# Barriers to utilize nutrition interventions among lactating women in rural communities of Tigray, northern Ethiopia: An exploratory study

**DOI:** 10.1371/journal.pone.0250696

**Published:** 2021-04-30

**Authors:** Selemawit Asfaw Beyene, Lemlem Weldegerima, Freweini Gebrearegay Tela, Omer Seid, Amal Tucker Brown, Afework Mulugeta Bezabih

**Affiliations:** 1 Department of Nutrition and Dietetics, School of Public Health, College of Health Sciences, Mekelle University, Mek’ele, Ethiopia; 2 Department of Nutrition and Dietetics, School of Public Health, College of Medicine and Health Sciences, Bahir dar University, Bahir Dar, Ethiopia; 3 Nutrition Section, UNICEF Ethiopia, Addis Ababa, Ethiopia; Tulane University School of Public Health and Tropical Medicine, UNITED STATES

## Abstract

**Background:**

While lactation is a physiological process requiring high energy demand to fulfill the nutrient requirements of the mother and the breastfeeding child, many factors affecting maternal nutrient intake can lead to nutritional deficits. Previous studies in Ethiopia have reported the prevalence of maternal and child undernutrition and related complications. However, qualitative studies exploring potential barriers to utilizing available nutrition interventions are limited. This study, therefore, sought to qualitatively explore barriers hindering the uptake of nutrition services among lactating mothers from rural communities in Tigray, northern Ethiopia.

**Methods:**

We conducted 6 in-depth interviews, 70 key informant interviews, and 13 focus group discussions among purposively selected community groups, experts, and lactating mothers between November- 2017 and January- 2018. Audio records of all interviews and focus group discussions were transcribed verbatim (word-to-word) and translated into English. Then, translated data were analyzed thematically using qualitative data analysis software Atlas ti-version 7.4.

**Results:**

The participants in this study perceived that lactating mothers in their study area are not properly utilizing available and recommended nutrition interventions, and as a result, their nutrient intake was reported as inadequate. Participants identified inadequate accessibility and availability of foods, feeding practices, cultural and religious influences, focus on agricultural production and productivity, barriers related to health services and poor access to water, sanitation and hygiene as major barriers hindering the uptake of nutrition interventions by lactating women in Tigray, northern Ethiopia.

**Conclusion:**

The uptake of nutrition intervention services was low among lactating mothers and was hindered by multiple socio-cultural and health service related factors requiring problem-specific interventions at community, health facility, and administrative levels to improve the nutritional status of lactating mothers in the study area.

## Introduction

Lactation is a nutritionally demanding stage of maternal life, and all nutrient requirements are increased during this period [1, 2]. Supplying the mother with an adequate diet that provides enough energy and nutrients to meet these requirements is essential for producing the optimum amount of breast milk, which is necessary for the health of the mother and the survival and development of her child [1].

Inadequate dietary and supplement intake during lactation can affect the nutritional status of the mother and also her breast feeding child [3, 4]. Inadequate dietary intake at this stage causes high rates of maternal and child morbidity and mortality. Moreover, poor nutrition among breast feeding children, mainly in the first 2 years of life, at a critical window of opportunity, leads to irreversible damage such as poor cognitive development and school performance, poor productivity, and intergenerational cycles of malnutrition [5–9].

Globally, maternal under-nutrition contributes to 800,000 neonatal deaths annually, associated with small for gestational age newborns (low birth weight). Moreover, stunting, wasting, and micronutrient deficiencies are estimated to cause nearly 3·1 million deaths annually in children [10]. Ethiopia is among the countries where pregnant and lactating women suffer from overall undernutrition. According to the Ethiopian Demographic and Health Survey (EDHS) 2016, 15.7% of women age 15–49 were thin (Body Mass Index less than 18.5) and about 24% of them were anemic [11]. Regarding child under-nutrition, there has been no significant reduction in stunting, wasting, and underweight since 2005. According to the 2019 mini EDHS, the national prevalence of stunting, wasting, and underweight was 37%, 7%, and 21% respectively. In the Tigray region, the prevalence of stunting, wasting, and underweight was 48.7%, 9.2%, and 30.4%, respectively [12].

Taking into consideration the high prevalence and severity of undernutrition among pregnant and lactating women, and children, the government of Ethiopia has been implementing evidence-based nutrition-specific and nutrition-sensitive interventions [13]. The country accelerated efforts to implement the second National Nutrition Program (NNP II) aiming for a reduction in the proportion of women of reproductive age with BMI <18.5% from 27% to 16%, a reduction in stunting to 26% by 2010, and zero stunting by 2030 [13, 14]. These targets can be achieved if the available evidence-based interventions, dietary diversification strategies, nutrient supplementation, nutrient fortification, nutrition education, dietary modification, and water, sanitation and hygiene (WASH) protocols [13, 15], are supported to be utilized by the community.

Nutrition interventions available for lactating mothers include the provision of nutrition education during the post-natal-care period, growth monitoring and promotion, and the well-baby clinic visits to facilitate dietary diversification, dietary modification, and utilization of iodized salt. Additionally, there are dietary supplementation programs for undernourished mothers and other indirect health services [13]. Despite the availability of these interventions in the community, their utilization by lactating mothers was reportedly low [16, 17]. While factors such as inadequate access to food, socio-cultural and religious influences, selling of food products, and poor access to WASH hindered program utilization in many low-income countries, evidence regarding the potential barriers to access such services in Ethiopia and specifically the Tigray region, is not understood well. While previous studies mainly focused on assessing the risk factors of low implementation/uptake of these interventions quantitatively, no study has been conducted to qualitatively explore community perceptions regarding the barriers to utilizing these high-impact nutrition interventions. Therefore, this study aimed to qualitatively explore barriers that could affect the ability of lactating mothers to utilize available nutrition services in the Tigray region of northern Ethiopia.

## Methods

### Study period and setting

This study was conducted in five woredas in the Tigray region, northern Ethiopia between November- 2017 and January- 2018. Tigray is the northernmost region of Ethiopia. The population of Tigray is estimated to be about 5,541,736 (CSA projection for 2019). About 80% of the population lives in rural areas. The region is administratively divided into seven zones, 52 districts, and 763 kebeles, the smallest administrative division. Currently, there are 2 referral hospitals, 14 general and 24 primary hospitals, 226 health centers, and 740 health posts. Details of the study design were reported elsewhere [18]. In brief, three food-insecure woredas (Ofla, Samre Seharti, and Tanqua Abergele) and two food-secure woredas (Laelay Maichew, Medebay Zana) were purposively selected for this study. Then two kebeles, the smallest administrative unit of about 5000 people, were selected from each woreda. Therefore, the study was conducted in 10 kebeles in the region.

### Study design

An exploratory qualitative study design was utilized in this study.

### Participant selection

Taking into consideration the principle of maximum variation, participants were purposively selected from different community groups and areas of expertise with the help of kebele leaders and health extension workers. For in-depth interviews (IDI), lactating mothers were selected; for key informants interviews (KII), participants from different community groups such as religious leaders, women development groups (WDG), kebele leaders, health extension workers (HEWs), agriculture extension workers (AEWs), and experts from various nutrition-sensitive and nutrition-specific sectors such as agriculture, nutrition, education, water, health, youth and sports services, and women’s affairs were selected. Lactating mothers, WDG, and women of reproductive age were selected for focus group discussions (FGD).

### Data collection tools and methods

Qualitative data for this study were explored from KIIs, IDIs, and FGDs. While KIIs and IDIs were carried out to explore people’s opinions towards potential barriers to accessing nutrition interventions during lactation in the studied communities, FGDs were conducted to investigate people’s interactions regarding the research objective and to triangulate the findings of the study from the perspectives of different community groups and experts.

After consulting experts from different disciplines such as public health, nutrition, agriculture, and food sciences and reviewing the literature, interview and discussion guides were developed to facilitate the KIIs, IDIs, and FGDs.

The guides mainly covered the types of nutrition interventions available and barriers to the uptake of the available interventions in the study area. The guides were developed in English, translated into the local language for the study area, and back-translated to ensure consistency. They were also pre-tested before the actual data collection was carried out, and comments identified during the pre-test were used to modify the tools. The pilot test was conducted in Ofla district; one FGD for each FGD category and one IDI each for district-level experts were conducted. KIIs, IDIs, and FGDs were facilitated by MSc/MPH holders with previous experience in qualitative data collection (with one note taker and one facilitator). While KIIs and IDIs were conducted in locations preferred by participants, FGDs were carried out either in a health post or community development center where the discussion could be held with minimal destructions. While the average time of KII and IDI was 45 minutes, FGDs took 70 to 90 minutes. All KIIs, IDIs, and FGDs were audio-recorded.

### Data analysis

Investigators listened to the audiotaped FGDs, KIIs, and IDI data several times to familiarize themselves with the data. All KII, IDI, and FGD audio records were transcribed verbatim and translated into English. Then, translated transcripts were imported into Atlas ti-version 7.4 qualitative data analysis software for coding. Following the principles of thematic analysis set out by Braun and Clarke, investigators applied line-by-line coding to inductively explore the potential barriers to accessing nutrition interventions during lactation in the studied communities. Following coding, identified codes were grouped based on similarities and differences to form categories. Finally, themes were identified. Participants’ quotes were reported directly as they were spoken, without editing the grammar, to avoid any loss of meanings.

### Trustworthiness

Member checks and prolonged engagement with participants were done to strengthen the credibility of the accounts. Data collectors, supervisors, and investigators spent a prolonged time in field work to facilitate sustained engagement with the study participants. For the member checks, participants who were willing to stay following each focus group discussion were asked to listen to the sound recordings and asked if they agreed with what had been said. Research assistants and investigators also conducted peer debriefing during data collection on a daily basis. To ensure the reliability of the data, data from FGD and IDI were triangulated. Coding was done by at least two investigators, and differences in coding were resolved via discussion. Transparency was maintained by recording each step taken from the start of the project until reporting the research findings. Researchers sought to set aside their assumptions, perceptions, and values, and prior knowledge to reduce bias during the collection, coding, and interpretation of data.

### Ethical consideration

The study was approved by the ethical review committee of the College of Health Sciences, Mekelle University, and permission to conduct the study was also obtained from Tigray Regional Health Bureau. Before conducting data collection, participants were informed about the purpose of the study, their right to participate and refuse at any stage of the study, and confidentiality. Consequently, verbal consent was sought from each participant. Verbal consent was audiotaped after information about the study was provided to study participants.

## Results

### Socio-demographic characteristics

Five IDIs, 70 KIIs, and 13 FGDs comprised the qualitative data collection. Seven FGD were conducted with lactating mothers, two FGD with women development army, and four FGD with men were conducted. In the lactating mothers’ FGD, the average number of participants was 11, with a minimum of six and a maximum of 12 participants. The mean age of participants was 26, with a minimum age of 18 and a maximum age of 40. The majority of the mothers had no formal education and were not formally employed outside the home. In the men’s FGD, the average number of participants was nine and ranged from six to ten. The FGD with WDA consisted of six and seven participants. Key informant interviews were conducted with 70 participants at the community and district level. About 47% of KII participants were female. Five IDI were conducted with lactating mothers, and their mean age was 31, ranging from 23 to 37.

The following themes were identified as the major barriers to the uptake of nutrition interventions by lactating women: inadequate accessibility and availability of food; feeding practices; cultural and religious influence; focus on agricultural production; barriers related to health services; and poor access to WASH.

### Inadequate accessibility and availability of food

The participants mentioned that inadequate availability and accessibility of food is a major challenge among lactating mothers. Mothers cannot access diversified food due to limited income, drought, limited access to markets, and scarcity of foods for purchase. Seed shortage was also one of the barriers hindering the production of vegetables for consumption.

*The main problem in the women of this community is a shortage of income*. *Even though the people have good awareness*, *they have a shortage of income to buy the required foods*. *For example*, *if they want fruit*, *they have to buy it and in order to buy the fruits*, *they must have the money*.

*(IDI*, *Agriculture and Nutrition officer*, *Samre woreda)*

*It is known that a lactating mother needs extra meals*, *but it depends upon the condition*. *If it is drought*, *she will eat little food*, *and if there is more production/harvest*, *she will eat more*.

*(FGD Women*, *Keyih Emba kebele)*

*They are affected by the absence of food items at the closest distance*. *For example*, *in our kebele*, *there is no vegetable and cereals produced […*.*] if mothers need these components of foods*, *they should go to the woreda city which is 30km from here*.

*(IDI WDG*, *Finaruwa kebele*, *Samre woreda)*

Even though experts advise the community to introduce home gardening to improve local nutrition practices by supplying fresh, cheap, and accessible fruits and vegetables, this was deemed impractical due to inadequate access to water and heavy dependence on seasonal rainwater. Moreover, the lack of gardening seeds near the community was also reported as a barrier to introducing home gardening into the community.

*What I have seen is there is no water at all*. *It takes 2–3 hours to fetch water in this community*. *Due to this problem*, *the community is challenged to work on nutrition through irrigation*.

*(IDI*, *Agriculture extension Worker*, *FelegeHiwot Kebelle*, *Tankua Abergele woreda)*

### Feeding practices and knowledge

Even where food was available to the household, feeding practices were identified to be inadequate. Mothers were resistant to change their dietary practices, despite receiving nutrition education and counseling by health care workers.

*We don’t use the homegrown foods appropriately as per the health experts’ advice*. *We don’t focus on the diversity and type of foods rather on the quantity; for example*, *if I don’t get hungry regardless of the food type*, *I don’t bother to address the variety and quality of the food types*.

*(KII*, *Lemlem Kebele*, *Saharti Samre Woreda)*

*They have all inputs in their home to get balanced diets*, *like they have egg*, *milk*, *and many other food sources in their home*, *however*; *there is a utilization problem due to a lack of awareness of the importance of diversified foods*.

*(IDI*, *Women Affairs Office*, *Medabay Zana woreda)*

On the other hand, most participants agreed that the mothers’ educational status was associated with dietary awareness influencing their feeding practices.

*The higher the educational status of mothers*, *the better their awareness towards the overall activities*, *like the feeding of diversified food and introduction of new agricultural technologies*.

*(KII*, *Agriculture extension*, *Finarwa kebele*, *Samre Woreda)*

*The women that have awareness can use the service better than unaware women because the aware women know the importance and the consequence of malnutrition then they will go to use the services*, *but if the women don’t know the importance and the consequences*, *they will not use the services*.

*(KII*, *head of Women affairs office*, *Laelay Maychew woreda)*

### Cultural and religious influence

Deeply rooted cultural norms and religious influences on nutrition such as how mothers should eat, excessive waste of food during ceremonies, the excessive workload of mothers, and fasting that could influence dietary practices and health were reported. For example, women do not eat quality foods in the absence of their husband, and others only eat food after their husband eats.

*As a culture*, *there is not any assumption that lactating women could eat better food than their husbands*; *if the husband doesn’t go home during lunchtime*, *the woman could stay fasting the whole day*. *This is very common in our community*.

*(IDI*, *head of agriculture office*, *Tankua Abergele Woreda)*

*In male-headed households*, *if the household head did not come home*, *the family*, *including children and pregnant women*, *will not eat; rather*, *they will wait until he comes*. *If the children and pregnant women eat*, *they will eat without sauce*. *[…] There is a practice that quality food is given to the husband and son*, *and the wife with her daughters will eat what’s left*.

*(IDI*, *Water Resource Office*, *Tanqua Abergele woreda)*

Participants mentioned that there is excessive wastage of food in cultural and religious ceremonies when farmers enjoy good productivity. This improper and exhaustive utilization of food whenever they have such ceremonies could lead to food shortage throughout the rest of the year, eventually leading to undernutrition and related consequences.

*There are wastages related to culture (ceremonies)*. *If their productivity is good this year*, *there will be many marriages and other religious festive meals*, *and they will end up with famine in the next year*. *There are some areas that required food support due to excessive wastage of foods in marriage and religious festive meals (“tezkar*”*) because the community awareness is low*.

*(IDI Agriculture and Nutrition officer*, *Samre woreda)*

The influence of religious fasting which prohibits people, including lactating and pregnant women, from consuming animal source foods was reported as a barrier hindering the uptake of nutrition interventions.

*Even if we advise them (health providers) that pregnant and lactating women should eat extra meals*, *they (religious leaders) do not allow them*. *Orthodox religion leaders will not allow women to eat in the important (religious) fasting times*.

*(KII*, *MCH expert*, *Tanqua Abergele woreda)*

Participants in this study mentioned that excessive workload for mothers, such as fetching water from long distance, was considered a norm in the study areas. Such excessive workloads lead to increased energy expenditure unless the energy lost is restored through adequate food intake and rest, which was not reported in the study area.

*Because we are living in rural communities*, *there are many activities that make us busy*. *We are very busy collecting animal feed like straw*, *forage*, *and the leftovers of sorghum*, *and since the animals are tied at home*; *we bring water from the river*. *In addition*, *I clean the animals’ feces*, *and all home activities are done by me*. *Thus*, *sufficient rest is not expected*. *After the child has been put to sleep*, *my husband and I are harvesting all our crops; thus*, *I am very much busy during the harvesting season*.

(*IDI women Lemlem Keble Samre Woreda)*

### Agriculture sector’s focus on production and productivity

Participants in this study perceived that the mothers’ dietary practice was affected not only due to the unavailability and unaffordability of food, but also due to the farmers’ market-oriented production and productivity. As a result, farmers prioritize selling their products rather than consuming the products, even though lactating mothers and other family members require such foods. This theme was mainly proposed by agricultural experts; their intervention is mainly focusing on advising farmers/producers to focus on products with high demand in the local market.

*One thing that is common and seen in our community is the selling of all agricultural products instead of using them as their food*. *For example*, *they produce honey*, *butter*, *and so on*, *but they take them to the market and sell it*.

*(KII*, *deputy head of Youth & Sport office*, *Tanqua Abergelle Woreda)*

*All the home-grown food groups are not properly fed to the lactating women*. *For example*, *home-grown products like honey*, *eggs*, *butter*, *nuts*, *sesame*, *and others are intended for market purposes*.

*(KII*, *head of the agriculture office*, *Tankua Abergele Woreda)*

Experts also recognized poor collaboration between the health and agricultural sectors. They believed that while the agriculture sector mainly focuses on agricultural products with limited attention given to nutrition-sensitive agriculture, the health sector also mainly focuses on nutrition-specific interventions, suggesting the need for strong inter-sectoral collaboration between agriculture and health sectors.

*The main factor is our poor understanding of the nutrition agenda; it was not our task and no concern was given to it; in the case of the health sector*, *it was their main task*, *but in the agriculture sector*, *the agenda of nutrition was not known*. *We only concentrate on the growth of agricultural production and the productivity of market-oriented products*.

*(KII*, *head of agriculture office*, *Tankua Abergele Woreda)*

### Health-service-related barriers

Despite an increase in health-seeking behavior from lactating mothers towards the available nutrition interventions in the communities, participants reported that utilization of the services was affected by two main factors; namely, poor access to health facilities and factors related to health care providers.

The unavailability of health facilities was reported as a key barrier to utilizing available services such as contraception. As a result, mothers reported that they are forced to either travel long distances to get health services or otherwise don’t access them. Participants in this study also perceived that the knowledge, commitment, and approach of health care providers affect the success of the service uptake by community members, including lactating mothers. They also reported that the quality of service in their community did not meet their level of expectation.

*Some of the mothers may not take the family planning methods timely due to the distance of the kebele*. *They don’t get the services quickly because some of them are living very far from the health post and they need somebody to come with them because they cannot come alone from very far places (they are expected to travel for more than two and a half hours)*.

*(IDI*, *HEW*, *Felege Hiwot kebele*, *Tanqua Abergele)*

*Though the professionals are there*, *the doubt in the community is in their competency*. *The clients worry and go back to their homes without utilizing the service*; *they don’t even want to visit there anymore*.

*(KII*, *School Teacher*, *Tanqua Abergale woreda)*

*This year*, *we were not screened*, *although we were pregnant and now lactating*. *Previously*, *they screened us every three months*. *But currently*, *no one is doing this*. *If we go to a health facility for other purposes*, *they measure us*. *But at this time*, *there is no campaign and community health day for screening*.

*(FGD women*, *Saharti Samre Woreda)*

Health care providers, mainly health extension workers, reported that because the kebeles are vast and households are scattered, they have difficulties accessing each household and counseling mothers and other family members per their plan.

*We didn’t conduct home-to-home follow-up visits continuously*, *since the kebele is vast and of difficult topography*.

*(IDI*, *HEW*, *Dinka kebele*, *Ofla woreda)*

### Poor access to water, sanitation and hygiene

Participants in this study identified water shortage as one of the main barriers to utilizing nutrition interventions to support lactating mothers and other community groups. Although some participants mentioned the availability of water in their area, they reported that either they have to travel long distances to access the water, or the available water is not safe/clean. The shortage of water was believed to present challenges to mothers in maintaining the hygiene and sanitation of themselves and their families. Moreover, it also made it difficult to prepare safe and clean food for their family, the lack of which could predispose them to infectious diseases and lead to undernourishment.

*Due to the shortage of water*, *they don’t hygienically prepare their foods*. *There is a critical shortage of water in this community*, *and the available water is very unpleasant and far*.

*(KII*, *HEW*, *FelegeHiwot kebele*, *Tankua Abergele)*

*There is a shortage of water*. *We know how much a woman needs water*. *The water source is very far away*, *and even the available water is not clean (it is river water)*. *(KII*, *HEW*, *FelegeHiwot kebele*, *Tankua Abergele)*

*Some may not have donkeys*, *and they fetch water on their backs; it takes about two to three hours for round trips to fetch water*. *In this case*, *even the pregnant and lactating women participate in the fetching of water*.

*(KII*, *head of agriculture office*, *Tanqua Abergele Woreda)*

## Discussion

This qualitative study was designed to identify potential barriers hindering the utilization of nutrition interventions among lactating mothers in the Tigray region of northern Ethiopia. The findings of this study identified not only barriers to nutrition interventions but also to nutrition itself, and highlighted that the accessibility and availability of food, cultural, and religious practices, feeding practices and knowledge, focus on agricultural production and productivity (weak nutrition-sensitivity of agriculture), barriers related to health services, and poor access to WASH were all major factors that hindered lactating mothers in the study areas.

In this study, the inaccessibility and unavailability of foods due to limited income, droughts, and dependence on seasonal rainfall, limited access to local markets, and scarcity of foods for purchase were reported as major barriers impeding adequate intake of different food varieties. Similar barriers were reported in studies from low-income countries and among low socio-economic status families in high-income countries [19–23]. A systematic review that assessed barriers to maternal nutrition in low- and middle-income countries identified that lactating mothers struggled to vary food intake sources due to low household income [19]. Other studies from the United States of America also showed that fruit and vegetable intake rates were low among low-income families [20, 21]. Such barriers may prohibit the adequate intake of foods in a given community, leading to under-nutrition and its related consequences. Though the effect of under-nutrition hinders the health status of the entire family, its impact is worse in the case of lactating mothers, as it affects the health of both the mothers and their growing children [9, 24, 25].

Feeding practice and knowledge were also reported as one of the barriers hindering nutrition intervention uptake by lactating mothers. Factors that could cause certain dietary practices could be explained beyond the availability and accessibility of foods. Diversified food intake is believed to be influenced by many factors other than only availability, such as an individual’s educational status, awareness, and readiness for change. In this study, even in families with adequate food access and availability, the practice of food source diversification was reported to be inadequate. While many studies acknowledge that education plays a significant role in utilizing nutrition interventions [26], it is well-known that education is necessary but not sufficient for the utilization of health interventions [27]. Similarly, individual resistance to changing dietary practices, despite having adequate knowledge and awareness is reflected by the understanding that not all education supports behavior change [28].

People place value on their culture and spirituality. As a result, their daily activities including household chores, fasting, and celebrating social ceremonies are governed by such cultural and religious norms. In our study, fasting among lactating mothers, giving priority to husbands and children in feeding, excessive wastage of food during social ceremonies, and the excessive workload of mothers were reported to influence the dietary practices and health of lactating women, and in turn, affecting the nutrition services provided. In line with this finding, many studies have revealed that cultural influences, community beliefs, and religious influences contribute to low uptake of nutrition interventions and have resulted in impacts on the nutritional status of mothers and children [29–31]. A study conducted in Tigray region, Ethiopia, showed that religious fasting negatively affected the diversified food intake of lactating mothers [32]. A systematic review revealed that domestic activities that excessively utilize the energy of lactating mothers’ were taken as normal practice in many countries, including Ethiopia [19].

The Ethiopian government is committed to the reduction of maternal and childhood morbidity and mortality by addressing the factors that negatively affect their nutritional status. As part of this commitment, the recently endorsed Food and Nutrition Policy was made to highlight the importance of multi-sectoral collaboration that promotes nutrition-sensitive and specific interventions for improving dietary consumption on the basis of quality and quantity and access to healthcare facilities [33]. In this study, however, poor multi-sectoral collaboration was reported as a major barrier to the adoption of nutrition interventions among lactating mothers. Farmers of the study area were mainly advised by experts in the local community to focus on products that are market-oriented, encouraging families to sell their agricultural and animal source products. As a result, it was less practical for lactating mothers and others in the community to consume diversified foods available at home. Similar studies from India reported that, despite the availability of such strategies at the policy level, grassroots implementation was limited [34].

Although some nutrition interventions were available in the study area, their utilization was compromised by factors such as distance and perceived poor quality of health care providers, commitment, and knowledge. The challenge of access to health care services which might hinder the utilization of nutrition interventions due to the aforementioned problems has also been reported in many other resource-limited countries [35, 36]. A recent study showed that healthcare providers’ limited knowledge and poor commitment were associated with poor utilization of healthcare services [37].

In this study, inadequate access to water for drinking, washing, and home gardening was reported as one of the major barriers to the local uptake of nutrition interventions. Although low utilization rates of all other nutrition interventions have an impact on the nutritional status of individuals’ poor access to WASH is particularly detrimental to the nutrition intake of mothers and children. Poor access to WASH could lead to infections such as diarrheal diseases, which ultimately cause under-nutrition. Moreover, poor access to water has an indirect effect on maternal nutrition due to the limited practice of home gardening that could lead to an inadequate intake of home grown diversified foods. The unavailability of water as a key cause for the low consumption of fruits and vegetables due to limited home garden practices was also reported in other studies [38, 39]. The impact of poor water access as a main predisposing factor for many infectious diseases that eventually lead to undernutrition has also been revealed [40–42].

### Strengths and limitations of the study

This study has several strengths. First, we collected the data from purposively selected community groups and experts from different levels and disciplines. Second, the findings of the study were triangulated from different perspectives: FGDs, KIIs, and IDIs and used validated qualitative approaches. However, the study also had limitations. It is difficult to avoid the effects of social desirability bias; in which participants might be reporting what they believe is acceptable to the facilitators. However, we have tried to minimize this through a detailed explanation of the research objective so that participants report what they feel.

## Conclusions and recommendations

The uptake of high-impact nutrition services was inadequate among lactating mothers and hindered by multiple socio-cultural and health-service. This study identified the scarcity and accessibility of food, inadequate feeding practices and knowledge, the focus on agricultural production for market, cultural and religious practices, health service quality, and poor access to WASH as major barriers to utilizing nutrition interventions by lactating mothers in the study area. Therefore, problem-specific interventions at the community, health facility, and administrative levels should be implemented to address these identified barriers.

## Supporting information

S1 File Interview guides. (ZIP)

S2 File Dataset. (ZIP)
