# Supplementary material for: Barriers to utilize nutrition interventions among lactating women in rural communities of Tigray, northern Ethiopia: An exploratory study
Source: PLoS One. 2021 Apr 30;16(4):e0250696. doi: 10.1371/journal.pone.0250696 (PMC8087028; doi:10.1371/journal.pone.0250696)
Supplement: S2 File — (ZIP) [file pone.0250696.s002.zip › S2_File.Doc/Community level Key informants/004_IDI_HEW_Dinka kebele_Ofla woreda.docx]

**Operational Research on Adolescent and Maternal Nutrition in Northern Ethiopia**

## In-depth interview responses with health extension worker

**Section A: Interview in details**

1. Zone: southern zone
2. Woreda: Ofla
3. Kebele: Dinka
4. Name of key informant: Zufan Kassie
5. Institution of key informant: Health post
6. Interviewer name: Mekonnen Haileselassie
7. Date of interview: 23/022010
8. Interview start time: 12:30
9. Interview end time: 2:00

**Section B: Socio-demographic and basic data of qualitative study participant**

| **Socio-demographic characteristic** | **KII** |
| --- | --- |
| Sex | Female |
| Age | 19 |
| Educational status | College education |
| Occupation/role in the community | Health extension worker |
| Service year | 9 months |

**Note:**

I: interview

P: participant

| **Section 1: common maternal (pregnant women, lactating women and adolescent girls) nutrition problems in the community** |
| --- |

**I**. What do women do to stay healthy in this community/woreda?

**P.** Special care and treatment is need for both the pregnant and lactating mothers. We insist the mothers to attend the health education program like using insecticide treated bed nets (ITN) to protect from malaria, check up during pregnancy.

**I.** What are the common nutrition problems in the community for women and adolescent girls?

**P.** The main problem in adolescents, pregnant and lactating mothers concerning nutrition is lack of awareness. They don’t put into practice what we learned them. For example, we give training about all types of nutrition and feeding system for a child above six months, pregnant and lactating women. But there is gap of working out at the ground. The shortage of fruits and vegetables and the community practical application of different health extension educations are the main problems of this Tabia. In our side, we give the education about nutrition in regular syntax. But there exist many practical problems when we observed at the ground. We educate about feeding practice, and to participate monthly in MUAC screening and we don’t think more feeding problems. But the community is affected by malaria, TB and the like. In pregnant women, we don’t face more disease related problems. But since it is hot area, abortion is our problem. In case of lactating mothers, we don’t encounter any problem.

**I.** What are the common nutrition problems in the community for women and adolescent girls?

**P.** We always give training about nutrition in this area, but they don’t apply into practice. There is back tradition in this area. We give training about nutrition like the demonstration of porridge by mixing the six types of food groups. Even though we showed them practically, there is a problem of implementation at their home that is why we face the severe and moderate acute malnutrition. These problems are not solved yet. We teach to prepare the six types of foods such as cereals teff, barley, sorghum, small millet, wheat, and mixing with pulses like bean, chickpea, lentil, (with a proportion of three part of the can/ሸንበር is cereals and one part of the can is pulses) then mix and make porridge for children and the pregnant women. This chemistry is important for the child and we teach the mothers monthly. Although foods like orange and banana are also critical it is not easily reachable. Farmers cultivate only teff, wheat and sorghum in the area. There are no other products of pulses like bean chickpea, lentils and fruits in the area. Transportation is also big problem. This Tabia is very far from Sesela which is the market place of our community.

In supplementation of micronutrients to protect night blindness, experts came from Mekelle and they were inspected and checked. And we didn’t face the problem of night blindness in pregnant and lactating mothers. Concerning anemia and blood pressure, it is not as such a problem of out Tabia. We didn’t observe any non-communicable diseases and goiter is not our problem. But one mother died at parturition due to diabetic problem this year.

Stunting is thin body condition (low height for their age) and under-weight is low body weight (low weight for their age). Stunting and under-weight is seen in some children in our Tabia. After screening the children with acute malnutrition, they have got supplementary feeding/phafa monthly. We advise the mothers to feed porridge their children. We also conduct monthly follow up to check whether they feed properly their children in their home.

**I.** Which women groups are most affected by these nutrition problems?

**P.** In my understanding the pregnant women are more affected since they feed the child in addition to themselves. The lactating mothers also affected because exclusive breastfeeding until six months are expected from mothers. Thus both pregnant women and the lactating mothers are sensitive to nutrition.

| **Section 2: Nutrition priorities in the peasants association** |
| --- |

**I.** Do you think it is necessary for your institution to get involved in work aimed at improving maternal nutrition? Explore for pregnant women, lactating women and adolescent girls.

**P.** Yes, it is very important to keep healthy for a child. In the health sector, nowadays it is given more emphasis to nutrition. The women development army with their cells get training about nutrition and the pregnant mothers have their own program and are given training to the pregnant and lactating mothers. But the focus area of nutrition education is only for pregnant and lactating mothers. Adolescent girls are not involved. We didn’t give any training to adolescents.

**I.** Why do you think this is necessary or not necessary?

**P.** For mental development, bright mind, and to have genuine thinking, the child should get balanced diet. I think mainly in the pregnant and lactating mothers is very important.

**I.** How can your institution at this level be involved giving examples from specific projects or work?

**P.** We are doing different activities about nutrition with agricultural sector. The agriculture experts advise the farmers to produce different crops and vegetables; and we have the knowledge about nutrition how to prepare and consume for their child and themselves. We also in collaborate with the head of the peasants’ association and the group leader to give training.

**I.** What kind of nutrition services do you spend most of your time on?

**P.** As I have discussed before, the service we are doing is like health education to create awareness and practically we demonstrate how to make porridge; in the health sector the out-patient therapeutic program is given and for moderate acute malnutrition we give plumpynut. In the nutrition area, we also try to create awareness among the community and to increase the consumption of mothers and children with diversified food. We teach mothers about the importance of balance diet food, we tell the mothers if not take nutritious food the mothers and their children become thin, not have bright mind, creating healthy citizen. We also show them practical based activities like porridge. Plus we call the mothers and give them health education monthly. The available resources for moderate acute malnutrition are plumpynut and ፋፋ.

**I.** Can you tell me some of the successful maternal nutrition interventions that you have implemented in this woreda?

**P.** The awareness of the community is improved every time. There was no any vegetable like carrot, Swiss chard, potato in the area. But after we have got training by regional experts about nutrition sensitive agriculture products, we introduce and mothers have got started to plant some home garden. This is the better work we have done. In the health sector, the service of distributing fafa is good. Creating awareness, porridge demonstration for pregnant and lactating mothers is very interesting. Iron folic acid service for pregnant mothers is given properly. But we didn’t give any responsiveness for adolescent girls.

| **Section 3: Nutrition interventions that improve adolescent and maternal health** |
| --- |

**I.** What kinds of nutrition interventions are in place to improve adolescent and maternal health in this woreda?

**P.** It is very interesting question. During antenatal care services, the pregnant have got all the available services in the health post. Like weighting, advising to eat balance diet food, if she eats two times a day before pregnancy, we advise to eat double (four times per day) or three times during the first pregnancy time. We advise to feed iodized salt. In our area only the iodized salt is available. Concerning rest during pregnancy, if we confirm that the mother is pregnant we write an official letter to the agriculture development agents not to involve in the water and soil conservation activities so as to get rest. The agricultural development agents are accepting our letter immediately. In this way we are doing in collaboration with them.

On the area of advising water, sanitation and hygiene services; being this is a rural area, they didn’t give more focus. Although we include in our teaching program, there is great gap on keeping their personal hygiene and sanitation due less availability of nearby water sources. Some community could get hand pipe water and some are not. We also distribute water guard (ዉሃ ኣጋር) to the community.

Regarding on the use of insecticide treated bed nets (ITN); everybody in this area is allotted the ITN. Being the area is endemic to malaria, the government has given great focus to distribute ITN. Thus accessing ITN is not our problem. Deworming is also exercised for children, lactating mothers.

Advising to mothers is equal for both pregnant and lactating mothers. In getting rest we write letters to agricultural development agent not to participate in the soil and water conservation until a child has got one year. Until the child has got strength, we advise the mothers not to expose for harsh environment. In collaboration with women development army, we have monthly discussion program for pregnant and lactating mothers about the overall health and nutrition issues. Either we show practically or give them education. Once we discus here with the women development army, they give education to their own groups/cells at their home.

Since the child could get affected by malaria, we advise to use the ITN properly for lactating mothers. We did nothing about nutrition in adolescent girls. But we have got good lesson now and we will also do in adolescent girls in the future. In the school, there is feeding program in the form of porridge during breakfast. Nutrition screening is also well practicing in the Tabia; and if the pregnant and lactating mothers are below the standard, they have given ፋፋ.

**I.** In your opinion, which programs are being implemented successfully (i.e. in the most effective way?)

**P.** From the listed once, monthly education is properly applied about the overall importance of nutrition; and it is implemented to each household at the ground. So in my opinion, it is successful in improving their awareness in the area of nutrition. Although they don’t applied practically what all they have learned. In deworming and vitamin A service at an interval of six months for children is also valued. More or less, we distribute all the available services properly. The community is also accepted for pregnant and lactating mothers as if they need rest. Pregnancy follows up; and both pregnant and lactating mothers request ፋፋ even they are normal or overweight. Because they thought ፋፋ is more balance diet to their child. But the home gardening practice is an infant stage in the area.

**I.** In your opinion, what are some of the reasons that the programs you mentioned are not effective?

**P.** Although we educate and show practically to the mothers, all what we learned them is not practically applied in their home. Even we go and try to show in their home in a practical way, most of mothers are not easily accept to apply what they learned. In the use of water guard (ዉሃ ኣጋር) is also a problem in some mothers. Reasons like bad smelling, heart attack are responded from mother why they didn’t use it. Keeping their hygienic status is also another problem. We advise them to keep hygienic, but in practice they do not apply it properly.

**I.** What are the implementation challenges that are specific to delivering the maternal nutrition interventions in the programs that we have been discussing?

**P.** In our problem, we didn’t go to home to home follow up continuously, since the tabia is vast and difficult topography. There is also a language barrier in between us. We speak Tigrigna whereas most of the mothers are not hear and speak Tigrigna. They are agowegna speaker. We use translator and it is not effective. The main concern of our idea is not translated properly. From the existing of four kushets in the area, only one kushet is Tigrigna speakers. Health extension workers are assigned based on lottery method. Thus the Agowegna speaker of health extension worker might not assigned into the area.

Some of the mothers are also resistant to accept our education. They considered simple and not as such important. We also educate husbands to help their wife during pregnancy and for lactating mothers. Most of them are helping their wives but some.

We have already discussed the main problem. Language problem, what are learned here is not practically applied at the ground. In case of supplies, there is no problem of iodized salt, iron folic acid or ITN; economically cereals like teff, wheat are available in the area but pluses like chickpea, bean, lentil, fruits and vegetables are the main problems in the area. because pulse are not growing in the area. Animal source foods are not consumed. They couldn’t slaughter animals for their consumption. Although they have livestock and livestock products like egg, chicken and butter, they are often destined for the market purpose and not for the household consumption, because egg, chicken, butter… are costly at market thus other priority agendas like school expenses, coffee, clothes and the like could cover from the sale of it. Relatively they consume milk since it is not sold and milk production is high.

Concerning collaboration between the nutrition sensitive and specific sectors; we give training in holistic approach. If we train about malaria protection, we also include nutrition, sanitation. Mostly we are doing in collaboration with agriculture experts and Tabia administrator. Our focus is on creating awareness on the area of keeping their health status in collaboration with the said stakeholders. But we didn’t do with education sector; that is why adolescents are not engaged in the nutrition education.

**I.** Which of these challenges are the most important?

**P.** In pregnant women, almost we all have discussed above and the main problems are properly practical application of what they learned. It is a great gap. Like taking rest, keeping sanitation and hygiene, taking extra food are other problems. Less availability of pulse crops, language barrier.

In lactating mothers, timely breastfeeding is a problem; in science a child should breastfeed about 10-12 times a day but the mothers are not applied accordingly. In case if the child is sleeping they never wakeup to the child to breastfeed even the whole day, this is contradictive to science. For them, the main problem is less practical application of what they learned. Language problem among us is also a problem. Economic problem is also another issue to get balanced diet; mostly their animal products sell to purchase other house expenses like coffee, sugar and the like. We give awareness about what they eat but it is not possible to convince mothers overnight. The mothers said that if we sale one goat we can buy a quintal of teff”. This is due to having large family size and economical gaps. But the change is relatively fair. We observed the improvement of consuming milk.

In adolescence, we didn’t do any education in the area of nutrition. Only there is food program that the students eat as breakfast in the school.

**I.** For these challenges that you mentioned, can you tell me of any successes or innovations that the Region/Woreda Offices have used to improve maternal nutrition service delivery?

**P.** Until now we didn’t discuss in details with other regional experts like what we have discussed today. But through time, we are starting to discuss with regional health bureau to solve the problems like the language barrier which is beyond to the district power. Still now, the problems are not identified appropriately; we are starting to detect it now. Therefore, we will try to place solutions in collaboration with respected bodies.

| **Section 4: Community factors affecting access to maternal nutrition interventions** |
| --- |

**I.** Can you think of barriers that prevent adolescents and women from using the programs and interventions that we have discussed?

**P.** As I have discussed above, they are not applying all things what we learned them; transportation is big problem. Less awareness, the nutrition activity is given more focus this year. So they do not clearly understand the importance of nutrition. Some of the individuals might not supportive. Language barrier also exists.

**I.** How can these barriers be addressed to improve maternal nutrition in the community/woreda?

It should be solved the language problem; others are already discussed.

| **Section 5: Other interventions that influence adolescent and maternal nutrition and health outcomes** |
| --- |

**I.** In your opinion, what programs or activities promote increased birth intervals in this woreda?

**P.** To promote increased birth intervals, awareness among the communities has been done. In the previous years, there was no more practice about long birth intervals in the community, but now they try to exercise in some of them. Relatively it is fair. The birth interval is 2-4 years.

**I.** What are the policy factors that affect age at first marriage?

**P.** Currently there is no marriage below 18 years in this Tabia. The rule is very tightly realized from the woreda to Kushet level. Underage marriage in this area is totally reduced. All the responsible bodies in this Tabia have great effort to protect underage marriage. We tell the community if there is underage marriage, she could expose to diseases like fistula. In this area we produce good awareness in the community.

**I.** In your opinion, what could be improved?

**P.** We teach the community about the consequences of underage marriage. The community clearly understands the problems faced during underage marriage and the opportunity when she marries at normal age. Therefore, awareness creation among the community and strengthening the present inclinations is very critical.

**I.** Can you think of any other opportunities to prevent early marriage and increase birth spacing?

**P.** The awareness of the community towards preventing early marriage and increase birth spacing is well recognized. The tabia administrator is doing well on this area.

| **Section 6: Multi-sectoral collaboration to improve maternal nutrition** |
| --- |

**I.** Which other sectors do you feel are necessary to work with?

**P.** For the improvements of maternal nutrition, we can’t cover all the activities by ourselves only. If all the responsible bodies like teacher, police, Tabia leader, agricultural experts are engaging; it is easy to convince the community and to apply accordingly.

**I.** How do you see the other institutions’ roles complementing your role in improving maternal nutrition?

**P.** It is good. The Tabia leader could convince easily to the community about nutrition importance. And mostly we are doing our activity in collaboration with the agriculture experts. Because they know what the farmer can crop during the cropping season. Therefore, to implement our nutrition education activity practically at the ground, cooperation with all stakeholders is critical.

**I.** What type of resistance to the needed change do you perceive or have you experienced so far with stakeholders?

**P.** It is our problem. We didn’t create strong integration among all stakeholders appropriately. We don’t have common plan.

**I.** How effective are the coordinating platforms in enhancing multi-sectoral coordination?

**P.** We are doing our activity with the agriculture experts like introducing of home garden vegetables, allowing pregnancy and lactating mothers to get rest during the soil and water conservation program. The same is true with the Tabia leader. We take the training about nutrition with agriculture experts that are why they are applying it. But we didn’t do with other respective stakeholders like education.

**I.** What needs to be done to improve the capacity of these bodies/platforms for effective coordination?

**P.** To continue the coordination among stakeholders, we the health sectors, agriculture, education, Tabia leaders (the four fronts) should be work in strong collaboration.

**I.** Do you have any other comments on anything that we have discussed?

**P.** I understand many gaps in our activities, I learned a lot from you. Especially we didn’t do anything in the adolescent girls about nutrition. Thus you direct us to work in collaboration with the four fronts (agriculture, education, health, and Tabia leaders). We have got good lessons. We have an opportunity to work together with stakeholders being we are in near office.

**I: Thank you very much for taking the time to discuss!**

**Summary of each section**

| **Section 1: common maternal (pregnant women, lactating women and adolescent girls) nutrition problems in the community** |
| --- |

The main nutrition problem in adolescents, pregnant and lactating mothers is lack of awareness. They don’t put into practice what we learned them. Additional foods like orange and banana are easily accessible in the area. Farmers are also cultivating only teff, wheat and sorghum in the area. There are no other products of pulses like bean chickpea, lentils. Water and transportation access is also a problem in the Tabia.

| **Section 2: Nutrition priorities in the woreda** |
| --- |

Focus towards to nutrition is increased by the government. The chain from health extension to women development army and the cells (containing pregnant and lactating women) get strengthen. But the focus area of nutrition education is only for pregnant and lactating mothers. Adolescent girls are not involved.

| **Section 3: Nutrition interventions that improve adolescent and maternal health** |
| --- |

We teach pregnant women to eat balance diet food, if she eats two times a day before pregnancy, we advise to eat double (four times per day) or three times during the pregnancy time. We also encourage getting rest during pregnancy. If we confirm that the mother is pregnant we write an official letter to the agriculture development agents not to involve in the water and soil conservation activities so as to get rest. And they accept our letter immediately. But keeping their personal hygiene and sanitation is compromised due to less availability of nearby water sources. Some community could get hand pipe water and some are not. Being the area is endemic to malaria, the government has given great focus to distribute ITN. Everybody in this area is allotted the ITN.

| **Section 4: Community factors affecting access to maternal nutrition interventions** |
| --- |

The Tabia is vast and difficult topography as the result we can’t continuously oversee through mothers’ home to home visit. There is also a language barrier in between us. We speak Tigrigna whereas most of the mothers are not hear and speak Tigrigna. They are agowegna speaker. We use translator and it is not effective. The main concern of our idea is not translated properly. From the existing of four kushets in the area, only one kushet is Tigrigna speakers. Health extension workers are assigned based on lottery method. Thus the Agowegna speaker of health extension worker might not assigned into the area.

| **Section 5: Other interventions that influence adolescent and maternal nutrition and health outcomes** |
| --- |

Currently there is no marriage below 18 years in this Tabia. The rule is very tightly realized from the woreda to Kushet level. Underage marriage in this area is totally forbidden. To promote increased birth intervals, awareness among the communities has been also created and satisfactory change has been shown in the Tabia.

| **Section 6: Multi-sectoral collaboration to improve maternal nutrition** |
| --- |

For the improvements of maternal nutrition, all the responsible bodies like teacher, police, Tabia leader, agricultural experts should be engaged. Currently we are doing our activity in collaboration with the agriculture experts. But we didn’t create strong integration among all stakeholders (health, education, and agriculture and Tabia leader) appropriately. We don’t have common plan.

**Additional comments**

I understand many gaps in our activities, I learned a lot from you. Especially we didn’t do anything in the adolescent girls about nutrition. Thus you direct us to work in collaboration with the four front (agriculture, education, health, and Tabia leaders) stakeholders.
