# Supplementary material for: Barriers to utilize nutrition interventions among lactating women in rural communities of Tigray, northern Ethiopia: An exploratory study
Source: PLoS One. 2021 Apr 30;16(4):e0250696. doi: 10.1371/journal.pone.0250696 (PMC8087028; doi:10.1371/journal.pone.0250696)
Supplement: S2 File — (ZIP) [file pone.0250696.s002.zip › S2_File.Doc/Community level Key informants/007_IDI_HW_Korem HC_Ofla Woreda.docx]

## Tool A

## IN-DEPTH INTERVIEW for Health Worker in Korem Health Center

| **Section A: Interview details**   1. Zone:__South___________ 2. Woreda: ------Ofla----------------------------------- 3. Kebele: ---------Korem--------------------------------- 4. Name of key informant: ---------Meron Shiferaw-------------------- 5. Institution of key informant: ------------Health Worker--------------------------- 6. Interviewer name: ----------Dejen Yemane------------------------------------ 7. Date of interview -----------November 3, 2017------------------------------------- 8. Interview start time: _________________________ 9. Interview end time: __________________________ | |
| --- | --- |
| **Section B: Interviewee professional information**   1. Gender    1. Female    2. Male 2. Age: ___25______ yrs 3. Highest level of completed education.    1. No formal education    2. Primary education    3. High school    4. College education    5. Diploma Certificate    6. Bachelor degree    7. Master’s degree 4. Current job/position: _____Clinical nurse in MCH clinic staff_____________ 5. How long have you been in the current job/position?    1. __3____ Months    2. ______ Years | |
| **Section 1** | **Common maternal (pregnant women, lactating women and adolescent girls) nutrition problems in the community.** |
| 1.1 | What do women do to stay healthy in this community/woreda?  **Participant:** In our community the Kebelle, Women development army, HEWs and health center give support to pregnant and lactating mothers and adolescent girls. Especially HEWs teach the community about health and health related issues in a home-to-home basis and the HC monitors their activities closely. So, there is support through the one-to-five network.  Regarding nutrition HEWs demonstrate preparation of porridge especially for under two children in selected central areas. In this event kebele administrative/executive bodies and health center staffs are involved. In addition, pregnant and lactating mothers get support from government like FAFA starting from the summer. The Health center also provide FAFA and PLAMPYNET for malnourished children.  Most of the time the focus is given to pregnant and lactating mothers than the adolescents. There are no activities/programs/interventions on fertile age group (to mean adolescent girls). |
| 1.2 | What are the common nutrition problems in the community for women and adolescent girls?  **Participant:** In our catchment the problems could be economy related. Always the problems are either due to economic problem or lack of awareness. In addition, lack of access to HEWs could be the problem. Because there is no enough number of HEWs that go and teach mothers and adolescents door-to-door.  Though there are many nutritional problems; severe and moderate malnutrition, micronutrient deficiencies, stunting and food insecurity are the main nutritional problems in our community. It is common to see anemic and hypertensive women, but it is less likely to get obese mothers and adolescents. Though, stunting and underweight is not comment in mothers and adolescents it is common to get stunted and underweight mothers and adolescents. May be the possible reason why stunting and underweight is not common in mothers and adolescents could be unlike in children in adults it is very difficult to identify stunting and underweight. |
| 1.3 | Which women groups are most affected by these nutrition problems?  **Participant:** As to me women groups most affected by nutrition problems are pregnant and lactating women. This is because though pregnant women should get extra meal due to lack of food supply they may not get enough food and sometimes due to poor economic status and negligence. In lactating women there is lack of self-support and care. Therefore, pregnant and lactating women are more affected by nutrition problems. |
| **Section 2** | **Nutrition priorities in the woreda** |
| 2.1 | Do you think it is necessary for your institution to get involved in work aimed at improving maternal nutrition? Explore for pregnant women, lactating women and adolescent girls.  **Participant:** Needless to mention, our health center gives support to lactating and pregnant women and it is essential/necessary. The reason why it is necessary for our institution to get involved in work aimed at improving maternal nutrition could be because we will create new generation/citizen and to grow properly we need to work with mothers to properly feed those children. To realize this our health center gives support to mothers based on the capacity we have.  Firstly, this is done by the community for the community and this needs community mobilization. Therefore, our institution is involved in community mobilization and awareness creation about nutrition and health in general as a citizen and health professional and this is our obligation. Thus, this way if we teach the community about what is nutrition, what to take and not take and what are the health problems related to nutrition, this is part of an involvement. |
| 2.2 | What maternal nutrition (pregnant, lactating and adolescent girls) interventions are the priorities in this woreda?  **Participant:** As a health center there is FAFA support program for pregnant, lactating and children in a monthly basis. In this program SAM and moderately malnourished mothers are included. Most of the nutrition interventions in place are focused in pregnant and lactating mothers and we spend most of our time on those interventions. Regarding the question what nutrition interventions have the most resources allocated nutrition interventions, I have no idea about the budgetary issue. |
| 2.3 | Can you tell me some of the successful maternal nutrition interventions that you have implemented in this woreda?  **Participant:** During pregnancy, starting from conception to they give birth there is a follow up and when they give birth it has its own way. In our health center, staffs contribute and saves money on a monthly basis.  As an experience we bye and store porridge flour. For this activity we have a separate room where mothers prepare their porridge and this way the health center supports the mothers. Then the rest will be their responsibility because they go home. In addition, we teach and council them what to do. |
| **Section 3** | **Nutrition interventions that improve adolescent and maternal health** |
| 3.1 | What kinds of nutrition interventions are in place to improve adolescent and maternal health in this woreda?  **Participant:** When a mother comes to our health institution, especially if she is pregnant we let her to start ANC at 5^th^ month. When she starts ANC, we advise her to follow her ANC starting from the first visit to the last visit and we council her what to take, for example we administer iron-folate in every visit. In addition, we administer Tetanus Toxoid and we council about their feeding. We also advise pregnant women to feed one additional meal per day, for example if she were feed 2 meals per day we advise her to take three meals per day. If they have their own house we advise them to garden plant source of foods, like salad and cabbage based on their capacity. On top of this we council them to get enough rest, at least they must take rest and sleep per day. Thus, when they come to our health institution we tell them the aforementioned advices and we do all the necessary things. When we measure MUAC, if we get mothers below the cut off/normal we enroll them to Outpatient Therapeutic Program (OTP) service or refer to Therapeutic Feeding Centers (TFU) if it is complicated SAM. Therefore, we follow mothers with below normal MUAC this way.  When lactating mothers come for post-natal care after 45 days we council them about feeding, vaccination, when they should come. In relation to this, we council them what type of food they should take, how frequent they should feed, how to breastfeed their child, and about family planning method.  Regarding iodized salt, because HEWs teach the community about utilization and benefits of iodine and all the community utilizes it and they now very well about its benefits. However, we don’t council about utilization of Insecticide treated bed nets because our area is highland. Whereas, regarding water, sanitation and hygiene and latrine utilization HEWs teach them in a daily basis better than us. In general, we give all-inclusive counseling.  Regarding adolescents, to tell you frankly, there is no strict follow up like pregnant and lactating mothers our focus is on pregnant and lactating mothers. For Adolescents most of the time we treat only for their complaint when they come for family planning, treatment or other purposes. Therefore, I don’t believe that we worked on adolescent nutrition. |
| 3.2 | In your opinion, which of the above programs are being implemented successfully (i.e. in the most effective way?) Why? **Explore for pregnant women, lactating women and adolescent girls.**  **Participant:** As I have told you before we focus on pregnant and lactating mothers and they are successful. This is because pregnant and lactating mothers are priority target populations of the government and the health institution and as a result the activities/programs outweigh to these target groups. Therefore, there is lack attention to adolescents due to either lack of prioritization or negligence.  About the successful interventions in pregnant and lactating mothers I can say all are successful. However, there is a long way to go on nutritional interventions on adolescents. |
| 3.3 | In your opinion, which of the programs mentioned above are less effective? Why? **Explore for pregnant women, lactating women and adolescent girls.**  **Participant:** For pregnant and lactating mothers, it is enough, but we need to work on the adolescents because we have a huge gap in this age group. Possible reason for the limited focus for adolescents could be lack of attention from the high ups/government. If you look at the pregnant and lactating mothers and children due to the presence of focused direction from the government, it is going well. Therefore, this gap in the policy and strategy makes a critical gap in the grassroot level. |
| 3.4 | What are the implementation challenges that are specific to delivering the maternal nutrition interventions in the programs that we have been discussing? **Explore for pregnant women, lactating women and adolescent girls.**  **Participant:** There are many challenges starting from prioritization. Firstly, there is coordination problem because there are circumstances where nutrition issues are seen as one sector issue due to lack of coordination and collaboration. If there is no collaborative thinking, sectors can’t know whom is working what and they will not help each other. In addition, there might be resource constraint based on our economic context and as a result there might be problem of blanket coverage on the maternal nutrition interventions. The other main implementation challenge is that lack of refreshing trainings for staffs because science is updating from time-to-time. These all are implementation challenges, but the bottom line is that lack of coordination and collaboration and sense of belongingness. Because there is a thinking that nutrition issue is health sector’s issue and health sector cannot address this issue alone. |
| 3.5 | Which of these challenges are the most important?   - Pregnant women   **Participant:** One of the challenges is clients (pregnant, lactating and adolescents), instead of looking the benefits of the services provided when we advise them they consider our advice as an order/obligation. Sometimes, when you nag them to utilize the services they even consider it as if you are begging them for your benefit. So, from the client side there is a problem of buying in the advices due to either lack of awareness or negligence and this a gap in our mothers.   - Lactating women   There is no difference, it is similar with the pregnant mothers. They are lactating, and they have postnatal follow-up, but they procrastinate their appointments by saying I will go tomorrow. Similarly, to the pregnant mothers, lactating mothers also did not consider the benefits that the timely follow up will give them. So, there is so many gaps.   - Adolescent girls   In adolescents as I have told you due emphasis is not given starting from the top level and when it goes down to the lower level of the health system it lacks focus. This is not because of the adolescents rather it can be by the staff or the policy maker, I don’t believe that that much attention is given. Therefore, it is our gap and it is also the gap of the higher bodies. |
| 3.6 | For these challenges that you mentioned, can you tell me of any successes or innovations that the Region/Woreda Offices have used to improve maternal nutrition service delivery?  **Participant:** As a government, meaning including actions taken on children, there are actions taken to support and help them (pregnant and lactating mothers). However, since we don’t have that much follow up for adolescents as a woreda or kebele. woreda is the leader and coordinator therefore, as a health center we work by giving due attention to pregnant and lactating mothers. But also as a town it is led by Mayor and like other sector as a wored and Mayor in general I don’t believe there is support. But since this issue is given to health sector woreda and health center support based on the capacity they have. Is it satisfactory or does it have universal coverage? it couldn’t, it doesn’t cover universally. As a woreda and health center capacity, they get support and help. |
| **Section 4** | **Community factors affecting access to maternal nutrition interventions** |
| 4.1 | Can you think of barriers that prevent adolescents and women from using the programs and interventions that we have discussed? **Explore for adolescent girls, pregnant women and lactating women.**  **Participant:** As to me I think in pregnant mother as a barrier; you council saying take rest, take extra meal, but she has children to care at home and she might be married. To fix this all she might have no enough time, and this could be one factor. In lactating, it is because of being obsidian. Sometimes mothers, traditionally perceive that it is enough if we breast feed the child once or twice. Therefore, now there is little awareness gap. They teach them, but all did not retain what they were thought, so it needs time.  Can you tell me additional information on the barriers?  Yes, for example, for not to start follow up during her pregnancy, the distance of health center from her house and time spent prevents adolescents and women from using the programs and interventions. In addition, not to expose her pregnancy to the public hinders not to come repeatedly to the health facility. But now it is better than the previous times. This time we ask them in a way it is comfortable for them. If they are from town it has no problem but if they are out of town health center also serves when they come from rural areas. Thus, it is in a way comfortable to them and the good thing is they did not forget appointment because they remind their appointment date when you tell them based on the holly days like “Maryam” and Gebriel”. We ask them comfortable date for them and we ask them for their convenience. When she says this date is comfortable for me we give them the appointment. In addition, there was a pressure by the community pregnant mothers not to eat good foods like meet but this time this thing is not that much in existence and they come properly. Therefore, we don’t take this as a barrier. |
| 4.2 | How can these barriers be addressed to improve maternal nutrition in the community/woreda?  **Participant:** Primarily what we must do is as I have told you we don’t prescribe because if we prescribe, it will not be based on what they have at home and surrounding, and it will not have a meaning. Therefore, instead of prescribing we discuss and recommend the appropriate action and she will decide based on the items she has at home. In addition, we identify factors/causes that hinder not to do these practices/activities by asking them. Then she will list the berries. Then we ask her what time is convenient for her just appropriate time for her. The after asking her what is convenient for her. In general, these problems can be solved by discussing with them. If we are able to solve those barriers by our capacity, we give solutions. |
| **Section 5** | **Other interventions that influence adolescent and maternal nutrition and health outcomes** |
| 5.1 | In your opinion, why would increasing the space between each births and delayed marriage (after 18 years) improve maternal nutrition and hence both maternal and infant health?  **Participant:** Now because be it birth spacing or what you have said it now, firstly by increased birth intervals they are the beneficiaries. Firstly, it is related with economy. It is the first issue. Secondly, it is related with their health. If pregnancy occurs within two years of delivery, there is increased risk of poor health outcome. Therefore, we tell them what benefits will they gain by birth spacing. Regarding the economy, if she gets pregnant and give birth while she has an infant it has its own economic factor. Therefore, we sit and discuss on these factors and we reach on to consensus.  Regarding Early marriage it is associated with negative health due to adolescent pregnancy and childbearing and rearing, divorce and economic consequences like loss of education and employment opportunities. In addition, young girls are not mentally and physically ready to give birth and take care of the baby.  **In relation to this question what types of activities/programs are in place that help to birth spacing?**  Firstly, family planning methods. There are different types of family planning methods and when they come after 45 days we directly link them from the vaccination room to family planning room so that to get family planning services. Because sometimes there are mothers who wants to stay and be a challenge for us but when we tell them the situation they simply understand it. |
| 5.2 | In your opinion, are these programs or policies effective? Why or why not?  **Participant:** As health center there are youths coming to our youth clinic and they have their own separate class. By chance they had taken training on pear to pear and they go to the community it could be in school or youth centers they give education. Therefore, as health center there is youth led and as a woreda it is led by youth office.  **Additional question, tell me if there are additional policies/programs regarding early marriage and birth spacing in relation with religion and politics**.  Here it is the contribution of all but be it religious or political leaders they are not working with that much attention. Therefore, as a health center as an independent institution and led by the woreda there are initiatives but as politicians and religious leader it is not that much. |
| 5.3 | Can you think of any other opportunities to prevent early marriage and increase birth spacing?  **Participant:** To space among births the best option is using family planning. It is to use family planning and the majority of the society living in urban and rural areas has access to family planning methods and utilize it. In this regard we believe that we are successful.  **In relation to this, are there factors that contribute early marriage in the community?**  Early marriage in urban is not that much, but we cannot say there is no early marriage in the rural areas. Though it is small in number, there is early marriage in the rural areas. There are students aged 15 years who came from rural areas for schooling and when they come to use family planning, we ask them why do they use family planning, they tell us that boys try to abduct us.  **So, are you saying that the policy is not implemented?**  Yes, there are implementation challenges.  **What must be done** **to prevent early marriage and increase birth spacing?**  To prevent early marriage, we can promote during meetings and teach at schools in a special case. This is not only issue of health, so all sectors must own it and work collaboratively and if teach at schools it will be successful. So, educating the public by giving special emphasis is needed. In addition, in order not to be boring you can teach the public using the volunteer youths.  **You have touched it in one or the other way but in can you think of any other opportunities to prevent early marriage?**  One of the opportunities is omen development army, women association, presence of constructional low against under age marriage. For example, the health center is the one who started it first and the community have good awareness about health and are willing to give support. Therefore, the first thing is using these volunteers and secondly as I have told you using the youths we can conduct health education and awareness campaigns. Either health worker or school teacher can promote prevention of early marriage especially using female students involved in clubs. Thus, you can share information and promote different messages. |
| **Section 6** | **Multi-sectoral collaboration to improve maternal nutrition** |
| 6.1 | Do you feel it is necessary for your institution to work with other sectors/institutions to address maternal nutrition?  **Participant:** From sectors for example there is social affairs office and this office has a relationship with the health center. But if you ask is this enough? Not. Because there is many pregnant and lactating mother therefore the coverage is too much and there is luck of supply. Therefore, it would have been better if other sectors help us though who is responsible for this issue will be determined by social justice office but is not satisfactory. For example, if we add education office we can do many activities with education even excluding nutrition we can do together on family planning, early marriage and others we can be successful.  **In relation to this, what is other institutions’ role in complementing your role in improving maternal nutrition?**  Starting from July 2016, the health institution staff by themselves has initiated the “one birr for one mother” initiative. This saved money can be used by the pregnant or lactating mothers. This was latter cascaded as an activity to different sectors and staffs of these sectors has fulfilled its obligations though we failed to sustain it. |
| 6.2 | For multi-sectoral action that effectively works to improve maternal nutrition at all levels, what kind of change in terms of the way stakeholders work together is needed? What type of resistance to the needed change do you perceive, or have you experienced so far?  **Participant:** Either the health center or woreda must schedule fortnightly or monthly community conversations thus you can teach health education, or you can support those who don’t start follow up, mothers who don’t have awareness about the community conversation could be identified and enrolled.  **What type of resistance to the needed change do you perceive, or have you experienced so far?**  To do so it needs capacity. You cannot succeed just by calling the community without nothing, so it needs capacity and support. The woreda also must support the health center by the possible capacity they have. |
| 6.3 | To what extent does your institution participate in the multi-sectoral nutrition coordinating body at the woreda level?  **Participant:** So far there is no nutrition related activities done in collaboration with other sectors. Except the self-motivated initiatives there is no collaborative work with other sectors.  **What needs to be done to improve the capacity of these bodies/platforms for effective coordination?**  Other than nutrition we have platforms where we meet with other sectors for other purposes. However, all sectors don’t actively participate by the sense of belongingness. |
| 6.4 | Do you have any other comments on anything that we have discussed?  **Participant:** From this interview as health center staff you asked me many questions and It indicated me to identify things that we should do that currently we are not doing. Specially in adolescents, we are not giving attention, and this is our bigger gap and we will do on this. In addition, I understand the government is committed to address the nutritional problems especially stunting. The other thing, we are doing good on the maternal nutrition interventions, but we will work to make it better. In adolescents we did nothing. Therefore, we are expected to do more and in general it is good. |

**Summary**

**Section 1**

- Severe and moderate malnutrition, stunting and food insecurity are the main nutritional problems in the community. Though, stunting and underweight is not comment in mothers and adolescents it is common to get stunted, underweight, anemic and hypertensive mothers and adolescents in the community.
- Most nutritional activities/programs/interventions are focused on pregnant and lactating mothers and there is limited attention to adolescents.
- Women to stay healthy they are networked and get education from HEWS.
- Malnourished pregnant and lactating mothers and their children get FAFA and PLAMPYNET.

**Section 2**

- The interviewee thinks that involvement of the health center in work aimed at improving maternal nutrition is necessary and obligatory. Thus, the health center is involved in community mobilization and awareness creation about nutrition and health in general.
- The priority maternal nutrition interventions in this woreda are pregnant and lactating mothers.
- The interviewee doesn’t know which nutrition interventions is taking much of the allocated resources.
- Regarding to the successful maternal nutrition interventions that you have implemented in this woreda, we save money on monthly basis to buy porridge flour for laboring mothers and we have separated a room for mothers to prepare porridge.

**Section 3**

- In pregnant women Iron foliate supplementation to prevent anemia, TT vaccination to prevent neonatal tetanus, counseling on one extra meal and rest during pregnancy, nutrition screening and enrolling to Outpatient Therapeutic Program (OTP) service or refer to Therapeutic Feeding Centers (TFU) if it is complicated SAM are in place to improve maternal health.
- For lactating mothers coming for post-natal care we council them about type of food they should take and frequency of feeding, how to breastfeed their child, iodized salt utilization and benefits, and about family planning method. They also inform lactating mothers
- They don’t council about utilization of Insecticide treated bed nets because the area is highland.
- Whereas, regarding water, sanitation and hygiene and latrine utilization HEWs teach them in a daily basis better than us.
- The maternal nutrition (pregnant and lactating mothers) programs are effective.
- The reason why they are effective is that because pregnant and lactating mothers are priority target populations of the government and the health institution.
- Whereas, nutritional interventions focusing adolescents are not effective due to either lack of prioritization or negligence.
- Adolescent nutrition is less effective, and this could be due to lack of attention in the from the policies and strategies of the government. Therefore, to address this critical gap it needs consorted effort at all levels.
- The main implementation challenge is lack of coordination and collaboration and sense of belongingness. But there are also other implementation challenges like lack of prioritization, looking nutrition as one sector issue, resource constraint, limited coverage, lack of refreshing trainings for staffs.
- Pregnant mothers don’t buy-in the advices and sometimes they consider as if we are obliging them due to either lack of awareness or negligence. Whereas, in lactating mothers there is procrastinating appointments.
- Regarding adolescent due emphasis is not given starting from the top level down to the lower level of the health system.

**Section 4**

- Barriers like work overload, traditional beliefs, lack of awareness, failure of mothers to retain what they have been thought, distance of health center, time spent, not to expose her pregnancy to the public can prevent adolescents and women from using the programs and interventions
- Barriers that prevent adolescents and women from using nutritional programs and interventions can be addressed by involving mothers on decision instead of prescribing, identifying factors/causes that hinder not to do these practices/activities by asking them.

**Section 5**

- Delaying pregnancy until the right time and spacing pregnancies can improve maternal nutrition and hence both maternal and infant health by minimizing poor health outcome, divorce, economic consequences like loss of education and employment opportunities, and inability to give birth and take care of the baby.
- Family planning and marriage laws are opportunities to prevent early marriage and increase birth spacing, though there is implementation challenge in early marriage.
- Therefore, to prevent early marriage and increase birth spacing, promoting during meetings and at schools is very important.

**Section 6**

- Working with other sectors in collaboration is necessary because nutrition is not one sector issue. Though it is not enough we are currently working in collaboration with social affairs, social justice and education offices.
- Though, there is no nutrition related activities done in collaboration with other sectors so far, the way stakeholders work together can by planning and scheduling together.
