# Supplementary material for: Barriers to utilize nutrition interventions among lactating women in rural communities of Tigray, northern Ethiopia: An exploratory study
Source: PLoS One. 2021 Apr 30;16(4):e0250696. doi: 10.1371/journal.pone.0250696 (PMC8087028; doi:10.1371/journal.pone.0250696)
Supplement: S2 File — (ZIP) [file pone.0250696.s002.zip › S2_File.Doc/Community level Key informants/021_FGD_Men of reproductive age_Hashenge kebele Ofla Woreda.docx]

**Operational Research on Adolescent and Maternal Nutrition in Northern Ethiopia**

## FGD for men participants

**Section A: Interview in details**

1. Zone: South Tigray
2. Woreda: Ofla
3. Kebele: Heshenge
4. Interviewer name: Mekonnen Haileselassie
5. Date of interview: 25/02/2010
6. Interview start time: 3:30 AM
7. Interview end time: 5:00 AM

**Section B: Socio-demographic and basic data of qualitative study participants**

| Name of FGD participant | Codes | Age | Marital status | Education level | Occupation |
| --- | --- | --- | --- | --- | --- |
| Mestofa Ali | 01 | 42 | Married | Illiterate | Farmer |
| Ibrahim Mestofa | 02 | 32 | Married | 5^th^ | Farmer |
| Hagazi Kalayou | 03 | 30 | Married | 4^th^ | Farmer |
| Said Aymut | 04 | 28 | Married | 7^th^ | Farmer |
| Humdi Mure | 05 | 35 | Married | 2^nd^ | Farmer |
| Hussen Eyasu | 06 | 31 | Married | Illiterate | Farmer |
| Darge Abadi | 07 | 27 | Married | 10^th^ | Farmer |
| Hassen Halefom | 08 | 35 | Married | Illiterate | Farmer |

**Key**

I = Interviewer

The numbers used are the respective codes of the participants

**Section 1: Common maternal (pregnant women, lactating women and adolescent girls) nutrition problems in the community**

**I:** What do women do to stay healthy in this community/woreda?

**01:** they undertake health checkup; during their pregnancy, they go to clinic for health checkup and they are advised to use hygienic toilet; fafa is also given for our children after measuring of MUAC. Thus we are treated in very good approach.

**02:** To keep our heathy, first we need sanitation, since the source of infection is due to the lack of sanitation; if we keep our sanitation we can protect from any disease. The women should also deliver at health center; mothers could not die while they have delivered at the health center. That is why all our wives are delivered at health center. In our time, no woman is allowed to deliver out of clinic in her home. We follow and apply as per the government’s direction/slogan; “one mother should not die while she gives life”.

**03:** If the child is required to become brilliant mind, the feeding system should be improved. Exclusive breastfeeding is critical until six months of the child. Even water should not drink until six months of the child age.

**04:** The understanding of the community towards the delivery of women at the health center becomes improved. The health awareness among the community is well established. For example pregnant women are strictly pursuing their health condition during their life of pregnancy at the health center. Free service is allowed for all pregnant women in the health center. After they deliver the child in the health center, exclusive breast feeding is recommended up to six months. The health extension workers are also advising the mothers to take rest and get help from their husbands. In soft net program, they are allowed to get rest until 10 months after delivery. But in the campaign of soil and water conservation, it is allowed to get rest for only six months after delivery. The children have got fafa from the health center.

**05.** The adolescents are young. No more attention is given for them. Sometimes they check for HIV AIDS.

**06:** There is a network in five groups and this group is not only discussed about health issue; but there are four main fronts (health, agriculture, education and good governance) and these groups are working for all sectors. In the development group, they discuss how to improve the awareness of the community in the health area, attendance student in school, agricultural development.

**04:** We are more benefited from the health center. For example my wife has delivered twins and they are now 11 months. Till six months, they never use food and water and after six months they use complementary foods like fafa from health center and we provide teff flour shake; but I don’t have milk source in my home and due to the lack of financial income I didn’t buy milk. Sometimes we give them vegetables and fruits since we have learned from the development groups and health extension workers. Now we are learning a lot from the health extension workers. But formerly, when a woman got birth she was treated culturally in her home whether she could survive or die; there was no nearby health clinic.

07: Cooperation among husband and wife becomes strengthen. The husband embraced his child while the mother makes porridge for the child. Child handling is not the task of mothers only. The pregnant women checkup in the health facility is mandatory. They always go to the clinic after four months of pregnancy.

08: It is clearly explained by others. There is large transformation in the treatment of pregnant mothers in the health facility. After delivery, balance diet food is given in the facility. Health education is also given in the schools like how to prevent HIV ADIS and unwanted pregnancy.

**I:** What are the common nutrition problems in the community for women and adolescent girls?

**04:** Since we are not educated, there is less strict follow up during pregnancy and we don’t give more focus to adolescent girls. Sanitation and getting balanced diet food is also the problem of the community. There exist, negligence of keeping their personal hygiene among the mothers.

**05:** We farmers are not educated. We don’t content our mandate; we negligence to accept what we learned. If we are called for discussion of some health issue by the health extension workers, we react not to attend; we give less credit for it. In the government side, there is scarcity of ambulance. There might encounter bleeding, thus treatment could carry through traditional fumigation at home rather than visiting to health center.

**03:** There is no goiter disease in our Tabia. It was common when we drunk unhygienic river water. But now it is totally eliminated. In case of getting rest in pregnant mother, still there is a problem. They are working until the end of pregnancy. Even after two months of delivery, we insist them to participate in different activities like weeding of crop, harvesting, and housework activities.

**01:** Night blindness in pregnant and lactating women is caused due to lack of butter for their hair. Head dryness is caused by lack of butter. The women should use butter for their hair and impatiens tinctoria (ሳሲላ) for their hands to protect disease. Even a man could not expose to disease if he uses butter for his hair.

**05:** Butter mixed with rue (ጨና ኣዳም) can protect disease like night blindness. For example we could see great practical difference between those who use butter for their hair and not use. Butter is a light itself. Based on the age of individual, vitamin A is also given in the form of pills. But it is given yearly in the form of campaign.

**06:** Health is crucial for human being. Nobody could fond if you are unhealthy. You can’t participate in any activities if not healthy. So the community has got great awareness from the health experts.

**07:** Health problem is associated with lack of sanitation. If you contact with cattle dung you could acquire disease. For example the acute vomiting and watery diarrhea is caused due to the lack of sanitation. But in our Tabia, there is no more non communicable diseases being this is rural areas.

**08:** Women are not taking alcohol except soft drinks. During women delivery, they should take Pepsi for the sake of bleeding protection. They could get relief, if they drink Pepsi during the sunny days.

**01:** In this Tabia, the common agricultural products are barely, sorghum and wheat. However, the most important food items are teff, tomato, potato, spinach, and the like. We could not produce all agricultural products at a time. We have not land to cultivate it. We are very much dependent on our family. For example we are four in our family and all are dependent in our mother’s agriculture land. We don’t have extra income.

**02:** The main problem is lack of balance diet especially during the July and August. For example my children are very thin, it is clear that they couldn’t get enough amount of food. We give half injera to each of them, this is due to scarcity of food because there is a say called “shortage is distant than sky”. As the result the children become thin and low weight. We use also less family planning.

**I:** Which women groups are most affected by these nutrition problems? Why?

**04:** In my opinion, mostly lactating mothers are sensitive to nutrition problem. Because they transfer what food consumed to their children during breastfeeding. They become thin and could develop respiratory disease and it changes into TB. They also participate in the household activities like food preparation, the overall agriculture activities. She couldn’t produce enough milk being she is exhaustively tired. At this time the child is exposing to environmental diseases. Therefore shortage of nutritious food occurred mainly in lactating mothers.

**03:** The problem could arise not only in lactating mothers but also in pregnant women; because during pregnancy, pregnant women could share food to their child inside the womb. For easily delver of the child, the pregnant women should have enough energy. Thus both the lactating and pregnant women could face similar nutrition problems.

**02:** In adolescent girls, there is poor body condition although there is no clearly observed the problem like in pregnant and lactating mothers. But they are not active in their educational status like the boys. The school termination is observed in girls. They are very busy in weeding weeds, handling child, preparing food, almost the burden is given to them.

**01:** I shared the idea of code 04. Lactating women are very busy in all household activities. For example it is easy to get milk if a cow is fully satisfied, if not, she could reject you and not treat her calf. The same is true in lactating mothers; if they are satisfied they could share to their child. In case of pregnant mothers, there is nothing to disturb them; but after four months of pregnancy, they should eat good quality and quantity of food like spinach, tomato, potato and reduce the workload. If they have energy, they will help to the doctor during delivery. If they have no energy it is difficult to get birth. At the time of delivery, referring to Maichew or Mekelle is due to shortage of quality food during pregnancy.

**02:** There is also no proper toilet and kitchen. Mothers are affecting their health by the smoke from kitchen when they prepare food. The smoke could cause trachoma. To keep the women’s health, we should think overall approach like water and environmental sanitation, supply of different food items, and health checkup in the health facilities.

**03:** Sometimes, the pregnant women get angry when they go to clinic; because the health clinic is not equipped. In some case, the health experts appointed the pregnant women for another day due to lack of enough drug in the pharmacy of the health center. Since the pregnant women came from long distance, they could not come back again; and they also think the problem exists all the time in the clinic. This is big problem in our Tabia.

**Section 2: Barriers to access and utilization of nutrition services**

**I:** What are the barriers to access and utilization of the maternal nutrition services enumerated below?

**08:** Other than the pregnant women, others that go into the health facility are after severe case of the disease. For example if a mother shows the symptom of coughing, we don’t consider as big problem and we don’t push them to go to health center. They also used to this type of habit. However, in case of pregnancy it is an obligation to go to health center after four months of pregnancy. They all have file in the clinic and checkup control is done accordingly. After they deliver the child, visiting is undertaken in their home by health extension workers to check whether they vaccinated their child three types of vaccine.

**05:** Cutup of electricity is common in the health center; if we called the electricity experts, they don’t respond us properly. You know, nowadays all activities are accomplished by electricity based technologies. As the result they don’t give any service to the mothers.

**04:** The problem in pregnant women is lack of balance diet food. They eat limited food types like injera and shiro only. Presence of high workload is also another problem; they participate in all activities like weeding, harvesting, food preparation.

Presence of less knowledgeable health experts in the health center is a problem. Example my wife was get delivered in this clinic, then the umbilicus of my child became swelling; and we brought to this clinic and referred him to go Korem then to Maichew; this is due to the lack of knowledge in the health experts. Immediately it was solved in Maichew hospital.

**02:** There is a program of pregnant women’s meeting day in this Tabia. There are three health extension experts that educate for two hours every month. They teach them how to prepare and consume of balance diet food so as not to reduce their energy, get rest, keeping sanitation, follow up to health facility and the like.

**01:** Although we have the knowledge of balance diet, the economic issue is other bottleneck. If you have not the source of animal and animal products in your home and no cash source in your pocket what are you going do? You are compelled to give the pregnant and lactating mothers only shiro and injera. You can’t fight with impossibilities. But we understand that the main food for pregnant and lactating mothers is teff by doing flour shake and porridge.

To prepare home garden in the homestead, it is easy at urban areas but in case of rural except very few individuals, nobody is benefited from home garden products. Since we have domestic animals, thus it is difficult to run both activities side by side; because we have shortage of land.

**02:** I oppose the idea of code **01.** I could say this is our weakness, if we don’t plant home garden due to the fear of browsers, we should use the animal and animal products for the pregnant and lactating mothers’ consumption. But mostly we are very much adapted to use the animal products for market purpose to cover our household expenses like coffee, sugar, schooling expenses etc.

**03:** Thanks to our government, we use hand pumping water. But it is still time consuming and we expect to introduce pipe water like the urban cities. If there is water, we can keep our environments and clothes in hygienic way.

**05:** Since our Tabia is highland, malaria is not our issue; that is why no distribution of ITN.

The available salt sold in our Tabia is also the iodized one. As it has been said before, the health extension workers and the development group are giving education to the household like how to keep healthy status of our home and environment.

**04:** Advice given by the health extension workers to lactating mothers is less compared to the pregnant once. After three months of delivery, the lactating mothers are participating in all household activities. The health expert does not give more pressure to the lactating mothers. Rather, they follow the child vaccination time. We have also a weakness to think for the mother and the child. We didn’t reduce the workload of them. Only we bother about our daily activity. This is due to our poor understanding. Therefore, we should think how to get rest and balance diet food which is very important to keep healthy to the mother and her child.

**03:** In case of adolescent girls, there is feeling of shame not competing with male adolescents, not talk freely, there is also the presence of family problem that we don’t scrape our culture like encouraging to participate in public forum equally with boys; more workload than boys.

**01:** We could say in adolescent girls, no especial treatment has been done in our Tabia. If the health experts give us advice to check up HIV ADIS, we are too much resistant to accept their recommendation; instead we considered as if the beneficiaries are the health experts. According to the rule of the government, the adolescent girls are not getting married. So we assume as if they face nothing problem. They have no more connection with health experts. The extension workers are giving more emphasis to the pregnant and to the lactating mothers.

**05:** In child treatment, the education is very strict in all the four fronts during the development group discussion. The problem is in adolescent girls, we families are also not treating them equally like male. Culturally we say like “why you eat more like male, we can’t plough you like an oxen”. So we undermine them as if they are not equal with male; and we don’t think as if they will mom tomorrow.

In case of water issue, there is no more problem in water access here; but we don’t keep our and environmental hygiene properly while there is an improvements. We don’t also practically apply the advice of health experts. Sometimes we sleep without washing of our body even we were ploughing the whole day. This is due to our negligence.

**03:** In my view, the community’s hygienic status is by far improved. There is an influence of the different movements that could force us to keep our personal hygienic conditions. We see different area exposures like visiting to Mekelle, Alamata, Maichew and other cities could make us an influence to become like them.

**04:** In my side, I didn’t hear any education about the health and feeding status of adolescent girls in our Tabia. The government does not give any attention to them. No development group and health extension workers are talking about the nutritional issue of them. We didn’t also develop any mechanism to feed our adolescent girls. We have got good lesson today and will give attention to our adolescent girls. You should also give advice to the responsible bodies like health extension workers, the Tabia leader, and the development groups.

**Section 3: Perceived needs of women for relevant services during pregnancy, lactation and adolescence**

**I:** What special things should a woman do to stay healthy during pregnancy, lactation and adolescence? Explore for pregnancy, lactation and adolescence.

**02:** To grow healthy child, the pregnant women should follow up their health status and deliver in the health facility. Since the child inside the mother get food from her, so she should get balance diet food. The balance diet foods are like fruits, energy giving food, fluids, teff flour flake.

**05:** If the pregnant women keep their personal hygiene and get balance diet food, she gives healthy child. After four months, they should follow to the health facility. In health facility, all service to the pregnant women is given freely. So she should use this opportunity and the child should get exclusive breastfeeding until six months and then complementary food.

**06:** If pregnant women get proper food, they don’t face anemia after delivery; and they don’t face any difficulty during delivery. The husbands should also support properly so as the pregnant women to get rest. We should also adjust their feeding status like teff, vegetables. If they eat diversified food types, they will have enough energy and they could deliver easily without refer to other hospitals. There is still an existing culture that without eating the husbands properly, wives are not eat; so we should encourage to eat equally with us. There is also the habit of more coffee drinking in women. It blocks their appetite and could cause anemia.

**01:** In lactating mothers, they give exclusive breastfeeding the child till six months. Thus the mothers should feed extra meal to compensate what they lost during breastfeeding. They should also share from the prepared balance diet food for children. Reducing the workload and should get rest so as to treat the child and get relief for themselves.

**03:** To grow the child properly, the mother should not leave with other bodies. The main task of the mother is treating the child. So she should get balanced diet food like flour shake, fruits, vegetables and mainly fluids. If we take the child to the agriculture field, the child could suffer by the bad weather and at the same time the mother suffers. Therefore, the husband should think about this issue, treat and help the mother in suitable way.

**02:** After my wife has delivered in the health center, she breasted her child by milking manually from her breast. As the result, my wife got rest and my child grew in good health condition. This type of approach should be introduced in other families. The mother only gives the milk without carry the child from place to place.

**07:** There are three types of vaccine that have given to the child; so during the visit time to vaccinate the child, some mothers are also has checked for their blood condition, appetite by the health experts. So postnatal checking to all mothers should be encouraged.

**05:** Once the adolescent girls get educated, they teach us the overall direction of our life. They prepare hygienic food to the family, being they are educated. They are our main helpers. Therefore, we should encourage them to get active and to participate in public activities. They should not feel shy and promote rather than to be confined in the house. Advice is very important from the family.

**I:** Do women in this community typically change their diets when they are pregnant and lactating?

**01:** There is an observation of great improvement every time. In our Tabia, we encourage mothers to feed extra food after she becomes pregnant. Relatively our awareness and our economic status are improved; and as the result we develop the knowledge how to support pregnant mothers in their feeding condition so as to deliver healthy and brilliant child. But at the beginning of their pregnancy, they reduce their food consumption level. Their appetite improves after they go to the health center at four month. They commonly adapted that they don’t go to health center before four months even they don’t feel comfort.

**05:** Now the government has given good awareness to the community. In the formerly, we didn’t care about the treatment of pregnant mothers and child; even after they had got pregnant they breastfeed the child and the child became ill; when they took to clinic and the cause of the child illness was consumption of colostrum. But now pregnancy is easily detected in the clinic. When they take their child to the health facility, they could get the chance of taking checkup for themselves.

**06:** After pregnancy, feeding is changed. At the beginning, they hate the common food that she was feeding it; instead she consumes green paper and teff flour flake. But if we prepare diversified food, they could get an alternative to select what they like unless they totally reduce their consumption level during pregnancy. Symptoms like nausea, spitting saliva, long uncomfortable sleeping is common during pregnancy.

**03:** In case of lactating mothers, extra food is very important. They get advice from the health extension workers what they could feed after delivery like porridge, teff flour flake, fruits, vegetables. We understand that lactating mothers should eat more than pregnant one. But in the rural area, we have only the access of barely, sorghum and wheat. There is no teff, and other fruits and vegetables.

**I:** What foods are recommended for pregnant women? What about breastfeeding women?

**04:** For the pregnant women, it is recommended to eat teff flour flake. For lactating mothers, it is good to feed flour flakes, milk, porridge, pumpkin, and hot fluid in order to stop bleeding and it is not recommended to eat injera; because it has no any contribution to stop bleeding and they have no any appetite to eat injera at that time. Since the lactating women has lost flesh from their body during their delivery, it could be nice if you slaughter sheep or goat to compensate the lost flesh of their. But our economy limits to get meat. Then again milk is mandatory either from your own milk or by begging from the neighbours. Sharing milk for lactating mothers from those who have milk is common culture in this area.

**03:** Although the government has banned the culture of fumigation due to the problem of deforestation, it was very important for both the mother and the child to easygoing of her body. Butter for their hair is also very important to strengthen the back of mothers.

**02:** I don’t have any different idea from code 03. Both the pregnant and the lactating women are shared what they feed to their child, we advise to feed three types of food like milk, flour flake and vegetables. We tell them to feed diversified food although it is not applied practically due to economic problems.

**I:** What foods do pregnant women avoid? What about breastfeeding women?

01: In our Tabia, everything is edible; except there is ሑሞድያ at the field, it is totally forbidden to go pregnant women to that area; because the ሑሞድያ is very danger to the fetus that causes abortion.

**02:** For lactating mothers, alcohol is prohibited to drink except soft drinks. Chat is also not allowed to chew. For example, I am chewing chat but not my wife; because it could affect her and her child.

**04:** I have no additional idea, in our culture only we know ሑሞድያ is danger for pregnant women which cause abortion. But others like soft food are recommended being it is easily swallowed and digested than injera, bread and any other dry food items; this is the interest of the women not culture. Fluid substance is more encouraged to drink for both the pregnant and lactating mothers. Even sugar cane is more recommended for lactating mothers to produce more milk to the child. And there is no any food item that consider as taboo to pregnant and lactating mothers.

**03:** I heard nothing about food types that are not allowed to eat during pregnant and lactating mothers. Only we know that ሑሞድያ which created from the crops is bad for pregnant women. If she goes today, at the next day abortion occurred. It is also bad for the crops.

**06:** I didn’t hear about food taboo.

**Section 4: Other interventions that improve pregnant, lactating and adolescent nutrition**

I: Have you ever gone for nutrition screening during community health days or routine service delivery? If you went, what was your experience? If you didn’t go, why not?

**07:** Yes; the pregnant and lactating mothers went for nutrition screening which was conducted during their checkup time like checked for their weight; they also checked the hands of children if they are thick or thin. If they are below the standard, they get the supplementary flour (fafa) and improve their body conditions. But this nutrition screening is routine service delivery in the clinic.

**03:** Pregnant and lactating women and children are participating in the nutrition screening but not adolescent girls. We don’t know the reason; nothing is raised by the health extension workers about the nutritional status of adolescent girls.

**05:** Our children are mainly benefited from this program.

**I:** Are pregnant and lactating women beneficiaries of the soft conditionality of the productive safety net program, PSNP?

**08:** Yes, more focus is given to the pregnant and lactating women; because the government thinks that they are the most vulnerable groups that need help. In order not suffer the child, and to get rest the mother, it is allowed to pregnant and lactating mothers get benefit from the soft conditionality of the productive safety net program without participating in the work.

**04:** It is declared legally from the woreda level that pregnant and lactating mothers are allowed to get benefit from the program without get involved in the work. Pregnant women should not participate in the work after six months even they are engaged in the program. And for lactating women, they are allowed to take rest for one year even they are engaged in the program.

**03:** This is clearly understood and a good lesson for us how much the government has great focus to pregnant and lactating mothers. So we learned a lot from the health extension workers as we should keep their health status so as to deliver a child with bright mind.

But in case of soil and water conservation, the rule is different; they forced the lactating mother to work after baptizing of their child. And the pregnant women are participating in the soil and water conservation unless they take letter from the health experts.

**01:** Being the pregnant and lactating mothers are engaged the soft conditionality of the productive safety net program without work, they are very much happy for this favour. From this program they are benefitting in two ways: one additional income and the other is a lesson for other sectors how much rest is important for pregnant and lactating mothers.

**Section 5: Understanding perceptions of age at first birth and birth spacing**

**I:** Do you think delaying the age at first birth to after 18 is better for the health of both the mother and the baby and is it promoted in the community?

**01:** Yes, currently there is clear rule that adolescent girls are not get married below 18 years. And the community totally owned as a culture. If somebody undertook under age marriage, everybody inform to the legal body and punishment is expected.

**04:** It is an obligation to have legal marriage certificate. For example we all have marriage certificate; so it is totally ban of less than 18 years marriage. This is very important for the girls’ organ not to hurt. It was first promoted by woreda lawyer and the community owns it as a culture; so everybody strictly follows if the case is happened. If under 18 years marriage is undertaken, they will take into prison. Nobody could also attend in their wedding ceremony. Some are punished as the result of less than 18 years marriage. Now it is known by everybody. Marriage certificate is also mandatory to get the groups; so controlling system is very tight.

**02:** The elders are also very supporting to this rule. Nobody could marry without accompanying of health examination.

**05:** If under age marriage is conducted, it could hurt the girls’ organ; they could also affect their mind. Nowadays, without having the consent of the girls’ interest, a family couldn’t force them to marry. We are appreciating this rule and all we in collaboration of the development groups support and encourage the strict law of banning less than 18 years marriage.

**I:** You may have heard that it is better for both mothers and babies if women wait at least 2 years after birth to become pregnant again. Do you think this message is being promoted in the community? If yes, can you tell us more about who is promoting this message?

**08:** Yes, family planning is very important; if enough space among consecutive birth interval is present; it is essential for the child and the mothers. For example if it is after four years of the first child then child becomes large and help you in treating the next child. You can also reduce your economic burden.

**01:** Mostly the birth interval among consecutive children becomes increase. Almost all the women are beneficiary of family planning. All the religious bodies are also supporters of this rule. Since it was planned for the sake of our benefit, we should apply practically in the ground accordingly.

**04:** Both less than 18 years marriage and space of the birth interval is popularized in the community and it is more practically applied in every household. Elders are also supporters of this rule. This program is given in the school. All the adolescence girls aware of this issue very well, so they are not voluntary to undertake early marriage.

**06:** About the family planning, all the responsible bodies through the channel from the individual to the development group, to the health extension workers and to health experts are discussing throughout the time. So awareness is created among the community; they also sense its importance like to grow the child in proper way.

**03:** We have clear line of task sharing among the responsible stakeholders and not difficult to apply practically at the ground. The community is very active to implement what is given any assignment by the government.

**Section 6: Understanding communication and information sources**

I: What kinds of community conversations or messages discuss women’s and adolescents nutrition in this community?

**01:** Communication among the family, how to develop the feeding status of the family; what special food type is important for pregnant and lactating mothers; how could get more rest than other family; how to develop our economic status; how the information gained from health experts and how it is practically applied at the ground; is communicated through the development group and the health extension workers.

**02:** The information how to help and handle pregnant and lactating mothers is gained from the health extension workers; so practical application is the important one; the husbands should support their wives and should have task share even inside the home activities.

**04:** There is women development army; development group; health extension worker; and these groups are working in collaboration to improve families’ health status.

**Section 7: Additional remarks**

**I:** How can we improve maternal and adolescent nutrition in this community?

**05:** Husband should take the lead and encourage pregnant and lactating mothers to take rest, balance diet food and give attention to adolescent girls

**03:** The health facility should be equipped so as to get full service without referring the women to other hospitals. Full drug, discontinuation of electricity is very critical.

**SUMMARY**

**Section 1: Common maternal (pregnant women, lactating women and adolescent girls) nutrition problems in the community**

- There is scarcity of ambulance. Bleeding could encounter due to late arrival of health centre, thus treatment could undertake like the traditional fumigation at home.
- Even after two months of delivery, we insist lactating mothers to participate in different activities like weeding of crop, harvesting, and housework activities. Their workload is high.
- Night blindness in pregnant and lactating women is caused due to lack of butter for their hair. Head dryness is caused by lack of butter. The women should use butter for their hair and impatiens tinctoria (ሳሲላ) for their hands to protect disease like trachoma.
- Lack of enough drugs in the pharmacy of the health center. Since the pregnant women come from long distance and if they face the problem, they could not come back again; and they also think the problem exists all the time in the clinic.

**Section 2: Barriers to access and utilization of nutrition services**

- Less awareness among the community and the presence of less knowledgeable health experts in the health center.
- There is the habit of more coffee drinking in women. It blocks their appetite and could cause anemia.

**Section 3: Perceived needs of women for relevant services during pregnancy, lactation and adolescence**

- They keep their personal hygiene and get balance diet foods like flour shake, fruits, vegetables and mainly fluids. If we take the child to the agriculture field, the child could suffer by bad weather and at the same time the mother suffers. Therefore, the husband should treat and help the mother in suitable way.
- Although it was banned the women fumigation at home due to the problem of deforestation, it was very important for both the mother and the child to easygoing of their body. Butter for their hair is also very important to strengthen the back of mothers.
- Pregnant women are not allowed to go to the agriculture field if there is ሑሞድያ; because the ሑሞድያ is very danger to the fetus that causes abortion.

**Section 4: Other interventions that improve pregnant, lactating and adolescent nutrition**

- The pregnant and lactating mothers are allowed to get benefit from the soft conditionality of the productive safety net program, without getting involved in the work.

**Section 5: Understanding perceptions of age at first birth and birth spacing**

- Early marriage is banned and it is taken as a culture by the community. If under 18 years marriage is undertaken, they will take into prison; nobody could also attend in their wedding ceremony.
- If women gives birth before the age of 18 years, they will be affected their organ which lead to fistula; and also the new born will not get adequate care from them.
- If there is birth spacing among the child, the first child becomes large and helps the mother in treating the next child.

**Section 6: Understanding communication and information sources**

- The information how to help and handle pregnant and lactating mothers is gained from the women development army, health extension workers

**Section 7: Additional remarks**

- The health facility should be equipped such as availability of full drug, discontinuation of electricity is very important.
