# Supplementary material for: Barriers to utilize nutrition interventions among lactating women in rural communities of Tigray, northern Ethiopia: An exploratory study
Source: PLoS One. 2021 Apr 30;16(4):e0250696. doi: 10.1371/journal.pone.0250696 (PMC8087028; doi:10.1371/journal.pone.0250696)
Supplement: S2 File — (ZIP) [file pone.0250696.s002.zip › S2_File.Doc/Community level Key informants/032_IDA-DA_AdishumBereket_Ofla wordea.docx]

**In-depth interview guide for with experts** using the Guide for Nutrition focal persons (**Tool A**)

**Introduction:**

Hello my name is Haftay. I am from Mekelle University. Thank you for taking time to speak with us today. We are doing a research on factors that influence the nutrition of mothers and adolescent girls in collaboration with the regional health bureau and UNICEF. Your participation is very valuable. The things that you tell us will be used to improve nutrition programs and services for women and adolescent in the region and in the country. Your names will not share when we report our results.

However, I will record the discussion using and audio tape recorder so that we can capture all the ideas that are shared. I have several questions to ask you that we have prepared in advance, and we will ask you to say what you think about each question. To ensure the privacy of everyone here, we ask you not to repeat what to discuss outside of this group. The discussion will last for 1-2 hours. Do you have any question before I begin? If you think of any question as we proceed, please feel free to let me know. If it is all right with all of you, I will run on the tape record now.

**Section A: Interview details**

1. **Zone**: Southern
2. **Woreda**: Ofla
3. **Kebele**: Adi shembereket
4. **Name of key Informant**: Asefa
5. **Institution of key informant**: Developmental association (Agricultural extension worker)
6. **Interviewer’s name**: Haftay Berhane
7. **Date of interview**: 06/11/2017
8. **Interview starting time**: 10:30 AM
9. **Interview end time**: 11:00 AM

**Section B: interviewee professional information**

1. **Gender**: Male
2. **Age**: 24 years
3. **Highest level of completed education:** College education
4. **Current job/Position:** Developmental association expert
5. **How long have you been in the current Job/position:** 2 years and 10 months

**Key:**

**I**: Interview

**P**: Participant

**Section 1: Common maternal (pregnant and lactating women), and adolescent girls` nutrition problems in the community.**

**I**: What do you think women should do to stay health?

**P**: If a mother is to be healthy, one; she should eat food that are good in nutrition containing different varieties for one thousand days with her child as the health workers always say it. The second one is, if she is living a stable life.

**I**: Let`s see these separately. What should a pregnant woman do to stay healthy?

**P**: The pregnant women should not be stressed and secondly, they should eat balanced diet and the diet should be ready based on their condition and need. Thirdly, they should not be engaged in heavy activities. However, they should not also stay immobile.

**I**: What about a lactating mother? What should a lactating woman do to stay healthy?

**P**: anyways, a lactating mother should receive milk, secondly, she should also take vegetables and crops. She should also visit health institutions for check-up for hypertension and so on. A lactating mother needs many things including rest and she should not engaged in activities just after birth. She should also take rest for about two months and take foods like porridge, hot soup made from teff and other cereals.

**I**: What about an adolescent with age range of 10-19 years? What do the adolescents do to stay healthy in your wereda?

**P**: Adolescents in the age of ten to nineteen needs food like children and adults. They should also work freely without any stress. The other thing is they need follow and support as they are in the fire age to live a stable life.

I: Well. Did you think the above-mentioned action are done in your wereda in women both lactating and breast feeding and adolescent girls?

**P**: Most of the above-mentioned activities are not on the ground. One; is lack of awareness by the community, second is since the community is less educated most of the activities mentioned are not done. I was telling you what the science tells but there is low implementation towards the activities mentioned.

**I**: In your opinion, do you think there are nutritional problems in this community especially in pregnant and lactating women and adolescent girls? Which problems are common in your area?

**P**: When we see nutritional problems in mothers, one; there is early death, the children born from these mothers will have abnormalities to mean those that have no normal body weight, secondly; in these adolescent girls of age 12-19 years there are many problems. These girls are engaged in work earlier than normal, since the community supports them to let them enjoy they tend to work and became very tired. Some of them leave the wereda because of work load. In mothers mostly one because anemia, and the other is there are lactating mothers who have swollen body because of shortage of food. I can explain the idea with these things.

**I**: In your opinion, how do you explain the risk of malnutrition for pregnant women?

**P**: The population high in this wereda. Those living in nearby town are somewhat better than those who live in rural areas. The percentage of malnutrition in pregnant women compared to the total population in our wereda is more than 50%. These women are at risk for malnutrition. This is because pregnant women in these community are being engaged in farm activities like weeding, harvesting, digging. Therefore, the problem is huge in pregnant women and it may account around 70%.

**I**: What about those lactating women and adolescents? In your opinion, did you think lactating women and adolescent girls are at risk of malnutrition?

**P**: If you see people living in this community take food items that can only satisfy their hunger. There is little awareness on the advantage of eating different food items. The risk of malnutrition in adolescent is lower than pregnant and lactating mother. This is because the adolescents are single and nobody is sharing then unlike the pregnant and lactating mothers. The prevalence malnutrition in adolescent could be as low as 20-30%.

I: Is there food aid in this community? Is there food support like fafa?

P: Yes, there is food aid given to households. However, it is difficult to predict how much the aid can reach the women and adolescent girls. It is very low. If you select an adolescent who needs food support or aid, you will get another adolescent who poorer than the first selected. The principle of the food support is to help the worst among the poor.

**I**: Who are eligible for fafa? To whom did you give this food aid?

**P**: This food aid is given to pregnant women and children. There are also mothers wo are eligible to take this support based on the condition they have. All pregnant women can get this support but all lactating mothers are not eligible for the food support.

I: What about PlumpNet? To whom do you think plumpNet is given?

P: PlumpNet is given to children who are underweight. However, there are many problems on the usage of the PlumpNet. When this food supplement is given to the child, the family members share this food to another child who is not eligible for the supplement. PulmpNet is not given to pregnant women as the health professionals said it is not allowed to be taken but lactating mother can take this supplement. The other problem is the supply issue. There is shortage of supply of PlumpNet. Therefore, there should be awareness creation to the community on the utilization of such supplements so as to prevent the shortage supply.

**I**: How common are the micronutrient deficiencies like anemia, night blindness and goitre in this community especially in women and adolescent girls?

**P**: Yes, they are common. For example, if you see night blindness it is commonly seen in children. Goitre is also common in females which is also assumed to be transmitted to men.

**I**: Who do you think is mostly affected by the micronutrient deficiency like anemia, night blindness and goitre? Do you think that women or adolescent girls in this community are suffering from micronutrient deficiencies like anemia, night blindness and goitre?

**P**: Goitre is mostly seen in women whether pregnant or lactating. Night blindness is mostly seen in children but anemia can be seen in any body be it male or female, or young or elder. Anemia is mostly seen in lactating women however, it could also be seen in pregnant women.

**I**: What do you think are the causes for these diseases? In your opinion, what could be the cause of these deficiencies like anemia, night blindness and goitre?

**P**: The cause of all these diseases is lack of balanced diet. In the balanced diet, night blindness is causes when a person is not eating vegetables and fruits that contain vitamin A like carrot and crops like teff. Goitre can also be caused by eating foods that does not contain iodine. It also depends on what you eat and the water you drink.

**I**: Is there any activity done prevent the occurrence of these deficiencies?

**P**: Yes. The agricultural sector doing to prevent the occurrence of these diseases in the community. The health sector is also doing activities on prevention of these diseases. We, agriculture, are doing in collaboration with the health sector. There is a team that works on nutrition which is composed of experts from agriculture and health which focuses on mothers and children. We are giving a training to mothers on how to prepare a food from carrot, potatoes and cabbage with the material for preparation but it is very low. It can be estimated that the training is given to only 10% of the mothers we have in this wereda. The supply from the government is also low. We explain it by this. We are educating mother on the prenatal and postnatal follow up on how to prevent night blindness. We are also showing them how to make a porridge pratctically. It is not only how to prepare but we are also training them what should a flour for porridge contains.

**I**: Do you think there could be any association between nutrition and occurrence of non-communicable diseases like hypertension and diabetes mellitus among women and girls?

**P**: I do not think there could be an association between nutrition and the occurrence of non-communicable diseases.

**I**: Let`s take some examples like diabetes and hypertension. Did you think diabetes can be linked with foods we eat like sugar or Hypertension with salt? How do you think so?

**P**: In most of the time diabetes is not caused by the consumption of sugar. It is caused by the ability of the body to resist thing. Otherwise it does not be caused by consumption of sugar.

**I**: Well. How did you think the non-communicable diseases are caused? Are non-communicable diseases common here?

**P**: Here there is no cases of hypertension however, there are cases with diabetes mellitus. In most cases, diabetes mellitus is caused by extensive work and stress. For example, I have seen in the previous time a child of age 19 years with diabetes mellitus in one of the kebeles in this wereda. If you see the cause of diabetes in this girl, it will not be the consumption of sugar because how will sugar be available in rural area. But the nature of the work will it lead the girls to diabetes. It is not linked to the problem of feeding.

**I**: How do you think the weight of women or girls in this community?

**P**: There are girls who are below the normal weight. There are many underweight girls. Even if this is done in collaboration with the health professionals, there many cases of women and girls who have low weight than the normal.

**I**: A weight of an individual may be overweight, normal or underweight. In which category would you put the women and adolescent girls?

**P**: In most the cases there nobody considered as overweight. Around 30% of women and girls are with normal weight however most of them which 60-70% of them are underweight.

**I**: Could we link the case underweight with nutrition?

**P**: Yes. It is directly related. If someone is underweighted means he/she is not getting foods with good nutrients. It is not only the fullness the stomach that matters. The important thing should be considered is not only the size we eat but should also bother about the content. A small bread may contain many nutrients than a large bread. The feeding habit is related with income.

**I**: Is it common to see individuals with overweight?

**P**: No. The prevalence of overweight is low which may be 2-3 percent.

**I**: Could we link the overweight with nutrition?

**P**: I do not think. If someone is taking good nutrient containing food, there is less likely to be overweighed. Therefore, overweight is not the result of over eating; it could be related with internal problem.

**I**: Is there a situation when the community suffers from food insecurity? Do farmers of this wereda harvest cereals that is enough to feed them for one year?

**P**: There is no as such appreciable number of farmers who produce farm products that can feed their family for one year. For example, farmers in this kebele are producing vegetables and fruits. However, these products will not rich to women and they used to sell it. Many farmers produce vegetables and they have enough money from the sale of these vegetables but this will not reach to Mothers and children immediately. The money will be lost just outside home either they drink it or lost it in other activities. I can say there is food insecurity as they cannot feed themselves for one year.

**I**: Are there food aids and programs like safety net?

**P**: Yes. There is food aid every year but the base is the quota we received from wereda. Based on the quota, we select the poorest of the poor. The benefits from safety net is also similar. But, apart from this in safety net program, we may go up to third stage of poverty. Therefore, in agriculture of productive safety net program, most of the farmers may not get benefited from the program.

**I**: What could be the proportion of the poorest of the poorest from the total population?

**P**: Around thirty five percent of farmers are benefited from the safety net program.

**I**: How frequent do you think food insecurity would happen and what are the main reasons for food insecurity; low productivity, small plot of land, or shortage of rainfall?

**P**: It is not about the size of the plot of land for farming instead, it is about production of food items with good nutrients. For example, in places with small plot of land, they think of production of carrot, cabbage, vegetables and crops. They can also think of potato and sweet potato. Therefore, the main reason is related with awareness. The farmers produce and take it to markets instead of using it. If we see, many farmers harvest farm products that may be sufficient only for about 10 months. The shortage of food is mostly common in the months of July to October and these months are moths of hunger.

**I**: Therefore, what could be the reason for the shortage of food in this case? Is it related to low productivity, small plot of land, or shortage of rainfall?

**P**: The shortage food could be linked to many things. One is the environmental condition which the rain. Now days, the rain is not consistent; sometimes it starts in Mid-July and stops on half of August or beginning of September and sometimes it starts on June and stops on Mid-August. The second one is related to agricultural sector. This is to mean that farmers are harvesting products that are not suitable for the land plot. Third is inability of using modified farm products that can grow fast. These are some of the reasons.

**I**: In your opinion, how do you think women and girls suffer from this food insecurity?

P: We have only few farmers that have food security. Around 60% of females are farmers. Therefore, females are highly affected.

I: If a household cannot secure their food that is sufficient for one year, what would be the impact of this food insecurity in the female when compared to the who household?

**P**: The impact is higher in females. If you consider a household with mother, father and children, mostly mothers are highly affected followed by child and then the father. This is because most father have the practice of eating pout side their home. For example, if they take beer, it has carbohydrate and the father getting food than the mother and the child.

**I**: Which of the above-mentioned problems do you think pregnant women are specifically at risk?

**P**: The main problem is malnutrition. The deficiencies like goitre, night blindness and anemia are the result of the malnutrition. If malnutrition is solved, the diseases like night blindness will disappear. Therefore, the main problem that affects females is malnutrition.

**Section 2: Nutrition priorities in the wereda**

**I:** In your opinion, what interventions do you think are the priorities of your institution as agricultural extension worker to improve nutrition for pregnant women?

**P**: There many interventions the agricultural sector can do. For example, in our kebele there is practical porridge preparation, diversification of the component of the powder used for porridge, vegetable home gardening. In vegetable home gardening we show them how to be done by ourselves. We also follow by visiting home to home to check how the farmers are applying the training we gave them. There is a follow up file in the agricultural activities. We train the farmers and inform them what the mothers and children can eat. We clearly train them what should a child eat in one thousand days. For example, in the past three days we have trained farmers on this issue.

**I**: Well. Do you think there is activities done by your institution targeting the pregnant women?

**P**: Yes, but it is lower than expected. We have done some activities for trial so that the farmers can own it apply it. In collaboration with nutrition team, the agricultural bureau is providing women with carrot, spinach, cabbage and sugar potato to cultivate them at home.

**I**: What interventions did you think your institution do targeting the lactating women?

**P**: It is the same with the pregnant women. There no different intervention done for lactating women except distribution of the above home gardens.

**I**: If there is any intervention done by your institution to improve nutrition of adolescents?

**P**: No. There is nothing done to adolescents. We mostly focus to mothers and children. We are giving training to mothers but nothing is done on the adolescent girls.

**I**: You have told me that your sector is focusing mothers and children. What do you think your sector should do to improve the nutrition of mothers and adolescent girls? Do you think it is necessary for your sector to get involved in work aimed at improving nutrition among women and adolescent?

**P**: If there are 300 pregnant women, we were supporting 30 of them. Two hundred seventy of them were not receiving the support. The adolescents are totally neglected. There should be follow up. There should be training, follow up and support to all pregnant women and there should also be experience sharing.

**I**: Did you think the nutrition interventions done by your sector for women and children are successful?

**P**: Yes. It is successful. For example, A woman in one village was exercising the home after we trained her. Therefore, there is improvement.

**I**: When you trained the woman, how their husbands are accepting the intervention?

**P**: If there is support and follow up, there will not be any problem. If you teach the farmer based on the scientific merit, there is good acceptance even after long time. All farmers are positive to help their wives if there is shortage of food.

**I**: How do you evaluate the success of your sector in improving the maternal and adolescent girls` nutrition in this wereda?

**Section 3: Nutrition interventions that improve adolescent and maternal nutrition**

**I**: Do pregnant women advised to visit health facilities for check-up and services?

**P**: This is done by the health sector. After weighing and screening, they advise them to take what they should took.

**I**: Do you think pregnant women receive advice on the need to get extra meal?

P: There is a manual that dictates how should a pregnant and lactating mother take their meal. For example, a pregnant woman should eat sufficient breakfast, lunch, extra meal on the evening if there is and dinner. It is not possible but it says. If you eat your lunch, you will meet at dinner time. For pregnant women we properly advise them what should they use and when but practically they do not apply it. It is difficult to the woman to eat alone while the father and children are outside. She will not also apply to what the science said. The main reason is shortage and not accepting what has been said and focusing on the daily work.

**I**: What about lactating

**P**: lactating mothers should take food items that can benefit them and can produce milk. But these mothers are not applying it one because of the lack of awareness and secondly because of shortage.

**I**: You have told me that mothers and children measure their weight by going to health facilities. Do the health institutions link with you if they are underweight?

**P**: We did not measure weight of mothers. The health professionals measure the weight of mothers and send a report to wereda. After they report, the wereda will send food support enough to mothers to health institutions. The complementary foods like fafa are not sent to agriculture while raw cereals can do.

**I**: Do you think the food support/aid considers the presence of pregnant or lactating or adolescent girl in a house hold?

**P**: No. When we distribute a food aid, we only consider the number of family members in a household. We do not consider whether they have women or adolescent girl. We do not consider the number of adolescent girls or whether the mother is pregnant or lactating.

**I**: Are lactating women getting advice about the nutrition sensitive agriculture such as home gardening?

**P**: Yes. We are giving training based on the program set by the government. There is a program when to train pregnant and what a lactating woman can eat. What a lactating woman should eat is one porridge, second is milk, third if there is barely flour shake. They should also take soup made from teff and wheat `Wefche` which can prevent anemia. Therefore, we are training them properly what the component of the diversification of a flour is.

I: Do you think all the component of a flour for porridge can grow here?

P: Yes. All are from the local. They can grow here.

I: What are the barriers that prevent you not to implement the nutrition sensitive agriculture such as home gardening?

P: The main reason not to implement this nutrition sensitive agriculture is one famers do not prioritize mothers. When you tell the farmer to garden for his wife a plot of farm for potato, this one for carrot, this for sweet potato and this cabbage, the farmer will allow. The farmer may say `I must produce a pepper for market instead`. There is also unfairness in the household. Nobody will listen the idea of the mothers. There is no respect to mothers. The father will dominate than his wife and he will not accept whatever golden idea is raised by the women.

I: How is the safety net program involved pregnant and lactating mother and adolescent girls?

P: In our wereda, if a mother is pregnant or lactating she will be benefited from the program without the involvement in the work. If a mother is known to be pregnant of three months or if she brought a result for confirmation of pregnancy, she will be given a 12 months rest before birth and 12 months rest while lactating with all the benefits of the safety net program.

**I**: What about adolescent girls?

**P**: No. The program does not give emphasis to adolescents.

**I**: Are women getting advice on personal hygiene, sanitation?

**P**: Yes. They always be advised but practically only few will apply it. When the woman is advised to keep her home hygiene, it may be difficult for her to do it because the home is narrow, she must move home materials from one side to other side and so on. Therefore, there is a problem on applying sanitation and hygiene. Even if the resource is available for example, water, they do not use it.

**I**: Why are they not using if resources like water are present?

**P**: The problem is personal problem. Lack of awareness could contribute. They may need support and follow up.

**I**: Do mothers get soap and other materials used for sanitation?

P: We do not give them soap but there is water guard and bishan gar for water treatment. There is a training on when a mother should wash her hands; before preparation of food, after cleaning their child, after visiting the latrine but there is no supply of soaps.

**I**: Do you think women apply the training given on sanitation and hygiene practices?

**P**: They do not apply it. Most of them did not wash their hands just after cleaning their child. I am saying this from my observation.

**I**: Are pregnant women getting advice on the need to use insecticidal treated bed nets?

**P**: They are getting advice but they are not applying it. For example, in one village the bed net was using for preventing Geleba from pests. They may make it different materials that will use them for the farming. The government is getting difficulty in those materials given for free. The farmers consider materials given for free are costless and with lower quality. For example, when insecticidal treated bed net is distributed for the households, the farmers consider that government is giving us because it is useless for the government. Therefore, there is a huge gap in the farmers.

**I**: Who is advising the mothers especially pregnant and lactating mothers to use the bed nets?

P: There is interface that is formed from agriculture and health. These interfaces will take a lead to teach them. For example, if a house is having five family members, they receive five bed nets. Just to use independently but they do not use it.

**I**: If the bed net is treated with insecticide, do you give them not to wash it? How is the supply of insecticidal treated bed nets?

**P**: The supply here is low. For example, I have seen distribution of the nets once in three years. I am not sure about washing practice of these nets. ..laughing

**I**: Are pregnant women getting deworming services? Have you ever seen a pregnant mother taking the drug called Albendazole?

**P**: No. I am not sure about this.

**I**: Is there a situation, which a pregnant woman to be eligible for targeted supplementary feeding?

**P**: No. There is no.

**I**: What about lactating mothers or adolescents.

**P**: When a mother is giving birth at health institution, there is a flour contributed from mothers for one to two-time porridge. Otherwise, there is no other targeted supplementary feeding for pregnant, lactating and adolescent girl.

**I**: Are lactating women getting vitamin A supplementation after she they give birth?

P: They are getting vitamin A supplementation but it is given in the health institutions.

I: What about for adolescent girls?

P: I am not sure.

**I**: What do you think the advantage of vitamin A supplementation?

**P**: Vitamin can increase the immunity of the mother, it will also help in growth of the child. It will also prevent night blindness. So, it good for their health.

I: Are adolescent girls provided school feeding in this wereda?

P: No

I: Is there school in this kebele and do students have school feeding?

P: Yes. There is school. There was feeding in the schools in the form of porridge. However, the schools included in the program are few. Schools in dinka and dara are included. Here, only school is included.

**I**: Why is this feeding program given to these schools? Why do you think the importance of feeding in schools?

**P**: The program is initiated to halt the shortage of food by safety children. The food contains all nutrients. It is there in the form of porridge, and ground nut.

**I**: Is this program targeting to adolescent girls or just is given to all students?

**P**: It is targeted. It is given to students in the school.

**I**: In your opinion, do you think this school feeding is important to out-school adolescents?

**P**: Nutrition is not only important for students. It is also important for others which are out of school. If those out school adolescents are taking enough nutrition, they may change their thinking and join or start schools. It will also help to undertake other activities outside school efficiently.

**I**: In your opinion, are adolescent girls linked to youth friendly services at health facilities?

P: The youth friendly service is there. But they are not utilizing it. In fact, is very important for them but they are not utilizing it.

I: Why do you think it is necessary?

**P**: It us know that our country is in development. If we are thinking to sustain our development, we must decrease the prevalence of HIV. The second one, is girls should keep their menstrual hygiene by themselves to prevent infection and contamination. This will also help them to prevent unplanned pregnancy. The third is, it will help them to meet their future goals.

**I**: In your opinion, which of the above interventions for the pregnant women like Vitamin A supplementation, target supplementary feeding, providing complementary foods like fafa, safety net program are being implemented in an effective way?

**P**: If we can programs like safety net, they are not women focused programs. Their focus is household. Most programs are not targeting women and we should work here.

**I**: Then, which intervention do you think is implemented effectively?

**P**: The most effective is safety net. In one hand, it will benefit pregnant and lactating women while they are at rest. There is also a training given to them on how to prepare their food. There is even a separate place devoted to mothers and children only. There is a chance for these women to enjoy and feel free from stress. I cannot say other interventions are more effective.

**I**: What about with the adolescents? Which intervention do you think is implemented effectively?

**P**: When we see adolescents in school and out school, the adolescent girls in school are more better. Adolescents in school know what they do even if the poverty is there. Their thinking is better than the out school adolescent girls.

**I**: In your opinion, which of the above interventions mentioned do you think is less effectively implemented?

**P**: Interventions that should be implemented but still not yet effective include the agriculture sensitive home gardening. The number one source of should be home. This home gardening may contribute much in preventing malnutrition. School feeding should also be inclusive to all schools. For example, students in the school near by this kebel are benefited from school feeding program however, students from other schools are not. The out-school adolescents should also be included in training and they should also be targeted with food aids.

I: What are the challenges to implement delivering the nutrition interventions that we have been discussing for women and adolescent girls?

**P**: The first challenge is our poverty. The second challenge is there is less number of literate people. The lack of enough resource is also a challenge.

**I**: What is the problem with the resource? How do we evaluate it?

**P**: There should be supply of the vegetables focusing women and children.

**I**: Who do you think should supply these vegetables? The government?

**P**: It is preferably good to capacitate the individuals to be self-sufficient. There should be awareness creation to the public first. It is good to train the public and enable to be self-sufficient and sustain the food security. The number one that should be capacitated is the farmer. The farmer should be trained. It is also good to support to those with disabilities and elders.

**I**: Do you think mothers know about the effect of malnutrition? The effect on themselves and their children?

P: Yes. The effect of malnutrition is clear to everybody. If a mother did not take her dinner, she knows what will happen to herself and her child. There are many mothers who have concerns on the effect of not taking food properly.

I: What about the adolescents? If they are not taking their food properly, would they think the long-term effect of malnutrition on their children?

P: Yes. There is a problem. They cannot forecast what will come later.

**I**: How could it be solved?

**P**: There should be education.

**I**: Who should provide the education?

**P**: It is good if everybody is aware of any thing. The education should be given by induvial who are nearby to the farmers. It could be given by agricultural sector or health or school. The main thing is the farmer should know it.

**I**: In your opinion, how do you evaluate the collaborative work between agriculture and health to halt the nutritional problems?

P: It is good. We have recently started working together. Since our start, it is going good. We are also using budget from the budget we have in supporting women. But it is not sufficient.

**I**: How do you evaluate the whole structure towards addressing the nutritional problems? Does it have

**P**: Nutrition have its own committee. It is led by kebele administrator and rural agricultural development officer will be secretary. Rural agriculture experts and nurses are members of this committee.

**I**: How do you think this committee doing?

**P**: Last time it has been evaluated as poor but currently it is going good. By educating you may not get immediate result but its long effect is good. The improvement is slow but if you educate one mother by today and two mothers tomorrow, they also help you in teaching the others.

I: Are there barriers that hinder the sector collaboration not to bring effective results quickly?

P: Yes. One thing is there is lack of well-educated professional. The support and follow up is very low. If you see in agricultural sector, we have different other activities. Our focus may not reach immediately to mothers and children.

**Section 4: community factors affecting access to maternal nutrition interventions**

**I**: in your opinion, what are the barriers you think are preventing pregnant mothers from utilizing the interventions that we have discussed above like Vitamin A supplementation, target supplementary feeding, providing complementary foods like fafa, safety net program? Lets` start with pregnant, lactating and then adolescent girl?

**P**: We already mentioned it earlier but there are many barriers. The first barrier is lack of awareness. Mothers are not given priorities, not considering the foetus they carry and lack of resource for example foods that are suitable for pregnant women. The other is inability to think as pregnant women need special food that contains good nutrients.

**I**: Do you think that there is connection between accesses of transport and utilization of these services?

**P**: Yes. Here the infrastructure is poor. The road is not suitable for car transport.

**I**: How do you explain the quality of service delivery during provision of the interventions for women and adolescent girls?

**P**: Very poor. The total population here is around nine thousand. There is only one nurse to give service. How could you think this nurse will train adolescent girls from the nine thousand population? It is very difficult.

**I**: What does the community beliefs looks towards the nutrition interventions for the women and adolescent girls?

**P**: It is very low. The farmers are producing many things but they do not use it. Honey and butters are products of farmers but they tend to take it to the market while the mother needs the farm products. There is also low production of vegetables.

**I**: What should be done to solve this issue? For example, what should be done to improve the nutrition of mothers and adolescent girls?

**P**: The main thing is just training. It is important to give training to the farmers so that they can utilize their products properly.

**I**: How should the training be given?

**P**: One thing, the training should be given to all society. For example, when you send a lady to a meeting, there is misconception in the community. Hence, there should be a training focusing the husband and wife. The husband then will understand how much the woman is suffering. This will help the husband to support his wife.

**Section 5: Other interventions that influence adolescent girls and maternal nutrition and health outcomes.**

**I**: How is the practice of early marriage or child marriage in your wereda?

**P**: Early marriage is common. For example, the family only observes the physical appearance of the girl instead of checking whether her age is above 18 years.

I: Why is it happening? Is it because of lack of schools?

P: There is training but the problem is lack of understanding. The farmer only observes the things on hand and there is no future planning.

I: Do you think there is a link between early marriage and nutrition?

P: Early marriage and nutrition are the same. For example, early marriage leads the adolescents to health problems. They may not reach the stage of pregnancy. If adolescent girls are married after 18 years and take appropriate nutrition, they will have good condition.

**I**: What about birth spacing? How do you link the relation between child birth spacing and nutrition? Why would increase the space between each birth improve maternal nutrition?

**P**: We can link it. Child birth spacing and nutrition are the same. Early birth and nutrition are negatively associated. For example, if a mother give birth immediately after 2 years of age, the first child is not well matured. Therefore, instead of two kids that are not well matured, it good to have one well matured in a household.

**I**: Are there activities done to promote birth spacing?

**P**: There is education. Contraceptives are given properly. First thing is to give the adolescent a chance to take measures to prevent pregnancy by herself. Currently it is good. There are changes about birth spacing. Especially, if we you come to urban starting from far rural area, there is good a change.

**I**: Do you think there are factors that affect the early marriage and birth spacing?

**P**: Yes. One time there was religious belief that dictates about birth spacing. If a mother is preventing pregnancy using contraception, it is considered religiously as sin. Since there is a ‘the creator will not let his creature stay hungry’, we can say it is strongly related with religion. There is also a culture of promoting child marriage considering as it is good to marry an adolescent while she is virgin. Therefore, there are conditions that promote child marriage like religious views. There is also a culture that considers being early married as she is from rich family.

**I**: Could you think of any other opportunities to prevent early marriage?

**P**: Yes. Just training.

**I**: To whom should the training for?

**P**: To children and family including father and the male child. It should be given to the father and mother in a household.

I: What about for birth spacing?

P: By the way, the culture should be known to everyone. However, the big responsibility could be taken by husband and wife. Therefore, there should be education.

**Section 6: Multi-sectoral collaboration to improve maternal nutrition**

**I**: Do you feel it is necessary for your sector to work with health sector to address maternal nutrition?

**P**: Yes. It is very important. I said this because one, health sector will talk about how the mother keep their health and the agricultural sector will also teach about what vegetables and fruits should have harvest. As we may not talk detail about health of mothers, they may not also talk detail about the necessity of the vegetables. Therefore, it is good to work together.

**I**: What about adolescent girls? What do you feel if you include about adolescent nutrition?

**P**: It is good. We are focusing about mothers as they are highly affected. But, it is also good to educate everybody including adolescent girls about nutrition.

**I**: Which other sectors do you feel are necessary to work with the present collaborative work between agriculture and health sectors?

**P**: Anyways, the first thing that is important is the farmer. If capacitated, the farmer will not be dependent. When most NGOs are focusing on mothers, the husbands may complain why they are not focused. It is good to build the capacity of the farmer but if the farmer is not capacitated, it is good to support and follow up. Any project can be valuable if it can support the farmers.

**I**: As the health sector is working with you, which sectors do you advise to be included in improving maternal nutrition?

**P**: One sector is the school.

I: What could be the role of the school in improving the maternal nutrition?

P: The school is a sector by itself. Since it is producing students which have scientific merit, if for example the school have 50 students and if 50 of them teach they family the result will be surprising. There is a higher attention from the government and it has also educated human power. Therefore, to teach a farmer, it will be good to use individuals who can read and write.

**I**: What do you think is the role of your sector in the present collaboration between your sector and health?

**P**: The farmer should be trained in all aspects to support the sustainable development of our country. The farmer should be role model in education, nutrition, products, relation, infrastructure, and producing market driven products.

**I**: How do you see the relationship of your sector with kebele, wereda and zone?

**P**: The responsibility of agricultural sector is big. But the relation is good. However, it is not like school or any other sector. Since the activities are very heavy, it may affect the relations.

**I**: How effective are the coordinating platforms in enhancing multispectral collaboration? What should be improved in the current structure?

P: The main problem as agricultural sector is when we see something new, there is a tendency to expand it. It is good to expand but, there is a problem. The second problem is aid. I do not like much aids. It may be good but it increases the sense dependency. It is good to build the capacity of the farmers to prevent dependency. There should also be daily meeting with the other sectors.

**I**: Do you feel the current governing bodies in kebele, wereda and zone are able to facilitate the effective collaboration?

**P**: These days, it is becoming good. Sectors should be led by professionals. The agricultural sector will better be managed by agricultural expert and the same is true with school. But, there is highly abled individuals in the higher structure and there are bodies that oppose the job of the higher body. For example, there are individuals who are working without freedom. There are highly educated individuals in remote area and it is good if they work in their profession. The problem is, there are individuals in the higher bureau that do not have the necessary educational level. There are 10^th^ grade with certificate who are working in wereda bureau. I know it is difficult but the job should be done by professionals.

**I**: Do you have additional point and idea on things that should be done to improve maternal and adolescent girls` nutrition?

**P**: Currently the government is primary focusing on especially on pregnant and children. However, the adolescents are the mothers for tomorrow. Therefore, there should be enough training and education, and things that improve their nutrition. Basically, you should focus on the base than seeing uphill. Therefore, adolescents should get balanced diet starting from childhood and then they may give birth to a good looking normal weight child. This is it.

I: Thank you for taking the time to discuss these issues with me today. I have learnt a lot from you. As I mentioned as the start of the discussion, I will remove all identifying information from the report of this conservation. I will make you sure that no one can identify your comments. If you have any concerns or questions, please feel free to ask me any questions. Thank you very much for your time.

**Summary:**

**Section 1**:

- Mothers and adolescents needs balanced diet, rest and free of stress to stay healthy
- Anemia and goiter are commonly seen micronutrient deficiencies
- Malnutrition is common both in women and adolescents
- Pregnant and lactating mothers are engaged in household farm activities like digging, weeding and harvesting on the top of preparing food of the family even if they are advised to take rest.

**Section 2:**

- Adolescents are not specifically targeted for nutritional support
- Nutritional support in the community is focusing pregnant mothers and children
- The agricultural sector`s priority interventions are training of farms on home gardening and training of mothers on practical porridge preparation, diversification of the component of the powder used for porridge, vegetable home gardening.
- Safety net program is one of the successful programs

**Section 3:**

- The attention given to pregnant women by the community is very low
- Supports provided by the government for free are assumed to be useless.
- Counselling and practical demonstration on food diversification is given to pregnant.
- School feeding is inadequate
- Supply of food support is not sufficient.

**Section 4:**

- Factors that prevent a pregnant woman from utilizing nutrition related interventions like vitamin A supplementation, targeted supplementary feeding include
  - Lack of awareness in the community
  - Poor infrastructure
  - Lack of awareness of farmers about balanced diet
- Training on awareness creation should always include both the mother and her husband

**Section 5:**

- Early marriage is common
- Birth spacing is affected by factors related to culture and religion
- The relationship between nutrition and birth spacing is direct.

**Section 6**:

- There is a live sectoral collaboration between agricultural and health sector
- There is a separate committee established to run activities related to maternal and child nutrition led by the administrator
- Maternal and child nutrition is the priority of the government
- School should be part of the multi-sectoral collaboration in improving maternal, child and adolescent nutrition
