# Supplementary material for: Barriers to utilize nutrition interventions among lactating women in rural communities of Tigray, northern Ethiopia: An exploratory study
Source: PLoS One. 2021 Apr 30;16(4):e0250696. doi: 10.1371/journal.pone.0250696 (PMC8087028; doi:10.1371/journal.pone.0250696)
Supplement: S2 File — (ZIP) [file pone.0250696.s002.zip › S2_File.Doc/Community level Key informants/036_IDI_Agriculture extention_Hashenge Keble_OFla woreda.docx]

**Operational Research on Adolescent and Maternal Nutrition in Northern Ethiopia**

## In-depth interview responses with agricultural development agent

**Introduction**

Thank you for your readiness of the oral informed consent form and for taking the time to speak with me today. I have questions to ask you which were prepared in advance. The discussion will take for 1-2 hours. If you have any questions before we begin please feel free to ask.

**Section A: Interview in details**

1. Zone: southern zone
2. Woreda: Ofla
3. Kebele: Heshenge
4. Name of key informant: Bihafta Birhanu
5. Institution of key informant: Agriculture extension office
6. Interviewer name: Mekonnen Haileselassie
7. Date of interview: 27/022010
8. Interview start time: 6:00 AM (local time)
9. Interview end time: 8:00 PM (local time)

**Section B: Socio-demographic and basic data of qualitative study participant**

| **Socio-demographic characteristic** | **KII** |
| --- | --- |
| Sex | Female |
| Age | 24 |
| Educational status | College education |
| Occupation/role in the community | Agriculture development agent |
| Service year | 4 years |

**Note:**

I: interview

P: participant

**Section 1: Common maternal (pregnant women, lactating women and adolescent girls) nutrition problems in the community**

**I:** What do women do to stay healthy in this community/woreda?

**P:** we have programs in collaboration with health extension workers, we teach the pregnant and lactating mothers how they feed properly and educate them how to produce the balance diet foods. We have clear work share demarcation in between us. Since the nutrition activity is not a one sector task, we found better findings when we become integrated. We create good awareness among the individuals of the pregnant women and lactating one; they start to be benefited from the integration of the two sectors like keeping the environmental sanitation, and the consumption level of the women increases every time. Now they start to think more focus towards for their own consumption than for market purpose.

The women to stay healthy in the community, they should keep their environmental sanitation. They should also keep their personal and water hygiene. Mainly they should also focus on their feeding status. Now there is improvement regarding nutrition in the community.

But still it remains one of the main public health problems of children and women; the production and consumption level is not balanced among the pregnant and lactating mothers; although they produce diversified food types like cereals, pulses, vegetables, fruits, eggs, fish, milk and the like; their consumption level is very poor; they mostly consume only injera with shiro.

**I.** What are the common nutrition problems in the community for women and adolescent girls?

**P:** As I have said before, the main nutrition problems in pregnant and lactating women are poor feeding habit, low accessibility of diversified foods and engaged in high workload. If they are enrolled in high workload, they couldn’t get enough time to feed properly. Mostly they are busy on agricultural activities like keeping of animals, cultivation of land, harvesting. There is a great problem in the utilization of the available food products for themselves. The quality food that produced in our Tabia like butter, teff, and apple are mainly intended for market purposes.

Other problems are the lack of diversified food accessibility in their home, less establishment of home garden and the supply of animal and animal products, less early food preparation for their pregnancy period. There is also an expectation of different donations from the agriculture and the health sector. For example, they expect free seed access like carrot, spinach, potato. If we gave for the poor freely, others are also expecting to get freely. Some are developing the mind of dependency.

**I:** Is there any enough awareness created among pregnant women now? If yes how?

**P:** Yes, we create good awareness among the women; but there is still a shortage; for example we have demonstration site in our office and also we use women who establish the home garden, will serve as demonstration site for others; especially this type of demonstration system is the most effective to create awareness among others; creating the sprite of competition among the beneficiaries is really effective to expand the introduced technology to others. Peer to peer training is also effective to develop awareness among the individuals. The benefits of home garden vegetables in some of the pregnant and lactating women become popular in this Tabia. There is also a program called sustainable undernutrition reduction in Ethiopia (SURE) that helps to have a plan on nutrition based agriculture production. We took a common training both the agriculture and health sector experts on how to promote nutrition and how pregnant and lactating women supported by the program. The health extension workers are selecting the most vulnerable groups and we the agriculture experts provide technical and material supports like seeds of spinach, potato, apple, carrot and we help the technical aspect how to plant these fruits and vegetables in the home garden. The health extension workers also provide training how the balance diet food prepare and how the women consume the food without lose its nutritional content. It was started since 2009 E.C.

Although I am an agriculture expert, I hope there is great malnutrition problem in our Tabia. Almost we all are short and thin. Because we don’t feed properly what we produced. I trust the burden is high in pregnant and lactating mothers; because they have extra load to feed their child. Relatively understanding about nutrition could better in adolescent girls’; since they could get different information like the importance of nutrition, consequence of malnutrition in the community and how to get balance diet from agriculture products; how to get ready vegetables as home garden and how to get animal and animal products at school and other medias like the farmers training center, experience sharing among schools. But still there is great consumption gap among all the communities.

Now, we the agriculture and health experts have a common plan with the objective of improving the nutritional status of pregnant and lactating mothers. We have good starting based on our specialty. But there is still a gap on the implementation of our plan at the ground. Some of pregnant and lactating mothers are resistant to apply the introduced technology at the ground; for example there is less home garden activity and less consumption level of diversified food, and developing the dependency mentality. If we call women for training, some of them are asking you about daily perdium before starting of the training.

But now we are observing improvements; for example some of them have started home garden production after they have been trained. In collaboration with the health extension workers, we showed how to make porridge from the combination of swiss chard, spinach, potato, flour. This improvement becomes after we provide a number of trainings to the pregnant and lactating mothers.

**I:** Do you think women/ girls in this community are suffering from micronutrient deficiencies (like anemia, night blindness, goiter and others)

**P:** This question concerns to the health experts. To my knowledge, anemia is common in pregnant and lactating mothers. This could be due to lack of diversified food consumption during the time of pregnancy and lactation. This is also associated to the lack of diversified agriculture food production or poor consumption of balance diet foods. The lack of poor integration among the agriculture and health sector could also a contributing factor for consumption of unbalance diet foods among pregnant and lactating mothers. We produce barely, wheat, teff, bean, vegetables and fruits; this Tabia is also potential in animal and animal products.

There is no any goiter disease in this area; previously we used ground water and goiter was highly prevalent in this Tabia; but now we use pipe water throughout of our Tabia; and the only iodized salt is consumed.

I didn’t observe night blindness and diet related diseases in our Tabia.

Stunting is very common in this area. It is caused by the lack of balance diet foods among the pregnant and lactating mothers and children. After six months, a child needs complementary foods like porridge prepared from cereals, pulses, milk, eggs, and vegetables. But due to reasons like shortage of these products, amount and feeding interval (how many times and how different food groups per day) could affect the consumption level of complementary foods during this age. Understanding the importance of complementary foods to the child was very poor among the community; a child could eat complementary foods before six months or after a year. But currently, the health extension workers are popularizing the proper feeding time of the child. They teach the mothers to feed their children with exclusive breastfeeding until six months of the child and complementary food is recommended after six months of age.

**I:** Is there a situation when the community suffers from food insecurity and nutrition problem?

**P:** There is food security problem mainly observed in female headed households; because they are not plough by themselves. Therefore, if they don’t produce enough amounts of food products, they could expose to nutrition problems; but we can also observe the nutrition related problems in the food secured one. There is great gap of proper feeding system among the communities; they don’t know the food type, amount and feeding frequency that could consume the pregnant and lactating mothers and children. Food insecurity is associated with the shortage of rainfall in the area. If there is no rainfall, no crop production and as the result mainly pregnant and lactating women, children and elders are affected; because they could not move from place to place for search of food.

In this Tabia, mostly food shortage encounters during the months of June-August; this is due to the shortage of rainfall; during this season there is a shortage of feed for their animals and no crop products. But shortage of rainfall in the woreda is not a serious problem; to my knowledge, it was only encountered in 2007 E.C. Food insecurity is also commonly observed in the community with no farmable land; if the family has no farmable land, they mainly dependent on the soft net programs. Therefore, the nutritional status of the pregnant and lactating mothers with no farmable land could be affected. Economic problem is a basic factor to obtain diversified foods. For example we have milk and egg production in our farm; and our intention is to provide these products to the pregnant and lactating mothers with fair price; however, due to their economic problem they don’t purchase it even with low price.

**I:** what are the common gaps?

**P:** Lack of awareness among the pregnant and lactating mothers are the other problem for consumption of diversified foods. We are giving training pregnant and lactating mothers in the farmer training center how they produce home garden vegetables and chickens with small land space, but to putting into practice are very low compared to the number of trainees.

The lack of strict follows up to pregnant and lactating mothers; since the integration between the agriculture and health experts are started in 2009, we didn’t address all the activities of nutrition at a time. There was no any focus to nutrition in our sector; rather we were concerning to market orienting agricultural products. We didn’t have common plan of nutrition issue and the task was totally run by health sector only. Formerly, only female experts were demonstrating the nutritious food preparation; but now the male experts are also participating in the activity.

There is a self-learning by showing practical activities such as home garden and poultry production among the community is promoted; because if we introduce the sprite of competition among the individuals, it is easy to get expand the home garden and poultry production among the women. This is also very important to solve the shortage of cultivated land among the community; since home garden activity and poultry production needs small land resource with high production. There are many chickens that introduced to the women. We also select and organize as association and prepare uncultivated land to boost their economy via apiculture, poultry production in small land, home garden activity. In case of adolescence girls, we didn’t do anything.

On time supply of materials such as vegetable seeds (carrot, spinach, potato) and agricultural materials (back hoe, water pour and the like) are a problem. There is also lack of water in some kebeles that could lead to use unhygienic water; toilet problem, personal hygiene.

Now some the sustainable undernutrition reduction Ethiopia (SURE) are starting to provide like poultry, home garden seeds to mothers in order to promote their nutritional status.

**Section 2: nutrition priorities in the woreda**

In your opinion, what interventions do you think are the priorities of your institution to improve nutrition?

In our Tabia, there are development groups that run the activities of nutrition in pregnant and lactating mothers. For example we get cabbage, spinach, potato and we first provide to pregnant and lactating mothers. If we bring the seed of apple, potato, carrot and spinach to our demonstration site we share and provide to pregnant and lactating mothers. Our focus is mainly to run the home garden by the pregnant and lactating mothers. The other good thing is improving the consumption habit of different vegetables and tubers like potato, carrot and spinach. These fruits and vegetables don’t need large land to produce. For example carrot needs very small land and no need of transplanting rather grow at the original land. Now awareness is created on the area of home garden activities.

Now our sector has developed great knowledge and emphasis on the area of nutrition. We have common plan and get common training with health sector. Previously, I didn’t have any knowhow on nutrition issue and I didn’t know what the health sector was doing. Regardless of the nutritional issue, we were focusing only production and market oriented activities. But now we are more concerning to feed by mothers than marketing purposes. Now the SURE program is starting and there are 21 Tabias in this district and we share equal participation with health sectors in the area of nutrition promotion. We have common plan and get training in common.

The agricultural sector supports in production of diversified food groups like cereals, pulses, vegetables, fruits, tubers with supporting profession based techniques starting and the health sector supports how the consumption of the produced agricultural products are promoted; such as how the food is prepare without losing its nutrition content, and the time and frequency of food consumption per day and the like; and now we have knowledge share in between of the agriculture sector and the health sector. Formerly this type of cooperation was not present; the agriculture experts was get training about the improvement of production and mainly market oriented production; and the health experts was get training the nutritional importance of different food items regardless of its availability and source. We didn’t have any common plan. But now it is totally changed; we are thinking to produce nutritious food items for the pregnant and lactating mothers’ consumption rather than market oriented. We have the files of all pregnant and lactating mothers in this Tabia; and if there is any opportunity in our sector that benefits the pregnant and lactating mothers, they are our first hand group.

The SURE program is working in nutrition areas in both the health and the agricultural sector. In the agriculture sector it mainly works in fruits and vegetables like apple, potato, carrot, swiss chard, and in the introduction of animals like poultry. Materials like shovel, hoe, and pitcher are also provided. There is a plan to provide mothers with three chickens for each. So our task is to prepare proper house for the chicken without mixing of the mothers’ house so as to keep sanitation.

We have also different fruits and vegetables like apple, carrot, swiss chard and cereals and pulses in our farmer training center. Thus it is easy to give training by practical demonstration of the activities for pregnant and lactating mothers so as to promote nutrition and food security. We have also chicken and dairy farm, and we sell to pregnant and lactating mothers at fair price.

Currently we are much effective in the area of home garden planting. We gave swiss chard for 32 pregnant and lactating mothers and we found them with successful result. The numbers of pregnant and lactating mothers who request us to run the business become triple now.

Compost production is also become very successful activity. The garbage substances at the homestead was collected and buried in the pit to produce organic fertilizer for the home garden vegetables. The pregnant and lactating mothers are becoming very happy as the result of introducing the home garden vegetables. During our activity evaluation with the health experts, we found that the consumption level of vegetables is increased by the pregnant and lactating mothers.

Once the SURE program was starting, the health extension workers are selecting pregnant and lactating mothers and we the agriculture sector provide them the said fruits and vegetables; and then we follow all the home garden products from the farm to the fork level; the common plans of our activities are evaluated weekly.

**Section 3: nutrition interventions that improve adolescent and maternal health**

**I:** What kinds of nutrition are in place to improve health of the pregnant in this woreda?

**P:** The most successful activity in the improvement of nutrition among pregnant and lactating mothers is the collaboration work among the health and agriculture sectors with having the common plan. We make a synergy to promote the nutrition among the community especially pregnant and lactating mothers. We create awareness among pregnant and lactating mothers about the production of nutritious food products. They start to request us whether we have seeds or not. This is an indication how much they aware about the agriculture products which is a big achievement for our sector.

Previously we didn’t care about pregnant and lactating mothers; we found farmers and talk about the increase of crop and animal production; how the farmers use an input like fertilizer; we didn’t know what type of crops are nutrition sensitive and how we give priority; we focused only about production growth based on the market focus.

But now many pregnant and lactating mothers are producing potato, swiss chard, carrot, and cabbage after they have been got training. On the same side the consumption of these products are increasing by the pregnant and lactating mothers. They also benefited from the chicken products like egg. We share the available kushets in the Tabia; and the health extension workers teach about the importance of different food types in terms of nutrition and we also educate the production growth such as home garden activity with small land but high production.

The pregnant and lactating mothers are benefited from the introduced products such as carrot, cabbage, potato. And the consumption level is increase since our collaboration between the health and the agriculture sectors are strengthening.

**I:** Do you think that pregnant and lactating women receive advice on the need to get extra meal?

**P:** The feeding aspect is followed by the health extension workers; but in our side we support them to produce different types of food items what I said above. So we encourage pregnant and lactating mothers to produce the agricultural products in their farm, and the health extension workers also educate to eat in different preparation approach such as porridge, soup and the like.

The home garden vegetables like swiss chard, carrot, spinach, mainly apple don’t take large farmable land but give high production. Now most of pregnant and lactating mothers accustomed to the activities.

In collaboration with health sector, we participation in environmental sanitation by doing compose; that is by digging pit one by one meter and all the garbage are put into the pit. In this case we are keeping the environmental sanitation and also the compost uses as organic fertilizer for the home garden. All the animal dung and any waste substance are disposed into the pit for the compost purpose. This activity could contribute in the prevention of acute vomiting and diarrhea in the community.

**I:** How about in advising the pregnant and lactating mothers to eat diversifying food?

**P:** This is predominantly the task of the health experts. But we insist them to produce diversifying products like cereals, pulses, fruits, vegetables and tubers.

**I:** How about in case of adolescent girls?

**P:** We don’t have any activity with adolescent girls in relation to nutrition. I trust the education sector is an ideal to educate about nutrition. Because tomorrow they will be mothers; so they need special attention at this age. Therefore, education about nutrition for adolescent girls is vital. They could have also an influence to educate their family about the nutrition and sanitation.

In our side we find the adolescent girls and boys above 16 years to engage in different agriculture activities like fattening once they become organizing in the form of association; they have given mountainous area to fatten sheep and goats and they become more beneficiaries.

**I:** In your opinion which of your interventions is being implemented in an effective way? Why do you think that it is effective?

**P:** The most effective activities in the improvement of nutrition among pregnant and lactating mothers are the collaboration work between the health and agriculture sectors with having the common plan. We make a synergy to promote the nutrition among the community especially pregnant and lactating mothers. We create awareness among pregnant and lactating mothers about the production of nutritious food products. They start to request us by themselves whether we have seeds or not. This is an indication how much they aware about the agriculture products.

Now many pregnant and lactating mothers are producing potato, swiss chard, carrot, and cabbage after they have been got training. On the same side the consumption of these products are increasing by the pregnant and lactating mothers. They also benefited from the chicken products like egg.

**I:** Which of your interventions of nutrition are less effective to the pregnant and lactating mothers? Why?

**P:** To say we are successful in our activities, all the pregnant and lactating mothers should get benefited from our inputs. But now few are benefited from the many once. This is because due to less understanding the importance of different trainings. Most of them give more stress to the daily incomes. For example if we arrange the training without perdium, they are not willing to attend the training. In this case, in all the pregnant and lactating mothers, we didn’t develop the sense of independency.

**I:** what are the challenges to implement delivering the nutrition interventions in the pregnant and lactating mothers?

**P:** in case of the pregnant and lactating mothers, they don’t tolerate high workload. The lack of farmable land and water access to their home garden vegetables is also a challenge. The supply of different in puts is not timely found; for example all the seeds of the vegetables and fruits, the materials used to run the activity of the vegetables like hoe and chickens. We didn’t also assess our potentials in the Tabia in such way that which activity is appropriate to become effective to the pregnant and lactating mothers. Assessing the unforeseen potentials of the Tabia for pregnant and lactating mothers is very important to implement accordingly. For example the fish products which is the most nutritious food products for pregnant and lactating mothers from the Hashenge lake is untouched.

**I:** How do you evaluate the resources available to the interventions?

**P:** As I have said before, there are many shortages such as lack of awareness, supply of plant seeds, chicken

The most important thing to work here is making awareness to the pregnant and lactating mothers. If they understand the benefits of our activity they could easy to engage in our programs and improve the nutritional status of the pregnant and lactating mothers. I highly advise to introduce the vegetables and fruits and chickens to address for all the pregnant and lactating mothers that could bring a change in their nutritional status. If the pregnant and lactating mothers introduce dairy cow either in group or individually, then it could boost or contribute in their economy and nutrition aspect.

Now we created good collaboration between the health sector and the agriculture sector. We have structures in the form of development group that coordinate the individuals at the household level. So it is easy to get and organize all the pregnant and lactating mothers.

Strict follow up to the pregnant and lactating mothers; giving training the health and agriculture experts and to pregnant and lactating mothers; supply of input for pregnant and lactating mothers; for example if I train the pregnant and lactating mothers about the home garden or chicken production, they need the supply of seeds and chicken immediately to introduce it. They need also encouragement for the model once in the form of reward so as to attract others.

Currently, there are some pregnant and lactating mothers have been provided the home garden seeds such as swiss chard, spinach, potato and carrot by REST and the SURE program and become started benefitted.

**Section 4: community factors affecting access to maternal nutrition interventions**

**I:** What barriers do you think are preventing the pregnant and lactating mothers and adolescent girls from using the interventions?

**P:** The level of education has great influence to accept the training and to put into practice. The higher education the pregnant and lactating mothers achieved, the better they become put into practice.

Skill and knowledge of the experts: building the capacity of the agriculture and health experts are very important so as to train the pregnant and lactating mothers in very convincing and innovative approach.

Compared to the previous one the awareness among the pregnant and lactating mothers is very encouraging. There is a development of good awareness among the mothers on the importance of consuming quality food in terms of nutrition. The training is given in the form of pictures which is best to convince easily except the problem of supply.

In case of the community, as I have discussed before the problem is supply and awareness but in some individuals are giving negligence. But the consumption level is improved.

**I:** How can the barriers are addressed to improve nutrition in the pregnant, lactating mothers and adolescent girls?

**P:** supporting the pregnant and lactating mothers through training and demonstration. Strong support from their husbands is very improved. Now the equality is already developed. In case of adolescence we didn’t do more. Since they are engaged in school, we are not expecting them to produce any activity, but making awareness about nutrition in the school is very important and they will serve as mediator to educate their families.

**Section 5: other interventions that influence adolescent and maternal nutrition and health outcomes**

**I:** In your opinion, why would delayed marriage and increase space between each birth improve maternal nutrition?

**P:** This is the questions to the health sector; but it is known that underage marriage causes delivery problem, stress, and fistula; however, if she is above 18 years she has already matured, there is no as such stress and other problems like fistula could encountered. In case of enough space among the consecutive birth interval, it has many advantages to the mother and the child; if not, the child could not get the favorite treatment like proper breastfeeding up to 2 years, timely supply of complementary foods and not keep the hygienic status of the child. The mother also becomes stressed to treat the children at the same time.

**I:** What programs or activities promote increasing birth intervals in this level?

**P:** Training is very important by showing the different between children born with enough space and not using colorful pictures; which is very explanatory to understand them.

In our Tabia, although this is the task of health experts, I have information that some individuals are punished as the result of early marriage. So it is a series issue in our Tabia. If under marriage is undertaken, the fathers of the two pairs could get punished.

For long space between the child birth, mothers use pill or injection, but there is a fear of remaining unproductive (sterility) among the mothers. This hesitation is not yet solved.

**I:** Can think of any opportunities to prevent early marriage and increasing birth spacing?

**P:** We have the opportunity that enough health center, schools to promote these activities; the awareness is created in every structure of the government.

**Section 6: multi-sectoral collaboration to improve maternal nutrition**

**I:** Do you feel it is necessary for your institution to work with other sectors/institutions to address maternal nutrition

**P:** Yes, in collaboration work is the most important preference to solve the problems. We have now an interface among all stakeholders. We share our plans that could work in collaboration with the respected stakeholders and evaluate the final results together. Our main stakeholders are health sector, water, security, education. We have also a visit program in the form of SMS (subject matter specialist) from each stakeholder (agriculture, health, water) to observe and evaluate the performance of each activity per our plan. For example we are doing very effective activity in collaboration with the health sector in the area of sanitation and compost production, home garden vegetables, in raw planting of potato, chicken farm and the women’s food consumption level. The same is true with water sector about the efficient use of water through dropping method to the home garden vegetables.

**I:** What needs to be done to improve the capacity of the stakeholders for effective coordination?

**P:** To all the stakeholders, there should get common awareness on the importance of integration. The integration of respected stakeholders is not only valued on the nutrition issue but also in other activities. For example to be more effective of enough birth interval among mothers, the education sector could promote very well through the students ; because most households have a student in the school and the family could get awareness through them. The Tabia leaders have also high power to convince the community.

However, there is some drawbacks of working in collaboration such as, the presence of less devotion to take others’ assignment; and holistic knowledge limitation of experts. Generally the main problem is less awareness among the experts; because we didn’t get common training. Therefore, experience sharing, common training among the stakeholders are important.

**Comments**

I learned a lot from this discussion. Many unforeseen activities are observed during this interview such as, the less attention on the nutritional status of adolescent girls.

I am very much satisfied on the occasion of working together with the responsible stakeholders. I can learn from the stakeholders’ activity and holistic approach. We have the opportunity to run in integrity way like, we have enough experts, and the Tabia is potential for fruits and vegetables like apple, spinach, potato. I learned additional lesson on the area of adolescent girls to involve in the area of nutrition.

**SUMMARY**

**Section 1: Common maternal (pregnant women, lactating women and adolescent girls) nutrition problems in the community**

- The quality food like butter, teff, eggs and apple which are produced in our Tabia is mainly intended for market purposes.
- In some mothers, there exist the sense of dependency mentality on the soft net programs
- Mothers don’t prepare/ store some quality food types for their pregnancy and early lactation period.

**Section 3: nutrition interventions that improve adolescent and maternal health**

- The compost production is found very effective among the mothers for their home garden vegetables as organic fertilizer and in directly helps to keep the environment healthy. All the garbage that are found in the homestead of the households are collected and put into pits to serve as organic fertilizer for the home garden vegetables.
- The production of different home garden vegetables such as potato, swiss chard, carrot, and cabbage are done by the pregnant and lactating mothers. Their consumption level of these vegetables is also improved.

**Section 4: community factors affecting access to maternal nutrition interventions**

- The skill and knowledge of the agriculture and health experts has an influence to encourage and train the pregnant and lactating mothers in the innovative approach.

**Section 5: other interventions that influence adolescent and maternal nutrition and health outcomes**

- There is a fear of remaining unproductive (sterility) among the mothers during the use of pill or injection to have long space between the child births.

**Section 6: multi-sectoral collaboration to improve maternal nutrition**

- We share our plans that could work in collaboration with the respected stakeholders (health, water, and education sectors) and evaluate the final results together.
- We are doing very effective activity in collaboration with the health sector in the area of sanitation and compost production, home garden vegetables, in raw planting of potato, chicken farm and the women’s food consumption level. The same is true with water sector about the efficient use of water through dropping method to the home garden vegetables.
