# Supplementary material for: Barriers to utilize nutrition interventions among lactating women in rural communities of Tigray, northern Ethiopia: An exploratory study
Source: PLoS One. 2021 Apr 30;16(4):e0250696. doi: 10.1371/journal.pone.0250696 (PMC8087028; doi:10.1371/journal.pone.0250696)
Supplement: S2 File — (ZIP) [file pone.0250696.s002.zip › S2_File.Doc/Community level Key informants/037_IDI_Kebele leader_Hashenge Keble_Ofla woreda (1).docx]

**Operational Research on Adolescent and Maternal Nutrition in Northern Ethiopia**

***Date: Nov 6, 2017***

**In-depth interview with Kebelle leader of Hashenge towm, Ofla Woreda, Tigray.**

**Section A: Interview Details**

1. Zone: Southern zone
2. Woreda: Ofla
3. Kebelle: Hashenge
4. Name of key informant: Abebe Tedeba
5. Institution of key informant: Hashenge kebelle
6. Interviewer name: Mengistu Mitiku
7. Date of interview: Nov 6, 2017
8. Interview start time: 8:43 AM
9. Interview end time: 9:46 AM

**Section B: Interviewee professional information**

1. Gender: Male
2. Age: 39 years
3. Highest level of completed education: High school
4. Current Job position: Hashenge Kebelle Leader
5. How long have you been in the current position: 6 years

**Main interview**

**I:** Interviewer

**P:** Participant [The key informant]

**I:** Good morning. I am Mengistu Mitiku from Mekelle University. Thank you for coming and showing willingness to participate in this study. We are doing research on maternal and adolescent nutrition in collaboration with UNICEF and TRHB. One thing I would like to say is that your opinions are very important. While you share me your thoughts and ideas, I will tape record our conversation as it is difficult to write all the things you speak. Moreover, our conversation may take 1 and half an hour to 2 hours. So, do you have questions before I proceed?

**P:** It is clear. I don’t have any question.

**I:** Okay. Please tell me anything that you would say is unclear.

**Section 1: Common maternal nutrition problems**

**I:** Thank you for being here and showing your willingness to participate in this study. Okay. Let us go to the first question. The first question is ‘What should mothers like pregnant and lactating mothers and adolescent girls do to become healthy/live in healthy state?

**P:** Ehh…*[participant trying to make his throat open to start the conversation].* Okay. One of the reasons why mothers should be healthy is to protect the generation.

**I:** Definitely. But, the question is what should mothers and adolescent girls do to stay healthy?

**P:** The first thing they should do is, having only one partner for those not married and having trust on their marriage. The second reason is having medical diagnosis every three months and the last third thing pregnant women and adolescent do is to consume foods that are of good quality and neat. The other thing they should do is to build latrine in the backyard they have, *[participant’s mobile started to ring out and trying to tell me what the sound is ~ it is message, it is message].* Mothers should accomplish all the things I mentioned so far.

**I:** Good. When we say mothers, it could be pregnant women, lactating mother and also we have another category ‘adolescents.’ So, could give detailed thoughts you have.

**P:** Yes, when we consider pregnant women, they should start antenatal care utilization services; they need to eat adequate and balanced food of different kind. When we come to lactating mothers, they need to eat adequate amount of food, like pregnant women; they need to make themselves free of any work load and therefore take rest, they need to keep their personal hygiene as this have a good return to the baby. Moreover, they need to make themselves free from alcohol.

**I:** What about adolescent girls?

**P:** If we consider the girls whose age ranges from 10-19 years, those we call them ‘\adolescent’ girls, I can many things. Especially when those girls are grade 8 and above, they usually face challenges like sexual harassment and under age marriage. To avoid such problems and challenges of adolescent girls, they themselves should be careful of their life, keep their protocol and if their age reaches 18, they should get married. That is it. That is our culture.

**I:** What is that ‘protocol?’

**P:** When I say protocol, I mean avoiding close relations with boys they don’t know, when they go to school, avoiding approaching males. At adolescent stage, knowingly and unknowingly, girls face many challenges. Perhaps the male may not harm their female counterparts. But, sometimes adolescent girls may initiate the harassment. This is not surprising as they are at their puberty period. Those at the late stage of adolescent, usually called ‘Tub Habet’*[Tub Habet refers to those whose breast has grown well ],* the stage they are in will force them to act. That is it.

**I:** When we come to the next question which is related to nutritional problems of mothers which includes pregnant women, lactating mothers and adolescent girls, what do you know?

**P:** Yes, I know some problems. When you say nutrition related problems, women face difficulty of delivery when they are malnourished. They face also anemia as a result of malnutrition. Anemia forces pregnant women go through prolonged delivery. If we consider those married under age, they face difficulty of delivery and even sometimes they encounter problems related to their sexual organs. Those things happen as under age adolescents do not have well-built, through good nutrition, body system. This situation goes to the baby coming from the womb and finally baby/babies will be stunted or will be born with a sort of illness. We have such problems related to nutrition which are currently occurring in our community.

**I:** What can you tell us about the state of ‘underweight’ in your kebelle?

**P:** It is what I told you so far. Those who don’t eat adequate food will be underweight, unstable ones with a much weaker state of mind where they don’t have a well-established schooling behavior and therefore don’t understand things well. This is linked with intake of inadequate amount of food and other helpful food items. Moreover, anxiety disorders will occur as a result of food intake inadequacy which leads to mental illness and therefore to madness.

**I:** How do you link mental illness with nutrition?

**P:** You mean the relation mental illness has with nutrition system? When a person doesn’t get what he/she needs, she/he will be disturbed and might therefore start to use cigarette smoking, chat chewing, ‘katikala’ *[Katikala is a type of alcohol]* drinking and other addiction will overwhelm him/her. All these are occurring due lack nutrition and of course due to anxiety. Nowadays, girls don’t marry with someone who have money or adequate resource though she is a beautiful adolescent, she doesn’t want to get married as unless she doesn’t have food security. If they don’t have access to good house materials, they don’t care about marriage. They stay for longer period uncoupled. Therefore, food security which could be expressed in terms of possessing adequate food and other capitals like good house furniture and land assets is must for adolescents to stay healthy and be mentally strong.

**I:** You told me that there are nutrition related problems with pregnant women, lactating mothers and adolescents. Which groups of women are commonly affected by those nutrition related problems?

**P:** At the first level, mothers will be affected. If she is affected by those nutrition related problems, the baby will not get breast milk and when breast sacking by the baby takes place, it is blood of the mother that will be sacked as she didn’t take enough amount of food. In such manner, the mother will have illness and the same problem might be transferred to the baby.

**I:** But, who will be more affected?

**P:** It is similar for all groups of mothers. Why I am saying like this is that when we take pregnant women and when the intake of food is not adequate, she will be in jeopardy and these problems will be transferred to the baby in womb or child at hand. It will not be good news for the baby and herself. Similar effect is also seen in lactating mothers. The more she takes food the healthier the baby will be. It is the mothers’ situation that determines the baby’s situation and health condition. Adolescent girls will be the last group who will be affected by nutrition related problems as they are a group capable of consuming better amount of food.

**Section 2: Nutrition Priorities**

**I:** Fine. Now, let us go the next question, which is maternal nutrition improvement related issue. Do you think that your kebelle’s participation in those priority maternal nutrition improvement activities is compulsory?

**P:** Yes.

**I:** How?

**P:** For example, when women and lactating mothers undergo nutritional screening procedures in health facility, those who are anemia and who encountered body weakness will be supported by our kebelle administration system. One of the means by which we support them is the Safety Net program, the other is food aid system and the third one is by exempting them from water and soil conservation activities. This gives them the opportunity to get rest. I think helping mothers in such a way is necessary. Community members are ready to work with any project like REST and SURE who are helping us a lot in funding women delivery related activities like giving ‘Mash.’ The main issue is that if the project is coming to serve our mothers in particular and our community in general, we don’t have commitment issues. We are all the time ready to get involved in any work intended to benefit the community. That is it.

**I:** What maternal nutrition related projects or intervention are given priority in your kebelle?

**P:** I know one project that supplies ‘FAFA’ to the community. This project is working in collaboration with REST and its main aim to alleviate food insecurity in our kebelle though there is no tangible result that shows their contribution. Along with our health extension workers, women association and women development army with a network of 1 to 5, we are currently working with this project in reducing maternal nutrition related problems. The movement of all these stakeholders take place ones a month where nutritional assessment of mothers and children will be organized and those with malnutrition problem will be detected with and ‘FAFA’ will be given to them. The other intervention is that fact that women are free from soil and water conservation activities.

**I:** Fine. Let us talk about the success of the interventions aimed at improving maternal nutrition. You told me that REST, SURE are assisting you in implementing interventions aimed at improving maternal nutrition. In your perspective, which programs do you think were successful and model?

**P:** When we consider NGOs working on maternal nutrition like SURE, we didn’t observe a big success. They are helping us simply superficially.

**Section 3: Nutrition Interventions that improve maternal and adolescent health**

**I:** Still we are talking about the nutrition interventions that are supposed to improve maternal and adolescent health. In the kebelle that you are leading, what types of interventions related to nutrition are being accomplished? Let us consider nutrition interventions that support pregnant women’s health?

**P:** When we consider pregnant women’s related activities, we have the initiative ‘one container of powder for one mother’ which currently is the common way of supporting mothers who come to health center for delivery of mothers in general. In this agenda, the whole member in our kebelle is participating. I said the whole community. What is being done is that the sorghum or wheat or any other powder collected from the community is in part brought down to the market and sold. In return, other necessary resources such coffee and sugar will be bought for the pregnant women and this will be kept at health center and served for when pregnant women come for delivery.

**I:** What about other interventions like council about family planning and others?

**P:** Yes, for example we have a sort of meeting that pregnant women undergo every month. In this meeting, they will be advised how to handle their pregnancy, what to do when they feel something unusual and they face a sort of bleeding. Moreover, they will be oriented what symptoms to look for and report. They basically exchange their pregnancy experiences so far. Such meeting of pregnant mothers is really bringing a good change in awareness and knowledge.

**I:** What about their nutritional intakes and general practices?

**P:** Yes. They also talk about the general aspect of food intake. They talk about the importance of vegetables, danger of alcohol and the food items they should avoid like those food items with sweet taste. They discuss also about the benefit of mixing different food items like salad, cabbage, potato and others. Moreover, they have the opportunity to learn other things like eating a diversified food that eating a single foot item.

**I:** What about lactating mothers?

**P:** It is similar. They are almost the same. The main objective is for mothers to produce milk for their babies. Therefore, for the lactating mothers to have adequate milk for their babies, they should eat diversified food. It is almost similar.

**I:** What about iodized salt utilization?

**P:** This is common in most households. Our community members have a good understanding of this food item, i.e., iodine salt and use it.

**I:** The other issues are home gardening, Safety Net and others. What can you tell me about all these, then?

**P:** Yes. It is right. Due to the involvement of agriculture, women are starting to cultivate vegetables in the backyard plot of land they have. Vegetable products like potato, salad, cabbage, onion, pepper and others are being produced in the very small plot of land at the backyards of every house hold. It is usual to seen vegetable production at almost every household. It is real. Apart from this, households are practicing to make their home and their surrounding environment clean, with some households using good waste management system like using containers for solid and liquid wastes. It really exists in our kebelle.

**I:** What can you tell us about lactating women’s usage of bed net?

**P:** We don’t have bed net distribution service. I know it is important but we don’t have that program, provision of bed net to households. Such service exists in Raya Azebo. Our area is skipped as it is assumed to be high land area and there are no mosquitoes.

**I:** Okay. So far, you talked about the nutrition interventions that could improve pregnant and lactating mothers’ health. Let us now come to adolescent girls. In this regard, what can you tell us about the nutrition interventions in place intended to amend the health of adolescents?

**P:** There are not well known interventions as far as I know. What we are doing for adolescent girls is that, as they are at younger ages during which puberty influence tightens and therefore start to create relations with their male counterpart, the need to train them and upgrade their awareness is very important. Because, we have the wory that adolescent girls’ innocence will expose them to diseases like HIV. That is what we are doing for adolescent girls at this time.

**I:** Okay. As I said so far, adolescent girls are the factory of future generations. So, what should be done in general?

**P:** Giving awareness creating trainings is important. You know, the first thing that should come is to build the mind of these adolescent girls either at school or community level. So, I believe that sustained trainings which could be conducted in collaboration with our stakeholders could bring about tangible changes. We need to advise them to eat at least three times a day, to utilize every product their household gets. For example, instead of bringing down milk in the form butter down to the market, they should be advised to consume it in its initial form, the milk. Instead of putting the butter on their hair and painting their body parts, their families have to be advised to feed their adolescents. Moreover, instead of sending products like ‘Teff’ and Honey to the market and converting to money, they should use it. Adolescents should eat breakfast, lunch and dinner. It is very important. In our kebelle, there are adolescents who eat breakfast and spend the whole day without getting a piece of food.

**I:** Well. So far, I have heard some information from you regarding maternal and adolescent girls’ nutrition and intervention intended to improve the health of mothers and adolescents. Among all those interventions, which of them do you think are successful?

**P:** As far as I know, there is no tangible work done and tangible result obtained. I cannot say this was successful and this was not.

**I:** So far, you told me that there is no successful and tangible work done and a good return obtained. Why is that? The challenges?

**P:** The challenge is that the leadership at zone and woreda level is poor. You observed that Hashenge is a good and fertile kebelle. But, there is no any work done to maximize the productivity of this kebelle. The need for strong leadership in our woreda is high as the current administrative body is performing badly. The focus is being given to Raya and the low land areas in this area, southern zone. However, since the wored adminditarive body believes that Hashenge is a kebelle where agricultural activitiy is good and productive, we are not getting what we are supposed to get. The pressure from the government is not allowing our pregnant women, lactating mothers and adolescent to get a sort of benefit from the fertile land we have. Overall, the big challenge we have is leadership. The presence of weak leadership is preventing us our mothers and girls not to get full support.

**I:** What do you think is the fact that the administrative bodies of your woreda are skipping your kebelle?

**P:** Not allowing our zone to be administered by its kebelle born scholars and individuals. Yes. The fact that our zone administrators are not dwellers of the woreda is bringing about a bad spirit in the minds of the community. This is real. The other challenge is, our area as you can see, is a best place for investment. There are many NGOs and other bodies pointing their fingers to invest around Hashenge. But, the path is closed at the upper level management. If this is the case, tangible and successful activities cannot be done and therefore the community cannot benefit from it. When I say community, don’t forget that mothers are the main members.

**I:** What kinds of measures are taken by your woreda to address the challenges?

**P:** As far as I know, there are no innovations and unique efforts the woreda has taken as a means to improve the maternal and adolescent girls’ nutrition status and of course their health as well. No effort made.

**Section 4: Community factors affecting maternal nutrition intervention**

**I:** You told me woreda related issues and efforts. What about the community related factors that hinder our pregnant women, lactating mothers to badly utilize the nutrition interventions you shared me so far?

**P:** I told you that backwardness is the big factor here. Lack of knowledge, locality oriented thinking are another factors. Moreover, community culture and belief have significant effect on the fact that mothers are not using nutritional interventions.

**I:** So, you told me a couple of community related factors. How can all the community related factors be addressed?

**P:** I would say by preparing forums. By creating spirit of struggle. Moreover, learning from model experiences and capitalizing on them is another means. Even bringing innovations to the ground can address the barriers. In addition to this, we have to evaluate our means, compare and contrast past and current progresses and results and learning from the differences. The big issue we might be missing is that we don’t have a habit of learning from previous experiences what so ever the result was.

**Section 5: Other intervention influencing maternal nutrition and health outcomes.**

**I:** In addition to the nutritional intervention we talked about so far, we believe that there exist other interventions that could improve maternal nutrition and finally bring about good health outcomes. You know that increasing birth spacing assumed to be one intervention. How could birth spacing improve maternal nutrition?

**P:** In my opinion, our female sisters are always undervalued. For males, the reverse is true. Males do come at any time and do sexual intercourse with their partners for the sake of fulfilling their feelings. Most of the time, they don’t think of the risk of their wives getting pregnancy. Thus, if such things occur, the possibility of achieving long birth interval will be terminated. That will bring harm to the mother itself and to the children born at a short interval as feeding both children will a heavy workload to the mother. However, when the spacing is long, the mother will have time to feed her child and herself. She will not be disturbed and therefore she will be mentally good and competent.

**I:** What about marriage after 18 years? What is its effect on the nutritional status and therefore on the health of mothers and adolescent girls?

**P:** The impact of early marriage was big. However, it is completely avoided in those days. We haven’t hosted under aged marriage since 1992 E.C. No marriage for under aged adolescents. This success is achieved as the system our government has laid was effective and the leadership at all levels had a big concern for female harassment. The role of the different stakeholders like the Justice bureau was also productive. The heath team did well. Any ways, the leadership role was the most influential one in this regard.

**I:** What do you think is the influence of religion on under age marriage and birth spacing?

**P:** The influence is big in our kebelle. In our kebelle, we have two religions: Muslim and Orthodox-tewahdo. These two religions strongly support the issue of marriage when the age of partners is above 18 years and when children to be born at wider intervals. Religious leaders support the directions of the government. As far as I know, almost every resident of this kebelle is easily influenced by religious leaders. For example, priests are leading actors in our kebelle.

**I:** You told me that you haven’t encountered under age marriage since 1992 E.C. what do you think was the driving force for such success?

**P:** The driving force which led us to such success was those who violated the rule that states that under age is strictly forbidden under the law. Couples who didn’t start sexual intercourse were taken from their wedding ceremony and imprisoned. They were forced by the law not to form a marriage. Those who played the main role were still imprisoned and the girl continued her education after which got the real opportunity to get married in the interest of her mind. The person who was forced to be imprisoned and who tried to get married was the girl was a farmer. This farmer had again spoiled his name as he again harassed another young girl and forced her to be a victim of fistula which finally resulted in death of the girl. She went west. These two cases always give us good lesson. The one who experienced fistula has gone west and the one who survived and become a teacher has got her parents punished for violating the law. We use these two scenarios to teach our community.

**I:** What do you think is the main community factors affecting under age marriage?

**P:** Backwardness. I said it is backwardness. It is due to bad belief and silly mind. But, it is completely solved.

**I:** To achieve marriage at optimum age level and wide-spaced birth interval, what do you think are the real opportunities your community have?

**P:** The fact that we can establish forums where community’s awareness could be increased is one opportunity. The fact that trainings could be conducted is another opportunity that really helps control early marriage.

**I:** What is the essence of the forum here?

**P:** When I say forum, I don’t mean forum for the whole community. It is impossible. The forum might be organized by using representatives from religions, mothers and adolescent girls. Then, the representatives will be made to act and cascade downward to the lowest level. Yes, it is in such a way that the community should be influenced.

**Section 6: Multi-sectorial collaboration to address maternal nutrition**

**I:** The last question I have is the issue of multi-sectorial collaboration to address maternal nutrition. Do you think that inter-sectorial collaboration is very important?

**P:** Yes, definitely.

**I:** How?

**P:** Let me tell you one saying. If you have a single stick and tried to do with it, it will be broken. When, on the other hand, three sticks are bundled together, the sticks will be straight and solid. The same is true. Working together will make you productive. When these nutritional issues related to pregnant women, lactating mothers and adolescents are worked on as one agenda, it will be good. When we consider lactating women and adolescent girl, as the adolescent girl will come to the situation where the lactating mother is in, it gives sense if the lessons are taken from the lactating mother. Definetely, the adolescent girl gets married after a year or two years. Similar lessons will also be learned from the pregnant women as well.

**I:** Okay. Right now we are talking about nutrition. When the term nutrition is talked about, it is the health sector that comes to our mind. For activities in health, it is not a wise way of thinking that only health personnel are required for its execution. In your opinion, what other sectors are required to participate?

**P:** The involvement of mothers themselves, non-governmental organizations, women’s association and justice bureau. Women’s affairs should also participate in this agenda. The involvement of religious leaders, women development army leaderships along with their system 1 to 5 and 25 to 30 networking could participate.

**I:** What can you tell me about the collaboration agriculture can have in the issue of maternal and adolescent nutrition?

**P:** The involvement of agriculture is very important. It is agriculture that produces different food items like salad, cabbage and others. It is from agriculture that milk is produced. It is agriculture that hybrids cows and come up with a better milk producing ones. When we consider hens, it is the activity of agriculture to handle them and produce eggs. The collaborative activity that exists among agriculture, education and health sectors is very mandatory for productive work.

**I:** If we believe that the collaboration that exists among the different sectors like agriculture, education and health is crucial, what change should they devise or improve to have a productive work as a whole?

**P:** Yes. What I am saying is that there are four governmental organizations that should work together. These are agriculture, education, health and leadership organizations. It is the coordination that exists among those sectors that helps. If all operate separately, it will be meaningless. The main of the collaboration is to transform the community. It doesn’t have other agenda. It is to bring better life for the community. If this is to be accomplished, the job that comes down from the higher level management should consider all the sectors in a fair manner. For example, when trainings are conducted, they should not be separately treated. The need to give training to all sectors together should be the issue. We are witnessing separately managed trainings. Therefore, there should be a common stage where all work together as a system.

**I:** What do you think is the role of your kebelle in the collaborative activity that the sectors like agriculture, education and health might have? The role of the kebelle you are leading?

**P:** What I would say about the collaboration my kebelle should have is that, we have four villages all with a good irrigation system. In all these four villages, there is a good opportunity to produce vegetables. When a call for meeting is made, all the villages will participate together so that one kebelle will learn from the other. All the villages will raise questions on the agriculture perspective, on health, education and leadership perspectives also. The issue of ranking also another agenda of the meeting. When work is done together, the coordination among the workers is also vital. We have four health extension workers for four of the villages. We have also agriculture focal person at each village. The same is true for education. Therefore, together with the leaderships of the four villages, the extent of collaboration will be good. Here, the role of school principals is mandatory as they are the ones who represent the community as scholars.

**I:** Good. For your kebelle and other sectors to have a good spirit of collaboration, what thinks need to be done to maximize your capacity?

**P:** What we need is to link our kebelle with different organizations like NGOs, to bring market oriented vegetable production. The other thing we need is to get training. We are not saying that we like to get training in Korem. We don’t have the stand that the training our kebelle members get should be in Korem. What we need is a well experienced trainer for the four villages I am administrating.

**I:** Let us come to the last question. Do you have anything you would like to say from what we have discussed?

**P:** You know the issue of pregnant women is getting attention; adolescent girls’ harassment is prevented.

**I:** What do you feel since harassment is avoided?

**P:** We are really pleased. The living standard of the community is being changed. We are seeing some changing their house from grass made to wood and metal made ones. The fact that there are still undone assignments in this community is a point that should be considered. We need to think to what level the community should be transformed. It is such concern which revives daily in our mind. Otherwise, we are happy to see many things done in our kebelle. Just we are thinking about the remaining assignments.

**I:** What about the lesson from the sectorial collaboration?

**P:** Still, we are happy. I am happy to lead this kebelle. This kebelle has produced many scholars due to the wise handling of our mothers and finally their children. Those children have grown well. I know there are doctors who come out from this kebelle and who are currently serving their community in Ayder hospital and Addis Ababa. We have scholars who are highly paid and get around 35,000 birr and who do their business even abroad. This gives us a big lesson. When mothers get good nutrition, good situation and good handling of their children, then the probability of getting a well refreshed mind will be high. Again, we are planning on the way forward. That is it.

**I:** Good. Now, we know that there are opportunities though we don’t make the best out of them. In your thoughts, what are opportunities do we have to improve the nutritional status women and adolescents?

**P:** We don’t have unmanaged opportunities.

**I:** Let us, for example, take Hashenge. Hashenge is a green and watery area. Therefore, can we say that we have taken the best out of Hashenge Lake in producing vegetables and other fruits?

**P:** Yes. There is nothing left behind. Those activities that should be done are done. We didn’t left any job behind that brings to our mind a sort of bad regression.

**I:** Even in the multi-sectorial collaboration?

**P:** Yes. Everything is tried and done.

**I:** At last, what do you like to talk a general message about maternal nutrition, then?

P: It is what I talked about so far. The ideas I have discussed are repeatedly coming *[participant a little bit laughed]*

**I:** Okay. I have finalized my agenda. Thank you very much for your time and for the massive information you gave me. Thank you.

**P:** I also thank you.

**Summary**

- For pregnant women and lactating mothers to be healthy they should eat adequate food and adolescent girls should keep their protocol.
- Mothers are the primary the first group of people affected by inadequacy of nutrition. The effect on the mother has a big effect on the baby or child.
- Safety Net, along with the effort of SURE and REST, are priority programs in addressing maternal nutrition related problems and their health.
- Backwardness in terms of education is the main community factor affecting the nutrition interventions intended to improve maternal and adolescent health.
- Multi-sectorial collaboration, involving the community as a whole, is key to the success of the different efforts exerted in improving maternal nutritional status and of course their health as well

The End
