# Supplementary material for: Barriers to utilize nutrition interventions among lactating women in rural communities of Tigray, northern Ethiopia: An exploratory study
Source: PLoS One. 2021 Apr 30;16(4):e0250696. doi: 10.1371/journal.pone.0250696 (PMC8087028; doi:10.1371/journal.pone.0250696)
Supplement: S2 File — (ZIP) [file pone.0250696.s002.zip › S2_File.Doc/Community level Key informants/048_IDI_WDA_Fina Ruwa kebele Samre woreda.docx]

**Operational Research on Adolescent and Maternal Nutrition in Northern Ethiopia**

**Introduction**

Hello my name is kiros, I am from Mekelle Universty; we are conducting a research on the factors that influences the nutrition of mothers and adolescent girls in collaboration with the regional health bureau and UNICEF. Year participation is very valuable; the information that you tell us will be used to improve nutrition programs and services for women and adolescents in the region and the country. We will not share your names when we report our results. The interview may take 1-2 hours and I would like to thank you for taking the time to speak with us today. You have the right to withdraw at any time and I will use tape recorder. Are you voluntary to participate for the interview?

**Yes** No

| **Section A: Interview details**   1. Zone: **South Eastern Zone of Tigray** 2. Woreda: **Seharty Samre** 3. Kebele: **Nebar Hadnet** 4. Name of key informant: **Miss Awetu Aebyo** 5. Institution of key informant: **Women development Armey** 6. Interviewer name: **Kiros Tedla** 7. Date of interview: **06/11/2017** 8. Interview start time: **11:00AM** 9. Interview end time: **12:37AM** |
| --- |

| **Section B: Interviewee professional information**   1. Sex    1. **Female**    2. Male 2. Highest level of completed education.    1. No formal education    2. **Primary education**    3. High school    4. College education    5. Bachelor degree    6. Master’s degree    7. PhD 3. Discipline or field of educational training    1. Agriculture    2. Health (MD, nurse, health officer, midwife, pharmacy, etc)    3. Nutrition    4. Public health    5. Food science    6. Other (specify):**Women Developmental Army** 4. Current position: **HEW** 5. How long have you been in the current job/position:    1. ______ Months    2. **1** Years |
| --- |

**I:** Interviewer **P:** Participant

**Section I**

**I**, **what do women do to stay healthy in this community or worerda?**

**P**. they keep their hygiene, they do health examination and vaccination. They should also take food particularly pregnant women they should eat what they prefer. However; females from village cannot eat what they prefer to eat because they do not have the resource. They also eat cereals when they are interested. Pregnant Mothers also follow their pregnancy in health facility and deliver here in order to avoid bleeding and save their children. This is because in the past a number of mothers were died because of bleeding and the community belief this was because of evil eye. But now mothers give birth at health facility and saved her life because there is no bleeding which was known as evil eye. This is if the mother is following her pregnancy in the health facility she will be given treatment for her low blood and give birth normally. The infant is also free from any disease if he/she is born in the health facility as the infant will be kept with clean clothes like blanket and washed by hot water. But if the infant was born at home; he/she will be kept using dirty clothes and washed using cold water which exposes the infant in to cold diseases “Kurree Mietaw”. They also assess overall health of the infant like they will check his eye, and his face; they also follow the mother and measure her blood; if become low they give her treatment or medication and become normal.

**I, Ok, you told me about hygiene how it is related with health?**

**P.** They should clean their clothes, and if they get they should also drink clean water. This is because if they drink dirty water they will suffer from diseases and they will also transmit the diseases to their child.

**I, How is food related with maternal and adolescent health?**

**P**. Mainly this is for pregnant women compared to lactating and adolescents as lactating mother as an example can eat what they get but pregnant women cannot eat any food as during pregnancy; these mothers loss their appetite due to the nature of pregnancy as they hate food during this time. When pregnant mothers visit health facility; they will be measured there nutrition and blood by the health extension worker and given treatment for their low blood. They also advice them to follow their pregnancy in the health post until six months but for above six months and above they will send them to the woreda or Samre.

**I, What about lactating mothers do to stay healthy?**

**P.** they keep their hygiene and their children. They are advised by the health expert or HEW to wash their hands and their breast before lactating their child. They are also advised to wash their hands when they feed their child and also wash after any activity to hold their children meaning they wash their hands before holding their children.

**Ok,** **I, What about adolescent girls do to stay healthy?**

**P.** they keep their hygiene as these girls only go to school and have no children and are healthier compared to mothers. They do not go to health facility except if they are to be married for testing to check whether she is above 18 years old or below. Except for this they are always on their education; thet is why students are always clean as their families take responsibility.

**I, what are the common nutrition problems in the community for pregnant, lactating and adolescent girls?**

**P.** there is a mother who has got pregnancy within four or five months of their first delivery in our kebele. As a result the infant is suffering from severe malnutrition and has extended abdomen. There are many children in our area suffering from moderate to severe malnutrition and some of these children are treated in the health post and given Plamplet and FAFA. But the other groups which are severely affected were sent to the woreda for better treatment and were given milk. However; the fafa is not enough in amount and even not distributed equally as there is health professional related corruption as it is not clear where the fafa has gone even though the woreda has allocated. As an example; we all women collected a petition and went to the higher officials to change the health professional and now they have changed the health professional. And even though they did not start measuring the nutritional status of the mothers as they had worked for two months; the new HEWs are very good in delivering health education on institutional delivery, environmental and personal hygiene like keep your toilet your body clean. They also teach the mothers to have separated rooms for animals, chicken and human being. They told us for WDA to be a model by working on the above mentioned things and teach the mothers during our coffee ceremony discussion.

**I, Ok, thank you; you told me that there is malnutrition on children like they have extended abdomen, what about on pregnant women?**

**P.** there are mothers who did not eat food and affected by malnutrition and becoming very thin. This is because they did not eat food appropriately and then their health will be affected. These mothers are not given fafa by the HEW and could not afford to get what they prefer to eat then they will be resulted in malnutrition. They also think of giving care to their children and their husband first but forget even their lunch or dinner.

**I, What about lactating mothers?**

**P.** there are mothers who could not produce milk and feed their children because of nutritional problem. The mothers were given fana previously but stopped now even though there are mothers who need the help. These mothers are not able to produce different foods in their home as they have lack of resources. For example the HEWs told the mothers to give them porridge and soup made of even “Taff” but this could not always present in the village. Even if present; it is not made with good hygiene and quality; hence the child will be exposed to parasitic diseases such as Ameoba and Gardia. Hence; there are children died or severely ill because of nutritional problems. There are mothers even severely affected by malnutrition mainly those mothers who were not eating well during their pregnancy because they hate eating even very well prepared foods but love to eat cereals which could not be available easily with low cost; as our area is law land we could not produce and get vegetables easily as well. Hence, there are mothers who died because of malnutrition; for instance I do have an information that there were two mothers died because of malnutrition in our kebelle.

**I, What about on adolescent girls?**

**P.** they are not affected as they can eat what they get and they can also eat by preparing for themselves as they do not hate food like pregnant mothers. They only go school and keep their hygiene as their families think and give care for them and the families are living for their children they give first to their children and eat the remaining. That is why adolescents are not affected by malnutrition. As an example; in this morning adolescents go to school by eating their breakfast what is available in the morning but the mothers will not eat as they should prepare food themselves and their family hence will eat after they are so starved.

**I, How do you express the extent of malnutrition on mothers?**

**P.** There are mothers who are severely affected by malnutrition as they cannot eat what should they eat. For example; as a WDA I lead 30 mothers and together with another male who leads the male; and here there is one pregnant mother who have got pregnancy within one year of her first delivery hence her child is suffering from malnutrition and have extended abdomen. I told her to come to this discussion but she refused as she is afraid of getting health professionals because her mistake. Even though she looks good compared to her baby she is also affected by malnutrition. There are also mothers who are affected by malnutrition as they could not eat food appropriately and because of giving birth early as they are ashamed to tell what they are interested to eat.

**I, do you know mothers who admitted to health facility because of malnutrition?**

**P.** Yes, including myself; they send me to the woreda health facility for better treatment because my nutrition status was very low. There are also other mothers with pregnancy of more than six months sent to the woreda health facility for better nutritional management.

**I, What about micronutrient deficiency like anemia, night blindness and gioter?**

**P.** there are mothers of both pregnant and lactating which are affected by anemia due to poor feeding or nutrition as we are living in the lowland which is very hot and having very temperature. There are mothers mainly of pregnant who have head ach and blured vesion during standing because of anemia.

**I, What about night blindness and goiter?**

**P.** there is night blindness but such mothers are given treatment from the health post and cured.

**What was the treatment?**

**P.** I do not know the name but it is drop of liquid from very small plastic container.

**I, what about goiter?**

**P.** there is also goiter but not very common like anemia and night blindness. This is because of shortage of iodine salt; but there is education that every mother should use iodine salt but not the old salt which have no iodine that is why the prevalence of Goiter is decreasing. In the past it was highly prevalent among both male and female. The old salt is only used to prepare “Shiro and Berbere”.

**I, Do you think there could be an association between nutrition and occurrence of non-communicable diseases mothers and girls? What are these in this community?**

**P.** there is no such diseases in our community.

**I, do you think the women or adolescent girls have height proportional to their age? Why?**

**P.** No, they are very thin and short. Because our area is not comfortable for living as it is lowland with very high temperature.

**I, Is it related with nutrition?**

**P.** Yes it is related with nutrition as these mothers do not eat very well but if they would have been eating good nutrition they would have very good body or fat. For instance; look at me I have two children but I am 18 years old and look like a mother with more than 30 years old; even the HEW told me that they could not believe you that you are 18 years old because I am looking very old women. Hence; this is the result of poor nutrition. We have no water around us we bring water from remote areas. As a result we cannot wash our clothes always and we cannot prepare our foods at any time and we cannot keep our body clean. Hence; we are affected by different diseases as we drink water coming from river.

**I, How water is related with your nutrition?**

**P.** yes it is related as if there is no water we cannot prepare different foods and keep our hygiene. For instance when I was pregnant there was no one to bring me water; hence I simply eat what I have got and give my child without washing my hand which exposes him to different diseases. So if there is water we keep our body clean, we keep our food materials clean as well. When I give my first birth; I delivered at the woreda because of shortage of food but in my second delivery I did not have difficulty as I was keeping my body clean and nutritionally good. Hence; water is equivalently important to other nutritional values. I have gone with some of the pregnant mothers to the woreda and I have observed that mothers coming from high land areas give birth immediately but those who were coming from low lands were giving birth very late and have a lot of sufferings and pain.

**I, Why this difference?**

**P.** I have raised this question during our meeting with HEW and they told us that the pregnant women coming from the high land areas eat vegetables, they do have enough water to keep their hygiene and to cook; they can also eat what they want like milk even though milk was highly produced in low lands now days there is scarcity due to scarcity of resources. But when we come to our area we do not have rest as we move with our animals; work with our partner or husband and travel very long distance to bring water; hence becoming exhausted and un able to eat food. This with the high temperature the mothers make very poor nutritionally and to become suffer from malnutrition.

**I, do you think the women or adolescent girls have weight proportional to their age? Why?**

Yes, mothers have light weight compared to their age. Example, lactating mothers are losing their weight during lactation mainly after six months; similarly also pregnant mothers. Because they do not eat appropriately as ordered by the HEW. But adolescents are relatively normal compared to mothers but when we compare with adolescents living in the town they are not similar meaning they have light weight as they travel long distance for schooling and because of the environment which is very hot.

**I, why do think is the weight of mothers decreasing with increasing age?**

**P.** this is because they born a lot of children; hence they could not eat food what they prefer as they have no time to prepare, they give priority to their children and they may also have lack of resource which could feed for both their children and themselves.

**I, do think the community have sustainable food for one year meaning can they feed their family without shortage for one year?**

**P.** there is no particularly this year we only got one rain in August and as you can see there is no crop production. Hence; the community is suffering from lack of food security because of lack of raining.

**I, How frequent is this happening and why?**

**P.** it happens alternatively mainly in the last five years between the years. As an example last year the raining was very good and there was no shortage of food as there was enough crop production. But this year there is no raining and the crop as well; if we do have enough raining we do not have food in security or famine**.**

**I, What do you think is the reason?**

**P.** I do not know exactly but I believe that it could be from GOD. But there are agricultural workers who told us that it is because of deforestation as we are cutting the forests for different purposes like for food preparation.

**I, Who is the most affected by the food insecurity?**

**P.** all are affected most notably mothers and adolescents. As I said it earlier mothers are affected because they are more concerned for their families than for themselves. Adolescents are also affected because next to mother adolescents give care to their families. For instance if I have one female she will help me in giving care to the children and have no rest. That is why they are also affected by food insecurity as both the mother and the adolescent cannot get the adequate food at the right time and they also give the available food to the children. But when we see the rich people in our community; as they have no problem in getting what they want like milk to drink or food like honey and meat to eat; they have no problem in nutrition.

**I, Which of the nutrition problems are risk for pregnant? Lactating mother? And adolescent girl?**

**P.** For pregnant women; they are highly affected by malnutrition and there are also anemic pregnant mothers as a result the infant in side will also become malnutrition. This is because they have a problem in feeding; HEW teach the mothers to feed three or four times a day during pregnancy and also to have extra-rest but they do not do that because of the reasons I have told you before. For lactating mother there is malnutrition because they focus on working not in feeding of their child and themselves; even though the child is crying because he/she had hanger; she will say to her daughter; please go to outside until I finish my work. Hence; this will result in malnutrition for both the mother as she cannot eat at the right time and her baby. Adolescents are also affected by malnutrition as most of our community had several children they cannot afford to feed them appropriately; hence the children including adolescents are affected by malnutrition as they are told to eat only a piece of injera. They are suffering from the consequences of malnutrition like wasting and stunting. But now there are changes because HEW teach the mothers and fathers on family planning but in the past a single mother could give up to 12 children where they are facing challenges on feeding and caring the children as they have no enough food, clothing and others.

**I, Which women groups are the most affected by the above mentioned problem?**

**P.** pregnant mothers are the most affected followed by lactating mothers and to some extent the adolescent girls as they can eat any available food.

**I, Why pregnant women?**

**P.** Because they are serving for two life meaning for themselves and the fetus inside as the fetus in side also needs to feed; hence they need extra-meal to eat. The other reason is they also give priority to their family than themselves to feed first and have no time to eat three or four times a day as they passed the time in preparing food to all their family and then they will feed only once.

**I, Why pregnant women?**

**P.** because lactating mothers are participating in multiple activities like preparing food, harvesting with their husband, and other agricultural activities working equally with her husband. Hence; they have no time to eat food even though they are expected to eight four or five times. Hance; the child will not get enough milk as she had not get the desired food.

**Section two**:

**I,** **what kind of nutrition interventions are in place to improve health of pregnant women, lactating women and adolecents?**

**P.** previously Plamplet and Fafa with oil were given to severe and moderate malnutrition mothers and children before two years ago but not now and last year. Children with severe malnutrition were given “mitmita” or Plamplet and fafa for moderate malnutrition child after screening in the health post. Mothers with pregnancy and those having less than six months of child and having malnutrition after measurement were given fafa. The mothers were advised to make porridge and soup at least three times a day for themselves and their child. This was given at our kebelle but had several problems as this was not distributed equally for all the children as there were children who are with malnutrition but did not get it because of corruption. But these interventions were very success full. Example; my brothers daughter was affected by severe malnutrition even though we give her milk and finally we brought her to the health facility and she had given Mitmita or Plamplet followed by Fafa and become healthy with good nutritional status.

**I, How do they give the interventions?**

**P.** they first ask them if they have any type of diarrhea or vomiting if they do not have any of this but he/she is very thin during measuring; they will give him/her the food. Mothers are screened using measuring material who put on their arms to measure nutritional status.

**I, are pregnant mothers advised to visit HS for check up and services?**

**P.** yes, they are advised by HEWs very well. Pregnant mothers are advised to visit health facility monthly. They are screened their blood and if have low blood they give them tablet I do not know the name to improve their blood, they also advice pregnant mothers above six months to go to the other health facilities for better management.

**I, Do you think it helps them? How?**

**P.** yes, because if they follow their pregnancy they will save the their life and the life of their child as they will get all the services like the treatment for anemia in which many of the mothers died because of blooding. They also advised on how to sleep and how to keep their hygiene.

**I, What about on getting extra-meal and rest for pregnant women?**

**P.** They get an advice as I have told you earlier. They are advised to eat three times if they were eating two times a day before during pregnancy. They are also advised to check whether they do have the resource before pregnancy if so they will take contraceptive until she has got the resources. They are also advised to get enough rest during pregnancy.

**I, What about on lactating mothers about extra-meal and rest?**

They are also advised similarly to eat three or four times in order to produce enough milk for their children. HEWs advice to the lactating mothers to eat as much as possible and get enough rest in order to produce enough milk to keep their children healthy and normal in nutrition.

**I, Are pregnant mothers get screened for their nutritional status? how? do you think it would help for?**

**P.** yes they are screened monthly for both pregnant and lactating mothers with child of less than six months. This will help for the mothers who affected by malnutrition to get fafa from the health facility and improve their nutritional status. Children are also screened their nutritional status and for those who are severly affected they will be given Plamplet and those at moderate they are given fafa. However; only very few are helped by these programs only two from our Kushet for example. Hence; a number of children and mothers are not getting the help because of lack of the resources.

**I, How do you evaluate the effect of the interventions?**

**P.** They are very effective particularly Plamplet it is very effective; Fafa is also effective and Children in our kushet love fafa very much. As an example; there are children who were severely malnutrition but after they are given Plamplet and fafa; they are very effective as the children had improved nutritional status and eat the local foods very good. There were two children who were severly affected by malnutrition and even the people where assuming as they will die but when they were given Plamplet and Fafa they become healthy and their nutritional status had improved very well. After this there are mothers who said the current government is creating chance to live again like GOD.

**I, How is the fafa prepared?**

**P.** It is prepared in the form of porridge and soup and given for both mothers and their children. They are advised to prepare three times; at the morning; at middle of the day and at night. But I cannot say that our community is using fafa to feed the children correctly as I have seen mothers give their children in the form solid which could not be eaten easily.

**I, About on adolescent girls; is there nutritional screening?**

**P.** there is no nutritional screening and intervention on adolescents.

**I, what about advising on food diversification during pregnancy?**

**P.** yes there is counseling and they are advised to eat what they want to eat and get rest. But mainly those pregnant mothers who have taken fafa are advised to eat four times a day like at the morning, at four, at eight and 12 hours until the fafa is finished and you to only eat yourself not with family members.

**I, On other types of food preparations and feeding?**

**P.** There is no any other counseling rather than the above mentioned.

**What is your role on these prioritized interventions in this Woreda?**

**P.** On pregnant women we will first assess and register all the pregnant women in our kebele during house to house visiting. After assessing we will improve their awareness through discussion on ANC and the importance of follow up. Then when they come to the health facility or health post we will start ANC for those with a pregnancy of 3 or 4 and above and we will screen their nutritional status. As I have mentioned above we will classify in to sever, medium and normal malnutrition and for these mothers with sever and medium they will be given FAFA. Similarly all children will be screened for nutritional status but first they will be vaccinated after 45 days. And then all the infants with age of less than five years will be screened for their nutritional status and we put under OTP according to the measurement I have told you before.

**I, What about on iodine salt ustilization?**

**P.** yes there is counseling and most of the people are using iodine salt. And we are advised to add the salt after we finish cooking in order not be melted and aired because of the high temperature.

**I, What about for adolescents?**

**P.** For adolescents we will teach them at school for those who are in-school adolescents and we have selected female leader there so we communicate with her and give them education on contraceptives. For out-school adolescents we will meet them during house to house visiting and teach them. We are also measuring for both the in-school and out-school adolescents their nutrition status by measuring their BMI but still they are normal but this screening is not for all adolescents.

**I, on getting advice on nutrition sensitive agriculture such as home gardening?**

**P.** Yes we are getting the advice from agricultural experts but we could not practice because of lack of water in our area. The government was trying to build water reservoir cannels but the community is afraid of malaria by cannels and also do not remain for a long period of time due to high temperature in the area; hence the water will be evaporated within a short period of time. The agricultural

**What about on the need to participate on the safety net program?**

**P.** Both mothers are participated based on their family status meaning if they are poor they are included but if they have two oxen and above they will not be included. Pregnant mothers with pregnancy of six months and above are not involved in the work done. Lactating mothers are also allowed to rest for ten months after delivery. There are also adolescents, elders and those who have no family are also included in safety net.

**Who provided the safety net program? Is there any other help?**

**P.** this is provided from the government through the kebelle and there is also emergency aid or “Hitsuts Hagez” because of the drought this year; and currently the government is studying the people who need the aid through the kebelle leaders.

**I, What is the difference between safety net and emergency aid?**

**P.** safety net is a five year program and the family members are given the aid for six months but emergency aid is given for 9 or more years depend on the drought status. But the criteria used during selection to include the people are similar for both programs.

**I, What about emergency aid for pregnant, lactating and adolescent girls?**

**P.** both pregnant and lactating mothers with children less than five years old are given first or prioritized for the aid.but the problem is that not all needy people do not get the help as most of the aid like fafa are remaining in the woreda. For example in 2008 E.C one full store of fafa was become out date.

**I, Is there any advice given to pregnant, lactating, and adolescent on water, sanitation and hygiene services?**

**P.** Yes, pregnant mothers are advised to keep their personal hygiene by washing their clothes and their body. Lactating mothers are advised to wash their hands and their breast before feeding their baby. They are also advised to wash with three days interval and keep clean their baby or children clothes. Adolescent girls are educated about hygiene in their school through their teachers.

**I, Is malaria common here? If yes, are pregnant mothers getting advice on the need to use ITN? Why? Who Advice them?**

**P.** yes there is malaria. Pregnant and lactating mothers having with infant or child of less one year old are given ITN first. This is because the pregnant women cannot given the treatment if had malaria immediately and the treatment may not be successful. Hence, she is aviced to sleep inside the ITN and to remove different unwonted plants or grasses, to illuminate smell pons having water and stagnant water.

**I, On lactating and adolescent girls about ITN?**

**P.** Lactating mothers are advised and given the ITN because of the child they have as he/she is very small. But there is no special treatment given for adolescent girls; they are equally treated with their other family members.

**Is there any treatment given to prevent intestinal parasites on pregnant, lactating mothers and adolescents?**

**P.** there is no treatment given to all mothers and the girls. For example; my son was suffering from Teania spp but I brought him the health facility and treated by my own money.

**I, Is there school feeding program for adolescent girls?**

**P.** there is no school feeding program but in 2008 EC there was school feeding program initiated by one teacher and students were given porridge made of fafa for those students below grade six.

**I, Which of the above mentioned interventions which is the most important for pregnant women? Why?**

**P.** the follow up and check up of pregnant and lactating mothers; Plamplet and fafa are very important to both mothers and children. Because as I have told you earlier they have shown very visible changes on the nutritional status of the mother and her child. Compared to milk; plamplet and fafa had produced very visible changes on the child nutrition but the problem is they are not available in enough amount to all the children.

**Section 3**

**I, What are the special things should women do to stay healthy in the community? ( during pregnancy, lactation and adolescence)**

**P.** pregnant mothers should follow their health in the health facility and get advice from HEW on how to get extra meal and rest. For example; if the pregnant mother is rich; she can eat egg and drink milk and also feed their children by feeding different combination of foods like vegetable, milk and cereals. Hence the child will become nutritionally normal and healthy. The lactating mothers should have enough rest and should not bring their children to the work place as he/ she will be bitten by mosquito or may be exposed to other injuries. They should also keep their hygiene.

**I, Do women in this community change their diets when they are pregnant and lactating?**

**P.** no they do not change their diet. They feed with their family similar to what they previously eat.

**I, But, do you believe mothers meaning both lactating and pregnant mothers should change their diet when they have got pregnancy? Why?**

**P.** yes, because eating with the whole family and eating alone is very different. If the mother eat alone she could eat as much as possible but if eat with their family she could not get or take the actual or the needed amount of food.

**I, What could you advice the pregnant and lactating mothers to eat?**

**P.** They should eat vegetables, animal products like milk and others, meat and other local crops like cereals.

**I, Is there any food which should not be taken by the mothers ( pregnant and lactating mothers)?**

**P.** I do not know.

**I, What affects women diet during pregnancy and lactating?**

**P.** They are affected by the absence of the food items at closest distance. For example: in our kebelle there is no vegetable and cereals produced if pregnant mother needs these components of food she should go to the woreda city which is 30km from this.

**I, What about gender disparities in women’s diet before pregnancy, and during pregnancy, lactation and adolescence?**

**P.** Both pregnant and lactating mothers do not eat or prepare food if their husband is not present because of our culture. And this is not only affects the mother but also the adolescents as they are not getting the food.

**Section 4**

**I, Have you ever gone for nutritional screening during routine service delivery? If you want tell me about your experience?**

**P.** Yes, I have been gone through nutritional screening and have been assisted or given fafa as my nutritional status was lower because I was not eating food as was not interested to eat food. Hence, I have given birth normally as my nutritional status was normal. But there are many other mothers who had got difficulty to delivery due to their poor nutrition. They were also given me advice on getting extra-rest. At least I have been screened three times within three months.

**I, What are the challenges related with attending routine service delivery?**

**P.** there are mothers who do not attend the routine services delivered by the health post. This si because the mothers thought that there is bias in service delivery by the HEWs like during distribution of the Fafa as they feel that their children had equal weight with the other children included in the aid or fafa and Plamplet.

**I, Is there any challenge related to the health service provider which affects the use of routine health service by pregnant and lactating mothers?**

**P.** There is no any problem related with the health provider as they are not the only one participating in the delivery system and selection of the affected children. We are also involved through the one to five channels. We get the information from the HEW and disseminate to the mothers as well.

**I, You have told me previously that lactating and pregnant mothers are given different interventions; do they know why they are included?**

**P.** yes, they know about their inclusion because they are also advised to come to the health facility for check up and advised to feed their child correctly as indicated by the HEW. For example: pregnant mothers are advised to increase their food intake so as to produce much milk and feed their child correctly.

**I, Does the given interventions improve their food intake and their health?**

**P.** yes, it improves the food intake of the mothers and the child. For example; lactating mothers had improved their food intake to the local foods after they have given fafa. There is also change on the amount of milk produced and their nutritional status when measured by the HEWs.

**I, are pregnant and lactating women beneficiaries from of soft conditionality of the safety net program?**

**P.** yes they are beneficiaries as pregnant mothers are excluded from the activities of the safety net program starting from four months of pregnancy and after delivery they are free from the activities done for 10 months of the lactation time.

**Section 5**

**I, Do you think that delaying the age at first birth to after 18 is better for both the mother and the infant? Is it promoted in the community? Can you tell me who is promoting?**

**P.** Yes I heard and it is well promoted in the community as there are education on such issues in schools with help of female affaires and all students are now becoming aware of the issue of early marriage. Teachers and leaders of the kushet are the primary players about early marriage. It starts from the kushet and then goes to the teacher and then to the kebele leader who gives the final decision. For example; there were many adolescents with age of lower than 18 years who were stopped by these leaders from early marriage.

**I, Do you mean they are working very well and there is no early marriage in this community?**

**P.**  yes, there is no early marriage now because the rules are very strict which includes jail and money punishment.

**I, Do you know about spacing of birth intervals or family planning? Is it promoted in the community? Can you tell me who is promoting?**

**P.** yes there is family planning now as there are family planning services given for one month, one year, three year, and it is well promoted the last 2 years as there are many mothers utilizing the services. But still there are mothers who do not utilize the service we do not know from where is the problem is whether it is related with their husband or themselves. Their reasons given were that the contraceptive is not appropriate for their health as it may affect their face, it may not goes with their restlessness as they have no rest, it may also can cause infertility in the long run.

**I, Can you think of any opportunity to prevent early marriage and increase birth spacing?**

**P. T**he WDAs as we are so close to the community and working in the community. We can also use the different celebrations or social events where mothers may be gathered like during Sunday in the church, WDA meetings with mothers, public meetings during safety net or emergency aid studies. For example, last week one health professional had been here to teach the community and we were mobilized all the community members of both male and female. And it had been very success full event. Both husband and wife were trained together.

**I, Do you think involvement of male is important? Why?**

**P.** yes, because there were male pressure in the house when the female tries to take contraceptive. Therefore; if they are aware of the importance of the contraceptive the female can easily understand him and helps her to take contraceptives. Then the mothers are advised to bring their husband during their visit to health facilities for family planning.

**I, what kind of community conversations or massages discuss women’s and adolescents nutrition?**

**P.** there is no discussion on nutrition

**I, Any other groups that provide nutrition information for women and adolescents in this community?**

**P.** there are only health professionals like HEW who teach on hygiene and how we can keep our hold clean and the materials but there is no education given regarding nutrition except for mothers who take fafa. She tell them how to prepare the food for their children and for themselves.

**Section 7**

**I, How can we improve maternal and adolescent nutrition in this community?**

**P.** I have no idea but if mothers have given enough rest starting from six months of pregnancy as they have no rest mainly in our village; they work every activities in the house hold. There should be enough amount and consistent aid of fafa and plamplet to all the children as there are infants with malnutrition but are not given. Pregnant mother should be the primary which should be helped as they had carried two lives; and then lactating mother as she is also giving milk to a child. Finally; adolescent girls are not at high risk compared to the mothers as they have no child they carry on. But compared to adolescents in the urban; those adolescents living in the village have poor living condition in terms of nutrition and health. This is because the adolescents living in our area are living in very low land area with high temperature and travel long distance for schooling. Hence; If we need to bring the needed change on maternal nutrition we have to work on providing aid to pregnant mothers together with the service constantly.

**Summery points**

**Section one:**

- Medium to severe malnutrition and Anemia are common among mothers and children..

**Section two**

- Free health services during ANC and delivery; Plamblet and FAFA for both pregnant and lactating mothers and their children are the interventions in this woreda.

**Section three**

- Pregnant mothers should visit HF for follow up and check up during pregnancy and should get etra-meal and rest during and after delivery.

**Section four**

- Nutrition screening is routinely performed as it is important to determine the nutritional status of the mothers and their children and to give them the nutritional interventions..

**Section five**

- The programs or policies targeting delayed marriage and birth interval are working very well.

**Section six**

- The only groups that provide nutrition information for women and children who had received fafa are HEWs.
- **Finally I have finished my questions and I would like to thank for your time, patience and answering all the questions. Thank you very much!!! Thank you!!!**
