# Supplementary material for: Barriers to utilize nutrition interventions among lactating women in rural communities of Tigray, northern Ethiopia: An exploratory study
Source: PLoS One. 2021 Apr 30;16(4):e0250696. doi: 10.1371/journal.pone.0250696 (PMC8087028; doi:10.1371/journal.pone.0250696)
Supplement: S2 File — (ZIP) [file pone.0250696.s002.zip › S2_File.Doc/Community level Key informants/050_IDI_agricultural extension_Fina Ruwa kebele_Samre Woreda.docx]

**Operational Research on Adolescent and Maternal Nutrition in Northern Ethiopia**

## In-depth interview responses of the agriculture extension head

**Introduction**

Thank you for your acceptance of the informed consent form and for taking the time to speak with me today. I have questions to ask you which were prepared in advance. The discussion will take 1-2 hours. If you have any questions before we begin please feel free to ask.

**Section A: Interview in details**

1. Zone: South Eastern Zone
2. Woreda: Saharti Samre
3. Kebele: Nebar Hadnet
4. Name of key informant: Meresa Birhanu
5. Institution of key informant: Agriculture extension office
6. Interviewer name: Mekonnen Haileselassie
7. Date of interview: 01/03/2010
8. Interview start time: 4:40 AM (local time)
9. Interview end time: 6:30 PM (local time)

**Section B: Socio-demographic and basic data of qualitative study participant**

| **Socio-demographic characteristic** | **KII** |
| --- | --- |
| Sex | Male |
| Age | 26 |
| Educational status | Bachler degree |
| Occupation/role in the community | Agriculture extension head |
| Service year | 3 years |

**Note:**

I: interview

P: participant

**Section 1: Common maternal (pregnant women, lactating women and adolescent girls) nutrition problems in the community**

**I:** What do women do to stay healthy in this community/woreda?

**P:** They have to get diversified foods; safe drinking water and they have to keep the sanitation and hygiene of their environments. We understand that nutrition is very important for the mothers and children to become healthy and active in their overall life. Therefore, we are doing some nutrition activities in collaboration with the health sector such as, environmental sanitation, different agricultural food production and the consumption level of these products by mothers.

**I:** What are the common nutrition problems in the community for women and adolescent girls?

**P:** Being the community is not well educated; there is a problem of understanding to take the inputs available in the woreda like fertilizer, chicken, good quality seeds. This Tabia is very vast in size compared to others in the region; therefore, it is difficult to communicate every household on time. It is also very hot area that the community is not as such active to apply what they have been trained.

Unbalance of height and their age which is low height among children in this Tabia is very common. Because this Tabia is very hot, most of our fat is melted by the existing high temperature. It is very difficult to observe fat children in this area. Therefore, stunting is caused by the high temperature of the area. In addition to the presence of high temperature in the area, there is no enough supply of fruits, vegetables and other diversified foods.

**I:** How the high temperature causes stunting?

**P:** The children of this area are forced to keep livestock at the grazing field by their parents. They don’t get food on time; the whole day the children are engaging in keeping livestock at the very hot field; therefore, the hot temperature with poor feeding style causes stunting to children. All the children of this area are assigned to keep livestock. Mostly they start keeping livestock from the age of seven years. You may observe the children at the field, and how much all of them are engaging in keeping of livestock at the high temperature area. Although children are enrolling to school, they run both businesses side by side. For example, if the children stay at school in the morning, they keep the livestock after they come back from school after noon.

The other problem in this area is the shortage of water source. The mothers both the pregnant and lactating once are traveling more than one hour to fetch water; so this is not difficult to guess how much the mothers are tired enough to eat food. Therefore the presence of high temperature in association with high workload of mothers in different activities could cause short and thin body condition.

**I:** How do you see nutrition in case of micronutrient deficiencies (anemia, night blindness, goiter…)?

**P:** Goiter is not our Tabia’s concern; I didn’t see any sign of goiter here in our Tabia being it is low land; where as goiter is common in highland areas.

But a disease like night blindness is observed; and it may correlate with the shortage of water. For example if don’t wash your face, many dirt particles could enter into your eye in a simple way. Many victims are observed in the case of night blindness in our Tabia. The presence of hot temperature could also an effect on the prevalence of night blindness.

Anemia is also observed in the pregnant and lactating mothers. In this Tabia, there is no access of fruits and vegetables; mothers couldn’t get these products. The local name of anemia is “dimbawe”; once the mothers’ body becomes edematous and they sense dizziness of the mind. Since there is no water source, there is no any irrigation activity. But in case of animal source consumption, the pregnant and lactating mothers have an opportunity to get milk although its amount is very low; mostly the other products like egg, butter and meat are intended for market purposes.

**I:** How do you evaluate nutrition and diet related non communicable diseases?

**P:** The diet related diseases are not common in our Tabia. Being there is no enough food consumption and the existing of high temperature in the area; diet related diseases are not common. Our problem is the shortage of food that I have discussed, such as the sign of edematous is clearly observed in pregnant and lactating mothers. In case of children, fafa is provided after they have been measured their MUAC. There are many children that are provided fafa. But I did know whether pregnant and lactating mothers are measured and get provided fafa.

**I:** How do you evaluate nutrition with food security?

**P**: This Tabia is the food in secured area; due to the lack of rainfall in the area, drought is commonly observed since the past four years. The community could not survive independently out of the food soft net programs. There are many problems that could face to the pregnant and lactating mothers as the result of food insufficiencies; they face high workload to search food for the family. They are doing the field activities like the husbands in addition to the house work responsibilities.

**I:** Which women groups are most affected by these nutrition problems?

**P:** Pregnant women are more sensitive to nutrition problems compared to others; because pregnant women have no chance of travelling from place to place to get what they needs. Especially after five months of pregnancy, they can’t go to markets. Our market area is very far to go easily. If the pregnant women need vegetables or animal products, they have no opportunity to get it easily. The minimum distance from one Tabia to the other is twenty three kilometers. Relatively the lactating mothers have the chance of traveling to far distance like market to get their needs. The same is true in adolescent girls who have the opportunity to travel long distance compared to the pregnant women. In adolescent girls, the level of their knowledge is comparatively better than pregnant and lactating mothers; they get information from the school or other Medias like radio, experience sharing among schools could contribute for their awareness in the area of nutrition issue.

**Section 2: Nutrition priorities in the woreda**

**I:** Do you think it is necessary for your institution to get involved in work aimed at improving maternal nutrition? Explore for pregnant women, lactating women and adolescent girls.

**P:** Yes, because the source of food is from agricultural products like; the crop and livestock production, fruits, vegetables, tubers and the like. These agriculture products are the basic diversified food sources for the pregnant and lactating mothers. Therefore, we produce the agricultural products in collaboration with the farmers; and the health experts could educate the pregnant and lactating mothers how the balance diet food could prepare and how the women consume the food without losing its nutritional content. In collaboration with health expert, we can produce the nutrition sensitive agricultural products at the farmers’ farm.

Currently we introduce high quality livestock breeds to the farmer like begait and Holstein Frisian breeds to get more milk production; therefore, if we agriculture experts educate the farmers to manage and produce high milk from the introduced livestock breeds and the health experts also provide training how the children, pregnant and lactating mothers consume these products to improve their nutritional status.

**I:** How can your institution at this level be involved giving examples from specific projects or work?

**P:** In the crop production part, we are doing the routine activities that we were undertaking before. But in the area of livestock production, we introduce the chicken and begait breeds to the community. Thus the pregnant and lactating mothers could have the opportunity to get benefit from the animal products. In our office, there is no especial focus to the pregnant and lactating mothers or adolescent girls; rather we give concern to the women as a whole to boost their incomes. We also evaluate the introduced livestock products in the households; and the consumption level of the community is mainly evaluated by the health experts. Only we provide especial favour to the pregnant and lactating mothers not to participate during the water and soil conservation campaign and the food soft net program so as to get rest.

**I:** What nutrition services do you spend most of your time on?

**P:** In the side of pregnant women, we focus mainly on the reduction of workload; in collaboration with the health sector like not to participate in the Tabia soil and water conservation campaign and get involved in the food soft net program without participating the work; but this is valued in lactating mothers if the child is below three years of age. Without any discrimination, we provide also special favours to the women in general such as, if a man performs three meters in the soil and water conservation then it is recommended the woman to work only half of him that is one and half meters.

In nutrition interventions, we didn’t allocate enough resources to address the nutritional problem of the pregnant and lactating mothers. By chance if we increase the product and productivity of agriculture, the pregnant and lactating mothers could be benefited; we didn’t also produce nutrition based agriculture products. In our office, there was no any focus towards nutrition; we were only focusing to increase production and to have market oriented agricultural products. But now as I have said before, we have already introduced chicken and quality cow breeds to women households and we are thinking to introduce the home garden vegetables for those who have nearby water access.

There are about one hundred poor women who engaged in the dairy cow and goats program this year; the REST in collaboration with agriculture bureau is working in this program. Therefore, this program will be strengthening by providing technical and material support. We are also working in the environmental sanitation in collaboration with the health experts; we fill up the breeding site of malaria during the summer season and we also undertaken the compost production.

**Section 3: Nutrition interventions that improve adolescent and maternal health**

**I:** What kinds of nutrition interventions are in place to improve adolescent and maternal health in this Tabia?

**P:** Mainly counseling on one extra meal and rest during pregnancy and lactating mothers is the health experts’ task. But in collaboration with the health experts, we allow the pregnant and lactating mother not to participate in the water and soil conservation programs so as to get rest. We provide training to all women through the development group about the production of different agricultural products and how women are getting rest during the soil and water conservation program. We also advise the pregnant women to eat a diversified food like milk, egg, cereals, and pulses which are produced in our Tabia.

Although we strive to introduce home garden vegetables, there is a problem of water source in our Tabia. At present, there are about thirty two women who are engaged in the home garden and natural resource development activities as a pilot project; thus we evaluate its result and if it will effective, the project will be strengthened. The same activity is done in the area of livestock production such as introducing the dairy and chicken production to women households; but livestock production is not a new technology and we found very effective.

The introduction of best stove (ምርጥ እቶን) by GIZ Ethiopia could also contribute on the health and nutritional status of pregnant and lactating mothers; they could not affect their eye and respiratory organ by the smoke of fire wood and they will not travel long distance to search of fire wood since best stove limit the consumption of much fire wood and indirectly it has an effect on the prevention of natural resource deforestation. In all our activities, we focus to the whole women rather than specific to the pregnant, lactating mothers and the adolescent girls.

In adolescent girls, there was food program (fafa) in their school but now it is stopped; in my view, the adolescent girls have the opportunity to get good understanding about nutrition from school and relatively they have better knowledge about nutrition compared to mothers regardless of their practical application at the ground.

We are doing a lot on the area of advising on water, sanitation and hygiene services. In collaboration with the health sector, we did on the prevention of acute vomiting and diarrhea disease in the community though avoiding the waste products around homestead by producing compost, and we fill up the malaria breeding site to prevent malaria prevalence and it is also important to preserve humidity of the farm; we also advice to use the water guard in each households.

In collaboration with water sector, we also advise the household to use water properly and efficiently. We have a common plan with all our respected stakeholders to increase the benefit of the community through the contribution of our efforts.

**I:** In your opinion, which of your programs are being implemented successfully (i.e. in the most effective way?) Why? Explore for pregnant women, lactating women and adolescent girls.

**P:** In the area of nutrition, we didn’t do a lot; since the topography of our Tabia is very challenging to introduce home garden vegetables; it is also very high temperature, needed long distance to get water source, and the awareness of the community towards nutrition is very poor. We agricultural experts had also poor understanding of promoting mothers’ nutrition.

We introduced best breeds of dairy cow and chicken to the household; but we didn’t evaluate their consumption level. We also allowed getting rest during the water and soil conservation program. We did also in the environmental sanitation and hygiene through compost production and fill up the malaria breeding area.

**I:** What are some of the reasons that the programs you mentioned are effective?

**P:** The awareness of the community is improved to demand the new technologies. The collaboration among different disciplines and sectors is increased to promote nutrition.

**I:** In your opinion, which of the programs mentioned above are less effective? Why? Explore for pregnant women, lactating women and adolescent girls.

**P:** In our side, we didn’t effective in the home garden vegetables; the consumption level of the community; the awareness of the community and poor understanding of experts on the nutrition area. Due to the presence of long distance among each Tabia, we have not the opportunity to address all households’ problem to undertake strict follow up. It is also difficult the mothers to come to our office to get advice and training.

**I:** What are the implementation challenges that are specific to delivering the maternal nutrition interventions in the programs?

**P:** Transportation access is the basic constraint to the community and the experts. There is very poor transportation access. We use the traditional ambulance using man labor for ill person and it is long distance from each corner to reach the main road. Our target was also to increase agriculture production and productivity as well as market oriented products; this was the gap of collaboration between the health and agriculture experts. The lack of awareness among the community towards the nutrition importance is also another problem. There is no any difference on the nutritional status of the rich and the poor; even the rich are more sensitive to malnutrition compared to the poor; because the families of the rich are more engaged in the keeping of animals at the field through traveling very long distance and their product is targeted to market.

Since the weather condition of the area is very hot, no one expert is interested to stay here; the turnover among the experts is very high. In this condition the alternation of different activities and documents are not properly transferred. Especially in the health sector, the turnover among the experts is very high; no expert can stay more than one year.

**I:** What challenges are the most important for the improvement?

**P:** Although we are striving to educate the pregnant and lactating mothers, due to the problem of transport we don’t address to the whole mothers; the size of the Tabia is also very vast to cover by the expert. The media of our communication is through the development group. We assemble to the networks and the networks are also to their cells.

Lack of awareness among the community, lack water source and transport access, sense of dependency, high temperature, lack of training to experts are the most problems exist in the area.

Resources like use of insecticide treated bed nets and iodine salt are not the problem of our Tabia.

**I:** For these challenges that you mentioned, can you tell me of any successes or innovations that your office have used to improve maternal nutrition service delivery?

**P:** We didn’t do more innovative activity that use to improve maternal nutrition service delivery; but we tried to provide an advice to the pregnant mother and their husbands’ to get rest during pregnancy. The introduction of best stove to mothers is also a good technology to minimize the problems produced as the result of traditional stove.

**Section 4: Community factors affecting access to maternal nutrition interventions**

**I:** Can you think of barriers that prevent adolescents and women from using the programs and interventions that we have discussed?

**P:** There is great different among the level of educational status of the pregnant and lactating mothers and adolescent girls in accepting the new technology. The higher the educational status of mothers is the better their awareness towards the overall activities like the feeding style of diversified food, introduction of agriculture technologies. There is no transport access to visit the whole Tabias; as I have said before transportation is the main bottleneck of this area.

Some pregnant women are participating in the same activities with their husband during the agricultural works. They cultivate and harvest with no any favour to them; totally they undergo with high workload like housework activities, agricultural work, traveling of long distance to fetch water etc.

Although the pregnant and lactating mothers are allowed to get rest during the water and soil conservation program, they could undertake the high workload activities in their house works.

Some pregnant and lactating mothers could engage in high work load activities and they could also travel more than two hours to work agricultural activities.

Some husbands also inhibit pregnant women from visiting to health centers for the sake of their advantage; they assume that she could stay in the health center for long time and they could face the problem of food preparation for themselves. But there is an improvement among the individuals from time to time.

**I:** How can these barriers are addressed to improve maternal nutrition in the community/woreda?

**P:** We should educate mainly the husbands through different innovative mechanisms like peer to peer training, preparation of award for those who treat their wives in a best approach, getting training from their school students, and repeated training by the experts.

About transportation, it needs the devotion of the experts to support the community. The government should also supply the transportation service and make reduce the size of the Tabias.

**Section 5: Other interventions that influence adolescent and maternal nutrition and health outcomes**

**I:** In your opinion, why would increasing the space between each births and delayed marriage (after 18 years) improve maternal nutrition and hence both maternal and infant health?

**P:** Keeping a woman healthy before she gets pregnant is important for preventing stunting. Healthy women make healthy and strong babies and communities. Therefore, if there is early marriage below 18, there could be delivery problem, fistula and stress to mothers and give birth of stunted children.

In case of birth spacing among children, it has many advantages to the mother and the child; if not, the child could not get the favorite treatment like proper breastfeeding, timely supply of complementary foods and keeping the hygienic status of the child. The mother also becomes stressed to treat the children at the same time.

**I**: What programs or activities promote increased birth intervals in this woreda?

**P:** There are marriage committees in the Tabia and the school director is also member of the committee so as not to give up her schooling; then the committees are inspecting her age before marriage whether she is above or below 18. There are also decisions not to give up her schooling and it is an obligation to check the pairs about their health status (HIV ADIS). If the marriage is undertaken under eighteen years, punishment is expected.

**I**: Can you tell me about any programs or policies in place in this woreda to prevent early marriage?

**P:** As I have said above, there is no underage marriage in our Tabia; the decision of the committees is already valued.

**I:** Can you think of any more programs or policies? Think about political, religious and other influences.

**P:** All the political administration and the religious person are strictly oppose the early marriage of less than eighteen years; that is why there is no any early marriage in our Tabia.

**I:** Can you think of any other opportunities to prevent early marriage and increase birth spacing?

**P:** We have the opportunity that the representative of the committees is devoted enough to prevent early marriage; the schools are also promoting these activities; awareness is already created in every structure of the government.

**I:** How about the space between each birth of children?

P: Here the rule is not strict; although the training is given to the women, its practical application on the ground is not fully implemented. Few women give birth after one year and most of them are giving birth after three years. But they do not want intentionally to delay up to three years. In my view the effect of nutrition could contribute to delay the activity of their cells.

**Section 6:** **Multi-sectoral collaboration to improve maternal nutrition**

**I:** Do you feel it is necessary for your institution to work with other sectors/institutions to address maternal nutrition?

**P**: The importance of collaboration work is unquestionable; we are doing in collaboration with health sector, water and education. We have now an interface to fix our activities among all stakeholders. We share our plans that could work in collaboration with the respected stakeholders and evaluate the final results together after the SMS (subject matter specialist) visit; we have a visit program in the form of SMS from each stakeholder (agriculture, health, water) to observe and evaluate the performance of each activity per our plan. Without supporting of each other it is very difficult to run all the activities of nutrition improvement in the mothers. But our visit and fixation of the interface is not specific to the nutrition improvement; it is the overall activities of the sectors.

**I:** Which other sectors do you feel are necessary to work with?

**P:** The sectors of health, agriculture, education and water are the main actors to improve nutrition among mothers.

**I:** How do you see the other institutions’ roles complementing your role in improving maternal nutrition?

**P:** We can do a lot of activities with health sector; for example to reduce the workload of pregnant and lactating mothers, we found letters from the health sector about their general health and pregnancy status then we allow them to get rest. We also collaborate in the production and consumption of different food types to the pregnant and lactating mothers.

Similarly the water sector visits to every Kushet check the quality of the hand pump waters so as to get healthy water to the pregnant and lactating mothers.

**I:** For multi-sectoral action that effectively works to improve maternal nutrition at all levels, what kind of change in terms of the way stakeholders work together is needed?

**P:** All the responsible stakeholders should set annual plan and share part of the plan to all experts; based on our plan we should visit the activities in each Kushets and evaluate the effectiveness of the activity; corrective measure is also undertaken for the poor performance one. Therefore, the common understanding among the stakeholders is critical.

**I:** What type of resistance to the needed change do you perceive or have you experienced so far?

**P:** In case of the health sector, they are busy in treating patients when you plan to visit the Kushets; this is due to the shortage of experts.

**I:** To what extent does your institution participate in the multi-sectoral nutrition coordinating body at the woreda level?

**P:** Since agriculture is the backbone of the region’s economy, we are doing in collaboration with all sectors. For example we are doing with health, water, and education sectors

**I:** How effective are the coordinating platforms in enhancing multi-sectoral coordination?

**P:** We didn’t face any coordination problem. Except the problem of the transportation access, we all are ready to help the poor with our profession.

**I:** What needs to be done to improve the capacity of these bodies/platforms for effective coordination?

**P:** We should have fixed day to visit and educate the mothers at their Kushet. The common training is very important to understand the subject matter of others’ activity.

**I:** Do you have any other comments on anything that we have discussed?

**P:** I have learned a lot about the nutrition problem in pregnant and lactating women and adolescent girls.

**I:** What lessons have you learnt regarding adolescent and maternal (pregnant, lactating and adolescent girls) nutrition in this woreda?

**P:** In my view, to educate about nutrition to the pregnant and lactating women and adolescent girls; it needs a sort of holistic approach; if they are given training with one sector it may be fragmented information to them; since it is broad and not one sector’s activity. Therefore, integration is an obligation rather than an option.

**I**: What opportunities do exist to promote multi-sectoral coordination of nutrition in this Tabia?

**P:** We do have very smooth relationship and our office is near to one another.

**I:** What opportunities do exist to promote maternal (pregnant, lactating and adolescent girls) nutrition in this Tabia?

**P:** We have the opportunity of very large size of farm land; we can produce a diversified food types during the summer season; and high livestock population that could contribute on the reduction of the pregnant and lactating mothers and adolescent girls in the Tabia.

**SUMMARY**

**Section 1: Common maternal (pregnant women, lactating women and adolescent girls) nutrition problems in the community**

- This Tabia is very vast in size compared to others in the region; thus, it is difficult to communicate every household on time.
- Children of above seven ages are forced to keep livestock by their parents; and therefore, the hot temperature with poor feeding style and high workload among the children causes stunting.
- Both the pregnant and lactating mothers are traveling more than one hour to fetch water; subsequently, the presence of high temperature in association with high workload of them could cause short and thin body condition.
- Night blindness is observed in mothers due to the shortage of water source among the community in the area.

**Section 2: Nutrition priorities in the woreda**

- We introduced chicken and quality cow breeds to women so as to be benefited them by the livestock products.
- We fill up the breeding site of malaria during the summer season and we also undertake the compost production by keeping the environmental sanitation.

**Section 3: Nutrition interventions that improve adolescent and maternal health**

- We allow the pregnant and lactating mothers not to participate in the water and soil conservation programs so as to get rest.
- The introduction of best stove (ምርጥ እቶን) by GIZ Ethiopia could also contribute on the health and nutritional status of pregnant and lactating mothers; by reducing their workload to search for fire wood. They could not also affect their eye and respiratory organ by the smoke of fire wood. It has also an effect on the prevention of natural resource deforestation.
- Every household mothers use the water guard to prevent any contamination of water

**Section 4: Community factors affecting access to maternal nutrition interventions**

- Transportation access is the basic constraint to the community and the experts.
- We use the traditional ambulance using man labor for ill person and it is long distance from each corner to reach the main road.
- Our target was to increase agriculture production and productivity as well as market oriented products; this was the gap of collaboration between the health and agriculture experts.
- Since the weather condition of the area is very hot, no one expert is interested to stay here; the turnover among the experts is very high.
- The size of the Tabia is also very vast to cover by the expert.
- Mothers cultivate and harvest with no any favour to them; totally they undergo with high workload like housework activities, agricultural work, livestock keeping and traveling of long distance to fetch water etc.

**Section 5: Other interventions that influence adolescent and maternal nutrition and health outcomes**

- The different innovative mechanisms to educate husbands in the promotion of nutrition like peer to peer training, preparation of award for those who treat their wives in a best approach, getting training from their school students, and repeated training by the experts.
- All the political administration and the religious person are strictly oppose the early marriage of less than eighteen years;
- Awareness is already created in every structure of the government about the ban of early marriage.
- Most of mothers are giving birth after three years of the successive children. But in my view, the effect of nutrition and high temperature could contribute to delay the activity of their cells rather than the mothers’ awareness.

**Section 6:** **Multi-sectoral collaboration to improve maternal nutrition**

- We have now an interface to fix our activities among all stakeholders (agriculture, health, water, school).
- We share our plans that could work in collaboration with the respected stakeholders and evaluate the final results together after the SMS (subject matter specialist) visit.
