# Supplementary material for: Barriers to utilize nutrition interventions among lactating women in rural communities of Tigray, northern Ethiopia: An exploratory study
Source: PLoS One. 2021 Apr 30;16(4):e0250696. doi: 10.1371/journal.pone.0250696 (PMC8087028; doi:10.1371/journal.pone.0250696)
Supplement: S2 File — (ZIP) [file pone.0250696.s002.zip › S2_File.Doc/Community level Key informants/051_FGD_Men_Keyih Emba Kebele_Samre Woreda.docx]

**Operational Research on Adolescent and Maternal Nutrition in Northern Ethiopia**

***Date: Nov 10, 2017***

**Focus Group Discussion with Men, Keyh Emba Kebelle, Seharti Samre Woreda, Tigray.**

**Section A: Interview Details**

1. Zone: South eastern zone
2. Woreda: Seharti Samre
3. Kebelle: Nebar Hadnet; Kushet : Keyh Emba
4. Interviewer name: Mengistu Mitiku
5. Date of interview: Nov 10, 2017
6. Interview start time: 10:40 AM
7. Interview end time: 1:20 PM

**Section B: Participants’ General information**

| **Participant** | **Age** | **Marital status** | **Educational level** | **Occupation** |
| --- | --- | --- | --- | --- |
| Participant 1 | 51 | Married | No education | Farmer |
| Participant 2 | 35 | Married | 4^th^ grade | Farmer |
| Participant 3 | 33 | Married | 6^th^ grade | Farmer |
| Participant 4 | 28 | Married | 3^rd^ grade | Farmer |
| Participant 5 | 25 | Married | 10+1 | Farmer |
| Participant 6 | 50 | Married | 7^th^ grade | Farmer |
| Participant 7 | 69 | Married | 2^nd^ grade | Farmer |
| Participant 8 | 43 | Married | 2^nd^ grade | Farmer |
| Participant 9 | 50 | Married | 1^st^ grade | Farmer |
| Participant 10 | 39 | Married | 7^th^ grade | Farmer |

**Main interview**

**I:** Interviewer

**Pn:** Participant [Focus Group Discussant]; **n** = Code assigned to the discussant

**I:** Good morning. I am Mengistu Mitiku from Mekelle University. Thank you for coming and showing willingness to participate in this study. This is a research on maternal and adolescent nutrition in Ethiopia being conducted by Mekelle University in collaboration with Federal government. This discussion can take as long as 2 hours. We will take any information from you because you know everything in this community. When we discuss, every idea is assumed to be correct and the idea of discussant could be criticized by another one. Thai is it.

**Section 1: Common maternal nutrition problems**

**I:** Let us start from the first question. It is about mothers and adolescent girls. So, let us start from pregnant women. For pregnant women to be healthy, what are they doing?

**P1:** No response. No sound. [Discussants remained silent].

**I:** Is the question clear?

**P2:** It is clear

**P4:** It is clear

**P8:** It is clear

**I:** Your ideas please; one by one.

**P8:** We are talking about pregnant women. The services pregnant women are getting are good. When we come to the agenda ’what pregnant women are doing’, of course it is their issue, the issue of preventing themselves from everything. I think they have a good way of keeping their personal hygiene good and even the nutritional stand they and their foetus have is being followed in good manner by pregnant women.

**I:** Continue number 9. The question is ‘what do pregnant women do to stay healthy?’

**P9:** Good. When we think of what mothers are doing, the first point is they are doing well in making optimum birth spacing. The second point is they are keeping their personal hygiene. The third point is they usually go to health centre every four month, six month to have their health status checked. There are such kinds of preparations in pregnant women. When we come to the way they wear their clothes, they are situational. They choose clothes depending whether they will go to big town or remain here in their village. These were my ideas. That is it.

**I:** Additional ideas. We are talking about what mothers do or are doing to be healthy? It could be pregnant women, lactating mothers and adolescents.

**P3:** Well. I am number three. What I want mention what pregnant women are doing is that they have a good understanding of long birth spacing. Mothers undergo health services follow ups every three months. When the time approaches, they go to health facility to give birth. It is in health centers that they give birth. Moreover, they keep their personal hygiene though it is not fully applied as the urea they are living in is a rural village. Anyways, they are doing such kinds of things. They are becoming well educated, they are becoming conservative in giving birth and they have developed the habit of going to health facilities for health checkups. These are my opinions.

**P7:** Pregnant mothers, like what has been described so far and what government directions are in place, have a program on the seventh of every month. During the program, every pregnant woman will check her weight and thinness. Moreover, they will check the health of their fetus. They will get advice and exchange idea among themselves. The stage the fetus is in, the position the estimated date of delivery and other things will be raised and discussed. Women were facing many dangers. Therefore, to prevent the problems and dangers, they have started to follow their health, the health of their baby in the womb which could be the position or the other conditions of the baby; they also check whether there is one or two babies in the womb. Of course, there are pregnant women we trust the Almighty GOD and ignore health check-ups. There are pregnant women wh don’t want health follow-ups at health center. They think of the way their parents have reached this time and want to follow this direction. We have such untransformed pregnant women who still didn’t avoid backwardness. Those pregnant women who faced a pregnancy-related problem and have the experience of the real problem will go to health center as they have the belief that they will be helped. So, they go in such a direction and provided they confirm that they are healthy, they will come home and keep following. Those with pregnancy related problem will remain in the health center and receive their medical care. Those who have pregnancy-related problem and children at home will receive the care either at home or health facility.

**I:** Additional ideas on pregnant women?

**P:** They remained silent *[P5: That is all. That is all. No new idea]*

**I:** Good. So far, many ideas are raised on what pregnant women should do. Why is that? Why do pregnant women do all the things you mentioned?

**P4:** The first reason is to keep their health in a good situation. The other reason is to make their baby healthy in addition to their health.

**P2:** Mothers take the principle that family members will be fine and healthy provided I am healthy. We know what has been the intention of mothers in the previous times. It is not only for the baby that they bother. They bother about the whole family. They assume that if they become sick, all members of her family will be sick. That is it. Whether she has five or six children, they consider the whole family.

**P5:** I will take the ideas so far described. So, as described about health and personal hygiene, I really agree. The other and main reason is that mothers want the baby they are going to deliver to be healthy citizen and leader of the next generation. That is it. That is why pregnant mothers are striving to be healthy.

**I:** Do you a different idea?

**P9:** That is it. What our brothers mentioned is the reason behind.

**I:** Okay. Let us go lactating mothers. What do lactating mothers do to be healthy? Your ideas, please.

**P9:** Well. When we consider lactating mothers, they undergo medical follow up every 7 month and they bring their babies to health facility to get food like plampnet. They work for their baby. They strive for their baby to get breast milk and other necessary foods like what I mentioned before. They also think of the avoidance of sex in the next six months after delivery. This is to make the baby healthy, to prevent the baby from breast milk free of colostrum. If a baby of child took colostrum, he/she will be weak in his knees; his/her back will be ineffective. Moreover, his brain will not be productive. Therefore, lactating mothers continue to fully care their child until the next six months. It is not only breast milking their baby, but they ensure the neatest milk. They properly breast milk their baby to make him/her healthy. In return, this brings joy to the mother.

**I:** Okay. Still we are on lactating mothers. What do lactating mothers do to stay healthy? This is the agenda. So, we have heard ideas from number 9. If there are additional ideas, you are welcome, please.

**P8:** What lactating mothers are doing is one they keep their personal hygiene and their baby’s hygiene also. The other is on food intake habit. They are good in such cases. If the baby is feed properly, he will be okay and finally the mother will also be okay. If the mother is neat, then the baby will get pleasant service from his/her mother. Anyway, personal hygiene should be given priority as it is a vital activity for the growth of the baby.

**I:** Okay. Number 8 has shared us what lactating mothers have to do to be healthy. He told us about personal hygiene and nutrition-the way lactating mothers feed themselves. Any other ideas?

**P6:** As he said, the main activity lactating women are doing is the issue of family planning. As mentioned before, a women she not become pregnant until her child become strong and healthy. This is being properly practiced among lactating mothers. That is all. Because a woman should be become pregnant until the youngest child becomes two years old. This practice is in a good condition among mothers. That is for the benefit of the child. If birth doesn’t take place until the next two years, the baby will be strong and healthy. In such practices, I can say mothers are being changed very well. That is about my opinion.

**P3:** Everything is mentioned. May be, I want to add on he feeding practice lactating women follow. In the next six months after delivery, there will not be any complementary feeding practices. Mothers feed additional food to their child after six months.

**I:** Excuse me; your idea is very good. But, could focus on the lactating mothers themselves, what they should do to remain healthy?

**P3:** Okay. If it is for themselves, to prevent any loss of blood from their body, they remain careful and if that happens, they directly go to health facility. Previously, they were facing the problem of blood bleeding. Really, it was a horrible time for pregnant women to face bleeding. But, these days, they are aware of most of the things and they really prevent is by visiting health centers ahead of time.

**I:** Additional idea or a different idea?

**P1:** Well. Of course I am a young man of the current day’s generation. I don’t know more than what our elder brothers and fathers here. For example, let us take my wife. She gave birth twice. But, these two children came to this earth with five your interval. Today’s ladies are aware of the burden of short birth interval. Our mothers were in a difficult situation in the previous times but sun has started to shine for us now days.

**P5:** They have to eat adequate food to produce more milk for their baby. They have to replace the milk they have given to their baby. They should help themselves and next they can help their children as well. They try to eat different types of food like ‘Kita’ and milk, porridge. Anyway, they do what helps themselves to be strong and if they are healthy and strong, their babies will be healthy.

**I:** What about adolescent girls? What are they doing to be healthy?

**P2:** Generally, today’s adolescent girls spend their time at school. They are lucky. The school says, we don’t want to let our students (adolescent girls). The kebelle administration says any adolescent girls should not get married before 18 years. Sometimes, fathers put pressure on their adolescent girls. If their wives don’t have one who helps her/his mothers, they put pressure. Fathers say there should be at least one girl who helps her mother. If she says no, then permission is given to her to go school and follow her educations. The overall message is that they spend most of their time at schools to get basic lines of knowledge. Moreover, they keep their personal hygiene and they are aware of the fact that they should get tested for HIV before they get married.

**P5:** What adolescent girls are doing is that they don’t care about the culture we have. They are ignorant of marriage until they reach 18 years. Most of them follow the modern day’s life. If they are requested to get married, they usually refuse because they want to complete their education and reach somewhere so that they will independent.

**P7:** What they are doing is mentioned by my brothers. The other thing they are doing to make themselves healthy is one they use family planning methods so as to make free from unwanted pregnancy. They don’t want to see any interruption in the academic path they are in. You know ladies always face challenges when they go out of their home. It is this fact that forces them to use family planning methods. Who knows, they may encounter sexual harassment by their male counterparts. Therefore, some of them are using contraceptive methods. Even when the need arises, they are using because they don’t want to terminate their school life. This is one of the different ideas I have. The other idea I have is when their parents force them to get married, they say no and force their parents not put pressure on them. That all about the ideas I have.

**I:** Any new idea?

**P9:** Today’s adolescents are undergoing immigration from their village to big towns and cities and finally to abroad.

**I:** You are telling us that adolescent girls immigrate from their village to big towns and cities. Does immigration make adolescent girls healthy?

**P9:** Yes. They migrate because they want to change their life using the better income they get in the country they migrated in. It is life. When they migrate to a country with a very good development, they really change their life as we observed from the experiences of their girls.

**Section 2: Common Nutrition related problem**

**I:** What are the common nutrition related problems in this community?

**P1:** One of our problems is that we don’t have adequate food items. We have really this problem.

**P4**: Before a couple of weeks and even at this time, there is a disease called watery diarrhea that resulted in because of lack of neat water and good nutrition. I mean that if we don’t use clean water and food, this disease occurs. The other problem is that our life is not food secured. If there is shortage of food, diseases like water diarrhea will occur as it is related to food contamination.

**P8:** Yes. This community has a problem of food insecurity and is worsened by the current drought**.** This food insecurity brought us malnutrition where some people with thinness morphology are being observed. The main problem is the occurrence of watery diarrheal disease which is closely linked with lack of personal hygiene. Many communities have become sick and some had gone west as the drought continues. There is no any improvement. That brings about malnutrition.

**P3:** Currently, there is no oil and sugar. Due to this, women are forced to eat food with no oil in it. This is really a very unwanted problem. If mothers do not eat adequate food which is pleasant to eat, that will harm their body as it is not confortable without oil. We don’t know what has happened to the disappearance of oil and sugar in this community.

**P5:** There is no good quality of water and if there is no good quality of water diseases like the watery diarrhea will take place. Another problem is thinness which is linked with eating inadequate amount of food.

**I:** Which groups of women are easily affected by the problems you mentioned?

**P10:** Malnutrition, for example, attacks everybody. But, the main ones are children.

**I:** Why children?

**P10:** Because children are not well developed and matured and therefore are easily attacked by shortage of food. They are very delicate and therefore are very prone to diseases related food shortages.

**I:** Additional ideas and thoughts?

**P7:** In addition to children, lactating mothers are also attacked as they breast milk their baby and lose many things from their body. Moreover, pregnant women are also attacked as their body is in a weak state due to the pregnancy they have. Therefore, all the groups of community members I mentioned are easily attacked by food related problems. The other group is elder ones as their body already weakened due to their age. People with disease already in their body are also affected by shortage of food and its consequences.

**P9:** Anemia is another disease.

**I:** How severe are the nutrition related problems that occur in mothers in this community?

**P7:** Due to the diseases related to nutrition, mothers couldn’t contribute in the development of this community. Since their body is weakened by those problems, they will be force to be out of the development related activities in this village. They simply spend most of their time at home doing some easy work activities. If they tried to work in some developmental activities in their village, they will further be weakened as their body is already affected. The weak state of body they have is also finally exposing them to dangerous problems which lead to death. At first, their body will be weakened and when they encounter any disease, the problem worsens and finally death will take place.

**I:** Okay. We are talking about women and adolescent girls. What about communicable diseases? Do women in this community have a problem of communicable disease?

**P9:** Well. The common communicable disease is, in the current situation is AIDS**.**

**I:** Is AIDS common in this community?

**P9:** Yes. It is common in this woreda.

**P6:** The common communicable disease in this community is malaria. Malaria is common here particularly in the months of June up to September. Females are easily affected by this disease as their body is weak due to different reasons.

**I:** How do you related malaria with nutrition?

**P6:** Malaria attacks you especially when you are in a state of starvation, particularly pregnant women and children. These groups of people don’t usually get adequate amount of food. So, they are prone to malaria. Of course, it is not only due to the fact that pregnant women and children don’t get food. Malaria occurs in its optimum season.

**I:** Another idea?

**P3:** Well. It is said by my brothers. But, I want to tell you that malaria doesn’t only affect those who eat inadequate amount of food. It also attacks many group of people. The main idea is that malaria easily attacks those who are malnourished. That is the fact behind. That is it. Yes, it is common in May and June and of course in August and September. All these months are when malaria occurs. Therefore, the attack that occurs in those who are malnourished and those who are not is different. It is the person who in malnourished state who will be prone to the disease and finally get the burden of the disease, malaria.

**P2:** Watery diarrhea is another communicable disease which is currently affecting people in this community, particularly pregnant mothers and children as these groups of people don’t get adequate amount of food.

**I:** What about diseases like stunting and wasting?

**P7:** Yes, we have such problem. In our village, most of the dwellers are wasted unlike those from urban areas. Ours are most of the time thin. There are also individuals who are screened and told to go other health facilities for further diagnosis and treatment and advised to consume more milk and milk products to recover from the nutritional status they are in now.

**I:** What about food security issues?

**P1:** Let me speak about this issue. All my brothers here didn’t taste the product of heir agriculture. There was no rain, no water and frankly speaking we are in a good position. We are in jeopardy. We have many unmet needs. It is this fact which is forcing us to be prone to disease. This is all I wanted to say.

**P4:** We spent time of drought this year. Government has been supporting us previously. But, this year we don’t have any aid that we received from our government.

**I:** What about your agricultural products, your own products?

**P3:** Even the agricultural product we have got is inadequate. Because you know, those areas that got rain in the summer are different from those who didn’t have rain. Due to this, the amount of food we eat has decreased. Those products that got adequate rain are good for food unlike those that didn’t get adequate rain. Anyway, we are disturbed as there is drought here in our village.

**I:** How do you describe the occurrence of such drought?

**P8:** Well. The drought we faced this year occurs rarely. Of course, we have spent times of good season in the last ten and twenty years. This year, we have got rain only in the month of August. We couldn’t get rain in the other months. The farmer has no option of expecting good product from the agricultural products. We simply have become desperate. It is really difficult. Anyway, we don’t need to disturb our farmers in this village. We expect our government to help us this year. As a leader of this village, we are giving our farmers a sort of hope as I have a good trust on our government.

**P7:** The other issue is the supply. We have been getting some resources for our daily consumption like sugar and oil as our government was distributing all those resources I mentioned previously. But, at this time, we couldn’t get such things.

**I:** What are the reasons for not getting such resources?

**P7:** Our kebelle is very far away from our village. The administrative bodies tell us to come to the centre of our kebelle which is Fina Ruba. To reach at this kebelle, you need at least three hours on foot. Even they tell us to come in groups to get sugar and oil. Anyway, it is difficult for elder ones and even the amount of money we pay for transport which is approximately 40 birr is very big figure for us. This is possible provided there is adequate transport. Due the distance to the centre of our kebelle, dwellers of this community are being served at this time. Our community has decided not to go to Fina Ruba. We know oil is a food. Sugar a very important commodity for children and those who are sick. There are people who spend their meal by taking water diluted with sugar.

**P9:** We have a problem of clean water supply for us for drinking and for our cattle. All the water sources we have are not functional at this time. We don’t have adequate food and water for our cattle, adequate water for ourselves. This is what I wanted to add.

**I:** Do you have additional thoughts?

**P:** P6, P9 and P10 responded [That is it, that is it, no new idea].

**I:** If there is no any different view, let us go to the next question. Okay. So far, many ideas were mentioned. Let us talk about nutrition related intervention for mothers and adolescent girls which could be health facility-based or community based intervention. In your community or woreda, what interventions are in place that support pregnant women, lactating mothers and adolescents?

**P4:** As far I know, there are no nutrition related interventions in this village. Previously, aids like provision of Fafa had been given to mothers and children. But, at this time, it has stopped. I remember there was a sort of Fafa aid to those lactating mothers who are malnourished before one or two years. From that time onwards, I couldn’t see any aid.

**P3:** I think what my brothers have said is correct. Previously, there was a targeted aid of body building food items for pregnant women and lactating mothers. Right then, the service was not interesting and today it has stopped. Due to the termination of the body building food items like Fafa to mothers and children a more severe problem has occurred in our village.

**I:** Others opinions on what your woreda or your kebelle is doing to improve the nutritional status of mothers and adolescents?

**P6:** I think the question is what your woreda is doing to improve maternal and adolescent nutrition. Previously, there was pregnant mothers screening and accordingly distribution of source food items like Fafa. But, it is stopped these days though mothers have become malnourished. The option pregnant women have when things related to nutrition is above their scope is to go to their family and get served. That is it. There is no any activity that our woreda is doing for mothers and adolescents. We don’t know the reason why the aid has disappeared.

**I:** Can we view the issue on the other way round – Do pregnant women, lactating mothers and adolescent get a sort of nutrition related assistances at health facility level?

**P7:** The help they receive is that they will undergo diagnosis. If even their children have a problem of malnutrition, they are advised how to feed their children and how to in general handle their babies. Previously, pregnant women and lactating mothers were undergoing screening so that the necessary food items which will build the body of the mothers had been given. These days, there is no such system at our health facilities. The screening will made and if malnutrition problem is detected, she will be advised what to eat, and what to drink at home. But, at home there is no any nutritious food. This area is a dry area. Therefore, the aid which our mothers were getting is decreasing and decreasing. Otherwise, they are getting every advice in health posts and health centers. We cannot deny the advice pregnant women and lactating mothers are getting from health professionals. They advise mothers to come to health center and get medical care. If the service doesn’t exist or if it is above their scope, they advise mothers to go Fina Ruba Kebelle or Samre for further diagnosis and treatment. But, for this community member it will problematic to manage such referrals.

**I:** What else? For example advise to use iodized salt?

**P8:** When I consider, for example, my wife, we have got an advice that iodized salt is very important for the development of the brain of child and even for us all. It is good to have such advice from the health center. Our farmers have learnt from such activity and right these days utilization of iodized salt is increasing and of the normal salt is decreasing. They advise us that iodized salt is very important for health and even they give us health education and advise us which is very important and which is very dangerous. Health centers are serving us to such extent. The issue of nutritional items provision at health centers up on undergoing screening was functional and when a baby is born, the same practice was in place in that we had the opportunity to go to health center and take food-like items for the baby. But, such services are not functional today. We don’t know the reasons behind.

**I:** What else? What sort of advises are being given? Such things are mentions so far like advising screening mothers. What else? Let us consider other things other than aids given or being given.

**P4:** They advise us that iodized salt protect from diseases.

**P2:** Anyway, the advice mothers are getting is that when we go to water and soil conservation activity, they are allowed to take rest. There are many pregnant women in our village. Healthcare workers go home to home to screen mothers and exempt them from water and soil conservation activity. This is a good benefit as our environment is sunny and hot. Of course, there are mothers who are reluctant to speak to health professionals that they are pregnant and there are mothers who undergo pregnancy testing by taking the initiative by themselves.

**I:** What else? What other services are mothers getting? For example about deworming?

**P7:** Yes, we are getting drugs of different kind. Mothers are getting such drug which is supposed to kill microorganisms in water and food. The drug is added to the drinking water and is given to every segment of the village. Mothers and under 5 children are given drugs. Even other drugs are given like those eye disease that are transmitted from other animals. The drugs are protect you by killing bacteria from your body.

**I:** What about services like going home to home and screening mothers for their nutritional status and acting accordingly?

**P9:** Well. Mothers get a sort of help. Children also get help from health centers. If his/her mother has gone west, they will be given milk powder for his/her survival.

**I:** What about mothers for themselves?

**P9:** For mothers, the provision of Fafa for moderately harmed was in place. But, at this time, mothers don’t have such services.

**I:** Why is such services terminated, the provision of Fafa for mothers who are screened and found to have nutritional problems? Why?

**P1:** Of course, it is not me but those at the woreda and Kebelle level that know the reasons behind. For me, I say that the administrative bodies of our kebelle are not good guys. I would say what our woreda has sent us disappearing in our kebelle. Of course, health professionals might have a good information about it. That is all my ideas.

**P6:** Okay. The reason behind the disappearance of nutrition related food for mothers and children is not clear. Yes, those children whose mother has died are being supported. But, we need to consider in the most general way. Previously, mothers and children were screened and their problems were acted up on and for those who are severely malnourished, they had been give plamphinet and those with moderate malnutrition are given Fafa and oil. This service in general is stopped. May be, it was in 2000 or around 2000 E.C., there was community based food aid like Fafa as there was a drought. But, this has disappeared with the reasons behind not clear. Our kebelle is at the boarder of region three. The center of our kebelle, though you don’t know it, is in Fina Ruba. We don’t have kebelles beyond that kebelle. That is it. The kebelle taken three hours to reach. Our main problem is that the reason for disappearing is not still clear. That is it. The healthcare workers undergo screening. But, the problem is that the provision of nutritional items according to the detected problem has stopped. Even the Fafa which was obviously collected in our kebelle has disappeared. This is what we are saying.

**P4:** I had been a guard here in this health post. More than 30 or 40 children had got their weights checked and more than 10 of them had the opportunity to get plamphenet and others will be promised to get Fafa. But, they don’t get. We don’t know what has happened. We don’t know where the around 40 quintal of Fafa is. The healthcare workers are doing their activity, checking the weight of mothers and children, but our kebelle is canceling every aid women and children might. Of course, there was such aid before two years, but it had been stopped by our kebelle for reasons of unknown kind.

**I: Other ideas,… other ideas**

**P8:** Okay. The main issue is described by brothers. That is all. Generally, we can blame the weakness of our kebelle but we have to be sure that we ourselves also have the weakness. It is good to note this. The issue of provider and client is clear. When our government sends us something that belongs to us, we have the responsibility to follow and ask. Residents are demonstrating a gap in that they don’t unite and go to the kebelle to make sure that their belongings are in place and will be given to them. I know that the kebelle is distant and they have the practice of delaying complaints coming from the community. There is a good supply from the government. The problem is in the kebelle. The condition of corruption is well on the surface. Anyway, there is a gap in the basic grounds of the administration of the kebelle. Administrative bodies of the kebelle are not working from the bottom of their health for their community. Really, the government is good in helping its people. The government participates during droughts and also exerts its efforts in this community for the sake of establishing good governance and the advice on how to utilize our property. That is it.

**I:** Good. Good. Now, it has been mentioned that many things are being done for mothers and the community as well, and many other things are also missing from the kebelle. The issue of advice for others by healthcare workers, the fact that women get advice to visit health facility and the issue of screening to identify which are malnourished and which are not. Even the health education given on the utilization of iodized salt is mentioned. Of all the services and interventions mentioned so far, which ones are the most important ones? Which interventions are helping mothers a lot?

**P4:** In this regard, I would say the only service mothers are getting is the routine screening and counseling service they have at health posts and health centers. There is no very helpful intervention that supports mothers.

**P4:** I share what has been described so far. Apart from that, the most important task I want to raise is the utilization of ambulance. The service given for pregnant and lactating mothers by ambulances is good and important.

**I:** What else? Women and nutrition related interventions

**P9:** The most important activities as to me are, one is the health education given on family planning. Previously, mothers were giving births six, eight and twelve months spaced. But, these days mothers are giving birth after five or six years of the previously born child. This is among the model activities done and as per the government’s plan. The other is the interventions done for malaria like through the use of drug and bed nets. These two activities have been done to keep mothers healthy by our government.

**I:** Okay. Let us generalize the idea. Many ideas are raised. The issue of nutrition counseling, health service utilization, sanitation, provision of complementary foods like Fafa. Many of the activities have been successful and some has failed. So, could you tell us why some of the activities were not successful, the barriers?

**P3:** The barrier is lack of concern, not showing the responsibility. The lack of concern of the administrative bodies of our kebelle is the big barrier. The people have chosen them to lead but they don’t care. They care about themselves, about their skin. They don’t care about the problem of the community. If they don’t care, what is the importance of choosing those bodies to lead the community? Why TPLF fighters have sacrificed themselves for the benefit of their people?

**P5:** It is lack of action. It is due to lack of action that many of the activities are not going well. Human beings, you know, fall down when they walk provided the road is not good. The same is true for this kebelle. There is a sort of barrier from the kebelle that prevented people of the village from getting work done for them. Therefore, it is lack of action. The Faf has disappeared because there is nobody who is concerned about the actions needed. Otherwise, the government is working well.

**I:** additional ideas?

**P:** No sound ***[****Participants remained silent]*

**Section 3: Perceived needs of mothers**

**I:** Let us go to the next question. Next, we will talk about the things mother perceive as necessary for them. What are the things pertaining to nutrition that mother say ‘this is what we need’?

**P1:** They say I need pepper, orange fruits and lemon when they become pregnant. They tell you to bring them different things, especially when after three or months of heir pregnancy.

**P7:** Mothers need rest though they go to work since they have nothing to eat at home.

**I:** Is that what they need?

**P7:** Yes. They need it. Rest is the main thing they need. The other things are like food clean water. As they are weak, they need all these things. They also need transport services as they couldn’t walk a long distance.

**P2:** What they think is necessary is that they want to use family planning methods. They need also clean water and personal hygiene.

**P4:** The adolescent girls need that when they finish 10 grade and were unlucky and were not promoted to preparatory school, they think the kebelle has to prepare a sort of work or activity for them. At 7 and 8 grade level, when they get married, they feel that their rights are violated by their parents and they seek other options.

**I:** Other ideas?

**P3:** What they think necessary for them, not what we think is that the assistance from healthcare professionals. They want healthcare professionals to serve them on time, to care them properly. Moreover, they want healthcare workers to think of them every think they need.

**I:** What is the role of husbands like you to improve the nutrition related issues of their wives? Your views please.

**P9:** The role of husbands could be like bringing clean water, good clothes and fire woods. Other things like preparing sauce and helping their activities in the agriculture. These are the things we assume should be roles of the husband.

**P8:** The role of the husband is to supply the house hold. The wife has the desire and ability to feed family members. The role of the husband, therefore, should be to provide every necessary thing to the mother. This should be the role of the husband.

**P5:** The main thing is pressure. Men have the practice of putting pressure on their wives. This should be avoided. The main thing should be to communicate and to understand to one another. That is the main point. The male counterparts should avoid this pressure so that everything will be good.

**P2:** What we need to help our wives is that when you come home from daily farm activity, wives serve every family member. They don’t even eat before we eat, our children eat. They simply bother about family members. Therefore, we need to make them free of any work activity as they are working in a sort of care for family members.

**I:** What should mothers do with regard to their food intakes during their pregnancy period?

**P4:** Our pregnant mothers do eat one type of food, food prepared from millet. That is it. They don’t change the food they eat because they don’t have.

**P10:** As far as I know, pregnant women in this community decrease their practice of eating foods. Even some of them will have a distorted appetite. They hate food.

**P7:** Well. Pregnant mothers are advised to eat additional food by healthcare providers. But, pregnant women in this community do not act like that. They don’t eat more food as they don’t have more. They cannot bring from downtown as it far way. The agricultural product we have is not adequate as this is a hot area where there is no rain and therefore no food. For example, my wife always don’t use sauce if she is pregnant, even I don’t help her to use it. She instead use and eat dry types of foods. She uses pepper and sometimes dispenses salt on injera and eats.

**I:** Why is that?

**P7:** She hates such sauces. Even I brought her to health center and she couldn’t. You see since we don’t help them, they are being affected. I have felt it for sure. I worry when I bring her to health facility. That is really difficult. The support we have for our pregnant women to consume more food is minimum. She simply tries being herself by eating like roasted grain and then skipping everything. When they don’t have the option, pregnant women eat everything they get. Otherwise, we don’t have the habit going to market to bring them better and delicious food

**P3:** Things are theoretically remembered. We don’t have the real practice as our community is uneducated and back warded. We don’t do it. We simply are putting pressure on them not eat the type of food they need. We should not say what we don’t do for our wives. That is it.

**I:** What about pregnant women, do they change the diet they use?

**P10:** Lactating mothers in this community do change and increase the food they eat as they give more to their baby and have a starved body. So, they increase their diet.

**P4:** What we know is lactating women increase their diet. They eat a different food from the one they ate before. Since they breast feed their child, they have the practice of eating variety of diet to increase their body strength and the amount of breast milk they produce.

**P5:** If they are lactating mothers, they change their diet. Provided they get adequate food items to be prepared, they even consume porridge with butter. After a couple of days, they start to consume roasted grain and bread which considered in this community as important foods. These are my suggestions.

**I:** What types of foods are not recommended to pregnant women and lactating mothers? Let us say mothers as a whole. So, what do you think are the food recommended for mothers and those foods not recommended?

**P9:** The recommended foods for mothers are honey, milk and porridge. All these are recommended energy providing foods. The other one diluted and boiled teff along with sugar. This is common in our community. The foods not recommended to our mothers are like meat as it makes delivery process very complex and difficult. Eating meat prevents mothers from giving birth easily. The other is injera prepared from millet as it is dry and makes mothers dehydrated.

**I:** Additional ideas? What food should be eaten what should not be eaten?

**P7:** When we talk about the foods that should be eaten, it is difficult to say something as we don’t have the information and the knowledge. We should not talk surely as we don’t have knowledge of list of foods recommended to pregnant women and lactating mothers. In our cultural setting, the foods recommended for mothers are red teff in the form of porridge and during pregnancy period, foods like milk and egg are recommended because they should not develop deficiency states. They are advised to take every thing they have at home. Regarding the foods not recommended for pregnant women and lactating mothers, we don’t have the information. We might have to read and able to know the different food items not recommended in the future. I myself don’t know them. I know that mothers have to eat different food items like vegetables and fruits.

**I:** Additional idea?

**P4:** What we know in this community is pregnant women should not drink ‘Areki’. Otherwise, we don’t have the information about the foods not recommended. It is a known culture to prevent mothers from drinking Alcohol like Areki. Otherwise, we know nothing.

**P3:** Okay. As mentioned so far, pregnant women should not drink Areki. Moreover, pregnant women should not utilize ‘shenafche’ which is powerful.

**I:** What factors affect pregnant women and lactating mothers’ intake of food?

**P4:** It is determined by the nature of the women. Some pregnant women eat more food and others eat less food. Even there are pregnant women who hate eating food. Anyway, it is the natural behavior of the person that determines when we talk about the factors that influence pregnant women’s nutrition.

**P1:** It is the health state of the mother that determines.

**P3:** We don’t have the idea. It is up to the healthcare workers to know. It is inside the women’s mind. We simply say ‘she has hated this food’ and therefore, we don’t have full information. This is something that should be confirmed by health professionals.

**P10:** What determines pregnant women’s diet during pregnancy is illness. When pregnant women go to health facility, they will be given tables that improve their food intake capabilities. Therefore, when pregnant women become pregnant, it is the additional disease the faced that protects them from eating a food. If you ask us what types of diseases are they, we don’t know. The only thing is that it is diseases which make them to have low appetite.

**Section 4: Other Nutrition related interventions that improve maternal health**

**I:** What other interventions do you know? For example like health service and diagnosis

**P4:** Pregnant women and lactating mothers have the habit of undergoing examination at health post, health center and woreda level. They go every three months and use every service they need. If the health workers at health post level have got an acute illness among mothers, they advise them to go to other health facility for further health services.

**P3:** Previously, mothers had the chance to get Fafa from our health facility. It was good service. But, provision of this energy giving food is now terminated and it is up to you find the solution. We don’t have factory to produce Fafa, oil and sugar. Therefore, our administration has nothing to improve the nutritional status of mothers.

**I:** What about community health days? Do mothers have the opportunity to be screened by health professionals at home at a sort of gathering?

**P8:** Yes, they have such services. Healthcare workers make pairs and go door-to-door to screen household members. After conducting the screening procedure, if a problem exists, they tell you refer you to other health facilities. They give health education on the measurements made and advise to come to health post health center for further action. But, there is lack of awareness of the way we use the health educations given by the health professionals who performed the screening process. However, there are some difficulties of using the health services given in this health post. This health post doesn’t have full material. Sometimes, as an administrative body of this kebelle, we tell them to bring te required materials from TRHB. But, they cannot and therefore, mothers will suffer at the health post or at home.

**I:** What do you think is the benefit that mothers are getting?

**P8:** Well. Since we are farmers with no education, we might not understand this issue well. But, when health professionals go home to home to screen and give advice accordingly, then mothers do have the benefit to get the health service quickly. This has the benefit to easily understand your health, extend your life and take adequate and selected diet properly. Those mothers who don’t know well will have the chance to be harmed severely.

**I:** Your ideas in this regard?

**P7:** The purpose of screening by going home to home is to identify children and mothers with nutritional problems. That is it. It does not have any other benefit. This is good. If you have a problem, they will advise you to come to health post for further service. But, when you go, there will not be other unique service. For example, my wife was screened and she had the problem of not eating food. They told her to come to health post but there was no a different service. The healthcare workers didn’t bring any change for the problem my wife had faced. Anyway, we understand that home to home visit of health professionals does have a huge benefit for mothers. It is clear. In addition, pregnant women have a meeting every month at the 7 day which helps them to get more information and clarity about their pregnancy in addition to the community health days. But, the issue of shortage of material is not good as we started to think of other options. We started to think of the ability of health workers in the health post though it is not the one to be blamed.

**P5:** Community health days are rarely seen. We know it is important to have such occasions but the fact is that such community days are disappearing. Otherwise, women are highly benefiting from such things**.**

**I:** What are the challenges of community health days? For community health days not to be effective, what challenges are there in place?

**P7:** One of the problems is distance. We have gained this health post after a tight argument and discussion among us. It is good to have this health post. However, those mothers and children who undergo screening in this health post will be sent to Fina Ruba to take things like Fafa and other energy giving foods. Even sometimes, the message that contains all pregnant and lactating women will be lost. For some of the mothers, going to Fina Ruba is too long and therefore they don’t get the energy giving foods. The issue of transport is also another barrier where there is not easy access.

**P4:** Mothers when referred to Samre health center, they will be forced to pay sometimes. This is also an obstacle. They don’t pay here in this health post. But, they are requested to pay in other health facilities. The other challenge is the shortage of medicine and vaccine in our health post. It is only medicines that will be consumed during pregnant women’s health day that will appear in the health post. Otherwise, there are no drugs. May be, the drug for malaria appears once every three months. The other challenge is the issue of insurance. 140 birr is being paid by insurance holders. When mothers go to other health facility, they are being requested to pay or bring insurance certificate.

**I:** What about safety net? Its benefits to mothers

**P9:** Mothers are getting aids in the form of birr and food items that will help them to lead the life of their children. Safety net is a good solution for the problems mothers have.

**P2:** Mothers don’t plough and do business in agricultural areas. For those who don’t have farm area business, safety net is a good option. Its importance is not for pregnant and lactating mothers only. It is also for elder mothers and even for males. So, in such a way, mothers are beneficiaries. I think so.

**P4:** Safety net has improved the life of males as well, not females only because it helps them 6 months a year. If the season is fine, then it will help them more. This is true when they are assessed and is confirmed that they don’t have adequate amount of resources. May be, there is a sort of problem in females rarely. If there is a little work to be done, they are made to work and even the fact that mothers participate in safety net programs when their baby is 10 months and above old. At this time, the mother will be forced to breast milk her baby with hot milk as the area is very hot. This is un solved problem of mothers in this community. Instead of helping mothers, we are making them busy and eligible to participate in soil and water conservation activity when their baby is 10 months and above old. This is not fair. May be, the aid in monetary amount for mothers is good. Otherwise, they are being forced to participate in soil and water conservation activities. That is not good.

**P3:** What I want to add is that though safety net program is a supportive program for mothers, it has also unacceptable system of involving mothers. The safety net program orders mothers to continue participating until their pregnancy reaches 6 months. Because we said is before that when pregnant mothers work, they will be weakened in addition to their weak appetite. Making pregnant mothers participate in water and soil conservation activities in the hot areas is really harmful. If don’t participate, they will get adequate assistance. Therefore, they are not being benefiting from the program. So, they need rest. Our government should improve this problem. We have many young males. If we want the next generation to be healthy, we need to help mothers, especially from such labor works. It is un fair to force them work under the circumstances I mentioned before.

**I:** Any other? Do mothers know that safety net program is in place for their benefit and other targets?

**P8:** The main of safety net program is to support those who are unable to lead their life. It is to create job opportunities for those who have a feeling to migrate. Safety net program prevents people from going out from their residency. That is it. It is to support individuals who don’t have job and therefore to contribute to the development of the nations, to prevent individuals from getting troubles in their life. This is the aim of safety net programs. That is all about my idea. Anyways, it is intended for those who are unable to support their life. It could be male or female. It depends. If there are males who need support, it will be practical and the same is true for females. It is the state of life they have that determines. Both have the chance to work. However, since females not males have difficulty of going out to site of safety net program, the things mentioned before to help mothers should be improved by the government. Anyway, the basic principle is that safety net focus on the fact that community members should work and get paid. It doesn’t focus on females. It is for all individuals.

**P7:** Generally, women don’t have the understanding that they should get benefit from safety net programs better than males. I have been working as Foreman in safety net programs for many years. In principle, it is indicated that safety net program is established for mothers and those individuals unable to support their life. But, the administrative bodies have not done an awareness creating line for females to get benefits from the program. The same is true for females. They don’t argue that safety net program is primarily for them. They are of course they are getting benefits though they don’t have the awareness about safety net program.

**P1:** For example, I could have 7 children along with my wife. But, safety net program only supports 5 members of household. This is not meaningful.

P9: Partly mothers are getting benefits and partly I doubt that they are benefiting. If a given mother has 10 children, she deserves to get the aid. However, only five they will get the benefit and this is 75 kilos. In such conditions, females are not benefiting well. This year, loans under the safety net program have been given to mothers. 75% of the loans were given to mothers. They are becoming aware of the benefits of safety net. But, the issue of five members of household to be included in the program should be solved.

**Section 5: Underage marriage and birth spacing**

**I:** If there is any new idea, let me proceed to the next question. The next question is about underage marriage and birth spacing. What do you think is the benefit of marriage later that 18 years?

**P5:** Under age marriage harms girls. In addition to this, the issue of economy is another problem. It will harm her and the family as well. If she is harmed, it will problematic.

**P10:** The question is the benefit if girls get married after 18 years. This result in girls’ maturity and even males will also be matured. When a women or a girl get married when 18 years and above, the will have the capability to manage her pregnancy. If she is underage, she may terminate her marriage. If not under marriage, she will stay healthy, the marriage will be fine and the baby will come of the womb with no. Moreover, the girl will be free of diseases. There are different diseases like HIV and even she will be safe of giving birth under operation.

**P7:** We are saying ‘let us create educated community.’ If there is no under-marriage, girls will not terminate their school life. The other benefit is that if girls continue learning as they are out of marriage, they will be happy. The economy of the country will be good. The economy of the girls itself will be fine. The health of the girl will be good. Under-age marriage, however, poses death threat to the mother as she is not matured and cannot tolerate things.

**I:** What about the promotion activities? How is marriage above 18 years and birth spacing promoted here in your community?

**P3:** This is a well promoted activity in this community. It is really being disseminated to the community. I cannot get community members with no idea of birth spacing and marriage above 18. It is not on yearly basis that this information is conveyed to the community. It is on monthly basis that this information is disseminated to the community.

**I:** Who disseminates this information?

**P3:** Health professionals and even community leaders are involved. It is promoted at village level and kebelle level and there are experts who follow it. Moreover, there is age examination. For example, my daughter has been here before a couple of minutes. I could have shown you. She get married after her age was assessed. It is in such a way that marriage is taking place. No girl gets married without going through the steps I mentioned. Anyway, it is positive.

**P9:** For the issue of girls’ marriage, it will take place when women development association, women affairs and leader of the kebelle, health and education office have all approved the issue. Otherwise, marriage will not take place. Regarding birth spacing, if a family has five children, it has to stop. For example, I have five children and I have to stop. I have decided to stop along with my wife. That is it.

**I:** What happens to the feeling of the community members as a result of promotion of birth spacing and above 18 years marriage?

**P2:** Previously, the feeling of the community was not good. But, the community is happy at this time provided marriage below 18 years doesn’t take place. If a given family is told that one of the members will not be getting married as her age is well below 18 years, the family will not complain. This is a fact in this community.

**P10:** Well. In some cases, there is still a gap that should be solved. However, most community members do not resist marriage related issues.

**P7:** Well. Let us ignore the good things as mentioned so far. There are community members who feel that protecting girls from underage marriage is a dangerous act. That is the feeling of some community members. Just stay until 18 years means meaningless. Why should she stay until 18? Our parents have come through such paths and why would we allow her until she spoils her personality? Why would we permit her until she spoils her maternity while she is at school? There are such community members that raise such questions. Anyway, the directions are good.

**I:** You told us that both marriage above 18 and birth spacing are being promoted in the community through different styles. Are there any other better ways of promoting marriage above 18 and birth spacing?

**P4:** My suggestion is the direction our government has brought is good and therefore it should continue as it is. Marriage above 18 years and birth spacing are very important for us.

**I:** You are right. But, the means of promotion is the questions whether it should it be improved or not?

**P9:** Yes, it should be well promoted using adequate human resource as my brother has described it.

**P7:** The community members are many. Of course, the decision should be passed by the members. But, in my opinion, the members should be 3 and at most 4. The community sometimes complains as there are committee members who you cannot get them easily. The community has known the essence of having this committee.

**P8:** Generally, it is described so far. The members are farmers and farmers focus on their life, their family. Experts should join them so that the committee will work in a nice manner. There should be one who coordinates the group and this coordinator should be a professional one. Otherwise, our farmers will go to farming activities though there is an issue of marriage that needs decision. Such disturbances will take place. So, there should be one who oversees the agenda.

**I:** What should be the birth interval?

**P1:** I have an experience for this. I have two children and the last one had come of the womb after 5 years. We understand the benefit. So, I have agreed with my wife that we have the third child after 8 years.

**P10:** Everyone has his own need. My opinion is 5 years. If it is 5 years, the child will go independently, will eat and drink by him/herself. Anyway, he/she will not be in jeopardy. But, in some families, they could have the feeling that they need more children as they are their helpers. And you have fear that you may not have more children if 8 years is the recommended time.

**P9:** It is better to have 4 years gap.

**P3:** 5 years is better.

**I:** Where did you get the information, 5 years?

**P3:** It is my opinion. We don’t have any health education related knowledge. They simply tell us that births should be spaced in order to bring about happy family.

**P4:** It is from our opinion. No other source.

**I:** Is there any community segment where birth spacing intervals are not promoted at?

**P5:** May be those who are unable to hear. Otherwise, I would say that there is no anyone who didn’t hear about the promotion. The message is conveyed at meeting occasions, churches and markets. Moreover, those who heard about the message convey it to others who didn’t have the information. There could be other better ways as everything is not absolute. Otherwise, it is good and confortable for me.

**P8:** On the issue of promotion of birth spacing, I would say it is being promoted in a good manner. There are established networks. The information flows from health professionals to kebelle leaders and then to women development army. So, I don’t think there is problem in dissemination of information.

**I:** I have from heard from you that birth intervals should be 4, 5 and 8 years. But, is it promoted in such a way?

**P4:** There is no such promotion. What is being promoted is that ‘have a well-spaced births.’ It doesn’t tell you how many years to have a space in between two children.

**P2:** We have never heard of the space that should exist between two children. The message is general. Birth should space only is the message conveyed to households.

**I:** What opportunities are there to manage marriage after 18 years?

**P8:** In our kebelle, there are 7 villages and each village has committees that follow the age before marriage take place. Though there are parents who, without the knowledge of the committee, try to conduct marriage of their under aged girl, the committee is effective and have the power. They can block the marriage and if this is above their scope, they will report to kebelle leader to have a sort of solution.

**P7:** Even the community has a good understanding on under age marriage. If under marriage takes place, the community knows that it could bring about a problem. Moreover, girls will terminate school life and even could encounter economic burden on the girl and its family as well. From experiences, the community has got a lot. Those girls who experienced under age marriage are considered as a good example. Those who faced such problem are suffering from different problems and those who didn’t face under age marriage and who continued their education have finally reached their destination. This is a good model path for the community. This is a good experience for us.

**Part 6: Communication and information dissemination**

**I:** Are there occasions when community members communicate about the nutrition related issues of mothers and pregnant women?

**P3:** Well. There is no such meeting, conversations for such issues only. May be, a comprehensive forum that includes many ideas for communication could be organized at kebelle level. Otherwise, there is no specific community conversation about maternal and adolescent girls’ nutrition related discussions to be frank.

**P7:** We cannot say that there is no conversation at community level. Pregnant women have a meeting with healthcare workers at the 7^th^ day of every month. The chance for health education is open then. Advices will be given. In conferences, the issue mothers most of the time is raised. Such as about maternal diet, delivery, clean water provision and other issue are discussed. However, meeting which really focus on mothers only, like agricultural activities, is not common. Even I can say such things are missing.

**I:** Where do pregnant women get information about nutrition related issues?

**P9:** The information is obtained from health centers. You have anemia, you have to eat such kind of food, you have vaginal problems… such information are usually commonly obtained from healthcare workers in our kebelle’s health post.

**I:** Barriers for information?

**P7:** The distance is the main barrier for information. As I said before, some of the pregnant women will go to health centers ignoring its opportunity cost and others will choose to remain at home as there is family issue that might not be ignored. They trust on GOD and remain home. They spend the whole pregnancy period without any information about nutrition. May be, they might get the information from a fortune visit by health professionals. So, the main barrier is having trust in GOD only and the other is distance.

**I:** What information do pregnant women and lactating women need to know?

**P5:** Overall, they need to know more information about the better preparation of porridge. The other is about the usage of honey especially when mother are at post delivery period.

**P9:** Mothers have the desire to have information about weight, blood pressure and may be about the different types of food necessary for her.

**I:** At the end, if there are concerns that we didn’t discuss them so far?

**P9:** Okay. The issue that should not be skipped and that we need to be conveyed is that the screening that had been done and the complementary foods given should be repaired. The other is the supply of sugar and oil should be restored. The third one is the government should consider the drought we have faced and should intervene as we don’t have something to eat and of course our cattle as well. The issue of safety net should also be considered as only 5 members of a given family get the aid and finally the issue of birth interval spacing should be better introduced in to the community.

**I:** Oh…[Oh is to indicate happiness as a result of completion of the long discussion]. Thank you very much. I have finished my questions. Of course, it is not me who finalized the long issue we had. It is you. Therefore, I would like to say thank you for the fine discussion we had. Thank you very much.

Summary

- Mothers in keyh emba village are focusing on health facility visit to make themselves healthy and therefore to make the health of their babies good.
- As there is drought, nutrition related problem like watery diarrhea and malnutrition are common in keyh emba kebelle.
- Healthcare workers advice is the main intervention mentioned by focus group discussants to improve maternal health and the fact that administrative bodies of the kebelle are arrogant and the overall illiteracy state in the community are barriers to access and utilization for nutritional services.
- Getting rest what most discussant mentioned as the main perceived need of mothers.
- Most discussants have understood that birth spacing is very important and the interval mentioned is 4-8 years.
- The dissemination of information about mothers’ related nutrition is obtained from health workers in most cases as mentioned by focus group discussants.

The end
