# Supplementary material for: Barriers to utilize nutrition interventions among lactating women in rural communities of Tigray, northern Ethiopia: An exploratory study
Source: PLoS One. 2021 Apr 30;16(4):e0250696. doi: 10.1371/journal.pone.0250696 (PMC8087028; doi:10.1371/journal.pone.0250696)
Supplement: S2 File — (ZIP) [file pone.0250696.s002.zip › S2_File.Doc/Community level Key informants/052_IDI_Health worker_Fina Ruwa kebele_Samre woreda.docx]

**Operational Research on Adolescent and Maternal Nutrition in Northern Ethiopia**

## **Tool A**

## **IN-DEPTH INTERVIEW GUIDE,**

## **School teacher**

Hello, my name is Omer Seid. I am from Mekelle University. Thank you for taking the time to speak with me today. We are doing research on the factors that influence the nutrition of mothers and adolescents in collaboration with the Regional Health Bureau and UNICEF. Your participation is very valuable. The things that you tell us will be used to improve nutrition programs and services for women in the region and the country. We will not share your names when we report our results.

| **Section A: Interview details** | |
| --- | --- |
| **Questions** | **Answer** |
| Zone | South east Tigray |
| Woreda | Sahrti Samre |
| Kebele | Member hadinet |
| Name of key informant | Tsegaye Birhanu |
| Institution of key informant | Fenarwa health center |
| Interviewer name | Omer seid |
| Date of interview | 09,November 2017 |
| Interview start time | 10:47 Am |
| Interview end time: | 12:33 Am |

| **Section B: Interviewee professional information** | |
| --- | --- |
| **Questions** | **Answer** |
| Gender | Male |
| Age in year | 25 years |
| Highest level of completed education. | Bachelor degree in midwifery |
| Current job/position | Health Center MCH |
| How long have you been in the current job/position: | 1 and 1/12 years |

**Section 1; Common maternal (pregnant women, lactating women and adolescent girls) nutrition problems in the community**

I: What do women do to stay healthy in this community/woreda?

P: Majority pregnant mother needs to exempt from works, and majority come to the health center for checkups for their pregnancy, then majority will do according our (the health center) advised. There is also cultural do they do to keep themselves healthy. Traditionally all pregnant mother applying externally or painting their abdominal area by leave juices to prevent “michi” or acute fever, which is a traditional treatment applied to prevent *michi.* Another thing the health center gives support letter for pregnant mother then they will exempted from productive safe net program developmental works for the seeks of giving time for rest.

I: What about feeding habit during pregnancy?

P: No change, the mothers will not change their food habit during pregnancy, they easts the accessible foods from animal sources like milk. She consumed the usual family food and has no change on her food consumption because of pregnancy. Mainly mother focus on taking rests during pregnancy but no modification on their food intake.

I: what about lactating mother?

P: Similar to the pregnant mother, traditionally it is recommended, lactating mother will set home and it is not allowed for her to go out of home before 12 days after delivery, and it true for her new born also because it bad for both child and mothers health. They come to the health center for immunization of her child. In general, Majority’s has responsible to do advises given by the health professionals. After birth traditionally the community come and visit the mother and have also bring different type of for the new born mothers

**I:** What do adolescents do to stay healthy in this community?

**P:** It is similar with pregnant mothers, no food change during adolescent, they usually eats the accessible family foods, like ground nut. The area is ground nut productive area and it is staple food of the community in the form of roasting.

**I:** What are the common nutrition problems in the community for women and adolescent girls?

P: For me the nutrition problem of this area is absence of food. During pregnancy commonly there may be loss of appetite but no action will be done by mother to manage it and majority of these mother prefer to tolerate it and take coffee for survival and these women’s comes to us and complain about loss of appetite, that why they are malnourished. There was FAFA supplementation for pregnant mother in our area but it is terminated six or seven months ago. We have also plump nut supplementation program for lactating mother child. For adolescent girls, there was FAFA supplementation before six or seven mothers but not now.

I: What about micronutrient deficiencies (such as anemia, night blindness, goiter)?

P:These are common problem add usually mother come to our health center by due to sign and symptoms of anemia, all pregnant mother have sign and symptom of anemia. For these mothers what we did is counseling and giving IFA. Goiter is not common and a bit minimal problem in our area. The major problem is anemia for pregnant mothers, rarely there is also varicose vain related lower leg edema I observed.

I: What about micronutrient deficiencies (such as anemia, night blindness, goiter) among lactating mother and adolescent girls?

P: It is not common problem we give plump nut for their child. For adolescent girls there was FAFA supplementation before six or seven mothers but not now. Most of the time the salt they used is not iodized and which is from Afar region.

I: What about stunting, underweight among adolescent girl and women?

P: They are fine and but there are short and underweight women’s and girls and I think it is mild problem in this community.

I: what about overweight?

P: laughing…. No such kind women and girls in this area.

I: what about Diet related non communicable diseases like, DM and hypertension?

P: It is not problem in this area

I: What is that, I think you told me that in some pregnant mother you observed varicose vain related lower leg edema

P: it is simple edema which is occurred due to standing for prolonged time of pregnant mother for working or due to prolonged waking for social gatherings like burial gatherings and it is miner complication of pregnancy.

I: What about food insecurity level of these women and adolescent girls?

P: No food shortage for take the problem is on its quality.

I: Do mean they cover all their food needs or do the government support?

P: With laughing then, the food they eat is from government support.

I: Which women groups are most affected by these nutrition problems?

P: Most of the time pregnant women’s are affected by this malnutrition. They come to our health centre and complaining about their appetites and has said eating only one meal per day and has taken only coffee, and even sometimes they reported that no appetite for coffee has eat nothing in the entire day. So because of this they are malnourished if they eat different type of food they will not be malnourished. Lactating mother are better in nutrition than that of pregnant mother because these women has too many food supporters form the family.

I: Do mean there is a food apriority for lactating’s mothers than pregnant in this community?

P: Yes, for lactating mother sheep or goat will be sloughed to feed her but no such thing for the pregnant mothers.

I: What about adolescent girl’s exposure for the above malnutrition problem?

P: It is fine, they are not exposed groups.

I: Why pregnant and lactating mothers are more risks for the above nutritional problems?

P: If the woman is pregnant, there will be low food intake which culturally accepted phenomena by the community. The community said low appetite is common problem across pregnant mother in the first two/three/four months, and then around seven/ eight months of pregnancy there is also pushing syndromes. According to the community, in the first four / five months of there will be low appetites for pregnant mother and the community has perceived it normal case, no nutrition support her. After seven/ eight months of pregnancy there will be pushing syndromes, here the community perceived it is normal problem being having not eat too much because of the syndrome and they said which is indicator that the mother has approaching to give birth because of this no nutritional support for pregnant mothers that way they become malnourished.

**Section 2; Nutrition priorities in the woreda**

I: Do you think it is necessary for your institution to get involved in work aimed at improving maternal nutrition? Explore for pregnant women, lactating women and adolescent girls.

P: It is possible to address malnutrition of pregnant and lactating women and adolescent girls. But it depends on the place. Late alone if you teach the community about diversified consumption, the community will not do it because do not have access for diversified food. No change or very minimal change in the community we achieved so far, that we know that all health works has going in to the community and has been doing teachings the community about nutrition and health but no change. It is difficult to bring big change on improving maternal nutrition.

I: Why?

P: you can imagine, when you teach them (mothers) because where could they get the diversified food, is it from ketema samre or Fenarwa, where could they get? It is difficult how they get diversified food, even there is no enough rain and it is dray area, and the area is not suitable for diversified croup production in this case it is not supportive for nutrition.

I: I think you are saying you are doing intervention for improving maternal nutrition currently, but there is minimal change, so you are already involving and in this regard why it is necessary to involving your institutions?

P: To reduce malnutrition

I: How or, what kind of intervention you are doing currently?

I: Health education, HEW and the health centres has demonstrates child food preparation for mothers, and health experts also going in to the community and teaches the community about feedings like iodized salt consumption. The community has using none iodised salts rock salts which is from Afar region and health workers have starving to change this by education. If all health works teaches the community like this malnutrition will be address.

I: what about other nutrition intervention like MUAC screenings?

P: It is done in the health centre, here we have CHD and in this program there is monthly screenings for all under two children, pregnant and lactating mother in the health centre. Now the problem is no FAFA here, we did only screenings, we do not have FAFA to give for the malnourished one. We have only plump nuts for children’s only but not for lactating and pregnant mothers.

I: What maternal nutrition (pregnant, lactating and adolescent girls) interventions are the priorities in this woreda?

P: In each monthly screening program if we get malnourished child we give plump nuts for its treatment. This assessment is done for all pregnant and lactating mother with their under two children, then priority will be given for the malnourished one based on the criteria for supplementations. Being having pregnancy is not criteria or not enough for supplementary foods, eg: If the criterion for supplementation is MUA =<18 or 19 CM which means the pregnant mother will not have supplementary food if her MUAC is 20 CM. so supplementary food priories will be given for the malnourished women’s.

I: What kind of nutrition services do you spend most of your time on?

P: On health education/nutrition education

I: what kind of education you give for them?

P: we counsel women’s about diversified consumption, appropriate consumption of the FAFA that we give, and then sharing it to other family’s member is not allowed.

I: as you told me you are doing CHD screenings and supplementation of foods so what nutrition services do you spend most of your time on in this case?

P: All activities are equal for us and we are doing equality, we don’t have special time for some specific activities.

I: Do you mean no other intervention except screenings and the supplementation?

P: There is prepared formula flour namely HAVENEY which is from the woreda, then we did is bartering, we give 1 kg the prepared HAVENEY for mothers and then they return 2 Kg unprocessed grains. Around sixty kg we received from the woreda, but have no that much demand from the community.

I: Now, where you spend most of your time is it on screening, distribution of supplementation….?

P: With laughing, distribution of the supplementation doesn’t take time, but doing screenings need too much time.

I: What do you do in this screenings?

P: Using MUAC we did screening for all pregnant, lactating and under two years children’s, then based on their MUAC order they will get FAFA for women’s and plump nut for children’s. eg: for pregnant mother FAFA will be given for both moderate and severely malnourished mother if we have enough FAFA, but it will be prioritized and given for only severely malnourished mother if we don’t have enough FAFA. If the mother has MUAC of 18 CM priority will be given for her than that of mother having MUAC of 19 CM, and If the mother has MUAC of 20 CM priority will be given for her than that of mother having MUAC of 21 CM. Up to MUAC of 23 cm it is allowed to give FAFA but not allowed to give mothers having MUAC of greater than MUAC 23 cm. This means mothers having MUACs of 23, 22 cm will not get the FAFA if we have too many mother in MUAC of 18 or 19 cm.

I: Can you tell me some of the successful maternal nutrition interventions that you have implemented in this woreda?

P: Mainly intervention that has done on lactating mothers is successful. Eg: pregnant mother are not using properly that the FAFA we give but lactating mother are using properly and successful.

I: Why you say lactating or how do you measure the successful?

P: Measuring their MUAC, then if they are well we will not give the FAFA

I: What about adolescent girls? Is there any intervention you are doing currently?

P: For adolescents it is the same, if they are malnourished FAFA will be given and we educate also nut no other service for them.

**Section 3: Nutrition interventions that improve adolescent and maternal health**

I: What kinds of nutrition interventions are in place to improve adolescent and maternal health in this woreda?

P: What we did is giving health education, doing nutrition screenings using MUAC, measuring weight of lactating mothers, giving IFA for pregnant mother.

I: so I need to know all activities you are doing for lactating and pregnant women’s and adolescent girls?

P: For pregnant mothers; we give education about daily feeding practice like, we advised mother:

- Talking at least five meals per day, ( taking to additional meals for the usual three)
- To prevent anemia we advised mother to consume foods cooked from red Teff, leafy vegetables like chard, lettuce and other like egg. Anyways, what we did is advising mother to eat homemade foods in the appropriate way.
- Taking rest, it is not mean totally set home in the while day, it means do not do long tedious walking, avoid heavy works and carrying heavy loads.
- We make the pregnant mother to bring her husband to us, and then we give him advice to supporting his wife in her pregnancy follow up.
- Giving IFA, and advising its importance.
- If the pregnant mother is malnourished, if we have we will give FAFA for her
- Advising about the danger signs and appointing the next follow up
- About child feeding in advance
- Testing HIV for her and for her husband
- Deworming at seven month of pregnancy
- Consumption of iodized salt
- Sleep in recumbent position and Advice on Insecticide treated bed nets (ITN) to prevent malaria. Pregnant mother become more anemic if they suffered from malaria and for pregnant mother it is not recommended to give the drug for the treatment malaria.
- About consumption of hygienic (clean) food and drinking clean water. We advised about toilet utilization and, water treatment methods because the community using river water so we teach use chemical or boiling for treating the water. This is to help them for preventing drinking of unsafe water.
- We give support letter for exemption of productive safety net program (PSNP) developmental works but, in behalf of her child or husband will do developmental.

**I: What about intervention for lactating**

P: For Lactating women what we did is advising about;

- Advising about family planning and if she need we give also
- About Immunization of her child and when she will come for immunization
- About hygiene practices, about child feeding like EBF in the first six months
- About her dietary practices, at least five meals per day. Most of the time accessing is the problem so we advised mother to buy from markets at Sahrity Samre city. For lactating mother this additional food is important for producing enough milk for her child.
- Using Insecticide treated bed nets (ITN) to prevent malaria and if they are infected by malaria mother will be anemic.

**I: what about intervention for Intervention for adolescent girls are?**

P: is it for pregnant adolescents?

I: For none pregnant adolescents?

P: About them, they come for **YCF** service and then they gets some advises

I: what are those advises

P: they gets advises like,

- About reproductive health related advises
- About family planning’s utilization and the prevention of unwanted pregnancy
- About food intake which is preparing them matured for future, then they will not be affected.
- HIV testing
- Teaching about the prevention of underage marriage

I: Is there school feeding program

P: No, it may not, have no Idea

I: do you have IFA for them?

P: for whom?

I: For adolescent girls?

P: No, we don’t have for them

I: Deworming services?

P: For Whom?

I: For adolescent girls

P: we don’t have this program

I: what about sanitation and hygiene’s?

P: yes we have this intervention programs

I: **In your opinion, which of the above programs are being implemented successfully (i.e. in the most effective way?) Why**? **Explore for pregnant women, lactating women and adolescent girls.**In your opinion, which of the programs mentioned above are less effective? Why? **Explore for pregnant women, lactating women and adolescent girls.**

**P:** I need to say successful program, services we give both here and also given in the community’s by going there like water consumption, sanitation and hygiene and this program is good to compare with other programs. In addition on child feeding, there is good improvement on EBF.

I: Do you have good successful program on adolescents?

P: About adolescent girls we did good job on family planning utilizations but no that much change on other nutrition related intervention.

I: Why you said these programs are successful?

P: The first reason is these programs are easily accessible by women’s so it is simple to intervention on these programs, but if we see other intervention it is difficult to make accessible for women.

I: Do you have any other successful programs? Why?

P: that is what I have

P: In your opinion, which of the programs mentioned above are less effective? Why?

P: still we are not changed the feeding practice of pregnant mothers. It is poor and less effective.

I: What about lactating and adolescent girls?

P: Feeding practice of lactating mother is good and about adolescent is medium.

I: why still the pregnant mother feeding is not improved?

P: Because no food access. We are continuously teaching them but no change on their food habit.

I: What about IFA intake of pregnant mothers?

P: It is good in utilization, but some pregnant mother refused to take it because of fearing that the child will be big in the womb, and then it will be difficult to born.

I: Does we take IFA supplementation as ineffective intervention in you ?

P: It is good program; because all pregnant mothers have come with anemia in our health service then we supplement them and have good improvements, so it is good intervention.

I: Do you have any other reason why dietary interventions on pregnant mothers are ineffective?

P: Another reason is related to its agro ecology that is the area is temperate, have no enough rain, so it is not supportive for nutrition.

I: **What are the implementation challenges that are specific to delivering the maternal nutrition interventions in the programs that we have been discussing? Explore for pregnant women, lactating women and adolescent girls.**

**P:** because of culture related barrier women will not accept some health interventions and do the intervention.

I: what is that? Could you tell me some examples?

P: Eg; to prevent acute fever pregnant mother uses to applied leaf juices (kitel mekebat) externally on their abdomen it is cultural related health problem

I: Do have any other cultural barriers for maternal nutrition? For example for whom food priority will be given; among females and male adolescent Vs female adolescents

P: Priority is given for females than male in our community, priority will be given for lactating mother than pregnant mothers in the community, from adolescent girls and adolescent male’s priority will be given for adolescent females.

I: Do you have Lack of resources/resource constraints to do on maternal nutrition’s?

P: We don’t have that much problem on resource.

I: I think you told me above there is shortage of FAFA, am I right?

P: Yes we don’t have FAFA for mother’s distribution. no problem with IFA we have enough for pregnant mother distribution and we don’t have also shortage on insecticide bed net and we distribute this insecticide nets in every in June.

I: What bout shortage of motorcycle, or budget?

P: Yes there is shortage on motorcycle, I do have very old motorbike which is very tough to use it while I need to go in to the community for education.

I: What about competing priorities?

P: Yes there is, if the issue is urgent, Eg: if there is AWD in the community, priority will be given for it than that of CHD.

I: It is during urgent situation what about in normal condition?

P: Not computing priorities in normal circumstance.

I: is there priority for nutrition?

P: No priority for nutrition most of the time, in our outreach activities in the community our priority are home delivery, ANC utilization and hygiene and sanitations.

I: Is that a problem, Lack of awareness on nutrition related problems?

P: It is good in all staffs; we don’t have that much gap and at least we have enough for pregnant and lactating mothers.

I: What about adolescent’s girls?

P: We don’t have separate intervention for adolescent girls but we have for lactating and pregnant mothers. For adolescents we have YCF program, or in the community we do give intervention with other community groups. We don’t have enough focus for adolescent’s intervention like that of pregnant and lactating mothers

I: Training needs/staffing/staff turn over

P: We don’t need that much training

I: What about the collaboration between the nutrition sensitive and specific sectors to improve maternal nutrition?

P: he is slight…. Don’t know the nutrition sensitive and specific sectors to improve maternal nutrition

I: I explain about the nutrition sensitive and specific sectors and then I asked him again?

P: there is a poor collaboration b / n nutrition sensitive and specific sector

I: **Which of these challenges are the most important**?

P: shortage of FAFA, and we don’t have coordination with other sectors, we have also shortage of staffs.

I: Which of these challenges are the most important among adolescent?

P: About adolescent girls they are not our focus, we don’t have enough focus for them.

I: Which of these challenges are the most important among pregnant women?

P: we have no thing for them, we did the screening in the CHD program then we give advice only, we don’t have FAFA for malnourished mothers and even if her MUAC showed the she is severely malnourished we give advising about dietary practice only.

I: Which of these challenges are the most important among lactating women?

P: About lactating women we don’t have that much challenge, but like that of pregnant mother if we get severely malnourished lactating mother we don’t have FAFA for them we give only dietary counseling’s.

I: is there any support for women’s by productive safety net program (PSNP)?

P: No specific support for them, the support is given for the whole family

I: For these challenges that you mentioned, can you tell me of any successes or innovations that the Region/Woreda Offices have used to improve maternal nutrition service delivery?

P: yes, for example we did report to requested FAFA but the response is no FAFA for us. We did report only, because no thing we will do except. About lactating mother we make HEW to work in the community so they give detail health education for lactating mother, and this is what we do only and we don’t have any other intervention for them.

I: As you said adolescents girls are not your focus, but have you ever tried to do adolescent girls?

P: Family planning or for pregnant mothers, but we do have separate adolescent girls focused programs.

**Section 4: Community factors affecting access to maternal nutrition interventions**

I: Can you think of barriers that prevent adolescents and women from using the programs and interventions that we have discussed? **Explore for adolescent girls, pregnant women and lactating women.**

**P:** One of the barriers is distance of the area. Mother is not getting the service in the nearby, eg: because of the fare distance may not come for four ANC. Even what we did is giving IFA for three months, because the mother may not come for the second the ANC because of the distance. It is also hard for us for doing home visiting to check wither she is taking or not taking the IFA because of the long distance.

I: What about other barriers?

P: low awareness is barrier

I: low awareness for what?

P: They will not do practically according to my advises. We give advises here but mother will not applied it in their home because culture related factors.

I: I am happy to hear specific examples, can do that Tsegaye?

P: It is not that much problem

I: so, what is that cultural barrier you mentioned? Am asking this for future good

P: It is not that much problem,

P: Mothers are not education and this is the barrier.

I: How could educational level of mother is a barrier?

P: Mothers are not applying the advisees’ they get for health worker, which is related to their low educational states. So their illiteracy is the barrier for not applying properly.

I: what about work load?

P: yes, mothers have too loaded works, which is evidenced by they come for ANC or PNC in market dies with doing their market shopping and during none working days. Because they are busy in working days.

I: what about the community beliefs?

P: yes, most of the time it is not allowed to go out of home for pregnant mother by fearing adverse health consequences locally known as *“Aynetila”*. It is not also allowed go out of home for lactating mother before 12 days of after delivery, because the community perceived sun exposure of mothers during this time is bad for her and for her new born health locally known as “michi”.

I: What is the problem relating to awareness?

P: The community perception towards our awareness creation interventions is not good. The community haven’t ear and has no interested to hear our awareness creation, rather they perceived we (health worker) do the awareness creation is for the sick of salary or benefit, because we (health workers) are recreated for that. According to them, if we do not do awareness creation we will not get salary or per diem. I think it is due to poor awareness of the community. late alone, it is not recommended to go out of home for new bone mother before 12 day of after delivery but if we teach the community to rectify this issue, i.e if we teach the community, this is a tradition and it is not correct, no problem or nothing will happen on mother and the child health if the mother go out of home before 12 days after delivery. The community may respond or say, it is oky, we know it, but you are doing what you are recruited for. So their perception is not good.

I: what could be the major barrier for not changing their behavior here?

P: This due to the community effect. It is impossible to teach separately each individual of the community. If we give advises for one pregnant mother, she is okay here, but she will not do according to the advises when she back to home which is due to community pressures. The mother knows the truth but because of the pressure that comes from her family or neighbor she will not do it practically, because her family or neighbor doesn’t have knowledge about the intervention or the issue.

I: Do you have any other barriers?

P: If you go to the community to teach about nutrition, the community will say our trip is for benefit ourselves only, because according to the community we will get per dime for the trip. The community perceived, we go there because it is for our per diem, not for community benefit. Even the community expected to have some composition/ incentives payment from us while they come and participated in our health education session.

I: what about quality of care as barrier?

P: It is difficult to give health education service in the community because of long distance and the area is very hot in climate so it is hard for health workers to go in the community. The health worker will not give good services, if he/she backs from such kind of tedious filed works. You will go long distance by foot to educate the community but If the community perception is not good and if you are back from such kind of fields, you will not be happy to serve the community, and will not give quality services in case. For example: we have Keyeh emabh kebell and the keblle has around 500 homes, if you need to go there to educate them you will paid 20 birr for a single trip transportation, then if the community is not interested and having the above like perception, you will not be happy, so this will significantly affects the service quality.

I: maybe can you mention some barriers for adolescent girls for not utilizing the service?

P: Relating adolescent so far we don’t have service for them

**I:** How can these barriers be addressed to improve maternal nutrition in the community/woreda?

P: The area is not suitable for pregnant and lactating mothers, which is it mountainous, so we should design home based case. The health care giver should go to mothers home and give home based care for pregnant and lactating mother because it is difficult for pregnant and lactating mother to come the health facility because of the distance and the topography of the area. Awareness creation is essential, through using churches because the community has strong attachment with churches so if the religious leads are well aware about the issue, they will teach their children, and by using community social leaders (*shemagles*) we can teach the community to create awareness. For pregnant and lactating mother we do teaching here but no behavioral change because of community influence, so we should do community awareness. If you call the community for health education they will ask you payment first to come and join, this is because of poor awareness of the community.

**Section 5: Other interventions that influence adolescent and maternal nutrition and health outcomes**

I: In your opinion, why would increasing the space between each births and delayed marriage (after 18 years) improve maternal nutrition and hence both maternal and infant health?

P: increasing birth interval is very important

I: What is that importance?

P: For example if the women have short birth interval in one or two year intervals, and mothers are responsible to cook food, to do farming activities like removing or un-weeding the crops and harvesting crops, so children’s have effect on these activities of the mother. Even for nutrition, the food for large family and the food for small family is not the same.

I: is there any health effect on mother’s health?

P; Yes has much effect on her health, eg; as I told you her nutrition is less, which means the mother will be malnourished because of shortage of food because of large family size and the mother will be anemic because of repeated birth she gives so it is not good for mother.

I: your opinion, why would delayed marriage (after 18 years) improve maternal nutrition and hence both maternal and infant health

P: First they are not matured and if they eat enough their MUAC and BMI will be increased so they will not be malnourished during pregnancy?

I: is there any health and nutritional problem related to early marriage? Let’s say a girl has healthy BMI and MUAC, and her age is 15 years, is there any problem in health or nutrition if she married?

P: laughing…Yes there will be a problem, even if she is well nutritionally but still she will face other health problems like fistula and other and psychological disorders.

I: Why fistula and psychological problem?

P: Because she is not matured so she will be stressed, pregnancy by itself is the worst thing so she will faced pregnancy related hypertension and for fistula also.

I: What programs or activities promote increased birth intervals in this woreda?

P: There is strong education program in this community about increasing birth interval and the privation early marriage. About family planning’s and it importance, and to prevent early marriage education about it has given in school and during females meeting. YFS we educate female adolescent about early marriage.

I: Assume, who will be responsible to control if one adolescent girl has made early marriage?

P: There is a team which established by peoples from the health center, from women affairs, kebelle leader and there are also other members that are from the community. this team is responsible to control early marriage in the community.

I: Can you think of any more programs or policies? Think about political, religious and other influences.

P: I’m happy if it (program/ polices) is culturally related, what I mean here the community will not come to the healthy facility just they will say it is evil eye so will not come to us. Even for simple cases if they suffer from diarrhea they prefer to go to traditional healer than the health facility. The community believed or trust the traditional healers (tenquay) so they will not come to the health center, this traditional ancient believe should be changed. To change this situation we should work with the religious institutions.

I: In your opinion, are these programs or policies effective? Why or why not?

P: If you tell father about increasing birth interval, they will not agree and responded you our children are our degrees like the degree that you have. So still they prefer to have many children’s. In this community still there is early marriage and the culture supports early marriage.

I: Can you think of any other opportunities to prevent early marriage and increase birth spacing?

P: still awareness creation is essential for its improvements. We should teach the importance and the consequences.

I: building awareness at schools?

P: yes, using the new generation of schools is essential for its improving.

**Section 6; Multi-seectoral collaboration to improve maternal nutrition**

I: Do you feel it is necessary for your institution to work with other sectors/institutions to address maternal nutrition?

P: We had got FAFA from Maret(REST) , so to address maternal nutrition like this we can work with REST. We will work on education and REST can support as FAFA in this case.

I: what about other partners?

P: we can with world vision, l10K

I: what about government sectors?

P: From the government; we can do together with agriculture sector, this sector will do on gardens, and identifying crops that suitable to produces in this area then will promote its production. We can work with schools, regarding to nutrition with the school teachers and director we can teach the nutritional problem of the area. Then to address malnutrition we can work with schools. With religious leaders we can teach the community about good nutrition, and the prevention of malnutrition.

I: For multi-sectorial action that effectively works to improve maternal nutrition at all levels, what kind of change in terms of the way stakeholders work together is needed?

P: If all sectors are coordinated we can change malnutrition, we can prevent early marriage, we increase birth intervals and we can prevent other problems.

I: yes you are right coordinated work is eventual so? What change will be done at the federal level?

P: At federal level it should control malnourished and no malnourished cases. At federal level it should identify the causes for malnutrition, if it comes from higher body, at our level we have to have enough staffs, the necessary imputes and budgets for education the community. The woreda should support, the region should support this activity.

I: To what extent does your institution participate in the multi-sectoral nutrition coordinating body at the woreda level?

I: I don’t have idea about it

I: Do you have any other comments on anything that we have discussed?

P: the community prefers to use traditional healer than that of our health centers, so awareness creation is essential in this case.

**Summery**

**Section 1; Common maternal (pregnant women, lactating women and adolescent girls) nutrition problems in the community**

Women do the following things to stay healthy

- Taking rest
- Doing checkups in the healthy facility
- Traditionally all pregnant mother applying externally or painting their abdomen using leafs

Common nutrition problems

- Moderate and sever
- Severe and moderate malnutrition
- micronutrient deficiencies- anemia is the very severe problem
- food insecurity
- wasting

**Section 2; Nutrition priorities in the woreda**

It is necessary to involve our institution to reduce malnutrition. Currently we are doing CHD screenings for pregnant and lactating mothers and children bellow two years. Most of the time we spend our time on health education

**Section 3: Nutrition interventions that improve adolescent and maternal health**

Nutrition interventions that are in place to improve adolescent and maternal health in this are

- Health education,
- doing nutrition screenings using MUAC,
- measuring weight of lactating mothers,
- Giving IFA for pregnant mother.
- Other

But adolescents are not the focus of the health center

Of these all intervention we did the most successful job on sanitation and hygiene

The implementation challenges that are;

- **s**hortage of resources
- no enough awareness
- Poor collaboration b / n nutrition sensitive and specific sector

**Section 4: Community factors affecting access to maternal nutrition interventions**

Common barriers

- Long distance
- low awareness
- educational states of women
- maternal work load
- Community believes
- Poor quality of care
- Relating adolescent so far we don’t have service for them

**Section 5: Other interventions that influence adolescent and maternal nutrition and health outcomes**

Short birth interval is not good the mother, she will be loaded by works, and if there is large family there will be malnutrition. Early marriage is not good because the girl is not matured enough so she will faced fistula and psychological problems.

**Section 6; Multi-seectoral collaboration to improve maternal nutrition**

We can do jointly,

- REST
- world vision,
- l10K
- agriculture sector,
- schools,
