# Supplementary material for: Barriers to utilize nutrition interventions among lactating women in rural communities of Tigray, northern Ethiopia: An exploratory study
Source: PLoS One. 2021 Apr 30;16(4):e0250696. doi: 10.1371/journal.pone.0250696 (PMC8087028; doi:10.1371/journal.pone.0250696)
Supplement: S2 File — (ZIP) [file pone.0250696.s002.zip › S2_File.Doc/Community level Key informants/056_IDI_HEW_Lemelem Kebele_Samre woreda.docx]

**Operational research on Adolescent and maternal nutrition in Northern Ethiopia**

**In-Depth interview with HEW**

**Introduction**

Thank you for your willingness and for taking the time to speak with me today. I have several questions to ask you that I have prepared in advance. If you have any additional questions or comments as we do the interview, please feel free to share them with me.

| **Section A: Interview details**   1. Zone: **South eastern** 2. Woreda: **Semere** 3. Kebelle: **Lemelem** 4. Name of key informant: **Mrs. Dlay Hayelom** 5. Institution of key informant: **Lemelem**  **Health post** 6. Interviewer name: **Abate Bekele** 7. Date of interview: **11/11/2017** 8. Interview start time: **10:55AM** 9. Interview end time: **12:21:27AM** |
| --- |
| **Section B: Interviewee professional information**   1. Sex    1. **Female**    2. Male 2. Age: **26 years** 3. Highest level of completed education.    1. **College education**    2. Bachelor degree    3. Master’s degree    4. PhD 4. Current position: **Health Extension Worker** 5. How long have you been in current job/position:    1. ______ Months    2. **___3___** years |

**I:** Interviewer **P:** Participant

1. **Common maternal (Pregnant, lactating women and adolescent girls) nutrition problems in the community**

**I: What are the common nutrition problems in the community for women and adolescent girls?**

**P:** This community has been thought on many health problems, and they also need much more education than the current level. There is also backwardness in this community. In this community, women are at risk of various nutritional related diseases like stunting, and thinness.

**I: How do you evaluate the level of these problems?**

**P:** There are few mothers affected by these problems, due to maternal malnutrition, the children born from these mothers are also affected by under-nutrition. Therefore, children will become stunted and thin like their mothers.

**I: How much sever is the problem in mothers?**

**P:** It is not that much. There are no mothers receiving plumpy-net, but there are children.

**I: Are there mothers who are receiving Targeted Supplementary foods like blended flour?**

**P:** Yes there are. Last year, the World vision provided it for them monthly five packs per month. Therefore, it was given for mothers who have mid-upper arm circumference less than 21 centimeter.

**I: How many mothers were affected last year?**

**P:** More than sixty received TSF. The number of women affected varied across the months of the year sometimes it was more than 60 but sometimes it was 60.

**I: In which month do the number of mothers affected is higher?**

**P:** At this month the case is higher, i. e. from November to December.

**I: Why it happens at these months?**

**P:** Because the farmers are on work/farm, they stay at desert without eating lunch, there children again feed only once per day. Therefore, the children and their mothers are affected by under-nutrition.

**I: What are nutrition related problems for adolescent girls in this community?**

**P:** When we see them at school they are fine, unlike mothers and children.

**I: What benefits do the adolescents get while they are at school?**

**P:** Because the education is given by various experts like teacher, and Health Extension Worker (HEW) and they stay on reading and writing daily, they understand the problem and bring it to their home or family. Therefore, these groups are better than other groups.

I: Are there thin adolescent girls in your community?

P: No

**I: Why?**

**P:** Since they are educated, as they take trainings at school and they can practice what they got.

**I: As you have said there is poor feeding habit in the families in the work season, and these adolescents are also from these families so how these adolescents do are well-nourished?**

**P:** Yes the feeding habit is the same for all, but as they are kids they are not expected to work.

**I: Is there any school feeding program?**

**P:** No

**I: Is there any micronutrient deficiencies cases like anemia and goiter among women?**

**P:** There is no Goiter in this community because the area is low land, but it can occur in high land areas.

**I: What is the reason for absence of goiter in this community/low land area?**

**P:** Hum…..

**I: What about anemia; is there women and adolescents suffering from it?**

**P:** There is no anemia in adolescent girls, but it is occurring in pregnant and lactating women.

**I: What are the reasons for that?**

**P:** Shortage of food intake

**I: Is that to mean no food source/crops?**

**P:** No, there are crops or agricultural inputs but they fail to use it appropriately. They take what they have to market rather than feeding at their home.

**I: What about non-communicable diseases like diabetes and hypertension, are they the problems in women of this community?**

**P:** No

**I: Do you have any mother who is receiving TSF this year?**

**P:** No, though we are screening them monthly by conducting home visit and we identified the cases, there is no organization to support TSF.

**I: What about the magnitude of under-nutrition this year?**

**P:** It is lower and better than last years.

**I: Why such improvement has seen?**

**P:** If there is TSF support, they don’t use what is produced at their home. They simply wait for TSF for feeding rather than using the products at their home. But, if the TSF provision is interrupted, they start to eat what is in their home and feed their kids as well.

**I: Are there women or adolescent girls who are overweight or obese?**

**P:** In this area, [shouted] NO...

**I: Is there any food insecurity problems?**

**P:** Regarding the crop supply, in some years there is drought then the productive safety net program that support them. The REST (Relief society of Tigray) also supports them. For example, currently the community can consume 3-4 times per day. Now there is no hunger.

**I: Why such improvement has come?**

**P:** It is because now there is increase in community awareness, there are also HEWs, agricultural extension workers, and there are fertilizer, improved seed, and so many things are there like again the poultry production, and others. Therefore, people are changing through time and considering self-support rather than begging. So, nowadays hunger is going to be averted.

**I: Which women groups are most affected by these nutrition problems, Pregnant or lactating?**

**P:** Lactating are highly affected than pregnant

**I: Why?**

**P:** The pregnant cares herself and think about her fetus. But, the lactating can have birth of twin or single birth and the breast milk may not be sufficient and she may not have adequate income, then she focus for her child and forget herself. And, bleeding during birth can cause especially home birth.

**I: Are there home births in this community?**

**P:** It is not many, last year there were 11 home births, but this year there are no.

**I: Why women are conducting home birth?**

**P:** Because they have no birth preparedness, and when the husband is on farm for work and lack any one who support her to reach health facility, when her kids are at school, and when she is alone at home when the birth starts. Otherwise, home delivery is not allowed for the mothers. But, if there is WDA member then she takes her to health center by calling for ambulance. There were mothers who gave birth on the road.

1. **Nutrition priorities in the community**

**I: What maternal nutrition (pregnant, lactating and adolescent girls) interventions are the priorities in this community?**

**P:** For adolescent girls, we teach nutrition education at schools, and the four major sectors such as Kebelle administrative, agriculture, Health extension, school director, and police, these all sectors go to school once monthly, to provide education on nutrition, peace, and others. On other side, we educate fathers at every gathering of people like on work for safety net such as soil conservation works which are done monthly every Wednesday.

**I: What are prioritized activities for women nutrition?**

**P:** Educating the mother on nutrition like by calling women in to one place in their village we were demonstrating how to prepare food/porridge from diversified food sources. We also teach them when they come to receive services such as TSF/blended flour, immunization, and when we go to soil conservation works/dam. But, always we teach them theoretically.

**I: What nutrition interventions have the most resources allocated to them?**

**P:** There is no budget for all activity. It is by ourselves; sometimes we may get it from government employees if there is any. It is prepared from milk, blended flour, egg, oil……

**I: Who provide this for you?**

**P:** These were supplied by NGO,

**I: Can you access them from the community?**

**P:** It can be taken from the community, but there is no irrigation. We can get only two-three cereals from this community, such as Sorghum, Teff, and “boloke”. They are not enough to prepare a blended food, but we buy the left food items from the market from Semere. Therefore, we can provide demonstration/education.

**I: With whom you are providing demonstration?**

**P:** Most of the time education is provided by me. Sometimes the agriculture extension workers if I called them are participating on the demonstration.

**I: Who provides money you to buy the crops or food items necessary to prepare a blended food for demonstration?**

**P:** It is me, nobody gives me money. If I have money I buy otherwise nobody gives me money.

**I: Do you think it is necessary for your institution to get involved in work aimed at improving maternal nutrition?**

**P:** Yes it is very important, because, agriculture can provide assistance in gardening; they give seeds for WDA and WDAs’ work on gardening. And they support on using irrigation to cultivate vegetables in the garden as there is spring water in the area. We also provide to the community to have gardening as it is important. Education/school is also important as students who thought can go back to the household and community and they act as main source of information for their parents. Kebelle administrative is also important to deliver information on meetings, associations, and work with religious leaders. So, since the administrative has acceptance by the community, he can deliver education there at community meetings.

**I: Can you tell me some of the maternal nutrition interventions that should be prioritized?**

**P:** Mothers should have adequate break. For pregnant women, we have communicated with agriculture sector and provided a pregnant women about one year maternity leave starting from 4 month of pregnancy from productive safety net program.

1. **Nutrition interventions that improve adolescent and maternal health**

**I: What kinds of nutrition interventions are in place to improve maternal health in this kebelle?**

**P:** To improve health of mothers in our community we are providing education on nutrition, and family planning. There is education on preparing nutritious food from what they have and incase when they have under-nutrition they are counseled to come health post and receive care like plumpy-net, and TSF. And, we support them to get these services. And, then when they discharged from the program, they are also told/ advised to feed blended food prepared at their home. So, they are advised on feeding diversified food.

**I: What kinds of nutrition interventions are in place to improve adolescent girls’ health in this kebelle?**

**P:** They are counseled to follow their education.

**I: Does it have relation with their nutritional status?**

**P:** Yes, as they may get married before 18 years, because in this area if the girl has no education, she is more likely to be victim of early marriage. Then they give birth, harmed by under-nutrition and they may die and can be ill. Therefore, we are promoting education.

**I: Is there any nutrition interventions done for adolescent girls?**

**P:** The education on nutrition is given at school by teachers, and nutrition club at school. We go to school monthly, and teach them through drama, and poem. We also show how to prepare balanced diet from various items. The school has no land for gardening, but last time the teachers provided crops from their home to demonstrate the procedure about blended food preparation.

**I: Do the pregnant receive counseling during pregnancy?**

**P:** Yes, they are counseled as they have to have clinical check-ups, place of delivery, to take tetanus vaccination, counseled to take extra meal, if she is taking three times per day before, she is advised to increase it by one.

**I: Do the lactating receive counseling?**

**P:** The procedures of feeding breast milk for their child, the number of times to feed, and the duration of breast feeding, and exclusive breast feeding, as she should introduce complementary food. They are advised to feed 8-12 times the breast milk. There is also nutritional screening for pregnant and lactating women monthly.

**I: Is there any nutritional screening program for adolescent girls?**

**P:** No, but we provide TT vaccination.

**I: Why there is no nutritional screening for adolescents?**

**P:** We cannot address all age groups since we have about 16 health extension packages to work on.

**I: Is there a job title to you to screen adolescent girls?**

**P:** No, for providing education there is structure that forces us to teach adolescents but not for screening.

**I: Do women are advised on nutrition sensitive agriculture?**

**P:** Yes, we advise them on it. There is spring water at 30-40 minutes distance from the community and they are told to fetch that water and cultivate spinach, and garlic. So, by considering this we are working with WDA. So, there is work started now.

**I: Are they advised about water, sanitation and hygiene?**

**P:** Yes. At WDA, women are advised to have separate liquid and solid waste management system, separation of house for cattle and humans, to keep her and children personal hygiene, to wash their hands before feeding their child and after handling the fecal materials of the child, when after the toilet. So, these are environmental and personal hygiene related education given for women.

**I: Do women are advised on the use of Insecticide treated bed nets (ITN)?**

**P:** Yes, as the pregnant and lactating women are vulnerable to malaria, they are advised to use ITN and how to use it, to avoid water pooling areas in their environment so these educations is given at DA levels.

**I: What about deworming and vitamin A supplementation?**

**P:** We provide vitamin A for 6-59 month children, and deworming for 2-5 years children. But, these services are not given for pregnant, lactating women and the adolescents.

**I: Is there school feeding programs for adolescents in school?**

**P:** No

1. **Implementation challenges and community factors affecting access to maternal nutrition interventions**

**I: In your opinion, which of the above programs are being implemented successfully (i.e. in the most effective way?)**

**P:** All the services we have discussed are very important. Deworming that has been provided every 6 month for children as it has been done as planned. Vaccination is also effective and the community is utilizing it very well. But, implementation of diversified food consumption is not that much effective because it needs enough number of trained HEW in the kebelle to reach all the villages and households by nutrition education. And, it would be better if one extension works at field and one at office. We are two but it is difficult to reach the entire community. But, if we were 3-4 HEW, we would go to each village and household and teach and demonstrate there and we would be effective. There is also inputs problem to consume and prepare a blended food in our community because the area is dry and there is no irrigation, therefore it is difficult to ask the community to bring the crop items important for preparing food from diversified food items. Therefore, it better to have its own budget.

**I: Why budget is needed?
P:** There should be budget to buy inputs to prepare blended food from various items.

**I: In your opinion, which of other programs mentioned above are less effective?**

**P:** There is ANC visit her at health post until fourth visit. But, it would be good if there is instruments, supplies and equipment’s for us to attend delivery her at health post. Therefore the community has easy access to the service.

**I: Is the health center is too far for this community?**

**P:** When I compare it with other setting, it is not far for our community as it takes 2 hours to reach. But there are settings in our woreda that take 1 day, and 6 hours to reach health center. But, it would be better if we have all supplies to deliver the service for mothers like delivery.

**I: How many mothers know as the nutrition is important for them?**

**P:** All women with in DA have knowledge on the importance of nutrition very well. They know what is needed for them. The majority of the community members have also awareness on the importance of nutrition for mothers.

**I: Is there a relationship between educational status of women and access to interventions?**

**P:** Educational status has no effect on utilization of the services for nutrition. The awareness is the same in both educated and uneducated women. However, the awareness that we have created is not satisfactory because if they were aware of nutrition very well, they wouldn’t sell their agricultural products rather they feed their children and themselves. Therefore, there education provided is not sufficient still we need more work to create awareness.

**I: What are other challenges to deliver the nutrition interventions?
P:** We have no challenge rather than the supply. Otherwise, the community is highly committed to use them as we are teaching them at every points we meet them like safety-net works, religious programs…

But, it would be better to show them practically as theoretical sessions are easily forgettable.

**I: What community related beliefs and norms are preventing access to interventions?**

**P:** There are no norms that prevent them. The interventions are culturally accepted.

**I: Why the interventions are acceptable culturally?**

**P:** In the past, there were so many diseases, there was no environmental cleanliness, there were so many people died and diseased by malaria, there was high child mortality. However, now there is good health in the community, no child death, healthy live births, by taking this all in to consideration, the community is highly supportive and take our education very well. And, they know as the nutrition interventions are helpful therefore, it is highly accepted by the community.

**I: Are the interventions are accessible to women and adolescents?**

**P:** There are 2-3 villages which are far that take 1 and half hour and 2 hours. But we go there and we teach about nutrition and services that are provided at health post. We inform them about services and schedule, so they can easily access the service.

**I: How convenience is interventions to the women?**

**P:** We teach at each village about nutrition monthly, assess nutritional status using MUAC, then it there are case of malnutrition, we provide education and schedule just to take care at health post.

**I: What other resources are needed to provide interventions?**

**P:** We need separate place to educate the community, and to put every material needed for nutrition interventions, as we don’t have it at the kebelle, even the FTC is together with administrative office and it is not convenient and has no materials for nutrition education. But, we have water pipe at health post, which may not be used for irrigation. There is spring water 30-40 minutes far, but it disappears at dry seasons like at November. So, this is again a challenge.

**I: How do you evaluate the commitment of the interventions providers in the kebelle?**

**P:** We are working all if we are healthy.

I: How the challenges mentioned so far can be solved?

P: Constructing separate room for nutrition interventions is good; therefore the woreda can manage it.

1. **Multi-sectorial collaboration to improve maternal nutrition**

**I: Do you feel it is necessary for your institution to work with other sectors/institutions to address maternal nutrition?**

**P:** Yes, I have mentioned the reason before

**I: Do you feel it is necessary for your institution to work with other sectors/institutions to address adolescent girls’ nutrition?**

**P:** It was good, but yet we did nothing about adolescent nutrition.

**I: You have mentioned necessary sectors for nutrition interventions, which other sectors do you feel are necessary to work with?**

**P:** Water supply office as it supply water and maintain the availability, and it is used for cooking food by mothers. They can also supply solar energy for cooking to improve health of mothers.

**I: How do you evaluate the level of collaboration among sectors in nutritional interventions?**

**P:** At school, we established nutrition club, and providing education so we would expect change has come if we work collaboratively. Every sector has involved and providing its effort to the success. But, the work is not that much satisfactory.

**I: Why it is not satisfactory?**

**P:** The environment is not smooth, because if a sector has plan to do other work, then leaves the plan of nutrition due to work overload.

**I: How this challenge can be addressed?
P:** They have to employee personnel that can handle issue of nutrition together with us.

**I: For multi-sectorial action that effectively works to improve maternal nutrition at all levels, what kind of change in terms of the way stakeholders work together is needed?**

**P:** We should mobilize the community with in short time and work together. So far, most of the time this work was done by HEW, but it would be better if every sector should be committed for this intervention. So far, I was begging other sectors to go to work together. All sectors should give equal attention to deliver nutrition education and intervention. So, there should be awareness creation for all sectors to give responsibility about nutrition.

**I: Do you have Joint plan for nutrition?**

**P:** Yes, we planned together for a year and the time period to accomplish activities was also set, then evaluation is made accordingly. We have interface for kebelle level nutrition intervention. Evaluation is made monthly on 12^th^ day of the month and have minute. We have put the role of each sector and evaluate them. During the evaluation we identify the non-performed activities and we set time period to accomplish and evaluate at set time. There is also weekly meeting (every Friday) to evaluate our weekly performances.

**I: What type of resistance to the needed change do you perceive or have you experienced so far?**

**P:** No, because we have all necessary thing in the community like milk, and egg. And, if the community use these resources it can be successful.

**I: To what extent does your institution participate in the multi-sectorial nutrition coordinating body at the woreda level?**

**P:** I have mentioned before, we have full participation. We have given education at different levels and personnel in the kebelle.

**I: How effective are the coordinating platforms in enhancing multi-sectorial coordination?**

**P:** It is too child, we need to work more hereafter.

**I: What needs to be done to improve the capacity of these bodies/platforms for effective coordination?**

**P:** Direction should be given for all sectors about importance of working on nutrition and all sectors should be made responsible to work on nutrition. All sectors should aware of as nutrition is no only work of HEW or Education rather it is the responsibility of all. So far, nothing is told for the sector as they are responsible on nutrition interventions. Other sectors say nutrition as “it is the work of HEW and we don’t have anything related with nutrition”. So, the work should come to all sectors from higher levels.

**I: What opportunities do exist to promote multi-sectorial collaboration of nutirition in this kebelle?**

**P:** The presence of all experts living in the same village or kebelle, we are neighbours to each other and nobody goes to other site, therefore, it is easy to work together.

1. **Other interventions that influence adolescent and maternal nutrition and health outcomes**

**I: In your opinion, do think delayed marriage (after 18 years) improves maternal nutrition?**

**P:** Yes, early marriage can have negative effect on maternal nutrition. The mother become thin and her child can be thin.

**I: How the nutritional status of mother affected?
P:** Because she is small and thin, she further exposed to thinness and fistula finally she may die.

**I: In your opinion, do think increasing the space between each birth improves maternal nutrition?**

**P:** Giving birth before, the previous child born is not well grown is not good. Because, the child cannot get balanced diet, but if the birth is after 2 years he can get enough complementary food. And, the mother will become thin and the milk production will be too small so her child cannot get sufficient breast milk then the mother will have psychological problem.

**I: What programs or activities promote increased birth intervals in this kebelle?**

**P:** The awareness creation is the only thing that we have. We provide education to plan birth after 5^th^ year of previous birth. We educate them on family planning and provide it.

**I: Can you tell me about any programs or policies in place in this woreda to prevent early marriage?**

**P:** We have a group/team that approves marriage.

**I: Can you think of any more programs or policies to prevent early marriage?**

**P:** Education at school, religious institutions and community meetings.

**I: In your opinion, are these programs or policies effective?**

**P:** Yet, they are very important because as when there is education there can be everything. No girl can marry before 18 years, if there is the case the girls report to teacher, HEW, or police and then the issue can handled and marriage is prevented. So, we have no girl that married before 18 currently. There is no forced marriage currently.

**I: Is there any girls who want to marry before 18 years?
P:** Yes, the request the kebelle team that approves marriage by saying “I am ready to marry and I can marry now” then the team cancels the request and prevent her from early marriage.

**I: What are the community factors that affect age at first marriage?**

**P:** In the past, there was community influence. Now there is no parental influence. In the past time there was misunderstanding as the 14-15 year old girl can marry and assume education has nothing to do for females, assume she leave them and go to other setting to marry.

**I: Can you think of any other opportunities to prevent early marriage?**

**P:** Presence of interventions at schools such as peer to peer (Acha la acha) clubs in school. And education is given in the club and learn each other, they know as there is no forced marriage. There is education on the health impact of early marriage.

**I: Can you think of any other opportunities to increase birth spacing?**

**P:** Presence of contraceptives, [thinking] that is it. Schools to provide education are also opportunities.

**I: What lessons have you learnt regarding adolescent and maternal (pregnant, lactating and adolescent girls) nutrition in this Kebelle?**

**P:** We trained training on maternal and children nutrition, but not on adolescent nutrition in the summer by NGO. We have trained on how, what and when to feed children and mothers. I have learnt feeding what is in the community.

**I: What lessons have you learnt regarding multi-sectoral coordination of nutrition in this woreda?**

**P:** So, women assume the TSF that they receive as one food item like the flour of a single crop, but they don’t know it as a combination of various food items. When we tell them to prepare such blended food from their own crops but they prefer to take their crop to market rather than feeding at home. They assume as they have only one item and want to bring other for their kids.

**I: What opportunities do exist to promote maternal (pregnant, lactating and adolescent girls) nutrition in this woreda?**

**P:** There is water point, charcoal, and the WDA that makes them friendly to each other, have also experts and inputs. So, we can do it more.

**I: Thank you very much for you time and detailed explanations!**

**Summary**

1. **Common maternal (pregnant women, lactating women and adolescent girls) nutrition problems in the community.**

- In this community, women are at risk of various nutritional related diseases like stunting, and thinness.

1. **Nutrition priorities in the community**

- Nutritional screening is done for pregnant and lactating mothers but not done for adolescents.

1. **Nutrition interventions that improve adolescent and maternal health**

- To improve health of mothers in our community we are providing education on nutrition, and family planning.
- There is education on preparing nutritious food from what they have and
- In case when they have under-nutrition they are counseled to come health post and receive care like plumpy-net, and TSF.
- Then when they discharged from the program, they are also told/ advised to feed blended food prepared at their home.

1. **Implementation challenges and community factors affecting access to maternal nutrition interventions**

- It is difficult to reach the entire community as we are only two HEWs
- There is also inputs problem to consume and prepare a blended food in our community because the area is dry and there is no irrigation,
- The awareness is the same in both educated and uneducated women.

1. **Multi-sectorial collaboration to improve maternal nutrition**

- At school, we established nutrition club,
- Every sector has involved and providing its effort to the success. But, the work is not that much satisfactory.
- The environment is not smooth, because if a sector has plan to do other work, then leaves the plan of nutrition due to work overload.

1. **Other interventions that influence adolescent and maternal nutrition and health outcomes**

- The awareness creation is the only thing that we have. We provide education to plan birth after 5^th^ year of previous birth.
- We educate them on family planning and provide it.
- Education at school, religious institutions and community meetings.
