# Supplementary material for: Barriers to utilize nutrition interventions among lactating women in rural communities of Tigray, northern Ethiopia: An exploratory study
Source: PLoS One. 2021 Apr 30;16(4):e0250696. doi: 10.1371/journal.pone.0250696 (PMC8087028; doi:10.1371/journal.pone.0250696)
Supplement: S2 File — (ZIP) [file pone.0250696.s002.zip › S2_File.Doc/Community level Key informants/059_FGD_Men_Lemlem Kebele_Samre woreda.docx]

## Seharti Samre Woreda, Lemlem Kebele- MEN FGD

| **Introduction:**  Hello, my name is Dejen Yemane. I am from Mekelle University. Thank you for taking the time to speak with us today. We are doing research on the factors that influence the nutrition of mothers and adolescent girls in collaboration with the Regional Health Bureau and UNICEF. Your participation is very valuable. The things that you tell us will be used to improve nutrition programs and services for women and adolescents in the region and the country. We will not share your names when we report our results.  However, we will record the discussion so that we can capture all the ideas that are shared. We have several questions to ask you that we have prepared in advance, and we will ask you all to say what you think about each question. To ensure the privacy of everyone here, we ask you not to repeat what we discuss outside of this group. The discussion will last for 1-2 hours. Do you have any questions before we begin? If you think of any questions as we proceed, please feel free to let me know. If it is all right with all of you, we will turn on the tape recorder now.  **Section B: Interview details**   1. Zone: South East 2. Woreda: Seharti Samre 3. Kebele: Lemlem 4. Facilitator’s name: Dejen Yemane 5. Date of discussion: November 12, 2017 6. Discussion start time: _________________________ 7. Discussion end time: __________________________ | | | | |
| --- | --- | --- | --- | --- |
| **Section B: Socio-demographic Information** | | | | |
| Name of the FGD participant | Age | Marital status | Education level | Occupation |
| 01. Abraha G/zgi | 40 | Married | 10 | Kebele propoganda |
| 02. Keshi Mebrahtom | 30 | Married | 9 | Youth league |
| 03. Alem Berhe | 78 | Married | Illiterate | Farmer |
| 4. Girmay Wuyu | 76 | Married | Illiterate | Farmer |
| 05. Hiluf Aynalem | 66 | Married | Illiterate | Farmer |
| 06. Tesfay Bula | 52 | Married | Illiterate | Farmer |
| 07. Weletekle Beyene | 41 | Married | Illiterate | Farmer |
| 08. G/tsaddik Gessesew | 34 | Married | 4 | Farmer |
| 09. Teklu W/gergis | 45 | Married | 4 | Farmer |

**Section 1: Common maternal (PW, LW and adolescent girls) nutrition problems in the community.**

- 1. **What do pregnant women do to stay healthy in this community? Why?**

**Participant #2:** there are limitation in our mothers. Our government has declared every mother to deliver at health institution and to avoid home delivery, to follow ANC until 4-6 month. This is alright and WDAs follow its implementation. But you might see mothers who don’t follow ANC and delivering at home. Regarding nutrition, in rural areas like ours, foods for pregnant, lactating and adolescents, there are 9-10 but there also drop outs from school especially if they are failed from education. In such circumstances, the farmer is not capable to pay for private education because the resource we have is land. There is a strict guide from the government that if there is home delivery she will be legally enforced by WDA and kebele council. If she delivers at home while she told to deliver at health institutions, we have set punishment from 50-100 birr. At the same time the one who assists home delivery will be also punished because instead of telling her to go to health institution. Regarding nutrition, what can they eat here? After baptism she will go for mowing and weeding carrying her child. There is no rest. On top of that she is responsible to prepare food for the family and fetch water. But the rules of the government are good and acceptable.

**Interviewer:** thank you participant #2. Any additional point. Okay participant #4

**Participant #4:** the issue is as it has been said, the problem is not fully solved. It is just about telling it back, regarding is it solved or not, it is not yet solved. Did they started or not, they have started? There are many wise women who go to health facilities for health services. Regarding nutrition, our capacity is decreasing from time to time because the area is affected by drought. The community is administered by government support unless otherwise, we don’t have anything to bet on.

**Interviewer:** thank you participant #4. Okay participant #1

**Participant #1:** as it has been explained, to explain on what do pregnant women do to stay healthy (…new participant was joining the group), in our community the idea is presented by our government and the health technical persons (to mean HEWs) and near to 40% of the community is following their advices. Women get services at health facilities and before that there are some limitations like we let them to work while they should take rest. One thing that we take it as a limitation is instead of taking rest and to recover their blood, with the strong sunlight there is food shortage. The good thing is that except few, pregnant women apply government initiatives like being screened every month and institutional delivery. In addition, regarding adolescents, we have learned from our government that adolescents should marry after the age of 18 years not to be challenged during delivery. We compensate the problems by applying the government initiatives and we are interested women to apply it. But sometimes adolescents might not score passing mark and parents face food shortage and lake of money. Overall, we are good at adopting and implementing health education in our community. Thank you.

**Interviewer:** you told me that if they fail to score pass mark adolescents will sit at home, so doth this lead to early marriage? If they fail, parents might be incapable to cover payments for private education and as a result they will sit at home. Is there an attitude that motivates the family to their early adolescent to marry?

**Participant #1:** parents want their child to learn but due to lack of financial capacity they might allow their child to marry.

**Interviewer:** additional idea. to add the question, in our community what do pregnant, lactating and adolescent girls do to stay healthy?

**Participant #3:** okay, the agenda is good, and we have informed our community after we hear that you are coming. It is about pregnant, lactating and adolescent girls’ problems. In previous regimes, as you all know women were dying due to pregnancy and delivery. But now due to his excellency let prim-minister Meles Zenawi saying “a mother should not lose her life while she is giving life” and our government’s policy death of mothers has decreased, and this is working well in our kebelle. Almost there is no home delivery except at health institution. Unless under difficult circumstances or due to unavailability of ambulance service, no mother is delivering at home. This has also happened in 2009 E.C, unless otherwise in the year 2010 E.C institutional delivery is almost 100%. Because of this, they will be free from bleeding, other related health problems and she will care her child. During pregnancy they have 4-5 times pregnancy follow ups and if they have shortage of food and weaknesses Woreda will arrange to the extent she will stay at the health facility until she recovers.

Other regarding work, with the farmer’s life, women work with the men. If it is mowing and weeding period, she will go with the men. Though it is not like the past they wander because to survival they must work.

Whereas in adolescents, there is vaccination in schools and they get enough advices. Traditional practices like early marriage, uvulectomy and FGM are also avoided due to the wider health education by HEWs and WDAs. These are conditioned and punishable in the constitution. There is no early marriage in our community. If an adolescent girl is going to marry, her family will report to marriage committee and marriage committee will either allow or prohibit the marriage after verifying her age.

If an adolescent didn’t score pass mark, she will stay at home and at the same time parents who have financial capacity also send their child to Mekelle to join privet or governmental colleges. But adolescents from poor families will stay here, and they look in to job vacancies. In this community as the priest said early marriage is forbidden and it is enforced by law.

**Interviewer:**  is the low community by law or governmental low?

**Participant:** it is governmental law and it is approved in the kebelle council. Parents who marry their adolescent girl while she was under 18 will be evaluated and accused though this doesn’t happen at our kebelle. Both the bride groom parents will be accused and to filter it out properly there are cases referred up to Ayder comprehensive specialized hospital. If she is 18 years old it is okay, if not they will take legal penalty.

Though, I have already explained it before, we have endorsed community by law for home delivery in our kebelle. Like what my brother has said the one who assist home delivery has no legal right to assist delivery, so she will be asked.

**Interviewer:**  how much will you punish for someone who assist home delivery.

**Participant:** for example, they were penalized at social justices and it was started up to 30 birrs but the kebelle council has decided more than that. But after discussing with Woreda, the main thing is convincing herself. Instead of going to health facility after giving birth at home, it is good to deliver at health institution to keep her life because they will give her first aid in health facility. Now the WDAs are also following it. For example, I am kebelle council member, we receive list of pregnant from each cluster. I have a list that show when will every pregnant will give birth and similarly priest Mebrahtom, youth representative has the list. There are community conversations with people coming from region to discuss on these issues. Thank you.

**Interviewer:** very good, thank you participant #3. Do you have additional points? If it is okay let’s proceed to the next point. Next, Participant #5 don’t you have different idea

**Participant #5**: I don’t have.

**Interviewer:** okay, if you don’t have different idea we will proceed to the next point.

- 1. **In your opinion, what are the common nutrition problems in the community especially in pregnant, lactating and adolescent girls?**

**Participant #6:** okay on the nutritional problems, yes there is nutritional problem because there is no food sufficiency in the lowlands. Nutritional problems happen because of unavailability and utilization problems. There are deficits and if there is deficit people will face diseases.

**Interviewer:** for example, what type of diseases can s/he will face?

**Participant #6:** if there is food shortage, there will be physical and mental deterioration. But we are mostly affected by malaria because this area is lowland. Especially cerebral malaria is common in our area. I am finished.

**Interviewer:** okay, Participant #3

**Participant #3:**  to shorten it, if you come to nutrition, because of health education and other interventions the community is in a better position compared to the past. Starting from its clothing and feeding there are few changes. But the main thing is nature. If they don’t have they can’t do anything and even those who have send their products to market. There are supports that must be given to especially elders, pregnant, children and adolescents. What should a pregnant women feed? For example, from the products that are send to a market like butter and egg. Even those who have animals, instead of using meat by slaughtering the goats they prefer to count them.

**Interviewer:**  there is a tendency of taking everything to market. What are the reasons that hinder us to use what we have at hand?

**Participant 3#:**  I have told you that the bottom-line is nature. The community did not say that let me eat what I have at home. Sending products to market is also associated to cover life expenses and REST (ማረት) debt. Even the well to do families wants to count the number of animals instead of knowing the benefits of eating meat and how frequent should they eat meat per month. There is lack of awareness in the benefits.

**Interviewer:** do HEWs teach the benefits of animal source foods

**Participant #3:** yes, they teach us. HEWs give us health education, especially on how a pregnant woman should feed. This is also presented by agriculture office and WDAs. Just to eat enriched food. There are also posters that show what kind of foods shall we take and how to prepare it.

Regarding the diseases, in elders and youths, it can cause bone slip, tiredness, night blindness up to permanent blindness. In elders due to lack of personal hygiene and in children they will have shrunken eyes and yellow color.

**Interviewer:** what about in pregnant women?

**Participant #3:**  it is also similar in pregnant women. They will have tiredness, especially during delivery they will be tired before 9 months and they will be admitted. There are pregnant women who are admitted 1-2 months until they deliver and provided supplementary food. HEWs inform mothers with low MUAC to stay at health facility because they have food shortage. However, had it been she is well nourished her blood and other vitamins will be in good status. Though HEWs teach what and how frequent a child and a mother should feed to be healthy, there are problems.

**Interviewer:** additional points, Participant #1

**Participant #1:** okay, Abraha has already explained it widely. But what I want to say is that mother who took adequate food will have full blood, be physically strong and she will not get difficulty to deliver. She can easily deliver. But in mothers who did not get adequate food, the fetus will be exposed to risk, and the mother will also be tiered. Thus, the mother will be subjected to operation due to lack of energy to push the fetus.

There is also lack of personal hygiene. Because we rush to agricultural activities and animals, and we also touch soil, then there will be infections that will lead to vomiting due to lack of hygiene. In addition, they will develop edema and leg thinness. This is not yet fully eliminated. Now we want to thank is, in the past people especially children were dying by malaria but now either by education or by treatment child mortality has decreased.

For health insurance we pay 142 birrs per year and if we are diseased we are easily treated. Because, once the family pays 142, he will get health services freely for a year. I have finished.

**Interviewer:** very good, thank you participant #1. Additional points,

**Participant #4:** it is already said by my colleagues. In this area we are affected by drought and water shortage. As you can see the farm due to shortage of rain and pest it did not bear fruit. We are affected especially by shortage of water.

**Interviewer:** if you don’t have additional points, do you think that stunting and wasting are common in your community? Stunting is shortness whereas wasting is thinness. Do you think these could be associated with their nutritional status?

**Participant #2:** we consider stunting and wasting as a natural phenomenon. But the constitution says that the one who grow in a good and bad way will not be equal. How come? There are many short and thin people, but we question why are people short and thin while they are eating and drinking? But it has its own reason. Those who are illiterate like us they say that somebody is always thin and short but what makes him short and thin is his/her way of living. We repeat what we have eaten yesterday (there is no food diversification), we have a problem in keeping our personal hygiene and latrine utilization. These all together will serve as source of health problems.

Last week there was council meeting, one who grow well and one who grow keeping animals will not be equal. The one who grow keeping animals and wandering here and there will not increase his/her height/weight proportional to his/her age.

**Interviewer:** very good, if there is additional point, is stunting and wasting common in our community? Do you think these could be associated with their nutritional status? Let me give the chance to participant #5. To repeat the question, is stunting and wasting common in our community? Does it have association with nutrition beyond nature?

**Participant #5:** we consider stunting and wasting as nature unless otherwise we don’t associate it with nutrition. But naturally there are short and thin people in our community.

**Interviewer #:** very good, participant# 3

**Participant# 3:** as it has been said by my brother before, we were considering it as a nature and hereditary. There are hereditarily good. If there is good caring, s/he will grow well. There is difference in weight and height. This is related with inadequate feeding. Last time L10K people were here and we have discussed well about nutrition and other related health issues focusing on children. We understand from this meeting that one who is adequately feed and the one who is not adequately feed are quite different. Leave alone human, if there is difference in feeding even two oxen born from one cow varies. The one properly feed will grow well and while the one who is not well fed will be weak and thin. Overall, although there is improvement in thinness and shortness are they are common in our community.

**Interviewer:** very good, if you don’t have additional points, I will proceed to the next question. Do you think that WOMEN in this community are suffering from Micronutrient deficiencies like anemia, night blindness, goiter and others? How do you see it? Okay participant #4

**Participant #4:** for example, I myself cannot see after sun set and this could be night blindness. But I could not get medication.

**Interviewer:** how do people see night blindness? Do they associate it with nutrition or aging?

**Participant #4:** I am associating with aging, but I was suffering even when I was young. So, when I see it now it is associated with nutrition. Because I have seen the manifestation starting from 1977 E.C when we were at the refugee at Sudan. Even though our government was distributing treatments, but since there were people worse than me I did not get any treatment that time. (laughing)

**Interviewer:** okay, participant #6. Did you get the question? The question is, do you think that WOMEN in this community are suffering from Micronutrient deficiencies like anemia, night blindness, goiter and others?

**Participant #6:** thanks to God we don’t have goiter in our community. But regarding night blindness, yes, our vision is impaired, and we are experiencing poor vision at night or in dimly lit environment. But my question is, why did you select us only? Why don’t you collect the community?

**Interviewer:** the recorder was paused and explained why they are selected for this study, because this participant joined lately after we finish the first question.

**Participant #6:** Regarding health of women, the government is doing good. They are promoting institutional delivery and it is okay. But be it from night blindness or anemia we have impaired vision.

**Interviewer:** any additional point. Okay, participant #3

**Participant #3:** okay, the question is regarding anemia, night blindness, goiter. This are the main health problems affecting our farmers. These are prevalent in our community. For example, goiter is common. For example, Mihreta has gone to health institution. Most of them suffer during their pregnancy.

**Interviewer:** what do you think is they experience during their pregnancy?

**Participant #3:** the community just simply understands goiter is worsen during pregnancy. Unless otherwise, we do not know the cause and its meaning. But my presumption during pregnancy due to blood pressure, work load, weight gain and tiredness could be the reason. In addition, I have also an assumption that this could be aggravated by taking foods not suitable for goiter.

**Interviewer:** in our community, do we have foods assumed to aggravate goiter?

**Participant #3:**  we don’t have, rather they associate it with pregnancy and delivery. Scientifically it might have but in general this is common in women.

Regarding anemia, it is common in the farmers and this is due to lack of nutrition. One of the participants was mentioning that he is suffering from night blindness starting from long time ago and this could be because in our community there is no food diversification. For example, there is no habit and awareness of feeding vegetables that are reach in vitamins that can prevent night blindness. Many young people have impaired vision in our community after 12 O’clock. This is night blindness. Health workers advises us to take red teff in the form of soup or porridge or in a kind of injera. If you go to health facility, you can get vitamins. So, be it the night blindness or anemia is associated with poor nutrition.

**Interviewer:** do you have any more to add? If not, lets proceed to the next question. Do you think there could be any association between nutrition and occurrence of non-communicable diseases among the women/girls? Why? Are there such diseases in this community? (silence) Am I clear?

**Participant #1:** okay, there are non-communicable diseases like stunting and others that disfigure human being. This kind of problems seldom occurs in this community.

**Interviewer:** to elaborate my question, non-communicable diseases are like hypertension, diabetes, cancer, overweight and others associated with nutrition.

**Participant #3:** overweight, hypertension and cancer are not common in our community. Because our community is busy of different activities and the feeding still did not invite for such kinds of diseases. But regarding diabetes, we have one with diabetes in our kebelle and he is taking medication. He is being followed by health workers and advised what kinds of foods to take. You can ask me on the communicable diseases.

**Interviewer:** you can continue your reflections on the communicable diseases associated with food.

**Participant #3:** for example, there is diarrhea and cough that occur in special seasons can be transmitted by leaving together and sharing drinking materials. If you take TB, it can be transmitted by food and breathing. Diarrhea can be transmitted by eating and drinking together. And HIV is also one of the communicable diseases, though it has no association with food.

**Participant #4:** it is already said by Abraha.

**Participant #1:** though it is rear there is overweight in one family and the community associate it with hereditary. For example, though they are poor, there are members of a family who are overweight in Gurare (it is village’s name). TB is also common in our community.

**Interviewer:** very good, the next question is, do women/girls in this community increase their weight/height proportional to their age? What do you think on why women/girls in this community would not increase their height proportional to their age? Could it have relationship with their nutrition?

**Participant #1:** okay, thank you. In this community, children are not increasing their height/weight proportional to their age when we compare them with children growing in urban areas. If you compare two children of the same age, children grown at rural is thinner and shorter than children grown at urban areas.

**Interviewer:** what could be the reason for not increasing their height/weight proportional to their age?

**Participant #1:** the reason could be difference in nutrition. Because rural children are not getting adequate food and hygiene.

**Interviewer:** does this problem especially wasting occurs in women/girls in your community?

**Participant #1:** it is similar. Because

**Participant #7:** Due to shortage of food children develop kwashiorkor and stunting in this community. This could be from poor nutrition, care and sanitation. Every human is equal but because of nutrition there is difference in height and weight. As it has been said rural and urban children are incomparable. Urban children grow well by the age of three whereas rural children are still growing down at the age of ten. The difference is because of nutrition and living style.

**Interviewer:** thank you, if you have any more to add

**Participant #3:** there is slight change compared to the past. There are rural children who look children who grow at urban. If you look at the rural youths, they are almost like their urban age equivalent both physically and educational performance.

**Interviewer:** Is there a situation when WOMEN suffer from shortage of food? In what situation do you think this happened? How frequent does it happen?

**Participant #2:** Government is teaching the community to space births. But there are mothers who give birth in every one-year interval. If there is no space between births, it will cause food shortage.

**Participant #3:** food shortage is common in the summer season on the months of June, July because farmers sow the seeds. During these times farmers will get tiered because they will spend whole day without feeding enough food. Similarly, they will also face food shortage on the month of September until they collect their crops from the field. That is why the community says “መስከረም ክታኣቱ ትኹስትሮም ክትወፅእ ተተስክሮም”. Because September is a transition month between the summer and the spring. In the year 2008 E.C there was a drought and there was drought relief assistance by our government. Similarly, we have sent detailed study of this year because there is a drought this year. Especially we face food shortage during drought times.

**Summary**

- Stunting, wasting, anemia and night blindness are common nutritional problems in the area where as non-communicable diseases are not public health problems
- In this community, children are not increasing their height/weight proportional to their age when we compare them with children growing in urban areas and this could be from poor nutrition, care and sanitation
- The community considers stunting and wasting as a natural phenomenon while they understand differently feed oxen grow differently.
- The kebelle is malaria endemic
- In this community households have animals, but instead of using animal source foods they prefer to count and sell as an income generation
- Due to drought and water shortage, there is food insecurity and as a result there is no food diversification

**Section 2: Barriers to access and utilization of nutrition services**

**2.1. What kinds of nutrition interventions are in place to improve health of the pregnant in this woreda? Where do they get it? Who provide it?**

**Participant #1:** regarding nutrition interventions to improve health of the women, there are supports from government and non-governmental organizations. For children they give Corn Soya Blend (FAFA) whereas for pregnant and lactating women they give counseling why and what to consume. Women are also applying what they have been thought.

**Participant #3:** the question is clear, and it is also partly answered by participant #1. Women get special through their WDA network. For example, there are nutrition screenings and HEWs take MUAC measurement to see whether the child or mother has shortage of food. If they are below the normal, they will be given Corn Soya Blend (FAFA) and they also teach us this can be prepared at home from maize, teff and barley.

Currently if you ask any woman she will tell you how to prepare enriched food by blending the different types of home available cereals. They also get advice how to care a child and the pregnant to take extra and diversified meal. After screening, if the mother or her child is found malnourished, s/he will be sent up to Samre health center and they will get special treatment.

**Interviewer:** what about for the pregnant and lactating mothers?

**Participant #3:** similarly, the mother will also take supplementations.

**Participant #4:** it is similar, HEWs and WDAs teach pregnant and lactating women to go to health facilities for ANC and PNC and to deliver at health institutions. They also advise them to feed adequate food.

Regarding home gardening, we have been told that home gardening ensures healthy diet because we have no problem in access to land.

**Participant #3:** HEWs repeatedly tech about personal and environmental hygiene because there was AWD in our community. The source of AWD is poor hygiene for example, drinking unclean water and environmental hygiene. We are thought to plant vegetables and fruits in our yard. Mr. Tesfay Bula is best example for us. He has garden and he produces red paper, and salad. There are posters in health posts that show what to eat for example, there is poster that teach iodine is important for brain development and health of children and when to add iodized salt when we prepare food. This information is told several times, but our community uses unionized salt coming from Berahle.

**Interviewer:** what is the reason for not using iodized salt?

**Participant:** though there are readily available iodized salts our community prefer to use Berahle salt because of difference in test and lack of understanding its importance. For example, I use iodized salt because I understand it uses. Iodized salt costs 8 birr per kilogram.

**Interviewer:** while we were discussing the first section, participant #4 told me that malaria is common in this community. What do you do to prevent malaria?

**Participant #1:** to prevent malaria, we drain stagnant waters.

**Participant #2:**  as it has been said by participant #1, on top of draining stagnant water we by clearing bushy areas and using ITN we prevent malaria.

**Participant #3:** yes, malaria is prevalent in our community. We use ITN, and government sprays insecticide two times on the month of June and September. But there are few villages that are not sprayed this year.

**Interviewer:** Which villages are not sprayed? And what was the reason?

**Participant #3:** Adi-akmada, Gurare, Adeshinhunko, and Hawramba, villages are the most prevalent areas. Everybody can tell you and the health office knows well. Their reason for not spraying those prevalent areas was according to their study they are less affected by malaria.

**Interviewer:** Which of the interventions listed above do you think is most important for Women/Girls?

**Participant #3:** to effectively utilize our time, all are equally important. Interventions like vaccination, institutional delivery and ITN utilization are effectively implemented in our community. Whereas, nutrition in pregnant, lactating and children is one of the less effective in this community. There is no diversification of foods because the community considers injera with sauce (ወጥ) is enough. The reason is due to lack of awareness. Though the community is educated by the HEWs and Agricultural Extension Workers, home gardening is less effectively implemented.

**Summary**

- HEWs provide services like health education and advises on, Hygiene & Sanitation, Family
  Planning, institutional delivery, ANC, PNC, vaccination, ITN and iodized salt utilization, to take extra and diversified meal for pregnant, lactating women and nutritional screening for under five children, pregnant, lactating and adolescent girls. But still there are limitations on implementation.
- Adi-akmada, Gurare, Adeshinhunko, and Hawramba, villages are the most malaria prevalent areas

**Section 3:** **Perceived needs of women for relevant services during pregnancy**

**3.1. What do you think is necessary for the WOMEN in your community? Ask them to customize it to the WOMEN in their community and themselves? For example, Basic facilities, Services Commodities and Others**

**Participant #4:** it would have been good if they take rest. But as it has been said minutes before they are wandering with the animals and as the same time they equally engage in the agricultural activities. That is why they are early losing their strength.

**Interviewer:** what additional things do you think is necessary for the WOMEN in your community?

**Participant #3:** it is up to us to exempt our wives from different activities, government can’t do that rather government can only educate the effect of doing extra work and importance of taking rest for pregnant or lactating women. But if food shortage occurs in a certain community, government is responsible to avail food support. During screening children and women with acute malnutrition are identified but due to supply problem most of them are not provided supplementary rations of Corn Soya Blend and oil. So, it is good to strengthen the nutritional screening by availing the necessary supplies.

**Participant #6:** our life is here and there, and it is difficult to give rest for our wives. Giving rest could be possible in urban, but in rural it is very difficult because of our way of life.

**Interviewer:** how do you see the role of husbands in improving maternal nutrition? For example, helping to take rest, extra meal, to follow ANC and PNC…

**Participant #2:** anyways the limitation is ours. As a household head, the husband is responsible to make his wife to avoid heavy work load and wandering with animals and to advise her to keep her personal hygiene, but it is rarely practiced in rural areas. We don’t realize their work load while they are busy to prepare food for the family. The reason is due to access and life style.

**Interviewer:** so participant #2 is saying husband involvement is limited. Do you have additional or contradicting idea on this regard?

**Participant #7:** mine is addition to what participant #2 has already said. Except do this and that we don’t have role in improving their nutrition. This is not yet solved. We don’t recognize the extra activities they have at home.

**Participant #3:** they have explained the main thing. For example, if a mother dies you can say that the household has died. It is not exaggeration to say a mother is the pillar of the household. For example, if you compare children who lost their father with those who lost their mother, children who lost their mother will suffer more. But we did not give credit to her role. Our community associates the death of a mother to the death of the household. So, the husband has a greater role in improving maternal nutrition. If a mother is malnourished, she will face difficulty during delivery, so we should work more on this regard because everything is at our hand. Our pregnant mothers are still involved in the heavy work while we can cover it by recruiting daily laborers selling one goat. This is lack of awareness.

**Participant #4:** we are told everything. If you are poor, you can’t do anything, and our community says, ‘a mother who has nothing is like a stone’ (ዘይብላያ እኖኻስ ዳርጋ እምኒ) but everything is at our hand. Our forefathers’ handover us farming only and we force our wives to work equally with us. Government can’t do beyond educating it is our responsibility.

**3.2. Do WOMEN in this community typically** **change their diets when they are pregnant? How is this diet different from when they are not pregnant?**

**Participant #2:** at the time of delivery, even those who don’t have will get milk and honey from the community for a week and more. But we don’t deliberately do this though we know the importance.

**interviewer:** what about during pregnancy?

**Participant #2:** there are few who change diet during pregnancy, but they are not that much. Though there is recurrent drought in the area, as farmers we are trying to diversify our production and livelihoods for improved food access and dietary diversification. There is a trial to blend the cereals at hand for porridge and soup preparation.

**Interviewer:** What foods are recommended for Women/Girls? What foods do Women/Girls avoid?

**Participant #1:** traditionally pregnant women is prohibited from drinking alcohol in our community. Nevertheless, there is no food avoided for Women/Girls. Rather they are recommended to eat diversified foods of animal (egg, milk) and plant sources if it is accessible.

**Participant #4:** as participant #1 has clearly stated alcohol is prohibited because leave alone for the fetus it is harmful for her. In addition, traditionally our community believes that, if a pregnant eat chickpea and roasted grain it will cause cramp both for the mother and the fetus. But I am not sure whether this is persisting or not.

**Participant #3:** what participant #3 is saying is not existing these times. Yes, in the past the community was perceiving that way, but this does not exist in the ground. But traditionally our community prohibit a mother to breastfeed while taking malaria medication for example, chloroquine. But we don’t have food that a women/girl should avoid.

**Interviewer:** Are there gender disparities in women’s diets before pregnancy and during pregnancy?

**Participant #3:** to make it short, we don’t know the gender of the fetus while she is pregnant. But traditionally the community predicts the sex of the fetus by looking at the shape of the pregnancy. For example, if the pregnancy shape is concentrated to the buttock the sex of the fetus is female, whereas if it is concentrated to the chest the sex of the fetus will be male. Overall there is no gender disparities in women’s diets before pregnancy and during pregnancy. But since female was oppressed in the previous regimes our community prefer male than female.

**Summary**

- During screening children and women with acute malnutrition are identified but due to supply problem most of them are not provided supplementary rations of Corn Soya Blend and oil.
- Husband involvement is limited in improving nutrition of pregnant, lactating and adolescents
- Though they know the importance of taking rest, extra and diversified meal, due to workload and food insecurity it is difficult to implement it
- Pregnant women don’t change their diets and they don’t have food taboos and gender disparities in women’s diets before and during pregnancy

**Section 4: Other interventions that improve pregnant, lactating and adolescent nutrition**

**4.1. Have you ever gone for nutrition screening? Where? Who provide you?**

**Participant #1:** sometimes Woreda Health Office deploy additional work force if not HEWs provides nutrition screening by going from village to village to provide the services. They give the services at convenient sites.

**Participant #3:** Every 3 months, the HEWs mobilize the community to health post to give de-worming to children under 5 as well as screening of children and PLW. Then malnourished children and pregnant and lactating women will be referred to the TSF to rehabilitate them.

**Participant #5:** it is the same.

**Participant #8:** yes, during outreach activity and household visits HEWs deliver vitamin
A supplementation, and screening to children and women present to the health post.

**Interviewer:** What are the challenges to attending community health days or accessing the routine service delivery?

**Participant #3:** for example, all eligible might not come during the community health day because work load and lack of information. The other challenge is everybody needs nutritional support regardless of their nutritional status. Instead of being happy for his/her child is not malnourished he/she looks at the support given to the malnourished once. Because of that mothers may not come to screening.

**Interviewer:** do pregnant/lactating women and adolescents know why they are targets for targeted supplementary foods and if it helps them increase their food intake. If not, why not?

**Participant #2:** yes, they know why they are targets. Targeted supplementary feeding is given for vulnerable groups such as children and pregnant and lactating women to mitigate the potentially negative effects of malnutrition

**4.2. Are WOMEN beneficiaries of the soft conditionality of the productive safety net program, PSNP? How?**

**Participant #3:** yes, they are beneficiaries of the soft conditionality of the productive safety net program. Around 100 mothers are integrated with HUB project of REST and they are subsidized around 4000 birrs per individuals. Regarding the question do pregnant women get safety net for free, safety net has its own criteria. It is aimed at enabling the poor facing chronic food security. To get safety net support able-bodied individuals must participant in productive activities such as soil and water conservation and those are identified in collaboration with social justice and agriculture offices. Unless otherwise, except the health supports being pregnant is not a criterion to be included in safety net support. She must fulfill the safety net criteria to get free support.

**Participant #4:** it is already said, only chronically food insecure household is included in safety net. Free support is given only for those who are identified as sick, old. But there is no criteria for pregnant and lactating women.

**Summary**

- Every 3 months, the HEWs mobilize the community to health post to give de-worming to children under 5 as well as screening of children and PLW. Then malnourished children and pregnant and lactating women will be referred to the TSF to rehabilitate them.
- During outreach activity and household visits HEWs deliver vitamin
  A supplementation, and screening to children and women present to the health post.
- Expectation of nutritional support regardless of nutritional status, workload and lack of information are challenges to attending community health days and accessing the routine service delivery
- Since targeted supplementary food is aimed at enabling the poor facing chronic food security being pregnant is not an eligibly criteria to get safety net unless she is identified as sick, old and poor.

**Section 5: Understanding perceptions of age at first birth and birth spacing**

**5.1. Do you think** **delaying the age at first birth to after 18 is better for the health of the women? How? What other benefits does it have for the women? What about for the baby?**

**Participant #4:** if she is married at her early age she will face problems. For example, her pelvic will not be fully grown and it will lead to miscarriage and birth complications.

**Participant #3:** the current policy supports a girl to legally marry at her 18 years and beyond to minimize birth complications. This is to wait until the body of the girl fully matures and develops. If she marries at less than 18 years old she will face fistula, subjected to deliver by operation, prone to anemia and she will be at high risk of debilitating illness and even death. But if she marries at her right age she will safely deliver.

**Interviewer:** Do you think this message is being promoted in the community? Who are working on it? How do they promote?

**Participant #2:** Through media campaigns and educational outreaches consequences of early marriage is provided. In addition, there are marriage committees who confirm the age of the to be married girl and allow marriage. If either of the bride or groom is below 18 years, they will be punished. It is very strict, and it is acceptable by the community.

**Intervention:** In your opinion, how could this message be better promoted? Who should be involved?

**Participant #8:** to stop early marriage policies must engage local and religious leaders to educate communities, and empower girls through education.

**Participant #1:**  it would have been better if messages are promoted by religious leaders and they are also promoting it.

5.2. **How many years do you think the gap should be between successive births for women? Why? What about if shorter than it?**

**Participant #4:** at least at 4 years. If there is short birth interval the child will not grow properly, and the mother will not recover from her previous pregnancy related problems. With the workload, below 3 years is not enough. but in our community mothers commonly give birth at 2 years and below. That is why children are short and thin compared to their age equivalent.

**Participant #8:** though there is improvement, short birth interval is common in our community. if possible better to increase birth intervals even beyond 4 years. But from the mothers, side, if they increase birth interval by 5 and 6 years there is fear of infertility. Husbands recommend increase birth intervals, but mothers don’t accept it.

**Participant #7:** these days it is the mother who refuses to increase birth intervals, unless otherwise husbands are interested to increase the interval. To keep health of the mother and baby it is good to space 5 years and beyond between births but there is refusal from the mothers.

**Participant #3:**  HEWs are giving advises and avail contraceptives. There is change in spacing between births in our community. mothers who were giving every 2-3 years are increasing the interval to 4 years. Birth spacing is important because it reduces maternal and child mortalities. Except few most use family planning. We get information on birth spacing from HEWs and health workers in health centers.

**Summary**

- Delaying the age at first birth to after 18 and spacing pregnancies can improve maternal nutrition and hence both maternal and infant health by minimizing poor health outcome

**Section 6: Understanding communication and information sources**

**6.1. Is there an opportunity in the community to discuss Nutrition for women? What are the opportunities? Probe: for community conversation, community mobilizations?**

**Participant #2:** we are given health education on nutrition and WASH by HEWs and WDAs. in addition, we get information during community mobilizations. The problem is we did not maintain the information. It is not from lack of education.

**Interviewer:** Which source of information about nutrition is essential for you?

**Participant #3:**  since it is for change, government should maintain deploy trained health professionals, HEWs and WDAs and must monitor and evaluate. Woreda and regional health offices must be monitored and evaluated weather the services are given by the manuals developed or not, weather professionals are serving in a right way or not. But the main thing is expansion of health facilities to every kebelle and village.

**Additional remarks**

**How can we improve nutrition during pregnancy? During Lactation? What about during Adolescence? What do you think specifically needs to be done to improve the nutritional status of the adolescents in your community?**

**Participant #2:** there is a kebelle named Zamra, which have no any road access. So, ambulances cannot reach to this area. Thus, laboring mothers come by traditional ambulance (stretcher). There are case who delivered while going to Samre health center by stretcher. We have presented this problem 2-3 times but is not solved yet. To alleviate this problem government must construct health center around.

**Participant #3:** we have water sources, but most are salty. So, it needs to discuss with NGOs to dig new water sources. It would have been good also to add chlorine to the well waters. As you have seen there is road problem, so to make it easy to ambulances it needs maintenance.

**Thank you**

Thank you for taking the time to discuss these issues with us today. We have learned a lot from you. If you have any concerns or questions, please feel free to contact us (contact info). Thank you very much for your time.
