# Supplementary material for: Barriers to utilize nutrition interventions among lactating women in rural communities of Tigray, northern Ethiopia: An exploratory study
Source: PLoS One. 2021 Apr 30;16(4):e0250696. doi: 10.1371/journal.pone.0250696 (PMC8087028; doi:10.1371/journal.pone.0250696)
Supplement: S2 File — (ZIP) [file pone.0250696.s002.zip › S2_File.Doc/Community level Key informants/063_WDA_Lemlem Kebele_Samre woreda.docx]

**Operational Research on Adolescent and Maternal Nutrition in Northern Ethiopia**

**Introduction**

Hello my name is kiros, I am from Mekelle Universty; we are conducting a research on the factors that influences the nutrition of mothers and adolescent girls in collaboration with the regional health bureau and UNICEF. Year participation is very valuable; the information that you tell us will be used to improve nutrition programs and services for women and adolescents in the region and the country. We will not share your names when we report our results. The interview may take 1-2 hours and I would like to thank you for taking the time to speak with us today. You have the right to withdraw at any time and I will use tape recorder. Are you voluntary to participate for the interview?

**Yes** No

| **Section A: Interview details**   1. Zone: **South Eastern Zone of Tigray** 2. Woreda: **Seharty Samre** 3. Kebele: **Lemelem/ Samre** 4. Name of key informant: **Miss Abriha Asefa** 5. Institution of key informant: **Women development Armey** 6. Interviewer name: **Kiros Tedla** 7. Date of interview: **06/11/2017** 8. Interview start time: **10:00AM** 9. Interview end time: **11:21AM** |
| --- |

| **Section B: Interviewee professional information**   1. Sex    1. **Female**    2. Male 2. Highest level of completed education.    1. No formal education    2. **Primary education**    3. High school    4. College education    5. Bachelor degree    6. Master’s degree    7. PhD 3. Discipline or field of educational training    1. Agriculture    2. Health (MD, nurse, health officer, midwife, pharmacy, etc)    3. Nutrition    4. Public health    5. Food science    6. Other (specify):**Women Developmental Army** 4. Current position: **WDA** 5. How long have you been in the current job/position:    1. ______ Months    2. **7** Years |
| --- |

**I:** Interviewer **P:** Participant

**Section I**

**I**, **what do women do to stay healthy in this community or worerda?**

**P**. pregnant mothers keep their health as they will follow their health in the health post starting from 4 months of pregnancy. They will eat food better than they eat before pregnancy like they eat four times if they were eating three times before. They also keep their hygiene by washing their clothes and their children. They also give birth in the health services and avoid bleeding as they are treated or take medication given in the health facility. They want to the health facilities by ambulance.

**I, What about lactating mothers do to stay healthy?**

**P.** they are vaccinated for themselves and their children. They are advised by the health expert or HEW to wash their hands and their breast before lactating their child. They prepare different foods to their children like porridge and soup consisting of egg, maize, lentils, thef and been.they also prepare and eat these different food.

**I, Why they eat different foods?**

**P.** because they are lactating as they need food to produce enough amount of milk for their children. They also need to feed their children different sorts of foods in order to have normal health and nutrition; they will also become critical thinkers and improve their cognitive ability and school performance. The mothers will also have good health and follow birth interval or spacing meaning utilize family planning.

**I, What is family planning?**

**P.** The mothers use different contraceptives to have delayed birth or space among the children. The contraceptives used are there is for 1 month, 3 month, six month, three year and even for 10 years. The government had worked here very well.

**Ok,** **I, What about adolescent girls do to stay healthy?**

**P.** they are given two times a year a medication in injection at school. This will help the adolescents to be healthy and cleaver in their education. They also keep their hygiene by washing their clothes and eat different types of food. Hence; they become good in school performance.

**I, what are the common nutrition problems in the community for pregnant, lactating and adolescent girls?**

**P.** pregnant mothers who are suffering from malnutrition, anemia, hypertension and their body swell up mainly around their arm and face because of poor nutrition. There are also mothers who are even died because of shortage of food or poor nutrition. But if they would have eaten food and care their child they would not have dead. There is also change among the mother’s interns of malnutrition because of the work done by the government as we do have health post around us even though it is not enough to all the “kushets” in our kebelle. Hence there is improvement on the mothers and their children.

**I, What is the health post doing to improve maternal nutrition?**

**P.** They give the pregnant different treatments like they give medication which improves their blood level; they also give them Plumplet for the child. The mothers (pregnant and lactating) and their child were also given Fafa to make porridge and soup; and they improve their nutritional status.

**I, How do they give the plumplet and fafa; are they giving to all mothers and children?**

**P.** They give to all pregnant mothers are affected by malnutrition and who could not feed well. The lactating mothers and their children who are severely affected by malnutrition were included in the aid. But now it is not given; it is stopped.

**I, Why it is stopped; is it because the diseases is stopped meaning no mother and child is affected malnutrition?**

**P.** No there are many mothers and children affected by malnutrition and who need the help. This month I have not seen any mother received the aid as we WDAs were told to send them to the health post but this month we did not send as I have six pregnant mothers.

**I, Why are pregnant and lactating mothers given the aid?**

**P.** pregnant mothers are given the help because they have infant inside who needs food to have healthy life and normal nutrition. The lactating mothers and their children are given the help by the government in order to improve their nutrition and their health as she is advised to give to their children in the form of porridge.

**I, How do you express the extent of malnutrition on mothers?**

**P.** There are mothers who are severely affected by malnutrition and send to the woreda health facility. These mothers are then came back with normal health because they were given treatment which improves their nutrition like different foods, injection, and keep them clean. There are also mothers who have moderate malnutrition and such mothers are given fafa in the health post after measuring their nutritional status.

**I, What about micronutrient deficiency like anemia, night blindness and goiter?**

**P.** there are mothers of both pregnant and lactating which are affected by anemia due to poor feeding or nutrition. Pregnant mothers are the main victims as they are living in the village where the mothers had got poor care due to different reasons like they cannot get all the necessary foods like vegetable as there is lack of vegetable here. But if they went to the health facility; they are given treatment to bring back their blood level or anemia in to normal.

**I, Do you think it is related with nutrition?**

**P.** Yes, it resulted from poor nutrition; because they did not eat different foods as recommended. But if they eat three or four times a day with the composition of the above mentioned sort of foods to themselves and their children; they would have normal health and nutrition to both the mother and the child or fetus.

**I, What about night blindness and goiter?**

**P.** there is night blindness but such mothers are given treatment from the health post and cured.

**I, do you think the women or adolescent girls have height proportional to their age? Why?**

**P.** No, they are very thin and short.

**I, Is it related with nutrition?**

**P. yes,** This is because of poor nutrition as they did not get enough nutrition but if for example someone had got good nutrition he/she will have very good nutrition status and school performance. But if they are starved; they become short and thin. There are children of poor family who are thin and short; there are also children who had very good body with normal height to their age at our kebele. This difference is mainly because of nutritional difference as the former one is from poor family where he/she cannot get the enough nutrition as they do not have the resource but the latter one is from rich family and can get the nutrition.

**I, do you think the women or adolescent girls have weight proportional to their age? Why?**

**P.** it depends on their health and nutrition of the mothers. For example if the mothers are healthy and eat very well; their weight will increase but if not it will be decreased.

**I, how do you recommend the mothers to eat to stay healthy with normal weight to their age?**

**P.** if they eat diversified foods for example if I eat egg in the morning; eat meat at lunch time and eat vegetable with coffee at night; I will improve my weight or nutritional status. So difference is in getting the balanced diet.

**I, do think the community have sustainable food for one year meaning can they feed their family without shortage for one year?**

**P.** there is no family who can sustainably feed their children currently because of the drought but before five years we were feeding our children as there were very good season.

**I, How frequent is this happening and why?**

**P.** we were very good after the 1977 drought or starting from 1979 up to 2004 and 2005 but starting from 2007 we are suffering from food insecurity as there were consecutive droughts as a result of drought due to shortage of raining. And now in 2010 EC as you can see there is no family who able to secure food and feed their family; we are also afraid of our animals as there is shortage of food and water to them.

**I, What do you think is the reason for the shortage of raining?**

**P.** I do not know exactly but I believe that it could be from GOD.

**I, Who is the most affected by the food insecurity?**

**P.** all are affected most notably mothers and adolescents. There are adolescents who had stopped their college education because of the drought as we cannot able to pay their education fee. There are also students who failed from grade 9 and 10 and becoming daily laborers like working on washing of clothes; because of the family cannot afford to teach their children due to the drought or shortage of food as they cannot cover the payment.

**I, So what should be done to solve the problem?**

**P.** the government should help us; as the society is the base to the government; the government should help and provide food aid to the society.

**How and what type of aid?**

**P.** the government can help the students and the farmers as we can also help by our capacity. The aid could be in the form education, food aid or health. Health like we are far from the health center so we need to have health center here in our kebelle.

**Section two**:

**I,** **what kind of nutrition interventions are in place to improve health of pregnant women, lactating women and adolecents?**

**P.** as I have told you previously; Plamplet and Fafa with oil were given to severe and moderate malnutrition mothers and their children before but now it is stopped.

**I, Who provide this? From where do they get it?**

**P.** The HEW provides this and the mothers get it here or health post.

**I, How do they give the interventions; are they giving to all mothers and children?**

**P.** no they only give to pregnant and lactating mothers and their children with malnutrition after measuring on their arm. In 2009 they were giving to all pregnant mothers as I have seen myself but in 2010 it is stopped.

**I, are pregnant mothers advised to visit HS for check up and services?**

**P.** yes, they are advised by HEWs very well. Pregnant mothers are advised to visit health facility, to keep their hygiene and eat three or four times a day. They also told them that they will give birth in the woreda and to feed their children very well after birth.

**I, Do you think it helps them? How?**

**P.** yes, because if they follow their pregnancy they will save their life and the life of their child by giving birth at institution. Hence, they will have good health for their child and themselves. The child will also be nutritionally normal as the mother had delivered as she is told how to care her child like to feed exclusively feed breast until six months and after six months to feed her child porridge and soup.

**I, What about on getting extra-meal and rest for pregnant women and lactating mothers?**

**P.** They get an advice as I have told you earlier. They are advised to eat three times if they were eating two times a day before during pregnancy. Lactating mothers are also told to eat very well to herself and child in order to have normal growth and nutrition to her baby.

**I, What about on food diversification meaning what type of food are advised the mothers to eat?**

**P.** they are advised; as an example for children they are advised to feed them a soft porridge having different compositions like a combination of been, lenticels, maize, and other available crops. They are also advised to add egg and vegetables but we do have scarcity of vegetables in our kebele as there is no water to do so. The mothers are also told to eat food four times a day consisting of meat, butter and others in order to healthy and normal nutrition.

**I, How are pregnant mothers advised to eat?**

**P.** they are advised to eat different foods like four times a day if they get; they should eat egg at the morning; meat at lunch time and vegetables at dinner. In order to have normal health and nutrition of themselves and their children.

**I, What about on iodine salt utilization?**

**P.** yes there is counseling and most of the people are using iodine salt as it protects us from goiter. And we are advised to add the salt after we finish cooking in order to avoid bitter test.

**I, on getting advice on nutrition sensitive agriculture such as home gardening?**

**P.** Yes we are getting the advice and we were producing vegetables in 2005 and 2006 but now we are not producing because of shortage of water due to drought. Even now I are trying to cultivate paupers or chelie but not effective as there is shortage of water and our animals also feed on it.

**What about on the need to participate on the safety net program?**

**P.** yes there is safety net program but not effective as it gives only for five family members even though the family size is very large like for example 12 family members meaning the remaining seven family members will not be given the aid. Both mothers are participated based on their family status meaning if they are poor they are included but if they have two oxen and above they will not be included. Pregnant mothers with pregnancy of six months and above are not involved in the work done. Lactating mothers are also allowed to rest for ten months after delivery. There are also adolescents, elders and those who have no family are also included in safety net. They are given 15 kilo per four or five months.

**Who provided the safety net program? Is there any other help?**

**P.** this is provided from the government and there is also the so called Digoma to those family members who are better than those who are very poor but had food security problem like those who have two or three cows. They are given 15 kilo of wheat, been and oil per month but not always.

**I, Is there any advice given to pregnant, lactating, and adolescent on water, sanitation and hygiene services?**

**P.** Yes, there is advice on personal and environmental hygiene and on how to keep water clean by the HEW. But the problem is we do have shortage of water as we do have only one functioning pump from six pumps; hence the people are suffering from the shortage of water.

**I, Where do get the advice**?

**P.** In the health post, holydays like Sunday in the church by HEW; we or WDA also give such education during our meeting with our neighbors in monthly ceremony like Tsebel. The topic of our discussion includes on how to defecate our stool that we have to use toilet, how to keep our environment and water clean.

**I, Is it helpful to discuss on hygiene? How?**

**P.** yes, for example if we defecate in toilet we will not be contaminated by house flies. Hence, our feeding utensils, hand, the enjera which we eat will be free from contamination by houseflies.

**I, Is malaria common here? If yes, are pregnant mothers getting advice on the need to use ITN? Why? Who Advice them?**

**P.** yes there is malaria. Last year there was no residual spring but this year there was residual spring. But there is still high prevalence of malaria in Adi-akmada, Adi-tsagina and Adi-sherba. The HEW is very busy now because high prevalence of malaria as many people are coming to the health post for treatment. Pregnant and lactating mothers having with infant or child of less one year old are given ITN first and then to the other farmers but this was not enough as there are many people who did not get the ITN. Hence; we have reported to the woreda but still no response.

**I, Why it is given first to Pregnant and lactating mothers and who provide this?**

**P.** ITN is first given to children in order to be healthy and not be bitten by mosquito. The pregnant mothers are given ITN first in order to give a healthy child. The health extension worker gives the education and the ITN.

**I, On lactating and adolescent girls about ITN?**

**P.** Lactating mothers are advised and given the ITN because of the child they have as he/she is very small. But there is no special treatment given for adolescent girls; they are equally treated with their other family members.

**Is there any treatment given to prevent intestinal parasites on pregnant, lactating mothers and adolescents?**

**P.** yes there is treatment or tablet (Shigut Knina) given to pregnant women and children with age of <10 years old to prevent intestinal parasites. We are also given deworming four times a year freely by the government. The treatment is given by HEWs to all pregnant and children at the health post and at each kushet if it is given to the community.

**I, Is there school feeding program for adolescent girls?**

**P.** there is no school feeding program but in 2008 EC there was school feeding program initiated by one teacher and students were given porridge made of fafa for those students below grade six.

**I, Which of the above mentioned interventions is the most important for pregnant women? Why?**

**P.** the follow up and check up of pregnant and lactating mothers; Plamplet and fafa are very important to both mothers and children. Because mothers are witnessing that these aids are very important as their children had shown changes on nutritional status compared to their nutrttiona status before the aid. Compared to milk; plamplet and fafa had produced very visible changes on the child nutrition but the problem is they are not given now.

**I, Is there any challenge which affects mother’s utilization of the interventions or given services?**

**P.** there is no any problem as currently every one is utilizing the service but in the past meaning before 2006 EC there were husband related barriers like the husband were not volunteer to use his wife family planning due to low awareness but now they are willing to utilize the service given. Hence, there is no problem in the community related with low level of awareness starting from 2006 as our community is utilizing the service as everybody is concerned on giving to his/her children by providing all the necessary requirements.

**Section 3**

**I, What are the special things should women do to stay healthy in the community? ( during pregnancy, lactation and adolescence)**

**P.** pregnant mothers should follow their health in the health facility and get advice from HEW on how to get extra meal and rest. For example; pregnant mother should eat egg, meat and vegitable and also feed their children by feeding different combination of porridge which contains vegetable, and cereals like been, maize and others. Hence the child will become nutritionally normal and will have excellent school performance. The lactating mothers should have enough rest and should not bring their children to the work place as he/ she will be bitten by mosquito or may be exposed to other injuries. They should also keep their hygiene. Currently; mothers are getting more rest compared to the previous once as for example they bring water using donkey from pipe water but before we were brought water by ourselves from very long distance and at night fighting with hyena.

**I, Do husband have a role on improving maternal nutrition? How?**

**P.** yes, because they bring their wives to the health facility for check up and counseling. They also give them rest and compared to the past now a day’s husband had improved role on caring their wives as they are also going with their wife during delivery to samre.

**I, Do women in this community change their diets when they are pregnant and lactating? How?**

**P.** yes, they do change their diet. Before pregnancy they do care and have no rest but after pregnancy they change their feeding style and they are actually doing in our community as everyone including the husband believes having very good nutrition is important for both the mother health outcome and child nutrition.

**I, What is the recommended food for pregnant and lactating mothers to eat?**

**P.** They should eat soups, meat and egg alternatively meaning at different times but if they are not pregnant they are not cared as the pregnant.

**I, What about the actual foods recommended for pregnant and lactating mothers to eat by the community?**

**P.** it depends on availability of the foods or capacity of the individuals; they eat egg, soup, maize, “teff”, and sorghum.

**I, Is there any food which should not be taken by the mothers ( pregnant and lactating mothers)?**

**P.** I do not know.

**I, What affects women diet during pregnancy and lactating?**

**P.** there is no as they can get treatment from the health facility if they have lost their appetite.

**I, What about gender disparities in women’s diet before pregnancy, and during pregnancy, lactation and adolescence?**

**P.** previously there were gender disparities as women do not eat foods like cooked chicken “Tsebhi Derho ” if the husband is not around at even if she is pregnant or lactating. But now this is changed as she can eat her part and left her husband part.

**Section 4**

**I, Have you ever gone for nutritional screening during routine service delivery? If you want tell me about your experience?**

**P.** no, as I have stopped giving birth long time before in 1996 as my last daughter is with age of 14 years old. Even though I used to give birth in the health facility there was no such nutritional screening services.

**I, Is there community health days? Do you think they are important? what about to accessing routine services?**

**P.** yes, previously all people were invited including young men were given a mass treatment but in 2009 there were no such but we are waiting that they will call.

**I, Are the people also screened for nutrition?**

**P.** Yes, mothers and young adolescents were also screened for their nutritional status.

**I, Do you think it is important?**

**P.** Yes, because the individuals who were screened and have night blindness were given treatment for their eye.

**I, Is there high prevalence of night blindness on mothers?**

**P.** The highest prevalence is among old people but among pregnant mothers I have not studied it but I assume it is low.

**I, How do you see the routine service given here?**

**P.** It is very good but the number of HEW is very low as we do have only two and now the one is absent because of disease. Hence; only one HEW is working for such high number of people with a disease and because of the shortage of HEW there are people who did not got the service but diseased as now the burden diseases is increasing. There are people complaining some times when they did not get here as she is busy and only one to give the services.

**I, What are the challenges related with attending community health days and routine service delivery?**

**P.** there is a community health day like during holy days meaning Sunday as a number of people are attending the church ceremony and the HEW give education here. There is no any problem related with the community related to health as they take the message to their home and practice.

**I, Is there any challenge related to the health service provider which affects the use of routine health service by pregnant and lactating mothers?**

**P.** There is no any problem related with the health provider but we do have shortage of human power in the health post; we need to upgrade the health post to health center as there are many people with diseases including pregnant mothers who need advanced services and we are far from the woreda health facility where the services are provided.

**I, Do you think mothers should be targets for food supplementary? Why?**

**P.** Yes they should be given as I have told you before there are mothers with the problem like for example there is one woman here who given birth now and her husband were died. As she is poor she should be given help for her child and for herself as well.

**I, are pregnant and lactating women beneficiaries from of soft conditionality of the safety net program?**

**P.** yes they are beneficiaries as pregnant mothers are excluded from the activities of the safety net program starting from four months of pregnancy and; after delivery they are free from the activities done for 10 months of the lactation time.

**Section 5**

**I, Do you think that delaying the age at first birth to after 18 is better for both the mother and the infant? Is it promoted in the community? Can you tell me who is promoting?**

**P.** Yes, if she gives birth at 15 or 16 years old she might be suffering from different diseases like Fistula but if she had given birth at 22 or 23 years old; both the mother and the child will be healthy. This is because the mother had become very strong physically so she can give birth without any problem for herself and the infant also will be healthy as she can feed him very well including the milk from her as she is mature. But if she had give birth at 13 years old the mother and the child will suffer from different disease and affected by malnutrition.

**I, Do you mean they are working very well and there is no early marriage in this community?**

**P.**  yes, there is no early marriage now in our community at age of 15 or 16 but only for above 18 meaning at 21 or 23 years old. For example; in 2006 there were individuals who had prepared to marry a 16 years old girl but we stopped her and they have done this after two years later in 2008 EC.

**I, Who are working to promote delayed marriage?**

**P.** the kebele, health facility and school are working on early marriage. These organizations give education early marriage and certificate of the age if she is above 18 years old. Currently everyone is aware and they bring their daughter to kebele and send for medical test to mekelle.

**I, How was the reaction of the community to programs? Any think which should be improved?**

**P.** The community had received it very well as everyone need to have healthy daughter and child. But for improvement I have no idea.

**I, Do you know about spacing of birth intervals or family planning? How much should be the gap between successive births? Is it promoted in the community?**

**P.** five year as the child is grown very well and can go to school so the mother can get pregnancy. This is because the mother and the child will be healthy as the mother is capable of managing her home and the baby is healthy. But if a mother give birth after one year of first birth both the mother and child will be unhealthy and nutritionally poor.

**I, Can you tell me who is promoting and you heard for the last time?**

**P.** The health extension workers give the education on family planning as there are different types of contraceptives like for one year, 3 months, 3 years and for 10 years. Hence, based on our preference we go and utilize the service or injection based on our preference. I heard all the time the HEW give education at the church and other community or maternal gatherings.

**I:** Who are involving on it?

**P:** We (WDA) and kebelle leaders are also involved in family planning and teach the community about health service utilization, on birth spacing and for men they are advised to allow their wives to use contraceptives. For example, we advice the husband of those who are not utilizing family planning to use the service and give birth at the right time.

**What better way do you think promoting better way? Is there community not addressed by the promotion?**

**P.** I have nothing as it is working very well except that the HEW is working alone we need additional HEW and the health center should be built here as the people is suffering from malaria. There is no also community member not addressed by the promotion.

**I, what kind of community conversations or massages discuss women’s and adolescents nutrition?**

**P.** yes there are discussions as I have told you earlier during Sunday at church by the HEW and the community. We discuss on how to feed our children that we have to give our children different foods in order to grow healthy and good in education. They also share their ideas and advice the community to make the education practical.

**I, What are the common sources of nutrition during pregnancy?**

**P.** The common nutritional sources for pregnant mothers in this area are sorghum, maize, barley or “Sgem” and “Adonger”.

**I, Do all women get the massage easily? What are the barriers for access to information for nutrition during pregnancy?**

**P.** Yes they get the massage easily. There is no any problem in getting the information about nutrition. The only problem is related with scarcity of resource.

**I: Which information is effective for you to change your practice in nutrition during pregnancy? Why?**

**P.** All are very important but the education given during Sunday is better as there are a number of individuals with both male and female available. But during the house to house education the mothers are only available but still very important.

**I, Which source of information about nutrition is essential for you?**

**P.** The information given to both pregnant and lactating mothers on ANC, food diversification, extra-meal are very important as they are the bases to have healthy mother and child in nutrition.

**Section 7**

**I, How can we improve maternal and adolescent nutrition in this community?**

**P.** at last I would like to say our people is suffering from shortage of food due to the consecutive drought; hence our government should provide us help or aid as the pregnant and lactating mothers and the elders are suffering from shortage of food. The health center should also be built here as I said before. As you are from University, we need class rooms to our children as they are learning in the open air without any shelter.

**Summery points**

**Section one:**

- Medium to severe malnutrition and Anemia are common among mothers and children.

**Section two**

- Free health services during ANC and delivery; Plamblet and FAFA for both pregnant and lactating mothers and their children are the interventions in this woreda.

**Section three**

- Pregnant mothers should visit HF for follow up and check up during pregnancy and should get etra-meal and rest during and after delivery.

**Section four**

- Nutrition screening is routinely performed as it is important to determine the nutritional status of the mothers and their children and to give them the nutritional interventions..

**Section five**

- The programs or policies targeting delayed marriage and birth interval are working very well.

**Section six**

- There is community based discussion on nutrition mainly during community celebrations like Sunday in the church.
- **Finally I have finished my questions and I would like to thank for your time, patience and answering all the questions. Thank you very much!!! Thank you!!!**
