# Supplementary material for: Barriers to utilize nutrition interventions among lactating women in rural communities of Tigray, northern Ethiopia: An exploratory study
Source: PLoS One. 2021 Apr 30;16(4):e0250696. doi: 10.1371/journal.pone.0250696 (PMC8087028; doi:10.1371/journal.pone.0250696)
Supplement: S2 File — (ZIP) [file pone.0250696.s002.zip › S2_File.Doc/Community level Key informants/066_KII_Kebele leader_Lemlem kebele_Samre woreda.docx]

**Operational Research on Adolescent and Maternal Nutrition in Northern Ethiopia**

***Date: Nov 11, 2017***

**In-depth interview with Kebelle leader of Kancha Ruba, Seharti Samre Woreda, Tigray.**

**Section A: Interview Details**

1. Zone: Southern Eastern zone
2. Woreda: Seharti Samre
3. Kebelle: Kancha Ruba
4. Name of key informant: Haleka Gebrehana
5. Institution of key informant: Kancha Ruab
6. Interviewer name: Mengistu Mitiku
7. Date of interview: Nov 6, 2017
8. Interview start time: 10:52 AM
9. Interview end time: 12:19 PM

**Section B: Interviewee professional information**

1. Gender: Male
2. Age: 52 years
3. Highest level of completed education: High school
4. Current Job position: Lemlem/Kancha Ruba Kebelle Leader
5. How long have you been in the current position: 4 years

**Main interview**

**I:** Interviewer

**P:** Participant [The key informant]

**I:** Good morning. I am Mengistu Mitiku from Mekelle University. We are doing research on maternal and adolescent nutrition in collaboration with UNICEF and TRHB. One thing I would like to say is that your opinions are very important. While you share me your thoughts and ideas, I will tape record our conversation as it is difficult to write all the things you speak. Moreover, our conversation may take 1 and half an hour to 2 hours. So, do you have questions before I proceed?

**P:** It is clear. I don’t have any question.

**I:** Okay. Please tell me anything that you would say is unclear.

**Section 1: Common maternal nutrition problems**

**I:** What are mothers in your community doing so as to be healthy is the first question? So, when we say mothers, we have pregnant women, lactating mothers and adolescent girls. Now, let us see them one by one. For pregnant women in this community to remain healthy or to be healthy, what do they do? This is the first question. Now, you can proceed.

**P:** Okay. When we see pregnant women in this community at this situation and as declared by the Ethiopian prime minister, ‘no mother should not die while giving life.’ This has been promoted through the health sector and other approaches. When a women becomes pregnant, what they should do is…

**I:** It is not what they should do; it is what they do.

**P:** Yes, it is about what they are doing. They keep their health in a good condition under the pressure they have in the current condition. It could be inside or outside their house. Anyway, what they do is they lead their life and their family members’ life either by any economic activity or water well preparation.

**I:** For example, what activities do they do? Let us say: their diet.

**P:** Regarding their diet, one they work for the sake of their energy and their babies’ energy building. They also undergo medical examination. If they undergo examination, they keep the health of their family and their own. Since they are the light of the family, they assume that keeping their health is equivalent to keeping the life of their family. They do have such initiatives

**I:** What else?

**P:** The other activity the do is maybe they do contribute to the family in the form of health education. They educate their family as it is their responsibility. The other thing is they use clean drinking water. If the water is not clean, they treat it with medicine. By doing so, they keep the health of their children as well. This is what women in our community do. In addition, if there is water they grow vegetables at their backyards and if not they buy from the market. This is another effort they have to keep themselves healthy.

**I:** What about lactating mothers, what do they do?

**P:** Fine. When mothers enter the lactation stage, gave birth and started breast milking their babies’, one they exert their effort to breast milk their children eight times a day so as to grow their child more healthy. When the age of the baby reaches 6 months, they start to use soup prepared from red teff and porridge.

**I:** What do they do for themselves, is the question?

**P:** If that is the case, it is what I told you previously ~ about the diet.

**I:** Okay. What else? We are talking about lactating mothers.

**P:** When lactating mothers breast milk their babies, they usually get their body weakened. So as not to lose weight and be attacked by diseases, mothers work to build their body. In addition to this, they keep the cleanness of their environment, their home and their latrine. They do what is important to their health.

**I:** If there are no additional thoughts about lactating women, let us proceed to the adolescent girls. What are adolescent girls doing to be healthy?

**P:** Adolescent girls are using folic acid in their school. Using such means, they prevent their body from any illness.

**I:** Who provides them the table, the one you told me and that supports blood production?

**P:** They are provided in their school. They continuously get it for 30 days. If the late adolescent, they refuse to marriage when their parents put pressure on them. They in return put pressure on their parents and make efforts not to terminate their education. They express their right by explaining that they don’t need to hear their parents. They, as to the plan they have, protect their health and express that they need to reach at the required level. They express their plan when to get married and give birth. They lead their own life by their own means.

**I:** What else can you tell me about adolescent girls?

**P:** If I have additional ideas, okay….*[key informant interviewee is indicating that there is no additional idea for the time being].*

**I:** Alright. Let us proceed to the next question. It is about nutrition related problems. What are the nutrition related common problems in your community?

**P:** Nutrition related common problems? When we talk about nutrition related problems, there is malnutrition. It is clear. In this community, there is a desire to consume more food. However, the current situation doesn’t allow this desire. Because in our community, which is a rural area, the need to consume milk, egg and even vegetables is high. The other problem is malaria which is related to the environmental condition.

**I:** Is malaria associated with nutrition deficiency?

**P:** When we think of malaria, it is associated with poor intake of food. Malaria attacks you when you are weakened as a result of poor diet. When your body becomes thirsty and weakened, the malaria which has been hidden in your body will revive. Therefore, it is in such a way that malaria and nutrition are related. Sometimes, the prevalence of TB and whooping cough is also common. These are common as the geographic condition is hot and dry.

**I:** How?

**P:** They are diseases which occur when the person is deteriorates.

**I:** You told me some disease related to nutrition. Which groups of people are easily attacked by the diseases?

**P:** The first groups of people attacked are pregnant women. It also attacks lactating mothers as this is complemented by blood lose, then small children. It attacks small children easily because they are thin and also due to the hot condition of the area, those occasions usually take place.

**I:** To what extent are the diseases found distributed in this community among mothers?

**P:** Due to the work done in the preventive aspects of nutrition related problems, there is no big problem that occurred, no deaths among mothers. The fact that the curative aspects of maternal services are also good. Anyway, more than three hundred people are examined for malaria. Because of the hot nature of the area, the issue of malaria is burning. The occurrence of diseases in lactating mothers is also another issue. The occurrence of cough like diseases in lactating mothers is a burden.

**I:** We are talking about the burden of diseases in pregnant women and adolescent girls. If you have additional ideas, let us continue.

**P:** The burden on pregnant and lactating mothers is what I mentioned it before. That is it.

**I:** Do mothers have micronutrient deficiency related diseases. For example, we can mention anemia, goiter…anyway, to what extent do these disease exist?

**P:** Yes, anemia occurs in pregnant women and lactating mothers because the group of individuals I mentioned can encounter bleeding and lack of adequate diet. It is a sort of nutrition related disease.

**I:** Let us consider communicable diseases. Are there commonly seen communicable diseases that mainly affect mothers?

**P:** What does a communicable disease mean?

**I:** Communicable diseases are those that are transmitted from one person to another. What looks like the burden of those diseases on mothers and adolescent girls?

**P:** Yes. There are such communicable diseases like cough and TB like diseases which are transmitted to mothers in this community. Malaria is also a common disease as it is easily transmitted from the infected person to the healthy one.

**I:** What else?

**P:** Okay. We talked about malaria, TB. These are the main diseases that occur in this hot area.

**I:** The condition that women and adolescent girls have no height and weight proportional to their age? Does such condition exist? You can tell me.

**P:** Good. The condition that women and adolescent girls have weight and height proportional to their age do exist. When I say it exists, there are mothers and children who grow well and children who are wasted. This occurs when households do not have adequate amount of diet to consume. This occurs in poor families and in some individuals, it is natural. Anyway, children will be wasted when their health is not good. These are the conditions in which the in balance between age & height and also age & weight exist.

**I:** What can you say about the food insecurity that exists among mothers and adolescents in this village? Do they have, for example, adequate agricultural products?

**P:** It is the absence of good agricultural product that mothers are suffering from diseases. In the agricultural area of the community, only ‘teff ‘and sorghum are cultivated. It is the fact that mothers have these two food items for consumption and of course are using only these two food items that they are facing food related problem. They don’t variety of food as this area is a hot environment. The agricultural product we have is not adequate. The community is in trouble and this is being aired through meetings and also now.

**I:** Do this community have such problems for longer periods?

**P:** No. For example, when we consider last year, it was a good season as the amount of rain we had was very interesting. But, we didn’t have adequate rain this summer. It was only for one month that the rain rained. Much of the agricultural activities are dismissed. Anyway, the output the community was expecting from the agricultural activities has decreased. As I said, the community is in trouble.

**Section 2: Access and utilization of nutrition services and barriers**

**I:** Let us go to the next point of discussion which contains some questions. The questions are about the nutrition interventions that focus on improving mothers’ and adolescents health.

**P:** Well. When we talk about the activities in place about maternal nutrition, mothers do prepare food from different food items. Moreover, there is an effort that helps pregnant mothers get different food items prepared from vegetables and other fruits in the form of soup. Mothers are being made to give birth at health facility and in order to prevent them from water born disease, the supply of clean water is also another intervention being implemented in our kebelle. There are such caring conditions to mothers.

**I:** Is there a system that makes mothers go to health facilities for health service utilization? Can you share me your ideas on this point?

**P:** When we consider pregnant women, they are advised to go to health facility until they give birth. They are advised to visit health facility least four times. This is to make their health good. The other issue is that they are also advised by health professionals to utilize variety of foods as this is one of the ways to keep the health of the pregnant women and of course of the baby fine. For pregnant women who gave birth and faced bleeding, they are advised to take variety of diet prepared in the form of soup and other types to replace the lost blood and also to make the child very healthy. Therefore, we can say that we have such activities supported by health education and of course we are implementing them.

**I:** What about extra meal, do women get extra meal when they become pregnant?

**P:** Mothers get health education on the amount of meal they should consume so that their baby will be healthy. They get health education. For lactating mothers, as we mentioned previously, they can face bleeding and therefore health education is given by healthcare workers to transform mothers’ health to a better stage. Health education is also given in meetings and other gatherings regarding maternal nutrition.

**I:** With regard to iodized salt utilization, what support are mothers getting?

**P:** Good. With regard to iodized salt utilization, mothers are getting support. When I say support, they have got the support to utilize iodine to make the mother healthy as well as the baby. To make the brain development very good, mothers are being made to utilize iodized salt.

**I:** Okay. Let us continue. With regard to backyard gardening, what sorts of activities are being done in your kebelle?

**P:** Okay. When we talk about backyard gardening, activities are being done. We cannot say it is implemented in every household. Anyway, those households with well water are cultivating crop products like salad, pepper and other vegetables. Those outstanding female residents are doing backyard gardening by carrying water by their own energy and therefore are benefiting a lot from it. They are benefiting themselves and their family. Thus, such efforts are being made to support mothers.

**I:** What about safety net program? Safety net is one activity linked with development. So, what can you tell me in this regard?

**P:** When we consider safety net program, it is a sort of work for food. Women are made to work in areas where there is flow of water and are paid every month for the work they do. In soil and water conservative activities, they work and are paid. For pregnant women with a three month period, they are exempted from any activity. Moreover, lactating mothers are also exempted from soil and water conservation activity for 10 and of course 12 months after she gave birth. This prevents the baby from facing a sort of discomfort. Educations for such things are given to mothers.

**I:** Let us focus on the safety net activity and of course other activities that are related to development as well. In this regard, what additional points can you share me?

**P:** With regard to development activities that support mothers I would say that for some mothers, who are household leaders, well water are being made ready and given for agricultural purposes. Last year, there were households which prepared wells by their own expense and supported by bureau of water and energy. There are such kinds of supports as well.

**I:** How is malaria in this community? Is it common?

**P:** Yes. It is definitely commonly observed here. Malaria is very common. It is very common as this area is a hot area. It was common in previous times. It started to inclined and now it has increased in prevalence. Almost the whole kebelle is attacked. Health extension workers are going door to door to treat cases. Plenty of mothers and children are spending their time in the health post. The number is almost huge and difficult to manage. When you ask them what they are doing, they say for malaria treatment. Therefore, malaria is common at this time. It is very common.

**I:** Therefore, since malaria is common, are mothers being given health education/advice?

**P:** Yes, it is being given.

**I:** What sort of support, advice is being given?

**P:** For the prevention of malaria, bed net is in use by mothers, household members so as to prevent from mosquito bite. Also, stagnant water is avoided in addition to the environmental sanitation and personal hygiene they are helped to implement. So, if such things are in place, malaria will decline. Such things are given in the form of health education indicating that if all the prevention methods are implemented, malaria prevalence will decrease. In the case of bed net, there was a sort of distribution to households, but right these days, the supply is terminated. Currently, small numbers of bed nets were here in our kebelle. Additional number of bed net was requested and still we are waiting. Mosquitoes are increasing their number and the previously distributed bed nets are torn apart. Otherwise, health education is being given to mothers.

**I:** Who is giving the health education?

**P:** First of all, it is the health team who can provide health education. It is the professional individual who provides the directions and we as a member of the management will distribute the directions. The administration of this kebelle will take its role and manage it. Anyway, the first team is ‘health’ and gives health education. The administration of our kebelle also makes adjustments to provide health education as well.

**I:** You told me about bed net. Who distributes bed net to this community?

**P:** During the last distribution round, every household has got it. It depends on the number of members a given household has. However, those bed nets are torn apart. When the report and complaint came, then inadequate number of bed net has come in the previous summer. Fifty percent of the community has got and fifty percent not. Therefore, malaria distribution is becoming worse and worse. Anyway, the health extension workers are responsible for the distribution of bed net depending on the list of household members every women development army has.

**I:** Next, what can you tell me about deworming? For example, drugs are distributed for the removal of some infections.

**P:** It is distributed. For example, last year a drug was given to community members for the prevention and treatment of trachoma. It was focused on all members of the community. Therefore, all have got it. Every member of this community has benefited from it. Moreover, antimicrobial drug are given for the treatment of water for drinking, not only for trachoma. Lead by health extension workers, tablets are given to treat water obtained from unsafe sources. Based on the things I told you, tape waters are also treated using the medicine obtained from health extension workers.

**I:** In this community or your kebelle, is there nutritional screening for mothers and adolescent girls and if malnutrition is detected, then supporting those with a malnutrition problem?

**P:** Okay. If state of malnutrition is detected through screening process, distribution of ‘Fafa’ is in place. Fafa is at hand and is brought here for malnourished children and mothers. There is food planned to be distributed to malnourished children and mothers. But, the amount is not adequate for the whole community. There is some amount of Fafa already made ready for distribution.

**I:** When is the nutrition screening program conducted? The screening of mothers and adolescents and TSF?

**P:** It is done every year. When the screening program is conducted every year, children and mothers are screened and given complementary foods like Fafa. Previously, when screening was performed for large number of mothers and children, there was adequate supply of the complementary food. Whereas right now, there is a shortage of Fafa. Even there are mothers who were screened and given cards. Still they didn’t get complementary foods. Screened twice and even three times, there are mothers who are still expecting to get the complementary foods like Fafa since they are promised that they will get it. Before some days, there was distribution of Fafa though it was for a limited number of mothers and children. It is not like the amount we had been receiving before some years.

**I:** You shared me that there are many activities or intervention intended to support maternal and adolescent nutrition. Of those, which one/s are very important?

**P:** The big intervention is health service. With regard to health service utilization among pregnant women and lactating mothers, it is known that you will keep the health of your children provided one’s health is kept safe. The fact that pregnant women are advised to have followed up at health centers and the use of iodized salt by pregnant mothers are the big and most important interventions. Of course, when we thank that mothers can easily be attacked by malaria, there are remedies like avoiding stagnant water and distribution of bed net. However, the direction that makes mothers to give birth at health facility is the most important one.

**I:** Good. You have told me the most important interventions and mentioned other activities. Now, we can say that there are implementation barriers for most of the interventions. What can say about the barriers?

P: The challenges are one low education and therefore lack of awareness. The other is inappropriate diagnosis made by health professionals. They tell pregnant women that they have some time left for delivery and therefore remain home as requested by healthcare workers, but at the end give birth at home. There are such challenges.

**Section 3: Perceived needs of mothers**

**I:** There are things that mothers declare as their perceived needs. It is not what any person, the government thinks for them. It is what they think about themselves, especially during their pregnancy period. What do you think are the needs?

**P:** Taking rest. Mothers express their need to be exempted from other duties. They say, ‘ we are becoming weak and weak and we should be exempted from heavy duty.

**I:** What about lactating mothers?

**P:** It is almost the same. They indicate that it is food what they need.

**I:** What about lactating mothers need with regard to visiting health facility and getting health service?

**P:** When they go to health facility, they sometimes perceive that they need the umbrella to protect themselves and their child. They say direct sun light is not good as think of linking it with illness.

**I:** What about adolescent girls. What do they perceive as a necessary for them?

**P:** They say ‘we don’t need marriage at this time. We need to keep going to school.’ They consider education. They consider economy of their nation and there are words they use in such cases. They rely on their own stand, not on the pressure of their family. They also rely on the advice they get from other people.

**I:** What is the role of husbands in reducing the nutrition related problems mothers and adolescent girls have?

**P:** Mothers have problems. Mothers become pregnant and finally give birth. They also exert efforts on breast feeding the babies. I advise, for example, my wife to go to health facility. The other role is that I communicate with my with and make her give birth at health facility. Moreover, I educate her during her pregnancy period and while she was lactating to consume variety of diet. The advice also. We give them good advice on how to use diet during pregnancy.

**I:** In your kebelle, what looks like the practice of other husbands?

**P:** When we consider our community, males don’t have the same practice. It is not the same. Those with a better education and better understanding usually support their wives. Some husbands are barriers. With regard to birth giving, the problem takes place. Males do not have the big role. There is a shortage of critical thinking.

**I:** When mothers become pregnant, what looks like their practice of changing the diet the use?

**P:** I have been talking about this issue so far. Anyway, they sometimes try to get extra diet. Of course there are poor members of this community. They don’t have and therefore the issue of extra meal may not be the case with them. In the health service utilization, the service is the same for all. Anyways, they try to eat some additional food items to make their health status good, especially in the households where there is adequate resource. In those where there is no adequate resource, of course they cannot fly and bring food related resources but they try.

**I:** Do they increase or decrease it is the question. So share me your ideas in this regard.

**P:** It increases because they need more for their baby.

**I:** Are there foods which are recommended for eating purpose? And foods not recommended? Let us consider mothers. What food should they eat what food should not?

**P:** Mothers are advised to eat some foods.

**I:** What are these foods?

**P:** Fine. One of the foods is soup prepared from red Teff as it generates blood. The other is vegetables as it gives protection for diseases. Meat and eggs are also recommended and given to pregnant women.

**I:** What about foods not recommended for eating?

**P:** I think ox meat could be one. Otherwise, I am not sure about the foods that are not recommended.

**Section 4: Other interventions that improve maternal and adolescent girls’ nutrition**

**I:** Do mothers undergo nutritional screening?

**P:** Yes. Pregnant women undergo screening at least four times and similarly lactating women undergo screening. Sometimes, pregnant women and lactating mothers are measured around their shoulder for the sake of knowing their nutritional status and this is done by going home to home.

**I:** Good. What are the challenges with such screening activity?

**P:** Though I cannot be fully confident to say there are no challenges, there are some. One of the problems is shortage of healthcare workers. By one side, health extension workers undergo home to home visit and by the other side, they stay at their health post to serve clients. They cannot do both things at the same time.

**I:** Do mothers believe that they are among the ones who get benefit from Safety net programs.

**P:** Yes. Safety net does have many benefits. It doesn’t facilitate migration. It allows individuals to get food for work. Safety net is an additional activity other than agricultural doings. Mothers therefore believe that they are benefiting from it.

**Section 5: Perception of age at first birth and birth spacing**

**I:** Good. The fifth issue we have is about birth spacing and marriage above 18 years. This is linked with mothers and adolescent girls. Thus, what do you think is the benefit of having not getting married after below 18 years?

**P:** Okay. For the questions ‘what do you think is the benefit of not marrying under 18’, I think when a given adolescent get married under aged, it will be problematic. The problems are; one-she will be terminating her school; two-when adolescents get married before their mind and physical body become strong it will be problematic. At this time, she may become pregnant and since their body is not well developed, they will face a huge problem. As a result, they may get operated while delivering. Such problems will occur. However, if she gets married when her body becomes strong and is on her own will, then will not face any problem. The pressure put on adolescents in previous times having been bringing all the things I mentioned, but now it is over. There is a situation now when adolescents where adolescent girls express their own stand and feeling. Even there is a condition that adolescents give health education about their experience when they grow up and reach the level they wanted.

**I:** What is the benefit of not having married under aged? You told me its benefit to the mother/adolescent girl. What about the benefit to the child who will come out in the future?

**P:** The baby may face the problems accordingly. For those who got married under aged….

**I:** We are talking about marriage above 18 years and you explained me about its benefits. What benefit does the baby get as a result of marriage above 18 years?

**P:** Yes! Marriage above 18 years does have a benefit. One-when they realized their physicality, their chance of producing more milk to baby will be good and the baby will be satisfied. Then the mother will have the chance to feed her baby having considered the advices and health education give to her. Since she is not immature, she is a mother.

**I:** Is the message ‘she should not get married under aged’ being promoted in this community?

**P:** Yes.

**I:** Who is promoting it?

**P:** It is being promoted by women’s affairs office, health office as I said by linking underage marriage to difficulty of uterus elasticity, the third one the administrative office. All these strive to convey the message at any meeting, at any message delivery point to prevent the occurrence of all the problems I mentioned so far.

**I:** What do they do when they convey the message?

**P:** The ways they consider are; one- when adolescents have reach grade 8, they have the chance to reach at high level education. Two – under age marriage brings about huge Physicality and uterus inelasticity problem during deliver. If there is uterus associated problem, it will bring another problem and if she get married after her physicality become strong, then that will help the mother to delivery safely and will again be good for the baby.

During the previous regimes, 12 and13 year old girls had the chance to get married. Now, it is by preventing such under marriage that the health of mothers and adolescents can be protected.

**I:** Therefore, what is the feeling of the community as such messages are being promoted?

**P:** Good feeling.

**I:** Why good feeling?

**P:** It is linked with current situation. When I say current situation, these days there is no any child who don’t go schooling. The second is the reality of previous times where there were many problems like maternal mortality. So, understanding previous situations are enough to just realize the happiness. Today, even there are situations when marriage is blocked provided it is under marriage. There are parents who by themselves bring their adolescent and request age estimation. This is done along with administration and women’s affairs and other bodies. It is the interest of the parents. Parents have good feeling.

**I:** Are messages pertaining to birth spacing and marriage above 18 years promoted and conveyed in the right way. Is there anything that needs improvement?

**P:** Good. The promotion is being done in the right manner is the main message. When I say it is being promoted, as I said so far, the message regarding the pressure on mothers, education of mothers are considered and when the promotion is done, the whole aspect of mothers will be conveyed and when the message is promoted, the right media in the right time is considered. Regarding birth spacing, it is related to situations and the environment where you are promoting the message. The capability of the family is considered if the baby planned to come out of the womb is to grow very healthy. If a mother gives birth at a year or two years interval, growth of one of the babies will hamper growth of the other and problems will take place. If babies are optimally spaced, their educational future will be pleasant, their growth will also be pleasant. Therefore, it is in such a way that the message

**I:** Therefore, are you telling that the message regarding birth spacing and not marrying below 18 is being promoted in the right way? Anything that needs to be promoted other than those you mentioned?

**P:** That is all. The community has a good understanding. The things mentioned so far have got acceptance by the community. There is no one who complains. There is no pressure from the community regarding marriage above 18 years and birth spacing. Almost, the community has formed a state of unity.

**I:** What opportunities do we have for birth spacing to be successful?

**P:** Regarding the good opportunities, yes we have good opportunities. Because individuals have got tablets and injections that help mothers give birth optimally. It is based on the interest of husband and wife. It is when we have well-spaced children, as a family, that we achieve good health.

**I:** When we say birth spacing, how many years is it?

**P:** In our community?

**I:** Yes.

**P:** The time one child should stay breast milking and with no upcoming child coming is, she should stay for three years and in the fourth year, she will may need to be pregnant and give birth.

**I:** What if the mother gives birth before that interval, before four and below years?

**P:** The problem that could come from this is the pressure it puts on families in addition to the hot condition we have here. Due to these things, mothers cannot buy clothes to their children as the life will not be balanced with the existence of shortly birth spaced children. It poses you problems, anyway. To let your children go to school and feed them, the number of children you have is a decisive factor. It puts pressure on families.

**I:** Okay. You gave the information regarding birth spacing to be 4 years. Where did you get this information from?

**P:** The health education we receive tells us the interval.

**I:** Is there segment of your community that still don’t have the information about birth spacing and marriage above 18 years?

**P:** In our community?

**I:** Yes,

**P:** I don’t think. Every member of the community has the information. The promotion reaches every household through health education.

**Section 6: Communication and information source**

**I:** I have the last question pertaining to information and communication. In your community, are there communications and gathering that focus on maternal and adolescent nutrition? What does it look like?

**P:** It is not clear. What was the question about?

**I:** That means, the nutritional issues pertaining to mothers and adolescent girls and the support the community might have for them. Do you have discussion forums?

**P:** Please pause your recorder *[participant needed explanation before he proceeds to the answer].*

**P:** It exists. We conduct meetings among participants selected from different categories to talk about mothers and children. It is about nutrition. Anyway, we do have such meetings. In previous times, there were many such activities through these days there is a sort of carelessness. Otherwise, there is health education.

**I:** Who gives health education?

**P:** The health education is given by health workers. May be these days there is some what a sort of irregularities in health education provision. Otherwise, the health extension workers here the health posts are giving health education. They gather family members and make their effort to reach the information to the whole population. Sometimes, they could educate 20 people at a time and so on. So, they deliver health education in such a way.

**I:** What sort of information do mothers need during their pregnancy period? The information.

**P:** The information mothers need while they are pregnant is, as we have been talking about so far, the information is intended to promote them to a better health. A pregnant women needs care until she gives birth. They need information pertaining to their visit to health facility, the need to give birth at health facility and again the need to replace blood lost through bleeding using nutritional foods. They also educate them about the basic behind balanced diet.

**I:** Okay. What else?

**P:** Other….[participant remained silent indicating end of the idea].

**I:** Well. If you don’t have additional information about the issue you talked me, where did you get the ideas you told me?

**P:** They get the information from healthcare workers. The workers have the norm that ‘any mother should not die while giving life’. This is Mele’s word and the education which ticks Mele’s [Mele is to call the late PM of Ethiopia] word is conveyed by health extension works and by kebelle administration at meetings and other gatherings.

**I:** Do you think that all mothers get such information?

**P:** It may not reach all mothers. But, health extension workers’ home to home visit probably is disseminating the information as there are pregnant women who cannot move easily. Anyway, the information reaches all mothers through house to house visit by health extension workers.

**I:** What can we say about the barriers that might be hindering the dissemination of information to some mothers?

**P:** Not going to health facility for health service utilization and therefore not getting health education is one of the problems that can occur. The second one could be is the fact that some have the belief to give birth at home.

**I:** What information can change the behavior in changing the diet of pregnant mothers?

**P:** The first thing that can change mothers’ behavior to undergo change in behavior is the health education they obtain from health extension workers and the other one the discussion that exists between husband and wife. If there is a good discussion between husband and wife, the probability of the mother from having a pleasant delivery is high.

If husband and wife have a productive discussion on how to take diet, then it is such thing that can change their behavior.

**I:** What do you think is the most important information that mothers need?

**P:** The best information, though we are repeatedly mentioning it, is that mothers should be given and be motivated by the information ‘it is the good health status of the mothers that brings about good health status of family members.’ It is such information that motivates them.

**I:** Fine. At the end, you can share me any other information pertaining to maternal and adolescent nutrition.

**P:** The information I would like to share is that there is shortage of diet. Therefore, like the supply of complementary food we had in previous times, we might need such supply currently as this community does have shortage of food items. The heath post we have is small and needs to be rebuilt as the service seeker from this community dictates.

**I:** Good. Thank you very much. You stay with me have enabled me to get plenty of information. Therefore, thank you very much. I have learned a lot from what you have shares us. If there is any information you need related to the discussion we have, you can contact us. I have finished. Thank you.

**Summary**

- The principle ‘any mother should not die while giving life’ is among the best motivating factor for mothers as repeatedly mentioned by key informant.
- Malaria is the disease that hugely affects mothers in this kebelle as the atmospheric condition is hot and convenient for the occurrence of the disease.
- Interruption of complementary foods has hindered the fight against malnutrition in mothers.
- Key informant has constantly indicated that birth spacing has a significant effect on the mother and finally on the mental development of the child as well.
- Community mobilizations which support communications regard maternal nutrition are not as such happening in the community.

The end
