# Supplementary material for: Barriers to utilize nutrition interventions among lactating women in rural communities of Tigray, northern Ethiopia: An exploratory study
Source: PLoS One. 2021 Apr 30;16(4):e0250696. doi: 10.1371/journal.pone.0250696 (PMC8087028; doi:10.1371/journal.pone.0250696)
Supplement: S2 File — (ZIP) [file pone.0250696.s002.zip › S2_File.Doc/Community level Key informants/067_Agriculture expert_Lemlem Kebele_Samre woreda.docx]

**Operational Research on Adolescent and Maternal Nutrition in Northern Ethiopia**

## **Tool A**

## **IN-DEPTH INTERVIEW GUIDE,**

## **Agricultural extension**

Hello, my name is Omer Seid. I am from Mekelle University. Thank you for taking the time to speak with me today. We are doing research on the factors that influence the nutrition of mothers and adolescents in collaboration with the Regional Health Bureau and UNICEF. Your participation is very valuable. The things that you tell us will be used to improve nutrition programs and services for women in the region and the country. We will not share your names when we report our results.

Do you agree to participate? **yes**

| **Section A: Interview details** | |
| --- | --- |
| **Questions** | **Answer** |
| Zone | South east Tigray |
| Woreda | Sahrti Samre |
| Kebele | Lemelm |
| Name of key informant | Tesfaye Hilemelecot |
| Institution of key informant | Agricultural development office |
| Interviewer name | Omer Seid |
| Date of interview | 11,November 2017 |
| Interview start time | 10:27 Am |
| Interview end time: | 12:23 Am |

| **Section B: Interviewee professional information** | |
| --- | --- |
| **Questions** | **Answer** |
| Gender | Male |
| Age in year | 26 years |
| Highest level of completed education. | Diploma, level four |
| Current job/position | Natural resource management |
| How long have you been in the current job/position: | 3 years |

**Section 1; Common maternal (pregnant women, lactating women and adolescent girls) nutrition problems in the community**

I: What are the common nutrition problems in the community for women and adolescent girls?

P: As far as I know no problem of plump nut and FAFA shortages here, I thought the woreda has enough plump nut so supply problem from the woreda. Plump nut is for lactating mothers child and FAFA is for pregnant and lactating mothers.

I: Do you know women that have supplemented pump nut or FAFA in this area?

P: yes there is. Plump nut is given for severely malnourished children’s and women’s. The health extension worker of the area has measuring tape that used to measure the nutritional states of the child. While measurement a child, if the tap meter showed read the child is severely thin or emaciated so plump nut will be supplemented. The HEW has doing home visiting and has made and checks the nutritional statues of children and mothers using the tap meter (which is MUAC meter). The plump nut will be given until the child recovered, has no plump nut supplementation if the child has recovered and get fat, but continuing the supplementation if the child is not recovering and still thin. One week plump nut ration has given for the malnourished child and then in every week the child will come to the health facility to collect his/her weekly the Plump nut supplements. If the child is improved it will be stopped to give, if not the supplementation will be continuing.

**I:** I think you are said FAFA is for women’s?

**P**: yes

**I:** This is the diagnosis of the occurrence of maternal malnutrition in this area, is that? Like, thinness shortness?

**P**: yes, there is

**I:** Why mother of these area are thin and short?

**P:** Most of the time this area community is drought exposed food insecure community. In this area there is persistence drought which occurred in every other year and even it is a drought year, this year. May be you might not see Teffes, sorghum and kidney bean plots in your ways while you are coming to here, because of the drought this crops has not given yielding and are harvested. In last year the season was good, has had no drought and frames has agricultural production, but this year there is drought. Thus, the drought has occurred in every other year, and hence the area has hot temperate climates, which means in each year no agricultural productions in this area. During drought year like this year, there is a food aid for the community, it could from productive safety net program or it could be from the government support through emergency nutrition program. Majority of this area resident community are poor in economically and if there is drought the pregnant mothers and children’s will be getting malnourished easy because they don’t have any access of diversified food for consumption and has no economic access to buy foods also. There is FAFA and plump nut supplementation in this area which is from the government because the area is food insured, and the community is poor, has no economic access to buy foods

**I:** This study focus is on nutrition of pregnant and lactating and adolescents girls, does micronutrient deficiencies (such as anemia, night blindness, goiter) are problem of this study groups?

**P:** What …what?

**I:** Does micronutrient deficiencies such as anemia, night blindness, goiter are problems for pregnant and lactating mothers and adolescent girls of this kebelle?

P: There is ambulance; majority of pregnant mothers has using this ambulance for transportation to Samere health center to give delivery, so no blood loss and anemia during giving birth. The problem is no diversified food consumption of lactating mother because they have no different cereals and grain for consumptions, may be sometime if there is milk, they drink. Last year we did study on pregnant and fewer than five children’s for FAFA supplementation and has found that anemia was not a problem. May be sometimes health works may get anemic pregnant mother while doing anemia test but am agriculturalist expert so I do not know more.

I: My question is, from your observation does anemia a problem among women?

P: No it is not a problem of our kebelle womens

I: What about vitamin A deficiency and goiter?

P: Majority of the community doesn’t have goiter, but if you visit each household of the community rarely you can get goiter affected persons especially from very old peoples.

I: Overweight, women and girls?

P: Smiling... no

I: What about diet related non communicable diseases like hypertension or diabetes, among pregnant and lactating mothers and girls?

P: No, hypertension or DM affected pregnant, lactating and adolescent girls in this area.

I: Which groups of the community most affected by the food shortage?

P: In this area old people has no potential or capacity to work so they are the most affected segment of the community by food shortages, so what we did is giving the productive safe net supported .

I: Which women groups are mostly affected by these nutrition problems?

P: Pregnant women and adolescent girls have husband and have brothers respectively so they will not affected by malnutrition easily, because their husband and brother will do food support by working abroad, but after giving birth (lactating) mother may not have food for consumption most of the time, and so they will be malnourished easily, for that FAFA and plump net will be given for this lactating women. The problem of malnutrition has occurred during lactating but malnutrition is fine or not a problem during pregnancy

I: Who will be most affected by food insecurity, women or male?

P: That is women’s

I: Why? Which women groups?

P: lactating women’s and old people have most affected by food shortages

I: why?

P: Most old people haven’t power to work by going abroad like Samre city or other kebelles for food. Pregnant and lactating mother are also risk groups for food shortage, because they will not going to Samere city to work for foods. eg. Take me, if am not teacher or a student, I will go to Samre city or somewhere else to do work to get money then I will send the money to my family for food. So, If the pregnant and lactating mothers do not have family members like me they will be food insecure easily, because nobody will go to Samere or other keblles for work and sending money for food. Pregnant and lactating mother have no potential or capacity to go and work in Samare city or other kebelles for food.

I: If there is a food in the household, for whom priority will be given, male or female?

P: It is given for females,

I: Does women of this area are short in height and thin in body size?

P: Yes, there are women’s with short in height and thin

I: Why they are short and thin?

P: Because agro-ecology of the area is sunny and has hot climate. In this community women are cow keepers so they will leave to home and going to fields keep cows and goats in the entire day. Unlike to other areas, girls of this area will not going to school when their age is at seven, which is schooling age, rather they will going out of home for cow or goat keeping in the field. Thus, this is the reason which makes women’s short and thin because they keeps cows/goat in hot climate out of home.

I: What will happen in hot climate? Or what is the relation between cow/goat keeping in hot climate with thins and shortness?

P: There is no adequate water so they will be thirsty and they will be hunger because they eat only one or two times per day (break fat and dinner only). Per day, if you are goat or cow keeper, you will have one meal in early morning at 6:00 AM, and then your second meal will be in the late evening at 6:00 PM. At least these women should eat three meals pre day for good nutrition, but they are not doing it in practically and that why they become thin and short.

I: I think it works for males, but does females do cow keeps usually?

P: It is the same for males and females. In this area both male and females will go to fields to keep livestock’s starting from age five or six. For example if the father has only female’s children and have no male they will going for keeping livestock’s in the field.

I: As you said there is drought in every other year, so where did the community get food during this time?

P: Mainly there is a food support from the government. In this kebelle we have 1019 families that are supported by productive safety net programs, and the rest are supported by the emergency nutrition program. If there is drought like this year we do report for the woreda about the productivity per hectare and then the worda will do the report to the region accordingly. Eg: in this kebelle, *Teff* production is three kuntal per hector, and then the food gap will be calculated by the woreda and it is converted to yearly demands or gapes of this kebelle then it will be send to the region for requesting food aids. In 2007 Ethiopian calendar there is a drought in this kebelle so the whole community of the kebelle had received food aids from the government. At that time it is given for one person 15 kg wheat, 2 kg pea/lentil and 1 litter oil for one month, and this supported was from the government.

**Section 2; Common priorities in the woreda**

I: What maternal nutrition (pregnant, lactating and adolescent girls) interventions are the priorities in this woreda?

P: Do mean our intervention?

I: yes, and in addition you can tell me also your woreda innervations.

P: There are pregnant and lactating women’s, we exempted these women’s from kebelle soil and water conservation developmental works and the productive safety net support will continued, thus, the woreda and the keblle have doing to give the food support of the productive safety nut for pregnant and lactating mother in to free.

I: Why you give in free for pregnant and lactating women’s?

P: To prevent these women’s from workloads and this area is hot climate area so to prevent mother from sun and pregnant mother may aborted while doing the developmental works so we allow her to prevent abortion.

I: Do you have any other priority interventions for adolescent girls (10-19)?

P: Mainly these age group (10-19) age young women’s are school students and has no work. In the school there is affirmative action to make them competent with their male counterparts and this done by school teachers

I: What is your specific role here (in the school intervention) for adolescent girls?

P: Our support is only for adolescents that have no family,

I: What do you do support for this adolescent girls?

P: We give the free food aids from the productive softy net program because they have not family

I: Do you have any other nutrition intervention in addition to productive safety net?

P: During community meetings or gathering with HEW we do promoted nutrition; good child feedings practices, pregnant mother feedings and lactating mother’s feedings and also old people feedings. There is CC association; during these CC meetings we do theses nutrition promotion.

I: what is CC?

P: CC means community conversation and has doing good activities in the community. In the community if someone is in crisis it could be female or male the community will do support this persons though the CC. The CC will collect the support from the community then will be donated to crisis affected persons; especially this is done during holydays. For adolescents (10-19 years) , who do have not family, the CC will collect 20 up 100 birr per households then will give them for their clothes and foods. If the government support is delayed the CC will do collected from the community then do support for those affected persons, and rich persons of the kebelle will also supports foods for the crisis affected persons.

I: Do you have any other intervention still?

P: There is no other intervention.

I: What nutrition services do you spend most of your time on?

P: Mostly we kill our time on teaching about appropriate feedings; that is about appropriate feeding of children’s during community gathering. During these gatherings, usually lactating and pregnant mother has made complain for us because they have no food and milk for appropriate feedings, and then they ask us plump nuts and they ask FAFA supplements. They complain why you give plump nut or FAFA for someone or Tesfaye and why not for me? Always they need FAFA or plump nut supplements. The health workers, HEW, has MUAC measurement for supplementation but mother are not confortable and has not agreed on this HEW measurement, rather mother has come with their subjective judgments on the nutritional states of her child and her own also , then fighting to argued us to get the plump nut or the FAFA. But, we solve this challenges through discussion, what we did is, doing detail discussion with community at the presence of the kebelle leader, reached in agreements

I: What is the content of that discussion?

P: The discussion is justifying the community why plump nut or FAFA is given for some children or women’s. The women complaint’s the HEW, they said there is unfair distribution of plump nuts by the HEW, and has said my child is thin but have not get plump nuts like the HEW do for other children. Mothers perceived the HEW has creating the biased and this is the usual complain which is from the community

I: What nutrition interventions have the most resources allocated to them?

P: Now we are strongly working on the prevention home delivery. We all (from health sector and agriculture) are going and visiting each household of the community then making the pregnant mother to give birth at Samre health centers. During the visit If we get the pregnant mother that has left only one week for giving birth, we do teach her and her husband about the importance of giving delivery at Samre health centers by skilled health worker.

I: Do you think it is necessary for your institution to get involved in work aimed at improving maternal nutrition? Explore for pregnant women, lactating women and adolescent girls.

P: Yes necessary. As the agriculturalist we can teach nutrition for the community which about diversified food consumption and avoidance of monotonous type of food consumption. If they eat flat bread, or enjra for the breakfast, we do teach the mother and sister to do “wet” from differ food items to make it diversified intake.

I: why you do teach diversified food consumption for womens?

P: Because it is important for health. Even if we take children’s they prefer to eat when the food is diversified but if the food is only *Enjera* in every day meals they are not egger to eat. Thus, if you eat diversified *Enjera* and *we*t you will be healthy.

I: But, my question is it necessary for your institution to get involved in work aimed at improving maternal nutrition

P: Yes necessary what we will do is improving productivity through using fertilizers, using row plating styles and using quality seeds for plantations and by doing water and soil conservation activities. Thus to get better yelled, starting from the month of May and June we do teach the community about appropriate fertilizer utilization, production using technology motor pumper like production of avocado , mango and vegetables like lettuce, and then we do teach its consumption for good health.

I: Can you tell me some of the successful maternal nutrition interventions that you have implemented in this woreda?

P: If we evaluate our nutrition intervention, in comparison adolescents has good understanding than that of lactating and pregnant mother when do promotion of nutrition’s.

I: Why this happen?

P: Because adolescents are literate but pregnant and lactating mother are illiterate’s and are farmers most of the time. Thus, it takes long time to teach pregnant and lactating mother, but it takes short time to tech adolescents. Because this we are using adolescent girls as media to teach lactating and pregnant mother. We do teaching adolescent girls about good nutrition, in the meantime we inform adolescent girls to transfer this nutrition message for their community pregnant and lactating women. So through adolescent girls we tried to reach lactating and pregnant mother

**Section 3; Nutrition interventions that improve adolescent and maternal health**

I: What kinds of nutrition interventions are in place to improve adolescent and maternal health in this woreda?

P: When I speak generally, jointly with HEW, schools we have been doing to improve nutrition. As the agriculturalist we do to improve agricultural production. Starting from the month of May and June we give support for farmer regarding to plot preparations, about row planting, using quality seeds and fertilizer to improve their productions.

I: What specifically you are doing for pregnant, lactating mothers and adolescent girls?

P: Therefor you need nutrition only

I: yes,

P: As agriculturalist to improve nutrition what we do for women’s and the community is promoting good production and supporting farmers to do land preparation for good productions , prevention of deforestation, hibreeding animal for getting better meat, milk and butter. With HEW we do promotion to prevent home deliver and family planning utilizations and the importance of increasing birth intervals and about feeding practice.

I: What you are doing on the feeding practice currently?

P: About appropriate feeding practice of children. Promoting the importance of schooling of young children, if young children are cow boys they will become malnourished because have no access to eat three times per day because they are out of home in the whole day. However, if they are students they have access to have three meals per day. There are some people that have too many livestock’s in our kebelle, and children of these people are not going schoolings because of keeping this animals in the field.

I: What about women’s?

P: If her child is student the pregnant mother will handover and responsible to keep livestock’s by going in to the filed but to prevent this, if we give poultry, or other small animals she can do the production at her home and she will not need to go out of home.

I: what specifically do for pregnant mother?

P: For pregnant mother with HEW we educate her to consume eat fruits like kale, avocado, mango. Counsel her to drink milk, do apply butter on her head, do not going long distances out of home, take rest and don’t carry heavy loads like fetching of water, do not sleep the whole day rather do some activities/exercise in the home. Pregnant mother will get the productive safety net food support in free without doing the developmental work and the same is true for lactating mothers.

I: Is that important to apply butter on pregnant mother head?

P: Yes, it is important to prevent sun exposure/ sun burn and to make soften her skin

I: What about lactating mothers intervention?

P: lactating mother has similar food intake like that the pregnant mothers.

I: What about iodized utilization?

P: We teach the whole mothers for iodized salt consumptions, which is to prevent goiter

I: What about productive safety net program for lactating mothers?

P: Starting from one month of pregnancy up to eight months after deliver mother will be exempted from the safety nut developmental works. Until the child starting to walk, the mother will get the productive safety net food support in free.

I: What about adolescent girls (age 10-19 years), do have intervention for?

P: is it nutrition intervention or another?

I: Nutrition?

P: After giving birth mothers do set at home, so to support this women nutrition we do teaching for her adolescents girls how to prepare and feed this delivered women. With HEW we do this activity, and then though her adolescent girl we tried to support lactating mothers nutrition.

I: What other additional support do pregnant women gets, in addition to the productive safety nut?

P: What they get is, in addition to the free productive safety nut support, currently for each mother five sheep’s has been given by the agriculture sector. The pregnant and lactating mothers are the primary targets and beneficiary of this sheep support, and next the targets are all females that have capacity or potential for reproduction of sheep will be benefited, based on their capacity three or five sheep’s has been given for reproductions.

I: What about bed net utilization?

P: It is locally known as *Zanzira* , primary this *Zanizira* is given for pregnant and lactating mother and if we have to much it is also given for other people of the community. Thus, the HEW do gives and teaches the bed net utilization and teach mother when the zanizra will be washed, but it is not our focus to do on.

I: In your opinion, which of the above programs are being implemented successfully (i.e. in the most effective way?) Why? P: We did successful interventions on adolescent girls.

I: What is that successful interventions for adolescent girls?

P: last year with school teachers we bought and give cows for school adolescent girls that have lost their father and mother. Then, this adolescent uses this cows for farming’s and has got good yelling’s in crop production.

I: Is that cow or oxen? Or do they do farming’s using cows?

I: Yes, possible to use cows for framings, if they are poor … (he responds with laughing). This support helps the adolescent girls to continuing they schoolings, if we don’t gave this support they drop their schooling and going out of the kebelle to find jobs. For pregnant mother we exempted from the safety net developmental works, but at their home they produce vegetables. Nowadays we have one sub-kebelle over there in our area, and there are pregnant mothers in this sub-kebelle, we do exempt these women from the productive safety net developmental works starting from one month of pregnancy. At her home she can do vegetation of kale, eat fruits like lettuce which is used for making diversified “*wet”* for their consumptions.

I: What kind of intervention you are doing for none school going adolescent girls?

P: We don’t have any intervention for this group.

I: In your opinion, which of the programs mentioned above are less effective? Why? I: We did less effective intervention on lactating mother. We do giving nutrition education for lactating mothers but are not giving the productive safety nut support for some of these women. There is also emergency nutrition supports but still we are not giving priority for lactating women. We do prioritize and give if the lactating woman is rich we don’t give these supports. Therefore this is what we identified and has taken as our weakness because we did not do too much on this. If the lactating mother has no husband, but if she is rich we will not give the food aids being as a female because she is rich and this is our weakness.

I: Am asking you, from the intervention that you are doing, which one is less effective?

P: I will go for all, for adolescents, lactating’s and pregnant

I: Yes

P: We are less effective on interventions of animal resources, eg. Let’s take poultry; it doesn’t require large effort for reproductions. But we did nothing

I: Oky, can you tell me the effect and the less effective intervention that you are doing for pregnant mother?

P: From the pregnant mother we do effective interventions on irrigations and through it the pregnant mother has producing vegetables and then consumes, but we did less effective intervention on poultry. We are not making the pregnant mother to consume eggs by having poultry.

I: What about the effective and the less effective intervention you are doing for schooling girls?

P: As the agriculture sector with the administrator of the keblle we give support for school going top ten adolescent girls. We supported exercise books, pens and cows, so we take it as successful intervention

I: What are the implementation challenges that are specific to delivering the maternal nutrition interventions in the programs that we have been discussing? P: We don’t have enough time for nutrition intervention works. Me my self is busy by other workloads. This kebelle has need four DA workers but we are two, thus we are busy. eg. I am natural recourse management experts, but am doing irrigations and AI activities also, which is because of shortage of staffs. In this case I don’t have time for nutrition interventions. Another challenge are: going in to the community in the temperate climate is so difficult for us while are going to teach the community. Community perception for nutrition is not good, they perceived they know about nutrition so they are not interested for nutrition educations, but in actual senior they don’t know nutrition and so because of the poor knowledge the community take nutrition as simple thing. Another challenge is expectancy/ dependency of the community, if we go with plump nut or FAFA the community will come to participate in the nutrition education but if we don’t have plump nut or FAFA they will not come, clearly they asked us plump nut or FAFA as a prerequisite to participate. Some don’t need to hear our education and has perceived and told us:

**Section 4; Community factors affecting access to maternal nutrition interventions**

**I:** Can you think of barriers that prevent adolescents and women from using the programs and interventions that we have discussed? **P:** There was barrier before now but now it is changed

I: What was that?

P: Before know the community will not take the food support by relating it with Protestantism locally named as *Bente and because the community* refused take the food, oil and the goat*.* Especially the priest (Kesis) strongly refused the food aid but know it is changed even the priest also takes.

I: What about the education states of women and its service utilization?

P: Yes education states determine the service utilization. Currently those 20-25 years old women’s are educated but above 25 years are not educated. If we see their feeding practice during their pregnancy and feeding of their child, the educated women’s has better feeding practice than that of none educated women. So dietary practice of educated women is better than that of none educated one which means the educated women accepts our nutrition education easily and better than that of none educated one. The older/ above 25 are not educated so they prefer to do culture related nutrition practices, like they do before. eg; if i advise none educated pregnant women about giving birth at the Samre health center by skill birth attendant, she may not agree and will say I have Marry (Marriam), she will keep me and no bad thing will happen, and then she may say I will give birth at home.

I: What about transport related problems?

P: we don’t have transportation related problems. About productive safety nut program, before now if you have 10 families you will get the support for the 10 families, but now it is changed and if you have 10 families, you will get support only for 5 families so families having more than 10 are not comfortable by this approach and has complaining a lot. Another challenge is the productive safety net beneficiaries are not interested to graduate from the program even they fulfills the graduation criteria.

I: What about the quality of care/intervention related barriers?

P: There is no problem on the quality intervention. Usually we do community participatory interventions so no such kind problem.

I: Do have adequate resources for maternal and adolescent girl nutrition intervention?

P: We don’t have scheduled nutrition intervention but the kebelle leader is the one who is responsible for scheduling the intervention program. So we don’t have mandate but the kebelle leader has for nutrition works. No community gathering program specifically for nutrition intervention or education, if the community has gathered for other agenda we do nutrition education as an extra agenda.

I: What about other barriers?

P: The woreda higher officials are not supportive for nutrition activities. Top offices will not ask and evaluate our nutrition interventions. I will be evaluated and graded by other activities but not by nutrition. The same is true for HEW, she will be evaluated by the home delivery but I don’t think so for nutrition. So no enough care for nutrition by the woreda, it seems secondary thing. I will not going in to the community only nutrition activities, but i can go to the community for agricultural activities, or afforestation developmental activities and then during the time I also transfer nutrition messages for the community.

I: Great, How can these barriers be addressed to improve maternal nutrition in the community/woreda?

P: No, we did nothing to addressed these barriers yet

I: What I mean, how could the barriers will be addressed?

P: The woreda should support the nutrtition works.

**Section 5; Other interventions that influence adolescent and maternal nutrition and health outcomes**

I: In your opinion, why would increasing the space between each births and delayed marriage (after 18 years) improve maternal nutrition and hence both maternal and infant health?

P: Early marriage is not good for the girls, because she will suffer to fistula during delivery, and other uterus related problem, and psychological related disorders

I: In your opinion, why would increasing the space between each birth improve maternal nutrition and hence both maternal and infant health?

P: Increasing birth spacing is important for the child to get time for breast feeding. If the mother has short birth interval she will be tired and will not prepare good food for her child and for her Owen. For example if the mother has 10 children she will be responsible to do food for all this children in this case she will be tired. If she has few children she will give better cares for her child and for her family. Children need frequent meals if she is tired she will not cook and feed her child frequently.

I: What programs or activities promote increased birth intervals in this kebelle?

P: Yes there are family planning methods that prevented pregnancy up to three years, but I don’t know its name, which is injectable. Another prevention method is tablets

I: Can you tell me about any programs or policies in place in this woreda to prevent early marriage?

P: To prevent early marriage there is committee in the keblle. The committee consists of kebelle leaders, kebelle police, kebelle women affairse, agricultural and rural developmental office. If someone tries to do early marriage first the committee will teach the person but if the person is not stopping still they will go up to court to stop the early marriage

I: In your opinion, are these programs or policies effective? Why or why not?

P: It is effective because now it is reduced. If the girl has relatives like uncles and brother she will not get married early because these people may not agree the marriage

I: Can you think of any other opportunities to prevent early marriage and increase birth spacing?

P: Religious persons are very influential so we can teach the community using them. If the “*nebsebat*” priest of the adolescent girl is not agreed no marriage at all so we should this priest. Majority of the people fears the law, so we should have strong law.

**Section six; Multi-seectoral collaboration to improve maternal nutrition**

I: Do you feel it is necessary for your institution to work with other sectors/institutions to address maternal nutrition?

P: Yes, thus, we can work with the CC, with kebelle leader and with the HEW and also we can work with local shools

I: What will complement the HEW?

P: We do with the HEW is to improve the nutrition. The HEW will teach pregnant mother about her feeding practice and then we do support the pregnant mother how to get this food items fo her good food consumption. Thus, we teach mother how to improve production by using composts, fertilizers, and by applying appropriate farming methods and by using quality seeds. With school teacher we can teach the school students about good feeding and farming’s then these students will transfer the message to their mother and father. The kebelle leader will support us by gathering the community then the HEW and we DAs will teach the community.

I: For multi-sectoral action that effectively works to improve maternal nutrition at all levels, what kind of change in terms of the way stakeholders work together is needed?

P: We started work jointly with HEW, school teaches but it is not strong. The kebelle leaders, HEW and the school should set to gather and develop action plans for joint works. We have to have one or two meetings per week for nutrition activities, the kebelle leader should control or evaluate our nutrition activities and each sector should have specific activities. If do like this we can do better nutrition activities.

I: What type of resistance to the needed change do you perceive or have you experienced so far?

P: If we work jointly together we will do good things for the community to improve nutrition. No resistance at all because here each expert is under the control of the keblle leader so if the kebelle leader is a coordinator of this multi- sectoral time nobody will resist. We all fear the keblle leader to resist because if we do resist it will be misinterpreted into the political direction which will be bad for each of the experts.

I: To what extent does your institution participate in the multi-sectoral nutrition coordinating body at the woreda level?

P: We do tray to work jointly with the health extension works. But it is not formally established coordination. We don’t have joint intervention planning’s but if we meet at the community, has it is by default, we will do jointly.
