# Supplementary material for: Barriers to utilize nutrition interventions among lactating women in rural communities of Tigray, northern Ethiopia: An exploratory study
Source: PLoS One. 2021 Apr 30;16(4):e0250696. doi: 10.1371/journal.pone.0250696 (PMC8087028; doi:10.1371/journal.pone.0250696)
Supplement: S2 File — (ZIP) [file pone.0250696.s002.zip › S2_File.Doc/Community level Key informants/069_Health worker_Lemlem kebele_Samire woreda.docx]

**Operational Research on Adolescents and Maternal Nutrition in North Ethiopia**

**Introduction**

Hello my name is Yemane , I am from Mekelle University; we are conducting a research on the factors that influences the nutrition of mothers and adolescent girls in collaboration with the regional health bureau and UNICEF. Your participation is very valuable; the information that you tell us will be used to improve nutrition programs and services for women and adolescents in the region and the country. We will not share your names when we report our results. The interview may take 1-2 hours and I would like to thank you for taking the time to speak with us today. You have the right to withdraw at any time and I will use tape recorder. Are you voluntary to participate for the interview?

**Yes** No

| **Section A: Interview details**   1. Zone: **South Eastern Zone of Tigray** 2. Woreda: **Samire** 3. Kebele: **Samire** 4. Name of key informant: **Sister Elfu Yigizaw** 5. Institution of key informant: **Samire** **Health center** 6. Interviewer name: **Yemane G/mariam** 7. Date of interview**: 11/11/2017** 8. Interview start time: **09:45AM morning** 9. Interview end time: **5:22 AM morning**   **Section B: Interviewee professional information**   1. Gender    1. **Female B.** Male 2. Age: **52 yrs** 3. Highest level of completed education.    1. No formal education    2. Primary education    3. High school    4. **College education**    5. Bachelor degree    6. Master’s degree 4. Current job/position: __**working in MCH Unit__** 5. How long have you been in the current job/position: **32 Years** |
| --- |

**I:** Interviewer **P:** Participant

**Section 1: Common maternal (pregnant women, lactating women and adolescent girls) nutrition**

***I:*** In your opinion, what are the common nutrition problems in the community for women? What about for adolescent girls?

P: As we know currently there is improvement in terms of women nutrition as compared to previous due to the awareness created about nutrition by health worker, HEW and WDA. When we say it has been improved it doesn’t means there is no problem/challenges at all.

**I: What do women do to stay healthy?**

P: In order to be healthy women need to keep their personal hygiene, well feeding and visiting health facilities. Pregnant women are following ANC and we are advising them what they must do to stay safe and healthy. During their feeding as much as possible they must eat varieties of food by exchanging the pattern based on their capacity for example they may have maize, sorghum, Teff and wheat therefore if all this are available in the house she can grind them and can have diversified food content. We counsel them to prepare necessary materials which may help them during and after pregnancy like clothes for the child and for herself, care giver and money. Since our government has given focus for pregnant women now we can say the service is good.

**I: What do pregnant and lactating women and adolescent girl do stay healthy?**

P: For pregnant women first she should check/tested for her pregnancy, must follow 4 ANC, to come to health facilities without appointment if she feels sick or discomfort for example if she see any fluid from her uterus, abdominal pain, movement of the fetus is reduced/stop and we counsel on feeding practice like eating extra meal.

After delivery before the she goes to her home we advised her to keep personal and the baby hygiene, to have extra meal on feeding porridge and soup. We counsel her to feed her child exclusively breast feeding for six months and the feeding must be frequently and not to give any food like water or any other before six months since sometimes they give water for the baby if he cries due to abdominal pain. It may causes to sick for the child if starts complementary feeding before six months.

She must vaccinate her baby based on the schedule by going to the nearby health facilities.

I**: What do adolescents girl do to stay healthy from your observation?**

P: Since these girls are under age they can be easily cheated by others or their peers therefore they must follow their education properly, vaccinated at school and may be exposed to unprotected sex therefore we provide them counseling.

**I: In your opinion, what are the common nutrition problems in the community for women? What about for adolescent girls?**

**P:** We advise adolescent girl not to get married below 18 years since it affects her during pregnancy and labour. Currently malnutrition cases are reducing previously most children were given milk food but now it is rare in our health center.

**I: When do you give milk for a child?**

P: We were have children with underweight coming from kebeles especially the past three years but now it is not that much seen.

**I: Is underweight common among pregnant and lactating women and adolescent girl in your health center?**

P: It has change and improvement previously pregnant women were admitted and taking milk due to underweight but now almost we don’t have pregnant women in underweight except sometimes rarely happens. When we measure the MUAC of the pregnant women almost all are normal except rarely it may be below normal but not common.

**I: When do you give fafa for pregnant and lactating women and adolescent girl?**

P: Mostly the scree ning is done by HEW in the health post if they are below 22 cm for MUAC they get fafa and oil. They received fafa if the one year child is below 8 kg since this is done by HEW I don’t have detail knowledge but generally from my observation there is improvement.

**I: Is their severe and moderate malnutrition among pregnant and lactating women in the woreda?**

P: I didn’t see any pregnant and lactating women with Severe and moderate malnutrition recently.

**I: Is there any micro nutrient deficiency among PW and LW and adolescent girls in your wored?**

P: Fro pregnant women iron and folic acid is provided since it helps for the mother and the child by reducing the risk of anemia. Of course there are PW who are too far from the health center and they get IFA from HEW in the health post.

**I: Is goiter common among women in your woreda?**

P: Goiter is not common in our woreda.

**I: Is there any non communicable disease among women in your woreda?**

P: Hypertension and diabetes mellitus patients are following in our health center.

**I: Is stunting is common in the woreda? Does it have relation with nutrition?**

P: Stunting is not common in our woreda and but if it happens it is due to nature.

**I: Do you think obesity or overweight is due to good nutrition? Why?**

P: It may or may not be due to nutrition; because sometimes there are people who don’t feed well but get over weighted and sometimes there are people who feed well get over weighted.

**I: Is there food insecurity in this woerda?**

P: As you know the productivity of farmer is dependent on the rainfall; for example last year production were better than this year therefore the farmers may suffer shortage of food as we observed and the farmers reported this is affecting the feeding practice and lead to malnutrition.

**I: Which group are more affected by malnutrition PW, LW or adolescent girl?**

P: Lactating mother more prominent to be affected by malnutrition since the mother is responsible for her and sources of food for the baby which can be easily affected by disease. Next pregnant women also can be affected by malnutrition; for example the food I take and the food pregnant women take is differ in its utilization. The food I take serves only for me whereas the food taken by the pregnant women is shared by the baby which helps for the growth and development of the fetus.

That is we advise her to eat repeatedly for the health of the baby and the pregnant otherwise she will be affected by disease and the baby will be low birth weighted.

**I: Do you think adolescent girl are affected by malnutrition?**

P: Everyone is at risk of malnutrition if they don’t feed properly the same is true for adolescent girl but when we give priority LW and PW are more affected by malnutrition.

**I: Do you think this health center is working in improving maternal and adolescent nutrition?**

P: Yes, that is why we are counseling mothers on the importance of extra meal feeding, giving ANC and delivery service since it has direct relationship with our services. We are working on child immunization, counseling on feeding of PW and LW as structure from top to bottom level. The attitudes of women are good on our service therefore will continue in giving better services this help us to double our effort and serve our community.

**I: What nutrition interventions are in place in your health center to improve maternal health and adolescent girl?**

P: Since our focus is on the women: we followed her starting from her pregnancy till the delivery by giving different services. We counseled her to come for immunization after 45 days of delivery to vaccinate her baby. And we linked the mother with the WDA and HEW to follow her on keeping personal hygiene and how to feed her baby and herself. In addition we advise them the importance of giving birth in health facilities and we were given delivery services for 100 pregnant women per months even though now it is reducing due the launching of samire primary hospitals. The reduction of home delivery is due to the effort of woreda administration, health worker, HEW and WDA.

The reason why we focus on women is: mothers are easily affected by different disease due to poor feeding and delivery related risks this may lead to damage to the fetus and the family as a whole.

Previously pregnant mothers were dyeing due to excessive blood lose during delivery wheras the community call it as “Serakin” known as devil spirit: whereas she is dying due to blood lose but the community has understood it and they are preferring to give birth in the health facilities.

I: **What is Serakin?**

P: I will come to this; previously our communities were not aware of the importance of giving birth in the health facilities therefore the mother were giving birth in the home. When the mother loses excess blood during labour or delivery at home we call it anemia but the community said it “serakin”. Serakin is manifested as retraction of the tongue to the throat and get difficulty of breathing if she died due to this they said “Serakin” has taken her life. Most of the times mothers are not dying during labour rather they are dying after delivery due to blood lose; that is why we teach the community the importance of health institutional delivery since they can get blood if bleeding happened.

**I: Which intervention is more consumed your time and resources in improving maternal and adolescent girl nutrition.**

P: I can tell only on my work since I don’t know the total budget but as we compared expenses of one disease which may cost 100 birr per service is very less as compared to MCH service which starts from pregnancy till delivery and postnatal care (45 days) and all services are free therefore the cost or resources allocated is very high.

**I: Which maternal nutrition intervention is more effective in your health center?**

P: Based on my service in this health center thanks to God; no mother is die due to pregnancy and delivery since we are working day and night even, if it is above our capacity we referred them with ambulance. After delivery we counsel them what to do till 45 days, tell her to come back if she and her baby have fever, chilling, the baby cry continuously and feeding practices.

**I: What nutrition intervention is in place for adolescent girl in the health center and in the woreda?**

P: We counsel them on family planning to use long acting method before they get pregnant when they come for abortion.

**I: What are the nutrition intervention services are giving for PW, LW and Adolescent girl?**

P: For pregnant women when they come for ANC we measures her blood pressure, examine the position of the fetus, weight, height and MUAC. In addition we advise her on nutrition like eating extra meal, diversification of food for example in our woreda there is Teff, barely, Pumpkin, sorghum and maize therefore based on the availability of crops in the home the mother should prepare and eat in the form of injera, porridge and “Qita”.

All foods are coming from the rural to the urban but the mother complain that there is no good food for them as compare to urban setting. For example a sauce of pumpkin is not important as the perception of the rural women whereas it has a lot of importance in its content like folic acid. This indicates first we have to use our products before we take it to the market. Therefore a pregnant should feed well to get healthy otherwise she will be affected easily as well as the fetus growth will be restricted.

We advise her on birth preparedness like preparing cloths for the baby and herself, assign someone who is responsible to bring her to the health facilities.

**I: Is their nutritional screening for PW and LW?**

P: Yes, we measure them their weight, height and MUAC.

**I: What do you do if her nutritional status is low?**

P: We admit her for follow up by giving iron folate, but mostly this screening is done by the HEW and they give them fafa and oil.

**I: Do they use Iodized salt?**

P: Now all most all the communities are using iodized salt since we are advising them its importance like reduced goiter and increase mental thinking. When they pour it must be after the end of cooking sauces in order to reduce the evaporation of the iodine.

**I: Do you advise for women on home gardening**

P: Yes we advise them, to garden in their home like salad and Swiss chard.

**I: Is their Safety net in your woreda?**

P: Yes, there is safety net program given as food for work.

**I: What are the interventions related to WASH?**

P: the first and most important is keeping personal hygiene and environmental sanitation which help us to be free of disease; if we couldn’t keep our personal hygiene and environmental sanitation definitely we will be exposed for different disease. Since HEWs are available in each community; they are giving advice on the importance of keeping personal hygiene and environmental sanitation. In addition now no farmer is without toilet at least they have temporary toilets to be utilized starting from children to adults this lead to reduction of communicable diseases.

**I: Is there ITN distribution?**

P: Yes, ITN is distributed; especially malaria risk area is receiving ITN as well as there is also in door residual spray (IDSR) for those malaria area kebeles.

**I: What services are given to adolescent girl?**

P: In school adolescent girl are getting service at their school about health related issues, like immunization (TT), reproductive health, early marriage, personal hygiene and family planning.

**I: Is their nutritional screening at school for adolescent girl?**

P: I don’t know and we are not giving nutrition screening at school but if government has bring it as a direction I think we can do it. No iron supplementation is given at our level for the adolescent girl but they may give them at woreda level.

**I: Which intervention is the most effective related to PW, LW and adolescent girls?**

P: We can say immunization of children is most successful. Home delivery is highly reduced even though still there is home delivery but I can say we are successful. This is due to the community has awareness on the importance of institutional delivery.

**I: What did you do to be successful for the above intervention?**

P: Previously mothers were dying during labour due to excessive blood lose the community has seen by his necked eye and hears it; the same is true the community has seen the pregnant woman giving births without complication when she delivers in health institutions. Now the community is the witness by his eye and ears on the importance of having birth in the health institution that is why home delivery is highly reduced due to the efforts of HEW, WDA, health worker and kebele leaders. As we know the direction of our government on woman is “no mother should not die while she is giving birth/life” therefore the health worker is more doing in preventing death of mothers due to pregnancy.

When a pregnant woman gives birth in health institution without any complication; she is the one who teaches other pregnant women to give birth in health institution by telling her experience.

**I: Do you think PW and LW are eating well as you have thought them? How?**

P: Yes, I can say they are eating as per our counseling but it couldn’t be 100% but the community has understood the importance of eating variety of foods. For example previously there was no education for children but due to the awareness created in the community now they are sending their children to school. The same is true since the community has awareness on the importance of eating variety of food I can say that they are eating accordingly unless they don’t have shortage of food.

**I: What nutrition interventions are less effective on PW, LW and adolescent girl?**

P: Even though we have good achievement in reducing home delivery we need to work hard to reduced it to zero level. We don’t have full ANC follow up among pregnant women who give births in our health center this could be due to geographical location some of them don’t have access for transportation to come to the health center. For example once they come for first ANC they may not come until delivery; when we ask them the reason why they didn’t follow the ANC, tell us it is too far to come on foot due to no transportation access. But generally they are following their ANC and give births in the health facilities. But we don’t have nutrition intervention for adolescents.

**I: What are that challenges and barriers affecting nutrition intervention for PW, LW and adolescents** girls.

P: As I have said our problem is no road to address some kebeles due to their geographical location but the health worker is commuted to serve his community. Previously each health worker was assigned one kebele and follows any pregnant women in his kebele till her delivery but for those geographical far were not assigning due to no road access for transportation.

**I: Is there any training related gap and turnover of staffs that affected nutrition intervention?**

P: Yes we can say there is lack of skills in handling nutrition related intervention therefore training on nutrition counseling must be given for health worker.

**I: What type of training area do you need?**

P: For example I need if training is given on MCH.

**I: What other challenges are there to provide your services for PW and LW?**

P: We don’t have shortage of iron foliate but we have lack of laboratory services for pregnant women we are sending them to hospital for the past two months. We provide them Vit A supplementation, counseling after delivery about ITN use and feeding of her and the child.

**I: What do you think the challenges of nutrition intervention for adolescent girl?**

P: I don’t know since we are more focusing on MCH service, I am not sure what is going on for adolescent girl. But for the future I think it must be done on the nutrition of adolescent girl.

**I: What do you recommend for the better improvement of women nutrition?**

P: Great, the government is doing his best but as I have told you even though there are improvement as compared to the previous in terms of road access and transportation in addition it will be good to have road access for the kebeles who don’t have road access for transportation as well as for ambulance. Continues support should be given for HEW and WDA since they are working at the community level and mobilize the community to counsel and educate women to follow their ANC and give birth in health facilities. Relate to vaccination since it is available in the nearby health post they can easily utilize without getting tired. And the most important is empowering and motivating HEW to full engage in the community and we are always ready to work with them.

**I: What are community related barriers that of the use of nutrition related services?**

P: Some individuals may not have awareness women nutrition due to no education but no barriers except I mentioned it previously like transportation due to no road access to some kebeles. Since HEW and health workers are teaching the community to utilize all available service they are aware of it but we health workers are responsible in securing the health of the community therefore we should work hard by having double effort.

**I: What is the importance of delayed marriage and birth spacing in terms of nutrition?**

P: Previously there was early marriage but now early marriage is not allowed. Early marriage can lead to pregnancy since the women is not capable of the fetus she may faced narrowing of the pelvic, fistula and may affect the growth of the fetus. But if it is above 18 years since the female is matured she can tolerate all physiological function starting from pregnancy till delivery without complication and the fetus will grows normally.

**I: What are the interventions related to early marriages?**

P: The community has awareness on disadvantage of early marriage, there is law related to early marriage they will be accused to the social justice and women leagues are closely following it.

**I: What are the interventions related to birth spacing?**

P: we counsel all mothers who gave birth in our health center to use family planning immediately after delivery by telling them its importance. If the mother give child again and again without giving space among the child both of the child and the mother will be easily affected by malnutrition. For example if two plants are planted nearby they couldn’t grow well because they are sharing the food or nutrients around their area with competition: the same is true for two consecutive children have not birth interval. Therefore there must be three up to 5 years of interval among the child since it has advantage to the mother in reducing burden, give chance to get rest, get chance for jobs that can generate income, become strong and beautiful and for the child they we be well nourished and grow without any with problem. Therefore based on the counseling they can choose their own interest of family planning method.

**I: What are the challenges or barriers that affects birth spacing and above 18 marriage?**

P: Most of the mothers are using family planning though some of them may give births without spacing due to negligence and early marriage is reducing even if when it happens as I have told women league, HEW and kebele leaders are following it generally I can say no barriers at all.

**I: What must be done to improve birth spacing and above marriage?**

P: It needs all actors’ effort like health worker, HEW, WDA and the community.

**I: Do you think working with multi-sectors can improve women nutrition? How?**

P: Yes, for example there are women league and affairs since they understand the burdens of women therefore they can work with us as partner in improving maternal nutrition. In addition we can take agriculture they are also our partner in improving maternal nutrition.

**I: What do you need from agriculture, women affairs and league to improve maternal nutrition?**

P: Women affairs and league help us in preventing early marriage, using family planning and to give birth in health institution. Agricultures are working with HEW and health worker in home gardening and other social activities.

**I: What are challenges and barriers affecting multi-sectoral collaboration in improving women** nutrition?

P: I don’t know.

**I: How do you the successfulness of the collaboration with women affairs and agriculture?**

P: It is promising not that much strong but whenever we need their support they are supporting us.

**I: What lesson do you learnt about maternal and adolescent nutrition?**

P: As my experience when we come to pregnancy and lobour it is very stressful but when you see the mother and the baby is safely delivered it gives you great pleasure and give you strength to serve more to the people.

**I: What are the opportunities to improve maternal and adolescent nutrition?**

P: There are women that can be model to other community when you show them something they immediately adapt and implement it. These are our opportunity to expand our messages to the community even if sometimes we become hopeless when the community resists for some intervention.

**I: What lesson do you learn due to working with other sectors?**

P: if you work together the result is more powerful than at an individual therefore workings with different sector will increases the result. For example we are reducing early marriage and home delivery due to the collaborative work of women affairs, law, HEW, WDA and the community.

I: **What are the opportunities to work with other sectors to improve maternal and adolescent nutrition?**

**P:** Since we have different meeting and discussion on the same agenda that help us to work together about women nutrition, family planning, early marriage and pregnancy related services.

**I: What additional points or remark would you like to add?**

P: I would like to say adolescent girl should get counseling on Sexual and Reproductive Health service and adolescent nutrition service should be given.

I: Dear Sister Elfu, thank you for your time and discussion, I have learnt a lot from your discussion thank you again. If you have any concerns you can contact me any time take my phone number (my phone number given to her).

Thank you very much for your time and information

**Summary**

Section 1:

- Keeping Personal hygiene and environmental sanitation are important for women to stay healthy.
- Road access is affecting the utilization of health services by some kebeles
- ITN and Iodized salt are available
- No routine nutrition screening at school and community level for adolescents
- Early marriage reducing
- Family planning utilization is accepted by the community.
- Women affaires and agriculture are working with health on early marriage, home delivery and home gardening
- The presence of early adapter is an opportunity to change the community
