# Supplementary material for: Barriers to utilize nutrition interventions among lactating women in rural communities of Tigray, northern Ethiopia: An exploratory study
Source: PLoS One. 2021 Apr 30;16(4):e0250696. doi: 10.1371/journal.pone.0250696 (PMC8087028; doi:10.1371/journal.pone.0250696)
Supplement: S2 File — (ZIP) [file pone.0250696.s002.zip › S2_File.Doc/Community level Key informants/087_IDI_WDA_Felege Hiwot kebele_Tankua Abergele woreda.docx]

**Operational Research on Adolescent and Maternal Nutrition in Northern Ethiopia**

## In-depth interview with kebelle women union’s head

**Introduction**

## Hello, my name is Amaha Kahsay. I am from Mekelle University. Thank you for taking the time to speak with me today. We are doing research on the factors that influence the nutrition of mothers and adolescents in collaboration with the Regional Health Bureau and UNICEF.

## So, do you agree to continue our discussion? 1. Yes 2. No

**Section A: Interview details**

1. Zone: South-East Tigray
2. Woreda: Tanqua Abergelle
3. Kebele: Felege Hiwot
4. Name of key informant: Tiemtey Desssaley
5. Institution of key informant: Kebelle administration
6. Interviewer name: Amaha Kahasy
7. Date of interview: 15/11/2017
8. Interview start time: 10:25AM
9. Interview end time: 12:22AM

**Section B: Interviewee professional information**

1. Gender
   1. **Female**
   2. Male
2. Age: 31 yrs
3. Highest level of completed education.
   1. No formal education
   2. **Primary education (Currently learning at grade 7^th^)**
   3. High school
   4. College education
   5. Bachelor degree
   6. Master’s degree
4. Current job/position: Head of kebelle women’s union
5. How long have you been in the current job/position:
   1. ______ Months
   2. 03 Years

**I: Wow! You stayed long in this position; so, how is that of being in position to you?**

**P**: This position is given to me by the people thinking that I will serve for them. So, as far as they gave me, I am serving; yet, I used to have a fear that it would have been heavy for me due to the family role that I have at home and due to the schooling that I am attending. But, if you believe that you can do anything, it is possible to do; that is why; I am working at it; because, the females believed at me and gave me the position; so, why don’t I work at it! And this is because the females believe at me and even the others believe too that I can perform it well; so, it has not problem at me.

**I: So, let’s talk again about your schooling, because it can be an exemplary for others; because, you have children and you reached the age of 31 years; you have also this position; but when I see other adolescents, they are failing at different grades like at grade 10^th^ being pretended by their peers too. However in the case of yours, you are now learning at grade 7^th^; so, what experiences can it give to others; who persuade you to do so; and how is it going on then, with all the other tasks you have?**

**P**: Anyways, one, age doesn’t limit for education; it doesn’t say adult or young; for all I know, I am young still; and again, I need to be a model for my children; that is, they have to say that what matters with us; our mom who gave birth to us is learning; so we have to learn well; as a result, I have my children at grade 10^th^, and also I have sent my two children for grade 9^th^ to the town (Yechila); and nobody follows them there; their father go from here and follow them well; for example, he said that forty birr will do nothing for me and he went to the town this week and he stayed there for four days; he communicated with the director of the school and he asked him if anything I need about the children; he also received the phone address of the director to follow them and also to give us a call if the children need anything or if they tend to worry so that to give them help. Again seeing my experience there are females who joined education at our kebelle and even at our woreda. I was also casted at magazine with the administrator Gebrehiwot; I have that magazine; but I left it at home right now. And it is even said that why don’t you take experience from her; and it is becoming an experience to them indeed. There are even adults who joined schooling with me; there is one priest who is called Gebru; there is also one guy called Shewit Tella; we are learning at one class and we compete to each other who will win to each of us.

So, me if I have business at woreda, I may go there; otherwise, I passed the day here at my school; yet, if I have work at woreda, I ask permission from my teacher and I go there; then, because I have my daughter who is learning with me at grade 7^th^, I take all the notes that I dint attend due to that another work. So, maybe that class-work may I miss; but nothing notes are missed that they learned it at the day; I took it from my daughter at night. Yet, there are females who are in doubt and who say that will this schooling be completed; but it will be completed as I thought it; it doesn’t have any problem! Now my husband for me is; indeed everyone is not equal; but he my husband about education because he saw many places, he says the main enemy of Tigray is absence of education; which then let present poverty. It is because guys quit schooling that are in poverty; so I will learn; I will join to grade 8^th^ in the coming year; thus, if that of grade 9^th^ will not opened here until that, I will go for grade 9^th^ out of here; it is my decision to go to Yechila and learn there; I will even learn up to grade 12^th^; I will even to continue more than that of grade 12^th^; we are already negotiated about it. But, to create females like me, I have to sacrifice a lot; yet currently, they seem to begin but not much; they dint join the formal education; but they joined the adult learning. For example, one woman called Kiros Belay, reached at grade 5^th^; but she quitted it now; thus, there are women who are following me. and the if there is education, there is change; but for me, they say where will she reach being learned; even for my husband, they say that damn it; if you let your children all at school, who will help you; and also your wife is all the day at school! They also were saying me that how does the time becomes enough to you; but it is enough; I do all what they do at home; they participate at unions; I participate at that too; they to remembrance (it is the activity committed as having celebration day for remembering angels or Saint Marry); I do that remembrance too; we work equal; my extra benefit is that of my school! My school is my extra benefit that I learn it alone. And I let them begin that schooling telling them that it is only heavy before you start it; otherwise, it goes easy when you continue it; I tell them at church; yet little they are joining! At least even that little mathematics that they can use at marketing, they can capture if they able to attend that of adult based learning for three years. So, at every Sunday, they have their own teacher that teaches them at any place wherever they choose; they are learning; thus for example, I am learning the formal schooling; yet, I don’t look down theirs; I go and sit down with them; the reason why is to please them; when I am asked, I give answers so that they will open their mouth (she means they will speak out); then I tell them no one of them is less than me; I am not better than you even by beauty; rather, our government gave us the chance of equality; and it is said females should be involved up to 50%; I tell them all these being with the teacher; and the teacher teaches them wherever they want being it at tree shelter or anywhere and I still attend with them when there is no that of formal education on Saturday and Sunday. Then if they need help from women’s union, I talk some; then, they thought as if I completed a lot; but I am not yet; I need to learn more; when I get someone beyond me, I feel it is heavy for me; and it is because I pass the time with the woreda and kebelle administrators that I know some; so, I tell them that if they are able to harmonize themselves with others, they can know some. So, I let them understand in this way; even they are worried then if I am separated from them; they say what we can do alone if she is not with us. But this is the way how we handle education at our home.

**I: Great! How many family members are you at home?**

**P**: I gave birth to six children; we are total of eight with four females and four males including my spouse and me.

**I: So, how many of your children are students?**

**P**: It is only my husband! There is nobody who doesn’t learn except him. and even the reason for him is that because he passed his life at military service; he fought for the Derg regimen; he also fought at the Ethio-Eritrea war; he came with grace; now he is retired and he is eating being at his home currently. He is helping us that of exercise books and pens for me and our children. But in my case, up to my grade three, I was to use form home; but since my grade four until then; a teacher called Giteom, who was working at Mykimem school has been sent ten exercise books in every year for me that he let me learn; he saw me at that school at grade-three and he said that I will help her as she is learning with her daughter after giving birth six children; he promised at woreda that to help me pens and exercise books up to my grade-eight; thus, the director of our school teacher Gebremariam receives the exercise books form him and gives me until then. I am not learning only by my husband; I have also many supporters.

**I: In which grades are your children learning?**

**P**: My elder daughter is grade 11^th^; my son is grade 10^th^; and one daughter and one son are at grade 9^th^; my daughter and I are learning at grade 7^th^, and last son is at grade 3^rd^ now.

**Section 1: Common maternal (pregnant women, lactating women and adolescent girls) nutrition problems in the community.**

**I: So, let me start from yourself again, you are learning at grade 7^th^ currently; then what health importance did it bring to you, your family especially to your adolescent daughters and may be to the women at your kebelle?**

**P:** This my being at schooling, leave alone by my children, it also causes pleasure to the others too. when a car comes form woreda and when I go using that car to deposit money of the union and or having other tasks with those woreda officials, and/or when car is sent to me from woreda if I have tasks to do at the woreda, and when those my children see me there travelling using that car with those officials, they become very happy; and this creates initiation to my children to reach where I reached; they say that our mom with many tasks and family responsibility reached here; so, what can hinder us to as such! And I tell them too that leave alone you, I am passing the day at school with my many tasks at home and outside; so, what can let you be carelessness; nothing! Thus, to be frank, they are so grown become youngsters; but I have not any one from them who simply stands at road (she means anyone who becomes hooligan); they are so sympathetic to what they are obliged to do; even my husband is also much respected to his speaks though he is not educated; for example, yesterday, he went to the school and made a speech there; and some ones asked that if he may have children; then others told them that those two are his children learning at same grade in that school (Yechilla); thus, they said to the children that how much they are lucky having such kind of father that could help them a lot; so, if someone has support from his parents, really he can understand well things. If children are given moral by their parents like be brave, be cleaver student then you will have better life in the future; you will live the life you deserve; if you say like this to children, it gives them courage; they become so cleaver. So, for these reasons, my learning is just very pleasing to them.

And at health, I think you asked me at health?

**I: Yes, what health importance does it bring you and to your family being learning at school and your all children being at school too currently, what importance does it have at your health and nutrition to you and your family?**

P: If someone is learned, he can understand the health well; you can understand the health; you can understand what to eat; someone may not understand his being wasted due to the problem of eating if he is not educated. A teacher can ask you that how we know either there is disease or not; you have to take him and let him checked. And as to me, leave alone at my own family, I help those pregnant and lactating mothers and their children who I lead them in this kebelle; I help them at checking their health condition not to be devastated, not to be sick. For those pregnant mothers, I give a call to the woreda to bring car; I have a small phone to my capacity bought by 200-300 birr; so, I let the car come and I take them to give birth at the woreda; I go myself with them and I follow their delivery there; I brought them back to their home successfully. The community is also able to understand that it is not good to deliver at home traditionally as it can cause much of bleeding and also transmission of unknown diseases; so, they themselves invite me to their homes to help them and to take them to the woreda; due to this condition, some of them even say that our children are the children of Mearway; as they call me Mearway (to mean my honey); they say that she saved us; it has gratefulness. So, if someone is educated, he can identify what is important or harmful to him. so, because I am learning, leave alone the health of my family, I have the responsibility of keeping the health of the community of Felegehiwot Kebelle; if an ambulance comes and passes via our area, I ask who is the woman taken, what happened to her; so, did she give birth, is she safe, did she get back home; I ask and check all these conditions by calling a phone. Maybe even a woman give birth at home by any means, I go to the home and check the condition; I give an advice about it is not important to give birth at home for the fear of excess bleeding; so, let her to go and be checked for that. So, we understand that being educated has importance; leave alone you reached at grade 7^th^, even those developmental armies that cannot read and write like me they understand it well about the health importance; they understand well about the government that it is standing beside us to help us. The government indeed needs to help us; it needs the community to learn; if a mother is not well, for example, I am leading eight family members at my home; so, if I am not present, that home will turn dark! The light of the home is a mother; if a student doesn’t know this, he is not really student; the light of home is mother!

**I: Very nice; thank you! So, the mothers to learn like you what should be done?**

**P**: I mentioned it earlier; if possible, they have to learn that of formal education; otherwise, they have to learn that of the adult based education for three years and they will able to understand it; because, in that of the adult learning session, the health (She means HEW) will go to them, I myself will go to them as women union head, the agriculture (she means DA) will go to them, and that of from water resource will go to them about drinking the water, and the HEW about the health, feeding and growth of their children, she will tell them there. So, if they learn for three years repeatedly, they will capture the education; it is only that certificate that they may not get from that education; otherwise, they have to understand at least for their home and family; it is because the health of the family can be good if mother is learned; if the mother is learned, she doesn’t want her children to be illiterates; because, she can understand that if I am able to understand learning these two to three hours, so, how about my children if they able to learn six to seven hours per a day, they will understand much; so, I let them understand that learning a mother from home is good.

**I: Good; so is the adult based learning present; is that accepted by the community?**

**P**: Yes, it is present. It has its own teacher recruited for it.

**I: Where is the teacher come from?**

**P:** When I was learning that, they were giving us teachers from this school during their par-time and they were teaching us; but currently, they recruited its own regular teacher that who completed grade 10^th^ from our kebelle and he is teaching that adult based learning; they let to compete three individuals both female and males; thus, the one who is called Mulushet Desta passed the exam and he is now teacher for adult based learning; he is now teaching for them the males and females during Sunday time; in addition, he let the mother decide convenient day for them then he teach them at their convenient days going to their respective three villages; if he give education at one village today, he goes to the another village tomorrow; and at the third day to the third one; it is he that goes to them carrying black board and chalk for the comfort of them; here in the formal education, we go to the school; no teacher comes to us; if we miss the class; will even be asked for it; but in the case of the adult learning, he goes to them at anywhere which is suitable time and pace to them so as not to disturb their own tasks at homes.

**I: When was that adult based learning begun here at your kebelle?**

**P**: I myself learned for three years in it; now I have reached grade 7^th^ coming out form it; so, it accounted ten years.

**I: What are the issues taught at the adult based learning?**

**P**: Firstly, we learn that of Ha…Hu… repeatedly (She means like that of ABCD…); again we learn numbers which don’t exceed that of 10s and that of addition and division; but lastly, I claimed that it has not certificate but learning year after year; and I decided I have to stop giving birth as I have six children; and I decided to join the formal schooling as I am young yet; at the end, they gave us certificate at our third year of the adult based learning; thus, I joined to the formal education.

**I: What else do they learn at the adult based learning about life?**

**P**: Out of it; that is, agriculture by its office, water resource by its office, and health by its office by the HEW, she comes and teaches about the health of the mother and her child supporting the teacher; she gives the education on many days.

**I: So, what importance and changes did it bring at the health of the mothers due to the reason they are learning at the adult based learning?**

**P**: One, to give birth at health facility; second, to have health checkups and to know about their health status; that is, the HEW teaches them to go to medical care and let check about the position of their fetus if they are pregnant; because, if they don’t do that, they may be hurt; so, they have to go two to one week before the onset of labor to the health facility; because, it is more worth to stay there for two weeks than not going there for delivery and leaving the world at all forever; she teaches all these things. Thus, we understand it all; it is for our life; even the government gave us car; the ambulance; so, what is the problem with us not to go there; nothing; we only pay ten birr per year for the wheels and fuel of the ambulance; thus, we are served very comfortably! We go having three four laboring mothers at once; we go for free; our right of equality is approved well; we have no difficulty about it currently!

**I: So, how do you see its necessity; may it be better to quit it or be continued?**

**P**: What’s wrong with us? The government showed us the good thing; so, how comes to interrupt it? Rather it has to be increased; the government showed us by the car we have that we are travelling for free and many mothers are being saved due to the reason that a mother giving life should not lose her life as said by our honored leader (she means late PM Meless Zenawi)! So, this has to be increased; not to be decreased even; we need rather additional cars yet.

**I: How about that of adult based learning?**

**P**: It has to be continued; for all I know, it has to be progressed into the formal education if possible and also it has to be expanded to other too; it is like the issue of that ambulance! There is no border for education; no border!

**I: Very good! Thank you; you gave me detailed evidence; so, mothers like those pregnant and lactating mothers and those adolescent girls here at your kebelle, what do they do to stay healthy including yourself?**

**P**: For example, if a pregnant knows that she is already pregnant, she is checked up; she tells to the health worker that she didn’t see her menstruation for these much of days; thus, after he examines her, he will approve her that she is pregnant then tells her to have follow up visits and she goes accordingly; and she gives birth with no problem. And this let them understand that if I able to handle well my life, this will be for the sake of my children; I have to live for my children; and also the HEWs teach that giving birth at health facility is good to prevent unknown disease transmissions during at home delivery; it is good; because you may say this woman is thin or emaciated; but you don’t know what is the disease with her; is that because she is in hunger that she is emaciated or is that because she is sick that she is emaciated; we don’t know! So for this issue, she has to be checked and she has to give birth at the health facility. So, if she is to give birth to healthy baby, she has to deliver at health facility and this is understandable.

**I: You said that they have follow-up give birth at health facility; what do they do to be healthy after they deliver during their lactation period like about their diets?**

**P**: Is that for the baby or for the adults?

**I: For both of them the children and their mothers too?**

**P**: Firstly, if they were to eat three times per a day during their pregnancy, they increase one times to it then as there is a baby; I increase one time as there is a baby; in addition to the food I eat with my husband and children, I have to add once for the sake of the baby; if the baby is to be born normally, I have to take additional food. Secondly, after you give birth at health facility and come back home with no bleeding, the baby has to take only breast milk of his mother; beyond that it can take additional food; but before that no water, no any food is taken; only that of breast milk; they teach us like this and we understand it. Previously, if the baby feels abdominal cramp, we used to give water, butter and others too which was very traditional; but now due to the government is sacrificing a lot to teach us starting from our 15s and 18s years of age, we understand it well that the baby has to take only breast milk up to his six months; after that he takes additional foods from at home available food sources like of those whole grains and cereals, and fruits. For example, I gave birth to six children; thus, I used to take them to get vaccinations timely; I let them be measured; and when I was told they are normal, I used to be very happy; I go home; then I got back to that but it is the same; and the folder was also checked and my husband name is Atalay Negash; thus, my children used not be said with lower measurements; and the reason why is that because if you able to understand little, as the government brings additional food for protecting food shortage for others, you can protect yourself about your children; you understand it well; and it doesn’t regret you as if something were left form you; it lets you be pleased; I myself personally, I used to be very happy when I got back having no food aid for the children due to they were normal. For example, when I was in grade-3, I was pregnant and I was so sick; due to this I was at lower weight and I was taking that of Faffa; I was then very disappointed taking that Faffa; then when I got back at the next month, I filled the measurement well; they said me that what can we continue due to your normal weight; I said to them that I was so happy for being normal.

**I: Those adolescent girls aged from 10 to 19 years of age are the ones who will be the future mothers who need good health currently; but they are not given focus yet; so, at your kebelle Felegehiwot including yourself, what is being done to make them healthy, and yourself as a model what are you doing to the health of your daughters?**

**P**: Anyways, what I am doing to keep their health after a I joined the school is that, one, I follow them not undergo early marriage; I give them education not to be pretended by them (she means boys); together with the teacher who is head of females’ affair in the school, we give them advises that they should not be pretended by husband; he may need them for the time being which may let them then to be the ones who cares only about their children like us; so, to be free from that you have to go to school and get back home wisely. Secondly, early marriage is not needed for kids; and you are kids; because, one, it causes problem during delivery; second, it causes different unknown diseases like that of fistula and this diseases happens for the one who marries at below age (she means early marriage); it is given them all such advises. One girl was to marry forced by her family on a day of Tuesday leaving her form the national exam of grade 8^th^ that would to be given on Wednesday; they fixed the date to be together with a holiday of (Saint Cherkos yearly remembrance); after that she came to me and told me that they forced her to marry leaving that grade 8^th^ national exam; then I went to the director of the school called Moges, and I appealed there; he asked me to give him a phone address and I gave him the address of the woreda; then he phoned to the woreda women union and affairs offices; then they asked him about my presence and he told them that I gave him the phone and as I were attending at grade 5^th^ but as I got the girl to be married quitted from her grade 8^th^; fortunately again the husband of that woreda women union head was driver and he arrived to the kebelle at the evening; I told him that Oh my dear! I have no phone to give a call to your wife but we came here being trained in Mekelle to protect the girls from early marriage and your wife is teaching me about the girls not to marry and for the women not to deliver at home ; but that girl is to marry forced by her family ; so what shall be done now; hence he gave me his phone to give call to his wife and I phoned to her home address with their own money; she then told me to go to the woreda by the next day early at the morning; otherwise, the girl wouldn’t be saved. Then, I went there and I told that I went to the home of her father having militia with me and I begged him not to let his daughter marry quitting from the schooling; but he refused; I told all these things to the woreda; then they came having their own car; they told me that they came having the car and they will go to his home; I said them okay; the man is at Rezina village; and I am at Guftalmo village; then they came and they went there; the bridegroom was there at the home; and they got the girl too there and they asked her if it was her interest to marry or not; they also measured her age and she was found her under-age; then bridegroom was fired to his home and the girl joined her schooling; she was casted in magazine and now she is learning at grade 11^th^ currently; she completed her age of marriage and also she married with her interest a boy who is student with her and now she is full of gold in gold e from her husband; hence around this month, she saw me in the way and she ran into me to hug me; and I asked her that do you like me or you hate me; what’s up to you; I don’t hate you; it was because I was interested to learn that I came to you and I asked your help; otherwise, there are many other who already married; currently I am learning well being with my spouse. So, it is because I told her not to marry at her under-age and let her to attend her schooling that she was casted in the magazine being said Waero, Waero (she means Lioness)! Hence, I do have that girl that I let her be model here! Again now, that Tirr (means January) is coming; thus, we are making study and that task will not be absent!

**I: When do we say the marriage is under-age?**

**P**: That of 15, 16 and 17; they have not to marry; they have not to marry before they completed that of eighteen; and they marry at their eighteen and above.

**I: What else beyond that early marriage is done to the adolescent girls to make them healthy?**

**P**: Again, when they are to marry, they have to be checked up together with their husbands about their health status; there is has not present marriage because someone is rich; the wealth is that of health; there has to be examination for him and her too; then they can marry.

**I: Great! What else is done like about their diets including yourself for your daughters as you have three school daughters; and how old are your three daughters?**

**P**: The one is 14 years old who went to grade-nine; and that one is eleven who is at grade 7^th^; and that of grade 11^th^ is above twenty years old.

**I: Then that of grade 9^th^ and 7^th^ are at their ages of 14 and 11 that are at the range of 10 to 19 years; so, what do you do for them especial to make them healthy?**

**P**: Is that about their feeding?

**I: Yes, about their feeding to make them healthy, if there is any that you do to your daughters so that we can take it as model for others?**

**P:** Yes, they can marry form the age of 18; but, if they are cared well from the age of their 15, if they are well dressed and if they have their pants; they can’t be startled; that is natural; that is it! (She is talking about the menstruation). This is very natural that they have not to worry about; except they have to keep their hygiene well, they have to wear clean dresses, they have to wear trousers; they have not to fear for what others say; because, when it comes first, many kids cry; they have not be ashamed! We have to teach the kids not to quit the schooling seeing that thing; because, they can fear hen their dresses are being tinged with that blood; even there may kids that hate seeing it let them not to go to school; so we have to let them know it is our natural wealth; and also our husbands can be complaint of that tinged dress thinking that as if the girl was doing another thing as they may not know about menstruation though living with us together (she means having sexual intercourse which then bleeding to the cloth); we have to let them understand that it is because of that our natural wealth that the girl begin to face it; rather, we have to let the girls wear clean dresses and trousers; they have not to dress not clean or thrown pants; they have not to wear someone’s pants; they have to wear their own clean ones; they have to be careful, they have not to have not good friendship; they have to complete their schooling wisely; if their age is then reached to the age of marriage, they can choose what they want as per their interest; if they need to marry, they can; and they lead it all together.

**I: What is the reason that they have not to wear someone’s pants?**

**P**: It is not worn another’s pant; without my health status, my friend has not to wear my pant. Because, liquid leaks to the pant and the same is true from that girl to that another girl. It has to be your own pant washing it very well and changing it timely; otherwise, even your pant is tinged with blood, you have to ask permission and you have to come out from the class to wash and change your own. Otherwise, it is not good to wear seeing someone is healthy at that time being; we teach them this issue very well for the adolescent girls; if they face any problem they have to ask their teachers and have to wash and change their own. For example, the adolescent school girls are friends to each other and share their problems to each other except with the boys; so, if she sees her friend is tinged with blood, she can say here I have clean pant and you can use mine pettily; but this has not be accepted or given; later you will also give it back to her washing it; this has not to be done because they like to each other; we teach them all this.

**I: What problem can it bring to them if they share it?**

**P**: In the case of health, all is not equal; they all are not examined; it is not known which of them are healthy or not healthy; they can have disease; maybe if all are healthy, there may not present problem; but if one is sick but another healthy, when that one wears the pant, disease can be transmitted; thus, this girl will be endangered.

**I: But, are there girls who practice like this?**

**P**: Ayiiiii (seemingly no), we just give the education; but we found no the ones who said we give it; we just give the education.

**I: How about at their feeding; about the type and frequency they should to take; is there any help they get for those adolescent girls?**

**P**: Do you mean at the community or at our home level?

**I: At the community level, does the community have knowledge about practicing it; and at your home level, what do you do for your daughters as a model for others, because, these adolescent girls to be good mothers for the future, they have to be cared at this their current ages?**

**P**: For our children at home, there is no anything that is said this has to be eaten by her or this has not to be eaten by her; if I have full, she has to grow eating full; but now you are asking me that is hot or cold not to be eaten by her may be?

**I: I am asking that what the focus is given to the adolescent girls like it is given to the pregnant mothers to increase their diets from three to four and that of lactating mothers to increase from four to five.**

**P**: For those adolescent girls, perhaps it could be said to them that they have to increase or decrease what they should eat; if I have, my daughter should eat variety of foods; she has not to b forced to avoid something and/or to stick to something alone; she has to eat anything you have being it honey or butter; due to this additional food, she learns and she becomes happy; so, I don’t think there has to be any food that is prohibited for her; she has to eat whatever she order you; unless, there is anything that I don’t know so that I have to learn about it.

**I: In this area, are there problems associated to deficiency of food to eat so that the mothers manifest like thinness to their body, goiter (Hifess), night blindness (Himma), and lower blood level (Anemia) and others that you may observed?**

**P**: So, in this case I have brought one girl being underweight here; and the same like this, previously they interviewed me and take me home to home visits to assess if there were such problems; they asked me to lead them where those wasted children were found at that time it was only about the children. But this girl, I have measured here by my naked eyes that she is emaciated as she gave birth to twin babies; it was at Temben that she gave birth and we were told to bring her back home and we brought her; thus, her one baby passed away but the one became her fate and she is growing well; but she was sick her breast and she didn’t tell me about it; when I tell her to take here for medical care, she refused me fearing that they would cut her breast; at first she told me that she has no money to go; I told her that I would to pay the money by myself and I would to call that she is lactating mother; she refused the; but she went at last; now she is fine but her breast decreased milk and her baby become wasted; then I took them to medical care and her baby was given that of Mitimitta (plumpy-nut); now she is fine. In addition to that, we found three babies who are wasted during our all home to home visits; thus, they took that Mitimitta (plumpy-nut) and they used it; now they are fine. So, they don’t bring the children to medical care when they are wasted; you have to persuade them; thus, I visit their home and I tell them that her baby is getting wasted thus has to take him to the medical checkup; or I take them myself and they get the service. And also there is one lactating mother who comes here who has nothing except that of safety net; so, as per our tradition, we are collecting two to three kg of cereals as per the capacity of individuals so that we will give her; she is beyond one month since she has delivered; and the one thing for her problem is that she quitted her education; the reason why she stopped it is that her mother is widow that she let her learn up to grade 9^th^; but she refused to continue for grade 10^th^ ; then she got boyfriend and delivered; she is not married; it is without marriage (shown at her face that she disgusted the way she did without marriage). Thus, what we do for such kinds of cases is that in that our traditional way that we collect anything available and share them; there was also union called social affairs of the keblle which was supposed to support up to 50 individuals; but yet it didn’t give them any to their hands.

**I: What does that social affairs do?**

**P**: There is money of the people; they give 24 birr per a year; thus, this money is collected at the kebelle and then used for those hurt who have not support from family members and who are ill and devastated by the illness.

**I: Who was organized by this social affair and when was that started?**

**P**: It is the government; and the time is, oh! It is long time; though it was not noticed; I myself remember since I have lead in the position for three years until then.

**I: So, is that functional working until then; are there individuals who are supported by it?**

**P**: There are individuals who are approved by the kebelle to be given; they are around fifty.

**I: What will they be supported then?**

**P**: They will be given the money collected from the community.

**I: How many individuals will be supported?**

**P**: It is as per the money collected; and yet all the people didn’t give the money; so, the kebelle currently has around twenty thousand birr at hand; so, that will be distributed. It will be distributed on the Michael day (on the 12^th^ day of November E.C counting) as it is already approved their names by the kebelle those who are hurt for really like those who are unable to see, and disabled are approved and their name is read to the people and already is understood by the people.

**I: Is this for the first time to be given or was given before?**

**P**: It was used to be given and comes like that until then.

**I: How much money is given to them then?**

**P**: I don’t know how much will it be currently; but in the previous times, it was around 200birr and like that for an individual.

**I: When was the time that the three wasted children you found during the home to home visit?**

**P**: It was in the previous year; in 2009 E.C. but I don’t remember the month.

**I: How about in the mothers and adolescent girls; may present mothers who are emaciated at your kebelle?**

**P**: Yes, those who are thin take Faffa; they ate present many at the kebelle level though I don’t know the exact number.

**I: Who is more affected from the pregnant, lactating mothers or those adolescent girls?**

**P**: Those adolescents, if they are not pregnant, they don’t take that Faffa; they are those lactating, pregnant and under-five children who take that. For example, I am single (not pregnant or lactating); so why not I am ten times emaciated; I am not given that Faffa; I go to medical care and get the treatment there.

**I: Is Faffa is present here; how much is given to the mothers?**

**P**: Yes it is present, and the children are given that Mitmitta (plumpy-nut); whereas, the mothers are given Faffa and they say they are given four; but I don’t know it exactly as I don’t take it.

**I: What if the problem of the mothers is beyond that of Faffa; what is done to them?**

**P**: If the child enters to the red measurement, he is given that of Mitimita, and it also given Mitimitta for test; and after taking htat Mitimitta, if he has not any change, he is referred. And if the mother is beyond the Faffa that unable to be fine, she is referred.

**I: So, are there children or mothers who are referred to higher medical care due to this problem?**

**P**: This one, you may get it from the HEWs.

**I: What do you think about the reasons that mothers are being thin and emaciated?**

**P**: The reason, how can we mention it; as we say it, it is because you don’t follow up properly; you have illness but you dint follow up properly; and also about better food from the products that we have form Teff, sorghum, maize, and barely that we grow them here though there is temporary drought currently; we are expecting our government to bring us maize; we sow wheat, barley, Teff, maize and sorghum here; so, the problem to them is that on the food preparation from what they have; the government that bring to us is food itself; it has not tablet inside; it doesn’t send us tablet; it is food like what we can prepare at our home (she is talking about the Faffa); we have to prepare mixing all what we have at home using oil; even the butter is with us at our home; instead of selling the eggs, we better use it at our home; for example, an egg is sold 3 or 2.5 birr here; I have an American hens and I sell eggs; but when it is fasting, why do I sell them; I give them to my children so then their bones and their health will go being built. But rather than selling that of egg, honey and butter and going to medical care, the first medication is at our home; it has to be if beyond this that we have to go to medical care.

**I: Okay; thank you; is there food shortage here due to drought?**

**P**: Yes for those who take Faffa; but especially this year, it is totally drought; nothing is harvested; nothing is present. You can see it; it is dry; no harvest is obtained; we are waiting for the government; what will do for us. Especially those widows who has nothing to sell, we have a fear that they will be in difficulty that we are worrying at our kebelle and even woreda level that we are thinking that how will the government help them. So, it is very sunny and the drought is hurting us extremely at this time.

**I: So, what is being don e here for the drought?**

**P**: Oh! We will be the load of the government then! It is the government!

**Section 2: Barriers to access and utilization of nutrition services**

**I: What kinds of nutrition interventions are in place to improve health of the Mothers and those adolescent girls at your kebelle; and what barriers are present that hinders you not get the services you deserve?**

**P**: So, the problem that hinders them not to go to the health and not to get the health service is that because they are not educated and lack of understanding; otherwise, they are not said that they should not to go to the health facility; rather, especially for females the woreda give education that we have to go and use health services; unless, those who may not understand it may miss the service; but no one says that this not needed to you; hence, it is free and clear that everything which is brought by government is to be used clearly for all; no body prevents for anybody to use the health services; it comes for all by the government; every one is the child of government; being it the vaccination and other service; everyone is mobilized to use; unless, they hear by one side and release it by another side and they don’t understand it or they feel that it is not important to them.

**I: Do the mothers and adolescent girls get counseling about their diets; you told me earlier indeed, but are these services present?**

**P**: Yes it is present; in the church it is given on every Sunday; and in this institution (she means HP), it is given in every month at the 16^th^ day of the month at Kidanamihiret day (Remembrance Day from the miracles of Saint Marry) (EC counting).

**I: So, what is being said there?**

**P**: It is about child feeding, about mother feeding, about antenatal checkup, about post delivery checkup; without saying this in every month and time the HEWs and the kebelle don’t release us; they even receive phone numbers for follow up.

**I: Who provides all these educations?**

**P**: When it is during that Kidanamihiret here, the HEWs provide the education; and at the church, if the HEWs come, it is well; otherwise, all the kebelle administrators provide the education all what they have received from what the HEWs said and reported; because, the HEWs report all the health and even agriculture related issues; so, all the kebelle administrators provide education being it all about health, education, and children is provided at the church on every Sunday for all the four villages; unless there may individuals who may miss the education due to they don’t come to the church.

**I: How about the religious leaders like those priests; do they work in collaboration?**

**P**: Yeah, at first, they are given more understandings about HIV and about the delivery of mothers; they were told that it is due to the priests that there were problems; but now, if there is one pregnant, first, the woman development army will be responsible; next the thirty houses women leader will be responsible and again her confessor priest (Abat Nisiha) will be responsible for her; thus, because it is revealed that the how many pregnant are found at someone’s group, when the confessor priest goes to the home for blessing, he tells them that they have to checked up and give birth at health facility; they are also able to get understanding at different meetings of the woreda; for example, one priest called Gebremedhin, in this kebelle, took his wife himself two times to the woreda and he let her deliver two times there to as model for others. So, there is support from the religious leaders too.

**I: Very good; may there any barriers that protect them to get services like to go to health facilities, and to get advising and counseling?**

**P**: What could be the barriers for them then? It is because of not educated only that is a barrier to them; they don’t understand it themselves; otherwise, if you let them understand it repeatedly, I don’t think, there will be present anything that could be a barrier to them. Previously during our parents, it used to be said woman has not to come out; she has to stay at home; but now, his is not present; everything is possible; unless, they may say themselves that they couldn’t perform that; even for the husband, if he is learning, she has to also I have to learn; she has the equality that is brought by our previous sisters who fought and die for our equality; and it is being said that 50% is for women; so, she has to use this equality by herself too; so, there may not present that hinders to her; but perhaps, who knows there may present one form hundred that hinder their wives to get what they need; and even for this if they whisper to us, we can go and let him understand by advising. You see; for example previously there were two women came here and I told them to go for training about improved oven; but their husbands didn’t send them; again yesterday, I assigned them both for training and I went to their home; and I told them that listen to me I am leading the kebelle, I am passing the day at schooling and also I am leading the home; what is missed at me; and what is the thing that you have worth to me; so, what is the problem if these women go and be trained; are you interested or not; I asked them; oh; previously we didn’t understand; now, why not they go and discuss they said; now they come for the training though I don’t know what will they talk after they get back home. So, if anyone is repeatedly told, there is nobody that can’t understand. Yet, when you firstly tell them, they say ohhh; and try to refuse; but how about those our previous sisters that went before the males to fight and die to bring us peace; it was them that went bare footed and shared little water among each other and fought against enemy which then brought us this current peace, school and shelter that we are looking at Felege Hiwot at this time; they let us travel by car; it is known that there was nothing at Felege Hiwot previously; it was sunny there was no school and there was no administration; but now there is school, green, and shelter which is brought by them; so, we let them understand this. So, if we let them understand repeatedly, there will understand it; unless it is the problem of let them understand; yet, as per my understanding; the kebelle is sacrificing and the HEWs are also sacrificing too. Yet, if you let anyone understand two to three times as there is no one day education; everyone can understand it well.

**I: You have already told me about the menstrual hygiene of adolescent girls; how about the water access, hygiene and sanitation practice of mothers here and the presence of malaria case in relation to it and the way how you protect it at you kebelle; can you tell me issues about these things?**

**P**: Anyways, our water source is river it is called Alikua, and it is of our hours travel on foot; in 2009 EC, the government let us drink bringing by truck; currently, the government made big dam around; but not installed for consumption yet; still we use for washing purposes from it; otherwise, our water source is very far which takes four hours to go there and get back totally.

**I: So, is that tap water there?**

**P**: No, it is by digging the river. It became difficult to obtain that; the government sacrificed a lot but didn’t get; now as an option, it built that dam.

**I: So, what is left with the dam not to b used for consumption?**

**P**: It is said that it is finished; but the water resource didn’t receive it yet.

**I: Why not received?**

**P**: The educated knows it!

**I: So, who fetches that water from that all four hours travel?**

**P**: That is as per what you have; mainly it is those donkeys that are taken to carry; thus with them, children those who are of the afternoon class go at the morning and bring that water; and in turn on tomorrow those will be of afternoon class will fetch water; as there are no children who don’t learn currently; but if there is on child, you will go yourself having the donkey. There no to say male or female that fetches the water; it is as per who is available at home; yet, mainly the females fetch the water.

**I: So, do you think this water scarcity has an impact on sanitation and hygiene of the food eaten at your kebelle?**

**P**: What can be said; we are not hurt yet which might be due to the high heat at our area which may let the river not to contain bacteria; otherwise, had it been cold like Addis Ababa, the water that we drink from dug river would have exposed us to many diseases; as per to my suggestion, it is because the river is so hot; unless, the water is so bad. It may be due to the heat that the bacteria are not occurred; otherwise, what; and until then there is water born disease that we are told.

**I: Are you given water guards?**

**P**: Since the previous two years, they have been bringing us that water guard in plastic and rubber; after it was said that an acute watery diarrhea was come, that water guard was not differed from us and that is why, there was no acute watery diarrhea at our kebelle; we escaped well from that disease.

**I: Do you think there is an impact to females due to the reason that the water is too far to fetch?**

**P**: In the previous times during our mothers, it use to have problems; but now what it reduced the problem is that, one, that donkey; second, the presence of grinding car; previously, they used to grind by their hands; but now it is by car which is grinded; and also you have your donkey and you take it to the water; so; you bring back loading on it. Because, there were mother who used to give birth on ways! But now, due to the government, everything infrastructure being it road, school, and even electricity is being extended; so, currently, there is no mother who grind manually on a stone; we let it being grinded by car; so, there is no any hurt to her. Even to the water, she goes carrying nothing as she have the donkey and she get back loading the donkey; so, she has not load to carry in that long hour travel; but previously, they used to carry on their back that long hour travels; in addition to that, after getting back from it, they used to be involved at home related tasks like that of grinding, that was the hurt. But now we use the flour grinded by the car to bake what we want; but the distance that we have to the water is our natural problem; the government tried three to four times digging wells; but not successful; and it tried to build that dam again though we are not using it yet for drinking; so, it may not okay to blame the government too as it is standing along our side.

**I: Is there malaria problem here?**

**P**: Yes there is malaria.

**I: So, what is being done to prevent this malaria; and who is being affected more?**

**P**: Even though there is malaria, one, there is free medication at our health post; there is tablet given with no any coin; we have three HEWs here with us that they provide us the tablets for free. Next to this, it was previously quitted; but now it came and we are given Zanzira (she means ITN) just in this month. It was in the previous times that children used to be hurt; now there is vaccination and no child is hurt or die; they also receive medication for malaria. But in the case of Rezina village, there are more malaria cases; because, we have spray for the malaria, we are given that ITN, and there is also medication; but at Rezina, it was assumed that it was better than us in the case of malaria; but they are worse than us in the case of malaria currently. And Misaza village is a bit fine like our village; but the problem is with Rezina village.

**I: What are the reasons then that Rezina village is specifically attacked by malaria cases?**

**P**: The reason is that it is not being sprayed with the anti-malaria sprays; because if it was sprayed and if the swamps were filled during September month, the mosquitoes would to be lost and that malaria would to be controlled; and again the stay out at somewhere desert areas so then they become attacked by malaria case. Anyways, even though it is not much malaria case like in the previous times; there is still malaria; for example, I was caught malaria on September.

**I: Why were you caught by malaria if you use that of ITN?**

**P**: It is because I travel to many places including that of Rezina; I go to Yechilla too and there are much mosquitoes there. But when I am at my home I use ITN very well; and my two children in Yechilla have their own ITNs now.

**I: Does all the community uses ITN here; and those pregnant and lactating mothers too?**

**P**: We thought it uses; but if there on adequate, the priority is given to the pregnant and lactating mothers to use; yet, the government is providing us for each of us individually; so, we use all individually that ITN. But when I went to Yechilla, I was left to use that ITN but when I see a mother with her baby not to have it; I left it for them and the mosquitoes bit me there; that was why, I brought the malaria; and I came back home, here I felt headache and they were let me be checked; thus, the malaria found and I took the tablets; now I am fine. I am fine now; but it is not because I am educated; disease comes to all!

**I: In addition to the Faffa they get when they are thin; is there any Targeted supplementary feeding for pregnant and lactating mothers that they get it here at your Kebelle?**

**P**: That is it the Faffa if they are thin; otherwise, there is no any special help given for them being it by the woreda or any other.

**I: So, from all the interventions undergone by the kebelle and government and any other bodies to make the mothers and adolescent girls be healthy; which interventions do you think are most successful as per your own judgment?**

**P**: Ehhh, what could be that; what could be that 100% successful; anyways, that diet is being affected by drought; so how can I say it is successful; but the good thing that I can say for us and it has to continue is that a mother giving life should never to lose her life; the car has to take them and get them back home; they have to have medical care follow up, a child has to be vaccinated; all these things has to be successful and I expect them to be successful too.

**I: Okay, you expect these services to be successful, how about what are currently successful interventions?**

**P**: Currently, the pregnancies follow-up and that of child vaccinations are successful; as it has never been said that a child is died of Shilimie (she means measles); now in the 2010, let 1000 or 5000 babies be born, the government is supporting them to grow well; and they will grow all; no problem will happen to them; because, immediately after they are born, they start vaccination and take medications; so, there is no baby who is said to be hurt; thus, this is successful.

**I: Very good; does the ambulance brings you back home?**

**P**: Yes, it brings us back to our home. Here it is decided for us by the government; for example, I took three to four laboring mothers to woreda; then after their delivery, they brought us back home with our newborns to our home. They may give priority for the one who is laboring; but after taking her; they do it for others too; but the drivers may get tired for themselves; otherwise, the government is doing good for us in this case.

**Section 3: Perceived needs of women for relevant services during pregnancy**

**I: Do you think there are special things that pregnant and lactating mothers and those adolescent girls should get at your community?**

**P**: As to me that can I say it is needed to mothers and those adolescent girls is that of education; they need education repeatedly; otherwise, saying they have to eat something or another something is not worth to me ; if they learn, they will eat it; that is it; what they need is that education. Education is boundless deposit that they have to learn it two to three times. This is what I understand!

**NB**. Other points in this section like the diets for mothers and adolescent girls are addressed in the above sections in detail.

**Section 4: Other interventions that improve maternal and adolescent girls’ nutrition**

**I: Do pregnant and lactating mothers and adolescent girls go for community health day’s services; and what services do they get there?**

**P**: They are vaccinated; and those pregnant and lactating mothers are also measured (she means the MUAC measurement); if she is lactating of less than six months, she is measured; if six months are completed, that is it; the baby is child; she will not be measured. If she is pregnant; starting from her time of pregnancy up to she brings up the baby in to his six months, she is regularly measured. They are all measured; there is no quota for measurement; thus, if they are found to be thin, they will get the service (she means the Faffa); then also they will be measured in the next month; and they will get the service too if they are still thin; it goes like this kind of round. So, there is no quota to measure the pregnant and lactating mothers; they all are measured when they come.

**I: Where are they measured?**

**P**: They are measured on every month being gone to their villages; for example, in this village they are measured in every Saint Gebriel day (Saint Gabriel) at the19th of the month (EC counting); and at Misaza village on Saint Michael day (at the 12^th^ day of the month in EC counting); and at Rezina village at the 7^th^ day of the month during the day of Silassie (the day of the Father, Holly spirit, and the Son; all the three together in EC counting); then they stay the day being measured.

**I: Do you think this community health day service is necessary?**

**P**: Yes we believe on it!

**I: Why it is necessary being undergone on each month at each village?**

**P**: For example now, there is a complaint by the farmers that how will I go to that kebelle with this all sunny condition; I am in difficulty and so on they say; thus, these two or three HEWs workers go to Rezina and undergo measurement there; and those who are okay will know that they are normal and will reduce their efforts from travel and it will also reduce their hesitation to come to the kebelle either to get the service or not; and also they will get their vaccination. Another thing is that gathering all of them in this kebelle and doing that entire job will not be fast; so, that one reduces the load of work and it facilitates the speed of the work; it gives the opportunity of job division. So, this is necessary; if they are measured at the Silassie 7^th^ day at Rzina, if they are measured at the Saint Michael day at Misaza, and if they are masured and vaccinated at Saint Gabriel day on 19^th^ here, this will be nearer service to the farmers; and also it will reduce that many hours of travel of the mothers to the kebelle; and also it will facilitate the speed of the work to be achieved in each village; rather than coming more than 200 people here and being measured. So, this is not hurt, rather serving the community to the nearest distance.

I**: You told me that mothers are safety net beneficiaries; so may there pregnant and lactating mothers there?**

**P**: Yes, there are.

**I: So, are they involved in the public work or how is that here?**

**P**: No, they don’t work; they eat for free.

**I: So, what do they do with that free time?**

**P**: It is allowed by the government that they have to be free so that they will not be hurt and devastated for themselves and their children too; from the time the she is pregnant up to her child grows.

**I: What is the time allowed the time to them?**

**P**: The pregnancy is clear as it s identified by the health worker; and it starts from that time; but for the lactating mother, oh; did I forget it…?!

**I: It is okay.**

**P**: But if the pregnant is identified even at her three to fourth month, she will take the rest.

**I: So, is that important for them; what does it benefit them?**

**P**: It benefit them; one, they would to pick stone beyond their capacity; they would to dig holes beyond their capacity; so, as it is allowed to them by the government due to the good administration, so that they will not deliver bad baby; they will not be distorted, and will not be hurt; which is then allowed by the government and accepted by the community for the benefit of these mothers; so we don’t even pick up things equally; so as it is allowed by the government, we also tell and follow that the pregnant and lactating mothers should not work.

**I: So, do they pass the rest at their homes or use it for other activities?**

**P**: That is, they lead their home and they try to have exercise; it is for the sake of not to be exposed to sun not to pick up stones and not to dig holes.

**I: Do they use it for medical checkups?**

**P**: That medical care is, they go either per a month or per two month once; they don’t go in every day; but that work is in every day! But in the case of medical examination, it is as per the health worker’s appointment that either he appoints them to come back at second month or at one month; otherwise, they don’t go in every day because they have that rest!

**I: I laughed much pleasantly, the way how she expressed it confidently; and she helped me with her economized laughing; it was near smiling!**

**Section 5: Understanding perceptions of age at first birth and birth spacing**

**I: You have told me in detail that early marriage is very prohibited and also you have told me that have successful experience up to going to Temben court in saving girls who were victims of that early marriage; so, who did promote this message to the community to be as such successful?**

**P**: The government; step by step from higher to lower levels, it teaches us; form region to zone form zone to woreda and from woreda to kebelle; it came step by step down; the woreda give us, and to them they are given by their higher levels; this is because it comes from higher level approved by council or others as a main plan to us; we do it as an order then.

**I: Do you think it has to be done beyond this about this issue?**

**P**: What, nothing is left about it; rather, it has to be continued as it is.

**I: How about that of birth spacing; how many years should it be between births do you think; and is there promotion about it here?**

**P**: Yeah, the farmers still have problem in understanding it yet; you can see, here are mothers who are pregnant but have children on their back too; all everyone is not the same. One, the husband and wife talk and understand it and should do it then; for example, if she gives birth after five to six years, it is good; but giving birth at three or two years is not okay; there are who are pregnant and also who gave birth; but for me, you have to breastfeed a child for three years; then you take rest of additional two years; then you become pregnant after five years; because your body becomes strong. Otherwise, you were breastfeeding a child; then you got pregnant when the baby begins toddling; so, when do you rest?

**I: What could be the consequence for the mother if the birth spacing is short?**

**P**: If you give birth soon and soon, one, your life will be distorted; and again, your womb will be loose; anyways, having baby over another baby does have many problems. Otherwise, if the baby is grown; if you breastfeed him well, his mind will be opened; he will be pleased; and when he speaks at his age of three to four years, it will like those of adults; he will be happy; as he has not anyone below him that can let him be greedy, he goes be pleased; and his mind becomes stronger. But if you have one child hug here and one child put there, that baby will cry all the day; when will, he walk erect? For example, if you see my son who is at grade3rd now, he looks like he is at his thirteen or fourteen years of age; but his is at his tenth years age now; you would to see him if he passes via this road; he reaches me; he plays and fights with me when we go together; this is because he has not child below him.

**I: Are your children clever at their schooling?**

**P**: Yes, they are clever; specially that young one at grade 3^rd^ is cleaver; but I have a doubt about the grade 7^th^ student. If a child is happy at his home, it is good to the schooling too. For example in my case, my husband brought me food to the school if I have meeting at the kebelle and if I have to enter to class directly from that meeting without going home.

**I: So, do you think a husband has an influence to the family; especially to the success of female, form the experience you have?**

**P**: It has a lot! All are not equal; let alone to send them to school, there are husbands who prevent their wives not to go to their neighbors. There are still who reaches up to divorce due to the hit them torture their wife all the day; we follow them and we tell them that they have to be taken into police station. Anyways, there will present two to three per hundred; otherwise, most of the community is understanding; due to the good administration extension; so, all is not the same.

**I: Do you think there may present other opportunities that better help for the success of early marriage prohibition and good birth spacing practice here at your kebelle?**

**P**: What can present other opportunity beyond education; the determinant thing is that education; if you educate that you will be hurt, your child will be in problem, if your daughter marries at early age and if she is not or he is not examined about their health status, all these things will be devastating; so, what can you do beyond teaching this; what can we put another option? There is no another option; but this education has to be very repeatedly; we have not to wait only that January; we have to also see during summer and autumn seasons that we may miss the girls being married secretly.

**I: Why they are married secretly (being hide)?**

**P**: Because they are not educated! It is not the girls that are hiding for marriage; their parents fear not to be seen by unions and others; so then, they do it secretly during holidays or other remembrance days pretending that as if it was the celebration for the tribute not for the marriage. Otherwise, the girl doesn’t know what is going on at her; she is kid!

**Section 6: Understanding communication and information sources**

**I: What kind of groups or opportunities would you expect to present at your kebelle to better discuss about the nutrition of mothers and adolescent girls here? because, if a plant is to give us fruits it has to be cared well and has to get all what it needs; the same is true for adolescent girls; they have to be cared now; to be the future mothers and to let continue the future generation; so, what opportunities do you expect to present for better maternal nutrition and health improvement here?**

**P**: As per my understanding, using the lessons given by the development armies and HEWs, we have to use what is all available at our homes; rather than taking it to markets; this is what I can understand. But beyond this, there are individuals who take the food as it is; for example, in the pace called Dagu, all pregnant mothers being thick or thin take the Faffa; but here in our kebelle, it is given for thin alone; yet, this food may be helpful for them; but, it would be better if they able to use the food sources that they have at their homes; they have to be taught how to use their own foods; there are even the rich mothers who are thin and taking Faffa.

**I: Why those rich mothers are thin?**

**P**: May be they go here and there; they are more thin; they have to be told that they should not take their sweet foods to market; rather, they have to let eat those pregnant and lactating mothers and those children too; this has to be improved in this way. This is what I understand; they have to learn; they have to know platforms; they have not to take the sweet foods into market; they have to eat what is available at home being it butter that or oil; for example, I may buy that of fifteen or sixteen birr of oil; but I have not to think that I can re-sell one small bottle of it for 100birr; I have to eat it for myself; it will give me energy; even that egg has to be consumed at home; they have to consider themselves as if they were sick and they have to eat all available at home; but have not to be expectants; that Faffa may be good if given to all pregnant mothers; yet, it is better if they use what is available at their home; and also the males has to be informed to be involved in helping the mothers; they have to care the pregnant mother; they have to help her home activities and all what she wants; and also the children should help her; thus, she will be happy mentally and she will be then thick enough; it is not only food that lets individual be thick; it is that pleasure; if you are pleased, you can be thick enough. In addition to this, if they are supported by development armies and HEWs, they will bring change; otherwise, saying that let’s give them this, let them be given that and so on; what will be helping them?

**I: So, are they given such kind of education?**

**P**: Yes, they are being given; for example, I have worked as development army for the whole ten years; and now I am centralizing the whole kebelle as a women union head; but I delegate another development army in my place; I support for all being in the center; and I used to praised many times that of dresses and exercise books that I still learn with it; I used to praised at kelbelle, school and even woreda level; we used to have discussion in every two weeks with the group and in every one week with in the five networks; and the woreda used to visit that group too. Whatever so, if you let others understand you, they will understand you; and this is good; now it is being said that Tiemtey’s group has to be asked.

**I: May be that we didn’t raised it earlier; mat there be the case of goiter and night blindness (Himma) here in this kebelle?**

**P**: We have one lady that has goiter; she used to give birth during that period; but now she quitted giving birth. After that, the HEWs told us that she has to use that of iodized salt not to increase its size; then at our group no bars of salt is used totally.

**I: Is that only your group that uses the iodized salt or what?**

**P**: As far as I know, most of the community uses that salt; that of in plastic packed salt from shop.

**I: Where is that found?**

**P**: It is found here at Hagoss shop;

**I: How much is its price; is that accessible?**

**P**: I myself bought paying ten birr per one pack; I took two; it is nine birr for one there at Yechilla; but here ten birr.

**I: So, for how much time will it serve you?**

**P**: It is as per your family and as per your feeding; for example, in this week, I took two packs; there may someone who can use it for a month, but I don’t think it will stay (she laughed). On pack is one kilogram and it is ten birr priced.

**Section 7: Additional remarks**

**I: If there is anything left that we didn’t raise it about the maternal and adolescent girls health improvement particularly at their nutritional conditions; let me give you the chance?**

**P**: What then; that is it; it is needed to be taught; especially those adolescent girls only look at child birth; they don’t see what problem will happen after the child; they only need to have more folks; they say that they need not to deliver only two or three; but more; yet, they have to know that they have to learn and they have to give birth by plan saying that I have to grow him well and I will let him learn. If she decides to deliver three children, she has to let them learn; she has to let them dress; otherwise if she has not capacity to do so, she has to leave giving birth by delivering only one; she was one previously; so, she has to let him grow well; next to that seeing her capacity and her home, she can give birth. But giving birth baby over baby saying that they will be grown by government has a problem.

**I: Very nice! And thank you heartily. You taught me a lot and you deserve what you are doing now, and we expect a lot from you for the future too; and long live to you.**

**P: Okay thank you.**

**Summary (home take messages)**

**Section 1: Common maternal (pregnant women, lactating women and adolescent girls) nutrition problems in the community.**

Adult based learning at Felege Hiwot kebelle is helping the mothers; one, to give birth at health facility; second, to have health checkups and to know about their health status; that is, the HEWs teach them to go to medical care and let check about the position of their fetus if they are pregnant; because, if they don’t do that, they may be hurt.

**Section 2: Barriers to access and utilization of nutrition services**

The barrier that hinders them not to go to the health and not to get the health service is that because they are not educated and lack of understanding.

**Section 3: Perceived needs of women for relevant services during pregnancy**

They need education repeatedly; otherwise, saying they have to eat something or another something is not worth to say; if they learn, they will eat it; that is it; what they need is that education.

**Section 4: Other interventions that improve maternal and adolescent girls’ nutrition**

Community health day service is important for reducing that of many hours of travel of the mothers to the kebelle; and also it will facilitate the speed of the work to be achieved in each village.

**Section 5: Understanding perceptions of age at first birth and birth spacing**

If there is enough birth spacing, the baby is grown well; if you breastfeed him well, his mind will be opened; he will be pleased; and when he speaks at his age of three to four years, it will like those of adults; he will be happy; as he has not anyone below him that can let him be greedy, he goes be pleased; and his mind becomes stronger.

**Section 6: Understanding communication and information sources**

As per my understanding, using the lessons given by the development armies and HEWs, we have to use what is all available at our homes; rather than taking it to markets.

**Section 7: Additional remarks**

The adolescent girls have to know that they have to learn and they have to give birth by plan saying that I have to grow him well and I will let him learn.
