# Supplementary material for: Barriers to utilize nutrition interventions among lactating women in rural communities of Tigray, northern Ethiopia: An exploratory study
Source: PLoS One. 2021 Apr 30;16(4):e0250696. doi: 10.1371/journal.pone.0250696 (PMC8087028; doi:10.1371/journal.pone.0250696)
Supplement: S2 File — (ZIP) [file pone.0250696.s002.zip › S2_File.Doc/Community level Key informants/091_IDI_Kebele leader_Felege Hiwot kebele_Tankua Abergel.docx]

**In-depth interview guide for with experts** using the Guide for Nutrition focal persons (**Tool A**)

**Introduction:**

Hello my name is Haftay. I am from Mekelle University. Thank you for taking time to speak with us today. We are doing a research on factors that influence the nutrition of mothers and adolescent girls in collaboration with the regional health bureau and UNICEF. Your participation is very valuable. The things that you tell us will be used to improve nutrition programs and services for women and adolescent in the region and in the country. Your names will not share when we report our results.

However, I will record the discussion using and audio tape recorder so that we can capture all the ideas that are shared. I have several questions to ask you that we have prepared in advance, and we will ask you to say what you think about each question. To ensure the privacy of everyone here, we ask you not to repeat what to discuss outside of this group. The discussion will last for 1-2 hours. Do you have any question before I begin? If you think of any question as we proceed, please feel free to let me know. If it is all right with all of you, I will run on the tape record now.

**Section A: Interview details**

1. **Zone**: Southern
2. **Woreda**: Tanqua Abergele
3. **Kebele**: Felege Hiwot
4. **Name of key Informant**: Birhanu Hagos
5. **Institution of key informant**: Kebele leader
6. **Interviewer’s name**: Haftay Berhane
7. **Date of interview**: 16/11/2017
8. **Interview starting time**: 11:05 AM
9. **Interview end time**: 12:30 AM

**Section B: interviewee professional information**

1. **Gender**: Male
2. **Age**: 29 years
3. **Highest level of completed education:** High school
4. **Current job/Position:** Kebele leader
5. **How long have you been in the current Job/position:** 02 years

**Key:**

**I**: Interview

**P**: Participant

**Fafa**:

**Section 1: Common maternal (pregnant and lactating women), and adolescent girls` nutrition problems in the community.**

**I**: What do women including pregnant and lactating, and adolescent girls do to stay healthy? What do they do practically?

**P**: Women eat balanced diet, visit the health facility for antenatal and postnatal care, do physical activity until they give birth as they should not always sit. The adolescent girls aged up to eighteen are in school. They are advised not to be exposed for harmful thing like HIV/AIDS. Adolescent girl is exposed to many things and adolescent girls are counselled by the health extension workers.

**I**: What about those out school adolescent girls?

**P**: Adolescent girls in this area are not still changed and they get married at the age of 15 and 16 years. These girls and their partner do not even do pre-marriage screening. The marriage is dependent on number of cattle and other wealth. Here, this is one of the tasks left undone to align with the governmental policy.

**I**: What is the reason why the pre-marriage screening is not being done?

**P**: The community is not changed and there is lack of awareness. There is understanding that a girl is virgin and no need of screening for any disease. There is lack of awareness as sharp materials can transmit disease. There is assumption that the girl is virgin and the partner is also do not do any sex because he is waiting for her. It is vital to do screening to prevent disease occurrence but still this is not done.

**I**: What do a pregnant woman do practically to keep her health or not to be sick here in this community?

**P**: One is the pregnant women should not do heavy work example like governmental work, food preparation. The second one is eating balanced diet as I have told you before, doing physical exercise. As to me this are the things that a pregnant woman does to stay healthy.

**I**: You were telling me about balanced diet. Do pregnant women in this community get balanced diet?

**P**: This is not practical. We were taking about what should be done but practically we are taking about it being at zero percent. If a woman in this community eats injera with shiro in the morning, she will eat the same in the lunch time and dinner time. We were talking about the policy. There are even women who do not get this shiro stew, just they eat dry injera.

I: My intention is to know what do these women including pregnant and lactating as well as the adolescent girls. In fact, you have mentioned some but I want to know more about this.

**P**: If a mother is lactating she will eat porridge with butter until one or more months. If the mother is pregnant, as I have told you before, she should take balanced diet. Anyways, I have already concluded that we do not do activities related to this. But for lactating women eats a porridge made up of wheat flour or sorghum flour with butter.

**I**: In your opinion, what are the common nutrition problems in the community for women and adolescent girls?

**P**: Since there are many diseases, nutrition related problems are mostly occurred in older adults. If the elder has shortage of food, there will be swelling. This what it is happening even when we are observing in person. This area is area of drought. It has been before ten years that this area repeatedly affected by drought. If the individual is hard enough, he/she may feed him/her self by engaged in work activities but the swelling is commonly seen in the females which are family leaders.

**I**: Where do you think the swelling occurs?

**P**: The swelling starts from her leg to this (showing his abdomen with his right hand). It creases its size then after. This is typically caused by shortage of food.

**I**: In which women does the swelling commonly observed? Pregnant, lactating or adolescent girl or all?

**P**: This mostly seen in pregnant women and older adults.

**I**: How do you think this shortage of food affects the woman`s health?

**P**: One, there will be anemia. If anemia is then occurred, the woman will die at delivery. If the mother is not getting service at the antenatal and postnatal period, there will be death.

**I**: You were telling me about anemia. How do you describe the occurrence of this anemia in pregnant, lactating or adolescent girl?

**P**: we can explain anemia, one, there is active engagement in hard work. If you get tired and you do not eat, then anemia will occur as there is no food to replace the blood. You will not get balanced diet. There will be shortage of food in the morning or afternoon and anemia will occur.

**I**: Have you ever seen a pregnant, lactating or adolescent with anemia?

**P**: The presence of anemia is confirmed by the health professionals. As leaders, we may hear the information that a mother is known to have anemia and ordered to eat some kind of food. It is difficult to guess the presence of anemia physically. This is because, anemia can occur in thin or fat individuals.

**I**: What about goiter, night blindness? Do these diseases commonly seen in the community?

**P**: Goiter is not seen in our area. We know it but, it is not commonly seen in our area. Night blindness is seen in older men or women with age of greater than 65 years. This night blindness can occur in human and animal after the sun sets and this is because of this is a lowland area.

**I**: What do you think is the cause of these diseases mentioned like anemia, goiter and night blindness?

**P**: I can say the main cause of these diseases is not getting balanced diet. Goiter is caused by one, not getting balanced diet and second, is eating non-hygienic foods like eating a meat dead goat.

**I**: What do you think should a food contain to be called as balanced diet?

**P**: One the food should contain meat and second, it should contain food items that can give energy like lentils, check pea. If the mother eats lentil in the morning, she must eat the three including salad, swiss chard and meat. This is what a balanced diet is. But is shiro in the morning and afternoon, I can consider this as unbalanced diet.

**I**: Is it common to see severely wasted pregnant, lactating or adolescent?

**P**: Severe wasting is common but it may or may not be due to disease. If an individual is very thin and there is no enough food at home, we consider this as because of shortage of food but if there is enough food at home, it may be because of other chronic illness.

**I**: Is it common to see overweight pregnant, lactating or adolescent girl?

**P**: [laughing]… There is no one with overweigh. Everybody here is underweight.

**I**: Do you think overweight could be liked with nutrition?

**P**: I do not know. It could be related to nutrition. If someone is eating delicious food every day and lives in a good environment, there could be increased body weight. But there is nobody with overweight in this area.

**I**: Do you think diet related non-communicable diseases like hypertension, diabetes mellitus common in the community?

**P**: The most common diseases caused by shortage of food are waterborne diseases like cholera, and eating non-hygienic food is also the same. Most diseases are caused by drinking unclean water source like from lakes, ponds. They are manifested by diarrhea.

**I**: What about hypertension and diabetes mellitus? Are they common here?

**P**: I am not sure about hypertension but diabetes is common. Even if I do not know the cause of diabetes, I think it caused by repeated consumption of sugar and its prevention could also be restricting from sugar. However, there are individuals with diabetes mellitus.

**I**: You were telling me as there is shortage of food, in what situation do you think this happened?

**P**: There is different evaluation of this food shortage every year. The main problem here is the lack of irrigation because there is no water. You can also see the water we get from rain during summer times is nil. In the year before last year, there was no harvest at all, last year there was somewhat better harvest which could be assumed up to 150 kg. Last year, the community also can feed animals without going to other areas for searching water and animal food. This year, you can also see the condition, is different from the year before last year only with the presence of straw for animals. The crops were hanged by an insect and there is no yield at all. No yield has been collected. There is only few yield on teff which could not be affected by the insect. This kebele is repeatedly affected by drought.

**I**: What other problems do women in this community suffer from?

**P**: Anyways, as we are living in lowlands, there is screening but only 50-100 individuals are benefited in the form of fafa with or without edible oil. This is supported by NGOs. However, I believe, there should be food support to most poor. After screening, whether the individual is thin or fat, many individuals are not benefited declared as not eligible. The good thing is, the screening shows the health status of the individual however, the community is not trusting the measurement and complaining that lucky individuals are being benefited.

I: Is this kebele benefited from safety net program?

**P**: Yes.

**I**: Who do you think are eligible for safety net program?

P: The priority is level of poverty. If an individual is poor with no cattle and able to perform a given task, s/he will be eligible for safety net program. If an individual is with two oxen and one cow, s/he will be eligible for emergency aid if there is drought. Therefore, this community especially felegehiwot is always under food aid.

**I**: What about pregnant and lactating mothers?

**P**: If the pregnant or lactating woman is poor, she will be benefited otherwise she will not. But If there is drought, she will be benefited from the emergency food aid.

**I**: Will the mother be benefited from the safety net program because of she is pregnant or lactating?

**P**: The only criteria is property. A mother will not be included in the safety net program because she is pregnant or lactating. If a mother is pregnant and eligible for safety net program, we only give her rest.

**I**: How many months will a pregnant woman in safety net program be freed from the program based work?

**P**: It is twelve months from the time of pregnancy. The rest is one year. After delivery, she will be given rest up to 6 months.

**Section 2: Barriers to access and utilization of nutrition services**

**I**: What kinds of nutrition interventions are in place to improve health of the pregnant in this kebele or wereda?

**P**: I do not think there are nutrition related interventions for mothers. There is nothing special done to pregnant and lactating women on nutrition related intervention in our wereda or kebele. We have many kebeles in our wereda but I am taking about wereda considering the other kebeles are getting similar intervention with ours. The only special thing is the provision of fafa. Few mothers are supported with fafa.

**I**: Do women advised to visit health facilities for check-up and services during pregnancy?

**P**: Women in these days are advised to go and check in health facilities. These days, there should no mother to deliver at home. The moto of our late prime minister Meles, ` No mother should die while giving life` is being implemented practically. Mothers are doing antenatal care every three months and postnatal care. There may be few mothers who did not go for check-ups but if they near to deliver, they will use the ambulance to go to the health facility for delivery. We have three ambulances to serve for three kebeles.

**I**: What are the reasons that hinder mothers not to go health facilities?

**P**: There no obstacle but she is having lack of awareness. If the mother is not changed, it is difficult. They believed that St. Virgin marry is in their home and no need to go to health institution. They said, God knows. This is seen mostly in mothers whose husbands are priests. The main agreement is as there is Virgin marry anywhere, at home or in the health facility. If mothers meet with health professionals, they will not be risk of bleeding and if needed caesarean section will be employed. Most of the community is aware of this issue.

**I**: What are the services given in the health facility for pregnant, lactating and adolescent girls?

**P**: The services include free transport service with ambulance, free check-up. If the pregnant is near to deliver, she will go to her home with ambulance. But if the mother is around six months pregnancy and does not get the ambulance service, she will come with public transport. For the lactating mother, they will directly take her with ambulance and after she get the required service in the health facility, they will bring her home with the ambulance. The service is for free. The ambulance is there for mothers to give transport service to and from the health facility for antenatal care and postnatal care.

**I**: Do you think that women receive advice on the need to get extra meal during pregnancy and lactation?

**P**: Yes. They are getting the advice because the health extension workers are always with them but the problem is the utilization. Therefore, more works are left to be done in this regard.

I: In your opinion, what could be the advantage of taking this extra meal?

P: If they take extra meal, there will not be any disease. They will get energy and the child`s health is also being checked as they are visiting the health facility. Therefore, the mother will not develop any disease during pregnancy or lactation. If the mother is having shortage of food or if she is not getting extra food that she must take, she will get sick.

**I**: Are Women getting counselling for food diversification during pregnancy and lactation?

**P**: Yes. They are getting advice but the problem is practical application of this advice.

**I**: What type of crops are growing here that we can use them for food diversification?

**P**: One is meat since we have animals for food, the second is the dairy products like better, milk, and milk products. There is also lentil growing here but the oil and pea will be bought from the market.

**I**: Is there advice for the need to use iodized salt?

**P**: Almost all the community is utilizing iodine salt. This is because there are counselling sessions arranged by the government to promote the use of iodine salt to prevent goiter. None is using the bar of salt because it is not hygiene. Therefore, use of bar of salt is nil and all are using iodine salt. They are using iodine one: because it is packed and may not have hygiene problem, second: it contains the mineral iodine and hence can have health benefit.

**I**: You were telling as there is shortage of water in this area. Is there advice on nutrition sensitive agriculture such as home gardening in collaboration with the agriculture?

**P**: Yes. I have told you as there is no water source here but the community is trying to plant the garden at home. However, the garden will not grow because of the warm nature of the area and lack of follow up. If you see specially this school (indicating with his finger), the community has tried to plant it many plants and many times but you can see there is nothing. The other problems of this area are the presence of termites and the shortage of water. The community will bring water for drink from four hours on-foot travel. Therefore, there is also selfishness to use the water only for drink.

**I**: Are women girls getting advice on nutrition sensitive agriculture such as home gardening? Do they try to grow carrot, potato and pepper?

**P**: There are few women who grow pepper and tomato but potato and carrot cannot grow in this area. There is no even harvest of these for market purpose. This indicates that it could be possible to grow such fruits if water is available.

**I**: You have tried to mention the importance of personal and environmental hygiene to prevent diseases like acute watery diarrhea. Are Women getting advice on sanitation and hygiene?

**P**: Anyways, they are told in different meetings and the only challenge is to bring it to practical. Since a woman is very crucial for hygiene and sanitation as she is handling all utensils for food preparation. She is the one who washed it or not. There is repeated education for woman to keep the environment clean including latrine.

**I**: Is there an advice given to lactating woman on how to handle her kid, what soap she should use and others?

**P**: I only know to keep their hygiene but not what type of they should use. The professionals are telling them whether it is life boy or medicam (type of soaps).

**I**: How are going to relate the lack of water with sanitation and hygiene?

**P**: In fact, there is no water but we do not have option. We are talking on what we have at hand. If a mother is pregnant and if she does not have somebody to bring water, as community, we advise the neighbours to bring her. Even if the required sanitation is not applied, she may get for drink and washing certain essential goods.

**I**: What about for adolescents? Is there advice given to adolescent girls on menstrual hygiene and related things?

**P**: The professionals are providing them advice since it is natural. There is repeated counselling on menstrual hygiene and family planning use.

**I**: Is malaria common in this area?

**P**: I think the source of malaria is here because it is a lowland. There is repeated malaria attack especially from July to end of November. In these months, there will be large waiting lists in health facilities. The cause of this disease is milk consumption and hunger. There is practice of using insecticide treated bed nets. However, most children have gone for herding cattle and when they pass the whole night there, they could be attacked.

**I**: Do you think there is sufficient supply of bed nets for the community?

**P**: There is supply of bed nets. In fact, the supply is not every year and there was no supply in the past two years. But this year, sufficient supply of bed nets is provided at this month. However, it would have been better if it were provided before two months.

**I**: You were telling me that malaria is common starting from the month of July. I can understand from this that it would be good if it was supplied on the month of July or before. Why do you think is the supply of bed nets delayed?

**P**: The obstacle is the miscommunication between the professional with the respected authorities. They must have supplied the required number of bed nets at the right time. After the bed net is arrived at wereda, it could be because of shortage of transport or another reason. But, it was two weeks before its arrival at our kebele while we heard the arrival of the bed nets at the wereda. The main reason is the professionals were not committed to understand the problem of the community and bring the bed nets in communication with the responsible bodies, be it zone or wereda. If it comes here, it will be distributed on time and the problem is at the higher officials. It may be stored for some time if only the farmers are not informed to come and take the bed nets. If this happens, we usually promote and communicate the farmers to take their quota. Therefore, the problem is from higher bodies.

**I**: Are women getting advice on the need to use insecticide treated bed nets?

**P**: Yes. Especially lactating women are advised given due attention because the child should not be exposed to mosquitoes and other flies. She is given repeated advice to use the bed nets alone. The pregnant should also prevent herself by using bed nets. Not only pregnant but the children should also prevent themselves from mosquito bites.

**I**: Who is advising them?

**P**: This advice is primarily given by health professionals. The political leader also can provide advice in detail as s/he is leading the community.

**I**: How do you see the practical application of utilization of insecticide treated bed nets?

**P**: There is a gap in utilization of the bed nets. Some of the users are using the bed nets for roping, for covering pepper not to be eaten by mouse. But, most of them do this after using for mosquito prevention for some time. However, used bed nets must be burnt separately. Anyhow, not all individuals are using bed nets and there is lack of awareness.

**I**: What do you think are the barriers that hinder the use of the insecticide treated bed nets? What are the main reasons for an individual not use bed nets after the supply and advice on utilization of these bed nets?

**P**: It is lack of awareness. There is no energy needed; it is for free and easy to use. I think the main reason for not using is lack of awareness.

**I**: What could be the solution then? You have told me that advice is given on how to use and why to use it. You have also told me as there are patients who are sick out of the community which shows the presence of the disease. How do you think should the awareness is created?

**P**: It is by providing continuous advice and counselling on it. There should be repeated counselling with the farmers on the advantage of using bed nets including the prevention of malaria and others, minimizes additional cost that could occur as the result of illness. Anyhow, there should be repeated counselling until things are properly done.

**I**: Do you think everybody here is aware as mosquito can transmit malaria?

**P**: Yes. The farmers are aware of the mode of transmission of malaria which is by mosquito bite. But, there is a gap on practical use of bed nets after they are advised to use the bed nets to prevent the mosquito bites.

**I**: Are women and adolescent girls getting deworming services?

**P**: Yes. Deworming was given especially when there was outbreak of acute watery diarrhea. However, the supply of drugs was not enough. Since the community was afraid of the acute watery diarrhea, it was using these deworming properly. But the supply was not enough.

**I**: How was the deworming given? Who was eligible to take the deworming?

**P**: Firstly, it was given by the health professionals and it was also given by water resource experts. It was given to the community in a meeting either by development army or in religious areas.

**I**: How do the out-school adolescent girls receive this service?

**P**: There is no special service for the adolescent girl. She will be using what others are getting. Therefore, the advice is given to the adolescent to use services like bed nets equally with the others.

**I**: You may get in school adolescent girls in their school, pregnant and lactating mothers may be in antenatal and postnatal care but where do you think you will get the out-school girls?

**P**: These days, there is networking and advice is given by the network. They could meet but they are not doing it. They do not come to meeting is someone needs to provide counselling on health related or agriculture. Sometimes these girls can come to the meeting but in most of the time, they receive messages from their mothers.

**I**: Why do you think is deworming necessary for women in this community?

**P**: The advantage of deworming is not only for women. But, if the mother is ill, she will lose her life. If a mother loses her life, the whole family will be a in dark season. That is why mothers are getting repeated advice and deworming services.

**I**: Do you think women in this community are benefited from Targeted supplementary feeding?

**P**: It is still not yet. If a woman is pregnant, she eats what she is having at home with her husband. She does not get any additional food because of her pregnancy. She is even more maltreated. She will be busy to feed her husband and her children. There is a time where she is not even get the normal share she should get.

**I**: What do you think the obstacles?

**P**: It is from the mothers’ side. There is shyness. Not only they do not eat special or extra meal but mothers may not eat before their husbands come to home. Husbands in this lowland area are not cheerful enough to help their pregnant wives considering as the pregnant mother need special and extra meal, which may be related to income.

**I**: Outside the shortage of food and drought, what other factors being lowland will contribute to this issue?

**P**: Education has been started in this area about ten years back. There is sand road right now but there were no infrastructures. Most people here were involved in herding animals. But I think the education will continue to bring behavioural change. Anyways, there is nothing difference from others except the things I mentioned. The sun is similar to all kebeles of our wereda.

**I**: Which of the interventions listed above like a mother should get advised to visit HFs for check-up and services during pregnancy, receive advice on the need to get extra meal during pregnancy and lactation, get counselling for food diversification during pregnancy and lactation, get advice on home gardening, get advice on personal and environmental sanitation, get advice on the need to use insecticide treated bed nets, get deworming services, do you think is most important for women and girls?

**P**: Anyhow, if women can use the services, all are important. Using bed nets and home gardening is important especially for lactating women.

**I**: If they are important, what are the barriers that prevent a pregnant woman from using the interventions mentioned above?

**P**: In general, the main challenge is in applying and using the interventions. The other is the income of the community is low. For a mother not to go for antenatal and postnatal care, the main barrier is lack of awareness and lack of commitment. Most mothers do not go because they say `God knows`. Husbands were also not interested to send their wives to health facilities for check-ups. But these days, husbands are becoming supporting in motivating their wives to visit health facilities for check-ups.

**I**: What about extra meal?

**P**: In extra meal, the husband will not bring and even if there is extra meal, the husband will take it first. Extra meal is not given because of her pregnancy or because she is girl. The barrier here is the lack of awareness. The community consider that there is no need for extra meal to be given to pregnant and lactating mothers.

**I**: If meal is prepared, who do you think will the meal first, the male or female?

**P**: This is known. The first to eat the meal is the male. Male is given first chance to eat because it is he the one who will go to herd the animals. He will even go first to school than a female.

**I**: What are the barriers that prevent an adolescent girl not to get a balanced diet?

**P**: The first obstacle for a girl not to get balanced diet is that the economy is controlled by her parents. She does not possess anything. She will eat the same diet as the other family members do. She will eat whatever the type of food is with her brothers and sisters. There is no diet prepared for an adolescent girl alone. Not only the adolescent girl but even the mother will face the same thing.

**Section 3. Perceived needs of women for relevant services during pregnancy, lactation and adolescence**

**I**: Do you think women need special services than the above interventions we mentioned?

**P**: The first thing is, women should receive quality health services in their areas with good supply of medicines. The second is the water supply. Women are the most affected and they responsible to bring the water. For example, if there were donkey carrying water, they would be driven by females. Males are not supportive. The water is fetched from four-hours transport on foot and there is no water around this area. If the water shortage is solved it will be good for hygiene. The supply of drugs should be given emphasis. For example, in this health post, there is only a drug for malaria and cholera. You need to pay a transport cost to get other health services than the one I mentioned.

**I**: Is there a health center near this kebele?

**P**: There is no health center here. It is found in the wereda which one and half hour`s car transport or around four hours on foot.

**I**: What are other barriers are there that prevent a mother not to go to the health center to get services than the remoteness of the area?

**P**: It is far and you will at least spend one night there. You will wait to get the service and to do this, you will need money for renting rooms, for food and so on. The money is not easily found at hand. These are the barriers which hinder an individual not to go to the health center. But the community is aware of the advantage of testing and follow up.

I: What do you think are the obstacles that hinder a mother not to get rest during pregnancy?

P: The obstacle is herself. When there is some sort of shortage in home, she will be involved by herself to solve the problem. Males are not supportive. Whether the husband or male child will not be involved to solve problems that happen at home. The male child will not bring water. Therefore, the woman will get involved without her willing to solve the problem that happens at home.

**I**: You have told me that a mother included in safety net program will be given a rest until six months after delivery. What activities has been done that helps the mother to get rest at her home?

**P**: The counselling is given the same as others but there is a gap in applying it. Because, the mother will do every home related tasks like food preparation by herself until the time of delivery.

**I**: What do you think should be done to solve such problems? How should we aware the family as she needs rest at home which is the same as the rest given in the safety net program?

**P**: The husband should be involved in counselling session. If the husband is convinced, it will be easy for the mother to get rest. I am telling you this because if there is a gap in home the husband will try to fill the gap than the wife. He can do every task done by mothers including the preparation of stew. Preparation of injera and water should also be done by other members of the family.

**I**: Is there any trial done until now to include husbands in counselling?

**P**: Not yet. It is still a gap.

**I**: Is there any barriers that hinders a woman not to get iron folate for prevention of anemia?

**P**: I do not think there is obstacle for this. The drug is available in the health post. But, there is a gap in visiting the health post to check the level of the disease and whether there is anemia or not. Any ways, this is simple and the community is aware of this. There is no need of counselling about this.

**I**: you have told me that husband should be involved in counselling. What do you think is the role of a husband in woman and adolescent girl nutrition?

**P**: Anyways, it is good if the husband is aware of the nutrition related interventions. The husband is responsible for overall control of the family and if the husband is convinced, it will be easy to be applied.

**I**: Do women in this community change their diets when they are pregnant?

**P**: Not yet. It is said that a clean food should be take but it is not yet applied.

**I**: Why do you think so?

**P**: It is related to shortage of food. But it is also related with lack of knowledge. However, it is difficult to be applied even if it is known. For example, if the mother should take vegetables, there is no vegetable growing here. Vegetable is expensive and it is good if these things are growing around your home.

**I**: What are the food items advices to be taken by women and girls?

**P**: As I have tried to tell you, there is no special food to be given for a mother because of her pregnancy. If the husband eats shiro in the morning, she will take shiro with him.

**I**: What foods do women including pregnant, lactating and adolescent girls in this community should avoid?

**P**: Foods that are not recommended for an adolescent girl includes raw milk.

**I**: Why do you think so?

**P**: It is believed that the consumption of uncooked milk gives heat and if she gains heat, it is believed that she will try to start courtship with males. Anyhow, this is done to prevent the girl from starting sexual intercourse.

**I**: Do the adolescent girls accept it?

**P**: Yes. As this is taken as culture, not only the adolescent girls but even their mother does not take uncooked milk.

**I**: What about for pregnant and lactating mothers? What foods should they avoid?

**P**: I do not know. I am not sure foods that are not recommended for pregnant and lactating mothers.

**I**: What food do mothers choose during their pregnancy?

**P**: It is known that pregnant mothers should take better food starting from the simplest food elements like pepper. Pregnant mothers need foods which contain butter. But, the problem is in the utilization.

**I**: Are there gender disparities in women’s diets before pregnancy and during pregnancy?

**P**: No. It is the same.

**Section 4: Other interventions that improve pregnant, lactating and adolescent nutrition**

**I**: Is there nutrition screening here?

**P**: Yes. As I have told you before, the screening is done by health professionals. There are many individuals benefited from the screening. But the community is believing as it is a chance but not based on result of screening. Thus, people are benefited from the food aids while they are fat. After screening, the thin may not be eligible while the fat is not eligible.

Anyhow, the screening is there.

**I**: Do you have any additional observation in the screening when the fat is becoming eligible and the thin is not? What is the community`s compliant?

**P**: If two neighbours are screened for nutrition, and one seems to be fat while the other seems to be thin, there are situations where the fat will be eligible for food support. The health extension workers are giving advice as the eligibility is based on the screening result but not based on visual observation of fattens or thinness.

**I**: Is there a day called community health day in this area?

**P**: I do not think there is a special day for health. But, the day for fafa distribution is at 16^th^ day of a month.

**I**: Do you think there is counselling on how to use the fafa during the fafa distribution day?

**P**: Yes. The cause of the repeated underweight result is related to sharing. Instead of helping the child to take the prescribed food and show remarkable improvement, the child is obligated to share what s/he has been given to other children at home. It is expected that the child with the nutritional problem will be supported to have a good development including brain development but the food support will be equally divided to all children. The lactating woman will also not eat alone. But the husband will eat first.

**I**: Do you think women and girls needs to be targets for supplementary foods?

**P**: Yes. Because, if they are specially treated nutritionally, their body will be well built. Since they are going to give birth to a child, if they are supplemented with balanced diet, they will give birth to a healthy child. Therefore, it is good if they are targeted for supplementary feeding?

**I**: You have told me that pregnant and lactating mothers are benefited from safety net program without involved in the work expected from this program until six months after delivery. Do you think the community is aware of this condition?

**P**: Yes. The mothers will bring a support letter and the rest will be given.

**I**: Is there any complainant on the community to be benefited from this safety net program?

P: Since it is provided based on governmental procedure, there should not be any compliant. But, there are complaints from the community as it is quota dependent. If there are two poor and if the quota is only for one, you will take the poorest of the two.

**Section 5: Understanding perceptions of age at first birth and birth spacing**

**I**: You have told me that out-school adolescent girls get married before 18 years of age. What about in school adolescent girls?

**P**: Early marriage is currently banned in school adolescents. It is not hundred percent but it is significantly decreased. Since the school here is up to grade eight, adolescents who passed to grade nine must go to the wereda and hence, the family pushes the girls to marry instead of sending them to the town. This is related to fear of the family to their girls not to do unprotected sex since they are going to be out of families control. There is also lack of awareness on the community towards early marriage. The out school adolescent girls were marring at the age of 15 and 16 years recently. But the police, women affairs and health extension workers will not allow the adolescent to marry. Whether the ceremony is get ready or not, the women affairs will not allow the girl to marry if under eighteen years of age and she will be tested in Abi Adi hospital. Last year, there were marriage ceremonies and the girls have been tested in Abi Adi hospital. There were also ceremonies cancelled because the girls were under the age of eighteen years.

**I**: Do you think the early marriage is completely halted?

**P**: We cannot say the early marriage is completely stopped. Most marriages occur in the month of January as mass. The current challenge we are facing is marriage in summer times. Since marriage on the month of January is known, they afraid not to be seen by the health professionals. Thus, adolescent girls are marriage on the summer times where they are free from school. We have observed this gap and we are planning to work towards this issue.

**I**: In your opinion, what does the reaction of the community towards early marriage?

**P**: If a girl is fifteen to sixteen years of age, the family tends to wed her because of the fear of losing her virginity. The community feels pride if the girl is married while she is virgin. The family do not about the problem that she is going to face like fistula and so on if she gets married before the age of eighteen.

**I**: What do you think is the impact of delaying the age at marriage after 18 is better on the health of the women and adolescent girls?

**P**: If the adolescent is married after the age of eighteen, she is already matured and there will not be harm to her and her uterus. In fact, she should undergo testing against HIV/AIDS.

**I**: What about for the child?

**P**: Since the mother is matured enough, the child will be fed well. This is because the one who marry before the age of eighteen year will face risk of fistula for herself and other diseases which is also seen in this area. However, she will give birth a healthy child if she marries at the age of older then eighteen.

**I**: Could we link this with nutrition? How could we link delayed marriage and nutrition?

**P**: If she is lactating, she should not take fafa but porridge. She should get porridge with butter and even injera should be taken with butter.

**I**: you have told me that if a girl marries at the age below eighteen years, she will get diseases like fistula. Do you think the community is aware of these negative impacts?

**P**: The community is aware of the condition since it is happening in the community. The community prays not to face such harmful effects if a girl is married being under eighteen. There is also counselling and those who face such problems also present themselves to the community to convince the community with their urine and feces uncontrolled. They talk to the community as they expression this problem because of early marriage.

**I**: What will be the community`s reaction towards this practical learning session?

**P**: The community accepts this learning forum very well.

**I**: Do you observe early marriage after such kind of scenarios?

**P**: Anyhow, this was happening previously but, the farmer is becoming well informed nowadays. Since most adolescents are engaged in school, the incidence of early marriage is very low.

**I**: In your opinion, could we have any other option that better promotes early marriage?

**P**: The better way to stop this early marriage is taking serious measures for those who commit such acts. There should be teaching on early marriage and its harmful effect. Therefore, if a family wants to marry an adolescent despite the counselling, the measure should be taken since it is supported by law.

**I**: How much would a family be punished if the family marriage an adolescent whose age is less than eighteen?

**P**: There is no one who has been punished but I assume the punishment would be high. The main thing is not punishment. It is better to create awareness than punish a given community. There should be continuous advice and if there are families who are not obeying as per the advice, they should be punished. This punishment could also be additional education to the community.

**I**: How is the practice birth spacing in this area?

**P**: The birth spacing has been started recently. We did not reach at its final but we are at the starting point. There are families who give birth with five, six and seven years gap between successive births. There are also families who give birth every year. But, there is repeated education about birth spacing. Almost all females are using injectable contraceptives.

**I**: How many years do you think the gap should be between successive births for women?

**P**: The minimum should be five to six years.

**I**: What will happen if the spacing gap is shorter than this?

**P**: The birth of one child over the other is harmful. One thing, this area is affected by drought and we are waiting support from the government. The child will not have a good mental development. The community has been learnt from the repeated drought.

**I**: What are the most common contraceptives most mothers are using here for birth spacing?

**P**: One is the injectable given for three months, one year and three years. There is also rhythm method but this is not useful because there is high probability of getting pregnant. The injectable is the better one.

**I**: Which methods are you using to promote birth spacing here?

**P**: Birth spacing is supported by evidence from the community. The community is observing whether family with spacing or those who give birth every year are being benefited and leading a happiest life. We are teaching the community by giving examples from previous droughts. There are families who came home leaving their hunger children on street in the year 1985. We are teaching thee community by reminding such happenings.

**I**: How are the religious leaders accepting this birth spacing and delaying of the age at first marriage to be greater than eighteen years?

**P**: Religious leaders are accepting this. The bad thing is abortion and this is not supported by the religion. Religious leaders are not against the child spacing. Aborting is forbidden by religion because, the pregnancy is life and vanishing this life is considered as sin. This abortion could also affect the mother and even the mother may die.

**I**: In your opinion, how could the message about birth spacing be better promoted?

**P**: The child spacing should be repeatedly advised to mothers by strengthening their networks and gathering. There should also be experience sharing among these mothers.

**Section 6: Understanding communication and information sources**

**I**: Is there an opportunity in the community to discuss Nutrition for women?

**P**: The opportunity to talk about nutrition is one; when women come to take fafa from the food support of the safety net program. Second; when women come to screening. Repeated education about nutrition to mothers is given in these two sessions.

**I**: You were telling me that husbands should attend counselling session on nutrition. Do you think there is favourable environment for husbands, mothers and adolescents to discuss about nutrition?

**P**: It is easy to educate adolescent girl or male about nutrition. As I tried to tell you, the main thing is the male will have a role in advising and convincing even if females are engaged in preparation of the food. Since they are together in work and meeting, I think providing education is easy.

**I**: What are the common sources for nutrition during pregnancy?

**P**: It is from the meeting. You should advise her about the advantage of eating different foods for herself and for the child`s heath by mentioning type food she should take. If you advise the mother practically and include the husband as he is the source of economy of the house, there will be real improvement.

**I**: What about mothers who do not come to meeting? How are we going to reach these mothers?

**P**: If we want to teach about nutrition, she will come with the help of developmental army which is established by the government. Then, there will be discussion and she will apply based on the advice.

**I**: Who is supporting you in teaching about nutrition in this area?

**P**: The health and agriculture experts. The non-governmental organization will also provide teaching during fafa distribution.

**I**: What are the key points the non-governmental organization is stressed during the education about nutrition?

**P**: It is about sharing and its disadvantages. The one who is measured and eligible to take the food supplement whether the mother of child should get it alone. There is also practical teaching on the preparation and utilization of the supplemental feeding.

**Section 7: Additional remarks**

**I**: If you have any other additional suggestions or comments on pregnant, lactating and adolescent nutrition in this community.

**P**: There is nothing but what I want to say about the nutritional support for pregnant women is on the nutrition screening. The screening is done in only fifty percent of all pregnant women. As pregnant women, all should be seen equal and children should also be seen equally. It is good to have a child with good overall development. If a food support could be enough to cover all children, I would advice to be given to all children. But especially on the pregnant women, whether it is sufficient or not, I prefer if they get equal distribution.

**I**: Thank you for taking the time to discuss these issues with me today. I have learnt a lot from you. As I mentioned as the start of the discussion, I will remove all identifying information from the report of this conservation. I will make you sure that no one can identify your comments. If you have any concerns or questions, please feel free to ask me any questions. Thank you very much for your time. You cannot say everything is clear and applied by the community but there is improvement from time to time.

**Summary:**

**Section 1**:

- Women should eat balanced diet, visit the health facility for antenatal and postnatal care, do physical activity until they give birth to stay healthy
- The area is highly affected by drought and there are nutrition related problems like wasting and anemia
- Overweigh is not a problem in the area
- Food insecurity is very common
- There is severe shortage of water in the area
- Pregnant and lactating women are benefited from safety net program based on the prosperity they have

**Section 2:**

- The barriers that hinder women not to visit health facilities include lack of awareness and religious beliefs
- Mothers are advices to take extra meal but the challenges are related to shortage of food, husband dominance, lack of awareness
- Nutrition sensitive agriculture such as home gardening are not applicable in the area because of lack of water and presence termite that destroy the garden
- The area is malaria endemic and most families use insecticide treated bed nets but the challenges to use these bed nets include lack of awareness, lack of access especially for the shepherds who stay the whole night outside their house, and lack of supply

**Section 3:**

- The attention given to pregnant women by the community is very low
- Supports provided by the government for free are assumed to be useless.
- Counselling and practical demonstration on food diversification is given to pregnant.
- School feeding is inadequate
- Supply of food support is not sufficient.

**Section 4:**

- The nutrition screening is not trust by the community
- Adolescent girls need to be targets for supplementary foods because they are tomorrows mothers
- Food support of safety net program is quota based

**Section 5:**

- Early marriage is currently banned in school adolescents but it is common in those out school adolescents
- The better way to stop this early marriage is taking serious measures for those who commit such acts.
- Almost all females are using injectable contraceptives to space births
- The minimum birth spacing interval should be five to six years.
- Religious leaders are not against the child spacing.
- There should be experience sharing among mothers with better child spacing.

**Section 6**:

- The opportunities in the community to discuss about nutrition for women is the time of screening, meeting, fafa distribution
- Involving male in nutrition counselling is vital
- Practical teaching on the preparation and utilization of the supplemental feeding is given to mothers

**Section 7**:

- Not all pregnant and lactating women are screened for nutrition
- Food supplement should be given to all pregnant women
