# Supplementary material for: Barriers to utilize nutrition interventions among lactating women in rural communities of Tigray, northern Ethiopia: An exploratory study
Source: PLoS One. 2021 Apr 30;16(4):e0250696. doi: 10.1371/journal.pone.0250696 (PMC8087028; doi:10.1371/journal.pone.0250696)
Supplement: S2 File — (ZIP) [file pone.0250696.s002.zip › S2_File.Doc/Community level Key informants/092_FGD_WDA_Felegehiwot Kebele_Tankua Abergele woreda.docx]

**Operational Research on Adolescent and Maternal Nutrition in Northern Ethiopia**

**FGD with WDA**

**Introduction**

Good morning. Welcome and thank you for taking the time to speak with me. I am Hailemariam Tekie from Mekelle University. I came here today to study the factors that influence the nutrition of mothers and adolescents in collaboration with the Regional Health Bureau and UNICEF. As you are from the community, you know the problems existing within the community and also the possible solutions for the problems more than anyone. So, your participation is very valuable. The things that you tell me will be used to improve nutrition programs and services for women in the region and the country. Your names will not be included in the report. But, in order to capture all the ideas that you share me, I will record the discussion. The discussion will take about one and half hours. Do you have any question before we begin? If it is all right with you, I will turn on the tape recorder now.

**Section A: Interview details**

1. Zone: South-Eastern Tigray
2. Woreda: Tankua Abergele
3. Kebele: Felegehiwot
4. Facilitator’s name: Hailemariam Tekie
5. Date of discussion: 16/11/2017
6. Discussion start time: 10:32 AM
7. Discussion end time: 12:52 PM

| **Section B: Socio-demographic Information** | | | | | |
| --- | --- | --- | --- | --- | --- |
| No | Name of the FGD participant | Age | Marital status | Education level | Occupation |
| 1 | Chiros Shankabelaw | 33 | Married | 0 | WDA |
| 2 | Amete Hagos | 28 | Married | 0 | WDA |
| 3 | Echiwat G/medhin | 30 | Married | 0 | WDA |
| 4 | Mamite Marie | 28 | Married | 0 | WDA |
| 5 | Lemlem Amare | 30 | Married | 0 | WDA |
| 6 | Meite Asefa | 38 | Married | 0 | WDA |
| 7 | Lemlem Gebru | 40 | Married | 0 | WDA |

**Key**

I = Interviewer

P = All participants

The numbers used are the respective codes of the participants.

**Details of the FGD**

**Section 1: Common maternal (pregnant women, lactating women and adolescent girls) nutrition problems in the community.**

**I: What do pregnant women do to stay healthy in this community? Why? LW, AG?**

**02:** Starting from pregnancy, two or three months, she has to go to the health center for check-up and she has to be sure that she is healthy until she gives birth. Similarly, after delivery, the mother and her child should be checked for their health conditions. In that way, their health will be secured. Then, the child should only be provided with breast milk until six months. After six months, the child will be provided additional foods such as porridge, gruel, and eggs so that he will be healthy.

**I: What about adolescent girls?**

**P:** [Silence]

**I: Additional ideas please?**

[Baby crying]

[Discussion interrupted][Mother of the baby worried]

**02:** Adolescents are also visiting the health centers for check-up to be healthy.

**I: So, to be healthy, they are going to the health centers for check-up but do they do anything before they go to the health center? I mean going to the health center may also be after you get ill. So, do pregnant and lactating women and adolescents do anything at home to be healthy?**

**02:** To be healthy, she has to keep the house hygienic, should not breastfeed her child before washing, and should not put a dirty cup in to a water tank, we need to tie the cup so that it will be used for taking water from the tank without touching the soil, should not drink rain water from the ground. We do not have quality water so that we bring and drink water from the spring but we use water-guard if available and if not we boil the water for drinking purpose. We keep our health in this way. In addition to that we use toilet without contaminating the environment so that we become healthy.

**I: What about others?**

**03, 05:** That is it. She has already explained it.

**I: Ok. In your opinion, what are the common nutrition problems in the community for Women/Girls?**

**03:** She has already explained it but it is also good to go to the health center and give birth there. Previously, it was very difficult that we were staying at home for seven days suffering but now thanks to the government there is hygiene to protect diseases, there is also toilet, and water. With regard to child delivery, we go to Yechila. So, there is access but our source of water is getting dried. The distance is too far and it takes us four hours to bring water.

**I: Is four hours for one trip or double trip?**

**03:** It is a double trip.

**05:** It takes five hours to fetch water. Four hours is for the youngster but for us it takes five hours.

**I: So, what are the problems, for example it can be a disease or workload, or any other problem that the pregnant, lactating, and adolescent girls are facing?**

**P:** [Silence]

**I: Would you please say something?**

**04:** They have already explained it**.**

**I: For example, if we did not follow appropriate feeding practice or because of diseases, there may be a problem of becoming underweight and then there will be a risk that comes after. So, what are the problems in pregnant women and others in the community?**

**P:** [Science]

**05:** Please say something. Why are you keeping silent?

**I: My question is what are the problems observed in women in these areas in relation to nutrition?**

**03:** If the women did not go to the health center and if she could not attend check-up during pregnancy, there will be a problem.

**I: What is that problem?**

**03:** If she does not go for check-up, there will be a problem because it can only be identified through check-up starting from the pregnancy. She will be dead if she does not go for a check-up. Is it not that mothers getting died because of these problems? It is because the mother could not care of her health that she is dying.

**02:** You do not understand what he is asking. His question is what are the problems or risks that affect the women? The problems that are common in these areas are one thing is that these areas are lowland and sunny and we do not have enough time working to serve for all and then there comes anemia. During this time if you go to the health center, they will provide you blood through injection. But if you do not go to the health center, you will be affected. It is a big problem for the mothers as the areas are lowland otherwise the government is supporting us a lot. It is lack of awareness that we are highly affected otherwise the government is doing well. The mothers are facing problems because of droughts and workloads.

**I: You mentioned that anemia is a problem that the mothers are facing. But what other problem do you know that mothers are facing in relation to nutrition?**

**I: Is anemia because of workload or other reasons?**

**05:** This problem is observed when there is drought that the mother could not eat enough food. It is good that pregnant mother has to take three times a day but it is not because the mother is lazy to prepare food rather there is lack of food due to drought conditions. Where can we get food to eat let alone three time, eating once in a day is not easy. As a result of this, the mother will get thin and then within four or five years she will face the problem of iron deficiency. In case she eats food, she will be good. But let alone three times, how can we eat once in a day? See the farm how it looks like. There is no food [*explaining her ideas in desperate*].

**I: How severe is the problem?**

**02:** It happens in some part of the community. Some households having better living standards, they will consume nutritious foods but poor households will be affected because of lack of food.

**I: Ok. As you said, there will be difference among different living standards but can we say most of the people in the areas are affected or what?**

**02:** The ones that do not have to eat are affected.

**I: Are poor households higher than the rich or vice versa?**

**P:** Waaaa! All households do not have enough.

**05:** It is difficult to say this is better than that one. Every household does not have enough food to eat.

**I: You told me that there is anemia. What about goiter?**

**03:** It is known as Enkirti (ዕንቅርቲ) or Hifes (ሕፈስ).

**P:** [Naming mothers that have goiter and discuss each other]

**04:** Goiter is not known in our locality but we have mothers here that are originally from other areas.

**05:** They came from Amara region.

**I: What is the cause of goiter?**

**05, 03:** Waaaaa!

**04:** It can be treated by traditional tattoo. If there is goiter, they make tattoo so that it will not be expanded.

**I: What about night blindness?**

**04:** It was happening in the earlier times but now it is ok.

**I: What is the reason that it was happening in the earlier times and now it is not a problem?**

**04:** What could it be? There is health service. If you faced that problem, you will take a tablet and get recovered. When it is dry, you may face that problem but when you put butter, you will get better. The other thing is that when you eat nutritious food, you will look good but in case there is shortage of food, it becomes difficult to see. When it happens, we go to the health center and take tablets to get better.

**05:** Night blindness is due to lack of food at home otherwise if you eat food, there will not be night blindness.

**I: How severe is night blindness in the areas?**

**05:** Though there are some, it is ok but thanks to the government, there is access to get tablets. Previously, most of the people were affected by night blindness but this time we are ok due to tablet. The problem is lack of food.

**I: What about non-communicable diseases like diabetics, high blood pressure, and others?**

[Goats disturbing us because they wanted to have shade under the tree]

[A woman become busy to through a stone on the goats to make them far away from us]

**01:** Ohhh! Till now, we are ok. The problem we have is the repeated droughts causing shortage of food. It would have been nice to work and eat from our own products but because of drought, we cannot do that. We have also problem of quality water. We have different problems in this area.

**I: So, do you think there is a problem that comes because of these reasons?**

**01:** There was a problem of cholera. When they drink unsafe water, they become sick but after going to the health center, they get recovered.

**I: Let me take you back again to the non-communicable diseases. Is there any incidence?**

**05:** Who is going to have high blood pressure? Everybody is deficient [*emotionally speaking*].

[All others laughing]

**I: What about diabetics?**

**03:** Wooo!

[All said no]

**I: What do you think on the height/weight proportional of women/girls in this community to their age? Could it have relationship with their nutrition?**

[Long silence]

**I: Is my question clear?**

**P:** Yes.

**03:** What can I say?

[All laughing]

**I: Say it. It is not a problem to describe the problem and find a solution.**

**05:** Say something. So, how did you deliver the child? It is just about the nutrition?

**I: The government wanted to understand the real problem of the community. So, do not worry to express the problems.**

**03:** Is it about children?

**I: No. It is about adolescents, pregnant and lactating women.**

**03:** So, what… ohh!

[All laughing]

**I: If it is ok, you tell me it is ok otherwise explain the problem that is happening in relation to the proportion of height/weight to age in these areas.**

**03:** Some of them who were taking FAFA for children did not provide them properly.

**07:** Waiiii..!

**03:** [Oath (ስጋኺ)]

**03:** For children!!

**04:** If for example, you take your child and get his weight, he may be lower than the child that is measured after your baby. This is how you need to respond in women.

**03:** If I have blood I will be lower and.

[Most of them saying woooo!]

**02:** If you have blood, you will be good.

**03:** Yes, I wanted to say that otherwise if she does not have blood, she will be lighter. If it is comfortable for me, my weight will be good. What will happen with the balance? It will settle, heavier person will get down whereas lighter person will float above. If I am getting food, I will be good.

**I: So, what is the current status in the community? Are they above the normal or less than the normal? I wanted you to tell me in relation to their weight and height.**

**03:** As they said if she gets lighter, she will take a card then they will say she is lighter and gave her a card otherwise those who are fed and kept his hygiene and if there is no diseases why should I take FAFA?

**I: If I got you, your idea is that those who are fed and kept their health properly, they will have normal weight and height proportional to their age otherwise; he will be lighter or thinner. I understand that and it is good. So, let me give you the chance if you have additional ideas and I will ask you later about the status of the community.**

**03:** If everything is comfortable and healthy and if you eat nutritious foods, and wash and care properly, there will not be any diseases. Otherwise, your weight will be lighter.

**I: She (03) has already told us the association of weight/height to age but now I wanted to know the status of the community.**

**05:** There is no food. Food is not available but it would be nice to improve the weight. But now there is shortage of food and it will not be possible to provide food to this and that. As a result of this, there is reduction of weight and also he will get short. If a child’s mother is dead, he will be affected by kwashiorkor. They are provided one injera for two and then get lighter in weight. But those that are getting food will increase their height, weight, and also he will not be affected by kwashiorkor.

**02:** They have almost explained it. They have explained what will make heavier and lighter. If health is ok, height will increase. If there is shortage of food, his stomach will be enlarged (kwashiorkor) as they said earlier; the body will be thinner, and weight will get lower and lower.

**I: What about others? Do you have anything to add?**

P: [Silence]

**I: Ok, What about overweight? Do women in this community suffer from overweight?**

**05:** Wooo! From whom could they get it? From whom could they bring and become overweight? Everybody here is thin.

**02:** No. everyone here is the same.

**I: Is there a situation when women suffer from shortage of food? I can see what is happening in this area. So, my question is in such conditions, what will happen specifically in mothers?**

**P:** [Silence]

**04:** What can we say about mothers? It is just the shortage of food. If they get food, they will be full of blood. Otherwise, there will be a problem if they do not get food. There is no difference from the others.

**02:** [Silence] and then [Laughing] we become speechless.

**I: What are the problems to the mother when there is shortage of food?**

**02:** Is your question what is the cause of food shortage?

**I: No. There is shortage of food, ok? Then when this happens, what are the problems that the mother is facing? For example, what happens in some areas is that they give food to males first in case there is shortage of food. As it may be different in different areas, tell me what happens in these areas.**

**05:** She will lose heartbeat and fainted and it will be difficult for her to speak because of difficulty in breathing. Because of shortage of food, she will give priority to children and then will get fainted and become sick. If she goes to the health center, she will be treated and come back to her mind.

**Section 2: Barriers to access and utilization of nutrition services**

**I: What kinds of nutrition interventions are in place to improve health of the pregnant in this woreda? Where do they get it? Who provide it?**

**02:** I have for example a child three years old. During pregnancy, I have been going for check-up but during delivery I was feeling bad because I was deficient of iron. I told the health extension worker that I am terrified. The health extension worker took me to Yechila woreda and I was provided with injection and then I gave birth with caring support of the health workers and I was given a tablet, injection, and also porridge. So, there is special service at woreda. Previously, I had short birth spacing but now I have three years child and now we are fine by using contraceptives.

**I: This is on the health side. What about other interventions?**

**P:** [Silence]

**03:** Say something.

**03:** Is it on me. [Silence] [ohhhhh!] It is good to give birth at the health post. For example, I gave birth three times in the health center and I found it good in the caring practices. I have also given birth two times in Yechila. It is good if you take care of your health.

**I: Why is it good?**

**03:** If you keep your hygiene, if the child is fed properly at six months, and if provided complementary food in case it is not drought, we can feed ourselves.

**I: Do women advised to visit HFs for checkup and services during pregnancy? What services do they get? What would it help to them?**

**P:** [Silence]

**02:** Previously, it was not known that you stay home but now we are aware of and making awareness to our youngsters to have a check-up on health, give birth at the health center so that now people are changed though some people murmuring on us saying “what are they doing?” but most of them are changed.

**07:** I had wife of my brother, she was pregnant and I was called to take her to the health center because her husband was not available. I told them that I am not healthy to take her but they told me to take her though I am sick because there is an ambulance to take us. But late after two hours they helped her to deliver. Hence, the government is supporting the people that they provided food and also blood through injection to the mothers, and there was swelling on the baby and then they treated it. There is support from the government so that she escaped from death and I brought her home back after delivery. So, thanks to God, she is now living. Had it been at home, she would have been dead. At home, she would have been dead while we simply focus on coffee, smoke, and others but thanks to God, she is alive with the help of the experts. So, the government is supportive. If there is high blood pressure, they will reduce but in case she did not go to the health center, she will only get dead.

**I: Do you think that women receive advice on the need to get extra meal during pregnancy and lactation? How? How do you think that it would help for?**

**01:** I do not know. I do not have a child. I stopped delivery long time ago. How can I know what is happening now?

**I: You may not have a baby but as you are with the community, you may have the information.**

**01:** It is said that pregnant women should take four times a day. But if there is no food, you do not take food the whole day. If I get enough food, I will give birth healthy but if there is shortage, it will be a problem for me.

**03:** [Silence]. They give them FAFA.

**05:** After measurement, if she is found to be under the normal, they give her FAFA but if she is normal, they tell her that you are ok.

**I: Do you think that the food provided is enough and appropriate?**

**02:** Sometimes it happens that the ones that are affected may not get FAFA while others that are at a normal condition may get FAFA. The other thing is that previously, a child was provided with full of fiber-bag (ጭረት) but now it is a small plastic bag for those who are taking FAFA.

**P:** [Side talks] There is no fiber-bag since long time ago.

**03:** If a mother is below the normal, she is provided FAFA by a short firer-bag monthly for three times. In case the child or a mother could not get better, for example I was sick by that time and I was pregnant, they gave me a card. Actually, a woman was complaining because I was given while she was not allowed. I told her that I preferred to be healthy rather than sick to take FAFA. During that time I took three fiber-bags for three months. But from that time onwards, I am fine. If you are healthy taking holy-water and getting health services, you will be fine. If you are good when measured by the health extension, you will be concealed from taking FAFA. Otherwise, if you could not improve your status, you will continue using FAFA even to the extent that you could reach on the red line.

**I: What about Plumpy net?**

**03, 05:** It is only in children. We have never seen women up to that level.

**03:** If the child improves his status, he will stop taking FAFA but if not, he may even reach to the red level and take Plumy net.

**I: Are Women getting counseling for food diversification during pregnancy and lactation?**

**03:** They give us education; we take vaccinations on time and use prevention mechanisms.

**I: Who is giving counseling? How are they providing?**

**02:** While they give us training, they tell us that a pregnant woman should take five times a day and not to repeat what she eat once. They educate us but because of shortage of food, we eat what we have at hand. We do not have foods to diversify. But they teach us not to eat at lunch time the food that we have taken at breakfast and not to eat at dinner the food that we ate at lunch time. They told us to eat diverse foods five times a day and if there is shortage three times a day.

**I: Are women getting advice for the need to use iodized salt? Why?**

**02:** We iodized salt. First we add onion and oil, and pepper, and then at the end of cooking process near to we add iodized salt.

**I: Do you think all the people use iodized salt?**

**P:** We use iodized salt

**02:** Previously, we used salt that comes from Arho (ganfur salt) but now we use iodized salt.

**I: Do you have a reason to use iodized salt than ganfur salt? What is the advantage of iodized salt?**

**02:** It will improve health.

**I: What kind of health? Would you please explain it?**

**02:** Iodized salt is prepared properly and distributed by the government but ganfur salt is found in the coastal areas of water which is collected by flood. When we break and see the ganful salt, we find mud and other waste materials.

**I: Are women/adolescent girls getting advice on nutrition sensitive agriculture such as home gardening?**

**P:** [Silence]

**I: I know that there is shortage of water in the areas but I wanted to hear from you if there are experiences or motivations and barriers with regard to home gardening.**

**05:** We do not have access to water to grow vegetables. We are suffering from shortage of drinking water. Let alone for vegetables, we could not get water for drinking.

**I: What about on the need to be involved in safety net programs?**

**05:** The safety net program beneficiaries are crying because they could not get the food on time.

**I: What is the reason that they did not provide to the beneficiaries?**

**05:** How can we know that?

**I: So, how is the community living if they did not get it for long time?**

**05:** They live by working as a daily laborer. They go out of these areas and work there for food. Without food how can they stay here? They cannot stay here to die.

**I: So, how do you evaluate safety net?**

**02:** Previously, safety net was good because whether your family size is 10, 8, or 12, everybody was considered and also the amount provided was good and delivered on time in every month. But now whether you are 6 or 8, you are considered as 5. Any family size above 5 is taken as a family of five members and the amount given is small.

**05:** When they pay in cash, it become better than before.

**01:** They give us after eight or nine months. When it is in raw materials, we were provided with wheat, lentil, and oil but now if there is wheat, there will not be lentil and if there is lentil, there will not be wheat. And the worst comes now that there is no wheat, lentil, and oil. Now everything is not available since long time.

**I: Are Women/Girls getting advice on water, sanitation and hygiene services? How they do get it? Its necessity?**

**02:** When we have enough time, we boil the water for drinking but in case it is not convenient for us, we simply drink the water without boiling. It is very difficult to do that but they also give us water-guards otherwise it is difficult.

**I: So, are you saying water-guard is better than boiling as it saves time?**

**P:** Yes

**I: Ok. Have you ever observed the difference between using water-guard and without using it on the health condition of the community?**

**02:** In our kushet, we are good. Our kushet is called Giftawo but there were 7 or 8 incidences of cholera in the kushet Misaza near to kushet Giftawo by drinking water from Tekeze River but because of the health facilities, they were taken by ambulance and get treated. So, when we use water-guard, we drink the water freely but when there is no water-guard added to the water, we do not feel good to drink it.

**I: Is Malaria common in this community?**

**06:** There are mosquitoes but because of ITN, we are ok.

**02:** We are highly interested to get ITN and the government is also supplying. If we sleep in areas where there is no ITN, the mosquitoes will bite us and then we will be exposed to malaria. If we use ITN, the mosquitoes will be out of the ITN so that there will not be a direct contact. So, ITN is very important. The other thing is there is a chemical that is sprayed to kill mosquitoes. Previously, the chemical was effective to kill mosquitoes but the since last two year, the chemical spray is not effective may be because of the reason that the chemical that they are spraying is expired. It does not have any smell or any other thing. So, ITN is more efficient than the chemical spray.

**I: Are Women/Girls getting deworming services?**

**06:** We were provided a tablet.

**P:** [*Side talks to discuss to remember what kind of tablet they are provided*]

**06:** We have taken two types: one is to protect night blindness (vitamin A supplement) and the other one is to protect parasites. It is very important. For example, I was feeling bad but after taking the tablet, I feel good. It is good to kill parasites in stomach.

[Side talks: it protects from ameba]

**I: Which of the interventions listed above do you think is most important for Women/Girls?**

**06:** Child vaccination.

**I: What about in mothers?**

**06:** Check-up for health conditions. The use of ITN and deworming are also important for us. I accept these because I understood their benefits.

**04:** The pregnant woman has to check-up herself on time, eat foods on time, and the child has to follow vaccination properly.

**I: What are the barriers in the implementation of these nutrition services?**

**06:** We are worried that there is no food because of the drought and our husbands has gone to other areas for work.

**03:** Poverty. Poverty is the major barrier. Poverty is bad and as you can see the land it becomes white salt. It was ploughed, sawn, and weeded like that of other areas but it is bare became bare land.

**02:** One thing is that there is a problem of reporting the problem of the community to the higher administration. I cannot think that the government will hurt us but the local leaders do not report that the community is suffering. That why we are lagging behind.

07: There is no one to report that we are facing problems. That is what I can say.

**Section 3: Perceived needs of women for relevant services during pregnancy**

**I: You told me earlier what a woman do to be healthy. Now tell me what should be the role of a husband to improve nutrition for LW?**

**05:** If he provides nutritious food to the pregnant woman, she will be good and the baby will also be good. She will not be exposed to problems but if she does not feel comfortable, she will be weak and unhealthy and then the baby will be wasted. This is what I have to say.

**02:** What he has to do is that when she wanted wood, water, and others, he has to help her. It is said that a man like that of woman can cook foods to help his wife but if he could not help her, she will be wasted while working in collecting wood, water, cooking, preparing food, and other activities at home. But if he helps her, she gets some rest. So, she needs a support.

**01:** She took my idea. If his wife is pregnant, he has to provide her animal foods, butter, and honey.

**I: Do women in this community typically change their diets when they are pregnant?**

**02:** If an adolescent girl is fed nutritious foods and grows well and if she gets married at the right age, there will not be problem for her. But if she gets married at early age and if she could not get nutritious foods, she will face problems.

**01:** If she is pregnant, she will take variety of foods. If she hates injera, she will take macaroni. There is also a habit of eating diverse foods like different cereals, cabbage, lettuce, and others if available.

**05:** Where to get these foods? It is only if available. Here in Felegehiwot, we do not have anything. What we have is sorghum. How can we get and diversify three types of foods? Let alone diverse foods, we could not get only one food with the required amount.

**I: What about the amount of food a women is taking during pregnancy or lactation? Does she eat More or Less food?**

**04:** If she gets food, she will eat but if not what will she do? The only option is to go to bed without eating.

**I: Do you think, it is dependent on the availability of food?**

**05:** The thing is we have only sorghum and that may not be available. It also takes us five hours to get water so that the time wastes without doing anything. As you can see, God could not be with us, the one that we expected to harvest is withered because of drought. So, we do not have anything to diversify.

**I: What foods are recommended for Women/Girls?**

**P:** [Silence]

**02:** During pregnancy, the pregnant woman will be happy if she gets foods such as organ meat, macaroni, and others. But this is only if available.

**I: My question is not what you like but what a pregnant woman is recommended to eat.**

**02:** Here in our area, there is no option. What we have is only sorghum if available and sometimes teff may be found as a variety.

**05:** If the mother eats gruel, porridge, and bread, she will have enough milk for the baby. If he is hungry, she provides him gruel. But if there is no anything, where can she get from.

**I: What foods do Women/Girls avoid?**

**05:** We say a girl should not take milk to limit her growth but boys deserve the milk. We do not also provide porridge to a girl because we do not want her to have good growth.

**I: What is the problem if she grows well?**

**05:** If she looks good and grow well, she may go with a man. As we are in lowland, we do not have enough so that we preferred to limit her growth to protect her from going with a man.

**I: What about pregnant woman?**

**P:** Nothing.

**I: Are there gender disparities in women’s diets before pregnancy and during pregnancy?**

06: It was just in the earlier times but now there is no any discrepancy. Everybody in the family eats together.

05: If you have enough food, you may give food to the male first so that they will go to work early and then females eat later because we are at home otherwise there is no discrepancy. As there is shortage of food, we eat together because I will not have anything to provide later to the girl.

**Section 4: Other interventions that improve pregnant, lactating and adolescent nutrition**

**I: Have you ever gone for nutrition screening? Who provide you?**

**06:** They are measured on their hand (MUAC). The measurement is conducted at the health post.

**I: When do the measurements conducted?**

**05, 06:** It has its own day. The day the measurement conducted is during Kidanemihret (every 16^th^ of the month).

**03:** Kindanemihret is for the measurement of women and Gebreal (every 19^th^ of the month) is for measurement of children where they are screened for FAFA.

**I: Do you think community health days would have benefits for the women? How? What about routine service delivery?**

**05:** There is community health day.

**I: Why and what happens during that day?**

**01:** They tell us not to give birth at home, go for a check-up, go to the health center for child delivery because there will be risk of death when you deliver at home, it will be healthy for the mother and the child as well.

**I: When does it happen? Does it has a schedule or any time the organizers needed?**

**05:** It happens during ‘Aba Gaber’ once every month (every 5^th^ in the month).

**I: Do you think it is important?**

**05:** We said it is important. It is important to give birth at the woreda (health center). We are saying it is important.

**I: So what is the benefit from community health days?**

[Silence]

**I: Is my question not clear?**

**02:** It is clear.

**I: If it is clear, tell me the benefit that you get or if not tell be the problems and better option to be conducted?**

**02:** [ohhh] It is difficult. We finished what we wanted to say.

**I: Do you participate on the community health days?**

[Silence]

**I: If you and others do not participate, what are the challenges to attending community health days?**

**P:** [Silence]

**03:** The health extensions come and teach us and also they give us training about proper feeding and prevention of diseases. Poverty is very bad but they tell us to check-up on time and give birth, use preventions on time, and they tell us everything. In addition to that the mother and child who are vaccinated are different from those who do not attend vaccination. If you do not attend, it will have a problem. Our sisters who are not attending the check-up and not getting the vaccination are dying.

**I: Do you think women needs to be targets for supplementary foods? Why and why not?**

**06:** Yes. Those mothers and children who are below the normal are provided FAFA. It is important that the child will grow healthy and the woman after giving birth will look good.

**05:** The FAFA is by itself a special food. So it is important for the lactating woman and also for the child. It is also important for the pregnant women. But the amount they give is small. Give your witness *(to the other participants*). Those of you, who have been taking, please say something.

**I: Are women beneficiaries of the soft conditionality of the productive safety net program, PSNP? How?**

**02:** If the woman is six months after pregnancy, she will bring certificate from the health center and provide it as evidence so that she will be allowed to rest until nine months of child delivery. But after nine months of child delivery, she will be involved in the safety net program activities. So, the government is supportive on this regard.

**I: What about its implementation? Do you think there are problems in its implementation?**

**02:** There are some problems. For example, if she gives birth at home unexpectedly assuming that it is not the time to give birth, she will not be allowed if she does not have a certificate.

**I: For the time being, let us assume this is her fault. What about the implementation problems if she has a certificate?**

**P:** No. there is no problem on the process of implementation.

**I: Do women in this community know why they are targets of the program?**

**02:** To protect the mother from harms. It is sunny and windy which will be difficult for the baby. They are working on digging the soil and let alone to the baby, it will be difficult for adults. So, to protect the child and the mother from the risks, she is allowed to get rest.

**Section 5: Understanding perceptions of age at first birth and birth spacing**

**I: Do you think delaying the age at first birth to after 18 is better for the health of the women? How? What other benefits does it have for the women? What about for the baby?**

**02:** When an adolescent girl gets married at the right age, she will have a good living, whereas an adolescent girl married at early age will not be healthy and will not have the resistance so that she will be exposed to different diseases. So, it should be at the right age that a girl has to get married as she will be able to manage her life properly. But if she goes at early age, she will face many problems.

**I: What kinds of problems? Would you please mention them?**

**02:** One thing is that she will have pregnancy at early age and then it will be difficult for her to give birth because of lack of energy and she will be exposed to deficiency of iron and then face a problem. Whereas, the one who got married at the right time will give birth on time, she will be healthy.

**I: What about on the child?**

**07:** I do not know.

**06:** There is a difference. As she is under age, her breast will be small to hold enough milk. She will not also use contraceptives because she does not have the knowledge. But the one who gets married at the age of 18 or 19 will be mature enough to care her child.

**I: Do you think this message is being promoted in the community? Who are working on it? How do they promote?**

**04, 05, and 03:** Yes there is.

**05:** To protect the mother from the problems, there is promotion. They call a meeting and then during the meeting, they promote not to send an adolescent girl before the age of 18. They also visit home to home to promote the message. We also visit home to home to tell the mothers on these issues.

**01:** Administrative leader of the kebele is also working to promote to prevent early marriage.

**I: In your opinion, what does the reaction of the community looks like to the promotion? Why?**

**05:** We accept the messages because it is for our benefit.

**06:** There is check-up to make sure whether the adolescent girl is above or below 18 years old.

**I: In your opinion, how could this message be better promoted? Who should be involved?**

**02:** [Can you allow me to go? I have another meeting. They are waiting for me.]

**I: [Just wait some minutes. We are just near to the end.]**

**I: So, how could it be better promoted?**

**P:** [Silence]

**03:** We are promoting not to send girls at early age. When it happens, the marriage is concealed.

**I: How many years do you think the gap should be between successive births for women? Why? What about if shorter than it?**

**05:** It is good. You will grow your child in a good condition. If there are many children, you cannot provide cloths and shoes but if you increase the spacing between births, you will handle your child properly and provide cloths and shoes.

**I: So, how many years do you think the gap should be between successive births?**

**05:** They give birth at a spacing of five years. For example, personally, I have been eight years since the last delivery. So, my child is in a good condition but those that are giving birth year after year are not looking good and they do not have clothes, shoes, and the food provided to them is not enough.

**I: From who have you ever heard? For the last time? Who are involving on it?**

**01:** The health extensions are promoting the message.

**I: What about others?**

**01:** There are also others promoting such as water experts.

**06:** Woreda parliament, Kebele administrations, health experts from woreda are also involved in these activities. We, as WDA, are also involved in the activity.

**02:** First, the government sends a message and then the health extensions tell and teach us about it. We also have discussions with them, then after we go to the public to tell the mothers to go for check-up, vaccination, and others. Now there is a big change on the community.

**I: What do you suggest promoting it in a better way? Which population in this community is not addressed with the promotion?**

**05:** It is told that mothers should increase spacing but I suggest not allowing mothers to give birth at all because there is shortage of food in our local areas. What will she provide to grow him? Let us first grow the born children properly.

**Section 6: Understanding communication and information sources**

**I: Is there an opportunity in the community to discuss Nutrition for women? What are the opportunities?**

**06:** We have discussion with the health extension during 5^th^ of a month.

**I: What do you discuss about?**

**06:** They tell us to convey the message to the community to use contraceptives, give birth at the health centers, go for a check-up, and increase birth spacing.

**05:** It just telling to promote increasing the birth spacing, to take them to the health center for child delivery, to encourage a pregnant woman to go for check-up.

**I: What about nutrition?**

**04:** Preparing a complementary food: using the spoon, take teff, wheat, maize, sorghum, any other food available at home will be added. If there are seven types at home, we add from all and then after cooling the porridge, we add iodized salt.

**I: Is the information accessible for all women?**

**05:** Yes. We are teaching them.

**I: What are the barriers for access to information for nutrition during pregnancy?**

**05:** There is lack of awareness. There are women who do not accept the message that we promote. They simply consider that we are specially benefited but on the other hand there are women that work hard for the improvement. So, this is due to lack of awareness.

**02:** [I am in hurry because they are waiting for me.]

**I: Ok, we are almost finished. Share your idea about this.**

**02:** We are taught and we teach them but some of the mothers do not think that we are working for their benefit. So, this is becoming a barrier due to their less acceptability because of lack of awareness.

**I: Which information is effective for you to change your practice in nutrition during pregnancy? Why?**

**01:** I am told to promote the activities and then according to the information I got, I promote them. All I have done in the community has been accepted.

**02:** What we have learned is to eat porridge from a mixture of teff, wheat and other things. Similarly, we provide for children by mixing all these and add dried meat if available. So, this kind of households will be more effective to be healthy.

**I: What about in mothers?**

**03:** there was a great change on the status of mothers taking FAFA, porridge and well prepared bread. This was effective in mothers.

**I: Which source of information about nutrition is essential for you?**

**06:** Vaccination is important that most people are willing to have it for their children. The other one is FAFA for children that many people wanted to take.

**Section 7: Additional remarks**

**I: Any other additional suggestions or comments on pregnant, lactating and adolescent nutrition in this community.**

**P:** [Silence]

**05:** The government has to consolidate on the activities of vaccination.

**02:** The support on the additional foods (FAFA) should be improved. As we are facing problems because of shortage of water, the government has to help us with this regard. The FAFA for children is reducing, so this has to be checked.

**I: Is it in mothers or children?**

**02:** For all, it is the same. So the government has to see this thing.

**04:** No idea

**01:** There is a problem on the safety net program. There is no one asking that we are not getting on time. There should be a solution to provide us on time.

**06:** You can see that our area is affected by drought. We are not doing anything. Our children and husbands are displaced from home for work. So, the government has to help us.

**I: In what way would you like to be helped?**

**06:** To provide us food aid

**Thank you very much for your participation and time.**

**SUMMARY**

**Section 1: Common maternal (pregnant women, lactating women and adolescent girls) nutrition problems in the community.**

- It takes five hours to fetch water.
- Let alone three times, eating once in a day is not easy even for the pregnant women due to drought conditions.

**Section 2: Barriers to access and utilization of nutrition services**

- Let alone for vegetables, we are suffering from shortage of drinking water.
- Poverty is the major barrier. The land was ploughed, sawn, and weeded like that of other areas but it became bare land.

**Section 3: Perceived needs of women for relevant services during pregnancy**

- How can we get and diversify three types of foods? Even we cannot get only one staple food, sorghum, with the required amount.
- As we are in lowland, we do not have enough so that we preferred to limit the growth of adolescents to protect them from going with a man.

**Section 4: Other interventions that improve pregnant, lactating and adolescent nutrition**

- Safety net beneficiaries are working on the soil with sunny and windy conditions which will be difficult for the baby and the mother. So, to protect the child and the mother from the risks, they are allowed soft conditionality.

**Section 5: Understanding perceptions of age at first birth and birth spacing**

- It is told that mothers should increase spacing but I suggest not allowing mothers to give birth at all because there is shortage of food in our local areas. What will she provide to grow him?

**Section 6: Understanding communication and information sources**

- There are women who do not accept the message that we promote. They simply consider that we are specially benefited. This is due to lack of awareness.

**Section 7: Additional remarks**

- You can see that our area is affected by drought. Our children and husbands are displaced from home for work. So, the government has to provide us food aid.
