# Supplementary material for: Barriers to utilize nutrition interventions among lactating women in rural communities of Tigray, northern Ethiopia: An exploratory study
Source: PLoS One. 2021 Apr 30;16(4):e0250696. doi: 10.1371/journal.pone.0250696 (PMC8087028; doi:10.1371/journal.pone.0250696)
Supplement: S2 File — (ZIP) [file pone.0250696.s002.zip › S2_File.Doc/Community level Key informants/094-IDI-HEW-Felege Hiwot kebele_Tankua Abergele.docx]

**In-depth Interview with HEW in Tanqabergele Woreda Felegehiwot Kebelle**

Zone: Southeast

Woreda: Tanqabergele Kebelle: Felegehiwot

Name of Key Informant: Letesilassie Assefa

Institution of key Informant: Health Post

Name of interviewer: Measho G/slassie

Date of interview: 15.11.2017

Start time; 11:00 AM End time; 12:30 AM

**Interviewee Professional Information**

Age: 25

Gender: Female

Highest level of completed education: College education

Current job/position: HEW

Work experience in the current position: 11 months

**I**: Interviewer **P**: Participant

**Section 1: Common maternal nutrition problems in this community**

I: Thank you. When I come to our first interview, how do you describe the common nutrition related problems in pregnant women, lactating women and adolescent girls of this community?

P: Nowadays they have better awareness on how to feed, when to feed, how many times a pregnant woman should feed and when a baby should start complementary feeding. However, to some extent, there is shortage of food access. Due to the shortage of water, they don’t prepare their foods in a hygienic way. There is critical shortage of water in this community and the available one is very unpleasant and far. So, the foods are not prepared in hygienic way and as a result the unhygienic food is not healthy and it will be entering to your body with the diseases. This community is highly affected by drought. It is almost seven years since the rain was stopped in this community. They try to harvest their agriculture in every summer, but they were not productive due to shortage of rain. It is almost a year since I come to this community and they told me that it is seven years in row since they produced their own agriculture and I have also learned that this is the first time they have seen grasses in the last seven years. So, this is what it looks like.

I: Good. Are there adolescents, pregnant and lactating women who have nutritional problems associated with the drought or other problems?

P: Yes, we have been screening the nutritional status of children on the 7^th^, 12^th^ and 18^th^ days of the month through going to the “kushets” (gots) to identify the malnourished children. After identifying the malnourished children and mothers, we let those who can be easily treated to enter to the TSF and we also treat those who can be treated in our facility (outpatient therapy). Therefore, we have been screening their nutritional status and also treating for their malnutrition.

I: Good. Besides the children, are there malnourished adolescent girls identified by your screening?

P: Yes, there are malnourished women. For example, we have identified more than twenty in this month and entered them in to the nutritional support.

I: Are the identified malnourished women within the adolescence age (from 10-19 years of age)?

P: We have not identified malnourished adolescent girls (10 to 19 years old). Most of them are more than twenty years of age. However, we have two girls whose age is from 10-19 years old and the remaining are more than 20 years old. Most of the time the problem is happening in the child bearing mothers.

I: Do you provide the nutritional screening service for all people?

P: We don’t screen all people. We only provide the nutritional screening and treatment services for the pregnant and lactating women and for the under five children.

I: Good. How do you describe the nutritional problems in the lactating women of this community?

P: What do you mean by the nutritional problems?

I: You have told me that you have been screening the pregnant, lactating and under five children for their nutritional status. So, from your observation, how do you see the problem of under nutrition and its associated problems among the screened lactating women?

P: Anyhow the problem is relatively better in the under five children compared with the mothers. The mothers have chronic and untreatable under nutrition. However, the malnourished children will quickly improve if you provide them the treatment and follow up. But the mothers usually stay under the nutritional treatment for the fourth or fifth times or until they are discharged without any change because their problem is chronic malnutrition and it cannot be changed. But, sometimes, there are mothers who improve after nutritional treatment, especially those women who had feeding problems due to their different temporary diseases or problems associated with child delivery. The healthy looking women identified to have under nutrition by screening cannot quickly respond to the nutritional treatments because their problem is chronic.

I: Ok, how do you see the problem of under nutrition and its associated problems among the screened pregnant women?

P: Generally, when we ask them, they have good awareness about feeding. But they don’t implement it. For example, a pregnant woman should eat an additional two meals from the normal feeding practice, but they don’t implement it. They know that they are expected to feed an additional meal, but they don’t do it, instead they give priority to their children and husband. Therefore, despite they have the awareness, they don’t implement it and as a result maternal under nutrition is common problem in this Kebelle. Almost any nutritionally measured mother in this community is below the standard.

I: You have told me that the mothers of this community are not implementing the good feeding practice though they have good awareness. So, why do you think they are not implementing it?

P: What can I say? They are giving the priority for others even if they know it is more important for them. If you ask them why, they tell you that “we know it is important to eat extra meal, but we have food shortage and we can’t feed ourselves without feeding our children, and “we would love to shared it had enough food was available”. In addition, culturally they don’t eat before their husband. According to the community culture, the wife cannot eat at all if her husband doesn’t eat by any means. First, she has to prepare for her husband and then she will eat later if there is anything left.

I: Good. Could you tell me any interventions being implemented to solve such kind of harmful traditions for the mothers?

P: After we assessed the cultural problems, we met and discussed with the women development army about the benefits maternal nutrition, for example, we told them that if the pregnant women do not get balanced diet, both the mother and her fetus will be affected by the nutritional problems, and we also advised them to give priority for maternal nutrition over her husband because if they mother failed to get proper diet, her child will be physically and mentally affected, and she may also face problems during delivery including blood shortage. Later we discussed with men development army on what we have discussed with the women development army. Then, when we counsel the pregnant women, we always discuss together with their husband at their home based on the family folder guideline. Hence, we are currently discussing with the pregnant women’s husbands on how wife and husband should feed, how he should support his wife and when and how many times she should take meals. Hence, we are counseling about the pregnant women nutrition not only for the pregnant women but also for their husbands.

I: So, are there observable changes on maternal nutrition after you started nutritional counseling both husbands and wives together.

P: First, you will get satisfaction by creating the awareness. Then, when we come to the implementation, despite it is not practiced in all women, there are very good changes in some pregnant women. There are also husbands who follow and take care of their wives in terms of discussing the problems of their wives and bringing them to the health facilities for diagnosis and follow up. There are some husbands who worry about their wives. Therefore, even though there are no big changes, we are observing good signs of change. However, the main challenge in this community is the repetitive drought.

I: Good. Are there severely malnourished pregnant and lactating women and adolescent girls who have been nutritionally treated in this community?

P: Yes, there are severely malnourished mothers.

I: Could you explain more about the severely malnourished women who have been taking Plumpy’Nut or treated in the health facilities?

P: We don’t have women who have been nutritionally treated in the inpatient of the health facilities yet, however, we had children treated by Plumpy’Nut in our Kebelle. For example, we had two children treated with Plumpy’Nut within the past three months. I have had a call today from my office to inform me about the availability severely malnourished child in our health facility. So, we have treated two severely malnourished children in the past three months. We usually refer severely malnourished women to the health center, but currently we don’t get severely malnourished women.

I: In addition, are there moderately malnourished pregnant, lactating and adolescent girls who take Fafa in this community?

P: Yes, there are women who have been taking Fafa in the community.

I: What types of women are taking the Fafa?

P: I already told you that we have nutritional screening services in every gots (“kushetat”). After screening, the women having greater than six month pregnancy and those who have less than 23cm MUAC are given the support every month.

I: Good. How do you see the deficiency of relevant micronutrients like anemia in this community?

P: I have not seen anemic women yet because we are following and advising them. However, the pregnant women who are hidden in their homes may be exposed to anemia, because they will not get the nutritional counseling and treatment for the prevention of anemia. Starting from my arrival in to this kebeles, we have not found anemic mothers.

I: Do you think there are pregnant women who don’t have antenatal care follow up in this community?

P: At least there are no any pregnant women who don’t have contact with us, but our main problem is we don’t have the diagnosis modalities and hence they are expected to go to Yechila Health Center for diagnosis.

I: Good. How do you see the availability of night blindness in the pregnant and lactating women of this community?

P: I have not seen women with night blindness in this community.

I: What about goiter?

P: Goiter is not common in this community, but there were some women who have goiter in my previous work area. However, I have not seen any women who have goiter in this community.

I: Where were you previous work area?

P: I was around Yechila, specifically call endarufael. There were around three women who have goiter there.

I: As you have told me before that you have been measuring the MUAC of pregnant and lactating women to measure their nutritional status. Do you provide the nutritional screening services for the in school and out school adolescents?

P: We screen only the pregnant, lactating and under five children. Before two years, we were providing iron supplementation for all adolescent girls living in the lowland areas, but it was given only for one year.

I: Why do you think the iron supplementation for the adolescent girls is stopped?

P: I don’t know why they stopped it.

I: In your opinion, do you think the adolescent girls should be screened for their nutritional status?

P: Yes, they have to be screened because they are around the pregnancy and child birth period. So, they have to be screened, but we are not screening them because it is not included in the program.

I: So, there is no program or budget from the top down for specifically screening and improving the nutrition of adolescent girls?

P: Yes, there is no any activity.

I: Good. How do you see the diet related noncommunicable diseases like hypertension, diabetes mellitus and others in this community?

P: I have not seen hypertensive people in this community, but we have one diabetic patient following for diabetes mellitus treatment. But, there are no women who have diabetes mellitus in this community.

I: Is there any education given for the community to prevent from diet related noncommunicable diseases

P: There is limitation in educating the community about hypertension and diabetes mellitus because we are not creating awareness about it. Generally, we have been advising the community to seek early treatment if they have the signs and symptoms of it. But, we have not discussed about the causes and prevention methods of such diseases.

I: Good. How do you see the magnitude of stunting in the adolescent girls?

P: “oh! Many!” most of them are short and thin. The chronic malnutrition happening in the mothers is due to stunting. A mother of this community is always under the Fafa support starting from her pregnancy up to six months after delivery. This is because the mother has long lasting chronic malnutrition. Usually the temporary problems (malnutrition) are easily reversed. So, stunting and thinness are common in our community.

I: What about underweight?

P: underweight is also common in this community.

I: Do you think the stunting and underweight are common in this community due to shortage of food or due to lack of community awareness on how to use balanced diet?

P: The community by itself believes that there were harmful cultures in the past, but now they have left the harmful cultures and replaced it by good feeding cultures. The stunting and underweight can be inherited from their mothers. A child of the stunted mother could be affected by under nutrition. So, the stunting and underweight can be caused genetically from their fathers and mothers, and it can be also caused by drought of this community. We have not come across with other causes of stunting and underweight. Hence, due to lack of awareness and bad cultures about feeding, the mothers in the past time were stunted and their children are also stunted and underweight. So, the first cause of stunting and underweight it is genetically transferred from parents to their children and the second is due to drought and shortage of food.

I: On the other hand, how do you see overweight?

P:”Esay” (Amharic/Tigrigna word)! It is impossible to get.

I: Ok, how do you describe the food security of this community? You have already touched it before.

P: What is food security?

I: In other words, does the community have food access from year to year?

P: Specifically this community from Tanqaberge has been suffering from drought and famine in the last seven years in row. There are also another two or three kebeles in this woreda similar with this Kebelle. Relatively this year is better than the past seven years; at least there are grasses and other foods for animals. Even there were no grasses let alone cereals in the past few years. Hence, this is food insecure community. Currently there are so many people who do not have anything to eat and there are also many women who spend days without eating (“tsomen tedefien zihadra”). For example, I remember we collected money and bought few amount of flour from the grinding mill for around three women during the New Year just for one day lunch. Later the government gave some support. So, many people are suffering from famine in this community.

I: Is there any government support for this community? In the form of wheat, oil or others

P: Yes, there is support, but it is not enough due to their large family size.

I: Ok, do you think women are especially at risk for malnutrition in this community?

P: Yes, they are at risk, because they cannot go out of their home for work by leaving their children at home. The women are suffering at home with their children. Their husbands are not affected because at least they will go out of their home for work and when you look at the husbands face or body they are relatively better because as I have told you they will go out of their home for different works. For example, almost all males in association spend the winter in fishing activity and they move from town to town to sell fishes. So, the males will not have any food shortage to eat. Therefore, the women are more at risk for malnutrition.

I: In addition, do you think the women in this community are more affected by stunting and wasting?

P: Yes, it is more common in the women. You can easily differentiate through observation that the women are more affected; even you can see the students.

I: Is there any specific reason on why the women are more affected by stunting and underweight, in addition to what you have descried before?

P: We have not studied more reasons apart from what I have described before.

I: You have told me before that the women in this community feeding priority for their husband and their children. So, from your observation, are there any reasons why the adolescent girls are more stunted or underweight than the male adolescents?

P: What can I tell you on why the adolescent girls are more affected than the adolescent boys? Anyhow, priority is more given for the boys than the girls. From our observation, they prefer to give less food for females than the males. If you ask them why, they will tell you that if the males are hungry their tendon will decrease over night. But, from my personal point of view, I doubt whether they really see their daughter and son differently or not. I don’t personally believe a mother could discriminate between her children. However, people say that if male eat less food, they will decrease over night, but the females have the capacity to tolerate it even if she eats less.

**Section 2: Nutrition priorities in the woreda**

I: What priorities do your institution (HEP) has in relation to maternal and adolescent health?

P: first we discuss with the women and then after identifying the problems we discuss with their husbands. Generally, the main priority of the health office is on women’s health because the women are the one who become pregnant and deliver the child, and if they are harmed, their pregnancy or child will be affected. So, if we give priority for the women, we believe that we can prevent most of the problems.

I: As you have told me the main priority of the health office is on women. So, what are the activities undertaken to improve the women and adolescent health?

P: For example for the pregnant women, there is good follow up from the time of conception up to two years after delivery and there is also follow up for the child up to his/her five years of age. The follow up is given on their nutrition, vaccination, family planning, sanitation and in general we follow and prevent all the possible problems for the women.

I: What about for the adolescent girls?

P: There is no priority given for the adolescent girls besides the health education. We educate them to get TT vaccination after 15 years of age and we also educate them to continue their education. However, apart from the health education given together with the male adolescents to use toilets and maintain their sanitation, there is no special follow up and discussion like what we do for the pregnant and lactating women.

I: What are the specific activities done by the health extension program to improve the nutrition of women?

P: We have been showing a demonstration for the pregnant and lactating women on how to prepare balanced diet from four different food items in the form of porridge from our own resources. For example we have regular meeting with the pregnant women and lactating women on every 16^th^ day of the month (Ethiopian calendar) to teach them on how to prepare the balanced diet and its ingredients and its benefit. In the next month, we discuss on who properly prepared the porridge containing diversified foods and we also evaluate the progress of the children. If the any child doesn’t make good improvement, we separately discuss with his mother if there was any problems in the food preparation and if the child has health problems.

I: Good. Do you think the priority given for the interventions of the women are enough?

P: Do you mean the education that we have been giving to them?

I: Not only the education but also the priorities given for all interventions of the pregnant and lactating women?

P: Despite we have human resource shortage, what can be done for the women more than this? We are providing the health education about almost all the problem a women are expected face. If there is enough human resource, there will not be service interruption for the community. Strict follow up and reluctant follow up are not equal and it is also big difference between visiting the household in a monthly manner and daily manner. Hence, we can only change the community culture by frequently visiting them. So, if there is enough human resource to visit and discuss with the community in a daily bases, it is possible to change its culture. If the community once adopted the new culture, the will implement it. For example, they were not washing in the past, but now they started to wear clean cloths because they adopted it and the culture is changed. In addition, they were using hair butter over the previous one without washing, but now you will not find any women who use hair butter before washing. Progressively there is change from the previous culture. First they are shy to implement the new activities, but through time they make it a culture.

**Section 3: Nutrition interventions that improve adolescent and maternal health**

I: You have informed me that you are educating the pregnant and lactating women to improve their nutrition through demonstrating how to prepare balanced diet. What other activities are in place to improve their nutrition?

P: There are agriculture and education working on maternal nutrition. There are three agricultural experts in the Kebelle; animal resources, natural resources and seed production. So, the seed expert helps them to use selected seeds, the animal resources also advise them to use egg, chicken, and other animal products. All sectors are giving priority for the women. Therefore, not only the health sector, but also all sectors are working on the nutrition of the pregnant and lactating women are working in their respective areas.

I: So, you mean that all sectors are working to improve the nutrition of women?

P: Yes

I: Ok, how do you describe the advice given for the pregnant women about their ANC?

P: By ourselves?

I: Yes

P: We have said that at this time we don’t have any pregnant women who don’t follow her ANC. When a pregnant women comes for ANC, we provide all the services like counseling, we provide her iron, we also advise her about her nutrition (what types of foods to eat, her current status, how many kg to add). We advise her everything relevant for her and finally we advise her when to come back to the health post. We are providing the services to improve the strength and health of the mother and to have sufficient development of her fetus. Our main drawback that we are struggling to improve is sending pregnant women for diagnosis to the hospitals and health centers.

I: You have told me that you have been counseling the pregnant and lactating women to eat extra meal. Do you think you are providing the counseling for all pregnant women?

P: It is a must to meet all pregnant women and we are providing the counseling for all pregnant women. We are here to provide services primarily for the mothers and we do the other activities in our remaining time. Since are meeting the pregnant women, we are providing all necessary services. However, the services are accessible for the adolescent girls.

I: What about for the lactating women?

P: It is similar with the pregnant women. We advise the lactating women to eat three additional meal from the ordinary people and to exclusively breast feed up to six moth age of their child and in addition, to provide complementary feeding after six months.

I: Do all the lactating women are screened for their nutritional status?

P: Yes, all pregnant women are screened and after six months, we screen their child.

I: Good. How do you describe the counseling given for the pregnant and lactating women bout diversified feeding?

P: I have already told you before that we are not only orally counseling them but also we are practically showing them how to prepare balanced diet. We demonstrate the preparation of diversified foods from our own foods. But, sometimes, to improve their understanding, we request the women development army to bring little quantity of roasted teff and roasted sorghum and others will bring pea, and we also request others to bring one egg and also another woman may bring a cup of milk. Then, first we tell the women who brings what and the types of foods prepared, and then we prepare the diversified food in the form of porridge under this tree (showing me a big green tree). But there is no any vegetable in the community for demonstration as well us for use but we advise them to use it whenever they get it. We are requesting the women to bring sample ingredient foods to easily understand it. We don’t gather all women for educating about the preparation of diversified foods; rather we show them in their nearby area. For example, we educate and demonstrate the porridge preparation in the 19^th^ day of the month in this area, in the above got, we prepare it in the 7^th^ day of the month, we also prepare for the other got in the 12^th^ day of the month. If it was not possible to educate them in the stated time, we just provide it in the churches. We have adopted each other and we are very strict in implementing it.

I: I: Good. As you have told me there, there is water shortage in this community. With the existing limitation, how do you see the efforts undertaken to cultivate home gardens in this community from the scarce water they have?

P: Advice is given but no one cultivates the home garden. Even we advise them to collect and use the water used for hand and coffee equipment washing. But, this is very sunny area and I am not sure whether it is possible to grow vegetable or not. We may try it in the house where we live, but there is no vegetable in this area. However, you may get egg because there so many chickens and though not many you may also sometimes get milk because this is lowland community. But you will not get any vegetables.

I: How about the counseling given to use iodized salt?

P: This community has been using iodized salt. Even the community was using the iodized salt before I come to this area and still they are using it. But, there are some people who don’t properly use it (they cook it with the stew). However, majority of the people know when to use the iodized salt and we are changing the remaining people who do not properly use it.

I: How do you describe the implementation of the safety net program in improving the nutrition of the pregnant and lactating women?

P: Yes, they are the beneficiaries of safety net program, but the support given is not enough. They recruit the very poor households to the program, but when you compare the support given by the safety net program with the other (“hitsuts”) it is like the difference between the sky and the land. Households who have some food shortage are included in the hitsuts program, but the households who do not have anything are included in the safety net program. But when you compare the support given for both; the safety net program supports only for six months, but the “hitsuts” program supports too much.

I: What about after six months? (For the safety net program)

P: They are supported only for the first six months of the year, but the program is sustainable for some years. But, the other one (those supported by the “hitsuts” program) gets too much support.

I: Who are eligible for the “hitsuts” program?

P: It is the support given for the community because of the drought, but the safety net program is given for the poor people whether there is drought or not.

I: Good, do the pregnant women get the food support from the safety net program for free or through working?

P: They are given for free

I: After how many months of pregnancy?

P: I think it is after four months, but I am not quite sure. I think they don’t let them to work if the provided them the pregnancy certificate regardless of the months of pregnancy.

I: As you now the pregnant women should take rest. From your observation, do the pregnant women of this community take rest?

P: They don’t like to work even those who have one or two months of pregnancy. They bring the pregnancy certificate from the health facility if they know that they are pregnant.

I: What about their workload in their home?

P: They do everything at their home. There is no anything a mother can’t do at home. There is no workload reeducation due to pregnancy at home.

I: Do they get counseling about workload reduction?

P: Yes, advice is given to reduce workloads, not to lift heavy equipment and not to engage in woks that affects their body. But they don’t implement it.

I: How do you describe the counseling about ITN utilization by the pregnant and lactating women?

P: If there is shortage of ITN, priority is given for the pregnant and under five children. Since this area is lowland and malaria endemic area, they are using it.

I: Ok, who are advising them to use ITN?

P: Only the health workers are advising them to use the ITN. But it depends on your approach; if you discuss together with the agriculture, education and water resources about the health issues, they are willing to share what they have to the community. However, if we don’t inform them what they are supposed to do, we will do it alone.

I: How do you see the deworming service in this community?

P: Deworming service is provided in every six months. Both deworming and vitamin A are provided every six months. Deworming is given for above two years children whereas vitamin A is provided for above six months children. The deworming and vitamin A supplementation are provided in line with our screening service. When we screen the children, we have complete information about them regarding when the child has taken it and then if the child didn’t take it, we give him during the screening.

I: Good. Is there targeted supplementary feeding service in this community? TSF is a special nutritional support (like Fafa, oil and other related support) for malnourished pregnant and lactating women and children.

P: Yes, as we have discussed before, those screed mothers are given monthly.

I: Good. What about vitamin A supplementation?

P: It is given for the delivered mothers and above six month children. We didn’t give it for the adolescent girls (greater than 10 years of age), but we have been giving it for the mothers immediately after birth and for the children above six months old.

I: Ok, It there school feeding service for students in this community?

P: Yes, but it is given lately after February or March, almost it is started at end of the year.

I: Do you mean it is given from around March up to the end of the academic year?

P: Yes,

I: Why do you think it is started at February or March?

P: I don’t know it

I: Is it given in every year?

P: Yes it is available every year, but it is started lately.

I: What do they provide in the school feeding? Have you seen it?

P: Last year it was a mix of peanut, rubbed wheat and rubbed maize. Fafa was given in the past, but now they are providing “titiko”

I: Good. Are there youth friendly services in this community?

P: we have not started it yet.

I: Why do you think there are no youth friendly services?

P: It is not planned

I: When we say youth friendly services, it could be related with the reproductive health services like family planning methods.

P: Yes, but it is not introduced in this community

I: Do the services available in your health post?

P: Yes, we have.

I: Which of the nutritional interventions we have been discussing (vitamin A supplementation, TSF, deworming, counseling to use ITN, Safety net program, water, sanitation and hygiene, counseling on extra meal for pregnant and lactating women, ANC and others) are successfully implemented in this community?

P: Almost majority of the interventions are successful. For example, there is no any child who doesn’t use vitamin A supplementation and albendazole.

I: What about the pregnant and lactating women?

P; Pregnant women don’t use vitamin A supplementation.

I: What about the lactating women?

P: It is only given immediately after birth, but it is not given months after delivery because the mother could have pregnancy. So, it is given immediately after birth for the mother and after six months for the baby and every six months then after. The mothers have already adopted it like any vaccination and they push you by themselves to give them the services. They tell you that my son has reached six months of age, but you forget to give the vitamin A. similarly ANC is good, but they don’t come by themselves. For example, no women come to the health facility for the antenatal care follow up by disclosing their pregnancy by themselves. We are finding them through going home to home and through communicating with the women development army. They diagnose for their pregnancy through going home to home assessment. So, they don’t come to the health facility by themselves.

I: what other interventions are successful in the pregnant women from the interventions that we have been discussing?

P: I have told you that there are no youth friendly services and there are problems in the sanitation and hygiene because there is shortage of water access in the community. It takes them more than four hours to get water. However, the other services are successfully implemented; especially they have better awareness in vitamin A supplementation than us.

I: What about the feeding of pregnant and lactating women?

P: They have good awareness about their feeding, but there are gaps in its implementation. For example, if a mother has eaten enjera with salt, we can’t say the mother has eaten food. So, the women have lack of access for food, though they have good awareness.

**Section 4: Implementation challenges and community factors affecting access to nutrition**

I: Good. What do you think are the main challenges for the implementation of the nutrition interventions for the pregnant and lactating women?

P: One, there is shortage of water. We know that how much a women needs water. The water source is very far and even the available water is not clean (it is river water). Second, there is drought.

I: What other challenges are there?

P: The others are good and the community culture is changing.

I: for example, let us see the challenges in three levels; are there individual related factors (like related with their educational status or awareness) affecting the intervention implementation?

P: The women have good awareness because we have been meeting with them from the very beginning, but they have food shortage. If they don’t have the food, you can’t identify other obstacles for not using balanced diet. As I have told you before this community has been suffering from drought for the last seven years. It could have been easily to identify the implementation challenges, had they have enough food at their home. So, there is food shortage in this community. Starting from this season, all males will go for fishing and only the women remains in their home. The husbands buy some food for their family by working and selling fish.

I: Do you think the educated or uneducated women are challenging to you for implementing the nutritional interventions in the community?

P: The educated women quickly accept what you advise them to do. For example, the housewives who stopped their education at 5^th^ or 6^th^ go ahead of you when you advise them. Even they maintain their hygiene and sanitation without giving them health education. On the other hand, there are uneducated women who do not wash their cloths despite you repeatedly advised them to do so. Therefore, the educated women usually rapidly implement the advices, whereas the uneducated women have low awareness.

I: Ok, what are the communities’ related challenges (related with their attitude, culture and traditions) for implementing the nutritional interventions for pregnant and lactating women?

P: They have better awareness when they talk with you, however they don’t implement it. The community culture gives favor for the men. Most of the women prepare and give meal for their husband before they feed for themselves, but there are few men whom take care of their wives. There are some husbands who have low awareness about maternal nutrition. They should at least follow whether their wives have eaten or not. We have been discussing with them that their wives should at least equally eat with them and we also advise them that the women is expected to feed two to three extra meal.

I: Good, do the women have access for the services we have been discussing?

P: What services?

I: The services are the interventions that we have been discussing such as, ANC, counseling services, the health post by itself and others.

P: Our health post has big problem. The problems are; it is old and made up of mud, and it is also dusty and the setup is not convenient for the customers. Even though we have enough materials for antenatal care follow up, we have shortage of materials for the services given after delivery and we also don’t have sterilizer. If there is no sterilizer it is very difficult to work. We don’t have stove and we also don’t have the sterilizer, but we have other materials. If we face delivering mother, either we assist her delivery by wearing glove or we refer her to yechila. So, the health post is not built in a convenient place, there are also rats and snakes around the building. So, there are uncertainties in the health post.

I: Ok, how do you see the quality of services given for the pregnant and lactating women? Not only in the health post services but also on the other services we have been discussing?

P: This kebelle is very wide and it is very difficult to get the mothers timely. The pregnant women who failed to come to the health post for delivery might be missed because of its wideness. For example, if I missed to provide the antenatal care service of a pregnant woman, I have to go to her home to provide the service, but it is very difficult to go to the pregnant women’s home without accompanying person or guard. Due to the very long distance, I am expected to beg the agriculture expert or Kebelle leader to travel with me. The area is also very hot and as a result the women many not get the required services.

I: How do you describe the commitment of the providers (like the HEWs, women development army or others) for improving the health and nutrition of women?

P: As far as we are here to serve the community, we are expected to provide the services with commitment, but are provides are not serving them. There are no any providers who have been working like the women development army. If the agriculture sector were working similar with what the health sector, we could have improved more than the current status. However, the sectors are not working in collaboration, instead they are independently working. We don’t work together; rather we just send the information for each other like a message. For example, I myself do not own the agricultural works, rather I send messages for them and the same is true for the agricultural workers.

I: Good. Are there other barriers for the implementation of nutritional interventions?

P: there are no other interventions except wide geographical area, unclean health post, lack of water access and drought.

I: Ok, what has been done to solve the challenges you have been mentioning?

P: there are no solutions for geographical setting, whether, and water shortage related problems. The health office is expected to solve the problems related with the health post and we are also struggling to provide sufficient services for the women.

I: Are there challenges related with TSF support?

P: They are coming by themselves to take it. After screening the mothers, we bring the eligible mothers to this facility. During provision, we advise them that the Fafa is a treatment, how to take it, for how long to take it, how much to eat per day, how to prepare it and when to come back to health facility.

I: Do you think the Fafa given for the mothers is taken by them?

P: No, they don’t eat alone. The mother cannot eat without sharing to her children. I will not belief her if she tell me that she ate it without sharing to her family. We are transferring the message but they don’t implement it.

I: sharing the food given for the women is a challenge by itself. What has been done or what should be done to solve this problem?

P: Anyhow, we have started to implement some solutions. For example, the Plumpy’Nut for under five children is given daily for a month.so, we make audit at mid of the month. Similarly, the Fafa is taken by ‘finjal’ (a coffee cup), and we can audit it at the mid of the month. It is also possible to check the quantity porridge prepared by its cooking equipment. We should not be nervous on them, rather we have to joyfully and systematically discuss with them. When I didn’t get 15 Plumpy’Nut while 15 days are left, I have to ask her why it is incomplete and I should advise her how such kind of malpractice could affect the health status of her child.

I: Good. What about for the foods given for them?

P: it is similar. Even though it is difficult to measure, we can estimate by the cooking equipment. We can also ask about how much is consumed, how much is left and we can also check the quantity of porridge prepared for this day. We can advise her to decrease the quantity of the porridge. We also advise her that if she doesn’t properly used it, her pregnancy will also be affected. But, we have not started this kind of advice for all women.

I: As we know our mothers are very kind. As you have told me, mothers give their food to their children and husband. Is there an advice given for the husbands to support their wives to use the fafa for them?

P: We didn’t give counseling about fafa for the husbands. However, if the fafa is completed ahead of time and if the husband is available during our home visit, we advise them together. We tell them that if you give this medication for another child, the malnourished child is not taking the prescribed medication and he will not improve from his illness. Therefore, there is no advice given for the husbands because, we trust the women and their husbands don’t usually see what is going on at home. However, when we suddenly meet their in their house we advise them together.

**Section 5: Multi-sectoral collaboration to improve maternal nutrition**

I: As you have told me before the agriculture is working for improving the nutrition of mothers. So, do you think different sectors should collaborate for improving the nutrition of woman?

P: Yes, unless they helped them by educating about the resources use, it will be difficult to implement the services. For example, the animal resources talks about the important animal products important for the women, the seed production expert also talks about the important seed they should cultivate and the health providers also talk about the women health. If there is multi-sectoral collaboration, the community will be changed.

I: Good. In addition to what you have mentioned, what sectors should collaborate for improving maternal nutrition?

P: Mainly it will be more important if agriculture, water resources and health collaborate to improve maternal nutrition. But, it is also important to include the health sector.

I: What else?

P: It is enough. If the education are working on the students, and if the agriculture and health are entering to the households we can improve the maternal nutrition. Most of the time, the health, agriculture and water enter to the households. Therefore, if we collaborate and work in the households, we can make a change.

I: Good. How do you describe the current collaboration between the health, water, agriculture and education in improving maternal nutrition?

P: As I have told you before, we all wants to aggressively work independently in our respective sector. Despite we complement each other; practically we believe that the other sectors are different and unrelated offices. So, we don’t struggle to collaborate together, instead we transfer messages as if we are not working for similar purpose. However, Tigray Relief and Rehabilitation Society was giving us a joint training with the sectors and we ( the health, agriculture, education and water resources) were working together to implement what we have trained together.

I: So, you mean that if Tigray Relief and Rehabilitation Society stopped the joint training, you don’t collaborate for improving maternal nutrition?

P: Yes, we only collaborate to implement our temporary assignment because we all want to aggressively work in our respective sectors.

I: Do you have a platform/system/ to work together?

P: we don’t establish the system to work together at Kebelle level. The sectors are centralized at woreda level (to mean that they have common working system at woreda level). The woreda administrator leads all the sectors. So, if the woreda administrator strictly manages the four woreda level sectors (the health, agriculture, education and water resources) and if he asked them what they have worked not only in their respective sector but also in the other sectors, the woreda level sector offices will strictly monitor us to work in collaboration. There is disintegration from the top down and they all are only concerned in their respective offices. So, the sectors are only accountable for what they have done in their respective offices. The problem might have started from the region or woreda.

I: Are there good opportunities for the multi-sectoral collaboration?

P: Everybody talks about collaboration and we all know that collaboration is important for all of us, and we also know that we will be successful if we work in collaboration, but we are not implementing it due to lack of common working system, communication and follow up. We are working independently in our offices. They (the woreda administration) tell four of us (health, agriculture, education and water resources) to work together. But the woreda “cabine” directly come to the Kebelle agriculture office (it has three experts) and critically evaluate their performances throughout the day. But they don’t ask what the health sector is doing and as a result we get disappointed. They don’t give time for evaluating the health related woks as much as they give time for the agriculture. But, the health sector should be given priority because we can only work in the agriculture if we are healthy. So, the woreda administration gives more priority to the agriculture. Health is our biggest resource, but the administration does not recognize this fact. So, the coordinating system from the top to the bottom is not steady.

**Section 6: Other interventions that influence adolescent and maternal nutrition and health outcomes**

I: How do you describe the benefits of delayed birth (after 18 years of age) for the mothers’ nutrition?

P: “What? what”?

I: What are the benefits of delayed first birth (after 18 years of age) for the women’s nutrition?

P: If the woman delivered after her 18 years of age, she will be strong and mentally ready for delivery. However, if she is below 18 years of age, her body will not be ready and her uterus will not be stretched and will result in difficulty for child delivery, and psychologically she will not be ready for child development. If the mother delivered her first child after 18 years of age, she will not have a problem to develop and feed her child.

I: Good. How do you describe the education given for the community to delay first child delivery to after 18 years of age?

P: Nowadays, the community awareness on the prevention of marriage under 18 years of age is increasing. If there is under 18 adolescent girl proposed for marriage, she will be seized by the women affairs and will be transferred to the Kebelle for further examination. He her age is proved to be under 18, the marriage will be cancelled. So, if the girls don’t marry in their early age, there will not be early pregnancy. For example, the marriage of two under 18 years of age girls have been cancelled last year after they wasted their resources/festive meal/. Currently, the women affairs are strongly working to prevent early marriage.

I: Good. How do you describe the benefits of birth spacing for improving the mothers’ nutrition and health?

P: Birth spacing helps both the mother and her child to become very strong. Birth spacing benefits the mother to have physical strength and also it helps her to have strengthened uterus. However, if she gives birth after birth, she will not get enough food because she will not have enough time to work for feeding her children, she will not be also physically strong.

I: Good. What are the activities being undertaken in this community for the women to increase birth spacing?

P: We have been providing the health education and family planning methods.

I: What polices and strategies are in place for increasing birth spacing?

P: We have been providing health education about birth spacing wherever we go and we have been also providing the family planning based on their choice. We don’t have another policy apart from this.

I: How do you describe the influence of religious institutions for birth spacing?

P: There are no challenges from the religious bodies about birth spacing.

I: What about for the influence of religious institutions for using family planning methods?

P: There are no challenges from the religious institutions except some of the mothers may not take the family planning methods timely due to very wide setup of the Kebelle. They don’t get the services timely because some of them are living very far from the health post and they need somebody to come with them because they cannot come alone from the very far distance (they are expected to travel for more than 2:30 hours). However there is no obstacle from the religious bodies, even we have been providing the health education about family planning in the churches together with the religious leaders.

I: As you have told me before the women affairs and other government bodies are strongly working to prevent early marriage. What is the attitude or understanding of the community about early marriage?

P: The women affairs alone are not identifying the girls proposed for early marriage, but the community by itself is identifying and reporting them for the women affairs. Then the women affairs further struggle to prevent it in collaboration with other responsible bodies. The community is reporting about the planned early marriage because they understood that it is bad culture.

I: I hope you know closely what is happening in the community because you are very near to the community. So, what things should be improved in the prevention of early marriage and bith spacing?

P: In the past there were trained and experienced family planning providers in each “kushet” (got), but they are not available right now. For example, they were providing family planning methods like the injection method for three months and the tablets. So, it will be very important to develop such kind of experienced providers in order to provide the services in their nearby community.

I: As you may know, in some areas, there are youth friendly services which help the youths to get their preferred services in their preferred place and by their preferred providers. So, are there youth friendly services in this community?

P: It is not available and I don’t know how it has to be established. I know it is available in some health centers, but I don’t know it should be established here. As you said there are schools in each “kushet”. It could be good not only for the students but also for the mothers, if the youth friendly services are established in the schools, because even the very far communities have schools.

I: As we have been discussing, despite there are no nutritional interventions for the adolescent girls, they are at risk for malnutrition, especially they are stunted and underweight. So, what should be done to prevent the adolescent girls from malnutrition?

P: If it is supported from the top and if highlight is given on how to implement it, then it will not be difficult to implement it. However, highlight should be given on what, how, and when to do it.

**Section 7: Additional remarks**

I: Finally, as I have told you in the very beginning, the main objective of this study is to identify the implementation and access barriers of pregnant, lactating and adolescent girls’ nutrition. So, if you have any suggestion about the main challenges for pregnant, lactating and adolescent girls’ nutrition?

P: The main thing for improving their nutrition is that the activities should be administratively centralized from the region up to the Kebelle level. If there is organized multi-sectoral collaboration, let alone nutrition many more things can be done. However, if we work independently, we will only focus to our own works. However, if there is centralized administration from the region to woreda and Kebelle, the woreda will be accountable for the region and the Kebele administration will be accountable for the woreda administration and all sectors in the kebelle will be accountable for the Kebelle administration and as a result we will work in the same level. Otherwise, if the health, agriculture and education work independently, they will not be effective.

I: What things should be improved from the community side?

P: We are talking in the area where uneducated community is found. If there are problems in the community, we have to educate them. Therefore, I don’t have anything to say about the things that should be improved from the from the community side. But, it is better to establish the youth friendly services in the community. Since we are taking about the uneducated community, there is no anything the community could improve because they can only improve it if we educated and followed them.

I: If you have any additional suggestion?

P: I don’t have.

P: Thank you very much for taking your time to participate in this study.

**Summary**

Section one: Common maternal nutrition problems in the community

- The community is food insecure due to repetitive drought
- A woman should travel more than four hours to get river water for drinking
- There is regular screening for the pregnant and lactating women and the under five children
- Chronic malnutrition is common in the women of this community
- Stunting and underweight are also commonly observed in the adolescents
- Culturally the women do not eat before her husband.

Section two: Interventions that improve adolescent nutrition

- Every pregnant women attends ANC in this community
- Beyond counseling, there is monthly demonstration and follow up of balanced diet preparation.
- There is no home gardening at all due to water shortage and low awareness of the women
- There is no nutritional screening and interventions for the adolescent girls except the rare school feeding.
- TSF is provided for the malnourished pregnant and lactating women

Section 4: Implementation challenges and community factors affecting access to nutrition

- The main challenges for the implementation of the intervention are repetitive drought, water shortage and inaccessible geographical area (very wide and far from the health post).

Section 5: Multi-sectoral collaboration to improve maternal nutrition

- The health, agriculture, education and water resources are working maternal nutrition and health, but have very poor collaboration

Section 6: Other interventions that influence adolescent and maternal nutrition and health outcomes

- The community has good awareness about early marriage and birth spacing,

Section: seven: additional remarks

- Multi-sectoral collaboration should be improved through improving follow up and accountability
- It is important to have trained family planning providers at kushet level to create access for the women living the periphery
- It is also important to establish youth friendly services in every schools.
