# Supplementary material for: Barriers to utilize nutrition interventions among lactating women in rural communities of Tigray, northern Ethiopia: An exploratory study
Source: PLoS One. 2021 Apr 30;16(4):e0250696. doi: 10.1371/journal.pone.0250696 (PMC8087028; doi:10.1371/journal.pone.0250696)
Supplement: S2 File — (ZIP) [file pone.0250696.s002.zip › S2_File.Doc/Community level Key informants/099_IDI_Religious Leader_Felege Hiwot kebele_Tanqua Abergele woreda.docx]

**Operational Research on Adolescents and Maternal Nutrition in North Ethiopia**

**Introduction**

**Tool B: IDI with Religious Leader**

**Introduction**

| **Introduction:**  Hello, my name is Yemane G/mariam. I am from Mekelle University. Thank you for taking the time to speak with me today. We are doing research on the factors that influence the nutrition of mothers and adolescent girls in collaboration with the Regional Health Bureau and UNICEF. Your participation is very valuable. The things that you tell us will be used to improve nutrition programs and services for women and adolescents in the region and the country. We will not share your names when we report our results.  However, we will record the discussion so that we can capture all the ideas that are shared. We have several questions to ask you that we have prepared in advance, and we will ask you all to say what you think about each question. To ensure the privacy of everyone here, we ask you not to repeat what we discuss outside of this group. The discussion will last for 1-2 hours. Do you have any questions before we begin? If you think of any questions as we proceed, please feel free to let me know. If it is all right with all of you, we will turn on the tape recorder now.  Do you agree to participate in the study?   1. **Yes** 2. No   **Section A: Interview details**   1. Zone:__**Central Tigray** 2. Woreda: ---**Tanqua Abergele** 3. Kebele: **Felege Hiwot** 4. Facilitator’s name: --**Yemane G/mariam** 5. Date of discussion: ----**Nov, 15, 2017** 6. Discussion start time: **4:00 Morning** 7. Discussion end time**: 5:06 Morning** | | | | | |
| --- | --- | --- | --- | --- | --- |
| **Section B: Socio-demographic Information** | | | | | |
| Name of FGD participants | Age | Marital status | Education level | Occupation | Informed consent/signature |
| 1. Priest Tekele G/mechael | 40 | Married | Priest can read and write. | Priest | Obtained |

**I:** Interviewer **P:** Participant

***Section1: Common maternal and adolescent girl’s nutrition problems in the community.***

***I: What do women do to stay healthy in this community/woreda?***

P: Since our area is malarias and sunny women like pregnant and lactating mothers go to health facility to get medication and monthly follow up on physical and clinical examination. In this area you may think of as you are health but you may suffer of headache, fatigue and tiredness this is happened due to anemia. Therefore pregnant women get tablets to treat anemia and have follow up. For example my wife has given births but she was suffered anemia while she was giving birth and has taken three units of blood during delivery in Ayder hospital and treated accordingly; this is my practical experience. Therefore pregnant women can be exposed to anemia due to malaria and sunny. Previously most pregnant women were giving birth at home since they believe in St. Merry and God and didn’t trust the medication the health facility but now as a church we are teaching them to give birth in the health facilities. Physicians or health worker are working to save life based on the well of God therefore every pregnant women should give births in the health facilities. For example I am priest when my wife start labour we call to ambulance and we go to Yechila (35 Km away from their Kebele) on our foot and from Yechila to Ayder hospital by using Ambulances.

They (Health workers) told us she is anemic and I think this is due to malaria, sunny and malnutrition. We didn’t feed the pregnant women due to our low capacity in terms economy even though we believed that pregnant women should get additional food especially starting from three till 9 months. In addition they are busy in fetching water during sunny time, workload at home and caring of their children till they go to school even if after doing all this they will go to keep the cattle. This led them to be malnourished and even though we believed they have to eat different food we don’t what are they. For example we have four cattle in our home the mother may drink milk even though the amount of milk is reducing. They eat only injera and milk if it is available in the home. Personally I believed all this happened is due to malaria and sunny. Even though there is no death due to birth related issues since our government has provided us ambulance and health facilities.

**I: What are the reasons for pregnant women not to eat properly?**

P: This is due to our capacity is poor to prepared foods that can help her to get strong and well nourished. Let alone our wife we will be so glad if we could feed others but we have don’t enough amount of food to feed them. Due to this she eats what her families eat and what they drink.

**I: Is there any food support given to pregnant women in your community?**

P: Till now we didn’t see any food support given to pregnant women but Fafa and oils are given by health extension worker once they are measured their weight, height and MUA (showing on their upper arm) for those who are very thin on weight and anemic. But this was given only for small amount of pregnant not to all of them.

I: **Who measure and give the fafa and oils?**

P: The HEW working in this health post measures and give the fafa and oils.

**I: What is the purpose of having Fafa and oils?**

P: Yes it has importances, it help the mother to gain weight since the fafa comes monthly and the HEW measures the women if they get improvement or not in terms of their weight. Once they get the fafa and measured their weight after one month some of the pregnant women will not be given the fafa. Practically I don’t Know why they decided since it is has meaning by the HEW even though we couldn’t see the change why they terminated to get the fafa and oils. We have discussed in different council meeting, this pregnant women are coming here since they are poor therefore why don’t we support them the fafa once they screened and get malnourished till the birth. As we know since they are human we couldn’t expect them (HEW) to be perfect they may have their own limitation in distributing the fafa and oil.

**I: What are the common nutrition problems among maternal and adolescent girls in your community?**

P: The common nutrition problems symptoms are swelling of the legs and when you touch it deepen and refill again when you remove your hand this shows or indicate the presence of anemia. Health workers also treat such kind of problem by saying this anemia and malnutrition and start feeding to the child or the women. I myself can understand the sign of anemia by looking the eye if its color become greenish and body become swell this show that it is due to anemia and malnutrition.

**I: What are other nutrition related problems like goiter and its causes?**

P:Yes, I know goiter or swelling of the neck but this is not common in our area but rarely it may happens but I don’t have detail knowledge on it and its magnitude and don’t know the causes of it.

**I: What do you think on why women/girls in this community would not increase their height proportional to their age?**

P: For example health and nutrition are not independent of each other’s they are dependent. Unless you give care for the child at the beginning once he get damage whatever you try to support him it is very difficult to bring it to the normal situation. When the child reaches to expose sunny and to support or help his family he couldn’t tolerate all this. I think this due to malnutrition at their child stage but if the child was grow with care and good nutrition no problem at all. But once they become stunted it is very difficult to gain the height rather they can gain weight when we improved the feeding practice therefore to reduce any stunting we have to do more during child stage except for those who are created natural with deformity which leads them to be short.

We can take an analogue of cow, when a calf well fed the milk of his mother and the one who don’t fed well his mother’s milk; the one with good milk feeding will get strong during fight as compared to the one with less breast feeding. The same is true on the calf; if the calf feeds less milk cannot grow widely but once the calf reach adult age and eat enough amounts of grasses and water the calf can gain weight but not height.

**I: What are the barriers and challenges of women not to eat/feed properly in your community?**

P: Most of the women are thinking o f their husbands, children and their families that is why they didn’t eat properly. But as a husband I told her to eat properly by saying if you are sick due to not feeding the night become darker in the home therefore you must eat well to make safe our family. But she refused to eat by saying “you’re (Husband and children) the one who get tired due to farming and other works I am so fine” even though she is hungry. But we couldn’t forced her to eat therefore the most actor who played in their nutrition intake is themselves. For example they didn’t eat till the husband come even though we recommend them to eat on time if we don’t come. I realized that if she gets sick I am the one who will get challenge as well as get sick. When we come to lunch or dinner on time no partiality we eat together.

**I: Do you think women are at risk of malnutrition?**

**P:** Yes, when we compared their work with their feeding it is incomparable; they expense lot energy and don’t replace it. For example when a woman gets pregnant and gives birth she has expensed a lot of forces and energy but if this is not replaced she may get malnourished and anemia. The problem is even though she released that she needs to eat during and after birth but no food in the house and the challenge will continued. For example, if I have I may provide her goat to eat during and after pregnancy but if I don’t have what I can do therefore her fate is determined on our capacity. The challenge is most of the time is due to our low capacity but sometimes even though the food is available they prefer to give it to their husband and children.

**I: Is there any food insecurity I your woreda?**

P: Yes; Our woreda is the most affected are of draught, thanks to our government this woreda is dependent on government support from year to year. For the past ten years our woreda is affected by draught due to shortage of rain or water. Especially during 2001E.C after we plough the land there were no rainfall. The same was happen in 2008 E.C except little amount of grasses were grow which serves as food for cattle. Where as in 2009E.C it seems better but most the farmers were not productive due to the shortage of rainfall. In 2010 the crops were affected by pests or insects which affect the production of the maize when it is ready to give fruits the pests were eating its leaf. Previously if the land gets one rain immediately it heals and become productive but now even if it gets rain once or twice it is not becoming productive. We are worry why this is happening. Now we realize when seed reach to give their fruits, no fruit at all when we saw it around 10 pests were observed in the maize’s. Due to this entire factor the production has been reduced in our woreda.

**I: How do government support you the food in the woreda?**

P: There is what we call safety net, they support by giving wheat 15kg per individual per months. This is given by different methods like food for work (daily working), for poor elder and during emergency preparedness if there were draught in the woreda they provide as food after they screen which farmer is really affected. This is mostly done during January once screening is completed they give us for six months.

**I: Is there any nutrition support for pregnant and lactating mothers in your community?**

P: No, nutrition support given to pregnant and lactating women except I told you previously which is given by HEW like fafa and oil after nutrition screening.

**I: Do you think the support given by the government is enough?**

P: First of all the effort of our government and should be appreciated but 15 kg wheat per individual for one month is not enough.

**I: When do you face more shortage of food in your community?**

P: We faced more challenges or shortage of food during the month of July to November. There is proverb which describes how the food shortage is common from July to November “the previous product ended and the coming production is too far.” In Amharic “ የፊተኛው አልቆ የሃላኛው ርቆ .”

**I: Do you think adolescent girls are affected by malnutrition in your community?**

P: We couldn’t know whether they are affected by malnutrition or not since they are running with us here and there, but they may be challenged during pregnancy and labour even though we don’t what is going on inside their body. Our limitation is we didn’t discuss focus about their nutrition since they eat what we have in the house. For example I have a daughter with age of 19 married one when she get pregnant she was following ANC in the nearby health center in Amhara region namely “Embadegue” due to their better service as compared to our health center but there were no difference in her feeding before and after pregnancy it was as usual. In addition we were closely following the health center till her birth monthly with me and her husband. She was taking drugs/tablets for her anemia and finally counseled her to come and give birth in their health center. Since there is no road for ambulance transportation; we called and go to Yechila health center and gave birth in the health center with no problem. But there is no enough support in the health center during and after delivery. Previously we were paying 10 birrs for ambulance and 5 birrs to feed mother after giving births but there were no services given to the mother after birth rather people who come for delivery give you different support like soup and porridge.

There is what we call “Seraqin” synonyms with thief of life, in my understanding when we say “Seraqin” it is associated with excess blood lose during labour and the mother become anemic, dry mouth and her tongue retracted and block the breathing system which lead her to die this is due to anemia and malnutrition but most of the mother think as this is “Seraqin” known as devil spirit most of the time this happen when the mother give birth in the home. But if the pregnant mother gives birth in health facility she can be safe of excess bleeding and even if it can be treated and replaced the lost blood accordingly.

**I: What are the different services given to pregnant, lactating mother and adolescent girls?**

P: They get tablets which help them to prevent anemia and the HEW give them (Pregnant and lactating mother) like fafa and oil but nothing else is given.

**I: You have told me that you wife and child were visiting health facilities what are the services they received?**

P: I didn’t know what service and treatments were given but they measured their weight and give them tablets and told them that they are improving their health during the different visits. I don’t know the details but I perceived the drug helps her to replace the blood and serve as foods.

**I: Did they get counseling on nutrition during pregnancy**?

P: Yes of course; especially when we followed the ANC in the Amhara region they were give us good care and counseling. For example let me tell you my experience we didn’t follow ANC for the first pregnancy of my wife and she was suffering a lot due to malnutrition and anemia. After we referred from Yechila to Ayder hospital and she was received three units of blood from this experience we were followed for ANC for the second birth of my wife and the first birth of my daughter since I realized the importance of having follow up. I was not using the nearby health facility simply we were using the service of Amhara region Health center.

**I: Why do you prefer to use the services in the Amhara region health center?**

P: I perceived the service given is better than the service given by our health center and in addition the husband of my daughter is from Amhara region that is why we were following in their health center.

**I: What can you tell me about Water, Sanitation and Hygiene of women in your community?**

P: When we say keeping sanitation and hygiene it is more related with HEW since they told us to use “Wuha Agar” or Chlorinated water to drop in the water because the water we brought from the river is not clean so when we add it in to the water it helps to clean the water as they told us.

I: **Do you think malaria is common among pregnant women?**

P: Yes it is common; when someone get hunger or don’t eat for long time immediately the malaria will be relapsed on everyone. But if you eat well you will be safe from malaria even if during September therefore the prevention mechanism is to eat well as to my perception.

**I: Do you use ITN in your community?**

P: Yes; the supply of ITN is interrupted and we are using it partially since most the women used the ITN for other purposes like to cover for their hair and to cover for chair and tables. Those who have bed are using it properly where us those with traditional bed “Medeb” are not using it properly. Currently we have received ITN but since the season is hot we prefer to sleep out of the door without using ITN. I perceived the ITN distribution is enough for example we are 7 families including the new baby they gave us three ITN.

**I: Do you use Iodized salt?**

P: Yes, we used Iodized salt since it is affordable even though I don’t know its importance the HEW tell us to use/pour it after the end of cooking but sometime we used the non iodized salt.

**I: What are the barriers and challenges that you perceived related to maternal and adolescent girls nutrition in your community?**

P: I couldn’t say nothing since the government is trying all best even thought couldn’t address the issues of maternal and adolescent girl’s nutrition I think it may due to the capacity of the government. But I believe pregnant and lactating need nutritional support even though they are not well supported when I compared pregnant mother need more food support than lactating mother. Showing sad face, we are working up and down in all our best to feed our family but we couldn’t solve it and the government is also facing the same challenge in which the nutrition problem that couldn’t be solved by us is difficult to be solved by the government.

Previously for those less than five years children fafa were given but now it has totally stopped for the last three years.

**I: Is there any nutrition intervention related to adolescent girls in your community?**

P: I don’t know. But three years ago adolescent girls were given tablets to prevent anemia and to help them during pregnancy after this I didn’t see any intervention related to adolescent girls.

**I: Do you think pregnant and lactating women and adolescent girls need rest, visiting health facilities? Why?**

P: Yes: because due to high workload she faced difficulty during labour. Not only in workload have they needed to be supported in all things. Feeling sad in his face, described that “we really very sad and don’t have power to see them with our necked eye when they (pregnant women) come nagging their head and has fast breathing due to lack of energy.” The pregnancy is not proportional as compared to their body weight and power to carry the fetus due to the poor life they lived. We see them with our necked eye only when they give birth.

I: **What must be the role of Husband in supporting his wife?**

P: The role husband must be reducing workload by exempting her from farm work, especially starting from four months of gestational age he must reduced her burden as government do for pregnant women to get maternal leave before giving birth. Now the burden on pregnant women is reducing in addition the husband must support her in bringing food for the pregnant but as I have said previously there is shortage of capacity. The mother is all the time busy at home as well as in the field (farm) therefore reducing of burden is very important since she will be challenged during delivery.

**I: What is the difference of feeding practice before and after pregnancy and lactating mother?**

P: There is difference in feeding practice before and after pregnancy, especially the feeding practice is less during pregnancy this is due to less appetite even though she has become interested to eat some food since she know the shortage of her house; may not ask her husband to bring what she like to eats and faced malnutrition. At this time we become busy in thinking of her, by advising please you have to eat unless it is not good for you and the baby. Rather the food intake is better during lactation than during pregnancy since they fill hunger they eat more even though the challenge is we couldn’t provide them with necessary foods due to our low capacity.

**I: Is there any food taboo in which not allowed for women?**

P: There is no food restriction for women; but Alcohol is not recommended especially during pregnancy. Previously women were not eating their lunch till her husband come but now we are telling them to eat if we are not available at lunch time. May be the meet inside the bone marrow is not allowed to be eaten by the women as culture and they themselves don’t eat it but I think this may help them to prevent anemia.

**I: Is there nutritional screening in your community? What is the purpose?**

P: Yes; the HEW measures their weight, height and MUAC (showing to their upper arm) and they give fafa and oil based on their measurement for the pregnant and lactating mothers. But when they distributed there is partiality in treating the women more they give for their neighbors.

The other challenge is when a mother gets fafa for herself or her child they give it to all the family or they share the food in the house hold.

**I: How can be solved the food sharing especially the fafa?**

P: The husband must forced her to eat for herself by advising her that she is the one who gets malnourished therefore she must to eat it properly. We must consider the fafa as tablets or drugs if so we couldn’t share the fafa as to that of others drug ordered to someone. But this is not practically implemented all the fafa and oils were shared by the family especially for children.

In addition plump net is not accepted by adults for its test therefore it should be given to children and the women to reduced sharing even though it may be difficult to reduced sharing among the children.

**I: Do you know who should get fafa and plumnet?**

P: I don’t know.

**I: Do you think women need special food support? Why?**

P: Yes, to make them to have strong body and can tolerate labour.

**I: What are the implementation challenges and barriers that affect women nutrition in your community?**

P: When we come to fasting a pregnant woman is expected to fast based on our religious but if she gets difficulty on her health the priest may allow her to eats whatever she likes but this is not allowed for all pregnant women rather it is allowed only for those pregnant who get sick and malnourished. But for lactating mother she can eats any time even if in fasting time.

**I: Do your wife were fasting while she was pregnant?**

P: Yes, she was fasting even if I told her to eat; she was fasting just like me for nine hours. But I decided she must eat during her pregnancy and consulted our father priest. And the priest punished me to fast and pray for her while she gets permission to eat whatever she like.

**I: What is the importance of having above 18 years marriages and birth spacing?**

P: It is clear and understood; if she gets married less than 18 years of age since her body is not matured, she may face difficulty during labour. If the penis of husband is not compatible with her body size may create fistula. It is true if female reach at age of 15 may show developmental stage of maturation like menstruation, enlargement of breast and become beautiful but the government has give direction the cutoff point to be above 18 years is mean of all women I think this is decided based on the merits of women therefore I don’t have any objection to this.

**I: What if the age of marriage is above 18 years**? **What is the importance?**

P: Laughing, above 18 years no restriction at all. Her body is matured and can be ready to get pregnant and give birth without complication. This information is given by the kebele leader and HEW together with religious leader in the church. They advise us to follow and report an early marriage and pregnant women while we go to teach our children home to home to inform the pregnant women to give birth in health facilities. I can say the information is reaching to the community but it lacks close follow up for its implementation.

**I: What are the interventions in your community to prevent early marriage?**

P: In our woreda there is close follow up on early marriage for example last year every marriage were get approval before the wedding happen and if she is under age money wedding or marriage have been cancelled even if food and drinks are already prepared. Medical result will be presented as evidences for her age regardless of her family witness. HEW and WDA if they have doubt on her age they call police and take her to health center to check the age before marriage. Based on this there are adolescent girls who have gotten permission to get married as well as some of them were refused to get married due to their under age.

**I: There is some rumor that family is getting married their children even if less than 18 years.**

P: Of course it may happen, but the community should understand that the government is bringing rules and regulations for the benefits of the community. Sometimes they are sending their daughter as holiday of saints like St.Mechael. But there must be close follow once after they prevent the early marriage till her age reaches 18 years since the families are marrying after the police and social just have gone.

**I: What is the age of limit for marriage in the church?**

P: I cannot tell you the exact age but when Hewan was created from the side of Adam she was 15 years old but we don’t have any opposition if she get married at the age of 18 years old.

Regarding to priest and deacon when they get married she must be virgin and they must know each other and must loved each other since divorce is not allowed.

**I: What is the importance of birth spacing?**

P: Regarding to birth interval we see it in two perspectives; one according to our holy bible it doesn’t allow to have birth spacing but as our government told us its importance in terms of the mother and child. For the mother birth spacing gives her rest and build their body also it helps the growth of the child to be strong and no malnutrition with no restriction for breast feeding. But if the birth interval is narrow the child will share the food and the breast and they may not get enough amount of food. The mother will be busy in carrying and feeding her children.

**I: Do you think the religious don’t support family planning to space the birth interval?**

P: What is the difference between birth interval and family planning?

I: Explanation given.

P: In our holy bible abortion is strictly forbidden or not allowed it is equivalent to killing someone since the fetus get life they claim or appeal to God why our parents are killing us without knowing our fate.

**I: Family planning is preventing the conception of fetus it is not equivalent to abortion, do you allow family planning?**

P: Regarding to family planning women are using but as religious leader we don’t promote or refused its utilization rather we have neutral position.

**I: What do you recommend to prevent early marriage in your community?**

P: Police, HEW and social justice should work together with religious leader. If priest trained on early marriage and every priest to teach his children about early marriage and the police should take the accountability to implement the law about early marriage.

**I: What do you think the role of the church in promoting women nutrition?**

P: I can’t say nothing about this but the community should implement what is given by the HEW and WDA.

**I: What community days are in place to improve maternal and adolescent girl’s nutrition?**

P: I don’t know. I have not seen any discussion related to maternal and adolescent nutrition.

I: What do you recommend to be done on maternal (Pregnant and lactating mother) nutrition in the future?

P: During ANC they should implement the advice and take proper feeding, must take rest and after delivery all service should be available. For example the health worker is looking her current health status but don’t know what is going on once she goes to her home therefore home to home follow up is important after delivery.

Let me tell you my experience my daughter was coughing after delivery and they gave her drugs (Tetracycline) and send us to home but I asked them saying “ I am going to far village she coughing how I can be sure that the drug is effective?” and they gave me three different drugs. Once we come home and she was taking the drugs according to their order but the coughing was not reduced and I preferred to stop the drugs and give her with fruits and vegetables like banana and oranges and she get cured immediately. The reason why I stop the drug is she was vomiting and the drug don’t stay in her abdomen, you see the health worker don’t see what is going on in the home I was tried to take her to health center but she was not reach seventh day of her birth. The health worker may used it for report purpose to know the number of women giving birth in the health center but no follow up after they send them to home on their health, feeding and child health.

**I: What are the challenges/barriers in utilizing the services by women?**

P: The two kebele ; showing their hand; haven’t road access if we need any health service first bring carrying them for long time till this road in which ambulance can enter which delays them to get the services. When we call to ambulance they came immediately but they missed us and returned since we are on journey carrying the mother.

**I: Do you think ambulance are taking and return the delivered women to her home?**

P: Yes now they are taking and returning them till their home it is introducing now. For example my wife was come by ambulance from Ayder hospital and to Yechila and from Yechila to Felege Hiwot.

**I: Do you want to add anything in which we don’t discuss or any other?**

P: I want to say the most important focus should be given starting from child hood on feeding, starting from colostrums and breast feeding but once they are not well nourished even if we feed them properly later on they only increases their weight not their height. Therefore the effort must be focused on the child hood.

I: Dear Preist Tekle thank you for your time taking and discussion, I have learnt a lot from your discussion thank you again. If you have any concerns you can contact me any time take my phone number (my phone number given to her).

Thank you very much for your time and information

**Summary**

- Malaria and sunny season is causes of malnutrition for Women (PW and AdolescentGirl)
- There is nutritional screening for PW and LW and given fafa and oil
- There is shortage of food in the community
- Pregnant and lactating women are not user of the sift conditionality.
- Stunting and under weight is common in the community
- Faring of fafa and oils is common among the family
- The woreda is repeatedly affected by draought.
- There is productive safety net program in the woreda
- No nutrition intervention for adolescent girl
- Women are not fed properly they prefer to feed their husband and children,
- ITN is partially utilized even though there is enough distribution
- Iodized salt is used even sometimes they used non iodized salt
- Husband role to PW and LW in giving rest and reduced workload
- Women eat more before pregnancy than during pregnancy
- Pregnant women is expected to fast from 6 to 9 hours unless she is sick
- Close follow up is necessary to prevent early marriage and not supported by the church
- Abortion is prohibited by the church but neutral position for family planning.
