# Supplementary material for: Barriers to utilize nutrition interventions among lactating women in rural communities of Tigray, northern Ethiopia: An exploratory study
Source: PLoS One. 2021 Apr 30;16(4):e0250696. doi: 10.1371/journal.pone.0250696 (PMC8087028; doi:10.1371/journal.pone.0250696)
Supplement: S2 File — (ZIP) [file pone.0250696.s002.zip › S2_File.Doc/Community level Key informants/105_FGD-Men-Hatsebo Kebele_Laelay Maychew.docx]

## FGD for men of reproductive age using Tool B

**Introduction:**

Hello my name is Haftay. My colleague is called Yasin. We are from Mekelle University. Thank you for taking time to speak with us today. We are doing a research on factors that influence the nutrition of mothers and adolescent girls in collaboration with the regional health bureau and UNICEF. Your participation is very valuable. The things that you tell us will be used to improve nutrition programs and services for women and adolescent in the region and in the country. We will not share your names when we report our results.

However, we will record the discussion using and audio tape recorder so that we can capture all the ideas that are shared. We have several questions to ask you that we have prepared in advance, and we will ask you to say what you think about each question. To ensure the privacy of everyone here, we ask you not to repeat what to discuss outside of this group. The discussion will last for 1-2 hours. Do you have any question before we begin? If you think of any question as we proceed, please feel free to let us know. If it is all right with all of you, we will run on the tape record now.

**Section A: Details**

1. **Zone**: North west
2. **Woreda**: Laelay Maichew
3. **Kebele**: Hatsebo
4. **Facilitator`s name**: Haftay Berhane
5. **Date of FGD**: 19/11/2017
6. **FGD starting time**: 9:00 AM
7. **FGD end time**: 11:25 AM

**Section B: Socio-demographic information**

| **S.No** | **Name of FGD participant** | **Age** | **Marital status** | **Educational level** | **Occupation** |
| --- | --- | --- | --- | --- | --- |
|  | Mehari Hagos | 52 | Married | Grade 8 | Farmer |
|  | Haile Gezahegn | 34 | Married | Grade 3 | Farmer |
|  | Legese Semere | 45 | Married | Grade 5 | Teacher |
|  | Kidanu Meresa | 51 | Married | Able to read and write | Farmer |
|  | Berhe Masresha | 43 | Married | Grade 2 | Farmer |
|  | Gbremedhin Sibhat | 39 | Married | Grade 3 | Farmer |
|  | Teklu Araya | 32 | Married | Grade 10 | Farmer |
|  | Zerisenay Tewele | 34 | Married | Grade 8 | Farmer |
|  | G/micheal Shishay | 48 | Married | Grade 5 | Farmer |
|  | Tesfay Berhe | 54 | Married | Grade 3 | Farmer |

**Keys**: **I**-Interviewer

**P**- Participant

**Ps**- Participants

**Section 1: common adolescent girl`s nutrition problem in the community**

**I:** What do pregnant and lactating women as well as adolescent girls do to stay healthy in this community?

**P-6**: If the question is what should females, be it adolescents or adults and up to neonates, do to stay healthy, the first thing is they should get health service in health facilities. To stay healthy and before nutrition, they should know their health status by screening. Then after that they need food. The food available in our setting is teff, wheat, and barely. These are the one that could be prepared in the form of injera. Now, sorghum is harvesting but the crops I mentioned them before are the main foods we have. We do not have other than these. If it is available, it is in small amount. For stew we use pea, check pea. If you eat these foods you will stay healthy.

**I**: Any else? Additional points?

**P-7**: The idea on what should females and adolescents should do to stay healthy is good. The thing that you are saying is what the government needs. There is no one who needs false report. There is no one be it zone leader, wereda who fears to talk what is on the ground. On the maternal, adolescent girls and child nutrition especially on the adolescent sisters, they should follow the immunization from the first until they give birth. They should also immunize their child. They should also eat foods that increase milk production like teff, wheat, as it has been mention before by my friend. There are balanced diets that produce milk and where mothers prefer to eat like porridge, crops. Crops are important for production of milk. Children should eat foods that can keep them healthy like mashed flour (Mtin) after measuring their weight. Mothers also should follow the antenatal and postnatal care. Other ideas will be forwarded by my friends.

**P-5**: It is good to present what is real in the ground. The false report has been hurting us until today. To talk what is on the ground, the first is what the feeding practice is look like. You have already finished about health. We eat teff, wheat, sorghum, and barely. Anyways, these will be prepared as injera by milling and mixing in the form of dough. We have to properly eat foods. Anyways, we do have nearby health facility and we go for treatment. The nutrition here is good. There are also different types of fruits and vegetables.

**I**: What should be done to stay healthy in addition to taking food? What should be done?

**P-5**: To keep our self-healthy, there should get tested and we should also eat balanced diet.

**P-10**: I want to mention about hygiene. The important thing is hygiene. If there is latrine. Hygiene is very important for females. There is also a mashed flour given by the government. As it is mentioned balanced diet, hygiene. The government has prepared us clean water, nearby health facility. Anyways, if there is hygiene, there will be health. The main thing is hygiene. If you wash yourself early in the morning, there will not be any disease. The health personnel always speak about this by visiting home to home. They also teach us to use latrine. Having planned family size. Each child should be feed like the animals we feed carefully. Giving birth with hygiene and based on plan. If you give birth with your plan, it will be easy for keeping the hygiene. The mother will be befitted if she spaces births. Her body will not be harmed if she gives birth at interval of five to six years. The bay will also be good. Pregnant women should follow up and visit health facilities. Pregnant are getting tested every month. They check every month. Currently there is ambulance unlike the previous times where the pregnant women were carried by man to deliver at health facilities. Currently, pregnant women are giving birth at health facilities without any problem. Pregnant mothers were taken to Axum and they were staying two to three days before delivery with severe pain. If the adolescent girls are married after they get matured, they will be fine. The government is banned the early marriage. It is supported by law. Adolescent girls are married based on their willing and therefore, there is no maltreatment. The feeding in this area is good because everybody has irrigation, where they produce energy giving foods and others like pepper, vegetables, salad. These days, everything is available and these is can help the child to build her/his body.

**I**: Others; what should the pregnant, lactating mother and adolescent girls do to keep themselves healthy?

**P-2**: A mother do one antenatal care, and second postnatal care to stay healthy. In postnatal care, the mother check herself and her child`s health. She gives birth in health institutions. The child should exclusively breastfeed from birth up to six months. To help the milk production, she should eat oil containing peas and the normal foods we usually eat like wheat, teff, wheat. She should breastfeed her child ten times a day. She should eat different foods three to four times a day. The adolescent girls are taking vaccines in school. Every individual is advised not to allow his/her daughter to marry before 18 years of age. Therefore, there is no cases of early marriage these days. Adolescent girl is married after 18 years of age if the family agrees to do so.

**I**: Others

**P-1**: The mother should undergo medical tests before pregnancy and then if she gets pregnant she should check her health everyone to two months. She should give birth in health facility. If she gives birth to female baby, the mother should take her to vaccination on schedule. If the baby finished her vaccine, she will grow by eating spinach, salad for those who have it. Since the baby grows, she should go to school and she should married after eighteen years. The husband should help the mother in every aspect. The food we eat include teff, sorghum, maize. Generally, our food is good.

**I**: If you have anything to add?

**P**-3: The adolescents are growing being from childhood. To grow in good condition, they must work what they can do it. I do not have irrigation but I used to buy spinach. There are lactating mothers who do not want to eat porridge but cereals. Then must buy these cereals. Previously mothers were giving birth at home including our sisters and wives. But, currently mothers are giving birth at health facilities. For example, my wife pregnant and she had the phone number of the health personnel. When my wife was about to give birth, I contacted the health personnel at night time and she came to my home. Then my wife delivers at the lowest time possible in the health facility. The government has done many good things but in case of school results, girls should not be evaluated as equal as boys. When these girls fail to pass grade ten, they are facing many challenges. Some of them are engaged as a daily labourer. The government should prepare an easy education for females. There are many girls who fail to pass grade ten including our children and sisters. Why is the government not providing them with jobs? You are asking as to give you how the adolescents grow but the question of the adolescents is not answered.

**I**: The question is very good but we are only studying, we may not give you the exact answer. If you have additional points on the point raised?

**P-6**: Females are helping us as it is raised before. They harvest with us. They can even carry some loads and help us in loading weights to donkey as you can see in front (showing a man was driving a donkey carrying 50-60 kg of straw). It will affect them later. Since they will bear three to four children, this will affect them during labour. The work load will affect their back bone. This should clearly be solved. We must solve it. Let alone mothers who bear two or three or four children, we know how it challenges us. We are using stick support to stand up after we stayed in weeding and harvesting.

**P-4**: it is to strengthen what has been said. Otherwise, I do not have new points. Since mother bear a child, they should not be involved in activities that need energy. They could not cope with males. They can help us but, they should not be involved in heavy activities. They should work activities that can handle it to stay healthy.

**I**: What are the common problems that are caused by taking unbalanced food you observe in this area? What are the consequences of taking unbalanced diet? Just from your observation in this area?

**P-2:** If children are not taking balanced food, they will be stunted and their thinking will be poor. They will be weak in school. Food shortage will affect mothers and children.

**I**: Others

**P-6**: The danger of food shortage as it is mentioned before. If two female and two boys are born on the same day, if the one is provided with good food, his height will be very nice. His mind will also be open. But if you do not provide him food, he will have problem of thinking and he will be short. There is a proverb saying as `it is always baby, if a donkey is short` [In Tigrigna; hatsarya adgis wetru elu]. Because of shortage of food, he will be always baby. His mind will be abnormal. There are many guys whose minds are abnormal. There are many individuals who get sick because of shortage of food.

**I**: What type of disease do they develop?

**P-6**: They will be paralyzed. Their legs will be paralyzed.

**I**: Others?

**P-7:** If you do not eat balanced diet, the baby born will be very thin. He will waste your time and energy to care upon him. Since he is not getting balanced diet, he will not grow fast. The mother will also be harmed. If she did not eat balanced diet, she may develop pneumonia and later changed to tuberculosis and other diseases. If there is a problem, this could be changed to tuberculosis, diabetes mellitus. If she did not eat balanced diet, her fertility may be affect.

**I**: P-7 has said, if a mother is not taking balanced diet she will face a problem in fertility. How do you think so? What do you observe?

**P-7**: My age is at 30^th^ and we are talking what was in the previous time. However, there is no problem in this time. Currently, the government is extremely jealous to support female especially pregnant and lactating. Leave alone pregnant mother, a female on leadership will be given a job that can be accomplished by her. Currently, there is no mother at risk of malnutrition since the government is seriously working in addressing these issues. But, in the previous times, their babies even were paralyzed and lead you to different unwanted expenses to take them to health facilities and holly water. The second is, the mothers themselves were affected because there was poverty, sunny times, poverty and these leads to tuberculosis and diabetes mellitus as well as other unwanted diseases. But, in the recent ten years, the government has strictly following this issue. A given pregnant mother at least visit to the health facility five to six times. Since the government has arranged health facilities to be nearby so that it will be good for delivery related issues. However, there is no problem in the current times unlike the previous times may be before ten years.

I: What do you observe if some for example myself do not eat iodine, carrot? What do you first observe in me or what am I going to develop?

**P-1**: If you do not eat a good meal from different vegetables and fruits, your eye will be weak for females.

**I**: What do we called this problem?

**P-1**: I do not know its scientific name but if you do not eat carrot, cabbage, vegetables, liquids, meat, crops, your eye will be weak. You will feel blurred vision, inability of seeing. There are elders who do not able to see after 4:00PM. This is caused by lack of balanced diet such as carrot. Carrot is used to clear your eyes. If they were being able to eat vegetables, salad, spinach, it would have been helped them. However, since they do not eat such food, there individuals who get difficulty in seeing starting 4:00 PM. If the females are not able to eat good food, they will not give birth to weak baby. If the mother eats food, the baby will be fine and if she does not eat, the baby will be weak. This is occurring in females if they are eating carrot.

**P-2**: The question about what would happen if someone do not take iodine, one the individual will develop goiter. This were seen in the previous times but not now. Carrot is important one in prevention of anemia. If you have anemia, you will feel vertigo, and fatigued.

**I**: Others? What are the consequences of not eating balanced diet? What would I be if I did not eat?

**Ps**: Silence

**I**: you have mentioned problems of unbalanced diet by yourself. One of the participant has mentioned as goiter was present in the previous times but not now. Is there wasting? Wasting is if I am for example, long but my weight is low. Is there underweight? Is there shortage of food? Diseases like hypertension and diabetes mellitus? Are these problems occurring in this area?

**P-10**: It is known that the idle will always be get hungry. There are a lot including the elders, those with small plot of land, but a clever farmer will busy in farming and will eat what he wants. Those individuals who have small plot of land will not eat what they want to eat because the land is not enough to harvest what you want. If somebody do not get what he wants, he will get hunger. But if he is busy working in different farms, he will eat much as he wants.

**I**: How many times do you eat per day?

**P-10**: the busy farmer will eat four times a day but the other will eat in the morning and in the afternoon.

**I**: When do you think these happens more frequently?

**P-10**: This happens more frequently during summer times. There is hunger starting July to September. If the farmer is clever, he will buy crops by selling products from the irrigation. However, there are also weak people who do not eat what they want. If this is so, they will be thin. There is no weight and abdomen [In Tigrigna; kbdet yela kebdi yela]. When we say, he should eat, we are to mean that he must work day and night. If he works, he will eat what he wants otherwise, he will not get food to be eaten. When we see some young boys that works for their own gets 120-130 birrs per day. These guys will eat with half of the money they get and will look good. There are also young guys who do not work the whole day and they get hungry. Therefore, the farmer`s feeding style is three or four types. A farmer should work hard to get what he wants. The farmer may at least chicken meat at every three to four months. Egg is also good but most farmers took it to the market. Some farmers may sell the egg for buying salt and pepper but clever farmers do not do this. There are farmers who sell hen, goats in general they are selling what is good. They are those who do not care themselves. There are even farmers that sell the white teff leaving one quintal for themselves to eat very economically. They prefer to buy two quintals of maize by one quintal of white teff. But this is not good because, the maize is roughage and does not contain vitamins as that of the white teff. The disease will be caused by hunger. If they want to eat while they are hunger, their appetite will be closed and develop a problem. I have finished

**I**: How do you think could shortage of food affects females including the pregnant and lactating mothers as well as adolescent girls? How do you think shortage of food will affect these women and adolescents?

**P-9**: If females do not eat balanced food, do not properly keep their hygiene they will get a problem. If they eat after washing their hand and if they eat food they prepared…. If they do not be involved in active work since they are pregnant mothers, if they move the baby will move and may face a problem.

**I**: To remind you the question. How do you think could females be affected by not eating a balanced diet? You can even compare with males?

**P-2**: Shortage of food for mothers if especially the mother is pregnant, if she does not take food, she will get a problem during labour that she will develop shortness of birth and she will not push the baby out. The baby will not get out and hence she will be taken to surgery. If the baby has shortage of food, the baby may come with abnormal position for birth because it will be easily change the position as it will be thin. Since the baby did not take food, it will not keep it position due to low weight. Therefore, the shortage of food will affect both the mother and the child. If the mother gives birth, her body will be open. If the mother did not get balanced diet, one the child will not get the colostrum and she will also get tired as the baby will breastfeed from what she has eaten.

**I**: Others? How do you think could shortage of food affects females including the pregnant and lactating mothers as well as adolescent girls?

**P-7**: If they do not eat, the first and foremost, there will not be talk about health. If there is no food you will not conclude as there will be health. The main thig here is the shortage of food affects especially females because females are somewhat weak by nature. What I mean by somewhat weak is to indicate that a female will not cope to run with you. Females are weak and soft from nature. Therefore, if they do not get balanced diet, it will be problem in growth and beauty.

**I**: You have mentioned that there is sight problem starting 4:00PM? What about anemia? Do you think anemia is common in this area especially on pregnant, lactating and adolescents?

**P-8**: In anemia, when you visit the health facility, you are advised to take soup of red teff, and hot injera with salt. Anemia is present and it is common. The mothers will not take the soup of red teff because of shortage of time to care the children even if they have the teff flour at home. She will get tired. Anemia is present, we ca not say it is not there. Mothers get measured and the result decreases every week.

**I**: Others? Anemia in the pregnant and lactating mothers as well as adolescent girls?

**P-9**: Anemia is common. If you take porridge and soup of red teff, you will be somewhat strong. When you stand from active work, you will feel vertigo like symptom and blurred vision. This is sign of anemia. If you take balanced diet, you improve. You eye will will improve and your energy will improve.

**I**: What about goiter?

**Ps:** We have already talk about this goiter?

**I**: Do you think those problems like goiter, anemia, and night blindness have relationship with nutrition? Are they really caused by shortage of food?

**P-3**: Night blindness is caused by one, food shortage. The second is, we are farmers and we could not clean our surrounding early in the morning. There are dusts in our campus. We are farmers and leave alone us, our children are also getting this dust. The dusts will contribute with the shortage of food. Therefore, blurred vision is present.

The dusts will contribute with the shortage of food.

**I**: Others?

**P-6**: As it is mentioned before, the cause of these problems is not only by shortage of food. But it was mentioned earlier. For example, if you defecate at open space instead of using toilet. If a flea come and sit in your food touching the defecate, you will be sick. It is to mean that it is not only by shortage but it could also be caused by others. The is also block of sand in front of houses that mast be cleaned. This sand is dusted by animals and child`s excrete. Then if the flea again touches your food, you be sick. This could also be the cause. It is not only caused by shortage of carrot, vegetables etc. but it could also be caused by lack of sanitation and hygiene.

**P-2**: Goiter can be prevented by consumption of iodized salt. The one which causes vertigo like symptom could also somewhat improve by taking carrot and beetroot. But if it is anemia. Goiter is currently decreasing. The night blindness is also common in males. There are many individuals who cannot see after 4:00 PM

**I**: Are non-communicable diseases like hypertension, diabetes mellitus and cancer common here?

**P-5**: The diseases that you mentioned are not common in our area. Hypertension is common in energetic and on those who have high volume of blood. But, we do not have such a person here. There is no case with cancer.

**I**: Others?

**P-8**: In case of diabetes, it is there, you cannot conclude as it is not present. It is common. Do not you remember the daughter of Araya, and others. Diabetes is related with hunger and cold. We cannot say it is not present. It is there.

P-6: The above-mentioned diseases are there. We cannot conclude as there is no. we cannot put as diabetes is not common here. in this kebele, there could be three to four cases of diabetes mellitus. There are even individuals with other disease like HIV. This could only be known by the health personnel. It is not also good to speak what we have heard from individuals. The cancer could also be present even if I am not clear what that cancer is.

**P-2**: There are no cases with cancer. This is seen in towns. But, I have known more than ten cases with diabetes mellitus. There are cases who are benefited from safety net program and HIV patients. As our belief, all diseases are communicable except the head injury.

**I**: Others?

**Ps**: silent?

**I**: What do you think is the causes of the above-mentioned diseases?

**P-5**: Diabetes mellitus could be caused by two things. One is related to hunger and the other is with comfort. Diabetes mellitus can affect both at state of hunger and comfort.

**I**: Others?

**P-2**: Cancer is common especially females. It starts on the breast. If you did not treat a would early, it could be changed to cancer.

**I**: Could these non-communicable diseases be caused by malnutrition? Could they be caused by overnutrition, or undernutrition? Just it is based your opinion

**P-10**: Anemia is caused by hunger and hypertension could be caused by comfort. Hypertension is caused when there is rest for those who were on active work. anemia can occur when you did not take the food that you like. Generally, if you take a food that is not comfortable for you, it will be changed to disease. The main thing is hygiene. It is not only what you eat, you must sleep in a good bed, you should wash your body. The disease is occurring because of being farmers. For example, those who eat a small amount of food with clear cloths in Axum will not get sick. But, we are getting sick because we are eating without washing our hands, sleeping in uncomfortable bed. The bed we slept may be uncomfortable to the level of bleeding. The blood could be high or low. Then, if you go to health facility, the doctor will tell you that your blood is dried. Your blood is dried without no movement or disturbed. Anyways, the hypertension is caused by rest and low blood level is caused by hunger.

**I**: Are there cases with overweight? Is it common to see overweight especially in women and adolescent girls here?

**P-2:** We do not have overweight but we have children with underweight.

**I:** Others

**P-5:** You are observing us as farmers, we do not have overweight. The man in front of you is the only huge person we have (indicating P-10)...Laughing…

**Ps**: Laughing...

**I**: Others

**Ps**- Silence

**I**: Ok. Is there a situation when women suffer from shortage of food? Those women who do not have food that is enough to serve them one full year?

**P-1:** Yes, there are. It is not appropriate to conclude as there are no women who are suffering from food shortage compare to myself. There are women who take rest three to four times to go to Sunday market. Yes, there are.

**P-2**: The food shortage is mostly seen in women who are house holders. Female house holders will only secure their food for about six months. Most of them are safety net program beneficiaries. They can only feed for six months. But, it does not mean all females are affected; it is the house holder females who are mostly affected by food shortage.

**I**: Others? Is it common to see females who suffer from food shortage?

**P-10**: We are going to repeat it. There are many females in this case. Females may have increased family size. The plot of land is very small. If the family size is five, they will be more affected. But, the government is supporting such women. They are always supported by the government. The farmer is also contributing 3 to 4 kilos for supporting these women. We are repeating it again but there are many females who do not even have enough for six months. There are five to six households who do not have food to eat. The government is promoting the support for females and there is a situation where we support from the kebeles. Females are more affected related to the family size and labour.

**I**: In what situation do you think food shortage or drought happened?

**P-3**: It occurs starting from May to September. The food shortage and drought is increased started from the month of May to September. Not only women, there are also males who face the drought and shortage of food. The women are being affected because they will have children from different husbands on which some of them help them but the other do not. Since females are our wives and our children, they are affected.

**I**: Others? When does drought or food shortage occurs?

**P-1**: The question is about when does a food shortage in females including those age up to 18 years occurs. The shortage is commonly see from September to November. This is because the mother will give the food to her child and without getting food for herself. Then, she will get sick.

**Section 2: Barriers to access and utilization of nutrition services**

**I**: Do women advised to visit health facilities for check-up and services during pregnancy?

**P-8**: Yes, it will be given. She will be advised to go to health post for check-ups. If the result of the check-ups showed that her delivery time is not yet, she will be given appointments but if the time for delivery is on that day, she will be taken to health center or hospital immediately with ambulance.

**P-10**: The health extension workers her are visiting home to home to identify each pregnant woman in households. The group leader is also asked to report if pregnant women is in his/her jurisdiction. The group leader with the kebele leader will go house to house to check pregnant woman. There may be new brides who do not want to expose their pregnancy but they will be known by the house to house visit. Militias’ are also participating with the heath extension workers in identifying the pregnant women especially those hide themselves because of shyness; these may be one out of hundreds of the pregnant women. The heath extension workers are three and they are doing the house to house visit by sharing the kebeles we have. These days, the community is considered as illegal to keep pregnant women without visiting a health facility. I told you one out of hundred because it is known starting from home by the militias, health extension workers, kebele leader and administrator. The advice is given to her starting early at around six months. I finish.

**P-4**: Good. Is advice given means, in the previous times the advice was given carefully and in coordinated way with the development army, kebele leader and professional. But currently, there is no house to house visit. The pregnant woman comes directly and until she gives birth, she visits the health facility by herself. Otherwise, there is no one who visit house to house. The visit was present in the previous times and now, individuals are using by themselves.

**P-7**: They have generally mentioned the idea, but the main thing here is the idea on does pregnant woman get advice. The advice is given by three or four types of advice. Because, one, there are health professionals. The health professionals will give advice. Each woman developmental army are there. They are also connected by the network. The health professionals will use this to provide the advice. Important idea like what should a pregnant woman eat, how she should sleep, any ways, as she swallows a life, they are advising her to keep herself and the life inside her be safe. Regarding the check-ups, they are checked until the final day for checking. If we see it in the eye of politics our kebele, the government is saying one mother, as our respected leader plans, should not miss one month for check-ups. If one mother misses one month without check-ups, she will be penalized. It was done in the previous times but now, the people is generally aware of it and hence mothers are going for check-ups by themselves. The people is accustomed it and no one is being shy on these days but there may be one or two individuals out of the total population. As everybody is accustomed it, there is even no need of advice to safe his/her own life.

**I**: Good. Let`s talk about feeding? How many times should a pregnant woman advised to eat per day? How many times do pregnant women eat per day practically?

**P-2**: The way they feed is like this. They eat their breakfast early in the morning and then they eat their lunch at about 10:00-11:00 o`clock. They eat at around 2:00 PM and then they eat dinner at night time. Totally, they eat four times per day.

**I**: Others

**P-10**: It is like this. The food is with them. The food is with them except the food that is brought from outside like what is done in town. The husband may bring banana and other from outside and these are the things that should be counted otherwise the food is with themselves. This is because they are passing their day being at home. If the woman is strong, she may eat food that is suitable for her. They are sending us out after giving us our breakfast in the morning. They may or may not eat with us but we are considering it as they eat with us. We are counting what we normally eat, but the mother may eat by mixing or soup. She may count what you have brought as in the town. We are eating four time per day; morning, afternoon, evening and night time. They eat equally as we did. But if they want to eat, the husband will not be with them in the day time. If a husband is out of home, he will drink or eat what he wants to but mothers can eat what they want. If she is clever enough, nobody will stop her from eating. Everything is with her whether it is good or not including crops, hen, egg etc. The difficulty may be in case of meat which may be brought once in a month. It is to mean the food with her.

**P-7**: It is as my brother said it. Mostly females as it has been said before, there are house holders where they have large family size. As it has been said, it depends based on their own ability. The female has everything with her. It may be because of her laziness and sometimes she may need advice as you been trying to tell as on how to keep herself. Otherwise, everything is with her. It is the number of times that we used to eat is four times. We are here eating our breakfast. We are not seeing them how many times they are eating. Anyways, the number of times we eat is four times and they eat four times with us which in the morning, afternoon, evening and night times. Today, individuals may not earn money but they can get the hand to mouth harvests and nobody will be affected by shortage of food. Therefore, females are not affected. This is the idea I have.

**P-6**: The idea mentioned about how many times females eat will depend on their income. If we say all females are eating, it will be very difficult. Females are not eating as it has been mentioned above. They give priority to their children. There are pregnant who do not even eat two times per day. This not because of their interest but because they do not have food at home. If we say how many times should be eaten, what is wrong if they can eat six times a day making sure that they can get it. They know what is important for them. There are vegetables nearby and they can bring by buying with two to three birrs. There is no body who can control them. As it mentioned before, I may go this side and the other will go that side and hence, they can eat they feel is important for them. The thing they can need from us is animal meat. If we do not bring them meat from Axum, they cannot go and buy this. We buy and bring them, and eat it as what they feel it good may be in the form of roasted, or cooked.

**I**: Others?

**Ps**: Silence

**I**: What about lactating mothers? How many times do lactating mothers eat?

**P-5**: not all mothers are the same. There are mothers who eat good and there are who give priority to other works and do not eat good even if they have the food at home. If the lactating woman gets different food, she can build her body early. If she gets foods like meat, milk, butter, vegetables, her body can easily get improved and she can better care her baby.

**I**: Others

**P-2**: As to me, lactating woman should take around five times per day. To increase the production of milk, not only foods that are eaten in the form of injera should she eat, but she should take crops. She also thinks about her kid and to produce milk, she will get different type of food. Even if husbands should feed their wives in the antenatal period but most of them tends to bring different kind of food just after delivery. This because, mothers will not go out of home before 40 days or before christening and the mother may decrease weight after birth. Therefore, I think the number of meals a mother can increases during lactation.

**P-1**: Lactating is like that of new bridegroom where he is feed with his bride. She should be feed well. This is because, she should not decrease her weight and as we have mentioned it before, the mother should eat good food to increase the milk production and the baby will get ample amount of milk. If you do not care the lactating mother, baby will not get enough milk. If she is not able to eat food, you should take her to health facility and get tested for not taking enough food.

**I**: You have told me that mothers are eating three, to five times per day during pregnancy and lactation. Are Women getting counseling for food diversification or foods that contain the mixture from crops, vegetables, fruits, etc during pregnancy and lactation?

**P-7**: Yes. It is advisable to eat balanced diet but since we are talking about females, they are given properly. We have professionals here hired by the government. Advices comes from these professionals will be distributed to each kebele. We have three professionals and they are assigned for four of kebeles. They have a plan when to go to which kebele. Women have their own day for meeting; it on 12^th^ and 27^th^ days of a month with the leaders of development army and health extension worker. There are many detail advices given at these days. The women development army is stronger than the men development army. This is because females are given advices by the health extension workers in this area. When you the women prepare a good meal more than the meal you get in downtown. No all women are the same, but when you see some home, women are preparing very good meal in their home. There are individuals who eats a food that can bring immediate change in your body. There are also women, as farmers, do simple food. Anyways, women are well advised on the issue.

**I**: Let`s add some examples with it. The first question is, are they being advised and let`s support it with examples.

**P**-6: Here we have a mush (Mitin) here owned by the laelay maichew wereda for children and mothers. It is composed of peas, wheat, sugar and cheek pea. The mixture is boiled, crashed and milled. This is prepared for lactating women. There is also a food prepared for babies who are started food after exclusive breastfeeding. This is done by female groups that contain 12-13 members. They are working by evaluating themselves every week. As it has been said, the health extension workers are visiting home to home to advice mothers to take porridge of red teff. Red teff is best food. This is because, this food provides blood. They are also advising to buy and take lettuce, spinach, beetroot, carrot.

**I**: Do you think all pregnant and lactating women as well as the adolescent girls?

**P-10**: The mash is given to those whose age is one year to five years. It is bad if we go giving you wrong information. This mash is taken by mothers at the time of labour to a child of less than five years. The others can work and eat. They are living with their family and if food is available at home, they can eat with their family. This a good century. The advice is given to everybody not only females but also for males at kebele level in groups. There are five females who are group leaders. The health extension workers with these groups will provide advice not only for the women and adolescents but for us, too. They are advised to combine and mill those foods available in house of farmers including peas, chickpea, Beans, wheat, barely. Lactating mothers are advised to prepare such mix since it has advantage. If they are interested, they can buy other foods they like by selling these food items. There are some lactating mothers who did not eat porridge until seven after delivery. These mothers are not interested to eat the food available at home. Lactating mothers will be fine while they are supported by their mothers or daughters until seven days and hunger is seen after the seven days where they start house related activities. It has been two to three years while it is started advices. Not only for females but the advice is also given to male farmers.

P-2: It is already described by P-10

P-5: The mash is used by children, pregnant and lactating mothers. It is already prepared and they will exchange one kilo of wheat or teff to one kilo of mash. They are properly using it.

**I**: Okay, good. Do women that we mentioned earlier get advice on home gardening? Or how each household gets advice on home gardening (backyard vegetation) to produce carrot and vegetables?

**P-8**: Regarding giving advice or not, it is given/told to produce carrot, beetroot, salad, lettuce and other spices. However, there are women who apply the advice and who do not apply. About this, they are highly advised and told. We are also advised and told. It is good. Thus, the idea that you are saying is highly advised and told.

**I**: Okay, others?

**P-6**: It is about do women get advice to eat and produce vegetables? Overall, if there is a farmland near to the river that you can water by digging 5 to 6 meters, it is said that it should not be left uncultivated but to be practically cultivated. Leave alone in that farmland, even advice is given to all to cultivate and produce near to your house in a 10 m by 10 m area using water that is used for drinking.

**I**: Okay, are they currently do that? Is that done currently in each house?

Number 6: I mean, they are given advice. Okay, let me answer while I am in that. I understand. However, if we say do they accept this advice or not, there are who accept and who do not accept. If there is water in the nearby, one or two may be benefited from the backyard vegetation. However, there are also persons who do not benefit leave alone from the backyard even in farmlands where you can get huge water by digging. As said earlier, some of those who said I have to work, they produce and then they eat or sell so that they earn a lot of income. On the other hand, those who slept (do not work), they didn’t dig to get water to cultivate and produce.

**P-1**: It is about do women told to produce backyard vegetation? It is told. Some individuals try to cultivate/produce in the late summer. Even me, I planted but the vegetables get dried after the rain stopped and the moisture of soil get dried. Any ways, I have irrigation and I have used irrigation but overall about the advice, it is given/told. They are advised and told to produce backyard vegetation from just brining something while you are pregnant, so this my idea.

**I**: If there is no any other, you told me that there are safety net program beneficiaries in this community. Who are the safety net program beneficiaries? What kind of persons? Are women beneficiaries of safety net program? Ehhh…P-10.

**P-10**: The beneficiaries of safety net are those that we told you earlier. These are women who are household heads with 7 children and 5 children. Don’t see it as benefit since the farmland is not sufficient to feed five persons. In addition, there are husband and wife with 3 or 4 children, who do not have their own farmlands but occupied small portion of farmland in their parents’ farm area. These are the ones who are benefited with the women. Thus, women are the beneficiaries of the safety net program but the rest of the farmers work by their own. Those families who have 6 or 7 household members get benefited since the farmland is not sufficient and it may help them from migrating to other areas. Thus, based on the government direction and after discussions at the Kebelle level, most of the beneficiaries of safety net program are women. Therefore, to those who are able to work, they are told to work. However, those women who are widowed and who have children are benefited by the program as the number of such individuals are increased in the Kebelle. So, women are the beneficiaries of the safety net program and not allowed to others.

**I**: Okay.

**P-5**: Safety net embraces/comprises two things. One, it embraces dependents. Dependents are those who disabled, very old individuals (elders) and orphans. The second is by work and it first includes female household heads, and then it includes those families who do not have farmland and who do not have any assets. Thus, safety net program supports these individuals if they are the ones who are affected.

**I**: What about others?

(All participants become silent)

**I**: Okay, good. Is there food support other than safety net in this community?

**P-7**: Regarding the food support, you know how it is? Safety net is a food support known by the government. About food support what is done as Kebelle is that there are what we called social affairs, right? Then the farmers pays 24 Ethiopian Birr or 3 Shehane (equivalent to 1.5 Kgs) in terms of cereals that is either Taff or wheat and all these contributions will be collected by the Kebelle. Now, as I told you before, there are 4 Kebelles and the quota for each Kebelle will be given by saying Kebelle this, this much is your quota and Kebelle this, this much is your quota based on the convenience made by the residents of that area. Then, the collected money or cereals will be kept at Kebelle level and there is its own independent office that audits this collected money or cereals at Zonal, woreda and Kebelle levels. And then, through the Kebelle structure/system as it was said earlier it will be asked at Kebelle level, who is food insecure? For example, me, I am called Mr X. Now, if it is said that Mr X is food insecure, then the Kebelle organizing committee (Kebelle Meseretawi Wudabe) will give me either 40 or 50 shehane (equivalent to 27 or 33 Kgs) in terms of cereals or 400 or 500 Ethiopian Birr. Thus, in this way food support will be given to such highly food insecure individuals for the sake of staying life.

**I**: One shehane is how much kg?

**P-7**: One kg is 1.5 shehane. Say it, 50 kg may be supported or 70 kg may be supported and it depends on the number of family.

**I**: Good. Is there advice/lessons given on water, sanitation and hygiene?

**P-10**: Currently, there are plenty of teachings/advises. Starting from ourselves, latrines/toilets and water, we are advised to clean. They advise us throughout the year to clean our premises (environment), to separately tie cattle, to have separate area for cattle, to have separate bed rooms for humans and to dispose the manure. Thus, teachings are plenty. However, as they are farmers, they did not apply them. Health extension workers visit house to house and no one inspects a house like health extension workers even the woreda officials and they inspect the house every month to check what the house looks like. They inspect everything including kitchen (stove), shelf, and nothing is left uninspected. They visit/tour every house. There is nothing spoken in the Kebelle except cleanliness/sanitation. Water is also in our backyard because of our government. We have about 2 or 3 tap water here around. In terms of water access, it is good. School/education is also here near to the gate of our house. The health post/center is also here in the nearby. The health extension workers visits every house and teaches. Even the woreda officials are here most of the time. Thus, it is told.

**I**: So, why farmers do not apply it?

**P-10**: You know what, why we do not apply it? Let if we constructed a latrine using the wood material, the latrine would be collapsed within two years as the wood get decomposed by worms and then when we tell to the health extension workers as the latrine is collapsed, they said use iron bar to construct the toilet. However, we urge with them by saying we don’t have capacity, so from where can we bring the iron bar? Thus, we asked them why you don’t give us iron bar …laughing…..give us the iron bar so that we can hardly construct the latrine within a year but they said to how many of you we can give and from where we can bring the iron bar and then they said, it is for yourself for your own private latrine, so we do not give you iron bars. Therefore, it is due to this, not since we are too busy. Any ways, latrines were constructed in every household but some of them become full while others are easily damaged and collapsed as the wood is decomposed by worms.

I: So, do worms affect the houses?

P-10: I am telling you the basement. The house has roofs above the ground…laughing…

**I**: Is there advice on water, sanitation and hygiene services?

**P-1**: “is education given” is your question; yes, it is correct; it is given. Advice on sanitation is given. You guys are clean.

**P-4**: the education is given. We have been advised and discussed about it. I have children. They wake up and want to go to school, he tries to eat and go without washing his hand, because the child does not know what to do. Thus. we parents have to advice to them: you have to wash before eating; we personally teach them. That way, they have taught us and now we are teaching to our children. Regarding the toilet, the wall is ruined but the slap is not; this is because the wall is damaged by rain. We are not able built toilet like the urban once, because our land is small, and it is not enough for our economy. Regarding hygiene we advised and in turn we are advising our children.

**I**: what else? Anyone with another idea;

**P-7**: Now, what it is: you are telling us what is in our heart. The good thing is, previously you have told us that you work in University. The university is helping well. You are making the government to exist and extend his network. Because, there are many universities working on seeds and they are helping us. Here on health issue, on your side, you are helping the community. And this is very good job. Now regarding the personal and environmental sanitation, it is like what they have said. What is here with farmer is: he did not attend school, including us. Some of them are because of lack of understanding. For example, if come to toilet, there are around 200 permanent latrines. There is no one who do not have temporary latrine. Those who don’t work even the temporary is because of lack of understanding on its benefit. If not, how it can be; and the government did not them any material (Tendino) to who build permanent latrine. That is the conclusion. Regarding the environmental hygiene, here there are professional and they teach the community. There was Atet (AWD) and other diseases; when a disease occurs, it is first notified to the rural community before the urban dwellers. This is done through the health representatives. In addition there other individuals assigned from the community for this purpose. Now, health professionals are telling us about personal hygiene. They tell us not to eat without washing our hands. “Do not touch a food before touching water” they are telling us this one. This is a personal hygiene. With regard to environmental sanitation, we are informed not to defecate in an open field; do not dispose waste in field. This is for environmental sanitation. At an individual level, you have to work individually. This is it and it is enough for me.

**I**: Is malaria common here?

**P-6**: Yes. If you come our kebele Hatsebo, one; there is grass land; it is low land and it attracts moisture (zebee). It includes from our home, here, to that area. There is another place are called ‘Adibala’ it is low land like ours. All areas across the edge of the grass land have malaria.

**I**: What else? Does the community use ITN?

**P-1**: Most of the community uses ITN. For example I myself utilize ITN. Even though I utilize, sometimes we move at night for work. At that time our children also waked up; they are not interest to sleep despite we told them to sleep. At that moment, the mosquitos bite you. After they bite you, they transmit malaria. In terms of utilization, we use ITN. However, because we wake up early in the morning for work, and lately enter to home at night, they bite us. But, while we sleep we use ITN.

**P-3**: In terms of its presence, yes, there is malaria. For the past three years, because we have been given education by the health professions, there, we know how to utilize the ITN, and we are still utilizing the ITN. We the material we have at home, be it mate or else, we put is under, and extend the ITN using stick. By doing so, we use the ITN.

**I**: Are there individuals who do not utilize ITN?

**P-8**: There may be. Regarding the preparation, utilization and extension of ITN, there may be individuals who do not use it. The government is providing the service, and it is very good. ITN is important; this year I did not take. I took ITN in the past three year. I extend three of them at three beds (Medeb), and we sleep under it. This year, there was a shortage of ITN, and some of us did not get ITN. For the next, more ITN should be distributed to us.

**I**: If there are individuals who do not use ITN, you can mention the reason. Because there is shortage of ITN, there are people who do not use ITN; what else is the reason?

**P-9**: There is no anyone who does not use ITN; there is anyone who does not have six or seven ITN; and all people use it.

**P-3**: Those who do not use ITN; I do not think adolescents are given ITN. “Do they utilize or not?” No, they do not use. For example, my brother Mr. ‘X’ has explained that he has three ITN. And he has three adolescent girls. I am not sure if they (adolescents) are given ITN or not; I do not think they are given.

**P-10**: ITN is distributed to family (household). May be if you (Mr. X) were not come, but the ITN was brought by two, three cars. It is given to family. May be if you were not come; but we are using it from the beginning. To one household, six or seven ITNs are given. If the family members are seven, he will be give seven ITN, and if the family member are eight, the same will be given. Regarding the presence of malaria, many people go to west (western Tigray) for job. At night people pass through this to go for daily work. At that time, he may not notice it, but later he may get sick of malaria. Some people also go to Humera to find job. Humera is risky area. Even the swampy area is a source of malaria. At summer, if the water in your home yard is not drained, it can bring mosquito. It can also live in the grass (Muguya). If you do not cut it, it is bad. Regarding the ITN extension (Mizirgah), it is because of laziness that people are not extending/using ITN. In a year, two or three ITN is given to each household. If two ITN is given, even the previous one will not get old. Therefore, we can use the previous one as mate. It is easy to make up the ITN. For example, Mr. X has three ITN; two of his children can one ITN and the other two children can use one ITN. We know in town that five, six individuals sleep together at one bed, separately for males and females. Here, the ITN is distributed every year. People are not using it only if they are lazy. It may be available at home; this is what I think. They (HEWs) were visiting home to home and found improper use: some people used it as cover to hey. Some of the ITNs were burnt and damaged. But the government provides us ITN every year. Besides, anti-malaria is sprayed every year. For example, this year, the river area was sprayed. It was not in ours but, it was sprayed in the river areas (low lands). It is swampy and malarias. We are given ITN every year. They (HEWs) also check whether it is extended or not.

P5: To those who do not use, the problem is in the person. For two persons in one household, one ITN is given. If he sleeps under it, it will benefit him. If they sleep separately, one of them will sleep barely. Anyway, we are given ITN every year. Let alone for us, even it can serve for others. The government is providing ITN, and the problem could be from the individual himself.

**I**: Who provide you advice on ITN utilization?

**P-1**: The issue is on the utilization. The question why it is not utilized well. The problem is the user’s problem. One ITN is allowed for two persons. If the two agrees to sleep together, it will be enough, otherwise, it will not be teared in to two. ITN is given every year. It is provided last and this year. There is more than enough ITNs at home. The government is distributed enough ITN but it could be the users` problem.

**I**: Who advise you on the ITN utilization? Who provide you advise on when to use and how to use?

**P-1**: The question is who advice you the extension and use of ITN. When the health extension workers advices us once, it was enough. They show us how to extend it and we are following it.

**I**: Are Women/Girls getting deworming services?

**P-6**: The medication is provided every year in each school. Students were told to bring with lunch as the medication was strong. All the students have taken the medication. We have four schools here around and students of every school has been taken this medication.

**I**: Others

**P-1**: P-6 was indicating about females however, not only females but all students including male students are also given. They are even told to bring their lunch.

**I**: Well. What about out of school adolescents? Do they take these medications?

**P-10**: the medication is provided to all females. The out-school adolescents are identified by group leaders. They are already studied. All my family including adolescents are posted on my door. Since the medication is supplied based on family number, they will be given. They are given in home to home basis like that of drug that was given for night blindness in the previous times. It is already studied. My family size is already in my file document. Therefore, since it is already studied, it will not be difficult to get even the out-school adolescents.

**I**: What do you think the advantage of this deworming is? Could it be linked with nutrition?

**P-3**: This drug is used to prevent the multiplication of the intestinal worms that can share the food they eat. It is also used to make them healthy.

**I**: What do you feel if women are specifically Targeted for supplementary feeding? For example, fafa?

**P-10**: There is no supplementation of fafa for women. In the previous times, fafa was given to school children. …Laughing… But now there is no fafa. It was on the regimen of derg where females wait for their husbands. But, these days, adolescent girls are eating at any time with their mothers. Nobody is controlling them and they can eat what they want from the food available at home. You will not meet each other during day times. You will come home at the evening. If the adolescent wants to take food to work area or school, she can do it. I was trying to mention it before; if you have, it is good to make it easy for swallowing.

**P-5**: If there is a chance that the women can get feeding targeting the women, nobody will oppose it. If there is any plan by the government on this regard, it is good if they can get the support. But, they are eating what we have and they can eat as whatever is convenient for them. But if there is special thing planned specifically for women, it is good to be introduced.

**Section 3: Perceived needs of women for relevant services during pregnancy**

**I**: Do you think women need special services than targeted food supplementation like getting rest or other additional food or medicine?

**P**-**8**: About the balanced food support, women are given advices to take rest. She should take soup of red reff than even wheat. Wheat seems good for eye but red teff is important to increase amount of blood. They are advised to take rest but they will be busy in home related activities.

**I**: From the above-mentioned interventions like distribution of ITN, deworming, targeted supplementary feeding, which one do you think are necessary for women?

**P-**1: If a female is to lead the best life, there should be a good food. If you have a problem you cannot bring a good food.

**Section 4: Other interventions that improve pregnant, lactating and adolescent nutrition**

**I**: We have mentioned some interventions for women like to take extra meal during pregnancy and lactation, to visit the health facility for check-ups, to use ITN, to keep hygiene and sanitation. What are the barriers that hinder women and adolescents not apply these interventions?

**P-10**: From July to December, there is no work to be done without females in farmers. They will not even sit by themselves. It is not good to speak what you do not really do practically. Therefore, females are highly affected in these six months. If the woman has children that can harvest, she may get rest.

**I**: Others? What are the barriers?

**P-1**: There is no obstacle for using ITN because the husband or child can apply it. But about the extra meal, it is an obstacle for female if they do not have food at home.

**I**: is there any additional point?

**Ps**: This is it. There are no additional points

**I**: We have said that females should take extra meal during pregnancy and lactation, visit the health facility for check-ups, use ITN, take rest. What do you think the role of husbands in the above-mentioned interventions? What is your role or contribution to your wives and daughters to implement the above interventions?

**P-5**: It is good to advise them before they get sick. But if unfortunately, they get sick, they must be taken to health facility for treatment early. The other is providing a balanced diet based on your capacity.

**I**: If your wife is pregnant, do you as, husband, assist your wife to eat balanced diet? to visit health facility on time? get rest? etc

**P-5**: Males should do during pregnancy. Because, it is the time you will advise your wife not to participate in difficult work, not to carry heavy things, and to visit health facility early. I, myself, do all these things.

**I**: Others? What is the real practice here?

**P-1**: We were talking about general things including the poor. But I want to speak my personal experience in these regards. If my wife is a pregnant and near to get deliver, I do participate in brining things from the market. I do this because, I am affected by different means if I am not help her. I must help her two to three times. If she is unable to fetch water, I should do it myself. If I have children that can fetch water, they can do it otherwise I must do it. I used to help her such things.

**I**: Others?

**P-3**: If you think critically, the problem come to you by any means. We are repeating the thing that has been debarred in the past times [to indicate the husband involvement in every aspect is now in practice]. If she gets sick, you will have expense of one to two thousand birrs. Even though we do not usually help her in the earlier time up to six months, it is our role to help her in bringing what she wants from the market on the months mostly after six months.

**I**: What about for adolescent daughters? As they are future mothers, do we give them food separately? How family helps their adolescent daughters exceptionally from that of males? Let me clarify it by examples from other areas. In some areas, the male is the one who should eat food first, should go to school. How is the practice her in favouring males than that of females?

**P-5**: in our area, there is no difference treatment for male or female in feeding. You will share what you have in home equally. There is no way to let a female stay to eat than the male. In our area, a female will eat equally with the other members of the family.

**I**: Others:

**P-1**: The thing that you mentioned is more traditional which is not current practice here. There is no way you favour to the male to provide him food so that he can plough, or go to school. You will equally feed by sharing whether you are male or female. There is no favour for husband, wife or male. I myself do not favour to anyone.

**I**: Which foods are advisable for pregnant women?

**P**-8: We are repeating it but for pregnant if there is carrot, beetroot, soup, porridge, vegetables are advised to be taken when she likes to eat. However, mother will not take by balancing these foods because she wants to share to her kids. The kids themselves are also nagging her to share them including the soup.

**I**: What about for adolescents? Which foods are advised to be taken by adolescent girls so that they can be matured or considering as they are tomorrows mothers?

**P-1**: Giving a food specific for adolescent girl is not started yet. We may start it if it has value but, it is not yet started.

**P-9**: Previously, there was no practice of giving special food for adolescents to be matured early but it is possible to start it.

**I**: Are there food taboos that pregnant and lactating as well as adolescent girls should not eat?

**P-10**: No there is no. there is nothing prohibited to be eaten in fact if it is available… Laugh… Females are not allowed to drink alcohol but they can drink soft drinks and eat any food.

**I**: In other areas, adolescent girls are not allowed to take milk because, it is assumed that taking milk will help to mature the girl early than expected and go for sex etc. How is the practice here? What foods are avoided not to be taken by females?

**P-1**: It does not matter what the adolescent girl eats including milk or others, it is the natural behaviour of the girl that matters. There is nothing that we prevent them from so that they can be weak and not looking for boys.

**Section 4: Other interventions that improve pregnant, lactating and adolescent nutrition**

**I**: Well. Is there a practice of nutrition screening?

**P-5**: there is measurement of weight and you will be measured by meter around this [showing the upper arm]. Mother, children are going for measurement to know whether they are decreasing or increasing.

**P-1**: I have additional points. There are measurements. All females who came for check-ups do measure their weight whether they are decreasing or increasing.

**I**: Is there a day dedicated and celebrated for community health? Or a day for meeting specifically for health issues? Or pregnant conference?

**P-10**: I may not remember the day but there is. There is a meeting of pregnant mothers and health nodes.

**I**: Therefore, do women participate in these meetings?

**P-9**: Yes. They go for participation. They are invited and then participate in the meeting.

**I**: What do you think they do in the meetings?

**P-1**: They have a meeting at 27^th^ and 12^th^ days of a month and they evaluate themselves on who was getting weak? who is not coming for check-ups? and who is not keeping her hygiene? They are meeting for evaluation two times a month to identify who is not working at the right direction, which houses do use the ITN and which toilets are dug and which are not.

**I**: well. Do adolescents participate in the meeting?

**P-3**: Yes, they do. The grouping is done to facilitate such activities. They have a day dedicated for ceremony. Even they are mothers who have saving account here.

**I**: Are females that do not participate in the meetings you have mentioned to me? If there are what is their reason?

**P-**9: the only reason they miss the meeting is if they are on the eve of delivery. But the adolescents are going for meeting every month. There are no obstacles for females not to go for meetings.

**I**: Are there barriers that hinder the adolescent girls not to go to the meeting? May be the families do not want or other factors?

**P-3**: This area is very near to town. Every adolescent can participate in any meeting that is arranged for them. Personally, I do not think families are preventing adolescent girls from going to meetings.

**I**: We have been discussing about safety net program. Do pregnant women benefited from safety net program?

**P-1**: No. Women will not be included in the safety net program because they are being pregnant. She will not be included because she is considered to be a wife a farmer that can lead a family.

**I**: From those who included in the safety net program, do pregnant women allowed to take rest?

**P-10**: If a mother is included in safety net program, she will not be involved in the work. She will not even participate in the free service of water and soil conservation. Pregnant women are not working in this support and even in the free service until christening which up to 80 days.

**P-1**: The condition in safety net is different. The law supports a pregnant woman to be free for one to two years. She will be supported by the safety net for free,

**P-5**: It is already answered.

**Section 5: Understanding perceptions of age at first birth and birth spacing**

**I**: Is there early marriage in this area? Is there marriage of girls below eighteen years?

**P-9**: There is no such incidence in our setting. There is no situation where females are getting marriage at fifteen and sixteen years of age.

**I**: Others?

**P-1**: It is additional point. There is no female who married before eighteen years.

**I**: Why do you think so? Why is early marriage not common here?

**P**-1: If a girl is married before eighteen years of age, she will be at risk of a disease which I could not remember the name of the disease right now.

**I**: Others? Why is early marriage not common here?

**P-10**: It is pro sidestepped by law. Moreover, those fortunate girls will continue their school up to grade 12 and some may join universities. They will reach at the age of eighteen years when they finish grade ten. There is also scarcity. An adolescent who is not having job will not married to a man who do not have any job. What should they eat if both of them do not have a job. But if the man has job, he might be married to female of age greater than eighteen because female less than eighteen years of age will not be allowed to be married as it is declared by the law and it is also females right. The male wants to marry female who has job. The female also wants to marry a man with job. Until they search for an individual who has job, the time will go and the female will reach at eighteen years or more. There are females whose age is 24 to 25 years but they are not yet married. Previously, early marriage was done because of the poverty. Currently, it banned.

**P-5**: if a girl is married early, one, she is not able to carry out the marital properly as she is young. The second, is she will face a problem during delivery. The government is not supporting because she will face these problems.

**I**: Do you think delaying the age at marriage to beyond eighteen years will have any benefit on the nutrition of the women?

**P-1**: If she is below eighteen years of age, she will not know the way of eating but if she is eighteen and more, she knows everything. If she does not know the feeding process, the marriage will be smashed. However, if she is eighteen, she knows the feeding process and the nature of her husband and will led a good life.

I: Other? We can also link with the coming baby from these girls? How could a delayed marriage will be related with improved nutrition?

**P-8**: Since the girl will go to school and for work, she may not see how food is prepared. If she can prepare a stew and injera, she may feed her husband by balancing the food when she is above eighteen. If the mother does not support her to see how it is prepared and send her to school, she will not be able to ready food properly. Therefore, whether the marriage is at eighteen or twenty-one years of age, if she did not able to prepare food properly, the marriage will be smashed.

**I**: Ok. How do you think the message about early marriage is being promoted in the community to prevent the occurrence of early marriage?

**P-5**: The community is being penalized by low not to marry at early age. The community is also not committing this early marriage. It is prohibited by low and this is being promoted in the kebele.

**I**: How much do you think one be penalized if early marriage is happened?

**P-5**: The law dictates a penalty starting 500 birrs to seven years jail.

**I**: Others? Do you know anybody being penalized?

**P-1**: We did not see anybody penalized with birr or jailed. We cannot talk about this since we did not see. There is no one who marry his daughter at early age.

**I**: How is birth spacing practically here? How are you doing with birth spacing?

**P-10**: We were telling you before. Those who have learn are applying it but those individuals who do not learn are suffering from the problem. Families who apply child spacing are properly caring their children, they will not face risk of hunger, and will have a capacity to led home. Instead of collecting mosquitoes who you would not feed them, it is good to care small family size which God gives you. The community is aware of this condition.

**I**: How many years do you think the gap should be between successive births for women?

**P-1**: If I have got a baby at this year 2010 E.C, I must add another baby by the year 2015 E.C. The next baby will be at good condition because I am adding a baby after the first baby is being matured. The mother also will not feel bad. However, if the mother gives birth within two years, the problem will affect me, my wife and the children. I am also being affected because instead of helping me she will be caring the two kids. I will then be going to carry the baby. Therefore, birth spacing is important.

**I**: How does the community looks accepts to the promotion?

**P-8**: The community accepts it and even the mothers have accepted it. Birth spacing is very good one, the children will grow well if God gives them health with current small plot of land. If there is no birth spacing, the child will be thin and the mother will be affected; her body will thin and exposed to other diseases. Her body will be weakened. Child spacing is very good.

**I**: How do mothers space births? Do they use contraceptives?

**P-8**: They are told to use [to mean contraceptives] to space births. They start using from the 7^th^ day after delivery. The injection is a problem. If you put injectable here [showing the place for implant on the hand] is hard. They will get dry. Since they do not take it all, it will be accumulated. But the tablet given for three months is better. There is a tablet for three months, six months and nine months. She can use this and this.

**I**: Which contraceptives do mothers prefer to use?

**P-5**: They use contraceptives for three months and six months in most of the times. There are different methods but they prefer to use the three and six months contraceptives.

**Section 6: Understanding communication and information sources**

**I**: Is there an opportunity in the community to discuss nutrition for women?

**P-1**: The question is about the day where women discuss about nutrition. Yes, there is. They come to the health post and discuss. After that, the leaders of the group will gather the members of the group and communicate them with the discussion.

**I**: Others? Is there an opportunity in the community to discuss nutrition for women?

**P-9**: Even if we do not know the exact date, they have meetings. They will plan a date for meeting and discussion. They discuss and evaluate themselves on how they have been done. But, I do not know the day.

I: We have discussed about the nutrition of mothers and adolescent girls. If you have any other ideas to be added in the area of maternal nutrition and adolescent girls?

**P-1**: The idea is about what should one up to eighteen years old adolescents and mothers be supported. For example, myself, I must ready a good meal to my daughters and my wife, to stay healthy and have good children. I should also give rest and good meal to my wife as well as I should help her. Therefore, females should get support from males.

**I**: Others? If there are ideas that are not raised during our discussion?

**Ps**: There are no other ideas

**I**: Thank you for taking the time to discuss these issues with me today. I have learnt a lot from you. As I mentioned as the start of the discussion, I will remove all identifying information from the report of this conservation. I will make you sure that no one can identify your comments. If you have any concerns or questions, please feel free to ask me any questions. Thank you very much for your time. You cannot say everything is clear and applied by the community but there is improvement from time to time.

**Ps**: Thank you! Hands on clapping…
