# Supplementary material for: Barriers to utilize nutrition interventions among lactating women in rural communities of Tigray, northern Ethiopia: An exploratory study
Source: PLoS One. 2021 Apr 30;16(4):e0250696. doi: 10.1371/journal.pone.0250696 (PMC8087028; doi:10.1371/journal.pone.0250696)
Supplement: S2 File — (ZIP) [file pone.0250696.s002.zip › S2_File.Doc/Community level Key informants/107_IDI_WDA_ Hastsbo kebele_Lalay machew woreda.docx]

**Operational Research on Adolescent and Maternal Nutrition in Northern Ethiopia**

**Introduction**

Hello my name is kiros, I am from Mekelle Universty; we are conducting a research on the factors that influences the nutrition of mothers and adolescent girls in collaboration with the regional health bureau and UNICEF. Year participation is very valuable; the information that you tell us will be used to improve nutrition programs and services for women and adolescents in the region and the country. We will not share your names when we report our results. The interview may take 1-2 hours and I would like to thank you for taking the time to speak with us today. You have the right to withdraw at any time and I will use tape recorder. Are you voluntary to participate for the interview?

**Yes** No

| **Section A: Interview details**   1. Zone: **Central Zone of Tigray** 2. Woreda: **Lealay Machew** 3. Kebele: **Hatsebo** 4. Name of key informant: **Miss Mulu Tadis** 5. Institution of key informant: **Women development Armey** 6. Interviewer name: **Kiros Tedla** 7. Date of interview: **19/11/2017** 8. Interview start time: **10:59AM** 9. Interview end time: **12:35AM** |
| --- |

| **Section B: Interviewee professional information**   1. Sex    1. **Female**    2. Male 2. Highest level of completed education.    1. **No formal education**    2. Primary education    3. High school    4. College education    5. Bachelor degree    6. Master’s degree    7. PhD 3. Discipline or field of educational training    1. Agriculture    2. Health (MD, nurse, health officer, midwife, pharmacy, etc)    3. Nutrition    4. Public health    5. Food science    6. Other (specify):**Women Developmental Army** 4. Current position: **WDA** 5. How long have you been in the current job/position:    1. ______ Months    2. **7** Years |
| --- |

**I:** Interviewer **P:** Participant

**Section I**

**I**, **what do women do to stay healthy in this community or worerda?**

**P**. pregnant mothers to be healthy first they will follow HF starting from 4 months of pregnancy and eat vegetables which helps to have healthy child. This also has benefit to the mother improving her health. The mothers should not also carry or hold difficult materials or heavy materials. We also advice the husband to help her as previously all activities were left for the women to do but now it is changed as the husbands are helping their wives like they bring water; thanks to our party and our government. The pregnant mothers also visit HF at least four times during pregnancy and should give birth at the HF. We WDA have 30 mothers below as so we mobilize the pregnant mothers to attend their pregnancy at HF and give birth here in the HF. We also educate the husband about the importance of following pregnancy at HF and to help his wife during cooking and child care. So the mother could have comparable rest.

**I, What about lactating mothers do to stay healthy?**

**P.** lactating mothers should feed their children like exclusive breast feeding before six months and immediately after six months she should give him balanced diet from home made foods or from what is available in the house. If she has no resource we communicate with the kebele leader and social affairs to be included in the safety net program. We do have an association here in the health post that prepare and provide a powder prepared from different crops found in our local area by an affordable cost. We also advice the mother to use iodine salt as it is important to the child to be healthy and improving his intellectual or school performance. We also advise the mother to feed extra-meal and take rest as if she does not eat very well she could not produce milk; hence the baby may become nutritionally poor or affected by malnutrition but we do not have much of such children.

**I, What do you advice the lactating mother to eat?**

We advise the mother to eat what is available in the house at least three times a day. As she is lactating mother she should take extra-meal and the husband should also understand this and help her to eat much.

**Ok,** **I, What about adolescent girls do to stay healthy?**

**P.** we and their family members advice them not to go to remote areas and to study very hard. All children above five years old to go to school and to feed very well to be healthy. They also keep their hygiene very well. Their families also follow them during schooling at our kebelle and when they move to the other areas for their high school education. We mobilize the community to send their children to school but most of the people are now aware the importance of education and are sending. I give myself as an example when I teach the adolescents about the importance of education as I would have been in the woreda if I was educated but my family married me when I was ten years old; and now see I am still here because of my illiteracy.

**I, what are the common nutrition problems in the community for pregnant, lactating and adolescent girls?**

**P.** there is a problem or disease on mothers resulted from both poor hygiene and feeding habit like swelling of the leg, eye infection and others which could put the mother up to hospital admission. This would have been resolved if we can eat with in our time from any available foods.

**I, so is there any mother or adolescent affected by malnutrition in this community?**

**P.** No we don’t have any mother of both pregnant or lactating affected by malnutrition now; there were one mother as I told you earlier. And she is already linked to the socials association for aid; we also give her some powder and become part of safety net program.

**I, What about children or mothers who are given Plumpnet or Fafa?**

**P.** there is no any children given plumpnet or fafa from my group but there are children who were given such help in other groups and also included in the aid provided by our association by preparing local powder. There are children who are below the standard weight in our kebelle when measured monthly at 16 or “Kidanemheret”.

**I, Who are measured during the screening process?**

**P.** all children with age of less than 2 years old, pregnant and lactating mothers are screened. There are no mothers of both pregnant and lactating who are affected by malnutrition but there are children who are affected by malnutrition and these children had improved their nutritional status after they are included in the help like our local powder or “Mtine”. Previously there were mothers who had been given Fafa but now it is stopped as we opposed it because it increases dependency or expectancy rate of the mothers. And we advised the mothers to use their locally available crops or cereals like been to feed their children.

**I, What about micronutrient deficiency like anemia, night blindness and goiter?**

**P.** there are pregnant mothers which are affected by anemia due to poor feeding or nutrition. Pregnant mothers are given medication but we advice them to eat the red “Taff” , vegetables like Karrot, none processed milk as the processed one could not replace the blood. We advice the mothers who have cows to drink the milk without processing at least a cap of milk immediately after milking the cow. We also device the mother to eat vegetables and soup of red “Taff” alternatively to improve her blood.

**I, What about on lactating mothers and adolescents?**

**P.** There is no anemia on lactating mothers as they are already treated during their pregnancy and the adolescents are also not affected by anemia as they are young.

**I, What about night blindness and goiter?**

**P.** there is no night blindness in our community. Night blindness is caused by poor nutrition due to lack of vegetables in our dish or during our feeding. So when we come to our community as we are reach in vegetables we eat vegetables that is why there is no such disease in our community. Because the community is advised by WDAs like myself and agricultural workers daily; hence, now the community had adopted the use of vegetables in a routine dish.

**I, What about goiter?**

**P.** yes there are mothers who have goiter. For example in my group there were two mothers who had goiter. The mothers had removed the goiter through operation and now they are free of the diseases.

**I, What do you think is the source of goiter?**

**P.** this is related with poverty as it a collected blood it is related with vaccination as there were no vaccination before but now there is no child affected by measles, Polio because of the vaccination. But now as the children are given vaccination immediately after birth like BCG and the community had use iodine salt the prevalence of goiter is decreasing.

**I, Is there non-communicable disease like diabetes in the mothers and adolescent girls?**

**P.** there is no in our community; there were one woman coming from the urban suffering from diabetes and now she is taking medications and she looks well.

**I, do you think the women or adolescent girls have height proportional to their age? Why?**

**P.** yes, they are proportional. The difference in the height is simply genetically but if their families are toll they become toll. For example, look at me I am short …………extended laugh…… but my son is toll as his father is toll. Therefore, height is genetically determined.

**I, Is it related with nutrition?**

**P. yes,** it related if someone did not get balanced diet may become thin, short with extended abdomen we call him “Zekfafa” because he drinks a lot of water. But when he get the balanced diet his nutritional status will improve and his abdomen also become normal. For example there was my neighbor here who has two children. She was very poor and one of her children become very thin, with extended abdomen and his head were very small. We advice her to give him soup of red “Taff” and finally he become normal with normal nutritional status.

**I, do you think the women or adolescent girls have weight proportional to their age? Why?**

**P.** yes, they do have weight proportional to their age in our community.

**I, why do think the children or adolescent and mothers have weight proportional to their age?**

**P.** this is because of continued follow up and care to the children. Before birth the children are followed in the health facility within their mother and after birth they are born in health facility and get vaccination and first milk of their mother or “Lgie”. They also get treatment for different types of diseases; hence children have weight proportional weight to their age.

**I, Is it related with nutrition?**

Yes, because children in this area have got foods containing vegetables and there is continuous education on child care and food fortification by health experts and agriculturalists the children have got weight proportional to their age. That is why we do not have a child with lower weight.

**I, Is there any children with overweight?**

**P.** yes there are some children having very high weight during delivery which is related with as the mother had been taking balanced diet during pregnancy. There were infants who were born with 4 kg and 2.5kg within my groups. The difference among the infants kg is because of difference in nutrition of the mothers. The former had been taken food very well whole the later might not take food very well. The health professionals in the health facility become happy when they see the child with 4kg and they this is very nice but practically the child with small weight will become very well when you see him after a month.

**I, do think the community have sustainable food for one year meaning can they feed their family without shortage for one year?**

**P.** no we cannot feed our children only from our farm rather we all members of the family work daily works to support to what is abstained from our farm.

**I, Is there shortage of food?**

**P.** No there is no shortage of food but there is no any rest as the male and female work together equally to fulfill their home. As a result there is no shortage of food in the house. For example I do have seven family members but our farm is very small which could feed us not for more than one or two months but everyone including me in the family works and support the family member.

**I, is there drought?**

**P.** this year there could be shortage of food as the crops were crashed with raining ice. Hence they do not contain any crop and we are afraid how the children and mothers could escape from poverty this year. We are expecting the government to support us food aids as we are left with no crop from the farm. The community is suffering from shortage of food and at this time the main victims will be mothers and children as the father can go to other areas to work but the mother will remain with her five or six children at home with nothing to eat. Hence; mothers will be the primary victims as the father will not be around even though the father sends 150 or 200 birr per month it could not be enough to the mother to feed her children. Hence, this year we are afraid but before we are not suffering from shortage of food.

**Section two**:

**I,** **what kind of nutrition interventions are in place to improve health of pregnant women, lactating women and adolecents?**

**P.** we do have a conference monthly at 12 or “Micheal” with pregnant, lactating mothers and their husbands. The HEWs give education on how to feed and teach the husbands to feed his wife when performing different activities and follow her during pregnancy for any discomfort.

**I, Who provide this? From where do they get it?**

**P.** The HEW provides this and the mothers get it here or health post.

**I, are pregnant mothers advised to visit HS for check up and services?**

**P.** yes, they are advised by HEWs very well. Pregnant mothers are advised to visit health facility, give their mobile phone to the HEW; we are also invited together with the pregnant mother. And they use ambulance service to go to the HF and back to their home. The ambulance riches at our village Hatsebo and there are also local ambulance (people who carries the mother) who gives support for mothers coming from village where the ambulance cannot reach. Thanks to the government it nice now.

**I, what services are given to pregnant mothers during their visit to HF?**

**P. M**others and their children are screened or measured for their nutrition and pregnant mothers are also measured their blood and if become anemic they are given treatment. They are also advised to eat very well. This is given monthly but if the mother had other diseases she may be appointed even after two weeks. Now the mothers are aware and following their pregnancy at HF and when there is any type of pain they phone to the health post or ask their husband to bring them to the health post.

**I, Do you think this is helpful to the pregnant mother?**

**P.** yes, because it protects the mother from excessive bleeding. In the past there were sinqakna meaning remained placenta. I do have experience about this when I was trained by Fana Health on delivery; there is a condition called sinqakna where mothers are affected by. This sinqakna is thin part of the placenta which remains in side. Hence, this may go up to the head or brain and may result up to death. This was a traditional believe which were practiced before but not now. To remove the sinqakna you have to be wise and careful with slow motion. But now there is such condition or sinqakna thanks to the health services provided. A number of mothers were died because of sinqakna; hence, the community is now using the health service to avoid death due to this. Therefore; giving birth at health facility had multiple benefits like avoids excessive blooding, avoids sinqakna, and the child will be kept clean and hygiene and this avoids diseases.

**I, What about on getting extra-meal and rest for pregnant women and lactating mothers?**

**P.** They get an advice as I have told you earlier. Lactating mothers are advised to eat many times when they feel hunger as they have empty stomach as the baby is already out. But pregnant mothers are advised to eat small amount reputedly as the baby is inside. The food content should be different like if she eat injera with vegetable now; she should eat injera with been or shiro latter. She should eat six times but at least she should eat four times in the morning, at lunch time, snack or in the middle of dinner and lunch and finally lunch.

**I, What is the importance of getting extra-meal and rest, and eating variety of foods?**

P. they will not suffer from anemia and malnutrition. The child or infant will also be healthy with normal nutrition as the infant gets his food from his mother through the placenta starting from four months of pregnancy. Lactating mothers will produce enough milk and the child will have good teeth, and healthy; the mother will also not disturbed by the child.

**I, What about on iodine salt utilization?**

**P.** iodine salt has different uses like it protects us from goiter and different psychological problems. It is put within a clean glass and mixed to the cooked at the last time of cooking in order not melt the salt and lost its contents. Hence, the mother and the child will be benefited from the value of the salt.

**I, on getting advice on nutrition sensitive agriculture such as home gardening?**

**P.** Yes we are getting the advice and we are producing vegetables like chili, salad, and others. We produce in winter and summer. In winter we seed by bringing water from river. We utilize the vegetables to ourselves.

**I, What about on the need to participate on the safety net program?**

**P.** yes there is safety net program and I am part of the program. Pregnant and lactating mothers are included in the safety net program if they are very poor and given the priority to them than the male as they are the high risk groups. As this aid would help together with what is available in the house; for example if she had one sheep she would have sold it if she did not get the help but now the mother will not sell the sheep and the sheep will become two or three and also feed the child.

**I, What is given in the safety net program?**

P. Money is given and it is 175 birr per individual per month but previously it was given 15 kilo of white per individual per month but they said 15 kilo but they could not be 15 kilo in reality. The aid is only for six months but the remaining six months it is not given. So the government should give the help for a year in order to bring the actual change. For example in “Adwa” they have given additional initial cost of 5,000 to build on it in addition to the safety net; it should be like this in our area as well.

**I, Is there any advice given to pregnant, lactating, and adolescent on water, sanitation and hygiene services?**

**P.** yes, everyone in our community uses toilet and open defecation is now becoming very rare. As there is education to avoid open defecation like any new comer should not get feaces in the open space and this is becoming taboo in the community. We also give education to mothers to keep their personal and environmental hygiene and to keep their children clean. If they do not keep their environment clean this will contaminate to what they are eating and become diseased. If they keep their hygiene the housefly cannot get the garbage as it is removed appropriately by putting in toilet and other containers.

**I, Is malaria common here? If yes, are pregnant mothers getting advice on the need to use ITN? Why? Who Advice them?**

**P.** there is no malaria in our area.

**Is there any treatment given to prevent intestinal parasites on pregnant, lactating mothers and adolescents?**

**P.** yes there is treatment given to children and the community with age of 18-45 years old for intestinal parasites. Pregnant mothers are given treatment when the pregnancy is above six months. Children with age of lower than five years are given and there is also school based treatment for students.

**I, Who provide this and for what purpose?**

P. HEWs give the treatment to the community and students at health post and at school. This is used to kill intestinal parasites.

**I, Is there school feeding program for adolescent girls?**

**P.** there is no school feeding program.

**I, Which of the above mentioned interventions is the most important for pregnant women? Why?**

**P.** pregnant mothers should eat-extra and take rest. The husband should also help her. The pregnant mothers should also strictly visit the HS and come even when they have head ach.

**I, Is there any challenge which affects mother’s utilization of the interventions or given services?**

**P.** there is no any problem as currently every one is utilizing the service.

**Section 3**

**I, What are the special things should women do to stay healthy in the community? ( during pregnancy, lactation and adolescence)**

**P.** she should deposit money as she may need money during delivery like she should deposit what she had got; it could be 50 or 10 birr as this may be very important during delivery in order to buy what she has needed or interested. She should also closely follow her pregnancy at HS and even after delivery for 45 days as the HEWs also follow her. She should also get extra-meal and rest; as she should not go out for 45 days.

**I, what is the role of the husband on improving maternal nutrition? How?**

**P.** he should bring water and should by from the market what the mother needs. He should not put her in depression and he should make her happy as she has his baby. We help them by advice; He should help her either by money or work and the mother will cook and eat for herself and her child.

**I, Do women in this community change their diets when they are pregnant and lactating? How?**

**P.** yes, they do change their diet. For example pregnant mother having pregnancy of two months will not eat maize if she was using this before pregnancy. This is because she hates eating maize because of the pregnancy, then her husband will bring another food like “Taff”and she starts eating. This food will not be shared by the family members; it will be eaten by the pregnant mother only.

**I, What is the recommended food for pregnant and lactating mothers to eat?**

**P.** mothers as I have told you earlier eat vegetables as we do have here in our area. This vegetable is suitable for the mother as it gives good health and they are advised with their husband in order to practice it in their home.

**I, Is there any food which should not be taken by the mothers ( pregnant and lactating mothers)?**

**P.** this is bad cultural believe and it was practiced in the past but not now. In the early times pregnant mothers were advised not to eat sauces containing red-pepper and they were also not allowed to eat meat as it was believed it may in large the baby and not allowed to drink broth (Merek). Adolescents were also not allowed to eat very hot sauces as it was believed it may in large their breast. But know all these are not on practice.

**I, What affects women diet during pregnancy and lactating?**

**P.** pregnant mothers lost their appetite during pregnancy or hate food which they were eating before but not for all as there are also pregnant mothers who do not hate. This is related with the health of the mother as if she is not healthy she would lost here appetite; hence she should eat what she has preferred to eat.

**I, What about gender disparities in women’s diet before pregnancy, and during pregnancy, lactation and adolescence?**

**P.** in the past yes there were such disparities like if the father is not around I would please eat the injera with salt. But now these believes are avoided as if there is sauces made of hen we will eat our part with my children by leaving his part for the father. Now there is no such practice as the children will not say yes for such believe.

**Section 4**

**I, Have you ever gone for nutritional screening during routine service delivery? If you want tell me about your experience?**

**P.** yes every time I have measured and they have told me that I do have hypertension and psychotic problem. I am also screened for my nutrition here in the health post and in the health center. But now they have diagnosed me with hypertension and I am suffering from head ach as the blood is coagulated in the brain and in my body.

**I, Who provide this?**

P. HEWs provide this in the health post and give medication like anti-pain. But they also refer to the higher health facility or Mayweyni or hospital.

**I, Is there community health days? Do you think they are important? what about to accessing routine services?**

**P.** yes, there is community health days to all community members weekly, at two weeks or monthly. As educated and non-educated do have difference in practice we educate during the community health days like to keep their hygiene, to construct and use toilet in their home, to have garbage container, if there is pregnant women to go to early to HF for follow up and check up, if lactating mother she should feed exclusively breast feeding until six months and after that to give food appropriately. There is also education on the husband should help his wife and those younger family members also should take their part and help. But now children with age above 18 years old are not ordered by the family members but they should take their part by any means. Here the father and mother should play their part to shape their children.

**I. During the community health days have you discussed about service utilization like institutional delivery?**

**P.** yes, for example if the husband is pressurizing his wife not to go to HF we will consult him not only in the community health days but also personally by giving him advice to send her to the HF and we left him after he believes that his wife is his children’s mother and he is happy to attend HF. We also advice them to send them at early stage of pregnancy and to make them happy and to avoid depression. For example; I may self developed this hypertension because I was so annoyed and irritated with my children when I came from meeting one day; and at that time I was in coma; admitted to hospital and developed this disease; now I am one month and one week since I become diseased. For every comes from absence of peace in the house if there is no peace in the house there is no health as hunger cannot kill by itself it may make you tine and put you in bed. And now thanks to the government we are resisting hunger and we can also buy from the market as everything is available even though it is costly. So if the mother has stress and not present in home; the children and the husband will be the first victims. Like for example if you have no light you cannot inter in to dark house and if your wife is not in home you will not inter in to the house. The same is for child as well if your child is not present you will not inter to your house even if your child is naughty. But father can live every where even though he has children; the burden is to the mother. There is one history my father told me; there was one female with seven children who leave her wife with her children to search for job. And he went to Humera and send 150 birr after a month but the mother were remained in a closed house with their children but with nothing to eat and this 150 birr were not enough and one of the children were swollen his leg and died because of hunger. House without mother or wife is dark; even a man with wife and without wife is different even in savings. The one with wife saves money but the one who have no wife may spent one or two hundred birr per day for drinking or other purposes.

**I, What are the challenges related with attending community health days and routine service delivery?**

**P.** there are individuals and women who do not attend regularly in the community health days as they say how many times we meet; I have already discussed the issues. There are old thinking’s like how our far fathers give births to us; why are they disturbing us by saying give birth at HF. So we work repeatedly to make them understand the importance and now the number is decreasing but still there are such problems. This is related with poor thinking or awareness or simply back ward thinking as for example if we change our clothes we say see what looks like this poor people for our neighbors. There is a problem mainly in general when people are becoming rich and start saying what do I ask them to do to me; I do have the materials and I know how to eat; as everything is with me I do get nothing from them new. Now we are targeting to such people with old thinking with kebele leaders by providing the health achievements like do we have mother did because of delivery, do we have children died now, do we have blind people and people with goiter. There were children who were undergo surgery mainly at the scrotum but now there is no.

**I, Do you think mothers should be targets for food supplementary? Why?**

**P.** this year we need because there is no crop production as it is crashed by ice. The mothers should also be given refreshment aid during their regular meeting at the health post as now they only get tee only. But permanently when we get children with lower nutritional status the government should provide the aid. As we are not sure that we may get a mother who is affected with malnutrition and have nothing to do. As you know you could not be sure even about your salary as the government can cut as they want. I can see from myself that during a training payment they cut at least half of the money and I always ask to self is the government know this as it is not rational. So if the government provides such aid we do not send the mother empty hand to her home but we could advice her please eat this and try to work and build on it. So if the government provide initial capital or help like family members are doing for their children when they start to establish family by providing the first materials like one quintal of crop. Government is also like a father or mother and below GOD so they need to bring at least the initial capital or help to protect the mother or child with malnutrition.

**I, are pregnant and lactating women beneficiaries from of soft conditionality of the safety net program?**

**P.** yes they are beneficiaries as pregnant mothers are excluded from the activities of the safety net program starting from six months of pregnancy and; after delivery they are free from the activities done until the baby is one years old. And after one year there are four or five female selected from the community to give care for the children at the work place and prepare food at the work place to feed the children. The care givers protect the children from snake bit, eating of soil and also provide shelter. Here the agriculturalists and health professionals work together and they give priority to children as they are the next generations they should not get sun light and should get shalter.

**Section 5**

**I, Do you think that delaying the age at first birth to after 18 is better for both the mother and the infant? Is it promoted in the community? Can you tell me who is promoting?**

**P.** yes, for female with less than 18 years old she is not physically fit and she is not even sexually ready as she is not reach the right time of maturation. There are females who were married early and now they hate their husbands because of their early marriage as they were not interested to have sex at that time. Hence; the girls hate their husband early. She may also get pelvic narrowness, Festula and she could not control her urine and others. The child is also not physically fit like his mother. But thanks to our government we do not have early marriage in our community; they married when their age is above 18 years old at 20 or 21 years old.

**I, what are you doing to promote delayed marriage?**

**P.** there is school based education on early marriage. I do have a daughter in grade seven and she told me that they are educated about early marriage and told me that they told us not to participate in any sexual activities and not to be deceived by men by providing money and if you have encountered any form of harassment use condom and contraceptive. There are also posters posted at different cites or places related to this. If there is early marriage the parents of female and the married male will be put in jail. The community is educated about this and knows it very well.

**I, How much should be the gap between successive births? Is it promoted in the community?**

**P.** it should be five years. Because the child to finish the care given by his mother very well even though it could not be stopped mothers care by their children. So if the child had got the appropriate care given by the mother he would become healthy or strong and academically active. But if the child does not get the appropriate care he would be affected by stunting even though he had full stomach. So child should get appropriate care from his mother by using contraceptives. There are mothers who are saying we become unable to give birth permanently due to contraceptive; the same thing also rose by their husbands by joking; as most of the mothers are utilizing family planning service. There are only very few mothers who give birth within two or three year gap but most are giving with four year gap.

**I, What is the effect of giving birth without gap?**

P. The mother will be suffering as she could not give care to both children at the same time. If the children were born separately the elder child will go with his father to the work place or even with his mother as he is strong. But if she gives birth without spacing or gap the mother will suffer as it is better to have twines than this. So this affects to both the mother and the child; the mother may be depressed.

**I, Can you tell me who is promoting and you heard for the last time?**

**P.** The health extension workers give the education using the WDAs who have 30 members below them.

**I, Why do think are some of the mothers do not use family planning services?**

**P.** they say that the treatment or contraceptive is not suitable to them and also say how can I use contraceptive after my first delivery; I should give my second birth or son. But lastly after they tested it by giving consecutive birth they came by themselves and say sorry I understand what you have told me before.

**I, Is there any barrier related to religion in utilizing family planning services?**

**P.** religion is already addressed as the religious leaders are consulted by the woreda leaders as well; it is not barrier. Rather they help us by giving education in the church.

**I, What better way do you think promoting better way? Is there community not addressed by the promotion?**

**P.** we should give education repeatedly during different meetings using posters containing the bad and the good practice of family planning like use of mothers who have carried a number of children due to birth without gap; poster of those babies born with or with out gap. WDAs are teaching like this to the mothers and to be effective and understand it very well we send WDAs for training to the woreda.

**Section 6**

**I, what kind of community conversations or massages discuss women’s and adolescents nutrition?**

**P.** We discuss about nutrition during community health days and we advice to seed and cultivate vegetables at their home by bringing at least 20-30 liter of water. We teach them like why do not you cultivate by yourselves in your home and if you don’t cultivate; bay from the market. Rather than filling the stomach with unnecessary materials it is better to fill with very small but quality food.

**I, Do all women get the massage easily? What are the barriers for access to information for nutrition during pregnancy?**

**P.** yes all mothers get the information easily including through written papers in each house hold. And there is no any problem related to this.

**I, Which information is effective for you to change your practice in nutrition during pregnancy? Why?**

**P.** the information given when all the community members are gathered is very important and effective. Because when you teach to all community members you can reach to all community members and you can easily take attendance through the WDAs but if you went through house to house visiting you may miss households. It makes easy to the health professional and other associations or structures as they can transfer their massage easily.

**Section 7**

**I, any other additional comment**

**P.** we WDAs are working very well and the community believes as we are working with perdium. But we do not have any payment or perdium our perdium is the life of the mothers when they are surviving with their children and living very well with their family members and our society at large. The community is saying they are calling as in order to get their salary or perdium but now days there is no perdium as before. Before health were paid highly but there is nothing now. In the 1990s there were trainings and I myself I have trained for one month but now there is no any training. We heard that there are trainings but nothing comes to the lower level; hence, the government should see us as we are working very well. Not alone for us those individuals are barriers for the success of the interventions should be asked individually. The government is not focusing mainly on WDAs even though we are working everything. We forget our home when others are working to strengthen their home. The government needs us only for his purpose to work but they do not consider us for payment or training at least once a year. We are not given the focus but they can evaluate our performance. The government is not present in the eye of the WDAs as we are not served.

**Summery points**

**Section one:**

- We don’t have any mother of both pregnant and lactating affected by malnutrition now.

**Section two**

- There is no any problem related to access and utilization of nutritional services as currently everyone is utilizing the service**.**

**Section three**

- Pregnant mothers should save money, visit HF for follow up and check up during pregnancy and should get etra-meal and rest during and after delivery.

**Section four**

- Mothers should visit HF during and after delivery and should eat vegetables with all what is present in the house.

**Section five**

- The programs or policies targeting delayed marriage and birth interval are working very well.

**Section six**

- There is community based discussion on nutrition mainly during community health days weekly, or monthly.
- **Finally I have finished my questions and I would like to thank for your time, patience and answering all the questions. Thank you very much!!! Thank you!!!**
