# Supplementary material for: Barriers to utilize nutrition interventions among lactating women in rural communities of Tigray, northern Ethiopia: An exploratory study
Source: PLoS One. 2021 Apr 30;16(4):e0250696. doi: 10.1371/journal.pone.0250696 (PMC8087028; doi:10.1371/journal.pone.0250696)
Supplement: S2 File — (ZIP) [file pone.0250696.s002.zip › S2_File.Doc/Community level Key informants/108-FGD-WDA-Hatsebo kebele_Lalyay Maychew woreda.docx]

Focus Group Discussion with WDA in Laelay Maychew woreda, Hatsebo Kebele

Zone: Central Tigray

Woreda: Laelay Maychew Kebele: Hatsebo

Facilitator’s Name: Measho G/slassie

Type of participant: WDA

Date of Discussion: 19.11.2017

Start time; 10:55 AM End time; 1:25 AM

Place of interview: Hatsebo

Participant Characteristics

| **S.N** | **Name of FGD Participant** | **Age** | **Marital status** | **Educational Level** | **Occupation** |
| --- | --- | --- | --- | --- | --- |
| 1 | Atsede G/wot | 40 | Married | Read & write | Farmer |
| 2 | Mamit Birhane | 35 | Married | 9^th^ grade | Farmer |
| 3 | Mulu Belay | 38 | Married | Read & write | Farmer |
| 4 | Alganesh Tadesse | 37 | Married | 4^th^ grade | Farmer |
| 5 | Yalem G/tsadik | 43 | Married | Read & write | Farmer |

**Interview:**

**I**: Interviewer/Moderator

| Name of FGD participant | Code |
| --- | --- |
| Atsede G/wot | P1 |
| Mamit Birhane | P2 |
| Mulu Belay | P3 |
| Alganesh Tadesse | P4 |
| Yalem G/tsadik | P5 |

**Section 1: Common maternal and adolescent nutrition problems in the community**

I: When I come to our discussion; from your observation, what do the pregnant women do stay healthy in this community?

P3: Well, I am P3 and I am the chairperson of the women development army. If I have heard or observed about the pregnancy of one woman in this community, I went to her home and I ask her slowly or through joking about her pregnancy and if she confirmed her pregnancy we advise her to get diagnosis and let her to register in the health facility. Later, we follow her whether she diagnosed or not and we also follow her to complete the four antenatal care visits during her pregnancy and to deliver in the health facility. After delivery, we advise them to; give colostrum, not to wash the baby, only breast milk until six months and of age and give balanced diet after six months of deliver. We also show them how to prepare balanced diet. I lead 28 women and I properly follow them if they are pregnant. We also follow the under five children and we advise their mothers to weight them, to maintain their hygiene and sanitation, to given them cooked meals, and also to give them balanced diet. We the WDA have been properly following and advising the mothers through going to their homes. We are educating them by saying, “a woman should not lose her life while giving a life”.

I: Good. What do the pregnant women do to stay healthy?

P2: To stay healthy, the women should get diagnosis from the date of conception to delivery and they should also deliver in the health institution. In addition, they should follow their antenatal and postnatal cares, and they should get health services by the health provides to maintain their health and their baby’s health. We have been also teaching them to vaccinate their baby up to nine month and to get all other services up their five years of age. So, we are teaching them starting from their conception.

I: Good, you are telling us that you are providing the follow up and advice for the pregnant women to stay healthy. But, what I want you to discuss is that what do the pregnant women do by themselves to stay healthy?

P1: Ok, I am P1. The pregnant women are coming to the health facility for diagnosis and the health extension workers have been giving them the required information about how many times a women should feed per day and to eat diversified foods including vegetables. They also advise them to feed three to four times per day because it is important not only for the mother but also for her baby. They have been also advising them to maintain their hygiene and sanitation through properly washing their hands, cloth and food equipment. We have been also advising them not to urinate outside their toilet and their toilet should also have soap and water for washing. So, we are organizing them to maintain their health.

I: Another opinion? Our main discussion issue is what do the pregnant women do to stay healthy?

P4: It is similar with what they have said. First we ask the suspected women whether she is pregnant or not and if she said yes, we advise her to get diagnosis in the health post. We also advise her to get HIV/AIDS diagnosis together with her husband. The health providers give her a tablet for blood shortage if she has the blood shortage. The pregnant women should get at least four antenatal care visits and she have to also deliver in the health post. The mother should give colostrum to her baby immediately after delivery and she shouldn’t wash the baby for within 24 hours after delivery. If the pregnant mother was eating twice a day, she must increase it to three or four times a day and she have to also eat from the egg, vegetables, milk and porridge. Anyway, she have to eat extra meal from the ordinary people because half of it for her baby. We have been also following the women for 45 days after delivery. The baby is also given immediately after delivery and after 45 days up to nine months. Exclusive breast feeding is given up to six months and additional food is started after six months. The baby is screened monthly and supplementary food is given if the baby has nutritional problem, but if the baby is normal his mother will be advised to keep on providing balanced diet like milk, porridge, vegetables and others.

I: Yes, this is what you have been advising the mothers to do to stay healthy. For example, there are traditional and cultural activities to maintain the mothers and their baby’s health. For example, I am from Raya and the mothers use traditional olivine smoke and butter to maintain their health. So, are there any traditional activities done by the women to maintain their health?

P1: What you have said is traditional practice and the “tush” (traditional olivine smoke) is not known in our community. The women may not move outside home before forty days of delivery because she may have some of bleeding associated with the pregnancy.

P2: As far as I know, naturally, the pregnant women do not carry heavy things and she don’t run like before her pregnancy and she shouldn’t share beds during sleeping.

P3: The delivered mother doesn’t have sexual intercourse with her husband within 80 days after delivery, she also don’t lift heavy things. If she doesn’t have economic shortage, she will stay in her house up to 80 days after delivery. So, there are no traditional practices like using smokes and others. Especially currently, the women maintain their hygiene and sanitation more than what we are talking. Our mothers were wearing a cloth with very narrow/tight at the abdomen, but now the women wear pajamas. Our culture is improving from time to time, for example, there is no any pregnant women who wear “shinshin” (a dress with very tight at the abdomen).

I: Good, you have discussed on what the pregnant women are doing to maintain their health. What do the lactating women do to maintain their health and their child’s health?

P3: We have a meeting with the lactating women and health extension workers in every St. Michael (12^th^ day in Ethiopian calendar) to demonstrate on how to prepare hygienic balanced diet. We prepare the porridge with two coffee cups of water and one coffee cup of balanced foods. The health extension workers also advise them to give this kind of hygienic additional foods for their child and it should be eaten immediately and if the porridge stayed some time, it should be heated before usage. So, both the lactating and pregnant women are given advice to maintain their and their child’s health. In the past our mothers were giving stone for distracting their child and the children were eating it. But now there is no similar scenario, instead the mothers travel with bottled water and food for their children. So, thanks for the Government we are very fine.

P1: The mother should have love for her baby and she have to support him at his back while the child is breast feeding. She has to also give the breast milk by swapping the breasts. So, the health extension workers and we are giving the education to the pregnant and lactating women.

P5: Starting from conception, they shouldn’t lift heavy pots; they shouldn’t carry heavy things while going to the market and they shouldn’t spend with the crowds during funerals, rather they should go early in the morning and return back before the crowd. So, they are eating balanced diet and they are maintaining their health very well.

P2: They should eat an extra meal from egg and other diversified foods. Her husband has to feed his wife through his hands because she may not eat for herself to breastfeed her baby.

I: Good. What do the adolescent girls do to stay healthy?

P3: If a girl reaches around 16 years of age, she can do everything. We advise them to use different protective methods like injections because they may face different accidents though some of them don’t listen to us. Nowadays, may God give us his mercy; the girls are having boyfriends at their early age. For example, may God give me his mercy, I have baby girls and I advise them to take care of themselves and prevent themselves by taking injections. There are no adolescent girls who stopped their education in our community and the teachers inform us in there are on the verge of stopping their education. If we have heard about the proposition of early age girl for marriage, immediately we call for the woreda and they come and cancel it, and they also advice the girls to continue their education. So, we are attempting to that extent.

I: Good, you have been educating the adolescent girls to stay healthy, but from your observation, what do the adolescent girls do by themselves to stay healthy in this community?

P2: The adolescents may face problems in their youth age, but we advise them to have condom and to get diagnosis for HIV/AIDS to prevent the problems.

I: What do the adolescent girls do to stay healthy in this community? What do you think P3?

P3: Just to repeat what has been said, we advise them to take care of themselves because it is very difficult to know what will happen and to differentiate who is having HIV/AIDS or not. In addition, we advise them to get diagnosis if they want have intimate friendship.

I: I want clarification on why the women shouldn’t have sexual intercourse within 80 days after delivery.

P2: Traditionally it is not allowed unlike the Muslim and if the mother delivered baby girl, it is prohibited by the orthodox religion to have sexual intercourse before 80 days after delivery. However, all people do not implement it. Let alone after 40 or 80 days, there are mothers who get pregnant during their delivery. For example, I remember a woman who gets pregnant before 40 days of her delivery in Mayweyni. The health workers had advised her to take contraceptive after during delivery but she didn’t accept their advice by promising to take it after baptism, but later she came back to the health facility after three or four months of her conception for abortion despite they were not willing to do the abortion service due to advanced age of her pregnancy. The mother suffered a lot due to her failure to use contraceptive method. Therefore, I have been advising all the women in my group to use contraceptive methods immediately after delivery and as a result my neighbors are using three or four injectable family planning methods. However, there are some challenging people who make an obstacle to our works. They tell us that you are working for your incentives and the contraceptive methods are not allowed by our religion. But, we strongly advise them that the pregnant women should not have a pregnancy before recovering very well from her delivery and to space between child births because if the woman gives birth after birth, both the first and the second children will not grow in a healthy manner. Hence, we are advising them and the women in my area are using the contraceptive methods after delivery.

P3: As it has been said, culturally and religiously (per the advice of the religious father) the women should not have sexual intercourse before 80 days of her delivery, but some stupid husbands may do it because she cannot say no to her husband. The woman is suffering from the problems associated with delivery and at the same time her husband may have the sexual intercourse because she cannot say no to her husband. Generally, this kind of problem is not common but there might be some stupid husbands.

P5: As it has been said, some people may not accept the prohibition of sexual intercourse before 40 and 80 days, but most of the women are using contraceptive injections that can protect them from pregnancy up to three or four years.

I: Ok, we will proceed now to our second discussion issue. What are the common nutrition problems for the pregnant women in this community?

P3: As far as I know there is no nutritional problem in our “kushet” because unless the women is lazy like me, the white teff, red teff, wheat, the legumes and the vegetables are in her hands. With the exception of rice all cereals grow in Hatsebo. I don’t know some women might be using the traditional practices otherwise every think is available in our community. So, I don’t think there are any women in Kebelle Hatsebo who have food shortage because every think is in our hands.

P2: We are fine, because education given every time in this community.

I: Education may be given for the women by the health workers, you or other providers. So, have you observed any malnourished pregnant or lactating women due to lack of awareness, shortage of foods or other reasons in your community?

P2: Some malnourished children are observed due to lack of awareness and we advise their mothers to take them to the health facility and they are given supplementary foods in the health facility.

I: What type of supplementary foods do they give them in the health facility?

P2: They give them the plastic packed foods and they become good.

I: Ok, what types of women are taking the supplementary foods in the health facility?

P2: The children of the uneducated and unable to understand our education are commonly affected. In addition, there are some women who entirely focus on their work by ignoring their child. However, nowadays, they are fine due to the education given to them. There was severely malnourished child in our kushet. I have visited their house five times. Later she gets improved after she took the packed food from the health facility and I was also preparing the balanced diet porridge for her. So, now she is healthy and the people are fine due to the education.

P3: I have similar opinion but to repeat it; the children from six month to five years old have monthly screening service for their nutritional status by measuring their weight and if they are below the standards, the health worker advise them to bring their child to the health facility and they give him the packed foods and the mother also feed feeds him additional foods at home. Unless the mother ignores the child, everybody knows it. If you don’t give clean water and hygienic foods using clean food equipment, the child will be at risk for diarrhea and vomiting. Feeding cannot protect them from nutritional problems unless you give them hygienic foods. So, if the foods contaminated by flies and you give it to the child, he will be sick from diarrhea and vomiting. You may take the sick child to the health facility, but you will not consider it as food related disease. Therefore, it is better to use toilets properly including the children and we have to also maintain the hygiene and sanitation of water containers.

I: Ok, you have told us that there are children who have been taking Plumpy’Nut from the health center or health post, so, are there pregnant women taking Plumpy’Nut?

P3: It is given only for the thin children who have low weight. It is not given for the pregnant women.

I: Do the pregnant women get screened in this community?

P3: Yes, they have been getting the screening services.

I: So, are there pregnant women who have been taking Fafa or Plumpy’Nut after nutritional screening?

P3: Fafa was given in the past, but there is no Fafa right now. Despite the mother is thin, she will not be given Fafa rather she will be advised to eat balanced diet to give enough breast milk for her child. She is advised to eat her own foods like different vegetables, milk and others because everything is with her.

I: Sorry P2 for the interruption, you can continue now?

P2: There were some women who have nutritional problems in the past, but now the problem is not observed due to the education given by the health extension workers. Now we haven’t faced either male or female who have nutritional problem.

I: As per your discussion, the nutritional problems are not common among the pregnant and lactating women. What about in the adolescent girls?

P3: There are no underweight or starved adolescent girls in this community. But, the problem is if they failed in grade ten, they don’t like continue their education instead they prefer to generate income by selling vegetables. So, they might be thin due to over thinking but not due to food shortage. For example, I have two daughters who engaged in vegetable trade after they failed in grade ten. In my opinion, their main focus is to sell the vegetables and save money. they are thin not due to starvation or disease, but it is due to their over thinking for their financial development.

I: Any other opinion?

P1: The adolescent girls do not have agreement with their parents as well as with their community if they fail in grade ten. They become nervous with their parents and other community members. But, I don’t have any awareness about adolescent girls who have nutritional problems.

P2: The adolescent girls do not have any nutritional problems. However, those who failed in grade ten are in crises. They don’t have agreement either with their parents or with their siblings. Those who have irrigation could be engaged in vegetable selling, but those who don’t have the irrigation are in crises.

I: Ok, Are there pregnant or lactating women who have blood shortage in this community?

P2: There were such problems in the past, where there was no health education, but now, thanks for the government we don’t have such problems in our community.

P3: I have not heard about those who have blood shortage in this community in my 20 years leadership.

I: Ok, are there pregnant or lactating women who have been taking tablets for the prevention or treatment of blood shortage in this community?

P3: We have been sending the mothers to the health facility, for example, I was given the red tablet for blood shortage five years back during my pregnancy in Axum. So, the women are taking the red circular tablet for the treatment of blood shortage in this community, but they do not like to take the while tablet because they believe that the white tablet increases the size of their fetus. When you say blood, we assumed that the blood directly given to the mothers, but the tablet is given to the pregnant women.

I: Is the tablet given only for the pregnant women or it is also given for the lactating women?

P3: As far as I know it is given to the pregnant women.

P2: The pregnant women are given the tablet for blood shortage while they come for diagnosis. Since they are taking the medication before delivery, we haven’t found any women who have the problem after delivery. This means, since they were taking the services during their pregnancy they would not face the blood shortage problem after delivery.

I: Is night blindness common in the women of this community?

P5: Yes, for example, I was not able to see anything after the sunset 18 years back during my last pregnancy. I had improved after they gave me drops from a blue plastic water container colored medications with necks. Later, I was also treated for trachoma. Sometimes still I have difficulty to see during the sunny hours.

P3: Sometimes, I have something that covers my eyes too. Occasionally, I couldn’t see the pen and paper to put my signature. When they hear that we have a meeting, many of them tell us to bring eye medications. Despite it looks normal they have something that covers it due to lack of butter (tsiediwina”) or others. So, there is a problem in this community?

P2: Most of the people have night blindness, but most of them have improved after they get treatment for trachoma. The people are now fine due to the services given by the government.

I: What are the services given by the government for the prevention of night blindness of the community mainly the women?

P3: The health workers have been advising us to wash our face with water and soap after using butter or other eye drops. In addition, some people say that they have improved very well after the trachoma service given in the community despite it is not completed eliminated.

I: Good. Is goiter disease common in the women of this community?

P4: The main treatment for goiter is iodized salt and it should be added after the stew is prepared. If the iodized salt is added during stew preparation, it will be evaporated by the heat. So, the main treatment for goiter is iodized salt.

I: So, is goiter common in this community?

P: There were people having very big goiter in the past, but I have not seen anybody who have goiter after the government has brought the iodized salt.

P1: There are still some women who have goiter. They are my close relatives and I have been advising them to use iodized salt and to get treatment for it. Their goiter increases during their pregnancy and delivery. So, it is commonly observed in our community.

P3: There are also a mother and her daughter who have very big goiter in this community. The size of their goiter is up their chest. Despite I repeatedly advised her to get treatment; she is not willing to get treatment services. Due to its very big size it is very difficult to cover it and she has also shortening of breathing during waking. There is also another woman who treated for goiter.

I: Why do you think goiter is commonly observed in this community?

P3: I don’t know it, her mother is very old and her daughter is almost equivalent with us. Her goiter was started during her early childhood.

I: Do they have other family members who have goiter disease?

P3: No, they don’t have it with the exception of herself and her mother. Her children and other siblings are normal.

I: How big is her goiter?

P3: She has very big goiter and even she has shortness of breathing during pregnancy.

I: P4 has tried to describe it before; do you think this community is using iodized salt?

P2: This community has been using iodized salt since very long time. There is no anyone who doesn’t use it. The health extension workers thought us to use iodized salt for the prevention of goiter and in turn we educated the women in our respective groups to use it. Currently every household is using the iodized salt. For example, there was a woman who had goiter in my group and I almost enforced her to go for treatment because she was not willing to go. After she got the treatment she becomes healthy and she is now blessing me for pushing her to get the treatment.

I: What about others? How do you see iodized salt utilization in this community?

P5: My daughter has invisible goiter. She has some feeling in her neck, tiredness and dizziness. However, she has been using the iodized salt in her foods including coffee for the last six years. But now she is relatively fine.

P1: Due to the strong work of the women development army, you will not get any household who don’t use iodized salt. The community is well aware that the iodized salt can prevent different diseases. We have been aggressively teaching the community to use iodized salts by going house to house as much as we have been teaching to construct and use toilets.

P3: The education about iodized salt utilization is given not only by the women development army but also by the Kebelle administrators and the FM is also describing about it in every morning. So, when we tell the community to buy iodized salt, they say that we already know it and we have been using it. They are using the non-iodized salt for cattle. The community knows how to use it properly (they use it after the stew is already prepared).

I: you have told us that the goiter increased its size during pregnancy and delivery. Do you know the reasons for its enlargement during pregnancy and delivery?

P3: I don’t know it. She says that the goiter becomes very large during pregnancy, but she relived little bit after delivery. I have been advising the mother and her daughter to get treatment but they are not willing to do so. She also says that I will only die by this disease.

P2: As to my understanding, the goiter is increasing during pregnancy because the pregnant woman’s capacity to withstand the disease is very low.

I: Are there any women who have diet related noncommunicable diseases like diabetes mellitus and hypertension in this community?

P2: I have not seen any women who have hypertension and diabetes mellitus in this community.

P1: I have not heard about someone who has hypertension.

P3: I have not heard about it and I don’t have it myself.

I: What other communicable diseases are common in the pregnant or lactating women of this community?

P2: HIV/AIDS and TB and my friend may add more.

P5: The problems are not common in this community, but my cousin in Axum was diagnosed to have goiter in Axum hospital and they referred her to shire for treatment. However, despite they have surgically removed it she is not still healthy.

I: What other communicable diseases are common in this community?

P2: He is asking us to discuss about the communicable diseases in this community and we have to understand the question.

P5: Yes, she becomes fine after she treated in the referral hospital.

I: P2 said that HIV/AIDS and TB diseases are observed in this community. So, why do you think the diseases are commonly observed in this community?

P3: The diseases are not as such common because, for example, there will be around 2 HIV/AIDS positive from 1000 people. The HIV positive people are those who have two or three wives in the town. If they are living in one to one relationship, the disease will not drop from the sky. So, if the husband is going from one to another, he will acquire the disease and transmit it to his wife living in his rural house. So, there might be 10 in 100 people who have HIV/AIDS. Unlike HIV/AIDS, you can acquire TB by itself without your mistake. However, you can only acquire HIV by your own negligence and it is like “calling for a dog to give meat”. We, the women are at risk for everything.

I: Well. Do you think the mentioned communicable diseases are related with nutrition?

P3: HIV/AIDS is transmitted by sexual intercourse, but I don’t think it is transmitted by foods and sharing beds and food equipment. However TB can be transmitted by sharing drinking equipment.

I: Good. The question is do you think the nutritional status of the one who have the communicable disease can be affected due to his illness?

P2: The HIV positive person may not disclose his illness, but we connect him with the health workers through smooth communication. Then, he will get the required nutritional and financial support from the government and health workers. So, he will not be exposed to nutritional problems.

I: Good. Are there stunted girls in this community? Stunting girls means those girls whose height is not proportional with their age.

P2: I have seen one stunted boy, but I have not seen the problem in the girls in this community.

P3: there are no stunted women in this community, but there is stunted boy.

P4: Yes, there is one person in this community who is very short relative to his age. He has a swelling in his back.

I: You have said that there are no stunted girls in this community. Do you think the stunted person you observed in your community is related with nutrition?

P3: His mother believes that the problem has been created due to the fact she was using contraceptive methods before her pregnancy. The feeding and sanitation practice of the women is so special and his siblings are very long.

I: Ok, are there another stunted people in this community?

P1: Yes, there is another similar girl who completed either 10^th^ or 12^th^ grade. She cannot move her legs and hands and there are also other disable people in this community.

I: We can see the disable people in a special case. Now I would like you to discuss if there are stunted people associated with under nutrition in this community.

P2: yes, there are two stunted males and one female in this community.

P5: The whole family of the stunted boy had acquired TB and the mother improved after making a surgery for her abdominal swelling, and the other family members has taken 60 injections each. Finally, one girl died and the legs and hands on the stunted boy that we are talking about becomes abnormal (it looks like a knife). But, he has made a little improvement after he got treatment in Mekelle.

I: In line with that, are there underweight or very thin adolescent girls in this community?

P2: Yes, there are so many thin adolescents who have swelling in their back, but I don’t think it is due to shortage of food. The swelling internally consumes their body. So, would nice if we get treatments for such kinds of diseases.

P1: Yes, there is very thin girl in our community due to food shortage. She was normal during her childhood, but later she becomes very thin and short as if she has sever disease. Now the school teachers are supporting her to continue her education. However, her twin sister is normal.

P4: There is also one girl in my community who has difficulty in walking.

I: Underweight means the weight of the adolescent is not proportional to their height or becoming very thin due to under nutrition.

P4: I was asking to know the reasons for her disability, but her problem is not related with under nutrition.

**Section 2: Interventions for improving maternal and adolescent nutrition**

I: What interventions or services are available in this community to improve the nutrition of women?

P3: The health workers have been advising them weekly in churches and meetings to maintain their personal and environmental hygiene like to properly wash their hands, their food equipment, to properly cover foods and to eat cooked foods. I also teach the 28 or 30 women assigned to me about their personal and environmental hygiene, not to get and stoles in our nearby home and to clean our house and house furniture. The health workers teach us and in turn we teach our respective groups about our personal and environmental hygiene and sanitation.

P2: If we want to maintain our health, we have to have a toilet because if there is no toilet we will defecate around our house and the flies my land in our foods and drinks from the faeces. In addition, if the pipe water is not transferred to clean water container it will be contaminated. Hence, we have to have very clean water containers and we have clean and dry our food equipment. If we maintain our sanitation, we will not acquire diarrheal diseases. So, they are teaching us and we are also teaching to our groups to have their own toilet and to maintain the sanitation of their foods and drinks.

P: Who teaches the community especially to pregnant and lactating women besides you?

P3: The health extension workers and other higher health workers teach the community through going house to house in the monthly holydays, for example, during 21^th^ and 27^th^ days of the month (in Ethiopian calendar). Together with the women development army they visit the pregnant and lactating women through going home to home.

I: Do the pregnant women get counseling about their antenatal care checkup?

P1: Yes, the health extension workers counsel them during the pregnant women meeting. They counsel them how many meals to eat per day and what types of foods to eat.

P4: In addition to what has been said, the pregnant women have been coming to the health facility on the St. Michael (12^th^) and Medhanialem (27^th^) days of the month (Ethiopian Calendar). In addition, the children and pregnant women have vaccination on every 16^th^ day of the month (Ethiopian calendar). During their meeting, the health extension workers teach them about their feeding, for example, if they were eating twice a day they teach them to eat three times a day.

P3: If we are talking about the pregnant women; in the past despite we were pregnant, we were equally competing and sweating with our husbands during sunny times, but now they are advised to take rests. In the past, rest during pregnancy was not known, but now due to the counseling given, they are taking rests and there is no any pregnant woman who travels without having umbrella.

I: Are there pregnant women who don’t follow their antenatal care?

P1: We don’t let them to stay at their homes. Since we are identifying and sensitizing them from the very beginning and we are giving them phone numbers, there is no any pregnant woman who doesn’t diagnoses and follows antenatal care. Even though all of them may not go to the health post, there is no any pregnant woman who doesn’t follows antenatal care either in hospital or health center.

P2: The health worker is following the pregnant women because we initially connect the pregnant women with the health worker. So, since the health working is following the pregnant women, she will not stop her antenatal care.

I: Good. How do you see the counseling given for pregnant and lactating women to prepare and feed diversified and balanced diet?

P: P2, P4 and I have been preparing the balanced diet for them in this house (indicating the nearby house). We have a meeting with all pregnant and lactating women during every St. Merry day (16^th^ day of the month in Ethiopian calendar). During our meeting we advise them to prepare the balanced diet by themselves by washing their own wheat, legumes and red teff, but if they don’t have the time to prepare it we advise them either to buy or to swap with other cereals from us. So, we have been selling the balanced food (locally call it “MITIN”) in every St. Marry (16^th^ day of the month in Ethiopian calendar) and they many of them come to buy it. Most of the time we advise the lactating women to take it for their baby, but there are also few pregnant women who take it for themselves.

I: Do the pregnant and lactating women get the advice to take the balanced diet for themselves apart from their children?

P5: The health workers have been educating the people to eat balanced diet in every Sunday at the church. In addition, they advise us to maintain our sanitation and to have separate areas for the liquid waters and covered toilets in our homes, and even she also teaches us to clean our nails.

I: Could you please describe how the MITIN (balanced diet) is prepared, who prepared it, its components and benefits?

P1: During the pregnant women education day, the health extension workers prepare a sample porridge using their own onion, egg and MITIN. They show us how to wash the equipment and how to prepare the porridge in a hygienic manner. They also advise them that it is important both for the child and for the pregnant and lactating women.

I: Anymore opinion?

I: You have told me that counseling and other services are given for the women for improving their nutrition. What services are given for improving the nutrition of adolescent girls in this community? When we say adolescent girls, they are the girls whose age from 10 to 19 years of age.

P3: There are eight to nine adolescent girls in my group. P1, P2 and I may advice our own daughters otherwise we don’t give attention for the adolescent girls. Even if the adolescent is very thin, we don’t ask her whether she has nutritional problem or not.

P2: Our main priority is for the pregnant women and children. We have not given attention for the adolescent girls. However, sometimes I advise my neighbors just like my daughters to have condoms with them because they don’t know what will happen. I also advise them to get diagnosed before their marriage.

P1: Our priority is on the lactating and pregnant women. We don’t give attention for the adolescent girls. Sorry I repeated what I have said before, when they failed in their education, they are suspected for going to the towns and for their relationships. If they went out of their home, their heart will be closed and they will go here and there. We are advising to our neighbor girls and others within our groups to prevent themselves from different accidents. However, we have not properly educated them. We can’t say we are educating them without doing it.

I: Why do you think attention is not given for educating the adolescent girls for improving their nutrition?

P3: The administrative bodies inform us to include them in different associations and groups. However, since they don’t inform us to monitor them why they are thin like the pregnant and lactating women, we don’t follow them. We may inform some of them to protect themselves from accidents, but we don’t advise them day and night as part of our job.

P2: We have been advised by the health extension workers to closely counsel the adolescent girl if she is suspected. But we have not used it as our main activity to counsel the adolescent girls. The health workers advised us that if you closely communicate with them they will talk with you. But we have not given attention for counseling them.

I: We have already touched it before; do you think sufficient advice is given for the community to use iodized salt?

I: We are not only talking about your work but also we are discussing about the general situation, for example, the health extension workers, other health workers and the Kebelle administration may have their own works on educating the community to use iodized salt. So, do you think sufficient advice is given for the community to use iodized salt?

P4: Yes, the health workers and we (in our respective groups) have been educating them in every meeting and as a result everybody is using iodized salt. The people use the non-iodized salt for cattle. Everyone understood its benefits. It is not only used for the prevention of goiter but also it is used for everything. So, education is given by the health extension workers and there are also other health workers who come from the woreda to visit the households in weekly bases to follow the availability of toilet, ITN, separate barn for animals and others. Generally, we have been giving the education to the community to have a toilet and of course there is no any household who do not have it. Hence, the health extension workers have been educating the community even in the churches.

I: Good. How do you describe the advice given to the women to cultivate home gardening in this community?

P4: Yes, during the summer season, we cultivate potato, spinach, cabbage and salad from the rain water. We have also irrigation for cultivating the vegetables during the winter season.

I: Do all the community have the irrigation?

P4: Yes they have. Those who don’t have the land are also cultivating by contracting lands.

P2: The health extension workers are teaching us to cultivate different vegetables in our homes. Accordingly, there is no anyone who doesn’t cultivate vegetables in his homes. We are cultivating different vegetables during the summer season and those who have irrigation continue their cultivation during the winter season.

P5: We cultivate the potato, spinach, cabbage and salad in the summer in our homes. After September, we again cultivate potato, spinach, cabbage and salad, red and while onion and others in our irrigation. We are also selling the vegetables in the market above our consumption. The health extension workers are also showing us how to prepare the porridge by adding spinach on it. We are using the home gardening.

I: Good. Is there productive safety net program in this community?

P3: Yes, it is available in this community. The poorest of the poor are included in the program. So, despite the included people have decreased due to their graduation, the program is available in this community.

I: Do the pregnant and lactating women specially supported by the safety net program?

P3, The pregnant women is not allowed to work. Does the pregnant woman have one year leave?

P2: Yes,

P3: The pregnant women have a one year leave from the safety net work and other environmental conservation works. The pregnant and lactating women are supported by the safety net program without working.

P2: The poor women households are included in the safety net program. If the women are pregnant, they will be supported for one year without work, but the people strongly resist their one year leave from the environmental conservation works. Some people are resisting their rest and they want them to work together with them after baptism of their child, but we didn’t let them to wok yet by communicating with different responsible persons.

I: After how many months of pregnancy are not allowed to work in this community?

P3: They say that they should work after forty days of delivery.

I: What about during pregnancy?

P3: It says they should work up to six months, but they don’t work at all because they will have some pains.

I: Ok. Do the pregnant and lactating women use insecticide treated bed nets in this community?

P4: Yes they are using it. There is no one who doesn’t use the insecticide treated bed nets during the mosquito breeding times while sleeping.

P1: We are living near the river in a cold area and all of us were sick with malaria disease and three or four of our family members including my husband and children were going together for malaria treatment. But now we are fine due to the insecticide treated bed nets and the house residual spray. The mosquitos are like bees, but we are using the insecticide treated bed nets. The pregnant women are using the ITN despite they have problems in constructing toilets.

P2: Despite we have shortage of the ITN; currently the pregnant, lactating and other people are using it. The priority is given for the pregnant women and the children. Malaria has dramatically decreased from the past due to the fact that we are using the ITN, residual spraying and we are also filling the areas where mosquito can breed.

P3: During the ITN distribution, priority is given for the pregnant women and children. The health extension workers are monitoring the utilization of ITN during their home visit. If there are some people who do not stretched the ITN in their bed, they discuss with us about the households who don’t use it. There was some shortage of ITN this year and we have also punished some pregnant women because they don’t prepare toilets. We told them that if you don’t implement our request, we will not implement your needs. There was malaria in the past, but now the problems are not available.

I: You have told me that there is not food shortage in this community. So, are there pregnant or lactating women who specifically targeted for nutritional support like Fafa and oil?

P2: Not available

P3: Before four or five years, the under nourished mothers and children were given Fafa and Plumpy’Nut, for example, I was told to have blood shortage and I was given Fafa in Axum health center. However, not even if the children are very underweight they are not given Fafa, instead they are given Plumpy’Nut. If the Plumpy’Nut is not available in this health post, they bring it from the health center. Therefore, there are no any women or children who take Fafa in this community.

I: Ok. Is there any medication given for the children or adolescents to prevent intestinal parasites and related malnutrition in this community?

P3: Yes a treatment of intestinal parasites is given every six months.

I: For who is given the treatment?

P3: It is given for the children every six months.

I: Good. Which of the nutritional intervention that we have been discussing are successfully implemented in this community?

P2: Prevention of maternal death like “a mother should not loss life while giving life”, prevention of child death from measles and cough. In addition, many people were dying from malaria though distending their abdomen. So, the biggest attention is given for the mothers and children.

I: Good. Let me remind you the interventions that we have been discussing and you will tell me the most successfully implemented interventions in this community; a counseling given for pregnant and lactating women on balanced diet, counseling on extra meal, counseling on antenatal care follow up, counseling given to use ITN, counseling given to maintain their hygiene and sanitation and others. So, which of the mentioned interventions are successfully implemented in this community?

P1: As it has been said, a mother should not loss life while giving life. Delivering mothers were suffering three to four days in their home, but now there is no any mother who delivers in her home. There is a change and we have made home delivery zero in our community. The mothers are delivering in the health post. The malaria is also eliminated by using the ITN and by residual spraying. So, everything is good. Our mothers were delivering at their homes just immediately after coming from harvesting, but now there is big change due to the availability of health facilities.

P2: Next to maternal and child services, toilet construction is also very successful.

P3: Our main back bone and result is “a mother should not loss life while giving life”. In addition as it has been mentioned, we also successful in toilet and hygiene and sanitation. Anyhow, all the interventions that we have talked are good.

**Section 3: Perceived need of women and adolescent girls**

I: Good. What types of services do the pregnant women need by themselves in this community?

P2: First, they have to build toile,

I: Well; what I want you to discuss is not what they should do, but what they need for improving their nutrition and health.

P2: The pregnant women say that “why don’t we deliver in this health post and why do you tell us to go Bringa, another time to Dego and other time to Myweini while we are suffering from the delivery pain”. So, the pregnant women want to deliver in their nearby health post and those women living in the areas where transport access is not available shout when we try to put them on the stretcher. Hence, the pregnant women are complaining about the long distance travel to get the health facilities.

P4: It is similar with what has been said, they want to get the health facility in their nearby and they want also to get electric power and other services like Myweini.

I: What about the lactating women? What do the lactating women need?

P1: When we advise them to take different treatments for their under five children in the health facility, some of them say that “why do we take our children to the health facility if there is no Fafa and you are nagging us for your own benefits.” Those who didn’t understand its benefit also say that weighting the child is meaningless unless they give them the Fafa and they also fear the evil eye. However, we have been advising them to see the status of their child through measuring his weight.

P2: The lactating women need rest to feed her child and she also needs balanced diet. They also need to maintain their sanitation and to vaccinate their child every time.

**Section 4: Implementation challenges and community factors affecting access to nutrition**

I: Good. What are the challenges you or other health workers have been facing while implementing the interventions that we have been discussing, for example, while counseling to prepare balanced diet, hygiene and sanitation, antenatal and postnatal care?

P3: There are very few resistant women in this community. When we advise them to vaccinate their child and to maintain their hygiene and sanitation, they say that “we didn’t grow up in such a way so don’t talk to us”. In addition, there are also some husbands who say that it is none of your business about the sanitation and hygiene of our wives. Therefore, despite there are very few women whom faze on our activities, the majority of the women are accepting our advices. Most of the community residents have been implementing our advices, but there are ten in hundred people who resist our advices.

P2: There are so many challenges from the women and husbands side. Even there are some husbands who are not comfortable with the vaccination of their child and diagnosis of their pregnant wives. For example, they say that your mother didn’t deliver you in this way. However, we have been taking the pregnant women for diagnosis after convincing her husband and herself. After repetitive visit and communication, we include them to the normal line.

I: You can explain the challenges in relation with the community culture and their level of understanding and attitude.

P1: They have already described it. Most of the community members have learned and understood it, but there are some women and males who resist our interventions. For example, when we go home to home, some of them say that “those women who belongs to the toilet and wastes are coming” (laughing). Some of them also demoralize us by saying walk through the ashes of others and you have a big position to tell us to dig a toilet. Hence, despite we don’t give up our confidence; there are some challenges in the community. I always laugh when somebody call me “belongs to the waste” (laughing).

I: What should be the role of the husbands for improving the nutrition of their wives?

P2: We have to educate them. If they are educated they will come to the normal line. But, it needs very strong effort because it is very tiresome to teach them.

I: what should the husbands do to improve the nutrition of their wives?

P4: We have to tell them repeatedly and they will come to the line.

I: We have already discussed before that the pregnant and lactating women should eat additional two or more times per day to improve their nutrition. So, what should be the roles of the husbands in improving the mothers’ nutrition?

P2: The husband have to tell his wife to take rest and if the women is lactating and breastfeeding her child, he should feed her through his hands. In addition, he has to reduce her workloads.

I: Good. Another opinion

P3: I have similar opinion; he has to advise her to take rest. All men are not equal and similarly all women are not equal. For example, some women may hesitate to eat again after eating together with her husband. However, if the husband is wise unlike me, he will advise her to eat more again for herself and for providing more breast milk to her child. Currently, the husbands are carrying their child, not the wives. They are too much caring for their wives. Even in the rural areas, the husbands are caring their children. He may tell her to feed herself and feed her child; otherwise everything is within her hand, and she is giving the food to her husband he is not giving her the food.

I: In our previous discussion, we have said that the pregnant women should eat two or three extra meal per day, but do the pregnant women implement is practically?

P3: We are teaching them to implement it, for example, the health worker is advising me to eat hygienic food and to use toilets, but they don’t see me what I am eating and where I am urinating. So, we are teaching them to eat very well and their newborns are very good. The children would not be good, unless the feed good. So, we are teaching them, but we don’t see them while eating.

P2: We don’t only provide the education but we can also see them how they are feeding. We have been teaching them in any meetings, social gatherings and any other invitations. We have been teaching them to feed properly not to loss capacity during delivery and to prevent blood shortage. So, they are feeding very well because had not been for that they wouldn’t have good newborn.

I: What types of foods are recommended for the pregnant women?

P4: If the pregnant was eating two times per day before her pregnancy, she has to eat four times from porridge, soup prepared from red teff, linseed and milk during her pregnancy. If she eats diversified foods, it will be good for her baby and she will not decrease her weight.

P3: It is good to drink milk if she has at her home, but if she doesn’t have milk, she has to use red teff, linseed, honey. Honey is very important for our uterus. It is good to take three tea spoon of honey in every morning. In addition, she has to eat porridge, and she has to sleep in the evening after she washed her legs and after she took soup. Since the spinach, salad and other vegetables are there, they are always using it. Nowadays, the women are very good in terms of using different foods and vegetables. Even if they don’t have the vegetable garden, they can buy from me or other neighbors.

I: Are there any foods which are not recommended for the pregnant women in this community?

P1: Despite the education is given and gender equality is declared, the rural life for the women is difficult because they have very big influence and they have big responsibility in their family. She will not have more time to prepare porridge or others for herself. In fact some of the women who have very small family size may prepare good foods for themselves. So, more education should be given for their husbands. Though their husbands say that the women are taking sufficient rest, practically they have more workloads at her house and agricultural areas, especially they spend the whole night working during the autumn season.

P2: Traditionally in the past, the pregnant women were not allowed to eat honey because they assume it is heavy meal for her. Almost all the testy foods like egg, meat and milk were not recommended by some people traditionally. However, now the pregnant women are advised to take all important foods and they are taking it as well. The women can take now small and diversified foods from honey, meat, milk and others.

P3: The pregnant women are allowed to eat roasted legumes especially pea because it has “chiramo”. Traditionally, whenever we get the pregnant women in social gatherings we have been advising them not to eat roasted pea, but there are no other foods which are not allowed for the pregnant women. I myself was not eating roasted pea while I was pregnant.

I: What is chiramo?

P: [All of them said abdominal pain at the same time]

P3: It is severe abdominal pain during delivery

P4: It is real. If you eat the roasted pea, you have severe abdominal pain for three days during your delivery.

I: Why do you think the roasted pea has an associated abdominal pain for the pregnant women?

P1: Because it is row.

P3: We don’t know it. Our mothers were telling us not to eat the roasted pea and we have been also telling each other in social gatherings or homes not to eat it to prevent “chiramo”.

P4: It is very severe pain like delivery, but it doesn’t kill you.

I: What other things influence the feeding of pregnant women?

P4: There are no other things with the exception of roasted pea.

I: Ok. Do the pregnant women screened for their nutritional status by the health extension workers and other health workers?

P3: Yes the pregnant women are being weighted in every St.Michael. We have different dates for different “kushets”. For example, the pregnant women and the children of Semere Kushet are screened on the 7^th^ day, Mishilam and Adi-Siye Kushet are screened in the 21^th^ day and Endakidaneminhet kushet are screened on the 16^th^ day of the month. So, we have monthly screening schedule for the pregnant women and children in their respective kushets, which means they are not supposed to come to this health post.

I: Good. Is there monthly community health day in this community? It is a diagnosis and treatment service at the community.

P3: There was a treatment campaign for the old people in every six months, but there is no any other service in this community. Even now when we talk about the treatments for the children, the old people request us to bring medications for them. They also inquire us that why the government dislikes the old people and they also say that the government only likes the women and children.

I: Is there a community health day for providing health services for the children, women and others in the community?

P1: Yes, there is eye diagnosis and treatment campaign by the regional health. They have provided the service twice or three times so far.

I: Do they come in monthly bases or not?

P3: They have come twice so far and they have provided the services in this health post and in Myweini. I have also heard that they will come on the16^th^ of November (In Ethiopian Calendar). So, they were only providing eye treatment services.

I: So, there is no community health day at Kebelle level?

P3: We don’t have community health day.

**Section 5: Other interventions that influence adolescent and maternal nutrition and health outcomes**

I: Do you think delayed first birth (after 18 year of age) is important for their nutrition?

P1: Since they are in their childhood age, they will be exposed to different risks. It will not have any benefit.

I: The question is delaying first birth to after 18 years of age?

P1: I thought it is before 18 years of age.

I: What are the benefits of delaying their first birth to after 18 years of age on their nutrition and health?

P3: If they deliver after 18 years of age (at 19 or 20 years of age), they will be healthy and they can control their child properly and their life as well. However, if they deliver before 18 years of age, they may face different accidents because they are not matured.

P2: If they married after 18 years of age they are matured and they know what is bad and good, and they will not face problems during delivery. However, if they delivered before 18 years of age, they may be at risk for fistula and other problems.

I: Another opinion?

I: You have discussed many health benefits of delaying first birth. What about its benefits for improving their nutrition?

P2: It will help them to maintain their health, diagnose themselves and they also deliver in health facilities. In addition, they also accept the education given to them and it will also help them to take balanced diet.

P3: since they have mature body, they will not face problems during delivery and they will have good feeding practice and management.

I: Do you think sufficient education is given for the community to prevent early pregnancy and early childbirth?

P4: We have been educating them to prevent early childbirth and to prevent early sexual relationship. If they married in their early age, they may be at risk for different problems.

P3: As we have said before, if a 16 or 17 years old girl is proposed for marriage, all concerned bodies including the woreda, women affair and group leaders will go to her house to prevent her from early marriage. Then, she will be advised to continue her education. We have been also closely advising those adolescent girls who are suspected to have boyfriend in Axum or Shire to protect themselves from different accidents. Early marriage is not allowed by rule. If there is early marriage, they will be punished around 5000 birr and it has also 5 to 10 years prison sentence. Currently, with the exception of few, many of them are not willing to marry before 22 or 26 years.

P2: First the awareness is given to us. If one early marriage is suspected, the women development army leader is expected to identify her. Then the information will be disseminated to the top bodies. So, the women development army leader is creating awareness in the community to prevent early marriage.

I: In your opinion, how many years should a women space between births?

P1: A mother should deliver in five years interval.

P3: In addition to what has been said, in the past we were delivering in three year’s interval due to lack of awareness. But, after we get the contraceptive methods, I have delivered in a four and five years interval, even this child (the child whose sound is recorded in this discussion) delivered after six years interval. Therefore, since it is very important, everybody has understood it. So, the correct birth spacing is four or five years.

P2: Childbirth spacing is very important and we have been educating them to space their births. In the past, the women were delivering in every year, which was very dangerous both for the women and its children. However, now we are advising them to deliver in four years interval and they are applying it.

I: What are the benefits of birth spacing for the improving the nutrition and health of the mother and her child?

P3: It is not equal between delivering every year and every four or five years. If the woman is delivering in every four/five years, she will not suffer so much and the child is also landed from her back. However if the mother is delivering birth after birth, the mother will putrefy from their urines and she will suffer in between the children without recovery. Neither the mother nor the children will be good. The mother will be suffering a lot with the four or five small children. But, if the mother is delivering in four or five years her backbone will be fully recovered and she will be healthy. Let alone human being, we have been told to conserve and not use similar seeds every year. So, the advantage of birth spacing is to improve the health of the mother.

P2: If the mother delivered birth after birth, she may end up losing her life. However, if she delivered in long interval, she may not loss her weight/body and she will not have blood shortage, and the child also grow up in a healthy manner. She will also be saved from death.

I: Do you think the message about child spacing is reached in the community?

P1: In fact the community has understood its advantage and disadvantages. In the past, there were so many mothers who deliver birth after birth. For example, we were advising a woman to take contraceptive methods, but she said that I don’t like the injections because I want to have more children. Later, she was seriously in danger after her baby was died inside her abdomen. Finally, she cried and regretted very much for her failure to use family planning methods. She also said that I almost left the rest of my children in dark to give birth for one additional child. Therefore, almost majority of the community are using the contraceptive methods. We don’t have now a mother who gives birth yearly or every two years, because everyone is aware of the benefits of contraceptive methods.

P3: We don’t have a woman who delivered in every morning. If there is a woman who delivered every three years, the people faze her that “you are delivering in every month”. There is also a relatively young woman in our community who is pregnant for her 10^th^ times. The people faze her that, “are you attempting to deliver dozens”. She is in my age, and they are joking at her that you are left with only two to fill dozen. Currently, we can only get a pregnant woman after searching many times, for example, I have only one pregnant woman in 28 women. Even the last child of the pregnant women is approaching to four years. So, starting from myself, almost most of them are using contraceptive methods. Now, you don’t need to speak about contraceptive methods, because they have been coming to the health facility to take contraceptive methods by themselves.

I: Are there good opportunities to prevent early marriage and for promoting childbirth spacing in this community?

P3: As I have told you, the woreda and Zone have given high emphasis for the early marriage. It is directly reported for the woreda and women affairs if there is early marriage in the community. As to the child spacing, they chose the contraceptive methods by themselves and they don’t need an advice it use it. We are not advising them to use contraceptive methods; because we are observing them that they have three years injective method in their hands and in their uterus. Nowadays it is normal to talk about the contraceptive methods they have been using, but they were hiding it in the past.

P2: The community has accepted it because it is our agenda in our women development army meeting. Hence, they have accepted the education given about it.

**Section 6: Understanding communication and Information Sources**

I: You or other health workers may teach the community about nutrition. However, do the community discuss about their nutrition?

P3: Yes, they have been discussing about their nutrition in every two or three weeks in the church. If the health extension worker is available it will be good, if not Kushet and group representatives provides their advice them about their nutrition during the discussion. *Let alone the human nutrition, they are teaching them about their cattle’s food I.e straw in the churches.*

P2: The health extension workers together with us have been teaching the community about their nutrition in every Sunday in the churches and they have accepted it.

I: What are the sources of message for women nutrition in this community?

P2: First the health workers teach the women development army and the women development army in turn teaches the community in his group.

I: Good. Another opinion?

P1: The health workers teach us in meetings and churches and we (the chair persons) in turn teaches our groups using our respective networks.

I: Good. You have been teaching the women to improve their nutrition, but from where could the community get nutrition related messages besides from you?

P2: They can learn from the health workers and from guests.

I: Any other source of message?

P3: They preferred the messages given by the health workers, but they don’t reject our messages too.

P2: The main source of information for the community is from the women development army.

I: Do you think the community; especially the pregnant and lactating women can get the messages easily?

P1: All the strategies, activities, vaccination and rules are transferred to the community by the women development army through their respective networks. Everything is smoothly reaching the community due the women development army and their connection with their networks. So, they are accepting it.

I: From your observation, are there any challenges to accessing the nutrition messages by the community, especially for the women?

P1: As we have told you before there are some obstacles in the community. There are some people who give us very bad feedback. Still some problems are not solved. Some people perceive that as if we are working only for our benefit. However, we have been convincing to the challenging people that we are strongly working to improve the health, sanitation and other problems of the community.

I: Finally, from your observation and experience, what types of messages are quickly accepted by the pregnant or lactating women?

P2: We have to convince them not by telling the information, but by going to their home and by slowly talking with them. If the mother says “I don’t have it and I don’t know”, you should not go back immediately because the mother is not getting the services. *However, if you want her to get the service, whatever she tell you, you have to enter in to her home and talk about her problems and discuss with her in a way she understands you.*

I: Good. Another idea

P3: It is similar.

**Section 7: additional remarks**

I: Good. As we have informed you in the very beginning, the main aim of this study is to identify the factors in influencing pregnant and lactating women and adolescent girls of this community. So, if you have anything to add or if you say there is any unmentioned thing you can reflect it. If there are any challenges which are not mentioned in our discussion, you can mention it.

P1: Everything has been described and we have also mentioned the challenges. As my sisters said, we have to discuss with them in kind without becoming emotional. As I have told there are some people who faze on us while going home to home. There are stupid people who say ‘those belong to the toilet are coming”. However, we advise them slowly that I am not going to use your toilets; rather if you properly establish and use the toilet, you will prevent from different disease.

P2: I think we have not described the activities done before and after toilets. We have to wash our hands using soap after toilet; we have to prepare waste disposal sites; we have to prepare also liquid waste disposal areas.

I: P1 said that there are some people who have negative attitude to the advice given to them. So, how should we convince them to accept us and who should advise them?

P2: the women development army should advise them.

I: How?

P2: since the guideline is cascaded to us, we have to educate them to maintain their sanitation in a way they understand it through repeatedly going to their homes.

I: One last question; we have said that the adolescent nutrition is not given due emphasis in this community. So, what do you think should be done on this issue?

P2: We have to appoint them in our respective groups to give them education.

I: Another opinion, if any?

P3: As my sister said (P2), if there is anyone who doesn’t understand it, we have to advise him the advantages and disadvantages of it. There is no anyone who cannot understand things if education is given to him. For example, I and those who spend in their homes are not equal. You gave me a lot of education, but the one who spend in her home will ask me about where I spend the day. If I tell her that we have discussed about the pregnant, children and adolescent girls, she will ask me about what I have learned from this discussion. Finally, she will understand me, if I tell her slowly about what I have learned about the pregnant and lactating women. If her husband tells me to stop what I am teaching to his wife, I will advise him more about what his daughter and his pregnant wife should do to improve their health. So, if I advise him in such away, he can understand it. However, he will not understand it, if I tell him to keep quite or to stop talking. Hence, they can learn if we teach them after we learned ourselves.

I: Thank you very much for scarifying your time to participate in this study.

P: [ Eleelelele!!! clubbing their hands]

**Summary**

**Nutritional problems**

- Most of the nutritional problems are not common in the pregnant and lactating women and the lactating women of this community, but they reported that night blindness and goiter are sometimes observed
- There is nutritional screening , but there is no targeted supplementary feeding for the pregnant and lactating women in this community
- There is no nutritional screening for the adolescent girls

**Nutritional Interventions**

- Almost all the nutritional interventions are available in this community except the TSF
- Among the interventions, home gardening is widely practiced in this community
- The pregnant and lactating women have regular monthly meeting to discuss about their nutrition and health
- The nutrition interventions are targeted for the pregnant and lactating women and their under five children but not for the adolescent girls

**Implementation challenges and community factors affecting access to nutrition**

- There are some resistant women and husbands in the community
- There are also some people who faze on the responsibilities of the WDA
- There are some food taboos for the pregnant women in this community e.g. roasted pea
