# Supplementary material for: Barriers to utilize nutrition interventions among lactating women in rural communities of Tigray, northern Ethiopia: An exploratory study
Source: PLoS One. 2021 Apr 30;16(4):e0250696. doi: 10.1371/journal.pone.0250696 (PMC8087028; doi:10.1371/journal.pone.0250696)
Supplement: S2 File — (ZIP) [file pone.0250696.s002.zip › S2_File.Doc/Community level Key informants/111_IDI_Health woreker_Hatsebo HC_Lalay Maychew woreda.docx]

**Operational Research on Adolescent and Maternal Nutrition in Northern Ethiopia**

## **Tool A**

## **IN-DEPTH INTERVIEW GUIDE,**

## **School teacher**

Hello, my name is Omer Seid. I am from Mekelle University. Thank you for taking the time to speak with me today. We are doing research on the factors that influence the nutrition of mothers and adolescents in collaboration with the Regional Health Bureau and UNICEF. Your participation is very valuable. The things that you tell us will be used to improve nutrition programs and services for women in the region and the country. We will not share your names when we report our results.

Do you agree to participate in this study? YES

| **Section A: Interview details** | |
| --- | --- |
| **Questions** | **Answer** |
| Zone | Central Tigray |
| Woreda | Laalay michew |
| Kebele | Mayweni |
| Name of key informant | Frewini Kassahun |
| Institution of key informant | Mayweni health center |
| Interviewer name | Omer seid |
| Date of interview | 19,November 2017 |
| Interview start time | 10:33 AM |
| Interview end time: | 11: 40 AM |

| **Section B: Interviewee professional information** | |
| --- | --- |
| **Questions** | **Answer** |
| Gender | Female |
| Age in year | 24 years |
| Highest level of completed education. | Diploma in midwifery |
| Current job/position | Maternal and child health unit care giver |
| How long have you been in the current job/position: | 2 year |

**Section 1; Common maternal (pregnant women, lactating women and adolescent girls) nutrition problems in the community**

I: What are the common nutrition problems in the community for women and adolescent girls?

P: The first one is women’s and girls’ didn’t know how to feed themselves. It is not because of absence of food, they have, but they don’t know the proper way to feed themselves. But really some woman doesn’t have enough food for feeing themselves.

I: Is there severely and moderately malnutrition women’s in this community? or women’s that are under that supplementation of plump nut or FAFA?

P: We have supplementation of plump nuts for children but have not for mother.

I: what about FAFA for women’s?

P: no, we don’t have. Am working here in the last one year and I never saw FAFA given for mother. A year ago, before I coming here I was working another woreda health center, there was FAFA for woman.

I: Where it is, you previous woreda?

P: Hawzen woreda

I: What about micronutrient deficiencies such as anemia, night blindness, goiter among pregnant?

P: Yes, pregnant mother of this area are anemic due to bad feeding habit. Feeding during pregnancy is not similar with the pre-pregnancy feedings. During pregnancy the mother should eat additional foods. During pregnancy there may be health problem which prevents food intakes of the mother as she like.

I: What is that health problem that affects the dietary intake during pregnancy?

P: Most of the time mother said, they dislike eating foods. There are some mothers that are not taking foods during pregnancy.

I: What about micronutrient deficiencies such as anemia, night blindness, goiter among lactating mother?

P: Lactating mother do not have anemia. Have no laboratory facility in this health center to assess anemia, in short.

I: Anemia, why not assessing by sign and symptoms?

P: We do assess pregnant mother by the sign and symptoms of anemia, but lactating mother uses health posts for health cares, they are not come the health center for health care.

I: What about the problem of anemia among adolescent girls in this area?

P: It is fine; anemia is not problem of adolescent girls

I: What about night blindness among pregnant and lactating mother and adolescent girls?

P: Eye problem, it is observed among old peoples. I didn’t see night blindness among pregnant and lactating women’s and adolescent girls of this area.

I: What about the problem of goiter among lactating and pregnant mother and adolescent girls?

P: Among pregnant and lactating mother there is goiter but I didn’t see among adolescent girls

I: Does women’s of this area are short (stunted) and Underweight?

P: There are short and thin women’s in this area

I: Why woman of this area is short and thin?

P: I think it is because of shortage of food, and genetic related causes

I: Is there overweight pregnant, lactating and adolescent girls in this area?

P: yes there are some overweight women in this area and, majority of women has average weigh.

I: What about diet related non communicable diseases like hypertension and DM among pregnant and lactating mothers, and adolescent girls?

P: Yes really there are hypertensive pregnant mother, and I don’t see hypertension among lactating and adolescent girls.

I: What about food insecurity women?

P: Majority of the community is food secured, but there is some food insecure. Majority of pregnant and lactating women’s do not have food insecurity problem, but sometime some mother has complaining problems of food insecurity.

I: Which women groups are most affected by these nutrition problems?

P: Pregnant and lactating women’s and under five children.

I: Why?

P: Because these group need additional foods compare to others. Off course all people needs balanced diet but pregnant and lactating mother and Childress need more.

I: What about adolescent girls?

P: They need also additional

I: Why?

P: Because it is necessary.

I: Yes it is necessary for everybody but why additional for adolescent?

P: Because adolescent is growing age and there is a physical change so they need additional food.

**Section 2: Nutrition priorities in the woreda**

I: Do you think it is necessary for your institution to get involved in work aimed at improving maternal nutrition? Explore for pregnant women, lactating women and adolescent girls.

P: Yes, it is good

I: Why?

P: It is supportive for them

I: What support?

P: What you say? If there is NGO for supporting supplements it is good to improve material nutrition

I: Do you have intervention to improve nutrition of women’s?

P: Yes, we are doing on advising of mothers consuming the available foods they have.

I: Do you have IFA for pregnant mothers, nutrition counseling for women?

P: Yes we have doing theses activities?

I: Good, I think you are doing nutrition interventions but I need further. How can your institution at this level involved to improve maternal adolescent nutrition?

P: Do mean as the health center

I: yes, as the health center?

P: We can give IFA for pregnant and lactating mothers, measuring the weight of pregnant mothers

I: What maternal nutrition (pregnant, lactating and adolescent girls) interventions are the priorities in this woreda?

P: For pregnant and lactating mothers, at the health post the HEW has demonstrated balanced food preparations systems. We have health education programs; we do educate one additional food during pregnancy for example if the women have three meals per day we do counsel her one additional meal. For lactating mother we do educate two additional foods form her usual food intake and also we do educate the mother about appropriate child feeding practices.

I: My question is interventions for women?

I: We do give IFA for pregnant mother, checking the pregnancy but because of lack of laboratory we do not do hemoglobin analysis, urine analysis and blood type analysis.

I: Why do you advise to use bed net? What is the importance of using bed net?

P: If they are infected by malaria, in particularly pregnant and lactating mother will be anemic. We advised the whole family should use if there is enough bed net if not priority will be given for pregnant and lactating women and under five children’s.

I: What about productive safety net intervention?

P: We don’t have

I: Advising family planning’s for lactating mother?

P: Yes we are doing

I: You can tell me any other intervention you are doing for pregnant and lactating mother?
P: How?

I: Oky, for example are you advising iodized salt utilization?

P: Yes,

I: Please tell me any other intervention?

P: Advising about nutrition, using bed nuts, counseling about danger signs of pregnancy, advising about hygiene.

I: What messages you give for related to the hygiene?

I: Educating mother how to keep cleanness of their home, their surrounding environments, and their personal hygiene, washing of their hands.

I: You said nutrition, what are the nutrition messages that you are advising for mothers?

P: Ya, I told you above. If the woman is pregnant we advise her to talk one additional meal from the usual, if the women is lactating we advised her to take two additional meals from the usual intake.

I: Regarding to rest?

P: Yes we advised, we advised mother to avoid heavy works.

I: What nutrition services do you spend most of your time on?

P: We give more time for pregnant mother intervention.

I: From pregnant mother nutrition intervention for what intervention you spend most of your time?

P: laughing …. Seems not comfortable with the questions, she said I need help form my staffs and said nothing.

I: Can you tell me some of the successful maternal nutrition interventions that you have implemented in this woreda?

P: Silent,

I: But she speak the general nutrition interventions

I: Can you tell me some of the successful maternal nutrition interventions that you have implemented in this woreda?

P: Silent,

**Section3; Nutrition interventions that improve adolescent and maternal health**

I: What kinds of nutrition interventions are in place to improve adolescent and maternal health in this woreda?

P: Do you have nutrition screenings for women’s?

P: Yes we have

P: Can you mention some of the nutrition intervention that you are doing for women’s?

P: Is it for pregnant?

I: Yes, and then you can tell me also for lactating and adolescent girls?

P: For pregnant mother we do measuring her weight. There is a problem if the mother reduced weight from her previous measurement and likely if there is excess weight gain from the previous measurement still the mother has a problem.

I: Do you have MUAC measurements for pregnant mothers?

P: Yes we have for under five children’s but we it is not for pregnant mothers. For pregnant mother we do weight measurements.

I: What about MUAC for lactating mother?

P: Yes it is done in the health post but not here. Here we are not doing measuring MUAC for pregnant and lactating mother but in my previous health center we do MUAC for them and then based on the measurement result we give FAFA/plump nut. In this health center nothing will be given for pregnant and lactating mothers so measuring their MUAC do not have importance.

I: Is that only for the supplementation of plumb nut or FAFA for measuring MUAC?

P: We are not measuring MUAC but we are assessing their weight, if mother weight is decreased from the previous we do counsel about appropriate feeding for its improvements, but if she has sever weight loss we do refer to Axum hospital.

I: What bout WASH activities?

P: Yes we give advice for both pregnant and lactating women. After birth we give IFA for lactating mothers, advising sanitation and hygiene and about family planning’s, counseling immunization of her child.

I: What about rest for lactating mothers?

P: Yes we are advising them

I: I wish to know the counseling’s you do for this women?

P: We advise to take rest with slight exercise for 30 minutes.

I: Do you have interventions for adolescents?

P: Health education about family planning’s that we give.

I: Do you have nutritional screening for adolescent girls?

P: No we don’t have.

I: Use of water, sanitation and hygiene services?

P: Yes we advise them though the health education program.

I: Any additional intervention for pregnant, lactating and adolescent girls?

P: For pregnant mother we have HIV test, taking all vital signs, measuring weight, giving TT vaccinations, giving IFA.

I: What about lactating mother?

P: Similar to pregnant, measuring all vital signs, testing HIV and giving IFA for three months after deliver.

I: Any other? Do you have advising about dietary diversity?

P: yes,

I: Do have deworming?

P: Right now we don’t have the deworming drug in the health center but before it is finished we gave after three months

I: Why deworming?

P: To prevent anemia

I: Have you vitamin A supplementation program for women?

P: Yes we have for lactating mother after delivery. We did give, but now we stopped to give because it is prohibited to give vitamin A after birth.

I: Why it is prohibited?

P: Do not know the reasons, but we have commanded to stop giving

I: Have you youth friendly services in this health center?

P: Yes we have

I: What is the role of youth friendly services?

P: Giving family planning, ANC follow-up, whatever the case she comes if the women’s have age between 14-24 years, she will get service in the youth friendly service units.

I: In your opinion, which of the above programs are being implemented successfully (i.e. in the most effective way?) Why? **Explore for pregnant women, lactating women and adolescent girls.**

I: Giving IFA and HIV testing are the successful intervention for pregnant mother. IFA to prevent anemia and testing HIV is important to prevent mother to child transition of HIV.

I: What about lactating mother?

P: It is similar

I: Which one?

P: The nutrition intervention is the successful

I: Which nutrition intervention?

P: All are successful…laughing

I: What about adolescent girls?

P: We do not have specific nutrition care for adolescents; sometimes we give abortion care only. Bay the way it is new health center that is the reason for not giving enough interventions for adolescents but still there is assigned person in the youth friendly service.

I: In your opinion, which of the programs mentioned above are less effective? Why? **Explore for pregnant women, lactating women and adolescent girls.**

P: Please go to the next question.

I: What are the implementation challenges that are specific to delivering the maternal nutrition interventions in the programs that we have been discussing? **Explore for pregnant women, lactating women and adolescent girls.**

I: Does Lack of awareness on nutrition related problems is challenges?

P: Doesn’t have challenge on this because we are the one who gives the service.

I: Do mean awareness level of mother doesn’t have impact on utilization of the nutrition services?

P: Not, am not saying that, mothers come to us, she could be award or not award about the service, and then we do counsel and gives the services for both mothers. So for both we give advices then they can use the service equally.

I: If you are not giving counsel how could they use the available nutrition services?

P: If they don’t have awareness how could they use the service? So, awareness determines the available service utilization. Even the literate/educated mothers will not use the service unless she has awareness.

**Section 4; Community factors affecting access to maternal nutrition interventions**

I: Can you think of barriers that prevent adolescents and women from using the programs and interventions that we have discussed? **Explore for adolescent girls, pregnant women and lactating women.**

**I:** Educational level of mother, is that barrier for service utilization?

P: The educated women can use the service better than none educated one. Educated person is better for the service utilization.

I: How or why?

P: It is not the same, the literate and illiterate person. The illiterate person needs small information to use the service but the illiterate person needs more information and has difficult to counsel the illiterate’s person. But here we give the same information for both the literate and the illiterate person about the service, so they will have similar awareness and then the will use it.

I: What community related beliefs and norms are preventing access to the interventions? How?

P: I don’t know, because mother has knowledge about the service in my opinion no such kind problem. Rarely some mother refused to take IFA and their reason is their blood will be increased when they take IFA and they have perceived it is not good for their health. For such mother we do counsel.

I: Are the interventions culturally acceptable? Why and why not?

P: Currently no culture related barrier. There was but nowadays it is changed.

I: What are these changed practices and how they changed?
P: Because it has been done strong awareness creation to change these behaviors, so the community awareness is good now. Formerly it was not allowed to give tea and chicken for young females but now it is changed.

I: Are the interventions accessible to the women and adolescents? ..Interims of transportation and cost?

P: Interims of cost the service for pregnant and lactating women is free so has no problem. But for adolescents it is not free, and I will not say all adolescents have equal access interims of cost. Some adolescent may not afford to use the service.

I: What about transport access?

P: There is transport problem, some mothers delivered before they reach to here, because of transport problem. Some mother has living a far from the health center so they give birth at their home because of the distance and lack of transportation, as a result last year we lost one mother because of home delivery because her home is too far she couldn’t come here as a result.

I: How conveniences are interventions to the women and the adolescent girls?

P: Yes it is convince for them…with laughing

I: How do you explain the quality of the interventions?

P: Yes it good, but not complete… with laughing. There is no laboratory facility so it is a gap.

I: What resources exist to provide the interventions and what do not exist?

P: We tried to give all service by using the available resources wisely. We don’t have enough rooms, but what we did is giving more than one service in one room, for example the outpatient department we give all services. We don’t have laboratory facility and laboratory technician’s professional. Relatively when we come to the MCH unit it is good.

I: How do you evaluate the commitment of the intervention providers at your level?

P: All staffs are committed and we give our time to give the service

I: Do have challenge on shortage of materials?

P: Yes there is shortage of materials. We don’t have laboratory .

I: For these challenges that you mentioned, can you tell me of any solutions that your institution have applied to effectively implement the interventions for women and adolescent girls? Specify the each solution done for each challenges?

P: We use the available resources wisely. We are giving four/five services in one room because of its shortage e.g. in the outpatient department room we do give also emergency services.

I: Can you tell me of any solutions that your institution have applied to effectively implement the interventions for women and adolescent girls?

P: Build rooms, availing laboratory facilities and recreating lab technicians

**Section 5: Multi-sectoral collaboration to improve maternal nutrition**

I: Do you feel it is necessary at your level to work with other sectors/institutions to address maternal nutrition? What about for adolescent girls’ nutrition? Why?

Probe for:

P: Yes

I: Why it is necessary?

P: To help pregnant, lactating mothers.

I: That good, which other sectors do you feel are necessary to work with your institution?

P: We can do with other respective institution

I: Can you mention some of these respective institutions?

P: I don’t know them

I: Ok, let me rephrase my question, can you work jointly with school to improve women nutrition?

I: Yes we can

P: What kind of intervention you will do jointly?
I: Giving health education for students in the school. Then students will teach their family while back to home

I: What about another sector or institution?

P: laughing.. and said Enough

I: What about the agriculture sector?

P: Silent

I: How do you evaluate the level of collaboration among sectors in nutritional interventions? Why do you think is so?

P: We are not working jointly nowadays, but it nice to work jointly for better achievement. Ya coordinated work is nice for good achievement

I: For multi-sectoral action that effectively works to improve maternal and adolescent nutrition at your level, what kind of change in terms of the way stakeholders work together is needed?

P: We should meet together it could be monthly or any other time, then doing work jointly with the education, agriculture and kebelle leader.

I: Is there coordinating platforms in enhancing multi-sectoral coordination in maternal and adolcent nutrition?

P: No we don’t have

I: To what extent does your institution participate in the multi-sectoral nutrition coordinating body at this level?

P: I don’t know multi-sectoral nutrition coordinating body

**Section 6: Other interventions that influence adolescent and maternal nutrition and health outcomes**

I: In your opinion, why would delayed marriage (after 18 years) improve maternal nutrition?

P: Early marriage should be stopped

I: Why?

P: Because if they married early; they are not ready and their body is under the growing age, they will exposed to fistula and it is tough for such mother to give care for their children (if they give birth).

I: In your opinion, why would increase the space between each birth improve maternal nutrition?

P: Increasing birth interval is import. Yes, increasing birth interval reduces the economic pressure of the family for growing the children and, if they have many children it become hard and has economic pressure for the family to grow those children. If there is low family size it nice for the come

I: What is the importance of increasing birth interval for the mother?

P: Yes, has importance on reduces workloads of the mother. The workload is not the same if we compare mothers having many children versus mothers having few children.

I: What is the importance of increasing birth interval for the child?

P: For children it is important, if children are few they will get the necessary cares from the family.

I: How do you explain early marriages in this area?

P: Has no that much early marriages

I: Why they do early marriages?

P: It is due to family pressure, not because of the girls interest. By wishing positive things for their girl the family do practicing early marriages, but I don’t think the family knows the consequences of early marriages. They family feel prudes if their girls get married, however it bad for the girl.

I: What programs or activities promote increasing birth intervals in this level?

P: Yes, we have giving family planning’s.

I: Any other programs/ or intervention?

P: We educate the importance of family planning, to reducing the economic pressure of the family

I: Can you tell me any other programs or policies in place in this woreda to prevent early marriage?

P: There are activities; telling the consequence of early marriages during community gatherings

I: Any other program?

P: Silent

I*:* In your opinion, are these programs or policies effective? Why or why not?

P: Yes, because nowadays it has nice states they are reducing

I: Can you think of any more programs or policies? Think about political, religious and other influences.

P: It requires additional efforts, if anyone interested to support

I: What additional efforts?

P: lounging…..

I; For example is that possible to do activities though using religious?

P: Yes, teaching the regions leaders and teaching the community together with religious leaders, the community has better trust for these peoples so the community has better accepted the massage from the religious person than from us.

I: Is that possible to do activities though using politicians?

P: Yes, they may be share responsibilities, so their involvements is nice

I: Can you think of any other opportunities to prevent early marriage and increasing birth spacing?

PROBE:

P: Awareness creation can be done at schools, religious institution and working with kebelle leaders.

Additional Remarks

I: What lessons have you learnt regarding adolescent and maternal (pregnant, lactating and adolescent girls) nutrition at your level?

P: Currently it nice because their family income is good

**Summery**

**Section 1; Common maternal (pregnant women, lactating women and adolescent girls) nutrition problems in the community**

Common nutritional problems are

- anemia
- Goitre
- Stunted and Underweight

Pregnant and lactating mother are more risk groups

**Section 2: Nutrition priorities in the woreda**

- Yes it is necessary to involve to do work aimed at improving maternal nutrition,
- Has nutrition intervention for pregnant and lactating mother
- Has no nutrition intervention for adolescents

**Section3; Nutrition interventions that improve adolescent and maternal health**

Intervention are

- IFA supplements, deworming
- Measuring weighing
- Counselling on one extra meal and rest during pregnancy
- Counselling on two extra meal during lactations
- Advice on water, sanitation and hygiene services
- Advice on Insecticide treated bed nets (ITN) use

Giving IFA and testing HIV are the successful intervention for pregnant mother. IFA to prevent anemia and testing HIV is important to prevent mother to child transition of HIV

**Section 4; Community factors affecting access to maternal nutrition interventions**

Barriers are:

- Educational level of mother
- community related beliefs and norms are preventing access to the interventions
- Shortage of transportation
- Poor quality of care

**Section 5: Multi-sectoral collaboration to improve maternal nutrition**

Can work jointly with

- Schools

**Section 6: Other interventions that influence adolescent and maternal nutrition and health outcomes**

Early marriage should be stopped because if they married early;

- Their body is not matures
- Mother will exposed to fistula
- Such mother will not give adequate care for their children

Increasing birth interval is import.

- Increasing birth interval reduces the economic pressure of the family
- Reduces workloads of the mother
- The child will get the necessary care
