# Supplementary material for: Barriers to utilize nutrition interventions among lactating women in rural communities of Tigray, northern Ethiopia: An exploratory study
Source: PLoS One. 2021 Apr 30;16(4):e0250696. doi: 10.1371/journal.pone.0250696 (PMC8087028; doi:10.1371/journal.pone.0250696)
Supplement: S2 File — (ZIP) [file pone.0250696.s002.zip › S2_File.Doc/Community level Key informants/112_IDI_Kebele leaderL_Hatsebo kebele_Laelay Maichew Woreda.docx]

OPERATIONAL RESEARCH ON ADOLESCENT AND MATERNAL NOTRTION IN NOTHERN ETHIOPIA

TOOL B

IN-DEPTH INTERVIEW GUIDE FOR KEBELLE LEADERS AND WOMEN DEVELOPMENT ARMY

PRINCIPAL INVESTIGATOR: DR. AFEWORK MULUGETA

DATA COLLECTION

IN-DEPTH INTERVIEW FOR KEBELLE LEADER IN HATSEBO KEBELLE, LAELAY MAICHEW WOREDA, CENTRAL ZONE, TIGRAY, NORTHERN ETHIOPIA

AXUM, ETHIOPIA

NOV 19, 2017

**Information sheet and consent**

I: Good morning. My name is Yasin Jemal. I am from Mekelle University. We are doing research on the factors that influence the nutrition of mothers and adolescent girls in collaboration with the Regional Health Bureau and UNICEF. Your participation is valuable. The information that you tell us will not be shared with others. However, the information will be recorded and used to improve nutrition programs and services for women and adolescents in the region and in the country. I have several questions to ask you that we have prepared in advance and we will ask you all to say what you think about each question. Ensuring privacy of everyone here not to speak what we discuss outside of this group is strictly not allowed. The interview will take 1:30-2:00 hours. Do you have any questions before we begin? If you have any concerns or questions as we proceed please feel free to let me know. If it is alright with you, I will turn on the tape recorder now.

Participant: Ok, I agreed to participate

= = = = = = = = = = = = = = = = = = = = = = = = = = = = = = = = = = = = = = = = = = = = = = = =

**Note:**

I: Interviewer

P = Participant

= = = = = = = = = = = = = = = = = = = = = = = = = = = = = = = = = = = = = = = = = = = = = = = =

**Section A: Interview details**

| Zone | Central zone |
| --- | --- |
| Woreda | Laelay Maichew |
| Kebelle | Hatsebo |
| Name of key informant | Tegenesh Tesfay |
| Institution of key informant | Kebelle office |
| Interviewer name | Yasin Jemal |
| Date of interview | Nov 19, 2017 |
| Interview start time | 11:37 AM |
| Interview end time | 12:53 PM |

**= = = = = = = = = = = = = = = = = = = = = = = = = = = = = = = = = = = = = = = = == = = = = = =**

**Section B: Socio-demographic Information**

| **Socio-demography of Key Informant** | |
| --- | --- |
| Sex | Female |
| Age | 30 years |
| Marital status | Widowed |
| Higher level of completed education | Grade 5 |
| Current job/position | Kebelle Leader |
| How long have you been in the current job/position | 00  Months    01  Years |

**= = = = = = = = = = = = = = = = = = = = = = = = = = = = = = = = = = = = = = = = = = = = = = = =**

**Section 1: Common maternal (PW, LW and adolescent girls) nutrition problems in the community.**

I: What do pregnant women do to stay healthy in this community?

P: To stay healthy, one pregnant women should get balanced diet. Second, cleanliness and the third is education. However, the most important thing before education is to obtain balanced diet such as vegetables to grow physically but to do this they required education. Thus, they learn two times a week. For example, women development army taught the pregnant women, lactating women and adolescent girls on balanced diet two times a week. So, to grow physically the fetus/baby in their womb, they should eat balanced diet and should change the food they ate during breakfast in launch time, and to change what they ate during launch in dinner time, and also to increase the frequency of feeding. Therefore, the women development army follow the pregnant in this way. One pregnant woman also requires medical examination after 4 months of pregnancy and to go every month to health facility for checkups and if the woman has some problems like anemia and others by then, she will get medication and they were given advice by the health workers to eat different foods to obtain what they have missed in their meals. Accordingly, there is follow up in this Kebele.

I: What else?

P: To keep stay healthy?

I: Yes

P: To keep stay healthy, they should have to make connection with women development army and the main thing that help them stay healthy is that if they are organized and to work with government jointly hand in hand. Cleanliness about food, cloths, body and the environment is also required. There should also be toilets. The environment should also be clean. There should be separate environment for animals (cattle). Keeping your environment in the way that cannot breed that of mosquito.

I: What do you add to stay healthy pregnant women?

P: They need insecticide treated bed nets (ITNs) not to be bitten by mosquito since she has a bay inside and thereby to prevent diseases. They also need balanced diet and the food should be clean to make the fetus/child grow healthy. Cleanliness is also required. The child also required vaccination every month to grow healthy. She should also take vaccination for herself if not her diseases will be transmitted to the fetus/child. The pregnant women should take vaccinations five times during pregnancy.

I: What else to adolescent girls?

P: To grow adolescent girls in care, the women development army gives advices to the mothers of adolescent girls to provide them balanced diet as they are the next generation (tomorrow’s leaders). In addition, advices are also given to themselves (i.e. adolescent girls).

I: What else?

P: I don’t have any other. Ehiiiiii (laughing)

I: Good. In your opinion, what are the common nutrition problems in the community for Women/Girls?

P: The problems caused by nutrition problems?

I: Yes.

P: There are many diseases caused by nutrition problems.

I: What are these?

P: Because a mother requires to prevent malaria. The other is kidney stone and is caused if a woman/girl eats salty foods. Another problem is that if they eat foods that has stones, it may cause stomach diseases. In addition, if they use non-iodized salt, they may develop goiter and to prevent this advices are given to woman/girls.

I: Is there goiter in this community.

P: Yes, there is.

I: What else?

P: There is kidney problem

I: What else in children?

P: If they are not vaccinated, they affected by measles.

I: I am asking you nutrition problems

P: Yes, measles is caused by lack of proper feeding and lack of vaccination. If the mother didn’t properly feed her child, the child will be emaciated, swell his stomach, become underweight and these cause complications to the child.

I: What else?

P: I don’t have any other

I: There are many nutrition problems such anemia, goiter, stunting and others. So, are these nutrition problems in this community?

P: Not this much. Actually, there are pregnant women who suffer from anemia. There are also women who suffer from shortage of food. Because of this, there are women who faint while they are sitting. This is also caused due to lack of feeding and poor food preparation in a hygienic way. Therefore, mothers and children are suffering from lack of balanced diet.

I: What else?

P: There are children who are short in the community. This is caused and seen in mothers who didn’t listen to get vaccinated or who terminate the vaccination while they are pregnant. The other cause is if there is “Ziwar” that is a traditional belief that happened when a pregnant women having coquina (Za’egol) rotates the other pregnant woman while sit down so that the fetus in womb will be affected/emaciated. There are also women who have diabetes in the community though I don’t the type but I taught that it is caused due to lack of proper nutrition. The other problem in our community is night blindness among elders especially in the previous time but it is not seen in adolescent, pregnant and lactating women after the start of the medicine that is given every 6 months and follow up by health workers before and after delivery. So, night blindness is reducing currently and the farmers/elders are now improving their sight.

I: In your opinion, how do you explain the risk of malnutrition for women?

P: The risk of malnutrition is that there are women who don’t have farmlands and still they couldn’t get farmland since there is land scarcity. These mothers have children and suffer from shortage of food. Thus, we have these mothers who are at risk of malnutrition and we thought that they are affected and suffering from shortage of food.

I: How sever the nutrition related problems you have mentioned are among the women in this community?

P: Woaaa… the question how sever the nutrition problem is that it may be severe in those female household leaders who have no farmland since these women are lactating mothers, they may suffer from nutrition problem though the problem is not this much (not common).

I: Do you think that women in this community are suffering from Micronutrient deficiencies like anemia, night blindness, goiter and others?

P: Now…..goiter is not common but limited. So, we have some in elders who are not the beneficiaries of the strategy in the previous time. However, goiter is not seen in adolescent girls from 10-19 years of age especially after the start of using iodized salt. Currently, all the public is using iodized salt since it prevents goiter and they not using the other salt (non-iodized salt). Therefore, it is almost reduced currently.

I: What do you think are the reasons for the deficiencies?

P: It is due to lack of proper nutrition. The farmers are not eating balanced diet because of lack of initiation to cook nutritious foods though they have the foods. The other is since they are too busy with routine activities, they will be tired to cook balanced diets plus their negligence. Thus, they suffer from these problems.

I: Are there non-communicable diseases (like diabetes, blood pressure) among women in this community?

P: Yes, there are like diabetes

I: Do you think diabetes could be associated with their nutritional status?

P: Yes, they are associated with nutrition. Second, diabetes is caused by eating high sugary foods. For example, if you add high amount of sugar in coffee, it will be changed to diabetes. Thus, high sugar is changed to diabetes.

I: Why women/girls in this community would not increase their height proportional to their age?

P: This is nature. It is nature for being tall or short and not caused by food (nutrition problem). For example, if I am short, I will have a short child and if I am tall, I will have tall child that looks like me. Thus, it is by race/genetic, not food. By consuming good (nutritious) food, you will not be tall. However, you will have good mind by consuming nutritious food. Thus, height is not resulted from consuming food.

I: Do women/girls in this community increase their weight proportional to their age?

P: Lack of having proper weight to their age is caused by poor nutrition. Those who eat proper food have good weight while those who have poor feeding will not increase their weight. Thus, this is associated with food and is caused by lack of proper nutrition.

I: Do women in this community suffer from overweight?

P: We don’t have

I: Is there a situation when women suffer from shortage of food?

P: Yes

I: In what situation do you think this happened?

P: Shortage of food is happened from August to September. It is caused when there is drought and shortage of rain.

I: How frequent does it happen?

P: There is shortage of food and we persons who suffer from but I don’t the number how frequent it is.

I: Okay, for example how frequent miss breakfast, or launch or dinner or how frequent eat only breakfast and dinner? In how many days?

P: Woa! Do you want to tell you in figure?

I: I mean, how they frequently miss breakfast or launch/dinner?

P: From the whole kebelle about 50 households may suffer from shortage of food.

I: Why?

P: The reason why they suffer from shortage of foods is poverty.

I: What is the cause of poverty?

P: From own weakness. Not working day and night tirelessly. Thus, they faced shortage of food. However, if they work they change.

**Section 2: Barriers to access and utilization of nutrition services**

I: What kinds of nutrition interventions are in place to improve health of the pregnant in this woreda?

P: Awareness is given by women development army. There is also called “Mitin” that is prepared by women association and is given to a woman by paying 20 Ethiopian Birr. This Mitin is balanced diet and supports to improve their status.

I: What any other intervention?

P: Vaccination, advice to eat/consuming diversified foods (orange and other plastic juice, soup made of red Taff, drink milk) and advice to take additional food.

I: What else?

P: To have cleanliness, have clean latrine, housing/environmental sanitation, drink safe water, separate compound for men and animals, and advice on water treatment chemicals, boiling of water and/or making cooled the water. Health extension workers moves house to house every two weeks to give advice and awareness to prevent diseases. In addition, women development army follow up the mothers every 3 or 4 days.

I: What is the importance of having clean water, hygiene and sanitation?

P: Previously, since there was no clean/safe water, worms were reproduced in the stomach so that the number of people who died (especially children) before their age are not easy but now women are drinking clean water since it is said drink clean water. Thus, clean water help us to reduce this and our farmers are benefited from this.

I: Would you tell me its importance?

P: Its importance to create awareness since our farmers didn’t know and to win poverty. So, it is needed.

I: Okay. What else? What other interventions?

P: To use and get advice on insecticide treated bed nets to prevent malaria. Mother and children should have to sleep under insecticide treated bed nets. In the previous time there was malaria but now it reduced and still there is malaria. Mosquito come from the rivers and wet lands and bite the farmers. Though most of our farmers taste the benefits/uses of insecticide treated bet nets (ITNs), there are few who are negligent to use (ITNs) so that they will be bitten by mosquito. Thus, in the last 4 or 5 years malaria is almost inexistent after the start of using (ITNs). In addition, every farmer in each development army is involved in filling stagnant waters with soils (draining stagnant waters) and removing/avoiding leafs to prevent mosquito breeding during start of September.

I: Where do they get it? Who provide it? Or who advise them?

P: Here, in the health post/health facility and we get the service by health extension worker. Woreda also gives directions to Kebelles and Kebelles by in turn teaches the farmers.

I: Are women/adolescent girls getting advice on nutrition sensitive agriculture such as home gardening?

P: Yes, they are getting advice on home gardening to produce vegetable lattice, salad, beetroot, orange and carrot (carrot is good for prevention of night blindness). Beetroot and carrot are also important for anemia and to replace blood lost during delivery.

I: What about on the need to be involved in safety net programs?

P: No need to get involved in safety net program.

I: Why?

P: This is begging from the government and is making bad your life. So, it is not good for your life but it may help for temporal needs for those who have shortage of foods. Or it may help to those who are sick/weak and elders. Therefore, for those who are healthy individuals who have good mind, safety net is not necessary. It is not a lifelong.

I: Are Women/Girls getting deworming services?

P: Yes, they are getting and given tablets.

I: Is there a situation in this community how you think that women need to be addressed through Targeted supplementary feeding (TSF) for women?

P: No. there is no. In the previous times (in 2012/13 G.C) there was corn soya support but not now. It is not coming at this time.

I: Why?

P: I don’t know. I don’t know the reason why about that

I: Do women advised to visit health facilities for checkup and services during pregnancy?

P: Yes. First the mother is vaccinated to herself during pregnancy five times but there is no vaccination after delivery.

I: What would it help to them?

P: To grow healthy and prevent risks. For pregnant women it helps to check the position of the fetus/baby and to herself to prevent diseases and risks. Similarly, it helps the pregnant women to check her health status if in case she bloods more during delivery and thereby to prevent complications.

I: Do you think that women receive advice on the need to get extra meal during pregnancy and lactation?? How?

P: Yes, they get advice and to have extra rest. However, without taking adequate test time if you start working, you will not look and your body becomes emaciated.

I: How much time?

P: Now, we give her time to rest. She has about 80 days (3 months) to rest.

I: How do you think that rest would help for?

P: For her?

I: Yes

P: It help her

I: How?

P: Taking rest will help her to return her affected body

I: Is the rest important for both pregnant and lactating women?

P: Pregnant women have 6 months rest period during pregnancy and 3 months after delivery/pregnancy.

I: What about advice in extra meal?

P: They should take extra meal to themselves and to grow the baby inside her womb. In addition, the lactating women should also take extra meal because if she didn’t take extra meal her back will become open and enters air and then she get sick and causes disease to her. Therefore, lactating woman requires the best food to prevent not to enter air in to her back.

I: Are women getting advice for the need to use iodized salt?

P: Yes, they are advised.

I: Why they are advised?

P: Not to fall at risk.

I: What kind of risk?

P: To prevent goiter.

I: Which of the interventions listed above do you think is most important for Women/Girls?

P: All are important. There is nothing that I can excluded as useless. Therefore, all are important to our farmers and our women.

I: But that you say the most important?

P: The most important is balanced diet such as vegetables and Mitin. Thus, balanced diet is the most important for women

I: What else?

P: Cleanliness of the house (housing sanitation), drinking safe/clean water, latrine utilization.

I: What else?

P: Using insecticide treated bed nets

I: What else?

P: To have regular medical checkups/examination and treatment.

I: What are the barriers in the implementation of these nutrition services?

P: At this time there are no barriers. However, people may not understand the importance and the other is negligence.

I: What else?

P: If there is poverty caused by own weakness and not working tirelessly.

I: What else?

P: Not applying what they have learnt due to negligence.

I: What about the barriers from service providers?

P: Nothing. We don’t have problem and there is no problem in giving the service.

**Section 3: Perceived needs of women for relevant services during pregnancy**

I: What special things do pregnant women, lactating women and Adolescent need in your community?

P: Which one? Those from 10-19 years of age.

I: Yes.

P: They want to be organized in association to work and benefited. Thus, if they get the opportunity, they want to be organized and develop to reach at the highest level. Now, these women need these things. The government also expands the work. For example, those persons living in areas where there is grass lands, they are organized in associations and the government gave them cattle as loan so that the persons will be benefited by producing milks and reproducing the cattle.

I: What special things do women and adolescent girls need in your community. For example, do they need to visit health facility, taking extra meal, taking rest and taking supplements?

P: Yes, they do. The women/adolescent do need to visit health facility, taking extra meal, taking rest and taking supplements. For example, they took vitamin A to prevent night blindness.

I: What else?

P: They do also need taking contraceptives to have birth spacing and taking balanced diets.

I: What should be the role of a husband to improve nutrition for lactating women?

P: The husband?

I: Yes.

P: He provides her food and other supplies. He works and provides her the supplies.

I: Do women in this community typically change their diets when they are pregnant?

P: Yes, they change. There are women who change up to 3 months. They change and hate Enjera (local food) while others hate coffee. Actually, it depends on your interest.

I: How is this diet different from when they are not pregnant?

P: It varies.

I: Why?

P: Before pregnancy, they didn’t hate foods and eat whatsoever they want. However, during pregnancy, they hate their own foods. For example, a pregnant women hates food prepared in her home and become more interested in food prepared in others home.

I: Do they eat more or less food?

P: There are women who eat more food and there are also women who eat less food. So, it depends. Some may eat liquid foods. For example, if the woman hates Enjera, they will eat Macaroni, Mitin, Carrot, Orange and Banana. The woman may eat enjera with shiro if she is okay. On the other hand, she may hate enjera with meat. Thus, the amount of food varies and depends on your nature.

I: What foods are recommended for women/Girls?

P: They are advised to take liquid foods and balanced diets as they are advised to eat foods that benefit both the mother and the child. Girls are also advised to eat 3 times a day.

I: What foods do women/Girls avoid?

P: There is nothing that women need to avoid especially girls. However, there is perception that girls not to drink hot drinks like tea.

I: Why?

P: Not to grow fast or not to get matured soon (laughing and smiling). Thus, in this community the girl should not drink tea if the girl is young.

I: What else?

P: Nothing except that.

I: You don’t have something to add?

P: The other is Liquor (Areqi) is not allowed to pregnant women. However, it is also believed that drinking liquor may make the fetus to have bold eyes.

I: What else?

P: Pregnant women are also prohibited not to eat hot peppers since the hot pepper affects the fetus in the womb.

I: What affects women’s diet during pregnancy?

P: What we can say?

I: What factors affect pregnant women’s diet?

P: Nothing

I: Are there gender disparities in women’s diets before pregnancy and during pregnancy?

P: Nothing

**Section 4: Other interventions that improve pregnant, lactating and adolescent nutrition**

I: Have you ever gone for nutrition screening?

P: Yes, I went to weigh my weight during pregnancy and delivery.

I: Where?

P: Hospital/health facility/health institution

I: Who provide you?

P: Health worker

I: Have you ever gone for nutrition screening during community health days?

P: Yes, we went to weigh our weight to know our status.

I: Do you think community health days would have benefits for the women?

P: Yes

I: How?

P: To know her status and then act accordingly. For example, a woman may know her weight and then she may be advised and she may learn what to do to have normal weight.

I: Have you ever gone during routine service delivery?

P: We didn’t go because we don’t waste our time going to health facility

I: Why?

P: Since we are busy with routine activity and not to make pending the activities. As we are rural, no one went except we are sick.

I: What are the challenges to attending community health days?

P: You know why the farmers are not attending the community health days while they are told by the health workers to come and attend for examination or advice? Since the farmers are not interested to make pending their work. They also consider going to community health days as wasting of time and obstacle to their work. They understand as they miss their activity. They don’t analyze the advantages and disadvantages of attending the community health days. Thus, negligence and lack of awareness are the causes for the problem.

I: What about to accessing the routine service delivery?

P: The same. It is lack of awareness and negligence.

I: Do you think women needs to be targets for supplementary foods?

P: Yes

I: Why?

P: Not to get affected since women are working in very hard work so that not to fall at risk. Therefore, as the women are working day and night, they should be targets for supplementary foods not to get affected.

I: Are women beneficiaries of the soft conditionality of the productive safety net program (PSNP)?

P: Yes

I: How?

P: A pregnant women should not work after 6 months of pregnancy and will be out of work till the child make one year while getting all the benefits but to do this the pregnant women should first bring medical evidence from the health facility/institution and then she will be allowed to have rest while she is getting all the necessary supports. Thus, she will receive all the supports for 12 months after delivery.

I: Do women in this community know why they are targets of the program?

P: Yes, they know.

I: How do they know they are targets?

P: They know. They said the government targets us by safety net program. However, women may not get support if they are rich or have wealth.

**Section 5: Understanding perceptions of age at first birth and birth spacing**

I: Do you think delaying the age at first birth to after 18 is better for the health of the women?

P: Yes

I: How?

P: At age 18 or before, the girl is not strong. Thus, better to think to have a child from 20-24 years of age and at this age she will not suffer from the risks.

I: What other benefits does it have for the women?

P: Nothing and that one is the benefit.

I: What about for the baby?

P: The baby is not also affected

I: How?

P: For example, if the girl become pregnant while she is 18 or before, the baby will be affected since the women is not physically strong.

I: Does this delay would have a benefit to the nutritional status of the women?

P: Yes

I: How?

P: Because it make her increases her weight and energy.

I: Do you think this message is being promoted in the community?

P: Yes, it is promoted

I: Who are working on it?

P: From higher to lower level up to networks by health workers and administrative.

I: How do they promote?

P: First, the health workers train the core persons such as women development army and then they teach them to promote the message to the public. The chairman also assists the cluster to teach his/her network. Thus, in this way they promote the message in each kebelle and village.

I: In your opinion, what does the reaction of the community looks like to the promotion?

P: It is good.

I: Why?

P: One it help us grow our child in good condition. Second, it helps the farmers/women not to suffer a lot as they required to have balanced diet and extra rest. They also advised us to be cleanliness.

I: In your opinion, how could this message be better promoted?

P: Previously women development army (network) was given training by the government but now there is no training. As a result, we have a fear not things become fragmented. For example, women development army in health were very effective since the women development army worked in promoting to have medical examination, institutional delivery (and making home delivery zero) and others activities and these army makes home delivery zero at the beginning because the army believe pregnant women may suffer if they gave birth at home. However, these development army are not working as in the previous time. Thus, to better promote the message training should be given to women devilment ary.

I: Who should be involved?

P: Women development army should be involved.

I: Who else?

P: Starting from the higher body up to the lower level and the network should get involved.

I: Can you think of any other opportunities for promoting birth spacing?

P: There is family planning service such as injections for 5 years, 3 years and 2 years.

I: How many years do you think the gap should be between successive births for women?

P: 4 years

I: Why?

P: Because if it is 2 years, the child will not be strong and the fetus in the womb will also be affected because both of them will not get balanced diet and grow strong.

I: What about if shorter than it?

P: 3 years

I: Why?

P: Because 3 years also good because they child become old and play outside and not be affected. However, the best is 4 years.

I: From who have you ever heard?

P: From health institution. The health works even said to have birth spacing up to 6 years and advised us to have only 3 children because we can care these 3 children and make them grow in good condition. However, our public didn’t accept and need this because having 3 children are not enough to give care to their parents. For example, the public said if 3 of them left for education or work, who will care me and because of this attitude the public need to have more children. For this reason, the public said 4 years is normal and I also heard 4 years birth spacing from the public. However, the health workers advised us to use contraceptives for birth spacing so that to have 3 children only.

I: For the last time?

P: I heard it since but still I heard it recently.

I: Who are involving on it?

P: Health workers, women affairs, religious leaders, model farmers and women development army.

I: What do suggest promoting it in a better way?

P: Women development army should be trained fully.

I: Why?

P: Why? To teach the public at lower level that is at the village level. It is women development army that make reach home delivery zero at each Kebelle. Therefore, the government should train women development army to make reach the lesson to all public because it is the women development army that disseminates the information.

I: Which population in this community is not addressed with the promotion?

P: There is no one who don’t get lessons.

I: Can you think of any other opportunities to prevent early marriage? How?

P: Public awareness is the opportunity, support from the religious fathers, presence of women affairs and follow up by women affairs (such as Timret) from higher to lower level to prevent early marriage is an opportunity and thereby to make the girls continue their education (i.e. not to stop their education).

I: What else?

P: Monitoring and evaluation about this issue by woreda administration is also an advantage.

**Section 6: Understanding communication and information sources**

I: Is there an opportunity in the community to discuss Nutrition for women?

P: Yes, there is.

I: What are the opportunities?

P: There is opportunity for women alone to get lessons in their networks every two weeks. Thus, they get advice/lesson to eat balanced diet such as liquid diets/foods. For example, if the women is lactating mother, she is advised to take meat and milk/fluids that can produce/generate and give more milk. Similarly, the pregnant women is also advised to eat balanced diet and checkup every month.

I: What are the common sources for nutrition during pregnancy?

P: Health institution. For example, there are women who work in association that prepared Mitin. Thus, women can get this food from health institution. The sources of nutrition during pregnancy are also vegetables (such as salad, lettuce, beetroot) and fluids that are obtained from market and/or produced from the agriculture.

I: What messages?

P: Advises about balanced diets, and examination and treatment. Thus, the lessons are obtained from health institution. Lesson such as women should take fluid diets. Here, women development army also involve in teaching to make aware the women. Health workers also revisit the households.

I: Do all women get the messages easily?

P: Yes, the get the message.

I: What are the barriers for access to information for nutrition during pregnancy?

P: Nothing but their own negligence due to lack of education and not accepting and taking the lesson as it is important.

I: What else?

P: Nothing else. That is it.

I: Which information is effective for you to change your practice in nutrition during pregnancy?

P: Advise from the health institution by health workers. Since I was women development army, I was trained by health workers and advised me to take balanced diet and I started eating.

I: Why?

P: Because the information is obtained from health workers/experts and these experts are trained by experts. Thus, the information is effective and helps the public not to get affected.

I: Which source of information about nutrition is essential for you?

P: All information given and believed by the government is essential. About feeding/nutrition, the information obtained from health workers/health institutions is essential. The next is from agriculture. The information obtained from our children who have diploma or degrees is also essential.

**Section 7: Additional remarks**

I: Any additional remarks that you want to add?

P: Training should be given to women development army about nutrition since it helps to refresh the knowledge and thereby to change the public.

I: What else?

P: Nothing except on that because they get lessons about agriculture. Thus, no need training on agriculture.

I: Thank you very much. I have finished.

P: Welcome.

**SUMMARY**

**Section 1: Common maternal (PW, LW and adolescent girls) nutrition problems in the community**

To stay healthy women/girls should eat balanced diet, keep clean their body, environment and latrine, use insecticide treated bed nets and should be vaccinated. Goiter, anemia, underweight, shortage of food and diabetes are common nutrition problems in the community.

**Section 2: Barriers to access and utilization of nutrition services**

Intervention such as advice to visit health facility, use ITNs, advice on water, hygiene and sanitation, deworming service, advice on use of iodized salt, advice on diversified foods, advice on extra meal, and advice on nutrition sensitive agriculture are in place. However, lack of understanding about the importance of the interventions, negligence and poverty are the barriers in the implementation.

**Section 3: Perceived needs of women for relevant services during pregnancy**

Pregnant women need to visit health facility, take extra meals, have rest and take supplements during pregnancy period. On the other hand, they are also interested to work in organized way such as in associations.

**Section 4: Other interventions that improve pregnant, lactating and adolescent nutrition**

The participant has gone for nutrition screening to weigh her weight during pregnancy and delivery. She also went for nutrition screening during community health days but not during routine service delivery. However, there are challenges that make her not to attend community health days. These challenges are being busy and not to make pending her work, not knowing the advantage and disadvantages of attending community health days, negligence and lack of awareness. On the other hand, being busy with routine activity and feeling healthy are the challenges not to go to health facility to obtain routine service delivery.

**Section 5: Understanding perceptions of age at first birth and birth spacing**

Delaying the age at first birth to after 18 is better for the health of the woman and the baby since the mother will not suffer and fall in risks. It also helps her to get balanced diet and thereby she will become physically strong. The maximum and the shorter birth spacing should also be 4 years and 3 years respectively. Awareness of the public, presence of religious fathers, presence of women affairs and follow up by Timret (parts of women affairs) are the opportunities to prevent early marriage.

**Section 6: Understanding communication and information sources**

Information obtained about balanced diet and medical examinations from health institution or health workers is most essential and effecitve. Other source of information are women development army, agriculture and won children who have diplomas and degrees.

**Section 7: Additional remarks**

Training should be given to women development army on nutrition.

THE END

REGARDS,

YASIN JEMAL
