# Supplementary material for: Barriers to utilize nutrition interventions among lactating women in rural communities of Tigray, northern Ethiopia: An exploratory study
Source: PLoS One. 2021 Apr 30;16(4):e0250696. doi: 10.1371/journal.pone.0250696 (PMC8087028; doi:10.1371/journal.pone.0250696)
Supplement: S2 File — (ZIP) [file pone.0250696.s002.zip › S2_File.Doc/Community level Key informants/119_IDI_Kebele Youth office_Selekleka kebele_Medebay Zana woreda.docx]

**In-depth interview guide for with experts** using the Guide for experts (**Tool A**)

**Introduction:**

Hello my name is Haftay. I am from Mekelle University. Thank you for taking time to speak with us today. We are doing a research on factors that influence the nutrition of mothers and adolescent girls in collaboration with the regional health bureau and UNICEF. Your participation is very valuable. The things that you tell us will be used to improve nutrition programs and services for women and adolescent in the region and in the country. Your names will not share when we report our results.

However, I will record the discussion using and audio tape recorder so that we can capture all the ideas that are shared. I have several questions to ask you that we have prepared in advance, and we will ask you to say what you think about each question. To ensure the privacy of everyone here, we ask you not to repeat what to discuss outside of this group. The discussion will last for 1-2 hours. Do you have any question before I begin? If you think of any question as we proceed, please feel free to let me know. If it is all right with all of you, I will run on the tape record now.

**Section A: Interview details**

1. **Zone**: North West
2. **Woreda**: Medebay Zana
3. **Kebele**: Selekleka
4. **Name of key Informant**: Merhawi Hagos
5. **Institution of key informant**: Selekleka Youth affairs
6. **Interviewer’s name**: Haftay Berhane
7. **Date of interview**: 18/11/2017
8. **Interview starting time**: 10:00 AM
9. **Interview end time**: 11:26 AM

**Section B: interviewee professional information**

1. **Gender**: Male
2. **Age**: 30 years
3. **Highest level of completed education:** High school
4. **Current job/Position:** Head, youth affairs
5. **How long have you been in the current Job/position:** 01 years

**Key:**

**I**: Interview

**P**: Participant

**Section 1: Common maternal (pregnant and lactating women), and adolescent girls` nutrition problems in the community.**

**I**: What do women including pregnant and lactating, and adolescent girls do to stay healthy?

**P**: In this area, there are women leaders that controls 30 households organized by the health center or there are also volunteers. There is network based teaching on nutrition, family planning and general health of a mother or an adolescent girl. There are also health extension workers in every kebele and they provide education by home to home visit. Therefore, there is good progress in this regard.

**I**: What do a pregnant mother do to be healthy?

**P**: A pregnant mother first keeps her hygiene to stay healthy. Keeping hygiene is very important because most diseases are caused by lack of sanitation and hygiene. As I have told you, the education is there in each kebele including education on environmental sanitation. There is education on environmental and home hygiene, eating balanced diet by the health personnel. I have been involved voluntarily in the health education. A mother should prevent a disease by the counselling provided to her.

**I**: What about a lactating mother? What does a lactating woman do to keep herself healthy?

**P**: She should visit health facility for follow up which we call it antenatal and postnatal care. A lactating mother performs what she has been counselled and advised by the health professionals. I have known many lactating doing according to the advice.

**I**: What about an adolescent girl? What does an adolescent do to stay healthy?

**P**: There are recently introduced organizations like peer to peer that deals who should be called an adolescent and what an adolescent should do. These are found in every kebeles. In order an adolescent girl to be healthy, there is a counselling provided by the peer to peer groups to the adolescent girls. However, I have not seen the adolescent girls doing it. It is difficult for me to talk about this because I do not have enough knowledge about what the adolescent girls are doing.

**I**: What is the peer to peer group doing? What does it deal with?

**P**: This peer to peer is focusing on health like HIV, reproductive health, issues related to tuberculosis, malaria, labour and delivery services. Anyways, it encompasses education on all health-related services in outside the health system.

**I**: What does it do with labour and delivery?

**P**: It indicates the services given to a mother who gave birth.

**I**: When we are talking about adolescent girls, we have agreed that the age is 10-19 years. who are eligible to be served by the peer to peer group?

**P**: The service is given to children and older adolescents. But the service is different for the two. You cannot educate children on HIV and others like labour and delivery however, you will educate those adolescent girls on HIV and tuberculosis and their transmissions.

**I**: Is there a learning session about nutrition in the peer to peer group?

**P**: Yes. There is session on nutrition. An individual should improve his/her nutrition to live and work. Nutrition is eating from different types of food like vegetables, cereals, and others.

**I**: What are the common nutrition related problems seen in this area?

**P**: If you do not eat balanced diet, your development will not be improved. However, I did not observe this condition in our area. But there are problems which comes from not keeping the sanitation and hygiene even if the food contains necessary nutrients. I do not know individuals who have problem of development in our setting.

**I**: Are there individuals who sick because of shortage of food or individuals who are supported with food like fafa?

**P**: Yes. There are studies done. As to my knowledge, all under five-year children are studied. All are also screened using weight. If a child is found to be with a problem of under nutrition, the child will be supported with food. I know there are children that are under this support. I may not remember the exact number but I know there are children who are supported.

**I**: What about those are sick and admitted to hospital because of food shortage? If there are individuals who are admitted to hospital and taking plumpy net?

**P**: Yes. There few children like those whose parents are died because of HIV/AIDS. They have strict follow ups. If a child is having food shortage, plumpy net may be given. There are two plumpy nets if I am mistaken. Those are plumpy sup and plumpy net. There are also supports from social affairs, world vision and other NGOs.

**I**: Do you think that women/girls in this community are suffering from nutritional problems like being thin or admitted because of shortage of food?

**P**: There is nobody who is admitted because of shortage of food in our kebele. I do not know individuals who are sick and admitted to health facilities from shortage of food [laughing]. I do not even think this will happen in our area.

**I**: You were telling me that there are individuals who are screened and supported with fafa. Are there pregnant or lactating women or adolescent girls who are given fafa because they are underweight?

**P**: Previously yes it was there. But, currently there is no such aids except for children.

**I**: In your opinion, how do you think the shortage of food will affect women?

**P**: If there is shortage of food, you will be exposed to different problems. You will be at risk of different diseases. This food shortage is mostly seen in patients with HIV/AIDS. This because you will see them being ill. This could be due to their thinking and adherence. But, it is mostly seen in this kind of patients. When you see them sleeping, you will conclude as it is because of shortage of food.

**I**: Do you think that nutrition related problems like anemia, night blindness and goiter are common in this community?

**P**: Previously, it was there but there are no individuals who are suffering from this. I did not see anybody suffering from these diseases. However, there are elders who have goiter and some of them have been removed it by surgery. Either because of good utilization of iodine or due to eating balanced diet, this goiter is not seen in the young generation.

**I**: What about anemia?

**P**: Yes. Anemia is there. There is also a tablet given in health center for treatment of anemia. If a pregnant mother is having anemia, there are tablets given for the treatment. There is also counselling to prevent the occurrence of this anemia. But women with anemia are given follow up services. Night blindness id mostly seen in elders. There is no eye problem in the young generation. There are some students who lost their eye starting from their childhood. But there is nothing seen in the current times about night blindness.

**I**: What do you think are the reasons for the deficiencies or diseases like anemia, night blindness and goiter?

**P**: Anemia is caused by not taking balanced diet. It also caused by nature. Whereas, goiter is caused by problem of nutrition. It is caused by consumption of salt without iodine. The eye problem is similarly caused by lack of hygiene like that of trachoma.

**I**: What do you think should someone take to prevent from the occurrence of anemia?

**P**: You should have enough knowledge about the transmission and prevention of anemia. I may not describe it well but, it happens on our day today activities. The symptoms of anemia are commonly known like vertigo, blurred vision, and other symptoms. The prevention of anemia include the food we take should include red teff in the form of soup and we should also apply preventive methods advised from the health professionals.

**I**: Are the diet related non-communicable diseases common like hypertension, diabetes mellitus, cancer etc in this community?

**P**: I know individuals with hypertension and diabetes mellitus. There is also a cancer seen in females. Therefore, three of them are seen in our community.

**I**: Do you think there could be any association between nutrition and occurrence of these non-communicable diseases?

**P**: If I am not mistaken, diabetes is caused by higher production of insulin in the body. The prevention method therefore is limiting foods that can facilitate the production of insulin like foods that contain sugar. In case of hypertension, limiting drinking of unfiltered local beer (Guush siwa). The main cause of hypertension is narrowing the blood vessels. If the blood vessel is narrowed, the blood will not flow as it should be. To treat the hypertension, the first thing is proper nutrition and physical exercise. When I say proper nutrition, it is to indicate that drinks like unfiltered local beers should not be taken. The other is limiting the rich peoples` foods that contain high fat content (Laughing). The crops have also different impacts. If we take red and white teff, they have different impacts on hypertension. The red will increase the risk of hypertension and we should eat white teff and crops like sorghum.

**I**: Is it common to see individuals in this community that have increased weight proportional to their height?

**P**: I think you want to indicate the BMI. There are overweight individuals. There are individuals who short but fat.

**I**: Do you Do women/girls in this community suffer from overweight?

**P**: It is seen in some individuals. There are young girls who are very fat. Therefore, it seen in women.

**I**: How could we relate this overweight with nutrition?

**P**: I think we should correct our feeding style. This can also be caused by stress. If there is stress, there could be unwanted overweight and it could be caused by overnutrition as well as sedentary life style.

**I**: You have told me that there are short girls with fat body weight. Do women/girls in this community suffer from not increasing their height proportional to their age?

**P**: There are no individuals suffered from this. The individuals I told you are not such very short. They are with intermediate height. I want to indicate that when compared to their weight, they height is somewhat short but not significant.

**I**: Do you think farmers of this community harvest sufficient foods that can be enough for one year?

**P**: In fact, this information could be better addressed by farmers but from my observation, farmers may harvest that can be sufficient for one up to two years. However, there may be fast farmers who harvest for three years and lazy farmers who may harvest for less than one year. It depends on the farmer`s activity.

**I**: Is there a situation when the community suffers from drought?

**P**: I do not think this area is affected by drought. There may be natural drought but, till now, there is nothing. The current situation seems good that can resist any drought. There are poor individuals. But I am comparing drought with situations that has been occurred in the past centuries like that of 1985. Otherwise, we have weak people like elders. There are also young girls who do not have support.

**I**: Do you think women/girls suffer from food shortage?

**P**: The poor girls are leading a temporary life by working in private houses or other area. They are benefited temporarily but this is not continuous. The percent of these women who are suffered from food shortage may be difficult to estimate as they are currently working under the control of rich people. But the real scenario is different. There are also women who are getting support from the safety net program. If the safety net is stopped, I could not guest what their fate will be.

**I**: For which one of the above problems like being thin, overweight, anemia, goiter, do you think pregnant, lactating women and adolescents are especially at risk?

**P**: Lactating woman is mostly affected by anemia. Most lactating women are using tablets for prevention of anemia when they are getting postnatal care. Therefore, lactating women are mostly affected by anemia. Goiter, overweight and other are not commonly seen in pregnant women. It may be seen in few but it is not common.

**I**: What about pregnant women?

**P**: Anemia is common in pregnant women, too.

**I**: What about adolescents?

**P**: We have heard that the adolescents are given iron supplements and therefore, anemia could be the problem that could happen in adolescent girls.

**Section 2: Nutrition priorities in the wereda**

**I**: As youths’ affairs office, what interventions has been done by your institution in relation to adolescent nutrition?

**P**: Education is given to adolescents in the form of training and counselling. We have two health extension workers the same as that of rural health extension workers. Their job is counselling about health. They give advice about nutrition including in schools. The education is being provided and the awareness is also good.

**I**: Do you think there are interventions done related to nutrition or others by your institution on the adolescent girls?

**P**: Our institution is at kebele level. There is one health extension worker and she reports the activities done on adolescents, adults and women related to nutrition and education. There are interventions on adolescents but I did not have the report at hand. Most of the volunteers are females. There are more than fifty-six volunteers in our town. They are facilitators and are linked in networks. Therefore, all are adolescent girls.

**I**: Who is organizing the volunteerism?

**P**: The volunteer organization is controlled by the kebele and led by the health extension workers. As youth affairs, there are no activities done in this regard. Last time, a training was provided to 25 adolescents by zone on nutrition. These adolescents are given training to their peers on coffee ceremony on nutrition, reproductive health and others.

**I**: Who was trained the twenty-five adolescents?

**P**: The trainers were from zone and it was basically on reproductive health. Fortunately, I was part of the training. It was recent focusing on the peer to peer training.

**I**: What are the activities done by the volunteers?

**P**: The volunteers are working without payment. The second is they are engaged on activities related to the sixteen components of health package. They identify a pregnant woman from which network she is and what important services she needs.

**I**: How is your working with the volunteers?

**P**: The volunteer is led by health extension workers as I have tried to mention it above. Our institution is supporting to the health extension workers. We arrange a conducive environment for the health worker for education and other services in every kebele. These health extension workers are working in the kebele and led the volunteer group.

**I**: What are the supports from your office given to the health extension workers?

**P**: The supports to the health extension workers include training, recognition and award to those who perform better such as continuing education and so on.

**I**: Do you think it is necessary for your institution to get involved in work aimed at improving nutrition among adolescent girls?

**P**: Off course yes. This is because, the adolescents will be encouraged as the government is doing in job creation and quality improvements. If volunteerism is encouraged as the government is giving attention to adolescents, I believe, there will be remarkable achievements.

**I**: As youth affairs, what should be fulfilled so that your institution will be engaged in improving the adolescent girl`s nutrition?

**P**: There should be a special hall dedicated to youths. This hall should be the area where youths can meet each other to talk about their issues. This is like school and other sectors. There should be an area dedicated for youth which includes area for training with all training materials.

**Section 3: Nutrition interventions that improve adolescent and maternal health**

**I**: What kinds of nutrition interventions are in place to improve health of the adolescents by your institution?

**P**: There is nothing done by the youth affairs office for the adolescent girls. The successful interventions are in loan and support. Otherwise there are nothing done for the adolescent girls.

**I**: You have been mentioned that there was a training for adolescents given by people from zone and you have been participated in the training. How was the training?

**P**: The training was interesting. The objective of the training was to work with the community practically. If the training is applied, it will bring a real change. There may be some gaps but if you are committed to start it, you will start from your home. You will start to teach about nutrition and reproductive health at home then distribute it to friends, and the community at large. If it is applied, I think it will bring a change.

**I**: What was the mission given to the trainees including you after the training?

**P**: We are trained and there is a community without the training. It was to train the untrained youth. We have grasped a lot of knowledge which we were not aware of and the objective is to train the untrained ones. We are doing on realizing our plan but we are still lagging.

**I**: What are the obstacles that hinder you not to go further? Why are you lagging?

**P**: Most of the youngster do not have constant job. If you are volunteer, you will work without any payment. You must have also money to teach others which are outside. You will not live without money and you will be busy to earn this money. There are also students and there will be discontinuation because these students will go to school. There are also many meetings. Therefore, these are the obstacles. To work continuously, a plan is prepared and the plan is documented in our kebele. Therefore, we will do based on the plan despite the obstacles.

**I**: Do pregnant women advised to visit HFs for checkup and services?

**P**: Yes. Every pregnant is known by each developmental army and the volunteers assigned. It is also known which pregnant is in which volunteer and how many pregnant women are there in a volunteer. There is a control mechanism and audit where how many pregnant women are visiting the health facility from a given volunteer. Pregnant women are known by the volunteers and there is follow up. There is counselling for pregnant women provided by the volunteers.

**I**: What do you think would it help to the pregnant women?

**P**: Since, the volunteers are part of the community, they are very familiar with the community. Mothers can give all information they faced to the volunteers without being shy. They can share all internal mysteries without restriction. The volunteers will be also tries their best to help the women in any aspect. The other thing is, the volunteers will help mothers who are pregnant and have chronic illnesses. They may help the mothers in bringing things like drugs and supporting many other things.

**I**: Do you think that pregnant women receive advice on the need to get extra meal?

**P**: Yes, they do.

**I**: How much times per day should they get?

**P**: If a mother takes meal two to three times per day, she will be advised to take four or five times a day when she get pregnant. The support may not be given but they are advised to take extra meal. They are advised to take meal at the evening, dinner, breakfast and lunch.

**I**: How do the pregnant women accepted this advice?

**P**: Most of our community is having intermediate income; not poor or rich. But it may depend on the condition of the pregnant mother. Some pregnant mothers tend to eat more than others. These mothers accept the advice very well. There are also pregnant women who are anorexic which eat only one or two times per day and these pregnant women are getting difficulty in taking as per the recommendation. But generally, they are accepting it.

**I**: What about lactating mothers?

**P**: It is similar with the pregnant women. They are advised to take extra meal to build their body and help the child for milk production. They are advised to take meal, even if I may not know the exact times they should take. They are given advice and support to take hot drinks in the form of soup.

**I**: You have been telling me that children are screened for nutritional status. Do women get screened for their nutritional status?

**P**: Pregnant measured not based on weight but based on the measurement at their hand (showing the mid arm to indicate MUAC). I have forgotten the BMI but they are followed based on this. They are therefore, getting screened.

**I**: What about adolescent girls?

**P**: Yes. They are similarly doing the same thing as that of pregnant women.

**I**: How can the adolescent girls addressed with such kind of interventions? It may be easy to advise pregnant women while getting the antenatal care follow up. Where do you think the adolescents get screened?

**P**: Do you mean for the pregnant adolescents?

**I**: No. adolescent girls that are not pregnant.

**P**: Adolescents are getting advice on the time of immunization for diseases like meningitis. But, I do not think there is a condition where these adolescents came to health facility for screening of nutritional status. I may not know it correctly. The pregnant women are given the advice while they are attending the follow up services but I do not have enough information on what happen to adolescent girls who are not pregnant.

**I**: Are pregnant women getting counseling for food diversification or for mixing of different foods in each dish during pregnancy?

**P**: Yes, they are. A model practice in health center is the mothers waiting room. When pregnant women came to the health center, she will learn how to prepare a diversified food. Previously, food was collected from each house hold that will be given for the pregnant women in health center. But currently, this is done at kebele level. Then, they are advised to teach the community what they have been taught. Many things have been done at each kebele towards this food diversification. There is education on this issue.

**I**: What are they advised to mix?

**P**: If we see the stew, instead of one, it is advised to prepare a mixture of pea, spinach, fruits and vegetables. The injera should be prepared by mixing different crops. These are some of the advises they are getting.

**I**: Which cereals do you think should be mixed to prepare injera?

**P**: Injera should not be prepared from white teff only. It should be a mixture of white teff, red teff, sorghum and maize. This what we know.

**I**: What do you think is the advantage of this food diversification?

**P**: The food diversification is eating balanced diet. You will get energy, it will also prevent you from disease, it also contains foods that builds the body. Taking from the mixture will help you to satisfy what an individual need to get from food that is energy, disease prevention and body building.

**I**: Are pregnant women getting advice for the need to use iodized salt?

**P**: Yes. Not only pregnant women but all the community are utilizing the iodized salt. Mothers are only using the bar of salt to mix it with pepper otherwise, they are using the iodized salt for stew. There is great achievement in utilization of iodine salt.

**I**: How do you think mothers in this kebele are using iodine salt? How are they adding to the stew?

**P**: Even if we do not know what is done in every home, as it is given as advice, iodine salt should be added after the stew is removed from heater. This is done to prevent evaporation of the content of the iodized salt. All individuals are utilization iodized salt but I am not sure with how they are utilizing.

**I**: How is the practice of growing home gardens in your area?

**P**: Vegetables in this area are growing in few farms. There are certain areas that produce vegetables for market purpose. If you see the river basin, it is given to youths to produce vegetables. You may have observed that the river basin here is cultivated with vegetables and fruits. Therefore, gardening is not done in every household.

**I**: Are famers getting advice on home gardening such as vegetables and fruits? Do they try to garden?

**P**: The urban agricultures are providing orientation on home gardening. They are advising to cultivate vegetables and fruits. The gardening is there but it is not enough. This gardening should be strictly followed unlike the situation happening currently. It should be considered as very important point to change the life of the farmers.

**I**: You were telling me that a pregnant woman should take vegetables and fruits. But, are there efforts done to produce these fruits and vegetables by herself at her home?

**P**: There is education about this but there is no practical application. There are limitations in applying it.

**I**: If a farmer is taught about home gardening, why is not applying it? What are the barriers for the implementation of these interventions?

**P**: The farmers are putting their elders as references. They say `our elders were using only one type of food but they were healthy enough`. This is may be related to lack of awareness. This could be the main barrier for lower implementation of home gardening.

**I**: What about things other barriers related to supply like water, fruits and vegetables for cultivation and plot of land?

**P**: In our wereda, there are many activities done in expanding irrigations. Budget is also allocated for expanding this irrigation. There are many ponds digging for water reservation or collection. But the farmers are not productive. There is a good support including sending experts to the area. This is not common in urban setting but the government is providing areas for cultivation of fruits and vegetables around the river basin here. But the community is doing what has been practiced culturally from previous times.

**I**: You were telling me about safety net program that there are individuals benefited from safety net program. Who are eligible for support from safety net?

**P**: The objective of safety net is to support those that can change themselves. The support is done by involving in active work. It is kind of linking to be self-sufficient. But this safety net is creating a kind of dependent mentality.

**I**: Are pregnant, lactating women and adolescent girls included in this safety net program?

**P**: Most of the time, those who are supported by the safety net program are those that are very weak. Those include individuals without support may be due to disease. Adolescents who are disabled or mentally ill are benefited from the safety net program.

**I**: What else interventions are there related to safety net program? It could be food aid or other activities?

**P**: Yes. There is capacity building package given by organizations like world vision who are capacitating in farming, poultry production and sheep and goat production. This is done in collaboration with governmental institutions like social affairs. Therefore, there are many individuals who are getting support from these institutions.

**I**: Are women getting advice on sanitation and hygiene services?

**P**: Yes. If we see the practically, students are applying it well. As we seen in some of the schools, they have a hand washing day. There are students who changed it to culture. They are believed to change the community with this practice. To be healthy, it is vital to keep environmental and personal hygiene. There is education on sanitation and hygiene but the practical application may be low. There is support and counselling sessions on sanitation and hygiene.

**I**: Is malaria common in this area?

**P**: Malaria is not common. There few cases but it is not exaggerated number. Since the government is doing good in preventing the malarial attack, you will not see people with malaria. In the previous times, there was many patients with malaria coming to health facilities but these days, there are only few patients complaining to have malaria.

**I**: What type of interventions have been done to prevent the malarial attack?

**P**: There was cleansing of the environment such as wells that can favor the multiplication of mosquitos. Other is distribution of bed nets and spraying insecticides that can kill mosquitoes. These are the preventive methods.

**I**: Do you think bed nets is distributed to all individuals?

**P**: Yes. Last time, there was a survey of households through the developmental army and all individuals have got insecticide treated bed nets. Bed net is supplied to each family especially in the town. There is improvement from time to time because of the follow up and support.

**I**: Well. Are pregnant women getting deworming services?

**P**: There many people who come from zone and federal level. Last year, there was provision of this service in schools and public meetings. It is given intermittently as a program.

**I**: Is there school feeding here

**P**: No. There is no

**I**: In your opinion, are adolescent girls linked to youth friendly services at health facilities?

**P**: The initiative is starting currently but there was no such services and the adolescent girls were not using these services. However, the service is initiated and I hope they will be benefited from the service from now onwards.

**I**: There is a separate room as outpatient department dedicated for services that are specific to youths in which there is education about HIV, reproductive health. How do you see these adolescent girls in using these services?

**P**: As I have tried to tell you, the twenty-five youths are doing this activity. There is a separate place in the health center dedicated for youths. There are all services like contraceptives methods for those users. These services were not present in the previous times.

**I**: Is the service already started?

**P**: Yes. It is already started.

**I**: Therefore, how do you see the service?

**P**: It is recently started service with plan. I think it is started before two weeks however the training was given before two months. This delay was related to lack of space in the health center. But, it is already solved. We have received the plan to start action. Therefore, we are ready to work for the next time.

**Section 4:** **Implementation challenges and** **Community factors affecting access to nutrition interventions**

**I**: What are the barriers that hinder women and girls not to use the above-mentioned intervention like youth friendly service, bed nets, deworming and health services?

**P**: There are no obstacles except those related to their own perception and awareness. It is dependent on their own opinions like that of shyness. I do not think there are other obstacles than the one I mentioned.

**I**: Do you think there will be a relationship between educational status of the women and access to the interventions?

**P**: Yes. But, I can observe in both in school and out school adolescents. They use the interventions equally. If the question is which outweighs, it will be difficult to assume but I can understand that all are utilizing the interventions.

**I**: Could transport be a barrier for access and utilization of services?

**P**: Transport is arranged in every kebele for example for delivery services but there are areas where the car services could be impossible because of the unavailability of roads. In these areas, there are traditional ambulances. Therefore, I do not think transport is a barrier to use the service.

**I**: How do you evaluate the service quality provided for the women and adolescents?

**P**: The services given to laboring mother is special. As we know mother should not die and special attention is given to them.

**I**: How does the beliefs of the community on given services for example youth friendly service? Do families allow their adolescent girls to utilize the youth friendly service or not?

**P**: This kind of activities are not accustomed by the community. To prevent these obstacles, the youth is involved in trainings. To further strengthen this, it needs to talk with community and community leaders. Any ways, this could happen may be because of lack of awareness of the community. But, I believe, this will be solved for the next time.

**Section 5: Multi-sectoral collaboration to improve maternal nutrition**

**I**: Do you feel it is necessary that your institutions should work focusing to address adolescent nutrition?

**P**: Yes. Everybody is aware of nutrition. But, there is a gap either related to economy or others. If we were serious in working towards this, excellent work will be done in our kebele.

**I**: I have heard that there is stirring committee in most of the wereda which aims in improving maternal nutrition. Is the stirring committee established here?

**P**: I am not stably working in the kebele. I am just telling you what I know from previous activities. All things are introduced. There is a command post, and other committees. I think it will be there but the follow up may have some inadequacies.

**I**: In most areas where stirring committee is established, the wereda administrator is a leader and the health personnel is a secretary. Others like women affairs, youth affairs, and etc are working in coordination to meet the objective of the stirring committee. What will be the role of your bureau in the multi-sectoral nutrition coordination? If you were member of the multi-sectoral coordination, what could be your role in improving the maternal nutrition?

**P**: Most leaders in kebele has many additional tasks. However, there should no task or other jobs without having health. If we were seriously committed on it, there could have been a good job done. Means, if we were participating better than the current involvement, there would be a good progress.

**Section 6: Other interventions that influence adolescent and maternal nutrition and health outcomes**

**I**: Do you think there is early marriage in this area?

**P**: Everybody knows that an adolescent girl should not marry under age of eighteen. This can be identified by recommendation from neighbors or others who observe it. There is also a law specific to early marriage. This is followed by women affairs. There are also representatives from law and governance. A trial will be done to solve the problem before it happens with development army and network. I do not think there is early marriage here.

**I**: What is the measure taken to a family whose adolescent is married at the age of below eighteen years?

**P**: As per the information I have, if a family allows their adolescent girl whose age below eighteen years, they will be jailed for a penalty of seven to ten years. The women affairs, and justice will stand against early marriage.

**I**: In your opinion, why would delayed marriage (after 18 years) improve maternal nutrition?

**P**: Yes. If an adolescent is eighteen years, she is matured and she has reached the fire age. It is considered that she has reached this age taking the balanced diet. Therefore, it will have an advantage.

**I**: How is the practice of birth spacing in this area?

**P**: Nowadays, there is a good job done in birth spacing.

**I**: How many years do you think the gap should be between successive births for women?

**P**: When we say birth spacing, there should be a four to five-year gap for a child from birth to the second pregnancy. It is four to five years until the first child develops well. Almost all are following this interval. There are improvements in this regard. You cannot see children born one over the other with shorter year intervals.

**I**: What programs or activities do you think will promote to increase birth intervals in this level? How do you think the community here is aware of this birth spacing?

**P**: Birth spacing is important to the first child to grow healthy and get balanced diet. It will have also economic impact. If the birth is one over the other, there will be a cost for growing these children. There will be impact on school attendance and the family will be face many challenges. This is my idea but there could be other ideas… (smile)…

**I**: What are the means of communication used promote the child spacing interval and prevention of early marriage and in this community? Meeting, women development army or others?

**P**: There is a situation where we can talk to the community especially those messages came from wereda. If the report about birth spacing shows a problem or gap, there will be a situation where people from the wereda come to address this gap in public meetings. Otherwise, the message could also be conveyed by the current networks via the volunteers or women development army.

**Section 7: Additional Remarks**

**I:** Do you have any other comments on anything that we must discuss on women and adolescent nutrition?

**P**: Things that should be done for youth has been raises by both of us. There should be separate office which coordinates the peer to peer for youths. There should also be supply of materials for training and support. The intervention given for pregnant and lactating mother is very good. But it should be given at each kebele than at wereda level. Most women around the town are using the kebele`s health center for labour and delivery but it was good if waiting area is established in every kebele which is near to every mother. There should also be a separate place in every kebele for youths.

**I**: Thank you for taking the time to discuss these issues with me today. I have learnt a lot from you. As I mentioned as the start of the discussion, I will remove all identifying information from the report of this conservation. I will make you sure that no one can identify your comments. If you have any concerns or questions, please feel free to ask me any questions. Thank you very much for your time. You cannot say everything is clear and applied by the community but there is improvement from time to time.

**Summary:**

**Section 1**:

- To stay healthy women and adolescent should
  - Keep their hygiene
  - Visit health facilities for check-ups
  - Participate in counselling on health-related issues
- Nutrition related problem like severe malnutrition and stunting are not common in the area
- Children with HIV and those elders are given food support
- Food insecurity is not common
- The micronutrition deficiencies are not common the community
- Anemia and goiter could be prevented by nutrition
- Diet related non-communicable diseases like hypertension and diabetes mellitus
- Adolescents are given iron supplements to prevent the occurrence of anemia

**Section 2:**

- School adolescents are given counselling about nutrition
- There are volunteers who facilitate the education and counselling about health including nutrition of women and girls
- There is a peer to peer education which could be a potential learning forum for mothers and adolescent girls on nutrition

**Section 3:**

- There is nothing done by the youth affairs office for the adolescent girls except arrangement of loans and related supports.
- There was a training for youths arranged by zone and its focus was on nutrition and reproductive health.
- All pregnant and lactating mothers are visiting health facilities without any barriers
- Audit of pregnancy and health facility visit is done by two ways not to miss any pregnant mother
  - By health extension workers and women development army
  - By volunteers
- A pregnant woman is advised to take four or five times a day
- There is a great achievement in utilization of iodized salt all households use this iodized salt
- Malaria is not common in the area

**Section 4:**

- The main obstacles for implementation of nutrition related interventions are wrong perception and lack of awareness
- Educational status will not affect the access of services
- Service provided for women is special and with acceptable quality

**Section 5:**

- Leaders at kebele level are busy with other administrative issues and give less emphasis to nutrition

**Section 6**:

- Early marriage is not common in the area
- Women affairs are seriously controlling the case of early marriage
- The recommended birth spacing interval is 4-5 years
