# Supplementary material for: Barriers to utilize nutrition interventions among lactating women in rural communities of Tigray, northern Ethiopia: An exploratory study
Source: PLoS One. 2021 Apr 30;16(4):e0250696. doi: 10.1371/journal.pone.0250696 (PMC8087028; doi:10.1371/journal.pone.0250696)
Supplement: S2 File — (ZIP) [file pone.0250696.s002.zip › S2_File.Doc/Community level Key informants/129-IDI_HEW-Hakfen kebele_Medebay Zana woreda.docx]

**Operational Research on Adolescent and Maternal Nutrition in Northern Ethiopia**

## Key informant interview with HEW

**Introduction**

Hello, my name is Amaha Kahsay. I am from Mekelle University. Thank you for taking the time to speak with me today. We are doing research on the factors that influence the nutrition of mothers and adolescents in collaboration with the Regional Health Bureau and UNICEF.

**Section A: Interview details**

1. Zone: North-West Tigray
2. Woreda: Medebay Zana
3. Kebele: Hakifen
4. Name of key informant: Wagaye Birhane
5. Institution of key informant: Health Post
6. Interviewer name: Amaha Kahsay
7. Date of interview: 21/11/2017
8. Interview start time: 03:25PM
9. Interview end time: 05:08PM

**Section B: Interviewee professional information**

1. Gender
   1. **Female**
   2. Male
2. Age: 31 yrs
3. Highest level of completed education.
   1. No formal education
   2. Primary education
   3. High school
   4. **College education**
   5. Bachelor degree
   6. Master’s degree
4. Current job/position: HEW coordinator of the HP
5. How long have you been in the current job/position:
   1. ______ Months
   2. 02 Years here; and 6 years at other two kebelles called Limiat and AdiGieshti

**Section 1: Common maternal (pregnant women, lactating women and adolescent girls) nutrition problems in the community.**

**I: It is known that you are highly burdened with the sixteen packages of the country that you are sacrificing a lot in achieving them; yet, if I am mistaken, I have heard that they are turned to be eighteen packages currently; and yet, it is you who is working at them and you knows all everything at the ground; so, in your opinion, what are the common nutrition problems in the community for women and adolescent girls at Medebay Zana woreda; especially at your HP level?**

**P**: Okay, what I can say it as a problem of mothers is that, though there feeding does have change from time to time, but when someone eats, it is not all about getting satisfied; but at eating the balanced diet, there is something unpeeled yet; when I say it to you, when someone eats, it is said that it has to take from all kinds of foods that we call, especially, from those four groups of foods. But even based on that, at some places, there is taking of limited foods; and I take it as problem.

**I: You told me that it is said that it has to be feed four groups of foods; may you mention them to me?**

**P**: Yes I can mention to you; from those four groups of foods; one, those which are called carbohydrates; then from those which are proteins; from those which are said disease preventers or called those vegetables; and from those ehhh…ehhh…we call them nutrients which mix from them. Thus, it is mean to the one who doesn’t feed these.

**I: Thus, very good; you told me that there is problem of improperly feeding at this area; at mothers and…?**

**P**: She interrupted my question and continued; now, there is a problem; but not at all; rather, at the ones which can the problem is be visible; like when it is measured by MUAC and by others, am I right; it can be visible at them; thus, we have a long way to do yet.

**I: So, we are saying that there is feeding problem to the mothers here; so, what kinds of nutritional problems due what are the problems that you get them here; because, mothers come to you and you screened them; you treat them; either you refer them; so, what are the problems here?**

**P**: Now what we call it a problem is that, because, we are here; what we do at mothers is, if she is pregnant mother, that of we call body mass index can be done to her by relating her weight to her height; but what we do here for her is that mostly it is MUAC; and if the MUAC of the mother indicates below 23, it indicates she is at malnutrition; hence here, many mothers are found. Previously, it was 21; but now, it is changed and if she is below 23, then it indicates that she has malnutrition; yet, it has its own levels; still it indicates she has that; thus, we found form the mothers that are found here.

**I: How many mothers do you get?**

**P**: Ffrom the totals?

**I: Yes; how man malnourished mothers are found here?**

**P**: From those who are measured mothers; for example, we measure pregnant and lactating mothers; we measure around 178 per month; that is as per our kebelle; so, if we measure this much per a month, thus for example, from these, from these, those who are around 42 are… (I have observed that she was begun be destructed by something; and that was here colleague HEWs); so,

**I: Please you better get out; I said to them.**

**P**: She laughed then. And they went out.

**I: So you said you measure 178 mothers per a month; then how many of them are endangered by the problem?**

**P**: 42.

**I: Which problem do they have then?**

**P**: These are those their MUAC is below 23.

**I: Which one are they again from those pregnant and lactating ones?**

**P**: More of them we found are those lactating.

**I: How man may they are?**

**P**: For example, those lactating in the previous month were about 28.

**I: Okay; do you mean the rest are pregnant?**

**P**: Yes.

**I: Which measurement cut off are you using then?**

**P**: That of 23.

**I: When did you start it?**

**P**: Since 2009 (EC counting)

**I: In 2009; on which month?**

**P**: I don’t remember the month. But, it was around the beginning of 2009; either on Hamlle (July) or… (She pause it here)

**I: Who did let use this measurement?**

P: The legislation.

**I: Which legislation then?**

**P**: The region’s guide line.

**I: Why I am asking you about this is not for another purpose; we were travelling to other places by this study; and they told us that they are not using it yet; we asked them why and they told us that it is because the legislation is not come to them; so, how comes, it arrived here for you alone; indeed it s nice that you are applying it; but why it becomes so different at different places that they are using 21cm but you are using 23cm cutoff point; I just need if any explanation? Is that you got letter, or training or legislation; what did come to you?**

**P**: It was training; we were trained; when we were trained at this woreda, for example, it is now being said that vitamin A should not be given to lactating mothers; this itself is given to us at training; and that is similar to this.

**I: So, when did you take the training of that 23cm cutoff point?**

**P**: It was around Gunbet (May) 2008 (EC counting); that we were trained.

**I: So, is that mean all at the woreda level uses this cutoff point?**

**P**: Yes, that is it.

**I: That is nice; what did you say about that vitamin A; can you tell me more about it?**

**P**: That vitamin A is also not being given for lactating mothers; because… (She would to continue, but sorry I interrupted her unconsciously)

**I: Who said so?**

**P**: Ohhh! (She laughed being surprised at the question); it is legislation; but what is the reason given as reason is that study was undergone between those who were given and not given; thus, what was found from the study is that it was not found any change among those who were given form those not given; thus, it is said that giving vitamin A for lactating mothers is meaningless; and then it is abandoned. Yet, we give for the children from six months up to five years.

**I: Since when was that abandoned?**

**P**: I think, it is since previous Miaziya (April) 2009 (EC counting).

**I: How about this, its legislation, who did bring it to you; let us stress here again?**

**P**: We were given training ourselves.

**I: Who did give you the training?**

**P**: There was someone from region who gave us training about nutrition. He trained us about nutrition; and it was told to us at that time.

**I: So, you have stopped since after that training?**

**P**: It was told us during that training; so that is it, we have never given since after that.

**I: And about that 23cm, was that like in such kind of training give to you?**

**P**: It same at training given to us; and also the woreda repeatedly gave us information.

**I: What did they, say to you?**

**P**: The format for reporting itself currently, says above 21 as it is old one; but we write it as above 23 by ourselves;

**I: Uhhh…Uhhh…at the…?**

**P**: Now at the format, because it is of the previous time, it says above 21 and below 21; but we correct that 21 to 23; since it has been told to us.

**I: How about that of Vitamin A; is there any direction came to you; what did the woreda say to you?**

**P**: It is only by that training; but the woreda told us orally that it is not given for lactating women.

**I: Very nice, uhh…so now, what change did you see after not giving that Vitamin versus giving it previously?**

**P**: We didn’t see; we didn’t see change.

**I: Are there mothers who request to give them and asking you why it is abandoned?**

**P**: Yes, they come; now what they ask is that, why that kind of tablet used to be given after delivery is lost? But when they ask, as per the training given to us, we tell them that that vitamin A medication is given to child from six month up to five years; but it is not given for you; and they ask why it is left; a study was given at those who were give vitamin A; and those not given; but didn’t have change; thus, if that medication that comes have not change in health, so it has not meaning; thus, it is let be used for children; we tell them this; thus, based on this, they stayed calm.

**I: So, up to when are they not to take it?**

**P**: What is here now is that, up to her six months, it is thought that it will be used us a protection for the baby; so, as it is said that vitamin A is the protection for the baby that she has to stay taking vitamin A inside her body; up to her six months of lactation; but, it is that she can replace it from those vegetables and other foods; it is that if she takes these, it is mean that the medication vitamin A is taken. So, because it is, we teach them to improve their feeding system; and what was present is that those below six months used to take that; but mostly it was below their 42 days, that we were giving them; if she was not to take at her 42 days, she was to take up to her below six months. Thus, that is the reason for abandoning it. It is left due to this; and it is that we have to go through feeding system.

**I: Okay, very nice; is another thing being left like that of vitamin A?**

**P**: No.

**I: You told me that there are mothers who are below 23cm of MUAC measurement; what else nutritional problems are present here?**

**P**: At limited place, there are who have Hiffess (goiter); but they are very limited.

**I: What does that they are limited; how can we mention it?**

**P**: For example now, those we say them lactating and delivering mothers at our kebelle are, around 320; those who are lactating and pregnant ones. Thus from these, that we know them until then, that those who have goiter are found to be around 31; that goiter could be either small or big; whatever so, those who have goiter swelling.

**I: From them, which of them have more from the pregnant or…?**

**P**: Ehh…those lactating mothers are more.

**I: What do you think it is more at them?**

**P**: Uhh?

**I: What is the reason, what do you think about it?**

**P**: The reason is about feeding; at the feeding, now are telling them that let us use iodine salt; the iodine salt, one, it let the baby be sharp; it helps for her mind to be sharp; in addition also, to extinct her goiter or Hiffess; am I right; so, it is because there is problem here, that it is still being observed; it is chronically staying problem though it may not happen currently; even if you see now, even from the ones who have goiter, the new generation being born currently, is going a bit better now; thus, it is that due to there was problem in iodine salt utilization; it was, the community was eating using that of the usual cattle’s salt (she means the bars of salt).

**I: What else problems like that of anemia, severe acute malnutrition or others are observed here?**

**P**: Severe acute malnutrition, at around 2009, we treated around four.

**I: Who are they?**

**P**: Severe acute malnutrition! (She said it strongly that seems to indicate that it is the problem related to children); I am saying children. Children, we treated around four. Thus, it is also currently present; not extinct; it is not said it is extinct. Little from what had been it is now better; per a year, they were to be treated sixteen or nine or like that; next, it has a bit change; but also now, it is still present.

**I: Why it is present then?**

**P**: Oh goodness! We didn’t improve our feeding system yet; we have long way to go yet; this is the reason.

**I: What is that feeding system, that why it is not improved yet; is that because no food to eat or we don’t give them education; what is the reason?**

**P**: Most of it is because of awareness problem; though you know again, there is problem of transferring it into practice; for that matter, even it couldn’t be problem of knowing.

**I: So, why it is not transferred into practice; what is the problem behind it?**

**P**: Most of it is that of problem of awareness; yet, little has shortage of food too. It is mentioned in two ways; for example, how is that is, of the rich though it has food access, it is caught by shortage of food (she means under nutrition); and of that poor, because it has not food access, it is caught food shortage (under nutrition); this is in two ways; but what is seen mostly is due to that of problem of awareness; that under nutrition which comes due to being poor is less than that of under nutrition that comes due to awareness problem.

**I: Okay; Wagaye (calling her name), it is known that nutrition is part of your packages; for all I know, it is there being as cross cutting issue that you get it anywhere; and mainly it is about creating awareness and screening activities; but here you are saying that there is feeding awareness problem of the community; yet, I am not saying you are not working at it; but what could be the reasons for this problem?**

**P**: May I explain it to you; now in the case of shortage of awareness; awareness can be mentioned in two ways; that awareness you tell going at one gathering place, and an awareness that you tell going to, home to home visit are so different; because if you need to be listened by someone, what you tell at gathering may be take like a sort of idea; but what you tell separating for an individual (she means at home to home) can say it is important and can hold it as key message. Thus here, where we go is in two ways; one, when we undergo home to home visits, we teach about nutrition very well; but when we teach there…! When I say awareness now, for example, when she has egg at home, she sells it and if bring to the baby those good for nothing like biscuits and like that, this is awareness problem still; though I told her too; this is what I am telling you about it; otherwise, it is not mean the awareness is totally absent or like that; otherwise, we are working as much as to our capacity; we are helping to each other with the development armies as much as possible; and the left one if we address it completely to 100%, the feeding system would to be also turned to be 100%; there wouldn’t have been a problem; but there is place still not addressed and there are also individuals who don’t hear you; these are the two ways.

**I: Do you mean; there is community that is not addressed yet; by you?**

**P**: Oh! You may go simply; but I am saying you have to go very repeatedly; you have to go again and again and let them understand; otherwise, there is no place that we didn’t reach; we go always.

**I: So, if you go always, for example, if you come today and teaches me, and if you come next month and teaches me, and so on; don’t you think I will be changed; or what else is the problem?**

**P**: Now, what do you see is that, the number has to be seen; for example now, if we visit to a household, at least we visit once per a month or we may not reach it; we have 1884 households; so, in each month for each household, we can’t reach form edge to edge; if it is nearby, let alone once per a month, we may go even five times per a week; if it is nearby; when we pass through it, when we come through it, we can tell. But those faraway households, we may not reach them per a month, and even there are that we don’t reach them per a month; thus, due to this reason, when you tell him one thing today, he says you okay; and when you get back after three months or like that and when you ask him what change is present based on what was told; it lets you get back to the same previous talk, it is some of those individuals; though we didn’t go much, I am talking about those who didn’t bring change. Thus, the problem is of all starting from me; one, we didn’t reach all to the community consecutively at each month; secondly also, getting adequate awareness, the community is not engaged into the practice; those who have, are not feeding well; and those who have not, are not also going in the right feeding way. Even currently, that absence of food can’t be reason even (she means there is food security); whatever so however … (she said nothing following this)

**I: What other problem like anemia, and Himma (night blindness), are they seen here?**

**P**: Ayii (no), they are not seen much here. What is here is that, the mothers are measured during their pregnancy; and they take also iron during their pregnancy. Thus, if they take and if they didn’t even finish it during pregnancy; they also take up to their 42 days after their delivery; thus, I personally, didn’t get and hear anyone with sever or any bad anemia until then.

**I: How about that of night blindness?**

**P**: In that night blindness, we have around two mothers that say it gets them dark at night; they tell you that during dusk; when it gets dusk, they say that it is difficult to them to see and like that.

**I: Are they two?**

**P**: Two.

**I: What? Are they pregnant or lactating?**

**P**: The one is pregnant and another is lactating.

**I: So, what was done for them?**

**P**: For them?

**I: Yes.**

**P**: For them; even now what we have to take is about the feeding system; now, on those we call them vitamins; those vegetables, we gave them advice that they have to feed those vegetables; ehh…we gave them that advice; there is no any other medication that we gave them.

**I: So, do you think it is enough to them; I mean this seems to be in the level of night blindness if I am not mistaken; thus, is that enough for them that of advice?**

**P**: Now what do you see is that, it was in 2009 (EC counting), thus, that one who said so, she took vitamin A during that period; because, vitamin A, was given up to that period; it was used to be given and we gave her. Whereas, the one was pregnant; and pregnant also can’t be given Vitamin A; thus, we advised her to correct her feeding system; thus, for it, additional medication is not needed; whatever so, if it is needed for it, it can be indicated (like she needs to say you can indicate me if needed).

**I: But, if a pregnant mother is found to be with night blindness; does it mean she has not to take vitamin A; utterly?**

**P**: She has not to take; I don’t know this until then; she has not to take; I don’t know this.

**I: In my opinion, if a pregnant mother is with night blindness, she has to take vitamin A; but the dose has to be lowered; I remember, I have read such kind of information; so, let us take it as assignment for both of us about it; okay?**

**I: So, are they fine now?**

**P**: They are fine now; they are now fine. Anyways, how the night blindness comes is that, due to those vitamins problems; thus, we informed them that they should take those vitamins which can be taken from the foods those vegetables and yellow colored foods; and accordingly, they are fine now little.

**I: You mean still it is present on them?**

**P**: Oh! Those who say it is dark to us at dusk, there are even elderly males; but because it doesn’t include them that we are talking about mothers.

**I: Okay, I understand; but are there males too?**

**P**: Yes, there are males too.

**I: So, what is done for them?**

**P**: Oh! We gave them vitamin A at that time.

**I: Okay; for how many times is given to them?**

**P**: For today; tomorrow; that is it.

**I: May there be that overweight; those who may be overweight or obese?**

**P**: How comes! There are no! Those, overweight; (She laughed very extensively)

**I: What makes you laugh then? With helping to her laugh too**

**P**: Those overweight! It is because I am surprised. (She laughed again); I thought that if you think you would to get them here.

**I: Why not, why don’t we get them here?**

**P**: Ayiii (like feeling of sorry); they are not reached there yet; they are not there yet;

**I: Why said yet; cannot be overweight here?**

**P**: We didn’t see with our naked eyes until then, that who say we are overweight and being stressed about it.

**I: Do you think that females are at higher risk to be exposed for malnutrition?**

**P**: Yes they are exposed at higher risk.

**I: Why, do you think?**

**P**: Why it is said is that, because it is mothers that serve the food; but how the mothers can be hurt is that, as it is known that our area’s tradition, due to the long stay backwardness, those better foods though it may be sporadic at this time, they prepare it saying for the husband; thus, due to the presence of such kind of thinking, they may wait for not eating alone; though some of them may eat alone yet; hence, even it may different as per different individuals; related to the culture, they think that it is for the husband; thus now, I think that there will that problem at our mothers linking with that previous backward culture.

**I: How about that culture, is not it peeled yet; that is, husband first, later children or like that?**

**P**: There is some; it is not peeled yet.

**I: Have you ever faced as such issue?**

**P**: Oh! Yes we faced; we face it yes; at some individuals.

**I: So, what do you do?**

**P**: We teach them letting them be together with the husband.

**I: So, are there individuals who are changed by doing so?**

**P**: Oh! Yes they are being changed; otherwise, it was all like this previously; yet, we can’t say it is totally changed.

**I: Would you tell me its weight that from how many males; do such kind of males present?**

**P**: This one; I have not it.

**I: Okay, how about heights of females right here; are they tall or short or what; how do you observe that; how do you see their height in relation to their age?**

**P**: Their height in relation to their age, at the current one, for example if you take child aged 14 years and if you relate him with the previous one; it is better; I mean the current one. But at the current mothers who get pregnant and who deliver; a bit the problem is present at some of them; there are some short ones.

**I: What do you think could it be its reason?**

**P**: The reason is that, that is the feeding as I told you. Our feeding system was backward; tat stunting was begun from it; even thought it may not be that visible stunting; the short stature is present.

**I: What do you do as a solution?**

**P**: Oh! This is being short; so, what can we do!? We tell to improve its feeding system for the next generation; but that stunting cannot be getting back immediately; because, it is irreversible.

**I: Is there any food shortage here; that of food insecurity?**

**P**: Food insecurity, it is present at some.

**I: How, in what conditions?**

**P**: When you say what kind?

**I: I mean, in which kinds of individuals is that observed?**

**P**: If we are to say we have food security, all the people, that the government is supporting us, has to come out all and has to get its own food something; am I right; is we need there is food security; otherwise, it is because there is food insecurity that the government is saying safety net and like other aids; thus, it is present!

**I: How about the farm products of the community; may it feed all the year round from its farm products or mat there be not eating breakfast or lunch due to food shortage?**

**P**: Currently, it is fine; though it may not being fed all the year round, due to the assistance that I am telling you; ehhh…there is no anyone who eats breakfast but never eats lunch; it all may not eat three times per a day; but those who eat down and dusk is, it can be said that it is almost all; almost all 100%.

**I: But, don’t you think all doesn’t eat three times per a day?**

**P**: All the people, how comes! It doesn’t eat! The people who eat three times per a day is that around sixty something; as the agriculture mention it.

**I: Oh! How about the rest ones?**

**P**: Two time per a day! It is 82 or like something percent, that perhaps, those who eat three times per a day.

**I: So, you told me the government is aiding here, what is that helping?**

**P**: There is safety net program here; thus, that safety net can be by work or by those disabled ones; based on this, it helps them.

**I: Okay; what is being given in the safety net?**

**P**: It is given cash and also crop; for example now, if it is due to snow destruction of cereals, there is a condition in which crop is given; but if simply poor though the crops are not destructed, there is a condition that cash is given.

**I: How much money is given per individual?**

**P**: I think, it is tow hundred thirty five birr if I am not mistaken, it is like that;

**I: How about the crop; what is given?**

**P**: Wheat

**I: How much kilogram wheat?**

**P**: Thirty or eighteen; or twenty or like that; anyways, I don’t know it for real;

**I: Don’t you participate at the safety net?**

**P**: At the safety net? How comes!

**I: Is that not important to participate?**

**P**: It is important; but we didn’t think it in this way. (She laughed)

**I: Is there drought here? We thought it is rich area?**

**P**: Indeed it is rich; but you do see here is that, it is even suitable for irrigation more form others; even it can improve nutrition; this place. Otherwise, if you go to Adi Ngsti kebelle, worse than ours that even you may get up to tens of malnourished individuals; it is a kebelle found far below from our kebelle; because there, the natural recourse works (she means conservation) is very dead, and similar that of irrigation; thus, the feeding system is very worse there in the kebelle; but here, it is better. This kebelle is better in its feeding system compared to that kebelle, though it cannot be said that it is rich; it is better food secure as those agricultures said it; and it is possible to ask them too. So, it is not far poor; or it is not far rich; there is a lot that has to be done yet; as to me it is not as such worse, it has irrigation access that produce those foods that we are supposed to eat.

**I: From those pregnant and lactating ones; which of them are at high risk of malnutrition?**

**P**: Now, it is more visible at those lactating ones; as far as I know at these lactating ones, because they lost their appetite during their pregnancy, then when the delivery comes too, that loss of appetite continues; after giving birth, her body gets down and her MUAC also lowers down; thus, it starts from pregnancy that of feeding; that it goes being degraded. Hence the advice that we give them here is that, to eat from all kinds of foods available at their home as per their interest; and even they feel sick or have vomiting during pregnancy, they have not to stop eating as much as possible.

**I: Is there anything that you work at the adolescent girls to improve their health and nutrition?**

**P**: Yes; we work.

**I: For example, what do you work?**

**P**: Now, at schools, we give them education about nutrition on how to feed; they could be from the age of thirteen to around twenty one years; anyways, as per the school is having them; so, we give them iron tablets too. In the previous year, we gave them in every six months; but on this month, we didn’t give them yet. But, we undergo to them in every six months; and we also have program at the kebelle level for the next to give them.

**I: For how many days is then given to them in that per six months?**

**P**: For three months in every six months.

**I: I mean, for how many days do they take the tablets?**

**P**: The days?

**I: Yes indeed, for how many days do you give them going there?**

**P**: That is it; there is that is something, that there are sixty tablets and we tell them to use it at morning and evening; because they are at menstrual period, that they can bleed more; thus, that is why; it is given to them. Thus, we give them to take it at morning and evening; and we give them for around two months; which is 120 tablets; they take this.

**I: Then, are there any nutritional problems that are observed at them?**

**P**: Oh! How it is then is that, we measure by MUAC if she is lactating or pregnant; but out of this, if we see any visible shortage of food (she means under nutrition) being her young or not lactating, we may measure. But, we didn’t see; because, the current generation is a bit better in its nutrition when it is compared from the previous one.

**I: Do you measure the adolescents like for the adult mothers you do?**

**P**: We didn’t measure them.

**I: Why then?**

**P**: We didn’t measure them.

**I: Why?**

**P**: Oh!! Nothing; we didn’t measure them! We don’t even know if it is measured. What we do for them is that we vaccinate them TT, we give them iron; we also give them health education about how to do; these are what we give them; bout hygiene works, we give them how to keep their hygiene; other than this, we didn’t measure and classify them where to be.

**I: But, do you think it would be necessary to do that for them?**

**P**: Oh! Yes it is necessary. But when I say it is necessary, my concern is that, when I measure her MUAC, she is child; how can I know her then; she is kid; let say for example she is 14 years; so, how much do expect her MUAC to be; that MUAC has not age; thus, how can it be measured then; I simply have that concern.

**I: Have you ever asked about it?**

**P**: We didn’t, but I internally have concern about it.

**I: Being concerned about nutrition of adolescent girls; may it have importance?**

**P**: Yes, it is important; because, why I say it is important is that, if she doesn’t feed during her adolescence, she will not have that adequate body something; if she has not adequate body capacity, she will be pregnant and give birth in the future; then the baby will be similar to her; thus, because this is it, working at adolescence is necessary; though the reason is as I am telling you; yet, it needs man power; because, when adolescent are seen at each something, they are much.

**I: Thank you, what else nutritional problems are seen in this area?**

**P**: Not others.

**Section 2: Nutrition priorities in the health post**

**I: Together with your colleagues HEWs, here in the HP, what are the priorities that you work at maintaining the health and nutrition of mothers and those tomorrow’s mothers (adolescents)?**

**P**: Priority…?

**I: I mean that you give them more focus?**

**P**: Okay, what we are doing in priority for mothers is that, at their feeding; that is about how pregnant mother should feed; we counsel her about her nutrition; in addition to it, she has to be checked up, she has to deliver at health center; it is because, if the mother is present, that we can say it all. Thus, as to the priorities we do, there is nothing that we do more than that of delivery; thus, the mother has to deliver at health center, the baby not to be hurt, we work at it giving priority. Again with that I have said it the nutrition; and that of vaccination, we work on these issues giving priority to the mothers.

**I: Very good; what do you counsel the pregnant and lactating mothers about their nutrition?**

**P**: What we counsel for pregnant mothers is that, we counsel them about to eat balanced diet; we counsel them that that of not eating eggs and soft foods in fear of the fetus will be big is, a backward thinking; she has to take different food sources; if she takes so, she can deliver healthy baby; if she delivers healthy baby, up to his 2^nd^ year of age how she has to feed him, what kind of food he has to take; and again, form what she used to eat, like if she was eating three times, she has to add one then has to eat four times; we give her this counseling. And if it was four times, she has to eat five times.

**I: What if she was eating only once per a day?**

**P**: That only once is not correct; anyone, at least he has to eat three times per a day; morning, noon, and evening; so, there is on such kind of feeding and any human can’t feed like that. Whatever so, using that average three times; we counsel her to use four times.

**I: But maybe, you told earlier that the community that is eating three times per a day is about 60%; so, what if she comes from those 40% who eat either two times or one time per a day?**

**P**: If she comes, what we say for her is that, the improvement at your nutrition is for your next generation; you have to improve your feeding habit. If we ask her how many times she eat; she can say us two times; if we ask her why, she can say us due to lack of appetite.

**I: What if she says I have not to eat?**

**P**: Leave that please, they can’t stay out of food. (She laughed). Whatever so, let her say she has not; currently, the community is giving another name even for their feeding system; as there are not individuals who can say I have not; then, that they pass the day with no food. but if it happens, we counsel the mother that, at least she has to eat that average of three times a day; and also she has to add some sort of snacks as per what is available at her home; we give this counseling; otherwise, telling her what is not available at home and can’t do it then is just good for nothing.

**I: What could be those balanced foods that she has to take?**

**P**: Those I have said to you balanced foods are those which can be made “Injera” are Taff”, maize, sorghum; again from those stews, those we call them proteins and they can be legumes; from the vegetables again, spinach, salad and kale; and can be also carrot, onion, and tomato; and from those we call them nutrients those vitamins and minerals are together with the vegetables; so, we counsel here she has to feed all these.

**I: How about in the lactating mothers?**

**P**: It is the same like that; she has to eat adding two times.

**I: How about to those adolescents? Do you advise them?**

**P**: Yes we advise them.

**I: What do you say them?**

**P**: Now, what we counsel them giving focus is that, one, about their nutrition that, not to be affected by the previous backward thinking of food is not needed for a female; second, we give them counseling that if a female feeds well, she will be healthy and also she will give birth to healthy baby. But mainly, we counsel to their parents; but for them it is the general condition about feeding that we counsel them.

**I: Why didn’t you counsel for the adolescent well for them?**

**P**: We didn’t do it.

**I: Why?**

**P**: What could it be then; when I think it personally; they are living with their family; thus, that we practice if the parent; thus, because I tell her, she may convince to her parents; but that who deed it is the parents being concerned about her future life too.

**I: What do you think if we able to do at themselves; would it be better?**

**P**: Working at both would be better; now how I do see is that, because the one who prepares and serve the food is the adult one who stays at home; so, talking about the foods with may not be as such something; yet, you are right that, working at them is also good.

**I: Maybe, what could be the reasons that we are not working well at the adolescent nutrition?**

**P**: It is not given focus; otherwise, it would to be done.

**I: What do you suggest for the future to be done about them?**

**P**: What can be for the future is that, once we enter to the family, we have to gather all the family members and they have to be given education for all at being together; because, what they want to ask, they can ask and what answer they need can be answered there; and that feeding system can be also well heard; otherwise, as to case, talking to the adolescent girls alone may not bring change still; but it could be better mixing them together; because, they (the parents) can even say to them what do you know about; so, teaching them alone is not as such…

**I: What is your perceived fear here?**

**P**: My fear is that, I can tell for a girl that she could be thirteen or fourteen years; if I tell her you have to feed in this way, she may say me okay; so, she will go home and will say the health worker told me that I have to feed in this way; and she will get the answer of what would have been happen even we adults would have been fed like your way; they could say her; but if it was with her family, they will hear it in person themselves; this is in my personal opinion indeed.

**I: So, do you think it is necessary to give focus and to work at maternal and adolescent nutrition?**

**P**: Yes it is necessary.

**I: How?**

**P**: Because we have to work at mothers; otherwise, working at others is meaningless; because, it is those mothers who deliver and be pregnant; and that can change generation. For example, if we are to eradicate that of stunting and wasting, we have to work at mothers; especially at those we call them adolescents; thus, at them again it will be needed to be worked in the way I told you.

**Section 3: Nutrition interventions that improve adolescent and maternal health**

**I: What nutrition specific interventions are being done at your health post level to improve maternal and adolescent nutrition, in addition what we have discussed above?**

**P**: From the works, we show food fair demonstration at each village; though the adults don’t eat the food, we invite the pregnant mothers and we show them how to prepare the food for their children; and we give the foods to the children of up to two years of age. And also we show her on how to prepare it at home and how to feed for her child after giving birth; and also how to feed hers too.

**I: What do you do and what do you show them in the food demonstration?**

**P**: We show them in each their villages; we show them once per a month. In this village we show them here in this health post; and at those two villages; there are places where they are convenient to them; then we do that there.

**I: Is that at home or where?**

**P**: No, it is at beneath of tree shelter; thus, one, we let the pregnant mothers come; and those development armies who coordinate them come too; we also let those lactating ones who have children less than two years come; then we show them each things what are needed to make that porridge; after that, we show them also about how it is made practically about everything that is being added to it; then next; we let them give their children to eat accordingly.

**I: Who eats then next; are that only the children?**

**P**: We give to the children; we also give to the mothers too.

**I: Where are the crops come from?**

**P**: From themselves; from their homes.

**I: Don’t they refuse to bring?**

**P**: They don’t refuse.

**I: Okay, how is its acceptance?**

**P**: Its acceptance is good; because it has taken wide time since it is begun here. It took around two years since it has begun here. Hence it acceptance is good; when I say this, the mothers see it here how to prepare; then, going home they prepare and give to their children.

**I: How is the behavior of the mothers in visiting health facilities like to this HP, and to health centers?**

**P**: Still the acceptance is good; but it is still after you go there repeatedly to their home and let them come; otherwise, thinking that it will be benefit them and having self initiation to visit to health facility still have a bit gap.

**I: Who initiates them then?**

**P**: We ourselves being together with the development armies?

**I: What do you do?**

**P**: For example, if she is pregnant and if she needs to be checked or if it is the time for her check up being it her first visit or other; we teach her in detail about how to go and what benefits will she get if she goes to health facility; thus, she is checked accordingly.

**I: What is then done for them here in addition to the MUAC measurement?**

**P**: Here, MUAC is done for them; second, that what we call it BP; ehhh…measuring their blood pressure; plus, what call it physical examination starting from their hair up to their leg swelling including listening of the fetus; then doing these, we send them to Selekleka health center; most of the time, they don’t undergo checkups here as some part of our kebelle is to the town side which is nearby to the town; thus, those who are nearby to the town undergo the four visits there, and those who are near to us, visit this health post; and we let them go via this to the health center.

**I: How about in the case of the hygiene of the mothers and adolescents; how do they keep that?**

**P**: To keep their hygiene, they are given education; base on that we give them education about when to wash their dresses, when to be washed their hair and their body too; accordingly, they apply it; but not all; there are also few who look dirty; then we give them counseling and then they apply it.

**I: Why don’t they apply it if they are advised?**

**P**: Oh! That is, the thinking of mankind is so much; I may receive fast or late; yet someone may receive late; someone is different from another one; that is the reason.

**I: How about that of backyard vegetable gardening, are there, mothers who practice this?**

**P**: Yes there are, and how they are, is that, it is being given lengthy trainings about that backyard gardening; if one mother is to improve here diet, she has to use vegetable gardening like those of vegetables; and thus, this is being done.

**I: About how many mothers practice this?**

**P**: Their number?

**I: Yes**

**P**: Ayii (no); I didn’t have it. But there are indeed.

**I: What benefits do they get then?**

**P**: About the benefit, they serve for themselves and also hey help to their neighbors; especially if they are alongside water sources, by producing vegetables, they help themselves, and also they can serve their neighbors by selling the vegetables to them; they are present as many as possible as this area is of irrigational place.

**I: So, what do they produce?**

**P**: They produce spinach, salad, cabbage, tomato, onion, mango and avocado.

**I: How about producing and self-use; do they take it to the market or use it at home?**

**P**: This is mentioned in two ways; producing themselves, they prepare it for their diets; there are also who produce but mainly they take them to the market; using for themselves whatever so anything. So, had it been all this changed, we wouldn’t have been talked about that of nutrition; so, this problem is present; there are who produce and take it to the market; yet, most of them they produce and use for themselves; they are higher in percent.

**I: How about de-worming, do mothers get this service?**

**P**: Yes.

**I: What do they get?**

**P**: For example, if she is seven or eight months of pregnancy, she is given that so called de-worming anti-helminthes tablet; she is not given if she is below 28 weeks. Is she is below 28 weeks and if she has symptoms of these helminthes, we send her to health center and get treated there.

**I: What kind of tablet are they given?**

**P**: Albendazole; given one time; one tablet; one of 100mg.

**I: Do they use it then or throw it?**

**P**: They use it; we give them here in front of us.

**I: Is there malaria here?**

**P**: Ayii, there is no much. Yes there is, but it is not much.

**I: How about that ITN then?**

**P**: ITN, in the previous year some was come being said for pregnant and lactating mothers; but now it is given to us for all the community; and we have been distributing it; even we have four pieces that are not given yet.

**I: Is there targeted supplementary feeding for mothers here?**

**P**: Okay; about that food support, there was no foods that was given to them until then; but now since the previous October, eighteen mothers are included by the save the children organization, and it is nutrition based inclusion; they are helped some supports to buy sheep and to practice backyard gardening to improve their nutrition. But before this, there was no any support given to pregnant and lactating mothers and two years age children.

**I: What was the support given to them?**

**P**: The sheep, the give them by buying; three sheep per each; and also they gave them that of the seeds of the vegetables like seeds of salad and spinach; and also those agriculture materials like hoe and others used for the gardening; and this is in process not begin yet.

**I: How about Faffa or like that?**

**P**: No, it is not present. It was around 2001(EC counting); but not after that.

**I: Why it is lost then?**

**P**: Oh! (Long silence); why it is lost is that, what is right then is that, we screen the nutritional condition; then, those who are critically undernourished or who are at the red measurement; if they are children, we follow them here by the OTP program; if she is adult, we refer her to Selekleka if it is critical; but those who are at moderate malnutrition, previously they used to get Faffa; but now, we give them only counseling on how to use the foods available at home; out of this, we have not Faffa or other things to give; but those who are at the worse kebelles like that of Adi Ngsti are included in the food security program; or else, we transfer them to safety net to be included there; that is if they are moderate malnourished and poor; otherwise; they have to correct their dietary practice for the things available at home; but if poor and moderately malnourished; we let him be included to the safety net by informing to the kebelle leaders.

**I: Do pregnant and lactating mothers get safety net services?**

**P**: Yes.

**I: Is that because they are pregnant and lactating or?**

**P**: No; it is if they are found to be moderately malnourished; or if they are poor then if the children are to be malnourished, they are included there. But if they are fine at their homes; because their nutrition can’t be changed due to safety net; we rather, advise them to use what they have at their homes.

**I: If they join safety net; are they let work publicly?**

**P**: No, they don’t join.

I: What is done for them?

P: Now if she is pregnant and if it is below four months, as we can’t know it; we send her to Selekleka; thus, if it is approved at Selekleka that she is pregnant; she is not forced then by the safety net program to join into work.

**I: How about after her delivery?**

**P**: After delivery, she rests for six months.

**I: What do they use with that rest then?**

**P**: Oh! What can they use it then; simply, they see their child. What else can they use then?

**I: Like coming to you and being checked their health?**

**P**: Yeah they use for it indeed; I thought you asked me what work do they work during that. They use it then for getting post natal service, for vaccinating their children, and for feeding their children; they use it for.

**I: Are there schools which do get school feeding?**

**P**: No, there are not. What kind of food?

**I: Porridge or like that?**

**P**: At school? (Being amazed); ayiii! not started.

**I: What is then done to those school adolescent girls?**

**P**: At school, we teach them about their hygiene and education about their dietary practice; otherwise, we don’t have food demonstration for them yet.

**I: From all your works about maternal and adolescent health and nutrition; which one of them are your most successful and less successful works?**

**P**: Okay; that I can call our successful work is that, we work fine at delivery and vaccination works; but, that I say, it has to be worked for the future, though I can say it weak is that but that needs focus for the future is, that of about nutrition which I already told you.

**I: What do you think are the reasons that the delivery and vaccination are successful?**

**P**: The reason that it is successful is that, starting from the higher level to the ground level; it has been given focus; that vaccination is long staying work and it took much time at work; and that delivery work is given focus from all higher levels and is being lead by focus too; and utterly it is linked to life, though yet that nutrition is also linked to life; that nutrition is indirectly linked to life; **whereas**, that one is directly and that is why it is given focus.

**I: How about that one; what do you think that it is not successful that of nutrition?**

**P**: Why it is not successful is that, that of awareness problem; for example, when you teach the community about delivery and nutrition, the acceptance level is so different; when you tell that the problem comes due to feeding problem; some of it may accept you though them says indirectly; and some of them refuse that it is not due to it; rather due to other things; it is due to this, that it is not successful until then; and also it is not given focus too.

**Section 4: Implementation challenges and community factors affecting access to nutrition interventions**

**I: Maybe, in your attempts to achieve the success of your interventions in all what you have tried until then at improving the maternal and adolescent health and nutrition, what challenges do you face?**

**P**: Okay, from the challenge that we are facing, that of the nutrition challenge is that, there is a bit less awareness on consuming balanced diet; because it is simply present that of the practice of filling your stomach with any kind of food; so, it has to be done for the future. Another that I can say a challenge is that of adolescent females who they would have brought better change at their nutrition; and also they would have convinced at least for their family; but it has not been done at them yet; and even utterly, it is those adolescent that they will deliver and be pregnant for the future; but not done at them yet.

**I: What other factors did hinder you working at these issues; being it like maternal education, religion, couture and others?**

**P**: That is the thinking problem; that of the long staying previous culture, in which it is has been said that those sweet foods for husband is still influencing us. And that of the previous belief about giving eggs and soft foods for pregnant mothers is still influencing us again due to the reason that it was not as an experience practiced in the previous culture.

**I: Is that of not giving eggs and other foods for pregnant mothers present until now?**

**P**: I may not say it is present; but that kind of perception is not peeled yet.

**I: Are there food tapes currently that are not given to pregnant and/or lactating mothers?**

**P**: That kind of thinking is present; and it is not on those who can be become pregnant or lactating; rather, it is found on those elderly mothers that they only hear that of the previous ones but not know about the current issues; thus, they say this one is not allowed for pregnant; and if there is such kind of thinking again, it is a concern that it can influence the future.

**I: How about in religion; may there any prohibition of foods and others to them?**

**P**: Ayii, I didn’t hear this one.

**I: So, to resolve the challenges you told me, what did you do by yourselves?**

**P**: To solve these challenges, if we face individuals in person who that have that kind of thinking, we convince them about the nutrition of mothers, and adolescent girls; especially about the need of eating well for the pregnant mothers considering the life of their babies and themselves. And also we teach about that thinking of good food for husband and not eating without husband has to be changed and the woman has to eat even being alone; and also that of thinking only for children but not about herself, has to be changed. We also invite the husband and we teach about these issues if we suspect or hear something about him.

**I: What do you think about how it would be better done for the future?**

**P**: Uhhh?

**I: For the future, how would it be better done?**

**P**: Is that there in the challenge?

**I: Yes.**

**P**: At the nutrition… (Long silence), if a change is to come in nutrition the education given by the federal ministry of health, it has to be given at family level so that it would better bring change for the future.

**Section 5: multi-sectoral collaboration to improve maternal and adolescent girls’ nutrition**

**I: Do you feel it is necessary for your health post to work with other sectors/institutions to address maternal and adolescent girls’ nutrition?**

**P**: Yes!

**I: Why do you think that?**

**P**: Why I say it necessary is that, for example, if we take agriculture, thus, if there is one pregnant mother and if we need to teach her about the balanced diet to eat; that food product has to be present at her home; and if these food products to be produced, there has to be present that agricultural professional or expert to give that advice. Thus, agriculture and health are never separable. If we take that education also, if we need to teach the mother, either there has to be present educated child or she has to be educated one; if she is able to write, the words you tell her; she can capture it very easily. If we say to the water resource also, there has to be present water; each household has to own water source; it is said by the government that there has to be present one alternative water source for each household; then for the nutrition as we said it; it can harvest earlier if there is water; but in the absence of water, either it will buy or not; thus, that nutrition will be lower. Thus, working together in collaboration is fruitful; and we are still working at some of it.

**I: With whom are you working currently?**

**P**: We are working; for example, with agriculture, we work together; for example, if we are to a mother about her nutrition, if she says that she has not irrigation, it is identified what the problem is that, either if it is due to inappropriate use of water or lack of alternative water source; thus, it is resolved accordingly and she is given then.

**I: Have you ever done such kind of solution?**

**P**: Oh! Yes.

**I: For how many mothers?**

**P**: Ayii, I don’t know their number.

**I: With which other sectors do you think is necessary to work together in addition to those you mentioned above?**

**P**: It is necessary to work together with the kebelle leaders.

**I: Why?**

**P**: What we do is the professional perspective; but that of about mobilization and that of nutrition to be effective in each home, it has to be followed by the propaganda and politics works; thus, working with them is fruitful.

**I: With whom else can you work together?**

**P**: That is it; others are not present here. (She laughed)

**I: Are there nutrition related platforms here; and have you ever participated there?**

**P**: Where?

**I: I mean, are there nutrition related platforms or sessions undergone here?**

**P**: Yes there are.

**I: Example?**

**P**: By the world vision, there are mothers included there; so, we participated there for about three times; in 2009 (EC counting).

**I: So, what was done there?**

**P**: It was about the nutrition; that we have problem; we have to improve for the future; because there is 40 % of that under nutrition currently; based on this, it was with the agriculture that we joined, thus, we got information about what has to be done at agriculture and at health too.

**I: Where else did you participate?**

**P**: About nutrition?

**I: Yes?**

**P**: No, we didn’t except it. We participated all of us at that one (save the children).

**I: So, was that productive for you?**

**P**: Wow! Yes it is productive; it is good to build capacity; learning in each time is good as you hear what is renewed in every time.

**I: What do you think to be done more than this then?**

**P**: What can be done more than this is that, what is now is that if it is to be done a lot at adolescence nutrition, it has to start from the person who works at higher level about nutrition; it has to have its own something from the higher level down to the ground; if it is to be productive for the future.

**Section 6: Other intervention that influence adolescent and maternal nutrition and health outcomes**

**I: Do you think if there is early marriage for a female, may it have impact to the nutrition of the female?**

**P:** Yes it has; if one female marries at her below-age, then, she will deliver at her early age; if she gives birth; her nutrition will not be to the fullest, it will be interrupted as her thinking is immature at that period; thus, it has influence that let her be in under nutrition.

**I: May there early marriage here?**

**P**: There were to marry; but we saved them.

**I: What do you mean, we saved them?**

**P**: What I mean is that, it is being tried that early marriage; but due to the direct evidence that is obtained via the kebelle women affairs and those development armies; that the head of the women affairs together with the woreda, they communicated; and then, two females are saved currently.

**I: How about before them; was there who already marred?**

**P**: Previously, there might be.

**I: What is your role in this early marriage prevention; what o you do?**

**P**: We give the information about not early marriage to be undergone; as if there is below-age marriage, there will be below-age pregnancy; then difficult delivery; and also fistula can come next; so, we teach in detail these are harmful things females’ age; and we work it together with them.

**I: So, how do you see this program; is that successful or still it has flaws?**

**P**: Utterly, that below-age marriage has never to be present as we know that what damages it has to the health of female; yet here, it needs working in collaboration from higher to lower levels; because, a female can be taken being masked by; but at this time there is registration of the vital events; which then, if she is to marry, it is checked at that registration when was she born; thus currently, it is going a bit easy to control; but previously, it was very difficult.

**I: What are the reasons that it is going easy to control that early marriage currently?**

**P**: Currently there is awareness; it is also given education regularly about not to undergo below-age marriage; in addition, if a girl is to marry she has to be registered in the vital event registration and also has to have marriage written agreement; otherwise, she has not to marry; she can’t get back later to be registered or else. Thus, when she is registered, it can be even identified with your naked eyes that how old she is; thus, this is the reason.

**I: So, do you think it is successful?**

**P**: Yes, it is successful; but it has gone sustainably for the future too.

**I: Is there any interruption done by the community to let it be less successful?**

**P**: What the community does is that, at some conditions, there are, who lay a lying; they calculate the age to be at eighteen; and they tell you that she was born on this date or like that; she was born in that calendar; she was there like that they talk. Thus, even witnessing by lying is crime; so, they have to be let back by giving them information.

**I: Do we have other opportunities to work more than this for better success?**

**P**: The opportunities that I can say we have are that, the time is so good; that there is registration for those who are born, married, divorced and died; so, if we register for those who are born, it will be known when was he born; then, it is to know the age by calculating when was born; thus, this has to be strengthened and continued for the future; so then it can abandon not only below-age marriage but also below age-pregnancy o f females; it has to be strengthened; and it also needs of all participation.

**I: Maybe, in the case of birth spacing, may you have any idea how it is going on?**

**P**: On birth spacing, it is being done on it; and birth spacing, one of the killers of mothers is that of narrow birth space; thus, though we may not know it in detail; but it is above a family panning method that can contribute to above 35% of maternal death reduction; thus, base on this they are educated also to use family planning; then there are who use family planning; and also present those who don’t use. Thus, it is needed to work, giving focus at those who don’t use.

**I: Is education being given about it; who gives then?**

**P**: Yes it is given; here, we give that education; because, family planning is one of the eighteen components; thus, education is also given by the development armies in each villages too; yet, that education has to be continued; because, if here is no family planning, there will be giving birth to baby over baby (to say with little space), if so again, there will be present maternal death and diseases too.

**Section 7: additional remarks**

**I: What lessons did you learn from the interventions that intend to improve maternal and adolescent nutrition at your Kebelle level?**

**P**: Is that of the current one?

**I: Yes, about all which are done until then?**

**P**: From what we have been doing, that we say it has a bit change is, that of nutrition; it has a bit change; for example in the case of awareness bring it pregnant or lactating mother, even about to eat additional meal, there had not been awareness; but now, at least she knows that she has to get additional meals. Even though there may present at eating the balanced diet or not, but there is a bit change at eating additional meals. Again at the pregnant mothers, there is a change about they have to have the pre-delivery follow ups; and that of at delivery works, I have already mentioned it earlier; thus, this is it.

**I: Very nice; how about at the multi-sectoral collaboration, is there any lesson that you learned at working together?**

**P:** Okay, at working together, it is when you work together that the work can be productive as I mentioned it earlier; being it with agriculture, water resources, education, and with those what we call them kebelle leaders, all these totally; it is needed to work together; because, if we need to improve the nutrition of one household, there has to be present the at least the food at home; thus otherwise, if there is no the food, telling them to eat could be a bit heavy; and even, as far as we talk base o what is available at their homes; thus, that question of what is available at home, has to be answered.

**I: If we get back to this woreda having all your recommendations to improve the services, what opportunities can we have then?**

**P**: Okay, what we call them opportunities are, to work like this for the future; then our area is conducive area for agricultural works as I said it earlier; thus here, if we introduce all the things that can be done at agriculture, and also if we identify those barriers of nutrition of the community and if we improve the awareness of the community, being it in group or individually; so, our nutrition can be improved.

**I: Thank you, if any idea left to be raised, let me give you he chance?**

**P**: Okay then, the thing that I can raise about nutrition is that, we mainly talked giving focus to those children less than two years of age; but about the nutrition that has to be considered for the future is that, we are saying female; so if female feeds well, thus considering the baby that she will give birth, we are saying she will give birth to healthy baby; but because we have to give it focus; thus, for the next we have to give focus also on those male children.

**I: Which age group of males’ children do you mean, to be given them focus?**

**P**: For example now, those up to 18 years are so called children; but what is here at them is that, at their nutrition there is problem; there is also beyond their capacity work; thus in relation with this, if we let him work beyond his capacity, and if we don’t correct his nutrition too, then in his future of his nutrition, it will leave to him a sequel. So, in our works of the adolescents, we have to work including them; though we may not go beyond that age.

**I: Thank you very much; it was a learning lesson for me; I am done here then.**

**P**: Okay then, thank you too; that you come all from Mekelle University to improve the nutrition of this kebelle, you came here sacrificing a lot; so, I thank you on behalf of the kebelle. Thank you.

**Summary (home take messages)**

**Section 1: Common maternal (pregnant women, lactating women and adolescent girls) nutrition problems in the community.**

Around 178 mothers (pregnant and lactating) are measured MUAC in each month as per the keblelle; form these, around 42 of them are endangered by under-nutrition problem.

**Section 2: Nutrition priorities in the health post**

As to the priorities we do, there is nothing that we do more than that of delivery; thus, the mother has to deliver at health center, the baby not to be hurt, we work at it giving priority.

**Section 3: Nutrition interventions that improve adolescent and maternal health**

If one mother is to improve here diet, she has to use vegetable gardening like those of vegetables; and thus, this is being done at the kebelle as the area is conducive for irrigation.

**Section 4: Implementation challenges and community factors affecting access to nutrition interventions**

One important challenge is that of adolescent females who they would have brought better change at their nutrition; and also they would have convinced at least for their family; but it has not been done at them yet; and even utterly, it is those adolescent that they will deliver and be pregnant for the future; but not done at them yet.

**Section 5: multi-sectoral collaboration to improve maternal and adolescent girls’ nutrition**

Working together in collaboration is fruitful; for example, if we take agriculture, thus, if there is one pregnant mother and if we need to teach her about the balanced diet to eat; that food product has to be present at her home; and if these food products to be produced, there has to be present that agricultural professional or expert to give that advice.

**Section 6: Other intervention that influence adolescent and maternal nutrition and health outcomes**

What the community does is that, at some conditions, there are, who lay a lying; they calculate the age to be at eighteen; and they tell you that she was born on this date or like that; she was born in that calendar; she was there like that they talk. Thus, even witnessing by lying is crime; so, they have to be let back by giving them information.

**Section 7: additional remarks**

For the future, we have to give focus also on the nutrition of those male children aged up to 18 years of age; because, if we let them work beyond their capacity, and if we don’t correct their nutrition too, then in their future nutrition, it will leave to them a sequel.
