# Supplementary material for: Barriers to utilize nutrition interventions among lactating women in rural communities of Tigray, northern Ethiopia: An exploratory study
Source: PLoS One. 2021 Apr 30;16(4):e0250696. doi: 10.1371/journal.pone.0250696 (PMC8087028; doi:10.1371/journal.pone.0250696)
Supplement: S2 File — (ZIP) [file pone.0250696.s002.zip › S2_File.Doc/Community level Key informants/132_IDI_Agriculture Extension Worker_Hakfen Kebele_Medebay Zana Woreda.docx]

## Medebay Zana Woreda_Agriculture Extension Worker KII

| **Introduction:**  Hello, my name is **Dejen Yemane**. I am from Mekelle University. Thank you for taking the time to speak with me today. We are doing research on the factors that influence the nutrition of mothers and adolescents in collaboration with the Regional Health Bureau and UNICEF. Your participation is very valuable. The things that you tell us will be used to improve nutrition programs and services for women in the region and the country. We will not share your names when we report our results.  However, I will record the discussion so that I can capture all the ideas that are shared. I have several questions to ask you that I have prepared in advance, and I will ask you to say what you think about each question. The interview will last for 1:30 -2:00 hours. Do you have any questions before we begin? If you think of any questions as we proceed, please feel free to let me know. If it is all right with you, I will turn on the tape recorder now.  **Section A: Interview details**   1. Zone: **Central** 2. Woreda: **Medebay Zana** 3. Kebele: **Hakfen** 4. Name of key informant: **B Teshome/H** 5. Institution of key informant: **Hakfen Kebelle Leader/Agricultural Extension Worker** 6. Interviewer name: **Dejen Yemane** 7. Date of interview: **November 21, 2017** 8. Interview start time: _________________________ 9. Interview end time: __________________________ |
| --- |
| **Section B: Interviewee professional information**   1. Gender    1. **Female**    2. Male 2. Age: **30/25** yrs 3. Highest level of completed education.    1. No formal education    2. Primary education    3. High school    4. **College education**    5. Bachelor degree    6. Master’s degree 4. Current job/position: **Delegate of Hakfen Kebelle/Agricultural Extension Worker at Hakfen Kebelle** 5. How long have you been in the current job/position?    1. ______ Months    2. **One month/Three** Years |

**N.B.:** Mr. B, the delegate of Hakfen Kebelle leader, is a loan officer at Medebay Zana Dedebit Micro Finance office. Once I started an interview with Mr. B(B in the document), while he was asking me to withdraw from the interview because he is not aware about the activities done in the Kebelle, there was a coincidence that Mis. H (H in the document), the Agricultural Extension Worker at Hakfen Kebelle interrupted the interview she had with Mr. Abate due to language barrier. Then not to miss especially Mis. H, I used to interview her while maintaining Mr. B to complement to each other in this interview.

**Section 1: Common maternal (pregnant women, lactating women and adolescent girls) nutrition problems in the community.**

**Interviewer: In your opinion, what are the common nutrition problems in the community for women? What about for adolescent girls?**

**Participant (B):** the commonest problem in rural area is lack of sanitation and what you eat in the morning is repeated in the dinner. Secondly you cannot get food that you need. For example, whether you like it or not you eat the same food day and night, and this could be the main problem for pregnant and lactating women. Especially in the care given to mothers, as a Woreda we have enough ambulances and the ambulances are giving 24-hour services to mothers at risk. In addition, there is ANC every three months’ time. We have also health center at kebelle level. During delivery and when a child faces any problem she/he will stay at operation room, then once the health status of the child is known then s/he will be returned to his mother. Then the child will be further followed by WDAs and WDAs will be responsible to identify the number of pregnant women in every village (ጎጥ), whom is following that pregnant women and when does a pregnant woman will deliver. So, they give special attention to institutional delivery.

**Interviewer: you told me that there is a problem in feeding balanced diet and sanitation practices, what are the reasons for such problems?**

**Participant (B):** when I say sanitation, especially this time we have 4 health posts in our kebelle. Latrine is constructed in each station and we can say that there is adequate sanitation. Especially our kebelle has better sanitation because of its proximity to the urban area. But in rural areas there might be utilization problems. Since they are farmers, even they have at hand they don’t invest on food and keep personal hygiene rather they prefer to save the money for wedding and memorial (ተስካር) ceremonies while they decay. Though our kebelle is better in different developmental activities, but there is a problem in the utilization and hygienic handling. Though it is difficult to change feeding balanced food, it is possible to change the sanitation practices. Currently utilization of ITN and others are becoming changed due to time. Previously the community was not using ITN and as a result they were bitten by mosquitos that lead them to disease. Still there are households who don’t properly utilize ITN, they use it for carrying straw and other purposes. Last time we burned the ITNs that are used for other purposes. This is to mean they don’t give for their health security.

**Interviewer: let me ask you the first question again, what are the common nutrition problems among pregnant/lactating women and adolescent girls?**

**Participant (B):** yes, regarding nutrition we have an expert on how to prepare enriched flour at Woreda level. Her name is Kiros, she prepares enriched flour and sell it 40 birrs per KG. In our kebelle, we don’t have stunting due to shortage of food. Stunting is observed in lowlands and mothers who don’t get enough care. Here we do have adequate health facilities and they check hemoglobin level. There are few women who face anemia during pregnancy and they get special follow up. Once she delivers health provider weigh the new-borne and since their weight is clear there is no stunting in our kebelle.

**Interviewer: What about wasting?**

**Participant (B):** there might be wasting. Because there are malnourished children referred to health facilities, feeding programs to get FAFA. If a child loses weight s/he will be given FAFA to recover from his/her weight loss.

**Where do you think is wasting common, in male or female, Muslim or Christian, rich or poor?**

**Participant (B):** it is most common in Christians. But there is no difference among rich and poor households. For example, in rural areas there are many problems that occur in rich households.

**Interviewer: Why in rich households?**

**Participant (B):** the reason why in rich household could be food might not be absorbed to your body. There are children physically good while they did not get adequate food. Except the women, children can accept any food unless you face food shortage. But in mothers, for example, a rich mother gave still birth because she was anemic. The child was wrongly positioned, and the mother followed, especially after 8 months she was followed every week and later she was admitted in hospital for further follow up. Though she is anemic the mother is still fine. So, as we have said, stunting occurs in both rich and poor households. But mostly it happens in poor households. There are people who could not get adequate food and cloth and there is probability of dying of cold exposure. Then this will expose to unnecessary diseases like stunting.

**Participant (H):** wasting in children and women is problem of our community. But stunting is not common. Whereas, wasting and anemia is common in pregnant and lactating women.

**Interviewer: where do you think is wasting and anemia very common?**

**Participant (H):** it is common in poor households. There is change from time to time on nutrition especially in feeding vegetables, and preparing enriched flour. But the poor households are supported like FAFA and other Aids though it is not complete.

**Interviewer: What about in children? Is wasting common in female or male?**

**Participant (H):** it is common in male than female.

**Interviewer: what could be the possible reason?**

**Participant (H):** this could be due to inadequate food of the mothers. Right after mothers conceive they do not get adequate food and children are not properly fed and this leads to wasting.

**Interviewer: if you don’t have additional idea, do you think micronutrient deficiencies such as night blindness and goiter are common in our community?**

**Participant (H):** though it is not prevalent there is goiter. There are individuals who removed their goiter surgically. The good thing is people go to health facility early and remove it. Regarding night blindness, it is not that much.

**Interviewer: Is a situation when the community suffers from food insecurity? In what situation do you think this happened? How frequent does it happen? Why do you think that it is frequently happening? How the women/girls do suffer from it, in particular?**

**Participant (H):** yes, there is food shortage. We do have very poor households who can feed themselves for three months unless they are supported by safety net program. So, there is food shortage.

**Interviewer: what could be the reason, because the area is categorized under food secured Woredas?**

**Participant (H):** most of the time poverty is common in females and this is because they don’t have anybody who will plough their farm land and due to poverty, they cannot administer it properly. It is less likely in male headed households. So, stunting, wasting and anemia is common in female headed households.

**Participant (B):** on top of what she has mentioned, the households led by the young generation lack farmlands and they are dependent, and in addition, there will be female headed households due to death of husband. Especially in this kebelle there are females who support their family by taking loans from Dedebit Microfinance and supported by World Vision. For example, there are significant number of households getting monetary support from government and the kebelle if supported by different NGOs. But there are households who don’t change their money to development activities. There is lack of understanding in most of the communities because there are individuals who prefer to eat using the money they get by support instead of changing it to development activities. In our kebelle we have more than 500 individuals supported by safety net program of this are unable to work. Most of the time back warded culture is killing us.

**Interviewer: What kind of back warded cultures are common in this area?**

**Participant (B):** wedding and memorial (ተስካር) ceremonies are one of the back warded cultures. The community invest unnecessary expense for wedding and memorial (ተስካር) parties and this is taking as a step back. Due to unnecessary expenses the family will finish what they have and will suffer of poverty. Then after it becomes difficult to the household to escape poverty. Even they devote borrowed loan to baptism party and later the child will suffer food shortage. This kind of wastage needs special attention.

**Interviewer: what did you do to solve such problem in collaboration with other sectors because it is not one sector’s issue? Because it is related to the food shortage**

**Participant (B):** what we do was, we restricted the number of oxen slaughtered for ceremonies should not exceed one.

**Interviewer: how do you see the implementation?**

**Participant (B):** it was implemented but later it is violated.

**Interviewer: what was the reason for violation of the decision?**

**Participant (B):** The community intentionally violated it. For example, in Tahtay Koraro there was a by law that punish individuals who slaughter more than 2 oxen. If you slaughter more than 2 oxen the community decided not to go to that party so that the food will be wasted. This has worked for almost 4 years since I was there. Then one rich man slaughter 9 oxen for memorial ceremony and he was imprisoned. Later the community violated the by law. In our kebelle, though there is change due to introduction of individual plate, but in the past a dish with excess injera was leading to food wastage and shortage.

**Participant (H):** he has already explained it. In the year 2009/10 E.C food wastage and ceremonies is minimized. Previously there was food wastage that leads to poverty but now it is better.

**Interviewer: Do you think there could be any association between nutrition and occurrence of non-communicable diseases among the women/girls? Why? Are there such diseases in this community?**

**Participant (H):** yes, there are. For example, there is occurrence of AWD but now it is better.

**Interviewer: Does it has any association with food?**

**Participant (H):** hygienic handling of food, and there is also problem in mothers in handling. There is a condition where by one eats without washing hands and feeding in unwashed plates. So, there are diseases transmitted by such things.

**Interviewer: what about non-communicable diseases** **like Diabetes, cancer, hypertension?**

**Participant (H):** it is rarely happened in few people but is not common.

**Interviewer: Is a situation when the community suffers from food insecurity? In what situation do you think this happened? How frequent does it happen? Why do you think that it is frequently happening? How the women/girls do suffer from it, in particular?**

**Participant (H):** when we say food shortage, farmers did not get balanced diet.

**Interviewer: Why? Because everything is in their hand**

**Participant (H):** though they have everything at home they did not utilize it effectively due to lack of awareness. Now there is a change but in the past enriched flour was not known. But now children and women are using enriched flour prepared from legumes and cereals.

**Interviewer: is there drought in this area?**

**Participant (H):** drought is not common.

**Section 3: Barriers to access and utilization of nutrition services**

**Interviewer: what nutritional intervention are in place to improve adolescent and maternal health?**

**Participant (H):** there are nutrition interventions like salad, swiss chard, carrot through home gardening. We teach them even to plant vegetables in their yard by the small jerrycan and every farmer have home gardening. Most of the women are changed through the support from Glimmer of hope and loans. For the out-school adolescent girls, we give nutrition interventions in churches and HEWs also teach and demonstrate about nutrition in their monthly meeting.

**Interviewer: do they get counseling to take extra meal and rest during pregnancy and lactation?**

**Participant (H):**  yes, they get counseling. They have a meeting every 21^st^ date of the month and we and HEWs counsel them to take extra meal and rest. In addition, HEWs, teach them in a home to home basis. As a result, the community is changing its feeding behavior.

**Interviewer: how do you see the iodized salt and ITN utilization?**

**Participant (H):** previously they were using ITN for carrying straw, but now every household is using ITN properly because they understand its importance.

**Interviewer: Do women have an opportunity to rest during pregnancy and lactation?**

**Participant (H):** they are told to take rest and they know the importance, but they have no chance to take rest.

**Interviewer: why?**

**Participant (H):** because they are engaged in the routine activities and some of the women even don’t know that they are affected due to not taking rest. Though they are thought they don’t implement it.

**Participant (B):** she has already explained it. Regarding home gardening, tomato, potato and onion are produced in our kebelle. If you take the rural community, they use hanged dipping material because their water storage is pot. So, there is a change by looking the outcomes of those who care themselves and those who don’t. regarding nutrition, farmer is richer than anybody else because they own hen, whit teff, and goat but they take it to the market and they save their money. For example, laboring mother will go to the farm to clear weeds before her seventh day of delivery. She might not fill the impact at that time but after time she will fill it because her tubes are opened then she will be exposed to cold weather. They only see the temporary benefit and they don’t predict the monetary expenditures once the mother is sick. But in this kebele it is not a problem. Except those who don’t have the rich eats well.

**Interviewer: we promote pregnant and lactating women to take extra meal and at the same time religious leaders promote fasting, how do you see it in your kebelle?**

**Participant (H):** regarding extra meal they don’t use enriched food. They go with the public for cutting grass and clearing weeds and they share the same food with them. Unless otherwise they don’t eat special food because they are pregnant or lactating.

**Participant (B):** if you see in urban areas, be it rich or poor you will slaughter a goat immediately even borrowing from others. Immediately they will take adequate food. in the urban areas they eat animal source food even in fasting time and faith fathers also supports such decisions provided that she will fast after she get recovered. But in rural areas, even though they own goats except in few households the laboring mother will be given only porridge. A laboring mother needs immediately goat and hen. Some due to unavailability and some due to greediness they don’t provide animal source foods to their wives.

**Interviewer: Which of the interventions listed above do you think is most important for pregnant women?**

**Participant (H):** Okay, starting from pregnancy follow up until she delivers, she must have linkage with health facility. In addition, she should take balanced food on time. If a mother was eating 2 meals per day she must eat 5-6 times per day. She must follow ANC and she must be measured on time and know the problem she has. Then she must not be separated from health facilities. Whereas, after delivery she must improve her feeding and if she is anemic she must deliver at health institution. After delivery, as it was said by B, she must use be it goat, egg or milk.

**Interviewer: do pregnant/lactating women use those animal source foods?**

**Participant (H):** yes, they use in few households. It is not that much. This could be the reason why women were wasted and anemic. But now this is improved. They meet with HEWs and if the issue is beyond their capacity, HEWs refer them and if it is of their level they manage it. This time we don’t have women who don’t follow ANC. So, every intervention is equally important.

**Interviewer: what factors can hinder from using these services?**

**Participant (H):** it is back warded thinking. They don’t want to expose their pregnancy. There is unchanged back warded traditional thinking. But now it is becoming changed.

**Intervention: okay can you mention other traditional thinking that can hinder maternal and adolescent nutrition? For example, female is prohibited from drinking tea and eating sweet things, and factors that hinder PNC follow up like prohibiting laboring mother from going out of home before 40 days.**

**Participant (B):** traditional thinking was there in the past. Females were prohibited hot drinks, yoghurt and milk in the past, but this kind of thinking is no more working. Now they have full freedom because the community is changed. the other traditional thinking is that, while you give service there might be death at health facilities. for example, we are struggling to avoid home delivery but still there are women who deliver at home. They associate it with Gods will and they say unless it is God will somebody was died In health institution. The clinical personnel cannot replace life, but s/he can investigate the women’s health status to minimize maternal and child mortality. He can only prevent, rather he cannot avoid it. Regarding the back warded cultures, it is already changed.

**Participant (H):** our faith fathers allow us to eat during pregnancy and so that we will take canon after birth. The grandmothers also say, if a mother is on pain, she should not go out of home. But this time this attitude is changed, and everybody goes to health facility if they see any sign. So, the traditional practices are changed from time to time.

**Interviewer: do pregnant/lactating women and adolescent girls get Targeted supplementary feeding (TSF)?**

**Participant (H):** yes, especially in health aspect they get many services. Especially poor households who have a pregnant woman and with children under two years of age/lactating women do get FAFA, oil. They are also thought and supported to plant swiss chard, carrot and salad in their backyard.

**Interviewer: what kind of Targeted supplementary feeding (TSF) do adolescent girls get?**

**Participant (H):** adolescents get health education because most of the adolescents are in school students. Adolescent girls are informed to report to the kebelle women affairs if there is any abduction/rape. In addition, we teach them to take care of cheating from posit sex and if it happens they should go to health facility for checkup.

**Interviewer: what about for out-school adolescent girls?**

**Participant (H):** out-school adolescent girl will be addressed in their village, because there are women gathering be it for sanitation or other issues. Sometimes we also call meeting and teach them. Currently no one can force adolescents for marriage before checkup, then they are told to check first to know their status.

**Section 3: Perceived needs of women for relevant services during pregnancy, lactation and adolescence**

**Interviewer: What special things should a woman do to stay healthy during pregnancy, lactation and adolescence? For example, Visiting health facilities, Taking extra meals, Taking rest, Taking any pills or supplements, Change in workload or Others**

**Participant (H):** since it is not due to unavailability much work is needed to change their awareness. Preparation of enriched flour (ምጥን) is implemented through grinding a mixture of 12 types of cereals and legumes. This should be the main because it contains many vitamins. Poor women who are supported by the kebelle be in monetary or other kinds are escaping from poverty. But there are few women who did not escape from poverty even they are supported.

**Participant (B):** on top of what she said, most of the supported households did not apply the support to serve its purpose rather they invest it for other temporary things. However, these supports should serve their purpose. We have models who were changed by the support they got from the government or development partners and they come to each kebelle to share their experiences. For example, we have models in kebelle Nefasit. But most of the community cannot be changed even you support them and because of poor utilization.

**Interviewer: Do women in this community typically change their diets when they are pregnant and lactating? Do they eat more food or less food during lactation and pregnancy than non-pregnant women? What foods are recommended for pregnant women? What about breastfeeding women? What foods do pregnant women avoid? What about breastfeeding women? What about the role of husbands?**

**Participant (H):** it is not about availability of food rather women are busy of routine activities. The problem is the attitude. Except engaging herself on routine activities the mother doesn’t care herself rather she cares for her son and daughter. So, she doesn’t change her diet and rather she eats the usual food. except the porridge she doesn’t get additional diet.

**Participant (B):** it is already explained. What is left is the husband role on caring their wives. Most of the time in rural areas except few who care for their wife especially when they conceive most are not aware about what care she needs. Excuse me, this would have been good if it is explained by H (H replied that it is okay). During pregnancy even, their behavior and needs is changed but in rural areas those needs are not fulfilled rather they nag them to go with them to clear weeds and cutting grasses. Due to pregnancy her appetite is not good, and she is not eating well then, she will face nutritional problem and the community is not understanding its future impact on the woman and her baby. In the new generation this are not problems because most of them are 10^th^ grade completed. The government is also struggling to end illiteracy by availing adult learning. Though there is change such problems like not taking food of her interest, happen in most kebelles.

**Interviewer: I have related question, in the past there was gender disparities in diet. Are there gender disparities in women’s diets before pregnancy and during pregnancy, lactation and adolescence.**

**Participant (B):** it is determined based on the interest of the family. There are families who wants to have male and in the other side there are also families who wants to have female. In the past there was culturally gender disparities in diet. For the men they provide good things and female eat the leftovers. This is like eating spoiled food. In rural except the husband women don’t eat spicy food and there are situations women wait until husband comes.

**Interviewer: Do this all still practiced in our community?**

**Participant (B):** in the past it was there, you cannot eat before the husband eats, and when you wait until husband comes the feeding time will go, and this will lead to appendicitis and other health problems but now it is changed. As I have said the female vs male is dependent on the decision of the family but still there is negative attitudes. As you have mentioned, if the husband is not around, you will eat injera with salt though hen sauce is prepared. But this is changed now except in some inclement husbands.

**Section 4: Other interventions that improve pregnant, lactating and adolescent nutrition**

**Interviewer: Have you ever gone for nutrition screening during community health days or routine service delivery? If you went, what was your experience? If you didn’t go, why not? Does the community health days or routine service delivery are considered to be just for children or if they perceive benefits for pregnant/lactating women and adolescent girls as well, and what are those benefits? What are the challenges to attending community health days or accessing the routine service delivery?**

**Participant (H):** in the monthly community health day there is no challenge because we have health post in our kebelle. Because mothers can get the services easily.

**Interviewer: do you think that community health day have benefits for pregnant/lactating women and adolescent girls?**

**Participant (H):** yes, understanding the benefits they are coming to utilize service.

**Interviewer: you told me that there is FAFA supplementation for those critically wasted, so do you think targeted supplementary food for pregnant/lactating women and adolescents has benefits. If not, why not?**

**Participant (H):** yes, it is given because it is important.

**Interviewer: Are pregnant and lactating women beneficiaries of the soft conditionality of the productive safety net program, PSNP (NB: pregnant and lactating women are exempted from the physical works of PSNP)?**

**Participant (H):** if you take our kebelle, if we get letter that confirm a mother is pregnant from health facility, we exempt them from any work until the child celebrate his/her 2^nd^ birth date rather she will care for her child.

**Participant (B):** if she get letter from health facility leave alone safety net we exempt them from water and soil conservation activities. So, we give special attention in this regard.

**Interviewer: pregnant and lactating women are exempted from the physical works of PSNP and water and soil conservation, but she is engaged in heavy works. Do the family exempt them from fetching water, mowing and weeding?**

**Participant (B):** we have said it before. Government is exempting them from physical works starting from the time she conceived until the child is 2 years old, but the family does not exempt them even in some families they don’t wait them until the 12^th^ day. Everybody know physical work will affect both the mother and baby, but they don’t exempt them even they grind until the eve of their delivery date. But the science recommends pregnant women should not engage in heavy works. So, I conclude that though the government exempt them from physical work they are engaged even in heavy work at household and this needs further efforts.

**Participant (H):** most of the issues are explained by B. The main problem is on the husbands, because even the women need to take rest husband force them to do heavy works. There are husbands that don’t understand even their pain. So, it is not yet solved.

**Interviewer: what did you do to solve this problem as a kebelle committee?**

**Participant (B):** pregnant and lactating women are specially followed by HEWs. Pregnant and lactating women are identified, registered and followed by WDAs. the pregnant women also follow ANC at health facility based on their appointment. The problem is you can’t see what is going on in the household. What we do is we exempt them from physical activities and promote institutional delivery, ANC and taking rest.

**Section 5: Understanding perceptions of age at first birth and birth spacing**

**Interviewer: do you think delaying the age at first birth to after 18 is better for the health of the women? How? What other benefits does it have for the women? What about for the baby? Does this delay would have a benefit to the nutritional status of the women? Participant (H):** we can say that there is no early marriage this time. If she is married before 18 years old she is not physically matured, and she can’t carry the fetus and during delivery she could face a problem and to the worst death. Because she is not capable and immature. She could also face excessive bleeding because of the immaturity of the ovary.

**Interviewer: Do you think this message is being promoted in the community? Who are working on it? How do they promote? Can you think of any other opportunities to prevent early marriage?**

**Participant (B):** the risk is because of the early marriage. Risk of early marriage is understood by the community. In our kebelle, except one case in 2009 there is no early marriage. The WDAs know what is going on in the community, which household is preparing for marriage, if the lady is under age we stop it in relation with the schools. Because every adolescent is in school and teachers also mobilize community to send their children to school. It is acceptable by the community except in few households and in those households, it is just to see their children engaged before they die. There is also wrong attitude that says if they are educated they would become prostitute. But overall, early marriage is almost zero in our kebelle.

**Interviewer: How many years do you think the gap should be between successive births for women? Why? What about if shorter than it? What other benefits does it have for the women and the baby? What do you suggest promoting it in a better way?**

**Participant (H):** regarding birth spacing, if there is 5-6 years interval between births the baby can get adequate food, you can fulfill his/her needs and the child can grow well. If there is short interval the children will be stressed and wasted because they will not be adequately breastfed. Secondly, if she gets pregnant at their 6^th^ months, they will get sick or die because they will drink colostrum. In our kebelle the gap between successive births is 3-4 years.

**Interviewer: What other benefits does it have for the women?**

**Participant (H):** the mother will not be stressed, it could lead her to anemia and birth related complications. The main reason for short birth interval was due to unavailability of family planning and disagreement of husbands. But now health center is available in every kebelle and mothers take family planning.

**Interviewer: How do you see the acceptability of family planning by the community and religious leaders?**

**Participant (H):** there is religious impact because they warn us as if we are killing lives. Similarly, old people. The community also perceives long acting family planning leads to infertility.

**Section 6: Understanding communication and information sources**

**Interviewer: What kinds of community conversations or messages discuss women’s and adolescent nutrition in this community? Who provides them, when, how are they communicated?**

**Participant (H):** most of the time women meet monthly at the 21^st^ date of the month and we transmit the messages during those meetings. We also demonstrate how to prepare enriched flour and preparation of porridge. So, most of the information is transmitted on the 21^st^ date of the month. In addition, such messages are transmitted in every village by the HEWs and WDAs.

**Interviewer: What are the most effective/successful activities in changing women’s health behavior? Who, what, when, where, why?**

**Participant (B):** regarding nutrition, we have an expert as we have mentioned before. The community attentively listen messages transmitted by people came from Woreda or Zonal offices.

**Participant (H):** since the community is familiar with us because we meet every day, they ridicule on us rather they accept information given by guests.

**Additional Remarks**

**Interviewer: Do you have any other comments on anything that we have discussed? What lessons have you learnt regarding adolescent and maternal (pregnant, lactating and adolescent girls) nutrition at your level? What lessons have you learnt regarding multi-sectoral coordination of nutrition in this Woreda? What opportunities do exist to promote maternal (pregnant, lactating and adolescent girls) nutrition in this Woreda?**

**Participant:** we have already said it:
