# Supplementary material for: Barriers to utilize nutrition interventions among lactating women in rural communities of Tigray, northern Ethiopia: An exploratory study
Source: PLoS One. 2021 Apr 30;16(4):e0250696. doi: 10.1371/journal.pone.0250696 (PMC8087028; doi:10.1371/journal.pone.0250696)
Supplement: S2 File — (ZIP) [file pone.0250696.s002.zip › S2_File.Doc/Community level Key informants/133-IDI_Religious leader Hakfen kebele_Medebay Zana woreda.docx]

**Day10: 12 /03/2010 E.C**

**Translation: In-depth interview of religious leader**

**Section A: Interview details**

Zone: Central

Woreda: Medebayzana

Kebele: Hakfen

Name of participant: Kes Hafete weld Kasa

Interviewer: G/medhin.B

Date: 12/03/2010 E.C.

Interview start time: 9:20 Pm; local time

Interview end time: 10:45 Pm; local time

|  | Socio demographic information | | | |
| --- | --- | --- | --- | --- |
| **Sex** | **Age** | **Marital status** | **Education level** | **occupation** |
| Male | 48 | Married | Read and write | farmer |

Position: Priest

**Section1: common maternal nutrition**

I: What do women (pregnant and lactating mothers) do to stay healthy in the community?

P: Anyways what I can say, today it is better than the previous years. This is because, in health service and others, the government has removed the backward things, and brought to peace and development. Pregnant woman goes to health facility every month to check the status of her fetus, and follow him if he is breathing and how he positioned like is he on horizontal or not. At the beginning, the community did not accept it well, but now he has adapted it. If she is pregnant women, she goes to health post every month and make checkup. Beyond this, when she arrives to deliver, there are no any women that delivers at home. She directly goes to health facility. So far because of lack of awareness and understanding, women were hided and there were a lot of problems: the baby nay come in malposition and even after delivery, she bleeds. There were a lot of incidents among women. As compare to that time, there is improvement in health in this time.

I: What else do women do beyond visiting health facility? Related to sanitation and nutrition;

P: Regarding the nutrition, if it is not related to shortage of food, they gave them education. If it is pregnant woman, this feeding style, and if she is caring a child, they tell her to feed different types of foods. There is a change, but it not complete. To proceed in a complete way, the economy matters. As we are rural dwellers, we have lack of money in our economy. Thus, if we say women are complete in nutrition, it is simply a false word. Second, there is difference between rural and urban community. We are not able to manage what we have. Even egg, we did not say I have to eat it rather than taking it to the market. We are serious to think that the egg can build me. This is not sill solved, there is low priority given to feeding. Even though it is not complete, it is better than the previous time.

I: What about the adolescent girls do? Both the in school and the out school ones; what do they do to stay healthy? Anything you observe;

P: what is there about them?

I: You can tell me anything you know. What do they do to stay healthy?

P: We do not know what they do and what they do not do. Regarding marriage in underage and normal age,

I: Okay that one has its own section. We will come back to it. Now, it is regarding their health, even if you can compare the current and previous likely of disease condition, and personal hygiene.

P: Regarding hygiene, they are clean. They kept their sanitation because when we see our children that we have born, they always wash and comb their hair; they wash their cloth and keep their personal hygiene. We are not comparable with the young generation. The way we grow is not comparable. Their hygiene is special. To prevent disease, they keep their hygiene. They care themselves. Even we are learning from them. They are also helping us; they wash our clothes too.

I: What are the common nutritional problems in this community for women and adolescents? They could be different disease, for example goiter; or mother with thinness or short stature or else those women encountered.

P: I do not know much about health other than the poverty existed as problem in community.

I: Anything you have observed;

P: We have only a problem of poverty, there is no any problem in health because of the good leadership of our government and because the health facility is expanding. When I compare the current and the previous one, the current is better. If a person gets sick, he will immediately go to health center, and he will get the necessary medication. There is no any problem seen in it.

I: What about related to nutrition? Is everyone gets heavy? Are all mother fat?

P: How can they become fat?

I: What about wasting? Are there thin women?

P: Yes there are.

I: How does it occur? What do the people say?

P: Because of short of economy; our income is not enough. Second, even when our income is low, there is lack awareness/understanding on how to use it properly. The primary reason is shortage in economy. You cannot do what you want, in rural, we have shortage of money. In addition to our shortage, we educate our children and we have other activities; we buy “Asbeza’ (food items) and there is also ‘Tihlo’... We produce cereals in a year and we are losing it. It cannot cover all things; we have many expenses’ Thus, we are facing a problem. Therefore, yes there many mothers who are thin and short, because, they care and educate children. While they are in problem, they tried to care their children well. They live in bad life. Your parents are not like you, because you are educated. For example if you look at me, I am sending my children to school, even if I am in difficulty. What should I do, I eat what I got, I cannot choose. My thought is to let my child learn.

I: Are there anything which make women at risk of malnutrition? May be because they do not eat food as they are overloaded or because of our culture they give priority to husband or other related things that put women at risk of malnutrition?

P: They do not have any special thing. They are not food. Even from the family, there is no any who suffers like her. If you say “why” she is head of household and I am her husband. She will say I will not eat before my husband. She will say I will not eat if my children did not eat. It is like the hen in which she gives to her chicken before she eats. The mother is suffering beyond that because she cares the whole family as she is head of the household. Whether the mother eats or not, the husband will pass by and get sleep. The child may not understand her, but the husband should do as she is a head of the household and she may suffer. I should say “my wife is suffering and she should not suffer” I myself did not mean it; I do not know the others. But, in majority, in our neighbors it is similar.

I: Are there many women who suffer from this (wasting and thinness) in your community, Hakfen kebele? May be pregnant and lactating women, those who do not have farmland or those who do not have husband;

P: Leave it that, even those who have farmland, they are suffering from shortage of food. But, many of them we can conclude they are fine. There are few that wasted. The determinant one is not ownership of farmland, it is about mentality. Even the one who does not have farmland may have better life because of his mentality. These who have poor understanding have poor life. Many of the people are good.

I: What about any problem among women related with anemia, night blindness and goiter?

P: Here, I do not know much about it.

I: Do you know what goiter is?

P: Yes, it swelled on the neck; it is related with salt.

I: With whom did you see it?

P: It is there in the town; we see when are passing through.

I: I your opinion, what do you think is the reason?

P: I do not know how it is comes but, we are told to use iodized salt. It heals it. They educate us like that, because iodine salt prevents goiter. They also tell us: iodine salt is good for children; it makes them active. Because of this, we use it.

I: Do people use the iodized salt?

P: I cannot say many people use it, because I do not want talk false. Anyway few people use it, but majority of them do not. Rarely, there is goiter and use iodine salt. It can prevent it.

I: Are there diet related non-communicable diseases among women and adolescents? Like women with hypertension or diabetes;

P: I do not know this; I mean I did not hear about it.

I: Okay, no problem!

I: Do you have knowhow about the diseases? Hypertension, diabetes; or ever heard about it

P: Yes indeed. Even there is a guy in our neighbor with hypertension. Regarding diabetes, I heard its name in chat.

I: What do you think is the reason for these diseases?

P: I do not this how it comes. What I know is there are individuals with hypertension here and in the town; but, I do not how it is caused.

I: How do you see the height and age of the women? Are there women who do not increase their height proportional to their age? Or are there youths whose is age 20 years but their height is short? We call it stunting.

P: I did see in our kebele. But, I saw in the market. She is a woman, and she is very short, below the knee; But, her face old. The same is occurred among man. But, in our kebele, I have not seen till now.

I: Do all women are tall?

P: They are fine, medium.

I: They should not be too short, rather, shorter than their counter parts.

P: The short is known; the one shorter than his people in community. I did see in our kebele.

I: You have told me that you have seen stunted women in the town. What do you think is the reason?

P: I do not know this.

I: Sometimes people relate it with nutrition and sometimes people relate it with nature and call this it is natural; from this aspect what do you think is the reason for being short (stunting)?

P: I category this as it is because of nature, a power of God.

I: This is a nice answer, it is good sand you have.

P: Every one cannot be short with lack of food; it is power of God that makes him hort.

I: What about in relation to weight? Some women might have low weight while their age is old; how is this here? Do they increase proportionally?

P: if I am to talk about weight, “when do I measure them?” while one person is small, he might have heavy bones, on the other side, when someone is tall, he might have light bones. I have not measure them. The one who measures know; I have not been there.

I: Yes, but, there are many people who are physical small, and call it even “the small one” this way is what I want, right? Even if it is difficult for you to identify from women, it can be easy for you to identify the problem from adolescent girls.

P: Yes there are, they cannot be equal; they are some who are huge and energetic and the others are small naturally.

I: What do you think its relation with food? Some say it is because of food, and some call natural because if the father small, the child can be small. On the other even if he is born normal he may be small if he is not well feed. How do you see these things?

P: That one is right. If someone uses food, he will be fat and energetic; this is what I believe. If he gets variety of food, if he eats enough food, and if his health is kept, he will be huge and he will be energetic, I believe in it. We are looking at our children, are they fine and are they better than others; we talk about it because the feeding habit and hygiene differs from one to other.

I: Do we have mother this problem? If so, is it because of shortage of food or lack of awareness on how to prepare and serve it?

P: The problem is because of shortage of food; if there is food, there is no one who does not know how to use it beautifully. It is said “I will learn from my neighbor” a woman said it. So if there is food, he can eat varieties including vegetable. The problem is he does not have enough budgets. He can’t eat all at a time having in mind the problem he will face tomorrow. What I have mentioned before regarding the completeness is just to the small food we have at home. If I have one Injerra, I eat it barely than think “what if I eat half of it by adding other varieties to serve balance diet.” We do not think this way. But our major problem is lack of food.

I: What about any problem related to food insecurity in this kebele? Is there safety net?

P: Yes. There are many people who are involved in safety net program.

I: Do we have women who suffer from overweight?

P: In the rural, there are not.

I: In your opinion, what do you think is the cause of overweight?

P: it can be from food or other; from radio, I heard that do not eat sweet foods like sugar, oil and coffee; over time it may cause problem like it blocks blood follow and can be clotted. I have not been learnt it; I just heard it.

I: In what situation women suffers from shortage of food? It may be good it time of autumn;

P: You know what, when you talk about food, now in autumn (harvesting time) you may get food in every plate. But, how far it will stay; it cannot stay from year to year. The problem is seen mainly during summer (July, August to September).

But now, even though they get food for two months, how far they will stay with that food for the whole year is the question. Because they (women) worry about the coming months, as she does not have food for the whole year. You cannot conclude it as enough. Women are counting months. She will not be stable with the food she collected in autumn, five, six quintal of cereals which possible can stay only two months. They thought of for the whole year. The worst is from July to September and October.

I: What else or other problems do women in this community suffer from? Other than the shortage of food;

P: There is no thing.

**Section two: Barriers to access and utilization of nutrition services**

I: What kind of nutritional interventions are in place to improve health of pregnant women in this kebele? E.g. do women advised to visit health facility and services during pregnancy?

P: Yes they get. But, I do not exactly what the services are. But they are advised what to eat during pregnancy, and after delivery, they are told to eat and during soup, a mixture of foods from bean and others. Feed the baby this way; body building foods and vegetables are important. If a baby is to born and grow, you have to eat well; the women knew it its details. The baby should be given body building foods; it is said like this. But, even though they lead us, we did not go more as we rural community we usually are hurry for other duties. Even though we have shortage of food, the care and follow-up we give to child is limited.

I: In this regard, do pregnant women get advice to visit health facility? Is it promoted widely at different places like church and meeting place?

P: Even the community himself tells to go health facility for examination. Previous, we do not know whether it have advantage or disadvantage; we just cover it and think it is natural, and say I will not take my wife to anywhere. But now, only those who used it knows; there are people who advice their wife to visit health facility. Besides, if she is near to give birth, they call for ambulance and take her to health facility. They care very much and bring her back to home.

I: Do you think women receive advice on the need to get extra meal during pregnancy and lactation?

P: Is it for them?

I: Yes.

P: Why do they tell me? Had I been there, I can answer it. They hold it for themselves. Therefore I do not know.

I: What about its importance, how do you see it?

P: This is for good; for feeding; to keep health; we saw it as good.

I: How do you see this in terms of religion if a woman be it regnant or lactating, is advised to eat extra meal? May if it is during fasting?

P: if the woman is fine, she has to fast; the rule will not allow her. But, if it leads her to danger or if she is going to die because of fasting, it is a sin. So, she eats at that time. When she became okay, she will fast and bow for it. She will be given penitence.

I: Do they get education? For example, do you education to women for whom you became a God father?

P: There is incident. For example, my wife was operated and she was eating at that time. The same was in other mothers, and I gave them penitence. There are women who become operated, and if she dies because of fasting, it is big problem. One for the woman, she is losing her life and same is for the baby. Then she eats and drinks what she gets. And later, she will receive her punishment.

I: What about for lactating mother? Does she have such options?

P: If she is good and her health is fine, it will not be allowed for her. It is possible only if she is endangered. Because she is lactating and caring the baby, there is no way she will be waived. If it does, it is about pushing the rule.

I: Are women getting counseling for food diversification during pregnancy and lactation by health professional or others?

P: Yes. The HEWs form mothers to use different foods, and eat body building food. They give the education; the failure is because of our laziness and weakness.

I: Can you list me as an example what exactly they tell to mother?

P: Here, it is not me who took the training. They are the mothers who have got the education; I only heard the information when they talk it others. I cannot exactly recognize it. They can answer this question, but I cannot answer.

I: At mass or at meeting, is there an education on maternal nutrition? Because, the food they prepare will not be eaten by only mothers but also by the family members. It is useful to the community. So, was it given in a forum where male have been involved? And even to religious leaders;

P: We do not give it attention.

I: Do you remember the last day where education is given on maternal nutrition?

P: No. even when they gave education, we did not give it attention?

I: Why do you think people are not giving attention? Is it because it has no advantage or is it the way they give the education is not attractive?

P: No it is not. It is the motivation; they say: it is not because we do not know how to feed; we know it. So, this is because of backwardness. Had we got enough food, we could have prepared balanced diet. So the reason why we do not give attention is because we know it.

I: You have mentioned me that it is not common to use iodized salt? Why do you think is there reason? Is it because of access or cost or taste?

P: I only know my family; how am I supposed to talk about other?

I: Okay! Can you get iodized salt everywhere?

P: Until now, yes it was.

I: Do you use iodine at home

P: No; previously we were using.

I: what about the other community?

P: There are some who use and there are some who do not. This is because of the habit. Second, the community is healthy. It is not about non acceptance, it is just because of the culture. But many of the community do not use iodine salt.

I: Do they inform you how to use it? When add to the salt, when to get it down; it is known by the community?

P: After the Tsebhi (Stew) is cooked and is put down, the salt is added.

I: Where did you get this lesson?

P: Here, in the health post.

I: Are women/ adolescents getting advice on home gardening? Is there a woman with home garden in your kushet?

P: We are given education the importance of vegetables, but I have not seen a women with home garden.

I: Who provide the education? Is it from the agriculture or from the health?

P: The health extension workers; the lesson is on the need to seed and plant vegetables, like potato and other. You can also take seeds for vegetation. This is all given by the agriculture.

I: How do you see the participation of adolescents here in home gardening; because girls are good to help their mothers? So if they are active at gardening or irrigation, they could bring change in family nutrition. So, how do you see their engagement in home gardening?

P: In their personal development, I do not see who is actively working. But in the change to prepare food, they buy either tomato or vegetables, like spinach, cabbage and salad (lettuce) or pepper when they get money. They are buying these things because they have the information from the modernization the saw. It is based that they are doing these things. But, I did see a girl gardening vegetables either at irrigation site or around home by bringing water. Even there is no water source for irrigation in our locality.

I: What is else intervention is here related to this.

P: No, even there are no vegetables or ‘Duba’

I: Do people use vegetables? Either from irrigation or from market; we know the resource is with the farmer; be it vegetables, cereals, honey, butter and others too, but we mothers taking these things to market. Beyond getting an income do mothers use these things at home?

P: No, they do not use. Be it honey, butter or vegetable, they take it to market to get money, and solve the temporary problem they have. They think of their children, and favors to their children. No single mother will say “I will eat this and that, and keep my health well, and refresh my mind” rather, while she is suffering and get emaciated, she will think how her children will get good position in the future. Even the simplest we can do is to eat egg but, we do not, because the mother is taking it to market and buy other thing. The reason is: one, lack of awareness; the second and critical one is poverty. You have asked if I am using vegetables because the resource is with us. Yes, the resource is we us however we do not use it. For you the government is giving salary every month. But we produce cereals, and these are not enough for children and food for ourselves. Thus, we face shortage. We have no experience to trade, and there is no job opportunity where we can work as labor. Therefore, we have poverty.

I: Are women involved in safety net program?

P: Yes.

I: How supportive is it? Does it bring change in their nutrition?

P: There, the work is hard, but because they are filling their abdomen daily, so we can call it good.

I: Are women getting advice on water, sanitation and hygiene services?

P: Yes, they tell us clean our compound, separate animals’ house, wash utensils. When you wake up in the morning, you have to wash your hand and face. They educate us such things.

I: How about regarding water sanitation?

P: They came and follow the water pump; to prevent from parasites, they treat it using chemical. The community is not such much changed, but, the education they give to us is good.

I: Who provide the education?

P: Regarding the water, there are experts from the water resource. The sanitation and hygiene is given by health extension workers.

I: Do you think the community have toilet? Okay, do o have toilet?

P: I do not have because this area is rocky and it became undoable. I myself want to have toilet, because we could use it when it is day, when there guest, but I can’t construct, because the land is rocky. They told me to build temporary one, but I did not. This is because it became undoable for me.

I: what about other community? Do they have a toilet?

P: Yes.

I: Do they utilize the toilet? Some people built toilet for sack of demonstration, but actually they do not use.

P: Yes they do; they utilize it. Because, they dag it deep and built it very well. Especial during summer, when there is cold, they utilize in it. I believe on the idea, but it become undoable for me

I: Do people put water for hand washing in the toilet including soap or ash?

P: They educate us frequently to dig and build toilet and to wash our hands after using toilet, but it is practiced by the community.

I: How common is malaria in this community?

P: Yes, there is malaria, especially on September.

I: How do see the risk of women to be affected by malaria? Are there many women affected?

P: it is not such much, but there few who get affected.

I: Is ITN distributed to the community?

P: Yes.

I: Are women or girls advised to use ITN? Why?

P: Yes, we are told to extend and hang it up the whole night to prevent from mosquito bite.

I: Some time people do not utilize ITN as dislike its smell. Are there situations where they use ITN to cover cereal and carry hey?

P: Yes, but it is when it get old. If is new, they used for themselves, especially if the women is pregnant of lactating, she sleeps under it.

I: Is it sufficient? The number of ITN given;

P: It is given for household; if some has family size of seven or eight, they are given three INTs, and if household has less than seven family members, they are given two ITNs. But, it may not enough to whole family.

I: Are women/ girls given deworming services, inform of tablet? This is in addition the chemical treatment of water by water resource office. Keep in mind that the drug different from the drug given for prevention of trachoma.

P: I have never seen it. I do not know; they may get when I am out home.

I: You have told me that there are women who are emaciated, and suffer from shortage of food. Is there supplementary feeding, like fafa (corn) and oil given for women/adolescents?

P: Now, there is no; but previously, these were in place.

I: When? Five or ten year back;

P: Wow, It had been more than five or six years. But now, I do not see anything; I do not know if they are taking now.

I: In your opinion, do you think there are women who are in need of supplementary feeding?

P: Yes, they need. If you give us, if our government gives us, we are happy.

I: It is not in this way.

P: He laughed very much.

I: What I mean is, are there women who very emaciated and are in need of fafa (corn) or plump net? Normally, this is not something to be given to all women in all area. It is given in areas where there is food insecurity and as result there is malnutrition like thinness and being short among mothers, both pregnant and lactating, and children.

P: Yes, and they are ample. There are mothers who have twins, and the babies are crying because of hunger. Beside to the mother’s milk, it would be good if they get such support. So far, the very small children were taking an aid. I forget its name. At that time, after eating that one, they become fat. So, it is good if that one come to our community. Even the mother could sleep without bothering.

I: Which of the interventions mentioned above do you thing is most important for women/ adolescents? (Advice to visit health facility, safety net, advice to use iodized salt, ITN use, WASH and other)

P: Giving birth at health facility is the one that brought change, because we have never heard woman dying during delivery. When woman starts labor, we immediately call for ambulance; it comes and takes her to health facility. Then they follow them attentively and assist them to deliver. If it can be managed her, she stays in but, if they cannot manage it, they refer her immediately. There is good follow up for pregnant women.

I: What are the barriers in the implementation of these services? (Counseling for food diversification, extra meal, checkup and services, ITN, supplementary feeding and advice on WASH)

P: All are not equal. Some professional care the community very well. Some, in the health facility, I do not know why, they just refrain to serve us; they are not committed to serve the community. They just say “GO! Wait me there” they are looking us, we are from rural. But, they consider as if they are the only knowledgeable ones. They just act as if they never come from rural. You are teaching us now, and your origin is rural. They just undermine us as if they are not from rural. It is not now; I observed it when my wife was operated at hospital.

This should be corrected, because the community is coming to be served. Second, many are young, and they can be changed over time.

I: What barriers are there in other service?

P: It is good, and I have nothing to say.

I: what are the barriers occurring while implementing the intervention? Or what are these? Anything to be corrected; it could be from the service provider, the way how service is deliver or from the community perspective.

P: our problem is we think “what can the HEW help, she cannot provide anything” for example I mention about the egg. The money we get by selling two eggs will not change life, rather it is good if we eat it; it can build us. We do not think in this way. The same is for honey, we cannot buy it. It costs more than 200 birr and it is high like sky. When we have the honey, we do not use it. But what I think is you should it and build your body. These are the education you could give; otherwise, there is no other problem.

**Section3: Perceived needs of women for relevant services during pregnancy, lactation and adolescence**

I: What special things should a woman do to stay healthy during pregnancy, lactation and adolescence? For example what do women want to visit health facility? It can be at health center or hospital; For example you have mentioned me before that compassionate care should be given in hospital; right? What else do mothers need?

P: That is right! What you are asking me correct. But here, I do not have anything to be added or corrected,

I: Okay, let me give you an example. In one area, women told me that there is no ambulance service to get back home from health facility after delivery; and as need they tell us it is good if a second ambulance is added; how is this in your community?

P: It is correct; but here, they take and return them back to home on ambulance.

I: What about in taking extra meal during pregnancy and lactation?

P: I do not know; they know it themselves.

I: Anything you can suggest as perceived need from the services;

P: For them (women)?

I: Yes.

P: They need honey, meat and vegetables. They should eat variety of food on time, so that their body can be relaxed and they can deliver easily.

I: Therefore, is advice necessary here? Should it be well given health professional or other bodies?

P: They are giving us the advice; what we lack here is the resource. And we understood it; if she eat well, the baby will be good. What we miss is the resources; we do not have any other problem.

I: What about related to taking rest? When women become pregnant, it is advisable for to get rest. Especially for the pregnant women, and for lactating mothers in their early period of delivery (postnatal period); what do women say, it the rest they get enough?

P: No. they do not get enough rest.

I: Why is that?

P: Their life is forcing them.

I: What else? Anything out of their personal life; just from the services or nutritional interventions, for example during safety net, water and soil conservation activities; do they get enough rest?

P: During water and soil conservation, previously, the woman was not working until a month, until baby celebrates epiphany. But now, do not have the data, but I heard, they have added them some days. In the previous time the rest was not enough. It was difficult for her to carry a stone on weak back. She did not regain strength in 40 days. She was working fearing the punishment, five birr payment. Even she may die. But now, the rest is extended although I do not know how much day is added. It is correct, the problem was there: if the baby is male, the rest was 40 days and if the girl is female, the rest was 80 days. After that they were forced to work. And that was a problem.

I: What about any need regarding the supplements? Vitamin A (green tablet) supplementation; Iron foliate, a red tablet, to prevent anemia, and others;

P: All are given. While they are pregnant, they take the iron, and after delivery, their children are given medication every month until six months. Now, even the community has understood it; the mother also goes to health facility on her schedule. The health care provides also move to home and give education in this regard.

I: What do women expect from the husband? What should be the role a husband to improve his wife’s nutrition? Many times, in terms of nutrition, we have come by when priority is given to man than woman; priority is given to boy than girl. So, to solve this, what should a husband do to help his wife improve nutritionally?

P: Is it to the women?

I: Yes, in addition, what should the health care provider advise to husbands?

P: We should look them equal. They are our mothers, our wives; they are our light. So, we should ask them if they eat, and tell them that: this is your share; you have to eat. Whether she has or not, the ‘Gibabo’ (the plate) is always covered. Thus, whether food is available or not, we, men, consider as if she (the mother) can eat at any time. Thus we should say: this is for you; share it equal. Even if there is no food, we should share it in equal. We do not have to insult her: why do not you do this and that. This is a problem. She is like us; she was at work with us. If we insult them, and intermediate them, by saying like where were you? What were you doing? We will lose our light. But, we should lose her; we have to think each other. Sometimes, we may say “will I going eat this?” and get angry with them. But, we should not talk them like that.

I: So, in your opinion, how are we going solve this? And who should work on this?

P: Regarding this, we teach them in church while we preach a gospel. We (Priests) teach the men to respect their wife, and the wives should respect their husband. We are equal; we are one body. Therefore, we should respect each other. Regarding this it is good if it is given at church; we can teach them in detail. And there is no problem if they are taught in the community. But at church, we can clearly teach them: women should not suffer; we should support each other; we are created by God for this. We should not undermine her. They should be taught in this way.

I: Okay, very good.

I: Do women in this community typically change their diets when they are pregnant and lactating? Normally, they have to eat, variety of food, because it is important for the health of baby. So do they eat something different?

P: In this rural area, “who is going to eat” There is one who eats different food, because she does not have. She may have an interest to change, but she lacks resource. There many women who do not eat when they become pregnant, because they say we are sick and they leave it. She may like other food, but from where can she bring; it is because of this they do not eat what they want. Not only to the child, but also if she want for herself to promote her health, she cannot because she lacks resource.

I: Had women have resource, which food item do you think are good for women during pregnancy/lactation?

P: like honey and vegetables; these are what I think. If there are others, tell us and let we know it.

I: What foods are not eaten by pregnant women/ girls?

P: I do not know. None edible which our girls are prevented to eat, I do not know. But because of their behavior, there may food which is not like by pregnant when they hate food. And, what they want may not also be available.

I: What about as culture?

P: But, as culture, there is no food which pregnant woman is not allowed to eat.

I: What about for adolescent girls? For example in one kebele, I heard that a girl does not eat hot Injera and does not drink tea. Is there anything related to this here? Be it pregnant or not;

P: I do not hear; I do not know. We are seeing our children eating Injera be it hot or cold.

I: What affects women’s diet during pregnancy? Related to with decision making or their husbands might not buy them food, and cultures, e.g. women do not eat hen’s meat (poultry) if her husband is not around.

P: There is no such thing now. Previously, let alone hen, even they do not eat the routine ‘Tsebhi’ (stew) made of meat. No one will touch her until her husband comes. We saw a condition in which let alone a women, a child was slept barely (without eating food). Now days, our children will not wait until we eat, and even they will not wait until they give them; they eat by themselves. Even for the Tsabhi (stew), my home experience, they will not whether the husband come or not. Thus, there is such things (food taboo) today.

I: Are there gender disparities in women’s diets before pregnancy and during pregnancy?

P: There is no.

**Section 4: Other interventions that improve pregnant, lactating and adolescent nutrition**

I: Do women ever gone for nutrition screening during community health days or routine service delivery? It can be at home or at health post.

P: Yes, they come to this health post; they get examined here, and if they are okay, they will get back. Again, the child will be measured, if he is in need, he will be given, if he not in need, he will get back.

I: Who are coming to this center for screening?

P: Is it for the women?

I: Yes.

P: Those who women who gave birth. At that time, all women who gave birth were coming to the health post.

.I: What about now?

P: I do not know; so far, when my wife was lactating, she was coming, and both she and the child were measured, but now I am not sure; may be it is continuing in that way.

I: In one or two kebele, what is being done is, every month women (pregnant women and mothers who have under-five children) are mobilized to one place for nutritional screening. Is there such kind of activity now in this kebele?

P: yes; previously, yes it was.

I: What about now?

P: Why for do I know it now? I do not know.

I: May be or in case you heard while it being mobilized.

P: I have not heard in this year. May be because, I frequently go out of home for other activities.

I: Are women beneficiaries of soft conditionality of productive safety net program?

P: I do not know it; only those who are involved knew it. Even I cannot know it. For elder, it is told that because your elders you will get support while they are waived for work; but if someone is identified and told to work for food, he will not get it, if he did not work.

I: What if the mother is of six months pregnant or lactating mother?

P: I do not know it, but I saw women going to work by carrying their babies.

I: Do women in this community know why they are targets of the program? Is there any criteria?

P: What do I know her? They are identified based on investigation, and she is included if she is proved as poor. This is the only thing I know. I do not know other criteria. They are involved based on their poverty.

**Section 5: Understanding perceptions of age at first birth and birth spacing**

I: Do you think delaying the age at first birth to after 18 is better for the health of both the mother and the baby? How do you, as a religious leader, see this?

P: It is good.

I: Do you support this idea? How?

P: It is acceptable because the girl will know herself; she will know when to sleep and when to wake up, and agreed to it based on her interest, the life style will be good. It is also good for both herself and the child. But, if she is married in her childhood, she may end up with unnecessary danger, and the life will not be comfortable for her. If it is after 18 years, she may do what she wants: either to proceed with her engagement or leave it.

I: How about to the health of the baby? Which is better for him? Birth to after 18 or birth to before 18 years;

P: What is its importance? Because, it has been long time since this thing has been changed. Girls are married when they become big physically, and the community has believed on it. And, many of the time, they are in education. The one that become a problem again is: the courtship; which is not supported by both religion and the government. They say “I love you, he loves me” and this has an influence; but there is anyone who marries while she is in underage in our community.

I: Do you think this message is being promoted in the community?

P: yes, it is promoted.

I: Who is providing this information?

P: This information is transferred by the kebele administrators and teachers. They follow them at school. These health extension workers also give information to prevent under age marriage. And, at school, teachers taught them, and tell them not withdraw from school and advise them to learn; and “after you finish your education, you can marry” these are what teachers told.

I: How acceptable is this information by the community? Is misconception?

P: No there is no such assumption in our community. Because, everyone understood and knew it; the girl should know her age and go to ever she wants; there is such thoughts in the community. No one pushes her to marry if she is underage. In this locality in every household, there is no anyone who did not send his child to school. Then when the girl is on job or she is ready for job, she will be married on summer. The trend has changed; marriage is carried out in summer.

I: In your opinion, how could this message is better promoted?

P: In this kebele, all have understood the message. Even when a parent let her marry in underage, he will be accused, and it will be terminated. Let lone marriage people will be asked if they let their child withdraw from school. Anyway, all have understood the information. There is saying called “a stored honey and an abstained girl will not miss a person to take her” Therefore parents are advising their children to stay strong and educated; get educated and study. They also advise them not play with males (mis wedi tebaetay aytdebadeba); this is not important for you. Parents give such information to their children.

I: You may have heard that it is better for both mothers and babies if women wait at least 2 years after birth to become pregnant again. How do you see this? Is education given?

P: Yes, it is given. Even there is a pill to space birth; second, economically, if the child has to grow well, birth should be spaced. The mother will not also suffer. And for the parent, it will be easy to manage if the birth interval is increased, and it should be increased. The community has believed on it and he is applying it. Even regarding the children, women have decided their number of children and take injection (contraceptive).

I: Therefore, how do you, as a religious leader, see it? Is this supported by the religion? Or do we have other option we can take?

P: What option can I say?

I: Okay, is it acceptable by the religion to take family planning methods? Or other options like the natural method: by follow the menstrual cycle; or the other way: considering children are our wealth; what are you reflection here?

P: How will a child be wealth; it is good only if you have good economy; he can support. Otherwise, if you not care him after birth; if you do not get him to good position; if he betrays his religion because of hunger and poverty, it has no mean as per my opinion. If you have economy, a child is good. Papas (priest) can be born; he can be a world leader, and he can live good life. But, after delivery, if you do not have enough food; if you did not care him; if he falls in danger because of hunger and poverty; if he goes to other religion, it has no importance. Therefore if possible, you have to withstand (abstain) and space your birth. You care your child well and inform his religion. And, for the child, make him to live a good life. So, in my opinion it is good.

I: So, in your opinion how many years do you think the gap should be between successive births for a woman?

P: The birth space should at least be three years or more. If it is before three years, it is just pushing the other child. But after three, it is a bit better.

I: How? How important is this to the child and mother?

P: if the child is to grow, he should breast feed well; and for the mother, the child should start walking. Else, if she giver birth in a year, one will cry, the other will not grow well; and the mother suffers.

I: Do you think this message is being promoted in the community? Do you think the community is applying the lesson?

P: The lesson is given by the health care providers and the community is using family planning.

I: For the last time, it could be in the past week or month or three months, any ways as per its plan, was education given on family planning?

P: It had been given in the past years. But now, had I been a meeting participant, I could have tell you what day it was. Otherwise I am a famer; I go to my work; I cannot continuously participant at meeting. Therefore, I cannot speak in this issue. Anyways we learnt it; there is pill; there is injection even that can serve for five years. They told us such things. Then, to some them it is suitable and to some them it is not suitable for them. After injecting a needle (contraceptive), a number of dangers (side effects) are occurring; because women are not eating good diet. Such things have happed. However, education is given to the community.

I: Are there a group of community who do not get education? Or is there a gap in proper utilization and promotion?

P: In this kebele, there is no.

I: What activities are in place to prevent early marriage?

P: What can I say for this to prevent underage marriage; for this, tell them not let their child marry in underage.

I: what else is need? E.g. Any legal protection;

P: If there is underage marriage, the person will be punished by the government.

I: What else? How do the community confirm the age of the adolescent girl?

P: She goes to Woreda and testifies her age using witnesses. Then, she comes and she will marry. If she is underage, she will stay.

**Section 6: Understanding communication and information sources**

I: In your kebele, is there community conversations or discuss on women’s (pregnant or lactating) and adolescent’s nutrition?

P: I have not heard this. At meeting, this, nutrition, is not discussed. Only the health care providers provide us advice.

I: Where do the HEWs provide us education? Is it home to home?

P: They tell them when the women come to health facility.

P: Do not you finish your question now?

I: Yes, I am about to fine; one question is left.

I: What are the barriers to access information on maternal nutrition? Do all people get the information? E.g. Men;

P: They are wives are telling them.

I: What is the problem if men are involved nutritional education for women? Is it because they are not invited?

P: Even if they are told to participate (invited), they will not come; they do not give attention. They say: we do not miss how to feed (we know it).

I: Which source of information about nutrition is essential for you? E.g. you told me you get information from your wife; or other source; which is good for you?

P: It is good, all is fine.

**Section 7: Additional remarks**

I: Any other additional suggestions or comments on pregnant, lactating and adolescent nutrition in this community

P: In safety net program, there are women carrying a child and work until seven unlock. Thus, we say: if charity organizations (NGO) gave the food, why do not the people simply take the food without any work. He is called poor and again he is order to work and get suffer. “What does the person do?” this is what we conclude. Even the activities done by safety are not such mush relevant. There is nothing done in practice. By working hard, there is no any development or facility they built in the area. The support is good, but there is hard work; they intermediate them: “if do you not do this, you will not get this.”

I: Any other information;

P: I do not have.

I: Thank you very much father!

**Summary**

**Day10: Summary**

**Section1: common maternal nutrition**

In rural are women give low priority to feeding.

There is poverty and lack awareness/understanding existed as problem in community.

People do not know the cause of stunting, and assume it is because of nature, from power of God.

.**Section two: Barriers to access and utilization of nutrition services**

There are people who advice their wife to visit health facility.

Fasting is a religious barrier for lactating mother to extra meal.

The community does not give it attention to nutritional education, because they think they know it well.

There are no women with home garden.

There are no deworming services given to women and adolescent girls.

Giving birth at health facility is the one that brought change, because there is no woman dying during delivery.

**Section3: Perceived needs of women for relevant services during pregnancy, lactation and adolescence**

Women need honey, meat and vegetables; they do not get easily at home.

Women are not getting enough rest at home. This is because of their personal life, and activities related with safety net, water and soil conservation.

A husband should look his wife as equal him, because she is his mothers, his wife and his light.

**Section 4: Other interventions that improve pregnant, lactating and adolescent nutrition**

There is no community health day for the mothers in this kebele.

Lactating women are not beneficiaries of soft conditionality of safety net.

**Section 5: Understanding perceptions of age at first birth and birth spacing**

Kebele administrators and teachers are key source of information to prevent early marriage

There is saying called “a stored honey and an abstained girl will not miss a person to take her” Therefore parents are advising their children to stay strong and educated.

For the sack of the mother and children it is acceptable by the religion to take family planning methods.

**Section 6: Understanding communication and information sources**

In this kebele, there is no community conversations on women’s and adolescent’s nutrition

Men do not give attention to nutritional education because they consider as they know it all.
