# Supplementary material for: Barriers to utilize nutrition interventions among lactating women in rural communities of Tigray, northern Ethiopia: An exploratory study
Source: PLoS One. 2021 Apr 30;16(4):e0250696. doi: 10.1371/journal.pone.0250696 (PMC8087028; doi:10.1371/journal.pone.0250696)
Supplement: S2 File — (ZIP) [file pone.0250696.s002.zip › S2_File.Doc/Community level Key informants/137_IDI_-WDA_Hakfen kebele_Medebay Zana woreda.docx]

KII with WDA in Medebay Zana woreda, Hakfen Kebele

Zone: Northwest

Woreda: Medebay Zana Kebele: Hakfen

Facilitator’s Name: Measho G/slassie

Type of participant: WDA

Date of Discussion: 21/11/2017

Start time; 3:00 PM End time; 4:25 AM

Place of interview: Hakfen

Participant Characteristics

| **S.N** | **Name of FGD Participant** | **Age** | **Marital status** | **Educational Level** | **Occupation** |
| --- | --- | --- | --- | --- | --- |
| 1 | Merek Gebrewahid | 45 | Married | Uneducated | Farmer |

**Key: I: Interviewer P: Participant**

**Section one: Common maternal and adolescent nutrition problems in the community**

I: First of all I thank you very much for your willingness to participate in this study. When I come to my first question; what do the pregnant women do by themselves to stay healthy in this community?

P: As women development army, we have been following them. Our follow up starts after their pregnancy is identified (around their fourth or fifth month of their pregnancy). During our follow up time, we advise them to go to the health facility for diagnosis and to prevent the occurrence of blood shortage, to anticipate the occurrence of any danger things. First they get the diagnosis in this facility (health post) and then they go to the health center for further diagnosis. Even though we are not educated, we advise them whatever we know, like to use soup (“sibko”) of red teff because it can fill our blood. We also advise them that if you take good foods, your child will be good, but if you failed to take food due to your tiredness, your child will be tired; even if you have low budget, you have to prepare different foods from what you have, for example, if you failed to eat enjera, you have to try soup; in addition, we advise them to take vegetables like spinach, as I have told you, it is possible to get vegetables like spinach from the market by paying one birr. If the mother eats properly, her child will be good. However, if the mother properly takes foods, she will give weak child (“sahmam”). Therefore, we have been teaching them to do so and as a result they check their blood shortage and under nutrition status in the health facility and they are maintaining their health.

I: As you have told me, you have been advising the pregnant women to eat balanced diet and to get diagnose service in health facility. But, what do they do by themselves traditionally or culturally to maintain their health?

P: With the exception of few, the pregnant women in our Kebelle are very fine because they do care themselves. From my observation, those who have the capacity to go, have been buying spinach from the market and those who cannot travel usually buy it by sending their children. Even if they don’t have high budget to buy other things, they usually buy and take the spinach and salad. They know the relevant things for their life and they are feeding in a good manner.

I: Good. What do the lactating women do to maintain their health and their child’s health?

P: When we come of the lactating women; the delivered mother don’t immediately takes foods. There are some mothers who don’t eat porridge immediately after delivery. Nowadays, the lactating women are eating to give sufficient breast milk for their child despite they don’t have good appetite. In addition, to improve their health and their child’s health, they take soups instead of eating the food they disliked most. Currently, they think not only for themselves but also for their child. There is a saying “the mothers should not loss life to give life”. Currently, the community awareness is increased very much. The women do not have any hesitation to take care themselves unless they have shortage.

I: Good. You have told me that the delivered mothers do not take meal immediately after delivery. Why do you think?

P: It is because the delivered mother will not have the appetite to eat due to the blood smell associated with the delivery. The mother eats after she left the area where she delivered. For example, they don’t eat in the delivery room, but they eat after they transferred to the lactating women room. Therefore, they don’t eat immediately after delivery.

I: Good. In addition to their feeding, what other things do they do to stay healthy?

P: After delivering in the health facility, they went back to their home by car and they spend in their home during the sunny times to prevent themselves from the sun, and dusty things, but they only go out of their house during the evening time or when the sun is shaded by clouds. Nowadays, Thanks to God, the women are very fine.

I: Good. You have told me what the pregnant and lactating women do to stay healthy in this community. What do the adolescent girls (from 10 to 19 years of age) of this community do to stay healthy?

P: The adolescent girls are using contraceptive methods to protect themselves because they are learning in the schools. Currently, the right of the females is protected and they are caring themselves. There is also follow up to prevent early marriage because if the girl married under 18 year of age, her body and uterus will not be matured and she will face many problems. We are following to prevent early marriage. So, early marriage in this community is not common, though some of the adolescents who failed in grade ten are marring in their early age.

I: Good. What else do they do to stay healthy apart from prevention of early marriage?

P: To stay healthy?

I: Yes, what do they do to stay healthy?

P: As to my observation, their focus is on their education, they don’t have any interest for marriage, and they don’t have interest for husband. When I see the small children, they don’t have any interest to have husband, but their interest is to continue their education. They want to properly write and study their education. They only focus on maintaining their personal hygiene and also caring their exercise book.

I: Good. What are the commonly observed nutrition problems in the community?

P: We have vegetables (“hamli”) during the June, but our area is not suitable for irrigation. Those areas near Selekleka have irrigations, but this community does not have irrigation. However, we have been talking the vegetables like tomato, onion and spinach from the market. We have been consuming the vegetables more than garden owners. Despite it is not available in our homes; we are buying from the market. Our problem is we don’t have our own home gardens, but we can find the vegetables in the market. The women do not have any problem.

I: Good, do you think the pregnant women of this community are at risk for under nutrition?

P: I have not heard any pregnant women who have food shortage in this community. But, from my observation, there may be some women who have shortage of budget. If she bought cloths for her children and others, she may have shortage of money to buy foods. If we sell some cereals, we may buy potatoes, onions, tomato and oils, and as a result we will be satisfied. But, if we don’t buy it, we will not be satisfied. So, there are no any women who said we have shortage of food, but as women development army, we think that some of them may have food shortage. The pregnant women are very fine, Thanks to God. They are delivery safely and they are changes foods with what they have.

I: Good, are there undernourished pregnant women in this community who are being given Fafa or other supports from the government?

P: It was given in the past, but currently it is not available. We have a meeting with the pregnant and lactating women in every St. Michael day (12^th^ day of the month in Ethiopian calendar) in our village (“kushet”). During our meeting, we prepare porridge from different types of grinded cereals, oil, spinach, carrot, potato and others to the children. I wish if you could see it, even people from the region have seen it. We have been showing it to the people of the village and Kebelle. Let alone to the children, we have been telling the mothers that you will be addicted if you test it.

I: Have you developing the porridge for sample or for feeding the children?

P: It is just prepared to show them how to prepare the diversified food (they call it “mitin”). We have been telling them to prepare it in their homes for their children and themselves.

I: What are the elements of the sample porridge (“mitin”) prepared in the meeting?

P: Red teff, maize and from all grinded cereals are first mixed and heated, and then potato, carrot, spinach and oil are also prepared. During non-fasting time, we also add egg otherwise we don’t add, because we are supposed to taste it in order to encourage the women to feed it. Finally, they will promise to prepare it in their home for their child.

I: What about for themselves?

P: Do you think they will not eat for themselves while they are preparing and feeding their child? (laugh). Thanks for the government, they have very good awareness and they are fine. Due to the plan he (The late Prime Minister Meles) left, currently the women are very fine.

I: Is there nutritional screening (weight and upper arm measurement) for the pregnant and lactating women in this community?

P: yes, they are being measured.

I: Are there undernourished pregnant and lactating women in this community?

P: As a chairperson of the women development army, I have been working together with the health extension workers. The children come to the health post for nutritional measurement. Despite similar food is given for different mothers, some of the children may have lower measurement. In that case, his mother will be advised to properly give him the provided food in the given month. We (the women development army) have been also teaching them. Then, within the given month, the child will come with improved nutritional status.

I: Do the pregnant women get measured?

P: Yes, they are being measured.

I: Is there any nutritional support (like Fafa) for the pregnant and lactating women who have under nutrition?

P: There was Fafa in the past, but now it is stopped. The women are advised to eat balanced diets at their homes. The thin children were given Fafa in the past.

I: Do you know why the Fafa is stopped?

P: I don’t know it. In the past it was given for the children who had visible tendons, but now we don’t have such kind of children.

I: Do you think the Fafa is stopped due to the fact that there is nutritional improvement in this community?

P: I don’t know why the Fafa is stopped, but it was working for the children. However, we told the women not request for Fafa because we told them that they can prepare Fafa in their homes. First we asked them to prepare and mix ten cups of different cereals and spices (one cup of white maize, one cup of red maize, one cup of barley, one cup of millet, one cup of pea, one cup of bean, one cup of wheat and others cereals) and then we told them to grind the mixed spices and cereals after adding one coffee cup of “ABAKE”( a local spice) and one coffee cup of “AWASDA”( a local spice) on it, then finally we told them that this is what Fafa means. I have tested it myself while I was lactating mother. Despite I have only around two children, they grew up very well. Initially, I have prepared the locally made Fafa by myself because there was conflict between the women to enter in to the government support. I just prepared it by collecting the relevant cereals from what I have and by purchasing from the market the important ingredient that I don’t have at home. After cleaning and heating the mixed ingredients, it became 10 cups and grinded it, and then I added sugar. Then, I was addicted by that food let alone my children. My child grew up very well by eating that food. Thus, I have been advising my group members to prepare such kind of Fafa in their homes.

I: Have you ever heard about the pregnant or lactating women who have blood shortage in this community?

I: Starting from last year, there was no any women who were told to have blood shortage. Even if they are told to have blood shortage, they are improving by properly eating in their homes.

P: In the past there were women with blood shortage and the government was supporting them. For example, there was a pregnant woman from this community who was saved after she went to the health facility.

I: When was that?

P: It was before a year. My neighbors told me that the pregnant woman is not willing to go to the health facility despite she was sick. When I visited her, she was looking black and I told her to immediately go to the health facility but she was not willing to go to the health facility with the intention to help her children. Together with the health extension worker, we pushed her to go and she was given treatment which costs up to 700 birr. So, her life was saved by the government. Therefore, starting from last year, the women of this community are fine.

I: Are there pregnant and lactating women who have night blindness in this community?

P: There were some women who have the problem. In the past, there were blue colored medications like the color of your cloth (indicating to the blue color of my shirt) coming to this community for the treatment of night blindness, but it is not available now. In the past, the lactating women were using it. When the women say that we have nigh blindness, I advise them to take the soup of red teff with sugar.

I: Why do you think the red teff is important for treating night blindness?

P: The medication that I have told was immediately eliminating the night blindness. If you have blood shortage, you will feel dizziness (I myself had dizziness). So, to prevent this kind of problems, the red teff is very important because it has blood. However, the white teff don’t have blood, but it is attractive for your eyes. If a women drinks soup of red teff, it works. So, at this time, there are no women who have night blindness.

I: Good. Are there any women who have goiter in this community?

P: Yes, there are women who have goiter, but we advise them to use iodized salt. There is no any woman who doesn’t use iodized salt because it is very important to create good brain of the baby. It also helps to eliminate goiter. Currently, there are no any women who develop the goiter due the iodized salt utilization, but there are some women who developed it in the past.

I: Good. Are there any communicable diseases which are common in women or adolescent girls of this community?

P: We don’t have it. There were some women who had problems in their uterus, but they have improved after treatment, thanks for the helpful government. In the past, there were some women, who don’t come out of their house, but they are now health, thanks for the government.

I: What was the disease of the women who were spending in their house?

P: They had uterus problems, but they have cured after treatment in the hospital. However, I have not seen other diseases in this community.

I: Ok, have you ever heard about some people who have noncommunicable diseases like hypertension, diabetes mellitus or others in this community?

P: There are some people who have diabetes mellitus and they are taking their treatment.

I: Do you think this kind of diseases is related with the person’s nutrition?

P: If the people who have this kind of disease get proper food, they have the chance to improve from their disease. This kind of disease requires foods like meat, milk which softens the body. So, the people who have the disease are fine due to their good feeding.

I: What should be our feeding practice to prevent this kind of diseases?

P: Those people who were bedridden due to the disease are now working due to the treatment and their good feeding.

I: So, how should we prevent the diabetes mellitus?

P: “Those who hide their illness are hiding their treatment”. Nowadays, the people are disclosing their diseases. There is improvement, because those who have the disease are getting the diagnosis and treatment timely and they are also taking properly feeding, thanks for the Government.

I: Do you know stunting?

P: Do mean short?

I: Yes, are there stunted girls in this community?

P: *Thanks to God, we don’t have stunted girls (talking with slow tone voice and impressively). It is observed in some women, but thanks to God, we don’t have it*. There are some sick girls who don’t come out of their homes. They are not supported by the government.

I: Do you think those sick girls who don’t get out of their house are due to food shortage?

P: It is from the will of God. Their parents are giving them foods. *People cannot be short due to food shortage; rather they can be short due to the will of God.* Their parents are giving them foods by working here and there.

I: What about underweight? Are there underweight or very thin adolescents or lactating women in this community?

P: They are good.

I: As I have told you in the very beginning, the aim of this study is to identify the common nutrition problems of this community and design appropriate services that helps to improve the nutrition problems of this community. So, this is not an evaluation.

P: Let me tell you here. As I am telling you, there are children who don’t come out of their houses. They didn’t get any support from the government, but they are only supported by their parents. Their parents are suffering to provide food, cloth, and treatment services for them.

I: So, how do you see the availability of underweight adolescents in this community?

P: With the exception of the three sick children, the adolescent girls are very good.

I: Good. Does the community have sufficient food from year to year?

P: Whether they have sufficient food or not, they don’t sleep a single night without satiety. Even if they have food shortages, they will not spend a night without daily food through working elsewhere. Let alone the farmer, the government will also has a problem. So, we are good.

I: So, you mean that there are no any women who have food shortage?

P: There might be some women who have the problem.

I: As women development army, I hope you know more about the community. For example, how many women are you leading in your community?

P: In women development army?

I: Yes

P: Thirty

I: You can think of the thirty women in your group. Are there any women in your group who have food shortage for herself as well as for her children?

P: There is one child, who has diabetes mellitus in my group, but he is being supported by the safety net program and he is also getting treatment for free. The women who are leading their family may have some problems, but generally I can say they are fine. Everyone should not be supported by the government, but we all supported to the child who has the diabetes mellitus to be supported by the government. He is getting support from the safety net program and he is also getting the treatment for free, as a result he has improved now.

I: It is good that the child has improved after the support, but the main targets of our study are the pregnant and lactating women and adolescent girls. So, I would like you to describe about the availability food shortage in the women of this community?

P: There is one elder women who have food shortage in this community, but I am not sure whether she is enrolled in the safety net program or not. I was requesting the responsible bodies to enroll her in the safety net program, but I am not sure whether she is enrolled or not. I have not asked her this week whether she is enrolled or not, because I was in Axum to visit my pregnant daughter there.

I: Is the safety net support given only for the people who cannot work or there are other people who are given the support by working?

P: There are people who are being given the safety net support by working

I: Are there pregnant women supported by safety net?

P: There are few poor pregnant women who are supported by safety net.

I: Do they get the support for free or they are working to it?

P: There is no any support for free. So, they are expected to wok to get the support.

I: Up to how many moths of their pregnancy do they work?

P: They have rest during their pregnancy up the baptism of their child.

I: At what month of their pregnancy do they start their rest?

P: In the past they were hiding their pregnancy, but now they don’t hide it. So, they have a rest up to one year during her pregnancy and after delivery.

I: So, when do the pregnant women take rest while working in the safety net program?

P: The pregnant women do not work at all starting from her conception. For example, I had worked in the environmental conservation while I was pregnant because I was shy to speak, but now they openly speak if they have pregnancy and as a result they will not be allowed to work. Therefore, we don’t allow a pregnant woman to work in the safety net and other works.

I: Good, why they are not allowed to work?

P: To prevent the mother and her child from different diseases

I: Well, you told me that the women are not allowed to work in the safety net program. But, do they get rest in their homes?

P: They don’t do heavily activities, but they can do easily activities like making enjera and prepare foods.

**Section two: Interventions for improving maternal and adolescent nutrition**

I: What nutritional interventions are available for improving the nutrition of the pregnant and lactating women in this community?

P: If the sleeping cloths of the child are not washed using soap and if it is not properly dried, it will create disease. Similarly, if the mother maintains her sanitation and she washed her cloths, she will be healthy and she will not be sick. In addition, she has to eat after she provided foods for her family and she have to take rest. She has to sit down and eat her lunch while breast feeding her child.

I: Good. How do you describe the counseling given for the pregnant women to attend antenatal care?

P: During antenatal care follow up, they are given information about not to lift heavy materials, not to takedown water filled containers from the back of the donkey. In addition, we have been advising them to get diagnosis to prevent blood shortage. So, you will not get any pregnant who do get diagnosis services. They do have good awareness and they follow their antenatal care until their delivery. Majority of them are also delivering in the health facility because to prevent blood shortage and some of them may have narrow pelvic (“mahtsen”). Since the health workers know all the problems, the pregnant women follow their check up properly.

I: What types of nutrition counseling are given for the pregnant women during antenatal care by the health workers or women development army?

P: There was a time where the pregnant women gets balanced diet in the health facility. The health workers were telling the pregnant women to stay in the health facility for some time and they deliver safely. So, the mothers are very good starting from last year.

I: What types of advice are given to them?

P: They are advised to take care of themselves. If they don’t care themselves, there will be pain and tiredness, others. So, information is given to prevent themselves form tiredness and weight loss and they support themselves based on the provided information.

I: How many times should they eat per day?

P: If they get good food, they have to eat three times per day. There is breakfast in the morning, lunch; there is also snack in the afternoon and dinner. So, she has to eat four times including dinner.

I: What is good food?

P: Good food in fasting time is composed of pepper, spinach, tomato, onion, oil and lemon. During the non-fasting time; if there is no shortage of budget, you have prepare fried egg. So, it is important to advise the women to feed in such away.

I: Is this kind of advice given for the pregnant and lactating women?

P: Yes, it is given for all of them. We advise the pregnant women to eat good foods. We tell them that if you eat good foods, you will deliver a good baby and we also tell them that the food you take is shared for your baby. Immediately after delivery, we advise the woman to take good food like “fino” and soft drinks in order to provide colostrum for her child. The child should take the colostrum and it should not be milked away. Immediately after birth, the child should take the colostrum, if the mother eats food after delivery, she will have sufficient colostrum. Currently, the pregnant women are caring themselves.

I: As you have told me, counseling is given for improving the nutrition of the pregnant and lactating women. Is similar nutritional counseling given for the adolescent girls?

P: We have little gap on the adolescent girls. They eat their breakfast early in the morning before going to their school. If they don’t eat their breakfast, they will not understand their education. They are also eating their lunch properly.

I: Do they get advice about their nutritional counseling like the pregnant and lactating women?

P: Advice for the adolescents is given by their parents about not share laughs with the males, rather to concentrate on their education. They are also advised that if they laugh with the males they will not understand their education.

I: Who gives the advice? The parents or…(interrupted)

P: We advise them not to disturb in class, to strongly study their education and to attend all classes through going to their school. But, since they have good awareness, they don’t like to; disturb, laugh with the boys, hunger and wear bad cloths.

I: Good. How do you see the advice given for the pregnant and lactating women to cultivate home gardens?

P: Our land is rocky and not suitable for home gardening. But, I had home garden like onion, spinach, and other vegetables for sample to teach the women on how to prepare the home gardens. The women were using their own home gardens after I showed them how to cultivate it. However, we have shortage of water. As I have told you we were using our home gardens in the winter, but we have shortage of water. There are not any women who don’t want to cultivate it, but we have lack of water for drinking let alone for gardens. Vegetables are important for softening the women’s body.

I: Do the pregnant and lactating women use insecticide treated nets in this community?

P: They are using the insecticide treated nets by stretching it using four wood stands. There is no anyone who don’t use the insecticide treated nets in this community. In fact the government is giving us the insecticide treated nets.

I: Is there any tablet given for the children and adolescents in every six months for the prevention of intestinal parasites in this community?

P: the red tablets?

I: A tablet given for the children and adolescents in every six months.

P: Yes, it is given for the treatment of abdominal worms. Thanks for the Government, they are caring them. The health extension workers are following them very well.

**Section three: Implementation challenges and community factors affecting access to nutrition**

I: Good. You have described that there are many intervention being implemented for improving the nutrition of the pregnant and lactating women; for example, a counseling given for pregnant and lactating women on balanced diet, counseling on extra meal, counseling on antenatal care follow up, counseling given to use ITN, counseling given to maintain their hygiene and sanitation and others. So, what are the challenges for implementing these interventions?

P: Our main challenge is water shortage, but we don’t have other challenges. If there was sufficient water in this community, we wouldn’t buy the vegetables from the market. As I have told you, we have tried in our home, but we stopped it due to shortage of water.

I: Ok, we can also see the challenges out of the home gardening, like the challenges related with the community culture, tradition or awareness on counseling given for pregnant and lactating women on balanced diet, counseling on extra meal, counseling on antenatal care follow up and others.

P: With the exception of water shortage, there is no any challenge in our community.

I: How do you see the sanitation and hygiene of the pregnant and lactating women?

P: Despite there is water shortage, they don’t have any problem with sanitation. For example, half of the water they bring (two “jerican”) is used for washing child cloths and the other half will be used for food preparation. Currently, they are maintaining their sanitation and their child too.

**Section 4: Perceived need of women and adolescent girls**

I: What types of services do the pregnant women need by themselves?

P: The ambulance is coming to the gate of their house. If there are any women who have a problem in this community, we immediately call to the health workers (if we have mobile if not we will call through other’s mobile) to send ambulance to our community to take her to the health facility. So, there is no any problem.

I: You are telling me that the women do not have a problem, but what do they need by themselves?

P: They need sanitation, but they are doing it. There might be child urine related smells after delivery, but if they get water and soap, the women will not have any problem. So, they need sanitation.

I: What should be the role the husbands for improving the nutrition of women?

P: If she prepared a stew of potato with onion for her husband, she will eat together with her husband. The wife can also eat what is left from her husband. But, currently the wife is eating not only what is left but also equally together with him. So, they don’t have any problem.

I: So, do the pregnant women eat what is left from their husbands in this community?

P: They eat together with their husbands. In the past, the women were providing the meal only for their husbands, but now they eat equally. Whatever prepared in the house like potato, locally prepared alcoholic drinks (“siwa”) are taken together.

I: You have informed me that the pregnant women should eat three or four times extra food per day? Do they practically eat three or four times per day in this community?

P: The husband may went to the market or other works by eating his lunch early in the morning, but the women cannot wait him until he returns back from his works. In the meantime, they prepare food for themselves. Then, she also eats together with him when he comes back from his duties. She cannot spend the day only by eating with her husband because he didn’t take the food with him.

I: I have heard in other communities that the wives do not eat any food in the absence of their husbands. So, how do you see in this community?

P: Wow! We don’t wait them. If we prepared chicken, it is correct, we wait them, but if we bought meat, we don’t wait for them instead we prepare and feed our portions with our children and we prepare and put some portion of it for the husband.

I: Why do the wives wait for their husbands if they prepared chicken?

P: It is based on the culture of our parents. Our mothers were not eating chicken in the absence of their husbands. It is cultural taboo, but we don’t wait our husbands if we prepared other foods and we also eat again with our husband. We were only waiting our husbands for every food in the past, when the time was mad and when we were not participating in the meetings. However, now we don’t wait them.

I: Good, what types of foods are recommended for the pregnant women in this community?

P: As I have told you, red teff, honey, meat, spinach, tomato and other vegetables are important for the pregnant women.

I: Good, what types of foods are not culturally recommended for pregnant women in this community?

P: None. How could she deliver good child if she doesn’t eat very well? How could she get energy to push down the baby if she doesn’t feed very well? She will be tired if she doesn’t feed well.

I: Good. But, are there food taboos for pregnant women of this community?

P: The pregnant woman eats every food.

I: For example, roasted pea is not recommended for the pregnant women… (Interrupted)

P: Pea is not recommended because the pregnant women will have “kiliwlaw” before delivery if she eat pea. Even, if the pregnant woman has “kiliwlaw” before delivery, the people inquire about her history of pea feeding.

I: What does “kiliwlaw” means?

P: “Kiliwlaw” is “chiramo” which is server pain during pregnancy. Despite it is not recommended to eat pea, currently the woman eats every food including pea. In the past, the people were advising them not to eat pea, but now they eat every food. Unless they have loss of appetite, they eat all foods including roasted cereals.

I: Good, is there community health day in this community?

P; Yes, we have monthly health care services in our village. There are monthly child vaccination services in the villages.

I: Do they provide other health services apart from the child vaccination?

P: Yes, there are also other treatments for the mothers.

I: Ok, what are the benefits of community health days for the pregnant and lactating women?

P: The vaccination given for the children will help them to prevent from whooping cough, measles, polio and other disease. If they take the vaccination, they will be completely healthy. The treatments given for the mother also helps her to prevent from cough and other disease. After they have got the treatment services, they become healthy.

I: Are there any challenges that prevent the pregnant and lactating women from using the health services in the health post and health center? It could be in terms of distance, cost or others.

P: No, we don’t have any challenge.

I: You have told me that there is a safety net program for the poor people. So, how do you see the role of safety net program for improving the mother’s nutrition?

P: It helps to improve their nutrition. For example, the child that I have told you before is now healthy due to the food support from the safety net program. The financial support from the safety net program could help the poor women to buy cloths, fertilizer and to educate their children.

**Section 5: Other interventions that influence adolescent and maternal nutrition and health outcomes**

I: Good. What are the benefits of delaying first birth after 18 years of age on the health of the mother and her child?

P: In the past, the mother was delivering in every one or two years, as a result, the children were dying. But now, the women are getting supports and benefits to implement childbirth spacing. If the mother delivered after six or seven years, the children will grow without envying and yearning. If the mother delivered birth after birth, the former child will be longing his mother.

I: At how many years interval should a mother give birth?

P: A mother should deliver in a six year interval in order to develop a healthy child. If they properly space their births, their children will be strong and they will have good feeding, cloth and they will be physically big. However, if they deliver birth after birth, both the former and the later children will be very weak. Currently, the women are using the contraceptive methods and they are also spacing their childbirths.

I: At which age should they deliver their first birth? Before or after 18 years of age?

P: A girl should deliver her first child after her 22 years of age in order to have mature body.

I: Why do you think?

P: At 22 year of age, the girl will have long waist. If the women has long and wide waist, she will carry the baby without difficulty and she will deliver a healthy child. However, if the girl delivered before her 18 years of age, she will be at risk for different problems and accidents, because her waist is not long and generally she is not mature to give birth. But, now the women are delivering their first child at 25 years of age.

I: Good. How do you describe the awareness of the community for prevention of early marriage?

P: Early marriage is now eliminated in this community because the people have schools at the gate of their houses. Even if their parents try to enforce them for early marriage, the girls will not be willing for the marriage. They focus on their education. The parents are also learned from their child’s previous early marriage. The farmers do not have the intention to propose their child for marriage; rather they want to educate their children. If the adolescent girl is married in early age, she will face many problems because hasn’t mature body and wide waist. So, the early marriage is eliminated and the main focus of the adolescents is on education. For example, if the parent asked her daughter bout whether she likes to marry or continue her education, she will insult him back. Their attention is on their education because they understood its benefits.

I: Good, are there any obstacles for the women for childbirth spacing? For example, are there any obstacles for using contraceptive methods by religion or others?

P: Whoever says what; there are no any women who don’t use contraceptive methods. Some people say that it is a sin to use contraceptive methods, but they are using it. No woman listens for that kind of people because they have good awareness about the contraceptive methods. Every woman has been using it. Even the married adolescents do not sleep with their husbands without using contraceptive methods. They remove their contraceptive methods to become pregnant after they get a good condition to do so.

**Section 6: Understanding communication and Information Sources**

I: Do the women discuss about their nutrition in this community?

P: The women?

I: Yes, the women.

P: Yes, they have been discussing whenever they meet about what types of foods to eat for improving their health. Nobody dislikes good food. So, they have been discussing about it.

I: Good, from where the pregnant and lactating women get the information about nutrition?

P: Information?

I: Yes

P: The chairperson of the Women Development Army has been giving information to them. She has been advising to those women who don’t go out of their home to attend meetings, if they are not willing to go she advises them at their homes to eat the important foods for their health.

I: What other sources of nutrition related information is available for them out of the women development army?

P: Information?

I: Yes, information about nutrition

P: Currently, the women have bright mind by themselves. They are ordering their husbands to bring what they need.

I: In order to increase their awareness about nutrition, they need information. One, the women development army are providing the important nutrition related information to them. What other sources of information are available?

P: The health extension workers have been also advising them about their feeding. During our monthly meeting, the health extension workers teach them about the important foods for their children and themselves and to maintain the sanitation of their cloths and body.

I: Are there any women who don’t get the information?

P: No. All of them are informed, unless they give less attention for it.

**Section Seven: Additional Remarks**

I: Good. The main aim of this study is to know the main barriers of access for nutrition information and nutrition services implementation. So, if there is anything left or if you have additional suggestions you are welcome?

P: Ayy! It was good, but I don’t know I may have given you incomplete information. I am just speaking here and there because I am not educated. You are working to solve the nutrition and health problems of the farmers. Everything is good. My two daughters have peacefully delivered their babies this year. In the past, there were many problems during our pregnancy and delivery, but everything has been changed. Nowadays, even we fear risks if a pregnant woman bends down little bit. So, we are very fin and everybody has the awareness.

I: Thank you very much for scarifying your time to participate in this study.

P: Yes, I spend here by leaving many holyday invitations from my relatives.

Summary:

**Section 1: Common maternal and adolescent nutrition problems in the community**

- Nowadays there are no nutritional problems in the pregnant, lactating and adolescent women.

**Section 2: Interventions for improving maternal and adolescent nutrition**

- The counseling is being given for pregnant and lactating women on balanced diet, counseling on extra meal, antenatal care follow up, counseling, ITN use, and to maintain their hygiene and sanitation and others.
- Home gardening is not practiced in the community due to water shortage

**Section 3: Implementation challenges and community factors affecting access to**

- Water shortage is the main challenge for the implementation of nutritional interventions
- Most of the time roasted pea is not recommended for the pregnant women due to fear of blunt abdominal pain
- There is gap in implementing nutritional interventions for the adolescent girls

**Section 4: Perceived need of women and adolescent girls**

- The women need sanitation

**Section 5: Other interventions that influence adolescent and maternal nutrition and health outcomes**

- The community has good awareness about the prevention of early marriage and child spacing

**Section 6: Understanding communication and Information Sources**

- The women development army and health extension workers are the main sources of nutrition for the women
