# Supplementary material for: Barriers to utilize nutrition interventions among lactating women in rural communities of Tigray, northern Ethiopia: An exploratory study
Source: PLoS One. 2021 Apr 30;16(4):e0250696. doi: 10.1371/journal.pone.0250696 (PMC8087028; doi:10.1371/journal.pone.0250696)
Supplement: S2 File — (ZIP) [file pone.0250696.s002.zip › S2_File.Doc/Lacatating women_IDI & FGD/010_FGD_LW_Sesela Kebele_ofla woreda.docx]

Date: Nov 2, 2017

**Operational Research on Adolescent and Maternal Nutrition in Northern Ethiopia**

Focus group discussion with lactating women in ‘Dasus’ Kebelle, Ofla Woreda, Tigray.

Participants’ General Information

| **Participant** | **Age** | Educational level | **Woreda** | **Kebelle** | **Occupation** |
| --- | --- | --- | --- | --- | --- |
| Participant 1 | 20 | No education | Ofla Woreda | Dasus | House wife |
| Participant 2 | 25 | No education | Ofla Woreda | Dasus | House wife |
| Participant 3 | 27 | No education | Ofla Woreda | Dasus | House wife |
| Participant 4 | 20 | No education | Ofla Woreda | Dasus | House wife |
| Participant 5 | 32 | No education | Ofla Woreda | Dasus | House wife |
| Participant 6 | 20 | No education | Ofla Woreda | Dasus | House wife |
| Participant 7 | 28 | No education | Ofla Woreda | Dasus | House wife |
| Participant 8 | 20 | No education | Ofla Woreda | Dasus | House wife |
| Participant 9 | 30 | 9^th^ grade | Ofla Woreda | Dasus | House wife |
| Participant 10 | 22 | No education | Ofla Woreda | Dasus | House wife |
| Participant 11 | 27 | No education | Ofla Woreda | Dasus | House wife |
| Participant 12 | 31 | No education | Ofla Woreda | Dasus | House wife |

**I:** Interviewer

**P:** Participant

**I:** I would like to thank you for showing your consent to participate in this study. Having said this much, I will directly go to the task we will do. Just feel free to share us your opinions and ideas. There are no wrong and right ideas. Just share us what you have. Now, let me go to the first question.

**I:** In this kebelle or Tabia or Community, what do lactating women like do to be healthy?

**Ps:** Participants remained quiet *[They look like they are confused].*

**I:** Did you understand the question? The question is ‘what do lactating women do to stay healthy?’ We would like you to share us one by one.

**P3:** Okay, my code is 03. We need to take adequate amount of food prepared from sorghum and wheat and we have to go to health post.

**I:** What any other idea can you say?

**P9:** To stay healthy, I have to have medical health services and I have to eat food and drink water.

**I:** What type of food should you eat?

**P9:** Food like potato, salad and teff.

I: Other idea? As you heard, number 03 said I have to seek medical care and I have to eat to make myself healthy. So, what additional ideas do you have?

**P 5 and P 6**: No, we don’t have additional ideas. Just they [P3 and P9] have said it.

**I:** What are the common problems related to nutrition in this kushet?*[kushet is to mean village].*

**P9:** If there is shortage of food intake, we will face illness.

**I:** When you say illness, what do you mean? I mean what type of illness?

**P9:** For example, anemia and malnutrition.

**I:** What else?

**P9:** I have finished my idea.

**I:** Any other idea? What are the common problems related to nutrition?

**P10:** Hum hum….

**I:** Do you have idea for the question I raised? Can you share us?

**P10:** No, I don’t have. I don’t know the problems related to nutrition. I am a housewife and a farmer. I know how to breastfeed my children and prepare food.

**I:** Number 07, you look like a person with a lot of ideas. Can share us please? Just you tell us anything you know. Don’t hesitate to share us your very important ideas.

**P7:** My sisters said it all *[bending down her neck and paying intention to her child].*

**I:** Additional ideas?

**P1:** If we lactating women do not eat food, we don’t produce much milk for our children and therefore, they [‘they’ means the children] will be malnourished and will be sick.

**I:** What do you mean by malnourished**?** Can you clarify it, please?

**P1:** Em….[participant remained silent thinking on the questioned asked]. I mean our children will be thin and weak.

**P11:** I have never heard of this idea.

**I:** Now, I want you to share me your ideas regarding the target population of nutrition problems. In your perspective, which group of individuals do you think are most affected by nutrition problems?

**P10:** Ourselves.

**I:** When you say us, what do you mean?

**P10:** I mean pregnant women, laboring and lactating mothers and children because if pregnant women do not eat food five times a day, we will face a problem and be starved and finally the child will refuse to come out of the womb during delivery. In case of lactating mothers, we know that lactating mothers have to breast feed their babies ten times a day. If we[we means lactating mothers] don’t eat food we will not have adequate breast milk for our babies and if this is so, our children will be starved and finally become weak and sick.

**I:** When you say our babies will face sickness, what do you mean; I mean what type of sickness/illness?

**P10:** I mean when my baby is starved, he will be thin. This is what I mean. I have finished my ideas.

**I:** Thank you code number 10. What other opinions do you have? So far, it is said that lack of food intake can bring about nutrition problem like anemia and other nutrition related diseases like thinness as mentioned by number 10. So, what else can you tell us about the target population of nutrition related problems?

**P9:** Those who are affected by nutrition related problems are our female population of this community and children.

**P7:** I am not sure which groups of population are affected by nutrition related problems.

**P6:** Mothers who have children and children themselves are liable to nutrition related diseases. For example, today we arrive here in this school to participate in this discussion and were waiting for you to arrive in time. But, you didn’t and therefore we become hungry. Thai is it. We become starved and our babies also as our breast couldn’t produce adequate milk. We spent the whole day waiting for you. We didn’t eat food until now.

**I:** Thank you again for your opinions. Now, I will take you to another question. The question is about barriers of utilization of nutrition services in health post and other facilities. What barriers do you face?

**P4:** As you can see, I have two babies (twins). Since I couldn’t feed them with adequate breast milk, they [they = refers to the health extension workers] have told me to bring my twin babies to health post to check their health status. They advised me to feed them plampnet as they are starved and weak. The health extension workers are doing what they can do. They give us health education by going door-to-door. They give us medicine for us and our babies. It is good.

**P12:** They tell us how to prepare food for our babies, how to keep them clean and neat though we don’t work for it in reality. It is good.

**P6:** We have no barriers with regard to the services. The problem, for example, I have is the distance to be travelled. I travel for one and half hour to get the services in health post. Due to this, I sometimes cancel my appointment by health extension workers.

**P10:** They [They = the health extension workers] advise us to use bed net to keep our babies from the attack of mosquitoes and they even tell us how to prepare food for our babies. We don’t have problems.

**P3:** We usually get advice to use iodized salt and variety food for ourselves and our babies. We have not problems.

**I:** What else?

**P8:** My sisters have already described them all. I don’t have a different idea.

**I:** What things do you do to stay healthy while you are in lactation period?

**P5:** We have to eat variety of food, visit health post for medical advice.

**P7:** We have to eat more to become healthy and get medical services.

**P1:** Yes, it is almost talked on by sisters. As they said [they = the group discussants], we have to eat, drink, get advice from the health extension workers and avoid travelling long distances. That is it.

**I:** What else do you do?

**P9:** What we have to do is talked on about. I don’t think there will be additional ideas *[she stood up signaling the time is running and is time to go home].*

**I:** Do you/women in this kebelle change their diet when they become lactating mothers? What are your ideas and opinions?

**P2:** I don’t do a different thing**.** I eat food like the amount I eat when I am not pregnant.

I: But, you know a lactating mother, like what have been talked about so far, should eat additional food to produce more milk for her baby. What do you think?

**P2:** We know what you said. What I am saying is I couldn’t make it practical as I am not economically strong and one from the well to do family.

**P1:** Mmm…., sometimes we eat more food and drink more water. Otherwise, our breast will not have milk for our babies.

**P9:** To have more milk in our breast, we have to eat good food and we have to drink clean water. This makes us to generate adequate breast milk for our babies and on the other side that milk make us strong and healthy. It is food. It is hiwet [hiwet = life]. Sometimes, as this is a rural area, we women prefer to accumulate our farm products with the thinking that it will help us lead our life when a challenging time comes.

**I:** What does challenging time refer to?

**P9:** I mean drought.

**I:** When you eat food while you are lactating, what types of foods do you prefer?

**P6:** Potato, Salad and Iodine.

**P10:** Potato, linseed, milk and cheese.

**P8:** I eat every food item.

**I:** But, which foods do you think are most important for you as a lactating mother? The point is lactating women.

**P2:** Mmm…, Linseed, honey and milk.

**I:** As lactating women, what types of foods do you avoid?

**P5:** I have no list to exclude. I eat all food types prepared and consumed in our community.

**P3:** Every food is a food. I eat every food item common in Dasus. That is it.

**P9:** I eat, like what my sisters said, every cultural food common in our village. Sometimes, we are told not to consume dry foods like Teresho [Teresho is a food prepared from Sorghum and is well dried by heat]. Otherwise, I myself don’t bother about which food to eat and which to avoid.

**I:** You said ‘we are told not to eat dry foods.’ Who tells you this, not eating dry foods?

**P9:** X and Y[X and Y refers to the two health extension workers who are currently working in the health post of the kebelle where this focus group discussion is conducted].

**I:** What else can you share us?

**P11:** I don’t know it.

**I:** Okay, you don’t know it. But, is it due to the fact that health extension workers don’t educate you while you visit the health post or what reasons can you tell me?

**P11:** Off course, the health extension workers give us health education. But, they specifically don’t tell us what type of foods to avoid. They tell us what foods to eat, what to do when we are lactating mothers; not what not to eat.

**I:** Have you ever gone for routine nutrition screening services? What can you share us in this regard?

**P9:** Yes. One day, I took my child to a campaign to receive vitamin A. It was done in our community by health extension workers and other representatives from woreda health office.

**I:** What challenge/s have you witnessed?

**P9:** Nothing. I brought him from and he got the service. May be the distance I travelled.

**I:** Who else can share us her opinion?

**P5:** After delivery, we go to health post for healthcare services. Most of the time, they advise us on how to use food and the amount and type we have to eat. This is a good service for us. It helps for us and our babies.

**P7:** One day, health professionals from Mekelle have come to our kebelle and measured the arms of our children and their weight. They told us that it is very important to feed our children with variety of food. They told us that proper feeding of our children will make them very strong and healthy.

**I:** Any other idea?

**P2 and P4:** They swing their head sideways.

**I:** Do you a new idea, P4?

**P4:** I don’t have. I don’t have additional ideas.

**I:** What about the safety net program ~ do you think that you lactating mothers are beneficiaries of the program?

**P6:** Definitely. Since we are free any safety net activity, we are benefieries. It is good for us.

**P2:** Yes. Our government is helping us by making us to benefit from safety net. It is additional source of income for us. We are happy.

**I:** Any other idea?

**P12:** In general, our government is helping us by safety net program. But, there are some problems as they don’t show fairness [they refer to kebelle leaders].

**Perception of age at first birth and birth spacing**

**I:** Do you think that that delaying the age at first birth to 18 years and after is better for the best of health of the mother and the baby?

**P6:** Yes. You know if we gave birth before 18, it is not good for the baby and the mother herself as it could bring about a bad consequences in terms of health.

**P9:** The idea is good because in our community, such kind of problem was very common especially in the last one decade where many adolescent girls were forced to get married to man who they don’t know it. It was a harsh time as the consequences coming from it were bad. Anyway, our sisters should get a sort of help from the government.

**I:** What else, any new idea?

**P1:** Our sisters should work to delay the age at first birth. Off course there are some factors that hinder this like religious and cultural issues.

**I:** How does religion and community culture affect the age at firth birth?

**P1:** Elders say a baby born of mothers at early ages will be active and healthy. The baby will be good in work and even in education. That is what our elder fathers and mothers say. It is good to hear such things as this have a bad result.

**I:** A different idea?

**P7:** That is it. I share all the ideas shared by my sisters. I don’t have a different idea.

**I:** Is the message regarding the age at first birth being promoted properly in this community?

**P12:** Em…..Yes, I don’t think the message is properly conveyed to the community. Of course, health extension workers sometimes conduct health education at community meetings and churches. But, we didn’t see any change at all. The effort is less and not effective. Even sometimes our women development army share us the message though the desired result is not still achieved.

**P8:** Health extension workers, to your surprise, are the only promoters of age at first birth. It is true. No other stakeholder is helping them. Therefore, I cannot say that there is a good promotion is being made in this kebelle as only the health extension workers are being concerned by the agenda of age at first birth and birth spacing. Almost in each of our visits to heath center, health extension workers tell us about family planning issue. It is good to get such service from the health extensnion workers. We are happy. But, the promotion activities are poor; only done by a single stakeholder, that is, health extension workers.

**I:** What additional promotional activities can be done?

**P5:** For me, the promotion is good but there are some issues that should still be addressed. Sometimes, the message of birth spacing and age at first birth should be advertised using a press and even through radio and TV services. Only the health extension workers should not be forced to work for this issue. Radio and TV messages are also very important. TV and radio are government agencies. They are of the government. Therefore, what is the problem with regard to this issue**?** I don’t think there is a problem.

**P2:** Yes. Sometimes, the issue of age at first birth and birth spacing should advertised using even posters and school messages where a very effective work can be done and a good result can be accomplished.

**P11:** For me, doing promotional activities pertaining to birth spacing and age at first birth at meetings, and other social gatherings is also very important. Definitely, a good job should be done in this regard.

**I:** In this kebelle, what type of things are done and being done to address the communication and information gaps that exist pertaining to women‘s and adolescent nutrition?

**P7:** It is only the health education that we get while we visit our health center. As to me, there is no anyway of conveying message to the community about maternal nutrition.

**I:** Any other idea? The discussants remain tight-lipped indicating that we our running out of time.

**P2:** The additional idea I have is it is time for us to go home *[participant looks like joking and laughing].*

**I:** Go on no 2; what additional idea do you have?

**P2:** Health workers are giving us health education, like three times per month. They are really with us. It is due to the rural nature of this community that we don’t grasp the issues they taught us. Otherwise, they are giving us health education three times every month. Additionally, they are giving us family planning pills for three months and injections for three years.

**I:** Well, I have finalized my questions. Thank you very much for your participation and for your time.

**P:** Okay. Thank you. Thank you.

The end
