# Supplementary material for: Barriers to utilize nutrition interventions among lactating women in rural communities of Tigray, northern Ethiopia: An exploratory study
Source: PLoS One. 2021 Apr 30;16(4):e0250696. doi: 10.1371/journal.pone.0250696 (PMC8087028; doi:10.1371/journal.pone.0250696)
Supplement: S2 File — (ZIP) [file pone.0250696.s002.zip › S2_File.Doc/Lacatating women_IDI & FGD/013_FGD-Lactating Women Dinka Kebele_Ofla Woreda.docx]

**FGD for lactating mothers** using the Guide for women of reproductive age and men (**Tool B**)

**Introduction:**

Hello my name is Haftay. I am from Mekelle University. Thank you for taking time to speak with us today. We are doing a research on factors that influence the nutrition of mothers and adolescent girls in collaboration with the regional health bureau and UNICEF. Your participation is very valuable. The things that you tell us will be used to improve nutrition programs and services for women and adolescent in the region and in the country. Your names will not share when we report our results.

However, I will record the discussion using and audio tape recorder so that we can capture all the ideas that are shared. I have several questions to ask you that we have prepared in advance, and we will ask you to say what you think about each question. To ensure the privacy of everyone here, we ask you not to repeat what to discuss outside of this group. The discussion will last for 1-2 hours. Do you have any question before I begin? If you think of any question as we proceed, please feel free to let me know. If it is all right with all of you, I will run on the tape record now.

**Section A: Details**

1. **Zone**: Southern
2. **Woreda**: Ofla
3. **Kebele**: Dinka
4. **Facilitator`s name**: Haftay Berhane
5. **Date of FGD**: 02/11/2017
6. **FGD starting time**: 7:00 PM
7. **FGD end time**: 8:00 PM

**Section B: Socio-demographic information**

| **S.No** | **Name of FGD participant** | **Age** | **Marital status** | **Educational level** | **Occupation** |
| --- | --- | --- | --- | --- | --- |
|  | Weizer Girmay | 35 | Married | None | Farmer |
|  | Aberash Asfaw | 23 | Married | None | Farmer |
|  | Belaynesh Mekonen | 24 | Married | Diploma | Teacher |
|  | Fintu Demeke | 37 | None | None | Farmer |
|  | Kasu Girmay | 24 | None | Grade 3 | Farmer |
|  | Alge Mery | 26 | None | None | Farmer |
|  | Chekolwa Adelu | 24 | None | None | Farmer |
|  | Mulu Getahun | 25 | Married | Grade 8 | Farmer |
|  | Asefu Fantaye | 36 | Married | None | Farmer |
|  | Gemebr Negash | 26 | Married | None | Farmer |
|  | Beletu Semi | 32 | Married | None | Farmer |
|  | Tiwres Getaye | 25 | Married | None | Farmer |

**Keys**:

**I**: Interviewer

**P**: Individual participant

**Ps**: participants

**Agewgna**: A language spoken by the village

**Section 1: Common maternal (pregnant and lactating women), and adolescent girls` nutrition problems in the community.**

**I**: What do you think should a mother do to stay healthy? What do women do to be healthy?

**P-5**: If I think something wrong is happening to my health, I should go to health institutions. If I became a pregnant, I will be going to health institution to start screening and examination. The follow up should go until nine months without any interruption. It is to mean until the time of my delivery.

**P-10**: The first thing is keeping your personal hygiene. If a mother is pregnant she should visit the health institution regularly.

**I**: What should a pregnant mother do to stay healthy?

**P**: She should visit the health institution every month. She should also eat different variety of food. She should keep away from mosquito bite and she should do this to be free of malaria.

F: (Reminding the question) What should a pregnant mother should do to stay healthy?

Ps: Silent

F: What should a lactating mother do to stay healthy?

P-12: If a lactating mother is keeping her personal hygiene, she should take her child for immunization and she should also be vaccinated. She should also away from pests. The child should also start complementary feeding after six months.

**I**: What should the pregnant mother do to keep herself healthy? I want just to know what she should do for herself?

**P-12**: She should take different variety food.

**I**: What type of food should the mother take?

**P-12**: She should take teff even if we do not have it.

**I**: What is the experience here? What do you do when you are lactating mother to stay healthy?

**P-8**: We eat what we have. We do not choose.

**P-3**: She should eat porridge. She should also take iron containing foods like teff.

**I**: Any other idea

**Ps**-Silence

**I**: What should adolescent girls do to stay healthy? Those girls of age 10-19 year who do not give birth and who never became pregnant?

**Ps**: Speaking each other with Agewgna (Most probably about the answer of the question raised)

**P-7**: (Speaking in Agewgna); One adolescent girl should take birth control

**Ps**- laughing

**I**- What else?

**P-4**: if an adolescent is going to be married…

**I**-What should the adolescent do to stay healthy?

**P-4**: She should keep her personal hygiene and environmental sanitation. There is no need to take birth control pills. If an adolescent wants to continue her school, we do not push her to get married. If she wants to be married, she will be weighted and then if she is ok, she will be married.

**I**- What do you think are the common nutritional problems that are seen in your community?

**Ps**- Silence

**I**-Is there any child or adolescent who is having nutritional problem here? Mean have you ever seen a child or adult who is severely affected by malnutrition?

**P-4**: A child may become thing if the child gets sick or if the mother is not having enough milk. If the child is thin, the group leader of the community will take the child to where supplementary food is given after it is assured that the child has a shortage of food.

**I**-Which of the following nutritional problems do you observe in your community? Are they individuals who are severely affected by malnutrition or those who become thin?

**P-4**: Yes, there are but we take them to health institutions to get food support.

**P-7**: Yes, there is since there is shortage of food.

**Ps**- Yes there is

**I:** What about anemia, night blindness or goitre?

**P-3**: Yes, there is. We have individuals with goiter

**I**: Is there a problem of Anemia, Night blindness and goitre in this community?

**P-7**: Yes, there is anemia.

**I**: Others? What do you think?

**P-2**: The problem is there in the community but we, those of us participating this discussion, do not have such problems.

**P-12**: For example, myself, I have a small goiter but it is very small.

**I**-Are there diet related none communicable diseases like hypertension, Diabetes mellites in this wereda or in this community?

**P-6**: Yes, there are

**Ps**:Yes there are

**I**: Is stunting common in this community especially in adolescent girls?

**P-4**: yes, there are small children for their age.

**I**: In most of the case, are these adolescents small or tall to their age?

**P-4**: There are many children who are small for their age. However, when I say small, it is difficult to predict where they are going to increase height latter or not.

**I**: Are there adolescent girls who are small for their age because of nutritional problem?

**P-8**: Yes

**I:** Other ideas?

**Ps**: Silent

**I**: Are there over weight adolescent girls in your community?

**P-8**: No, there no. Almost all of them are short and thin. There are few who are normal otherwise most of them are thin.

**P-4**: There are three mothers who are obese.

**I**: How is the food security in this area? Did you get enough cereals from your farm that serves you for a year?

**P-3**: Most of us are dependent on farming. It is not bad.

**I**: Did you think you have collected enough food for this year?

**P-2**: The farm product of this year is lower than last year.

**I**: Was there enough food from your farming in the previous year?

**P-2**: Last year, it was enough but this year there is shortage of yield.

**P-4**: Here, only sorghum can grow. Our land does not grow any other cereal than sorghum. Last year there was enough sorghum for a year but it was destroyed by pests. However, this is year there is not even good yield of this cereal.

**P-5**: There is no enough food this year even compare to the last year.

**P-9**: There is no enough yield by this year.

**I**: who do you think is mostly affected by the nutritional problems? Pregnant, lactating mother or adolescent girl?

**P-8**: The lactating mother is mostly affected?

**I**: Why do you think is lactating mother mostly affected?

**P-8**: As she is breastfeeding a child and the child may also disturb her not to eat enough food. The child is sharing her.

**I**: who do you think is mostly affected by food insecurity or nutritional problems?

**P-12**: If a mother is lactating the government may help her otherwise she may go for food search to other areas. However, if the mother is pregnant, she will not go anywhere and then she will eat being in home.

**I**: What about others?

**P-11**: Pregnant is mostly affected by nutritional problems?

**I**: Why do you think is pregnant mostly affected by the nutritional problems?

**P-11**: Pregnant cannot work and she will become weak.

**P-4**: Both pregnant and lactating mothers are affected. This is because, if a mother is lactating the child will need food which will share the food she has. But The lactating mother is mostly affected.

**P-3**: Shows signs of disagreement. The most affected is the pregnant one. Because, the pregnant will need different variety of food. She cannot eat all food items. The pregnancy by itself will prevent her from eating all types of foods. However, lactating woman can eat whatever she got. Therefore, the pregnant one will be mostly affected.

**Section 2: Barriers to access and utilization of nutrition services**

**I**: What are the barriers that hinders a pregnant mother not to access maternal services? Eg. Do pregnant women receive an advice about what type of nutrition she should eat and how she should prepare?

**Ps**: with low tone; Yes, it is.

**P-9**: They advise us to take liquid, honey if it is available. They told us to take clean food. However, we did not get all these things.

**I**: Did you receive a tablet to prevent anaemia when you were pregnant?

**P-13**: Yes, I took

**P-9**: yes indeed

**I**: Is there any obstacle that prevent you not to take the tablet for prevention of anemia?

**P-7**: when we manifest with head ache and sign of falling, we go to health institution and they give us a tablet. It is after we receive the tablet that we get relief. We have enough access to these medications.

**I**: Do you have barriers that hinders you from getting these medications?

**P-12**: I was having headache even when I had a honey at home. I was drinking more than a jerican water. When I went to the health institution, I was fine with short period of time.

**I**: What are the obstacles then?

**P-13**: There is no obstacle.

**I**: Did you receive any deworming while you are pregnant?

**P-13**: I brought a medication for deworming of my child but he is still not fine.

**I**: Do all children in this community measured their weight?

**P-2**: Yes, all children are weighed.

**I**: Did you receive enough rest while you are pregnant?

**P-4**: In our community, there is soil and water conservation and safety.net. If a mother is known to be pregnant after she screened in a health institution, she can take 12 months of rest while she is pregnant and 12 months rest while she gave birth.

**I**: Do you think all mothers and, adolescent in your wereda screened for nutritional problems?

**P-8**: Yes, there are many things they do for them. If they are weighed and found to be with nutritional problem, children can receive a feeding (indicating Plumpy Net). If we go being sick to the health institution, they give us only tablets to swallowed. If a child develops an eye disease, there is no eye ointment.

**I**: What about you? Did you all weighed while you are a pregnant?

**P-11**: Yes, if there is a nutritional problem, they are proving us plumpyNet but if it is not severe they give us `Fafa` (a food supplement for MAM). They then follow us every month and if you are ok, they stop supplying these supports.

**I**: Have you ever get an advice on healthy eating, diversified meal to you and your child?

**P-5**: We are getting an advice on how to eat diversified meal prepared from variety of cereals including wheat, teff, and bean to be mixed with egg and prepared in the form of porridge. However, there little chance to get these foods in our community.

**I**: What about others? Do you think different type of food is found here in the community?

**Ps**: No, it is not found.

**I**: What type of salt are you using?

**P-12**: We use iodized salt.

**I**: Is iodized salt available widely?

**P-4**: All communities are utilizing the iodized salt. It is widely available.

**I**: Have you been told about the nutritional sensitive agriculture such as home gardening, production safety net program, food security, or work load reduction to prevent nutritional problems?

**P-4**: I want to tell to shorten the discussion. We were happy to plant home garden. But we do not have water source starting from September to June. We brought fruits and vegetables from the near bye town like korem, zata and sesela (nearby villages with good water source).

**I**: Others

**P-7**: Since we do not have water we cannot do it.

**I**: How do keep your personal hygiene, and of your kids?

**P-2**: The first health workers were telling us how to keep our personal and environmental hygiene but now, there no hygiene.

**I**: Why are not keeping your hygiene right now?

**P-2**: The current health workers are not advising us to do so.

**I**: Other Idea? Why are not keeping your personal and child hygiene?

**Ps**: Cross talk

**P-3**: They are not answering what you are asking them. You are saying something but they are replying another thing. I think they are not understanding it clearly. (She tries to translate the question to Agewgna)

**I**: Is there anything that hinder you not to keep your, your child hygiene?

**P-3**: The problem is lack of water. For example, we use water by buying from far area. If an individual is poor, she may not buy the water and she will not keep her personal hygiene.

**I**: Did you use insecticidal treated bed nets?

**Ps**: Yes, we use it.

**P-12**: We were using it but not now.

**P-4**: We have been using the bed nets but it was given to us before three years. Since it can be dirty, we used to wash it and it will no more be useful. We understand the advantage of the net but currently there is no supply.

**P-2**: We are using it but since we washed it I think it will not be effective.

**I**: Why are you not using new insecticidal treated nets? Is it because you did not go to the health center and request for new one?

**P-4**: Every has been using these nets. The nets were distributed before three to four years but currently there is no supply from the region.

**I**: What do think should be done to improve the above-mentioned issues, for example, shortage of insecticidal treated nets, iron folate, deworming and nutrition?

**P-3**: There should be new supply of bed nets. For example, a pregnant woman should not be bitten by mosquito and since the chemical of the bed nets is gone by washing, there should be supply of new bed nets.

**I**: Others

**P-7**: There should be a clean water supply. There should be a clean water in this area because our children are suffering from intestinal parasites since we are drinking un cleaned water.

**P-4**: I haven`t seen medication for deworming here. I am hoping you guys will bring to our health center here. you may not be responsible to bring this but report to the higher bodies

**Section 3: Perceived needs of women for relevant services during pregnancy, lactation and adolescence**

**I**: Now you are going to tell me what should be done to adolescent girls, pregnant and lactating mothers? What should be done to these to visit the health institution and get screened.

**P-3**: Education should be given to the mothers by health extension workers.

**I**: Where did you think the appropriate place for the education?

**P-3**: The mothers should have gathering for this education.

**I**: Others?

**P-4**: we have gathering with the pregnant and lactating mothers every 15 days for immunization and the education can be given in the gathering. For the adolescents, there should be a meeting with them and they should be told about how to keep their health, screen for any disease and advise them as health is about everything. There could be a meeting with the adolescents every month.

**I**: Good. What should be improved for a pregnant and lactating mother to take extra meal?

**P-12**: If the pregnant women get wheat she can make it porridge and if she can get teff she can make it bread. It is also good if she gets foods in the form of liquid. If the government helps us, and support us with the supply of foods, we will be healthy.

**P-11**: If a mother gets pregnant, she should eat soft foods.

**I**: In this area, when a woman gets pregnant, do you think she changes her diet?

**P-1**: Since we do not have foods for change we usually eat the same food.

**I**: Other ideas?

**P-8**: We used to eat only sorghum.

**I**: Others

**P-2**: She may change the diet as she is pregnant and may need variety of foods. The issue is on the availability of food for changing.

**I**: What did you personally when you were pregnant? Have you been eating the same when you were pregnant? Did you think lactating mothers should change their diet?

**P-3**: I was eating different food types while I was pregnant because, not every food was suitable for me. But now as lactating mother I am eating whatever food I get.

**I**: Other ideas?

**Ps**: Silence

**I**: Did you need more or less amount of food while you are pregnant or lactating?

**P-8**: I was getting less food while I was pregnant. When you are pregnant, you may need variety of food but if you get a small and clean food that is enough.

**P-12**: I used to eat small amount of food while I was pregnant. This is because, I was full with the fetus. However, I was eating more after I give birth.

**I**: Which foods did you think are advisable for pregnant women?

**P-12**: Small bread, soft drink and this will help her later during delivery.

**P-3**: A pregnant women should take variety of food like teff to prevent occurrence of anemia.

**Section 5: understanding perception of age at first birth and birth spacing**

**I**: When should a mother have a next pregnancy? You may have heard that it is better for both mothers and babies if a woman waits at least 2 years after birth to become pregnant?

**P-5**: The birth spacing should be 2 years. For example, my first child is 4 years and this one is two years; I have born this child after 2 years.

**P-12**: The birth spacing is three years in my experience.

**I**: In this community, when is an adolescent getting married?

**P-2**: Adolescents are getting married just after 18 years.

**I**: Over all discussion was good. If you have anything to say before I close the discussion?

**Ps**: Silent

I: Thank you for taking the time to discuss these issues with us today. We have learnt a lot from you. As we mentioned as the start of the discussion, we will remove all identifying information from the report of this conservation. We will make you sure that no one can identify your comments. If you have any concerns or questions, please feel free to ask me any questions. Thank you very much for your time.

**Summary:**

**Section 1**:

- There are many nutrition related problems in pregnant, and lactating mother as well as in the adolescent girls.
- In their perspective, lactating women are mostly affected

**Section 2:**

- There is lack of awareness
- There is lack of water
- There is also lack of supply

**Section 3:**

- There is no habit of changing diet during pregnancy and lactation
- Rest and visiting health intuition are things that should be done to pregnant mother to stay healthy

Section 4:

- Pregnant mothers are beneficiaries of safety net program
- All pregnant women are supported with a nutritional program

**Section 5:**

- The common age for marriage in adolescent is 18 years and above
- Mothers are applying child spacing protocols using family planning

**Section 6**:

- There is meeting every 15 days which include education about nutrition
