# Supplementary material for: Barriers to utilize nutrition interventions among lactating women in rural communities of Tigray, northern Ethiopia: An exploratory study
Source: PLoS One. 2021 Apr 30;16(4):e0250696. doi: 10.1371/journal.pone.0250696 (PMC8087028; doi:10.1371/journal.pone.0250696)
Supplement: S2 File — (ZIP) [file pone.0250696.s002.zip › S2_File.Doc/Lacatating women_IDI & FGD/015_IDI- Lactating mother_Hashenge_Kebele_Ofla woreda.docx]

**Day2: 25/02/2010 E.C**

**In-depth interview of Lactating woman**

Woreda: ofla; Kebele: Hashenge

Socio demographic information

|  | **Socio demographic information** | | | |
| --- | --- | --- | --- | --- |
| **Sex** | **Age** | **Marital status** | **Education level** | **occupation** |
| female | 37 | divorced | No formal education | merchant |

Translation 01 – IDI

**Section1: common maternal nutrition**

Q1, I: What do women do to stay healthy in the community?

P: is this for only me or for other women?

I: All women including you

P: from the beginning during conception, woman should be examined, and take contraceptive three or four times and prevent pregnancy. Then to keep her health, she needs to keep her environment clean, keep personal hygiene and wash her cloths. Besides, constructing toilet and utilization properly will let the pregnant women healthy and will delivery safely. After delivery, she has to eat foods three times, four times. At morning breakfast, lunch and at night dinner is necessary. After delivery, for the baby, he must be vaccinated three times until 9 months. And until 6 months he must be breast feed, without drinking water, and taking no food. After 6 months, a ‘mitin’ composed of three fourth from local and one from other, that is from misir, bean, Ares and other should be prepare for him.

I: what about for lactating mothers? What do they do? For example, you are lactating mother, and what do you do to keep your health and your child’s health too?

P: For our self, I breast feed my child daily and I do not have to let him feed and hug by other people. I have to taker of him myself, wash him a care him always.

I: what about for the mother?

P: she must be clean, and wash her body.

I: what about in terms of nutrition?

P: To breast feed her baby, she must eat a variety of foods and must. Sufficient and proper feeding is good until the child starts to walk. My baby, for example is one year and two months old. Because I feed him well for 6 months only breast milk. After 6 months, I provide him additional different foods two to three times usually ‘porridge (GEAT). Now he can walk and run independently and he is fine. If an individual did not keep his health, we are observing a lot of things happening in our community. If people did not keep his personal and environmental health, if they do not have toilet and if their babies are not clean, there are a lot of health problems occurring.

I: what about for adolescents that are 10-19 years old, especially girls, what is being done to keep their healthy?

P: first, they must be well educated and be clean. At young age, they should care of themselves. At age of 18, marriage is possible. I have a girl, and she is student and now I was sick entire begging of my girl for marriage by other people. At grade eight, when two year is left to complete, I let her marry to someone and now she already withdrew it. “This is what a woman does. Because I was divorced from my husband after I gave birth to her, I care her lonely for 18 years as if it is one day. “Uhfffff” It has been 6 years since her marriage, and I was helping her husband to learn. Because I am woman, he prefer to divorce. Now she always criticizes herself and we feel as if we lost our respect.

I: what are the common nutritional problems in this community for women and adolescents?

P: So far, what we observe is a lot of problems. There was malnutrition in many households. This is related with not having a food and not knowing how to prepare it. But now, an educated people like you are coming and telling us what to do, there are a lot of improvements here and in other areas. But some years back, there was a number of problems duet to hunger. But now, because people are work to eat, there is no any problem.

I: Are there children who take ‘fafa’ and other who are screened as being thin and taking plump net?

P: Yes, there are a lot.

I: What about other?

P: Yes, there are, especially women who give birth. For example, I was taking ‘fafa’ three times when I give birth and I ate it in the form of Porridge. But, when my baby improved, I stop. And there are a lot of women who take for themselves and for their children.

I: what about any problem related with anemia, night blindness and goiter?

P: In our surrounding, people come to health facility when they have the problem.

For those who are too stunted children, health works are giving them ‘fafa’ and to other who are sick, we are getting treatment here in this health center. In our kushet, had it in previous scenarios, there could have been a lot of problems, but now there is improvement.

I: what about problems related to underweight, overweight? And other non-communicable problem on women and adolescents?

P: It is fine

I: what about any problem related to food insecurity? Do people have enough food that can serve from year to year? Or is there any shortage in summer? Or any one on nutritional support?

P: yes, there are. The government is giving support some time. Everyone is not similar in having sufficient food. There are people who work for food, and there are some who cannot. Thus, there individuals on safety net, those are insecure and are not eating daily. Mainly, the old people and some lactating mothers are getting support.

I: Which groups of women are affected by the above nutritional problems? Pregnant women, lactating or adolescent?

P: The problems are common among lactating mothers. In terms of support, all women are not getting it. Fafa is primarily give to women who have severely ill children and those who are highly devastated women. All are not getting support, eg if there is a child in need of it, why do not we give him might be raised, but it sot, it primary focus to those who chronically affected child and his mother as t given from the government. So the problem is common among mothers and lactating ones.

**Section two: nutrition priority in the woreda**

I: Do you think that you can contribute in solving at the above problems on maternal nutrition? At least to your neighbor and community on their nutrition and health by providing advice and information

P: In my surrounding! Yes, I do. First, I have to be to myself in keeping my environment and children. When there is someone who sits next to me, we are too much friendly, and I tell them to build toilet to these who do not. I advise them to clean their environment and not to dispose any waste in home yards. The disposed wastes my expose us to disease. Last week, people from Woreda came and checked it and they got nothing dirty as we had cleaned and disposed it in a pit. We advised to people as per our experience.

I: what interventions are being done in this community/woreda to improve maternal nutrition? It could be from the health sector or agriculture sector

P: is it by the government?

I: yes

P: the government is providing us fafa, direct support, and safety net. And these are what I know. These are what the government is helping us. The fafa is very important to children, and the quota (direct support) is important to who are poor and on hunger. These are the three things what I know. I did know another.

I: which one is most widely given intervention from those you have mentioned so fa?

P: safety net. Quota (direct support) is given when there insecurity.

I: is there enough resource for the safety net?

P: it is based on your number of households, if you have two family members; you will be given for two. If you have ten, you will be given for ten. So, it is based on family size. If you are one, you will be given for one; on addition, no decreament.

I: what are the maternal nutritional interventions that brought change in your community? For pregnant women, lactating women and adolescent?

P: In the near past one to two year, safety net brought change. In the past, we were given money, but now we are getting cereals, fafa, Kinche and oil. This year , safety net is bringing change in the community. When there harsh condition like what is happened last year in Raya, the Woreda gave two to three quintal of cereals to each household. Fafa is given to pregnant and lactating mothers. They are use in the form they want. But last year it was not available. Now we benefitting form it.

I: who are more beneficial for this?

P: children and pregnant women, and later lactating ones. Lactating mother should use for herself to benefit her child. After six month, the child will be given his quota. If the food is prepared by an excellent mother, it will be enough for her and the baby. The child is improving when using fafa after 6 months. So it is very important.

**Section3: nutritional interventions that improve adolescents and maternal health**

I: what nutritional intervention in place for pregnant women?

Such us, advice on ANC services (eg Iron folate supplementation, deworming, and weighting?

P: Yes, the measure them and gave them fafa accordingly.At month three, they give us a lot of drugs eg, the red one they gave us in ‘derzen’ for anemia, . They gave us three times in a year. At 9^th^ month, they give us food in the form of medicine.

I: what about advice / counseling on extra meal and rest during pregnancy? How many times to eat, when should she get rest?

P: Pregnant woman should eat food of different types three, four times. That is breakfast, lunch, snack and dinner.Over all, four times so that you will not be endandere while giving birth. They told us all the aforementioned information.

I: what about nutritional screening or measurement on their upper arm?

P: yes they made the measurement. All pregnant women are screened well all the time.

I: what else? Example, use of diversified food, what is beign told and advised? Use of Iodinized salt, home gardening?

I: Do get advice on these?

P: Yes, we get. In the irrigation we have, we produce tomato, paper, and everything is there and we used it. We used the packed salt what is called ionized. We use vegetables.

I: who gave you these advises?

P: healthcare workers. They go home to home, they deliver us such information. They advise us, to use cabbage and tomato when eating Injera.

I: What about advice on water, sanitation and hygiene services?

P: There is a packed water treatment medicine, and they advised us to one tablet to the pot and use the water. When we finish, we add one drug to one Jerikan of water. By doing so, we keep our health.

I: What about ITN distribution to pregnant women?

P: We do not have ITN.

I: Why? Is there mosquito here? Is malaria common here?

P: there is no mosquito and malaria. We hear that there is malaria in Raya/Mokoni. But, there are some people who own ITN. They bought and they pack and put it. No one distribute ITN in our community. In Raya ITN is distributed, because it is malarious. When they sleep under it, the mosquito will not enter.

I: Are all these intervention we discuss in place to lactating women like you?

Example, counseling on extra meal and rest, MUAC, use of iodine, food diversity

P: I am doing all these things, including the women surrounding me.

I: how many times have you been nutritionally screen?

P: All the time; usually when the health extension workers come to home, they examine us and advise us.

I: what about vitamin A for your eye: the blue and small drug to be swallowed?

P: To treat water, they come every month to give us the drug. But, for my eye, I was given nothing.

I: What about deworming service?

P: If we have parasite, we go to the health center and got treated there accordingly.

I: what about at home to home or at any communal place?

P: No deworming at home. Just when they came home, they tell us to go to health facility if we get sick. Besides, when we want contraceptive for three, 6 months, 9 months or 3 years, the came to our home and give us the service.

I: Do lactating women participate in food support, productive safety net program (PSNP)? Or did you food support freely if you are lactating?

P: No, there should be someone who can work for you; he can be your husband or son/daughter.

I: how about adolescents? Do they nutritional screened?

P: yes

I: what type,

P: They are advised at school, to use contraceptive.

I: what about weekly provision iron folate?

P: I did not see/hear about this, because I do not have adolescent now.

I: what about deworming service?

P: I did not exactly know on what happen, what I know is they (HEW) always inform and educate people.

I: what about the use of Water, sanitation and hygiene services?

P: they are informed at school

I: what about to the out school girls?

P: Their parent might advise them, because they can observe what is happening on their neighbor?

I: In your opinion, which of the above programs are being implemented successfully in pregnant women, lactating women and adolescent girls?

Example: Iron folate supplementation, deworming, and weighting? Counseling on extra meal and rest, food diversity, WASH and nutritional screening, etc in pregnant women, lactating women and adolescent girls in your kebelle/Woreda.

P: Fafa, safety net. Government is supporting the people. In the long past years, there was not fafa. I have two children without this baby, so then, I did not receive any support. But now, there is fafa and safety net. It is with all its ‘Tsebhi’ and salt. The government has improved it. In one side to pregnant woman and in onside to lactating women, and if the child is well feed and satisfied, it is good.

I: why do think is the reason behind the success? It because of the HEW? Or Woreda or kebele? What is the reason?

P: yes it the strength of the government and an educated people like you are helping and informing the community. Now, I am thankful to government.

I: which of the programs mentioned above are less effective?

P: there is no weak program

I: think program that need to be done to pregnant, lactating women and adolescent

P: for example, if my baby is getting fafa, other child may strive to be given; so it is good if fafa is given to all children in the community. The sick is eating, what is the problem if the healthy ones eat too? If the government brought it, it must be give all children home to home.

I: any issue on pregnant

P: No problem because pregnant women are getting every service.

I: what about adolescents?

P: there is no problem.

I: what about for out-school girls? how do they get the service

P: Health care workers come home to home give health information on WASH and vaccination.

I: are there challenges in implementation the nutrition interventions for pregnant, lactating mothers and adolescents? It could be lack resource, example shortage of ‘fafa’, lack of awareness and training gap? Others?

P: No, in my observation, I have not seen any problem. If a baby is born sick, he is being treated. Pregnant women are being followed properly starting from here in health center to the korem for referral. They are very cooperative to pregnant women. In this one to two year, everything fine and good.

I: Are there challenges specific to pregnant women? Example on advice on antenatal care services (iron-folate supplements, deworming, counseling, weighing)

P: Yes, there are problems. For example, there is bleeding during delivery at an individual’s home. But there are efforts to decrease the problem. But if she is dead we can do nothing.

I: what about any challenge on advice to take extra meal and rest?

P: there is no gap, they always tell us every morning on diet how to prepare and eat.

I: what about nutrition screening (using MUAC measurements and manage accordingly)?

P: yes they are measured every pregnant.

I: is it always? Or is there any challenge that you herd from your community?

P: now it is fine, even when she has bleeding, health care works will let her stay at health facility to treat her.

I: What about on the use of variety of foods?

P: ‘Do not think all do have it’ every do not have ample food.

I: what about those who have all the necessary foods?

P: with all the advices we get to eat ‘this and that’; those who have can eat 4 times, 5 times. But those, who do not, will eat only once or twice if possible. But there some people who are tired and lazy to prepare variety of foods despite the availability. But, if it is good, because it for myself, I can prepare it well. Generally, it is not equal for everyone, my dear. People say ‘I will not eat’ is because they do not have it. For example if they want to buy an onion, potato, and tomato and other things, “how can I do it if I do not have money?” it will not be fulfilled. If I can do it, I will do it. The poor may say “I will eat” but “from where is he going to bring it all?” just nothing to do.

I: how about challenges related to water, sanitation and hygiene services?

P: no problem at all, they are always telling us to be clean at all.

I: What about all the above issues to lactating mothers? Is there any challenge?

P: yes, there could be problems with mothers, but the services are okay. HEWs are all the time with us.

I: what about on adolescents? Do they have similar focus like pregnant and lactating mothers?

P: what kind of focus?

I: for example pregnant women will be continuously screened and advised accordingly, and she even can be provided fafa. What about in case of adolescents?

Is there school feeding at school? Is it continuous? Thus I want to tell me any challenges regarding this and others?

P: At school, student get different information and health information, but I do not know whether there is school feeding or not.

I: what about regarding weekly iron folic acid supplementation?

P: I do not know about it

I: When there are challenges, what do the Woreda have used to improve maternal nutrition service for pregnant women, lactating women and adolescent girls?

P: For some mothers and old people, the Woreda provide direct support sing quota system. For the adolescents, there nothing to be offered to them. I do not know if there is. But for mothers who have child there a quo to given to them.

**Section 4: Community factors affecting access to maternal nutrition interventions**

**I:** Can you think of barriers that prevent adolescents and women from using the programs and interventions that we have discussed? May be because of Education, transport, workload, awareness, quality of care, community beliefs, others?

P: yes, there are. For example those who don’t have money to cover transport may not get service.

I: what else?

P: Those who do not have money is because of their poverty, but those who the potential are not using the programs because of lack of awareness. But the poor should be supported by the government, and the community should help each other as other people’s help is important.

**I:** How can these barriers be addressed to improve maternal nutrition in the community/Woreda? For pregnant women, lactating women and adolescent girls

P: To whom?

I: To all type of mothers

P: To the poor, support is very important, people should collaborate each other. And to the one who Lack information, she must be supported informed accordingly

I: can you add more in relation to pregnant women, lactating women and adolescent.

P: For pregnant women, they need different foods, they have to eat and drink and screen for their status. And need support on these issues. Pregnant women should eat three times, four times.

**Section 5: Other interventions that influence adolescent and maternal nutrition and health outcomes**

**I:** with your understanding, why would increasing the space between each births and delayed marriage (after 18 years) improve maternal nutrition and hence both maternal and infant health?

P: To give birth, it should be after staying three to four years until the baby grows well and be strong.

I: what about the advantage to the mother?

P: Within three years, the mother will breast feed one child than two, and she will not weaken physically. Thus it is good both to the mother and child in general.

Regarding the marriage, for example I was married at age of 10 years. Even though it is no happed on me, there are many who face difficulty while giving birth and may face fistula. In the past girls were marring at age 10 year if they are daughter of a rich person. At that time, they were facing a lot of problems. But now marriage is allowed only after eighteen years. If she is married at 18 she may give birth at 19 and she will be health for herself. So, it is advantageous to get married at 18 years for both the woman and the baby.

I: What programs or activities in place to promote these things in this woreda? That is to increased birth intervals and to prevent early marriage?

P: They teach us while they came to our community. If a woman wants to increase birth interval, there is contraceptive to be used for three years and even more, starting from three months based on her choice. If she wants for three years, there is injection for three years and if she wants for three months, there is injection for three months. And to the girls, they are advised to be careful and attend their education to avoid unintended pregnancy.

I: What happen to parents if they let their girl marry below eighteen years? Is there a law/policy that prevents this?

Eg what a girl/boy is married at age of 15 years?

P: there is punishment and jail at the woreda.

I: what about any religious influence? Is it supported or what?

P: the law is accepted by all, and no one will support under age marriage including religious leaders. Girls will be checked and weighted in health facility if she real is 18 years. If she fulfills, she will be married. If not, she will stay. There is no anyone who will marry his/her undercover.

I: Do you think that these programs or policies effective than before?

P: yes

I: Why?

P: In the past, girls were marring any one that she did not like. There were a number of divorces. But now, after they get married, they immediately give birth and they are living together. This time things are good.

I: what about the community? How do they determine age to marriage? Having in mind that the law determines the age to be eighteen?

P: The community agrees with age of 15 for girls, because she can give birth. This is what we have received form our parents. But now, it is 18 year as the governments declares it.

I: In your opinion, what could be improved to increased birth intervals and to prevent early marriage?

P: no thing. All are fine and these are bringing change in the community.

I: what can be done at school to prevent early marriage and increase birth spacing?

P: Girls should be advised to take contraceptive

**Section6: multi-sectorial collaboration to improve maternal nutrition**

I: Do you think it is good if different sectors/institutions work together to address/improve maternal nutrition? E.g. agriculture with health or justice.

P: No, they should work independently their own jobs. Because they have different activities, what is important is if they work individually.

I: I am asking you at sector level, for example which do you think is important, if health and agricultural extension workers came together or separately to advise you on nutrition and home gardening; which option do you prefer?

P: It is okay for me if they came together or separately, what is needed is the information that is delivered to me.

I: people argue that it is good if different sectors work together, how did you see if health and agricultural extension workers came at different time to you home for advice, would it be fine to you? Or are there any challenges that affect collaboration?

P: Yes it may work, but it is good if they came separately. This is because, they community will listen them well. If they came both sectors, it may work temporarily. But if it wanted to be understood, it is best if one of them inform the community.

I: take an example of planting carrot and eating carrot, what the about collaboration of health and agricultural extension workers? Does it sound to you?

P: No, I prefer if they teach me one after the other, so that I will not forget the information.

I: is there a condition when health and agricultural extension workers can successful collaborate to improve maternal nutrition?

P: Yes, it may be helpful to attend community health events at all at a time.

I: Is there anything that you want tell me to improve maternal nutrition (pregnant, lactating and adolescents) and overall health of the community

P: No. All things are mentioned. Pregnant women are getting care, adolescents are involved in school, and mothers are well supported. So, everything is fine; I do not have any point to be mentioned as the government is supporting the community.

I: Do everybody in the community know women’s health including their nutrition is a priority issue?

P: Yes, the community know it all and the government is supporting women and had provides all services.

I: ay thing more

P: NO

I: Thank you for your time and energy.

***Summary***

**Section1: common maternal nutrition**

To keep women’s health, they have to keep their environment clean, keep personal hygiene and wash her cloths. After delivery, a woman should eat foods three times, four times.

And until 6 months a baby must be breast feed, without drinking water, and taking no food. Lactating mother must eat a variety of foods and proper feeding is good until the child starts to walk.

There is malnutrition in many households. And it is common among women and children. Old people and some lactating mothers are insecure and are not eating daily; they are getting support.

**Section two: nutrition priority in the woreda**

The government is providing us fafa, direct support, and safety net. The fafa is very important to children, and the quota (direct support) is important to who are poor and on hunger. This year, safety net is bringing change in the community.

I: children and pregnant women are more beneficial from the support.

**Section3: nutritional interventions that improve adolescents and maternal health**

All pregnant and lactating women are nutritional screened all the time.

In the irrigation we have, we produce tomato, paper, and everything is there and we used it. There is a packed water treatment medicine, and they advised us to one tablet to the pot and use the water.

There is no mosquito and malaria. In this kebele and no one distribute ITN in our community.

Lactating mothers are not given vitamin A and no deworming at home.

At school, student get different information and health information, but I do not know whether there is school feeding or not.

At community level, there nothing to be offered to the adolescents.

**Section 4: Community factors affecting access to maternal nutrition interventions**

Mothers who don’t have money to cover transport may not get some nutritional service. Support is very important to poor women and, people should collaborate each other. Pregnant women should eat three times, four times per day

**Section 5: Other interventions that influence adolescent and maternal nutrition and health outcomes**

It is good to both the mother and child in general if woman give birth after staying three to four years until the baby grows

There are many girls who face difficulty and fistula while giving birth when they marry below age of 18 years.

If parents let their girl marry below eighteen years, there is punishment and jail at the Woreda.

**Section6: multi-sectorial collaboration to improve maternal nutrition**

Everybody in the community know women’s health including their nutrition is a priority issue and the government is supporting women and had provides all services.
