# Supplementary material for: Barriers to utilize nutrition interventions among lactating women in rural communities of Tigray, northern Ethiopia: An exploratory study
Source: PLoS One. 2021 Apr 30;16(4):e0250696. doi: 10.1371/journal.pone.0250696 (PMC8087028; doi:10.1371/journal.pone.0250696)
Supplement: S2 File — (ZIP) [file pone.0250696.s002.zip › S2_File.Doc/Lacatating women_IDI & FGD/026_FGD_lactating Women_Hashenge_Ofla woreda.docx]

Date : 05/11/2017/15/02/2010 E.C

Time:9:35-10:57 Am(Morning)

Palace: Hashenge Health center

Moderators: Beyene and Gebremedihin

Sociodemographc information

| Participant code | Name | Age | Marittal status | Education | Occupation |
| --- | --- | --- | --- | --- | --- |
| P1 | Nuru ayni ebrahim | 22 | Married | Illiterate | Housewife |
| P2 | Atrigya siraj | 23 | Married | Illiterate | Housewife |
| P3 | Alganesh Tilaye | 35 | Married | Illiterate | Housewife |
| P4 | Hilifti kebede | 21 | Married | Illiterate | Housewife |
| P5 | Gedete Girmay | 30 | Married | Illiterate | Housewife |
| P6 | Zenebu Birhanu | 18 | Married | Illiterate | Housewife |
| P7 | Sofiya mohamed | 19 | Married | Illiterate | Merchant |
| P8 | Wesek Abadi | 20 | Married | Illiterate | Housewife |
| P9 | Mebrahte Reda | 39 | Married | Illiterate | Housewife |
| P10 | Belaynesh Kashi | 37 | Married | Illiterate | Housewife |

M: Moderator

P: Participants

**Section one: Common maternal nutritional problems in the community**

M: OK thank you for coming voluntarily. The first point for discussion will be what people in Hashenge did to remain healthy? Is it clear? i mean what people do to keep their health. it can be mothers, lactating mothers or other members of the community?

P4: Ok! to be healthy people should eat clean and wear clean. they should also use iodine in their food.

M: Can you elaborate more on that?

P4: It can be cabbage. so it must be kept clean.

M: Ok p4 has mentioned her view

P7: First of all your environment should be clean, the house for the human and animal should be separated as it can cause a cough. then you should eat more vegetables . iodine salt shall be applied after the stew is made.

M: Do you think iodine is to be applied only at stew?

P7: Ya in stew. where can be used. It cab added to bread, milk

M: Well P7 tell us her view. others?

p2: rich will not develop illness?

M: How?

p2: He will be clean. then he cannot get ill and if you can feed what you have you will get ill

M: What else? keeping environmental hygiene were mentioned so far?

[children were disturbing at this time]

M: What did you say i cannot hear you please P2

P2: Your body should be clean, the animals[tirit] should have separate house. we are the same

M: Participation is very important. give more idea before i proceed to other point

Section two

M: I will go to the second question. what are the nutritional problems commonly seen among mothers, lactating women in this area.

P3: Regarding feeding , the infant should take only breast feeding until 6 months. he should not get any other food. then he has to take some additional food which is mixed type. made of wheat, sorghum etc. it can be given in the form of porridge. ever water should not be given until 6 month.

M: Well i come to issues related to infant feeding but now what are the common nutritional problems seen in lactating and pregnant women. for example, the condition of being thin due to feeding problem and getting treatment at health center in the form of planpnet, provision of Fafa for those who failure to gain weight, food support for the household etc. does these conditions present in your area?

P5: My child had this problem. he had low weight. then they gave me Fafa and i start to prepare and provide him 3 times a day and he now fine. the porridge was made of three parts of water and one part of Fafa. additionally i was preparing a food from the mix of pea, sorghum and wheat. now his appetiet is good.

M: When was this happened?

P5: After six month my breast mild was not sufficient to him and become thin. the once he start to take fafa he become fine. now he is 1 year and seven months old.

M: I am asking this kind of experience. others tell us your idea? Any child who is admitted for having low weight and problems relate dot feeding, children who are evaluated by health workers to be low weight or households who are food secured in hashenge. Let the children be free, they are ours but give your attention as well. is that clear for you. i am asking about any health problems caused due to nutrition such as being thin, low weight, slow gain of weight. are there children who use the packed food of that Fafa.

P8: Yes there are. we get these and tell us to feed them.

M: Are the number of this people large enough.

P8: Yes. i am taking Fafa currently

M:Why are given?

P8: I am pregnant.

M: Others please tell us your observation about these problems. do you see these kind of problems in your neighbor?

P8: No

M: Do you know that a person with malnutrition can face health problem too? i.e can pregnant, lactating women get health problem due to malnutrition?

P8: Yes

M: Who can get ill, the well nourished of the malnourished one? [no response]

M: Do you agree that malnutrition will cause a disease.

P45: Yes

M: if it is so, from adolescents, lactating, pregnant, infants which one are most affected by malnutrition. who are vulnerable to malnutrition?

P4: As to me the mothers breast milk might not be sufficient in amount for the infant. so the infants less than six months are more affected for such problems.

M: p4 is saying less than six month infant are more affected. this is her view. what about others?

P7: Malnutrition is due to feeding problem. it is not only loss of weight but also their body can swell. i see a case his body swells and was shining. He was referred to Michew and he was diagnosed as having malnutrition and get treatment there for two weeks. the health workers disposes the mother.

M: What do you mean by the mother was dispossessed?

P7: They said why you fail to feed him. what were you doing. you were supposed to feed varieties of food. After 2 weeks of treatment he recovered from the visible swelling and become normal.

**Section two: Barriers to access and utilization of nutrition services**

M: P7 said that malnutrition is not only weight loose. I see a child with body swelling and having malnutrition. [This is her person experience]. And p4 has said the infants are the affected group. what about the others? which group are most affected? the adolescents, pregnant, lactating or infants. if you have additional ideas on this point? Well, from different types of nutrition interventions that are put to tackle problems due to malnutrition in pregnant and lactating and improve their nutrition status such prenatal care, nutrition education and counseling about variety, frequency of food, use of iodine etc. So, what are the challenges to implement these services to the community? similarly what are the factors that hinder the community to receive these services? you can say repeat the question as long as it is not clear for you. for example provision of deworming, environmental hygiene, nutrition screening etc. i am saying what are the problem to provide this kind of services? please participate.

[P2 encouraged the participants to participate and express their idea]

M: ok p2 is requesting you to participate. i will repeat the question. there are different services provided to women and lactating women to improve their nutrition status such as screening, education etc . what are the challenges to deliver these service and by the way let me change the question dies these services available/provided to you?

P4: Our problem is in the community not in the government. they thought that weighing a child is not good. Of course this attitude did not exist is now due to efforts to reach home to home. two health workers gather the community near their village and identify by their weight and provide them fafa and if their condition is worth, they give plenpenet. it they weight is well, they continue to check every month since there is no guarantee for the next month.

**Section three: Perceived needs of women for relevant services during pregnancy, lactation and adolescents**

M: pregnant and non pregnant or lactating and non lactating women, can you compare these in terms of food requirement. which once need more? do you get my idea. does these group need the same kind of food?

P6: A pregnant needs variety of foods. this will help her during labour and for the fetus. a lactating women should change her cloth twice a day as the cloth she wear will be dirty due to urine of the infant. it will cause a disease.

M: what about in terms of feeding, can you tell about feeding?

P6: She need to get different food s for the market. she need to visit health facility and if her appetite is not good they will give her a tablet

M: P6 give us her view and the others?

P4: Regarding feeding, a pregnant women must increase her feeding frequency by one from non pregnant state or one extra food is required for pregnant. Likewise, for lactating women need more feeding since the infant food is breast milk and the source is the mother. she need more feeding. Until six months the infant food is only from the mothers, therefore more feeding is needed for lactating women every.

M: So, you mean lactating needs more food and one extra food should; be given

P4: Yes

M: what about others?

[a child fall around at this moment but he was fine ]

M: Let's continue

p1: pregnant needs more food

M: Why the pregnant needs more food?

P1: For the health of the mother and her child. she must be clean and the food should be good

M: Well if you have additional point here. which one needs more food, the pregnant or non pregnant, the lactating or the non lactating

P7: the pregnant should take different foods. the child is dependent on her until six months but afterwards he did not need much from her. before six month he needs form her. therefore she must feed more.

M: Now you are lactating, how did you feed yourself?

P6: we use hot drinks such as gruel, coffee and different types of food. the baby will be good. You must wash the bay and get adequate sleep. when the baby wakes up and if you want to hold the baby, you must wash your hands. otherwise the baby might get a disease.

M: P6 mentioned her idea. tell us your own experience. If the question is clear to you. P5 did you hear me.

P5: Yes

M: what about

P8: A lactating women should consume different types of food until six months. she need to drink milk. the baby will use from his mother

**Section four: other interventions that improve pregnant, lactating and adolescent nutrition**

M: Would you please tell more on that. what are the problems in feeding during lactation period considering your own case? P2 can you tell me? well what are the food that are allowed for the lactating women. explain me well on this point. i mean the foods prohibited for pregnant and lactating women in this area. P8 can tell me.... well i will come to you. P5 If you have idea?

P5: well you should select and eat. that is it.

M: Yes that what is supposed to be and this what might be taught but we want to know what you actually eat during lactation period. people tend to encourage eating some type food while discouraging you eat some others. i mean what are these foods which are allowed or not allowed by the community.

P2 would you tell me

P2: no thing

M: Sure, there isn't a food which is tabooed in this area

P2: There is no food taboo in our area.

M: what about others?

P6: in this area a lactating women is not allowed to eat roasted crop, Injera with stew until labor honeymoon period. In this period she will eat porridge

M: how long is this labor honeymoon period?

P6: If the bay is girl it is for 3 months and if male it will be one month. That is after their date of Baptisim. The reason for not eating the roasted crop is that she might experience dental problem'' Keykokuba''.

M: What do you mean by Keykokuba?

P6: her teeth will be loose and feel discomfort after lactation. So she will eat porridge

M: I am not asking what told to you from health professionals, rather what actual you did during lactation? Ok p2 continue

P2: During pregnancy nothing is prohibited. she can eat what she like to eat but as she(p6) said during lactation she must be limited to some foods.

M: You mean During pregnancy nothing is prohibited

P2: Nothing is prohibited during pregnancy. we eat what we like. during lactation we do not eat the roasted drop and the Injera with stew.

M: What else? P1 can you tell me

P1: i do not know any prohibited food

M: Ok. is Shinbira(chekpea) allowed?

[as a group they said we do not produce it]

M: What! you mean it did not grow here?

[yes]

M: Okay, you do not know it. what about Tiktiko?

P7: We eat Tiktiko of wheat and pea

M: So Tiktiko is allowed

P7: In this area spike of wheat and roasted crop are not allowed for pregnant women. They induce cramp to the women.

M: How much common are this kind of practice. you are lactating did you experience these advices/actions on you ?

P7: I gave birth during harvest time.

M: What did you say?

P7:The time is harvest time

[They all laugh together for seconds]

M: Oh! do you mean i gave birth at a time when spike is not available. did you do this intentionally?

P7: No it is my first birth

M: Ok, i understand it. i will continue with question. it there any difference in feeding between boys and girls. tell us if there is difference between them practically. who took more?

P6: The girl is responsible for making the food, so she is free to eat. it up to her but the boy are not supposed to make food. they simple eat what is given to them. this is a difference.

M: Do you mean the girl can eat whenever she want?

P6: yes because she at home. she can do what she like but the male eats a prepared food.

M: in terms of volume who eats more or for whom you give much comparing male female?

you shall be free and give us your idea. Is there difference between male and female ?

the number of feeding per day

P7: There is no difference. they will eat breakfast together and a lunch up on their return from the school. they will be given the same.

M: By the way what do you call those boys and girls in the age 10-19 years. i am saying young

P9: The young is age below 18 years.

M: So, do you thing better feeding is important for adolescent as we did for pregnant and lactating women? for example it there any education for adolescent about feeding?

P9: we did heard about that

M: P4 has mentioned some of the foods tabooed in this community. May i conclude these are the only foods considered taboo in this area? have you ever participated in nutrition screening program, or attend a session for detecting nutrition problems?

P1: we never face this kind of problem

M: I mean do you get this kind of services

P1: Yes we use these services. Our children gets weight measurement and if they are found low, they give Fafa to children and they give also ORS[zibtsbetse]

M: What is Zibtsebetse

P1: Maychew[to mean ORS]

M: Where did you get that

P1: At health facility

M: Others, those of who came lat. of course we discussed a lot. Anyway it is about feeding condition. it was mentioned that children will be screened and if the bay is found low in weight, they will give us Fafa. So, i mean have you participated in such activity?

P9: Yes we participated. My child was low weight last month[September 17). i give her foods and after one month(October 17) i took her to health center and her weight was corrected. she now 7 months. the problem happened when she become six months.

M: What did they do/ what did you do?

P9: I gave her milk, and porridge and she took these food well and she gains weight and Birhan[HEW] said me she is fine and i feel happy. But i was afraid at the beginning. if they are not fed well , "Nifase Gebeleo"( they will be exposed to illness).

M: What do you mean by Nifas Gebelo

P1: A child who is not feed well he will experience illness if additional food is not given after six months.

M: Well you told me about infants. can you tell me about lactating mother?

P9: if she is economically good , there s no reason to feed well. those who have farm might not have problem on this regard but those who do not have a farm, who can they feed well. this true only for those of us who have a farm. those of us who have farm we used to eat porridge, Kita and gruel. it i have i will eat five times a day because it will bring change in breast milk and the baby will fed and grow. But, if you do not have food how comes. if the women wants to work, it will be difficult because of the baby care . whatever if i have food at home , i will not forget to eat(i will not be lazy to eat)

M: What is the main problem? do you mean there food shortage is common in this area?

P9: Yes

M: Is there a safety net program in this area?

P9: Yes

M: What Kind of people are involved in the program?

P9: People who do not have animals. here the problem is that even though the work for safety net, they crop is given too late.

M: Can you elaborate a bit , when will the work start and when do you get the crop?

P9: The work is always but the food distribution will be late due to the release of budget form the source

M: By the way is it a support or safety net

P9: it is safety net

M: Do pregnant and lactating women get benefits from safety net program?

P9: Whether she is pregnant or not, if she has animals and farm she will not be given benefits from the safety net program. the same is true for lactating. only individuals who did not have animals will participate.

M: Do you mean they did not work or they did not get benefits.

P9: If she is pregnant or lactating and do not have animals, she will get all the benefits for the program until 10 months.

M: Others please tell us. was there a campaign about nutrition screening, have you ever told to screen your child from health professionals in Hashenge?

P1: we did not receive a message from them. we go to health facilities for check up

M: Do you thing that safety net has benefit for prevention of nutrition problem

P2: Regarding the safety net i do not have any animal but i am not involved in the program. i am not beneficiary.

**Section five: understanding age at first birth and birth spacing**

M: continue to share your experience. that is an important point Well, have ever heard that marriage before 18 years is not good for the women.

P9: Yes

M: from whom?

P9: This kind of message is communicated during meetings. but i am not sure if mothers are understanding or not. it did not reach down to mothers. But, marriage is allowed after 18 years.

M: So, you mean mothers are not getting the messages or is limited at top level. what can be done to reach mothers with messages regarding early marriage and birth spacing?

P9: mobilization and meeting at lowest level is important.

M: others, what do you mean by birth spacing for you?

[P9: encourages the participants to participate]

P4: A mother should give birth at least once every three years. if p[possible every five year but on average she must use contraception for 3 year. if you give birth in short interval, the second child will compete the first child and this will affect him

. **Section six: understanding communication and information sources**

M: Who give this kind of messages?

P9: The message is form health center. a mother should start family planning after four months of lactation. This lesson is taken from health center at least once a month.

M: Is there any discussion with mothers and adolescents? May in the form of meeting. P9 you told me that there meetings at top level. so, can told me similar events further if any.

P9: Yes there is a meeting . The participants are development groups, the young and the women. we have also received education at kebele level.

M: Improve the health of mothers by provide proper care for mothers children will make children healthy and ethical and the child who is grown well will produce a health child and this in turn will build a nation. therefore, to influence the health behavior of mothers or to improve their health practice such as proper feeding, personal and environmental hygiene and family planning what should be done? so far P9 has said a lot. Now please others give your idea. what shall be done to improve the health of mothers. the discussion is open. we coming towards the end. we are left with one more point of discussion. P6 you had good participation before but now your keep silent. what can be done to improve the nutrition of mothers and adolescents in general. let us forward our ideas. Few minutes ago you said health professionals are sources of information. Are the they only sources of such information? ok p9 tell us

P9: look if the mother feed well, she will give health baby. feeding is important

M: Does the women development army teach about feeding?

P4: They did not teach us.

M: Others, for whom you are referring when you are saying a health workers. do you mean a health extension workers of health professional

P9: The health professionals. When we go to health center. They teach women development army at health center. The others coordinate the provision of Fafa

M: Who is the coordinator of that?

P9: There are assigned women at the health center. They screen and refer them to with diagnosis of low weight or normal weight to these in kebele.

M: Who are these in the kebele?

p9: Those are female students who fail at tenth grade. Menemene Yibila Dimilala (Their role is that reading the name of beneficiary). Birhan (HEW)will identify the thins and send to them. They call us to collect Fafa" Fafa Niatiken Yibila". They give us and tell us to prepare porridge and feed our infants.

M: Are this people trained about feeding?

P4: [we do not know] but, They teach us how to feed, they say prepare the porridge in the ration of 3 parts of water with one part of Fafa and feed four times a day.

M: That s for children. what about for you?

P7: If pregnant she need to take porridge five times a day and for the infant four times.

M; What about for lactating:

p3: For lactating she will use food available at her home only.

M: What kind of crop is given for safety net?

P3: It is wheat.

M: How much is given to you?

P4: It is 10 Tasa (15 kg)every month for one individual. But, in practice they do not give us this amount. we will not receive this amour every month. we get it every at least five six months . At six month they will calculate 10 Tasa by the number of family members and give us. If the family size is five they will get 50 Tasa.

**section seven: additional remarks**

M: Anything that you want to say for me. i was simply guiding you to discuss but you can mention what every you want say about it.[keep silent] Well if you do not have any more idea. i thank you for being with us for more than 1 hour and 20 minutes. though some of you did not participate as we want but we believe that we have got important messages from you and thank for that in the name of Mekelle university. we have finished the Discussion. Thank you!

Summary

- To be healthy people should eat clean and wear clean. they should also use iodine in their food. Iodine can be applied at stew, bread and milk Environment should be clean, the house for the human and animal should be separated.
- The community thought that weighing a child is not good. Fafa and plenpenet are given to children according to their nutritional status which checked every month.
- A pregnant needs variety of foods. this will help her during labor and for the fetus. a lactating women should change her cloth twice a day as the cloth she wear will be dirty due to urine of the infant. it will cause a disease.
- A pregnant women must increase her feeding frequency by one from non pregnant state and lactating women need more feeding since the infant food is breast milk and the source is the mother.
- Food taboos for lactating women include eating roasted crop, Injera with stew until labor honeymoon period while porridge is allowed. During pregnancy nothing is prohibited.
- The girl is responsible for making the food, so she is free to eat but the boy are not supposed to make food.
- Women are not satisfied with food distribution following safety net program. there is delay and there is problem in selection
