# Supplementary material for: Barriers to utilize nutrition interventions among lactating women in rural communities of Tigray, northern Ethiopia: An exploratory study
Source: PLoS One. 2021 Apr 30;16(4):e0250696. doi: 10.1371/journal.pone.0250696 (PMC8087028; doi:10.1371/journal.pone.0250696)
Supplement: S2 File — (ZIP) [file pone.0250696.s002.zip › S2_File.Doc/Lacatating women_IDI & FGD/045_FGD_Lactating women_Keyih Emba kebele_Samre woreda.docx]

**Day 4: 30-02-2010 E.C.**

Translation 01 – FGD of Lactating women

**Section B: Interview details**

Zone: South Eastern

Woreda: Saharti samre

Kebele: NebarHadnet

Facilitator’s Name: Gmedhin B

Date of discussion: 30-02-2010E.C.

Discussion start time: 4:40 AM local time

End Time: 6:25PM local time

| **Section B: Socio demographic information/ All Pregnant Women** | | | | |
| --- | --- | --- | --- | --- |
| **Code** | **Age** | **Marital status** | **Education level** | **occupation** |
| P1 | 18 | Married | 8^th^ grade | Farmer |
| P2 | 25 | Married | 5^th^ | Farmer |
| P3 | 20 | Married | 8^th^ | Farmer |
| P4 | 20 | Married | 6^th^ | Farmer |
| P5 | 16 | Married | 8^th^ | Farmer |
| P6 | 20 | Married | 7^th^ | Farmer |
| P7 | 19 | Married | No education | Farmer |
| P8 | 30 | Married | 8^th^ | Farmer |
| P9 | 23 | Married | 2^nd^ | Farmer |
| P10 | 19 | Married | 6^th^ | Farmer |
| P11 | 25 | Married | 5^th^ | Farmer |

**Section I: Common maternal nutrition problems in the community**

I: What do the lactating women, including yourself, do to stay healthy?

P1: To keep our health, when we wake up, we first wash our hand and face, and at the same time, we wash our baby’s body. Then prepare food for our baby; it could be soup or bread (KITSA). If we gave him soup in the morning, we will prepare ‘Injera’ using ‘Shirofor’ for his lunch. At night I give me soup. As a mother, I have to drink enough soup and milk so that I will produce more breast milk for my baby.

P2: For me I drink soup and Maycheba(milk); if the baby do not drink soup and Maycheba, we prepare and give him a fried egg.

P7: complementary feeding is important for the baby. And for the health of the mother, we wash our body sing soap and we also wash our baby’s body too. We feed our baby on time and there pa procedure on how to do it. But the mother, although we do not have enough food in our community, we cannot list more like the people from urban. However, with what we have, that is Teff, we will prepare thick bread for the baby. For the mother, we usually drink soup at night. Besides we prepare Injera from the Mashla (maize) and eat it. This is what we do.

P3: That is what is mentioned by others. In our village, we do not have any varieties to use alternatively. We commonly produce Teff and Maize. From this, we also eat ‘Kolo’ as an extra meal beside Injera. When get milk, we drink. To the baby, we prepare him porridge.

I: What are the common nutrition problems in the community for women and adolescent girls?

P6: There are a lot of people who do not have food. E.g I know some on in my neighbor.

I: Let me explain to you, are there women who took fafa, plump net, because of malnutrition? You can tell more related to nutrition

P2: Yes, previously, I was told that I was wasted. I was taking fafa used it in the form of porridge, using one cup of water and two cup of fafa. I ate it without even sharing to my children. Then, after a month, you brought change, you would stop, if not, you will be given additional.

I: Explanation: other nutritional problems, it could be in your family or in your community, for example, it could be anemia, night blindness or goiter, else others.

P7: Even when you became lactating, your feeding depends on your household food security. I know a lactating woman in my neighbor, who has gynacologic problem. We simply and repeatedly say development army for group of 30 women, but no one oversees and no one has visited and understand her problem for a long period of time. Later, she went to Ayder hospital and got examined and treated there. Here, we talked about her in meeting if there are options to help her. I remember one day, it was mobilized in the community, to let inform, if there is anyone with swelling, be it gynecologic or goiter. Then we identified her, but because she was severely sick she finally got died even though she went to Ayder Hospital. Here in our community, there are a number of people who are sick, including lactating mother that are wasted. There are many lactating adolescent who discontinue their education because of their parents. There is no as big as such problems if the government is assessing to identify the community problems. Now days, there is a term called ‘friendship and love’, adolescent girls are getting pregnant before 18 years. This way, they are putting their family in trouble. It also affects their health. Furthermore, there is also problem in lactating mothers and their baby’s nutrition because, there is scarcity of food. We don’t have vegetables in this area as it is sunny and hot. There is no water source to irrigate.

I: What about related micronutrient deficiencies (such as anemia, night blindness, goiter)?

P3: In our kushet, there are women that are affected by goiter, and no consider it as disease. We assumed it as naturally gifted sign. They are still living with it and none of them tried to get treatment at health facility. It is taken as habit in our community.

I: what about diet related non- communicable diseases? Like diabetes, goiter and others?

P4: I did not see and any one with diabetes or goiter, but protect our health form these diseases, we have to keep our health, keep personal hygiene and environmental sanitation. Besides, if you are sick you have to go to health post and explain your problem.

P5: I have never seen such incidents.

I: Are women/adolescents in this community who do not increase their height proportional to their age? And weight to age? Could it have relation with nutrition?

P5: yes, this is because they are unhealthy. Their height become short naturally; it is their nature.

P4: A short person may have something he feels inside. How can I know the reason if I did not ask him? I do not usually ask this question. But if he gets diseased, you may ask him how it happens. For example, when some has affected by ‘MENDAETI’ (has hump on his back), he grows down. It swells on his back, and because it pushes him down, he becomes short. There are many such occurrences (being short) in our community. And this is because of disease.

P7: Having short height is very common. It could be due to 1) an internal disease and 2) it could be natural. There are many people who are short naturally. For example I myself, I do not know what they can say about me, I was very fine both my entire body and height before I came to here, to my mother-in-law. Because of disease, I had gone to Ayeder hospital and I had operated there. I am weak now. I cannot stand up easily from my seat, if I worked something. These all decreases height. Height is determined by health and disease.

P8: Yes, there are. I know one woman whose age 60 years but she is too short. This can because of poor development during her childhood or May be her mother had poor feeding during pregnancy, or maybe she did not take enough food after delivery. Because of these things women can get stunted.

P9: In our family, we have a stunted daughter. Even though she is 15 years, she is still short. There are many others whose height and weight is lower than their counter age groups and even her youngsters. The mother could have some problem inside her or we do not know because there was education that time. Either it could be due to hunger or disease or lack of screening or natural deformity; we did not know it yet. The girl is 15 years old and she is her height is short, and is still small in her weight too

P6: I have an 8 year hold daughter, and she stunted; her younger brother is taller and heavier than her and we call this a disease.

I: Is there a situation where mothers suffer from shortage of food?

P11: Because the land is not productive, for example in household, we do not have food harvest that can be consumed from year to year. Because we are working for food and despite we are not severely hungered as the government is supporting us, but there is a shortage as our farm is small.

I: Is there a situation where lactating mother do not eat food because of shortage?

P7: Yes, every person is not equal, one eats and the other does not. Not only are those who do not farm but also the farm owner are starving. The farm is simply consuming fertilizers, if not, it is not productive. There are a number of people who migrate and bring loan from other areas, and finally fallen in problems. Besides, those mothers who do not have husband used their land with share. The one that plough the woman’s land may not give her the harvest properly. Because of this, there are a number of women who are in trouble and facing shortage of food. There is a condition where a lactating woman does not eat lunch. Even when we produce enough food, we sell it to pay back government loans/debit: for fertilizer and others.

I: In what situation do you think this happened? When? Summer, autumn or else

P3: In autumn, we get from here and there as it is a harvesting time. In summer, together with cold, there is hunger. We weed without eating, we give the pies of bread have to our children. Hunger is commonly occurred in summer.

P8: Food shortage occurs in August and September; when we have some, we prioritize and give to husband and children, and we sleep without eating.

P9: Anyways, we do not have a woman who dies of hunger. We do not lost what to eat drug the sunny season, but, there is problem in summer. There is a proverb called “Eat when you do not have a child”. This is true, because we give attention to our children and husband.

**Section2: Barriers to access and utilization of nutrition services**

I: What kind of nutrition interventions are in place to improve health of lactating women?

P8; ‘Fafa’ is given to children who are wasted. This is done after measurement, and if they are underweight, they will be given fafa.

I: what about to the mother? The lactating one

P8: If her child is under six months and the mother is wasted, she will be given fafa for herself. After six months, the baby will be measured, and he will be given according.

P1: In our community, much is not done to improve nutrition of lactating mothers. But, we the mother goes to vaccinate her child, she will be measure her MUAC and if she is wasted, she takes fafa. And when the baby is stunted, he takes plum net every month until he improves

I: Anything additional? Okay. Do lactating women advised to visit heath facility for services?

P5: Yes. To prevent disease, there care providers come home to home and vaccinate us on arm. They also educate us to clean our compound ad dispose waste in pit. This is what Health extension workers advise us.

P6: HEWs teach me, and I prepare milk in morning, porridge in lunch time to my baby and egg at night; I do not have enough breast milk.

I: what about to visit health and advice on nutrition?

P4: HEWs and women development army frequently give us advices. We cleanly prepare a meal from what we have. We also keep the sanitation of our environment. They help us with the capacity they have.

I: Do you or other lactating mothers get advice to use extra meal? How?

P3: Yes. A pregnant woman should eat one extra meal and after delivery she needs to eat two extra meals. And when her baby is older than six months, the mother should give him complementary foods.

I: who is giving such advices?

P3: Health works. They inform us every time we go for vaccination.

P11: yes, the teach us to add one more if we are pregnant. The weakness could be with mothers, but HEWs telling us every day.

I: Do lactating women screening for their nutritional status? Be it in the form of campaign, or scheduled?

P11: This year, we are not screened, although we were pregnant and are now lactating. Previously, they screened us every three months. But currently, no one is doing this. But, if we go to health facility for other purpose, we measure us. But in the time, there is no campaign and community health day for screening.

I: other, additional? May during pregnant mother conference or else

P8: My home is in Zemed, it is far from here. Before two years, there was nutritional screening; they measure us on our arm. But now, be it because we are not heard of it or they are not coming to here, there is no screening at all. Now days, because our home is far, HEWs came and informed us to come to here.

I: what about the use for food diversification? Do on this you get advice regard?

P3: Yes we get. For example, here in our area, we have teff, maize and sorghum. These are three varieties. To use balanced diet, “do not eat these at different session rather it will be good if you serve it at the same time”. This is continuously advised by HEWs. For a child it will be a balanced and complementary food too.

I: any other? Okay. What do you think is its importance?

P2: Rather than simply eating only injera, she has to add others like,’Tiktiko’, ‘kolo’, bread and ‘Hanza’. This is done to increase production of breast milk. If she eats only injera, she cannot produce milk. Health care providers provide us such information, and after we comprehended it, we practice it.

I: Any more to be mentioned as benefit of food diversification?

P9: There are body building foods (proteins) and energy rich ones. To your health all are important. So, you need to eat different types of foods.

I: Do lactating women get advice to use iodized salt? Why?

P10: yes, we use it to prevent goiter. It has been two years since we left use of the ordinary salt. We are using the iodized salt, because it has chemical as we have got advice on this.

I: do women know how to use it? Have they been demonstrated it to you?

P10: We add iodized salt after we put down the Tsebhi(Wot)and we have to cover it. This is done to prevent evaporation of the chemical found inside the salt.

I: Others? Hoe about it’s (the salt) access?

P3: yes, the salt is easy accessible and we are getting it easily where at any time. Even its cost it lower that the ordinary (non-iodized or Gamfur) salt. The Gamfur one is costly and it has no advantage and we are not using it.

I: any other? Okay.

I: Do lactating women get advice on nutrition sensitive agriculture such as home gardening?

P8: yes we get, but we are not practicing it. This is because there is no water in setting, we are thirsty of it. HEWs advise us to plant pepper, salad, tomato and other vegetables as these are source of energy and good for child.

P7: That is true what they have said it. HEWs inform us plant vegetables in our home yard. The food what we prepare to adult and child should be different. Vegetables are complementary foods to children. But here in our kushet, there is no water since it is Kola. Even though we did not give attention, they give us the education.

P9: it has been five years since development army is established. We had undermined the use of pepper. But after advice, I plant pepper and I cover my consumption, especially during fasting or dry season. I water it continuously and it has been two years since I left buying from market.

I: how about its access? For example if you a seed, do you get what you want? May be the HEWs may tell how to prepare food from it; how about how to plant it, from the agriculture side?

P1: yes, we get. For example pepper, I prepare seed from what I have bought for suck of consumption. I bring pepper form Samre and I use from that. We are being advised to plan vegtables, but we did not home garden more as we want, because, the condition is sunny and the water source is too far.

I: what about related to safety net? Are lactating women involved?

P11: Yes, when they are pregnant, they are given rest but for the lactating one, they have rest for ten months; after that they work for food; and yes, they are involved in safety net. Regarding the vegetables you mentioned above, no one gave us seed and even the agriculture extension do not give us; we just prepare our self. E.g. pepper. But, we do not practice for tomato, potato and onion although we had been advised.

P10: That is what they have mentioned. If she is pregnant and is confirmed in health facility that she is in her six month of pregnancy, she will get rest. The lactating one will get food support freely until ten months. When her baby is ten months old she will work for food.

I: any more on safety net?

P2: yes. If she lactating, she is getting food support freely until ten months, and if you’re wasted after MUAC measurement, she will get fafa. And for the baby, plum net will be prescribed for him.

I: what about advice on water, sanitation and hygiene services?

P6: we do not dispose waste everywhere, we put it in pit; we wash our hand before eating.

I: any more on latrine construction and utilization, and hand washing at critical times or more?

P3: After we wake up from sleep, , including our children, wash our hands and face before eating breakfast. We wash before and after eating meal; after touching any waste. We are also advised not dispose coffee’s ‘Daka’ everywhere; and to use clean jar while pouring water. Besides, they tell us to keep our personal and environment clean to stay healthy

I: who do provide this and where?

P4: This information, health post and in the meeting with development army leader. They taught us on environmental hygiene and waste disposal system. Water handling and food preparation are also included in the lessons.

I: Is malaria common in his community?

P9: Yes, malaria is commonly incident here, because it is ‘kola’. For this they gave us ITN. During autumn, we sleep under the ITN. And regarding the sanitation you mentioned before, I want to add some; Health care providers educate us well. We separate a compound to our animals and to our hens. If we do not get soap, we wash our hand using ash. We also wash our children. We also put jar inside the pot which tied using rope to fetch water. In these five to six years, there is such follow up in our community. We have also separate toilet.

I: Who inform how to use ITN? How frequent is it given to you? How much? Is it enough?

P1: Malaria is common in summer and autumn. In summer, mosquito will bread if there is swamp area around if it holds water. Along with the grass, it comes at night. In summer, people come and spray insecticide to our home.

P11: The swamp is, as it explained earlier, should be drained. The grass should be cut. The ITN may or may not present. If it is only one, priority should be given to women. Our home is sprayed insecticide every year.

I: How much ITN do you have? How frequent is it changed?

P11: we have three. It has been three years since it is changed.

P8: It is true that mosquito comes during summer. HCWs inform us to properly use ITN. Besides, they tell us that malaria transmitted through mosquito bite. Cleaning our compound and using ITN is important.

I: how common are lactating mothers affected by malaria?

P8: Yes there are many women who are severely, moderate and mildly affected ones.

I: What about regarding targeted supplementary feeding like provision fafa and Plump net?

P2: The mother use fafa until six months. After six months, she will not be given whether she bring change or not. If the baby is still under measurement (severe malnutrition), he will be given plummet or milk. If he has little improvement, he will be given fafa.

I: what about to the mother if she has still severe malnutrition after 6 months?

P2: She will be treated by her own expense. No support is given to her?

I: Are lactating women getting deworming service?

P3: Deworming give to only children, not for mothers. Every three month under five children are taking drugs for parasites, but for the mother, she will be Measured her MUAC, and if she is low, she will take fafa.

I: which of the above intervention do you think is most important?

P11: safety net is the most important for me as we are supported for ten moths with working.

P10: use of ITN and draining watery/swampy areas is good. The malaria is transmitted by anopheles mosquito, and it may lead cerebral malaria. We are preventing this disease. So this intervention is also good.

I: what are the barriers in the implementation of these nutritional services?

P7: Any ways, the government is providing every information, but everything may not be fulfilled. A chemical is entered to the hand pump water, and we have also ‘wuhaagar’ at home to treat our water. Everything is provided, may be in our side that the people can say the water’s taste is changed, and others. In our area, there is no any water borne diseases. For me everything is fine.

I: what else other barrier is here? (Food diversification, extra meal, checkup, services, advice on WASH, ITN etc.)

P10: everything is fine. They tell how to feed our child and a pregnant women should eat one extra meal and eat four times, and lactating women has add two extra meal. The food should have varieties: form vegetables, teff, maize and wheat.

We also prepare porridge for our child. Thus, I see no problem at all.

P3: A mother is not getting supplementary feeding if her baby is greater than six months. Thus, procedure should be corrected, because the mother should take ‘fafa’ if her MUCA is low irrespective of the child’s age.

The mother should get deworming service together with her child. But this is not inplace.

**Section 3: Perceived needs of women for relevant services during lactation**

I: What special things do lactating women need in your community?

(Visiting health facilities? Taking extra meals? Taking rest? Taking supplements? )

P9: A pregnant woman is working in the water and soil conservation activity until 6 months of pregnancy, so we want this to be corrected. As per our need, she must get rest after three months of pregnancy.

P1: Pregnat woman are getting in troubled while working during pregnancy. Before six months the fetus is bloody and unsettled, so the mother should not work any heavy activity. And after six month, he has developed his organs and the mother needs extra rest. So, we want this to be corrected.

I: what about for lactating mothers.

P1:for safety net, the mother beneficial without working. The problem is during the water and soil conservation. If she celebrated “Kirstna” on Sunday, she will get out on Monday for work in water and soil conservation. With this small infant on back, the work is tough for mothers and for the health of the baby. It would be good if we are on birth leave until six months, so then the baby will be on complementary feeding.

I: what else? E.g Taking supplements like vitamin A.

It has been long time (five years) since it got discontinued. Currently, it is only giving to under five children. Regarding the rest for pregnant women, special focus should be given. I know a woman who has been working until six months pregnancy, and final the fetus got aborted. This happened because we carry stone and to gether with the sun, that woman got miscarriage. Because pregnant women are shipping a stone, we are exposed to disease.

P9: for example I myself was working while I am pregnant to this baby. I had frequently fallen down after I get back from work. This is because I am too tired by the water and soil conservation activity during the entire day. This truly hurts our health and must be corrected.

P5: There are wasted mothers and children. However, the provision of fafa is interrupted. As a result, there is no any improvement in the mother’s nutritional status. Thus, it must be supplied.

I: what should be the role of husband to improve maternal (LW) nutrition?

P10: The husband should slay and prepare meat for his wife starting from her pregnancy. Because males are involved in some discussions on women’s health, they have better understanding than before. He also must save money for transportation purpose and other expenses. And after delivery, he must buy other food staff needed for his wife.

P9: that is what has been mentioned.

P2: There is no such a care. What is not left is he buys honey and butter for her during her delivery. He fortunately just slays and prepares meat only if a goat dies.

Because of our poor economy, husbands are not supporting more.

I: Does he buy goat from market or is it from what you have?

P2: It is from what we have and only if a goat dies. But, he must buy a honey and butter from the market.

I: Do women in this community typically change their diets when they are lactating? It could be in type, in amount and number of meals.

P2: Yes, what a different diet we add is like bread and porridge. But the husband eats only injera.

I: what about in terms of amount? More or less food?

P3: It is known that lactating mother needs extra meal. But, it depends upon the condition. If it drought, yes, she will small food. And if there is more production/harvest, she will eat more.

P4: After delivery, our feeding is base on our resource. We use soup. But you have, even you can eat variety of foods. But, we commonly face scarcity.

P11: You need to be healthily to bring change. If you are healthy and eat more, you would be fat and good looking. But, if you are not healthily, even you may get wasted, though you are eating more. Any ways, the one who eat clean and more food is better.

P6: lactating mother eats porridge, ‘Titiko’ and bread. At night, she must drink soup.

I: What affects lactating women’s diet?

P8: Now days, there is no problem. You can eat you want if you have.

I: Are there gender disparities in women’s diets during lactation?

P11: The food is the same, but the mother should eat one extra more than the male.

I: what about in terms of size for girl and boy?

P11: for the children, there is no difference for male and female. We give priority to the younger baby.

**Section4: Other interventions that improve pregnant, lactating and adolescent nutrition**

I: Have you ever gone for nutrition screening during community health days? Where?

P6: it is almost a year since it is interrupted.

I: Had the community health day benefited lactating mothers at that time?

P6: we went there, and after measurement, they gave us four packs of ‘fafa’; we prepared porridge using oil and were feeding our children. It was only one time and we were not after that event.

P7: Even though there is screening now, there variety of screening tests done in our community, E.g. HIV test and MUAC measurement. None of it is present today. You can go to health facility either if you are sick or your baby is sick and at that time care providers will examine you and may measure MUAC if it is necessary.

I: what about during the routine service delivery? Is there nutritional screening?

P5: In the previous years, there was screening sessions and inform their status, and mothers and children were given fafa and plum net. But now there is no screening program. They only measure to children.

I: How important is nutritional screening to mother?

P9: These days, even when I am educated, or my sister knows the condition. We are measured our MUAC, but they did not tell us our nutritional status. Even we ask to our self why they did not say anything about me. I think this has to be known. So far, if I am wasted, the give I paper. Now, if they mobilize us to the kebelle, they do not measure us again and do not tell our improvements. As we are all lactating, we want to know our nutritional status. It would be good if we are told where are in terms of MUAC.

P3: If I am sent and travel for three hours to kebele because I am under nutrition, and if they sent me back without any support, it quite unfair. That was the situation existed so far. Had we been screened in our locality, in the health post, it would not be tiresome to us whether we receive fafa or not. I will not be offended if they did not give if I have improvement. I will also be happy if they give fafa if I am wasted. But, previously there was a problem, as we were coming back barely without anything, after travelling for three hours, if I or my baby has improvement in MUAC.

**Section5: Understanding perceptions of age at first birth and birth spacing?**

I: You may have heard that delaying the age at first birth to after 18 is better for the health of both the mother and the baby. How do you see this?

P5: it is not good to marry before the age of 18 years. Because, there could be problem while giving birth. But she will be safe if she marries after 18 year. It is because of the backward thinking that people are practicing it.

P6: it is good if a girl marries at age of 18 or 20. If it is bellow she will be physically low too.

P11: if she marries under 18 years, she might face fistula or difficulty during delivery. This is because her womb and hip are not wide.

I: how about its relation with her nutritional status?

P11: Because she is young, the baby will be very small (underweight).

I: what else? Okay.

I: Do you think this message is being promoted in the community?

P3: Yes, it is being told through development army, at school. If a girl preparing to marry, everyone knows about it, they have a fear that they could have been caught or punished accordingly. So there is no underage marriage.

I: how acceptable is this message by the community? Or is there anything to be improved on underage marriage?

P1: in our community, age to first marriage is fine. This is supported by law. It is first checked by the community leaders and after confirmation, she will marry.

P4: This day, this information is widely known. Previously, marriage was determined by the pressure of parents. But now, in school girls have enough information and development armies are creating awareness in the community.

Because of his or her children everyone here are aware.

I: How many years do you think the gap should be between successive births for a woman?

P7: it is good if birth interval is three to four years so that preceding child will be healthy and well developed. If not, it has pressure on the mother, and the baby will not get enough care.

P8: Three to five year interval is good. Because the baby well breast feed until three years, he will be physical and mentally developed. This is how they educate us.

P5: Greater than three and four year’s birth interval is okay.

I: what if in shorter interval, e.g. one year birth interval, what would be the problem?

P5: The child will be sick and he may have frequently abdominal cramp, because they feed him colostrum.

I: From whom did you get this information? Where do get such information?

P2: it could in health center or me if I have education. In time of our former mothers, there was no contraceptive. Now, because the mother is aware, there is an injection to increase birth space. If a mother wants family planning service, and determine to care her children, there is follow up the government, and an injectable contraceptive.

But, if she refuses to take, she will get pregnant and give birth after year. One will on her back and on will be under her arm. This time, the baby will be hungered and she will also be malnutrited.

I: What can be done to prevent short birth intervals, and prompted the message more?

P8: Alost all women knew it, but there are some who don’t use saying “injectable is not suitable for me” and they give birth every year. But, if it suitable, there is injectable that can even prevent for three years. But all have the knowledge because health care providers had taught us.

I: else, or any other information?

P11: Previously we were giving birth every 2 years, but now because the education expanded to the community, and there is a pill, there are women who deliver before four years. In such case, the child will have normal growth. HEWs are informing us in every session we met. Thus, there is improvement.

**Section 6: Understanding communication and information sources**

I: Is there any opportunity in the community to discuss nutrition for women? What are these? What should be done in general to improve maternal nutrition?

I: even among the intervention we have mention above, or else? Even need of resources?

P8: On the safety net, it is 10 months that a woman is allowed to get the benefit without work. The same way, it must be applied in the water and soil conservation activities. Meaning, a lactating women should be waived the water and soil conservation activity until her baby celebrates ten months. I, myself, was working in water and soil conservation while my child is 45 days old, and I was suffering. These we want a focus here.

I: what about related to community conversation? Is there community conversation that talks about maternal nutrition?

I: Or on home to home visit? Food preparation, etc

I: or on information dissemination to the community on maternal nutrition?could be in meeting.

P9: Regarding all the issues we have discussed, on pregnant women and children and lactating women, like the safety net program, water and soil conservation program should consider lactating women, and this should be promoted in meeting in our kebele. This has to improve for females.

P3: for lactating mother, it is advised that they should eat extra meal and give support for it. This should be promoted well.

I: any additional suggestion or comment for mothers?

I: thank you very much for your information and time.

Good day!

**Summary**

**Section I: Common maternal nutrition problems in the community**

In our village, we do not have any varieties to use alternatively. We commonly produce Teff and Maize.

Even for lactating, your feeding depends on your household food security.

We simply and repeatedly say development army for group of 30 women, but no one oversees and no one has visited and understand women’s problem for a long period of time.

In our kushet, there are women that are affected by goiter, and no consider it as disease. We assumed it as naturally gifted sign.

Stunting is common and it could be due to a disease or natural.

Food shortage occurs in August and September; when we have some, we prioritize and give to husband and children, and we sleep without eating.

**Section2: Barriers to access and utilization of nutrition services**

The weakness in adding extra meal could be with mothers, but HEWs telling us every day.

Here there is no campaign and community health day for nutritional screening.

The cost iodized is lower that the ordinary (non-iodized or Gamfur) salt.

Women do not practice home gardening, because there is no water in setting,

Regarding the vegetables, no one gave seed to women and even the agriculture extensions do not give them.

If she is on severe malnutrition after 6 months,she will be treated by her own expense. No support is given to her.

A mother is not getting supplementary feeding if her baby is greater than six months. Mothers should get deworming service together with her child.

**Section 3: Perceived needs of women for relevant services during lactation**

A pregnant woman is working in the water and soil conservation activity until 6 months of pregnancy, so we want this to be corrected. As per our need, she must get rest after three months of pregnancy.

It has been long time (five years) since vitamin A supplementation is discontinued.

It is known that lactating mother needs extra meal. But, it depends upon the condition. If it drought, yes, she will small food. And if there is more production/harvest, she will eat more.

**Section4: Other interventions that improve pregnant, lactating and adolescent nutrition**

It is almost a year since nutrition screening is interrupted.

These days, even let alone I am educated, my sister knows its importance. HEWs measured our MUAC, but they did not tell us our nutritional status.

**Section5: Understanding perceptions of age at first birth and birth spacing?**

If she marries under 18 years, she might face fistula or face difficulty during delivery. This is because her womb and hip are not wide.

Three to five year birth interval is good. Because the baby well breast feed until three years, he will be physical and mentally developed.

All women have the knowledge on contraceptives because health care providers had taught them.

**Section 6: Understanding communication and information sources**

For lactating mother, it is advised that they should eat extra meal and give support for it. This should be promoted well.
