# Supplementary material for: Barriers to utilize nutrition interventions among lactating women in rural communities of Tigray, northern Ethiopia: An exploratory study
Source: PLoS One. 2021 Apr 30;16(4):e0250696. doi: 10.1371/journal.pone.0250696 (PMC8087028; doi:10.1371/journal.pone.0250696)
Supplement: S2 File — (ZIP) [file pone.0250696.s002.zip › S2_File.Doc/Lacatating women_IDI & FGD/057_FGD_Lactating women-Lemlem kebele_Samre Kebele.docx]

**Operational Research on Adolescent and Maternal Nutrition in Northern Ethiopia**

## FGD with lactating women

**Introduction**

## Hello, my name is Amaha Kahsay. I am from Mekelle University. Thank you for taking the time to speak with me today. We are doing research on the factors that influence the nutrition of mothers and adolescents in collaboration with the Regional Health Bureau and UNICEF.

## So, do you agree to continue our discussion? 1. Yes 2. No

**Section A: Interview details**

1. Zone: South-East Tigray
2. Woreda: Seharti Samre
3. Kebele: Lemlem
4. Facilitator’s name: Amaha Kahsay
5. Date of discussion:11/11/2017
6. Discussion start time: 10:29AM
7. Discussion end time: 12:56AM

| **Section B: Socio-demographic Information** | | | | |
| --- | --- | --- | --- | --- |
| Name of the FGD participant | Age | Marital status | Education level | Occupation |
| 01. Mesert Belay | 22 | Married | 12^th^ | Hose-wife |
| 02. Kidan Tensay | 42 | Married | 0 | Hose |
| 03. Nigsti Gezu | 35 | Married | Attended adult education | Hose |
| 4. Freweyni Teabiyo | 21 | Married | 10^th^ | Hose |
| 05. Ayinalem Woldie | 37 | Married | 0 | Hose |
| 06. Mulu Woldeharat | 35 | Married | 0 | Hose |
| 07. Gideta Ayene | 35 | Married | 3^rd^ | Hose |
| 08. Ayiney Birhanu | 30 | Married | 0 | Hose |
| 09. Desta W/tekle | 34 | Married | 0 | Hose |
| 10.Tiwures Libelo | 30 | Married | 0 | Hose |
| 11. Komihen Birhanu | 39 | Married | 0 | Hose |

**Details of the FGD**

**Section 1: Common maternal, lactating women nutrition problems in the community**

**I: What do lactating women do to stay healthy in this community at your Lemlem Kebelle?**

**I: It has praise for the one who starts the first talk!**

**03**: Let me take the praise! With laughing! So, for our children and ourselves to be healthy, firstly, we deliver at health facility. Going there, with good health follow up, if there is blood increase, it is let reduced. If any happens to your health, you are checked; if your baby is with less capacity, it is checked and helped there. Then, you come home with very improve health; then, the baby is served with different foods at home. Firstly, he is vaccinated three times; at his ninth month, given fourth vaccine; then before his six months, he doesn’t drink water; after his six months complete, we prepare different foods from available foods at our homes; any different foods we prepare and we feed him; and we care him not to be sick.

**I: Thank you so much! Have you heard her; she told us all about what is being done for both mother and her child in very detail! Like that of the delivery at health facility, the care given to the baby there and at home too before and after his six months; so, what else do lactating mothers do to be healthy here in this kebelle?**

**02**: If our life is to be kept, first, the health packages (she means to HEWs) follow us from our pregnancy; Then they let us go to health center; thus, keeping our promise, we go to the health center and we are let have four times follow-ups. Then we give birth there in the health facility; they care us well; if our blood decreases and if we bleed, they give us helping food to our blood (she means IV-infusions) they give us all these foods with no any five cent of payment; the government take us by ambulance and our baby is treated there too; he is also given three injections there. They are also injected at their ninth month; again up to their fifth year of age, they are given injection in every year. We also give them our care from mixing different foods like being it lentil, flax, legume and others; we mix and prepare porridge and when they are six months and above we feed them this porridge based on our capacity. And may be during delivery, all could not be healthy; if they are those who gave birth, they may develop trembling, and fever; thus, they go to health facility. And about the danger signs of pregnancy, maybe I brought it late, if the pregnant woman has pregnancy danger signs like that of swelling of her legs, blurring of her eyes, and bleeding; this is danger pregnancy! So, she goes to medical care. Whatever so, we may not capture it all; but, there is no flaw to the care we get; unless, we are not educated; we are fine due to our government.

**I: Thank you again; for the clear and detailed discussion she provided us; maybe here talking and appreciating the government may not be our ultimate goal; what is good deed is known; but what we want now is that what is really happening in the ground and what has to be improved about to improve maternal health; so, what else is being done by you lactating mothers here to stay healthy; anything left?**

**07**: That is it; nothing is left. Except our student children are not getting jobs. Otherwise, there is nothing left. At medical care, for all I know, it is exceedingly present; when you feel sick, the ambulance comes and takes you to the medication. But in the education case, our students are not getting jobs as soon as possible.

**I: Which students are they those who are not getting jobs?**

**07**: Any of the students who learn at Samre; from Samre to Mekelle. We are giving them our money to learn; but no job they are obtaining; those who completed to grade-12^th^ and colleges too; for example, many females went to Mekelle for college education; and even this one is ready to go to learn (the grade 12^th^ participant); but there is no job for them. But as to the medical care, it is even exceedingly being undergone.

**I: Oaky, let’s talk in detail here; why do not they get jobs?**

**07**: It has to be to the government to be evaluated!

**I: So, have you ever asked to the government why they are not getting jobs?**

**07**: Indeed, we didn’t ask about it.

**I: It’s okay; let’s talk about how they pass their days if they are jobless at your kebelle?**

**07**: Indeed, they do anything they get for themselves; they will not be in problem for their day and night needs; they can search any kind of work. But the problem is that they are not getting salary and payment by the government for what they have learned. Yet, they are not at difficulty of getting for their daily needs; but they are not invited by the government to be recruited and be paid a salary for their jobs.

**I: Are there many who are jobless like females who completed their education but no job at your kebelle?**

**07**: Yes there are many; like those females who completed their grade-12^th^, are many but no job.

**02**: But now whatever so, the good government is persuading and letting all learn and learn; and we are giving birth and we are growing them. And now it is must for them to learn; there is no means that they have to go into dusk (she means being older) working at this farm. But now at the education, the point is increased from time to time; thus, many are failing; they fail from 10^th^, and 12^th^; there are also individuals with knowledge but not learned due to lack of capacity; then become farmers; yet, there are also individuals who have capacity and then learn with their own expenses. But there is a problem here; maybe there are many students, the government may not get enough places to put them into their jobs; maybe there are many students, the government may lack adequate place thus those with higher profession may occupy the place. I mean, what else can be said then? but, the guys are present at the town moving here and there as she already said it; they may get any work that can be hand to mouth but not worth beyond that; so, as she said it there are many who have not jobs at our kebelle. So, if government has to see it widely this is it; unless, we left it thinking that it is beyond our capacity.

**I: Okay; those students who already completed their schooling but have not jobs; may this have impact to their health; especially females? So that we it can be witness for our government that our students are being affected due to lack of jobs if any?**

**07**: Yes, there is; the hurt is that for example, there those who completed either grade-10^th^ or 12^th^ and also those graduated ones. But those graduated one due to absence of job recruitments, they are entering into other directions like that of anger, being caught by diseases, and even hanging one’s self, being drunk though they don’t come here and being checked their health status..

**I: Are there guys who are hung themselves at your kebelle?**

**07:** There are two students at our kebelle.

**02:** No, it is not because of lack of job that they hung themselves. (Argumentative)

**07:** Yes, it is because; the government didn’t follow them well! (Argumentative)

**02**: Let me talk here, firstly these students didn’t get adequate points at grade-12^th^ to enter to university; due to this, that their point is low not due to they didn’t get salary that they are hanging themselves, they take malatine too.

**07**: You see my sister! It is because the government is raising the pass mark to university that they are committing suicide!

I: How many students did commit suicide here in this area?

**02**: One from Kebelle Gerhi and another one from kebelle Samre at 2010E.C died due to that unable to join university.

**03**: And the third one is at university that was killed himself; in 2009E.C due to the reason that he requested money from his parents to send him for his graduation; but they didn’t send to him; thus, he killed himself there.

**02**: Yes, the problem was up to his mother and father that they didn’t send the money; yet, it can’t be done anything if that Satan comes to him; he is deceived by Satan! He was from kebelle Hadinet. It was in 2009EC; I think from Mekelle.

**06**: It was up to his thinking deficit that he hung by himself!

**07**: But from those who completed their education, but not get job, there is nobody who hung himself; rather, they let themselves be annoyed, anger which then let them devlop gatric problem. And also many females are flocking to Mekelle to learn at colleges paying their own money because they didn’t get enough points either at grade-10^th^ or 12^th^. This is because, the government is fluctuating the passing mark from time to time for them to join the university. for example, this year all the females of our kebelle at grade 12^th^ are failed; now they are going to Mekelle, and the tuition money for college is increased from 200 to 300-400ETB per a month. So, may God help them!

**I: So, will they get job after they will be graduated from those colleges?**

**07**: So, that is the problem; they don’t get job sooner after graduation!

**02**: Those who are clever are getting jobs; we have not totally blame the government! Those who are graduated from universities by the government are getting jobs.

**07**: Yes, some are getting jobs.

**I: Is there anyone who is graduated from university but not has job at your place?**

**02**: There is no anyone; they all get the job; yet, the problem is not many students are joining the university due to the higher entrance point; the problem is sustaining the entrance of the students to the university. Otherwise, if the able to enter and sustain at the university, they get the job after completing their education. The problem is that those who are learning paying their money are not getting jobs; they are moving here and there!

**I: So, what do you think shall be done to reduce the jobless students especially those females that are not entering to university but joining to private colleges paying their own money; what would you do if you were given the authority like if you were the government and ministers to help them?**

**07**: Job! All the participants and me laughed much happily! Then after stopping the laugh, we would have created job sooner to them had you been selected us to do so.

**I: What kind of jobs would you to create for them then?**

**03**: There reason why our students are moving here and there without anything is that if we are to do what we are talking, the government is creating an opportunity to all the students to learn without any discrimination that all the children of the poor and the rich, blacks, the reds, the tall and the shorts without any difference are learning and being mobilized currently. But, equally to this mass education mobilization, there has to be equal access of jobs created so that everybody could be involved.

**I: For example, what kind of jobs would you mention?**

**03**: For example, those already educated have to get jobs so that they can teach to those who are not educated.

**I: For example, would it be okay if they are involved in agriculture production; otherwise, may it be okay if e put them all as teachers as there is no place for all to be as teachers?**

**03**: That agriculture is enough! We are the ones who are cursed that we are living in this agricultural work! But for those who are educated the government should increase job opportunities so that they have to be involved there.

**02**: I don’t agree here! The difference is that the government is providing adequate teachers and many students are learning by helping and providing assistances to the teachers and the students too; there is no problem here. But here, learning is getting knowledge; so, he has knowledge, he will be merchant, he will be able to be farmer; even to be a farmer needs knowledge; it is the farmers that are getting good incomes at this time; it is because they are taught by the benign government. But whatever so, do all the educated ones get job by the government that may not be true; that is up to your chance! Yet if you are educated, you will never fail; even from the farmers, it is those educated farmers that win to those blind (she means non-educated) farmers in the productions; because, they know it what and how to do things. So, any one educated can work at office work s, some at trading and some still as farmers; all can’t work in office; so we can’t blame the government. Whatever so, the ones who are educated never die; unless, it is us that we are not educated.

**I: So, why don’t those so-called jobless students plow and harvest here with their parents?**

**05**: Some of them are plowing and harvesting.

**06**: Unless they started any work at towns, they come and work at agriculture with their family. Yet, some are still moving here and there with on jobs.

**I: So, are they successful at their farm works?**

**07**: Do you think the one who plows is not successful? Farmers never fail by any means; every one even the educated ones are relying on the farmers.

**I: In your opinion, what are the common nutrition problems in the community for pregnant women?**

**03**: There are thin women here that will answer this question. (She laughed)!

**I: Yeah, what is really happening to our mothers related to their nutritional conditions, are there any problems, and diseases, due to they don’t get adequate food to eat? Let’s talk then?**

**05**: what if there is who eats well but not changed! (She laughed). May be due to thinking deficit, you eat your breakfast early at the morning; next if you eat your lunch at 2:00PM, your body will not accept the food; it will not work properly; it has its own time to eat. Then eat your breakfast at 8:00AM then you eat again at 10 to 11:00AM; then it will assimilate to your body; otherwise, it will not work even if you eat. And in the case of being wasted and thinness, there is natural one and also it happens due to food shortage; the natural one is permanent that you can’t change even if you eat all the day; from where can you bring? But that wasting and thinness due to food is known. But for all things, eating properly is very important; you have to eat at appropriate time without passing your hours of eating.

**I: Okay, you told me that there are natural and food based thinness; so, are their mothers who are thin and wasted due to food shortages here in the kebelle like what you have told me about** the problems to the students earlier?

**02: We are okay due to our benign government (she called it red government)!**

**I: It is okay; but our intention is not to talk and evaluate the government; whatever the government does, whatever is here done to you being it by your husbands, yourselves, and others, what really is happening t you our mothers is the concern; so, let’s talk about it?**

**02**: Now, when we say government, it is government. But ourselves we are not able to feed our children timely due to our thinking deficit; as a result, there are many children who are screened for Faffa; and the government was used to give them well all the Faffa, Mitmita (she means to Plumpy-nut) and milk too; the same was also given to mothers. But now all that is abandoned. There are also mothers with nutritional problems that were given that Faffa. But this happened due to our thinking problems that we are not accepting the orders come from the government about how that there are many mothers and children exposed to Faffa ration. And this is because we don’t care our children and our selves; we don’t care!

**I: What happened to the mothers in your kebele; are there lactating women with nutritionals problems?**

**05**: Yes, there are and it is because they don’t give adequate care to themselves. This is probably there is food shortage at home so that they give priority to their children or they may have thinking problem about their food taking; this is it whatever so.

**I: So, what is happening to the mothers due to the food shortage or the thinking problem hey have if you understand me well?**

**06**: There may be a problem at home that the mother thinks about feeding her children leaving herself without a food when there is food shortage; but due to the government that provides us aids, we are eating fine. But now, as it is said there is natural thinness which happens to the mother natural and which can let her be critically sick and even let her die. Yet, if they are identified to be thin, they go and receive Faffa and they use that properly. So, when do our own business we talk about the ones who receive Faffa that it because they are thin that they receiving it; otherwise, we don’t know it really what it’s about, unless educated ones like you come and identify it. Yet, we simply talk and hear that there are mothers who receive Faffa; and for their children if the Faffa is not okay for them, they are given Mitmitta (she means plumpy-nut) and they are referred and given milk for their children at Samre; then we say is that because of hunger or are they very thin; we talk this when we are together at our home.

**I: How many mothers are given Faffa at your Kebelle?**

**06**: Children are many that receive that of Fffa and Mitimitta (Plumpy-nut); but I didn’t hear about mothers who take that of Mitimitta (plumpy-nut) and milk; yet, they were many while the Faffa was used to be given; but I don’t know their numbers.

**I: How about the mothers; are they pregnant or lactating ones who used to take Faffa?**

**03**: Most of them were those of mothers who had less than six months of children

**I: How many children are given that of Faffa and Mitimitta at your kebelle?**

**06**: I don’t know them for real. Please if you know them tell him well so that we can manage our time!

**07**: Those who were taking Faffa are many children; but those who were given Mitimitta (plumpy-nut) are few; who are around two to four. But for the Faffa, they were many children and mothers.

**I: How many mothers do you think given Faffa at your kebelle?**

**07**: They are both the pregnant and lactating mothers; they are many.

**03**: I think if there are 100 mothers the 50 will join that Faffa.

**02**: I don’t think all the50s, may be 20 to 30 out of the 100 mothers will join. It is based on our kebelle that can take that Faffa, so, they couldn’t be 50:50. It doesn’t exceed that of 30!

**07**: Yes, that is it; it is about 30. But even one mother is wasting!

**I: How about now, is the provision of Faffa and other services available?**

**03**: Currently, it is not available; as a result, it is not known that who is thin, and wasted from who is not! They closed the service.

**I: Why do you think it is closed?**

**03**: I don’t know.

**02**: Maybe it became beyond the capacity for the government; the problem is perhaps much. It is quitted since June 21^st^ (EC counting)

**03**: Indeed, we dint ask for the government; but if we ask even coming to this health; we are said that why are we begging why are we so expectants form others. If we ask why Faffa is not present, they told us that we have not to be expectant and we have to b self-sufficient; so, we don’t ask such things.

**I: Who is advising you not to be expectants and so to be self-sufficient?**

**03**: Oh! Any health professional who comes here for any work tells us like this. For example, you came here and told us your name; but others don’t tell us their names; they come here and we come here too; hen we are vaccinated and we ask them about it. Their answer is, doing be as such expectants!

**I: So, what are those thin mothers doing currently if there is on this service currently?**

**03**: They are still getting wasted! (She laughed and others too)

**07**: They may it what they have at home.

**06**: It is because we are given Dugoma (emergency aid) and safety net that the Faffa is not present. So, the pregnant and lactating mothers might be using that service.

**I: What is given in that emergency aid and safety net here at your kebelle?**

**06**: The safety net is given for six months per a year and it is of five years program. Thus, anyone who enters at first being it by chance or else, will stay for five years in this service.

**11**: It is given cash for two months and what and legume for four months.

**I: When are the two months of cash, and that of cereals?**

**02**: The cash is given during Easter fasting months; whereas, the wheat is finished up to June to July of the year. And 225ETB is per individual is given per a month; whereas, 15kg of wheat and 4kg of legume is given per individual if it is in cereal.

**I: Does the safety net come on time to you?**

**02**: it comes late; and sometimes it is given us at once. It was given to us on June-5; now we are waiting for the coming Easter fasting.

**07**: The cash comes on March and April; then after the wheat is given for May, June, July and August. However, the ration of July and August was given to us on June collectively and we are chewing that.

**I: Okay, you are saying that the Faffa is left due to the safety net and emergency aid presence; so what is given by emergency aid too at your kebelle?**

**09**: It is given what 15kg, legumes 4kg per individual and food oil of 4 liters per 8-9individuals. It is given for seven months per a year. But, it comes sporadically; it is not regular which sometimes comes late and sometimes comes fast.

**I: Who is beneficiary of emergency aid and that of safety net at your kebelle?**

**02**: The one who is poor in its properties enters to safety net program; whereas, the ones who are have around one pair of oxen, a cow and little goats are going to join to the emergency aid.

**I: How about the pregnant and lactating mothers, where are they classified?**

**02**: Let me clarify it; for example in 2009EC, I myself gave birth and I was given much Faffa; even my children were using from the Faffa I was to be given. And those other pregnant and lactating mothers were also given Faffa three times in that year from March to May in 2009EC; and it was given for all the pregnant mothers.

**I: How about now, how many of you are at safety net and emergency aid?**

**03**: I am out of any of the safety net and emergency aid service; though I don’t know why. Yet, we are working and eating. I think, it is due to we have two oxen and around ten goats that they let us out from the services.

**02**: Five of us are safety net beneficiaries, and two of them are emergency aid beneficiaries.

**I: So, does it mean all of you don’t have two oxen like hers that you are beneficiaries?**

**10**: No, we may have two oxen; but she exceeds us by the goats. (They all laughed pleasingly)

**I: Do you think lactating mothers are especially affected by malnutrition?**

**03**: Yes, in my opinion, it is due to shortage of food; it is common at our area; when you get one item, you don’t get another item, when you search for one food type, you miss another food type; you can’t get all you want at our place; this is reason in opinion.

**I: What else do you think cold be the reasons to be especially at risk of malnutrition in lactating mothers like you?**

All didn’t give response.

**I: Okay do you think that lactating mothers in this community are suffering from Micronutrient deficiencies like that anemia, night blindness, goiter and others?**

**03**: We are fine to our eyes; but there are women who have swelling to their necks. I have seen women that have big swelling which does have multiple dimensions at their neck; thus, they said the feel pain; as a result, they went to Samre to be treated; but, they referred them to Ayider. However, they didn’t go due to lack of capacity to go and be treated there. The one that I saw is at child bearing age that she can give birth if she recovers from her illness and she is here from our kebelle at Lemlem.

**I: How about anemia/low blood level to the body to lactating mothers in this kebelle?**

**08**: That low blood is not stay longer; if it happens, we go to the medical care, and we take tablets; then we become safe. There is no body that sit-down having low blood to her body.

**I: What is given to lactating mothers with anemia/low blood level?**

**03**: They are given food via their hands. Then they are also given red tablets for the low blood level and they come back home having that tablets.

**I: So, do they take the tablets all; is there any one from you who did take that tablet?**

**07**: Yes, I myself took that tablets; I brought medication for my stomach and that tablet and I took it already in these days; I am done with it at this moment. I finished all the syrup of for my stomach and that tablet for the low blood.

**05**: By now no throws that tablet away; it was in the previous times when we were not aware of its benefits that we used to leave it away; but currently we take it all; it fills our blood well then. So, how comes that we through that tablet when the government is bringing it to help us; this is unthinkable!

**I: How about night blindness (Himma) at lactating mothers in this kebelle?**

**05, 07:** We didn’t see!

**I: Are there mothers who are obese at your kebelle?**

**03**: Is that being healthy or due to disease?

**I: Maybe, they eat more; due to other reasons but who are very thick in their appearances?**

**02**: There are women who terminate giving birth; but now they are taking medication for hypertension. One is from village Kacherwa, another from Havramba village and third one is from Adi Akimada village who excessively swollen her body and her blood is overflowing but she is younger that she is at her child bring age like us.

**I: What do you think is the reason for their hypertension?**

**03**: Their blood is so ill; when sun hits them their blood overflows; they are told so by the medical care; they are told not to eat salt; if they eat salt, it let their body be swollen and let them fail. They are told not to drink coffee, and ‘Tella” (alcoholic local drink).

**I: How about that of diabetic mellitus (Shikoria), are there women at your kebelle with this case?**

**08**: There are no women that we know; but there is one man with that disease who is around 70 years of age; and he lives at Kachero village.

**I: So, do you think these hypertension and DM is related to feeding abnormalities?**

**06**: We don’t know! What did we learn about to mention about this; but what they have been said is like what we said it that they are told by doctors. They tell us that they should only eat that powder red pepper without salt and they have not to drink also that of ‘Tella’. Otherwise, what do we know about it. You know it those who you are educated.

**02**: Uh! As to our opinion, we said it again it may happen due to excess rest that they have; though we don’t know what professional will say about it; but we say it is because they have not adequate work activities to do, they have excess rest. Had they had adequate activities here and there, they wouldn’t have been caught by it.

**I: Do you think women/girls in this community would increase their height proportional to their age or are they tall enough or short including yourselves?**

**03**: You already recorded our age; and you are looking at our heights too; so; measure it using that. (She laughed pleasingly). Don’t you do that?

**I: I need to hear it from you?**

**03**: So, as to us, someone who is young may have tall height; and someone older may have short height; it is not same to the age in both males and females.

**I: But, why this happens so; that is young with tall height and older with short height?**

**03**: God created it; God is so autonomous to create everything; it is all at his hand.

**I: So, how do mothers look like to their heights at your kebelle based on your observations?**

**02**: we have medium heights; why we said so is that due to the area where we are living and due to the living standard that we have, for all we know, we are strong and it is even appreciated that we are walking erect! (They laughed). So, it is good height that we have.

**I: Do you think diet may have relationship to our height for being short or tall?**

**02**: Why not! There is awkward living not an awkward human! There are individuals who were short then turn to be tall enough due to they get comfortable living environment; once someone is tall, there is no problem; it will be fat enough and then the body will be strong; whereas, the shorts, when they get conducive environment, they are being changed. But that awkward livening is not even okay for the tall; it let his bone be crushed, and his meat be gone.

**I: What do you mean by awkward living?**

**02**: An awkward living is that always rushing for life but no adequate production; not free of begging, not having adequate rest; not having the food we want to eat; and this is due to shortage at home; more over to this, we are giving birth beyond our capacity because Saint Marry give us to deliver; but we have not the capacity to share what we have with our children; we have the deficit of supply.

**I: How about the issue of food insecurity; are their households who have food shortage, who may not eat their lunch or dinner or not at all?**

**09**: Is that about now alone or of the total condition?

**I: All about the situation here at your kebelle?**

**09**: There is no food; it is suffering; what kind of food do we have! For example, in this previous summer (rainy season) having our pair oxen, and our children even let them quit from schooling, we plowed and suffered a lot to sow the land we have. But now at last, we didn’t even get that of straw for our cattle let alone to get harvest for us! This is the indication of food insecurity.

**I: Then what is the community doing for living here in this kebelle?**

**09:** Sometimes, we harvest a bit fine for ourselves and also straw for our livestock; but since the previous two years and including right now, there is no harvest that we obtain; even the straw for our animals is relatively fine at this time that little is present as you can see it; but nothing we got for our consumption; now, even the animals are becoming load for us; we are worrying what we will feed the them for the future.

**I: So, how is the community living, what solution can be done then?**

**09**: We can do nothing; but what we have to do is that we have to let our children learn. And we are sending them to school to come out from our naughty living and to get better life for the future; and we are telling them to learn and to escape from this life. But in our case, we can’t go anywhere, we are done here!

**I: Now, there is no any harvest here that I am looking at; so, what is the community eating?**

**07**: We are really worried; the government is aiding us.

**09**: When a baby is in hunger whom to does he cry? The same is true for us! Those June and July are already passed; we suffered a lot during those months to harvest right now; but there is nothing! So, the same as the baby, when he cries, the parents brings him anything to eat from anywhere; the same is then for us that we will cry for our government.

**Section 2: Barriers to access and utilization of nutrition services**

**I: What kinds of nutrition interventions are in place to improve health of the lactating mothers in this Lemlem Kebele?**

**02**: Even that of Faffa for children and for those who are in hunger is already lost. Yet, our government is flawless though we become so resistant to be changes to the government. However, there is on any support to the pregnant, lactating mothers and children in this year of 2010EC.

**I: Do mothers visit to health facility; for example, do you visit to health facility here?**

**10**: In this health post there is no medical service except that of drug for malaria and for headache.

**03**: There is drug for headache, for diarrhea, and for malaria here in the health post.

**02**: No, there is no medication here; even that for diarrhea is that of English salt (she means Epsom salt) which replaces our food; this is health post. When we are critically sick, we go to Samre.

**I: So, what is done t you here in the health post?**

**02**: That is, they care us well and they send us to Samre giving us paper; Because firstly when we feel sick or when our children happen something, we come here; thus, they let us go to Samre to the health center. But here if malaria happens to us, we get that drug; we also get the drug for headache as pain killer and also if the diarrhea is critical we are given that of English salt to replace our lost food.

**I: How about for the children, what is done for them here if they feel sick?**

**02**: The drugs for eye and head here are bought by price; but that of malaria is given for free; but for others you pay your money. Yet, the other services like Faffa and plumpy-nut (Mitmitta) are not present at the health post currently; they are abandoned services; and we are unable to ask why; we are afraid of asking the government due to the reason that may become to the government beyond its capacity.

**I: How about in Samre, what services do you get there?**

**09**: At Samre, there is health center and also hospital; and stool and blood test is done to us and we are treated accordingly. If it is beyond their capacity and very critical, we are send to Ayider.

**02**: There is all examination there; if there is TB, they give medication.

**I: How do you go to Samre?**

**02**: The worst is taken by Ambulance.

**I: Please other, why don’t you talk; do you think you have to be represented only by one mother (02)? Let’s talk?**

**03**: You better give us the chance by yourself?

**I: That will not be an appropriate; it will seem like forcing you involuntarly; so, you better speak to your will.**

**03**: So, we are talking the one who are worried about the things!

**02**: Why worrying; it is because we understood our government’s teaching that we are talking!

**I: So, how much hours does it take to Samre on foot?**

**07**: One and half an hour

**08**: Two hours

**03**: The ones who carry take them three hours; for those elders two hours and for those children who can run one and half an hour.

**I: How about for mothers?**

**03**: We travel carrying either goods or child; so, it takes us three hours to arrive there.

**I: How about when mothers are sick and referred to Samre?**

**03**: If she is pregnant, she will be taken by ambulance; but if I feel sick or this my kid feels sick, I have to go on foot. It is allowed only for the pregnant to be taken by Ambulance.

**I: So, do you get all the services like diet counseling when go to Samre including at this health post?**

**06**: We are given the advice about what to do for our baby when he gets sick; they tell us we are getting sick because we are not eating. But when we are given the medications, what you call it, the medical insurance, we are registered at it and we are supposed to get the medication for free in the health center; but, when we go to the pharmacy with our prescription paper to that pharmacy in the health centre, they tell us it is not present thus let us go outside stamping out paper so that we buy it outside from our expense; this is the challenge we have.

**I: Who are they that let you to do so?**

**03**: The health workers; they give us some of it; but they tell us that some of it is not found with them; but, we already paid 143ETB for the medical insurance for the whole year to be treated for free.

**I: How much is paid for the medical insurance here?**

**03**: It is 143ETB

**02**: No, it is 250 ETB

**03**: That is of the current payment; but what I have paid in the previous year is 143ETB and I have to talk about what I have already paid!

**I: No problem, we can talk about the past and right now payment?**

**02**: So, in the previous it was 143ETB; but currently, for those who don’t pay on time it is said they will pay 250ETB including the payment for punishment; or, we will see if they let us all to pay 250ETB in this 2010EC.

**03**: But the problem is that when we have already paid the payment for it but we are not getting the free medical service; for example, this my baby was sick and I took him to Samre; then it was ordered to him five injections and fifteen tablets; thus, they gave the five injections at the health center; but I was send to outside for the fifteen tablets; then, I bought them from my pocket.

**07**: It happened the same issue for my child too; when I went to bring the medication for my gastric problem; I also took this my baby for treatment as he was sick; thus, they gave my medication for free but they charged me the price of the medication for my child. But we don’t know the reason why.

**I: Have you ever asked them why they send you outside to buy medications if you have paid the medical insurance fee?**

**03**: They told me that the tablet that is important to treat the illness was not present with them. They said they have not such kind of tablets with them.

**I: For example, how much did you pay outside?**

**03**: Twenty birr

**07**: The price for the syrup was 24birr; but I paid 12birr which is half of it; as I was worried about my child; I simply paid that half and came home; but I do not know why I paid only that half of its payment. It was inside that hospital which is the hospital of Ayider which is found at Meda Samre (one place part of the Samre town).

**I: Is there any place where you are given counseling about your diets; how do you get that diet counseling?**

**07**: When we come here to let treat our sick children, the health extension workers tell us about it; they tell us about how to feed them; they let them drink and eat. But in the case of going to the medical centers there in Samre, let alone to tell us about how to let them drink and feed for our children, even that medical care service that I got was worthless. They only give you that of tablets; no education and counseling at all.

**I: Is that at the hospital or at the health center?**

**07**: To the hospital, I went only once for this baby; but the problem is to the health center.

**I: What do you think that they are not giving education and counseling?**

**07**: I don’t know; but I came home paying my money.

**I: Have you ever asked them why you pay money if you are registered to the medical insurance?**

**07**: No, we dint ask!

**I: So, whom do you think will ask about your rights if you are not able to ask when it immediately faces you?**

**02**: Right here? If you they don’t go having their photos at hand and if you ask that whose problem is it, it is their problem; for example, I went to that health facility due to this my baby was sick at her 79^th^ days of age at the eve of her baptism just at the night time of 9:00PM. Thus, we went to the medical care, she was unable to breast feed; she was crying and unable even to eat. Then, at the beginning we had paid 142 ETB for the medical insurance; our problem was then her photo was delayed; whereas, photo of all of us other family members were posted in the folder; why it was delayed was because she was born after the 142ETB payment of the medical insurance; that was after we paid and we posted the other photos; hence, we went and we said to them that we have already paid; at the health center they said us that we have to bring them an evidence for our kebelle; then it was night and we paid to the health center; and they also referred us to the hospital there at Samre for further investigation to the baby; again there in the hospital they asked us to pay the money but to bring evidence about the baby. Because, the list of the eight of us is at the folder but this ninth baby is not registered there; so, we were to do nothing. Then we got back home paying our money and let treated our baby; and it was great that our baby is fine. And he kebelle even told us to take the evidence about our baby and so that to return our paid money; but we dint went back there; they even told us to get back and to take our money; it was possible to bring back our money but we didn’t do it. This is what I have faced in my case, we left the that of 80birr as our baby was fine and because it was bust time for us as it was the period of her baptism; and also we thought that that money was already sent by them to the finance minster so that might be difficult for them to bring it out back for us. So, may these be the cases for them too that they are raising them as problems is the concern I have.

**I: So, as you have already heard her, she is saying that if there is a photo of each family member in the folder, there will not be present the extra payment; unless, there will be payment for the one who is missed in the list; so how were your cases?**

**07**: My child has already the photo in the medical insurance folder.

**03**: In my case, what I am saying is that the medication is not present in the health center, thus, they gave me the paper being stamped and sent me outside; thus, I bought those fifteen tablets by twenty birr; mine is so different. I am saying that they have shortage of medications at their center; but I am not saying about not being registered in the medical insurance. I have asked to the one who gave me the injections and he told me that it is because it is not present that he send me outside; but the payment was not to be paid back by the medical insurance; I have already paid it.

**I: Which salt is better do you think?**

**04**: The iodine salt.

**I: Why do you think is it better?**

**09**: For health!

**11**: For abdominal cramp

**10**: To be protected from Guegueti (she means goiter).

**I: How many times do you think lactating mother like you should eat per a day?**

**02**: Five times; the pregnant mother should take five times per a day.

**I: How about the lactating mothers.**

**02**: The same is for hers too; for all I know, it is more critical for her. I think, pregnant mother should take five times per a day; and that of lactating mother should also take five times per a day by preparing home available mixture of food items and also enriched stew like form egg, and even flax and what she has all at home. So, mixing all these things together if she eats and also gives for her child, she will not die; we are taught well by the health packages though not by the health facility at there, Samre; but we may not capture it all what the health packages teach us as we are not educated. There in Samre, they only provide us the medication, but here the health packages advice us well even beyond parents’ advises!

**07**: Yes, these health workers counsel us very well here.

**I: What do you think will be the importance of eating diversified diets for your health?**

**09**: Do I excrete milk to my baby if I eat or not; it is when I eat. But if I don’t eat, let alone to excrete milk, for example, if we stay the day with no food, the babies will shout and we also be unstable. So, even though we don’t care well about ourselves, if we care well and if we eat well, it will help for our health; the baby will breastfeed well and will grow well. And does the one who eats well goes well or that not eats well; it is the one who eats well that goes well!

**I: Okay, great! And are there mothers who practice backyard vegetable gardening from you?**

**02**: It was used to be practiced well during 2007 and 2008EC by many of the farmers as there was fine rain seasons during those periods and the farmers were intended to work at developmental works. Even though there were not gardens at nearby homes, there were vegetables and fruits practiced by many farmers at nearby temporary river basins; but due to some cruel individuals who were accustomed to stealing the fruits and other plants; farmers become less interested working at such gardens and also the water scarcity happened due to the drought; now, there is on any practice of gardening at our kebelle.

**I: Who is stealing them and is there not any security here that can be appealed?**

**07**: For example, there is guava at the dried river basins which is not ripe yet; but they steal it being green; they are those who can’t see thing s properly; but we don’t know them who they are.

**I: So, do you mean you are not practicing any vegetable gardening like that of spinach, salad and others at your homes?**

**10**: How comes; there is no water!

**09**: Right here please! Let alone to get water for gardening, we are fetching water on our back for our consumption travelling many hours as our tap water was broken; so, it is unthinkable to practice that vegetable gardens.

**I: How is water access here; how many hours do you travel to fetch?**

**02**: Our water tap was broken; yet, we are dependant at it; our main water tap was broken a day before yesterday. Until then, we are fine; we don’t travel much distances; it is found at the center of the village.

**01**: May be it takes less than one hour for round trip

**04**: May be it is around thirty minutes.

**09**: Right here please! In this village there are around 200 households; what we are used to use are two water taps; there were many but broken most of them; yet form the two, the one is so salty which is not used. Whereas, the second one, when you intend to fill for two jars, it takes you at least three hours long.

**06**: No, it is about that mater of turn!

**09**: So, that is what I am saying; it is because we have not adequate water taps!

**08**: But, we are not fetching from rivers.

**02**: We don’t bring from rivers.

**09**: I am not saying from river; we have appointed guard in that water rap and he let us use turn by turn properly that single water tap; but if we send our children to bring water at 8:00 o’clock; they stay there up to 10:00 o’clock; that is the problem we have.

**I: Who fetches that water?**

**07**: Just females

**11**: If you have children, they will fetch.

**03**: If the children go to school, the one at home will fetch (mother); but the children being female or male if present, they can fetch. It is brought on donkey back or on one’s own back if on donkey.

**I: So, how many water taps are working currently?**

**02**: Now, only one water tap is working; the rest five are broken; they slept! Enough! And the problem is to the water resource office that they are unable to repair them and let us use. For example, one of the taps is of less damage which can be repaired easily using cement; it is not entirely dead; but they dint do anything!

**I: Had there been practice of backyard gardening at your homes, what would have benefited you?**

**05**: It would to be so delicious diet; it would to be assimilated to the body if we ate vegetables. It would to be good diet; but we are not practicing it due to lack of water.

**I: So, how is your hygiene and sanitation practice going on if there is no adequate water; and are you being counseled about it?**

**02**: Yes, the health packages (She means HEWs) counsel us well about how to keep our environmental and personal hygiene and to use latrine; they tell us that we have to clean our surrounding, we have to clean our children, and we have not let our children not be so clean. They also advise us about not to be so weak in our cleanliness not to be seen any feces in our surrounding; and this is practically being done.

**I: How is malaria problem at your kebelle?**

**02**: There are some cases of malaria illnesses currently; it is observed in the areas where swamp is found. And especially in the village called Adi Akmada, there are many cases of malaria, due to the area, is not sprayed. And also at the village called Kacharwa in our kebelle, there are some cases observed though it is relatively fine. But in the case of Adi Akmada, there are many individuals much suffered from malaria and they come here to the health post to collect drug.

**I: Why is that Adi Akmada Keblle being victim of many malaria cases?**

**02**: It might be due to that of shortage of water and the area is very hot too. There is water there; so, what can we say then?

**I: Is there not any protection material for malaria given to the community in these kebelles?**

**02**: There is Zanzira (she means ITN); but we are not given; there was some on Gunbet 2009EC (May 2017) came to our kebelle; but some individuals took it. It was few, then few individuals took; but many of the community didn’t take that; for example, I was newly lactating mother during that period and I dint take it.

**I: For example, is there anyone who doesn’t have ITN (Zanzira)?**

**07**: No, we have not it! For example, I myself brought my three children to this health post because they were caught malaria; but they give me their treatment; they said they were caught malaria. For example, the sprayers come to our Kebelle around Hamle 25 to Nehase 2009EC (July to August 2017), but during that period I was at the health post taking all my three children one of them hugging at my chest, the second one cling at my back and the third one catching his hands, then they got the treatment; and when I got back home I found the sprayers at my home. But because the children were infected before the spray due to, I was not given that Zanzira, I come to the health post three times. Currently, they are fine but they were hurt by the malaria all the summer!

**I: How about now, is there malaria occurrence at your kebelle?**

**09**: Yes, there are cases; they fall down and go to treatment; we didn’t say it is totally avoided. There are cases still; for example, I brought my children here to the health post and the health extension gave me tablets which have human picture at their covers and which have their own hours to be taken. She gave me based on their ages; but I dint get any hope; again she said that it is malaria and she went to Samre and brought tablets from there and she gave them again based on their age; now they feel better though they are bit disturbed if it is cloudy condition. Yet, they are now fine and able to stand and move.

**I: What do you think the Zanzira was not adequate while it was distributed?**

**03**: They said it was not adequate; it was given only to those who were at public work during that distribution date. But we the pregnant and lactating women during that period were not able to get it as were at home; they were not able to mobile the community to take it as it was not adequate to be distributed for all.

**02**: It was given on around Ginbot (May) 20s 2009EC when I was newly lactating mother; and it was said that was little. Indeed, we shouted at them why they didn’t give us but they said it was too little.

**05**: It was those who were in the safety net work that they were given as they were there during its arrival.

**I: How many individuals may be caught by malaria at this kebelle?**

**10**: Oh! How do we know them; the HEW has the report.

**02**: I remember one day, when we have had meeting here in the health post, there were two children and three men who came for malaria treatment.

**I: So, from the many interventions done here for mothers until then, which ones do you think are the most successful; for example, health, nutrition, WASH and others too?**

**02**: It has not sustainability! The main thing is that it is it is dwindling! The reason not to blame it is that due to it is entangled with many tasks; otherwise, even the health doesn’t have sustainability; even the water is like what we have said it. However so, we don’t know; either it is due to it is beyond capacity or we are all waiting from it; it let us be calm not to blame it completely. Otherwise, there is on sustainability; it is dwindling!

**I: What does that dwindling mean?**

**02**: It is only the start that is seen; there is on finishing!

**I: What else could you say about this issue; others?**

**03**: The reason why we are saying it good collectively is because when we compare it with the period of our mothers that were many emergencies during delivery; they were facing many accidents and also they were dying during that period; otherwise, if we see that Ambulance at this time, it is transporting both the pregnant and lactating mothers to medical care. But for example, I myself went on foot to Samre when I was pregnant to give birth there; this was due to there is ambulance sacristy and it was unable to rich for my need at that time; and this happens due to the reason that we are many but the ambulance service is little. So, when we say it is good, it is to mean that the government is thinking about us though it has not adequate service to address all what we need; that is why, I went on foot to Samre thinking that I would not get the ambulance waiting at home due to there is scarcity; so, I went there and I gave birth; but it doesn’t mean the government is not worrying about us. But the name called is that we are going there by ambulance and we are getting back home by ambulance! Yet, the ambulance is serving well for emergency conditions too.

**07**: We all go to the delivery n foot; but the car is said to be for pregnant. But you guess; if you fear that the car will be present due to other conditions, you go no foot and deliver there; for example, I went on foot to deliver at Samre; but I got back home by that car.

**02**: Right here please! The ambulances were so many in numbers; but if we need to see it widely, where are the cars; they are crushed all! They were about three or four; but only one left. In my case, I dint go to the health setting prior to my laboring like what my sisters have said it which is important to protect any problem occurrence staying at health center in advance to the labor. But I didn’t do it due to my own problem; but suddenly, Saint Marry come to me (she means laboring happened) then I was waiting Ambulance that phone was called to many offices including the water resource office; at last, they said ambulance is not present; it is referred to Mekelle; then I decided to go on foot slowly to Samre; they said me to wait until it comes from Mekelle but I refused and travelled. Whatever so, I arrived at Samre on foot being in pain; but, I gave birth at the town at my brother’s home before arriving to the health center. It was while I was giving birth to this baby who she will be six months of age on the Michael day 12^th^ of the month (EC counting). Then after I gave birth at brother’s house, the Ambulance helped me and took me the health center; and I was treated there; my placenta removed and cleaned well there; I was fine. So, the problem is that those non-functional ambulances and the current one is only one. But mainly, the heath extension tell us that since there is no adequate ambulance to take us to Samre, we have to go one to two weeks in advance to our labor initiation to be safe form ambulance shortage. But in our case, we don’t attempt to go leaving our children at home; we worry for them not to be in hunger of their lunch and dinner. But had we gone there in advance to our labor there, we would have gotten adequate rest; we would not have sacrificed a lot at home for preparation of lunch and dinner for children. This is our concern and problem. Whatever so, this could not be a blame to the government.

**I: What barriers are present in the implementation of the service that you would be expected to get?**

**02**: We would expect the services to be full and sustainable! We would expect the emergency medical care to be here for treating snake bite and that of cattle swelling (she means to anthrax); we would be happy if that of water taps were repaired and handed over to us! We don’t know either the government is waiting us to repair it by ourselves as it already gave us once; the same us the parents let marry their children then expect them able to help themselves by their own efforts. Yet, the medical care needs much to be worked; we are not satisfied yet by it!

**I: What are the things that are not let you satisfied in the medical services?**

**02**: That of snake bite, that swelling (anthrax) which are very emergency conditions; had emergency medical care been here; there would not have been an accident to any individuals here.

**I: What is that swelling about; can you tell me in detail?**

**09**: It is called of cattle; it swells anywhere except at our teeth which might be it would have been slide from it (Laughed). Then it doesn’t give time until we go to Samre; it kills! It starting with itching, then if it is scratched, its periphery seems to be healing but it middle becomes black and creates inside hole. If you drink Sawa (the local alcoholic drink), it kills you.

**03**: It can kill within you three to seven days. And it attacks all male and females.

**06**: It is said of cattle which have been said cultural from our previous parents. But we don’t know why it is said so. It is also called Megerem!

**02**: It is given injection to treat it. For example, a two years of female child was caught and she was given 16 injections two years ago; now she is four years old baby.

**I: Is there snake bites here; are there individuals who died of it in this kebelle?**

**07**: My daughter died of that snake bite in the previous year; she was 13 years of age and she was grade-8^th^. And four hens were died due to that snake bite; that is, after the snake bite her, the site is incised by blade and the live hen, was but by her anal orifice to that bleeding area so that here opened anus will such the venom and will let save the girl; but four hens were tried but all died due to the venom; and she was not relived from the poisoning as it was disseminated to her brain; after that we took her to Samre health center; they sent her back to the hospital there in Samere; she was treated there but not saved; I lost her! Here is also young boy is died of snake bite ten days before her at our kebelle. Others are also being bite but may not die if they get the treatment sooner.

**Section 3: Perceived needs of women for relevant services during pregnancy**

**09**: Right here please! In the case of health service, there are real problems; first, the guest from high level tell us that there is a killer disease that kills within 24 hours so that we have not send our children far away to the bush areas; otherwise, they will die; so, what shall we do; what if we are caught with that disease; how can we arrive there within 24 hours then? second, why individuals are dying due to snake bite is that due to absence of emergency medical care at our keblle and due to shortage of ambulance that when somebody is sick or being bite we go out to search for someone who can carry and travel; then the death will happen; third, the delivery happening at the way to Samre is again due to lack of delivery access at our keblle and shortage of ambulance. So, these things have to be resolved.

**02**: We need also to have good road access; another challenge for our health service is the bad road that we have. It is so weak! Yet, there is positive change when compared from the previous times that there was even the road that we have now; however, it needs improvement; it has not sustainability.

**09**: We have also another problem that we need to be addressed; that is, if we need to teach and feed well our children there has to be present school here; so, currently there is junior school up to grade-8^th^; but it has to be extended at least up to grade-10^th^. Even the current class-rooms are ruined and the students are learning being in difficulty; so, even the present one has to be renovated.

**Section 4: Other interventions that improve lactating mothers’ nutrition**

**I: Have you ever gone for nutrition screening during community health days; and what is done there?**

**02**: Yes very well. I said it earlier about the uder-5 years of age children; those who are thin go to their Faffa; and those who are fat enough take their other medications (she means like vaccinations) and they get back home. There is also a medication said to be for eyes which comes either once per a year or like that which is given for all being it adult or child; so, they (she means the HEWs) treat us well. And that of screening and measurement for children and pregnant mothers come once at six and/ or three months; thus, all are screened and measured those pregnant women and the children; then those who take vaccination take that and those who may be thin are send to Faffa.

**I: Where is that community health day undergone?**

**11**: It is here in the health post.

**02**: they undergo it in each village of the kebelle; because this is our village, they invite us here to the health post. They also go to another village and they measure them being under shelter; and same under shelter they measure at another village.

**I: So, what is given after the screening and measurements?**

**08**: They give medications like tablet, droplets, and also Faffa. Those who deserve Faffa are transferred for faffa and those who are healthy take their medications. Then again in the next three months or second round, they measure us all and they recheck our status; thus, if there is someone to repeat the Faffa; they repeat, and if healthy be thanked and come back home.

**I: Do you think this community health has importance for your health?**

**04**: Yes indeed, the one who is health and fat enough becomes happy when she is said she is healthy and again get happy and happy; because nobody hates to be healthy. But the ones who are thin will be blamed and will be claimed that hwy they are thin or if their children are thin they will be asked why they let them be so; why they didn’t care them well. Yet, if they care them well they will be thanked and let back their home. And the one who is not thin, will be happy and will get back home having her other medications to take at home.

**I: You said there is no currently that of Faffa for pregnant and lactating mothers and children too at your kebelle; do you think it would be important if it had been present?**

**10**: Those children who have already got that Faffa are grown well; but we don’t know what will happen to those children who are currently thin ones and not getting Faffa.

**07**: It helped the children to grow well.

**03**: Those children who used to be thin become so fat and smart. And those children, who were even ugly to be seen, brought them beauty to their body.

**I: In the safety net program, do pregnant and lactating mothers participate at public works?**

**01**: Any mother who is pregnant, starting from her pregnancy up to the tenth month of her lactation doesn’t participate at public works.

**I: How many months is a pregnant mother given a rest from public work in this kebelle?**

**08**: It is form her sixth month of pregnancy for three months up to her delivery period.

06: Then she is given ten months of rest after her delivery; the baby has to get ten months of rest.

**I: So, do you think his rest is important for the mothers, what does it have?**

**04**: Is that questionable about its importance to the health of mother and the child; if they able to get the rest and if she able to breastfeed her baby being free from wind and sun!

**10**: Yes very well; we care and clean our babies; they tell us not to let our babies hurt by sun and wind. Then after the tenth month, we start the work.

**I: So, shall it be continued or let it be stopped that exemption from public work for pregnant and lactating mothers?**

**07**: Let it be continued!

**09**: Rather, for a good thing, it has to be added another good thing; how do you think it has to be so! It is the bad thing which has to be stopped; not the good thing! (Laughed)

**Section 5: Understanding perceptions of age at first birth and birth spacing**

**I: Do you think delaying the age at first birth to after 18 is better for the health of the women, and is promotion is provided here at your kebelle?**

**I: Please don’t you say something about it? For the number (01, and 05)**

**03**: Yes, why don’t you talk about it, you are grade 10^th^ and 12^th^, you can talk more about it; let alone you, we the non-educated ones are talking a lot!

**02**: Indeed, they are students yet, they don’t know the social life of the community yet. They are not politically ripe yet. So, we better not nag them much. (Laughed)

**I: Okay; so, let’s talk? Is there promotion about this preventing early marriage here?**

**02**: They don’t marry before 18 years of age; if they need to marry; they have to be passing via women affairs and the health checkups about their ages. They have to tell their ages before going to the marriage.

**07**: Yes, they have to be checked first.

**I: So, who is promoting this message at this kebelle and what is said about it?**

**02**: About the early marriage, if the girl marries before her 18 years of age, she will be pregnant and she will face accidents during delivery. So, don’t send your daughters before their 18 years of age, it has to be testified by women affairs they said to us.

**I: Who are those who teach this message?**

**02**: It is those kebelle leaders and those health extension workers. Especially that women affairs’ head is mainly working at this issue; and also all the kebelle leaders; they tell very well. And even now, they are telling us to register them since their birth; and we begin to register them. And also the HEWs are letting us register our babies so that it may help them to trace back their age later if they need it to testify for marriage. And I think it will be convenient for all of us as it is already started.

**I: Do you think this early marriage prevention is important for the health of women?**

**02**: Yes, it is important to determine their ages once it is registered at the folder; we don’t worry about remembering their ages; because it is said that silly and paper never lose what they have caught! So, it is important for us the non-educated and for those even educated ones that they don’t need any witness.

**I: How about not marrying below 18^th^ year of age; is that helpful for the girl?**

**02**: Yes it helps her!

**I: Who else can tell me about this issue?**

**I: Okay!**

**Section 6: Understanding communication and information sources**

**I: are there any other additional groups that can teach about maternal nutrition for you that this kebelle?**

**02**: Those development armies both females and males; those who lead one to thirty and one to five.

**I: What do development armies tell you about nutrition?**

**02**: They tell us about child feeding; whatever, they help the HEWs that they tell us to keep our personal and environmental hygiene, and to keep the cleanliness of our children; and the women development armies too; they teach us like this. One tells for her five women in the network and that another one also tells for her thirty members in the network; the government has already joined us to each other; but, we are still learning the Ha, Hu (She means like the ABCD…).

**I: May this network helpful?**

**02**: The things that would have been tiresome individually are able to be solved simple being together in network. So, it is important.

**I: Is the education via network accessible to all women?**

**07**: Yes, it is accessible?

**I: Who are your most successful educators about your health, diets and hygiene from the many groups that comes to you?**

**09**: They are teaching us well abut handling of our goods at home, cleanliness and our health; and we are doing accordingly. (She means the HEWs)

**Section 7: Additional remarks**

**I: Okay, any ting left that you want to say?**

**02**: It is enough for us now.

**03**: If any left, you know it and you can tell us! (Laughed)

**I: So, to speak honestly, thank you so much giving us your golden time being with your kids! God bless your kids and long live to you all!**

**02:** Let’s clap our hands!

They clapped all!

**Summary (Home take messages**)

**Section 1: Common maternal, lactating women nutrition problems in the community**

That agriculture is enough! We are the ones who are cursed that we are living in this agricultural work! But for those who are educated the government should increase job opportunities so that they have to be involved there.

**Section 2: Barriers to access and utilization of nutrition services**

Let alone to get water for backyard gardening, we are fetching water on our back for our consumption travelling many hours as our tap water was broken; so, it is unthinkable to practice that vegetable gardens.

**Section 3: Perceived needs of women for relevant services during pregnancy**

Emergency medical care should present at the kebelle; because, individuals are dying due to snake bite is that due to absence of emergency medical care at our keblle and due to shortage of ambulance that when somebody is sick or being bite we go out to search for someone who can carry and travel; then the death will happen.

**Section 4: Other interventions that improve lactating mothers’ nutrition**

Those children who have already got that Faffa are grown well; but we don’t know what will happen to those children who are currently thin ones and not getting Faffa.

**Section 5: Understanding perceptions of age at first birth and birth spacing**

Registering the age of girls at their births is important for us the non-educated and for those even educated ones that they don’t need any witness in determining the age for early marriage.

**Section 6: Understanding communication and information sources**

Those development armies both females and males; those who lead one to thirty and one to five networks are playing their role in addressing nutrition counseling to mothers.

**Section 7: Additional remarks**

Let’s clap our hands!
