# Supplementary material for: Barriers to utilize nutrition interventions among lactating women in rural communities of Tigray, northern Ethiopia: An exploratory study
Source: PLoS One. 2021 Apr 30;16(4):e0250696. doi: 10.1371/journal.pone.0250696 (PMC8087028; doi:10.1371/journal.pone.0250696)
Supplement: S2 File — (ZIP) [file pone.0250696.s002.zip › S2_File.Doc/Lacatating women_IDI & FGD/065_IDI_lactating women_Lemlem Keble_Samre Woreda.docx]

**Operational Research on Adolescent and Maternal Nutrition in Northern Ethiopia**

## In-depth interview responses of the lactating mother

**Introduction**

Thank you for your acceptance of the informed consent form and for taking the time to speak with me today. I have questions to ask you which were prepared in advance. The discussion will take 1-2 hours. If you have any questions before we begin please feel free to ask.

**Section A: Interview in details**

1. Zone: South Eastern Zone
2. Woreda: Saharti Samre
3. Kebele: Lemlem
4. Name of key informant: Gideta Glselassie
5. In-depth interview participant: Lactating mother
6. Interviewer name: Mekonnen Haileselassie
7. Date of interview: 02/03/2010
8. Interview start time: 4:20 AM (local time)
9. Interview end time: 6:00 PM (local time)

**Section B: Socio-demographic and basic data of qualitative study participant**

| **Socio-demographic characteristic** | **IDI participant** |
| --- | --- |
| Sex | Female |
| Age | 25 |
| Educational status | 4^th^ grade |
| Occupation/role in the community | Farmer |
| Marital status | Married |

**Note:**

I: interview

P: participant

**Section one: common maternal lactating women nutrition problems in the community**

**I:** What do lactating women do to stay healthy in this community?

**P:** We are farmers and if women need to be healthy, there must be improve their feeding style by eating different cereals and pulses; being we are farmers, our feeding style is not new; we eat what is obtainable at home. We eat bread, injera with salt and አደንጎር; but we don’t give great value for the consumption of diversified food although there is not such food access problem in the community. We don’t give value for the consumption of variety food and we have less awareness about the importance of consuming balance diet food. We eat the common foods offered at home; but all the pregnant and lactating women are networked and get education from the health extension workers about their feeding style.

**I:** In your opinion, what are the common nutrition problems in the community for lactating women?

**P:** There is shortage of edible oil at the public market; if you want to prepare food for our consumption we face the shortage of oil; although this year is a drought season, we produced adequate food from our agricultural farms and it is sufficient for our family. I am a lactating mother and if I want to eat porridge, how could I eat without oil; there is no oil access at the market; so due to the lack of oil I eat injera with salt or only bread for the sake of survival. If the oil is found in Kebeles, only a liter is distributed among many households. For example we are five families in the households and we use one liter for more than one month.

**I:** Why shortage? What is the cause of oil shortage in lactating women?

**P:** There is no edible oil at market although the reason is not known. If there is no oil, I don’t eat and prepare the diversified foods; I only eat bread and I do have less appetite for other foods; this could contribute to become thin and poor body condition, and we could easily affect by diseases. Naturally I don’t like shiro wot, I used bread and if I find oil I could prepare ስልስ. Therefore, due to the shortage of oil at the market, it could cause less consumption of diversified foods by the lactating mothers.

**I:** How sever the nutrition related problems are among the lactating women in this community?

**P:** There is no program based feeding system among the lactating mothers. Although the root cause of thinness is not known, most of the lactating women in this community are very thin. In my view the thinness of most lactating women could be due to the engagement of high workload in the agricultural activities, poor feeding system, travelling of long distance to fetch water. I travel more than one hour of round trip to fetch water and at least two round trips are expected per day. To visit the community market, we also travel more than five hours of double trip. Having this long distance of journey among the lactating women, it is difficult to have good body condition.

**I:** Do you think that lactating women in this community are suffering from micronutrient deficiencies (anemia, night blindness, goiter and others)

**P:** Yes, for example I am the victim of anemia. During my pregnancy time, I took iron folic acid after the health experts were confirming my health status in Samre health facility. I found a change of improvement after my consumption of the iron folic acid.

**I:** What was the cause of anemia?

**P:** As per the health experts’ education, it was caused as the result of food deficiency. The lack of timely feeding system and lack of getting diversified foods are the main causes of anemia. As per the recommendation of the health experts food types like salt, potato, meat, shiro wot, bread, injera, porridge, egg and milk are main food items that could prevent from anemia.

I did not come across with night blindness; in our community, it is mainly observed in elder women. Goiter is not our problem; it is mainly observed in highland areas. Here in the community, everybody uses iodized salt and it uses for the prevention of goiter.

**I:** What diet related non-communicable diseases are common among lactating women in this community?

**P:** In our community, this type of case was not observed. Only the high blood pressure is observed in one elder woman of our community. She is more than seventy years and she is fat. Now she stopped drinking of coffee. I don’t know the cause of this disease; but it is very bad disease that blocks the blood vessel.

**I:** Why lactating women in this community would not increase their height proportional to their age?

**P:** You can easily observe everybody here in our community that there are unbalance height and weight to their age proportion. We also get awareness from the health experts in the health facilities, how much we are thin and low body weight. The main reason of stunting in children is due to poor treatment of the child since its infant age. The lacks of timely breast feeding, quality food, cloth, hygiene are the factors that cause child stunting. Therefore, the mother is the main actor to treat her child so as to prevent child stunting. Once the child has transferred into food after six months, you should give the child meat, water, a cup of soup, porridge, and the like. It is not necessary that you could bring new things beyond your capacity; rather you prepare the home grown foods in a hygienic way. Compared to the previous time, the feeding style of the mothers and children is better now and every woman treats her child properly. For the benefits of their child, the lactating mothers become consume in a better way. Generally due to the training given the health extension workers and their strict follow up by visiting to home of mothers, it is observed better improvement in the mothers’ body condition and their child development. Formerly, the child was growing with big head, pumped stomach, edematous leg and thin neck; but now this type of symptom is not observed in our children in the community. For example I have a chicken and if my child starts feeding, I will feed him properly as per the health experts’ recommendation like egg, porridge, soup, bread etc. in addition to the breastfeeding until three years.

**I:** Is there a situation when lactating women suffers from shortage of food?

**P:** Yes, for example I eat mostly bread and injera with salt; although we produce teff, sorghum, pulses, I only eat bread and I don’t have good appetite to eat food; this is due to the lack of oil. Mostly we didn’t eat a balance diet foods; sometimes the life style of our community could not invite to eat diversified foods; this is due to the engagement of high workload, lack of spices that give appetite, no vegetables and we don’t give more focus to eat a balance diet foods; we only eat for maintenance and diversified foods are considered as luxurious foods. There is also a shortage of food in some seasons and as the result we could suffer by hunger unless we get emergency aid from the government.

**I:** How frequent does the shortage of food happened? Why? What other problems do lactating women in this community suffer from? Why?

**P:** Unless, there is drought in this community due to the shortage of rainfall, the products from our agricultural farm is sufficient to feed our family; now days the drought is occurred at every other year. Shortage of food is very common during the drought season and mainly the pregnant and lactating women and children are the vulnerable groups to food shortage.

Awareness of the lactating mothers about the importance of diversified food is poor. We lactating mothers mainly focus on our routine activities and we don’t give value for our consumption of balance diet foods. We want only to fill our stomach regardless of the quality and quantity; we eat only bread and injera with shiro wot. The shortage of fruits and vegetables, and our poor practical application of what we have been learned by the health extension workers are the main problems of our community.

**I:** How do you evaluate nutrition with food security?

**P**: As I have discussed before, agricultural farm production depends on the presence of rainfall. This community is the most food in secured area; drought is commonly observed every other year due to the lack of rainfall. The community could not survive independently without some emergency aids and the food safety net programs. There are many problems that could face to the pregnant and lactating mothers as the result of food insufficiencies; they face high workload to search food for their family; and when the husbands go to other areas for search food, the pregnant and lactating mothers could serve as the head of the households. Therefore, drought mainly affects to pregnant and lactating mothers including their children.

**Section 2: Barriers to access and utilization of nutrition service**

**I:** What kinds of nutrition interventions are in place to improve health of the lactating women in this Tabia? Where do they get it? Who provide it?

**P:** There was a food aid package for pregnant and lactating women from the health center like fafa and plump nut. During my pregnancy, I had been given five kilograms of fafa after I had got screened; but now it is stopped it. There is no any food aid to the lactating mothers; however, the advice is given every time from the health extension workers how to prepare and consume the home grown foods so as to substitute the fafa that was given by the health center. They also advise us to keep our personal and environmental hygiene, and to drink hygienic water. We have been given an advice for lactating women to vaccinate our child every month. For example I bring my child to this clinic every month to get vaccination; until now my child has been vaccinated three times. I also came to this clinic to get drug for birth control. I took also the measurement of MUAC and I became below the standard and then get advice to feed balance diet foods like soup, milk, egg, vegetables; but due to the lack of time and food resource like oil and sugar; I didn’t apply the advice of the health extension worker that is why I am thin now. For the malaria prevention, they provide us insecticide treated bed nets. But this we didn’t get the whole community due to the shortage of the bed nets.

Compared to the two communities, more advice is given to the pregnant than the lactating once. For example I was measured the MUAC during my pregnancy and I had been given five kilo gram of fafa and plump nut. But now during my lactation time no fafa ws given after I was measured the MUAC; only they advised me to consume the home grown foods like porridge, egg and soup.

**I:** Do you think that lactating women receive advice on the need to get extra meal? How?

**P:** The health extension worker gives us pieces of advice, what and how the balance diet food could prepare and how we consume it. After they measure us the MUAC, they advise us to eat the diversified food types like egg, milk, the porridge of red teff, they also advise us to use vegetables. But due to the lack of water source we don’t have home garden vegetables. It could take around one hour of round trip to get water from the source.

**I:** What about on the need to be involved in safety net programs?

**P:** I don’t involve in safety net programs. This program is intended only for poor individuals with no any domestic animals. In case of us, we have agricultural farm and domestic animals like oxen, cow, donkey, chicken; and that is why we didn’t involve in safety net programs. We only engage in the emergency aid during the drought season.

**I:** Do lactating women get advice on water, sanitation and hygiene services? How do they get it?

**P:** We get advice on the personal and environmental hygiene and sanitation from the health extension worker. Based on their advice, we keep our personal hygiene properly. All the waste products like the leftovers of animal feed and the ash mix and dumped into the pit to produce compost. Although we have the shortage of water, we don’t compromise to our personal hygiene. I bring water at least two round trips per day. Therefore, our personal and environmental hygiene is well kept. Since our source of water is brought from the river then to avoid any contamination of water, we are provided the guard water to add to our drinking water. We also use a tippy tap technology to facilitate our hand washing interests. The tippy tap was built using jar can, string and stick; by tying one end of a string around the neck of the jar can and the other end of the string near the bottom of the jar can; and by hanging the jar can on a stick with a Y-shaped end; the stick is securely in the ground and stable enough to stand holding the water-filled jar can. This technology was shown by the health extension worker. Due to the presence of this technology, we are not exempting to wash our hands and face; we are always washing, after using any waste substance, after cleaning young children, before preparing and eating food, and after eating food. In this case, our personal hygiene is kept very well; and for sure we are not exposing to any disease like acute watery diarrhea.

**I:** How about the prevalence of malaria?

**P:** Since this Tabia is a low land area, there is high malaria prevalence; the health extension worker provides us insecticide treated bed nets for the prevention of it. But this year, we didn’t get enough amounts of bed nets. For example, this year I didn’t get the net; unless you are keeping your turn first, you could miss the service. The service is not given priority to pregnant and lactating mothers; rather it is provided with the principle of first come first served.

**I:** Is there a situation in this community that you think lactating women need to be addressed through targeted supplementary feeding? Why?

**P:** Yes, during my pregnancy, I had been given fafa and plump nut after I was screened; but in lactation period I didn’t get the service. I screened once and I was found below the standard; then she advised me to eat quality foods like porridge, egg and soup in my home. I hope the targeted supplementary feeding service is given to pregnant rather than lactating mothers.

**I:** Which of the interventions that you listed above do you think is most important for lactating women?

**P:** Pregnant women should conduct the medical checkup at the health service to know their health status. For example I took fafa after screening in the health facilities. Fafa that was given during my pregnancy is the important food item, that improved my body condition and I was feeling very energetic. The other important advice is keeping personal and environmental hygiene; we can easily protect from different diseases like acute watery diarrhea, malaria. If the environment is cleaned, no mosquito could breed around your home.

All the advices that were given by the health experts are very important to us. For example, we are advised to eat diversified and extra foods during pregnancy and lactating time; so it is essential to get healthy the child and to the mothers. For example, as per the advice of the health extension workers, I ate more foods during the first weeks of my lactation time like porridge with butter, honey, bread; at that time I was fat although I am reducing now. The stunting is caused by the mothers’ lack of eating diversified foods.

**I:** What are the barriers in the implementation of these nutrition services during pregnancy and lactation?

**P:** The barriers to eat diversified food are due to the shortage our economical source; for example we mostly encounter the shortage of oil; if no oil all my appetite is blocked. We couldn’t get vegetables, meat, butter and the like in the easy way. Access of different food types at nearby settings is also another problem. For example our market site is far away from our home; it takes more than five hours for round trip. Our awareness on the importance of nutritious food is also poor. We don’t use the home grown foods appropriately as per the health experts’ advice. We don’t focus on the diversity and type of foods rather on the quantity; for example if I don’t get hungry regardless of the food type, I don’t bother to the variety and quality of the food types.

**Section 3: Perceived needs of women for relevant services**

**I:** What special things do lactating women need in your community?

**P:** As I have said before, we are advised to attend the health facilities after post-delivery of the mothers. For example, I came four times to the health center mainly to vaccinate my child. I came only once to use the birth control. Although we are advised to come to the health center, we more active during pregnancy rather than at lactating period; because we have less understanding about the importance of visiting to the health post after delivery; we thought that already the stress is over during pregnancy. Had it been the fafa aid during lactation like the pregnancy time, it could be nice to attend. But if my child gets common cold, I visit to clinic to treat him.

In case of extra meal, we are advised to eat more food during lactating time like porridge, bread, egg and these foods could help to open her breast nipple; since these food types are very important for the child growth. Most of the time we didn’t apply practically the advices of the health experts because there are a number of factors that inhibit to apply it such as; shortage of food sources, lack of transport access, high workload among pregnant and lactating mothers.

In case of workload; being we are living in the rural communities, there are many activities that make ourselves busy. We are very much tight in the collecting of animal feeds like straw, forage, the left overs of sorghum and since the animals are tied at home we bring water from the liver. In addition to this, I clean the entire animal’s faces, and all the home activities are done by me. Thus sufficient rest is not expecting in such phenomena. After the child has been slept, I and my husband are harvesting all our agricultural crops; thus I am very much busy during the harvesting season; even I don’t have enough time to treat my child properly during this season.

**I:** What should be the role of a husband to improve nutrition for lactating mothers?

**P:** My husband is very cooperative; he shares his part to do in the home activities like bringing the fire wood, in collaboration with me, he collects the agriculture products, sometimes he brings vegetables from the market and the like. But I am doing all the agricultural field activities equal to my husband in addition to my house work responsibilities.

**I:** Do lactating women in this community typically change their diets during lactation?

**P:** Relatively the preparation of extra food for lactating women is better than for pregnant ones. However, the preparation of foods for lactating mothers is only for the first two weeks of lactation; for example I ate meat, packed juices, porridge with butter and soups. For lactating women, porridge is important to strengthen our body; thus we should eat porridge until the baptism of her child.

During lactation eating injera and drinking water is not recommended; until the first seven days of lactation, eating injera is not advised; because it has no any energy that could give strength to mothers. Drinking water has also an effect to exacerbate the bleeding of mothers; instead it is recommended to drink local alcohol (ስዋ) which uses to increase the milk during breasting of the child. During pregnancy also ቀሎ፤ ንፍሮ are not recommended to eat which could cause pain to the child.

**I:** Are there gender disparities in women’s diets during lactation?

**P:** In past time, this type of culture existed and it may be also exist in some households still now; some women prepare the especial food like chicken, egg and butter for their husband; but in our family, there is no any disparity in between husband and wife; we eat together the whole families at one dish. We eat four times per day; we eat morning as breakfast, lunch, tips and dinner; but we eat almost similar foods during the breakfast, lunch and dinner which are bread. We also eat only lunch and dinner if we are busy in some agricultural activities.

**Section 4: Other interventions that improve the nutrition of lactating women**

**I:** Have ever gone for nutrition screening during community health day? Who provide you?

**P:** I have been screened two times, one at pregnancy and the second during my lactation period. After I was screened during my pregnancy, I took five kilo gram fafa and the plump nut; and the health expert advised me to eat alone; I showed great change in adding my body weight. The same after my delivery, I was screened and get found below the standard; but since there was no fafa in their store, the health expert didn’t provide me fafa; instead she advised me to eat five times per day from the home grown foods such as porridge of red teff, egg, butter and soup so as to improve my body weight and open my breast nipple to my child; as per her advice, I ate porridge and I showed a little change in my body weight.

During the children vaccination, we mothers are also screened and get advice as per our results. Sometimes we could forget the routine service delivery or we may not get the access of some drugs like birth control drugs; we also busy in house activities and by giving less credit to get the routine service, thus the health experts prepare all the necessary drugs at the community health days and we could get benefit at that time.

Some challenges during routine service delivery are lack of some services like fafa, insecticide treated bed nets, birth controlling drug; in community side also there is less follow up to the health facilities.

**I:** Do you think lactating women needs the targeted supplementary foods? Why?

**P:** The importance of targeted supplementary foods to both the pregnant and lactating mothers is unquestionable; I told you that I was benefiting from the fafa that was given during my pregnancy. I increased my body weight and it could get benefit to the infant but I didn’t get during my lactation time.

**I:** Are the lactating women beneficiaries of the soft conditionality of the safety net program? How?

**P:** This program is intending for the poor once; I am not participating in this program since I have livestock like oxen, cow and donkey. In this program, pregnant women are not participating in the work but after nine months of delivery, lactating women are involving in the work. There are programs that help for the elders, poor and emergency aid for the whole community which is provided during the drought season. There is no especial aid favour for pregnant and lactating mothers.

During the water and soil conservation, pregnant women are not participating in the work but the lactating women are participating after baptism of their children.

**Section 5: understanding perceptions of early marriage and birth spacing**

**I:** Do you think delaying the age at first marriage after 18 is better for the health of the women? How and what other benefits does it have for the women and the baby?

**P:** Yes, if there is early marriage below 18, there could be delivery problem, unbalance sexual intercourse and stress to mothers and affect the health of children. Currently, early marriage is almost ban in our community; everybody passes through the health facilities before s/he gets marry. It is allowed only after confirming their age is above 18 years by the health experts. I remember that there was cancelled the marriage program after the pairs were confirmed under age; during the confirmation, all responsible bodies were called to check the age of the pairs like the priest, the families of the two, the development group, the elders and teachers. These groups had great contribution to guess the age of the pairs. There is a punishment like prison for the fathers of the two pairs if there is under age cheating marriage.

**I:** Does this marriage delay would have a benefit to the nutritional status of women?

**P:** Yes, there could be divorce after marriage since there was no affection among the pairs; it was done only by influence of the two families. The other problem is prejudicing the organ of the female being it is not matured, it could also affect the health of the child like delivery of underweight and less treatment after delivery by his/her mothers.

**I:** Do you think the underage marriage is promoted in the community?

**P:** Yes, the promotion of avoiding early marriage is important for the sake of our selves. Unless we owned as a culture by the community, it is difficult to keep always by the government. If the female is above 18 years and the male above 22, then the pairs get married and they could not face the problems like food insecurity, and dependency on their family.

There are also decisions not to give up her schooling and it is an obligation to check the pairs about their health status (HIV ADIS). If the marriage is undertaken under eighteen years, punishment like prison is expected. Therefore, almost underage marriage is stopped in our community.

The presence of birth spacing among the consecutive children is important for the mother as well as the child. The child could not get the appropriate treatment like timely breastfeeding and supply of complementary foods and keeping the hygienic status of the child. The mother could not also get free time to become stress-free.

**I:** What does the reactions of the community looks like to promote it?

**P:** Now, the understanding of the community towards the acceptance of birth spacing is improving. For long space between the child births, we use pill, and some mothers fear of remaining unproductive (sterility) among the mothers. But they see tangible evidences from those who use the birth control that become delivered. Some religious people are also strictly opposing the early marriage of less than eighteen years; now it is totally rejected the early marriage among our communities.

I: How could the ban of early marriage is promoted?

P: Now early marriage is not the problem of our community. We have owned the demerits of early marriage for the child and the mothers. If early marriage is conducted, everybody has the right to inform for responsible bodies; almost the trend of early marriage is stopped in our communities.

**I:** Can you think of any other opportunities to prevent early marriage and increase birth spacing?

**P:** We have the opportunity of accepting the community that early marriage is considered as taboo and the community are devoted enough to prevent early marriage; the schools are also promoting these activities; awareness is already created in every structure of the government; and we have health post at nearby the community.

**I:** How about the space between each birth of children?

P: Awareness about birth space among children is given by the health experts but there is no tight rule and we didn’t handover like early marriage by our communities. Although the training is given to the women, its practical application on the ground is not fully implemented. Few women give birth after one year and most of them are giving birth after three years.

I: How many years do you think the gap should be between successive births for women? Why?

P: In my side, I use the birth control medicine and I got birth after four years. But according to advises of the health experts, the birth spacing between successive births of children are accepted above three years. The advantage of birth spacing among children is already discussed. It could prevent the child from stunting since the breastfeeding time could prolong up to two-three years and the mothers could prepare complementary foods in a proper way; mothers could have enough time to treat the child and for herself; feeding and sanitation is compromised and their economic burden is not reduced.

I: Who is the source of information?

P: The main source of information for birth spacing is the health experts. But the women development groups are also advising to the individual women in their home. Now having getting information is not the problem of ours; but the existence of resistance among some women and less practical application at the ground. Some women miss the program of birth control at the health center and sometimes the drug is finished at the health facility. But to my observation, there is an improvement on the use of birth control every time.

I: What can be done to promote it in a better way?

P: All the women should use the birth control; all the community should aware through their family like their husbands, their children from school; the health extension workers, and only one priest is advising us about the importance of birth spacing.

**I:** Can you think of any other opportunities to prevent birth spacing? How?

**P:** we have health post at nearby the community; training is given about the importance of birth spacing by the health extension workers;

**Section 6: Understanding communication and information sources**

**I:** Is there an opportunity in the community to discuss nutrition for women?

**P:** There is a discussion among pregnant and lactating women every month at the day of 12, who are organized by the women development group and the health extension workers; they prepared and eat bread during their discussion; they discus about their personal and environmental sanitation, feeding time and type. The women development groups disseminate the information through home to home visit for those who didn’t attend.

**I:** What are the common sources for nutrition for lactating mothers?

**P:** We have the problem of water source and we couldn’t produce home garden vegetables; but the common summer crops grow in this community are teff, sorghum, bean, wheat; almost every community in this rural area have livestock like cow, sheep and goats, ox, donkey and chicken.

I: Do all women get the message easily? If not, why?

P: Every women is received the information during the discussion. For example, I am not participating in the discussion but I didn’t miss any information even I am at home.

**Additional comments**

We learned a lot about pregnant and lactating women’s nutritional status and the importance of diversified food and reducing workload for mothers.

**SUMMARY**

**Section one: common maternal lactating women nutrition problems in the community**

- There is a shortage of edible oil at the public market and we require eating bread only; this could contribute to become thin and poor body condition.
- I travel more than one hour of round trip to fetch water and at least two round trips are expected per day. To visit the community market, I also travel more than five hours of double trip. Having this long distance of my journey, it is difficult to have good body condition.
- We usually eat food for maintenance and eating the diversified foods are considered rather as luxury foods than essential component of daily household nutrition.

**Section 2: Barriers to access and utilization of nutrition service**

- Compared to the two communities, more advice and follow up is given to the pregnant than the lactating once. For example I was measured the MUAC during my pregnancy and I had been given five kilo gram of fafa and plump nut. But now during my lactation time no fafa was given after I was measured the MUAC; only they advised me to consume the home grown foods like porridge, egg and soup.
- We use a tippy tap technology to facilitate our hand washing interests; and to avoid any contamination of water, we are provided the guard water to add to our drinking water.
- The insecticide treated bed net is not given priority to pregnant and lactating mothers; rather it is provided with the principle of first come first served. For example, this year I didn’t get the bed net.
- The barriers to eat diversified food are due to the shortage of our economical source; access of different food types at nearby settings; our awareness on the importance of nutritious food is also poor; if we don’t get hungry regardless of the food type, we don’t bother to the variety and quality of the food types.
- After my child has been slept, I and my husband are harvesting all our agricultural crops; thus I am very much busy during the harvesting season; even I don’t have enough time to treat my child properly during this season. I am doing all the agricultural field activities equal to my husband in addition to my house work responsibilities.

**Section 3: Perceived needs of women for relevant services**

- Although we are advised to come to the health centre, we more active during pregnancy rather than at lactating period; we thought that already the stress is over during pregnancy. Had it been the fafa aid during lactation like the pregnancy time, it could be nice to attend.
- Relatively the preparation of extra food for lactating women is better than pregnant ones. However, the provision of quality foods for lactating mothers is only valued for the first two weeks of lactation.
- Until the first seven days of lactation, eating injera is not advised; because it has no any energy that could give strength to mothers. Drinking water has also an effect to exacerbate the bleeding of mothers; instead it is recommended to drink local alcohol (ስዋ) which uses to increase the milk during breasting of the child.

**Section 4: Other interventions that improve the nutrition of lactating women**

- There are programs that help for the elders, poor and emergency aid for the whole community which is provided during the drought season. But there is no especial aid favour for pregnant and lactating mothers.
- During the water and soil conservation, pregnant women are not participating in the work but the lactating women are participating after baptism of their children.

**Section 5: understanding perceptions of early marriage and birth spacing**

- Currently, early marriage is almost ban in our community; everybody passes through the health facilities before s/he gets marry. There are a confirmatory body that ascertains the age of the females like the priest, the families of the two pairs, the development group, the elders and teachers.
- We have the opportunity of accepting the community that early marriage is considered as taboo and the community are devoted enough to prevent early marriage; the schools are also promoting it; awareness is already created in every structure of the government.

**Section 6: Understanding communication and information sources**

- The information source is disseminated by the health extension workers and the women development groups through home to home visit of each mother in our community. Every women is received any information on time.
