# Supplementary material for: Barriers to utilize nutrition interventions among lactating women in rural communities of Tigray, northern Ethiopia: An exploratory study
Source: PLoS One. 2021 Apr 30;16(4):e0250696. doi: 10.1371/journal.pone.0250696 (PMC8087028; doi:10.1371/journal.pone.0250696)
Supplement: S2 File — (ZIP) [file pone.0250696.s002.zip › S2_File.Doc/Lacatating women_IDI & FGD/090-IDI_ Latatating women_Felege Hiwot kebele_Tangua Abergele woreda.docx]

**Day7: 06 /03/2010 E.C**

**Translation: In-depth interview of lactating mother**

**Section A: Interview details**

Zone: south eastern

Woreda: Tankua Abergele

Kebele: Felege Hiwot

Name of participant: Amete G/hiwot

Interviewer: G/medhin.B

Date: 06/03/2010 E.C.

Interview start time: 5:00 Am; local time

Interview end time: 6:0 Am; local time

**Section A: Interviewee professional details**

|  | Socio demographic information | | | |
| --- | --- | --- | --- | --- |
| **Sex** | **Age** | **Marital status** | **Education level** | **occupation** |
| Female | 23 | Married | 8^th^ Grade | Farmer |

**Section1: common maternal nutrition**

I: What are the common nutritional problems in the community for lactating women?

P: The problems seen here are: one, for example myself, during pregnancy, I have to eat extra meal than my family or my parents. If they are eating three times, I do not have extra to eat. This is related with shortage of food in area, which is occurred repeatedly. Because we do not more; the problems occurring among mothers and children is because of shortage of food. Here in this area, the government says “mother has to eat four times”, but we, because we have shortage of food in the area, are exposed. Because, we have little at home, we are facing a lot of problems.

I: What are the problems? E.g. wasting

P: There is wasting. In our kebele, kusht Misaza, because of shortage of food, we heard one woman died as a result of hunger. There are such problems in our community. Because of hunger, we feel dizziness, we fallen down because of it. I saw such things.

I: What about among adolescents whose age is 10-19 years. How do you see them? Is there nutritional problem? They could be short as per their age or with low weight?

P: Is it related to malnutrition?

I: Yes.

P: yes, there are.

I: What about waste ones?

P: There are adolescent who are wasted, like me, they are thin, but tall.

I: what else do you observe?

P: It is being thin and short physically. I do not know their problem. But, when I see things in terms of myself, the problem is cause by under nutrition. Both disease and wasting is caused by malnutrition.

I: Have you ever encounter anemia, night blindness or goiter, in yourself?

P: I was not affected by goiter. I do not exactly know if it is malnutrition, but, when I was pregnant, and immediately after delivery, I had a feeling of dizziness. And, I was given a medicine. I am not sure if I was cured by it, anyways I feel good after few weeks. And, we think as if we are cured. Thus, I can say, anemia is seen in the community.

I: what do you think is the reason?

P: it is because of hunger. E.g. if I have an onion, I do not have oil, I do not have meat. Thus, it is because of the shortage of food.

I: Is there adolescent your house?

P: Yes.

I: So, how do you the above problem with her. You are in one family and may not feed differently.

P: Anyways, she is like the community, she is not thin and she is not fat. Besides, she is a student, and she washes on time and do everything on time; she does not seem, she has a problem. But, she is thin. Because she does not tell me her problem, I do not know more. But she is clean but thin.

I: Do you see any one with hypertension or diabetes or else diet related non- communicable diseases?

P: For example I have a mother, and she has a goiter. When she went to health facility and she was advised to use iodized salt. But, she did not bring any change. The goiter is not too much visible but still now she has goiter.

I: Does she use the iodine?

P: Yes, but it is as usual.

I: what do you think is the cause of goiter?

P: At that time because I was a child, I do not know how get the goiter.

I: what about now, what do you think is the cause?

P: This day, it could be because of anemia or malnutrition. This is what I guess.

I: Are women/adolescents in this community who do not increase their height proportional to their age? They could be lactating women or adolescents?

P: Yes. There is one lady that I know her. She is very short; I do not know what the cause is, But, she give birth like others. When I compare her while walking, she is short and I am tall. Her age is old, but we do not know why she is short.

I: Could it have relation with nutrition?

P: I did not think about it. But people say it is natural. Because, it is said “if one short, he give born short”. So, I can say it could be because of it.

I: What about the proportionality of weight to their age? Take yourself as example, you look tall, but do you think your weight proportional to your age? Your observation could be about other mothers too.

P: Now I am thin. There is one who is fatter than me. And some of us are equal. Our weight defers. The difference is the fat one uses good diet and has good life in her house. And this happens.

I: You have mentioned me that there is shortage of food. In which situation do you think this happened? How frequent does it happen? Do people have food that can serve for a year?

P: Teff is commonly produced here. If get or produce Teff, sorghum, Adengor (bean) and Selit (sesame). Let alone for ourselves, we can prepare variety of diet. But, we have shortage of food, because of drought. This year, we do not have any think, and we are saying “How are we going to live”. Let alone to use variety, even we do not have one food item to eat. We are striving to live. Because we can eat what we want, we will be exposed to malnutrition and disease like anemia.

I: In which situation do you think this happened? Is it in summer or in dry season? When do you think many people suffer from it and need government support?

P: Now, there is shortage. It starts from July and August. Currently, there are many adolescents suffering from hunger. The people are very scared now. The shortage starts in summer and decreases in autumn, and this is only if he has produced some. Had we have we could use it. But actually, now, there is no any production in this time.

I: How frequent does it happen? Is it many times?

P: Yes it happens many times. It is more frequent to be dry than it rains.

I: What other problems do women in this community suffer from?

P: The other problem is lack of medicine for children and mothers. Until the ambulance comes, for a mother, if more fluid outflows during delivery, there will be a problem. To go early, there is no health center here. In this health pots, there is only anti malaria drugs. When we have a problem and do not have money for transportation, we go to a health center in region three. If you go to region three, there are variety of drugs inform of capsule. If you tell them your illness, they will give you what you. For children, there is also syrup of different kind. But, here, if you are sick, there is treatment for only malaria. There is no other service here. This makes children to be late for treatment. Because you lose time until you travel to other health facility. Because of this they are affected and hurt.

I: What about adolescents? Could be access to education, health service or nutrition related problems

P: yes, there is a problem; for example last year at school, there were two female students who were sick at school; other students carried and took them to health facility. I am not sure what the cause was, be it because of hunger or not. I hear this as I have brother, a student, at the school.

**Section2: Barriers to access and utilization of nutrition services**

I: What kind of nutrition interventions are in place to improve health of lactating women and adolescent girls? Or to solve the above problems, be it from Woreda or kebele leaders

P: I do not know what interventions are done. At school, there was support for girls. I do not know the organization that made the support. But, to in school adolescents that are frequently absent from school and interrupt schooling, money was given to them. Then the students bought ten sheep for each. It was assumed that they are interrupting school because of shortage of food at their home.

I: what else, related to nutritional support?

P: Yes, there is plump net for children and fafa (corn) for women as food support.

I: where do they get it?

P: It is here in the health post.

I: Do you get advice for you and your child. E.g. vaccination, on nutrition to use balanced diet, to visit health facility for checkup during pregnancy

P: yes

I: who provide you?

P: Health workers, I suspect pregnancy. I go to health facility and made checkup. They tell me that I am pregnant and they also check my blood. To prevent anemia they advised me to eat extra meal than my family. When I go for checkup at 6^th^ month, and confirm that I have malnutrition, they give me red tablet; I lost its name.

I: is it iron?

P: yes. It is iron. And then, at 8^th^ month, I will also check if I am increasing or decreasing. If I get improved, it is good. And if not, they also give that drug. At ninth month I will go to health post for delivery.

I: After delivery, what type of service is delivered related to nutrition? Advice to use of food diversity or other advice for you and your baby;

P: Yes we get; after delivery up to 40 days, we are advised to use variety of foods. But we do not practice it. Because we do not have much food, we get rest for seven days or maximum of 14 days. Because of the resource in our home, a limited one, we do not apply what the HEWs advised us. We get the advice but we have not anything for implementation.

I: what about for adolescents (out school and in school girls)? Related to visit health and advice on nutritional screening;

P: I do not know if they are getting advice or not. But, I heard that if a girl is greater than 15 years, she is given contraceptives. But I do not know the other information.

I: if mothers are getting counseling for food diversification, could you tell me about it?

P: They tell us to prepare from what we have. If you have Teff, make it soup; and prepare ‘Tsebhi’ (stew) with variety. Honey provides energy. But we do not have in home. “If you have these foods at home, you should eat”, they always counsel us. If you eat what you have, it will give energy to your body.

I: Do lactating women get advice to use iodized salt? Why?

P: yes.

I: Do you use iodized salt? Can you tell me how to apply the salt?

P: Yes I do. Once we combine the oil, onion and others, anyways what I have at home, I will add the salt when it is time to get it down. Once I add the salt, I immediately step it down from the fire.

I: what do you think is the advantage of iodine?

P: “Waeh’’ they told us that iodine prevents from goiter.

I: Do you think there is change in goiter among adolescent, once the community starts to use iodized salt? Is there a decrement in goiter in the community?

P: There is decrement. It is only seen among older women. The youngsters are free of goiter. But among elders, e.g. in our locality, I know two, three mothers with goiter. There is no goiter among the young ones.

I: Do you believe on the efficacy of iodized salt? Did you advice to your brothers?

P: yes.

I: Do you get advice on home gardening? It can be during summer or via bringing fetching water?

P: is it by agriculture?

I: it could be through the agriculture extension or health professional or both. It is to plant vegetables like tomato, onion, salad cabbage and others to benefit the mother.

P: Yes there information given by the agriculture extension and they provide us vegetables. Even though the government is supporting us, there is a shortage of water here, and the vegetables will not survive, Because of this we do not plant vegetables.

I: what about related to safety net? Are lactating women involved? What about you.

P: Yes I am involved and there are also mothers who benefit from it.

I: what is given there?

P: Previously, 20008 EC. We were given a bean. Last year, in 2009 EC, We were given cereal, but we were not given bean.

I: Do you work? Or are you waived for work because you are lactating mother in the safety net program?

P: now, what they told us is, your baby is older than ten months, the mother will work. If baby is less than ten months, the mother will not work.

I: Any more on safety net? What about for adolescents, both out and in school girls? Do they get support?

P: Is it for head of household?

I: No, just for the adolescents?

P: They are not given any support.

I: what else like the emergency support?

P: yes, there are people we get emergency support. Even now they are took the support. But, safety net is not started yet.

I: are lactating mothers involved in emergency support?

P: no they are not.

I: who is getting the emergency support?

P: Those people who some food at home and are identified to take emergency support.

I: what about for the elder people who are unable to work and even lactating mother, orphans?

P: these are included in safety net.

I: is there anything you can tell me more the support?

P: There is no.

I: what about advice on water, sanitation and hygiene services? Or how to prevent from disease; did you get such advices?

P: Yes, and this is given by the health care providers; there is no other body who can deliver such information to us.

I: How about on latrine construction and utilization?

P: I have latrine, and we utilize it. When we wake up from sleep, we go to toilet and defecate. After we get out the toilet, there is water and we wash. When we get back to home we wash using soap and water.

I: what about water for drinking? It is accessible in short distance?

P: The water source is far from our home

I: how long does it take?

P: It takes four hour to go to source and get back to home.

I: Do not you have water source here?

P: Yes, we do not have it?

I: what do you think is the reason? Is because there is no potential area or responsible bodies are not working for it?

P: Here, it is difficult to dig out by human energy; may be using machinery from the government. The groundwater is too far. It said that the nature of the ground water is too far and it can’t be dig out using human energy. Here the land is not ‘Netae’ (There is no surface water) that can be easily traced.

I: So, how important is this to you? Is there any complaint from mothers or else?

P: Yes we request for water. For example in 2007 or 2008E.C, water was supplied on tanker to the community. There was thirsty of water. But now, you bring yourself either on donkey or on your back, and it takes four hours.

I: Are there adolescent who are absent or interrupted from school because of the above problem?

P: Yes, there adolescent girls who withdraw from school either because to fetch water or because of shortage of food at their home.

I: How about their (for adolescents) nutritional status and health?

P: “Way ehm” anyways they are medium.

I: Is malaria common in his community?

P: Yes, there is malaria. There are many people who are affected by it.

I: When do you think it is common?

P: in September.

I: Do you have ITN? And do you properly use it?

P: Yes I have, and I use it. For example, I have four family members and I was given two ITN. Then, one is for me and one is for my children.

I: who do you think is at risk of malaria? Mothers, children, men or adolescents; who is commonly affected in your community?

P: Malaria mainly affects children.

I: So do you give primary focus for your baby?

P: Yes, to prevent her from malaria, after dinner, she sleeps under the ITN.

I: Who inform how to use ITN?

P: health care providers

I: Are lactating women getting deworming service? To prevent from parasites, have you take a drug for it.

P: No I do not. I swallow a drug used to prevent eye disease.

I: What about for adolescents? Do they deworm at school?

P: yes, I heard students saying we swallowed a drug, but I do not know what the drug is.

I: Who provide them?

P: I do not know. My son, a grade three student, told me that “they gave drug, and I feel head ache and dizziness”. That is what he told me.

I: is it the drug given for eye?

P: I do not know. But, that is what told me; the drug was heavy and I felt dizziness. The students were given twice. At that time, I was pregnant, and I was advised not to take a tablet, rather I was given an eye ointment.

I: Is there anyone who is affected by parasites? May be some, mothers, with diarrhea; because of use of dirty or contaminated water? E.g. acute watery diarrhea

P: Yes, there is diarrhea. But, I am not sure if it is waterborne or not. Acute watery diarrheal at this time was not seen in our Kushet. When people are temporarily affected with diarrhea, they are given ORS.

I: what about lactating mothers, and their children?

P: yes mother get diseased by diarrhea. The same is true for children?

I: How is the necessity of deworming to mothers? It is acceptable by the community?

P: There is no anyone that does not accept if you convince him. We he see it, he will understand it benefit. The first thing is that he did not get the service, but if they are going give it, we will accept it.

I: Do you or lactating mothers screened for nutrition; MUCA measurement. And get support, like plump net fafa (corn) and oil.

P: Yes, I took.

I; are you getting the supplementary feeding now?

P: no I am not, but may baby girl is taking. In last month, she took plump net.

I: What was the reason why you baby is taking plump net?

P: HEWs explained to me “she has decreased, and she needs complementary feeing.

I: Does she bring change?

P: Now, she is good than before.

I: Do the above nutritional intervention (deworming or supplementary feeding like fafa to adolescents (10 to 19 years old girls?

P: No, they do not give them.

I: which of the interventions above do you think is important for women?

P: How?

I: e.g. it could be the provision of fafa to women and children, involvement in safety net, access to safe water, ITN provision; do these things have benefit to mothers.

P: To call it important, all are not complete in the service delivery, but to strengthen, it is good if shortage of water is solved. If so we can wash our body, our utensils, drink clean water. Yes it could be better if we have enough food. Example, what people from urban and rural differ are these things. The life we have. In town, they can be scholar, or government workers or having good resource at home; this is why they look good. Had we been like them, it would have been very nice.

I: What are the main challenges here that prevent the implementation of these nutritional services? You can say: had these things been solved, there could be change.

P: the barriers are, one, we do not have water in our locality. Besides, we wish to have town here, because, we think, if we are living in town we will be changed. And the younger population will changed by having job. The primary problem here is shortage of water. The people are selling their animals for survival. The people are suffering because, there is shortage of rain. Then the people are in debit with government loans. Individual sell their animal to eat; when he wants to buy them, they are expensive even though they are cheap now. It is in this time they get cheap. These all are challenges.

I: what else? Sometimes people have sufficient food, but they can be in malnutrition, because of low awareness, or lack of advice or else; Use only one type of food; mothers( lactating and pregnant)do not eat extra meal despite availability; do not use ITN and affected by malaria; do not use latrine and frequently affected by disease; are there such conditions?

P: yes, there are. Let alone other, there is a women we give birth two children within a year. This means, the woman gets pregnant while the first baby is three months old; I have a sister that gives birth with in year, then the infant died. She is in pain while the baby is sucking the breast. This is because of low awareness. Now, she is in pain and the baby is crying to sucking. She is hurt and the baby is hurt. So this is because of low awareness. There people who do not understand, and there people who easily understand. There are also people who have money but they do not use it properly; neither have they eaten and look good nor they save it and utilize it properly.

I: How are these problems happening? E.g. Your sister, she gave birth two in a year; is because she is not advised or is it deliberately even after counseling?

P: It was unknowingly. We are advised to use contraceptive after 40 days of delivery. I give born my second child after five years, and this one was born after four year. Birth spacing is important for the mother and the child. In case of my sister, it was sudden. There are half that did not understand it even after you talked to them. They say “The child will correct everything”. Thus, there is lack of awareness.

**Section 3: Perceived needs of women for relevant services during lactation**

I: What special things do lactating women need in your community?

E.g. To visit health facilities, you told that it is far from this and this health post only has antimalarial drugs. So, how long does it take to you to reach at the health center? Is it in Yechila?

P: Yes, it is far from this.

I: So, what things should be fulfilled here in this health facility (health post?

P: All delivery services should be available here. All necessary drugs should be available here for mothers. If I got early treatment here, people will take lesson and say one woman is treated here and she is okay now. The second is, if a child has cough, it will be good if he is early treated in a near place than travelling to Yechila (it is a town in Aberegele Woreda), and he will be cured. For children, one, there a capsule to be swallowed; there is syrup, and everything what you like, what the clinician gives you; all these are available in other health centers. But here, if you are sick, they will examine you, and give you drug by explaining that you have malaria. There is no other drug. If all drugs are brought, it will be good. Here, only anti malaria is available.

I: How about in giving advice to take extra meal? Is there something missed? Sometimes people do not eat extra meal though they have enough food. Is this because they are advised or is it resistance? And what should be done/ what is needed?

P: Is it something that can be done?

I: yes

P: “WAY ehi”. For this, there is extra meal that is given by the government, but only five cartons of fafa (corn) are given that are small. But, as to me, I do not think they can bring change. They said they can bring change because, they have measured it. But, I do not think they can bring change. The fafa (corn) give is small. There is also for children, a plump net. These are 15 for 30 days, sorry, those are 30 plump nets for 30 days. For example, this baby is given one plump net per day, and it will be early finished. They told us to add other complementary food form ours. But now, we do not ours; had I have food, I would feed her properly. It is only she (the baby) that started supplementary feeding, but none of my others children did start feeding it. If I am going give her by preference, from that and this food item, food should be available at home. But now, “How is she going to fulfill by eating one plump net?” emmm, I do not know.

I: Regarding rest, do women get enough rest during pregnancy or lactation? Do you take rest? E.g. during safety net; is the 10 months waiver enough?

P: yes; if there is someone who can carry the baby, he may stay despite he cries. But, if not, the students will be absent from school and care the baby. The work place is too far and the weather is sunny. Thus, it will be good if extra 4 of 6 months are added; at least the child with start to eat injera by himself.

I: what should be the role of husband to support maternal nutrition?

P: No thing, because they are not helping us.

I: what do you expect from your husband?

P: What we found in house, we eat together. They say “let us eat and go for work”. They do not advise us, because they do not it in detail.

I: In some areas, women do not eat poultry even when they hungry if her husband is not around. Do you accept this?

P: yes I do.

I: Is this right? What if you are pregnant, you are lactating and feel hungry?

P: I believe in it, because woman should not eat dish of poultry alone. If I get, I will eat the dried injera or bread, But, I will not eat the poultry.

I: What is the problem if you and your children eat half of it? How are you going to produce milk for this baby in this way?

P: “Whah!|” I will eat the other food, and after he come, we will eat together, else we will eat alone. We can eat the other food items like onion and tomato.

I: what do you expect from your husband? Sometimes men eat what they want in town, but they do not help their wives; how do you see this or what do you expect from your husband?

P: what are you going to do for them?

I: Health extension worker may give education separately for them. So, what do you expect? You can express what you feel.

P: Anyways, may be his wife, because they can understand each other. But, I do not think people can convince him.

I: Do women in this community typically change their diet when they are pregnant? Now you are lactating, but did you change diet while you are pregnant? Adding extra meal, and variety; even do you have awareness in this? Or do you get advice on this?

P: Yes, I had been advised. If she is pregnant or lactating she has to eat variety of foods. If she eats something in morning, she do not have repeat it at night, rather eat different type of foods. We are advised to eat what we have and eat one extra meal than our family. This is what we have taught.

I: If so, what is the challenge to practice it?

P: The problem is we do not have resource at home. But, in terms of education, it is clear.

I: Are there gender disparity in women’s diet before and after pregnancy. Is there a diet which woman did not eat during pregnancy?

P: No, there is not.

I: what about in adolescent girls?

P: No, there is not.

**Section4: Other interventions that improve pregnant, lactating and adolescent nutrition**

I: Have you ever gone for nutrition screening? Where? Who provide you?

P: Up to six months from delivery, I was being measured MUAC. After six months, the baby will be measured.

I: what about for you?

P: The do not measure on me.

I: What if you have malnutrition?

P: No! No! Never! Whether I am wasted or not, they will not screen me after six months.

I: Okay, until six months, who made the screening?

P: health care worker

I: how frequent is it? Is it monthly? Is it inform of campaign or schedule one?

P: There is one day appointment. It is done monthly.

I: Is this at individual level or to entire women?

P: At kebele level, people mobilize and tell the fixed date to pregnant women, lactating ones and those who have children. Then all of will come at the same day?
I: How frequent is it in a month?

P: It is done once in a monthly.
I: How about to adolescent? Will they be included?

P: No, they will not.

I: Do you think the community health day have benefited mothers?

P: For mothers, I do not think that it has benefit and bring change. It is tasty while eating it, but I personally did not bring change.

I: what about for other? Are any other women that describe the benefit because they have improved?

P: For children, yes it has benefit.

I: what about the nutritional advices you got there so that you can apply it at home?

P: The advice is good. But, what it brought by the government (fafa) did not bring change to. And I do not know if the change if because of the fafa or the food that I ate at home.

I: what about during the routine service delivery? May be when you come for child vaccination or other service, Is there nutritional screening? MUAC measurement;

P: Yes they examine us until six months after delivery. After six months, they will not screen us.

I: what are the challenges to attend community health days? E.g. distance; what about in accessing routine health service?

P: Now, for example, I came for child vaccination at 8:00 o’clock. Then said “we will not vaccinate your child because you are late” and then I went back home. We were three and all of us went back. Because it rural area, and the HEWs also fixed specific hour, they left us without service. We came back in the next month and let vaccinated our children.

I: is it far? How long does it take to you to get the health post?

P: Just one hour.

I: Are women benefiting from the targeted supplementary feeding (the fafa (corn) and plump net given)? Even though you have mentioned to me that it does not bring change for you; how is it for other?

P: It is good; especially for children, the plump net is good. Even it would be good if it is added.

I: Regarding safety net, you earlier mentioned that a mother gets support with waiver starting from 6^th^ month of pregnancy up to 10 months lactation. What are you supposed to do during this time? Or any advice you get;

P: is it after we are waived from work?

I: Yes it is. So any advice you get at that time?

P: Once our pregnancy is confirmed, they advise us to have checkup at health facility to prevent hypotension (anemia).

I: What about for lactating ones?

P: for lactating, I don’t remember.

I: During the ten months of waiver, what precondition is set for lactating mother like the conditions put pregnant woman to attend health facility for follow up?

P: They tell us to properly feed children.

I: How do you see the implantation of the preconditions (soft conditionality)?

P: It is good.

I: Are there women who do not visit health facility while they are waived from work in safety net program.

P: In previous years, there were some women, but now it is fine and every mother is getting follow up.

I: Do women in the community know they are targets of the program.

P: Yes. They thought that is for our self for our benefit.

**Section5: Understanding perceptions of age at first birth and birth spacing?**

I: You may have heard that delaying the age at first marriage to after 18 is better. How do you see this in terms of the health and nutrition of the mother?

P: If the girl is 18 years and above, one, she will be in love with her husband. For her, because she is married by her interest, nutritionally as well as the relation with her husband and his families will be fine. But if she is under 18 years, she will face difficulties and she also my hate her husband and led to another dispute, because she has no interest. Thus, underage marriage is bad.

I: What about that delaying the age at first birth to after 18? Is it better for the health of both the mother and the baby? How?

P: It is heard that a girl that marries under 18 years faces difficulty during delivery. But I do not know. But if she above 18 years, as her body is strong, it is good.

I: How about to the child health? Is there difference in birth below 18 years and after 18 years; which will be better to the child?

P: Is it to guess?

I: No, do not know anyone in your community with this scenario?

P: No, I do not know.

I: What about in yourself? You told me that you give born at age of 15; how get the situation in your baby, at first birth, during delivery? How do you describe it? Was there a difference with those who gave birth at after 18 years?

P: I do not know the difference between me and others. But, based on the education they give us, there is difference between births at below 18 and the after 18 years.

I: Were you fine when you give first birth at underage?

P: yes.

I: How old were you?

P: fifteen years old.

I: Do you think this message is being promoted in the community? That is, to increase birth space and prevent early marriage;

P: yes

I: who are working on it?

P: Health care worker and health cadres educate to the community.

I: how do they promote it? Where? Is it home to home or in mass?

P: They educate us at church, and in meeting

I: how does the reaction of the community looks like to the promotion?

P: Now days, because many times the community has understanding about it, they are stopping marriage.

I: For example, if someone is married at age below 18 years, what will happen to him? Is there any punishment or any legal action taken?

P: yes there is, now for example, if the girl is 15, 16 or less than 18 years, and is married, he will be punished 300 birr

I: After all these information (health education is given and law is set for it), are there adolescents who marry in underage?

P: No there are not.

I: what about at school? May be if a girl has boyfriend, is a situation when she gets pregnant and gives birth?

P: Not any more.

I: even any information you heard; do not you?

P: No

I: In your opinion, how this message could be better promoted? Or is it enough?

P: It is enough; people have understood it and they are learning from each other. So, it is enough.

I: During the promotion, are there individuals who do not accept it and you feel that we need to use other opportunities to address them? It could be involving religious leader or local community judges or kebele and kushet leaders

P: At church, I do not know; I usually go on Sunday and get back to home. May be if the priests have to be educated or else the other community knows it.

I: what about for student? Do they get information from school teacher or other body?

P: When there is parent’s day event, the educate us.

I: what about for the students? Do teacher educate about disadvantage of early marriage at school? May be if your sister tells you;

P: Yes, she tells us.

I: Is she married now?

P: No, she is at college now.

I: Regarding birth spacing, how do see it, does it have any advantage?

P: Yes, it is good. One, to me, the first child will grow before the new one comes. Thus, increasing birth space is good to the mother and child. One, my body will be strong; second, the baby be will strong. If you have two children at a time, I will not be comfortable and the children will not also be in comfort.

I: How many years do you think the gap should be between successive births for a woman? How many years do you stay before giving birth to this child?

P: This baby girl is born after four years.

I: How many years do you think the gap should be between successive births?

P: For example, I gave birth after five and four year’s interval to my second and third child, respectively. I would say four years or more gaps between successive births are good.

I: what is its advantage?

P: It is good to me and the child

I: How is it important to you and the baby? Can explain it more?

P: For the baby, if it is only she, I will care her very much. And for me, if I give birth before I replace the blood I lost; it is not good for me. And I can be easily affected.

I: Who is providing such information?

P: The health care providers

I: when was the last time you have heard about it?

P: Every time during delivery; they (HEWs) also teach me while I am pregnant. And after delivery, they told me to use contraceptive after 40 days. And I say okay, and I have started.

I: Are you currently using any contraceptive?

P: Yes I use.

I: Do other women, like you, use contraceptives?

P: Yes, there are

I: How about adolescents? Because they are young (fire age), they might boyfriend, and to avoid unwanted pregnancy, do they use contraceptives?

P: Yes.

I: What do you suggest to promote it in better way? Do all communities hear about this message?

P: yes, it is.

I: where do they get the information?

P: It is given at kebele and church

I: How frequent is it? Who is educating the community?

P: Health cadres and kebele leader.

I: is there any population in the community who is not addressed with the promotion.

P: There is no anyone who did not heard the promotion, but there may be a problem in understanding.

I: Can you think of any other opportunities to prevent early marriage? Sometimes, parents may induce girls to marry when she looks big and tall, or if a Deacon wants to marry a girl who is virgin or other reason. Thus, to prevent such incidents, what can be done?

P: Now, everyone understood it. Many people know it and it is not difficult.

**Section 6: Understanding communication and information sources**

I: Is there any opportunity in the community to discuss nutrition for women? On proper nutrition, is there any discussion?

P: yes there.

I: Who is involved? And what do they say?

P: The health care providers tell us to use different variety of foods, to eat extra food, and others

I: Memo: the participant is too tired.

I: Okay, Who are the sources of information? You have told me: the health cadre in your kebele and HEWs. Who else serves as a source for information?

P: Development armies,

I: What messages are commonly delivered?

P: The educate us to build a toilet; to deliver at health facility if there are pregnant women; to get screened and checkup; vaccinate your child; utilize ITN. These are all what they teach us.

I: Are there people who do not access information, because their house is at remote area or missed during mobilization and promotion?

P: No, everyone accesses information through development army.

I: Which source of information from the above is effective for you?

P: The nearest source that is development army.

I: Why?

P: Because the development army is in every GOT, they can convince at morning and evening to the people.

I: How frequent do women development army meet you?

P: two times per month.

I: What do they tell you when you meet with them?

P: The teach us about delivery; for the pregnant one, they tell her for early checkup; for the lactating one , they tell her to have follow up care for herself and the child. Besides, they also meet us to inform about latrine utilization.

I: any additional suggestion or comment that you want added? I am asking for more ideas, because we want to know the existing problems here in the community. So it is good if you mention me anything that has to be solved by the responsible body. Tell me this this and that.

P: I, as to my observation, one, we have shortage of water; thus we need the responsible body to build any water source. The second is, because this time it drought. There are a lot of adolescents who are hungry staring from August, and the local community gave cups of cereals to them for survival. Thus, the government should give enough food support in recent. But, the worst problem is the shortage of water. Even though we can’t dine water, it is very important. If I am hungry I cannot bring water. But if I ate and get full I can carry and bring water from anywhere. Thus, the main challenges are shortage of food and water. The water source is too far.

I: Does it frequently interrupted?

P: which one?

I: Rain water, the natural.

P: When it rains, it rains heavily. And, because we have vast farm land, we can collect many cereals. But, much of the time, it is dry over many seasons.

I: anything else you want to add on the nutritional interventions and related to health services

P: other! The advice and education given by health care provider is good. But when you go for service, there is no medicine (drug) in this health post, and thus the medication should be brought. Other: I do not have.

I: thank you very much for your information and time.

Good day!

**Summary**

**Section1: common maternal nutrition**

Wasting is common problem and there shortage of food, one woman died as a result of hunger.

There are many wasted adolescents; they are thin, but tall.

The Lactating woman do not know the cause of goiter

Stunting is natural. Because, it is said “if one short, he give born short”.

Lactating woman do not use variety food, because even we do not have one food item to eat.

There is lack of medicine for children and mothers in the health post.

**Section2: Barriers to access and utilization of nutrition services**

For in school adolescents that are frequently absent from school and interrupt schooling, money was given to them. Then the students bought ten sheep for each.

If a girl is greater than 15 years, she is given contraceptives

Youngsters are free of goiter.

Even though the government is supporting women, there is a shortage of water here, and the vegetables will not survive in the area.

There adolescent girls who withdraw from school either because to fetch water or because of shortage of food at their home.

In September, malaria is common in his community. Malaria mainly affects children

There is no deworming service lactating women

There is no nutritional intervention (to adolescents (10 to 19 years old girls

**Section 3: Perceived needs of women for relevant services during lactation**

Woman does not eat poultry even when they are hungry if her husband is not around.

Women are advised to change their diet when they are pregnant. The problem is they do not have resource at home

**Section4: Other interventions that improve pregnant, lactating and adolescent nutrition**

Lactating mothers are not measured their MUAC after six months of delivery; only the baby is measured.

For mothers, I (lactating woman) do not think fafa (corn) has benefit and bring change.

**Section5: Understanding perceptions of age at first birth and birth spacing?**

Marriage after 18 year, increases loves between couples; this is because the girl is married by her interest, nutritionally as well as the relation with her husband and his families will be fine.

After delivery, they (HEWs) inform mothers to use contraceptive after 40 days of delivery

**Section 6: Understanding communication and information sources**

Health cadre in kebele, development armies, and HEWs are main source for information, of which development army (nearest source) is effective one for mothers.

**Additional remark:**

There are a lot of adolescents who are hungry staring from August, and the local community gave cups of cereals to them for survival

The worst problem in the area is the shortage of water.

Over all, the main challenges are shortage of food and water. The water source is too far.
