# Supplementary material for: Barriers to utilize nutrition interventions among lactating women in rural communities of Tigray, northern Ethiopia: An exploratory study
Source: PLoS One. 2021 Apr 30;16(4):e0250696. doi: 10.1371/journal.pone.0250696 (PMC8087028; doi:10.1371/journal.pone.0250696)
Supplement: S2 File — (ZIP) [file pone.0250696.s002.zip › S2_File.Doc/Lacatating women_IDI & FGD/093_FGD with Lactating women_Felege Hiwot kebele_Tankua Abergele woreda.docx]

**Operational Research on Adolescent and Maternal Nutrition in Northern Ethiopia**

**Introduction**

Hello my name is kiros, I am from Mekelle Universty; we are conducting a research on the factors that influences the nutrition of mothers and adolescent girls in collaboration with the regional health bureau and UNICEF. Year participation is very valuable; the information that you tell us will be used to improve nutrition programs and services for women and adolescents in the region and the country. We will not share your names when we report our results. The interview may take 1-2 hours and I would like to thank you for taking the time to speak with us today. You have the right to withdraw at any time and I will use tape recorder. Are you voluntary to participate for the interview?

**Yes** No

**Section A: Interview details**

1. Zone: **South East of Tigray**
2. Woreda: **Tanqa-abergele**
3. Kebele: **Felege Hiwot**
4. Interviewer name: **Kiros Tedla**
5. Date of interview: **15/11/2017**
6. Interview start time: **9:00AM**
7. Interview end time: **11:o6AM**

**Section B: socio-demographic Information**

| Name of FGD participant | age | Marital status | Educational status | Occupation |
| --- | --- | --- | --- | --- |
| Amete Menegn | 20 | Married | Illiterate | House wife |
| Tures Gebru | 20 | Married | Illiterate | House wife |
| Brhane Guesh | 28 | Married | Illiterate | House wife |
| Amete G/hiwot | 23 | Married | Grade 4 | House wife |
| Yeshi Woldie | 20 | Married | Grade 3 | House wife |
| Kiros belay | 27 | Married | Grade 5 | House wife |
| Enzosh Gebru | 25 | Married | Grade 5 | House wife |
| Meskelu Atekalign | 28 | Married | Illiterate | House wife |
| Hunayesh Alemayoh | 30 | Married | Illiterate | House wife |
| Abriha G/medhin | 19 | Married | Grade 8 | House wife |
| Enzosh Wedih | 30 | Married | Illiterate | House wife |
| Mulu Ayele | 19 | Married | Grade 10 | House wife |

**I:** Interviewer **P:** Participant

**Section I**

**I**, **what do women do to stay healthy in this community or worerda?**

**P**7: to be healthy we drink filtered water, take treatment and eat diversified food.

P4: I have to start from keeping hygiene of my house by cleaning the house; I have to separate the human living and animal living houses including for hen. I have to have a place to put the materials used to eat and keep clean.

P2: we have to eat diversified food; we have to separate the living house of animals from human living. We have to keep clean all the materials used to eat and drink. We have to also keep our children keen and the materials used to feed them.

**I. Why are you keeping your hygiene?**

**P9:** because it protects our children from diseases as if we keep our children clean and feed them at the right time and send them to school they will not be affected by infection or diseases. Thanks to the government we are good with the exception of that we are only getting anti-malarial drugs but not other medications like shirop so we need the government to bring as clinic to this area as we are far from the health center and the child will be very much affected until we arrive there. But the other think is similar to what was mentioned by the others like we have separated our living house from the animal. But as you come from the region please suggest the need of the clinic strongly.

**I, what are the common nutrition problems in the community for pregnant, lactating and adolescent girls?**

**P9.** for example mothers give birth and the baby become very thin; hence they will bring to the fafa program and he will be given “Mitmita” or Plumpnet and respond very well. It is very nice even though I did not practice it; it good.

**I. What was given? For whom?**

**P9:** I think it is given fafa and plumpnet. And it is given for very thin children, lactating and pregnant women; but I am telling you because I heard from the experienced women but I do not have the experience. But if the mother is becoming very thin they are given fafa and shown improvement or changes.

**I. Why mothers are affected by malnutrition?**

**P4:** this is related with poor feeding habit or shortage food. First the mother should start follow up and if she had anemia she will be given red circle drug (iron). The first visit is to check pregnancy and if pregnant she will then go at the six month for second round and if she has anemia she will be given the drug and then she will also visit at the 8^th^ month and get the medication again. So if she had followed her pregnancy at the health facility she will not be affected by malnutrition. If she had seen the birth indicator she will go to the health facility and give birth in the health facility and the baby will feed the first milk as it makes the baby healthy and protects from malnutrition. And after six months if she feed him soft porridge made up of eggs and oil he will grow very well but if the baby is not feeding very well like in our area as there is no resource; he will suffer from malnutrition.

**I, How do you express the extent of malnutrition among lactating mothers in this community? Why?**

**P9:** we have not seen them but I think there is more on children and almost half of the pregnant and lactating mothers are thin as they are given Fafa. I have not seen them taking I am telling you by guess. (She was amazed by the question as she replied “TEWSAKEY” to mean I do not care; not my duty). This should be replied by those who are utilizing the service.

A big silence as they were talking said ways

P9: there are mothers who are utilizing the service but they will not talk as they are ashamed.

P9: they are very thin because they are not eating very well because they have no resource to eat diversified food. For example: if they are interested to eat sorghum or teff or other three or four foods like meat; they could not eat as they have no these foods or they have no many to buy; mainly in this area as it is lowland we cannot get all what we need to eat that is why the mothers are affected by malnutrition. But if they can afford these materials or foods they would not be exposed to malnutrition.

P3: the main reason why lactating mothers are affected by malnutrition is due to lack of resource or being poor and lack of awareness like preparation of diversified foods like for example there are individuals including me who do not know how to prepare a mixture of sorghum with other types of crops. There is also lack of resource; she laughed…. and said it is enough for me.

P3: related with lack of knowledge it is sometimes but most of the time it is related with lack of resources like if you have got onion you will not have oil; if you have teff you will not have sorghum to mix with the teff. The main problem is related with lack of the resources and that is the reason why mothers are becoming thin or affected by malnutrition. I have finished!!!

**I, What about micronutrient deficiency like anemia, night blindness and goiter?**

**(**Silence followed by laugh when I say please participate do not afraid as I am your brother)

P8: yes there are anemic mothers as they have head ach mainly during pregnancy and are given treatment for that.

P2: yes she right; pregnant mothers are suffering from head ach mainly when they stand up because of anemia. Hence; they went to the health service and take the medication for the anemia. They are given two types of drugs red and white; the red is for anemia and the white is for anti-pain. They take the medication after they have taken soup and different foods.

**I. As you are lactating mothers how common is anemia? Why?**

P2: It is also common among lactating mothers related to excessive bleeding and she will suffer from sever head ach problem even unable to stand as she is affected her back bone; it is also related with poor feeding as our area is lowland and village in which she could not get everything to eat due to lack of resources and then suffer from malnutrition. And after birth the mother is given similar drug she had taken during pregnancy period and they are measured their blood and taken injection and come back.

**I, What about night blindness on lactating mothers?**

**P11:** we are fine with night blindness but we are suffering from anemia and get treatment form the health post.

**P10:** yes, mothers are affected by anemia and having head ach as they do not eat very well. Hence they went to the health post and get advice on how to feed and to drink soup.

**I, What about goiter on lactating mothers?**

**P1:** no answer

P11: there is no goiter now as the people are utilizing iodine salt but common in the past.

P6: it is not present now but was common previously because we are drinking water after boiling and use iodine salt provided by the HEWs.

**I. What is the cause anemia among lactating mothers?**

**P8:** this is because we are living in a very hot please or due to high temperature as it causes head ach when we ago to bring water as it is far away from our home.

P9: as she said it is because of high temperature as we have no water closely we travel six hours to bring water; the mother will stand up early in the morning to bring water without eating any food and then come back after five or six and become starved and even start to lactate her child without feeding where the baby will not get the milk but lactates her until he/she gets milk but even some times the child may take blood as she has no milk. And after this she will start to prepare the food and until she prepare the time will become nine or ten; hence the mother will suffer from head ach and finally fall down or coma and then go to the health facility and given drugs for medication. But mainly this area is highly affected as is lowland and had shortage of water but our mothers were living better than us; as they told us that there were plenty of butter and other resources. But now we are suffering from multiple problems; as the government is not doing consistently; they will start something but they do not finish it; they left it away. So the lactating mothers are suffering from the disease because of lack of resources and water.

P2: yes it true as she said that it is because of the temperature and lack of water as we travel long distance to get water. Thanks to the government; we do have dam around us to use to drink our animals. But we travel long distance for ourselves and the mother standup early at 4:00 or 5:00 PM to bring water; hence she will come back after five or six o’clock and after coming back she will first think about her child and starts lactating her child and then give food to other child or family member then the breakfast time will pass here and then she will eat her breakfast at lunch time. So problem in our area Felegehiwot is very complex due to shortage of water; that is why mothers are becoming anemic and affected by malnutrition

**From where do you bring the water?**

All the participants responded to the question at the same time and said “ Way to express their amazement of the distance and water type and said from river”

P2: we bring the water from “Hariqa” name of the river; there is running water at the summer but now we bring by dinging on the river and filter the water at home.

**I. Is there any non-communicable diseases such as diabetes?**

P6: there is no any problem.

P1: there are no any diseases.

**I, do you think the women or adolescent or children have height proportional to their age? Why?**

**P2:** No, they are not proportional as an example when we see here among ourselves we do not have the same proportion of height to our age. There are people who are short but their age is higher.

**I, Is it related with nutrition?**

**P2: no,** it is not related with food as there are short individuals with very good body condition or fat. For example I am short but my body is normal but there might be another female with less than me in age but longer than me but thinner. This is because GOD created us like this.

There were words like way to express their opposition to me when I said any opposite idea to this suggestion followed by laughing.

P9: start by laughing….. and the with the word “way” ; how can we give opposition as it is because GOD created as short and long again extended laugh by all the participants. Being short and long is because GOD make them like that; for example I will feed my two children similarly but the one may become short and the other long so I will say they are different in their height because GOD made it like that but nutritional difference. But there could be nutritional difference if there is dose difference like the one with longer height might take higher amount of food but the other or shorter might take small amount of food and could have another disease. The other problem mainly in our village is related with insulting the shorter or thinner by saying you eat but you do not get improve in your weight or body weight; you see your younger brother is above you.

P5: no answer

P7: Manayesh had already mentioned it; GOD could create as one with short and the other with fat or normal body weight. It could also be related with disease.

**Disease how?**

P7: because it affects internally and increases its temperature.

P3: it is natural to be long and short but there is difference in taking the amount of food consumed among thin and fat; we give them the food for both to eat together and the one with high weight might take the higher amount and the thinner will be left with small amount of food to eat; but we did not give additional food hence the thinner will become very thin as result of this. So being short and long is natural or from GOD but being fat or thinner is because of diseases.

**I, do you think the women, adolescent or children have weight proportional to their age? Why?**

**P.** no it is not proportional. As for example one year old child might have higher weight than a two years old even though the former had lower age than the latter child this is because the latter or with lower weight compared to his age might have different diseases.

**I, how do you recommend the mothers to eat to stay healthy with normal weight to their age?**

**P7.** if they eat diversified foods for example if I eat egg in the morning; eat meat at lunch time and eat vegetable with coffee at night; I will improve my weight or nutritional status. So difference is in getting the balanced diet.

P9: the children might have different kg which could be related to their feeding habit. If for example the mother with a child more than six months feeds by making soup to drink but the mother of the thin might not provide. As an example we all here lactating mothers have no similar type of caring to our children as I may care or feed my child very well but she might not feed her child so their kg will become different as main will measure better than her child.

**I, what type of food should children or mother feed to have normal weight per age? Is it feeding similar food or diversified food?**

**P9:** it should be different food at different time like feeding different foods at the morning, at the lunch time and at the evening or at night. And washing his body and sleep him; and will have normal nutritional status or weight.

P10: they have no proportional weight to their age. This is because they do have shortage of food. They do not take extra-meal and luck of hygiene as they are exposed to different diseases.

**I, Is there any mother or child with overweight?**

**P9:** I have not measured them but she laughed and said how could this children become overweight

………………there was also a supporting sound and laugh by others to show their support…

P3: laughed and said how could this children be overweight at this temperature and area.. and said “EHIMM Zenbotbet de-ama” to to express how difficult is the situation to become overweight at this please for children…..

And the other participants also accompany her with a huge laugh which seems they are amazed by the question. And said it is nice if they can arrive at their home with very slow movement forgetting to be overweight or normal weight.

**I, do think the community have sustainable food for one year meaning can they feed their family without shortage for one year?**

**P2.** there is no family who can sustainably feed for one year their children currently because of the drought. But we are living by aid from the government and by selling animals.

P3: it the same as do not have any crop cultivated for us and our animals as well.

**I, How frequent is this happening and why?**

**P3.** Starting from three or five years we are living with aid from the government.

P6: the last six or seven years here in Felegehiot there was no crop production as there was no raining. As you can see there is even no for our animals to eat and we are baying crops from the market by selling animals like goat but it is very costly as we pay seven birr per one killo of any crop and we are living with aid coming from the government which is not efficient. So we are suffering from the drought and doing the same thing for many years particularly this Felegehiwot; but the other areas they might get at least small amount of crops but mainly this year we have nothing to eat for water we can bring from the river if we can full our stomach our main problem is hungry now.

P10: yes there is drought every year; and we have got aid from the government. Mainly this year even we have no food for animals.

**I, What do you think is the reason for the shortage of food?**

**P9:** the main reason is poverty due to shortage of rain. In the lowlands we are dependent in land or agriculture but now we cannot produce crop due to shortage of rain. We are frightened what we can feed to our children as we have finished the animals by selling. We as Felegehiwot we do have credit or dept to fulfill and feed our family members. But it true that eating is very nice if we get but here the problem is we do not have the chance even to get something to eat. As we did not get what we saw we simply get very small from the sorghum and teff and we limit our children to eat very small food which is one fourth of the “injera” by saying it is enough for you; even for our husband we say please eat this only. But if we get we know it is very nice to eat like meat and drink milk if available. The last six years we do not have any milk production it had gone with our fathers and mothers.

**Section two**:

**I,** **what kind of nutrition interventions are in place to improve health of pregnant women, lactating women and adolecents?**

**P9:** fafa is given by the government. There are mothers come to the health facility and could not get the aid and go back empathy hand as they were normal after screening. So the government is giving fafa and Plumplet still now without stopping. Lactating mothers are given five killo per month if they are thin during the measurement until six months.

P3: they are given fafa for three months during pregnancy starting from six months of pregnancy and for lactating mothers for five months regardless of her nutritional status at the six month they will transfer in to the child.

**I, Who provide this? From where do they get it?**

**P3:** The HEW gives the list of the mothers who need the aid by screening and there is provider or distributer to the mother. The mothers get it here or health post.

**I, are LW advised to visit HS for check up and services?**

**P8.** yes, there are advices targeting to mothers starting from pregnancy as they told us to deliver in the health center to get fafa. They advice us to came for check up every month.

P9: yes, they teach us even sometimes coming from woreda on how to feed our children and told us to feed our children very well. They also teach about washing of a child with Gujile lemat. They also advice to pregnant mothers to go to HF for check up and delivery and the community is also receptive and go to the HF. Hence, many of the mothers give birth at health facility as the government was also helping for mothers who give birth at HF by providing fafa for porridge for the delivered mother. But now the last three years the government had stopped proving this as there was misconduct from the community or the mothers themselves like robbery. Hence, the government is trying to help the mothers by providing ambulance.

**I, What about on getting extra-meal and rest for lactating mothers?**

**P7.** They told us to come monthly for nutritional screening of our children but they did not give as information or education on the need of extra-meal and rest.

P9: it is lack of resource but if we get; the farmers had no problem of knowledge regarding this. Followed by extended laugh ……..the problem is not with eating as I have to lactate my child I have to eat at the morning very well and different food at lunch time and wash my body and sleep and the same thing at night so myself and my baby will become healthy and nutritionally normal. So the child will not need the help if we feed appropriately as in our area or village we lactate at least for one year but in urban it is for six months. So if we get we feed our children very well and become normal but if we cannot get nor have lack of resource and we cannot feed the child and ourselves and the child will become thin.

**I, Does LW get screened for their nutritional status? How?**

P9: yes there is screening starting from three months of pregnancy monthly but when they say you are normal we do not go again as they have told me that I am normal even though you are suffering from malnutrition

P2: yes, she is right there is screening for example I have screened when I was pregnant starting from six months and she told that I was normal and I said thank you if I am normal. And after delivery I went to other HF and measured and told me that I am normal with good nutritional status with my daughter. Immediately after delivery I have measured and I was normal; I came also after six months for measurement I was normal again and from that time I do nt come as I consider as I am normal because of the previous screening result. At my first daughter she was fine when measured and my second daughter she had good nutritional status until September 2017 but at this time she had disease and was given treatment like shirop and give me a paper and told me to communicate with HEW and they provided me plumplet. And now she is very nice and looks good.

**I, What about on food diversification meaning what type of food are advised the mothers to eat?**

**P2:** there is advice for both the mother and on how to feed her child. They advice us like you have to feed your child like this and yourself. But give focus or first to our child rather than ourselves and lack of the resources but they give the education even through Gujile lemat. For example I came monthly to take the plumpnet and I have taken 30 plumpnets and they advice me to feed one per day together with other foods. They also told us to prepare to our children soup and soft porridge made of egg and others. The problem is lack of the food components like if have egg; I would have no teff and when I have got teff I would miss the egg; so this is the problem not education as HEWs are teaching us.

P9: they give education very well like prepare powder of teff or sorghum, been from market and mix this and add milk if available or egg then make porridge then feed the baby at the morning and similarly at mid-day. So they give education like this. The problem is the community or the farmer had lack of resources as they cannot afford to have these materials but the HEWs give education every time.

**I, Is it helpful for LW and her child?**

**P9:** yes it is helpful as it makes your child nutritionally normal and improves his nutrition and remains in his home.

P5: yes it is important as it helps the child to get extra-food. For example for thinner baby if you feed him very well he will become normal.

**I, What about on iodine salt utilization?**

**P8.** yes there is counseling and most of the people are using iodine salt as it protects us from goiter. And we are advised to add the salt after we finish cooking in order to avoid bitter test.

P2: we add the salt at the end of cooking in to avoid melting and not to be also so cold. If I add the salt when the cooking is hot it will melt and cold (to mean testing without salt).

P9: it is good to use iodine salt as it gives bright brain, and heart; and we told that it is very good.

P3: it has value as it is tested or packed and clean but the old one was not packed meaning open and dirty.

P7: it protects us from different diseases as it is packed and tested.

P11: previously we were using the old salt which comes to this place using donkey and it was not clean; hence we were exposed to different diseases like Goiter. But now we are using the tested iodine salt and there is disease like Goiter.

**I, on getting advice on nutrition sensitive agriculture such as home gardening?**

**P9:** there is no education on vegetation and the area is not good for vegetation as there is no water even for our drink.

**I, Do you think eating vegetable is important?**

**P9:** yes it is important for our health but it was stopped because of acute diarrhea in this area and even if we want to eat we cannot get it easily.

P8: we cannot cultivate here as there is no water even for our crops and we cannot buy vegetables because of the different diseases occurred last summer as it may transfer the disease as house fly may rest and contaminate the vegetable with the disease.

P7: the first thing it cannot be cultivated here as we do not have water but could have been cultivated if would have small ponds. So we cannot use water for vegetation as we do have shortage even for ourselves to drink.

**I, What about on the need to participate on the safety net program?**

**P7:** yes there is safety net program; I am part of the program as I do not have any animal and I am poor. They give us the aid as they went as they like; sometimes they give as monthly and sometimes after three months or four months. There is no constant time in getting the aid. The amount depends on the number of family in the house and they give as 15 killo per one individual per month.

**P3:** they told them for one year but they provide to them for six months and it is 15 killo per individual per month. There was also been and oil given but the bean is stopped but the oil is given still now.

**I, Is there special consideration for LW in safety net program?**

P3: there is no special consideration, the mother will be considered during the help if the whole family needs the help according to the criteria and given 15 killo per month for six months and continued for one year. There is no safety net targeting lactating mothers.

**I, Who provided the safety net program? Is there any other help?**

P7: it is provided by American organization through their representative here and his name is Milaw.

P3: it is through kebelle but I do not have full information as I am not in the safety net program.

P7; we are told by the kebelle leaders and go to the woreda and take the aid.

P9: there is no safety net for lactating mothers in the kebelle. It is given by studying your family but not as being lactating mother. But there is no any other help to both pregnant and lactating mothers except the provide fafa for both mothers if they are thin. There is no one from the kebelle or government side who tried to help mother considering the burden the mothers had in giving care to their family or considering the number of children that she is suffering from scarcity of resource. There is only aid for all of the family but they only provide the aid for five family size but not more than five and we take the aid from woreda.

P2: yes she is right; as there is no any aid for LM. Even the aid is given at different time interval and they give not to all the family members but if five they give to three of the family members. Hence; the two family members left will share from the three members given the aid which could not be enough. We also use this aid to pay our depts.; so even getting the aid is not enough.

**I, Is there any advice given to lactating mothers on water, sanitation and hygiene services?**

**P10.** yes there is education on sanitation as they told us to separate the living house of human and animal as you have to prepare separate house for hen and other animals. They also tell us to make small cannel for water reservoir and to construct and use toilet. We are also advised to keep our personal hygiene by washing our body.

P9: I have no different idea from her, as she said we are advised to separate the home for animals and human. We are also advised to keep our personal hygiene and sometimes there are individuals coming from the woreda to give education on preparing separate room for animal and human and to construct a place or shelf to put our materials or equipments. They also advice us to use only one cup or jar to withdrew water from water storage material like jerican.

**I, Who give this advice?**

P9: Most of the time HEWs and the kebelle leaders and community farmers during our meeting or community discussions also give advice on hygiene.

**I, Is malaria common here? If yes, are lactating mothers getting advice on the need to use ITN? Why? Who Advice them?**

**P5:** yes malaria is common and we are getting the advice on how to control malaria like we use ITN and clean our environment.

P8: we have to use ITN and sleep on bed. These are the control mechanisms of malaria.

**I, Are lactating mothers getting ITN first?**

P8: we all get ITN and we sleep inside the ITN with our children.

P9: we are given ITN to sleep with our three children this is to avoid biting by the mosquito. If having malaria we went to the health post and get screened by taking blood from the finger if positive they give us treatment and become normal.

P6: the ITN is given to all people living here but not exclusively.

**I, Is there a situation in this community how that you think that LW needs to be addressed through Targeted supplementary feeding for LW? Why?**

**P3:** it is not given to all LW but based on the measurement if they are measured lower then 12 or like that they will be given the Fafa. But it is not given to all lactating mothers and pregnant as they have a measurement.

**I, Any problem related to this or additional?**

P9: we cannot say there is problem as we do not follow them and check if there is problem. But I know they mobilize mothers of pregnant and lactating to make nutritional screening. And I heard complain from some of the mothers who did not get fafa as some of the mothers get fafa but not the other one. I do not know from where is the problem as it could be due to shortage of the resource from the above leaders or from the HEWs themselves.

**I, Are the mothers denied the service when they are nutritionally poor or having malnutrition?**

P9: yes some of them are denied even though they are very thin and need the help.

P3: I do not know as I am not receiving the aid. There are mothers saying someone is getting the aid who have better nutritional status then me. But we do not know whether it is true as we are illiterate and unable to read or measure their nutritional status and even I do not know exactly where the problem is.

**I, which of the above mentioned interventions is the most important for LW? Why?**

**P9:** LM with malnutrition should be given fafa as this helps the mother to feed her child with good milk. The child also should be given fafa as the child will become healthy if given porridge exclusively or without sharing to others. The mother with malnutrition will also eat the fafa exclusively and become nutritionally healthy or show improvement. So the government should strongly push on the nutritional supplementations to mothers and children.

P2: the government should help the LM to get food as if the mother is nutritionally healthy or not affected by malnutrition; she could care her child well as she can produce enough milk. If I am pregnant and get the right food; the baby will become healthy and well nutritionally. If I have got and cooked per the recommendation of the HEW and eat; I would become nutritionally normal and healthy. Similarly if I feed my child without sharing exclusively with hygiene; he will become nutritionally normal and respond very well.

**I, Is there any challenge which affects mother’s utilization of the interventions or given services?**

**P9.** she started with laugh…. All individuals are not the some … and laugh… all husbands are not similar as there are husbands who oppose for example birth spacing and say why are going to HF and utilizing contraceptive; why don’t you give birth always. Mothers nowadays are becoming aware about family planning as it is important for your child nutrition and health but when they decided to utilize but the husband will say you will not go. Regarding feeding mainly during pregnancy when you ask your husband to bay food for you in which we are interested with; he will reject it and say shat up. There is also health provider related barrier as they are also becoming feed up with you and say please go away; she passed her time by getting the aid “ Weskata” to mean who do not show any change with her child and why should she eat by herself. But the problem is she has no the resource or being poor.

P3: extended laugh… are this will be resolved if asked to be normal? There are husbands who stop to their wife from going to the HF to get family planning by saying what is your problem in giving birth as I am here to feed my children but this is from their mouth as they have no capacity to feed all the children appropriately and even say after birth please give him injera with salt. When I say the child had no cloth; he will replay when we were children we were walking without trouser.

……………….Extended and very hot laugh………………………………..

P3: There are also husbands who are cooperative and there are also mothers or women opposing their husband when they told them to utilize contraceptive by saying it is not your business as I am responsible for lactating and caring. So there are mothers who give birth within one year of birth spacing and the children are suffering from different disease and becoming put in difficulty. Should also send the contraceptives frequently as they are saying it is finished.

P8: using contraceptive is important and I am using it as it makes me healthy with my child.

**Section 3**

**I, What are the special things should women do to stay healthy in the community? ( during pregnancy, lactation and adolescence)**

**P9:** visiting health facility is very important as the mother makes to know the status of her child and if diseased get treatment. But the problem is here in the health post we are only getting anti-malarial drugs but not other drugs for other type of infections. For example; if the child had got common cold we could not get medication here in the health post but move to the health center which is very remote from this area and give as different types of medications and the child responded very well. Hence; it would have been very nice if we can get the medications here and very large health facility. So how can I visit to the health post as could not get the medication?

P3: she should get extra-meal and eat early in the morning with their children; and eat again at lunch time and extra after lunch to produce enough milk to her child. But this could not be done as we do not have the capacity to do this; as eating is very important if available not alone the lactating mother but also to others.

P6: we know it is important to eat extra meal but the problem is we do not have the resource.

P2: mothers should get extra-meal and rest but the problem is they should have a child who gives them the extra-cervices like for example bringing water, keeping animals like goat and other activities which exposes to the mothers to different diseases like head ach.

P7: yes we know getting extra-rest is important but it is very difficult for us as we bring water from very remote area or river.

**I, Do husband have a role on improving maternal nutrition? How?**

**P11.** Yes as everyone needs help to each other but the problem is the male has no time to work the outdoor activities. But we could have been very happy if they brought us water and woods to prepare food but he is very busy as he also keeps the animals as well.

P9: yes we need help but the problem is they do not have the knowledge and practice like for example if we order them to cook; they could not because they have no the ability to cook. But I would like to help me starting from early pregnancy up to delivery including the delivery period to bring me water and wood; so that I will not be exposed to high temperature and remain working only the indoor activities. If he has got different type of food he left it for me to eat and tell me that I should it very much as I am pregnant or lactating in order to be healthy.

**I, Do women in this community change their diets when they are lactating? How?**

**P8.** No difference as I do not have any choice because of lack of resource. Hence; I will eat what I have got what matters is getting the food.

P7: we do not differentiate the food; we eat as it is as we do not have choices.

P3: we know it is important to eat different types of food during lactation or pregnancy but we could not get it.

**I, What is the recommended food for pregnant and lactating mothers to eat?**

**P7.** They should eat orange, banana and meat. Then they will be very healthy with their children.

P3: mango, and other types of foods like milk, meat, orange and butter and banana; if available all types of food are very important. But the problem is we cannot get these foods easily.

**I, What affects women diet during pregnancy and lactating?**

**P3:** there is nothing affecting diet after delivery or during lactating for example I myself I was eating everything available even I can eat the wood if it would have been edible.

**P9:** we do not have any problem in diet but the problem is there is no food to eat or resource scarcity or being poor. Appetite we have full appetite and if we get and wood would have been edible we would eat. If we get for example honey we can eat together with “Kita” of wheat powder but we cannot get it but with “Kita” of sorghum with honey. But we cannot do this as we don’t have the resources like honey and wheat powder. Mothers like such feeding to produce milk and get rest and sleep but could not be practical.

**I, Is there any food which should not be taken by the mothers ( pregnant and lactating mothers)?**

**P9.** There is no any food which could not eaten by LM. They eat every type of food.

**Section 4**

**I, Have you ever gone for nutritional screening during community health days? where?who provide it?**

**P.** they give education by mobilizing the community in the health post. They teach to all the community about extra-meal and rest; food diversification and others.

**I, Is there community health days? Do you think they are important? what about to accessing routine services?**

**P9.** Yes, previously all people were invited including young men were given a mass treatment but in 2009 there were no such but we are waiting that they will call.

P2: there is no screening during community health days but only during monthly visit. But there is education on lactating mother like they should be helped and get extra rest and meal, the husbands to help their wives in bringing water and woods. If she give birth she should be kept safe in house in order to avoid disease like pneumonia. The LW should be helped until the baby is one year old.

**I, Do you think it is important?**

**P3.** No I have not participated in the community health days because I did not head about that.

P9: yes it is helpful as you can do as what was told during the discussion. At least you will catch up some or half of the discussions and practice in your home or share with your children about the discussion. Not alone with community it is very important if we discuss with two or three; so it is very important.

P6: she looks so tired to speak. Yes, they (HEW) give as the education during community health days.

**I, What are the challenges related with attending community health days and routine service delivery?**

**P3:** lack of awareness or knowledge as I thought as if I could not get anything new except the high temperature and wind. And I prefer to stay at home which is because of lack of knowledge.

P9: this is related with laziness and lack of awareness as it is important to attend community health days than remaining at home. The one who remain at home regrets when we tell them about the discussion. But if participated we will try to practice it at home.

**I, are pregnant and lactating women beneficiaries from of soft conditionality of the safety net program?**

**P9:** previously LM was working and was not given rest but this year they are allowed to take rest for ten months. But previously they were working similar to the other non-pregnant or normal community members. They went to the field with their child or left the child at home.

**Section 5**

**I, Do you think that delaying the age at first birth to after 18 is better for both the mother and the infant? Is it promoted in the community? Can you tell me who is promoting?**

**P9:** there is no early marriage in our community but early marriage can cause problems to the mother and child health as the mother was not ready physically. For example if she gives birth at 16 years old she may face difficulty during delivery due to narrowing of the pelvic.

P4: yes it has as she described earlier if she gives birth at 16 years old: the first thing is she will be damaged, she may face pelvic narrowness and the baby may also have anemia as he takes blood from his mother. But if she give birth at 20 and above years old she would be physically fit and have normal body; hence the baby would be normal as well.

**I, Do you mean they are working very well and there is no early marriage in this community?**

**P9:**  yes, there is no early marriage now in our community at age of 15 or 16 but only for above 18 meaning at 20 and above years old but previously yes there were early marriage.

P4: yes it is working very well.

**I, Do you know about spacing of birth intervals or family planning? How much should be the gap between successive births? Is it promoted in the community?**

**P7:** yes, giving birth at one or two year is different from giving birth after five or six years of first birth. This is because if you give birth within one year difference the pelvic will not be strong or become loose but if you give birth with intervals or spacing your pelvic will not be damaged and become strong.

**I, What about to the child?**

P7: He would be affected as he could not get appropriate care if I give birth within one year difference or birth spacing as to which I should care. Hence; both of the children will be affected but if it would have been born with spacing I could give him full care and the child will grow healthy.

**P4:** yes there is difference between those who give birth within one year birth spacing and those who give birth by spacing. The mothers who give birth within one year difference may be exposed to excessive bleeding and other diseases. But if she gives birth after four or three years of the first birth she would not be exposed to different disease as she can keep her hygiene and the baby will also be healthy as the mother can give him full care. When the mother gives the birth within one year difference the baby will not gate the actual treatment and may eat soil and exposed to different diseases.

**I, Can you tell me who is promoting and you heard for the last time?**

**P4.** The health extension workers and kebelle leaders or cabine give as the education about family planning.

P9: the HEWs give as the education or promoting about family planning and there is no in our community who do not use family planning. They teach us to use family planning as it is helpful to the mother to keep herself and the community is practicing it very well they have utilizing the family planning services such as for three and four years contraceptives.

**I, what kind of community conversations or massages discuss women’s and adolescents nutrition?**

**P8.** I do not know and I have never participated in such conversations.

P4: yes we are given education during their house to house visit and to the total community during community health days to the total population in the health post and in church on Sunday.

P9: yes they give education during any meetings of the community. There are also kebelle cabine who are involved during the discussion.

**I, Do all women get the massage easily? What are the barriers for access to information for nutrition during pregnancy?**

**P4.** yes they get the massage easily. There is no any problem in getting the information about nutrition. The only problem is related with scarcity of resource.

**Section 7**

**I, How can we improve maternal and adolescent nutrition in this community?**

**P4:** in order to keep our children, our eating materials clean; and to avoid infection we need water. As we do not have water here; we bring water from very remote area. The other is we do not have health center here to get full diagnosis and treatment to our children and ourselves; we get only anti-malarial drugs from the health post. So we need health center around us.

P9: yes we do have water problem as we travel more than six hours to get water and we need also health center as we could not get medications like shirop here in the health post. We ask the government to provide us these services. The government should also support fully to mothers and their children who are affected by malnutrition as there is shortage.

P2: yes it is similar to what the others said. We do have shortage of water and we could not able to keep our children clean. The health post has also only anti-malarial drugs.

**Summery points**

**Section one:**

- Medium to severe malnutrition and Anemia are common among mothers and children.

**Section two**

- Free health services during ANC and delivery; Plamblet and FAFA for both pregnant and lactating mothers and their children are the interventions in this woreda.

**Section three**

- Pregnant mothers should visit HF for follow up and check up during pregnancy and should get extra-meal and rest during and after delivery.

**Section four**

- Nutrition screening is routinely performed as it is important to determine the nutritional status of the mothers and their children and to give them the nutritional interventions.

**Section five**

- The programs or policies targeting delayed marriage and birth interval are working very well.

**Section six**

- There is community based discussion on nutrition mainly during community celebrations like Sunday in the church.
- **Finally I have finished my questions and I would like to thank for your time, patience and answering all the questions. Thank you very much!!! Thank you!!!**
