# Supplementary material for: Barriers to utilize nutrition interventions among lactating women in rural communities of Tigray, northern Ethiopia: An exploratory study
Source: PLoS One. 2021 Apr 30;16(4):e0250696. doi: 10.1371/journal.pone.0250696 (PMC8087028; doi:10.1371/journal.pone.0250696)
Supplement: S2 File — (ZIP) [file pone.0250696.s002.zip › S2_File.Doc/Lacatating women_IDI & FGD/106_IDI_Lactating Woman_Hatsebo Kebele_Laylay Machew woreda.docx]

**Operational Research on Adolescent and Maternal Nutrition in Northern Ethiopia**

**An In-Depth Interview with Lactating Women**

**Introduction**

Good morning. Welcome and thank you for taking the time to speak with me. I am Hailemariam Tekie from Mekelle University. I came here today to study the factors that influence the nutrition of mothers and adolescents in collaboration with the Regional Health Bureau and UNICEF. As part of the community, no one knows other than you about the problems existing within the community and also possible solutions for the problems. So, your participation is very valuable. The things that you tell me will be used to improve nutrition programs and services for women in the region and the country. Your name will not be included in the report. But, in order to capture all the ideas that you share me, I will record the discussion. The interview will take about one and half hours. Do you have any question before we begin? So, if it all right with you, I will turn on the tape recorder now.

**Section A: Interview details**

1. Zone: Central
2. Woreda: Laelay Maichew
3. Kebele: Hatsebo
4. Name of key informant: Nebarit Kahsay
5. Institution of key informant: _______
6. Interviewer name: Hailemariam Tekie
7. Date of interview: 19/11/2017
8. Interview start time: 10:27 am
9. Interview end time: 12:25 am

**Section B: Interviewee professional information**

1. Gender:
2. **Female**
3. Male
4. Age: **37 years**
5. Highest level of education:
6. No formal education
7. **Primary education (Grade 6)**
8. High school
9. College education
10. Bachelor degree
11. Master’s degree
12. Current job/position: **House wife**
13. How long have you been in the current job/position?
14. _____ Months
15. _____ Years

**Detail of the In-depth Interview**

**Key:** I – Interviewer ; P - Respondent

**Section 1: common maternal (pregnant women, lactating women and adolescent girls) nutrition problems in the community**

**I: What do women do to stay healthy in this community/Woreda?**

**P:** To be healthy, first we go to the health center for child delivery and then we breastfeed him the colostrum, wash him after 24 hours, breastfeed him frequently, then the health extension workers check his weight and follow him even by coming to home, they give drops to his eye at the health center after delivery, they gave him drop through his mouth, inject him to protect the navel from bleeding, I took a drop after two weeks and follow monthly vaccination, and now I am left with the ninth month. With regard to nutrition, in order to have breast milk, I eat additional one or two foods and he has to breastfed and now he has started additional food as he is above six months old. We also come to the health post to check the weight.

**I: Is it for you or your child?**

**P:** Why should I check my weight?

**I: Ok, we will talk about child later on but tell me what you do to be healthy?**

**P:** For me to be healthy, I eat diverse foods and I have taken vaccination to protect a disease to the child, what should I do others? That is what I do.

**I: What about other than eating foods**

**P:** We have irrigation and produce vegetables, we have lettuce and all other vegetable and we do not purchase any vegetable.

**I: What are the vegetables that you produce?**

**P:** Spinach, lettuce, cabbage, onion, garlic, tomato, we have all in our farm.

**I: So do you use them for consumption?**

**P:** Yes, I consume vegetables.

**I: What are the common nutrition problems in the community for lactating women?**

**P:** The child may be exposed to different diseases, he may be less in weight, may face diarrhea and thinness.

**I: What about the mother?**

**P:** It is if I get diseased that the child will also be sick. If I do not eat properly, he will not get enough milk, then he will be exposed to diseases and for me if I am not getting good, what will happen?

**I: Which women groups are the most affected by these nutrition problems?**

**P:** I think it is the lactating women.

**I: Why?**

**P:** What a reason can I have? We do not have an experience before this time, we are in a good condition but if someone is not in a good condition, he will be exposed to diseases. It is a must that he will be exposed to diseases. That is it. But if you come to the health post and check your health as they are also giving us information, there will not be a problem.

**I: It is true that if the lactating women or the pregnant women, or anybody could not get food, there will be a problem. So, which women groups (LW, PW, and adolescents) are the most affected by these nutrition problems?**

**P:** The pregnant women.

**I: Why?**

**P:** If the pregnant women do not have many check-ups, she will be exposed to death.

**I: Why is she exposed to death?**

**P:** Because of lack of care.

**I: Do you think that there are other problems to the lactating women related to nutrition?**

**P:** It is like that of pregnant women. She may feel dizzy. [Laughing] what should I say?

**I: What other problems?**

**P:** The child will not also be grown well. If she faced problems, there are many problems. If the mothers could not provide a child a breast milk for six months (*exclusive breast feeding*), he will not grow normal.

**I: What other problems can be observed on the mother herself? May be you can share me if you have an experience related to this.**

**P:** I have no any experience related to this. In our areas and in our community, it is good that we do not face this kind of problems.

**P:** You brought me a difficult education. [Laughing].

**I: You told me about dizziness which may be related to lack of iron. Can you also tell me about goiter and night blindness?**

**P:** There may be goiter and night blindness. In our local areas, it is ok but sometimes it happens in the elders. They tell us that the cause of goiter is the sun. When they go outside during sunny, they face the problem of goiter. But as I do not have that problem, I cannot understand how it happens. Anyways, we are using iodized salt because it is believed that iodized salt treats goiter.

**I: What about night blindness?**

**P:** Nowadays we are feeling bad because of the wind but night blindness is ok this time. You may find some elder people affected by trachoma but now it is not common.

**I: What is the reason that there was an incidence but now it is reduced?**

**P:** Now there is vaccination and we are provided with tablet to prevent eye disease and drops for children. So, as there are health services and the government is following these issues, there is an improvement.

**I: But what could be the cause? There are health services if you are sick, but what could be the cause of trachoma?**

**P:** I do not know the cause.

**I: What about non-communicable diseases such as pressure, diabetics, and others among women/girls in the area?**

**P:** As to me, it is ok in our local areas though you may get 1 out of 100. Anyways, most of the people are healthy. I can say we are ok with that but I do not know the cause. What I heard about diabetics is that if the sugar is not heated well and eating some sugary foods.

**I: What do you think should be to prevent it?**

**P:** It has to be heated before eating and keeping hygienic conditions.

**I: What about high blood pressure, if there is?**

**P:** What should be done if there is high blood pressure? It is reducing the foods that increase blood pressure. What could be another reason?

**I: So, you mean the cause for blood pressure is the food?**

**P:** You know it is not about eating extra food or eating nutritious foods. The nature is different for different people. God did not create all the same. Some of us may have high blood pressure while we are eating the same food at the same time and some of us may have iron deficiency. Anyways, it is said that iron deficiency can be replaced (corrected) but high blood pressure is difficult but I do not know in the health point of view.

**I: So, if I get your point, you are saying that anemia is related to food but high blood pressure is related to the natural condition of individuals.**

**P:** Yes, high blood pressure is a problem and I do not believe that it can be treated by yourself.

**I: What do you think about the proportion of weight or height of lactating women to their age?**

**P:** You know, you may be short due to disease but shortness or tallness is given by God. The brain activity and health can be different if there is difference in feeding practice but to be short or long is the creation of God. You cannot say that I have long child because I fed and cared him properly and you cannot also say he become short because of less care given to him. So, someone may be short due to disease but I do not think he will be short because of food.

**I: What about proportion of weight to age?**

**P:** In the case of weight, it could be true. He may get kwashiorkor (enlarged stomach) and get sick and there could also be diarrhea on the child. Otherwise, height can be short only because of God.

**I: What about lactating women?**

**P:** In the case of weight, you know for example there are lactating mothers in urban and rural areas, but we cannot say I have to leave work as I am lactating mother. Though we eat the foods, we try to accomplish all activities so that all lactating women may not have the same weight. As, to me, you should be in the middle; you should not be seriously harmed and as we are in the rural areas, I do not want to say our weight is good or I do not want to say also we are this and that. But if you are in the urban areas, lactating women are respected, for example if she has a job and you are educated, you may tell her to stay at home assuming that your salary is enough so that the child will grow well until he starts eating food. But in the case of farmers, it is different because of livelihood status.

**I: Do you think this is the problem in many households?**

**P:** Thanks to God, we have not seen bad in our local area. There are some who can have child delivery through surgery but some 1 or 2 or 3 in 100 may be found. It is ok that most of the people give care to their health unless there is a problem.

**I: What about overweight in women/girls?**

**P:** With regard to this, I have not seen like that. I mean we are ok. I have not seen bad.

**I: Could you tell me about food security in the kebele/woreda?**

**P:** If you simply try to be careless, you will face shortage of food but if you try to work hard devaluing yourself, you will not face shortage of food. So, sometimes there may be a shortage of food.

**I: Would you explain it in detail please?**

**P:** For example, I am a farmer and I have a baby. If I say I have to get rest, how can I eat nutritious food that I wanted? But if I eat and work hard, I will eat more and more nutritious foods. Otherwise, you may not also breast milk your baby. It is true that he will be exposed to sun and wind and he will not be like that of treated ones. If you are son of a farmer, you will know that son of a farmer whether he is from the rich or poor household, will be treated less compared to the others. So, there will not be a benefit by getting rest otherwise what will you eat if you do not work? It will not make sense.

**I: Of course there will be a difference among households. But generally what can you say about food insecurity in the local area?**

**P:** There could be food insecurity in the area. Actually, in our local area, food security condition is ok. We have never seen and heard of about someone son of or daughter of this or that family is hungry. We have never seen any young or any other in our kushet about hungry. There is no any drought in our areas. We have never been affected by drought and the government is supporting for those that are poor through safety net program.

**I: But do you think that there is a situation where some face the problem of shortage of food? In what situation do you think they are facing the problem?**

**P:** I do not know. There may be a shortage of food probably due to negligence otherwise there is no drought. But there is no that much shortage in the area. They do not complain on God and the government.

**I: So, you are saying it is good?**

**P:** Yes, it is good. The government is also supporting the poor through safety net.

**Section 2: Barriers to access and utilization of nutrition services**

**I: what kind of nutrition interventions are in place to improve health of the pregnant/lactating/adolescent girls in this woreda**

**P:** The health extension workers are teaching us on how to eat nutritious foods. For example, they showed us how potato is cooked and provided to children after six months. We are also taking their lessons.

**I: What other intervention do you get other than this?**

**P:** So far, we are not supported. I have never been supported by the government. But in terms of education, we have been learned. Actually, education by itself is support because without education, you will not be learned and changed. So, we have learned and taking that education, we have been working otherwise we are not supported.

**I: Ok. What about other interventions?**

**P:** We are advised not to shorten birth spacing. They tell us to use contraceptives to increase birth spacing. Anyways, it is to decrease the family size. So, there is counseling at kebele and other levels.

**I: Would you please explain it?**

**P:** If the birth spacing is short, the mother will be affected.

**I: What about check-up and other interventions?**

**P:** We go to the health center and check our weight or I mean take the children every month for check-up. What will be better than this check-up?

**I: What about extra meal during pregnancy and lactation?**

**P:** Why should I talk about others? I am not carrying baby without getting pregnant. Anyway, as a pregnant woman, we come to the health center for check-up for about four times. That is what pregnant women do.

**I: Would you tell me explicitly about yourself what you do when you are pregnant?**

**P:** When I am pregnant, I have got check-up four times since four month of pregnancy. So, I have visited four times for check-up.

**I: Did you get counseling or you went there by yourself?**

**P:** They give advice and counseling but once someone makes it clear for you, you should not wait until they said it again. Why should you wait? They do not say that you must come only in a month. You may come after two weeks or any other convenient time for you. It is good to check your health and the status and position of the baby. I remember, it was at mid night, 12:00 that I went to the health center because i feel the indications of child delivery so that I delivered at 1:00 after mid night.

**I: You started check-up when you were four months pregnant. Is it because there is no need to go before that month?**

**P:** They advise to go to the health center for check-up but why should I go there when it is only blood.

**I: So, do you mean it is not needed?**

**P:** I do not mean like that but you may not even know whether you are pregnant or not for 2 or 3 months. So, you cannot go by this time. Actually, they do not tell not to come for check-up. So, it is my own idea that I go there four or five months after pregnancy.

**I: What is the benefit of this to the baby?**

**P:** It has a benefit for the baby. How could there be a person without a reason? It is beneficial for the baby. If the child gets nutritious foods since conception until five years old, he will reach where you wanted. We have also been provided a book that tells about this. After all, he will get food from me. If I eat good food, he will be good and if I do not eat good food, he will not be good. So, it will be beneficial for his body and also for his mind.

**I: So, do you get screening in the health center?**

**P:** Yes, I am measured. They measure us. I was measured and told me that I am good. Actually, I forgot how much my weight was but they told me that I am normal and they told me also that my blood is good.

**I: When was that?**

**P:** It is when I was pregnant.

**I: Do you think that it makes a difference?**

**P:** Let me tell you frankly that I gave birth only two times in the health center. During the earlier time, there was no much education or it might be due to childhood (lack of knowledge) but when I was giving birth to this baby and the baby before this one; I believe that it is good to check whether the position of baby is good or not though I did not go to the advanced check-ups using computer systems. So, it is important and beyond that. If you have high blood pressure, they tell that you have high blood pressure and if you have anemia, they tell that you are anemic so that they recommend you to use treatments. You will also be confident enough if you know about your status. If you are told that you are normal, you will be happy. It is very important.

**I: You told me about the nutritious foods like that of potato but would you please tell me in detail if there is food diversification during pregnancy and lactation?**

**P:** Let alone a pregnant woman or lactating woman, any person not getting pregnant or not lactating is expected to eat nutritious foods in order to keep his health better and protect from disease incidences by taking fruits and vegetables. So, they are giving education on these issues so as to protect us from different problems. As we understood the benefit of this, we take the lessons.

**I: Can you give me examples of eating nutritious foods for different problems related to nutrition?**

**P:** For example, it is good to eat lettuce to protect from disease, that is, your body will not be exposed to diseases. The other things are ahhhh, I forget to mention the names. Anyways, all of them have their own benefits. For example, some of them could be for the brain, some may be to protect from diarrhea and other diseases, and it could be to build the body. It is because of their different benefits that diverse foods are recommended to eat. Why could they explain us to eat diverse foods if they could not have different benefits? There is no food that does not have its own benefit either to protect from iron deficiency or other preventions.

**I: What about iodized salt in the community?**

**P:** If we are going to talk about the community, let us say our parents, they did not take education and they did not reach the level that we have got. Still they are using the salt that is not iodized. But the ones like me that are getting education, I think they are using iodized salt. It is the new generations that are using iodized salt but not the elders. But, the good thing is that they are not exposed to the diseases. It is the new generations that are getting the education. So, if say all the people are using iodized salt, it will be false.

**I: You told me earlier that you have vegetable gardening. What about other people in the community in the kebele?**

**P: T**hose that have access to water for home gardening are producing and those that do not have access to water do not produce but they purchase from farmers producing like ours and there is no one that does not consume vegetables this time.

**I: Do you think many of the farmers produce vegetables or what?**

**P:** I cannot say most of the farmers produce vegetables. Probably half of the households or I think it is most of the people with no home garden vegetables.

**I: If most of the people do not have access to home gardening, what about consumption?**

**P:** In terms of consumption, thanks to God, they consume by purchasing from here and there because of the market expansion. I can say, there is no one that does not consume vegetables.

**I: What about Safety net in the local areas?**

**P:** Yes, there was safety net program but I do not have the information for this year because I am not good in communication. Otherwise, I know that the poor are working on the safety net program and getting support for the household. There are many people that have been improved. Those that show an improvement are graduated and they are replaced by other poor households.

**I: What kinds of individuals are involved in the program?**

**P:** Those that do not have enough foods and the land was not productive because lack of fertilizers. There was no good agronomic practice otherwise the land is itself. The land could not increase but it could be decreased because we are increasing children. There was no education on how to utilize the land properly so that it was not treated well. We were simply applying inappropriate fertilizers and it was giving us equivalent to our practice. But now, through the safety net, they ask us to take fertilizers according to the standard. They tell us to take the fertilizer required for the specified farm size. It could be one or two quintal depending on the land size. They give the farmers assuming that they will be benefited. Though the farmers do not accept the benefit of the fertilizers, they are forced to take it otherwise they will be removed from the safety net membership. So, as they are afraid not to be excluded from safety net, they start taking the fertilizers. They were forced to take the fertilizer and apply it on their land and the land become productive in producing grains and others. As a result, they get enough food to satisfy the households. So, though the farmers were urged to take fertilizer, now they are demanding more fertilizers. If the land is treated and become fertile, it will increase the yield and become productive. So, now yield is increasing from time to time. Now, they are involving individuals that do not have agricultural land. Those that are married but do not have land are involved in the safety net program. But most of the people are improved and are out of safety net program.

**I: What are the improvements from safety net beneficiaries or what are the changes?**

**P:** You know change mean is that if they had only one house for a family, now they will have two or three houses or the improvement can also be on the fences and doors. The other thing is those that had one ox will have two oxen or those that had one ox will have ox and cow, and fulfill other necessities and are called models. As I said earlier, the land will also be productive giving twice the previous yield. So, there are changes otherwise how can you say there is an improvement if some could not improve house, cloth or others. It is visible to anyone within the community, God, and the government.

**I: So, you are saying safety net is beneficial.**

**P:** You know, the government is saying you should not be dependent but what I am saying is that they are told to be excluded from safety net if they do not take fertilizer for the specified land size. They will take because they do not want to be excluded but the fertilizer become significant and then after they consider the use of fertilizer, they will observe the importance of the input. This is because of safety net. Actually, there are educated households that understand the benefit from the beginning and use it including myself. Personally, I have never been in safety net program because we work hard to improve our livelihood. We do not have the hope to be supported or aided or getting a loan from any one. So, it is only those that do not have a land that are under safety net program by this time. Most of the other groups of people in the community are not members of the program because it has been a lesson how to improve your livelihood.

**I: Are there other similar interventions that support the community in these areas?**

**P:** We do not have anyone supported.

**I: I mean it can be governmental or non-governmental organizations?**

**P:** You now, for example they may organize to have a meeting; the meeting could target the models but the meeting may not be carried out in the presence of models that the government is intended to have discussions with because there is partiality. They may say why not I should attend the meeting than sending others. I am for example a model but we do not participate on the trainings organized for us. So, though the government has many activities, it is only those that are working around the office are the beneficiaries. If for example, they are told to send participants, they send their wife or sister or wife of his brother. It is just partiality.

**I: What about water, sanitation and hygiene practices?**

**P:** The government has provided us two or three water pumps around one area. This is well designed in that the waste water is going in one side and collected in well prepared cement made basin for cattle drinking purpose in the summer season. So, we are drinking quality water. We are not considered to be different from the urban areas.

**I: What about sanitation and hygiene practices?**

**P:** I can say most of the sanitation and hygienic practices are in a good condition in the areas. We had a simple toilet and after that it has been two years that we are waiting to receive the cement made sitting material of the toilet though we have also prepared the house for the toilet. Now we are using open defecation.

**I: So, as they could not provide you the materials you do not have a toilet.**

**P:** Yes, actually we had a toilet but now we do not have it because of their delay.

**I: Ok. Who is promoting the messages for these activities?**

**P:** It is the health extension workers. They tell us to build a toilet properly. Now we have built a house for toilet and it has been two years since we paid 240 birr for the toilet material.

**I: Who is going to facilitate the provision of the material?**

**P:** You know there were two or three materials that came to the kebele and my husband went there to bring it but they told him that there will be another that will come near to your local areas as these ones are to the beneficiaries around here. From that day on, we are waiting till now.

**I: So, are you waiting the kebele to get it?**

**P:** What they are saying is we cannot transport the materials for each of you by a car. So, increase the number of beneficiaries 2, 3 or 5 and we will provide you. But the problem is that the people could not be involved probably due to the money to be paid for.

**I: So, can you explain the benefit of sanitation and hygiene practice?**

**P:** There are many benefits. If you have waste material around your area, there will be an ameba and exposed to other diseases from what you eat or drink in case they were infected. For example, if that was infected with flies, you or your child will be exposed to diseases.

**I: What about malaria in these areas?**

**P:** Malaria is only when the children and youth are going to Humara that they are affected. Otherwise, in our area it is good in terms of malaria. There are no swampy areas suitable for mosquitoes. They are also supporting us ITN. There are actually cases during the rainy winter season of July and August that the land becomes moist because it is clay soil otherwise, most of the time there are no mosquitoes.

**I: Do you use ITN during the rainy season?**

**P:** Yes, we use ITN. It protects mosquitoes even if there are some that sounds (mosquitoes sound) inside but it does not cause much problem.

**I: Who is promoting the use of ITN and provided to you?**

**P:** It is starting from you, from the region. The region gave to the woreda, the woreda gave to the kebele, and the kebele to the local health posts. So, we get it from the health post.

**I: Is it accessible for all?**

**P:** I use it for myself but I do not know whether there is enough ITN supplied for the community or not. I do not know but it has never been inaccessible for me.

**I: What about deworming in children?**

**P:** Yes, they give us tablet but I am not sure whether it is for deworming or anemia. Previously, they were giving us and later on they were also giving us during pregnancy but I do not know what. The problem is from ourselves that we eat foods unhygienic. It will also be a problem if the utensils are also not clean where there are flies. So, it is because of these problems and I do not think is will come by other reasons.

**I: You told me earlier that you are measured and you were good but what will happen if some –one is underweight?**

**P**: There is no underweight. Previously there was FAFA provided to children. But since 7 or 8 **y**ear ago, there is no anything provided to children. What was happening previously is that mothers who are thin were provided oil or something that I am not sure now because it is long time ago. But thanks to God, I and children have never been exposed to that problem. So, I have never seen anything in the last 6 or 7 years. But I do not know if they are doing something secrete. Whether it in children or mothers, there is no anything unless there is partiality to provide them secretly.

**I: Do you think that there is no support because there is no problem like you are?**

**P:** I do not say that there is someone under who is in need of support in our area and I do not wish too. I am sorry if I made a mistake but we go to the health center for vaccination and check-up every month. I have never seen a child who is under rather I can say they are better and normal children. But previously, there were children and the mothers as well who are underweight and very thin and were provided with FAFA and oil. Now, I have never seen like that.

**I: What about targeted supplementary feeding on pregnant women?**

**P:** No one dislikes to be supported. You know, for example parents educate their children to reach at some point and in the case of illiterate, parents provide their children something after they are married and advise on how to run the rest of our life. Then after the child should implement what has to be and should not wait his parents to do that for him. So, I do not want the government to give me an aid. For me the education that the government is giving is enough and that is the support expected from the government and I have to do the rest by myself. I told you that I have irrigation; do you think that our parents have the access for that? Now it is because of the education that there is increased number of educated individuals that we are advised to harvest two times a year. So, I do not want to be supported and I should not minimize the chance of support for others. There could be someone but I do not want to be supported.

**I: Which intervention do you think is most important for pregnant and lactating women?**

**P:** There is no one that is not important. The education should be increased and I would like to implement practically the education that we are provided. It will be difficult for me to differentiate by saying this is good and that is bad. Anyways, everything is good and I would like to say let us not miss the advices. The advice should be continued as people can forget them but if you refresh them, they will accept and implement the activities as new.

**I: But, can mention the most beneficial one?**

**P:** The most beneficial is child feeding. Actually, for pregnant women, check-up and eating nutritious food is known. But we need to also focus on what food is needed for the child, how to grow the child, and other so that I would like to have education on these issues.

**I: What about lactating woman?**

**P:** It is true that lactating women should also be educated. You cannot say everybody is aware of and is educated. Probably it happens that they have tried but they need education.

**I: What are he challenges that limit the implementation of the interventions in relation to nutrition of pregnant and lactating women?**

**P:** Though we have paid, there is lack of supply of toilet facilities. The other thing is that water is not accessible for all. Even for us, the water that we are using for irrigation will get dried in the dry season. Actually, the government has tried to search for water using machines but it was not found.

**Section 3: Perceived needs of women for relevant services during pregnancy, lactation and adolescence**

**I: What special things should a woman do to stay healthy during pregnancy, lactation and adolescence? Explore for pregnancy, lactation and adolescence?**

**P:** You know, we need to get education. What other support do we need? But education for women during meetings is special thing. We have to be supported in that way.

**I: What do you think would be the role of a husband to a lactating woman?**

**P:** In the case of pregnant woman, he has to do the activities that are difficult for her that may cause the change in the position of the baby. In terms of food, it may be impossible for a husband to prepare and provide her [Laughing]. I do not think there is a husband to do that. Anyways, every husband wanted his wife to have healthy baby. I do not think there is a husband who wanted her to face problem and get harmed.

**I: What about in lactating women? What should he do after delivery?**

**P:** After delivery, they have to go to the health center for child vaccination on time. It is just similar to that of care during pregnancy. The husband has to provide also nutritious foods to protect the child from the problems and provide additional foods to the mother. Actually, it is the husband that works hard to get and bring foods to the house. Who others will work and bring food for the woman? He will also work to provide additional food for her. He has to also help her not to do activities that may affect her, and encourage her to provide enough milk for the child.

**I: Tell me about yourself. What is the condition in your family?**

**P:** Ours is like that.

**I: What is the support that your husband provided to you?**

**P:** We are doing what has to be done. We discuss and then distribute the activities. I may be involved in the activities by myself if I believe I have to be otherwise I am not forced to do if it is not recommended for women.

**I: Do women in this community typically change their diets when they are pregnant and lactating?**

**P:** I change my diet. I have to eat a variety of foods and also when I was taking two times, I have to make it three times a day. That is the change. How could it be the same for lactating women and others? For the lactating women, food is prepared that is easily edible and convenient for her. Everybody knows what foods are important and not important for her. There is no one who is not getting prepared during that time unless there is an economic limitation.

**I: Ok, can you tell me the foods that you add after delivery?**

**P:** When you give birth to a child and when you loss that much blood from your belly, you have to take foods that can replace the loss.

**I: Could you give some examples?**

**P:** For example, porridge is provided for pregnant women every morning. She is also provided gruel and others. But it depends on the livelihood status of the households. Personally, I take porridge in the morning and also I take hot drinks.

**I: What about when you are lactating?**

**P:** As the child is getting food from the mother, she has to take diverse and nutritious foods that are fresh or cooked. So, if possible, you have to select the foods that are good to the child. When you were in the bed within the seven days after delivery, everybody will be provided with porridge. After these seven days, you have to prepare and eat additional diverse and nutritious foods which may be injera or any others. This is what I do until six months to eat nutritious additional foods. It is just after six months that I went out for additional activities. So, as he is taking food from me while I take egg, meat, and other foods, I have to eat nutritious foods.

**I: What foods are recommended for pregnant and breastfeeding women?**

**P:** What they recommend us in the health center is to take at least one additional food. There is no recommendation that tells you to eat injera, bread, or porridge. But there is education about to eat one extra nutritious food.

**I: What about foods that should be avoided for pregnant and breastfeeding women?**

**P:** There is no food which is not important for the women unless there is shortage. If there is availability, everyone wants to eat nutritious foods.

**I: What about foods that may affect pregnant and lactating women?**

**P:** I heard about alcohol (*areki*) during a social occasion, not from the health extension, that this alcohol dries-up breast milk. Personally, I was taking alcohol but after six months pregnancy of this child, I stopped drinking alcohol. Different persons did not accept this but I accept that alcohol is acid that will dry-up breast milk though I did not get this information from the health extension.

**I: What about other foods that may affect the woman or the child?**

**P:** It is the shortage of food (hungry) that affects but I do not think there is another that affects.

**I: what about gender disparities in women’s diets before pregnancy and during pregnancy, lactation, and adolescence?**

**P:** It might have been in the earlier times but this time I can even say women are eating more than men. Oh, men, they are only eating once and stay the whole day. So, I do not think that there is disparity among men and women. I have not seen in my parents and I do not differentiate males from females at home. This time you eat together so that if it is enough, you will satisfy together otherwise, it will be lesser for all together.

**Section 4: Other interventions that improve pregnant, lactating and adolescent nutrition**

**I: Have you ever gone for nutrition during community health days or routine service delivery? If you went, what was your experience? If you didn’t go, why not?**

**P:** There is community health day. I cannot say everyone in the community is involved in the community health days but most of the people are involved in the education. During the community health days, we are learned about keeping hygiene and sanitation.

**I: At what conditions are the community health days organized?**

**P:** There is no fixed day. It is just when they wanted to convey some messages.

**I: So, are there screening during this day?**

**P:** Ahhh! I think I did not understand your question. I was telling you different.

**I: My question is that are there health day at community level in your areas?**

**P:** This is not that much common. I do not want to tell you something that is not really happening. I have to tell you openly so that it will be practically organized for the future as it not happening now.

**I: Do you mean that there were no community health days at all or it is only recently ignored?**

**P:** I have never seen community health day that is organized monthly.

**I: It may not necessarily be monthly but are there community health days even if it is once in a year where there is also screening for lactating women?**

**P:** Do you mean for mothers or at household level?

**I: The community in general.**

**P:** I do not understand that. There is about a year like rehabilitation meeting to talk about health. The health extension may have like that but as we do not have job there, we have never been called for the meeting. They may assess and learn to each other during the meeting.

**I: What about, for example the health extension coming to the community to teach about vaccination and others?**

**P:** Yes, there is. There is a program that they may have a schedule to call children 2-5 years old and therefore, they are told to do so depending on the schedule of the program. When the program comes, we have a trend to take 2-5 year old children for vaccination.

**I: What about for adults?**

**P:** Yes, there is also for adults but it has been long time.

**I: Do you think that it was important?**

**P:** You know, you cannot say whether we are healthy because of that or not. What i believe is about vaccination starting from childhood. Earlier times, children were highly affected such measles and others but now due to vaccination children and including myself, I was vaccinated while I was a child, there are no problems like that of measles. So, it is very important.

**I: do you think that all the community participates in the program?**

**P:** Now, the community has observed it on their children and understood what was before and how are we now? So, they have accepted it and participate on the program.

**I: Is it only about vaccination or there are also other interventions?**

**P:** There is screening and then they recommend what has to be done and what has to be provided. They give the medicines and other treatments whether it is a tablet or drop based on the results of screening.

**I: What about diet related interventions after screening?**

**P:** Yes, with regard to diet, they are measured to check whether there is a change or not.

**I: Are pregnant and lactating women beneficiaries of the soft conditionality of the productive safety net program?**

**P:** I have never been safety net beneficiary. I am not sure but as to me generally pregnant women does not work on activities of safety net program. Lactating women may work but I do not think pregnant women are involved in the activities. The advantage of safety net is that if one is involved in the activities, all the households will be provided. So, that is the benefit of safety net and I do not think pregnant woman is involved in the activities but I do not know about lactating women. I do not understand these things. It is the development agents that have the full information.

**Section 5: Understanding perceptions of age at first birth and birth spacing**

**I: Can you explain that delaying the age at first birth to after 18 is better for the health of both the mother and the baby?**

**P:** Now, according to the law, there is no one who can let his daughter to marry before the age of 18 years. I do not think that there is any who did it after 1984 EC. There a great difference for a matured and immature girl for marriage. If she is immature, she cannot manage the household. If she does not finish her childhood, she may not improve the livelihood of the family. How can it be the same for mature and immature person? That is why the government put the law to protect early marriage.

**I: What about in relation to nutrition?**

**P:** That will be dependent on her livelihood status. There is one proverb “you can eat from your own food-table without asking but you have to ask for eating if it is your mother’s food-table” (ጊባቦኻንያ ሓሊፍካ ጊባቦ ኣዲካኻን ጠይቕካ).

**I: My question is what could be the difference below and above 18 year old marriage in the nutritional status like what you mentioned the difference in relation to the health condition?**

**P:** There will not be a problem if she is above 18 years old. She knows what is important and not important for her. She is also getting married after completing education grade 10. But if she is under 18 years old, she is immature. Her thinking capacity is incomplete and her brain will be divided into many. Finishing your childhood with family and going outside with your childhood in new household life are different. She will not be like that of the matured girl.

**I: So, what could be the reason? Is it the skill that is received from the mother that makes the difference to prepare food or any other reason?**

**P:** There is a difference. When your mind is broad you will know what is important and not important and also you need to know what is good and bad for you. If you are below 18, you may not even know what you talk are good and bad let alone the work that you do. You may talk what is not important and you will not also able to manage the family. So, she will harm herself and the family as well. It is a double burden. But, this is not happening this time.

**I: What could be the reason for the change?**

**P:** In our local area, early marriage is as I told you earlier. Mainly, she is the one losing. She cannot manage a family and she will be affected. Anyways, our government has set a law that protects a girl not to get married before the age of 18 years old and the community has accepted this and there is no one having early marriage.

**I: Is it the law or the community that could make the change?**

**P:** There is a law and then the community accepts and carried out for the effective implementation of the law. The community understands the importance of the law and implements it effectively.

**I: What could be the reason that this community accepted for the great change while it is still happening in other areas?**

**P:** Let me tell you taking myself as an example, if I could have stayed up to 18 years old for marriage, I would be like you. I was married at 11 years old as the promise of my parents to the family of my husband. It was in 1984 EC that I got married and in 1985, my husband was in school at grade 4 and because of my husband I was enabled to write and read and then attend school up to grade five. So, I missed the chance of getting salary and comfort but it was not difficult for me to manage the family. I could have been in comfort and my mind would be broadened but now I become ignorant. This is what can happen but I do not think there will be another problem.

**I: What about increasing spacing between each birth?**

**P:** I give the first birth at 16 years old but there is nothing bad on me. There is nothing that happens because there is God beyond anything though it is good to accept the law of the government. But whether it is death or illness it is at the hand of God. The government is under the God.

**I: It is true that everything is under God, but do you mean that it is not a problem even if it is under 18 years old?**

**P:** No, I do not say it is not a problem this time. They have to get the education. How can they get education after marriage, they will stay ignorant. So, as to me it should be at 18 year old.

**I: What should be done to increase birth spacing?**

**P:** It is good to increase birth spacing because how could it be good to have children every tomorrow? With regard to birth spacing whether you like me or hate me because you know it is good to tell the reality. I have enough children and I was using and now I am also using contraceptive because I am enough. I was giving birth at three years interval and my children were not facing anything bad. But as the government said, there are mothers giving birth at 5, 6, or 7 years interval. But you know, you cannot say this is good and that is bad because as I understand the government telling us to increase the family size is to give care by reducing the family size rather than growing up of children with limited feeding practices by increasing family size but individuals are different in their living styles. For example, as I told earlier, I have irrigation. This irrigation needs a family; it cannot be done only with husband and wife. Otherwise, you will be taken up by glutton. So, I was following short birth spacing; I do not want to lie assuming that you will hate me. As you are here to help us, I have to tell you the reality so that you will also advise me if I am mistaken. As I have children, half of them are irrigating the vegetables and half of them are keeping animals. So, what this mean is that we cannot say there is no needs of child especially for farmers like us, child is very crucial. If there is no shortage of food and if there is work to be done, they have to give birth and be supported. It is only this child and his elder brother that are not involved in the activities otherwise, the others starting from five years old, they are tilling the land. There is no one eating food without working. But increasing the birth spacing is also important because there are individuals that do not have farm land. If this kind people with no land increase the family size without having enough to feed, it will be difficult. But for us, it was important to have children though now I am enough.

**I: Who is promoting the message?**

**P:** The health extension workers in the health post. They are telling me to stop giving birth. They were also telling me before I gave birth two or three children. I told them that you are civil servants and you can limit the number but for me I need children that will help us keeping goats, cattle, and other activities. They told me that we are telling you for your benefit because you may be a feeble woman. I understand that they wanted to help me but there is a difference that I would be benefited if I have children. So, I was continuously giving birth until now.

**I: What do you suggest for those that do not have enough to increase birth spacing?**

**P:** Most of them are using contraceptives but there should be enough education. The second thing is that the beneficiaries should practically apply the education that they are offered by the trainers. They have to be also convinced to accept by themselves rather than applying it by force.

**Section 6: Understanding communication and information sources**

**I: What kind of community conversation or message discuss women’s and adolescent nutrition in this community?**

**P:** Yes, there are but you know the thing is that there are people with different behaviors. For example, when you have 10 women to mobilize the intervention, some of them may accept your idea and those that do not accept will say it is because she has something to get. All may not accept but you need to do to make all people accept it.

**I: So, what kinds of community conversations do women or adolescent nutrition is there in this community?**

**P:** I do not know. I have never been involved in the community conversations. It is the development agents, the networks and other groups that are involved on these issues. We only come to this health post for check-up or similar activities and they do not ask me to participate. We do not have any means of conversation other than coming to this health post for health services.

**I: What are the challenges in promoting the massages to women?**

**P:** There is lack of providing repeated education. And the community has to seriously check-up health status. The other thing is poverty because as a result of poverty, you will face problems related to nutrition due to shortage of food. I do not have other challenges that I can say.

**I: What are the most important messages that you easily accepted?**

**P:** All are important messages, especially check-up of lactating women. Previously, there was no child that does not stay for six months without tasting foods. If a child is baptized, you will be in hurry to give him food. But now, it is understood that it is not important for him and it does not help him to increase his mindset. Now, there is an education that it is the exclusive breastfeeding that makes him to grow-up healthy. I believe that the child will be active if exclusively breastfed. Let alone the brain which is the most important thing, we know how much we were changing their cloths because of diarrhea when they were provided food within six month but we have never considered that the cause of that problem is the food. The other important thing is the advice to care a child and provide him nutritious foods.

**Section 7: Additional remarks**

**I: How can we improve maternal and adolescent nutrition in this community?**

**P:** It is wrong to say ‘we did for what we did not’ and it is also wrong to say ‘we did not for the things that we did’. So far, I do not think there are things that I do not understand. As our heart is divided in to so many things, probably, I may have a gap on explaining otherwise, I do not thing there are thing that I do not understand. Everything is good. I do not have a reason that I want anything to ask you to know as I do not have something that I do not understand.

**I: What about for the future? You were just teaching me; it was you not me. I came here to gather information and to learn from you. So, would you please comment on the things that have been done, what should be improved, what should be done better for the future, and others to improve maternal and adolescent nutrition? It can be from what we discussed or anything that we did not cover during the discussion.**

**P:** As to me, the things that are important for the community are repeated additional educations/awareness like this. We should not sleep assuming that this community is active and is aware of the issues. For the community, though it might not be monthly, there should be education at least once in a year.

**I: So, you are saying the most important thing that should be improved is education.**

P: Yes, it is the education because if there is education, there will be everything.

**I: thank you very much for sharing us the information and time**

**SUMMARY**

**Section 1: common maternal (pregnant women, lactating women and adolescent girls) nutrition problems in the community.**

- The brain activity and health can be different if there is difference in feeding practice but to be short or long is the creation of God. So, someone may be short due to disease but I do not think he will be short because of food.

**Section 2: Barriers to access and utilization of nutrition services**

- As the poor farmers are afraid not to be excluded from safety net, they start taking the fertilizers and apply it on their land and become productive in producing grains and others. As a result, they get enough food to satisfy the households.

**Section 3: Perceived needs of women for relevant services during pregnancy, lactation and adolescence**

- When you give birth to a child and when you loss that much blood from your belly, you have to take foods that can replace the loss.

**Section 4: Other interventions that improve pregnant, lactating and adolescent nutrition**

- In the earlier times, children were highly affected by diseases such as measles and others but now due to vaccination children are not exposed to these kinds of diseases.

**Section 5: Understanding perceptions of age at first birth and birth spacing**

- As I have children, half of them are irrigating the vegetables and half of them are keeping animals. So, what this mean is that we cannot say there is no needs of a child especially for farmers like us.

**Section 6: Understanding communication and information sources**

- Let alone the brain which is the most important thing, we know how much we were changing their cloths because of diarrhea when they were provided food within six month but we have never considered that the cause of that problem is the food.

**Section 7: Additional remarks**

- There should be repeated education because if there is education, there will be everything.
