# Supplementary material for: Barriers to utilize nutrition interventions among lactating women in rural communities of Tigray, northern Ethiopia: An exploratory study
Source: PLoS One. 2021 Apr 30;16(4):e0250696. doi: 10.1371/journal.pone.0250696 (PMC8087028; doi:10.1371/journal.pone.0250696)
Supplement: S2 File — (ZIP) [file pone.0250696.s002.zip › S2_File.Doc/Lacatating women_IDI & FGD/141_FGD_Lactating Women_Hakfen kebele_Medabay Zana Wored.docx]

OPERATIONAL RESEARCH ON ADOLESCENT AND MATERNAL NOTRTION IN NOTHERN ETHIOPIA

TOOL B

FGD GUIDE FOR LACTATING WOMEN

PRINCIPAL INVESTIGATOR: DR. AFEWORK MULUGETA

DATA COLLECTION

FGD REPORT FOR LACTATING WOMEN IN HAKFEN KEBELLE, MEDABAY ZANA WOREDA, NORTH WESTERN ZONE, TIGRAY, NORTHERN ETHIOPIA

SELEKLEKA, ETHIOPIA

NOV 29, 2017

**Information sheet and consent**

I: Good After noon. My name is Yasin Jemal. I am from Mekelle University. We are doing research on the factors that influence the nutrition of mothers and adolescent girls in collaboration with the Regional Health Bureau and UNICEF. Your participation is valuable. The information that you tell us will not be shared to others. However, the information will be recorded and used to improve nutrition programs and services for women and adolescents in the region and in the country. We have several questions to ask you that we have prepared in advance and we will ask you all to say what you think about each question. Ensuring privacy of everyone here not to speak what we discuss outside of this group is strictly not allowed. The discussion will take 1-2 hours. Do you have any questions before we begin? If you have any concerns or questions as we proceed please feel free to let me know. If it is alright with all of you, we will turn on the tape recorder now.

Participants: Ok, we agreed to participate

= = = = = = = = = = = = = = = = = = = = = = = = = = = = = = = = = = = = = = = = = = = = = = = =

**Section A: Interview details**

| Zone | North Western zone |
| --- | --- |
| Woreda | Medabay Zana |
| Kebelle | Hakfen |
| Facilitator name | Yasin Jemal |
| Date of discussion | Nov 21, 2017 |
| Discussion start time | 03:08 PM |
| Discussion end time | 4:32 PM |

= = = = = = = = = = = = = = = = = = = = = = = = = = = = = = = = = = = = = = = = = = = = = = = =

**Section B: Socio-demographic Information**

| S.No | Name of FGD Participant | Code | Age | Marital status | Education level | Occupation |
| --- | --- | --- | --- | --- | --- | --- |
| 01 | Sindayo Tikabo | P_1_ | 19 | Married | 5^th^ Grade | Farmer |
| 02 | Beriha Matyos | P_2_ | 24 | Married | 8^th^ Grade | Farmer |
| 03 | Abeba Leul | P_3_ | 22 | Married | 4^th^ Grade | Farmer |
| 04 | Letay Guesh | P_4_ | 20 | Married | 10^th^ Grade | Farmer |
| 05 | Alem Negash | P_5_ | 22 | Married | 4^th^ Grade | Farmer |
| 06 | Haymanot G/Hiwot | P_6_ | 25 | Married | 9^th^ Grade | Farmer |

= = = = = = = = = = = = = = = = = = = = = = = = = = = = = = = = = = = = = = = = = = = = = = = =

**Section 1: Common lactating women nutrition problems in the community.**

I: What do Lactating women do to stay healthy in this community?

P_4_: For the question what they do? They should eat balanced diets.

I: Okay, what else?

P_4_: They should keep their hygiene and by keeping their own hygiene, they should also keep their children’s hygiene.

I: What else? What other cleanliness

P_4_: They should keep clean their clothes.

I: What else? What other cleanliness

P_4_: They should also fulfill cleanliness of environmental in general.

I: Okay, any additional idea? What do lactating women do?

P_3_: Their food should be balanced diet and then they should keep clean their cloth and their environment. They should also visit health facility for medical checkups and vaccinate their children. In addition, they should also wash and care their children.

I: What else?

P_2_: They should have medical checkups during post natal period. They should also feed their children in hygienic way (by keeping their hygiene). In addition, they should eat balanced diet.

I: Okay. What else?

P_6_: First, they should keep their personal hygiene. They should also wash their hands using water and soap before they breastfeed their children and the child should breastfeed on regularly. The other is to keep their environmental sanitation.

I: Good. Do you have something to add? Or that you think left?

(All participants were silent)

I: Okay. In your opinion, what are the common nutrition problems in the community for lactating women?

P_4_: From what?

I: From nutrition problems? That is problems caused by lack proper nutrition.

P_4_: Malaria is commonly seen.

I: Why malaria is commonly seen?

P_4_: Due to lack of eating balanced diet, lack of having good beds such as unclean bed, being wet, lack of rooms as well as lack of money.

I: Okay, what else/

P_2_: If the mother is not eating balanced diet, the child should will be affected.

I: What happened to the child?

P_2_: Becomes small and emaciated.

I: Okay, P_3_?

P_3_: Yes. The mother may also reduce the amount of breast milk production if she didn’t get balanced diet.

I: So, what common nutrition problems are seen in this community?

P_3_: The children become sick and worms develop in their stomach due to lack of sanitation. Thus, if there is no balanced diet and no proper care of children in hygienic way, the children will be getting sick.

I: Okay. What are the common nutrition problems in lactating women caused by lack of proper nutrition? Problems caused due to lack of eating balanced diet?

P_6_: Goiter

I: Okay, what else?

(All participants were silent)

I: In your opinion, how do you explain the risk of malnutrition for lactating women?

P_4_: Lack of land (farmland) and lack of education since the public is not educated. These are the risks.

I: Okay. Any other?

P_3_: For farmers, it is lack of land (farmland). Currently, we don’t have farmland and we are in worry since there is scarcity of land.

I: Why scarcity of land is the risk of malnutrition?

P_4_: Since we are the late generation, the land is occupied by the old generation and it is not transferred or given to us. The land will be given or transferred only if the land owner get died. Even the owner got died, his children and grandchildren says the land belongs to us. Thus, there is land scarcity and the youth is worried/concerned. The other is not having education (lack of education) or they stop their education.

I: Okay, P_6_? What are the risk of malnutrition?

P_6_: Having large family size. Thus, they should control births.

I: Oka, what else? Other risk of malnutrition?

(The participants were silent)

I: Okay, P_3_?

P_3_: It is having large family size rather it is land scarcity.

I: P_6_ said if we have many births, it will affect the economy

P_3_: Yes, that is right. It affects the economy.

I: What else that you think not mentioned?

P_3_: That is it.

I: Good. Do you think lactating women are especially affected by malnutrition?

P_4_: Yes

I: Why?

P_4_: Due to the reasons we have mentioned.

I: What are these? Tell me?

P_4_: Land scarcity/lack of land

I: Okay, since there is land scarcity.

P_4_: Yes, there is land scarcity…..laughing…..

I: Okay

P_4_: Yeah, since there is land scarcity. …Smiling…laughing… and as P_6_ said there is no birth control. The other problem is not getting married at the right age rather get married while underage and then giving births. …Laughing….so, these are that affects us.

I: Okay, P_1_?

P_1_: They are like that.

I: Say your own? No problem, add your own?

P_1_: …Silent…

I: There is shortage of hand

I: What does it mean shortage of hand?

P_1_: Shortage of food at home or not getting food at home.

I: Okay, P_4_?

P_4_: We seek persons who are better like university graduates or 10^th^ grade complete to be assigned (in the meanwhile she is smiling and laughing) in order to teach the elders since they don’t understand, so elders may change if such persons are assigned otherwise they never be changed.

I: What else? Okay, P_5_ say something and let is your voice?

P_5_: As they said there is shortage of food, lack of land and even if there is land, there is lack of oxen and other. Thus, leave alone there is shortage of food for lactating women, it may also affect the children though you have food for children.

I: Okay, good. How sever are the nutrition related problems that you have mentioned among lactating women in this community?

P_4_: They may cause diseases and then cause death since they do not get medication/treatment on time when they get sick and when you get sick, they need food but there is no food so that it leads to death.

I: Okay what else? Okay, P_2_?

P_2_: Wayyy! They may be exposed to diseases due to lack of economy and if they don’t have money for medication, they will be exposed to diseases.

I: Okay. Do you think that lactating women in this community are suffering from micronutrient deficiencies such as anemia, night blindness, goiter and others?

(Some said there are)

I: Okay, let’s talk one by one. P_5_, are there these problems?

P_5_: There are.

I: Which ones are present from that I mentioned?

P_5_: Night blindness and anemia is the main.

I: Are they seen among lactating women?

P_5_: Yes, there are.

I: What about goiter?

(A participant said yes)

P_5_: Ayyy, yes there is also goiter.

I: What is their causes?

P_4_: Due to lack of vitamin. You know how? Since, the persons don’t eat varieties of foods…..Laughing…since the public eat only shiro (local sauce). They don’t have the capacity to change food, what they get if used for shiro. …Smiling and laughing.

I: What else? P_3_?

P_3_: About what?

I: What are the causes of goiter, anemia and night blindness? Reasons of deficiencies?

P_3_: Yes, it is due to lack of proper food and whatever it is, they are caused due to lack of food.

I: Okay, P_5_?

P_5_: Since iron is not present in cereals, she should eat enjera made of Taff and take soup and the likes and not to be exposed to anemia, lactating mothers should drink/take any fluid//liquid foods but due to shortage of money (lack of capacity), they didn’t use.

I: Okay, any other?

(All participants were silent)

I: What do you think on why women/girls in this community would not increase their height proportional to their age?

P_5_: Due to lack of proper nutrition. If pregnant women didn’t eat properly as well as lactating women, they will not properly breastfed their children due to shortage of food. Thus, the children will become short, thin/emaciated and the children may face any problems.

I: Okay, good. Any additional?

P_2_: Since there is child marriage and then giving many births closely. Not using birth control (contraceptive) due to backward tradition.

I: Do women/girls in this community increase their weight proportional to their age?

P_4_: No

I: Why women/girls do not have weight proportional to their age?

P_4_: Due to lack of food. They do not have food and have scarcity and since they give many births closely. In addition, they are not educated and do not know.

I: Okay, what else. Any additional?

(All were silent)

I: Good. What diet related non-communicable diseases are common among lactating women in this community?

P_4_: Like malaria.

I: Malaria is communicable diseases. Let me explain it. Problems like diabetes and blood pressure. Are there these problems?

P_4_: Is that after we ate the food?

I: Yes, non-communicable diseases caused due to lack of proper nutrition. Are there diabetes and blood pressure among lactating women?

P_2_: Is that communicable or non-communicable?

I: Non-communicable such as that ones I mentioned such as diabetes is non-communicable and blood pressure is non-communicable. Are there these diseases?

P_4_: I don’t think blood pressure among lactating women but may be in elders.

I: Is there a situation when lactating women suffers from shortage of food?

P_4_: Why not? Yes, they are affected.

I: Why they are affected?

P_4_: Since there is shortage of food.

I: What causes that?

P_4_: The shortage of food?

I: Yes

P_4_: We were saying due to land scarcity, lack of education and due to large family size and the family again do not learn.

I: Oaky, P_6_?

P_6_: What was the question?

I: Is there a situation when lactating women suffers from shortage of food?

P_6_: I said they are not affected this much.

I: Even in one? If there is, there is. If there is no, there is no.

P_6_: There is no

I: Okay. P_6_ said there is no but P_4_ said there is shortage of food. Okay, P_3_/

P_3_: What do you say about?

I: Is there a situation when lactating women suffers from shortage of food?

P_3_: Yes, there is. They may focus on activities and may not eat

I: But is there a situation that lactating women do not eat due to shortage of food?

P_3_: Yes, there is and they face

I: What is the reason?

P_3_: Not getting food.

I: What is the cause of that?

P_3_: Lack of land and low production such as Taff.

I: Okay, P_4_?

P_4_: P_3_ has already mentioned all.

I: Okay. How frequent does it happen? Okay, P_2_?

P_5_: They eat 2 times a day since there is shortage of food. However, lactating mother has to eat always but she cannot not eat due to shortage of food and 2 times a day is not sufficient.

I: How many times she has to eat?

P_5_: If possible up to 4 times. Even what is that?

(The participants were laughing and one of the participants said she is right)

I: Okay, what other problems do lactating women in this community suffer from? I mean problems that lactating women suffering from in this community from your observation?

P_5_: Isn’t that lactating woman can protect herself if she care during pregnancy so that she can give birth safely and care her child to grow?

I: Ehhhh…I am shaking my head...

(Some participants are laughing since they are impressed by P_5_ expression)

P_5_: If she didn’t use health service (ANC service) and didn’t care the child during pregnancy, how she can give healthy baby? Thus, lactating mother should eat and a pregnant women should also use the services. Any ways, the main thing that affects us is that since we don’t eat during pregnancy due to shortage of food (as we face shortage of food), we face difficulties/problems even during labor (delivery) and it may also lead to death or it may cause problems like anemia.

I: Good.

**Section 2: Barriers to access and utilization of nutrition services**

I: What kinds of nutrition interventions are in place to improve health of the lactating women in this woreda?

P_5_: Is that not to get sick?

I: Yes.

P_5_: In our Kebelle?

P_5_: The health workers give us ITN and they come to our Kebelle and taught/advised us to keep our environmental sanitation.

I: What else? Tell me other services or interventions you get?

P_5_: Yes, the health workers also advised us to eat balanced diet. They also follow us and see us in good manner.

I: Good. What else? Other services/interventions you receive? Okay, P_6_?

P_6_: If we have anemia, the health workers give us medicines/drugs. They also gives ITN especially if we are pregnant women.

I: What else?

P_2_: They (the health workers) also advised us to have medical checkups

I: Any other? P_3_:

P_3_: Yes, the health workers advised us to visit health facility and have medical examination/checkups, and advised us to keep our sanitation/hygiene.

I: Okay, P_6_?

P_6_: They also give special support to those who have evidence/paper for a year

I: What type of support?

P_6_: Isn’t there support to a family member whom get sick in order to get free treatment for a year by paying 140 Ethiopian Birr. So, there is also such service.

I: Okay, P_1_?

P_1_: It is like that

I: Okay. Where do you get these services/interventions?

P_4_: In Kebelle

I: Where in Kebelle? Is that in Health post or health center?

P_4_: Health center

I: Okay, P_5_?

P_5_: Here in this kebelle and showing me the health post

I: Okay, who provide you these interventions/services?

P_5_: Mr X (she mentioned the name of the person but do not written here for the sake of ethical issue)

I: Are they health workers or agriculture workers?

P_5_: Health workers

I: What services do the health workers provide you when you visit the health facility?

P_4_: There is nothing except drugs/tablets.

I: What else?

P_3_: They give us drugs

I: Others that are not mentioned?

P_3_: They (the health workers) measured us our weight. They taught and advised us to eat this and this foods. They also give us ITNs not to bitten by mosquitoes and they make follow ups.

I: Good, what any other services you get?

(All become silent)

I: Okay, what would it help you to your health, for example, when they measured your weight, gave you drugs and advised you to use ITNs?

P_5_: Weyleke (a kind of surprise to express felling). To prevent anemia if we have and to prevent any problems in case we have. Thus, they follow us.

I: So, what would it help you?

P_5_: To our health. It help us to improve our health as well as to improve the health of our children too.

I: Is that important to the baby?

(All participants said yes, it is important)

I: What about it benefits to the baby? Okay, P_3_.

P_3_: It helps the baby not to be bitten by mosquito so that not get sick with malaria. And not to face the child any problems or health consequences, they make follow up.

I: Okay, P_5_/

P_5_: To the baby?

I: Yes.

P_5_: To keep him clean, use ITN not to be bitten by mosquito and not to expose/acquire malaria,

I: Do you they give you advice on the need to get extra meal?

P_5_: Yes.

I: How?

P_5_: For example, if you have anemia, they advised you to take like soup made of Red Taff with sugar, to eat lettuce and salad. So, we also use that one.

I: What else?

P_4_: That is. Nothing else.

I: Okay, P_6_? How they advise you to take extra meal?

P_6_: To eat foods prepared from varieties of cereals, vegetables and fruits by mixing them up and to feed 2 or 3 times the child in addition to breast milk.

I: Okay, any other?

(All participants were silent)

I: Do the health workers give you advice on the need to use iodized salt

(The participants said yes)

I: For what can be used iodized salt? Okay, P_1_?

P_1_: To prevent goiter

I: Okay, what else? For what can be used?

P_3_: For mental development of children and helps to increase the height of children. So, if they use iodized salt, it is good.

I: Do lactating women getting advice on nutrition sensitive agriculture such as home gardening?

P_5_: Yes, they are given advice

I: What they tell you to do?

P_5_: To produce/cultivate vegetables like cabbage, lettuce, salad and tomato. They also advised us to feed children with 7 types of foods in order to give them strength.

I: Okay P_4_?

P_4_: Though they said like that, it is not practical. You know why? Because there is lack/shortage of water. She is smiling….

I: You always smiling while you speak. Interesting!

(They all laughing)

I: Okay, P_2_?

P_2_: They taught and advised us to dig water wells (E’la) to produce vegetables. Thus, they always teach us.

I: What they advise you to produce?

P_2_: Like lettuce, salad, tomato, onion and other different types.

I: Good. Do they make lactating women get involved in safety net programs? Okay P_4_.

P_4_: Woa. There is no for lactating women but they said to poor or elders and so on. Thus, there is no support for lactating women.

I: Okay, P_3_?

P_3_: Yes, of course. It is like that. Support about safety net? No no (ayey) they don’t support us.

I: Who provide you advice to produce vegetables in home gardening?

P_5_: Agriculture

I: Any other? Is there another one?

(They all silent)

I: What else interventions related to this? I mean other than home gardening and safety net programs.

P_5_: Additional what? Is that irrigation activities?

I: It might be irrigation activities or food support, so what else? Okay P_5_?

P_5_: We don’t have any other interventions but if there is water wells, they advised us to produce vegetables like lettuce, salad and told us to get loans but we didn’t take loans because of lack of capacity.

I: Okay, P_4_?

P_4_: There is no support. Woa.

I: Okay, good. Do lactating women getting advice on water, sanitation and hygiene services? Do they give you advice?

P_3_: Yes, they told us to drink tap water.

I: What they tell you to add if there is no tap water?

P_3_: To add iodized salt.

I: Iodized salt?

P_3_: Ehhh…what was the name. The health workers told us. Please bring/give me the name (Please remind me the name)…

I: Wuha agar?

P_3_: Yes, yes, yes, that is right/correct. They told us to add wuha agar.

I: What about the tablet?

P_3_: Yes, they give us.

I: Okay, P_4_?

P_4_: Though they said, it is not practical/implemented. Yes, they told us like that but since there is economic problem………not completed the sentences…

I: Do you buy and use the water chemical (wuha agar)?

P_4_: Which one?

I: The water chemical used for treatment?

P_4_: Yes, of course.

I: You buy and use?

P_4_: No, it is distributed by support

I: So, do they give you advice on how to keep clean water, sanitation and hygiene?

P_2_: Yes, they give us. They also monitor/follow the cleanliness of the borehole water developed in our Kebelle and they add water chemicals and we drink up to 6 months.

I: Okay, P_3_?

P_3_: Yes, it is like.

I: Okay, P_6_?

P_6_: They told us to take the water chemical during summer. However, if don’t take, we will boil the water and use.

I: Good, any other?

(They all were silent)

I: Okay, do you think it is necessary advice on water, sanitation and hygiene service?

(Some of the participants said yes, it is necessary)

I: Okay, let me start from P_1_?

P_1_: Yes, it is necessary

I: Why it is necessary? For what it would help us?

P_1_: For cleanliness and to prevent diseases

I: Okay, P_3_?

P_3_: It would help us not to expose to diseases

I: Okay, P_5_?

P_5_: To get rid of from worms

I: What else?

P_5_: And to be free from malaria and other different diseases

I: Okay, P_6_?

P_6_: It is like that

I: Okay, P_4_?

P_4_: It is like that

I: Okay, good. Is there malaria in this community?

P_4_: Laughing…Yes, there is

(Here, P_2_ and P_3_ also said, yes, there is)

I: What else the rest of you?

P_6_: Yes, there is malaria but since we use ITN, we are not affected.

I: So you get the service?

P_6_: Yes

I: Who give you the service?

P_6_: Mr X (She mentioned the name of the person but not written for the sake of ethical issue)

(Other participants were also answering similar)

I: Who are they? Are they health workers or agriculture workers?

(All participants said health extension worker(s)

I: Okay, good. Are lactating women getting deworming services?

P_2_: Yes, we are given. They also give us drugs for trachoma.

I: Okay, P_6_?

P_6_: It is additional, they gave us.

I: Okay, good. Who provides the service?

P_6_: Health post workers (health extension workers)

I: Is it necessary for lactating women?

P_6_, P_3_ and P_4_ said, yes, it is necessary. P_1_ and P_5_ also said the same.

I: Why it is necessary?

P_3_: Not to be exposed to diseases

I: Okay, P_6_?

P_6_: To be safe our health

I: What else?

P_4_ and P_5_ also said it is like that (the same)

I: Okay, do you think that lactating women should need to be targeted through Targeted supplementary feeding (TSF) in this community?

P_3_: Yes, we need.

(Here, P_4_ is laughing)

I: Why?

P_3_: Since there is problem, by feeding that a mother will get benefit to herself and her child.

I: Okay, is it necessary?

P_3_: Yes, it is necessary

I: Do they target/support you?

P_3_: No, they don’t support us

(Here, others also complement P_3_)

I: Okay, P_4_?

P_4_: No support

I: Why?

P_4_: I don’t know whether it is shortage (lack of capacity) or not, I don’t understand the reasosn.

I: Okay, P_1_?

P_1_: It is like that

I: Okay, Never ever given corn soya support here?

(All participants were talking and saying not given)

P_5_ and P_4_ said never given in Hakfen, we don’t know it.

I: What do you say P_2_?

P_2_: Previously it was coming but it stop before 3 years and we don’t know whether they changed to other or sold it.

I: Okay, P_3_?

P_3_: We don’t know it

I: Okay, P_4_?

P_4_: We don’t know it

I: Which of the interventions listed above are most important for lactating women?

P_6_: Insecticide treated bed nets (ITNs)

I: What else?

P_6_: The other is….what we called the one added to water….I forget it the name.

I: Wuha agar?

P_6_: That is also important and given to us to use

I: What else P_5_?

P_5_: Like food/diversified food is most important

I: What else?

P_5_: The other is also advice on water, sanitation and hygiene, ITN use for mosquito control,

I: Okay, what else? P_4_?

P_4_: The most important is provision of clean water

I: What else?

P_4_: In addition to that, side by side, ITN is most important since it helps us keep our health. Thus, it is necessary.

I: What else?

P_2_: Clean water is most important

I: What else?

P_2_: Balanced diet

I: Okay, P_3_?

P_3_: It is like that. No, additional.

I: Okay, P_1_?

P_1_: It is as they have been said it and also utilizing iodized salt is important.

I: What are the barriers in the implementation of these nutrition services?

P_5_: Lack of land/ land scarcity

I: What else?

P_4_: Lack of water

I: What else?

P_6_: I don’t understand the question?

I: What are the barriers in the implementation of these nutrition services?

P_6_: There is no barrier

(Here, P_4_ is laughing…..and said she don’t understand the question)

I: Okay, P_3_?

P_3_: Having lack of land. We didn’t do it by digging water well since we don’t have land. Thus, we did do it.

I: Okay P_2_?

P_2_: It is shortage of land (land scarcity)

I: Okay, what else other than land scarcity? For example, there may be health service but having land scarcity may not be a barrier to use the health service but it may be a barrier to shortage of food. So, let say if you are asked to get treatment while there is health center, what is the barrier not to use the service?

P_4_: It is lack of money and nothing else.

I: What else? Such as not to get ITN, not to use iodized salt, not to keep your personal hygiene, and so on. What are the barriers?

P_4_: lack of money since the providers asks for money.

I: Okay, any other?

P_4_: Lack of education/awareness

I: Any other?

P_2_: Lack of economy

I: P_1_?

P_1_: It is like that

**Section 3: Perceived needs of women for relevant services**

I: What special things do lactating women need in your community?

P_5_: Yes, they advised us to visit health facility, take different foods such as vegetables, to prepare porridge and eat, not to give food the child before 6 months but after 6 months to feed/give them different types of food.

I: Okay, P_4_?

P_4_: What is that?

I: What special things do lactating women need in your community such as visiting health facility, taking extra meal, taking rest and taking supplements?

P_4_: Yes, they are advised

I: Do you need?

P_4_: Yes, we need but no due to economy. Any ways, they told us.

I: What else special things need to do?

P_6_: We need to eat at least 4 times a day if we are lactating mother

I: What else?

P_6_: We need to keep clean our water, sanitation and hygiene as well as to keep our children. Thus, they give us advice.

I: What should be the role of a husband to improve nutrition for lactating women?

P_4_: It is low

I: Why it is low?

P_4_: Since they didn’t learn and not educated. We also don’t learn.

I: Okay, any other P_6_?

P_6_: Those educated, they gave us support even when we are in postmortem period. Thus, they slaughter goat and provide us meat. And husbands think that lactating women do not get hungry. Thus, they brought us what they can by working up and down.

I: What else P_5_?

P_6_: They also make engaged us to work in easy activities

I: Do they really practice it?

P_6_: Yes, but not all

(Here, the participants are laughing)

I: Okay, P_5_?

P_5_: Woa. Due to lack of awareness, they didn’t practice. Isn’t it? Weyleke!

(All participants are laughing)

Here, P_5_ said, you know why? They see us in safety net I mean they follow us up to 6 AM and then after they don’t follow us.

I: Okay, P_2_?

P_2_: Lack of capacity or economy but we can eat what is available.

I: Okay, P_3_?

P_3_: Laughing….it is like that. Laughing…like that. No one prevents for what is available but there is lack of capacity/economy.

(They all laughing)

I: Do lactating women in this community typically change their diets during lactation?

P_6_: Till the child make 6 months, we can change but after that we will be like our family. Thus, before 6 months, we can change diets.

I: What do you eat?

P_4_: Like soup, porridge and others

I: Okay, P_4_ do you have additional?

P_4_: No, that is it.

I: Okay, good. What foods are recommended for lactating women?

P_4_: Meat and the other is…..laughing…to eat enjera (local food) made of Taff, and cereals. Thus, such thing is needed.

I: What else?

P_4_: The other is to drink alcohol

I: What kind of alcohol?

(Almost all participants were laughing)

P_4_: Laughing…..if possible…laughing...like soft drinks since it helps to produce breast milk. So, it is also preferable.

I: Okay, P_5_?

P_5_: To eat by changing foods

I: What type of food?

P_5_: As P_4_ said to eat meat, soft drinks, vegetable and fruits, and cereals that can produce breast milk

I: What affects lactating women’s diet during lactation as to be recommended/

P_6_: Lack of economy

I: What else?

P_4_: Economy and diseases that is the health status of the women.

I: What else?

P_4_: The others are lack of money and lack of land.

I: What else?

P_4_: Lack of education/awareness on how to prepare

I: Are there gender disparities in women’s diets during lactation?

P_4_: Yes, there are

I: How?

P_4_: How? Laughing…For example, if meat is cooked/prepared, it is given to husband. Laughing…Best foods are given to husband.

(Others were also laughing)

I: Why it is like that?

P_4_: Wayyy…I don’t know

I: Okay, P_2_?

P_2_: Currently, there is equality

(All of us were laughing)

I: What else any disparities like she has to eat this or since you are lactating, you shouldn’t eat this or husband should eat this, or like since you are women, you shouldn’t eat this or I should eat first but you should eat later, what disparities are present?

P_3_: In household, there is no. Laughing…there is equality…Laughing…Any ways what is available is eaten together. Laughing…

(Others were also laughing)

I: Okay, P_5_?

P_5_: Due to lack of awareness she can wait her husband for food rather than eating by reducing part of the food especially when meat or shiro is prepared.

I: Is that due to lack of awareness or gender disparity and since a husband must eat

P_4_: It is the existed problem and it is said a women should not eat before husband. It is like a culture.

I: Best foods are also given to husband, right?

P_4_: Yes

I: Okay, what else P_5_?

P_5_: It is like that.

**Section 4: Other interventions that improve lactating nutrition**

I: Have you ever gone for nutrition screening during community health days?

P_6_: Yes, I have gone especially during pregnancy starting from 4^th^ month up to delivery, we went to health facility and check our weight.

I: Good. Any other?

P_5_: We participate as P_6_ said.

I: Have you ever gone?

P_5_: Yes, I have gone

I: Why?

P_5_: To check and keep my health

I: Okay, P_3_?

P_3_: Yes, I have gone

I: Why?

P_3_: Not to get sick, not to get exposed to diseases, not to be emaciated and not to suffer from lack of blood. Thus, we went to denote and get measured. So, they check/monitor our diseases and others.

I: Okay, P_6_?

P_6_: They also check the positioning of our baby whether it is in normal way or not. They also give us vaccination that helps the baby in the womb.

I: What else P_2_?

P_2_: We went during pregnancy.

I: What about after pregnancy?

P_2_: We went for vaccination of the child

I: What about P_1_?

P_1_: We have gone during pregnancy and they examined us

I: Where?

P_6_: Here in the health post and health center. We also deliver in health center and there is no home delivery

I: Where do you go?

P_5_: First we checked at health post and then we went to health center to check any concerns

I: Okay, P_4_?

P_4_: It is like that.

I: Who provide you?

(All participants said health workers)

I: Do you think community health days would have benefits for the lactating women?

P_5_: Yes

I: How?

P_5_: To know whether the baby is in normal position or not, to know whether we have anemia or not, to have follow up for medical checkups. Thus, the health workers told us this so that we also apply it.

I: What else P_4_?

P_4_: It is like what P_5_ said it. It is know the positioning of the baby and to know our health status.

I: What about routine service delivery?

P_4_: Who would go? If you only get sick

I: Okay, P_2_?

P_2_: During Antenatal care every, we went every month

I: Okay, P_5_?

P_5_: It is like that

I: Good. What are the challenges to attending community health days for lactating women?

P_4_: Since all people are not educated and there is lack of awareness, we spent the time at home. In addition, if you are with elders, they said, why you go to health facility, health facility is nothing unless Saintly Mary come herself. Thus, it is due to the public is not educated and since the elders do not understand. However, if it is emergency/urgent, you will go quickly. Otherwise, you will not go if you are good.

I: Good, okay P_2_?

P_2_: Since there is backward tradition

I: What else?

(All participants were silent)

I: What are the challenges to attending community health days for lactating women?

P_5_: Since we have lack of awareness

I: What else?

P_5_: Woa… Since we focus on our routine activity and thereby to solve our household problems

I: What else?

P_5_: That is it.

I: Okay, P_6_ what are the barriers not to attend in community health days?

P_6_: No barriers, however, in case if it is production season, we may be busy of collecting cereals/products. The other may be routine home activity otherwise we don’t have any barriers.

I: Okay, P_3_?

P_3_: It is like that

I: Okay, P_1_?

P_1_: the barrier is home activity

I: Okay, what about the challenges to accessing the routine service delivery?

P_6_: Being a farmer

I: What else?

P_4_: Lack of money though there is support starting from this year by paying 140 Ethiopian Birr. Thus, you will pay once and help you get service for the whole year. However, since this wasn’t present before, if you get sick, you will suffer. Thus, due to lack of money, you will not get medication or you will be at home. So, you will be at bed till it takes you to death or become very severe.

I: What else?

(They all become silent)

I: Do you think lactating women needs to be targets for supplementary foods?

P_6_: Yes

I: Why?

P_6_: To those who have shortage of food, it may help them

I: Okay, P_3_?

P_3_: Yes, it is. They have to be targeted/supported.

I: Okay, P_5_?

P_5_: Yes, it is needed.

I: Why?

P_5_: If lactating women get measured and become thin/emaciated, they have to supported/targeted with supplementary foods. Thus, they should be given food support.

I: Okay, P_4_?

P_4_: Yes, it is needed because the food contains eggs, vitamins, sugar and other. Thus, it is very important for children to improve their weight.

I: Okay, P_2_?

P_2_: P_5_ has already mentioned

I: Okay, P_3_?

P_3_: It is like that

I: Are lactating women beneficiaries of the soft conditionality of the productive safety net program, PSNP?

P_4_: I don’t know why lactating women are not beneficiaries. Is that due to high population?

I: Okay, P_6_?

P_6_: Most of the time it is to those who are weak, elders and poor, not for the sake of lactating women

I: Okay, P_1_?

P_1_: It is like that. It is given to elders, weak and poor, not for lactating women

I: Okay, P_3_:

P_3_: It doesn’t come for support of lactating women since the providers said Mr X or Mrx Y has this much, and doesn’t belong him/her and the other may be due to partiality. Thus, they will not give us food support.

I: Do women in this community know why they are targets of the program?

P_4_: Yes

I: P_5_, what do you say?

P_5_: Yes, I know.

I: Okay, P_2_?

P_2_: Yes, we know but there is pressure

I: How do you know you are targets of the program?

(Here, P_4_ is laughing)

P_4_: How do you know you are targets?

I: Yes. How do you know you are targets?

P_4_: Is that to have supplementary food support?

I: Yes.

P_4_: By education

I: You will not say me that I have learnt it in education, do you mean you hear it

P_4_: No, it is not like that but there are guidelines coming down

I: Okay, P_2_?

P_2_: We know since it says those who have no land should be supported

I: Okay, P_3_?

P_3_: It is said like that but they do not support lactating women who are poor.

**Section 5: Understanding perceptions of age at first birth and birth spacing**

I: Do you think delaying the age at first birth to after 18 is better for the health of the women?

P_4_: Yes, it is.

I: How?

P_4_: It helps her to become physically good, becomes strong and mature mentally.

I: What else?

P_6_: It helps her to get ready

I: What else?

P_1_: It is like that. It helps her to become physically look good.

I: What else?

I: Good, what about to the baby?

P_6_: Yes, it is important

I: How?

P_6_: To provide food properly so that it helps the child to grow in good condition

I: What else? Okay, P_5_?

P_5_: It is like that

I: Okay, P_4_?

P_4_: It helps the child to grow in a good condition since the mother thinks and analysis properly.

I: Any other?

(All become silent)

I: Does this delay would have a benefit to the nutritional status of the women?

P_4_: Yes, it benefits.

I: How?

P_4_: The woman will not be affected and looks good.

I: Okay, P_2_?

P_2_: The woman will not be exposed to diseases. P_4_: also added the woman will not exposed to diseases.

I: Do you think this message is being promoted in the community?

P_4_: Though it is promoted, it is not given in regular basis but you may hear the message bypass

I: Who are working on it?

P_6_: When we have meeting with women development army, the women development army taught us many lessons. For example, they taught to have follow up in order to delivery properly, to follow the positioning of the baby, and to have birth spacing at least 4 years.

I: What else? Okay, P_4_?

P_4_: It is like that and we may participate in the meeting but others are at home and they may not be informed and hear the message since they do not come to the meeting and are busy with household activities. Thus, no one tells them what is discussed in the meeting. Therefore, it will be good if it is told in churches to disseminate the information.

I: But, who are working on it?

P_4_: Women development army (WDA) and it is told in meeting with WDA. You will also get the message if you only attend the meeting otherwise you will not get.

I: Who else P_5_?

P_5_: Health workers

I: How do they promote?

P_5_: Weyleke! They said if you get married at 18, don’t give birth till you become 20 or 21 years but they said give birth when you fulfill/get the required ones and after you become physically strong.

I: In your opinion, what does the reaction of the community looks like to the promotion? Why?

P_4_: This is understood and no one in urban didn’t heard. It is promoted every morning and even it is promoted to students too.

I: Okay, P_6_?

P_6_: Especially what WDAs taught and advised us, it helps us to use family planning service. Thus, we accept what WDA told us.

I: What do you tell us P_2_ how the community reacts to the messages?

P_2_: Most public didn’t accept the message since they are illiterate. However, there is variation at individual level that is some may accept while others may not and encourage to give births closely.

I: In your opinion, how could this message be better promoted?

P_6_: Teaching the importance of using family planning and increasing birth spacing as this is helpful to provide balanced diet and clothes to the children. Advising not to have more than 4 or 5 children.

I: What else? How this message be better promoted?

P_4_: By involving teachers since they are assigned and working in each Kebelle. In addition, to participate students.

I: Okay. Who should be involved?

P_4_: Teachers and students. Since students also teach their parents.

I: Who else?

P_4_: And also health workers in health centers/posts

I: Any other? Additional?

(All become silent)

I: Good. Can you think of any other opportunities for promoting birth spacing?

P_2_: Family planning service

I: What else?

P_4_: We should also need to have awareness though there is family planning service and they please use family planning.

I: What else other opportunities to promote birth spacing?

P_5_: To know her menstruation cycle and then by identifying some days, she can sleep with her husband

I: Any other?

(All become silent)

I: How many years do you think the gap should be between successive births for women?

P_4_: 7 years

I: Why?

P_4_: Since it helps you to care your child till the child starts education. After that you can add but you should care the child to grow in a good condition so that to make reach the child at the highest level

I: What about shorter one?

P_4_: It should be 4 years

I: Why?

P_4_: Since our public did not give birth less than that

I: Okay, any other?

P_6_: It is like that. The maximum 7 years and the shorter 4 years

I: Why 7 years and 4 years?

P_6_: if you give births in number of years, the child will not face shortage of food and gets balanced diet, and the mother also gets time to rest that is from always caring children and from being pregnant.

I; What else P_5_?

P_5_: It is like that as they said it.

I: How many years? Tell me your own?

P_5_: It varies based on their attitude

I: So, tell me your own?

P_5_: It should be 5 years maximum and 3 years minimum.

I: Why?

P_5_: 5 years is good to care the child not to get sick but if she wants to give birth after 5 years, she may face delivery problems. However, if it is 3 years, her uterus is open and she doesn’t face any problem. Therefore, above 5 years is long and she may have operation (surgery) since she is late to give birth

I: What else P_3_?

P_3_: It is like that.

I: How many years?

P_3_: Maximum 5/6 years and minimum 4 years.

I: Why?

P_3_: Not to get stressed by the pregnancy and since it is a problem if you give birth without spacing/closely.

I: Okay, P_1_?

P_1_: It is like that.

I: How many years?

P_1_: Minimum should 3 years

I: Why?

P_1_: It should also be 6 years maximum

I: Why you say 3 years minimum?

P_1_: Helps the child become good

I: What else?

P_1_: To yourself also to have rest

I: Good. What do you tell us P_2_?

P_2_: The maximum is up to 5 years

I: Why?

P_2_: One, if she give births without spacing, neither the mother nor the child will be in good condition. There may also face shortage of food so that they may be exposed to diseases. However, if there is birth spacing, one, the child will start walking. Second, the child grows and reachs at good level. After that she can think to have another child and she can give birth 4 or 5.

I: What about the minimum?

P_2_: The minimum should be 3 years

I: Why?

P_2_: Woa. For the question why is……Laughing…

(The participants were also laughing together)

(Here, P_3_ said to let the child walk on foot and P_2_ complements P_3_ by saying it is to let the child walk on foot)

I: Okay, P_2_?

P_2_: Since it also requires economy.

I: From whom did you get the information?

P_4_: From teachers

I: From whom else? Okay, P_2_?

P_2_: From health extension workers

I: Okay, P_4_?

P_4_: From teachers and health extension workers

I: Okay, P_6_?

P_6_: As P_4_ said it, we had learnt it from teachers and again now we have learnt it in health facility during delivery. Thus, the health workers taught us in good manner. And advised us to use birth control/contraceptives. In addition, by looking at our body, they said you have to wait this years and told us not to give birth.

I: What can be done to promote it in a better way?

P_6_: To promote in a better way to make grow the child without stress, we have to accept it.

I: How can we promote this in a batter way P_4_?

P_4_: By creating awareness and teaching the public so as to control population growth since there is lack of land and water.

I: Okay, P_5_?

P_5_: Through learning and we should learn by health workers

I: Can you think of any other opportunities to prevent early marriage?

P_4_: Teachers. Teachers should give special support to women after class. Form Monday up to Friday they learnt from period 1 up to 6. So, after that they teachers should teach women separately by excluding men to convince them about the impacts of early marriage and thereby to prevent it. Thus, you can convince the girls but the parents no more to accept.

I: Any other opportunity to prevent early marriage?

P_5_: There is punishment. If they get married while they are underage, parents/the husband may get prisoned and thereby parents get lessons Thus, there is law.

I: What else P_4_?

P_4_: It is like that. They get prisoned and punished. Thus, they fear not to be punished.

I: What else other opportunities to prevent early marriage?

P_3_: It is like that. If they get married, the parents will be punished.

I: So, you mean there is law?

P_3_: Yes, there is law.

I: What else?

(All become silent)

**Section 6: Understanding communication and information sources**

I: Is there an opportunity in the community to discuss Nutrition for women?

P_6_: Yes, we discuss in women development army meetings and WDAs advised us to eat vegetables and fruits, to drink fluids like milk since it helps to produce breast milk.

I: Any other?

(They all become silent)

I: What are the opportunities?

P_6_: presence of women development army

I: What else?

P_4_: Health extension workers and teachers

I: What are the common sources for nutrition during pregnancy?

P_5_: From fluids

I: But what are the common sources of nutrition?

P_5_: From husband

(Al of us were laughing)

I: Okay, P_3_?

P_3_: If it is available at home, it is already available if not we will buy the fluids and the foods from market

I: Okay, P_4_?

P_4_: It is like that. We buy from urban. Actually, if you are in rural, you can prepare soup and the likes. However, if you are in urban, you will buy soft drinks and others by your own money if you have.

I: Who is your source of information?

P_4_: From health centers

I: From whom you get the information P_5_?

P_5_: From health center

I: Okay, any other?

P_3_: It is from that. The health workers told us

I: Okay, P_2_?

P_5_: It is like that

I: Okay, P_6_?

P_6_: It is like that

I: Okay, P_1_?

P_1_: It is like that

I: What messages?

P_6_: From health center?

I: Yes, from health center

P_6_: To keep the health of the child, they advise us to have medical checkups starting from the 4^th^ month and they also advise us everything to keep our health.

I: Okay, any other what messages?

P_6_: They advise us to take soup, vegetables and fruits

I: Okay, P_4_?

P_4_: It is like that. They said it is good if your enjera is made of Taff and advise us to take like soup and others.

I: Okay, P_2_?

P_2_: It is just to exchange and use from your economy…Laughing. If you eat sauce in the morning drink then to eat shiro at night, and if you drink coffee in the morning then to take soup at night and the likes

(They all were laughing)

I: Good. Do all women get the messages easily?

P_4_: Not all. They are limited. Only those who involved in the meeting get the message but not those who didn’t get involved and no one tells them by going to their home. There are mothers at home who don’t come to meeting because of their household activity so that they do not get the messages.

I: What do you tell us P_6_?

P_6_: It is like that.

I: What are the barriers for access to information for nutrition during pregnancy?

P_6_: Lack of education. Since we are educated, we understand everything starting from schools up to the current one that they teach us here and it is understood. Thus, we follow medical checkups/health messages and others attentively. However, those who are not educated, they said what we did before when we eat and what we did before when we care children to grow up.

I: Okay, P_5_?

P_5_: It is like that

I: Okay, P_1_?

P_1_: It is like that

I: Okay, P_2_?

P_2_: It is like that. Laughing

I: Okay, P_3_?

P_3_: It is like that. Laughing

(Others participants were also laughing)

(Her, one participant said…Eyyyyy…Our mind is somewhere else)

I: From whom the information you get is so effective?

P_4_: Information from teachers

I: Any other, P_2_?

P_2_: From health extension workers

I: Okay, P_6_?

P_6_: Lessons we learnt from health extension workers is the best

I: Why lessons learnt from health extension workers is effective

P_5_: Since it is useful.

I: How is it useful?

P_5_: How is it useful? We can get medical examination, and since we run to health centers not to cause the diseases any harm. So if health extension workers do not organize and cares us, where should we go?

I: Any other, P_4_:

P_4_: It is like that

I: Okay, P_3_:

P_3_: It is like that

I: Which information is effective for you to change your practice in nutrition during pregnancy?

P_4_: Information from health extension worker

**Section 7: Additional remarks**

I: Any additional remarks that you want to add? Something that you think not mentioned?

P_4_: Nothing. That is it.

I: Okay, P_5_?

P_5_: It is like that

I: Okay, P_2_?

P_2_: Nothing is left. However, women requires special support

I: What kind of special support?

P_2_: As mentioned earlier like corn soya and others. Laughing

(Others also laughing)

I: Okay, P_3_?

P_3_: Yes, food support is needed to women since they don’t have land as mentioned earlier. Thus, we need to be supported.

I: Okay, P_4_?

P_4_: Advice is also needed

I: What kind of advice?

P_4_: Advice like do this and this so that their mind will be refreshed if not they will be confused since they are always at home

I: So what should be done?

P_4_: To be told to come to meeting ……Ehh…

I: P_4_ please complete it?

P_4_: I have finished. Laughing.

(Others were also laughing)

I: I didn’t hear you the last one?

P_4_: Any ways it is like that

I: Okay, P_1_?

P_1_: It is like that

I: What do you say P_6_?

P_6_: I am also with them

I: Okay. Thank you. I have finished.

All said. Thank you.

**SUMMARY**
