# Supplementary material for: Barriers to utilize nutrition interventions among lactating women in rural communities of Tigray, northern Ethiopia: An exploratory study
Source: PLoS One. 2021 Apr 30;16(4):e0250696. doi: 10.1371/journal.pone.0250696 (PMC8087028; doi:10.1371/journal.pone.0250696)
Supplement: S2 File — (ZIP) [file pone.0250696.s002.zip › S2_File.Doc/Lacatating women_IDI & FGD/142_IDI_Lactating Women_Hakfen kebele_Medebay zana woreda.docx]

**Operational Research on Adolescents and Maternal Nutrition in North Ethiopia**

**Tool B: IDI with lactating women**

**Introduction**

| **Introduction:**  Hello, my name is Yemane G/mariam. I am from Mekelle University. Thank you for taking the time to speak with me today. We are doing research on the factors that influence the nutrition of mothers and adolescent girls in collaboration with the Regional Health Bureau and UNICEF. Your participation is very valuable. The things that you tell us will be used to improve nutrition programs and services for women and adolescents in the region and the country. We will not share your names when we report our results.  However, we will record the discussion so that we can capture all the ideas that are shared. We have several questions to ask you that we have prepared in advance, and we will ask you all to say what you think about each question. To ensure the privacy of everyone here, we ask you not to repeat what we discuss outside of this group. The discussion will last for 1-2 hours. Do you have any questions before we begin? If you think of any questions as we proceed, please feel free to let me know. If it is all right with all of you, we will turn on the tape recorder now.  Do you agree to participate in the study?   1. **Yes** 2. No   **Section A: Interview details**   1. Zone:__**North west** 2. Woreda: --**Medabayzana** 3. Kebele: Hakfen 4. Facilitator’s name: --Yemane G/mariam 5. Date of discussion: ----Nov, 21, 2017 6. Discussion start time: ___9:00 afternoon 7. Discussion end time: ____10:07 Morning | | | | | |
| --- | --- | --- | --- | --- | --- |
| **Section B: Socio-demographic Information** | | | | | |
| Name of IDI participants | Age | Marital status | Education level | Occupation |  |
| 1. Letensea | 32 | Married | o | Farmer |  |

**I:** Interviewer **P:** Participant

***Section1: Common lactating women nutrition problems in the community.***

***I: What do lactating women do to stay healthy in this community/woreda?***

***P:*** We lactating and pregnant women have meeting every week or two week to discuss on our health which is facilitated by women development army (WDA). This helps us to be safe from any danger during pregnancy and lactating even for the adolescent girls they teach them how to protect themselves from different challenges. Pregnant women come to this health post and they will go to health center if necessary, this helps the pregnant women easily to avoid anemia, to improve their feeding to reduce under weight and overweight. During delivery; thanks to our government now the health center is good in providing ambulance and services. We are near to the health center when we call to ambulance they come take us to health center on time now we are fine and the baby are also fine. And the baby exclusively breast feed for six months and after six months she must start additional complementary feeding. When she feed her breast to the baby first the one breast must be empty before she shifts to the other breast since the most nutritious part of the milk is come at the end whereas the first coming milk is more of water. When the baby become six months she must prepare variety of foods based on the available foods in the house by mixing them when she prepare porridge and soup. When the baby gradually grows we feed them milk, egg, vegetables and meat.

**I: Can you tell more focused on lactating women what they do to keep themselves healthy?**

P: First I wash my hands using soap not to transmit the dirty in my hand to the food during food preparation to keep myself and my family from any diseases. I cover all food containing materials and water containing materials because if it is open it can be polluted by fly or other pests and may result for diseases if it is eaten by our children. Therefore if I wash my hand, wash my children and keeping the cleanness of the food we will be free of disease. The diseases come when we don’t keep sanitation of our environment with animal dung and child faces.

**I: What do you do to keep your environmental sanitation?**

P: What do you mean! We stand early in the morning and clean it since we have compose we will take it the waste into the compost. And when my baby defecates in his cloth or in open field I have to clean and wash him otherwise he will be invaded by the fly and can be infected for different diseases.

**I: In your opinion, what are the common nutrition problems in the community for LW?**

P: Till now I have not seen LW with malnutrition problem in our kebele due to food shortage.

**I: What type of nutrition problem do you know?**

P: she may face nutrition problem if she didn’t use properly the wealth (available food) in the house by selling to the market like honey, teff, maize and others, but since she is PW or LW if she eats the hone, teff and maize her baby as well as herself become healthy and safe. Since pregnant and lactating women are feeding well, visiting health center to be measure their weight and height and give births that is why I didn’t say any malnourished mother.

**I: Do you think LW is at risk of malnutrition? Why?**

P: In our kebele I don’t think so to have malnutrition. Because when they are measured their weight and height till we haven’t her someone is getting below weight or below normal therefore we don’t think we are at risk of malnutrition.

**I: Do you think that LW in this community is suffering from Micronutrient deficiencies?**

P: Previously we were heard of blurring vision but now since all pregnant and lactating women are following or visiting health center they are taking red tablets and other treatments due to this now we can say no blurring vision among mother. We come every months on the day of 07 namely Trinity and take also tablets to prevent anemia.

**I: Is their swollen of the neck (goiter) seen in your community among LW? What is the reason?**

P: I don’t have goiter and I haven’t seen in any of the PW and LW in our kebele whereas previously polio which paralyze the leg and hand was common among the children but now it is totally not seen.

**I: Is there any communicable diseases in this community?**

P: HIV is communicable disease that can be transmitted from person to person through sharing sharp materials like needle and blade as well as unprotected sexual intercourse. Tb (tuberculosis) is communicable diseases that can be transmitted through drinking water without washing the cup once an infected person has drunk with it we have learnt on education.

**I: Is there any non communicable diseases in your community?**

P: What does it means non communicable? I: explanation was given about non communicable.

P: I know diabetes is non communicable but in LW I haven’t seen any non communicable diseases.

**I: What do you think, why women/girls in this community would not increase their height proportional to their age? Could it have relationship with their nutrition?**

P: Yes of course! When a woman is not increasing her height while her age is increasing this is definitely due to malnutrition. Because if you well feed a child with less than five years his height increases but if he doesn’t eat well he become short and short without increasing his height as his age is increasing. Regarding women we do have short women and this could be due to malnutrition or may be due to hereditary from their parents.

**I: Do women/girls in this community increase their weight proportional to their age? If no, what do you think the reasons would be?**

P: Yes, even though there are women who are thin as compared to their age but not common in our kebele. For example no one is thinner than me.

**I: Do you think that you are thin?**

P: Yes, since the life of world is difficult I think I am thin and not comfortable.

**I: Have you measured your upper arm and your weight?**

P: Yes, they have measured me my weight and MUAC (by showing her upper arm) and they said I am normal. We were measure our weight and MUAC every months during pregnancy and till six months of after delivery.

**I: Do LW in this community suffer from overweight?**

P: Smile on her face and after laughing, in rural area overweigh is not common.

**I: Do you think over weight is due to good nutrition?**

P: I don’t think it is due to good nutrition but since women in rural area are busy in work I haven’t seen any over weighted women.

**I: Do you think shortage of food is occurring among the family for the past one year?**

P: Thanks to our government no shortage of food.

**I: What do you mean thanks to the government how does government support you?**

P: The government support for those who don’t have capacity to work like liver (black abdomen) diseases patient in cash or wheat.

**I: Did you get any food support while you were pregnant and lactating?**

P: Why they support me, I have not got any food support during pregnancy and lactation.

**I: Is their productive safety net program in your woreda?**

P: Yes, they recruits based on their criteria like those who haven’t ox and land to be plough.

**I: Have you been in safety net program?**

P: No, why me since I am not poor they don’t include me.

**I: Do PW and LW get food support?**

P: No food support given to PW and LW.

**Section 2 :Barriers to access and utilization of nutrition services**

**I: What kinds of nutrition interventions are in place to improve health of the LW in this woreda? Where do they get it? Who provide it?**

P: When I was pregnant and went to health center for delivery they provide us porridge made of butter, paper and balanced diet “Mitin” before two weeks of delivery we go to the health center the government feed us till we give births in the health center. They give us clothes and soaps for the baby.

**I: Why do they give you soap?**

P: The soap is given to wash my hand before feeding him my breast and to wash for the baby.

**I: Have you gotten counseling on nutrition? What are they?**

P: Yes, they tell us we have the right to get services from the beginning of the pregnancy till 45 days after delivery for example urine tests, blood test and “it is on my tongue but I am forgetting it”. They advise us not to hide any symptoms or sickness and we must to got health center

**I: If what type symptoms do you see will go to health center according to what they advised you?**

P: If we have bleeding within 45 days of delivery and to take injection for family planning after 45 days of delivery.

**I: What type of family planning do you know?**

P: Tablets (pills), injection either for one year or two year based on your interest.

**I: Currently are you using family planning? What type?**

P: Yes, I am taking tablets (pills).

**I: What advice did you received while you take the pills?**

P: They told me once if stop using the tablets I may see excess bleeding and not worry when I see like this.

**I: Who provide you this information?**

P: Health worker from health center and health extension worker in the health post.

**I: What kind of nutrition advice have you received?**

P: While I was pregnant they told me to eat extra meal one in the morning, second before lunch, third during lunch, fourth before dinner and fifth are in the evening. And I was eating accordingly and when I went to health center and measured my weight he was appreciated me for weight gaining.

**I: What type of food were you eating in the five schedules?**

P: I was eating “shiro” sauce, next “siles” (it is sauce more of tomato content), again shiro and later silse.

**I: Do they tell you like this to eat? Why not you followed it?**

P: No they told us to eat different food in the five schedules. Even though we have work load to do all variety of foods but we are using by mixing teff and maize while we make injera.

**I: Is their nutritional screening for LW? What is its importance?**

P: Yes, they measured us our weight, height and MUAC (by showing her upper arm) in the health center as well as in health post. It has importance unless why do they measure us if we are normal no problem if our weight is below normal they advise us on how to gain weight by eating balanced diet and increasing the frequency since we will suffer challenges during and after delivery if we are below normal weight. Once we eat and gain weight based on their advice they appreciated and encourage us to continue like this that is why we are not facing challenges during and after delivery.

**I: Do you use Iodized salt?**

P: Yes we use iodized salt now everyone has stopped using non iodized salt; the iodized salt must be poured after the cooking is ended to avoid evaporation of the iodine and not taken by the wind.

**I: What is the importance of using iodized salt?**

P: The iodized salt sharpens the mental thinking of the children and reduced the swelling of the neck (goiter) by the way I have lost my concentration due to thinking of my children and the cattle but I can remember more than this.

**I: Do you get any advice on home gardening? What?**

P: Yes they teach us on the importance of home gardening like vegetables that we can use it even if we produce more we can sell it to the market that can have small support to your home.

I: **Do you have home garden?**

P: Yes, I have home garden, we eat it for ourselves and the remaining will be sold. We eat cabbage, salad, and chili with yogurt and milk. We get this information from health worker and HEW from health center and health post.

**I: Are you while you home gardening gives you fruits?**

P: Yes, that is why we are coming here when they (HEW) need or call us.

**I: Do you think LW need food support like free safety net?**

P: It is not common in our woreda but it will be better if they are supported.

**I: Do LW getting advice on water, sanitation and hygiene services? How they do get it? What is its necessity?**

P: We have access to pump pipe water which is nearby our house and they treated with white tablets to kill insects and pests; they inform us not to use the pump water for one day.

**I: Do you treat water at your home?**

P: Yes, I could not remember the name (from her description chlorinated water AKA “wuhaagar”) but we add it to the water and we stay for some time and we drink it. They (HEW and WDA) advice us to clean our environment and remove stagnant water to prevent malaria.

**I: Is malaria common in your kebele?**

P: Yes, but since we use ITN it is not affecting PW and LW. ITN is distributed to all households and when a pregnant woman goes to health center they give her ITN in addition to her family has.

**I: Is their Deforming?**

P: Yes, they gave us red and white tablets by HEW in the health center and health worker.

**I: Do you think it has importance?**

P: Yes, it cleans parasites in our abdomen.

**I: What do they do if PW or LW is below normal during nutrition screening?**

P: In our kebele I haven’t seen for PW and LW but for children when they measured for MUAC if the measurement is on the red they take fluid food packed with sack (Plump net). Even though children are not less than the normal range therefore it was long time to see malnourished child for me. Our kebele is capable of feeding themselves that is why food support is not given since we can feed ourselves.

**I: Which of the interventions listed above do you think is most important for pregnant and lactating women?**

P: Using iodized salt, keeping environmental sanitation, ITN utilization, feeding Varity of foods likes vegetables and fruits using home gardening all this are good.

**I: Why do you say the above listed activities are good?**

P: Since our government has working on maternal issues therefore the health workers and HEW are implementing it.

**Section 3: Perceived needs of women for relevant services**

**I: What do you think is necessary for the LW in your community?**

P: As I know, everything is good.

**I: Do you think PW and LW need rest? Why?**

P: Yes, especially during pregnancy and lactation she need to have rest. Taking rest helps the PW and LW to protect their child and themselves from any danger or to strengthen them.

**I: Do you believe eating balanced diet and having extra meal benefited LW?**

P: Yes that is why we are eating extra meal and balanced diet as they teach us.

**I: What do you discuss in your community day? When?**

P: As I have told you previously we have discussion with our network WDA every month on 07^th^  day. We discuss on feeding practice, we ask whether pregnant or lactating women are following or visiting their appointment during our discussion. And it has good progress sin motivate her to go to health facility by creating links with the health extension workers.

**I: Do you need additional intervention on nutrition for LW? What are they?**

P: We are getting Vit A supplementation, trachoma treatment and nutrition screening.

**I: What is the role of husband in supporting LW?**

P: Husband must support his wife in different way for example starting from the time that she knew her pregnancy till her delivery as well as lactation period by reducing workload, helping her to eat diversified food and follow whether she eat or not and advice her to eat.

**I: Does your husband has supporting you? How?**

P: Yes, that is why I am telling you the above is from my experience.

**I: Does the support of your husband have brought an importance to you? How?**

P: Yes, do to my husband support I haven’t sick and I give birth without complication. For example when I become term to give birth my husband take me to the health center and after delivery he was with me till we come to home.

**I: What foods are recommended for LW? What foods does LW avoid?**

P: She is recommended to eat red teff, porridge and soap and shouldn’t eat sorghum because it doesn’t strengthen her if she eats sorghum only. Since she may have lost her blood to replace it she needs to eat red teff during lactation.

**I: Is there any food taboo for LW?**

P: After long laughing, today no food is forbidden for women we eat equally with all but during pregnancy sugar is not recommended since it may increase the weight of the baby.

**I: Do LW in this community typically change their diets during lactation? How is this diet different from when they are lactating?**

P: LW must eat more additional meal because she is responsible for the baby as well as for herself and the baby is dependent on her.

**I: If you want to eat but your husband is not in your home do you eat without him?**

P: Yes, if I get hungry I will eat.

**I: Are there gender disparities in women’s diets during lactation?**

P: Wa! After laughing, no gender disparities in our community even though previously there was gender disparities now we are equal.

**Section 4: Other interventions that improve pregnant, lactating and adolescent nutrition**

**I: Have you ever gone for nutrition screening? Where? Who provide you?**

P: Yes, I have screened for my nutritional status they come and measure by home to home and sometimes if the WDA gather us they measure us in our village. I couldn’t remember the day but they come and measure us. During home to home HEW measure all the family members.

**I: Do you think it has importance?**

P: Laughing… if it doesn’t has importance why they are getting tired. It has importance for us.

**I: Do mother improves her or child when she comes for the next screening once she is known as under weight?**

P: Yes, but as I have said previously under weight is not common in our community.

**I: Do you think nutrition screening has acceptance and the community believes it is for his benefits?**

P: Yes, everybody is volunteer and they come for the screening all the time. When the HEW come and measure home to home once the community see their coming they gather and bring their children even from farming for sheep keeper child.

**I: Do you think LW needs to be targets for supplementary foods? Why and why not?**

P: Unless they are poor and have not income generation no need of supplementary food because the women have enough food in her home.

**Section 5: Understanding perceptions of age at first birth and birth spacing**

**I: Do you think delaying the age at first birth to after 18 is better for the health of the women? How? What other benefits does it have for the women? What about for the baby?**

P: Yes, it has importance that is why our government has introduced it. For example if she gets married earlier than 18 years she will get difficulty during delivery since her body is not strong for sexual intercourse may develop fistula whereas if she married above 18 years she is matured and can get pregnant and give birth without any problems.

**I: How many children do you have?**

P: I have six children

**I: What was the birth space interval among your children?**

P: The birth interval between each child was three year but now I have planned to stop giving birth that is why I am taking family planning.

**I: Do you think birth interval has benefits for the baby as well as for herself?**

P: Having birth interval is important for the baby and for the mother since it gives chance to care for their baby without burden but if she has more children side by side she become busy and get tired to feed her children as well as herself.

**I: Do you think you were burdened due to having more children?**

P: laughing… till now I am ok but for the future I don’t know.

**I: Do your community support early marriage?**

P: Wow! It is very tough to get early marriage in our kebele unless they are 18 and above.

**I: Do the community accepts early marriage as taboo or forced by law?**

P: The community has accepted early marriage as it is not good rather it is very difficult to control by law or force.

**I: Who have done more to prevent early marriage in your community?**

P: Kebele leader, HEW, WDA and the students themselves (Adolescent girl) have played a great role.

**I: Do the community accept birth spacing or interval? What family planning method they used? How long is the interval?**

P: Yes the community has accepted since it has importance for example if you have two Childs with no birth spacing both children will not be feed properly and may become under weight and sick. The birth interval must be four to five years. If the birth interval is wide the child becomes healthy and well nourished.

I: What do you recommend to have birth interval or spacing? Do you think women in this community give births with interval?

P: They must use family planning. Most of the women are giving birth space or interval before they birth the next child at least three years of interval up to four, five and six. I know one woman she give birth space till six years.

**Section 6: Understanding communication and information sources**

**I: What are the sources of information for you? Which one is/are more effective and trusted sources of information?**

P: Kebele leader, health worker, HEW and WDA. And we trust them all.

**I: Why do you say all are good?**

P: Since they teach and follow us our activities.

**I: Do they follow you about keeping sanitation and personal hygiene?**

P: That is why I am telling you they are good.

**I: Is their demonstration on porridge preparation in your community?**

P: Yes, we have demonstration on porridge and soap preparation which comes from government which helps for demonstration.

**I: Continue please tell me.**

P: Sorry since I am not educated I couldn’t describe all.

**I: Please don’t worry your telling me a lot of information.**

P: We are coming for the demonstration with good interest if we are not clear about the messages sometimes we ask and sometimes we simply go and learn from repeated observation.

I: What do you recommend to have better information communication means about maternal nutrition?

P; all is good.

Additional remarks;

**I: Any other additional suggestions or comments on pregnant, lactating and adolescent nutrition in this community.**

P: I don’t have to add, since I haven’t seen any under nutrition among PW, LW, Children and adolescent everything is fine.

Summary:

Section I:

- There is regular meeting with their WDA network.
- No nutrition problem
- No micronutrient deficiency
- Over weight and underweight are not common
- No food shortage
- No food support for LW

Section 2:

- There is nutrition screening for LW
- Used iodized salt
- Used ITN
- They have home gardening.

Section 3:

- There is Husband involvement
- No food taboo for LW

Section 4:

- There is home to home nutrition screening

Section 5:

- No Early marriage
- Birth spacing accepted by the community between 3 to 6 years.

Section 6:

- HEW, WDA, Health worker and kebele leaders are trusted sources of information.
