# Supplementary material for: Barriers to utilize nutrition interventions among lactating women in rural communities of Tigray, northern Ethiopia: An exploratory study
Source: PLoS One. 2021 Apr 30;16(4):e0250696. doi: 10.1371/journal.pone.0250696 (PMC8087028; doi:10.1371/journal.pone.0250696)
Supplement: S2 File — (ZIP) [file pone.0250696.s002.zip › S2_File.Doc/Woreda level and above key informants/001_IDI_Agriculture office_Ofla woreda.docx]

**Introduction**

| **Section A: Interview details**   1. Zone: **South Zone of Tigray** 2. Woreda: Ofla 3. Kebele: 4. Name of key informant: **Mr. Gebru Teklay** 5. Institution of key informant: **Agriculture** 6. Interviewer name: **Kiros Tedla** 7. Date of interview: **03/11/2017** 8. Interview start time: **09:37AM** 9. Interview end time: **11:15AM** |
| --- |

| **Section B: Interviewee professional information**   1. Sex    1. Female    2. **Male** 2. Highest level of completed education.    1. No formal education    2. Primary education    3. High school    4. College education    5. **Bachelor degree**    6. Master’s degree    7. PhD 3. Discipline or field of educational training    1. **Agriculture**    2. Health (MD, nurse, health officer, midwife, pharmacy, etc)    3. Nutrition    4. Public health    5. Food science    6. Other (specify): 4. Current position: **Deputy Head of Agriculture and Extension coordinator** 5. How long have you been in the current job/position:    1. ______ Months    2. **5** Years |
| --- |

**I:** Interviewer **P:** Participant

**Section I**

**I**, 1.1 **what do women do to stay healthy in this community or worerda?**

**P**. When we see the topography of our worerda, is mostly high land, there are also lowlands and 48% of semi high land with mountains and becoming low soil fertility due to degradation. Because of this our woreda is one among other woreda in the region related with problem in maternal and child nutrition. As agricultural sector we are working to improve the feeding habit and nutrition of mothers through education and modern feeding but still there are implementation problems. There are also very good practices as example we have done pilot study using 1449 mothers and we have got very promising results regarding maternal nutrition. We have also observed changes on farmers, children and pregnant mothers because of the trainings.

Regarding to this topic there is change through time regarding nutrition in the community compared to the past; however; the change is very stagnant. We still have people have the necessary nutritional foods such as egg, meat, milk and milk products, we have also fish but they do not utilize them and they eat meat only during holydays; they only produce them for marketing purpose. So there are cultural and other barriers related with nutrition in the farmers. As an example; in this woreda there is high fish production but the community do not use it because of cultural related barriers. Therefore; we need to bring a change on the awareness of the people to nutritionally healthy community.

**I, 1.2 what are the common nutrition problems in the community for pregnant, lactating and adolescent girls?**

**P.** The problem related with malnutrition in this community is because of the feeding style of the farmers they only depend on one commodity like the farmers most of the time use wheat but not others like vegetable, meat, fatty acids, honey; still there is a gap or problem on the farmers on the use of those foods. Even though there is an improvement in the production; there is a big problem in utilization of the food products for themselves either from their own or from the market. They only produce for marketing. Severe malnutrition is common on mothers but on adolescents it is better as they can get information particularly regarding to vegetable production or home gardening during schooling and farmer training centers as there are trainings and demonstrations for schools. There are also experience sharing among schools and there are also kebeles with better performance due to this but still we do have problems in practicing those particularly on pregnant and lactating mothers. Previously there was high prevalence of Goiter in our area particularly around the hashenege bassine but when we see currently as an agriculturalist we use to types of fertilizers nitrogen and phosphorus but now there is a “boron” shortage as manifested in new burn back bone abnormalities or neural tube defect due to poor agricultural practice. Anemia is becoming very common on mothers as indicated during delivery; there are also other problems like shortage of zinc. Hence, these and others are the result of poor nutrition resulted from poor agricultural practices. To improve these we are trying to introduce new fertilizer and ways of house gardening practices for vegetables and working on farmers to utilize these products for their own or by cashing small amount of money from the market. Stunting and underweight are very common in this area but overweight is not the problem of this community. There is also anemia as manifested on children and mothers. Example children had got very difficult to see when you put pen in front of them due to blurring of vision and mothers had got difficulty during delivery due to anemia. These all are the result of shortage of high energy giving nutrition and vitamins. When we talk about food security, even though the production is improving; it is mainly focused in one commodity; but still it cannot solve our nutrition problem. So in one house if they can produce the four blocks of foods; we can say there is food security in the house; but know they are only producing only one block of foods but not the different foods which are suitable to our body.

**I**, 1.3 **which women groups are affected most among pregnant, lactating mothers and adolescents by the above mentioned nutrition problems?**

**P**. Our main problem related with nutrition is among pregnant and lactating mothers but not on adolescents. Example; we have sampled from “SURE” utilizing mothers; we have got a mother who can’t feed or lactating her baby due to nutritional problem. Particularly when mothers are pregnant they lose appetite and lactating mothers are also restless and as a result they are the once who are affected by malnutrition at most. Therefore; if we can work on mothers meaning lactating and pregnant mothers and improve the awareness of adolescent girls we can be success full.

**Section two**:

**I,** 2.1**do you think it is necessary for your institution to involve to get involved in the work aimed at improving maternal nutrition?**

**P.** Yes, because it is one of the three sensitive issues such as nutrition, climate change and gender equality. These are the main prioritized agendas by the regional government and the woreda as to have healthy community or leader you should have healthy feeding or nutrition and then you will have very innovative visionary leaders. Therefore; as a woreda we are working on improving nutrition by considering as one of the sensitive agendas. We use all our farmers training centers and we demonstrate them on all the agricultural components such as hen farming, crop production, vegetable like potato production and karot, animal production. We also train “Gujle Lm-at” and work with them for better performance and change. We are also already working on nutrition; starting from planning to bring a change and up to demonstration on vegetable cultivation and others. We have established a program working on selected very poor farmers to improve their production and nutrition by giving training and very small amount of incentives. The program also works on rich famers able to introduce the agricultural components. Therefore; if we can get donors or other organizations we use this existing channel and get a better result. If there are other experiences which can be implemented here; if there are donors who can give or assist the cost of the vegetables seeds or who provide the vegetables so that the economically poor farmers can get the vegetables as well as we can also able to avoid the shortage for all farmers. On the other hand; if organizations like yours (Mekelle University) able to provide us a training to build the capacity of the DA and Gujile lemat working at the kebele level and if there are photographs, posters and images on maternal nutrition to teach the farmers.

**I. 2.2 what maternal nutrition interventions targeting pregnant, lactating mothers and adolescents are the priority in this woreda?**

**P.** On our worerda the main priority is on house gardening or vegetation as most of the farmers are able to produce wheat by themselves and have animal production. We also focus on hen farming. From soil content perspective of having important minerals to human; our most time is spent on improving the mineral content of the foods like boron, iron, zinc, and phosphorus which is reflected on bone abnormalities, by changing the content of the soil particularly using fertilizers; so that the farmers will have improved production and nutrition. They will also be advised on the use and cultivation of house garden on vegetation to minimize the effect of micronutrients on vision, anemia. Our main resource is allocated on introduction and expansion of fertilizers to enhance production for marketing and for in house utilization. Secondly on expansion of small farm irrigations to produce vegetables such as peppers, cabbage and cauliflower.

**I,** 2.3 **can you tell me some of the successful interventions maternal nutrition interventions which are implemented in this worerda?**

The most effective intervention with our stakeholders is concerning pregnant women and child nutrition which is the introduction of animal production like hen farming, milk production and utilization from both sheep and goat. But compared to the above, house gardening and vegetation of both permanent and the short time vegetables is less effective. Even though the permanent vegetables are not important immediately for pregnant women they are important for adolescent girls so we need to work on it. We need also to work on awareness and those we did not rich at the time being or the missed once for both mothers and adolescents (example there could be adolescents out of schooling so we need to work on awareness).

**Section 3**

**I, 3.1 what kind of nutrition interventions are in place to improve adolescent and maternal health?**

**P.** On pregnant women they will be followed on all aspects of their health but coming to agricultural sector we work on safety net program like if they are members of safety net program we will make them free of work and let them take rest. But concerning other health related issues we will follow them communicating with the health sector particularly on feeding habit and types of exercise but mainly we focus on resting and giving first to them. There is also nutrition screening practice and all the materials are introduced in the health service. Pregnant women are then followed and based on these findings they will be given the different interventions like for example of they need rest. Supplementary feeding and iodized salt yes there is education but still need to be worked on it.

Pregnant women will be studied and if they were participating in safety net they will be leaved from work after they get signature from the health professional. Regarding on use of vegetables or house garden products; pregnant women able to buy from market enable and train them to buy and eat. For those who cannot by themselves they will be helped to cultivate at home through training by “Gujile Limat”. On hygiene, water coverage is increasing so that the community is advised to use pipe water but not from rivers. They are also educated on personal hygiene through demonstration and to wash the vegetable before eating or cooking; to wash all feeding materials. In this area malaria is very rare even though there were malaria before. The community is also aware about how to use bed nets and how to control the vector by eliminating the water containing grounds. Supplementary feed safety net like fafa and plamplet by health. Through agriculture we try to provide vegetables with the support of “sure” and we work also to improve the awareness of the community trough training and demonstration in the FTC in order to produce the vegetables in their home. And those who are unable to produce and economically poor will be given the back yard vegetable seeds from the FTC. We declared that all agricultural experts or practitioners should help all pregnant women and secondly those economically poor pregnant women are assisted in different agricultural practices like during plowing, harvesting, trashing and giving different trainings.

On lactating mothers our intervention includes resting after birth from safety net continued the treatment during pregnancy until the baby is two years old. And when they bring the children we have prepared a waiting space for their children. On feeding practice we have worked with health sector on how to feed and keep safety of their children.

Regarding nutrition sensitive agricultural practice we work mainly on both pregnant and lactating mothers. We train and demonstrate them on the FTC about home gardening and help those economically poor pregnant women by providing vegetable seeds from the FTC by low cost. But on adolescent girls we mainly work on awareness creation and we also make the students to cultivate vegetables and share their experience among themselves and to the community by selecting the model schools. About school feeding program we have done previous year because of the drought but now I have no knowledge on it wither it is working or not. But during the drought period had given the students together with health experts in order to see the quality and safety of the food program.

**I, 3.3 other comments or suggestions regarding interventions in this worerda**

**P.** We have to work also on male as 33% of the population of our country is male. So we have to participate young male in order to achieve our objective. This is because including mothers and adolescents is important but to make stronger it is important to involve particularly young male with the adolescents as they are parts of the life of the mothers and adolescents.

**I, 3.4 which of the above mentioned interventions are implemented successfully or effective and why?**

**P.** The most success fool intervention among the three categories; the intervention targeting mothers is success full; this is because the problem related with mother mortality during delivery is decreased; only one or two mainly per year because of technical problems. This is due to increased follow up during and after delivery, counseling on feeding and resting, improved water quality and house hygiene. The children are also very different compared to previous once that they are becoming very active, improved cognitive statues and IQ because of improved feeding and quality of mothers by the interventions. On adolescents there are changes as they are trying to practice at their home what was given by the training. But our big achievement is mainly on pregnant women; they have a monthly scheduled meeting to discuss on ANC, feeding and the above interventions and this will be continued after birth as well. All these activities have been done in coordination with DAs, HEWs and Gujile Limat. The main strategy is the government strategy and we do have a stream committee at a worerda level to discuss in all sectors regarding women affairs. They discuss on the performance of agriculture and other sectors regarding their role and achievement on enhancing women economic status. This discussion will be continued up to the lower level or kebelle with Gujile Lmiat and discuss on participation of women on all affairs. As a worerda we have improved women saving which improves the economic status of the women.

**I,** 3.4 **which of the above mentioned interventions are less effective and why?**

**P.** We did not reach all mothers in need of the services and on adolescent girls it is very poor only it is a trial in some areas. The other is on involvement of men in maternal and adolescent nutrition as men should help their partner or wife in all works particularly during pregnancy and delivery this is because a mother who had given birth should not cook herself immediately; the man should take care of his wife by cooking for his wife. That is why men should be an integral part of the maternal and adolescent nutrition.

**I, 3,5 what are the implementation challenges that are specific to delivering the maternal nutrition interventions that we have been discussing above?**

**P.** The problem starts from ours as we were not able to provide continuous training at the lower level and we focus on routine activities and then give poor attention to the program achievements. The implementation problems are related with resource scarcity like lack of well prepared land, different equipments, Shortage of vegetable seeds, high turnover of man power as most of our trained DAs move to other places. Distance and geographical location particularly the areas found with the border of Amhara region like Dinka are very far from Korem or the worerda are resource poor areas naturally that is why we are an able to implement the interventions like vegetation or home gardening.

**I, 3.6 which of these challenges are the most important?**

**P.** The most important challenges are lack of commitment from the workers of the sector starting from lever DAs up to higher officials including the stream committee. There are also problems related with selection of the inappropriate trainers at the lower level and farmers related problems like unable to implement or practice the training like home gardening, feeding style and crop production after the training due to carelessness.

The main problem related with the nutritional sensitive agriculture is related with men. Even though the mothers believe that NSA is very important the male is reluctant to practice it and they only left it to the women like the home activities. They believe that they should do it by themselves. There are also problems related with feeding habit and fertilizer utilization which could improve the nutritional output of the seeds.

**I, 3.7 Any Region/Worerda related success or interventions used to tackle the challenges and to improve maternal nutrition delivery?**

**P.** We have trained a round 238 farmers using model male and female on nutrition for three days. The training includes production and utilization of crops, vegetables or NSA, and feeding style through demonstration and practiced on how to prepare the foods from all components of the agriculture products. These models were also involved in training and experience sharing to the local communities of different areas or kebelles. We have practiced school based vegetation in selected schools such as “Menkere” and Zanta were these schools have water. And using these schools we have tried to give trainings to other schools and surrounding communities. There is also experience sharing among the schools and there are adolescent girls learned from this and start home gardening.

**Section 4**

**I, 4.1 Any barriers that prevent adolescents and women from using the programs and interventions discussed above?**

Yes there are problems related with mothers like they select interventions themselves among all other interventions even though the interventions are equally important to them. Here they will only practice their own choice but avoid the other interventions. As an example, from agricultural interventions; they do not to cultivate permanent vegetables; all most all needs to involve on short term utilized vegetables like cabage and hen farming. Similarly, adolescents also have similar problem. Problems related with service delivery are not very much but sometimes scheduling problem during meeting among health sectors, the agriculture and the community. There are some barriers related with the community as well. As an example; the area is rich in fish but not much consumed in the community because they believe that it is smelling, there are problems related with eating of meat and other animal products even they do have because they believe that meat is eaten during holy days only but it known that pregnant women needs meat. Other believes like eating vegetables are related with poor people so that they avoid eating vegetables like potato and others. We have manifested this during this summer.

**I, 4.2 How can these barriers be solved to improve maternal nutrition in the communityor worerda?**

**P.** First it should start from the way of thinking that human should eat balanced diet to live healthy so that we should work to change the way the community perception on balanced diet starting from the worerda up to the lower level through trainings and using model farmers. We also use video to show them about food preparation at night during meetings with Gujile Lmat and we advice them to prepare the samething in their home. So that we can make nutrition the issue of all the farmers and improve the perception of the farmers. We should also include all agricultural components which fulfills all nutritional valuable commodities so that we can do very good job regarding nutritional problems.

**Section 5**

**I, 5.1 Why would increasing the space between each births and delayed marriage improve maternal nutrition and health of both mother and infant?**

**P.** Giving birth at 18 years old and after four years meaning at 22 years is very different. It is better if she gives birth at least after four years of marriage. This is because at 18 years the mother is not well nutritionally which affects both maternal and child nutrition; she is not well in protecting herself and the child in giving care. Her sexual desire is also affected by this early marriage. We have seen this practically many times in this area that mothers who married at 18 years are challenged during follow up, delivering and after delivery as well. These mothers are challenged in feeding for themselves and their children so that both of them become nutritionally poor. So that it is better to give birth at later age. It is also better to give birth at different intervals as this is not good for both children and mother. Mothers should think of how to grow a nutritionally good baby rather than giving birth within a short period of time as this very good for the health of the mother and the child as well. Birth programs promoting birth intervals are mostly worked by the health sector but in our sector (agriculture) we work related with safety net, if mothers give birth and are members of the safety net we give them rest and give nutritional counseling on balanced diet. Safety net is one program in our sector which includes mothers, male farmers but more focus on mothers we give education on HIV, nutrition and gender. Early marriage is mostly worked on schools through drama and by giving practical examples in the community who had experienced the effect of early marriage. There is also committee working on early marriage at school level. This committee is working also on how to use modes to the female students in collaboration action AID Ethiopia and Agriculture. This has improved school attendance of the females and avoids absenteeism. In relation with politics to space between each births and delayed marriage are main pillars as all the stream committee members have political leadership.

**I,** 5.2 **are the programs related with birth interval and delayed marriage effective? Why or why not?**

**P.** Yes there are changes from time to time on both early marriage and birth intervals. But, still there are very surprising and heart teaching events particularly related with early marriage and gender violence. For instance, there was one very teaching event around the “Sesela” violence. Regarding birth interval the farmers are now understood its importance and start practicing it. Community related barriers mostly manifested at family level as there is promising among the family members for marriage relationship before giving birth that they will engage their children after birth based on only family interest. Still there are also early marriages among the farmers by hidden; they name the marriage ceremony as holly day or “tsebel” and send their child early; this is also practiced among farmers who have been perceived as having good knowledge about early marriage. There was one girl married early and became up normal and still suffering from the problem. Regarding policy, the policies have no any problem in relation with birth interval and early marriage but the problem is related with on the implementers. The main problems related with implementation challenges is because of higher resistance from the family members when you tell them that it is better to teach than marry her. But they could not listen you because of the previous cultural barriers. For instance, I have a good experience regarding early marriage here in “Maymado simret”; I do have the evidence that she is below 18 years but the father said to me that I am her father; I know the age of daughter better than you. To avoid these barriers everybody in all sectors should work and bring changes on females pregnant and on below age school girls. Particularly if we work at school level; if we able them to discuss with each other and bring practical examples from the community who are affected due to early marriage to tell how early marriage affects the life of the girls like we do against HIVAIDS in the early times and did it consistently we can end early marriage in the community.

**I, 5.3 can you think of any other opportunities to prevent early marriage and increase birth interval?**

**P,** Yes, we do have opportunities like there are girls who denied early marriage and live with their relatives at cities and continued their education; now they have finished their schooling and are at a very good condition. Therefore; we use such girls as models or best practices teach the community practically or though video. As I have said before we do have around 28,000 students and if we use these students to educate about nutrition and early marriage we address not only the students only we also address their family as well. We do have different clubs working in schools like female club and “Timret club” working on female affaires so if we can use them and educate through literatures like poem and drama we can bring the denuded change but the main thing is that everyone should own it and work for it.

**Section 6**

**I, 6.1 Do you think it is necessary for your institution to work with other sectors to address maternal nutrition?**

**P.** Yes, agriculture cannot work separately, all the other sectors such as health, finance, religious leaders, elder community members and others stakeholders are also have very important role as more than 80% our population in our area are farmers. Therefore; if we work collaboratively with all those sectors to change the awareness of pregnant women and adolescent girls at school level; we can bring a change. The role of Agricultural institution is mainly on empowering the women to produce all the necessary three or four commodities having nutritional values from their land and improve its utilization for themselves in their home. Here, the role of the health sector is counseling on how to feed their children and for them. The gender affaires office if they can work on equity in service utilization from trainings and materials of both agriculture and health as well as on gender violence. Educational sector if they work on improving the awareness of women and the finance to bring all the logistic related issues and religious worker if they work on the cultural barriers in the community meaning if all the sectors work in collaboration we can create nutritionally secured community.

**I, 6.2 for the multi-sectoral actions working effectively, what kind of change in terms of the way the stakeholders work together is needed? any type resistance to the needed change do you perceive or experienced before?**

**P**, we have to strengthen the stream committee found at the worerda and kebele level which contains 12 different sectors. And to work more with educational sector at schools level; here even though we have done it with some schools still we do have a lot to do on both in school and out school learners. We need also to use educational channel of both in and out learners to educate on health related issues in collaboration with health, education and agriculture to improve adolescent nutrition. We should also need to follow both in school and out school adolescents whether they have practiced it practically in their home about the training given and expand adolescent schooling to all areas of our worerda. Therefore, if we able to work on all the strategies coming from the region and the country without neglecting; we can achieve our target. There are challenges particularly on the stream committee, we do have a constant meeting schedule but all the sectors do not avail themselves meaning there are missing and sending new people in to the meeting where this are new to the agenda as well there are also expert related negligence or giving less focus to the area but simply working the routine activities on both local and worerda level experts. The local stream committee, similarly there are absenteeism from both farmers and local agricultural experts by prioritize other there on activities. There is also a problem related with sectoral collaboration as the sectors focus on their own sector only but giving less attention to collaborative work but here there are also some promising things like performing some meetings during reporting.

**I, 6.3 does your institution participate in the multi-sectoral nutrition coordinating body at woreda level? to what extent?**

**P.** Yes, With health we have no any difficulty we are working very nicely and meet in different occasions but our main problem is with education sector as we do have limitations and difficulties even though there are works started and with gender affairs we are doing well. But education we need to work at school level and bring change as the students are not only benefited for themselves but their families as well.

We work with SURE project working at local level; they do have their own one focal person and other one person from agriculture office. We have done review meeting recently and they have found big gaps like what is happening at the worerda level there were lack of coordination and working together among the lower branches of the sectors or stakeholders. Therefore, even though we could able to create very strong coordination at worerda level it would not be effective if we are not able to produce good coordination and performers at the local level we will not bring the demanded change at the community. This is because the main pillars are the local experts who are working closely with the local community and identifies the farmers. We do have problems at worerda level related with supervision as well; we do not supervise the lower level experts per schedule and regularly to identify the strength and weakness at the lower level in order to work on the weakness and enhance the strengths. As an agriculture sector we have been working on health related with nutrition and we do this together with the other activities but on health sector there is one person responsible for nutrition only, hence this brings conflict of interest among the sector experts. The difference in responsibility and cost might be responsible for the collaboration as there are feelings like I am working extra-lead without payment where others are paid for it. Therefore, We need to give equal treatment and responsibility to the experts working on nutrition of all the sectors from agriculture, health and education

**I, 7. Do you have any other comments on anything that we have discussed?**

**P.** We have put three pillars like nutrition, climate change and gender equality. The main problem as an agriculturalist is lack of sustainable focus on all the prioritized issues starting from higher bodies; example on climate change we are working more than what is demanded by the region but still there are in and outs here as well. Similarly also on nutrition the main problem is lack of consistent focus and sustainable monitoring and evaluation. We have to identify the strengths and weakness so that we can build on strengths and work on our weakness. The other is giving training to farmers to improve their awareness on the need, why we work on nutrition as a centeral pillar so that we can bring the farmers in to the required level. To sum up, the interview is in one of the major issues or problem which is nutrition sensitive agriculture. Hence, agricultural sector should works not only in improving production but should also be balanced agricultural commodities. This could not be worked by agriculturalists only but should involve other stake holders like Mekelle University and other Universities in strengthening NSA in their localities.

The main thing is we have to work on mothers, if a mother had given birth but could not lactate her baby; there is no bigger mourning than this as any child has the right to lactate and get nutrient so everyone should take the responsibility and work on mothers. Therefore; I need to underline that to get good citizen with a leader ship capacity we need to work on mothers. We have to work also on adolescents in creating awareness as these are the bases because they can also change their families and are mothers for tomorrow. We can use schools, religious leaders and mass-media to teach those adolescents. We have to also work to avoid the cultural barriers of the community.

Multi-sectoral and stakeholders, we have worked before two years with save the children and Pop-Geldof on animal farming starting from hen farming and then upgraded to sheep farming and there are also mothers researched at cattle or cow farming. The mothers also able to feed their children to feed the balanced diet consisting of milk, house garden vegetables and egg. We have also done very nice job in students through school based demonstration, training and experience sharing. The same thing was done for farmers at FTC and we have created model farmers who invite us for evaluation and feed back; these were done in coordination with the health sector. There are works done through worerda based budget for collaborative sectors such as health, education, gender and agriculture on lactating mothers and changes had been registered particularly on child care. Now they feed their children from milk, egg, and vegetables.

We do have common plan and the stream committee which enables as to work with other sectors. We had also worked the last one year with AGP which works mainly on nutrition through capacity building in our worerda and there is also SURE program and we work also with them. The other is the safety net program who is established by stakeholders to work maternal nutrition. So above mentioned stakeholders are the opportunities for our success on some of our interventions but still there are works to be done. There is no any farmer saying it is not important for me when you provide the necessary information the problem is scarcity of resource.

**Summery points**

**Section one:**

Severe malnutrition, Anemia, Stunting and underweight are very common in this area but overweight is not the problem of this community.

**Section two**

Main nutrition priorities in this woreda are house gardening or vegetation, on improving the mineral content of the foods like boron, iron, zinc, and phosphorus which is reflected on bone abnormalities, by changing the content of the soil particularly using fertilizers.

**Section three**

On pregnant women they will be followed on all aspects of their health but coming to agricultural sector we work on safety net program like if they are members of safety net program we will make them free of work and let them take rest. But concerning other health related issues we will follow them communicating with the health sector particularly on feeding habit and types of exercise but mainly we focus on resting and giving first to them. There is also nutrition screening practice and all the materials are introduced in the health service.

**Section four**

There are problems related with mothers like they select interventions themselves among all other interventions even though the interventions are equally important to them. There are some barriers related with the community like problem related with eating of fish; they believe that it smells. Other believes like eating vegetables are related with poor people.

**Section five**

Use of exemplary girls who denied early marriage and live with their relatives at cities and continued their education; now they have finished their schooling and are at a very good condition. Therefore; we can use such girls as models or best practices to teach the community practically or though video to improve health outcomes of mothers and adolescent girls. We do have around 28,000 students and if we use these students to educate about nutrition and early marriage we can address not only the students but also we also address their family as well.

**Section six**

Multi-sectoral and stakeholders, we had worked with save the children and Pop-Geldof on animal farming starting from hen farming and then upgraded to sheep farming and there are also mothers researched at cattle or cow farming. There are also works done through worerda based budget on collaborative sectors such as health, education, gender and agriculture on lactating mothers and changes had been registered particularly on child care. Now they feed their children from milk, egg, and vegetables.
