# Supplementary material for: Barriers to utilize nutrition interventions among lactating women in rural communities of Tigray, northern Ethiopia: An exploratory study
Source: PLoS One. 2021 Apr 30;16(4):e0250696. doi: 10.1371/journal.pone.0250696 (PMC8087028; doi:10.1371/journal.pone.0250696)
Supplement: S2 File — (ZIP) [file pone.0250696.s002.zip › S2_File.Doc/Woreda level and above key informants/003_IDI_women affair_Ofla woreda .docx]

**Operational Research on Adolescents and Maternal Nutrition in North Ethiopia**

**Tool A**

**IDI with Women Affair Ofla**

***Section A: interview details***

1. Zone: South
2. Woreda : Ofla
3. Kebele:-------
4. Name of Key Informant (KI): Aster Niguse
5. Institution of KI: Women Affair of Ofla
6. Interviewer name: Yemane G/mariam
7. Date of interview: Nov 03, 2017
8. Interview start time: 3:17 Morning LT
9. Interview End time: ***4:58 Morning***

***Section B: Interviewee Professional Information***

The head of the Women Affairs was Female with age of 35 and BSc in Agricultural Economics having a total of 12 years experience with 3 years of experience in the current position.

***Section1: Common maternal (pregnant, lactating women and adolescent girls) nutrition problems in the community.***

***I: What do women do to stay healthy in this community/woreda?***

P: Thank you, If women need to be healthy, there must be change in feeding practice; the farmer has vegetables, cereals and crops. Regarding to maternal and adolescent nutrition previously our farmers was selling their production to the market but now they didn’t sell it. Previously Mothers didn’t not prepared foods for pregnancy rather they focused to prepare foods for lactation period but now women didn’t sell their production to the market rather they prepare food with different composition of vegetables, cereals, fruits, crops and other with respected amount of ratio. The change was due to the involvement of Health extension worker (HEW), women development army (WDA) and pregnant conferences on feeding practice of the pregnant and lactating mothers. A total of 21 kebeles are now involved in nutrition programs which focus on feeding practice on frequency and amounts to be taken during and after pregnancy but we could not say we have fully improved the feeding practice.

The nutrition focus is given to mother and under 2 years child but regarding to adolescents we didn’t yet give an attention on their nutrition, simply they eat what their family eats the routine ways.

**I: What are the Common nutrition problem among women and adolescents?**

P: Malnutrition is common among women especially on pregnant and lactating mothers. For example if a pregnant woman was malnourished the baby would be low birth weight. Stunting and underweight are common among women in our community as well as anemia is common among adolescents due to early marriage which lead to uterine rupture, bleeding and fistula as complication.

Even if there is enough food like hen, goat, sheep, milk and eggs mothers and adolescents are not feeding them as expected that helps to build our body.

I: Which groups of women are most affected by these nutrition problems?

P: Mostly pregnant and lactating mothers are at risk of nutrition problem because the pregnant mother is responsible for the growth of the fetus during pregnancy but didn’t eat additional amount of foods.

The lactating mother is responsible to feed the child but due to work load she didn’t eat properly and they harm themselves and the child, because once the child sleep she become very busy in home activities which lead her to workload.

**Section 2: nutrition priorities in the Woreda**

**I: Do you believe you institution involvement in improving maternal nutrition is necessary?**

P: Definitely it is necessary to be involved in the improvement of maternal nutrition. Since all nutrition programs are important specialty to women. A woman will become pregnant, deliver and lactate if not well nourished the next generation would be affected. Since a woman is the responsible one in creating the future generation, focus must be given to them to solve nutrition related problems.

Now we are working with different sectors focusing on children, pregnant and lactating mothers related to with nutrition intervention/programs in our woreda and we are ready to work with everyone who is interested to work with us.

We are working with health office on child feeding, with women league we work on Pregnant women specially on ANC, place of delivery, immunization, feeding practice, post partum follow up using the WDA and HEW.

I: What are the maternal nutrition priorities in your woreda?

P: We don’t have a big deal with adolescent nutrition since they can eat as their families eat. As I have said previously since we need to create a better generation maternal nutrition is our priority we would like to work more on feeding pregnant and lactating mother.

In our woreda all women are networked with their respected developmental army. Among the intervention we used demonstration on each health centers and kebeles, on how to prepare porridge using the composition of food (diversification of food) using different ratio or proportion of vegetables, fruits, cereals, crops and others by using pregnant women conferences and nutrition day.

We give most of our time focusing on feeding of child, pregnant and lactating mothers. Now a new nutrition program called Sustainable Under nutrition Reduction in Ethiopia (SURE) is introduced in our woreda. Currently we are working with different sectors like health, agriculture and community police and we are ready to work with any sector if we get capacity building and supports to promote the nutritional status of child, pregnant and lactating mothers.

As women affairs office we are working on linking women association, women league and other sectors from woreda till the community level on supporting child, pregnant and lactating feeding by using community mobilization even though sometimes we are busy in meeting.

We are partners to other sectors as you we focuses on women benefits from education, agriculture and health. Specifically we focus on nutrition of child, pregnant and lactating mothers by creating community awareness even if very difficult to bring change immediately. Husbands are now supporting their wife by taking to health facilities and fulfilling foods in the house.

As well we have to fight in creating awareness to fully implement the nutrition intervention on pregnant and lactating mothers. When we come to resource allocation 50% of the Woreda budget is planned to support for women in the Woreda for different intervention especially on health and agriculture but we don’t have any budget for this from our office.

I; Can you tell me successful maternal nutrition intervention implemented in your woreda?

P: Regarding to nutrition intervention we are highly working in creating community awareness about maternal nutrition, as I have said even if it is very difficult to bring changes as we need. In addition we provide demonstration, manuals on feeding practice about pregnant and lactating mothers on the preparation foods to the community in collaborating with health, agriculture and others sector.

**Section 3: Nutrition interventions that improves adolescents and maternal health**

**I: What are the nutrition intervention in place to improve maternal (pregnant, lactating and adolescents) nutrition in your woreda?**

P: During pregnancy a mother will be advised to visit the health facilities for ANC, during and after pregnancy, advice on feeding practice to at least one extra meal three times even four times per day as well as increasing the amount to be eat twice of the previous. We use pregnant conferences which held every month using experience sharing among the mothers.

We advise her to go to health facility for examination whether the baby is in good condition in terms of position, heartbeat, growth normal, or not and to screen for other consequences even if this is done by the HEW.

In addition we advise the mother on sources of carbohydrates (energy giving food), protein (body building) and vitamins to have safe and normal baby which will not be easily affected by stunting.

During lactation we advise her to have EBF for six months and to have more feeding practice as compare to the pregnant women by taking soup, porridge and other foods since the baby gets food from her. And she must start complementary food after six months till 2 years using proportion of different cereals, crops, vegetables and others which is given by the HEW.

She will be advised on full Immunization for the child till nine month and counseling on family planning after post natal to chose whatever she likes. ITN must be used during pregnancy and lactation, to use iodized salt after the cooking is ended.

But we don’t have special focuses on adolescents regarding to nutrition simply they eat as like to their family. Rather we focus on reducing early marriage, unwanted pregnancy and school attrition.

I; What are the **most effective nutrition interventions?**

P: We are effective on child feeding, reducing home delivery which leads them to have excessive bleeding even to death and good family planning utilization.

Related to nutrition we have implemented in all the 21 Tabiya even though it is difficult to say there is 100% achievement but as compared to the previous achievement it is good. This is happen due to awareness creation in the community using pregnant women conferences, WDA and HEW.

If the community perceived the issue women is the issue of the community and if the women have participated in their issue we can bring a lot of changes. The woreda has also focuses on women nutrition by assigning one focal person working on maternal and child nutrition.

I; what are the **less effective nutrition interventions?**

P: We have promising start on maternal nutrition but as women affairs we didn’t have close followed up on adolescent nutrition this could be our focus on adolescent is on prevention of early marriage and advice them on home they can support themselves.

Related to pregnant women we feel that we are not still effective in reducing home delivery. In addition we are not fully implemented maternal nutrition due to lack of awareness or less focuses to eat even if they have enough amount of foods in their home. The pregnant mother don’t eat balanced diet during pregnancy time rather they prepare balanced diet food for her delivery.

For home delivery the geography is difficult to reach health facility, delay at home, delay at transportation, delay at health facilities, those who were not following ANC are more at risk but this is controlled by close follow up of the WDA and HEW by recording reporting the number of pregnant women.

I: what are the Challenges to implement maternal nutrition intervention in your woreda?

P: Regarding to training if the woreda allocate budget and training is given a lot of improvement will be achieved but there is lack of training on maternal and adolescents nutrition and those who trained must train the other staffs and focuses were not given due to other priorities like to make the farmer more productive, empowering economy, improving agricultural production and seasonal work but focuses on maternal nutrition should be given at all level. We don’t have any problem related to coordination of different sectors.

The farmers were selling their products to the market but we are working that the products should be consumed at household level unless it is excess product.

Regarding to ANC it has good acceptance, but to have a good relationship pregnant women prefer if the health professionals are female. The others are well implemented in our woreda related to maternal nutrition whereas we have challenges in implementing the adolescent’s nutrition since our focuses are on preventing violence, unwanted pregnancy and early marriage.

I: **What is the most successful approach that has used by your woreda to improve maternal nutrition service delivery?**

P: Even though we couldn’t say we have successful intervention but we have started and implemented a pregnant women conference which is scheduled every month and the participants are pregnant women in which they will share their own experience in their community. Using this pregnant conference we have bringing changes in pregnant nutrition improvement. For lactating mother we teach them on feeding practice when they come for immunization.

**Section 4: Community factors affecting access to maternal nutrition intervention**

**I: Can you tell me the barriers that affects or obstacles the implementation of maternal and adolescents nutrition in your community?**

**P:** Well, even if the 21 kebele or Tabia have transportation road it is very difficult geographically or topology for cars or ambulances to reach there and the mothers take too long time to reach to the health center and health post till they get transport. We have six health centers in our woreda but we have only three available ambulances unfortunately one of the ambulances is not working now due to accident. If ambulances are available in each health center they will be available in the woreda office. Therefore if any one calls either to women affairs, to health office we will send the ambulance to the community. The better one is each ambulance will be standby in their health center this will help to reduce delivery at home as well as delivery on the way to the health center.

Education is the main factor in the community that affects the implementation of maternal and adolescent nutrition due to low awareness since most of the community members are illiterates. These results in resistance for ANC follow up since they prefer female health worker than male.

And delays to the health center during labour is common due to her families recommend her to stay the labour is not yet strong. Workload and negligence are common in our community especially the pregnant and lactating mothers. They prefer to drink a cup of coffee than cooking her food. Even if she is busy in preparing food for her husband and her children she doesn’t eat with them properly.

I can say 90% of the health care is good but when mother come later than their appointment due to workload and the health workers may shout at them for their delay this may create dissatisfaction among the mother and may not come again.

**I: How can we improve the barriers on maternal nutrition?**

**P**: Our community should create sense of ownership that every service in the health facilities is mine. The same is true health worker should respect the community and must create awareness and involve the community like maternal, women and the farmers in the nutrition programs. For lactating mothers they must come based on the appointment and schedule to get the services whereas the health professional should understand and respect that she may miss the day and schedule. If any health worker disrespects the mother in the health facilities she will never come back. For adolescents we counsel them on family planning methods and advantages as well as the disadvantages by approaching them like friendship. The HEW asks for the adolescent when was her menstruation and check for pregnancy based on the result counsel her on how to use family planning

**Section 5: Other interventions that influences adolescents and maternal nutrition**

**I: What is the importance of birth spacing and marriage above 18 years in improving maternal and infant health?**

**P:** If women married below 18 ages since her uterus is not matured to carry the fetus as well as she gets difficulty during labour and may develop complication like uterine rupture, bleeding and fistula. If the woman is greater than 18 years she can get pregnancy and normal delivery but if it is less than 18 years it is very difficult for her. Therefore the marriage age preferred if the woman and the man is above 18 or 20 years age.

We are working with different sectors called stream committee namely health, police, justice, and women leagues that meet together to discuss on current issues. For example now January is coming in which a lot of wedding ceremony are conducted, so we teach the community about unnecessary wastage of resources for the wedding and the limit age of the women must be above 18 years. If we have budget we give training in our office for the community about early marriage but we face challenges on shortage of budget to give the training in Tabias or kebele for the community even though we believe this is very important.

We are working with community police, WDA and HEW that help us in controlling the early marriage. Previously the approval from the tabia but now we are following the vital event registration and she will be checked for her age if the age above 18 the wedding will precede if not it will be stopped by the police. In addition the WDA and HEW report to us if there is any marriage in the community.

Previously the community passed the wedding by naming as “Tsebele” or saints holyday like Trinity day, St. Marry and St.Micheal if the she is less than 18 years. In order to avoid this HEW and WDA will report to us if such ceremony or early marriage is going on.

**I: do you think preventing early marriage and birth spacing is effective in your Woreda?**

**P:** Yes of course, it is effective because we are close following early marriage and birth spacing. As we know early marriage is causing for high attrition rate from school due to early marriage and early pregnancy. Even though the marriage is above 18 years still she may terminate her school due to pregnancy and birth. Therefore we teach them to use family planning by using our structure and ourselves wherever we get an opportunity during community meeting or any other events.

Even though we are working on reducing on early marriage still now we couldn’t work more on reducing the school attrition rate since once the marriage is happened the families urges or forced her to have birth which lead to termination of the school.

We give advice the couple to use family planning and continue their school though some of them accepted and some of them refused.

To reduced early marriage and attrition from school there are different clubs at school like girls club, boys awareness club and in our woreda we have 21 violence prevention committees consists of students, community elder and leader, religious leader and women is established in all Tabia or kebele .

At school level teachers have their own role in creating awareness for the community during parent’s day at school. Even if it is difficult to bring dramatically changes but through time changes will come.

**Section 6: Multi-sectoral collaboration to improve maternal nutrition**

**I: If your institution works with different sectors, do you feel it will improve maternal nutrition?**

**P:** Yes of course I feel it will improves maternal nutrition working with different sector, as I mentioned previously they help us in controlling early marriage, reducing attrition rate from school.

Our partners are education office, police office, justice office, water resource office and agriculture. We expects from health office to teach the community on maternal nutrition during pregnancy and lactation, ANC, early marriage and importance of education for adolescents.

From agriculture we expect to teach women on economic development and creating 50% of the women get advantages from the programs and budget. For examples if women have farmable land they will check whether she has used fertilizer or not and follow if she has becoming productive at the end of the season.

From water resource management office we expect to introduce new technology to the community especially for women on using of biogas, solar and water technology.

From police and justice office we expect them to prevent any violence, conflict and harassment on women and we will work with starting from the bottom to the top level structure that already exists in our woreda.

Finally we will evaluate the work we did with our partners whether our work has secured the benefit of women in our woreda.

Currently they (parteners or sectors) are working effectively with us since our government has given focuses on the maternal nutrition. Health office has helped women to utilize health services properly, agriculture office advised women to feed themselves properly based on what they produced rather than selling to the market.

**I: Can you share me what lessons has learnt regarding maternal nutrition and adolescents in your woreda?**

P: We are working on the benefits of women, pregnant and lactating nutrition in our woreda.

More focus is giving for women nutrition by working with different stake holders and sectors to empower women on economic development and political involvement because it is very difficult to have successful achievement in all achievement unless women are not involving in the process. Sense of ownership should be created by the community that without women involvement it is very difficult to be successful and they have to be benefited 50% of the programs budget.

Adolescents should be involved in community demonstration, in school as well as in their home in creating about maternal and adolescent nutrition.

When WDA or HEW are demonstrating feeding practice to the pregnant mother since the adolescents also come and participated in the demonstration this helps adolescents to demonstrate their skills in their home by helping the mother. Now we have assigning one focal person from each sector.

We have awareness on maternal health as women affairs what must be do for women off course we didn’t give much focuses on adolescents nutrition in which are going to work on it. As we know these changes were not come dramatically they passed a lot of challenges to work on. We may have any gaps during the implementation process we will try to improve them.

The opportunity in our woreda related to pregnancy and lactating mother nutrition is mothers who have used from you the benefits will be used to teach for the community about the importance of following ANC, institutional delivery, PNC and feeding practices.

**I:** Can you share me what lessons has learnt regarding multi sectoral collaboration of nutrition in your woreda?

**P: Yes,** We have good coordination within the different sectors that is why we have assigned one focal person from each sector to nutrition intervention committee in our woreda. As women affairs we are highly involved with different sectors related to women, pregnant and lactating mothers.

It doesn’t mean the focal person is not the only responsible one related to maternal nutrition rather we close follow what is going on the ground and we provide them support.

Since we are working with the health sector we have get awareness on pregnant and lactating mother’s services like ANC, Feeding practice, during labour examination, PNC, child feeding, immunization and breast feeding are obtained due to working with health sector.

I: Dear Aster thank you for your time taking and discussion, I have learnt a lot from your discussion thank you again. If you have any concerns you can contact me any time take my phone number (my phone number given to her).

Thank you very much for your time and information

Summary

Section 1: To stay healthy women are involved in different community demonstration, get advice from HEW and WDA about nutrition, hygiene and how to become productive. The common nutrition problems are malnutrition among pregnant women since they care more for after delivery than during pregnancy.

Section 2: The woreda give more attention for pregnant and lactating mother nutrition while there is less focuses on adolescent’s nutrition.

Section 3: To improve maternal nutrition 21 of the Tabias (kebeles) in the woreda has implemented nutrition intervention where as it doesn’t more focuses on adolescents nutrition.

Section 4: Transportation, topology or geographical, low awareness of the community due illiteracy, lack of ambulances and poor approach of health care providers are some factors that affect the implementation of maternal nutrition.

Section 5: In the woreda early marriage and early pregnancy are common which lead to high attrition rate from school as well as affects the nutritional status of the mothers since the adolescents are already ignored or forgotten in the adolescent’s nutrition.

Section 6: The woreda works collaboratelly with different sector in working with maternal nutrition where as not working with an adolescent’s nutrition. Rather on adolescents issues like early marriage, reducing violence and counseling on family planning they are working good even if they are unable to reduce high attrition rate from school.
