# Supplementary material for: Barriers to utilize nutrition interventions among lactating women in rural communities of Tigray, northern Ethiopia: An exploratory study
Source: PLoS One. 2021 Apr 30;16(4):e0250696. doi: 10.1371/journal.pone.0250696 (PMC8087028; doi:10.1371/journal.pone.0250696)
Supplement: S2 File — (ZIP) [file pone.0250696.s002.zip › S2_File.Doc/Woreda level and above key informants/008_IDI_head for Youth Office_Offla_Woreda.docx]

**Operational research on Adolescent and maternal nutrition in Northern Ethiopia**

**In-Depth interview with Head of Youth Office**

**Introduction**

Thank you for completing the informed consent form and for taking the time to speak with me today. I have several questions to ask you that I have prepared in advance.

If you have any additional questions or comments as we do the interview, please feel free to share them with me.

| **Section A: Interview details**   1. Zone: **Southern** 2. Woreda: **Offla** 3. Kebelle 4. Name of key informant: **Mr. Solomon Tumey** 5. Institution of key informant: **Offla woreda** **Youth & sport affairs office** 6. Interviewer name: **Abate Bekele** 7. Date of interview: **2/11/2017** 8. Interview start time: **03:47PM** 9. Interview end time: **05:08:48PM** |
| --- |
| Section B: Interviewee professional information   1. Gender    1. Female    2. **Male** 2. Age: **38 years** 3. Highest level of completed education.    1. College education    2. **Bachelor degree**    3. Master’s degree    4. PhD 4. Current position: **Head, Youth and sport office** 5. How long have you been in current job/position:    1. ______ Months    2. **___08___** years |

**I:** Interviewer **P:** Participant

**Section 1: Common maternal (Pregnant, lactating women and adolescent girls) nutrition problems in the community**

**I: What do women do to stay healthy in this community/woreda?**

**P:** In our woreda to be healthy, first the government has given special attention to women. Therefore, making women to feed balanced diet, and to follow the health check-ups like vaccination are the main works of government. At women side, as community member, to be healthy, they are using balanced diet and working to prevent themselves from disease as programs designed by the government. The government has been working on pregnant women by promoting them to have health facility follow-ups starting from the pregnancy, therefore mothers have been learning on important health topics by health professionals. Thus, majority of pregnant mother follow health care so every pregnant mother is following health care as recommended. Therefore, the majorities are doing what has been recommended by professionals like visiting health facilities for medical check-ups. Actually, I am not health professional to tell you in detail about health check-ups that women in our community are doing. But, I know the pregnant women should do exercise sometimes, they are visiting for screening about hypertension, not working heavy works, and counselling about healthy practices. For this, women in our community are visiting health facilities as these things would prevent complication during delivery.

**I:** **What about lactating women do to stay healthy in this community/woreda?**

**P:** I am telling you about what is done by the government and women in our woreda. The majority (more than 95%) of women of this woreda either they be pregnant or lactating, they have health check-up follow-up visits to health facilities. For example, until 06 month as the child should take only breast milk, and then after the child needs the breast milk together with additional food, and the women has been informed to feed the balanced diet for themselves. So, the women of our community are practicing these. Based on their level of income, they are also educated to use balanced diet. But there are challenges of using balanced diet in the community.

**I: What are challenges that hindered the women to use balanced diet?**

**P:** However, not only in those women who have low income but also in those women that have surplus crops/income, using balanced diet is poorly practiced in our community. For example, if they have high wheat production, they use wheat for consumption in the morning, lunch time, and nights though they can sell some wheat and buy other food groups to have feeding of diversified crops. So, I cannot say there is no problem of such food diversification in our community rather there are awareness problems. And, such habit is practiced in both low and high landers of our community especially in the low landers the problem in using balanced diet for all age group such as children, women and men is worse because they have no surplus production. To solve such problems the government has been planned and developed programs though there are limitations on implementation.

**I:** **What about adolescent girls do to stay healthy in this community/woreda?**

**P:** The adolescents in our woreda have duties in their households like fetching water, providing “enichet- local name for materials used for cooking, cooking and go to school. So, our woreda adolescent girls do have duties similar with other woreda girls.

**I: Do the adolescent girls do any activities to be healthy?**

**P:** Basically, in our community, people go to health facility if and only if they get sick/ diseased. Otherwise, adolescents are spending their time in working to their families and they don’t have any program or plan regarding to their health. Let alone adolescents the adults even are seeking health care/check-ups and want to visit the health facility when they get sick. Therefore, we don’t have a community that work with attention towards his health.

**I: What are common nutrition problems in the community for the women and adolescents?**

**P:** We do have 21 kebelles and all of them have no similar nutrition problems. For example, in majority of kebelles, there is surplus production but there is problem in using balanced diet. They have cereals and other agricultural products at hand but they didn’t use them as needed.

**I: What is nutrition related problems occurred due to lack of diversified food eating?**

**P:** In the low land areas of the woreda, there is problem in getting food throughout the year. Therefore, there have been nutrition related deficiencies in inhabitants such as children and adults. On the other hand, due to failure to feed balanced/diversified diet though there is surplus production, the high land area people were also affected by nutritional deficiencies. Regarding the clinical presentations, it would be better if the health professionals give you information on specific nutrition related problems in our community rather I am giving you the general issue that I experienced in my community. For example, there was drought in 2007 EC, there were nutritional problems like stunting in low land areas but in high land areas since the community is using only single food item there was nutritional deficiency problems identified in these areas with surplus production.

**I: What are problems related with food security?**

**P:** In our woreda, we have 21 kebelles, and in most of them there is increase in production and productivity. They have started to have surplus production so it good. But, the problem is still there in low land areas, 3-4 kebelles around Sekota border face drought as they may not get timely rain. Otherwise, majorities of our kebelles have no problem with food security.

**I: Which women groups are affected by nutrition problems that you have mentioned above?**

**P:** Most of the time, children were the highly affected groups. The pregnant were also the most affected because they need much as they should feed for both themselves and child. So, they were also among the most affected groups in our area. But, I don’t have any information the adolescents affected.

**Section 2: Nutrition priorities in the woreda**

**I: Do you think it is necessary for your institution to get involved in work aimed at improving maternal nutrition?**

**P:** Yes, indeed. We as youth and sport office as you know youth/ young are from 15-29years women and men. At this age if the women have good nutrition, they will have good thinking ability. Therefore, if we are involved on work to improve women nutrition, we are preparing a good base for next generation. Therefore, it is important for us to get involved in this work.

**I: What roles does your institution can have if it involved in work aimed at improving pregnant women nutrition?**

**P:** Actually, we have been working on this. In this regard, the youth especially in rural areas where there are no modern transportations, they have prepared traditional ambulance to take labouring mother to health facility. And, we are providing education for adolescent girl to make them to be users of health services such as HIV/AIDS prevention, and contraceptives. For also pregnant young women we are also supporting them to have health check-ups. So, our office is working with health office to make women get appropriate health care and we will work together as it is our duty.

**I: What maternal nutrition related interventions are done in this woreda?**

**P:** The intervention is done on the mothers as they should take foods that are recommended for them especially the balanced diet. The women were measured for their nutritional status then counselling has given for them to feed balanced diet. Basically, they are advised to feed balanced diet prepared from locally available food items.

**I: In which group of women are these interventions focused?**

**P:** By the way, the interventions mainly focus on pregnant women. To be honest, we are not working for adolescent girls and these age groups are the forgotten issues. We are working in promoting them to finish their education, then after working to make them economically empowered and to create them work opportunities. Otherwise, we are not working towards improving the health of adolescents rather than educating them to protect them from unwanted pregnancy and HIV/AIDS. Therefore, we are not working towards improving the nutritional status of adolescent girls. Even the health sector is also focused on pregnant women; otherwise I haven’t seen effort made to address the adolescent girls.

**I: What are nutrition related works done by your institution?**

**P:** Nothing is done rather we focused on empowering women economically. May be, currently there is a work related with nutrition that has come through health office this year (2010 EC), we are the members of committee established in our woreda. But, before this year, nutrition related works are done by health and education sectors. Otherwise, the youth and sport office has been only working to prevent the sexually transmitted infections and HIV/AIDS.

**I: Please tell me about the nutrition committee that you are taking part this year?**

**P:** We are not yet started the actual work now. There is an NGO that has come to work on nutrition and we are the technical committees. The work is aimed at improving the nutritional status of women. But, we haven’t started the work but there was a single meeting and I was not the member but our vice head is a member of the committee. So, he may tell you a detail about it. Overall, we are not working on nutrition.

**Section 3: Nutrition interventions that improve adolescent and maternal health**

**I: What kinds of nutrition interventions are in place to improve adolescent and maternal health in this woreda?**

**P:** Regarding nutrition, by identifying various items like vegetables, sorghum and others, there is food prepared given for children as porridge. For pregnant women, there is work done to promote feeding of balanced diet by these women based on what they have at hand. Education on importance of balanced diet has been given for all people but especially this work is done with special attention for pregnant women. But, there is no work focused on nutrition of adolescent women in our woreda.

**I: What specific nutrition interventions have been done to improve pregnant women health in this woreda?**

**P:** For pregnant mother, there is counselling on what is recommended for her like how to be healthy, feeding, to have check-ups for herself and foetus. Therefore, there are the pre-delivery services and post-delivery services like vaccination for her child and follow-up of her general health status that should be given for pregnant women. The pregnant women are also screened for nutritional problems and there is intervention if any problems of nutrition happened. So, these are what I know.

**I: What specific nutrition interventions have been done to improve lactating women health in this woreda?**

**P:** It is about the child feeding, as a child should feed only breast milk until six month then after additional food is needed. In prenatal and postnatal time good works have done to improve health of mother and her child. Otherwise, I have information gap to provide you detailed activities done on mothers.

**I: What specific nutrition interventions have been done to improve adolescent girls’ health in this woreda?**

**P:** Nothing

**I: In your opinion, which of the above programs are being implemented successfully?**

**P:** In my opinion, the nutrition works done at prenatal and postnatal period are most effective because there is comprehensive monitoring of mothers nutritional status from time of pregnancy until the child is age of 5 years.

**I: Why the programs you mentioned are effective?**

**P:** The pregnant women are checked for nutritional status as if the mother has nutritional problem then she will have unhealthy child and she herself can have health problems like hypertension. So, these all things are checked during follow-ups therefore the women will be healthy and have a healthy baby. For example, mothers with HIV are made to have healthy baby. But, in the woreda most of the nutrition related tasks are focused on children.

**I: In your opinion, which of the above programs are less effective?**

**P:** This is difficult for me to tell you, because I don’t have a look in this regard. I am telling this all from my exposures as a member of the community otherwise I haven’t done yet on nutrition.

**I: What are the issues that challenged you to get involved on nutrition related works?**

**P:** Now so far what I have answered for you are not because I am working on nutrition, but as a member of this community I trying to respond the questions rose as much as possible. My office has no program on nutrition, and we don’t have job position and no one is also asking us about nutrition.

**I: What are the implementation challenges that are specific for delivering the maternal nutrition interventions in the programs that we have been discussing?**

**P:** For example, regarding place of delivery, mothers are told to give birth at health facility then there are situations that these mothers could give birth at home. Regarding nutrition, there is lack of awareness in our community to feed balanced diet. Even mothers waste most of their time in works rather than caring themselves. They don’t give value for their health. But, in low land areas there are shortages of food items due to lack of adequate agriculture productions. For me, the main reason for not using balanced diet in this setting is lack of attention.

**I: Are there any competing priorities for not giving attention about nutrition?**

**P:** Yes, there are. Having multiple harmful traditional ceremonies like weeding and preparing memorial festival for their lost/died family members. Then they finish their products once that were planned for long time. Therefore, this is the main challenge that prevents households from feeding balanced diet in our woreda.

**I: Is there lack of awareness on nutrition related problems?**

**P:** Exactly, this is the main challenge again. They community is ready to go to health facility when they get sick.

**I: What are the reasons for not working on adolescent girls’ nutrition in your woreda?**

**P:** In our level, we don’t have duty given to work on adolescent girls’ nutrition. It may be due to our field of specialization as majority of our staffs are graduates of management. So, they mainly have a capacity to mobilise and organise the youth to be productive. Nutrition is basic for the health of everybody; therefore those youth who don’t have health cannot lead their country in the future.

**I: What should be done to your institution to work on adolescent nutrition?
P:** We don’t need new structure, rather strong collaboration on nutrition with heath sector is needed to work on nutrition. We can work on creating awareness for adolescents about the importance of nutrition by mobilizing them. So, we will have healthy youth. So, the main problem was lack of attention in our side and failure of other higher levels/officials to give us adolescent nutrition as a duty of youth office.

**I: Do you think working in collaboration with other sector will help you to work on adolescent nutrition?**

**P:** Yes, already we have young girls and most of our community are rural inhabitants so majority of youth are also participated in the agriculture. So, they can support the community and promote nutrition. Again to create awareness for adolescents about nutrition, we can easily mobilize the youth and then the health sector can provide nutrition education. Therefore, if agriculture, women affairs, health sector and others will unit and work together we can address nutrition in adolescents.

**I: Which of the challenges for implementation of nutrition interventions for women mentioned above are most important?**

**P:** Lack of awareness of the women on importance of nutrition is the main challenge. There is problem in changing what they have advised in to practice.

**I: Why awareness is lacking in your community?**

**P:** Provision of advice to women is not enough to create awareness and change it into practice; there is gap from the professional’s side as they failed to critically monitor and support the community.

**I: What challenges have you faced on advice on antenatal care services?**

**P:** We don’t work on such aspects skip this question.

**I: For aforementioned challenges, can you tell me any successes that the region/ woreda offices have used to improve maternal nutrition service delivery?**

**P:** In our woreda, majority have adequate crop production, but in some low land areas there limited resource. Otherwise the high landers even didn’t use balanced diet. So, such problems are reducing today due to continuous education (i.e. there are improvements). Majority (>95%) of pregnant women follow antenatal care and these mother were given education about nutrition. For low landers, as I said there were problems of inputs. For this, the government has introduced production safety-net program to increase food security though there are still challenges. To increase the production and productivity we are implemented agriculture via irrigation. We are also working to educate women to create awareness on nutrition.

**Section 4: Community level factors affecting access to maternal nutrition interventions**

**I: What are barriers that prevent adolescents and women from using the programs and interventions that we have discussed?**

**P:** In our woreda, access to transport, lack of access for roads from kebelle to kebelle), distance from health facility as there are settings which are 3-4 hours far from health facility, lack of awareness and attention. The government sectors are also have no attention towards adolescent nutrition rather they are focused towards maternal and children nutrition. For example, failing to give nutrition as one of sectors duty is the main barrier.

**I: How can these barriers be addressed to improve maternal nutrition in the community/woreda?**

**P:** Regarding lack access of road, to improve maternal nutrition in the community, we do have women development army. These groupings of women should be strengthened as if we are working in each woman groupings; indicates we are working on each woman. And, to teach women, the males also should know what has to be done for maternal nutrition so that they can support their women. So, awareness on nutrition should be created at both women and men to successfully implement women nutrition. Regarding transport, already the government has started to work on access to transport through community participation to construct road from kebelle to kushet (the smallest administrative unit). So, this problem can be solved in through such community participation.

**Section 5: Other interventions that influence adolescent and maternal nutrition and health**

**I: In your opinion, why would increasing the space between each births and delayed marriage improve maternal nutrition hence both maternal and infant health?**

**P:** In my opinion, birth spacing has importance to help the infant to grown up with all necessary supports for his growth then in turn an infant be healthy and then he/she will be productive to his/herself and his/her country. But, if there is no birth spacing there may not be adequate support to children to be healthy and also the mother health is compromised because if she gave birth without spacing as supporting a child growth is too tough so that it compromises mothers’ health. Therefore, in my view, birth spacing has both maternal and child health benefit.

Regarding delayed marriage, if a woman is pregnant before 18 years of age then she is not matured to give birth therefore, both she and her infant will have a health problem. As she has no capacity to care her child, it would be difficult to expect good child care from her. So, such marriage again will results in health problems of both mother and her kid.

**I: What programs or activities promote increased birth intervals in this woreda?**

**P:** As youth office, we don’t give advice to get married before 18 Years. So, we recommend them to marry at above 18 years of age. We advise for birth spacing on regard with its economic importance though what we work is not adequate as there are short birth interval practiced at the grass-root level. However, the health sector teaches about the health benefits of birth spacing. But, there is no good monitoring of such activities at lower levels. There is also community miss-understandings like some think as “God has allowed me to give birth so why I am limiting birth?” From the religious perspectives, some women also refuse to use contraceptives like condom and others. There is also lack of awareness about importance of birth spacing.

**I: What should be done to address such misunderstandings and awareness problems?**

**P:** It needs adequate education for our community on its importance. It would be better to deliver education with religious and community leaders on the way they would accept our education.

**I: Can you tell me about any programs or policies in place in this woreda to prevent early marriage?**

**P:** Yes. By the way, practicing less than 18 years marriage is violation of law, so that those who did will be accused as per law. So, there are procedures to allow a marriage. And, the political leaders have also role in executing implementation of such laws.

**I: In your opinion, are these programs and policies effective?**

**P:** Yes, especially regarding prevention of early marriage as the policy has been implemented. On the birth spacing again there are programs and policies, but we don’t have problem of policy rather the problems of working bodies/ executives limited the effectiveness of the programs and policies. So we do have problems in creating awareness to our community about importance of birth spacing as responsible bodies are not working with attention. Otherwise, we have good policies.

**I: In your opinion, what could be improved?**

**P:** The community awareness can be improved with intensive education as we may not get effect without continuous education.

**I: What are any other opportunities to prevent early marriage and short birth spacing?**

**P:** The existence of policy is the first one. The second is presence of leaders/ executives that can follow the implementation of the policy.

**Section 6: Multi-sectorial collaboration to improve maternal nutrition**

**I: Do you feel it is necessary to your institution to work with other sectors to address maternal nutrition?**

**P:** Yes it is important. Because, as youth office, we do have young women and we can do awareness creation works on them. Since we are with them and we can create awareness on importance of nutrition for women. So, if the health supports us technically by providing nutrition education I hope we will be successful.

**I: Which other sectors do you feel are necessary to work with?**

**P:** The health, education, women affairs, agriculture as they are important to increase production and productivity, and social affairs are important. The water sector is also important as they work on supplying clean water as if there is no clean water there is no nutrition. It is also important to supply water for gardening of variety of crops.

**I: For multi-sectorial action that effectively works to improve maternal nutrition at all levels, what kind of change in terms of the way stakeholders work together is needed?**

**P:** By the way for nutrition, under AGN, there is ENGIENE project that different sectors except ours have been working together. It is to mean that, to solve nutrition problems if we focus on awareness creation works, I feel there could be change in nutritional status of women. We don’t have much resource problem rather than problem of utilising it. Therefore, if we create appropriate awareness at community level, the women affairs, the youth affairs, the education, health and the likes can take their share. Therefore, if we all work collaboratively, we can get improved achievements regarding maternal nutrition.

**I: What type of resistances to the needed change do you perceive or have you experienced so far?**

**P:** When we discuss here now, I felt that we have to focus on nutrition of adolescents because these adolescents will be mothers for the next time. There has been focus on nutrition of children therefore such focus has to be given again for nutrition of adolescents. So, it is to mean we have forgotten the adolescents and I would say, let us do for them now. On nutrition, health has been engaged but if the effort of youth office will be added it would be successful. So, it should be done collaboratively. Regarding resistance that might be faced, prioritising seasonal commitments for example when some assignments come from higher levels then you will miss nutrition and shift to that activity. Then, you may not work as per plan and schedule but you may come for reporting only.

**I: To what extent does your institution participate in the multi-sectorial nutrition coordinating body at the woreda level?**

**P:** Yes, we have started to work together and have made one meeting. Our office vice head is a member of the nutrition technical committee. They have planned and have prepared schedule to work together. But, we did nothing so far on nutrition together. But, we were working on traditional ambulance and disease prevention activities with health sector. Otherwise, regarding nutrition we did nothing so far rather what we have started this year.

**I: What needs to be done to improve the capacity of these bodies for effective coordination?**

**P:** Primarily, they should plan and schedule their activity. If they monitor and evaluate what has been done timely, they will be effective.

**I: Do you have any other comments on what we have discussed?**

**P:** By the way what we have discussed is a big agenda; the importance of working on youth about nutrition attracted me a lot because these youth are the next mothers that could give birth. Therefore, if we focused in these groups, it means that we are focused on children and mothers. So, this is a good work program so that as an office of youth I would say we can work collaboratively.

**I: What lessons have you learnt regarding adolescent and maternal nutrition in this woreda?**

**P: S**o far, in our woreda nutrition related works are mainly focused on children and mothers. However, now I have noticed working on adolescent nutrition is important.

**I: What opportunities do exist to promote multi-sectorial collaboration of nutrition in this woreda?**

**P:** We have the capacity to work on different aspects to address nutrition.

**I: Thank you very much for your time!**

**Summary**

1. **Common maternal (Pregnant, lactating women and adolescent girls) nutrition problems in the community**

Let alone adolescents the adults even are seeking health care/check-ups and want to visit the health facility when they get sick. There was drought in 2007 EC, there were nutritional problems like stunting in low land areas but in high land areas since the community is using only single food item there was nutritional deficiency problems identified in these areas with surplus production.

1. **Nutrition priorities in the woreda**

Overall, the youth office is not working on nutrition.

1. **Nutrition interventions that improve adolescent and maternal health**

Now so far what I have answered for you are not because I am working on nutrition, but as a member of this community I trying to respond the questions rose as much as possible. My office has no program on nutrition, and we don’t have job position and no one is also asking us about nutrition.

We can work on creating awareness for adolescents about the importance of nutrition by mobilizing them. So, we will have healthy youth. So, the main problem was lack of attention in our side and failure of other higher levels to give us adolescent nutrition as a duty of youth office.

**Section 4: Community level factors affecting access to maternal nutrition interventions**

In our woreda, access to transport, lack of access for roads from kebelle to kebelle), distance from health facility as there are settings which are 3-4 hours far from health facility, lack of awareness and attention by both the community and sectors.

**Section 5: Other interventions that influence adolescent and maternal nutrition and health**

Birth spacing and delayed marriage have both maternal and child health benefit.

**Section 6: Multi-sectorial collaboration to improve maternal nutrition**

It is important to work together to improve maternal ntrition.
