# Supplementary material for: Barriers to utilize nutrition interventions among lactating women in rural communities of Tigray, northern Ethiopia: An exploratory study
Source: PLoS One. 2021 Apr 30;16(4):e0250696. doi: 10.1371/journal.pone.0250696 (PMC8087028; doi:10.1371/journal.pone.0250696)
Supplement: S2 File — (ZIP) [file pone.0250696.s002.zip › S2_File.Doc/Woreda level and above key informants/027_IDI_Head of Women Association_Southern Zone_Tigray.docx]

**Operational research on Adolescent and maternal nutrition in Northern Ethiopia**

**In-Depth interview with Head of Youth Office**

**Introduction**

Thank you for completing the informed consent form and for taking the time to speak with me today. I have several questions to ask you that I have prepared in advance.

If you have any additional questions or comments as we do the interview, please feel free to share them with me.

| **Section A: Interview details**   1. Zone: **Southern** 2. Woreda: 3. Kebelle 4. Name of key informant: **Mrs. Serkalem Mearge** 5. Institution of key informant: **Southern Zone Women Association** 6. Interviewer name: **Abate Bekele** 7. Date of interview: **07/11/2017** 8. Interview start time: **11:20AM** 9. Interview end time: **01:09:24AM** |
| --- |
| Section B: Interviewee professional inrformation   1. Gender    1. **Female**    2. Male 2. Age: **35 years** 3. Highest level of completed education.    1. College education    2. **Bachelor degree**    3. Master’s degree    4. PhD 4. Current position: **Head, Women Association Office (Delegate)** 5. How long have you been in current job/position:    1. ______ Months    2. **___05___** years |

**I:** Interviewer **P:** Participant

**Section 1: Common maternal (Pregnant, lactating women and adolescent girls) nutrition problems in the community**

**I: What do women do to stay healthy in your community?**

**P:** I can classify what women do to stay healthy in to two. The first, they work in group and the other is individual work. For group work they have the so called development army (DA) comprised of 30 women. Among which the five are leaders. Among the five leaders one is leader of health. So, with in the DA comprised of 30 women, she monitors the group not to give birth at home, to prepare and give balanced diet for her children, to vaccinate her children and other many health services are oriented and organized by the leader of health. Regarding nutrition, we do have poster showing how to prepare food from variety of food items/products to feed her children and demonstrating others then again represent our community at the regional level.

**I:** **What about pregnant women do to stay healthy in this community?**

**P:** Out of the 30 DA member women, primarily the pregnant will be identified by the women representative of the health among DA together with health extension workers. For example, if DA has two pregnant women, then the health leader follows these mothers in terms of various health related activities such as she monitors whether these women are following ANC and PNC or not by going the health facility. And, to prevent death, she also follows the pregnant to not allow them home delivery. On the other hand, she also follows the type of food necessary for pregnant women to prevent foetal deformity as there are nutritional problems that lead to congenital malformations. Then, every two weeks, the DA have meeting and then the health extension worker (HEW) teaches the necessity of health facility follow up before and after birth. Even the members of the DA follow the pregnant mother to check whether she is making appropriate clinical follow-ups and to promote continuous follow-up. And, if the pregnant fails to follow appropriate clinical service she will be penalized by DA. Such action has been learnt well in this community. It is recommended that each mother should clean her village at 5km distance radius and it is highly recommended that all members of the DA should fulfil the all 16 packages of health extension program. So, failing to do so penalizes the mother.

**I:** **What about lactating women do to stay healthy in this community?**

**P:** They always do have screening for cancer to prevent health problems due to cancer. They also have clinical follow up after birth as what pregnant do. Overall, they are members of the DA and lead in a way what is mentioned above.

**I:** **What about adolescent girls do to stay healthy in this community?**

**P:** They are not members of the DA rather they are followed at schools and colleges. There together with women affairs there are staffs who teach the adolescents to prevent abortion, for example, many times this group face abortion, so before occurrence of such event, the staffs create awareness by teaching them, and in case when they face such challenge there are guiders that communicate with women affairs and then we together with health professionals create a means to perform safe abortion. Otherwise, they don’t have a group work like what is done by DA. But, they have their own grouping at respective schools. There is the so called girls club, they have teacher that teach them every week or two weeks to prevent drop outs due menstruation so that the school have modes (pads), contraceptives and other necessary materials are there and we follow them critically. For example, they have gotten education on how to use condom to prevent HIV to prevent loss of their life. They have also discussion sessions at school and at the community. They have also 1-5 networks and there they also help each other, but most of the time we give trainings on the challenges that adolescents may face quarterly at the school.

**I: What are common nutrition problems in the community for the women?**

**P:** There were nutritional problems like lack of adequate food source to consume at previous times in some women. But, currently, the government has given supports especially health bureau gives food items like spaghetti, macaroni, and there are supports given by health sector. Some women with poor living status have come to our office and we were linking them to health sector. May be the problem is worse at rural areas. But, in urban area, the women themselves, for example, if there is a woman with poor socioeconomic status in the development army, the members take part in sharing some support for such woman. So, they have a culture of helping each other. For example, when a woman gives birth, not the woman that prepares foods to be consumed at the postpartum period rather the members of DA prepare it. Even they care here until she deliver by going to health facility together and then after they give support in supplying what is needed at postpartum period. In rural areas, lack of food supplies, for example, most of the time we buy food items mainly from rural setting, the awareness is not that much good. For example, we use from them but they benefit other people but not themselves. So, they have big awareness problem on the importance of various food items, for example, a rural woman sells an egg and buy other food item rather than feeding that egg to her kids. Actually, about 80% (guess) have good awareness currently. However, the urban community have no utilization or awareness problem rather they might have no adequate income.

**I: How sever the nutrition related problems you have mentioned are among the women?**

**P:** We don’t have many people affected by malnutrition in our area. But, we have elderly women, and malnourished due to illness. Otherwise, social affairs helps such people at each kebele, we have also list of women who are very poor who were identified and supported monthly up to 400-600 birr and then also supported by rich merchants as they give wheat flour and oil. But, in other rural settings there might be problem in awareness and the village they are living sparsely populated so may difficult to help each other. In urban area, the women can reach and help their entire peer with in a day. But, in rural area, currently there is the so called safety-net program, so the women are helped by this program and therefore they are changing their lives. It is not aid, aid was stopped, but it is the payment for their work at the community.

**I: How sever the nutrition related problems you have mentioned are among the pregnant women?**

**P:** There might be pregnant women who suffer and they can be vulnerable for nutrition related problems. But, so far we didn't noticed a pregnant who suffered from nutritional problems. However, there are pregnant women who are reported as they are told having HIV unfortunately and facing psychiatric problem come to our office. Then we and the women affairs will work on bringing psychological calmness of such women. Otherwise, we didn’t notice any nutritional problems in our city. Even only one women was died due delivery within 3 years period so far.

**I: What are common nutrition problems in the community for the lactating women?**

**P:** The lactating women can have increased body weight, because most of the time those who were using contraceptives were said to increase body weight. Especially, those women who took injectable contraceptives were said to have increased body weight and again it said increase the weight is the nature of the drug. The women like it as they think increasing weight is luxury/comfort rather than considering it as health problem. But, I didn’t notice obese women that much in our area.

**I: Do you think that women/girls in this community are suffering from micronutrient deficiencies?**

**P:** There was goitre long ago currently it is not the problem. But, many women are suffering from anaemia. For example, when the women go to hospital there are women who need blood transfusion. Otherwise, currently there are only few who were undergone surgery for goitre in our area.

**I: Why do you think these women are suffering from Anemia and goitre?**

**P:** Goitre is caused due to unclean water use. But, anaemia is due to failure to feed adequate food items, for example, a woman might not get all food items as economic status differs across individuals, the women are thinking for only to fill their stomach but don’t consider the nutrient contents of the food they consume. So, many mothers do this, when they eat and fill their stomach that is ok for them to live and give birth at ninth month because they have limitations both on access and feeding behaviour.

**I: Do you think there could be any association between nutrition and occurrence of non-communicable diseases among the women/girls?**

**P:** Yes, for example, diabetes is said to be hereditary but most of the time risk factors for diabetes are feeding fatty foods that can cause diabetes in women. So, foods that could produce much energy/sugar should also be limited to prevent diabetes. If you care on intake you cannot be affected by diabetes. And not eating excessive carbohydrate prevents diabetes, for example, sometimes there are children who look like 18 years while actually they are 10 years. Even I know there children of rich households, that do like so. This happened in these children due to careless eating habit like they dislike eating vegetables so that they eat most of the time meat and fatty foods therefore, I can say it is due to fatty food consumption.

**I: Are there non-communicable diseases (diabetes, hypertension and overweight) in your community?**

**P:** Yes there are. Overweight is not common problem and there are only few who affected by obesity. The diabetes and hypertension are problems in our community. Even we do have hypertension that starts at early childhood.

**I: How much sever are these diseases in your community?**

**P:** The problem is now good unlike previous times because people are taking care of themselves when they consume foods, avoiding fatty foods as currently there is a good awareness at community level. So, the community understanding has changed and there should be some other intervention.

**I: What do you think on why women/adolescents in this community would not increase their height proportional to their age?**

**P:** Sometimes, there are dik-daka (very short locally) women that is due to malnutrition. Actually, they are not many, but 3-5 are there in their community. They are not only short but when you see they are very short. This is caused because poor feeding habit and the feeding problem of mother at time of pregnancy. Rather it is not due to physical attack. After birth it could be lack of body part. But we consider it as it is due to failure to eat what is needed at the time of pregnancy. So, that is why we counsel pregnant women to have healthy and mentally good baby. Currently, women think a baby born through caesarean section(C/S) is normal as the women don’t be stressed when they give birth through this route. But previously, giving birth through C/S was horrible for mothers even they assume it as death but now that is totally changed and need to have good and healthy baby. In the past, it was said to be “the child will be grown up by his chance”. Therefore, currently, the mothers are okay to give birth via operation. This much the understanding of women has been changed now.

**I: Do women/girls in this community increase their weight /height proportional to their age?**

**P:** In this regard, there is currently established center that is sport club which encompasses gymnasium to reduce weight and to shape body structure especially for youth. This done by support of government that provided the area for it. So, when people get a little bit fatty they go to the site to shape there body. So, currently there is awareness that being fatty is harmful and as it is disease. In the past, people think it as wealth but currently it is considered as disease. So, they want to reduce their weight rather than becoming fatty.

**I: Is there a situation when the community suffers from food insecurity?**

**P:** Yes there were problems in the past. There were youth girls who suffer, but nowadays since the government have made good opportunities by allowing loans mechanisms for this group. For example, when the girl come our office and if she is suffering from food security us together with women affairs we will solve immediately. There are many women that come to search a way to change their lives. In the past, there was no government structure to support women in capacitating the income generation, but currently immediately up on their request the government supports them. Even the process doesn’t finish one month and they can take credit up to 100000-500000 birr and then change their lives quickly. But, no women is facing challenge as what was in the past like when they lack something she was crying and stay at home and no women is currently facing hunger at home, and if we estimate a daily labourer women even can earn 100birr daily. We have been linking them with industries, universities, but there are elders who still need support and the government is supporting them. There is no safety-net program in urban areas but it in rural. But, these elders would be supported if they were in rural setting.

**I: Are you saying there is food insecurity in your community?**

**P:** Yes, even we (the government workers) are living a hand to mouth lives but not that much adequate to support our families. There are no people living in comfortable situation, but there are no people who didn’t eat at all even though there is variation in feeding style as someone can get variety of foods but the other didn’t.

**I: How frequent does the food insecurity problem happens?**

**P:** At summer time there are food insecurity problem especially in rural areas, because the rural community use food items from their agricultural products but at summer season it would be difficult to them to harvest and get food.

**I: For which one of the above problems do you think pregnant women are especially at risk?**

**P:** I don’t think many women are affected by the problems. For example, when we see the drought occurred in 1977 EC, the women were at hunger and they were pulling their feet, unable to talk, unable to see but currently we don’t have such nutritional problems I can be a witness. But, there could be variations, the living status of entire women were not same. In the past the women can get through hard works, after many up and downs but now, those are in trouble come and shout to get support. Otherwise, there was begging in this area currently. This is also an indicative of changes has become. There were people who beg even when they have good income. So, this begging has stopped otherwise the students of the church might beg. Otherwise, I haven’t notice a woman died of hunger so far though she might not have adequate income.

**I: For which one of the above problems do you think pregnant women are especially at risk?**

**P:** Most the time, though they have adequate food source, there are women who will become thin. Sometimes some might become too fatty and others become too thin. This is because the roughness in the relationship between them and their husband like disagreement, conflict and there could be other issues. So, we have been counselling the pregnant to not be stressed and complain, feed diversified food. And, in case when she has stress we invite her to come to ours. Most of the time, woman can have comfort at home but there are women whose husband harm them and that were committed suicide then died, and took poison. Most of the time pregnant women were fatty and in some women were thin. But the good thing is that there is a clinical following up starting from 4^th^ month of pregnancy. So, she will tell what she has faced daily to the WDA. For example, the five talks together and at every two month they will have meeting again with WDA. Therefore, the women themselves are participating in solving their problem. Otherwise, we can say the challenge has been changing/ solved so far.

**I: For which one of the above problems do you think lactating women are especially at risk?**

**P:** Most of the time, they become thin as this group give attention to health of their kids, and failure to eat the balanced diet. In our area, they become thin she cannot eat what she expense for her child through breast milk due to lack of adequate food supply and awareness. After birth a woman focuses on her baby as she doesn’t understand the risk of failing to consume appropriate food for her. So, this is way we are working on creation of awareness.

**I: For which one of the above problems do you think adolescent girl are especially at risk?**

**P:** Most the time, this group don’t want to be fatty, they want to maintain their posture well to keep their body structure. Otherwise I haven’t seen any other risks in adolescents.

**Section 2: Nutrition priorities in the woreda**

**I: In your opinion, what interventions do you think are priorities of your institution to improve nutrition for pregnant women?**

**P:** We (the women association) only work on formation of women groups (the WDA). Then, we encourage them to change their lives, and then to educate their children and have their own money pool. For example, we have built up team at university level currently. Then, women are working there by supplying enjera, and other supplies. So, this the way we support them. So, they cannot be harmed as they are generating income. So, we are working to change their lives through income generation, through creating awareness. We working in making they to generate their income and therefore they don’t want any support from government, rich and other individual. Even the women who were in front of us when we meet you were working production of poultry, and they were HIV patients. Therefore, the can sell the hens and eggs and generate income. They were asking to get further loan to generate much income as they faced challenges to buy some supplies. For example, in the past, HIV patient were staying and crying at home but currently they are forming group by 10-15 individuals, and generating their income. So, having HIV does mean nothing currently. So, all these patients are utilizing the opportunity and taking the credit up to 500000Birr. Therefore, they are changing their lives and we cannot expect they are harmed. Those harmed women have their way to handled. So I am not considering such issue for time being as the focus is maternal nutrition but it doesn’t mean there is no assault.

**I: What interventions do you think are priorities of your institution to improve nutrition for pregnant women?**

**P:** For example, there is a day called a forum of pregnant women, which is organized by women association, women affairs and health bureau in every month. We prepare soft drinks and others necessary resources for the day. Then they share their experience regarding how and what they were eating during pregnancy. Therefore, they support each other, for example, mother that may bleed tell the effect of it and its management to the women come to celebrate the day. All the women who celebrate the day are pregnant. Therefore, there is monthly celebration of this day at each Kebeles.

**I: What interventions do you think are priorities of your institution to improve nutrition for lactating women?**

**P:** For lactating, we teach on feeding their newborn like advising them to avoid bottle feeding, exclusive breast feeding till 6 month, using clean child feeding instruments, and such works are done by us. But, mainly we work again on the DA level as among the five leaders in the DA; one leads the health issue together with health extension workers, one works on education, one works on peace, and the likes. Each of these women follows their respective sector related issues/interventions. For example, the health issue leader woman, identify women who are pregnant, lactating, vaccinating and the likes at their DA. So, each of the five women of work in their respective sector like the education sector leader work on identifying mothers who didn’t send their children to school in their DA.

**I: What interventions do you think are priorities of your institution to improve nutrition for adolescent girls?**

**P:** To be honest, as these girls are educated and can learn nutritional problem through biology course, so occasionally our experts teach about nutrition sometimes. But it is not that much unlike what we have done for adult women because they are educated. Even we are still back to create the knowledge that the adolescents have regarding nutrition to women in DA. For example, the adolescents can learn and read and write hence can easily understand the nutrition.

**I: What nutrition interventions have the most resources allocated to them?**

**P:** The women association don’t have any budget rather it is freely working institution. The institution is free from any political commitments, any women who can be a member of any political party, for example, a woman who is not a member of current political party comes to our institution I should serve as what she want. It is a right for any women to use the benefit from our side. Therefore, we don’t have any budget as we are non-governmental but women affairs have its own budget as it is governmental organization. So, the women affairs mainly allocate budget on issues related with women and children because a woman who want to bear child should have protection for her life. For example, what is said by our late Prime Minister Mr. Melese Zenawi “No mother should die due to delivery”. So, not only us but every sector gives attention towards pregnant women health. For example, if we ask the DA about who is supporting them, the definitely say the health sector. The main topic of discussion for women is about health. And the DA army is organized for health works by women association as WDA is responsible to our organization. But we don’t have any allocated budget from the government, but we work with WDA together. For monthly holiday celebrated by pregnant women the budget comes by both the health sector and women affairs and sometimes the administrative.

**I: Do you think it is necessary for your institution to get involved in work aimed at improving maternal nutrition?**

**P:** Yes. We are highly supportive, for example, these women are directly linked with us and together with health we can easily deliver education to them. As woman is your wife, mother, sister and daughter everybody should work with us and we are happy. We are working to create healthy mothers and children and then to have good nation.

**I: What is your institutions role in work aimed at improving maternal nutrition?**

**P:** We organized women in groups called WDA. Therefore, not only health but all other sectors can schedule and work with the WDA. We supervise the WDA at regular basis, to sustain the groupings for example in Mayichew town there are 152 DA that comprised of 4560 women. We also facilitate transfer of experiences from one kebelle to another through the DA for example our women may go to Mekelle to get an experience we search funds from other partners.

**I: can you tell me some of successful nutrition interventions for pregnant women that you have implemented in this Zone?**

**P:** The highly successful intervention for us is there was home birth in the previous times, but it is stopped now. There was also death at home but now this has also stopped. Due to great efforts made to create awareness. There were also women who have nutrition problem but nowadays we have women who praised for their good work in Addis Ababa for their achievement in health. These women are praised due to their achievements in health and their nutritional status. Even we have such posters in our office that you can see their work on child and women feeding. What has been done regarding health in our area is reduction of home birth and death. There was time in which many women die due to pregnancy like 30/year but currently one maternal death has occurred per three years. But, much of nutrition related works are mainly done in children and little has done on maternal nutrition so far.

**Section 3: Nutrition interventions that improve adolescent and maternal health**

**I: What kinds of nutrition interventions are in place to improve adolescent and maternal health in this Zone?**

**P:** There are works that have started recently, on creating household that can feed diversified foods. There are works done at school level to demonstrate how to prepare diversified food. The students are demonstrated on how to prepare a porridge using diversified food items and then they taste it. There are works done by diversifying foods such as cabbage, spinach and flour. But, it is not that much expanded. There are also some women who have started implementation of such practices. However, sustainable use of diversified foods is not practiced yet. The initiative is led by the health sector and the DA and the administrators work together with health.

**I: Do pregnant women advised to visit HFs for check-ups and services?**

**P:** Yes

**I: What services do they get there at HFs?**

**P:** The have monthly appointments, they are screened for diabetes, anaemia, hypertension and psychological stress and all necessary check-ups have been done. Unlike the past times, in which the women were only palpated their abdomen then go back to their home but currently detailed clinical check-ups have been done. Currently, there is also screening for cancer, TB and other infection disease like acute watery diarrhea therefore a pregnant woman is told to clean her hands with soap before eating. They also advised to avoid heavy works and these are services given for pregnant women at HFs among many others. They are highly advised in many health related issues. Sometimes some women who cannot afford ultrasound come and ask us for support then we link them with doctors who provide free care for such women. Then they will be told whether there is position problem of the foetus and told to come early before a week of initiation of delivery to hospital to prevent complication. They also advised on feeding style. Unlike what is done in past times like only telling the estimated date of delivery but currently every check-ups and all laboratory test are done too pregnant women. That is why currently the maternal death is highly reduced. Nowadays, if there is any health related problems she will be told. However, for example, in past times, when a woman get pregnant of twins were not told as she have twin pregnancy because they thought as they may face stress due to multiple pregnancy.

But, currently, she has informed and get ready for what to be potentially done for her even she might be get ready for surgical delivery. This has occurred because efforts have been made in creating awareness on health related issues.

**I: Do you think that pregnant women receive advice on the need to get extra meal?**

**P:** Yes, for example, if she has anaemia, she will be advised to take foods that replace her blood such as foods and vegetables.

**I: Do you think that lactating women receive advice on the need to get extra meal?**

**P:** They also advised to take extra meal especially fluids as I said they get thin.

**I: Do you think that pregnant women get screened for their nutritional status?**

**P:** In the cities, there are women who try to check their fat level. But, in our setting when women visit HFs they used to have hypertension, haemoglobin, white blood cell count and the likes otherwise most of the time they don’t visit health facility for nutritional problems. When she faces reduced appetite she consults her doctor and takes medication unlike other big cities that used to have overall check-ups. Otherwise, the doctors themselves check pregnant women whether they are well nourished or not. Even the doctor gives the so called…… [Thinking]….hum, plumpy-net and other additional foods is given for malnourished women. Even they can be advised to prepare and feed some food type at her home.

**I: How do these check-ups would help for?**

**P:** For example, issues identified during delivery are fatal, so if she knows here sickness/disease early she can take care of herself. For example, if a woman has surgery while she has asthma that she didn’t know till surgery and then she may face a challenge. Likewise, if she has hypertension while she has undergone surgery, she will die immediately. Therefore, caution is very important. So, telling her as she has health problems early is very important for her life.

**I: Are pregnant women getting counselling for food diversification during pregnancy?**

**P:** Let alone hospital they used to be counselled at kebelle level in their WDA. They have been preparing balanced diet from various/ diversified food groups around 9 food items. And, this blended flour will be prepared and used by the women even after delivery. The DA members prepare the blended flour and cook it for pregnant women.

**I: What is the importance of using such blended flour?**

**P:** It has various uses for her body, as her baby need his food from her body. So, her baby will be healthy as he gets balanced diet from her body and she may not face excessive bleeding during labor, and improves her appetite.

**I: Are pregnant women getting advice for the need to use iodized salt?**

**P:** Oh the women know everything even more than us, for example, if we invite on or two members of the DA, they will tell you about everything even more than a doctor tells us. They know everything and they have gone even beyond our scope. For example, I don’t know whether the porridge can be included during preparation of porridge or not but a woman has to me that as it is possible to add cabbage in porridge because she has advised by the health professional. Among all other works done in our region the health work has shown significant improvement in our community.

**I: Are pregnant women getting advice on nutrition sensitive agriculture such as home gardening?**

**P:** Okay, for example, to grade woman as “A”, she should have various vegetables in her garden such as cabbage, spinach, garlic, and others. If the woman has such gardening and fulfilled all the 16 packages of the health extension program are considered as model and sore “A”. Unless a woman is living in rent houses, she should have gardening to be considered as “A”. They will not buy vegetables from market to be called as “A”. So, there is high competition among the 30 members of the DA. Even if they fail to understand it importance, they are doing it to become grade “A”. So they are using this opportunity.

**I: Which pregnant women need to be eligible for targeted supplementary feeding (TSF)?**

**P:** They were given blended flour, and oil. There is something that is measured and weighted to identify them. For example, the normal haemoglobin is 12-14, but if it is less than 10, and if they have under-nutrition and they can be identifiable. So they have such problems they are eligible for TSF. The tool to measure children nutritional status was something that is put on the arm [Showed on her hand]. But, for women it is by comparing pre-pregnancy weight with weight during pregnancy.

**I: In your opinion, are adolescent girls linked to youth friendly services at health facility?**

**P:** There is the so called youth-youth discussion. Most of the time, they discuss on abortion, HIV, and migration. So, such practices are there at health facilities. But it works together with youth office.

**I: Why linkage of adolescents to youth** **friendly services at health facility?**

**P:** Yes it is very important. For example, a girl may get infected with HIV and she may face unwanted pregnancy hence unsafe abortion. These all problems need the support from professionals at health facility. They can be advised to take care of themselves as they are in the fire age they may face several assaults and many others.

**I: In your opinion, which of the above interventions for pregnant women are being implemented successfully?**

**P:** I have said already, for me, in health aspect several successes have gotten. The maternal death reduction and what has been done regarding child nutrition are promising.

**I: Why do you think that it is effective?**

**P:** Because, for example you cannot look while your mother die and everybody when he/she gets a pregnant women the caution given for pregnant and other people are totally different. And, even you see the care given for mothers and children in the hospital it is different than the care given for others. There is strict follow-up and accountability mechanism like for example if something happened wrong on them, the doctors/professionals will be chased out from their career and may be accused. Therefore, the effectiveness is due to strong monitoring and follow-up. We have been discussing each and every point in the care provided for the mother when something wrong on her. But, the stunting, mal-positioning and congenital deformities like there are children born with lost body parts are still there that may need strong work. We consider the cause for such congenital malformation as nutritional problem and there is a need to focus on maternal nutrition. So, we are not effective in intervening nutritional problems. Otherwise, even the HEW will be penalized about 3-4 month salary if a mother gives birth at home as it is considered as death because nobody cared here. So, clear accountability mechanisms have put in place.

**I: In your opinion, which of the above interventions for adolescent girls are being implemented successfully?**

**P:** Regarding adolescents, as I said there are girls club at each school level and they thought on assault. There are also teachers who work with girls and the leader of the club is that teacher. The teacher supports at every aspect of their lives, for example, in the past, a girl that might be on menstruation and may leave class and she will be shamed and drop-out from the class. But, nowadays this has solved due to efforts made. At each school, there are rest rooms for changing modes (pads) and they discuss each other.

**I: In your opinion, which of the above programs are less effective?**

**P:** Regarding health service use the community has well understood the importance so I don’t think there is problem.

**I: What are the challenges to implement delivering the nutrition interventions that we have been discussing for pregnant women?**

**P:** The first, they didn’t have awareness, for example, when we promote consumption of diversified foods then they were not understanding it well and therefore they prefer to use only single food item. They didn’t know the importance of diversified foods to our body. So, this was a challenge. There are women preferring some food items due to they may not be comfortable. They were refusing to use contraceptives by saying “I dislike it” and they also were saying “the religious father has prevented here”. Actually, this has solved currently. There was also saying “St. Marry is in home but not in the hospital” to refuse hospital delivery. But currently, a woman clearly knows the consequence of home delivery. So, even at night, she goes with either ambulance or Bajaj to give birth at health facility to be free from the penalty and other rumours by her DA teams.

**I: How do you evaluate the resources available to the interventions on nutrition?**

**P:** In our side, we don’t have budget. What we need is for demonstration and we don’t want many resources to implement the intervention. Even for monthly women forum celebrations, the women themselves are sometimes promising to provide soft drinks as they like this day since it is aimed at discussion with each other. They have discussion about their overall health including that of foetal positioning on the day. They also debrief on the date of birth.

**I: How do you explain the awareness of pregnant women on nutrition related problems?**

**P:** Currently, they are good in understanding the importance of nutrition. They can identify food items helpful for her health. They know well but they may not get it when they only lack money to purchase it.

**I: How do you evaluate the priority given for the interventions for women?**

**P:** We are here to improve the use by women and enable women to generate their income. We are also working to change their lives and enable them to lead their household by themselves without any aid from the government.

**I: Are there any factors related to skilled care provider that may affects implementation of the interventions?**

**P:** There are no shortages of skilled man-power. Currently we have even more preferred and higher number of doctors. But, sometimes from rural setting due to distance and failure to refer timely, a labouring women may come after prolonged labour then when she reach the hospital she may die. This was a gap regarding the skilled professionals. Regarding nutrition related activities, there are small enterprise sector experts that can demonstrate how to gardening. And feeding style and food preparation is followed by HEWs. They educate and demonstrate about gardening and balanced diet preparation with in each DA. So, the women and the experts are highly skilled now.

**I: How do you evaluate the coordination and collaboration between the nutrition sensitive and specific sectors?**

**P:** I would say it is very valuable and we are effective when work together. But, most of the times we work together with health sector that is why we are effective regarding health related activities. Otherwise, others are still back and not engaged collaboratively. But, it designed that every sector should implement its activity with the DA. For example, if you are an agricultural expert then you go to the DA and there you can implement your activity together with WDA. So each sector (such as health or education and etc) work together with WDA at the community level. So, the women in DA are active participants of the work each sector has planned. The WDA are the back bone for each sector activity. Therefore, each sector goes to the WDA and support the implementation of respective activity.

**I: What other factors are inhibiting implementation of the nutrition interventions?**

**P:** We don’t have any problem. Sometimes we may have work overload, meaning if we go and support and show to the WDA they themselves can implement easily.

**I: Can you tell of any successes that your institution has used to improve delivery of nutrition services for pregnant women?**

**P:** With available extra time, even if they have their own schedule, additional support time were used to guide them. Political work is too difficult as we work in many aspects of women and there are many assaults, like I was planned to meet you in the afternoon, but I have gotten another commitment therefore I will go that in the afternoon. We again have responsibility to supervise HEWs. We evaluate and monitor them based on their performance.

**Section 4: Community level factors affecting access to maternal nutrition interventions**

**I: What are barriers that prevent adolescents and women from using the programs and interventions that we have discussed?**

**P:** As I told you before, there were community cultural beliefs that hinder them to use health service, but that are broken currently. Therefore we don’t have any community related barrier that prevents them from using health service. They say “I dislike health service and I don’t want to go there as I have setan”. To break such misunderstanding or beliefs we have gone through the religious life father - “locally called yenifes abat”- to tell her to refuse to her father again unless she birth in health facility. So, there was such a big challenge. However, currently we have no such case at all.

Otherwise, there are some husbands that don’t allow their wives to take contraceptives, those who cannot waiting the challenge that she will face if she get pregnant in short interval. This is the problem currently still existing. There are woman that take contraceptives concealed from her husband. To care themselves for not giving birth before 3-4 years of previous birth, they are doing such great effort. To halt this problem in case where there is very challenging husband we call him to our office and advise him on the risk of having repeated short interval pregnancy.

But, there are also good husband in other way even who are taking male contraceptives to plan his family. There is males contraceptive, in Adishu city currently, by support of NGO to prevent pregnancy. There were 6/7 males who had taken male contraceptive last year. So, we do have such a good husbands again.

**I: What are barriers that prevent adolescents from using the programs and interventions that we have discussed?**

**P:** Yes, there are some parents who prevent their daughter from getting health service when she became pregnant because they ashamed of what happened on their daughter. Recently, even I had my daughters friend who were pregnant at university and come back to home in which her mother didn’t let her out of her home even not allowed her to visit the health facility till the 7^th^ month of the pregnancy. Then I have taken her from Korem city to my home her Maiychew to take her to health service. Then she have gotten the care necessary for pregnant and delivery safely. So, the awareness of parents in this regard is poor. Meaning, we have community that don’t support their daughter when they become pregnant. Therefore, to keep their respect the parents let their daughter to go to other settings to give birth and the child comes as he is a child of other women. This process lets the adolescents for unwanted health problems including the psychological problem.

**I: How do you explain the educational status of the women and the girls in relation to using the services?**

**P:** The educated mothers are more likely to visit the service, but currently as there are educated mothers in DA they are all good in access the health service. But, the educated one has good visit to health facility as those uneducated one have still some cultural beliefs and misunderstandings like St. Marry is not in hospital, and if they fill their stomach by any food they feel as that is enough. In general, uneducated have low awareness than educated this is why they don’t visit as many visits as educated women.

**I: Do you think that there is connection between accesses of transportation with utilization of the services among women?**

**P:** There are problems of transportation in rural settings. But, in urban transportation is not a problem. In rural setting, there are hard to reach areas therefore the labouring mother come by stretcher. Then they may die before reaching the health facility.

**I: How do you see the quality of the services?**

**P:** Currently, the quality is good.

**I: What does the community beliefs look like towards the nutrition interventions for the women?**

**P:** There was problem in understanding the importance of feeding blended foods even in both literate and illiterate women. Even the rural women development army are better in understanding the importance and practicing it. They again cook by mixing food items that the urban and literate women don’t know. But, their problem is rather than consumption they prefer to sell.

**I: How can these barriers be addressed to improve nutrition among pregnant women in this community?**

**P:** Creating awareness for those who prioritise selling than feeding is very important.

**Section 5: Other interventions that influence adolescent and maternal nutrition and health**

**I: In your opinion, why would delayed marriage improve maternal nutrition hence both maternal and infant health?**

**P:** Early marriage can cause fistula. Meaning, it occurs in their urination tube due to early marriage. This in turn leads to psychological problem. For example, if a girl urinates without control over her body, she will become psychologically torched. She may face again many problems. Therefore, for those who are less than 18 years it is not recommended to marry. We also accuse those who committed early marriage through legal ways. It can also affect the nutritional status of the girl. She can have poor nutritional status so that she cannot care her baby well.

**I: In your opinion, why would increase the birth space between each birth improve maternal nutrition hence both maternal and infant health?**

**P:** Everybody knows the importance of birth spacing. For example, the child will grow in good condition and the mother herself doesn’t be harmed. She doesn’t face economic crisis. Many… many…things, the child grows well, she keeps herself well nutritionally, but if give birth yearly she will be harmed, can become thin, she can be easily vulnerable to disease.

**I: What programs or activities promote increasing birth interval in this level?**

**P:** At DA level, a mother is identified about type of contraceptive (whether it can be implant, injection or tablet) used. The women also assessed and recorded based on the type of contraceptive they are taking. But, if the husband is prohibiting his wife from using contraceptive we call and give advice. So, in this way the birth spacing is promoted. So, the contraceptive given is based on the woman preference. The HEW can inject it there at woman setting no need to go to higher levels. If she prefers Norplant, she should go to hospital to insert it through surgery.

**I: can you tell me about any programs or policies in this Zone to prevent marriage?**

**P:** Yes, if a girl is less than 18 years, it is totally forbidden to marry, and again the HIV test also should be done for every marriage. So, these are government’s policies.

**I: Can you think of political influences on early marriage?**

**P:** There is political commitment; even the governments’ attorneys are working on accusing those who committed assaults. If under 18’s want to marry, the attorney argues and prevents their marriage. So, we do have the protection by law to prevent early marriage.

**I: Can you think of religious influences on early marriage?**

**P:** The religious leaders are part of our team; we work together with Muslims, Christians, and catholic. So we have monthly meeting, for example, if a woman wants to go to holy water while she is taking a drug. Then she may refuse to take drug, then the religious leaders counsel them not to stop drug while they take holy water. There many such case, like HIV patient that refuse to take drugs, then we tell to religious leaders help us and the patients will be made to take both simultaneously.

**I: In your opinion, are these programs and policies effective?**

**P:** In my opinion they are effective because in their implementation is effective.

**I: What are the community factors that affect age at first marriage?**

**P:** There were community misunderstandings previously, but now they are not. However, sometimes there are cases that girls especially 16-18 years are married to husband because the husband wants to get her parents wealth. There was again culture, the parents can say as they promised to give their daughter to their friends’ son. But, currently this has solved and there is the so called the social affairs in kebelle levels to approve marriage after critical medical check-ups, and then if the health confirms as the girl is greater than 18 then the social affairs approves marriage. Then the boy and girl should sign agreement after again HIV test result. Therefore, there should be approval letter for marriage before weeding day. There are marriages that were cancelled even after a week of weeding day if the girl is less than 18. There are also kidnapping happened in around Raya this year.

**I: What are the policy factors that affect age at first marriage?**

**P:** No, rather there is policy that promotes age at first marriage should be greater than 18 years.

**I: What are any other opportunities to prevent early marriage and short birth spacing?**

**P:** There are boys and girls who want to marry girl/boy of rich man due to they are poor. These people come to consult us, then we will tell her not to marry him and we show a way that she can generate income. So, we have many women who reached good position/able to generate income. Political commitment, presence of other sectors working together, and presence of attorney are opportunities. Regarding short birth spacing, we have WDA that is our opportunity. And, they have high commitment. And, they have savings about 100-500 birr/week, like “equb”. Presence of awareness creation at schools which is done by staffs of women affairs that follow the students.

**Section 6: Multi-sectorial collaboration to improve maternal nutrition**

**I: Do you feel it is necessary to your institution to work with other sectors to address maternal nutrition?**

**P:** Yes it is important because every sector is working with us not only the health. Therefore, we are the backbones and it is important to work together.

**I: For multi-sectorial action that effectively works to improve maternal nutrition at all levels, what kind of change in terms of the way stakeholders work together is needed?**

**P:** We need to have commitment like health sector. Many times even daily, we meet with health sector through available means of communication. So, each sector should be committed well to improve maternal nutrition.

**I: Do your Zone have nutrition coordinating body?**

**P:** Yes, the health sector leads and we and women affairs, youth office and all other are the members.

**I: To what extent does your institution participate in the multi-sectorial nutrition coordinating body at the woreda level?**

**P:** We are participating in planned sessions and activities. We evaluate and monitor activities related with women and children and get justification on achievements. If not achieved we give feedback and put the way forward together. We have joint plan and minute. We regularly have meeting (monthly), every two weeks we visit the WDA and the WDA evaluate themselves weekly.

**I: How effective are the coordinating plating forms in enhancing multi-sectorial collaboration?**

**P:** We are effective, and we are happy to do and the community has a need for change. They are not using powders from supermarkets but blended flour prepared locally is used for feeding their children.

**I: What needs to be done to improve the capacity of these bodies for effective coordination?**

**P:** There should be adequate budget as if there is budget we can do what we want. But if you have doing with bare hand, it would be difficult to mobilise women.

**I: What lessons have you learnt regarding adolescent and maternal nutrition in this Zone?**

**P:** I have learnt from WDA, for example, I have learnt about how to prepare and use blended food. I have learnt many things such as the importance of blended foods to prevent the children from stunting, and to have healthy and mentally well children from WDA but not from education.

**I: What lessons have you learnt regarding multi-sectorial coordination of nutrition in this Zone?**

**P:** I have gotten many experiences from the team, supporting each other, and the importance of collaboration on women.

**I: What opportunities do exist to promote multi-sectorial collaboration of nutrition in this Zone?**

**P:** The presence of programs on public holidays like March 08 (the women day), commitment and there is government support.

**I: Do you have any other comments on what we have discussed?**

**P:** Nothing

**I: Thank you very much for your time!**

**Summary**

1. **Common maternal (Pregnant, lactating women and adolescent girls) nutrition problems in the community**

- The adolescents don’t have a group work like what is done by WDA. There are dik-daka (very short locally) women that is due to malnutrition.

1. **Nutrition priorities in the woreda**

- We (the women association) only work on formation of women groups (the WDA). Much of nutrition related works are mainly done in children and little has done on maternal nutrition so far.

1. **Nutrition interventions that improve adolescent and maternal health**

- There are works that have started recently, on creating household that can feed diversified foods though it is not that much expanded.

1. **Community level factors affecting access to maternal nutrition interventions**

- There are some parents who prevent their daughter from getting health service when she became pregnant because they ashamed of what happened on their daughter.

1. **Other interventions that influence adolescent and maternal nutrition and health**

- Early marriage can cause fistula.
- If a girl give birth yearly she will be harmed can become thin, she can be easily vulnerable to disease.

1. **Multi-sectorial collaboration to improve maternal nutrition**

- Multi-sectorial collaboration is important to us because every sector is working with us not only the health. Therefore, we are the backbones and it is important to work together.
