# Supplementary material for: Barriers to utilize nutrition interventions among lactating women in rural communities of Tigray, northern Ethiopia: An exploratory study
Source: PLoS One. 2021 Apr 30;16(4):e0250696. doi: 10.1371/journal.pone.0250696 (PMC8087028; doi:10.1371/journal.pone.0250696)
Supplement: S2 File — (ZIP) [file pone.0250696.s002.zip › S2_File.Doc/Woreda level and above key informants/029_IDI_woreda head of education office_Ofla Woreda.docx]

IDI with head of education office at Korem

SOUTHERS ZONE

Name of key informant: Haylay G/gergis

Age: 49 years

Gender : Male

Education: masters

Service year 8 years

Position: Head of district education office

Interviewer: Beyene Meressa

Date 27/02/2010 E.C

I: Interviewer

P: Participant

**Section one: Common maternal nutrition problems**

I: Ok, thank you for being voluntary to be interviewed. When i go to the first point of discussion. What women in Korem in general do to stay healthy? What activities they perform?

P: For a woman to be healthier, one they have to take balance nutrition must be in place. But, in practice, there is a problem in feeding of women in the community. One reason is economical problem. Due to economical problem and secondly due to lack of knowledge, there is feeding problem. Considering this if the women is not feed well, her baby will be harmed psychologically and physically. The government is working on feeding together with women affair and in collaboration with stakeholders. Even at Tabia level they are working focusing on nutrition. There were six Tabias where nutrition intervention were implemented.

M: Well, regarding the multi sectoral activities on nutrition we have discussion towards the end. Now, as an introduction you mentioned that feeding is one practice. Further, what women do to be healthy? Including the pregnant, lactating women and adolescents.

I: Are you asking me what themselves do or what the government did for them.

M: i mean by themselves

I: Oh if that is the case, there is an education from the government and those who accept they show change where as those who did not the result is seen in their children. more mothers are delivering at health facility, these feeding practice is also improving due to increase understanding in the community. In the past people use to eat one stew( food ) item but currently, they start to eat two to three types of food. They were not using oil. now they are using. They were not using sugar. now they are using. An increase in the economy will increase communities understanding. This is the existing condition regarding feeding.[phone call at 3:59]

M: It is good that you mention some the practices in the community. what are common health problems due to feeding problem in this area?

M: One is malnutrition. The community eat what they found. There is problem with eating balanced diet. You need to eat three time a day but i the rural they do not apply this due to the economic problem. Feeding depends on the resource that you have. Particularly in the areas with water shortage, this problem is common. In these areas children are not physically active and they become absent from school and if they come they sleep. Their mental is not active and their performance is not good particularly the far places. [the office was opened by the customer and a wait me message was given and discussion continued]

M: So those you mentioned some a problem due to feeding problem such poor school attendance, poor physical problems. these are usually long term effects. what other problem can happen to women and adolescents duet o malnutrition? especially immediately following poor feeding

P: You can get better information from health professionals. they can give better. i can only reflect general observation.

M: But, do you thing that it will cause a health problem?

M: Yes it will cause. it can cause abortion, the new born will have low birth weight.

M: For example problems such as anemia. goiter, night blindness. do you observe these problems in this area. we want to know your personal experience and reflections. There is no right and wrong answer here. This question will be asked to farmers as well.

P: In this area there goiter due to deficiency of iodine. there is problem of dwarfism or stunting .

I: How common are this kind of problems

P: Oh, i cannot tell this. it needs to be supported by study. if you want concrete information, you can get for health office. Do not take to more technical details of it. i can give you general ideas. There is study, but due to malnutrition there stunting can happen. they are short and they are poor in school. this can be seen in mothers and children.

M: what about wasting i mean imbalance between height and weight. stunting is imbalance between height and age.

P: I do not know about it.

M: Do you thing that malnutrition can cause non communicable disease?

I:Yes if you do not have resistance, you can easily get ill. It your food is not nutritious, you will be exposed to illness. we observe communicable disease in some of the kebeles. health professional go and address the problem. mainly due to shortage of water.

M: How do evaluate the food security of this area. i mean do they have sufficient food for weeks, months or years? it is of two type there are areas which are not secured for year and this will be more vulnerable at the time of drought. even now. when the crop production is good the emergency support will be interrupted. The safety net is program is operating in all area and at all time.

I: when did the drought occur. is it frequent?

P: Ya in normal condition the rain my stop. this season it is fine as a district level. The agriculture has confirmed this. The evaluation of this season showed that it is in good status.[customer opened the door and the interview was paused]

M: continue. we were talking about the frequency of the drought. is the drought happened frequently in this area. you please cloth the door. I told you, in 2008 there were a drought and there was support from the government. sometimes it(food insecurity) can happen without drought. hey do not allow people to the office. it can occur due to natural disaster. At times of good condition, it will be fine. when it is drought due to snow, flood , it will affect the product. there are places which are repeatedly affected by drought

I: Where are these places

P: For example. The lowlands are affected. Guara, Dinka, guara, gualmerkorous, Maymaedo places like this.

I: in your observation which ones are more affected by malnutrition. Food secure and insecure, in which area are the nutritional problems such as stunting, wasting happen

P: Oh i told you. That is the information that I have. I cannot give more than this now you leading towards health. it is better to ask for health. it you have questions targeted for education i can answer for you. I: Ok there is a question

P: I am giving you general information. You are going in to the details.

I: Do not worry what we need is only what you know. i you do not know about it. i can understand and pass the question. as head of the education office the ideas that we need form you is that, we believe nutrition is not the concern of one sector there will be questions that you can address well but now we are at the introductory. Well, from the women, lactating and adolescents which ones do you think are more exposed for malnutrition? from the those listed who are more affected for malnutrition?

P: Children, mothers and old age are affected

I: Can you explain more on that, why you said like that?

P: If the mothers are not supplied well with foods, it their husband cannot provide them, they can be affected. one there is pregnancy, delivery which are additional. mostly they need more food. there is tradition. they face problem of death and low birth weight.

I: Well, what kind of intervention for improving feeding of women and adolescents are in place here?

P: One there is a work owned by the health office. It is nutrition centered. They have their own work. There are health extension works. via these group there is work on nutrition. that formal government intervention. In areas that are not covered by the government, the nongovernmental organization support on nutrition. they did not cover all areas. That are focused on some projects particularly, in the area of nutrition. Like home gardening, educating women to eat egg, vegetable and fruits and use of porridge and the women affairs also did job on this area?

I: Now you are head of the education office, so do thing that involvement of your office in nutrition intervention is important and what a school can do in this regard? what the education sector can do in the mentioned targets for nutrition intervention? what kind of participation will you have?

P: This an institution where children get education. Many children stay in school. So, even though, school alone may not do nothing but together with the other sectors there are children who have feeding problem. Their parents do not have the capacity. they did not receive balanced food. here we work together to provide this children. With health and agriculture and others. if we want to keep their health there are works together with health, there is treatment program.

I: What is your specific role when you work with the stakeholder?

P: Our role will be identifying children who need support and give to them. we also provide them at school and if we are asked we will provide the information. for example students may dropout from school due to lack of food. the drop out is the result of lack of food. so we will identify where are these areas and provide them. Secondly we give pen and exercise book for those who cannot afford. all most all children born join school, so we provide support in school.

**Section two: nutrition priorities in the Woreda**

I: Now i will mention some examples of intervention in place to improve the nutrition of mothers and implemented by the concerned stakeholders for example antenatal care service, educating and providing the services , screening for malnutrition and providing necessary service, educating them to eat diversified food, personal hygiene, the use of bed net etc. We know these activities are being implemented by different concerned sectors. so, what is your experience about this or your observation? i know these done in other areas. are they present in this area?

P: In schools mainly the services are focused on menstruation, contraception, environmental hygiene. there are clubs and health professionals also teach and then if an illness happens, we inform to health professionals and manage together. we encounter pregnancy at schools in rural, they are taught to check. there are this kind of education in school. we have experience of doing together.

I: At school is there any service for detecting malnutrition in adolescents.

P: This service is not available at school. screening is made only during the event of communicable disease and related to HIV/AIDS. There is no other screening service in school.

I: How do you see the need of additional food for pregnant and lactating.

P: are you asking me the need to eat additional food?

I: Yes

P: yes it is needed, because there are areas with food shortage. A mother will not get what she is supposed to get. That need special support like what the government is doing. Though they are health they are being supported in areas of food shortage. They provide them by going to their Tabia

I: now, interventions for treatment for anemia, vitamin A have you seen this kind of interventions here

P: I do not know this

I: Ok, what kind of person is involved in safety net program? tell me in relation to pregnant and lactating women.

P: well safety net is for the poor, for the for in secured. they will be given a place and when they perform they will be supported and they will be transferred (considered as food secured). this for both male and female. It has its own criteria. They will be selected from Tabia. when they improve, they will be transferred. there poor females who need support will be given priority. those who do not have and who can work are given the chance. older and others even the community also provides support.

I: What about for pregnant and lactating women

P: They will not work but hey will get the support similar to others who involved.

I: What about school feeding: who is school feeding . what kind of schools are eligible for feeding. I think you have some information about his. i want you to share me?

P: This year there is no school feeding. in 2008 there were some schools because there was a drought that time. children were likely to dropout from school as their parents cannot afford feeding. Then the government identifies 31 schools under feeding support "Migibina". Two types of food was given to them.

I: What are the two types of food.

P: The first one porridge and the second is "Kinche". This were the foods. They is made from wheat, corn which supplied from cooperative unions. The community will support water and wood and they also serve the students where as the government will facilitate transportation of materials. NGOs like action aid and save the children were involved. it was like this.[the door was opened].

P: Hey Assefa please cloth the door

I: you were tell me about school feeding

I: You told me that the feeding program is not there. Why is that?

P: There is no drought this year. but he feeding is very important. they are asking why it is interrupted and many students become absent from school related to feeding. It there is feedig they will come for the sake of the breakfast. they did not get that at home

I: do you mean that there is an assumption that at a time of good harvest food security is granted

P: Now there is no supporter for this. The government brings it as a project every year. only at the time of drought, they discuss about it. there is no formal strategy to support schools with feeding.

I: It is not via the government's strategy.

P: It is not a planned program

I: How can out of school children be reached by nutritional interventions

P: There is an association called "Timiret Kinkin". They collect crop from the community and when the mothers give birth at health center, they get feeding there. secondly any poor will also will be supported. it will be collected form the community. they will provide exercise boor, cloth etc. if it is beyond the scope the community, the Woreda will support.

I: is there any education about feeding at school

P: Yes it s included in science curriculum. additionally there a topic about feeding in science. the health professionals also provide health education.

I: Does health extension worker provide health education at school

P: yes they meet with school directors and they inspect. They also provide separate education for female. They observe sanitation, any communicable diseases and provide support.

I: The interventions like we were mentioning before such as school feeding, education and sanitation which are meant for improving nutrition. Are they effective in addressing malnutrition?

P: Yes there is change. When they get balanced food there will not be school absent and the will attend the school properly. The result will be better. If school feeding is well introduced at school children. it will contribute for good physical growth of the child. it will increase their mental capacity and increase their understanding. If the resource allows and feeding program is implemented based on study, it is highly important. we have seen the change in 2008. they will be absent from school. We set a target of less than 1% school dropout and this was achieved by all schools. and they will be clever ad competent. this year the program is not in schools and we have observed some absent in schools. we said to school directors that there is no drought this year. Particularly, around Guara, Dinka, Maymaedo which are highly affected. The children form this areas are short. it is very important to have school feeding according to our evaluation.

I: You mentioned that school feeding is important. what are the challenges in the implementation of these services aimed to improve maternal nutrition

P: Those who are feeding the children are volunteers, they are selected by the community. They do not have salary. they need have salary. They start working and left soon. There is problem in budgeting, as long as they do not have salary the quality will be compromised. there is gap in that regard. The school can arrange wood and place for cooking. the problem is there shortage of water. It must be supplied by reservoir(Roto) in the school. transportation is another challenge. this is given as an extra job. the responsibility is given to the Office only. there is assigned person to follow this. There is logistic problem. we give attention to prevent any problem related to feeding. the Woreda has taken the commitment and make suffering considering the benefit for the children.

I: of course You have tried to mention it before. There is service related to menstruation. is there any service where school girls are targeted particularly on nutrition. Are there any club in the school

P: Yes there club in all schools

I: What is the name of the club?

P: It is called sanitation and health club. within it contains several activities for example: for female there is a room where they can get rest during menstruation. And they provide modes. family planning, water sanitation activities are activities done by the club. There are model schools. they work about HIV, even there are school who produce vegetables such as carrot, cabbage etc. for feeding. They produce and send to the local community.

**section three: Community factors affecting access to maternal nutrition interventions**

I: What are the main challenges in the implementation of nutrition intervention. you have mentioned some for the challenges one the absence of adequate resource. For example, how educational status of the women, service quality can influence utilization of services?

P: Most of he women are out of school. There is no child bearing women in the school. the case can happen in secondary school. They get pregnancy at 9th 10 and 11th grades.

I: What i mean how the education status of the women and service quality can affect their own feeding?

P: Ya , I have told you before. it is repetition. it the person is not educated there will be problem with feeding one due to lack of knowledge. and the second is due to problem in thing The educated once are improving their feeding. There change from time to time. There change in feeding in clothing. this will affect the students. the uneducated ones are uneducated.

I: In your opinion what shall be done to solve problems in nutrition?

P: Females should be educated. the problem is related to knowledge. Considering he resource they have they need if women eat balance diet, they will not get illness and their children will not face problem. To avoid the problems in nutrition, firstly the community need to learn. is so the health professionals as well should involve in teaching. And it should be delivered seriously in school curriculum. Secondly the family should be supportive. even those who have or the rich do have problem in feeding. despite they have eggs, they won't feed their children. They took to market instead of eating. Now there are efforts from women affair, education, health. Because of this the maternal mortality, child mortality and communicable disease are decreasing. so education and awareness creation activities should continue. beyond awareness creation it is important to rake part and to provide support. it food supply is found necessary it should be done based on study. in the selected areas.

I: For pregnant and lactating?

P: Yes, based on study if the mothers, lactating and children. This support should continue until it issue come in to the right track. education and feeding support are essential. "Seeing is believing". they have to see in action. There are good things in practice and it is possible to change. the women are doing it.

**Section Five: other interventions that influence adolescent and maternal nutrition and health outcomes**

I: School adolescents were not targets for nutritional intervention and emphasis was not given so far. so, what can we do to address their problem? like the HIV education, menstruation, family planning services what can be done in nutrition? why i am saying like this is that she is the mother of tomorrow.

P: In school they need to learn. there is sexual practice in schools. Educating the adolescent female that this is not correct.

I: Is there any education given to females. it can be about marriage under 18

P: Yes, there is but it is not specifically for females. they have their own club; girls club. they are told not to get pregnant, to use condom. Condom is available at secondary schools. this kind of education is given particular to female. But there is diffidence in that regard. For example, there shortage of modes, no sufficient room, sanitation materials. It is symbolic. Even the health professionals come rarely. no one comes with commitment. it is not planned. for example, take first aid it is important and functional in the past. Nor there is no first aid. sanitation materials as well are not available at school. women are supposed to be supported but ther is no such support.

I: Who is responsible for that?

P: It is the schools responsibility.

I: The school or the health office

P: The schools. if there are donors, they will support some times. otherwise it is the schools responsibility to fulfill form its budget.

I: Even for fist aid materials

P: Yes we request for health office.

I: Who provide education school?

P: The health workers because it is more technical. they have contact teachers who lead a with club.

I: It is known that early marriage has negative effect on the health of the women. Is there education about it?

P: Yes

I: Who provide this

P: mainly the women affair and then there is screening committee.

I: Who are the members of the steering committee.

P: Oh i told. the women affair, water, health, agriculture and school. These are the members.

I: Is the head or other person become the member of the steering committee

P: The heads of each office are the steering committee and the same is true at Kebele/Tabia level. Early marriage is taken seriously. It there are girls who are to marry due to parts influence. it will be reported to women affair. they will deal with attorney and prevent the marriage.

**Section six: multi sectoral collaboration**

I: Ok i will present you the last question. It is about multi sectoral collaboration, the problem of malnutrition cannot be addressed by education alone, by health alone or agriculture alone. so multisectoral approach is required. Therefore, what can be the role for the school in the collaboration. what looks your level of participation and discussion at the steering committee. The steering committee will have agenda, so can be done in adolescent at multi sectoral level.

P: about the other you will get form others but as a school one increasing school participation of female. Their participation should be 1 to 1 ratio at registration. All age eligible girls should come to school. To avoid attrition they need special support. we provide support in English, moths and natural science based on the voluntariness of school teachers. secondly when they are in school to bring them to leadership assigning them as a monitor; one male and one female monitor in a class. it can also be unit leader, class room teacher etc. secondly, to protect them from abuse, we hear their complaint. it they tried out side of the school. For menstruation problem they have room.

I: What about in nutrition. how do you see the activities in nutrition. are performing well in that?

P: Our role as school in the steering committee is to teach. the school will make ready and the health professionals will provide the education. This is the collaboration. we cannot say the collaboration regarding nutrition is strong. It is theoretical.

I: So, who can we make the collaboration strong?

P: The main issues is Budget. budget shortage should be solved. If you want to work at school about nutrition, the necessary things should be supplied. In the area of Health, the same is true if we want to support them. they did not get nothing outside their home but education is given more or less and they learn in their school. We teach vegetable, fruit, protein and fat. did they get at home. No, so at least they have to get at school. But this is absent which is the minimum

I: Who the chair of the screening committee

P: The women affairs. of course the administrator is the chair but the women affair is leading

I: Do you raise any agenda about nutrition during the meeting of the steering committee. for example you raise issue related school problems.

P: Yes we raise . we discuss about a student who become absent and we ask why? they become absent due to food, due to early marriage, etc will be evaluated. if there is early marriage, the school, will report and we will discuss with the family and bring her back to school.

I: Is there follow up on early married.

P: Yes

I: Who follows?

P: The women affair/the committee

I: Ya they usually engaged to early marriage from school

P: The school will report their absence, then her age will be checked and the girl herself will say i am forced. it is not my interest. the attorney will also look at the issue and if they are under 18, they will not be allowed to marry.

I: Ok. As a conclusion to improve the nutrition of women, pregnant and adolescent what should be done?

P: That is what i told you before. there is thinking problem. There must be a support. the committee need be strengthen, budget should be available, and female need to be supported. but the main problem is with thinking. if you work on that both rich and poor can improve with in it capacity. basically, there are areas which are food in secured naturally which is exceptional. we have areas with repeated food insecurity in our area and they get support. in this regard support s need until it comes to the right track. With increasing awareness it will improve. But, now children when there is feeding and when there is no feeding, there is difference. even though they at their home they migrate with their children. child spacing is important, there women who give birth without limit but they are poor. the main issue is that if you teach and if you can guide the majority of the community can be changed. Then for those who need economic support, support is necessary. Thank you i have finished.

**Summary**

- There change in feeding habit. the community are currently feeding two to three types of food. oils, sugars are added to their food but this is not true in rural. This was relate to increased economy the community.
- the consequences of poor feeding include, not physically active and they become absent from school , sleep inside the class and poor school performances. These are visible in far places of the district.
- Two type there are areas in the district; those not secured for year and more vulnerable to drought and food secured. the lowlands are more affect area example: Guara, Dinka, guara, gualmerkorous, Maymaedo
- The role of the school is identifying children who need support
- School feeding contribute for better nutrition and school attendance. But it is an occasional intervention supported by NGOs. Porridge and Kinche are the type of feed
- There is steering committee form district to kebele level but it is not properly addressing nutrition intervention and it is not well coordinated.
- There is no nutrition education at school but there is health and sanitation club which can do this task if supported by the stakeholders
- Poor females who need support will be given priority. Those who do not have and who can work are given the chance to work in safety net.
