# Supplementary material for: Barriers to utilize nutrition interventions among lactating women in rural communities of Tigray, northern Ethiopia: An exploratory study
Source: PLoS One. 2021 Apr 30;16(4):e0250696. doi: 10.1371/journal.pone.0250696 (PMC8087028; doi:10.1371/journal.pone.0250696)
Supplement: S2 File — (ZIP) [file pone.0250696.s002.zip › S2_File.Doc/Woreda level and above key informants/030_IDI_head for Woreda Health Office_Ofla Woreda.docx]

| **Introduction:**  Hello, my name is Dejen Yemane. I am from Mekelle University. Thank you for taking the time to speak with me today. We are doing research on the factors that influence the nutrition of mothers and adolescents in collaboration with the Regional Health Bureau and UNICEF. Your participation is very valuable. The things that you tell us will be used to improve nutrition programs and services for women in the region and the country. We will not share your names when we report our results.  However, I will record the discussion so that I can capture all the ideas that are shared. I have several questions to ask you that I have prepared in advance, and I will ask you to say what you think about each question. The interview will last for 1:30 -2:00 hours. Do you have any questions before we begin? If you think of any questions as we proceed, please feel free to let me know. If it is all right with you, I will turn on the tape recorder now.  **Section A: Interview details**   1. Zone: **Southern** 2. Woreda: **Woreda** 3. Kebele: ------------------------------------------ 4. Name of key informant: **Weldegergis Hailu** 5. Institution of key informant: **Ofla Woreda Health Office** 6. Interviewer name: **Dejen Yemane** 7. Date of interview: **November 6, 2017** 8. Interview start time: _________________________ 9. Interview end time: __________________________ |
| --- |
| **Section B: Interviewee professional information**   1. Gender    1. **Female**    2. Male 2. Age: **35 years** 3. Highest level of completed education.    1. No formal education    2. Primary education    3. High school    4. College education    5. **Bachelor degree**    6. Master’s degree 4. Current job/position: Ofla Woreda Health Office head 5. How long have you been in the current job/position?    1. ______ Months    2. **3 Years** |

| **Section1** | **Common maternal (pregnant women, lactating women and adolescent girls) nutrition problems in the community.** |
| --- | --- |
| 1.1 | What do women do to stay healthy in this community/woreda?  **Participant:** it is good, for your information this year we have targeted 4776 pregnant women and as a woreda we have 990 women development army (WDA) and we have 29158 members. To maintain the health of women, before 5 years we have launched WDA as a woreda and kebele. And this network is functioning well by taking women into consideration on vaccination, delivery and especially starting from 2008 E.C we are centrally working on nutrition to improve their feeding behavior. Through the 74 HEWs of our woreda in coordination with the WDAs there are health education sessions for the identified pregnant, lactating and adolescents. |
| 1.2 | In your opinion, what are the common nutrition problems in the community for women? What about for adolescent girls?  **Participant:** the common problem in our community is that they produce for sale than for consumption. Most of the time they produce market centered crops the profit in mind. There is a big gap in utilizing the animal and animal source foods and cereals in our mothers and in this regard, we took a blame that this could be due to the limited health education and monitoring activities.  **Interviewer:** As you have mentioned there is a tendency of market centered production in the community and if that is, so the pregnant women, lactating women and adolescent girls will not get the required nutrients. So, what are the common nutrition problems in in your woreda especially in pregnant women, lactating women and adolescent girls?  **Participant:** due to lack of proper utilization, there is anemia in our mothers. Even though iron supplementation is administered by our HEWs, at least there are mothers who don’t utilize it. Secondly, when we screen them around 33% of mothers have less than 23cm Mid Upper Arm Circumference (MUAC). And this increases the susceptibility of mothers to different diseases. In addition to the improper utilization of plant and animal source foods they don’t use micronutrients given by health professionals appropriately. Thus, this exposes them for different diseases.  **Interviewer:** what are these diseases? Can you tell me please?  **Participant:** for example, anemia and physical weakness  **Interviewer:** What about others?  **Participant:** there are reports from hospitals that indicate children are born with birth defects especially due to improper utilization of iron supplements. Unlike the successful achievements in institutional delivery, we have evaluated ourselves that we have limitations in nutrition.  **Interviewer:** you have said that there are micronutrient deficiencies. What could be the possible causes of those micronutrient deficiencies?  **Participant:** as we have said, one iodine utilization. Especially these times the utilization of iodized salt is almost 100%. Secondly, with iron supplementation there are limitations. For example, pregnant women take iron folate tablet for 3 months and the issue of putting the tablets in a comfortable place and taking them appropriately is the questionable. When you take children, even though we administer Vitamin-A they might vomit it. So, the issue of proper administration of the nutritional supplementations is very important.    **Interviewer:** you told me about pregnant and lactating women, what about in adolescents? What are the commonest nutritional problems?  **Participant:** most of the time adolescents are not target groups for the nutrition screening. Because, now a day we don’t have underage marriage so minimum if the adolescent girl did not come as pregnant she is not target for our HEWs. May be those 19 years old adolescents who are married. Unless otherwise, the area of adolescent nutrition is not well studied. Since the eligibility criteria for WDA networking is >18 years old, they are not targets. Besides, this time it is very rare to get pregnant at this age. But during home-to-home visit in educated family you might get home gardening. Unless otherwise, when you see the utilization of nutritional interventions in this age group, they are not target groups like the pregnant and lactating women.  **Interviewer:** Do you think there could be any association between nutrition and occurrence of non-communicable diseases among the women/girls? Why? Are there such diseases in this community?  **Participant:** when you say does malnutrition our community’s problem, you cannot say it is 100% it is our problem. At least the main target should be the health professional. Be it communicable or noncommunicable nutrition related disease are almost neglected in our country due to lack of due attention. Due to lack of attention we did not teach our wider community. And this can be taken as the limitations of the health professional and the administrative bodies. Our main limitation was failure to creating platforms to make nutrition as a multi-sectors agenda. Even in the communities where our HEWs are involved, the community didn’t give due attention.  **Interviewer:** do you think non-communicable diseases is problem of our community? For example, like cancer, obesity and cardiovascular diseases  **Participant:** yes, they are our problems.  **Interviewer:** is that common?  **Participant:** generally speaking if you see their prevalence non-communicable diseases are not our problem. As a region wasting is common. But obesity and overweight are not common. But in general, the failure is shared between the health professional, administration and the community because in the community there is a problem of applying what they have been thought by the HEWs.  **Interviewer:** which segment of the population is most affected by non-communicable diseases?  **Participant:** non-communicable diseases common**ly affects** children, in pregnant and lactating women. In the national nutrition program failure to invest in the first 1000days, conception to the 2^nd^ birth date can lead to non-communicable diseases. Pregnant are not taking extra meals and rest, they are not properly caring to their children. In our screening programs we are looking mothers with non-communicable diseases and this is an indication that they are properly feeding.  **Interviewer:** Do women/girls in this community increase their weight/height proportional to their age? Could it have relationship with their nutrition?  **Participant:** pregnant and lactating women are not increasing their weight/height proportional to their age. Before two years we were enrolling mothers with <21cm MUAC to TFP and now we are using MUAC of <23 cm as an eligibility criterion for TFP and we have many mothers with MUAC of <23 cm indicating that they are not adequately feeding. When we look the percentage of wasting for example it outweighs on mothers compared to children. The bottom line is inadequate feeding due to work overload, not taking extra meals and nutrition insecurity. But overweight is not the problem of our community.  **Interviewer:** Is there a situation where the community suffers from food insecurity? In what situation do you think this happened? How frequent does it happen? Why do you think that it is frequently happening? How the women/girls do suffer from it, in particular?  **Participant:** yes, there is food insecurity problem, especially in the year 2008 E.C and it is common in the lowlands kebeles (Guara, Dinka, Dara and Gual-menkorios) around Tsilare basin. Especially these four kebeles are food insecure and most affected by drought. Recognizing this, the government has provided support for both the community and their animals. In addition, we installed water thankers and trucks were distributing water for two months. During these times the most affected were children and women and there was nutrition emergency through the health program by Save the Children. Currently, though it is not equivalent to the central Ofla kebeles these kebeles are food secured though we have 47,000 Safety Net Program supported population and this includes the elders, disables and pregnant and lactating women.  The lowlands kebeles (Guara, Dinka, Dara and Gual-menkorios) around Tsilare basin have also critical shortage of water both for drinking and for agricultural purposes. The good thing is, these kebeles have good animal resources.  **Interviewer:** from the nutritional problems that you have mentioned like anemia, goiter, stunting, wasting to which one do you think are mothers most exposed?  **Participant:** pregnant and lactating women are most affected by wasting and anemia. Especially we don’t know is that due to supply, access or utilization of iron supplementation anemia is common in our mothers. |
| **Section2** | **Nutrition priorities in the woreda** |
| 2.2 | In your opinion, what interventions do you think are the priorities of your institution to improve nutrition for pregnant women? Why? What your institution is doing currently related to the priority activities for pregnant women?  **Participant:**  our priority intervention is nutritional screening. HEWs in coordination with the 990 WDAs do nutritional screening (MUAC and oedema) every month for Children from 6 to 59 months and pregnant and lactating women and our target is not to measure their nutritional status rather it is about are we treating SAM cases as OTP or refer them to TFP.  **Interview:** What about for the micronutrient deficiencies?  **Participant:** it is related with our screening activities. For example, if she came for screening we did not only take anthropometric measurements, the HEW looks for all the components including the counseling services.  **Interviewer:** what about the lactating women?  **Participant:** it is similar, because they are our targets. For example, the HEW will check monthly how much iron does a woman has, how much is left, and did she utilize properly when she come for screening.  **Interviewer:** what about the adolescents?  **Participant:** regarding adolescents as I have told you before they are not target groups for nutritional interventions. But you can address them in their WDA network if they are 18 years and above or while HEWs give health education in schools because HEWs has a schedule to teach students on HIV, nutrition, WASH monthly.  **Interviewer:** What nutrition interventions have the most resources allocated to them? Could you tell me in detail with examples?  **Participant:** this are not budget allocated services. For example, iron supplementation, deworming and vitamin A supplementation are routine services. We are part of the SURE program (sustainable undernutrition reduction in Ethiopia). There was a forum before 2 weeks and in this forum, we have identified job description of Education office, Agriculture office, Women affairs, health office etc. in nutrition and we have evaluated as a woreda the imbalance between our productivity and the perception of our community towards adequate nutrition. Then we took an assignment kebele leaders to mobilize the community, HEW to monitor the health issues and Agricultural extension worker to improve productivity starting from home gardening. So, we reached in to consensus that nutrition is a multi-sectoral issue. |
| 2.3 | Can you tell me some of the successful nutrition interventions for pregnant women that you have implemented in this woreda? Why do you think they are successful?  **Participant:** our screening activities are successful. In our woreda, apart from women we have food demonstrations for men in health facilities, where model men show food demonstration. In addition, we celebrate the 6^th^ month of a child to congratulate the child for starting complementary foods. But we need to conduct studies to see the impact of the interventions.  **Summary**   - Wasting, and anaemia are the most common nutritional problems in Ofla Woreda. - Adolescent girls are not target groups like pregnant and lactating women for nutritional interventions - The main limitation of the Woreda is failure to creating platforms to make nutrition as a multi-sectors agenda. - Nutrition related non-communicable diseases are not major problems of the Woreda - Pregnant and lactating women are not increasing their weight/height proportional to their age - There is food insecurity problem, in the lowlands kebeles (Guara, Dinka, Dara and Gual-menkorios) around Tsilare basin. - Nutritional screening is priority nutrition intervention and it is successful. - Nutritional interventions are not budget allocated services, they are routine services. - In Ofla Woreda there is common consensus that nutrition is a multi-sectoral issue. - Apart from women there is food demonstrations for men in health facilities |
| **Section3** | **Nutrition interventions that improve adolescent and maternal health** |
| 3.1 | What kinds of nutrition interventions are in place to improve health of the pregnant in this woreda? Where do they get it? Who provide it?  **Participant:** There are many types of nutrition interventions to improve health of pregnant, lactating and adolescent girls like nutritional screening, Vitamin A and iron folate supplementation and deworming. These are given in all the 31 health facilities in our 21 kebeles. These nutritional services are routine service given at the 31 health facilities and sites. Vitamin A supplementation is given for lactating whereas the rest of the interventions are given for all.  **Interviewer:** do we have pregnant, lactating or adolescent girls targeted nutritional services?  **Participant:** the service starts from ANC and during ANC follow up HEWs give nutritional screening and counselling. The main thing is giving advice on extra meal, reducing work load to the extent they right letter to agricultural agents to be exempted from water and soil conservation activities and to be included in the Safety Net Programs.  **Interviewer:** what about in adolescent girls? Where does adolescent girls get nutritional services?  **Participant:** they will get at their site. We will not discriminate rather we serve when they come to us. Monthly we have screening and WDA mobilize eligible mothers, so f they come to the site they will be served. But our center for adolescents is schools. Last year we had organized a forum for school directors to discuss on nutrition agenda only and we reached in consensus to make nutrition as their agenda because if they get nutritional education at school they can easily take home.  **Interviewer:** Are pregnant women getting counseling for food diversification, iodized salt during pregnancy? Could you tell me about it with examples? Why do you think it is necessary?  **Participant:** by the way the WDA leaders has monthly meeting and they also gather their respective members every two weeks. Every WDA has manual on nutrition and other interventions. So, based on their manual they give health education on how to prepare enriched food, importance of taking extra meal, food diversification. We provide additional food (6Kg) for mothers with <23cm MUAC and we inform them that this is not quota, so she must not share this with her family because it is given for her like a medicine. Sharing medicine prescribed for someone is prohibited, like that additional food given for malnourished mothers or children must not be shared among the family because it is medicine. But we cannot say that all mothers are appropriately utilizing it. This can be attributable to the communication and convincing skills of the WDAs and HEWs. So, we need to equip the 74 HEWs with the knowledge, attitude and communication skills required to train and support WDAs in order to improve the health of families and the community, with emphasis on pregnant, lactating and adolescent girls’ nutrition.  **Interviewer:** Are pregnant women getting advice on nutrition sensitive agriculture such as home gardening? What about on the need to be involved in safety net programs? Who provide them? What else interventions related to this?  **Participant:** agricultural agent is responsible to identify eligible individuals for safety net programs. Regarding home gardening, after identifying 1200 poor pregnant and lactating women having less than 2 children, SURE program has distributed apple, mango, carrot, cabbage, and potato seeds to insure food security and food adequacy. Before 2 weeks we were demonstrating with the help of audio visual materials. There were mothers who were complaining for not getting FAFA after screening. Then after several discussions with the community FAFA is not a support rather it is given for people having nutritional problem and now the community understands that who is eligible for such supports.  **Interviewer:** Are pregnant women getting advice on water, sanitation and hygiene services?  **Participants:** if you take teachers, they are subject specific (mathematics, English, chemistry and the like) but HEW is all rounded and the center of the health extension package is WASH and communicable diseases. This year WASH was the major agenda because there was acute watery diarrhea (AWD). So, promoting sanitation, hygiene and safe water handling is routine activity of our HEWs. There is a consensus that health knowledge of mothers is better than men because of the works of our HEWs and WDAs.  **Interview:** Are pregnant women getting advice on the need to use Insecticide treated bed nets (ITN)? Why? Who advise them?  **Participant:** malaria is not our problem because our woreda is highland except few kebeles. So, instead of distributing insecticide treated bed nets we give general health education on how to prevent and control malaria by draining stagnant water and clearing bushy areas. Because the resource is allocated to woredas with high burden of malaria. But before two years TRHB has sent ITNs by mistake and we distributed them to the lowland kebeles around Tsilare basin namely Adishumbereket, Sesela, My-maedo and Zilat cluster for around 13,000 households. Side by side with the distribution we were educating the community to give priority to pregnant and children. But, since malaria is not a problem the was utilization problems like using ITN to cover grasses.  **Interviewer:** Is there a situation, which pregnant women need to be eligible for Targeted supplementary feeding (TSF)? Why? Could you tell me specific examples?  **Participant:** yes, they are eligible for Targeted supplementary feeding (TSF). For eligibility to TSF, we use 3.4 and 3.1 conversion factor for pregnancy and delivery. So, based on this Targeted supplementary feeding.  **Interviewer:** In your opinion, are adolescent girls provided school feeding? Why is it necessary?  **Participant:** yes, it is school feeding is very important especially for school attendance and performance. In relation to the 2008 E.C drought there was school feeding in 36 schools by the help of Action Aid and Orthodox Tewahdo Church but now we don’t have school feeding at all. Regarding feeding interventions targeted to out-school adolescents, since the school feeding program is in schools it doesn’t target out-school adolescents.  **Interviewer:**  In your opinion, are adolescent girls linked to youth friendly services at health facilities? Why is it necessary? What about out-school adolescents.  **Participant:**  we have low performance in linking both in-school and out-school adolescent girls to youth friendly services. In this regard Hashenge cluster is the best performer. Transforming Primary Health Service (TPHS) program previously called FHP is working on this area. We have youth clubs. but it is difficult to say all adolescents are addressed.  **Interviewer:**  Which of the interventions listed above do you think is most important for pregnant women?  **Participant:**  all are complementary to each other. For example, iron supplementation is equally important with WASH. If she doesn’t have sanitation facility she will be exposed to different communicable diseases. For example, if she fails to wash her hand in the critical times she will get diseased and these communicable diseases affect most mothers and their children. But if you create awareness nutritional interventions are very easy to implement. Because if you take WASH, the community associates with cost. |
| 3.2 | In your opinion, which of the above interventions for the pregnant women are being implemented in an effective way? Why do you think that it is effective? In what way was it implemented?  **Intervention:**  Screening, deworming and vitamin A supplementation are most effectively implemented interventions. But from the client side implementing advises given by HEWs is not satisfactory. |
| 3.3 | In your opinion, which of the above interventions mentioned are less effective to the pregnant women? Why do you think that it was less effective?  **Participant:** Hygiene and sanitation are less effectively implemented interventions because the community associates it with cost for slabs and superstructures. In 2006/7 E.C almost 20 of the 21 kebeles were certified as open defecation free kebeles, but due to lack of monitoring and evaluation it has failed. Because it is not a onetime activity done by campaigns. The other in effective intervention is home gardening due to lack of water. Similarly, linking adolescents to youth friendly services is also very poor and this can be lack of attention from woreda. |
| 3.4 | What are the challenges to implement delivering the nutrition interventions that we have been discussing for the pregnant women?  **Participant:** ineffectiveness of our health education delivery methods.  **Interviewer:** what do you mean by ineffectiveness of our health education delivery methods? Could you elaborate it?  **Participant:** okay, when I say ineffectiveness of our health education delivery methods, we don’t know how much time is needed to transmit a certain health message. The other is lack of continuous monitoring and evaluation activities. In addition, in a kebele we have only two HEWs and these HEWs are responsible for 10,000 population and as result there is accessibility issue. Road access, distance of health facilities, geographical conditions and lack of awareness can be also mentioned as implementation challenges. Acute watery diarrhea is best indicator that the community is not keeping its personal and environmental hygiene and poorly handling its water.  **Interviewer:** How do you evaluate the resources available to the interventions? How do you explain the awareness of the pregnant women on nutrition related problems? What about the lactating women? The adolescent girls? The care providers?  **Participant:** we don’t have a problem on supplies except distance of health facility, topography and HEW to population ratio.  **Participant:**    Probe for pregnant, lactating and adolescent girls:  **Interviewer:** How do you evaluate the priority given for the interventions for the women?  **Participant:** they are our priorities it is governments direction.  **Interviewer:** Are there any factors related to skilled care provider that affects implementation of the interventions? Tell me more about it?  **Participant:** though we did not assess it is good to sensitize WDAs and HEWs because they are the one who meet with community and are the one supposed to bring behavioral changes.  **Interviewer:** How do you evaluate the coordination and collaboration between the nutrition sensitive and specific sectors?  **Participant:** there are good starts for example in the SURE program, but it is not satisfactory. Though there is a consensus that nutrition is not one sector issue we cannot confidently say there is coordination and collaboration between the nutrition sensitive and specific sectors. |
| 3.6 | For these challenges that you mentioned, can you tell me of any successes or innovations that your institution have used to improve delivery of nutrition services for pregnant women?  **Participant:** to be focused in nutrition, we discuss in different forums and reached in to consensus that nutrition is not only the responsibility of mothers but also the responsibility of fathers. That is why in our woreda, apart from women we have food demonstrations for men in health facilities, where model men show food demonstration. Because we believe that without involvement of men it is difficult to overcome the nutritional problems. The other is the community is considering vaccination and institutional delivery as their norms.  **Summary**   - Nutritional interventions like nutritional screening, counseling, Vitamin A and iron folate supplementation and deworming are in place to improve health of pregnant, lactating and adolescent girls - Equipping HEWs with the knowledge, attitude and communication skills required to train and support WDAs in order to improve the health of families and the community, with emphasis on pregnant, lactating and adolescent girls’ nutrition. - There is SURE program, a multi sectoral program for improving nutrition - In the year 2008 E.C there was school feeding in the 36 schools by the help of Action Aid and Orthodox Tewahdo Church - Screening, deworming and vitamin A supplementation are most effectively implemented interventions - Hygiene and sanitation, home gardening, linking adolescents to youth friendly services are less effectively implemented interventions. - Ineffectiveness of health education delivery methods is also observed due to lack of continuous monitoring and evaluation activities and unavailability of standardized time to transmit a certain health message - Road access, distance of health facilities, geographical conditions and lack of awareness were mentioned as implementation challenges - To improve delivery of nutrition services, there is involvement of men in food demonstrations. |
| **Section4** | **Community factors affecting access to maternal nutrition interventions** |
| 4.1 | In your opinion, what barriers do you think, are preventing pregnant women from using the interventions that we have discussed? (**remind them**) What about the barriers for lactation women? What about for adolescent girls?  **Participant:** lack of awareness attributable to educational status of the women and the girls in both ANC and PNC services and distance of health facility, lack of ambulance, connection between accesses of transportation in PNC can be mentioned as barriers for utilization of nutrition interventions. For example, during the summer season there were home deliveries because there was heavy rain and the road was closed in four kebeles. There are also community beliefs that affect utilization of PNC, like a mother should not cross rivers before 45 days, though they are addressed now a day. Besides, absence of health problems during previous pregnancy, uneventful previous pregnancy experience can also prevent pregnant women from using the interventions.  **Interviewer:** what about quality of care?  **Participant:** yes, there are problems in quality of care especially in delivery but this cannot be considered as barrier because they know the importance but they raise it in meetings. |
| 4.2 | How can these barriers be addressed to improve nutrition among pregnant women in this community?  **Participant:** by creating awareness through community conversations can improve nutrition among pregnant, lactating and adolescent girls. On July 2017 we have had a community conversation with 4538 women by HEWs, Health Center and Woreda health office staffs on ANC, PNC, Nutrition, WASH and communicable diseases.  **Summary**   - lack of awareness attributable to educational status of the women and the girls in both ANC and PNC services, distance of health facility, lack of ambulance, connection between accesses of transportation, absence of health problems during previous pregnancy, uneventful previous pregnancy experience are barriers that prevent pregnant women from using the interventions. - Community beliefs like a mother should not cross rivers before 45 days also affects utilization of PNC. - barriers that prevent pregnant women from using the interventions can be addressed by creating awareness through community conversations |
| **Section5** | **Other interventions that influence adolescent and maternal nutrition and health outcomes** |
| 5.1 | **Interviewer:** In your opinion, why would delayed marriage (after 18 years) improve maternal nutrition?  **Participant:** yes, delayed marriage improves maternal nutrition. Scientifically under 18 years old adolescent is not physiologically matured. So, she must wait until maturity and to understand how to care child. Adolescent pregnancy is also associated with low birth weight.  **Interviewer:** In your opinion, why would increase the space between each birth improve maternal nutrition?  **Participant:** if there is no birth spacing it will lead to adverse nutrition outcomes for the mother and child. For example, the mother might face anemia, underweight and birth complications and she will give birth to low birth weight child. If there is low birth weight it will increase maternal mortality.  **Interviewer:** What programs or activities promote increasing birth intervals in this level? **Level indicates kebele/woreda/Zone/Region**  **Participant:** we promote family planning while mothers come to health facilities for services and in home to home basis through HEWs. As a woreda we have good performance in family planning. Most of the time mothers use Depo-Provera but we are convincing mothers to use long acting contraceptives like IUCD. In utilizing long acting contraceptives, Lat Health Center is a model institution and that is the work of the Midwife working there.  **Interviewer:** Can you tell me about any programs or policies in place in this woreda to prevent early marriage?  **Participant:** at woreda level early marriage is legislatively prohibited.  **Interviewer:** Can you think of any more programs or policies? Think about political, religious and other influences.  **Participant:** It is politically and religiously supported. |
| 5.2 | In your opinion, are these programs or policies effective? Why or why not?  **Participant:** yes, they are effective, and this is included in the 2^nd^ GTP. In, addition it is a multi-sectoral agenda. |
| 5.3 | Can you think of any other opportunities to prevent early marriage and increasing birth spacing?  **Participant:** creating awareness at schools and religious institutions. In addition, presence of WDA networks at each village, women associations and leagues. In each Kebele there are 21 steering committee focusing on mothers and children.  **Summary**   - Adolescent pregnancy is associated with low birth weight. - Short birth spacing lead to adverse nutrition outcomes (anemia, underweight, maternal mortality and low birth weight) for the mother and child. - Promoting family planning increases birth intervals - At woreda level early marriage is legislatively prohibited. - Improving maternal nutrition through increasing space between each births and delayed marriage (after 18 years) is included in the 2^nd^ GTP and it is a multi-sectoral agenda - Presence of WDA networks, women associations and leagues at each village is an opportunity to prevent early marriage and increasing birth spacing |
| **Section6** | **Multi-sectoral collaboration** **to improve maternal nutrition** |
| 6.1 | Do you feel it is necessary for your institution to work with other sectors/institutions to address maternal nutrition? What about for adolescent girls’ nutrition?  **Participant:** yes, it is necessary because maternal nutrition cannot be addressed by only one sector that is why we are trying to work with other sectors.  **Interviewer:** Which other sectors do you feel are necessary to work with your institution?  **Participant:** education, women affairs, social justices, agriculture, woreda water committee |
| 6.2 | For multi-sectoral action that effectively works to improve maternal nutrition at all levels, what kind of change in terms of the way stakeholders work together is needed?  **Participant:** now we are appreciating multi-sectoral collaboration for nutritional interventions, what is needed is just strengthening and letting it to function. The only change needed is that, adding construction sector to the woreda steering committee, because if it is included it can facilitate in the construction of infrastructures.  **Interviewer:** What type of resistance to the needed change do you perceive, or have you experienced so far?  **Participant:** So far, we did not face challenges and resistances |
| 6.3 | To what extent does your institution participate in the multi-sectoral nutrition coordinating body at this level? **Level indicates Kebelle, woreda, zone and region**  **Participant: the** participation of our institution in the multi-sectoral nutrition coordinating body is good. For example, we are part of the technical committee in SURE program. But it needs further work.  **Summary**   - **To improve maternal nutrition** our institution must work in collaboration with other sectors, because maternal nutrition cannot be addressed by only one sector - Education office, women affairs office, social justice office, agriculture office and woreda water committee are our collaborating sectors. - The only change needed in the existing multi-sectoral collaboration is that, adding construction sector to the woreda steering committee, because if it is included it can facilitate in the construction of infrastructures |
| 4.5 | Do you have any other comments on anything that we have discussed?  **Participant:** I don’t have comments it is comprehensive and all inclusive. |
| Thank you for taking the time to discuss these issues with me today. We have learned a lot from you. As I mentioned at the start of the interview, we will remove all identifying information from the transcript of this conversation. We will make sure that no one can identify you from your comments. If you have any concerns or questions, please feel free to contact me (contact info). Thank you very much for your time. | |
