# Supplementary material for: Barriers to utilize nutrition interventions among lactating women in rural communities of Tigray, northern Ethiopia: An exploratory study
Source: PLoS One. 2021 Apr 30;16(4):e0250696. doi: 10.1371/journal.pone.0250696 (PMC8087028; doi:10.1371/journal.pone.0250696)
Supplement: S2 File — (ZIP) [file pone.0250696.s002.zip › S2_File.Doc/Woreda level and above key informants/031_IDI_MCH&Nut focal Person_Woreda Heath Ofiice_Ofla woreda.docx]

**Day3: 27/02/2010 E.C**

**Translation: In-depth interview of MCH and nutrition focal person**

Zone: south

Woreda: ofla; korem

Name of participant: Kiros

Institution of key informant: Woreda health office

Interviewer: G/medhin.B

Date: 27/02/2010 E.C.

Interview start time: 2:55

Interview end time: 4:30

Socio demographic information

|  | **Socio demographic information** | | | |
| --- | --- | --- | --- | --- |
| **Sex** | **Age** | **Marital status** | **Education level** | **occupation** |
| male | 47 | married | diploma | Government employee |

Position: MCH officer plus nutrition focal person

How long have you been in the current job/position: 12 years

**Section1: common maternal nutrition**

I: What do women (pregnant, lactating an adolescents) do to stay healthy in the community?

P: Okay, to keep the health of the pregnant or lactating, the first thing is the knowledge they have about nutrition. They need to know ‘what is balanced diet is’ is the food they have balanced and do they know how to prepare it matters. If they know all these, they can prepare and eat there by keep their health. For example if we see in the case of pregnant one, a woman should add one extra meal beyond her previous eating habit. This is because she has fetus inside her. So, she has to balance her nutrition by adding one extra meal. She does not have to take heavy foods, especially hard water. The woman should use iodized salt. In our set up, the proper utilization of is not consistence among all women. Sometimes, they just add the salt before they putdown the ‘Tsebihi’ and while it is still hot. In such case the iodine will evaporate, and the dish may end up without content and is left with the taste of the salt only. These things should correct.

During pregnancy, what she needs to do is to attend focused ante natal care in health facility. She has to go to health facility at least four times and get screened and her status. She can make all rounded examination and take necessary things for her health. From the beginning of her pregnancy, she has to take iron. But, when some women took iron, because it has a side effect of nausea, they did not continuously use it. But it has big advantage associated with bleeding that may encounter during delivery. Thus, there is some sort of problem in utilizing iron.

I: what about regarding the lactating and adolescents?

P: For adolescents, there are a lot of things that are not being done. Had we intervene on them, I believe we can stop the cycle of stunting. Therefore, we did not do anything for adolescents. There were little efforts e.g. provision of iron for adolescents in town, but now, it is not in place. Thus, it will be good if adolescents became part of nutritional intervention. Even the potential is with them, you know, they are doing a lot of hard works. The energy adolescent lose is not balanced with the energy they gain. Because it affects them, we need to work here more. When we look at lactating mother, mostly she is eager to feed her baby; she did not give care to herself. The assumption that “if I eat well, I can produce a lot of milk to baby” is not well understood by lactating mothers. There is a gap here, and we need to work more in this regard. My breast is not producing milk is a common complaint of mother and there by tend to provide additional food to the baby before six months. There is a knowledge gap on “if I eat well and drink well, I can produce sufficient milk to my baby”. So if we work on this, on nutrition, we can better perform in improving women’s health.

I: what are the common nutritional problems in this community for women and adolescents?

P: I cannot call it common or talk more on the adolescent, because, there is no nutritional intervention implemented for them. So, we need to assess, we need data. But, regarding mothers (lactating and pregnant), we measure MUAC. Most of the mothers, almost greater than 80%-85%, who come for measurement, are those who are chronically and nutritionally affected and ill ones. Those who perceive themselves as healthy do not come. This is a change that should be addressed.

Thus, the nutritional problems are common among mothers, especially lactating ones.

I: what about the type of nutritional problems, beyond lower MUAC, e.g. both macro and micronutrient deficiencies, like goiter and others?

P: when we observe the micronutrient deficiencies, look for example how we develop, we are poor to memorize what is being told. We are affected by the malnutrition cycle. In the past, goiter was widely observed in the community, but after intervention, like provision of iodine tablet and others, goiter is not common now. The biggest problem here is in creating attitudinal change. Once you give health education, people are not alert to attend it. This is related with the background of the community, and lag to bring psychological and physical change. Here is point where we want the community to develop. And the visible one is malnutrition after we measure MUAC. But it is not epidemic level, because it has highly decreased. Because our Woreda is ‘Degua’ (temperate zone), what we commonly observe is waterborne diseases. And the malnutrition is not common. There are only few kebeles that are Kola in climate. This is what I observe.

I: Is there micronutrient deficiencies (such as anemia, night blindness, goiter) in adolescents?

P: yes, it is not as common as previous ones. But, all rarely occurred in the community. Even goiter, night blindness is seen in some areas related with improper use of iodine. Use of iodized salt is extensively expanded. If you go a one household and asked her “Do you use iodine?” then she will immediately say “yes”. The problem is up on proper use of iodine. Because of these, there are few challenges.

We you come up to night blindness, the problem are occurring due to weak hygiene and sanitation practice. It is not commonly related with vitamin a deficiency. We provide Vitamin A twice a year, every six month, so very child is provided. Because such interventions are changed from campaign to routine health activity, night blindness associated with vitamin A is not a problem. As you know our Woreda is one of the areas which have high burden of trachoma related to the poor personal hygiene. People do not prefer to wash their body due to fear of cold.

Though things are not complete, we cannot more on adolescents, and we need detailed assessments on both macro and micro nutrients.

I: Do you think there is relationship between nutrition and no communicable diseases? Why?

P: Absolutely true. They have relationship. If you have any event/disease, you are not able feed enough. In this time, take a lactating mother, if she is sick and she is not taking food, and she is breast feeding, she will be severely affected in terms of nutrition. So, I believe that nutrition and no communicable diseases are highly correlated.

I: Are these problems (diet related communicable diseases) resided in the community?

P: Yes, if you go down to the community, you will find these problems.

I: what do you think on why women/girls in the community would not increase their height proportional to their age? Is it related with their nutrition? How?

P: When we look at stunting, it can be related with heredity. If astounded woman got pregnant and is not well nutrited, they will give birth to underweight baby. This time the system (cycle) is not broken. If we did not break the cycle, the problem could be intergeneration as the mother might get it from her parents. These, if pregnant woman attend the focused ante natal care, eat balanced diet and perform all advices and services given by care provider, such as taking iron and after delivery, if she immediately breast feed including the colostrum to her baby; if she exclusively breast feed the baby daily 10 to 12 times up to six months, after six months, supplementary feeding is important. And it must be balanced diet composed of one from cereals and thee from barley and vegetables, and if she feed him properly, the cycle of malnutrition could be broken. This is not commonly implemented because, 1) due to poverty, and lack of food, people may eat only one variety of food 2) the mother be burdened with a lot of tasks and then she will be tire to prepare variety of food; this is mainly due to lack of awareness.

I: What about issues related to overweight?

P: we have awareness on overweight, as malnutrition refers to both to under nutrition and over nutrition. But, our setting can be best explained by under nutrition. Over nutrition is not a problem here. Even I have never seen in my life time.

I: is there a situation where the community suffers from food insecurity? Why?

P: Now, food security issues are being strengthened using safety net and direct support. Only in case of climate change, e.g. before two year, there was drought and food insecurity was a challenge. But last year, there was enough rain, and the production was good. This year, thought it was not consistent, there was rain. On food security, this day’s things are good and are changed. Now, there is no condition that a woman sells an egg and butter, and buys oil in return. People are working hard and there is improvement. The government is also providing a support to those who are in need.

I: How about the impact of food insecurity on women/girls.

P: Last week we had a forum with office of agriculture to create awareness regarding pregnancy. If a woman is pregnant, she needs extra rest and decease a work load. Here we have little pressure. People have a tendency to withhold the benefit of safety net if pregnant woman did not work until 6 month of pregnancy.

Despite we are working to reduce the burden, there are few inconsistencies in its implementation. Such things are not the work of one sector; we rather need to collaborate with other sectors to avoid the problem.

I: Which groups of women are highly affected by the above challenges? Lactating/pregnant or adolescent?

P: Only pregnant women and mother on postnatal care up 45 days are supported by the safety net program without working. Yet, other pregnant mothers who have infants must work to get aid. So, the challenge is high for the lactating one. Working while carry baby, if the child did not breast feed on time; the advice to breast feed a baby 12 times does not mean he should touch, it rather must be real. So, this way, they had a pressure to their food security issues.

**Section two: nutrition priority in the Woreda**

I: In your opinion, what interventions do you think are priority of your Woreda to improve nutrition for pregnant?

P: Here in our Woreda, even though we are part of the Sekota declaration, the is a program call ‘SURE’ that works on pregnant women and under two children. In this program, we clearly know the number of pregnant women, under two years’ children and under six month’s infants. And it planned to improve their nutrition. This is done in collaboration with agriculture where mothers are advised for introduction of home gardening. Yes, because, if a woman has vegetables in her garden, she can prepare a balanced diet and feed her baby and herself. This intervention is in place in some households, around 10% of the poor. We provide them potato, apple and other vegetables. It is practically implemented. The task is started, and as far as the organization is present, it will be continued. As Woreda health office, we work on under-five nutrition, and SURE is specifically working on under two children and pregnant women. So we are working together.

I: what about for adolescent girls?

P: This is the area that we did not work yet. Currently, there is a plan, for example, Maret (REST) has conducted an assessment. And has a plan to provide an iron in the form of tablet or something to be drunk. I am not exactly sure on what it is, but there is an initiative. But, it is not yet begins. Because we did not work on adolescents, I cannot talk on what is strong and weak.

I: What nutritional interventions have the most resources allocated to them?

P: If we work hard on pregnant women and under two children effectively, we can bring change and even break the cycle. We can change the generation.

I: is there enough resource? Please explain it to me!

P: Now a day’s, a woman may not have all at a time, if she has three cereals, even she could have five, six varieties. These could be enough to prepare balanced diet.

Even though we put criteria to prepare balanced diet, a woman can do it from the available foods in her house. Therefore, I believe, it is good in terms of resources. If we work till the grass root level, we can achieve it and we will not discontinue because of shortage of vegetables, pulses and cereals. Even when food security is weakened, in each household, a mother has ‘teff ‘ she can sell and buy other variety and can prepare a balanced diet with what she has, that is, one from pulses and three from cereals.

I: in your Woreda, are there any activities done to improve nutrition among pregnant women? What are these?

P: Yes, we have. We usually give them education during the conference of pregnant women. It is done once in a month. In that time, there is a demonstration on how to prepare ‘porridge’ as diet which is balanced.

I: what about to lactating and adolescents?

P: we educate and demonstrate to all women who are pregnant and lactating. But, we do not have any specific intervention for adolescents. For pregnant and mother with under-five children, women development armies have also role in demonstrating how to prepare porridge (GEAT).

I: Among the nutrition interventions which one was successfully implemented and can be scale up? Why?

P: conference of pregnant women. This is because if a women is not screened, she will be advised to so during conference. Besides, we discuss on women nutrition and advice the women to deliver alt health facility. After delivery, we earlier tell her to breast feed her baby and inform her not to drain the colostrum as it is important to the baby. Generally, there significant change, but we can declare that we are free of malnutrition.

I: What about the lactating ones?

P: it is similar, except few mothers that did not adhere to the advice given by care providers, if you probe a woman for “do you give water to your baby if he has abdominal cramp?” she will say “yes” and she will justify that it because of the inducement of her mother-in-law.

**Section3: nutritional interventions that improve adolescents and maternal health**

I: what nutritional intervention in place for pregnant women? Where do they get it? Who provide it?

P: For pregnant women and lactating mother who have under-two children, 1) introducing home gardening at each household; if it is not possible at individual level, we demonstrate and educate women at the FTC (farmer training center) and health posts. But, as I said it before, there is nothing done for adolescents.

I: Do pregnant women visit health facility? What services do they get?

P: Yes, most of them get service. We have 31 health facilities of which six are health centers and 25 are health posts with two health extension worker at each health post. During the conference of pregnant women, they will be asked if they had been screened or not. If a woman did not visit health facility yet, she will be advised to go and make all the laboratory examinations at her nearby health center. The rest visits (3^rd^ and 4^th^) to health center or health post can also be made by initiation of the mother. Health extension worker are delivering all these advices at the community level. When we look at the delivery at health post, it is good though we cannot say there is no home delivery.

I: how many of the mothers attend ANC four at health facility?

P: Before last year, it was around 50% but, last year it was above 80%. Now, there are no women who discontinue ANC after 1^st^ and 2^nd^ visits as many are attending the 4^th^ ANC visit. We did not reach 100% performance, but there is always significant change in terms of ANC coverage.

I: What about in getting advice on extra meal to both pregnant and lactating mothers? Do they really do it during your supervision?

P: “Extra meal” imm: we just advise the science, to take extra meal, but on ground, there is a gap. It could be because of being busy in preparing food to her children and family. As a result, on time breakfast, on time lunch and on time dinner and extra food are rarely practiced. Only few women who have enough resource are exercising it. While some children are eating at morning and evening, the rest may only eat one times.

I: What about lactating mother?

This situation is similar. For instance, if the bay is slept, the mother would be happy as she is getting time to work other activities in the house than letting her baby wakeup and breast feed or providing him food.

I: Do women get nutritional screening?

P: first, we plan and call for screening as per the number of pregnant and lactating mother in the community. What is surprising here is that, only mother who speculates themselves as wasted will come. And after screening, all most majority, greater than 80% to 90% have malnutrition in many kebeles. In some kebele, hundred percent have MUAC less than 23cm. What you can conclude is that only women who suspect themselves or their child is stunted/wasted are coming while those who assume themselves or their children is health are left unscreened.

I: unknowingly, does it seem targeted screening?

P: yes it seems. If 60 mothers came and all became wasted, it is difficult to conclude. For example if you see a four month report of this year, out of 192 mothers, 121 had MUAC less than 23cm.

I: what in the case of lactating mothers?

P: it is similar: We screened them all at the same time. We mobilize and call for pregnant women and mothers who have less than six months old infant. And the findings are similar and seem targeted screening.

I: Despite that it seems a targeted screening; it is nice that they are being identified. What do mothers expect/benefit after screening?

P: We give them advice. After screening, we tell them that your MUAC is decreasing or your child’s MUAC is low. We advise to eat balanced and variety of foods. And tell to avoid sharing foods. Generally, we give advice and services. There some women who directly implement the advice and came up with change in the next session. We tell her again on how she regained her status.

I: How frequent is this nutritional screening done?

P: it is done every month. All children less than 2 years, pregnant and lactating mother are all screened.

I: What about food diversification during pregnancy and lactation? Even on those who are food secure, how important is this?

P: On food diversification, we frequently counsel mothers while visiting home to home. HEWs advise mothers in every opportunity. There many mothers who took it seriously and implement it, and at same time, there are women who do not really give attention to the advice. But the advice is given at meeting, through women development army and during home to home visit. But, the implementation differs according to the women.

I: what do you think to change the scenario so that every woman will implement food diversification?

P: Each woman cannot comprehend the information uniformly. Thus, we need to inform and support them continuously. Then the mother may think “they are doing this for the benefit of me and health of my child” and thereby brought change. The big thing is that sustainable health education and practical demonstration. These days, talking many times may not change individuals. It only works to wise person. But, for many of the women, it good if we show them in practice: this is cereal, this is pulse, and this is how to prepare a porridge; if possible using milk, if not using the water where vegetables had been cooked in, and the time to add iodine, will help mother to understand easily. Furthermore, we show them how to feed, how measure the size based on their age and advise them to prepare in the same procedure in their home and feed their children. The mother should not only cook one for the entire day, she rather have prepare for maximum of four hours, and she to prepare three or four times a day.

HEWs may also demonstrate at home if the woman is told to prepare the necessary material ahead. A mother may not possess a meat (Quanta), but we do not want her to worry, because she can prepare using the varieties she has at home.

I: what about on the use of iodized salt? What is the community’s’ preference?

P: I can say about 90% of the community have iodized salt. The coverage is good, except, in times when there is scarcity; few may use the ordinary one. The problem what we observe is improper utilization.

I: Are women using home gardening? What about their involvement in safety net programs?

P: Now we have started it. At kebele level, both health and agricultural extension work together; they go home to hoe together to visit under-two children. We are introducing gardening at household level. There could be limitation of land in some households, but general it being implemented nicely and resources are also being distributed. We cannot generalize that all women who have children are involved and those involved are beneficial. There could be few gaps but can corrected through continuous follow up and support.

I: what else nutrition intervention is needed here?

P: As we have described so far, as an intervention, there are BCC and safety net activities associated with pregnant women and nutrition. Women should take enough rest, have proper nutrition and visit health facilities for checkup and follow up. When we look at safety net program, it is done together with agricultural experts. When one woman is identified as pregnant, she has to get enough rest and at the same time, she will get all the benefits of safety net program without working. There was some inconsistency in some kebeles, but now, we (both sector) came to agreement.

I: What about regarding WASH? Are women getting advice on water, sanitation and hygiene services? What about the implementation?

P: Look! Here are cereals and vegetables, if it has no sanitation, it has no meaning. Because of diarrhea, and other diseases, it will lead to malnutrition. Thus, both things must go in parallel. Both nutrition and hygiene are the two faces of one coin, and it is not an exaggeration. But this is not on ground. The hygiene practice in our Woreda is too weak. Feces are not considered as bad, and is justified as this is a child’s feces and considered a harmless. Although these habits are decreasing, hygiene practices do need extra efforts. A woman should wash her hand before serving food and after using toilet. These are told to mothers and to other population. Any one should wash before eating; a woman should wash before preparing food. All these information is delivered continuously.

I: What about the use of ITN among mothers, especially among the kola (hot in terms of climate) kebeles?

P: For many year, we, an entire Woreda, had not being given ITN. But, last year ITN is distributed to the community.

I: what the reason why it is not given continuously?

P: I remember one day, ITN was given to our Woreda and is put in the warehouse. After one day, it was mentioned that it is sent wrongly, and the region immediately took it. But, in some kebeles where their climate is ‘kola’, there are incidents of malaria. E.g. in sesela, Dasus, but it is not common in terms of magnitude and there was no epidemic at all. The utilization of ITN is not as expected and there is a problem, like using it as mate, and used it to collect hey.

I: what about services relate to deworming to pregnant and lactating mothers? Who provide the service?

P: The deworming is only to children who are two to five years old. We do not have deworming service to adults including mothers. May be pregnant women in time of their 3 to 6 month of pregnancy, they may it in the health facility, But as general, we only give vitamin A and deworming service to children of six to 59 months . If a baby is given in July, he will be given in January after six months. Deworming service is delivered twice a year. In the RHD (routine health delivery), we identify eligible children and provide the service based on the schedule. But, in some health facilities, they gave them once for three months, and such system seems campaign. We evaluated it, and took actions.

I: Are pregnant women eligible to get TSF (targeted supplementary feeding)? Why?

P: Yes, they got. Mothers whose MUAC is less than 23 cm are getting supplementary feeding. TSF in involves both children and women (pregnant and lactating). The Woreda itself is part of the TSF target.

I: what about for adolescents?

P: leave it, nothing is done for them.

I: Are lactating women getting Vitamin A supplementation after they give birth?

P: At postnatal care, until 45 days, the mother is given vitamin A?

I: who else is eligible for vitamin A supplementation?

P: Children of 6to 59 months are also given vitamin A supplementation. As a treatment it can be given to others at IMCI clinic based on the algorism. Of these condition, it will not be given to any one including adolescents.

I: In your opinion, are adolescent girls provided school feeding?

P: few years ago, there was a school feeding program I few schools. But now, I do not think it is in place.

I: how do you see its necessity?

P: To decrease school absenteeism, it is good, but nutritional is not too much as it is only one variety, that is, rice. The feeding is not diversified.

I: Does the feeding program consider out school girls?

P: No. even it was not implemented in all schools. The school feeding program was in place only in areas which were severely affected by drought.

I: because of their age, adolescents might be exposed to different things. Are they linked to youth friendly services at health facilities?

P: so far, there is no specific service given to adolescents, they just get any health service like the other adults. Despite it is not separate adolescent are getting service e.g. if she pregnant, she will get the focused ANC, and STI treatment. But, there is no focused nutritional service to adolescents.

I: Which of the interventions listed above are important to women? Meaning, if we work on them, you feel we could bring change.

P: even though I could not say more, I believe iron provision to adolescent girls is best. Awareness creation is also important to mothers. Although there are resources, awareness creation is essential to bring behavioral change. With the multi tasks care providers have, enough information might not be given to women. When pregnant women do not come to community health day or health facility, we need to go to her home, and advise her accordingly, and in this regard, there are few things remained behind.

I: Which of the above interventions are effective to women (both pregnant and lactating)?

P: Usually, complementary feeding effective. It is started at 6 months for most of the infants. And feeding extra meal form ‘mitin’ is also a best practice.

I: why do you think that it effective? What special thing is done among women?

P: nothing is done special. Naturally, a mother favors to her child. When the mother is lactating and advised her to take extra meal, she will not eat, but she can correctly breast feed her baby. For example in few women I had observed that “if a mother is feeding her baby on one of its breast and on the same time if the other breast leaks, she will immediately switch it. This is a wrong practice. But now, we have educated them not to switch it as the first drops are watery in content rather they should keep feeding on using the first one. She can switch if the first one has stop draining. Generally, mothers are not good enough to feed themselves, but they are excellent for their babies. Mother do not recognize the importance of well feed other to a child.

In addition, mothers are good in breast feeding a baby 10 to 12 times a day.

I: Anything you can talk on adolescent (in and out school girls) as success stories?

P: No, we do not have.

I: Which of the above interventions are in effective to women (both pregnant and lactating)?

P: On performance, there could be variation, but all are running smooth. The limitation is the care given to women by her. Besides, there is no any intervention we do for adolescents.

I: What are challenges to implement nutrition intervention among women and adolescents?

P: Unable to address each household is a barrier, health care provider’s motivation lower as compared to the previous one. HCW are fed-up. In the community, the mother, her husband and her mother in law do not properly implement the given advice.

I: How do you evaluate the resource available to the intervention? The awareness of mothers and care givers

P: In utilization of resource, the first thing is focus or priority given, starting from federal, region and down to Woreda. If we work on nutrition, we can bring change and decrease burden of stunting. Because of improper nutrition, we observe that if five individual are dead, three are because of malnutrition. To decrease child mortality, nutrition is important. In our Woreda, we are supported by SURE.

I: How do you see the priority given for the interventions for women at Woreda level/community? Are any computing activities?

P: I do not there computing activities for nutrition. For example, if you compare HIV screening versus nutrition, nutrition should come first. There is no thing which can compute. If you see hygiene, it can go in parallel to nutrition. If we work on nutrition without hygiene, it has no meaning. There are things which can go in parallel and integrated. For example, if pregnant woman come for ANC, we can attach her to PMTCT service a time. So things are complementary to each other. If we work on nutrition, we can address the others, because they are additional. If an individual is affected by disease and he is not taking a diet he will not cure. So, nutrition has to well address till the low level; besides, we can do easily if we eat balanced and clean diet with what we have.

I: Are there any factors related to the skill care provider that affects the implementation of the intervention? How?

P: with health care provider, 1) every one do not have uniform awareness 2) even though he has awareness, he may not reflect all what he has. Firstly all providers must be skilled; this must be across all the structure: region, Woreda, kebele, heath extension and women development army. It is not enough to have only skill and just educate people; it must be practiced to bring change than simply being theoretical. If we practiced it, the community can easily understand us.

I: What about the coordination and collaboration between nutrition sensitive and specific sectors? E.g. how jointly do health and agricultural extension works together? How about its implementation?

P: The collaboration is fine. And on ground, because health and agricultural extension works together,1), the DA will receive a lot of thing s from the HEW and the HEW well get other information from the DA, e.g. how to work on home gardening, and other one o how to prepare porridge. Both are supplementary to each other.

I: Can you tell me any success or innovation that your organization uses to improve maternal nutrition that can be taken as benchmark to others?

P: you mean best practice; together with digital green, and SURE, we have video to demonstrate on how to garden and prepare food at home. We will expand this.

Preparation of porridge is started her, ‘Mikimas’ (celebrating time to start complementary feeding) to infants at 6^th^ month is innovated in this Woreda. This even is like birth day celebration. We had best practices, but there is limitation in preserving and sustaining the activities.

**Section 4: community factors affecting access to maternal nutrition interventions**

I: Are there barriers that prevent women from using the nutrition interventions like MUAC measurement, use of extra meal, deworming, any food taboo etc.? What are these?

P: so far, there was a challenge on eating sweet food. This is because “if she eats sweet foods she assume the fetus will grow and become big and she cannot give birth easily” But currently such things are solved. The second challenge is, there is gap in the food security, and people do not have more food to use as per our advice.

And the third is there is low awareness and inconsistency in implementation. Mothers think this way: “I gave birth children without any problem in the absence of heath facility” so what is the importance of your education, is a common challenge from the community. Although these challenges are decreasing, there are in a significant number.

I: what about any challenge related to the educational status of women and girls?

P: When you look at school, girls’ education good and may not have impact, but as long as we did not have focused intervention among adolescents, it can create a problem.

I: Do you think there is connection between access to transportation and utilization of services among women?

P: we have six health centers, and one health center has 5 to 6 health posts; women may not get all necessary services at health post: In such cases, there is a transportation problem when mother are going to health centers. Services give at health post and health center are not similar. But, as general health and maternal nutrition, all services are cascaded and available at the lower level. When there serve acute malnutrition with complication, there is referral, there could be a challenge in accessing transportation.

I: What about related to quality of care? Do women retreat from HF because of poor quality of service?

P: Yes, there is limitation and we addressing it. One day, it has been reported that there are many women and children with severe malnutrition. When we go and checked it, the number was low as compared to the reported one. Thus, we have to work on quality of service in addition to clean and quality data we need.

I: What about any challenge related to community belief a culture?

P: this is not common problem. But, as one the supplementations has milky content, women who are fasting are not willing to take them. We inform mothers as it has no problem when they are sick.

I: what can be done to address the above barriers to improve maternal nutrition?

P: One thing is the belief. The community should believe on the service and strive to seek it at any health facility. If we increase our efforts on health education, we can address it. Then the community can understand it. With the overlapping activities we have, our focus may be narrowed. Some of the challenges are beyond our scope. E.g. if there is no access to transport, we can construct road, besides, some need enough resources.

**Section5: Other interventions that influence adolescent and maternal nutrition and health outcomes**

I: In your opinion, why would delayed marriage (after 18 years) improve maternal nutrition?

P: that is the main point. The girl should marry after 18 years. This is because of the nutritional cycle. There are observation where there is under age marriage. There were many events that had been disengaged in connection with women affair office. The community wants honor of to their family. There is fear that the girl may start relation with other if she grows well. Thus, they wrong tell their age as if she is 18 while she is actually 16 years. Due to this there is pressure on girls. But, all there are rare observation. If we look it in nutrition perspective, if a girl is married, she will immediately be pregnant with in a year. Then, if she did not follow ANC, if she did not properly use food, and after delivery, if she does not care herself, it would be like ‘a child having a baby’ is very challenging. What I am explaining is it has effect on maternal nutrition.

I: what about related to increasing space between each birth and maternal nutrition?

P: Previously, there were problems, including among religious leaders; there huge gap in utilizing contraceptives. But, this time, there is improvement. Short birth intervals are seen among few families in remote areas. Mothers are now deciding themselves that they will give birth after two or three years.

I: What programs or activities promote increased birth intervals and to prevent early marriage in this Woreda?

P: to increase birth space, we are providing all resources needed for family planning; it could be long or short acting contraceptives. The program is already cascaded to kebele level. We provide all necessary training to care providers. Following that, health education is important to increase utilization. In doing this we are working with religious leaders. They really are helping us because they start at their home as being a model to the community.

To delay under age marriage, a girl should have a certificate form health facility for confirmation. When the girl comes for checkup of her age, she will also be linked to VCT service, and then she will be sent to Woreda justice office. We did this together with women affairs office. In this regard, we truly are working on it.

I: what about in terms of law if someone violets this?

P: there is law, and he/she will be punished accordingly. For example last year many planned marriages were disconnected.

I: In your opinion, are these programs or policies effective? Why?

P: very much! Because in every opportunity, through health extension workers, at schools, and additional, the girls are getting education, they also inform us.

I: What are the community factors that affect age at first marriage?

P: commonly, it is the need of the family. There is also a pressure from the family of the male too. When they saw the girl physical good, they had fear that she can start intercourse with other men, and to avoid this, they prefer to let her marry early. The community knows that age to marriage is 18 years, they are deliberately saying she 18 when she actually is 16 years old. Every farmer knows this fact, but to he wants marriage if girl looks physically mature. This is because they thought there could not be a problem even if she gives birth.

I: what about in terms of law? Policy factors that affect age at first marriage

P: To take action, they must be recognized first. If it does, they just reverse the marriage. We have a structure to follow this age checkup and HIV test are done together at health center, when there is dilemma, the girl is referred to hospital.

I: is there any other opportunity to prevent early marriage and increase birth spacing?

P: one thing to delay early marriage is to strengthen the current activities. Extensive awareness creation at school and involvement of office of justice and other sectors like women affairs is important. To increase birth space, provision of enough resource for contraceptive and awareness creation on the benefits each method is needed.

**Section 6: Multi-sectorial collaboration to improve maternal nutrition**

I: Do you feel it is necessary for your institution to work with other sectors/institutions to address maternal nutrition?

P: now, we have started it through SURE. There is a team from youth office, women affair, and social affair, and education, public relation including agriculture, finance and health. This is a technical team from seven sectors, if we work properly in all structure, we can change maternal nutrition. We have joint plan. If al, we strengthened these activities, we can bring change in maternal nutrition.

I: Is there anything consider for adolescent during the multi-sectorial collaboration?

P: No, but from now onwards we will do more, especially with education both in and out school, I hope we can work with the youth office.

I: is there any other sector that can potentially collaborate with your institution to improve maternal health? E.g. The different association

P: I am not sure, but regarding resources and finance, we need the help of non-governmental organizations and association.

I: How effective are the multi-sectorial coordination? For example with a agriculture

P: What is best for me is home gardening and It is going smooth and we need to extend it more. Others are supportive. E.g. if we look at women affair, the mother is already member of the association. If we have problem in food security, we are working with the office of food security.

I: Is there any challenge that needs to be improved during the multi-sectorial coordination?

P: Not yet, because we are staring multi-sectorial coordination. But, with agriculture, there are some inconsistencies. Sometime the HEW and DA are working independently, and we had evaluated it together. If we work in parallel we, can solve the problem occurring on maternal nutrition.

I: Is there any resistance at lower level, from the expert and fro the community?

P: There is no resistance. Rarely, there is scarcity of land for home gardening, overlapping of activities to low level providers, not giving attention. All these may compromise the performance.

I: How active is your institution during multi-sectorial coordination? In taking the lead as it for mother’s health

P: During budget allocation, we had evaluated the status of maternal nutrition. We had also check list for follow up.

I: What needs to be done to improve the capacity of these bodies/platforms for effective coordination?

P: I believe that we have to work capacity building activities at all level including the community. Thus we can perform more.

I: Do you have any other comments on anything that we have discussed? Lessons learned. E.g. Adolescent nutrition, multi-sectorial coordination etc.

Regarding multi-sectorial collaboration, it is on the beginning, and we have to strengthen it.

And on adolescent nutrition, we have to start and work more including resource and budget. We can change the generation if we strengthen all activities.

I: what opportunities are there?

P: we have a working structure starting from the community through health extension workers, women development army and men development army; we have all these structures. And at to level, there is also multi-sectorial collaboration. Beyond that, it is present at regional too. What is needed here is resource, i.e. budget. At Woreda level, the issue of budget is beyond its capacity, thus if we get support, everything is possible.

I: Do you have any other comments that you want to add?

P: You know what it means. Regarding nutrition, there are a number of partners who wants to help and sill are working. Previously, some start and discontinue, and interventions are stop. But now, because it is involved within the government system, I do not there will a challenge. If we work, we can achieve it. Because of the structures we have, we are better. The big thing is budget constraint which limits the effort we want. Beside this, there is a problem in access to transportation in some kebeles. We also need enough supply, if so we can do more.

Thank you.

I: Thank you very much for your time and energy!!

**Summary**

**Section1: common maternal nutrition**

Knowledge about nutrition is important. Women need to know ‘what is balanced diet.

Most of the mothers, almost greater than 80%-85%, who come for MUAC measurement, are those who are chronically and nutritionally affected and ill ones

Night blindness is occurring due to weak hygiene and sanitation practice; it is not commonly related with vitamin a deficiency.

Over nutrition is not a problem here, and lactating mothers are commonly affected bb food insecurity.

**Section two: nutrition priority in the Woreda**

In this Woreda, there is a program call ‘SURE’ that works on pregnant women and under two children. It is done in collaboration with agriculture where mothers are advised for introduction of home gardening.

Adolescent girl’s nutrition is the area that we did not work yet.

During the conference of pregnant women, there is a demonstration on how to prepare ‘porridge’ as diet which is balanced.

**Section3: nutritional interventions that improve adolescents and maternal health**

Home gardening for each household is demonstrated at individual level and at the FTC (farmer training center) and health posts.

Nutritional screening is done every month. All children less than 2 years, pregnant and lactating mother are all screened.

The deworming is only to children who are two to five years old; we do not have deworming service to adults including mothers.

As best practice, together with digital green, and SURE, we have video to demonstrate on how to garden and prepare food at home. Besides, preparation of porridge is started her, ‘Mikimas’ (celebrating time to start complementary feeding) to infants at 6^th^ month is innovated in this Woreda.

**Section 4: community factors affecting access to maternal nutrition interventions**

Eating sweet food is a challenge: “if pregnant woman eat sweet foods she assume the fetus will grow and become big and she cannot give birth easily”

Low awareness of mothers and limitation in quality of care are affecting access to nutritional services

**Section5: Other interventions that influence adolescent and maternal nutrition and health outcomes**

There is fear that the girl may start relationship with other men if she grows well. Thus, parents intentionally tell as if she is 18 while she is actually 16 years.

To delay under age marriage, a girl should have a certificate form health facility for confirmation.

To increase birth space, provision of enough resource for contraceptive and awareness creation on the benefits each method is needed.

**Section 6: Multi-sectorial collaboration to improve maternal nutrition**

There is multi-sectorial collaboration started through SURE. There is a technical team from youth office, women affair, and social affair, and education, public relation including agriculture, finance and health.

We have a working structure starting from the community through health extension workers, women development army and men development army. This is an opportunity to address women nutrition through multi-sectorial collaboration. But, the big thing is budget constraint which limits the effort we want.
