# Supplementary material for: Barriers to utilize nutrition interventions among lactating women in rural communities of Tigray, northern Ethiopia: An exploratory study
Source: PLoS One. 2021 Apr 30;16(4):e0250696. doi: 10.1371/journal.pone.0250696 (PMC8087028; doi:10.1371/journal.pone.0250696)
Supplement: S2 File — (ZIP) [file pone.0250696.s002.zip › S2_File.Doc/Woreda level and above key informants/035_IDI_Head Women leage office_Ofla woreda.docx]

**In-depth Interview with Women League Leader in Korem Woreda**

Zone: South

Woreda: Ofla Kebelle: 03

Name of Key Informant: Azmera Tadesse

Institution of key Informant: Women League

Name of interviewer: Measho G/slassie

Date of interview: 06.11.2017

Start time; 8:36 AM End time; 10:08AM

**Interviewee Professional Information**

Age: 30

Gender: Female

Highest level of completed education: bachelor degree

Current job/position: Women league head

Work experience in the current position: 8 months

**I**: Interviewer **P**: Participant

I: Thank you very much for your willingness to participate in this study; when I come to our first discussion topic; what do women or mothers do to stay healthy in this community?

P: Currently, whether a women or other any community member has to correct their feeding practices to stay healthy and to live longer. When we say feeding, it is not about filling your abdomen rather you have to eat balanced diet from all what you have or from your resources. In addition, maintaining personal and environment hygiene is very mandatory for health. Nowadays if they eat unhygienic foods, they will face many problems. We can create a healthy citizen if they used balanced diet from their available resources and in addition if they maintained their personal and environmental hygiene. The good thing we have currently is that almost you will not get anyone who couldn’t understand the importance of balanced diet. The information about balanced diet is well disseminated in the community and schools. The community has well understood about the importance of balanced diet, and they know that eating food is not for filling the abdomen rather it is used for maintaining their health. Beyond that, the community associates some foods and drinks with the intelligence of the people. For example, they assume that if they eat balanced diet they will be strong, healthy and intelligent. So, based on their capacity, they have to eat diversified foods from the cereals, fruits and vegetables. Therefore, they can maintain their health; first by eating balanced diet and second, by maintaining their personal and environmental hygiene.

I: Good, as I have told you before, the main focus of this study is on the nutrition of pregnant women, lactating women and adolescent girls. So, could you describe little more about what pregnant women should do to stay healthy in this community?

P: In the past, for example before I come to this office, including me our understanding about pregnant women was poor. We were considering the pregnant mother’s feeding just only for herself. But now everybody including the layman person can tell you that the pregnant mother’s feeding is also important for her baby. Therefore, it has been well promoted in schools and other clubs that pregnant mothers should eat balanced diet from conception to delivery. Without exaggeration, currently there is very good awareness about the importance of feeding during pregnancy. Of course it is not only awareness but also they are practically applying it. Their husband’s attitude is also changing. They are supporting their wives for better health of their child and wife. In the past, important foods were only prepared and stored for their delivery. Even after delivery, the prepared food was not given for the delivered mother instead it was given for others who supported her during delivery as festive meal. For example, as far as I know, meat was only prepared after delivery, however now they (husbands) are preparing meat for their pregnant wives. Hence, feeding during pregnancy is mandatory. However, as I have told you before, the food should be balanced and diversified, but not just for the sake of filling the abdomen. Practically we are observing good feeding practice during pregnancy as per their resources. But the problem is if they have gastritis disease, they many not eat meat or other balanced diet. I am sure there will not be 0.5% pregnant mothers who do not eat balanced diet. There may around 0.11% who does not use balanced diet during their pregnancy. So, practically we are observing them while using balanced diet. This indicates there is an attitude change in the community. Even it is not only an attitude change; rather they are using it practically. This change is because of the awareness created in the community by the health workers, women affaires and other sectors.

I: Good, in addition to the pregnant women, could you describe about what the lactating women should do to stay healthy in this community?

P: Currently, you will not get a pregnant woman who doesn’t follow antenatal care. They are also following their postnatal care. The postnatal care will help to monitor the health status of both the baby and the mother. So, they are following their postnatal care.

I: Ok, in addition to what you have described before, could you describe about what the adolescent girls (within the age group of 10 to 19) should do to stay healthy in this community?

P: In my understanding, both the adolescent girls and boys should be given more attention about their nutrition. Still now, awareness about adolescent girls’ nutrition is not created. We have been more working on the under seven children because we have been informed that the nutrition for the under seven children is more important for their mental development. But, there is no any special work done on the adolescent girls’ nutrition.

I: Ok, why do you think special attention is not given for the adolescent girls’ nutrition?

P: I don’t think the adolescent nutrition is understood from the top to the bottom. We have been working to protect them from rap, migration early marriage through establishing women affairs and women union (“awedadiba deki anestiyo”) in schools, because most of the adolescent girls are found in schools. So, we have established the women affaires from the Kebelle to the Woreda to protect the adolescent girls and our main priority was protecting the women from the above mentioned problems. But, we didn’t work on the adolescent nutrition.

I: Good, when I proceed to the next interview; what are the common nutrition problems in this community for the women and adolescents?

P: There is a follow up for the children born from the economically poor family. Currently everyone goes to the health facility if they have any health problems. The health workers usually measure the children to know their nutritional status, and if they are underweight, they will be given fafa. Since the fafa by itself is not enough, their mothers are counseled to prepare additional foods for their child. As a result, there are good changes in our community with regards to children nutrition, because most of the time we are closely working with the kebeles.

I: Good, you have told me that there are underweight children who are taking fafa in this community. How do you describe the average number of underweight children in this community?

P: They are very small in number. It is observed only in two of our Kebelles. So, it is not common in most of the Kebelles, because, as I have told you before the awareness of the community is improved. If the community understood the importance of nutrition, they will implement it. For example, in relation to the health care, mothers living with HIV/AIDS delivering babies free of the virus and they are also following proper feeding practices. So, the children in this community are healthy.

I: Well, you have told me that under nutrition is observed in some of the children of this community. How do you describe the nutrition problems in the pregnant mothers of this community?

P: There is no any nutrition related problem in pregnant women in this community.

I: Ok, are there any pregnant women who are given fafa after measuring in their upper left arm (Showing her in my upper arm) in this community.

P: No, I have not seen any pregnant women taking fafa in this community.

I: In addition, how do you see the nutritional problems in the lactating women and adolescent girls in this community?

P: The nutrition program has its own focal person and budget in the health sector. In addition, since all responsible bodies are aggressively working on it with the aim of creating healthy and productive successor citizens, I have not seen any nutritional problems in our community as per our evaluations and reports.

I: Good, the nutritional problems in pregnant and lactating women and adolescent girls are commonly observed in other communities. However, you told me that the problem is not common in this community? Why do you think it is not common in this community? I would like you to tell me the special activities and strategies implemented in this community to prevent nutritional problems in women and adolescents.

P: As I have told you before, we have not done anything on adolescent girls’ nutrition. But, the main reason for the absence of nutrition problems in this community is due to the fact that all responsible bodies are responsibly working on it in collaboration with all stakeholders. As a result, as I have told you before, there are few children (“I don’t know their exam number right now”) with nutritional problem and they are currently taking fafa, but we don’t have any problem on the mothers. As for the adolescent girls, we have not worked so far, but we are planning to consider it in the future.

I: Good, you told me that your office is planning to work on adolescents’ nutrition of this community. Could you please tell me on what you are planning to do on adolescent nutrition?

P: It is the adolescent girls who are expected to give birth by tomorrow; in addition, the child is more near to the adolescent mother than his father. Even though they are far from pregnancy and child delivery, they should know the importance of adolescent and pregnant women feeding. We have agreed to create awareness about adolescent nutrition; despite we have not started it yet. The adolescents are very important parts of the community because they will deliver and develop a child by tomorrow. Even though both the father and mother are equality responsible for child development, the mother is more close to the child. So, we are planning to make it one of our priority areas.

I: Could you describe an example of activities that you are planning to do?

P: For example, we have established strong adolescent girls’ networks (“nay dekianestiyo wudabe”) in schools. Therefore, we are planning to give them a training on adolescent nutrition so as they will educate their family members. We have also created a good coordination between the women affaire of the Kebelle and school girls network. So, we are planning to use the existing networks to create awareness about adolescent nutrition in the community.

I: Ok, are there pregnant women or lactating women or adolescent girls who use Plumpy’Nut in your community?

P: The plumpy,Nut is given for some of the children who have nutrition problems, but we don’t have any women who takes it. There are no as such nutritionally harmed women who require Plumpy’Nut.

I: In addition, there are very important micronutrients for better health of mothers like vitamin A for night blindness and a tablet for prevention of blood shortage. So, are there pregnant women who take the mentioned micronutrients in this community?

P: Yes, there are pregnant women who take either the tablet or in the form of syrup to prevent blood shortage. But, most of them take the tablet.

I: Who are taking the tablet?

P: The pregnant women take the tablet to prevent blood shortage; however, if they have gastritis or if they don’t like it, they will be given in the form of syrup.

I: Ok, are there lactating women or adolescent girls who take treatments for blood shortage or night blindness in this community?

P: I don’t know.

I: How do you describe the magnitude of goiter (caused by shortage of iodine nutrient) in this community?

P: In the past (not recently), goiter was common in this community; especially it was more common in selected areas like “HUGUMBRDA and SHIMBRKIT” due to the water they drink. Even the residents of that area knew that the goiter is caused by the water they drink. They know the water sources that cause the goiter disease and that of the water sources that doesn’t cause the disease. In the past, the community was using a salt without iodine, but nowadays, majority of the people are using the iodized salt because they have seen the advantages and disadvantages of it. But the people have to check it while buying the iodized salt, because the sellers my cheat them by providing a grinded non-iodized salt in the shops. In my childhood time, almost majority of the people had goiter, but now it is almost eliminated due to the fact that they are using iodized salt. As I have told you, people say that the water of the above mentioned areas were causing the disease, but now due the government is constructing spring water and due to the iodized water, the problem has decreased.

I: Good, from your observation, do you think all residents of this community are using iodized salt?

P: I can’t say 100% of the people are using iodized salt, because there might be few people who don’t use it. But, I think most people prefer the iodized salt not only due to its health benefits, but also due to it is processed (unlike the other one, it doesn’t require grinding and preparation).

I: From your observation, can you tell me why majority of the people use the iodized salt and why few people prefer the non-iodized salt?

P: As I have told you before, majority of the people use the iodized salt. But at the same time they use the other salt (“gamfur chew”) for the preparation of spices, pepper and others. So, it is wrong to say that 100% of people are using the iodized salt. I don’t think the people are using the non-iodized salt; rather it could be due to their adaptation or familiarity with the other salt.

I: Good, do you think there is association between nutrition and noncommunicable disease?

P: People may acquire disease due to different reasons. Some people may be sick due to shortage of food, while others could acquire it from other sources.

I: Could you tell me some of the noncommunicable diseases associated with nutritional problem?

P: For example there is stunting. If you roughly compare the people from Tigray and other regions, we are little bit shorter than the others. Proper food is fundamental to create brilliant and innovative citizens. So, we have to give more attention for the diet of the pregnant mother and the children, mainly from conception until the child reaches seven years.

I: Good, you have reflected that shortage of food can lead to stunting. How do you see the magnitude of stunting among the adolescent girls and women in this community?

P: It is not as such common in this community. For example, when I observe people in other places during my field trip, they are too long compared with the people of our community. I always ask myself on why we are relatively shorter than the other people. It may not be due to shortage of food, but still there is difference from one community to other community. For example, from my observation, when we compare Ofla with other woredas or Tigray with other regions, the other people are relatively bigger [laughing]. But, I have not seen a severely stunted adult person which needs extra support; rather I am talking my general overview about the community.

I: Ok, In addition to stunting, how do you see the availability underweight or low weight for their age women or adolescent girls in this community?

P: As I have described in the beginning, there are no underweight women or adolescents in this community. There is big difference between the past and the current living standard of the community.

I: Good, how do you describe the overall food security of this community?

P: Well: food security of this community is good. From my previous observation, most of the community members were food insecure some years before, especially during October month. But not, you will not get any household which have food shortage. I am telling you from my practical experience, I was born in this community, I had also attended my education here and my previous working institution was also in this community. So, I perfectly know how this community has been changed. When I was a student, almost all people whether they are rich or poor were suffering from food shortage during October, but now there is no anyone who have food shortage. For example, we are now in October moth, I have not seen anyone who has food shortage. The main reason for the food security in this community is that their working culture and awareness has been changed. They are working hard by borrowing money and as a result they are getting profit and eating without any problem. All responsible bodies are also closely working in collaboration with the relevant stakeholders to ensure and maintain food security of this community.

I: Good, do you think food insecurity will more affect the pregnant women, lactating women and the adolescent girls?

P: Most of the time food insecurity affects primarily the women because most of the women spend their time in their home. If they don’t have enough food for their children, ultimately they will be psychologically harmed. When we come to the adolescent girls, despite most of the problems are solved currently, most of the time the girls spend their time in their home while the male can go anywhere. So, the girls could also be affected psychologically.

I: Good, is obesity common in adolescent girls or women in this community?

P: No, how it will come to this community [laugh]

I: Ok, what interventions do you think are the priorities of this woreda to improve the nutrition of pregnant and lactating women in this community?

P: The nutrition program has its own focal person and it has also its own budget in the health care, and many stakeholders are collaborating on it. Nutrition education is given for the community by the nongovernmental organizations and health workers. So, all responsible people and sectors are working on nutrition.

I: Good, can you tell me successful interventions implemented by this woreda and particularly by the women’s league for improving pregnant women’s nutrition?

P: The woreda level women affairs and stakeholders have included women’s nutrition in their plan. In addition, the woreda has fronts with the sectors and stakeholders. So, the fronts have plans on women’s nutrition. The front’s plan is also cascaded to kebeles and further to the women development army. Therefore, responsible bodies at all levels are working based on their plan.

I: Ok, could you tell me any specific activities included in your plan related with improving pregnant women’s nutrition?

P: One activity included in our plan is improving pregnant women’s nutrition through engaging them in the safety net program. They can benefit themselves by working in the safety net program. They are equally paid with others in the safety net program. In addition, the women development army are educating the pregnant women to prepare and eat balanced diet based what they have in their home.

I: Good, what you have told me, what are the specific roles of women’s league in improving the women and adolescent girls’ nutrition?

P: As I have told you before, we have different women unions (“timret”) like women association, women affairs and women’s leagues at woreda and kebeles levels. The woreda level union’s plan is cascaded to the kebeles. All of the union’s work in collaboration and they are also integrately working with the women development army. As a women union, we are working on health of the community among the others, especially on the nutrition of women and children. As I have told you before, we have not worked on the nutrition of adolescent girls. However, the nutrition of children and women are one of our main priority areas. We don’t work separately, rather we work intergrately as a union and we are also working with different stakeholders to achieve our goal.

I: Good, can you tell me a specific intervention in place with allocated budget for improving women nutrition?

P: As to the budget, it is allocated by the health office and we have health extension workers in the bottom. The good opportunity with the women related work is that we have so many stakeholders like Grad and Action Aid. So, we are closely working with this stakeholders and the health extension workers and you will not get any plan of this offices which doesn’t include women and children nutrition. We are also following the implementation of the plans.

I: Good, you have told me that your office is working in collaboration with different stakeholders. So, could you please further describe the stakeholders and their roles in improving women nutrition?

P: For example, we are not preparing the fafa and PlumpyNut for women and children rather we are supported by the stakeholders.

I: Ok, could you tell me specific nutrition related interventions that your institution has not worked yet but has a plan to work?

P: Our main priority area is on women. Since nutrition is fundamental for all citizens, we are expected to work on improving women’s nutrition. We will also work on the nutrition of the adolescent girls because we have not worked yet on this area. In addition, we will expand the children nutrition because we are given a big responsibility to produce good citizens. Everyone should answer any questions related with nutrition, which means awareness has to be created in the community.

I: Good, can you tell me some of the most successful nutritional interventions for pregnant and lactating women in your woreda?

P: There are no nutrition related problems in pregnant and lactating women of this community. This indicates the nutrition interventions are properly working in this community. In addition, we are practically observing the changes. The pregnant women are following their antenatal care and the health workers are also measuring their nutritional status. Moreover, the health workers and health extension workers are educating and following them about their nutrition. In addition, all sectors and stakeholders are responsibly working on it.

I: Good, you have told me that the health workers and health extension workers are providing relevant nutrition services for the pregnant women. Could you tell me some of the nutrition services given for the pregnant women by the health extension workers and other health workers?

P: I don’t have detail knowledge on this issue and I don’t like to tell you what I don’t know practically. In fact we have reports on antenatal and postnatal follow up and child delivery, but I don’t have the knowledge on the specific nutrition services during follow up or delivery.

I: How do you describe the nutritional counseling given for pregnant and lactating women in the health facilities?

P: let alone the mother went to the health facility for pregnancy follow up; the nutritional education is given in almost every gathering or meetings. They should not eat for the sake of abdominal filling instead they should eat balanced diet. The balanced nutrition includes vegetables, fruits and others. They may not get all food types at the same time and from the same place, but at least they should use it at different times. So, the nutrition education is given by health extension workers, women development army, woreda stakeholders and other responsible sectors. Moreover, the education is given even at small gatherings. The repeated education has changed the community understanding about balanced diet. In the past, the people were not eating nutritious foods like honey, onion and oils, but now the community are eating balanced diet due the nutritional education given to them.

I: Ok, you have told me that many nutritional interventions like nutrition education is being given for pregnant women in this community. What about the interventions for lactating women?

P: It is similar with the pregnant women. The nutrition education is given during delivery, postnatal follow up, during meetings, during discussion. Let alone for the lactating and pregnant women, every person is advised to eat balanced diet.

I: Ok, do the pregnant women screened for their nutritional status?

P: Yes, they get holistic services during their antenatal care. During their ANC follow up, they get screened for shortage of food and if they have shortage of blood they will be given a tablet or syrup. They also make a blood diagnosis for HIV/AIDS and Hepatitis in the health facilities.

I: Ok, how do you describe the counseling given for pregnant and lactating women about food diversification?

P: They are advised to eat meat and meat products, vegetables, fruits, and cereals like barley, wheat and legumes. If possible, it is advisable to eat from all types, if not; we have to properly use what we have.

I: Ok, in fact you have well told me about iodized salt utilization in this community. Now, I would like you to tell me about the advice given for pregnant women on iodized salt utilization?

P: Advice is given for the community to use iodized salt. Iodized salt is very important not only for the prevention of goiter but also it is used for decreasing pain during menstruation.

I: Good, do the pregnant women advised to engage in nutrition sensitive agriculture such as home gardening?

P: Yes, they are advised to have home gardening and to engage in irrigation, and of course there are many women who have home gardening and irrigation. There are also households who sell the home garden products beyond their consumption. In the past, the community was only selling their honey, but now they realized that honey consumption can improve their health and as a result they are consuming it by themselves.

I: good, how do you describe the need to involve pregnant and lactating women in the safety net program?

P: The safety net program has been helping the women to benefit from working. The pregnant women work in the safety net program for some months and later she will get the benefits of the program for free during her pregnancy and maternity leave. The program has improved the economy and the work culture of the women and other community members. Once a community member is working in the safety net program, he/she is expected to graduate after some times and he/she will not be involved in the safety net program again, which means the household has improved his economic status.

I: Ok, for how is a community member expected to stay in the safety net program?

P: I don’t know it for a time being.

I: Are the pregnant women given the support for free or they are expected to work?

P: Why they should be given for free? They are expected to work. A three, four or five moth’s pregnancy cannot prevent them from working. May be they are expected to get rest after 6 or seven months of pregnancy.

I: Good, What other related interventions are there in the community for improving pregnant and lactating women?

P: they have the awareness, but I don’t know other similar interventions in the community.

I: Good, how do you describe the advice given for pregnant and lactating women on water, hygiene and sanitation?

P: As I have told you before, we have the health extension workers in the Kebelle. As their main role, the health extension workers are expected monitor in-house and environmental sanitation, toilet utilization, availability of hand washing facility and availability of solid waste disposal site. We are also integrately monitoring the implementation of the hygiene and sanitation packages using our structures from the top to the bottom; woreda women union, Kebelle women union, women development army, cell leadership (“wahyo amerarha”) and women league. They are also educating the women to use modern kitten in their home.

I: How about the sanitation related education given for adolescent girls?

P: “The environment by itself is a teacher”. So, the adolescent girls are getting health education from the schools and they are implementing what they have learned in schools in their homes.

I: Good, how do you describe the advice given for pregnant women, lactating women and adolescent girls to use insecticide treated nets?

P: Most of the kebeles of this woreda are highlands, in which mosquitos are not found. However, the communities (whether pregnant or not) living in the semi-highland areas are educated to use the insecticide treated nets. Generally, the education is given for the community to use the ITN to protect children not only from mosquitos but also from other insects. But, unlike the other low land areas, the ITN is not given for this community for free with the exception of the few semi-highland kebeles because the disease is not common in our area.

I: Ok, are the pregnant women given deworming services?

P: Yes, a holistic diagnosis is given for the pregnant women. Health education is given and they have also antenatal care follow up. During the antenatal care follow up, the made holistic diagnosis and they are given necessary treatments accordingly.

I: Good, are there pregnant or lactating women included under targeted supplementary feeding in this community?

P: There are no pregnant or lactating women supported by targeted supplementary feeding in this community. But the antenatal and postnatal care follow up is very impressive in this area without exaggeration.

I: Good, are lactating women getting Vitamin Supplementation after they give birth?

P: It depends. If she has vitamin A deficiency, she will be given or if she has vitamin B deficiency she will be give vitamin B. During delivery, the health works know what the mother needs and he gives her what she needs.

I: Good, are the adolescent girls given school feeding in this community?

P: School feeding is not available in this community. It is given in few schools found in other communities. But, there are schools who give shoes. For example, ADELA, MENKER/ BATSIH have given shoes for the students. The school feeding is given in the communities which have food shortage.

I: what about the school feeding or other support for the out of school adolescents?

P: Almost all adolescents are attending their education. The women development army in collaboration with health extension workers and Kebelle administration are providing education about adolescent education to the community in everywhere including in religious churches.

I: good, in your opinion, are the adolescent girls are linked with youth friendly reproductive health facilities?

P: Repetitive health education is given to the girls to prevent themselves from health problems like HIV/AIDS. We have not worked on their nutrition, but we are aggressively working health education of the adolescents.

I: Ok, do the adolescents get their preferred family planning methods in their preferred place?

P: There is condom everywhere including in schools and they can get the pills in the health center.

I: Good, in your opinion, which of the above interventions for the pregnant and lactating women and adolescent girls are effectively implemented in your community? 1:04

P: Among the intervention, antenatal and postnatal follow up are very effective in this community. We are practically implementing the quote “Women should not die while giving life”. In addition, children nutrition for preventing stunting and wasting is effective in this community. The main gap we have is on adolescent girls’ nutrition and we are planning to work on it in collaboration with different stakeholders. Hence, we are effectively working on women (during pregnancy, delivery and after delivery) and under seven years old children. We are also effective in providing youth friendly services. For example, they can get condom anywhere in their schools and in our offices to protect them from different health problems. Whereas, they can get the pills from the women affaires office in the schools and they can also get the injectable methods in the health facilities.

I: Good, what do you think are the main challenges to implement the nutrition interventions for the pregnant, lactating and adolescent girls?

P: In the past there was an awareness problem in the community, but now it is no more a challenge. In addition, to implement the interventions, it requires a capacity, but if they have good awareness it can be implemented based on what they have.

I: As you have well explained before, the nutritional interventions for adolescent girls are not well implemented. What are the main challenges for its implementation?

P: Lack of giving attention. It could have been implemented like the other intervention, had it was given attention by all responsible offices.

I: Ok, how do you evaluate the collaboration and coordination between different sectors and stakeholders in implementing the nutrition interventions?

P: the stakeholders are working on it with full sense of ownership and sense of integrity. So it is very good.

I: Ok, can you tell me any innovative approaches or exemplary experiences that your office has worked to improve pregnant and lactating women in your community?

P: I am not sure whether it is an exemplary experience or not but we have made impressive changes on women and under seven children nutrition.

I: Ok, what barriers do you think are preventing the pregnant, lactating and adolescent girls from using the interventions that we have discussed before?

P: The main barrier from using the nutrition services is shortage of resources or capacity. For example, if the mother does not have capacity, she may not eat balanced diet like meant and milk products. So, the nutrition service use depends on what you have at home.

I: Good, how do you describe the barriers for the nutrition services use in relation with the community awareness?

P: There is no awareness problem. Almost everyone knows the importance of pregnant and lactating women nutrition.

I: Ok, do you think transport access is a barrier for the nutrition services utilization in this community?

P: When we see access for health services, we have six health centers, we have also health posts. In addition we have health extension workers at Kebelle level who are responsible to follow the pregnant and lactating women in the Kebelle. In fact we have shortage of ambulance and some of the kebeles are difficult to reach during summer time due to mud roads and are far from the center. But with all the infrastructural and facility challenges, we have not faced major problems yet.

I: Ok, how do you see the quality of nutrition service provision for pregnant, lactating and adolescent women?

P: The health extension workers, women development army and stakeholders are working together and they complement each other. If one fills the gap of the other and vice versa. There is also enough manpower to provide the nutritional or other services. So, the quality service provision in this community is good.

I: Good, what does the community beliefs looks like towards the nutrition interventions for the women and adolescent girls?

P: Currently, they have good attitude towards the interventions. The people have well understood about the relevance of consuming balanced diet whether to prevent themselves from stunting, underweight or other problems. However, the preparation of food depends on what they have at home. As a result, I have not seen major nutrition related problems both in the women and children.

I: Now I would like you to describe the community beliefs towards gender equality in nutrition and the availability of associated food taboos in this community?

P: In the past, there were bad beliefs in the community. For example, the children and adolescents were eating the leftover food of the adults, but now the children are not willing to eat leftover food. The good thing about the current generation is that they are changing due to the health education given by radios, televisions and other Medias. Currently, there is no separate food prepared for adults, children, men or women. If a household has one boy and one girl, there is no feeding difference between them. In the past, the term “let him eat” was commonly accepted in the community, but currently this concept is eliminated. The students have full confidence and democracy in their school and they are practicing is at their home as well. I cannot say the problem is 100% solved, but you don’t see the problems most of the time. There was a time where the children are expected to wait outside the house until the adults finish their dinner or lunch. Therefore, currently there is no feeding difference between children, men or women.

I: Good, how can some of the barriers you mentioned before be addressed to improve nutrition of pregnant and lactating women and adolescent girls?

P: Well; changing the community understanding means changing their mind. You cannot bring radical change in short period of time; however continuous education is being given for the community and we are solving the barriers step by step. It could be difficulty to totally change the community barriers at the same time. Let alone the community, persons born from one mother are different. So, it is difficult to say 100% of the community is changed.

I: Good, how do you describe the importance delayed (after 18) marriage for improving maternal nutrition?

P: If she married after 18, she will have the strength and capacity to work and feed herself and if she eats properly she will give a healthy child. As I have told you we are producing citizens. So, to produce productive citizen, delayed marriage and maternal nutrition should be given more emphasis. Nutrition is mandatory for all, but the nutrition for the girls and women is even more important.

I: Good, can you tell me the strategies, policies or activities in place in this woreda to prevent early marriage?

P: Early marriage is prohibited by our law. First, before going to punishment, it is important to increase the awareness of the community on early marriage. Second, we are continuously educating the community of each kebeles about early marriage and its punishments. But the main and the most important is that the schools are actively working with women affairs and unions to eliminate early marriage. When we see the monitoring of the early marriage, we have different responsible bodies at Kebelles starting with the women development army, administrators (“fetsemti”) and women unions (women affairs and women league). As per our constitution, early marriage is an attack on girls. We are very successful in preventing early marriage; despite we didn’t work on the adolescent nutrition.

I: What is the punishment for those who practiced early marriage?

P: “We hung each other before marriage” [laugh]. In fact it has punishment (even they may be imprisoned), but we are mainly working on its prevention than the punishment. We focus on preventing the early marriage through collecting information about the candidates for marriage and their age from school and others in collaboration with women union of the kebelle, woreda and other stakeholders; because we cannot save the early married women whether we punished the parents or not.

I: How do you describe the awareness of the community of early marriage?

P: Almost it is totally changed. The community has understood the problems and even the criminality of early marriage, but still there could be very few people who still practice it. But sometimes we face difficulty to confirm the age of the adolescents and lack of genuine testimony in the community because currently most of the adolescent’s girls are long though they are still under age. As a result, we are closely working with the health facilities in confirming the age of the girls.

I: How is your collaboration with the religious organizations about early marriage?

P: The religious leaders are working with us in educating the community about early marriage.

I: Ok, how do you describe the importance of child spacing on maternal nutrition?

P: If the give birth over birth, the child will be affected. As you know the baby should take only breast milk up to six months of age. So, in order to give enough breast milk, the mother should take balanced diet. So, spacing is important both for the mother and her child. We are also educating the community to space their delivery and even the religious leaders are encouraging child spacing. In addition, there are important long and short term family planning methods for child spacing. As a result, the community are spacing their children.

I: What specific interventions are in place at all levels from zone to kebelle to encourage child spacing?

P: capacity building training is given step by step at all levels. Example, the woreda provides training for the Kebelles leaders, the kebelle providing training for the women development army and finally the women development army provides training for the community.

I: Good, you have told me before that many sectors and stakeholders are currently working on pregnant and lactating women nutrition. What do you think the relevance of the multi-sectoral collaboration for improving pregnant and lactating women and adolescent girls’ nutrition?

P: I have already described the importance of the multi-sectoral collaboration. It is very important to collaborate in producing healthy citizens. Nutrition is very important for physical and mental development of the children and adolescents. It also helps to produce successor productive citizens as nation, region or woreda.

I: Good, so what are the roles of the other sectors in improving the women and adolescent nutrition?

P: The other sectors and stakeholders are responsibly working on women and child nutrition with full sense of ownership.

I: Are there any challenges or barriers for the multi-sectoral collaboration in pregnant and lactating women nutrition?

P: There is no big challenge for the multi-sectoral collaboration in this community. The degree of engagement may vary from sector to sector, but there is no significant challenge.

I: You have informed me before that your office is working on pregnant and lactating women nutrition in collaboration with different stakeholders. So, what things should be done to further improve the nutrition of the pregnant women, lactating women and adolescent girls?

P: One, we have to work on the adolescent nutrition. I told you before about our plan to work on adolescent nutrition and we are expected to work day and night to implement our plan in our hierarches.

I: Finally; I would like you to tell me anything that you feel is relevant for this study?

P: I have not anything left. Attention should be given for the pregnant women, lactating women and adolescent girls from the top to the bottom, because it is very important for developing physical and mental strength and to produce healthy citizens.

I: thank you very much for taking your precious time for this study.

P: thank you.

Summary:

- The message about balanced diet utilization is well promoted in the community and schools
- The community associates balanced diet consumption with the health and intelligence of the people
- It has been well promoted in schools and other clubs that pregnant mothers should eat balanced diet from conception to the time delivery
- There is good awareness about the importance of balanced diet during pregnancy
- In the past, important foods were only prepared and stored for their delivery. Even after delivery, the prepared food was not given for the delivered mother instead it was given for others who supported her during delivery as festive meal
- Awareness about adolescent girls’ nutrition is not created
- There is no any specific intervention on the adolescent girls’ nutrition
- There are some children who have been taking FAFA and Plumpy’Nut in this community, but the there are no any pregnant or lactating women who take either fafa or FAFA and Plumpy’Nut
- There is good integration between the women affairs, women league, women development army and health extension workers to disseminate information about women nutrition in the community
- Majority the people are using iodized salt, but at the same time they use the other salt (“gamfur chew”) for the preparation of spices, pepper and others
- Goiter was more common in selected areas like “HUGUMBRDA and SHIMBRKIT” due to the water they drink, but it is decreasing now due to the fact that the people are using iodized salt and they are drinking treated water from the spring.
- Some years before, the community was food insecure especially during October, but currently there is not food shortage in the stated community.
- The woreda level women affairs and stakeholders have included women’s nutrition in their plan. The plan is also cascaded to the kebele and women development army
- The safety net program and women education are successfully implemented for improving the pregnant women’s’ nutrition.
- The women union (women affairs, women league and women association) in collaboration with the women development army and health extension workers are actively working on pregnant and child nutrition.
- The women league is working on women and children nutrition in collaboration with some nongovernmental stakeholders like Grad and Action Aid are responsibly working on pregnant and child nutrition
- Nutritional counseling for pregnant and lactating women is successfully implemented in this community, but the nutritional counseling for adolescent girls is totally neglected.
- The education given for pregnant and lactating women about iodized salt utilization is successful, and the advice given for pregnant and lactating women about producing home gardening is successful.
- The women League are also integrately monitoring the implementation of the hygiene and sanitation packages using our structures from the top to the bottom; woreda women union, Kebelle women union, women development army, cell leadership (“wahyo amerarha”) and women league
- The adolescent girls are getting health education from the schools and they are implementing what they have learned in schools in their homes .
- Most of the kebeles of this woreda are highlands, in which mosquitos are not found
- There are no pregnant or lactating women supported by targeted supplementary feeding in this community.
- School feeding is not available in this community
- Repetitive health education is given to the girls to prevent themselves from health problems like HIV/AIDS
- Among the intervention, antenatal and postnatal follow up are very effective in this community
- There is no awareness problem. Almost everyone knows the importance of pregnant and lactating women nutrition
- The woreda health office have shortage of ambulance and some of the kebeles are difficult to reach during summer time due to mud roads and are far from the center
- Currently, they have good attitude towards the interventions
