# Supplementary material for: Barriers to utilize nutrition interventions among lactating women in rural communities of Tigray, northern Ethiopia: An exploratory study
Source: PLoS One. 2021 Apr 30;16(4):e0250696. doi: 10.1371/journal.pone.0250696 (PMC8087028; doi:10.1371/journal.pone.0250696)
Supplement: S2 File — (ZIP) [file pone.0250696.s002.zip › S2_File.Doc/Woreda level and above key informants/071_IDI_head of education office_Semere woreda.docx]

**Operational research on Adolescent and maternal nutrition in Northern Ethiopia**

**In-Depth interview with Woreda Education Office**

**Introduction**

Thank you for your consent to take part in this study and for taking the time to speak with me today. I have several questions to ask you that I have prepared in advance. If you have any additional questions or comments as we do the interview, please feel free to share them with me.

| **Section A: Interview details**   1. Zone: **South-Eastern** 2. Woreda: **Seharti Semere** 3. Kebelle: 4. Name of key informant: **Mr. Belay Molla** 5. Institution of key informant: **Seharti Semere Woreda Education office** 6. Interviewer name: **Abate Bekele** 7. Date of interview: **13/11/2017** 8. Interview start time: **09:15AM** 9. Interview end time: **11:17:35AM** |
| --- |
| Section B: Interviewee professional information   1. Gender    1. Female    2. **Male** 2. Age: **32 years** 3. Marital status: **Married** 4. Highest level of completed education.    1. College education    2. **Bachelor degree**    3. Master’s degree    4. PhD 5. Current position: **Curriculum implementation expert** |

**I:** Interviewer **P:** Participant

1. **Common maternal (pregnant women, lactating women and adolescent girls) nutrition problems in the community**

**I: In your opinion, what are the common nutrition problems in the community for women?**

**P:** Regarding nutritional problems in our community, the first is thinness, and having height which is not proportional to age i.e. being short so these are the common ones in our area. Then they are also vulnerable to disease, for example, those who have nutritional problem are affected by marasmus and kowashirkor, this is observable problem that we see when we go to field.

**I: In your opinion, what are the common nutrition problems in the community for adolescent girls?**

**P:** Yes, there are cases; particularly the so called stunting is observed in adolescents, very short those who have no proportional height to age especially in kola – low land – areas of our woreda, they are affected by it. Therefore, there is thinness and they don’t have proper body since they are affected by marasmus and kowashirkor.

**I: What about other nutritional problems like goiter, anemia and night blindness?**

**P:** There is anemia, for example, at school when I was teacher there were women and adolescents and still there are cases. But, for goiter there was a study conducted on it by Mekelle University once then they have taken sample, among the study participants 50% of students had goiter. Actually, it was not visible but by using samples 50% had goiter. And, in adult women there is also the case of goiter. But it is on women living in dega – high land –areas of the woreda the goiter is visible. It is big swelling at or around the neck so this is visible. But, regarding eye problem, I haven’t concerned as a problem in our set-up but there are cases particularly the elders, mainly above 60 years have eye problems and I guess there is nutrition related cause; otherwise I haven’t seen in students and children.

**I: What are the possible causes of nutritional problems you have mentioned so far?**

**P:** The cause for goiter is known, the salty portion of the land especially in highland areas when there is water flow, the salty soil on the upper surface goes eroded, and therefore the community consume non-salty foods, which results to goiter. The other, the thinness and stunting are due to the feeding. In our community there is problem in utilizing products of agriculture. For example, in this area there are milk, honey, and have income and everything else, but all the products have taken to market. Therefore, there is no trend/culture of using honey; there is no trend of using milk at home, there is no trend of using butter at home. So, the improper use these products is one of the factor for the occurrence of marasmus and kowashirkor. The use unbalanced diet, consuming same food items always, for example, our community is consuming is “Adengore” – in Amaharic, the “wot” of this crop is staple food in this area. They don’t use milk or butter therefore when we use only one food item, our body cannot bear what it intended to do so. Therefore, I have a stand to say these are causes of the nutritional problems.

I: How sever is the problem in women and adolescent girls?
P: In mothers, I haven’t seen it that much. But, there are cases in in school adolescents and out-school adolescents. The adolescents have no proper physical structure, there is thinness, and there is shortness. These problems are common in women particularly at primary schools it is highly visible. And, there are still cases at high school and TEVTS. For out-school girls who are daily labourer have no the right physical body structure. Therefore, I have intention that nutrition has caused such loss. It is also visible and in adult women though the percentage share is lower than that of adolescents, there are such diseases there could 2-3% mothers who are under-nourished.

**I: Is there a problem of overweight in this community?**

**P:** There are the cases in the urban but in rural setting we cannot watch women who have overweight rather they are thin or short. Most of our community is living in rural setting commonly they have thinness, but in this city there are also very huge women. That is why we are saying malnutrition as two tips, under and over. For example, in the city there are about 4 overweight women who are very huge. And, some of these are also unable to move. But, in rural areas you may not watch this problem.

**I: What are the causes for the overweightness’?**

**P:** Sometimes there is physical inactivity, for example, they consume food, then they stay sit and the food they consumed is again too much but no food utilized because already they are inactive. There is a woman I know, but she moves and has work/ merchant but I perceived as she has no proper feeding as she has good income that is why her feeding habit too much. As I said earlier, getting balanced diet has two things i.e. feeding lower than recommended causes thinness, whereas feeding more than recommended leads to over-weight. Therefore, these women in urban area have fed above the recommended level. Whether the women have made activity or not if she fed more than recommended she will be overweight. And these I said 4-5 women are very big and I belief they are overweight due to overeating and they are often inactive too. So, these are the causes.

**I: Why the community would like to sell their agricultural products rather than consuming them at home?**

**P:** This could be due to lack of awareness on nutrition. For example, by health and any other sectors, there is education about nutrition. But, that is not enough, for example, they may not consume if you tell them to consume, but how to utilize and eat foods should be focused. For example, I was giving training on nutrition for community, when I teach the value of egg when it sold and they consume at home, many mothers inspired. For example, two eggs can be consumed by two or more family members, but if they sell it could value only 5 birr, so what can be done by 5 birr. So, I told them why you don’t use them at home you have eggs, honey, milk at your home, and you can eat egg with bread for breakfast and you may feed other at lunch. Then they accept and understand it. But they already have a tradition of drinking alcohol and then they want money and the woman again sells what she have at home and buys’ coffee, for example, when we see not this woreda, in our country, there is a tradition of giving priority about coffee. For example, we sell what we at home and we will buy the coffee. Therefore, the calorie present in coffee and in egg is not clear for the community. Otherwise they only fascinated in coffee, they don’t have concern to use honey, milk or egg. Therefore the main issue should be awareness and then there is gap in practicing, simply we tamed therefore it needs much time to change. For example, we work on nutrition at school; there is program on nutrition so we have participated together with health sector; we educated them about the food items and their nutritional value with their cost. We prepared food from egg, milk, spinach and others, then the cost was estimated the cost and we have seen the cost is too small, but the value it has was very high, this was the way we thought the students. But, what I assume is, they may have problem of internalizing the education very well.

**I: Why these communities are not practicing though there are various awareness creation sessions?
P:** In our community, when I talk as an individual and as the woreda, we are good orators but we are not people of practice. I talk what is recommended but do I am practicing it in my home? No, for example, I am talking about what should be on the foods we consume, but if you come to my home there may not be such good food. So, the two things are not in-line, rather too different, and then it becomes a paradox. Therefore, when I say this, like if I drink alcohol at hotel or alcohol vendors, the farmer I have thought can say “what this person was saying? He told me not to drink but he is doing that” because they think alcohol has importance on what the teacher has thought them. Therefore, they may think why not I drink alcohol by selling what they have. This is my perception, and to avoid such actions, due to we are not practical so there could be problem in changing our community. So, we the teachers of nutrition and different experts should practice it first. As the community see us what we work always, they can see feeding only one item, so they may perceive as the teachers of nutrition is feeding only single item and they can assume to feed only single item. For example, we have gotten the community that assumes himself as better than the government employee in terms of feeding diversified foods. In our community, somewhere, a woman has prepared a various food items for us. Therefore this indicates how much she understood the importance of balanced diet. So, they can say we the farmers are better than the government employee. Therefore, the feeding habits of government employees such as for example at kebelle level, the HEW, agriculture experts, teachers are not in line with what they teach the community. What the community believes is the employees are better but if we fail to do what we teach them, so they assume the practice is not necessary. Always, we are calling awareness for community but we are not getting people who practice. Therefore, first we have to change ourselves. For example, when I was having training on nutrition in Wukiro city, the Dr. who was giving us training was asked us to set him daily menu for food for a week, then all participants planned to eat meat always, meat... meat …meat…. ; all the participants were assuming as getting adequate nutrition by eating meat always. Therefore, they planned to eat meat at breakfast, lunch and dinner plus some planned to drink beer in addition. So this is to say, we should be a role model for the community by practicing diversified food prepared from various items so that we can lead them to practice it. Telling the community about the stunting and wasting cannot be a guarantee to change them and their problem, rather there problem and practice can be changed if we actually do what we teach them like being a role model. For example, if I have over-nutrition and under-nutrition problem in my home, then if the community has such case, it is not their problem rather mines too. Meaning, the community is not guilty, rather I am guilty. Therefore, it is only practiced, if we are role models that the community can practice what we teach them. Therefore, all government employees and responsible workers of the community at kebelle level should become the role model first. And these workers in turn watch the higher level government worker like us at woreda level, therefore everybody at all levels should be a role model for the community level workers and the community. When we give training for them and if he comes to my home didn’t observe what I am teaching in my home, he then learns as it is only for talk therefore he then talks for the community and don’t expect the practice from the community. Then the community can only have knowledge but cannot practice. Therefore, we should be role model first. Otherwise it is still difficult to change our community.

**I: Is there any food insecurity problem in your Community?
P:** Yes there is food insecurity. The agriculture sector has a work division for food security. The mothers with food insecurity participate in community works and then they will get food support.

**I: How sever food security is in the community?**

**P:** It is too many, for example, if the rain fails to fall for a very few time, I am sure 80% will be affected. There are more than 100,000 populations in this woreda, though I don’t know the actual numbers who are in productive safety net program, I guess they are many. They mainly get supports like wheat and oil. The support is assumed to be given appropriately, but what actually done is different, for example, the administrative of kebelle uses such support in indirect way, they community always complains like he takes the support by his wife’s brother name or in another person. The area is not food secured, the community is known in begging for example when we see the high land areas actually it was not due to shortage but it could be learnt behaviour. The high land area community are called productive, ye indeed they are productive but after this they get wheat and oil as support by productive safety net program. They are many, though I don’t have the actual data; many people are supported by the safety net. They are those who said to be graduated, but they didn’t graduate actually as they are still in secured. Without they get secured, they simply graduate from the program. The criterion for graduation is the household can secure food by himself. Meaning each household believed to have adequate production and self-sufficient, then they will be discharged from the program. So, this community is not food secured and they are living with support from the safety net program.

**I: Why the community is food in-secured?**

**P:** The first assumption is the community has learnt the support by the government, majority perceive there is support by the government. For example, the support can be 20-40 cups of wheat, this is very minute cannot serve anything for the household but they learnt the support in fact the area is highly affected by insecurity as it is not productive, for example, the area is not suitable for production. For example, there is shortage of rain, it is low land area therefore lacks rain for several times. There is rain once per year however it is again not adequate as it stays for short duration. For example, this year others was a better rain but the half the woreda has no rain at all thus it is drought at the areas. Because the area is mainly exposed for such shortage of rain, there is food insecurity. Therefore, it is not suitable area for productivity. In high land areas, the area is densely populated, the farm land area is small but the population is densely populated. But, in the low land areas, the people are living very scattered and they are living in cluster fashion, here there could be 2 or more households, whereas there somewhere there could be 20 households and etc. therefore they can have very wide farm area and if they got rain, they can produce good production. But in highland area the land is too small, and the population is high therefore the production and the population level cannot be matched therefore they become food in-secured due to the imbalance. But, the main reason for food insecurity is the nature of the rain. There are no options like irrigations to replace for rain, because there is no water source in the area. There is no water for both cattle and humans there are cases that take about 1 hour to fetch water, it is very far. Therefore, due to this reason the food insecurity is prevalent.

**I: Why do you think women are especially at risk of malnutrition you have mentioned above?**

**P:** The first is; there is male’s superiority in a sense females cannot run money as they want. The males have total control on the money and the female has no role in any decisions. For example, if they have sheep of goat when the male sells them he doesn’t consider something important for his wife, for example he may produce sorghum, Teff or wheat then he assumes as his wife to eat either of the crops in whatever ways for consumption, but doesn’t considers what his child, and wife has eaten. In this community, you can watch the farmer come to city and drink alcohol, and waste his money but doesn’t say what to buy for his wife and he takes nothing for his wife from the city. Therefore, the males don’t think as their children and wives shouldn’t be fussed by food. However, the women loss many things in her life time like when she get pregnant and give birth then again she fed her child, so she loss much energy then she need much food, but the males/husband don’t provide such appropriate food. Therefore, this is the number one reason why women are at risk of malnutrition in the community. Moreover, there was no focus on maternal and adolescent girl’s nutrition so far even from a national level, but there are some movements recently. So far, regarding nutrition it has been targeted at children. Therefore, recently there are sensitization and mobilizations of community to act on maternal nutrition, but yet it is not implemented. The women should own her production, for example, if the household has cattle, they should make decision together to sell or to do something, they should agree each other and after he sell them, both should decide together on its use. For me, this has not been done in both legal ways/law and in creating awareness very well. We talk much on gender equality but that is not happening at ground level, i.e. she has been kicked at home yet. For example, I have wife, but when I took my salary then it is given for her when she ask me about 200-300 birr, but I don’t give her like I don’t say “I have gotten this amount of money so you can have this”. So, the problem starts from me the educated one. Therefore the literate has also such problem. This affected the women. Therefore, we all should have a perception that a woman should get much food than males.

1. **Nutrition priorities in the woreda**

**I: What priorities do your institution has in relation to maternal and adolescent health?**

**P:** As school sector, we are working towards the females as they can perform as males. The students well perceived the gender equality, and the teacher again well perceived the gender equality. Regarding their health, we are working with health sector, currently schools have garden, to use the products from the garden for children and mothers. We have started at one school, then that kebele become model. Women also have gardening and they are using the products as well as selling it. So, this achievement is after we started the work at school with health professionals. Moreover, for at schools for girls, we have prepared a separate room to girls to have menstrual hygiene. As I said, majority of students are rural, they fear, so that separate room is prepared but it is not done at all schools rather it is implemented in few schools. There is a plan for each student with expected date on menstruation. This is done by the team of student called the health team. There are two health clubs at schools the first on is the health club working on menstrual hygiene among many other health issue and the second club is nutrition club. So, the health club have savings to buy menstrual pads and support each other in health issues. Mekelle University again supported the supplies like modes/menstrual pads, and pants for some schools. But in other schools the schools themselves buy the supplies by their own budget. They know the expected date of menstruation and supplies will be requested as the number of girls that can have menstruation at a time. Otherwise the girls may be challenged, for example, recently there was a girl that left the school due to menstruation even she has gone through the fence. We have asked the school but the school informed as they have prepared separate room and other facility for the girls, and said she might not have awareness about it. So, we have such activity in schools but it not in all schools. For example we have 60 schools, 35 are 1-8 (primary) school. And may not be 10 schools in which such activity has started but about 3 high schools have started and we planned to expand it to all schools. Regarding the health of women, we don’t have work done that I can tell you, but we work with health at schools with health sector for reducing the maternal and child mortality. The students have the data about pregnant and children. For example, at kebelle level the students know the number of pregnant women. Then they link them with the teacher who is chair of the club to link them with health professionals. Moreover, there is nutrition day at schools, on that day we call the mothers and demonstrate balanced diet preparation together with health professionals.

**I: What nutrition interventions have the most resources allocated to them?
P:** We don’t have budget, we are serving free. We are doing without budget. As education sector, for example, the schools have clubs but we don’t allocate budget for them. The school itself covers the budget. For example, for menstrual hygiene pads and pants, the school allocates the budget but we the woreda office allocate general budget as the school grants to schools. Otherwise, we don’t give on job training for women and adolescent girls. As woreda level, there is no budget as there is scarcity, for example we request budget always for nutrition and life skill training. Otherwise, NGOs such as the Relief society of Tigray (REST) and world vision are sometimes supporting us.

**I: Do you think it is necessary for your institution to get involved in work aimed at improving maternal nutrition?**

**P:** It is very important because our institution we have large number of staffs, we have more than 30,000 students, more than 1000 teachers, again there many supportive staffs, so we have around 2000 staffs in education sector only. And we have 38,000 students and around 30,000 are adolescents as the majorities are from the rural setting and have delayed school start. For example, when I participated in activities and trainings I will give it to the director, my colleagues/ teachers, and then for students and then there flow of experiences through chain to the community. If the experience reaches the students which means it simply reached the community. Generally, it is to mean it is important to get involved in activities related with nutrition.

**I: How do you evaluate the priority given for the interventions for women?**

**P:** We have planned to work on nutrition as priority and we are working on it because if there is good nutrition, there will be healthy community therefore this is why we are currently prioritized to work on nutrition. Currently, we are choosing additional potential areas to implement it to be efficient. If there is nutrition, there couldn’t be children death, meaning the death is natural process but death due to nutritional problems will be reduced. Therefore, we are more concerned at working on this. We are working to create healthy students and the students in turn will work on making their family healthy. So, nutrition is a base for every work and success.

1. **Nutrition interventions that improve adolescent and maternal health**

**I: What kinds of nutrition interventions are in place to improve adolescent and maternal health in this woreda?**

**P:** In this regard, due to our profession, we may not know specific activities because we are not that much familiar with health activity. For pregnant what we know is follow up, the women go to health facility every time. Then, they have to be checked about nutritional status. A woman should be assessed on the gestational age, counseled on type of food that should be fed by pregnant, and advised on feeding style. These all services do exist in our setting and it is provided for women. I assume the follow-up is the main activity for pregnant women. But, such activities may be difficult to work at schools.

**I: How the access to these services?**

**P:** There couldn’t be problem of access. For example, for access the main challenge is awareness. The main challenge for mother to access the health facility is the geography. It is full of chains of mountains and the pregnant woman can take 2 hours to access care on her kebelle health post. Therefore, this is difficult for pregnant to follow clinical check-ups. But there is outreach service by health professionals to check the pregnant at her home. So there is access to health posts problem. And, the number of HEW is small (they are only two for 7000-8000population), therefore it is difficult for them to administer the service.

**I: What kinds of other nutrition interventions are in place to improve adolescent and maternal health in this woreda?**

**P:** They are advised on taking extra meal during pregnancy and lactation, and iodized salt. They are activities that should be given and the health professionals are working on these rather than sophisticated treatment procedures. Their main work is also hygiene and sanitation, the food the women are eating are clean, the cleanness of the food, and the cooking of the food are among many other tasks of the health professionals. Nutrition is also the main task of them particularly on maternal and child nutrition.

**I: Is there advice on nutrition sensitive agriculture for women?**

**P:** Yes, but it is difficult on implementation, for example, we are just making campaign, rather, for example, the FTC should be the model of excellence. But, when we see the FTCs, the center has no plants, has no cultivated improved crops to demonstrate for them, but the agriculture and other experts talk about improved seed, deforestation, but the center that should be exemplary is just empty or has nothing that someone can learn so why we are forcing the community? Research means doing something new and showing how good is something then the farmers can learn and want to do by themselves - “they can say why we don’t do”. Otherwise they think as they know more than the educated ones about the agriculture, then if you advise them to take fertilizer or improved seed or whatever you say they don’t practice it. Likewise, for women rather than talking about nutrition for days, demonstrating and cultivating the crops at garden around the health post is enough. If they demonstrate gardening and preparing food from various crops at health post or facility, the 3-4 women who have come for the service can use the experience and use it very effectively. So, we don’t bother and waste our effort unnecessarily**.**

**I: What is the problem to gardening at health posts?
P:**

The main problem is said to be human power, for example, in a kebelle there are two HEWs but they serve 7000-8000 population. On the other hand, there was access problem to pregnant women villages from their health post as it may take about 2 hours, therefore, the spend their time at journey and may not get time to work on gardening. This is the problem but it is simple, there could be guard and he could keep the gardening or takes care of it. Therefore, rather than teaching about nutrition, demonstrating the practice about nutrition is better.

**I: Do the women are receiving targeted supplementary feeding?**

**P:** Yes they receive it; there is women association for preparing blended flour, and they are providing for women with low cost, and the women are consuming it as porridge. This intervention lasted long since the health sector started it. The women have understood the importance and are utilizing it. So the women association is selling the flour, but such flour may not be provided by government and cannot be found packed at health facility. Primarily, they have learnt and then they are producing and using it as source of income. It had been used for pregnant woman when she comes to give birth then the government supplied it to woman at health center. The flour has every nutrient such carbohydrate, protein and others. For malnourished there is also called plumpy-net given at health facility. I think there is also blended flour/targeted supplementary foods. But I don’t think women are receiving such flour currently. Likewise, mainly the plumpy-net is given for children, but I haven’t seen when it was given for other age groups like adolescents. Even sometimes I am hearing as it is forbidden for other age groups. But now I felt as it should be given because once whoever gets affected by under-nutrition should replace his deficiency.

**I: Is there youth friendly service for adolescent girls?**

**P:** I think this is a peer-peer service; this is present at schools and health centers. I have been working on education system for long time, we are using this initiative in life skill training – a training given to prevent transmission of HIV, there is peer pressure that promotes the transmission of sexual transmitted diseases. Therefore, it is provided through game by making them fun, but show how HIV would be disseminated, for example if there are 50 trainees, you show them how these 50 get infected by HIV once. Adult women such as commercial sex workers (CSWs) also receive such training and they have good awareness. When we were giving training for youth and adult commercial sex workers, the CSWs feel as they are better than youth to preventing HIV as they are always taking the preventive measures. They were also telling for energy replacement as during sex they were losing much energy. So, they are talking about the importance of nutrition. But they feel as youth are committing unsafe sex.

**I: Which of the above listed interventions do you think is most important for pregnant women?**

**P:** For me, the number one important is nutrition, because when mothers feed it indirectly reached to children, and then it goes to the whole nation, for example, a woman has fed food appropriately means she is the one who provide food for her kid and the community. Always if women are well-nourished, they provide better nutrition for males and others. So, the nutrition is the basic and otherwise it doesn’t mean other services are not necessary for them rather nutrition is basic to all services.

1. **Implementation challenges and community factors affecting access to maternal nutrition interventions**

**I: In your opinion, which of the above programs are being implemented successfully (i.e. in the most effective way?)**

**P:** Now, I have said nutrition is important but its implementation is very weak. Though the awareness creation among mothers and adolescents are good, and we are doing in better ways across the health, agriculture and education sectors, the main work done so far is awareness creation. Therefore, if you ask somebody about nutrition and clinical follow ups for women and adolescent girls, he/she can talk much but in practice it is too child. For example, maternal mortality reduction has been implemented fully; the women who give birth at home are very low, it is very rare. If there area is very far, the family members are in case at field during initiation of labor then a woman may practice home birth otherwise she delivers at health facility. But the main thing that a delivered mother should get better nutrition, such understanding is present in urban setting, what makes it better; this is to mean the husband slaught sheep, as they consider this replace the blood or energy lost during delivery, but actually the only meat cannot have such input. But the husband says “I slaughted sheep”, so there is misunderstanding. There is assumption that nutrition is fulfilled if they buy specific food item like only egg, only meat, only honey or butter. For example, how the butter only can be eaten. Therefore, we are more working in creating the awareness but the only awareness creation cannot change therefore we should go to implementation. So far, we are too early in the implementation. But, the successfully implemented activity is maternal follow-ups like clinic visits because most of the women have check-ups.

**I: What are challenges to implement nutrition interventions?**

**P:** The first factor that influences its implementation is culture, and religion. Still, there is a culture state that females shouldn’t eat good food rather good and good foods should be for males. Hum … [laughing] to your surprise, I got married few times ago, my wife was educated but I faced incredible thing. The “doro wot”- the chicken - was cooked on a day before I go to field then the chicken was not eaten until my return. She was saying “My husband was not around, so how can I eat alone”. I was ashamed because I know she is literate and expecting she knows bad and goods. Then, when I guess her mother is even a teacher and well educated, I don’t where such behaviour has come. But later, I understood that it is familial influence. She knows but when I guess and asked here the reason, she told me as she is waiting me to eat. I understood, does she think me as I have the support for such culture. She was thinking as; I will ask her why she ate the food in the absence of husband. Then, we discussed and then after not only field when I stay in my office about 30 minutes in lunch time, she eat her lunch alone. So, there is this culture in the community which is rooted in my home. Therefore, this is a dip rooted culture that may not be easily halted. For example, in rural areas, the children are not eating food, there is a culture that says: unless the father prays and present at food time, it is unlikely for other households to eat food. This culture has both traditional and religious base. For example, fathers’ prayer for food is religious, and waiting for husband to eat is tradition. They assume father as a big pillar of the household. So, these are the big cultures in this community.

**I: How aware are the women and girls on the need to get interventions?**

**P:** Now, women are aware of the interventions and they are motivated on the implementation of interventions. They know as nutrition should be focused on them, for example, when I give life skill training, we have discussed about slaughting the cattle, we were arguing on males or females or both can be slaughter, I was asking why not both can be slaughter, we were discussed from religious base but agreed as both can slaught because nothing is written in Holly Bible and Quran. There was a woman who said “if woman reach the level that males reached, she can slaught”. So, this is again another cultural practice. As I said, controlling wealth is given for husbands. However, now there is promising progress in creating awareness on females on owning of their properties. Currently, there is some improvement.

**I: Is there a relationship between educational status of women and access to interventions?**

**P:** In my opinion, they are the same in accessing the interventions. For example, in rural areas there are outstanding women. They practice gender equality in a good way. They have good administrative ability. But in urban setting, they are better at meetings but have nothing in practicing. They simply know and talk for symbol, for example, in household where the couples are government employee, when they return from work, the husband goes to bed while his wife goes to cook but she don’t call him to support her. I have a staff lady who beliefs women are equal with males. But when I ask her about her husband, she told me as she didn’t try yet to call him for works at home. Talking outside is nonsense rather should be stated at inside the home. There are many talks outside home, but there various works left to be done. Somebody may say urban setting is better but actually they are not.

**I: What community related beliefs and norms are preventing access to interventions?**

**P:** Yea, I have already said, there are beliefs that make/push women to alcove.

**I: Are the interventions acceptable culturally?**

**P:** Yes they very acceptable, for example, in religion the Holly Bible and Quran states, woman is crown, i.e. she deserve care. The problem was the past tradition which advocates the males’ superiority. Otherwise, it is not the religion that forced people to do such culture. For example, when boy is born there is seven times “Elilita” – in Amharic to represent celebration by women but it is three times for female birth, however this is not written in Holy Bible or anywhere else. The gender equality begins here but they cannot advocate gender equality because they believed in the superiority of males i.e. superiority complex.

**I: Are the interventions accessible for women and adolescents?**

**P:** Nutrition is for all though we are working for women and children most of the time. But, no nutrition interventions focused/ concerned adolescent girls. For example, those who aren’t married, adolescents, and other groups need nutrition too. I don’t know where the nutrition program made to focus only on specific groups, I think at ground level it focused on pregnant and children and training has been given for pregnant and child nutrition. The adolescents are neglected. I think this happened incidentally. In practice again, this training is only focused for mothers and children rather than concerning adolescents too.

**I: What about cost and transportation to access the interventions?**

**P:** Currently, we are intervening in kebelles in which the community can access them easily. The interventions are done at their kebelles. We preferred the place which is easy to access and then it is also a center/an average place for all.

**I: What about the quality of the interventions?**

**P:** This could difficult, we have started the intervention as a sample, but the quality should be assessed at education sector level only, the school don’t know about the quality of crops and their handling very well, but the agriculture expert and the health experts should do that, but this has not been yet done we simply started at school, therefore the quality may be poor.

**I: What resources exist to provide interventions?**

**P:** The water accessibility is very challenging, though we want to expand the interventions. Majority of our schools have no water supply. We were planning to make our schools center of excellence in nutrition. The second is shortage of the improved seeds of spinach and others, otherwise we have adequate manpower, for example, students bring water for gardening.

**I: How you planned to solve water supply problem?**

**P:** We are working with water and energy office, but they need much budget. Therefore, budget is the main problem to construct. So far, the water point has been installed by one wash and kowash projects. No school has water installed by woreda’ budget, the schools currently have water were either they had water initially or installed by NGOs. We are trying our best, we have bought roto/tanker for schools, and we distributed 30 tankers for schools to collect water.

**I: How do you evaluate the commitment of the interventions providers in the kebelle?**

**P:** For example, when we intervene at a school, it expanded to kebelles. So, currently, the households are made to have gardening, the kebelle was taken as model in nutrition, started from school it diffused to kebelle, there is NGO called world vision we worked together and provided seeds from school to kebelle. Therefore, there is a culture of participation at school. There are the health professionals, and the community mothers work together at school. Therefore, their commitment is good.

**I: What solutions that your institution has applied to effectively implement the interventions for women?**

**P:** I have told you about water, and the problem of seeds is already we asked REST as they have project on nutrition they provide seed. They have a program called L-10k (last 10 kilometre) to work, meaning they work on within 10 km radius from the health facility. They have supported as seeds.

**I: What do you think needs to be done to better address the challenges you have mentioned like budget?**

**P:** This is difficult; working in collaboration with stakeholders is a key to solve the problems. We can prepare roof catchment using water tankers, to collect water when rain comes. Working together with agriculture, REST, and water can solve as they can solve water accessibility problem. In our side, we can support by our students by providing them separate crop production areas for different grade levels to cultivate crops. For example, you can give some meters of land for grade 5, so they can provide water for the crops, so this is how to solve the water shortage problem simply. But, the issue of budget may not be solved at this time so our option is to use our students. Therefore, if we work like this, we currently are selecting schools that can be appropriate for gardening so as to use it as model to disseminate the experience to other kebelles.

1. **Multi-sectorial collaboration to improve maternal nutrition**

**I: Do you feel it is necessary for your institution to work with other sectors/institutions to address adolescent nutrition?**

**P:** Yes, from sectors it is better to work with youth and women affairs office, because these institutions have budget for youth and women works, they have allocated budget therefore from that budget if we work on nutrition it would be better. Not only these, there is sport office and other’s like NGOs, for example, world vision works with youth therefore if we work with such institutions I hope we can do better.

**I: How do you evaluate the level of collaboration among sectors in nutritional interventions?**

**P:** Most of the time we are working in collaboration with other sectors for other programs. But, we didn’t yet start to work together with others on maternal and adolescent nutrition. However, in other activities, for example, we have work that we work with health, with women affairs, with social affairs, with agriculture and with land desk and area development. For example, due to our work with land desk we work development woks like schools be made ready for production because they help us by training while we provide human resource and produce crops. They work on identifying land suitable for production and water supply. We are complementing each other. For example, if the health capacitates us, for example, if every education graduate has skill of attending birth, I don’t know whether the Mekelle University has started or not, it is planned to provide sexual and reproductive education for all teaching graduates. It is a course aimed at enable teachers to attend delivery through home visit. The curriculum has been made ready to start at all universities and I think Mekelle University has started it. Therefore, if the SRH has introduced to teachers, then the teachers easily deliver it to the community because teachers have close relationship with community. Because as I have mentioned above, there are only 2 HEWs at kebelle, s they cannot address the whole population the kebelle, but there are more than 10-30 teachers per kebelle, therefore they can support them. Whenever there is health problem they can support. So if there are such capacitation’s for our students as well as for teachers, we would be effective. For example, if we work in this way, why not we address the nutrition of adolescent girls too? Therefore, risk of vulnerability for nutrition related problems will be reduced for adolescents. The females are vulnerable for nutrition related problems, and then they cannot be productive in whatever position you invited them. It is also not good for biological process like in her offspring’s; a girl can be challenged for giving birth. Therefore, if there is no reduction in vulnerability of these girls, we could face challenge in productivity.

**I: For multi-sectorial action that effectively works to improve maternal nutrition at all levels, what kind of change in terms of the way stakeholders work together is needed?**

**P:** Our collaboration shouldn’t be only paper based rather it should go beyond. For example, we have several interface and intra-face agreements so far, but when we look agreement some of them were not correctly created. I only put my organization work there but totally missed to what other should be supported by me/my organization. Therefore, we have put such agreement and then we discuss then we will implement it. Therefore, it should be exercised well. Therefore, our collaboration should be towards implementation/in practice. Collaboration by itself doesn’t mean the work has done. For example, we have understood, and taken it written on paper, but when somebody comes from region and ask about collaboration we say just we have made interface/agreement, so the supervisor has only interface on his supervision checklist, but this should be done. Otherwise, the checklist should go beyond yes or no response and should evaluate the work of each sector like what has been done by health sector? And it should also show a place for where they can observe what has been done. So this can show the correctness of the collaboration. Otherwise, it shouldn’t be only on talks rather we would go through in practice, thus our collaboration will be productive till the ground-level.

**I: Why such evaluations done?**

**P:** The first, when I go to lower levels, I am to ask whether the school has plan but my checklist don’t allow me to check and observe what has done. Even it can say check and observe, for example, I may not have good knowledge in planning activities therefore I focus in points where I am familiar. So we may have knowledge gap, therefore when we supervise we should go together with experts of each sector. Currently, a good work has started we go together with health, administrative, and others. They go together to kebelle and supervise for example, if it is said that FTCs has made productive, for FTC the education expert may not know details, but due to the presence of the agricultural expert he can observe the FTC by various parameters. This has to be practiced. It helps as to take action on those who didn’t achieve. Otherwise, if it is only on paper, the region may come and talk then those orators can talk very well and but shouldn’t be like that. Because we have to make a prospective nation, for example, if somebody may die this might be nothing for me but it is really hurting the community at lower level. The farmer works there hard and his wife again works hard and then she may not get nutrition compensation for the energy she lost therefore it should be done on nutrition even by considering the amount of energy each food item can produce again be better explained to them. For example, Adungore wot (bean stew) is common in this area, but if she eat it always she may not be protected from disease. In this community, a farmer stays a whole day by a single enjera with adangore wot, but he may need 2000kcal/day but he has eaten 500kcal, therefore we should tell them how it is nutritionally.

**I: What type of resistance to the needed change do you perceive or have you experienced so far?**

**P:** You are leading a sector then to work on nutrition, for example, I am free today, tomorrow I may not be free while the health is free, on the next day the agriculture can also say I have another commitment. Therefore, the collaboration work has a problem such as lack of the same plan for all sectors, and the time may not be fixed together to monitor and evaluate what has been implemented at this woreda. Therefore, for example, we have meeting before you come to our office here, today my program was not here to stay with you, I have planned to support one school I was ready to go. Unfortunately, I was called her by my boss, we have discussed and done some activity, thus some occasional assignments from the higher level though it is important but it may not be a time to be engaged on it. Today I missed my program, therefore this will be postponed to next week then again in that week I may face another occasional assignment. Therefore this is a bottle neck for the collaboration because we are not meeting; while we miss our program can be postponed to years then her it comes to lie or to give false information. Like by simply my colleague from health sector goes to community and bring me some information about activities done which are not detailed and may not be right enough, then you can simply guess the things snap shots. But if you get a good supervisor, you may not be passed simply by lie, he may dig and ask many points then you are known as not done.

**I: So, how can these can be solved?**

**P:** It is simple; there is time when various sectors stay free of work. Then by considering such time you can arrange your supervision time. The supervision may take 1-2 weeks and then the rest is compiling report so it can be done overnight. Therefore you should arrange schedule timely and inform all sectors prior to the deadline because without program you cannot go haphazardly. Therefore we should have schedule and the schedule should be communicated to woreda administrative, so, let say we are four sectors we should inform the administrative what to work prospectively, then my boss and administrative know me as I have schedule or program to conduct supervision therefore they may order me to go to my work even though there could be meeting at woreda level and I couldn’t be blamed because I have gone to work what I have planned primarily. Therefore it is simply cancelling training calls. At training you may get 500-1000 birr, you can work invaluable thing if you go to the community. So if we work in this way, it would be better.

**I: Do you have joint plan with collaborators?**

**P:** No, this is what I am saying so far. There is simply an interface, otherwise no common plan you simply call others if you want to go for supervision. We have started to supervise in team last week, my friend was to go to it but due to meeting calls from our office it was cancelled. The meeting was said the administrative had been with us and said to be finished with 10 minutes but we stayed more than 5 hours. So after 5 hours you can go for supervision as the weather is too sunny and it takes 2 hours to reach the community. Otherwise, we plan to go together by ourselves but we have no good chair to organize just to notify and manage the team. In NGOs it is good to work together, but it is difficult to work together in government intuitions, for example to work collaboratively, the regional education bureau may call us for meeting while we have another pre-planned activity in our office though we have sent our plan to region at the beginning of the fiscal year because they may not see our plan. For example, there is supervision season, this time is known at regional level, but they call you for meeting thus you cancelled your supervision and then you go to meeting and immediately the come to supervise us and ask the supervisions done by me and they question “why you didn’t do supervision?” Therefore this issue should have some norm like there should be a good organizer and chair for the activities, hence we bring change.

**I: To what extent does your institution participate in the multi-sectorial nutrition coordinating body at the woreda level?**

**P:** It is limited somehow because there high workloads so that I missed the involvement for a while. But I don’t say there is consistent involvement in the implementation.

**I: How effective are the coordinating platforms in enhancing multi-sectorial coordination?**

**P:** It is not effective because it is only established but not yet started to implement. At the end of last year, it is newly initiated from higher levels and this year only once we made supervision. So, hereafter it may start to work strongly but so far nothing has done.

**I: What opportunities do exist to promote multi-sectorial collaboration of nutrition in this woreda?**

**P:** it is difficult to say; the opportunity… [Thinking] sometimes there is time to go to field together, in that opportunity you can observe the whole works done at community, for example, if health, education, agriculture and women affairs experts go together even for another duty like you may go to convince the farmers to use fertilizer or irrigation though it is not present currently, this thing was done at night when the farmers come back from their work, but on days you may stay asleep but we can do supervision of other activities then after at night you can teach the current issue. So, we have such opportunity. Since the community is engaged at different activities you may not access them easily and therefore the sessions should be arranged at night and during day time we should go to works that don’t want community discussion like institutions. As education sector, supervision is started at August, because the class ends in June and July is a break and August is a preparation phase to New Year then after the monitoring and evaluation phase starting from November and it ends at mid-December because you have made supervision in November and provided feedback to take some actions then class ends at January and at the beginning of the second semester in February we have one supervision to check whether the feedback given in November was corrected or not to see the change in health, students, and awareness on nutrition are checked. The cycle goes in this way. For example, we have quarterly supervision plan and others do like ours.

1. **Other interventions that influence adolescent and maternal nutrition and health outcomes**

**I: In your opinion, do think delayed marriage (after 18 years) improves maternal nutrition?**

**P:** It is also supported biologically because the maturation is attained after 18 years. For example, if a 14 or 16 or 15 year old girl has married, i.e. 14year at marriage means she give birth at her 15^th^ year and 15 year marriage means he give birth at 16^th^ year. That means she is not matured again mentally, and while she give birth it means “a baby is to care a baby”. She is a child and again she has a newborn; therefore she treats two things therefore she will be hurt psychologically because not only she lacks knowledge of the importance of nutrition but also she is still child that need the want the one who cares her. The age of adolescence is a period for confectionery consumption of her childhoodness. As it needs maturity, it affects her nutrition negatively. Primarily she needs care and she doesn’t know how to care her child and what he wants. And her uterus is also not ready to bear a child; the food she eats might not be enough as her child shares it. But those who are older than 18 years are matured and well developed body structures even our bones become compact after 18 years meaning they become strong. But a girl who is lea than 18 years cannot bear the weight as her hip joint even weak. Let us take, a child born at 18 and at 24 years, the child of 24 years mother is mentally alert than that of 18^th^. The hip widens even after the age of menarche, and it affects the birth. So the maturity affects nutrition.

**I: In your opinion, do think increasing the space between each birth improves maternal nutrition?**

**P:** Yes, for example, when a mother gives birth yearly she becomes weak, nutrition can be innate and acquired, for example, we may have some protein level in our body, so this woman spend much of her time at caring her kids if she has frequent births, her mind is on only her kids.

**I: What programs or activities promote increased birth intervals in this Woreda?**

**P:** The first is health education to space birth. There is information dissemination through posters and banners at schools. So the cons and pros are visible there.

**I: Can you tell me about any programs or policies in place in this woreda to prevent early marriage?**

**P:** Early marriage has stopped. Currently, there is no such practice completely. Because we have development armies, the women affairs are also here, and at school there is also child right/affairs club to protect children. This club critically follows the girls to prevent early marriage if there is a rumour they immediately notify to the kebelle administrative then the administrative writes to development army, so we have such chained and organized work. The students are completely understood the issue and notify for their teacher or school director and then he follows her until her parents. So the female students are highly capacitated and they call for help, rather than taking the marriage order of their parents that was in the past times. Therefore there are punishments until detaining of her family because there is legal concern.

**I: Can you tell me about any religious issues in this woreda to prevent early marriage?**

**P:** Currently, religious leaders are working as they are influential. These people are taking training many times as they are influential and are best contributors to stop early marriage.

**I: In your opinion, are these programs or policies effective?**

**P:** Yes, it is about two years since we heard about early marriage. Though I cannot say it is completely eliminated, sometimes there is a system to conduct marriage secretly by avoiding the wedding ceremony because if it is known, they will be accused/ due to the fear of law. There are people in jail.

**I: What are the community factors that promote early marriage?**

**P:** Yes, there are community factors among which culture is the one. They know as they (her mother or father) might be married at 10 or 12 years of age. Therefore, they assume as their daughter is an adult while she is at 14 years and assume she might be unmannered if she is not get married at this age. The other issue is her husband to be can force her to have marriage, as he could be old enough like 30 years, so he pushes her to get married. The next is her parents might be economically good/ rich, so this guy wants her to be economically empowered.

**I: Can you think of any other opportunities to prevent early marriage and increase birth interval?**

**P:** We have strong development army. These development groups are very strong as they highly against the early marriage and frequent birth even they don’t excuse for their family too and they seriously follow the situation. At school, there are clubs, school director and student who are working always actively. Most of the time November and December are the major seasons for early marriage so the clubs, school director and student are actively search to prevent child marriage. So then they all work on identifying cases that are ready for January at the end of the first semester as most of the early marriage happens during this month to make it secret from students and teachers. Al teams including the health professionals and the women development army are active at this time. The health sector proves whether the girl’s age is above 18 years or not and the religious leaders also help us in estimating the age of the girl by using the religious events done for her.

**I: What opportunities do exist to promote maternal (pregnant, lactating and adolescent girls) nutrition in this woreda?**

**P:** We have nutrition club at school, and we are using holidays to teach our community. Whenever there is holiday there are always community discussions then at that moment the health extension workers and education sector representatives go the community and teach them. Las year, we had discussion at school with women of the kebelle at nutrition day, and we provided education to completely eliminate the harmful traditional practices.

**I: Why the FTCs are not the demonstration sites for the community?**

**P:** Leader is influential. Surprisingly, the government has given a good opportunity for agricultural extension workers just to cultivate the crops in the FTCs and use the half of the product for themselves without any cost by cultivating the improved seed there as they are conducting an experimental study. For example, the farmers are doing in their farm and the experts are cultivating at their FTC then they can demonstrate how to be more productive by using fertilizers. So the farmers can see the difference in crop production using fertilizer and without fertilizer therefore the work itself teaches the farmers rather than having additional educational session. But the experts are not doing like this because there is no push over them from the higher level supervisors, and I also doubt whether these people are aware of the aim of this intervention. Every year we are looking the same thing at the FTCs, no change has made. For example, schools are better, the school is green and have gardening but the FTCs not. The reason is complicated and cannot be easily understood so it may need further exploration.

**I: Is there school feeding program?**

**P:** Yes there was school feeding program three years back during El-Niño as there was no rain. But it was for one year since the rain has come then after.

**I: What lessons have you learnt regarding multi-sectorial coordination of nutrition in this woreda?**

**P:** As to me, on nutrition rather than knowing it theoretically, I have seen it practically. I understood as the nutrition has an impact in productivity of our nation. I understood nutrition is very essential. Even I evaluated myself as I was eating food haphazardly but I didn’t have know-how about nutrition rather we are simply engulfing and filling our stomach. I felt if all sectors work collaboratively we will be highly successful because it enables us to fill individuals’ gap as my knowledge fills the knowledge gap of me and vice versa. It also increases creativity.

**I: Thank you very much for you time and detailed explanations!**

**Summary**

1. **Common maternal (pregnant women, lactating women and adolescent girls) nutrition problems in the community**

- The first is thinness, and having height which is not proportional to age.
- Because we are only good orators but we are not people of practice.
- This community is not food secured and they are living with support from the safety net program.
- The main reason for food insecurity is the nature of the rain and lack of options to get water.
- There is no water for both cattle and humans there are cases that take about 1 hour to fetch water, it is very far. Therefore, due to this reason the food insecurity is prevalent.

1. **Nutrition priorities in the woreda**

- Regarding their health, we are working with health sector, currently schools have garden, to use the products from the garden for children and mothers.
- If there is good nutrition, there will be healthy community therefore this is why we are currently prioritized to work on nutrition.

1. **Nutrition interventions that improve adolescent and maternal health**

- I assume the follow-up is the main activity for pregnant women. But, such activities may be difficult to work at schools.
- The main challenge for mother to access the health facility is the geography.
- The area is full of chains of mountains and the pregnant woman can take 2 hours to access care on her kebelle health post.

1. **Implementation challenges and community factors affecting access to maternal nutrition interventions**

- Maternal mortality reduction has been implemented fully; the women who give birth at home are very low, it is very rare.
- So far, we are too early in the implementation of nutrition.
- For example, in rural areas, the children are not eating food, there is a culture that says: unless the father prays and present at food time, it is unlikely for other households to eat food.
- This culture has both traditional and religious base.
- For example, fathers’ prayer for food is religious, and waiting for husband to eat is tradition.
- They assume father as a big pillar of the household therefore they cannot eat food if the father is not around.

1. **Multi-sectorial collaboration to improve maternal nutrition**

- It is better to work collaboratively.
- Most of the time, we are working in collaboration with other sectors for other programs.
- But, we didn’t yet start to work together with others on maternal and adolescent nutrition.

1. **Other interventions that influence adolescent and maternal nutrition and health outcomes**

- Delayed marriage and increasing birth interval are important for women nutrition.
- It is about two years since we heard about early marriage.
- I cannot say early marriage is completely eliminated,
- Sometimes there is a system to conduct marriage secretly by avoiding the wedding ceremony because if it is known they will be accused/ due to the fear of law.
- There are cultural factors that promote early marriage.
