# Supplementary material for: Barriers to utilize nutrition interventions among lactating women in rural communities of Tigray, northern Ethiopia: An exploratory study
Source: PLoS One. 2021 Apr 30;16(4):e0250696. doi: 10.1371/journal.pone.0250696 (PMC8087028; doi:10.1371/journal.pone.0250696)
Supplement: S2 File — (ZIP) [file pone.0250696.s002.zip › S2_File.Doc/Woreda level and above key informants/072_IDI_MCH expert_woreda health office_Samre woreda.docx]

**Operational Research on Adolescent and Maternal Nutrition in Northern Ethiopia**

## Key informant interview with Woreda level MCH expert

**Introduction**

## Hello, my name is Amaha Kahsay. I am from Mekelle University. Thank you for taking the time to speak with me today. We are doing research on the factors that influence the nutrition of mothers and adolescents in collaboration with the Regional Health Bureau and UNICEF.

## So, do you agree to continue our discussion? 1. Yes 2. No

**Section A: Interview details**

1. Zone: South-East Tigray
2. Woreda: Seharti Samre
3. Kebele:
4. Name of key informant: Woldeabezgi Kahsay
5. Institution of key informant: Woreda health office
6. Interviewer name: Amaha Kahsay
7. Date of interview: 13/11/2017
8. Interview start time: 10:00AM
9. Interview end time: 01:02PM

**Section B: Interviewee professional information**

1. Gender
   1. Female
   2. **Male**
2. Age: 25 yrs
3. Highest level of completed education.
   1. No formal education
   2. Primary education
   3. High school
   4. College education
   5. **Bachelor degree**
   6. Master’s degree
4. Current job/position: Woreda MCH nutrtion expert
5. How long have you been in the current job/position:
   1. ______ Months
   2. 02 Years

**KEY: I = Interviewer P = Participant**

**Details of the KII**

**Section 1: Common maternal (PW, nutrition problems in the community**

**I: In your opinion, what are the common nutrition problems in the community for women and adolescent girls at Seharti Samre woreda?**

**P**: Yeah, at our Woreda we have malnutrition protection activity which includes urban nutrition that aims at reducing both the over nutrition and under nutrition. But our main concern is at the under nutrition which includes stunting, wasting and underweight. And we asses these conditions using nutrition materials like that of anthropometric materials, secondly biochemical method, thirdly clinical method, and the fourth method that we use is dietary method that we check about diversification to see either the diet is balanced or not. So, we work based on these four methods; but our more focus is on that of anthropometric and clinical methods; and diversification of food is the responsibility of all being it about children and mothers that it is also used commonly. And as nutrition expert of the woreda, we use these all even up to the lower levels of the woreda. Thus, those with severe and moderate malnutrition are found both the children and mothers including pregnant and lactating ones. We get them by screening; previously before I joined here as an expert before previous two years, there was a kind of practice which is called CHD because I knew it when I used to work at the lower level being HEWs supervisor before those two previous years; so, that community healthy day used to be done once in every three months which used to take one week for each session and it was used to be done by health staffs. What was done right there was that of de-worming, vitamin A, and screening of pregnant and lactating mothers and those of under-5 years of children; thus, those who were <11cm are severe acute mal nutrition and 11-11.9cm were moderate and if 12cm and above are used to called normal classification. And this is what we are using at the ground currently. But associated with PSNP, there is something changed but not implemented to the ground; for example, to be said severe, it is said up to about 11.4cm; 11.5 to 12.5cm said to be moderate and that of 12.5cm and above is said to be normal; but this is simply put in the guideline; not implemented practically to the ground.

**I: Why this new guideline is not implemented then?**

**P**: It is new yet.

**I: You said PSNP; did you mean about safety net program?**

**P**: Yes, PSNP means, those pregnant and lactating mothers in order to have ANC follow up, and in case of not to weakened their health services due to the public work, and also not to be overloaded by work, they are given rest starting from their pregnancy up to one year of the age of the baby; this is associated with PSNP. Yet, the manual says like that even we have one guy who took training in the previous year; so, I can even show you the manual later.

**I: So, why didn’t you implement the new manual of nutrition screening guideline to your work?**

**P**: Let add about the mothers too, earlier I told you about the measurement for children; and what we have used for mothers is that if they are around 20cm, they are called moderate; and we were using 21cm as cutoff point. But in the new guideline, it says the cutoff point is 23cm; thus, if below 23cm, they have to be said moderate. And the aim of the manual is that it indicates that we have to work by widening the measurements. Because, due to our limited capacity, we were working being limited to acute malnutrition; but by widening the level of screening, it shows that the way how mothers and children should be treated and the ways how an awareness should be created to them too widely. And if it worked accordingly, I have a good believe that it could be good; because, those mothers who were at the border of the measurement were only given advice on their health and diets then being sent to their homes.

And yet, even those are found to be in the problem of malnutrition are used to have wrong perception about it; that is, if you screen them again and if you told them that they are normal, they ask you that why they become normal and even they argue they need not to be normal; and I think this comes from the thinking of being expectant to be aided; they correlate that being severely malnourished is source of some gift so that they need to be malnourished then they will be given as a gift in relation to it. But, we let them understand it well; that is we advise them that the ones who are getting the aid are because they are victims of food shortage (malnutrition); and that malnutrition (under nutrition) comes in two ways; the one is due to there is on food to eat and the second one is there food but there is no knowledge on how to use it properly; thus, why are normal is that you have on either of these problems then it is not important to be said so to get this little aid! You tell here to use the home available food products including those vegetable gardens that protect disease and to give her child enriching those foods; you let her understand it is because the others didn’t do as such that is why they are undernourished; thus, they understand you sooner; then, they come back to the thinking of helping oneself, they say I become self-sufficient. Thus, it indicate us a lot has to be done on awareness creation about it, though it has been done much by CHD and RHD; yet, we have not to sleep saying it is already finished; more focus has to be given to the awareness creation of the mothers for the next too.

Now, why we are not using the new manual is that it is said that it has not to be begun until official letter is send to us from the federal all throughout the country on the same way, thus, from the region to us respectively too. That is why; it is not started yet. Indeed, training is already given to the wreda level experts and other low level professionals in the previous year from the nutrition budget that was given for it from the region,; so, it is already being aware by us; but it is not implemented yet at the ground; had it been about a pilot implementation at a given woreda, we would have applied it sooner. But this is given to all woredas and may be to all regions which may also needs common understanding among all; as per my understanding.

**I: By whom was that new manual come; anyways?**

**P**: It says blended, it is prepared by health bureaus and the organizations like UNICEF.

I: Thank you for your detail discussion; and in the case of SAM and MAM, can you tell me the gross number of individuals who are suffered from those problems?

**P**: The number is present in the report, though I can’t remember the numbers; so, I can give you from the report; the number is present there.

**I: How about in the case of micronutrient deficiencies; to what extent is that visible in this woreda?**

**P**: Pertaining to micronutrients, the nutrients are macro and micronutrients; those that are needed in much amount are macro nutrients; they are protein and carbohydrates. Those micronutrients are those vitamin and minerals and can cause iron deficiency and vitamin A deficiency if they are taken low; especially connected with pregnancy via the placenta to the fetus; so it is worked a lot about the iron status during pregnancy. It is also being worked in vitamin A. But there is still problem about them due to the awareness gap; and it has to be worked a lot about these issues. Yet, it has been done in this way about the macro and micronutrients here. And about that vitamin A, in addressing that 17% coverage is being done; it is being done about Albendazole. Screening is being done that of Vita A, de-worming, at pregnant and lactating mothers, and per each month totally. That CHD was once in every three months and there was also EOS which I knew it as information but I didn’t face it at work; yet, it was undergone once per six months. so, you can observe here that the government was working in every six months; then changed into every three months, and again now changed into every one month of screening; this shows that the government is giving focus to nutrition and able to understand how much nutrition is so crucial; and also indicates us that the government needs to create awareness to the community about nutrition. Because, the follow up was in every six months, then narrowed to every three months again to every one month; and who knows, it can be also changed to be in every day follow up! So, there is good follow up currently.

And as to the acute malnutrition that I have told you, it is stunting which is measured using height to age and then it is shortness which shows chronic malnutrition; wasting is also thinness which is measured using height over weight; and underweight is one of the chronic which can happen from during pregnancy up to the newborns and it has both the chronic and acute malnutrition characteristics. So, our focus has to be in the micronutrients deficiencies as they can cause these problems even indirectly. If the community is unable to use the micronutrients like vitamins and minerals, it will happen like anemia and vision problem due to vitamin A deficiency and also skin and immunity problem related to vitamin A; diarrhea can also happen due to Zinc deficiency. So, focus is needed in the use of these micronutrients; even when you compare their availability with the macronutrients in the farm products, those macro nutrients are easily available in the cereals; but less vitamins and minerals. So, visible focus has to be given also to the availability of those vitamins.

We as health part, we work at the immediate cause in disease prevention; there are three cause of malnutrition; immediate cause, underline cause, and base causes; thus, we are working at the immediate cause in preventing disease due to malnutrition by undergoing screening. But that of underlying and base causes are related to nutrition sensitive sectors which is 80% and is being worked there; ours is more focus at 20%.

But the main challenges to talk about right here is that 80% is only name; enough! That 80% is only name which is being hung to health; though there is little change about nutrition until then, there has to be successful change yet; and that comes only if the sectors work together; like if we work with agriculture, education to create awareness; thus, the agriculture which it produces can be known easily; the health can work then at the immediate cause easily; so that the health can work at the prevention areas so that the 39% stunting according EDHS 2016, can be reduced to 26% by 10% reduction; indeed, we are not saying also the health is 100% working at the disease prevention. Yet, in the same fashion like the health tries to address that of the 20% policy if the other sectors work at that of 80% by knowing their clear roles; that is, what they have to do has to be clearly known; 80% is gross figure; it is common figure; it has to be specific if it has to be done; otherwise, it is difficult to monitor and evaluate. For example, if they need to work 80% at nutrition, for example, how will you measure that education is working 80% at nutrition; how do you decide either education is working or not? Uhhhh…How do you evaluate either agriculture is working or not? How do evaluate either women affairs are working or not? So, it has to be specific; like what has said 20% for health; if it is 10% for education, it has to be 10%; if it is 30% for agriculture, 30%.

**I: D o you think mothers including pregnant and the lactating ones, and those of adolescent girls are especially at risk of both macro and micro nutrient deficiencies here in this woreda?**

**P**: The pregnant mothers in the case of wasting, when we measure by MUAC, they are at risk of it; and even the lactating ones though relatively limited. And as to the adolescents, it seems relatively to be started currently; otherwise, it is the most ignored one; even in that of screening is ignored to them; that adolescence is more ignored even in the case of malnutrition. But in the case of the pregnant mothers, they have higher demand; even they carry two lives; on the other side, there is placenta and there is the fetus; so, they are at high risk of wasting when we screen using MUAC; though we don’t know about their stunting condition; and the BMI is not done for pregnant; but it would to be done in adolescence; yet, we are not doing that, so, it needs focus for the future. What we are giving attention currently are the pregnant and lactating mothers and those children even up to the infectious diseases like HIV; but not for adolescents like the focus we give to pregnant and lactating mothers; and this is our mistake that we have to correct it for the future.

**I: But, why didn’t you give focus for adolescents’ health/nutrition?**

**P**: The reason why we didn’t give attention is that, first; it was not come from above being led as a priority agenda; for example, in the case of reporting, we take our reporting formats from zone or region; but what if it has no the format for adolescents? What if it is only about pregnant and lactating mothers, and children; do you think you can work it as a priority task? What will you do it even you work it well if it has not reporting format; this shows you it is not targeted by the government and not focus of priority by the government; so, we don’t give it focus in the same way! Nobody asks you report about that work; indeed, it wouldn’t have been worked for report only; but what we work at is those adolescent who come as pregnant; because they come pregnant, they may get the pregnancy related services. But, what would have been better done is the period of adolescence before pregnancy; because if you work at adolescent, she is the one that gets pregnant three to four years later; so, if you work well at here nutrition, she will not hurt by wasting and those micronutrient deficiencies like vitamins and minerals. Thus, for the future, the government has to work at it giving an attention...Uhhhh (being surprised); yet, it doesn’t mean our woreda is working at it!

**I: May there the problems of anemia, goiter, night blindness and others at this woreda; how do you see their occurrence as per your expertise level?**

**P**: In the case of goiter, it was relatively higher in the previous period due to the iodine salt consumption problem; but now it is relatively decreasing due to an increase in the iodine salt consumption; but it doesn’t mean it is extinct; even that of consumption of non-iodized salt is present; that of spoiled and expired iodized salt consumption is present; which happens due to less awareness of the community; indeed, the community tells you that the packed salt is important for its health; but if you see what is the salt packed; is that properly stored, spoiled or expired; not known well; there is a problem here.

**I: In which group of women from lactating, pregnant mothers or adolescent girls do you think is goiter more prevalent?**

**P**: Mostly, it starts from adolescence; but it may increase due to pregnancy or lactation effect and others later; but more it is observed in the adolescents as per my observation.

**I: What do you think about its reasons?**

**P**: Its reason… (Bit pause observed) uhhh…if you see the community, it tells you it comes from the parents; they tells you that my mother was like that; my grandmother was like that; but if you come to the science, you may see their father and mother have goiter; but if the children are out from that area to towns, they may not have that goiter; and this shows it might be due to iodine salt consumption; and also due to environmental reason like the water you drink if it is salty confined at one place; whereas, that tap water may be fine. Even the geographical location and the water consumed from one place may be the reason; for example, the water in the Seharti Samre, you see guys who drink water from one place being caught by goiter ; whereas, no form other place; so, this needs further study!

**I: Did you observe individuals who are caught by goiter who drink water for a specific place here in Samre?**

**P**: Here in this woreda? Alas, I dint see; what I am telling you is from my…Uhhh (he jumped to the next justification). It is because, when you see many individuals with goiter and if you ask them why it happened so, they tell you it is due to the water they take; so, this is it. But, to be perfect, it needs study!

**I: How about the issues of Overweight and obesity, which are the other sides of malnutrition** opposite to what you have told me earlier in detail; how is in your woreda?

**P**: At our woreda? At these times, due to the living improvements, it is being observed! (Smiled)

**I: Is that much?**

**P**: Not much indeed, but it is being seen even that of hypertension related with DM; even if you check BMI of your friend simply and informally, you may get someone who is overweight (smiled); it is observed especially at those with improved living those governmental workers; however, it is not that much at others.

**I: Great! How about in the case of food insecurity, how much of the community is food insecure in Samre as it is one of the food insecure woredas already known; how do you evaluate this especially to mothers as per your expertise?**

**P**: I may not talk about the percentage; but to take about the guess, in the case of feeding there may not present anyone who never eats dinner; and I don’t think so too; unless, there is any especial and personal problem. But the main concern is that does anyone feeds as per the proper feeding practice; for example, if you say we have to eat three times, butt you may eat two times; in addition, when we come to the quality, amount and frequency, do those two diets contain the proper contents of the appropriate diet; that is another concern. So, we can’t not judge it in blind; it may not be that of increasing from one time eating to more than once per a day; rather all the concerns should be addressed to be said food secure; so, I can’t guess simply; and I don’t think even it will preset; however, it has to be proved by study either it is present or not. But in my case, that amount and frequency matters, for example, let say I eat three times per a day; but if it is only vegetable, I can’t say I am food secure; I may face any deficiency; because, I am not taking from the animal products; I am not from the energy source foods; so, energy deficit may happen to me; and the reverse is true; so, the diversification matters too; then the frequency and the type of diet is important; these are the two important things that can let us be said either food secure or food insecure as our woreda level. Otherwise, it is difficult to judge totally as food insecure or food secure due to someone is not eating dinner or someone is eating. So, we have to see the cumulative of whole day consumption as if to see the food security; not that of the onetime eating pattern at dinner or like that.

**I: Okay, how about in the case of stunting, how do you see the mothers at your woreda; are they stunted or not; when you see them as per your expertise level?**

**P**: Yes, there is stunting!

**I: Can you elaborate it well to me?**

**P**: Stunting is normally determined by the anthropometric measurements scientifically; yet, when you assess it clinically from the previous four methods, it is present at those pregnant mothers though it is difficult to dare that they have all stunting; because, you find them at delivery, at family planning, and at vaccinations; you find them in different ways; even those mothers that you get them at the family planning, may it be because they are married at their early ages and give birth so that they may not increase their height due to that or I don’t know why; but I thought personally if it has any relationship; but there is still stunting at our woreda. Even, if you compare the total stunting in both males and females of the previous with the current population, I think, stunting is higher in this population; may it be because it is only given concern to the wasting currently; or is that the knowledge of the population; I don’t understand why; but, the foods that they used to feed is not present currently for us; for example, the animal product foods used to be present at each house previously; but not present currently; I can speak it surely; in addition, those agricultural products which are expensive like honey and milk used to be present more in the previous time; but not now; and again those indigenous animals and farm products are almost being extinct currently; it is those called ‘begayis’ cows that are present and even these ones are expensive pertain only for those who can afford the payment to buy.; which then even owned by those rich at towns then they sell milk to the governmental workers in contract so then the mothers have milk contract for their children and they may not breastfeed their children. On the other hand, we are creating an awareness that it is begun to develop economic businesses that the products used to be wasted are being used effectively currently; opposite to this again, you have all sources at home but if you don’t use it, if you take it all only to the market, it is mean to the nutrition issue is not worked yet. Again, nutritional sanitation is being practiced well currently; but we are not out of stunting yet.

**I: If you compare stunting according gender, in which of the population from males or females are more affected?**

**P**: Stunting is more at females; in my opinion, it is in the nutritional practice that for example, in the case of husband feeding, it is not totally destroyed; just minimized; for example, if a food is served at home, the mother says I am at home I don’t go nowhere; for example, if there are only two “Injeras” at home, that one Injera will be given to the husband because he will out for business and that another Injera will be given to the children as they will go to school; so if she is invited to join, she will say no problem I am at home I don’t go anywhere; then what happens to her; she will have other works at the home too and she waits only for the lunch not eating her breakfast. Still if you see the adolescents, the concern is given to the boys as they are assumed that they go to work outsides so then they are given food packs having it with them to the work places; but that female is said to be she is at home; yet, she goes to fetch water; then her breakfast passes; she waits for lunch only; then if that right feeding time passes, the nutritional feeding will not work; even our ancestors say so; as well scientifically it works. Then they are overloaded with works; then the time passes due to the work; also pregnancy may come later; so, if not worked during the adolescence and if they are overloaded by work and if their nutritional issue is not given attention, they will be then stunted. Even the adolescent males can eat outside if they have money; they can even see it as fashion; but the female adolescents do not do that.

**Section 2: Nutrition priorities in the woreda**

**I: What priorities have your woreda health office to improve the maternal and adolescent health and nutrition?**

**P**: One, to improve the maternal health and nutrition, we are working at awareness creation via the focused ANC; together with this, we are working with agriculture to reduce their workload so that they will use their time for focused ANC and their health performance will be improved too. And there is nutrition counseling in the focused ANC too. In addition, there is family planning service that may contribute to the economic balance of the family by creating birth spacing; for example, if you need to have two children, you have to have adequate food to feed them which is balanced one; otherwise, if you have many little children, they will not get adequate food then will be malnourished; thus, we are working at it well. The third one that we are mainly working is that of 1000days of life from pregnancy up to two years of age of the child; then the mother should increase her diet at least once during pregnancy and two times during her post natal as she loses more; and she gets the services during her pregnancy and at postnatal care as we said it earlier; so, we strive more to create awareness so that to create link with other sectors like agriculture and education for addressing that in which ways would they help us in the 1000days of life. In the case of the child, it starts from the birth in which it has to start with taking of the colostrums with in thirty minutes to one hour duration; kangaroo method has to be also applied so that the baby will get its temperature and also it will help to increase the child to mother love; he will also breast feed there that will be improved its nutrition; and colostrums we call it first immunization and the community is understanding it well and it is the success that we achieved it well. In addition, we are also working at good attachment and position of the breastfeeding to the baby. But the challenge which happened here is that when the lower lip of the baby is attached to the breast to cover all the lower part of the areola, the mothers have fear that the lower lip of their babies will be large; thus, they resist for the good attachment due to their perception about the lower lip enlargement of their babies; it is still observed at occasional ways of mothers especially at the remote areas. Moreover, to the good attachment and positioning, we also work well at the frequency of breastfeeding that the baby has to be breastfed at least ten to twelve per 24 hours; we counsel the mothers starting from their fourth ANC and also when they come for family planning; and the challenge here is also they didn’t pick-up the baby to breastfeed if they get him sleeping; for example, if she is at work and the baby is slept, it might be his time to breastfeed, but she doesn’t do that then, that of 10 to 12 breastfeed frequency will not be reached; by the way, work is everything for females; they are found in everything at any kind of work; thus, this hinders the frequency of breastfeeding of the baby. In addition, we work at the first six months of the baby that it has not to take even water and other foods before his six months of age; and the reason is that the issue of the GI and stomach maturity to digest foods; he can’t digest foods; then when you advise them hey accept you 100%. But starting from his sixth months of age he can be given complementary feedings; but the problem here is that, that of the promotion of saying that starting optimal complementary feeding after six months is really confusing that is being promoted by the mass media promotions that we have to follow them strictly and even we are saying it ourselves; but this is wrong if you see it; because if you say it has to be started after six months of age, that ten month is after six months, that nine months is after six months and even that one year is after six months; so, if you ask a mother of one year age of child either she started complementary feeding or not to her baby, she told you that she didn’t start yet; because it is started after six months; and that one year is earlier after six months yet! So, the time when should be strictly addressed in Tigrigna as most of the mothers consider that of after six month is normal up to one year of age of the child. Indeed, we are working at it well and it might not be present as such much; but, it is not worth to say something is totally avoided; rather, better to say I reduced it but I have to search it further; thus, it has to be worked a lot in reducing these challenges too. There were also challenges on the cleanliness of the umbilicus of the babies that it was put like mud, butter, and others to it for the purpose of lubrication not to be dried when it is newly cut; but this is almost avoided currently. there was also a challenge of washing newborn in below 24 hours of birth; but is not recommended to be so as it hurts the baby by hypothermia; and this is also being avoided by the mothers as they are able to understand that the white liquid being born with the baby is harmless.

So, we are practicing it well and our mothers are accepting it with full understanding all about the timing of breastfeeding initiation, and complementary feeding at sixth month of age. For example, our best experience in the initiation of complementary feeding is that we began candle lit welcoming at the sixth month of age of the child to say him welcome from the age of only breastfeeding to the age of complementary feeding; and we tried this candle welcome at health post together with the HEWs and the development armies and it is being welcomed by the mothers too which can show them that the accurate time of initiating complementary feeding is sharply on the sixth month of age for the child; this is because the candle can be bought with very less cost but its effect to remember the date for complementary feeding initiation is big; yet, the coast for celebrating birth day is more which may not worth to the candle lit role to its nutritional role to the child; thus, this is being expanded from development army to another army and from kebelle to other kebelles; but, I am not sure if it is totally implemented at each households yet; so, this is the best experience that has to be extended to other woredas too; we already tried it at Myramoko health kebelle at its hgealth center; and it is also extended to Adishishay and Mykana health posts there; Gijet and Esret health centers are also practicing this experience.

When we come to the complementary feeding, we let them begin with enriched porridge classifying their ages into two; that is, those children from 6-11 months are let take very loose porridge which is as loose as the honey with no residue (Wolela); and it has to be enriched with milk and well chopped and powdered meat so that not to be chocked. But the challenge here we face is that the mothers have fear of chocking of their children if we tell them to give meat or those vegetables; yet, the problem is on our counseling technique; that we have to let them know how to give them by chopping it well and make it clean and cooked well too; thus, they accept it well; and this indicates that it is due to our communication gap that may let them fear. In addition, the food they eat has to be enriched from the cereals of three fourth, and legumes one fourth, and others too, including those micronutrient source that can be given to them as snacks after their meal. Moreover, we also give practical trainings for mothers that have micro-garden accesses; that is, if they have vegetable gardens, they use those vegetables and other home available farm products so then it is demonstrated to them by the HEWs and the WDAs about how to prepare that porridge and to give to their children; and it has good influence to the practice of the mothers about feeding their children. This all what we do about the feeding practices of children up to their 2^nd^ year of age; and also, we undergo de-worming and vitamin A provision during this period of age and also that of screening service so that it is linked to the nutritional condition in which we able to see what change is present due to the breastfeeding and complementary feeding practices to the children.

**I: Great! You told me in detail all about what you do in the first 1000days for children; how about for mothers there?**

**P**: About mothers in the 1000days of life is focused on the ANC follow up that firstly we undergo study on the LMP and EDD of the mother; so, if this is known, you tell her when she will deliver and you will follow up here together with the HEWs, WDAs and the religious leaders and other stake holders too. so, we expect them to begin the ANC at their three to four month of pregnancy; but the problem here is that there is delay that they may not even begin ANC follow up to their sixth month of pregnancy; and if they begin late, they may not have those four ANC visits; yet, we work also about her nutritional conditions like measuring her weight in each visit so then we able to see the changes; so also, we give her dietary counseling based on that finding. Yet, the challenge here is that the diet and the family number, but, we tell her not to jump her breakfast, to eat lunch and to take her dinner too. in relation to the fasting too, there is a challenge; but we deal with them up to telling that it is even allowed by religion for those pregnant mothers not to fasting as they are carrying two lives; we even deal with the religious leaders about it; I even remember that there has been a negotiation at regional or federal level with the religious leaders that allows for pregnant mothers not to fasting; I have seen a manual about it; so, we tell them accordingly. For example, if she is going to fasting for forty days in the case of Ester, she will lose much in her nutrition condition for that much days; so, we advise them it is allowed even by religion and also we advise them to eat like the way they prepare for their children from varieties of food sources; they have to eat together with their husbands too. We also deal with agriculture on to reduce their workload during public works so that they can get adequate rest and can use it for other health services just starting from her pregnancy period; we also deal with their husbands about reducing workload at home about getting her additional diets at home and also about screening HIV/AIDS together; and even about family planning what use after delivery agreed together which one is convenient to them.

Yet, we also advise the pregnant mothers to increase their diet by one from what it had been before pregnancy; but what my concern is that how much times did she eat before pregnancy; what if it was once per a day; are we saying she has to eat two times per a day then; so, this is mean to it is accepted by the government and then we are using this principle; or are we sure that the community is eating three times a day then it is undeniable to talk about the additional diets; this is for your consumption and research that I am talking indeed! The same is during lactation, it is said that she has to increase two times per a day; how much times was she eating during pregnancy is the concern then. So, I think, we have to ask first if she was able eat her breakfast, lunch and dinner; so, we tell her to add once during her pregnancy; anyways, what we advice for pregnant mothers to do about diets is that it has to be small but frequent, it has to be clean and hot. Because, eating more only once may not be worth for her; for example, eating much once with her husband but not repeating later alone is not okay for her; rather, she has to eat small but frequent times, clean and hot, so that her nutritional condition will be good; and that is we advise them. But in the case of the increasing one diet to the pregnant is doubt; for example, are we saying we do have same socio-economic status; do the pregnant mothers who live at woreda Seharti samre, at rural and town eat same three times per a day? (Surprised and laughed). So, you have to do research and you have to correct and bring it back to us (opened his mouth like laughing and indicating request for researching the issue).

**I: Thank you for the detail! So, when you do all these nutrition related interventions at your woreda; do these nutrition interventions have their own budget allocated?**

**P**: What do you mean by budget?

**I: I mean are resources allocated for the nutrition related activities you perform here in the woreda?**

**P**: Yeah, even it is send from the region said for nutrition; we also have nutrition specific budget that if it is planned to give any nutrition related training or activity, we do it separately; it is not mixed with other activities; but, as to the human resources there might present difference when it is performed by one alone or by tow together.

**I: But, maybe here the activity for nutrition may be separate; but how about the money, car, time, and man power like it is allocated for other programs like TB, and HIV at woreda level; is there such kind of distribution for nutrition too?**

**P**: I think it comes from UNICEF and Tigray regional bureau; and if the budget comes for nutrition, it is never be used for other issues as nutrition is being given focus by the government; for all I know, it can be even used from other programs if there is opportunity of excess budget there or if nutrition related emergency or urgent condition is occurred too. Otherwise, at our woreda level, the budget allocated for nutrition is very limited; unless, it comes from above!

**I: Do you think it is necessary for your woreda health office to participate in this nutrition related interventions that you mentioned them above; or is that because it is compulsory to work that you are working it?**

**P**: Especially that of child and maternal nutrition, it is beyond necessity which has to be even as a mandatory to be done and it has to come to us even as an order to work it; it is crucial! I don’t know in what words I shall tell you! Unless there may present any budget constraint and other reasons, it has to be even priority of the country to work at nutrition; because it is that of nutritional issues that lags you back from many things; so, it is undeniable about its necessity to work and it has to be even our mandate working at it. I told you earlier the focus that is present is at lactating and pregnant mothers and under-five years of age children; but that adolescence is forgotten; for all I know, the government has to shift its focus to adolescence; because, if it is worked there, there will not be present any problem during pregnancy later; for all I know, it has to be increased more than what is supposed to work by us currently; nothing has to be left not worked related to nutrition issues if possible.

**I: So, how would you convince about it necessity if someone comes to you and arguing you that it not necessary to work at nutrition; in few words?**

**P**: There may not present anyone who argues about it is not necessary; yet, why necessary is that, one, when we work at nutrition, the productive human power comes from it; because, if a pregnant mother doesn’t feed well, she will not give birth to a normal baby; and he will not physically and mentally grow appropriately; if so, he will not be for himself; and if not for himself, he will not be for his country too; and this results failure; the, nutrition is crucial to the country if you need to create those who are knowledgeable and can do research who are smart and concentrated. You can’t be even great leader without good nutrition, so, nutrition matters for political and economic and social issues of a country.

**Section 3: Nutrition interventions that improve adolescent and maternal health**

**I: Because we have already addressed it all about what nutrition intervention are being done and what challenges are present at your woreda; we will not go through them in this section; but I need you to explain me about how is iodized salt consumption practice here, because I have seen practically individuals who travel on foot with their loaded donkeys of that non-iodized salt which is that of bar of salts, here in our ways from Samre to Abyi-Adi?**

**P**: Yeah, in ht case of iodized salt, as I have said it earlier there is change but we are not totally out of that problem until then; and there are reasons for this; one there awareness is not disseminated to the entire community the main reason that we say; second, the iodized salt is not easily available to the community; but they get that non-iodized salt very easily when it compared with that and this is that let us be in difficulty to manage the problem.

But if you ask us what we are doing about it; one, we are doing about checkup of the iodized salt; and we get problems there like that of lacking reagent to check the iodine in the salt; and the presence of that bar of salt in the market is also another problem. Thus, when they lack that iodized salt at the market, they take that bar of salt saying that this is our all time salt that let us grow until then; thus, let us take this; and they take that. Yet, we teach them about the importance of the iodized salt consumption to brain development and the way how it has to be cooked in the food and they understand it well; but the problems are; one, the production of that bar of salts are increasing but that of iodized salt is decreasing in its availability and quality; even the one which is available is fake; which may not be appropriately produced; even it was our agenda of discussion some time ago; strictly, it has to be known officially who is producing that iodized salt; how much is its quality addressed; and also its expire date has to be checked before consumption. Otherwise, there is no understandability problem by the community; but there is access to that bar of salts in the market; then, they doubt that had it been that iodized salt important, that one would have been prohibited; but not! So, they buy it. Thus, if it is not important for us, either let’s quit it totally that iodized salt; or otherwise, let us work hard at avoiding that non-iodized salt and increasing the iodized one to the market; it has to be known who is producing it; how is its quality; and it has to be addressed to the rural community; it has to be accessible to the community at the rural areas if you understand me.

**I: Who is bringing the iodized salt to your woreda?**

**P**: It is those merchants.

**I: So, don’t you have any controlling mechanisms about approving its quality and accessibility and prohibiting that bar of salts?**

**P**: No, except raising it as an issue in some meetings. For example, it is that so-called Shewit salt which is thought to be iodized which comes here; but I don’t know if it has expire date labeling; and also how many are reading that expire date accordingly is another issue. I remember also there were some ones who sold fake packed iodized salt at around Raya; and it was raised as an issue in a meeting at Maychew. So, there has to be a checkup mechanism for those who are producing that salt and their quality has to be seen and approved; and also, if any mishap happens, they have to be let accountable for their mistakes; because, they can even increase its quantity if it doesn’t have good quality when they pack it.

**I: May there any mechanism that you can do to control them?**

**P**: I don’t think we cannot let them stop their work, it may not be our mandate; or either I don’t know it if we have the mechanism; either you have to study it and let us know; or if you have something to tell me, you may tell me later. (He laughed in a kind of requesting gesture). Even the mothers ask you that they need to buy that iodized salt from shop; but then they don’t get it; hence, they buy that one.

**I: How is backyard gardening practiced in your woreda; are there, mothers who practice this micro-gardening?**

**P**: Yes, there are sporadic individuals who may use that; but it is where those who have irrigation to practice it; otherwise, it is not practiced due to the shortage of water here.

**I: Is there not any means to dig water wells so that the community can practice the backyard gardening?**

**P**: Indeed, water wells, are being dug; but it will not be adequate to use it for gardening beyond the domestic consumption.

**I: How about in the case of malaria, and the habit of using ITN by the community and the mothers in particular at your woreda?**

**P**: The protection of malaria in our woreda starts form the sanitation system in which the community is educated to clean its environment by draining any confined ponds, filling and dumping swamps, cutting grasses and other small bushes; which then avoids all the convenient condition for mosquito breeding; and this is developed first by awareness creation to the community. And the second way of malaria protection is using that ITN in which it is distributed to the community by giving priority to the pregnant and lactating mothers and those of under-five years of children; yet, it is distributed to all households if there is ample amount of ITN; otherwise, the priority is to them. Then if someone is sick passing these protection methods of keeping environmental sanitation and using ITN, he will come to treatment and will get medications accordingly; this is how we do.

**I: Is there de-worming service for mothers?**

**P**: De-worming Uhh…Albendazole?

**I: Yes, anything that could be given for lactating or pregnant mothers?**

**P**: I think there is for pregnancy mothers; previously it was said there is contraindication; but now since the second trimester, they can be given drugs for de-worming service; I think so.

**I: How is your woreda doing at providing additional food services to the pregnant and lactating mothers which is that of targeted supplementary feeding?**

**P**: That of supplementary feeding is given in two ways that of blanket and targeted one; that blanket supplementary one is for all; whereas, the targeted one is specific for pregnancy and lactating mothers and under-five years children; but there is no any service that they are being given. It may be at schools;

**I: But why that TSF is not present for the mothers and children?**

**P**: Previously, there was Faffa which was given to them; but either is that due to economically or due to we bring change why it is stopped; I have no knowledge about it. It was given in the previous time for those who used to have moderate malnutrition; for all I know, as I told you earlier, there was even a perception by the mothers that they used to come thinking that they will get that Faffa instead of coming to be checked and to be said normal; they were not feel okay if you told them that they were normal; they used even to complain that how comes, that women is more thick than me but she is given; why not me?! There were such kinds of complains too. And also those children who come out from severe malnutrition from OTP, used to take that of Faffa; but all is not present currently.

**I: So, what is being done for those with moderate malnutrition currently?**

**P**: We encourage that of complementary feeding; that is it.

**I: What if they are severely malnourished?**

**P**: They are sent to the OTP centers, and if they are beyond the OTP, they are referred to the TFP centers.

**I: How about youth friendly services, is that present in your woreda?**

**P**: Youth friendly service Uhhh…yes it is present; but it is not as per to the level of its demand to be done due to lack of room to undergo the service. It is not worked much; it is not focused; because, YFS is a package that aimed at those 15-22 years adolescents and it has to be done at its own room and OPD having its own materials for checkups and its focal person which would to be intended to have its own special nutrition; but there are many problems starting form shortage of rooms up to other services to be given separately; though some health centers began it partially.

**I: So, what are the clear challenges that let you not work at the YFS properly?**

**P**: The one thing is that lack of materials like the registration formants that it should have its own reporting and tally sheets for the packages provided there. Another problem is that lack of rooms to undergo it; thirdly lack of those screening instruments is the challenge; yet, that of family planning and HIV screening is partially provided at this time. Yet another problem is that there is shortage of human power in addressing the services for these adolescents even that of family planning provision.

**I: Is there any school feeding service at your woreda?**

**P**: Yes, all the students were given Faffa in each day during the drought periods of 2007 and 2008EC and it was given to them in the form of flat bread (Kitta); and currently, it is planned that it will be continued though not started yet.

**I: From all the interventions that we have been talking about, especially those focused at pregnant and lactating mothers, which one do you think is the most successful?**

**P**: Yeah, from the successful ones, the one is the ANC; and next to that is that of breastfeeding.

**I: Why is ANC successful?**

**P**: Why I said so is that due to number of visits that it is improved from time to time; they are able to have four focused visits many of the pregnant mothers which is currently comparable with the number of deliveries undergone; that is why it is successful.

**I: How about that of breastfeeding?**

**P**: Breastfeeding, its failure was that of colostrums initiation at earlier time and that of only breastfeeding up to six months; now it is almost improved; and that of from six to twelve months of age is also going well in both the breastfeeding and complementary feeding practices.

**I: How about the intervention that you are not successful in the achievements?**

**P**: From those which are not successful programs is; one, that of reduction in severe and moderate malnutrition is not moving well as per the government’s program and policy to reduce it; even it was expected to reduce it up to zero level; but if you get similar numbers and cases at every time and month, this shows the intervention is not successful.

**I: What can we think about, why this is not so successful?**

**P**: Why it is not successful is that one, it is in the complementary feeding; second on the production problem of the food source and third it is due to the provision problem of these foods to them. Another reason is that there may present awareness but not supply or there may present supply but on awareness. And also in the case of breastfeeding, though there is that of 52% of breastfeeding up to six months; there is still interruption by the governmental workers and the same for the complementary feeding that the babies are not given at appropriate times.

And all this failure came from our failure in working together with other sectors; because to reduce that SAM and MAM, its 80% is needed to be done by other sectors; but there is on specific roles allocated to them yet; there is no visible performance plan; thus, you fail to monitor and evaluate it; you can’t measure whose success or failure is that about; even we don’t know to what extent is our working together with the other sectors, though we may say we have achieved about 50% or other things about it; so, that multi-sectoral collaboration needed to be re-worked again if we need to bring change! (Uhh…laughed as the sign of looking down the situation of the collaboration)!

**Section 4: Implementation challenges and community factors affecting access to nutrition interventions**

**I: You have told me in detail about the interventions related to nutrition and what challenges are present behind them; yet, may there other challenges and factors specific to the mothers at individual level due to difference at their educational levels; and at community level like that of culture, religion, and others; what can you tell me here; if any left from what you have told me already?**

**P**: fortunately, that of fasting related to the pregnant mothers is being resolved; that it is not a problem currently; yet, I doubt; if we see it in the religion side; it may present secretly that there may present who are fasting; for example, the problem is that in the case of Muslims, if they are fasting, they have to stay with no food all the whole day; relatively that of Christians is up to around 9 hours per a day; so this has to be assessed yet; as both of the religions are found at our woreda level.

But as to the culture, we have not as such weird culture of feeding; thus, there is no feeding problem related to our culture. But in the case of education level, you can see it in two ways; maybe we might be 50:50% or it can be checked by study; but if you see it to during the counseling session, the ones who never are educated accepts you every ting you tell them very openly when you counsel them using the steps by GALIDRA and ORRPA approach; but those who may learn any, when you counsel them based on those steps, they don’t want to accept you; they resist you; maybe it is due to the little knowhow they have or not, but they don’t want to take it all; they resist you correlating to their ancestors’’ practices. Yet, if you see it widely, those who are educated ones better understand you at least if you ask them back what you have said them, they can tell you back well; though it is not known about its quality that either they capture it all or not! But you can’t conclude that only those educated ones are more understanding than those non-educated ones; because, you can see that those who are learned some may not able perform more than those non-educated; which still maybe due to nutritional effects or natural defect.

**I: How about the interventions themselves, do you think they are full of quality when performed at your woreda level, how do you see them to address the need of the community?**

**P**: In the quality aspect, it is not done to the fullest; when I say this, for example, the successful is intervention that we said is ANC; but there are still gaps that are not delivered to the mother when she comes in need of that service; then reduce its quality; for example, she would have checked for syphilis case at her first visit just to continue the next three visits properly; but not, because, there is shortage of man power; there are no materials for diagnosing that syphilis; thirdly the interruption of electricity which may distort the reagent for test; thus, though there may present professional and reagent; if no light, the mother will not get that service; and again there may present that light and professional but if on reagent, the mother will not get the service; so, she doesn’t get the service to its 100% at any time. So, if this much is missed in the service that we said it the most successful intervention; what will you imagine in the programs that we are not working them successfully!?

Especially, we have to work well at the breastfeeding and complementary feeding of the 1000days of life; even in the case of breastfeeding, we advise more during her pregnancy about it and about her feeding; but after her delivery we give focus only to the feeding of the baby forgetting that mother during her post natal care; we ask her only about the feeding of her baby; but not about hers; so, this has to be done; and also focus should be given to the complementary feeding of the babies; because, you may demonstrate the sample preparation; but if the production is not sustainable, you can do nothing about it; by the way, the complementary feeding can be successful if and only if that multi-sectoral collaboration is functional; that is, health can’t do alone anything; but if it is needed to be addressed to all the community, the agriculture has to sustain the production of the food sources; the education sector has to have nutrition education policy so that all the generation has to have knowledge about nutrition and it has to be sharpened about it from the very beginning; but with the current situation it may be difficult to address effective breastfeeding and effective complementary feeding.

**I: So, in addressing all the challenges what we have discussed them here; what is your woreda health office doing; any sacrifices that you underwent?**

**P**: So, what we have done about nutrition is that, one, as our agenda is disease prevention as up to the 20% allocation, so, we are creating awareness to the community on diseases prevention in which it is also about nutrition as nutrition is about disease prevention; even going to the meetings; so they are able to tell you about even what nutrition is to them; but the problem is on its practical implementation; they are not able to practice it. So, in order to bring the practice, every sector has to take share and responsibility; for example, in the case of education sector, it has to see why students drop out their schooling, what does their nutritional condition looks like, and it can be part of study as it can also show us the nutrition condition of the community as almost every community member may have a student that go to school. So multi-sectoral collaboration is needed; otherwise, when you are working at the 20%, again if you intend to work together at the 80% and/or if you insist them to that 80% work alone without their involvement, this will lastly creates despair and failure to the nutrition success. So as to me, like it is given the 20% allocation to us; that 80% of contribution has to be specifically allocated to each sectors so that they can take their specific roles even by undergoing any study about it; and they can be evaluated accordingly; they have to have annual plan and report so that they will be checked about their achievements; otherwise, who will be responsible for that 80%; who will be asked if that failure happens; is that the education sector, is that the agriculture sector, or are thy those unions? For example, if that 20% fails, he health sector will be responsible! So, either there has to be a way that sectors can send their report to us so that we can send it accordingly or they have to have level to level reporting system from ground to above levels inside their respective sectors. So, in addressing the underlying and basic causes like the social, economic and political factors by the 80%, it has to be worked out by strongly involving the political leaders too.

**Section 5: multi-sectoral collaboration to improve maternal nutrition**

**I: We have talked a lot about the necessity of multi-sectoral collaboration for the success of nutrition issues for the community and mothers at large; so, which sectors do you think are necessary to work with your woreda health office?**

**P**: When we come to the sectors, especially the need of agriculture and education is great; that education is needed for awareness creation; and if the awareness is changed into practice, it will work to the health. Women’s affairs are also important to create influence as they have chain with the females up to the ground level; in addition, that of related to the justice of children and their rights, the social affairs can let respect the schooling of these children, and can also address their basic needs so that their diets can be addressed there; moreover, political leaders have to be involved which can then see and lead the specific activities allocated to the sectors either they are achieved or not, then, that of TVET is also needed; because they emerge different unions and trainings like of metal works and female unions; then it can be easy to create awareness on those unions as they work and live together so then they can take it down to the ground again which makes ways of communication about nutrition very easy if such unions are able to have awareness about it. Another important one is that of water sector, which it is important to practice micro-garden and agricultural works; if there is water access, the mothers would to produce vegetables at their gardens.

**I: So, if you were given the chance to make the multi-sectoral collaboration live and more effective, what do you think to do about it?**

**P**: Aha (Laughed)! One, these sectors have their own budget; so, if it is possible to have its own budget for the nutrition too like them; that coordinated work would have been done successfully. Second, if this is not possible, that 80% of role has to be allocated to each sector; thus, they will allocate budget for it from their own budgets by any means; because, if I am in the political lead, either its own budget will be allocated or they will allocate from theirs so that they will develop plans in their leading priorities about nutrition; for example an agriculture will be linked with water resource by hook or crook for the sake of water and micro-garden productions; health will be linked to education sector by hook or crook to create the awareness. So, it would be possible to create a system of coordination that would run by itself; rather than moving haphazardly.

**I: So, if we intend to create such kind of multi-sectoral collaboration, what resistance would you fear to be happen?**

**P**: If it is possible to create this collaboration, it will have its own budget and focal person in each sector that can run it smoothly so that they can see and resolve their challenges there; yet the feared challenge could be that they can excuse that they have overloaded tasks which then can reduce the focus to nutrition; yet, if they are strictly given as their specific task, they would to do it by any means integrating to their daily activities. But because it is in its whole at this time; no sector is able to take the responsibility which let them waiting to each other without any work about nutrition.

**I: Do you have any nutrition related coordination platforms at your woreda level; does you woreda health office coordinate or participate at nutrition related platforms?**

**P**: Our woreda health is invited to participate at nutrition related platforms at zone and regional levels; as well our woreda health undergoes nutrition related awareness creation and technical training platforms to the lower level professionals. We also use others’ platforms; that is opportunities from other sectors’ platforms that we take little time and we transfer our few messages about nutrition which might happen either shortage of budget or time to coordinate such kind of platforms; for example, during the meetings of religious leaders, women affairs’ and those youths;, we use their stages and transfer our messages; this is because the youths are productive man power, the women affairs are part of the women development armies and those religious leaders can be linked to the feeding practices and fasting.

**I: Great! Do you think these platforms are successful or not?**

**P**: The success at awareness creation from region to zone and zone to woreda is successful; everyone comes with adequate knowledge; but the problem is from woreda to lower level; and another problem is that of lack of budget to run different platforms separately for different segment of populations like that of religious leaders females so that they would to take it clearly in to their other group members; if you are not able to monitor for the ones you give them training, it can’t be said successful. There are even individuals who are trained well but apply nothing from it even at their home. Yet, the level of understanding of individuals is so different that some one can understand easily and may apply it; whereas, others may need very repeated information to understand it well; so, what we are giving here as short trainings are for those who can capture it at shorter time period; which is then mean to there are individuals who may not understand it as well; and his is not successful then.

**Section 6: other intervention that influence adolescent and maternal nutrition and health outcomes**

**I: It is known that not marrying and giving birth before the age of 18 years old for girl is prohibited by the government; so, do you think it has relationship with her nutritional condition, and how about that of birth spacing?**

**P**: It is true that of not marrying below 18 months of age for a girl is being promoted; not only promoted it is also used as a slogan; yet, it is not only promoted by health; it is done by all the community too; other bodies like the women affair, youths, and the administrators too. So, its importance is that, one, it is without interest of the girl so then it causes her psychological depression; it also causes obstructed laboring; the problem of abortion and fistula; and other problems like cesarean section and related to fistula; bleeding during the procedure and even may causes her death. If there is fistula, it may let her be isolated from the community which again causes her to be psychologically depressed; which then let her to her thinking of had not I married earlier, I wouldn’t have be interrupted from my aim; I would have continued my education; which then again causes her not to lead good marriage life and let her be despair. So, early marriage has both physical and mental influences; even it has influences t her nutrition.

**I: How on her nutrition be influenced?**

**P**: One thing, she is not interested at the marriage; so, it causes her poor bond to her spouse as she may think about the interrupted aim; so, this in turn causes her not to eat properly not to have proper dietary practice due to that disturbed life. So, this is a danger life would it be.

**I: How about that birth spacing, how do you see it?**

**P**: It is related with that; if there is on birth spacing in addition to that of early marriage, those all above mentioned problems will happen and it will also degrade the economy of that household. So, birth spacing is very important; one, in the case of nutrition, if the mother is to provide effective breastfeeding and complementary feeding in the 1000days of life, she has to have birth spacing; otherwise, you may get mothers get pregnant at their sixth month of lactation and one year of lactation; this means, the baby is not breastfeed the first two years in the1000days of life which then let him fail both mentally and physically improvements; then this will be lead to the country and even for himself that can’t create anything.

**I: So, what kind of policies do you think can promote these issues very well?**

**P**: Which one?

**I: I mean to improve the current situation of breast feeding, birth spacing and early marriage?**

**P**: For example, in the birth spacing what can make it successful is that an awareness creation; unless you don’t say me that I am inclined, what I need is that it has to be included well in the education policy and all the adolescents have to know it very clearly about what is the problem behind marrying before 18 years of life; if they know it well there during the adolescent time like the focus given to the pregnant and lactating women, it is those adolescent girls that later become pregnant mothers; so, we have to let them know and feel it well; thus, change can really come; we have to teach them all about pregnancy and its impact about early marriage and its impacts and if she has already married, about how to use birth spacing and its importance; because, she doesn’t know where to go and what to do; we have to lead her.

Anyways, the way how we are doing at family planning has to be so different from the previous; because, we are saying that our 855 of population is living at agricultural works; but on the other hand, the land is getting narrow and narrow (Laughed with surprise)! For example, nowadays it is only that of half of hectare that is allocated to a household; and that is why; youths are now given land in union at mountain sides currently. so, what is better now is, one, the youth has to be educated; because, what will happen if someone has eight children and if they divide that of half of hectare to all of them; they will pass their time being fought to each other; this has its own problem at the security. So, if there is fighting among brothers, it will cause fighting among villages; then it will cause fighting among kebelles, it will then causes fighting among woredas, then again among regions; then it will come to the security of the country like what we are observing at our country currently. so, as far as there is on option about the land we have; it’s becoming narrow; so, family planning and that of birth spacing are best choices; and it is if we work here that nutrition can be succeed again. Because, anyone can work and help himself and learn and innovate tings too if it is nutritionally well; but if we don’t work at the adolescence time, there will be jobless youth and that will be the risk for the security of the country too. And this is what I can say and thank you (He laughed happily; I think, it is for his deep elaboration)!

**I: So, what do you think the factors or challenges at community and policy level that may hinder us not to undergo these issues of nutrition with family planning and early marriage promotions to their maximum achievement?**

**P**: If there is no good birth spacing, there will not good nutrition to the children; and this can also let them to early marriage engagement; because they don’t understand, they are not mentally developed and let them need that marriage earlier; they will not have interest at education. The first thing here is that the birth spacing problems; then they are not fed well due to the narrow land and its low productivity; thus, their nutrition is hurt; then, during adolescence, they intend to do those weird activities which are not appropriate. This is how the chain of problem it goes. This is because, there is burdened problems here that he is not fed well; he is not educated well; he is not grown well; so, it causes problem during adolescence that need to marry earlier but not interest at schooling. Thus, birth spacing is very important at addressing the issues of nutrition, education, and security of a country. And that is why, even children are not successful at their schooling which might b due to they are not getting their basic needs like that of food due to absence of birth spacing which let them not to get adequate foods at home. So, there has to a generation created about who needs to learn and who needs to innovate and can be benefited from that, rather than who waits that agriculture; because even that modern agriculture may have its own doubt as there is no still adequate land to practice it; so, the next generation has to march for better education and better technology innovations; and this can be achieved working at birth spacing and nutrition.

**Section 7: additional remarks**

**I: What lessons did you learn from the interventions that intend to improve maternal and adolescent nutrition?**

**P:** Related to the maternal nutrition, it has great lesson that if you work at mother it is mean to you already worked at the total population; it is mean to, you are designing the next generation to be more innovative and educated. If you care a mother from her period of pregnancy, it means you are caring about her baby, then that baby will learn well, and that will created educated generation that can build the country; and our ultimate goal is seeing developed country; so, this can be achieved if w work at mothers; so, being work together with agriculture, education and others; our main target is at the mothers and this is to create better generation for the country. Currently, the mothers themselves are witnessing that there is good work at them; they are saying to the adolescent girls that they are lucky that there is education and health at their home; and the mothers wish as if they were adolescents at this time so that they would to use all the available opportunities for better change.

**I: what opportunities are present at your woreda to make successful changes to the maternal and adolescent nutrition and health as per the directions you gave us?**

**P**: Fortunately, the community is eager to be changed if you are in a position to bring change; this is one opportunity in our woreda; another one, the staff is so young that you can work with it together for better change.

But before that, if I dint mix it together; related to the policy challenge, in the case of quality education, in my opinion, the government is not moving appropriately; especially since the previous two years and like that, there is no quality education; it is only being talked as quality education; but no quality education is present!

**I: Can you tell me some evidences about why you said it lacks quality?**

**P**: The one that I can assure is that, the previous time grade one student when compare with the current grade-fifth student; their knowledge is incomparable; that of grade-fifth student may not able even to write his name well; but that of grade-one student in the previous time at our period may able to write and understand things well. Even you can observe form the health professionals who used to be produced in the previous times and those who are produced at these times. So let me come to the nutrition, we are saying there is a community who can accept and understand us well; but there is no manpower who can let the community understand well and who can bring change to the community; because, if the professionals are not well trained and knowledgeable, they will not convince the community; rather, they will be convinced by the community! We have reached a time in which the community can challenge the educated ones and can change their thinking as we are creating awareness to the community. But, the man power being produced currently is free of skills and adequate knowledge. For example, those who are coming to us at this time, when you order them to do one thing, they say you that they dint take the training; but they are diploma professionals said to be passed that of COC. So as to me that of simple pass from grade to grade like from grade one to two, three, four and like that has to be stopped; we are pretending the generation; we are not healing it! This is my personal opinion. So, we are saying we will do many things about nutrition for the future; but where is the human power to do so; where is the human power that we are creating!? So, if you able to add what I am saying all, even that cheating at exams is becoming as our culture at this times; and why this cheating is coming is because there is promotion form grade to grade with no knowledge to be promoted; why not I repeat two to three times if I have not knowledge to pass! Where is failing of students at this time!? And this is also related with the result-based evaluation scores (Wutsiet Tekor) of the teachers too; the teachers are becoming careless at their evaluation to the students; because if you evaluate your students correctly, and if you let fail two to three students, you will be asked why you did so, and even you will be said it is because of your laziness they failed not because of their laziness; but you know that you are the best teacher; yet, the student may not understand you because of their nutritional problem or birth spacing problem; you will be forced to let them pass then; because you will be evaluated as poor teacher; then you will not get promotion; you will not get scholarship opportunities and also you will not get salary improvement; then, your option is killing the generation; because, if I lay and let paass the students, I will be given that of 100% result-based evaluation score; but I am killing the generation; whereas, that teacher who is working at creating better generation by working the right thing will be given zero and will be depressed then; and even he will be commented, blamed and even will be fired from his job too. So, the teacher is developing the conceit of what do I care who ever passes; quality no matters; quantity matters; so, all students are passing. Them what is now happening to the government, youths are becoming joblessness; what is the problem right here?! Uhhh…those who are jobless youths at this time would have searched their fates to be like merchants and those rich ones when they were at their grade 8^th^ and 10^th^; had it be well qualified way of education delivery and passing from grade to grade at that their period. You got me? All these jobless individuals would have owned their jobs at this time; but now everybody passes being collected together; then what comes next is that the complain of saying we have on jobs; they say that the government taught them; let them graduated but not gave them job. Then this is the problem created by the government that is becoming a problem to itself the government. So, I believe that if this policy of education is corrected, that of joblessness will be resolved. Otherwise, what is the importance of letting individuals to complete grade tenth and/or they have their diploma but sat down. And this is becoming a challenge to the government; and the youths may go to different malpractices like joining opposition parties

And creating chaos and insecurity to the country to if they are jobless; even if the leading party needs to continue, it has to work hard here; as to me, that of the 1990s policy is appropriate one that we have to use. What will wrong if someone who doesn’t know repeats at one class; nothing! And even those adolescents has to be learned all about nutrition by incorporating it in to the education policy as I told you earlier; if so, they will not cause adversity to let them understand during the adolescence period; they will understand it well. Thus during adolescence, when you advise them, it will be like review to them; that will be about provision of direction to what they have been already taught about family planning, birth spacing, and nutrition; so, if it continues like its present situation, it will be a failure; if it is done better, it will be success.

**I: Thank you so much; in addition to the detailed data; I have learned a lot from you.**

**P:** Thank you too.

**Summary (Home take messages)**

**Section 1: Common maternal (PW, nutrition problems in the community)**

Stunting is more at females; in my opinion, it is in the nutritional practice problem; that for example, in the case of husband feeding, it is not totally destroyed; just minimized; for example, if a food is served at home, the mother says I am at home I don’t go nowhere; for example, if there are only two “Injeras” at home, that one Injera will be given to the husband because he will out for business and that another Injera will be given to the children as they will go to school; so if she is invited to join, she will say no problem I am at home I don’t go anywhere…

**Section 2: Nutrition priorities in the woreda**

Of the nutrition problems is that how much times does a woman eat before her pregnancy; what if it was once per a day; are we saying she has to eat two times per a day then; so, this is mean to it is accepted by the government and then we are using this principle; or are we sure that the community is eating three times a day then it is undeniable to talk about the additional diets; this is for your consumption and research that I am talking indeed! The same is during lactation, it is said that she has to increase two times per a day; how much times was she eating during pregnancy is the concern then.

**Section 3: Nutrition interventions that improve adolescent and maternal health**

The production of that bar of salts are increasing but that of iodized salt is decreasing in its availability and quality; even the one which is available is fake; which may not be appropriately produced.

**Section 4: Implementation challenges and community factors affecting access to nutrition interventions**

In the quality aspect, it is not done to the fullest; when I say this, for example, the successful is intervention that we said is ANC; but there are still gaps that are not delivered to the mother when she comes in need of that service; then reduce its quality; for example, she would have checked for syphilis case at her first visit just to continue the next three visits properly; but not, because, there is shortage of man power; there are no materials for diagnosing that syphilis; thirdly the interruption of electricity which may distort the reagent for test; thus, though there may present professional and reagent; if no light, the mother will not get that service; and again there may present that light and professional but if on reagent, the mother will not get the service; so, she doesn’t get the service to its 100% at any time. So, if this much is missed in the service that we said it the most successful intervention; what will you imagine in the programs that we are not working them successfully!?

**Section 5: Multi-sectoral collaboration to improve maternal nutrition**

In addition to the other sectors collaboration, political leaders have to be involved which can then see and lead the specific activities allocated to the sectors either they are achieved or not.

**Section 6: Other intervention that influence adolescent and maternal nutrition and health outcomes**

Early marriage can causes her to have poor bond to her spouse as she may think about the interrupted aim; so, this in turn causes her not to eat properly and not to have proper dietary practice due to that disturbed life.

**Section 7: additional remarks**

As a policy challenge, in the case of quality education, in my opinion, the government is not moving appropriately; especially since the previous two years and like that, there is no quality education; it is only being talked as quality education; but no quality education is present!
