# Supplementary material for: Barriers to utilize nutrition interventions among lactating women in rural communities of Tigray, northern Ethiopia: An exploratory study
Source: PLoS One. 2021 Apr 30;16(4):e0250696. doi: 10.1371/journal.pone.0250696 (PMC8087028; doi:10.1371/journal.pone.0250696)
Supplement: S2 File — (ZIP) [file pone.0250696.s002.zip › S2_File.Doc/Woreda level and above key informants/073_IDI_ depeauty head for Woreda health office_Samre woreda.docx]

Date:13/11/2017

IDI with WHO at Samre distrcit

Name of KT: Nebiyat Desu

Place : WHOFFICE

POSITION: Vice head

Education: BSc

Experience: 3 months as vice head but 1 year of service

Interviewer: Beyene

**Section one**

I:Thank you for being voluntary for the interview. the first point of discussion is what are the common forms of malnutrition in Samre area?

P: yes among women, particularly in pregnant and lactating women there are several problems related to malnutrition. we conduct a screening every month and for those whose MUAC below 21. when we look at this , more women are affected by goiter/iodine deficiency.

I: is it common here

P: Yes it is common

I: why is that goiter common here. is it related to the non use goiter?

P: This area is mostly high land. this why they are affected.

I: the coverage of iodine use is 96%. It one component of the 16 health extension packages and health education is given.

I: What about the sever forms of malnutrition such as marasmus and kwashiorkor in this area.

P: It is better in this area

I: Is these condition happen in children of this area

P: It is not commonly seen. we measure them, if their MUAC is below 11 they will be linked to OTP, TFU.

I: Are there this kind of cases? i mean marasimic of kwashiorkor children who are managed at health facility

P: It is better

I: You can tell me by comparing different Tabias

P: When we se at the TFU it is zero. There are children under OTP in somes Tabias. It is zero in some of them. one or two cases in a month

I: What will be done in OTP?

P: OTP; their MUAC will be measured and if they are found less than 11, they will be provided 14 plampnet to be taken twice a day for seven days. they will be under follow up to see for complications. if they develop complications, they will be admitted and be given amoxicillin. Otherwise with OTP; they will take it at home. They will come to the health facility after seven days.

I: What about the TFC that you mentioned before. where are these center?

P; We have three; at Adi-Kaela, Gijet and Samre. Adi-kalela is new. it is started with the ART service. Gijet and Samre are the old centers.

I: How much are the number of case/ users of OTP. You will have a report about that

P: Yes, OTP, in Samre it is zero. we consider there is better feeding. from the other Tabias there are few cases which come rarely. In 2009/last year there were three cases from the TFC. They were transferred out to Mekelle. the same is similar at in Gijet. Majority will be treated with with OTP.

I; Is the number of cases managed by OTP high?

P: It is fine, it is moderate, during screening we only find two or three who have sever forms

I: Is there a means to screen malnutrition among pregnant, lactating women and adolescents.

P: There was what we call EOS(Enhanced outreach service), then screening of women, deworming and vitamin A were given. this was done once every six months. then it is changed to CHD which is made every three months. Deworming was given after 12 months but in CHD it is changed to 24 months. it was done every three month in the form of campaign. The health workers together with health extension workers go to each Tabia/center.theywere providing screening service and vitamin A.

I: Was there a mobilization activities to gather the community to the center?

P: It is scheduled. They will be informed about it. in the last two or three years , it is changed to RHD(routine health day); it is done every day. Before it was every three months and it is called community health day. The health extension workers run it on day to day basis.

I: How is the Routine health day implemented. tell me about that?

P: It is done every three months. There it is changed to routine health day. it is already considered as daily task. They will be screened every day.

I: Where is the place?

P: At the health post and health center

I: What about the CHD

p: Every three months and done at Tabia. Now it become routine.

I: Do women get the screening services? It is commonly done for children but for the women is was not as such. do women get weight measurement to determine their nutritional status. How is their participation in the routine health days. and what challenges do you face in that regard?

P: Women are receiving the screening service using MUAC. If they are fine, they will go home but if they are found moderate of sever they will be given Fafa..many mothers are using this service. The challenge we face is mothers seek to use the food although they are fine. the same is true in children. They brought to us assuming he lost his weight. Then when we say he is fine. they complain as if we do not want to give them. The mothers themselves also have similar complain.

I: is there a weighing scale at the health post

P: Yes we have MUAC and weighing scale. they measure and mange them. i can say it good in this regard,

I: Well, can you tell me more about the micronutrient deficiencies in the this area. You have mentioned about goiter

P: There is anemia. pregnant women came to us in anemic state since there are is malaria endemic.

I: Is malaria distribution high?

P: yes it is high

I: How is ITN use?

P: The use is good but they use it for other purpose after few time አድጊ መፅዐኒ ይገብርዎ

I: How is ITN distribution

P: In the high lands we will give to the priority y Tabia but in the lowland there is 100% ITN coverage

I: Do you mean all household heads have ITN

P: yes

I: Is it one per household

P: If their six two and below we will give them 1

: How is Vitamin A deficiency

P: It is fine, it rarely happen.

I: What about the chronic forms of malnutrition such as stunting, wasting and underweight

P: They are not as such common. It is not visible.

I: are there Short or very thin children?

P: It is rare

I: How do know this? do you have a mechanism to check these conditions

P: The health extension workers did this to rule our malnutrition.

I: Do the health extension have the skill to identify malnutrition by measuring weight and height. it has its own diagnostic criteria using the WHO graph, you can determine by comparing his height, weight and age . Do they have this skill practically. do they have the materials and is the condition convenient to do so? they have several duties focused on prevention. can they get time to screen?

P: They are better. they are trained. they are treating infant before 2 months. they can detect malnutrition.

I: I guess they can detect in children but can they do for women

P:Yes they can do but there could be fluctuation in the measurement. the is measurement error. they might make it mild, moderate or severe by mistake. This is could happen rarely. It is can be taken as good.

I: You know malnutrition is not only mean under nutrition. over nutrition is also a malnutrition. how is the situation of overweight here. Is this problem seen in children and lactating?

P: The community can be categorized as moderate. in terms of overweight and underweight

I: How common are communicable and non communicable disease in this area? particularly those that are related to diet such as hypertension, diabetes.

P: hypertension and diabetes we have some cases

I: How are their magnitude is there a tendency of increasing from time to time during your stay here

P: It is fine. There are people who do not get screened. it varies some time it becomes high and sometimes none

I: Up on checking for malnutrition those with malnutrition might have superimposed infection. there are several patients attending health facility. do you check for malnutrition at OPD? For example a patient with malaria who came to get treatment, did you assess him for malnutrition?

P: We do not do this. Particularly in adults we did not assess for malaria if they come for malnutrition.

I: How do you see this. Infection can lead to malnutrition. so, for every patient who came for treatment, what if they are checked their nutritional status on top of providing treatment

P: It is good. we were taking abut it always. particularly for male. for women they will be assessed when she get pregnancy and they do have monthly follow up. We need to work on the other group. There are things to do

I: How is the food security of this area? is the food produced sufficient for their consumption

P: It is good. the rural and agricultural office follows this issue. the areas is mostly lowland. they are food in secured. their crop is sorghum. There is no Teff. when there is good rain in the summer, they will get better sorghum product.

I: It only the sorghum they they can produce, so how can you advice them about food diversification? don't you think this will be problematic if only one type food is produced?

P:'Yes, when we teach them to sell half of their product and buy another crop and mix them to make the flour

I: Do they do actually. our society is more prefer to stay longer period by eating what they have produced than selling some and buying another food with better quality. How do you see this?

P: There is problem. but there is some improvement. sorghum alone can be used to make Injera. in this areas they buy red teff and mix is with the sorghum they have for consumption.

I: From where did they buy

P: There are some lowland areas which produce Teff. For example Finarwa, the areas is dry. hence, they grow ground net. Their cost at the market are better. they will sell it and buy teff

I: How do you evaluate the food security of the area

P: It is seasonal. this year we visit one village "Abiyi adi shushay", they are affected by drought. they come for complain. they have corrected together with agriculture.

I: What do you do when there is drought?

P: We work with agriculture. agriculture deals more on this area. They go for emergency aid.

[Office telephone call]

I: Well we were talking about what will be done during drought

P: There is emergency aid. there is also support from CC(community conference) operating at Tabia level. They collect money and support for those who face a problem.

I: Continue, what they did.

P: emergency aid will be given and money will be provided from the collected

I: From the different forms of malnutrition we mentioned before, which once are the main problems in your area; stunting, wasting, micronutrient deficiencies and macro nutrient deficiencies such as marasmus and Kwashiorkor. Particularly in women

P: As to my observation in women, women are affected more by goiter and anemia in pregnant as i told you related to being malaria endemic are and the pregnancy effect

I: Have you ever see stunted women

P: It is rare

**Section two**

I; I will go to the second idea, what did you do to improve the nutrition of women and adolescents as an office

P: In the area on maternal health we work on immunization particularly TT for women

I: Tell me the reason for working on these interventions?

P: Because it is given to prevent tetanus for herself and the baby. she is usually exposed to sharp objects and this may cause tetanus to her. hence, the vaccine will be given to her to prevent form tetanus. The non pregnant also get the vaccine from school

I: What else

P: We also work focusing on deliver and maternal and child feeding. we advice to give birth at health facility to prevent complications

I: What do you do in maternal nutrition

P; one we give health education, they will get screened for malnutrition and other illness

I: When do you give health education?

P: It is given routinely. we give them everywhere in health center, church or market

I: is it planned? health education is should be planned. the message should be appropriate to the audience et. is there a planned health education for women

P: Yes we have a monthly meeting of pregnant women. They make coffee together.

I: Who is leading the meeting

P: if it is at health center the MCH focal persons, if it is at Tabia, the health extension worker. They will be given about birth preparedness, breastfeeding, nutrition, EPI

I: there several interventions to improve women nutrition. of course we will see it later. but how do you see the participation of your office in these interventions?

P: what the health office can do is that coordinating the NGOs. World food program is supporting women by providing Fafa. We can do mobilizations, Monthly follow up and report can be done by the office. we also facilitate the link of women with credit institutions like Relief society of Tigray. It is safety net. they will be involved in simple activities that can harm them, they will take their children to the work place and some who will care their children will be hired. They will work to their capacity and in the mean time they will care their children. they also get support for the food security office under agriculture and the relief society of Tigray.

I: What else? For those services you mentioned before about maternal nutrition, How is their implementation. what are the specific challenges there?

P: Regarding immunization among mothers, it is good they receive up to TT5 but in adolescents the coverage is low. only 46 % of girls get vaccinated. TT in non pregnant women is low.

I: Why it becomes low?

P: It is due to lack of awareness and again little attention is given by the health sector. It is not like maternal and child health services. It is not like other health extension packages. it might be due to their expectation getting the service during pregnancy. The adolescents do not have adequate awareness. Regarding screening it is good and about 85% of the women are screened.

I: Is there screening for school girl age 10-19 and lactating women. Do you have this kind of service for these groups. for pregnant women there is a service to be given during ANC.

P: The lactating women are better than adolescents. They get some services during post natal . but in school this kind of service lacks .

I:What specific services do lactating women receive

P: All women during first visit will be checked for anemia and they will take Iron regardless of whether she is anemic or not, she will be measured her height and weight and educate how she should be fed. she will also be informed how to decease weight. the pregnant are better. there s waiting room for those who come from far. They will stay to see for any complications. I can say it fine in this regard.

I: Do you provide advice about extra meal and how they should fed themselves to pregnant and lactating women. you told me that they will get screening service.

P: yes if she is a pregnant, she will take one extra meal from the normal. if she is lactating, since she producing milk she will take two extra meal for the normal. they are getting counseling by professionals.

I: Did you advice about food diversity. i want you to tell me in detail what you are doing for the mother. As seen from studies, most women get first screening, take Iron folic acid, measure her blood pressure, and some blood test. But do really they get checked for their nutritional status and get proper advise how she can prepare food. how many times should she eat.

P: yes during the distribution of Fafa, the health extension workers demonstrate how to prepare some foods. there is some interruption but at the health center there is demonstration. There a kind a Mash called "Habeney"(the name given to the type of Mash). This is prepared locally and it available in health centers. They did food demonstration.

I: Who did the demonstration. Is the health extension worker did this?

P: The health extension workers, the pregnant women and some women development army work together. But there is interruption and it is not complete at all.

I: can you tell me more about the use of iodine. Is there ant message given to them about this?

P: Yes, during food demonstration we teach them about iodine use . iodine salt is one of the 16 components of health extension package. everyone is aware of this.

I: You told me that goiter and anemia are common in this area. Is the education address these issues. Are anemia and goiter included in the message?

P: Yes the give health education always during market day. there will some gaps in the utilization of iodized salt, there are some who use non iodized salt.

I: where did they get the non iodized salt? Is it available at market?

P: They commonly use for making the pepper flour. They manly use for cattle. it is sold in market.

I: Well you raise important point which i did see before. they add salt to pepper and the type od salt is non iodized one. so do you think that this would have effect on the its use .

P:Yes of course that add to the pepper and to avoid the risk they add iodized salt on top of it. It is not possible to say they all are using. In some places it is not accessible. The shop will be too far.

I: is goiter in children?

P: it is not

I: You know why people are using the salt. it is give taste to the stew. It is to avoid the water test. So, it they add to the pepper at the beginning and if that amount gives the desired test to the stew. i guess if they are not to add additional iodine salt to the stew ass using that it is enough feel that a kind of education to correct this think is important How do you see this?

P: Yes it is important because they are using the non iodized salt to increase the volume of the pepper they are preparing.

I: Is the cost of iodized salt affordable for them

P: It is lack of awareness. they can afford it.

I: How is the safety net program in this area. in other places we heard that there is consideration for pregnant and lactating women. they do not allow them to work. How is it here?

P: From the time that pregnancy is noticed Until one year after delivery they should not work.

I: What did you do about sanitation for mothers.

[phone call]

I: What kind of service are given to mothers

P: Sanitation is also one component the package. they provide health education about personal and environmental health . there is also education about toilet by health extension worker and women development army. particularly the exception WDA did best on this.

I: I were asking mentioning the service. what other service you can mention? let me add more; what about deworming?

P: we were providing deworming for lactating women but recently as per the letter form the region, we are not giving to lactating. it is not as such important for them unless they have the disease. It can be given only as therapeutic. We treat them by albendazole. we also provide deworming for children age 24 months to 59 months. Vitamin A fro 6 months to 29 months

I: What about for lactating

P: It is better in lactating. mainly it is given therapeutic

I: Do pregnant and lactating women actually take deworming service similar to iron folic acid

P: You will give them as therapeutic

I: Is there targeted supplementary feeding. For example when the women who undergo screening found some deficiency, do you give foods like Fafa

P: Yes we provided them through the support from world food program and world vision. The world food program is supporting us till now but world vision is not currently.

I: how do they support you?

P: They were directly providing the support to health center.

I: Did the support reach to mothers?

P: yes but the problem is that there is measurement error. the is gap.

I: which one the problem; is it classifying the normal as abnormal or abnormal as normal

P: mostly the problem is classifying the normal as abnormal

I: Is there youth friendly service?

P:Yes, it is available in all health centers. they provide services targeting the youth such as family planning, nutrition, physical activity

I: How do you reach the adolescents out of school and provide the youth friendly services

P: yes, they do have representatives in the youth friendly service. They are receiving the services. of course there will some gaps in addressing the whole.

**section three**

I: From the services that we were mentioning which once do you think are very important and needs much attention for pregnant, lactating and adolescents.

P: Strengthening the youth friendly service is essential because the youth can teach his family. then distribution of Fafa. there is interruption on that. we appoint them monthly. They come but return with our getting the Fafa. The world food Program shall make necessary follow up and provide the support uninterrupted. the other services are fine. they are being adopted by the community.

I: Which one of these services do you think are effectively implemented

P: One is the first antennal is attended well but the fourth is very low when we evaluate as a Woreda, region or nation. The other we also good in terms of immunization and deworming for children and vitamin A

I: What about for in school adolescents? you have mentioned that there is no service in school. P: P:There is gap in that area particularly on TT

I: Do you have service that you provide for out of school adolescents. is there a system that address this group

P: There is gap on that aspect. we do not assume they are getting health services

I: Well, what are the community factors that affect the nutrition of women?

P: One is their income. Again lack of awareness

I: How do income and awareness affect

P: having large family will cause difficulty to provide enough food for the family, particularly during drought time. The mother cannot eat well even at the time of pregnancy. in rural priority is given to children and men. This mother will be exposed to malnutrition and other illness. their awareness also contribute for malnutrition. Although they have good income they won't use it.

I: How do the education status of the women affect malnutrition. How is their educational status.

P:Majority are illiterates. now a days there are mother who complete grade ten. This will affect their understanding and acceptance of services. there problem in implementing the recommendation.

I: Do mother know the service they you provide to them

P: Yes they know, the problem is with implementing it.

I: What beliefs in the community can affect the use of nutrition service of women. these factors can hinder the use of nutrition services

P: From culture, fasting is one factor. there is also food restriction. it is not fully addressed

I: What are the foods that are not allowed for women.

P: I do not know much. but there are some foods restricted for pregnant and lactating women. women themselves even they need to practice fasting at the time of pregnancy.

I: How the situation of transport for women

P: This is our main problem. it is very wide district. women travel 4 to 8 hours on foot. due to this they miss some of the essential services. we want them to stay here at the waiting room. some men are problematic. they say how she come to health facility leaving her children. i get about three cases and brought myself justifying the impact of the maternal death to the children.

There transportation problem in mothers

I: Can they get the service in the nearby area such as health post.

P: They can get but the Tabias in this area are too far from the health post. They can not get the service as they need.

I: How do you see the quality of services regarding nutrition and how is the commitment of health workers

P: There is conference of pregnant women once a month. These women development army are trained about family planning, nutrition. they are part on the conference. They share the information they have when we come to the drugs, there is problem is visiting health facility on time. some come to health facility at the time when they are supposed to make fourth visit. They come at six month. They took iron at six months.

I: There is an established network to household level. they know each other. so if there pregnant women somewhere, is that not possible to take essential drugs to where they are. Can't the health extension worker provide the check up service at their home? Their main role to go house to house not only to remain at the health post. How is this condition

P: of course they go home to home. They went home to home during outbreak and during post natal. they did not go for checking ANC. There is no complete follow up to pregnant women and letting them to visit four times.

**section four**

I:what are the problems that hinder for providing service to maternal health services

P: Income, awareness and transport

I: Do men provide support to their wife?

P: Not all but religious leaders are getting training because of their influence of feeding and family planning. some did not support while some provide support

I: What can it be solved the problems mentioned

P: Transportation, strengthening development army and bring the mother to health facility if transport access is not possible.

I: What about their income

P: I do not have say on income

**section five**

I: Well how did you work with other sectors regarding nutrition, How is your participation. what do you do as a team

P: As a health sector, we identify pregnant women and link them with relief society of Tigray and agricultural food security offices. We involve pregnant and lactating women in safety net program. They will be told what to do. the health extension workers screen them.

I; What other sectors are working with you other than agriculture and relief society of Tigray

P: we all work together, we share report about malnourished children in OTP with food security every week but we did not sit together and evaluate our work. There is gap on this area

I: Do you have any job with school

P: It is not strong

I: What should be done by each sector to improve these services?

P: Currently to have strong and coordinated work we do not need anything. we have to be strong and evaluate our job

I: Who should be involved

P: Water resource, agriculture, education, health. i think social affairs should also be part of he team. women affair.

I: Who is coordinating body

P: Yes the food security office. they have taken training about nutrition and involving women in safety net. But there is gap here

I: can i say there is a team which formally talks about nutrition of women in Samre?

P: You cannot say confidently. There is a team but it lack working together. we only meet weekly with food security office. there is no formal meeting among sectors and evaluating activities.

I: Do you think this possible to do?

P: Yes but the offices are overloaded. that is why they fail to meet regularly

I: This is the last question; it is about early marriage and birth spacing. what do you do on this area. how can it influence the nutrition of the women.

P: About early marriage. There is marriage committee at Tabia level. they will see each case and she will marry after approval. health is one of the members in the committee. There exceptional people. They force her to army intentionally while she is under age out of law. They will acused and punished. the marriage committee is strong

I: Who are the committee members?

P: Women affair, health, administrator, the kebele leader

I: This could be in Samre(name of the district). what about in the Tabias

P: I told the kebele level. i were in "Finarwa"(Name of the kebele). this committee exists there. several girl come to the committee and be evaluated and if the qualify the will marry

I: you said there people who marry the girl in hidden manner

P; Yes. this happens, the girl will be exposed to Fistula. as a Woreda this will cause a problem to herself. she is kid and she is going to have a child. it is difficult for her and it will affect her feeding. If she give birth at optimum interval the children will get sufficient and good food. if there is spacing, they be fine. otherwise they will be exposed to malnutrition

I: What did you do to delay birth

P: We work to create awareness. the focus areas are, family planning to increase birth interval. some women say "Saint marry will allow me to get pregnant every two years. we encounter this kind of cases.

I: Did that happen in realty

P: Yes some time it happens and sometimes not

I: Do you have additional points that you want say to avoid this practices?

P: To avoid early marriage. there must be awareness about it. some parents said" think that she will be rude and that is why i forced to get married her". we need to create awareness on this regard. another job creation. the job will make them busy.

I: What can be done at school

P: In school there are clever female students who are forced to be out of school because of marriage. There were such cases where a marriage is canceled. This students should well informed so that they can influence their parents

**section six**

I: So as last point, if the feeding of women should be improved what do learn from your experience and recommend to be improved

P: One if multi sectoral is strengthened we can do great job. this sectors do have huge arm under them. it they collaborate and reach every women and if the same collaboration goes dowm it can solve the problems

I:What else other that what you mentioned

P: what I can add is the pregnant women are taking Fafa from health center coming from far place. they are asking us why the Fafa is not given to us from our Tabia. they are taking it from health post as well but there still far from the health posts. whyn they did not come to us. They asked to us.

I: Can vehicle reach to their area.

P:In some of the Tabias it can reach

I: we have made long interview. i have learnt a lot. thank you. i have finished

P: Thank you too

**Summary**
