# Supplementary material for: Barriers to utilize nutrition interventions among lactating women in rural communities of Tigray, northern Ethiopia: An exploratory study
Source: PLoS One. 2021 Apr 30;16(4):e0250696. doi: 10.1371/journal.pone.0250696 (PMC8087028; doi:10.1371/journal.pone.0250696)
Supplement: S2 File — (ZIP) [file pone.0250696.s002.zip › S2_File.Doc/Woreda level and above key informants/074_IDI_Water Resource Office_Tanqua Abergele woreda.docx]

**Tanqua Abergele Woreda – Water Resource Office KII**

| **Introduction:**  Hello, my name is **Dejen Yemane**. I am from Mekelle University. Thank you for taking the time to speak with me today. We are doing research on the factors that influence the nutrition of mothers and adolescents in collaboration with the Regional Health Bureau and UNICEF. Your participation is very valuable. The things that you tell us will be used to improve nutrition programs and services for women in the region and the country. We will not share your names when we report our results.  However, I will record the discussion so that I can capture all the ideas that are shared. I have several questions to ask you that I have prepared in advance, and I will ask you to say what you think about each question. The interview will last for 1:30 -2:00 hours. Do you have any questions before we begin? If you think of any questions as we proceed, please feel free to let me know. If it is all right with you, I will turn on the tape recorder now.  **Section A: Interview details**   1. Zone: **Central** 2. Woreda: **Tanqua Abergele** 3. Kebele: **Meare** 4. Name of key informant: **Aregawi G/kirstos** 5. Institution of key informant: **Water resource, Mining and Energy Office** 6. Interviewer name: **Dejen Yemane** 7. Date of interview: **November 13, 2017** 8. Interview start time: _________________________ 9. Interview end time: __________________________ |
| --- |
| **Section B: Interviewee professional information**   1. Gender    1. Female    2. **Male** 2. Age: _________ yrs 3. Highest level of completed education.    1. No formal education    2. Primary education    3. High school    4. College education    5. **Bachelor degree**    6. Master’s degree 4. Current job/position: **Deputy head of Water resource, Mining and Energy Office** 5. How long have you been in the current job/position?    1. ______ Months    2. **One and half** Years |

**Section 1: Common maternal (pregnant women, lactating women and adolescent girls) nutrition problems in the community.**

**Interviewer: In your opinion, what are the common nutrition problems in the community for women? What about for adolescent girls?**

**Participant:** okay, of course my field is not related to health. However, it is possible to reflect from observations. From my observation, when I compare children grown in a Woreda where I grow and in this community, I think they have growth differences. This is to mean that from childhood to adolescent age (up to 18 years), growth of both male and female is different. For example, if you take Degua Temben Woreda, where I was born and grown, growth of children is fast, and they are physically fit. Whereas, in Tanqua Abergele Woreda, children are stunted and wasted.

**Interviewer: why are children of this Woreda stunted and wasted?**

**Participant:** it has many reasons. First the area is lowland, and it is prone to malaria and exposure high sun intensity. Secondly, when I relate my profession, the soil is not fertile compared to highland areas.

**Interviewer: what is your parameter to say it is not fertile?** **Does it have any reason with your profession?**

**Participant:** related with my field, geology, if you take areas like Degua Temben Woreda from Enda-Mariam to Alaasa and in Southern Zone of Tigray around Raya and Maychew, it is rich in minerals because the soil is made up of igneous and basalt terrain. So, the fertility of the soil is high and if you feed on foods grown on these areas it is full of minerals and it is obvious that it will support the growth and development of the body. Whereas in Tanqua Abergele Woreda, they produce sorghum and as to my understanding this is only rich in carbohydrates. In addition, Tanqua Abergele Woreda, is rich in animal resource. It is ranked in the forefront by animal resources. However, because the community are lowlanders they did not adapt quickly to the technology and global market. So, when you compare them with the other areas they are more stunted and wasted.

**Interviewer: For example, you told me that the area is rich in animal resources. If that is so, animal source foods are accessible and scientifically these foods have high quality protein and micronutrient that are essential for healthy growth and development. so, what could be the reason for being stunted and wasted?**

**Participant:** yes, it is right that if they feed animal source foods, you expect they will grow healthy, but this are not enough. Because there is crop variety from area to area. For example, teff growing at highland and teff growing in this area are different in content. Similarly, they don’t produce more wheat, barley, and legumes compared to other areas. They can get it from market, but they wouldn’t get diversified foods like areas who produce variety of crops. In addition, the area is not yet irrigation user. May be from my perspective, I believe that from the six food staffs, most of them are missed.

**Interviewer: just I have a follow up question. You were comparing highland verses lowland and you told me that in lowlands they might not get the required minerals due to infertility of the soil. In relation to that tell me the common health problems associated with nutrition in you Woreda. When you tell me the problems try to give me the difference between male vs female, pregnant vs lactating, pregnant vs adolescent, illiterate vs literate, rich and poor.**

**Participant:** it is clear. If you compare nutritional problems in those who have and who don’t and being male and female, there is a difference. Those who don’t have, and male are more affected by undernutrition. Because if the household is rich, even they did not produce it, they can buy from market. If you see in this area communities who are near to urban have better nutrition when compared to those who are living in rural areas.

**Interviewer: what could be the reason?**

**Participant:** the reason could be, awareness of the community, but in rural households are not interested to leave their birth places. This time there are much improvements. They enroll their children to school at their right age. The government is strictly following, and the community are also sending their child to school accepting the importance of education. Most of the time, instead of farming they lead their life by livestock breeding. But they are deceived by their quantity without quality. So, they are nomads. Since the area is frequently affected by drought, they practice a limited kind of agriculture, moving their herds periodically from place to place in search of water and pasture.

**Interviewer: how frequent does a drought happen in this community?**

**Participant:** out of the 4 years since I have been here, the three years were drought years. Except the 2008 E.C worst drought the rest were similar. Overall, drought occur every year in this community and as a result the productivity is decreasing. So, there is food insecurity. You can check that in majority of the schools there is school feeding (FAFA) supported by NGOs.

**Interviewer: in how many of the schools did you implement school feeding program?**

**Participant:** maybe it is challenging for me to know the number and I may not support it by data, but parallel with the text books there is FAFA and oil. Due to these supports students did not drop out of school because of food compared to other areas. To avoid drop outs there are number of governmental supports.

In addition, stunting and wasting is different when you compare male with female.

**Interviewer: whom do you think are more stunted and wasted?**

**Participant:** male is more stunted than female, because female is relatively better. Though it is difficult to present scientific justifications, female grow in a faster rate than male. But due to workload males are more stunted and wasted. Because families’ handover their agricultural activities to those kids. But females have also their own role in the agricultural activities. Even the household heads are not active participants in agricultural activities. Because the kid will hand over their activities. The other issue is, like highlands there is no preparing your farmland for future production?

The main reason is some of them are diseased like malaria, but now the government has controlled it by distributing ITN and draining stagnant water.

**Interviewer: do they properly utilize ITN?**

**Participant:** the utilization is seasonal and lacks consistency.

**Interviewer: what could be the reason for the inconsistency?**

**Participant:** it is lack of awareness and since the area is arid it is difficult to be covered. Because it is hot. But the ability of ITN to kill other insects the community understands its importance. In some areas they use it to cover grasses but due to frequent health education on the importance and its cost there are improvements from time to time.

**Interviewer: to whom did they mostly give priority at a household when they use ITN? To child, adolescent, pregnant, lactating or to the husbands?**

**Participant:** yes, this can vary from area to area but generally speaking there is good awareness. Because there are two HEWs per kebelle and the Woreda administrative also working focusing on health, education, water and agriculture. As a result of consistent monitoring and evaluation by the Woreda the is rise of awareness in the community from time to time. The community gives priority to children and pregnant women. Due to the advices and concealing given by health professionals home delivery is almost none, although there are some.

**Interviewer: what is the reason for home delivery?**

**Participant:** as a Woreda there was a challenge in the religious leaders due to lack of awareness.

**Interviewer: could you give me example?**

**Participant:** For example, there was a tradition that priest’s wife should not uncovered and touched by health professional. In addition, pregnant women also feel humiliated when they assisted by men on delivery. They also believe that everything is God’s will and Saint Marry will help them. But now they understand that next to God, science has its own contribution in saving their lives and every woman is properly getting the health services.

**Interviewer: How did you solve such kind of problem?**

**Participant:** we solved it by organizing frequent conversations with the religious leaders by giving them examples. After frequent discussions there were early adaptors who served as models. In addition, women were giving frequent health education to decide on their health and utilize health services given by HEWs free of husband influence. Now they deliver at health institutions and get ANC and PNC services by discussing with their husbands.

**Interviewer: What type of services do pregnant, lactating and adolescents get at HF?**

**Participant:** our Woreda is included in the Sokota Declaration and Sustainable Undernutrition Reduction in Ethiopia (SURE) project.

**Interviewer: why is your Woreda included in Sokota Declaration and Sustainable Undernutrition Reduction in Ethiopia (SURE) project?**

**Participant:** the reason why our Woreda is included in Sokota Declaration and Sustainable Undernutrition Reduction in Ethiopia (SURE) project is because there is high prevalence of stunting. This is based on evidence. Though I don’t have the data at hand you can get it from health center. This shows how stunting looks nationally, regionally and specifically as a Woreda. Now our community also started comparing children who get adequate food with children who didn’t get adequate food, what to feed before and during pregnancy, where to deliver. They have also started to use animal and plant source foods. For example, in this area instead of using animal source foods they prefer to sell animals to get income.

**Interviewer: Many small and medium farmers have animals and they have considerable income through production and marketing of animals and their products, but still there is high prevalence of stunting and wasting in this area. Do families buy food items from market after they get income by selling their animals and animal products? In addition, tell me your stand on which pathway is good for nutrition, income or consumption pathway?**

**Participant:** it is good idea. Stunting and wasting are becoming alleviated in our community these times. Most of the households are pastoral instead of farming they bread herds. For example, there are kebelles in this Woreda like Felegehiwot kebelle which don’t have water resource except one water source. Recognizing the problem, government gave them an alternative to reside in a kebelle of their choice, just a kind of villagization. But since they have livestock resources they fear lack of grazing land and the community rejected this idea. Because they prefer to have wide grazing land than farming land. In this area people need to be famous by the number of goats they have. They believe on the number of animals they have but they are not quality breads compared to other areas. Especially in the year 2008 E.C, due to drought animal price was reduced. In this area except in holydays, the animal is dying or old enough they don’t eat meat whereas an employee eats even out of holydays. Coming to the income vs consumption pathways, people around this area enjoy investing their money on ceremonies like memorial of their family died 10 or 15 years ago instead of buying diversified foods. This is not the problem of this community alone, rather it is the problem of the region. In our region people face food shortage not only due to poor production rather due to excessive wastage of food in ceremonies like wedding and baptism. For example, in the year 2007/8 E.C, there was surplus production but due to unplanned ceremonies the community faced food shortage in the drought season. Since sorghum is at their hand, instead of buying cloths, teff, and other cereals, they invest their money on ceremonies for celebrity. Due to health education urban residents are better in this regard compared to rural residents.

**Interviewer: while the resources are at the hand of the rural residents compared to urban, what do you is nutritional status become worst in rural areas?**

**Participant:** though availability and accessibility are one factor the bottom line is the awareness. If you didn’t utilize and manage what is at hand it is a problem by itself.

**Interviewer: where do you think is stunting and wasting more common even when you compare rural residents having many herds with few herds?**

**Participant:** it is common in the poor, the one who have few herds. If you have at hand though it depends on your awareness you will use it. For example, it is incomparable children of households having milk year-round and who don’t have.

**Interviewer: what are the common nutritional problems in pregnant, lactating and adolescents?**

**Participant:** since I am not health professional, it may challenge me to answering this question. You can identify well-nourished and malnourished women by looking on her face. Most of the time you see wasted pregnant and lactating women, because most of the time the mother performs most activities even during her pregnancy and lactation periods. For example, Vitamin A is given to children and lactating women in health facilities through governmental and nongovernmental aids.

**Interviewer: What additional services do pregnant and lactating women get at health facilities? (silence) for example do they get IFS, deworming, ANC, PNC…**

**Participant:** yes, they get IFS. For example, if a woman is bleeding, she will be provided blood supply.

**Interviewer: do you think nutritional problems like anemia, goiter and night blindness are common in our community?**

**Participant:** of course. The reason is, if you did not get proper nutrition you will not get essential nutrients for your body. For example, goiter is widely common in this community especially in women.

**Interviewer: from your observation, in which group of women does goiter occur prevalently?**

**Participant:** from my observation it is common in the elders than younger women. I don’t know, it could be the sorghum but there is also dental problem. They lose their teeth early.

**Interviewer: what could be the reason? What do communities say about dental problem?**

**Participant:** they call it መንቀርሳ but as to me it could be due to lack of dental hygiene and their feeding habit. Because they only fed sorghum. Head dandruff is common in children and this could be due to lack of personal hygiene. If there are such opportunistic infections, they will compute nutrients on top of the food insecurity and this will impair their growth.

**Interviewer: Do you think noncommunicable diseases are associated with our diet?**

**Participant:** may be with hygiene and sanitation, unless otherwise they are not associated with diet.

**Interviewer: I was asking on noncommunicable but tell me what types of communicable diseases can be transmitted because of poor personal hygiene?**

**Participant:** if food is not hygienically handled it can cause ameba and typhoid. If you eat food which is poorly handled and unhygienic you might be affected by intestinal infections. Though, children grown in urban is better in hygiene compared to children grown in rural areas, resistance to infection is better in rural children.

**Interviewer: why is resistance to infection is better in rural children than in urban?**

**Participant:** maybe it is because rural children are physically active than urban children. Because the rural child grows toddling in animal dungs and that exposure may help him/her to develop resistance. Whereas, urban child gets necessary care and when he/she exposed to infection, he/she will be affected easily. Overall, there are number of diseases associated with personal and food hygiene. For example, if we share blade, head dandruff can easily transmit. At the same time if you share cloths there is possibility of transmission of fungal diseases. Therefore, the transmission of such communicable diseases is aggravated due to carelessness and lack of personal hygiene.

**Interviewer: you told me about communicable diseases like typhoid and ameba, what about noncommunicable diseases related to diet like diabetes, hypertension, cancer and others.**

**Participant:** these diseases are traditionally called rich people diseases. These diseases are common in people who frequently eat sugary foods. But here in our community people eat roughage. Without exaggeration, what we eat here is just simply to fill our stomach, so it is less likely to see people with noncommunicable diseases. When we say it is likely it is to mean it is rear. Even traditionally our community avoids sugar to prevent hook worms. In our area the commonest disease is cerebral malaria.

**Interviewer: you told me that children do not increase their height/weight proportional to their age, what about in women/girls? Do women/girls in this community increase their weight/height proportional to their age? If not, what do you think on why women/girls in this community would not increase their height proportional to their age? Could it have relationship with their nutrition?**

**Participant:** from my observation, I was associating stunting and wasting with genetic makeup of your family and the environment where you live related with behavior and health. There is no gender disparity in nutrition both female and male eat together in the same plate. Regarding the question does stunting and wasting has a relationship with nutrition, yes, it is related. In men headed households, if the household head did not come home, the family including children and pregnant women will not eat rather they will wait him until he come. If in case the children and the pregnant is to eat, they will eat without sauce. Such traditional thinking is still persisting, though there is some improvement. There is a practice that quality food is given to husband and their son and wife with her daughters will eat the leftovers. Apart from the leftovers, what so ever the food is, men and sons eat first and wife with her daughters eat later. As a result, women/girls are more affected by wasting compared to men. Thus, women/girls’ immunity status will be diminished and easily affected by opportunistic infections like malaria.

**Interviewer: For which one of the health problems you mentioned do you think are pregnant women are especially at risk? What about the lactating women? What about the adolescent women?**

**Participant:** most of the time they are at risk of malaria. Even in the urban parts of the Woreda, regardless of their age and sex they are prone to cerebral malaria. Though there is reduction in malaria prevalence due to environmental management, insecticide spraying and ITN utilization, malaria is still a problem in elders. Especially cerebral malaria has long lasting effect. So, cerebral malaria is dominant in this area and the rest will come associated with it.

**Summary**

- Common pregnant/lactating women and adolescent girl’s nutrition problems in this community are goiter, anemia, stunting and wasting because the Woreda is prone to malaria, soil is not fertile, and it lacks crop variety compared to highland areas.
- Head dandruff, malaria, and AWD are the commonest communicable diseases whereas, cancer and dental problem in the woreda
- People from poor households, male sex and rural residents are more affected by undernutrition because of lack of awareness, workload, being pastoralists and their limited agricultural practice.
- Food insecurity happen almost every year in Tanqua Abergel Woreda.
- Unplanned investment on ceremonies lead the community to food shortage
- There is school feeding in this woreda and due to that students did not drop out of school.
- One of the challenges for institutional delivery was religious leaders due to lack of awareness
- The Woreda used early adaptors as change agents to change behavior of the community
- Though there is high animal resource, instead of using animal source foods they prefer to sell animals to get income.

**Section 2: Nutrition priorities in the Woreda**

**Interviewer: In your opinion, what priorities do your institution has in relation to maternal and adolescent health? Why?**

**Participant:**  as a water resource office, like other sectors we have direct and indirect activities on to improve maternal and adolescent health. Most of our activities have indirect effect. For example, if you take mineral, we give due emphasis to women empowerment that will contributes to improved nutrition. If you take the health sector, they have direct effect by providing services like vaccination and vitamin A supplementation. Whereas our institutions’ mission is to facilitate provision of clean water and women's empowerment which affects their income. So, most of our activities are related in empowering women. As stakeholders, our institution also participants on awareness creation activities. But most of our mission is related in empowering women through increasing their income.

In this regard we work to make women beneficiaries of irrigation/water conservation systems, for example, home gardening; mining; and access to clean water and energy. For example, women to beneficiary of renewable energy to prevent respiratory diseases affecting the lung we promote to introduce ‘Mirt Eton’ (ምርጥ እቶን) to avoid biomass that emit excess C_2_O. in the past we were using lamps but now we are encouraging them to use solar energy.

**Interviewer: What is the importance of using solar energy?**

**Participant:** it has three to four importance. First, it reduces carbon emission as part of the green economy policy of the country and this promotes the health of the community. even if you see cost wise, for example, a farmer can get solar light for several days at the cost of 250 birr. As you can see the area is endowed with 13-months sunshine including the summer season. So, they use solar energy for charging mobiles and lighting purpose. Compared to households using biomass energy, households using solar energy has better health benefits because those who use biomass energy will be exposed to trachoma and respiratory health problems due to carbon emission. We work on this regard through WDAs by convincing women to serve as models. So, women will not be affected by respiratory health problems if they use renewable energy sources. In addition, they are also contributing to the green economy strategy. As Africans we are more affected by drought though we don’t have industries contributing to carbon emission, rather the global carbon emission is significantly affecting our weather patterns (temperature, precipitation, or wind). That is why we are affected by El Nino and La Nina. As water resource office we are working on realizing green economy to promote health and reduce deforestation.

Specially in lowlands, fire wood was available everywhere in the past. But now you could not get even traveling long distance. Thus, our mothers are kidnapped while collecting fire woods. But now because of using renewable energy, pressure on mothers is reducing. As a result, their time and energy are saved, and this can be used in ensuring their health and nutrition. In the past mothers were responsible to grind, collect water and fire woods by themselves. Every work was performed by the mothers and husband only complain about not getting their food on time. But now days such attitudes are minimized due to continuous health education. Because instead of going long distance to collect fire wood there is solar energy at her disposal. For example, in area where there is no electricity they use solar energy. In the past, we were exposed to social, health and economic crises because of using imported fuel for lamps. But now our community has started using solar energy and as a result health is becoming ensured.

In addition, if the woman is irrigation beneficiary, there is higher probability of getting balanced diets because she will have vegetables and fruits. For example, in our Woreda there are organized women who produce mango, banana and papaya fruits.

With the water distribution and the involvement of the community in irrigation activities we need to work more especially in women participation. For example, our Woreda is divided in to three clusters namely Agbe cluster around Temben, Yechila cluster, and Gigiket cluster around Tekeze hydroelectric power. But the irrigation coverage is different from cluster to cluster and Agbe cluster has better irrigation coverage.

**Interviewer: what makes Agbe cluster to perform better? Is that due to water availability or mobilization?**

**Participant:** they have both. They have river of Gereb-Giba and youths from Eastern zone are also benefiting from this area. Now women are becoming productive by engaging in irrigation activities and are also supplying to market. Such women are becoming models and awardees. Down the river Gereb-Giba, due to reduction on the amount of water and poor extension activities, irrigation activities in Yechila cluster is poor.

In GTP-I as a water resource bureau and as a Woreda, we have a strategy to avail water sources at 1.5km radius. According to government strategies water resources must be led by women because women are responsible for collecting water. This is to strengthen better protection and care of water resources. Currently, in GTP-II we are working to avail water resources at every 1km radius considering per capita consumption for rural and urban areas. Therefore, there is condition where women go long distance to fetch water. So, because of access to safe drinking water close to their home, women are saving their time and energy.

**Interviewer: Do you think it is necessary for your institution to get involved in work aimed at improving nutrition among women and adolescents? Why?**

**Participant:** it is good. Be it as a Woreda or region, women accounts above 50% and any developmental activity can’t succeed without involving women. Not only because of their numbers, but we need to involve women. Coming to your question, it is mandatory to involve water resource office. Because water is life it is mandatory. Without water, it is impossible to think health, agriculture, education and other sectors. You can go for more than three weeks without food, but the maximum time an individual can go without water is very shorter.

From our experience, when we involve women, they will be benefited and at the same time our energy, mineral and water activities become successful. So, we have learned that without involving women, leave alone success it is impossible to march even.

**Interviewer: How do you evaluate the priority given for the interventions for the women?**

**Participant:**  comparing the number of female population and their participation, we are below expectation. For example, availing water in 1km radius is not yet achieved. Because as a Woreda we have water scarce kebelles, how to improve nutrition among women and adolescents leaving in such kebelles, how to benefit women who are not involved in irrigation activities are big questions that need answer and collaborative work. So, overall, we need to work more to benefit women by optimally utilizing available resources in the community.

In our mining activities, we aim to involve above 50% women. But since the identifying and organizing is out of our office’s mandate, fully involving women and following their performance is still lacking. Therefore, in this regard it is not satisfactory, and it needs extra work.

**Interviewer: what type of minerals do you have in your Woreda that can benefit and increase women’s income?**

**Participant:** for example, we have sand and gravel resource especially in Agbe cluster. We have many youth enterprises benefiting from this resource. The youths whom I was discussing before you were those enterprise members. In three months they earn more than 150,000 birrs. Of this, 150,000 birrs they are expected to pay only 3% tax whereas the rest 97% will be their profit. In such activity even, the women can do it because it is not labor intensive.

**Interviewer: how do you see their participation in the existing enterprises? Because it needs endurance.**

**Participant:** this is the prevailing attitude in the community that needs to be solved. But when you see water and soil conservation, women are frontline actors and they are equally participating for its success. And mining does not require much effort than water and soil conservation. Though there change in attitude in the women side. Now they believe that, they can improve their nutrition by participating in different income generating activities.

On top of sand we have gravel, and gold in our Woreda. In addition, we have requested to all universities in Tigray, except Raya University to study the mineral resources in our Woreda.

**Interviewer: I am happy with the information that I am getting, anyways, lets proceed to the next question.**

**Summary**

- Water resource office work to improve pregnant/lactating women and adolescent girl’s health and nutrition by increasing women participation on irrigation, home gardening and mining activities; availing water sources at 1.5km radius to minimize time spent and traveling distance to fetch water and availing clean and renewable energies that reduce carbon emission and respiratory health problems.
- Involving water resource office in work aimed at improving nutrition among women and adolescents is not only necessary rather it is mandatory
- Availing water in 1km radius is not yet achieved

**Section 3: Nutrition interventions that improve adolescent and maternal health**

**Interviewer: What kinds of nutrition interventions are in place to improve health of the pregnant to your level? Where do they get it? Who provide it? For example, services given at HF ranging from advices to curative activities.**

**Participant:** there are ideas sharpened from time to time by government and development partners. For example, there is at kebele level there is adult education especially for older women. This focus on performance based education on development packages. Thus, they will develop their literacy level and at the same time they will implement government directions like increasing the beneficence of women on irrigation, water, and other activities.

As a Woreda, 40% of the leadership is occupied by women. They are serving as office head and deputy heads and at the same time they are leading women affairs and league at kebelle level. Coming to the health issue, health services at kebelle level is given by HEWs. These HEWs are working day and night to raise awareness of women. They are mobilizing pregnant women to follow ANC and vaccinations. Because of their unreserved effort, the health seeking behavior of our mothers is increasing. They develop a trust that if they use health services they can minimize pain, morbidity and mortality. HEWs are also giving home-to home services and counselling.

**Interviewer: what type of counselling and advises dose a mother get?**

**Participant:** for example, they get advices to use ANC and PNC services. Related to nutrition, they advise them to properly fed in order to produce milk especially for breastfeeding mothers. In areas identified as having lack of awareness, even male health professionals are assigned to disseminate health information. Health center staffs identify areas with high wasting and stunting rate, low ANC and PNC follow up, etc. Once they identify their target groups the give advice on taking extra and diversified food, rest, and other services. They give personal and general advices at health center. On top of advices they administer Vitamin A supplementation and screen children and women. They also deliver FAFA to children and women identified with acute malnutrition during screening. But Plumpy Nut is given to children only. They also teach them how to prepare foods blending from cereals and legumes. The community is close to media.

**Interviewer: what type of media?**

**Participant:** in the rural area most of the community use Dimtsi Weyane Radio program where as in urban, Kana TV is dominating. But in rural areas, the community is hand and glove with Dimtsi Weyane Radio program.

**Interviewer:** **Does Dimtsi Weyane Radio program broadcast about nutrition?**

**Participant:** yes, but it is not only about nutrition, it also broadcasts about agriculture and WASH issues. They know even the schedule. If you have exposure to listen Dimtsi Weyane Radio program, majority of the participants on radio discussions are from rural areas. Almost all the community have radio and mobiles.

**Interviewer: you have mentioned mobile, in some areas we came to know that people were listening radio programs but this time they are not totally listening because they are listening to music in their mobiles. How do you see this, do the community here listen to radio programs?**

**Participant:** not only mobile, even the radios are with memory cards. It is an exaggeration if you say youths follow media. When we say they follow radio programs, it is for the farmers and war veterans. In such segment of the population, they have 2-3 radios. Some of them also use their mobiles to listen radio programs but this is not that much because of battery usage. The WDAs also transmit health information in the form of dramas.

**Interviewer: Do pregnant women get advice for the need to use iodized salt? Why? Do they properly utilize it because in some areas they still use the uniodized salt?**

**Participant:** yes, because it is lately introduced, we cannot say that it is fully utilized. Though iodized salt is better content wise the uniodized salt is better in test and because of that there are many households who use uniodized salt. In addition, the reason for using uniodized salt is it sourer than the iodized salt. These and other attitudinal problems were the barriers for iodized salt. But these times there is a shift towards iodized salt due to the consistent awareness creation by HEWs. In the past, every household was owning traditional grinding mill and when need arises they were grinding salt at home but this time since there is no traditional grinding mill they are obliged to grind in modern mills and instead they are using the iodized salt. Through time utilization of uniodized salt is becoming changed, but still there are communities who use it. The good thing is availability of iodized salt in the market is making the community to utilize it. On top of that they know when to add iodized salt during cooking because of the health information dissemination through HEWs and medias. To avoid loss of iodine due to food processing they add iodized salt

Especially in our Woreda it is important to use iodized salt because the soil is eroded due to deforestation and overgrazing which leads to increased loss of iodine from the soil. Though utilization of iodized salt is changing over time, but still there is utilization of uniodized salt. You can see uniodized salts in the market places and that is used for animal’s consumption. Especially in dregs they add uniodized salt to increase their feed consumption.

**Interviewer: Are pregnant women getting deworming services? How do they get it? who provide the service?**

**Participant:** the questions are difficult, but I know that there are vaccinations at school. Specifically, measles and TT vaccines are provided.

**Interviewer: In your opinion, are adolescent girls provided school feeding? Why is it necessary? Is there any such feeding interventions targeted to out-school adolescents?**

**Participant:** school feeding is not given at all schools. For example, there are 64 schools in our Woreda, and this all schools are not supported for school feeding. Be it government or development partners do help furniture and text books for some of the schools instead of school feeding. However, in the elementary schools having school feeding program they provide the service for all the students, regardless of sex, age, distance from where s/he came from. The importance of school feeding programs is to alleviate short-term hunger and helps children concentrate on their studies and enable them to gain increased cognition and better educational outcomes. But there is problem of adaptation to the foods by few students. Regarding out-school adolescents, I don’t know any intervention given for this segment of population.

**Interviewer: Is there a situation, which pregnant/lactating women and adolescent girls need to be eligible for Targeted supplementary feeding (TSF)? Why? Could you tell me specific examples?**

**Participants:** you better ask this question to the health sector. What I know is that there is counselling, and administration of vitamin A and iron folate supplementations. But I don’t know what pregnant/lactating women and adolescent girls need to be eligible for Targeted supplementary feeding.

**Interviewer: in our next question, I will ask you two questions. Are pregnant/lactating women and adolescent girl getting advice on nutrition sensitive agriculture such as home gardening to generate income and promote food diversification and security? What about on the need to be involved in safety net programs? Who provide them? What else interventions related to this?**

**Participant:** most of the questions are related to agriculture. Especially promoting women to participate in development activities is task of all sectors. To benefit women, there are plans how to empower women in livestock, irrigation and home gardening. For example, there are nutrition educations to reuse wastewater for home gardening and there are successful women who produce homestead food. Taking such women as models we educate the public to introduce home gardening. So, we promote women to take the lead and to improve their household decision making through homestead production activities. If the women are empowered men are beneficiaries. Because she is responsible for food preparation. Though there is still male dominancy there are many activities done to empower women by agriculture, women affairs and water resource offices. As a water resource we work on availing the supplies for example, the irrigation schemes. Whereas, women affairs office has a mission to empower women and they plan to empower women in collaboration with other sectors. So, to benefit women there are integration works. For adolescent girls, they have gardens for demonstration purposes in their schools that helps them to learning more about nutrition practically.

Regarding safety net program, there are two conditions in safety net program. First, household-level targeting focused on households with high levels of food insecurity that need emergency food aid related with drought. Though there are inconsistencies, women are beneficiaries from such programs. Secondly, vulnerable households who have a pregnant woman and with children under two years of age/lactating women are PSNP beneficiaries. The second one is done by health sector. Here the health sector identifies eligible pregnant and lactating women with malnutrition problems. Therefore, pregnant and lactating women are exempted from heavy workloads instead they will visit health facilities to get health services. Even in water and soil conservation activities, children are handled with care. In every catchment, pregnant and children are placed under shade and pregnant will take care of the children.

**Interviewer: Are pregnant/lactating women and adolescent girls getting advice on water, sanitation and hygiene services?**

**Participant:** yes, especially in sanitation we have WASH project. In addition, as a Woreda there is introduced system led by health sector. We are working to install sanitation and water facilities in every institution and smoke free stoves in every household. Especially the smoke free stoves minimize the pollution of the indoor environment and related health problems. Women can easily prepare food and ensure health of the family at all. This is responsibility of every sector; water, education, agriculture and health. They support each other to increase economic productivity and ensure health of the community. ensuring health is mission of health sector, whereas suppling water is water sector’s responsibility. Not only providing water supplies, water sector works on homemade treatments like boiling to ensure quality of water. In collaboration with health sector they also take samples from the water sources to check its quality and based on the findings we treat water sources. Though our share differs WASH activities are done by water, agriculture and health collaboratively. Especially women get WASH related information by WDAs and HEWs.

**Interviewer: In your opinion, are adolescent girls linked to youth friendly services at health facilities? Why is it necessary?**

**Participant:** it is better to ask this question to the health sector because I have no idea in this regard.

**Interviewer: Which of the interventions listed above do you think is most important for pregnant/lactating women and adolescent girls? We have seen many interventions, to list some; ANC, PNC, IFS, VAS, iodized salt and ITN utilization, taking extra and diversified meal, taking rest, deworming, home gardening, WASH, counselling and others**

**Participant:** all are equally important. If you take our area, you cannot say one is better than the other because every intervention has its own importance. Rather we are looking for other necessary intervention.

**Interviewer: In your opinion, which of the above-mentioned interventions for the pregnant/lactating women and adolescent girls are being implemented in an effective way? Why do you think that it is effective and if not, why do you think that they are not effective? In what way was it implemented?**

**Participant:** almost all are successful but there might be a difference because their starting time is different. Some of them are started lately. In addition, weight given to the interventions also is one determinant for the success of the interventions. In my opinion the most successful intervention is institutional delivery. It is an evaluation criterion for every council member. The moto is not reducing maternal mortality related to delivery, but no mother should deliver at home. This is a top priority agenda and even professionals and responsible bodies are penalized when there is home delivery and maternal mortality at health institutions. Interventions like iodized salt utilization, home gardening, and counselling on taking extra and diversified meal and rest are also successful because they are parallelly given if a mother came to health institutions. But the slogan “a mother should not die while giving life” is bold in every meeting and the community is promoting this slogan. To realize this slogan, the government is working to avail ambulances and road access to every kebelle. In the absence of road community by itself carry pregnant mothers to health facilities where they can get skilled attendants via traditional ambulance (stretcher). We are successful in this regard but still the other interventions need to be equally handled and given attention like institutional delivery. But in WASH related practices we are lagging behind for example, acute watery diarrhea (AWD) is a result of poor WASH. This year only there was two AWD episodes. In our Woreda, there was AWD starting from the end of 2008 E.C, the month of July to the beginning of 2009 E.C until the month of January. The worst AWD episode has happened in the end of 2009 E.C and it killed around 80 individuals. This year also there are 2 deaths due to AWD. The main cause of this disease is due to lack of personal and environmental hygiene and poor water quality. Though AWD was happened in many Woredas, had we been worked hard like the other interventions we would not face such preventable problem. This has happened because people were using surface water without treatment and even hand pump users also due to poor handling and lack of maintenance they were using contaminated water. The most difficult attitude is that they associate it with their sin and they say they have always lived in this way and so did their parents and grandparents before them. Overall, though there is improvement we need to work hard to change such poor practices that would expose the community to preventable diseases.

**Summary**

- HEWs give personal and general advices at health center. On top of advices they administer Vitamin A supplementation and screen children and women. They also deliver FAFA to children and women identified with acute malnutrition during screening. But Plumpy Nut is given to children only. They also teach them how to prepare foods blending from cereals and legumes.
- Rural residents use Dimtsi Weyane Radio program.
- Though iodized salt is better in content wise the uniodized salt is better in test and because of that there are many households who use uniodized salt
- There is school feeding programs and it’s important to alleviate short-term hunger and helps children concentrate on their studies and enable them to gain increased cognition and better educational outcomes. In addition, for school going adolescent girls, they have gardens for demonstration purposes in their schools that helps them to learning more about nutrition practically. But there are no interventions for out-school adolescent girls.
- Vulnerable households who have a pregnant woman and with children under two years of age/lactating women are PSNP beneficiaries
- The most successful intervention is institutional delivery and related interventions whereas WASH related practices are unsuccessful interventions.

**Section 4:** **Implementation challenges and** **Community factors affecting access to nutrition interventions**

**Interviewer: What are the challenges to implement delivering the nutrition interventions that we have been discussing for the pregnant/lactating women and adolescent girls? When you answer think of individual level factors, community level, intervention level**

**Participant:** as I have stated it before lack of awareness is one of the implementation challenges. Sometimes though you created awareness there is budget constraint. This is due to lack of prediction because we don’t learn from experiences. There also professionals who lack commitment, they do things for the sake of getting salary instead of to contribute to their community. There is also associating things with religion and culture.

**Interviewer: what kinds of religious and cultural aspects that could be considered as implementation challenges for pregnant/lactating women and adolescent girls nutrition?**

**Participant**: for example, though it is changed there was a belief that priest’s wife could not be investigated by male health professional. Our community also prefer to go to holy water instead of health institutions and those sites are source of AWD because people having such problem may go to those sites. According to the survey done, 76% of AWD is caused by poor water quality and according the trend of the people caught by AWD, most have history of visiting holy water. Instead of going to health facilities people go to holy water. Secondly, they associate the health problems with their sin and God’s wrath. For example, they say river Gereb-Giba has been there for long time and we have been using it for decades and so did their parents and grandparents before them unless it is God’s wrath how come water will cause health problem.

**Interviewer: how do you see the cultural acceptability of the nutrition interventions?**

**Participant:** now our community is aware of the good and bad cultures. They are maintaining the good once and avoiding harmful cultures. In the past performing your work on Sunday was prohibited but now such thinking is changed, and mothers are preparing food for their children and families.

**Interviewer: how do you see the convenience of the interventions for pregnant/lactating women and adolescent girls? What about the quality of service and commitment of service providers?**

**Participant:** in our Woreda we have 20 kebelles. Of these 20 kebelles there is only one kebelle which has no road access that connect with the Woreda and other kebeles. Though topographically it is difficult the rest kebelles has road access. There is lack of preplanning. For example, instead of calling to ambulances ahead of time the community call once something happened. Unless otherwise, there is health center at every kebelle, health posts in every cluster and Yechila health center is upgraded to primary hospital. Mother who were travelling to Abyi-Adi and Mekelle are getting services at their proximity. In addition, there is expansion of health institutions from year to year and equipping with necessary materials, human resources and ambulance services. There was lack of ambulances, but we solved it by dedicating ambulances to serve pregnant mothers and laboring mothers through recruiting drivers to work by shift to serve them day and night. But still there are problems related to fuel and maintenance cost and ambulances are called at a time. So, we can say the services and interventions are accessible and convenient to pregnant and lactating women. Regarding resources as I have mentioned since maternal health services are priority agendas there is commitment in availing necessary resources though there is still constraint.

**Interviewer: For these challenges that you mentioned, can you tell me of any solutions that your institution has applied to effectively implement the interventions for women and adolescent girls? Specify each solution done for each challenge? While your institution tries to solve the challenges, what problems do it faced? What do you think needs to be done to better address the challenges you have mentioned? How they can be addressed better?**

**Participant:** as a water resource office especially in the water activities there are attempts to provide clean water to the communities in their proximity in collaboration with other sectors. We identify and discuss at Woreda level where are the areas with low water supply coverage to prioritize our interventions. Then once we identify the areas we develop proposals and apply to either REST, UNICEF or regional Water resource bureau. In addition to installation of new water supply schemes we also apply for maintenance of nonfunctional water schemes. Especially during AWD episode we have tried to maintain the nonfunctional water schemes, but the spare-parts are not available in the market and this was one of the challenges for our maintenance activities. We always present proposal to the Woreda council but based on the budget constraint and the urgent societal need sometimes water may not be a priority. Because the Woreda council out way which to do first, constructing health center/post, farmers training center or water. In addition, capacity of the sector also hinders our activities. Though there is budget constraint we do a lot of activities by public labor. For example, in rural areas we don’t request budget to construct health facilities and water and soil conservation rather we mobilize the public.

**Summary**

- Lack of awareness, budget constraint, unavailability of spare-parts for maintain water supply schemes, religious and cultural factors are mentioned as implementation challenges affecting access to nutrition interventions

**Section 5: Multi-sectoral collaboration to improve maternal nutrition**

**Interviewer:** **Do you feel it is necessary at your level to work with other sectors/institutions to address maternal nutrition? What about for adolescent girls’ nutrition? Why? Which other sectors do you feel are** **necessary to work with your institution? How do you see the other institutions’ roles complementing your role in improving maternal and adolescent nutrition?**

**Participant:** it is known that working alone will not take you anywhere and working with stakeholders is successful. When we evaluate our works, activities done alone and with collaboration with other stakeholders are quite different. Especially in impowering women be it in health or economy it is good with other stakeholders like agriculture, health, women affairs, Woreda administrative, and other concerned development partners. Activities done in collaboration with those stakeholders will be successful. For example, activities done in reproductive health area UNICEF and Save the Children have done a lot; by material support and capacity building in different health centers. We value the capacity building given by those development partners more than the material support because the knowledge will sustain for long time. Development partners are working on issues where the Woreda cannot reach. As an office we participate in water and irrigation activities. If water resource did not avail water, **i**t is difficult to speak about irrigation and clean water. Thus, our office is working in collaboration with health, agriculture, and the other sectors which I mentioned before in fund raising, mobilizing public labor or in attitudinal change. Because if you convince the public, they can do things by themselves.

**Interviewer: How do you evaluate the level of collaboration among sectors in nutritional interventions? Why do you think is so?**

**Participant:** we have steering committee led by women affairs at Woreda and kebelle level. We are members in those committees to ensure women beneficence. Things are presented in the larger committee after it is evaluated in steering committee. The larger committee is a committee led by the Woreda administrative head consisting of heads of different offices even those who are not members of the steering committee. Then the larger committee evaluates and discusses the progress and way forwards up to giving technical support and punishing low performers. Except not following the schedules the committee actively evaluates activities done be it health wise, politically, socially and economically by every sector.

**Interviewer: do you discuss nutrition related agendas in your committee?**

**Participant:** yes, since health part of the committee we discuss issues related with nutrition like the importance of eating fruit and vegetables. For example, agriculture in their irrigation activities they promote farmers to produce cereals diversified cereals. They technically support farmers what to sow based on the type of soil and availability of water. In the irrigation activities, role of water resource, agriculture and other concerned bodies is identified.

**Interviewer: For multi-sectoral action that effectively works to improve maternal and adolescent nutrition at your level, what kind of change in terms of the way stakeholders work together is needed? What type of resistance to the needed change do you perceive, or have you experienced so far?**

**Participant:** I don’t see problem in the existing collaboration because there is good integration and when we discuss at committee level we discuss at individual and sector level in a way that corrects our drawbacks. So, in my opinion it is good to continue as it is. But there is overlapping of activities with temporary programs.

**Interviewer: Is there coordinating platforms in enhancing multi-sectoral coordination in maternal and adolescent nutrition? To what extent does your institution participate in the multi-sectoral nutrition coordinating body at this level?**

**Participant:** Yes, as I have mentioned before, in SURE project each sector has taken assignment. Activities to be done by water resource, agriculture, health and other sectors is listed in detail. Then what is left is contextualizing to your own sector. Then to end undernutrition each sector should play its role. Water sector participates in availing water for drinking and irrigation purposes. Because water is critical in ending undernutrition. Because of SURE project we have a platform that enhance multi-sectoral coordination in maternal and adolescent nutrition. We meet in the 7^th^ date of the month. Regarding the participation of our institution it is active participant. In addition, we have other platforms to meet for example, in malaria and HIV/AIDS committees.

**Interviewer:** What needs to be done to improve the capacity of these bodies/platforms for effective coordination?

**Participant:** due to critical shortage of human resource we could not assign focal person for every activity. For example, in agriculture, assigning gender expert to empower women and look for every gender issues.

**Summary**

- It is mandatory to work with other sectors/institutions to address maternal nutrition and agriculture, health, women affairs, education, Woreda administrative, and other concerned development partners are mentioned sectors necessary to work with water resource office

**Section 6: Other interventions that influence adolescent and maternal nutrition and health outcomes**

**Interviewer: Do you think** **delaying the age at first birth to after 18 is better for the health of the women? How? What other benefits does it have for the women? What about for the baby? Does this delay would have a benefit to the nutritional status of the women? Do you think this message is being promoted in the community? Who are working on it? How do they promote?**

**Participant:** well, when a woman leads the household she sacrifices a lot. First, she is not physically mature and psychologically she is not ready to shoulder mother’s responsibilities. Then physical and psychological immaturity will lead her to stress. When we compare with the past, in the past you stay with your family until you physically and psychologically mature whereas this time after weddinging you independently lead your life. But in this regime, there is low that prohibit early marriage and you will be punished in kind or you will be imprisoned. And this is accepted by the majority of the communities. If there is early marriage both sides will be accused and will be responsible for that. Then if the adolescent is a student she will continue her study, because we can say there are no out-school adolescents. Anyways women affairs work in collaboration with kebelle and Woreda leaders and schools to prohibit early marriage because it will lead to teenage pregnancy that would lead to miscarriage due to immaturity of ovary, increases fistula and leads to school dropout. Attitude of the community and lack of awareness are factors that leads to early marriage. Because the community wants to see their child happiness and to get fame by investing a lot for the wedding ceremony. In the past, especially the poor were forced to give their child to rich once to share wealth. But this time, delaying early marriage till age 18 is promoted by HEWs, women affairs and schools.

**Interviewer: Can you think of any other opportunities to prevent early marriage? How?**

**Participant:** our integration could be best opportunity to prevent early marriage. If you get someone is to be married before the age of 18, her age will be verified at health institution. There may be approved proclamation, but I recommend approving proclamations and including it in the criminal code to prohibit early marriage.

**Interviewer: How many years do you think the** **gap should be between successive births for women? Why? What about if shorter than it? What other benefits does it have for the women and the baby? What do you suggest promoting it in a better way?**

**Participant:** increasing birth interval helps a child to get his/her mothers’ love and the mother also get enough time to recover from her previous pregnancy and to get ready for the upcoming pregnancy. Because women who give every year and who give at intervals for example every 3 year are different in their health and social life. If she gives birth at short intervals she will be absent from different social events like wedding and funeral ceremonies and meetings because she is always responsible to care, her child. In addition, the child will not adequately breastfed if she gives birth at short intervals. If the child is not adequately breastfed, s/he will not grow well. I have observed it practically. So, birth spacing is beneficiary for both the mother and the child. It helps the child to be health and strong.

**Interviewer: Can you think of any other opportunities** **for promoting birth spacing?**

**Participant:** the national policy is governing. Then one of the opportunities is family planning interventions is important to promote birth spacing. In addition, understanding the impact of unprecedented population growth can also lead to birth spacing. In addition, the commitment of the government to save lives of women and newborns is an opportunity to birth spacing.

**Interviewer: how do you see the religious and political influence on birth spacing?**

**Participant:** it is known that though religion and politics are different they have similarities in some points. Because they all promote to be loyal and faithful. Anyways they all don’t promote early marriage and birth spacing though there are awareness differences among the religious leaders because someone can raise a question if they don’t support it, why was there was early marriages. Overall, except the positive influence there is no negative influence.

**Summary**

- The benefit of delaying the age at first birth to after 18 are; it helps the women to be physically and psychologically matured and minimizes miscarriage due to immaturity of ovary, fistula and school dropout rates. Delaying early marriage till age 18 is promoted by HEWs, women affairs and schools.
- Gap between successive births helps a mother to get enough time to recover from her previous pregnancy and participate in different social and economic activities. In addition, it helps the child to be adequately breastfed and grow healthy and strong.
- Family planning and commitment of the government to save lives of women and new-borns are mentioned as an opportunity for promoting birth spacing.

**Additional Remarks**

**Interviewer: Do you have any other comments on anything that we have discussed? What lessons have you learnt regarding adolescent and maternal (pregnant, lactating and adolescent girls) nutrition at your level? What lessons have you learnt regarding multi-sectoral coordination of nutrition in this Woreda? What opportunities do exist to promote maternal (pregnant, lactating and adolescent girls) nutrition in this Woreda?**

**Participant:** I have already said it and I don’t think there are things left because you were presenting it in a summarized way. But what I want to add is, after you write a report it is good to brief the findings to the public.
