# Supplementary material for: Barriers to utilize nutrition interventions among lactating women in rural communities of Tigray, northern Ethiopia: An exploratory study
Source: PLoS One. 2021 Apr 30;16(4):e0250696. doi: 10.1371/journal.pone.0250696 (PMC8087028; doi:10.1371/journal.pone.0250696)
Supplement: S2 File — (ZIP) [file pone.0250696.s002.zip › S2_File.Doc/Woreda level and above key informants/075_IDI-Water resource office head_Samre woreda.docx]

**Day6: 04 /03/2010 E.C**

**Translation: In-depth interview**

**Section A: Interview details**

Zone: south eastern

Woreda: saharti samre

Name of participant: Siyum Tedela

Institution of key informant: office of water resource, mining and energy

Interviewer: G/medhin.B

Date: 04/03/2010 E.C.

Interview start time: 4:00 Am; local time

Interview end time: 5:25 Am; local time

**Section A: Interviewee professional details**

|  | Socio demographic information | | | |
| --- | --- | --- | --- | --- |
| **Sex** | **Age** | **Marital status** | **Education level** | **occupation** |
| male | 37 | Married | Bachelor degree | Government employee |

Position: Head

How long have you been in the current job/position: 4 years

**Section1: common maternal nutrition**

I: In your opinion, being a member of the community and being office head and as an expert, what are the common nutritional problems for women in the community? What about for adolescent?

P: In our community, there is a problem in providing balanced diet; there is lack of awareness in the community. There are opportunities related to child care and home gardening and are in place at early stage, thought these are not expanded. All balance diets like egg, meat and other foods that are scientifically recommended to children and follow up is limited.

I: what about in terms of type? Example stunting, disproportion of height to age and weight to age, and others

P: regarding to stunting, the first problem is not serving a balanced diet, and in addition, lack of birth spacing; when it important to have birth space of four to six years, there is a situation when women get pregnant within a year. This is a critical challenge even there are choices, there is poor utilization of contraceptive. This is associated with backwardness. Besides, exclusive breast feeding is not implemented among all mothers. Even after six months, there is a problem in preparation of balanced diet to child as meal to prevent stunting.

Regarding adolescents, girls’ enrollment to education is in a good status. Their proportion at school is higher than males. Reciprocally, early marriage is common among adolescent girls. There are efforts to prevent this, but it not yet completely solved.

I: what about problems related with micronutrient deficiencies (such as anemia, night blindness, and goiter) in adolescents?

P: Here, to prevent goiter, the community know it. Use of iodized salt is good and the community has good awareness in this regard. There is good progress than the previous. In supplementing iron, there are things done by the government but, in the awareness in the community is low. Therefore it should be promoted.

I: Are anemia and goiter common in the community?

P: No, not now. Previously before five years, there were incidents of goiter among women. But, this time, since my enrollment in this office, I have never seen a case.

Regarding night blindness, there are many people who are blind in adjacent Woreda of region three and some in this Woreda. The awareness is growing from time to time. Thus there are cases of blindness.

I: what about no communicable diseases like hypertension, diabetes, and others? In relationship with nutrition

P: Rarely, there are people with diabetes and hypertension. Even I saw a situation with blood clotting (thrombotic stroke). The case of blood clothing was among my relative. The community is not losing hope because of illness. Because it is a time civilization and globalization, people are seeking care even at private health facilities. Regarding mothers, during pregnancy a women attends/ has follow up every month in health facility. This is what I saw even though I am male. I have opportunity to meet health professionals, like nurses and health extension workers; and I lead one cluster. When we look at holistic approach, there is good awarness.

Women, at time pregnancy, have good screening and examination practice. Antenatal and postnatal care is good. But the big problem which is not solved yet is home delivery. Sometimes it goes forward and sometimes it gets backward. Here, what is targeted is not well achieved. There is a problem in follow up. At low level, even the wife of an expert is giving birth at home. Thus, there is a gap in continuous awareness creation program.

I: what about stunting and underweight (low height/ weight for age) among others?

P: Here, there could be natural cases; because one can be short or long. But I could have shortage of knowledge to identify who is getting pregnant: the short or the taller.

I: Your observation can be in your neighbor or community; woman whose height is shorter than their counter age mates, and low weight in relation to her age…

But in relation to nutrition, the problem is not prominent. Because of the awareness increasing, the magnitude is low. At age of 18 years, her height may seem like 12 year old girl. But I did not see. But I think there might be in other kebeles as I did not go around to all areas.

I: What about issues related to overweight among women?

P: no.

I: is there a situation where the community suffers from food insecurity? Do people have food that can serve them for a year?

P: On food security, there is change from year to year. Even though there is climate change, the community bringing change. However, there are some households who have food insufficiency.

I: Can you guess how many of the community is on food support (emergency, safety net?

P: In our Woreda, the population size is about 151 thousand. Of which 35, 000 is benefiting from safety net. Within this, there some who work for food and some on direct support. This number can be changed every year. Together with the people on emergency support, the number goes to up to 48 or 49 thousand. Therefore, in the community, these on direct supports has good attitude. These are mainly elders and people with disability. The support stays from six to ten months.

I: How about women/girls?

P: There women who are in food support, though I do not have the exact number.

There are female head of household who benefit from the safety net program. There are different options there; they have potential to graduate from the safety net and finally they will support themselves.

I: How do you think women are especially at risk of malnutrition we have mentioned above? These can be pregnant, lactating or adolescent girls to comparison to men;

P: we can see the risk in two ways. 1) There are women who have land for farming and are also involved in safety net program. 2) There are women who have no option: they can be prostitute, may be merchants who do not have land, and are involved in safety net. In both of the groups, there is risk for the mother and child.

After work, when the mother gets back to home, there is a problem in caring the baby, as she usually gives her breast to the baby without washing her hands. There is also a situation when mother are not using the available opportunities. E.g home gardening which they can easily do it is limited.

**Section 2: Nutrition priorities in the Woreda**

I: what priorities d your institution has in relation to maternal and adolescents health? E.g. In specific to your office could be about access to safe water;

P: Our focus to mothers is related to our work that is water. The second is creating water potential for irrigation. In these issues, regarding on water for drinking, in every side, the committee that controls the water pump is led by women, because women are facing many of the challenges. With child on her back, she lit a fire, bring water, carry a pot and do all things. To solve the challenge of water, we gave authority to women to control the water source at both kushet level and kebelle level. We are trying and making service women friendlily at all level with respect to water. But, there are still challenges to be addressed by our office. Besides, we build alternative water source for women that is wale (Aela). We were build wales in the previous year and we have plan this year too. If a women is head of household she have a land around her house, she has to home garden either vegetables or cereals at her nearest site. This way, she has to work rather than looking other for help. This direction we give to women. We also introduce alternative energy source. The woman is advised to leave the traditional way of cooking and substitute it with new technologies. This is also our priority that we currently working on it.

I: How are these interventions you mentioned before are really benefiting women in relation to their nutrition?

P: Here, agricultural extension works give them training, and we, together with other partner, also create conditions to provide seed for home gardening. However, all women did not get these opportunities. At the model site or pilot area, we are beginning the intervention. If there is a damaged wale, we coordinate the kebele community and they maintain the wale. Here, we get good support from Maret (REST=Relief Society of Tigray), Mekene eyesu: it is an organization and world vision Ethiopia. These are our supporting partners in this Woreda. And in biomass, energy saving, it is related with nutrition and health. It is related with fire and smoke. Because she carries a child at her back while cooking, it is not comfortable for her. In collaboration with GIZ, we are building biomass.

I: is your institution collaborating with either health office or agriculture or other sector to improve nutrition among women and adolescents?

P: we are a partner with women affair, health or agriculture. In addition we are member of stream committee. Thus, with issue of nutrition, stunting and early marriage, we are in line with the health office. But, there is something left and it is not complete. We are not changing the community.

I: what are the limitations?

P: One, once we produce water, there is no follow up if the community is using it appropriately. By considering home gardening as an activity of agriculture, there is a gap in working jointly with agriculture office, because we have to work in an integrated way rather than independently. In previous evaluation, this is noted as weakness. And in others, like preparing balanced diet to child and preventing him from stunting and keeping child health, there is a gap in implementation. For the next, we have to follow it. This is not only the issue of agriculture, health and water resource and women affair but it is also an issue of other sectors. If we work together and strengthen the activities, we can bring change.

**Section 3: Nutrition interventions that improve adolescent and maternal health**

I: what nutritional intervention are in place to improve women health? Be it by your institution or other sector.

P: it is primarily done by health extension workers along with women development army leader. As culture, all the different food varieties are collected and they demonstrate how to prepare porridge at the health post. They also give education to women. If this is strengthened in the future, there will be good change. If you see at television, mixing with salad and egg, women in Raya are seen preparing porridge. In our Woreda, there is resource, and had we worked here, we could have brought change. At low level, these observations are there. The HEWs are transferring skill to women. But there is limitation in expanding the demonstration to all women.

I: What else?

P: At school, education is given on home gardening by HEWs

I: what about to the out-school girls ? What is being done for them and the entire community?

P: To the general community, there is a program called “education for adolescents”, this is given not only by teacher but also by water expert agriculture and health expert. Here, adolescents are fast to participate in the session.

I: what are the lessons given at the session?

P: Regarding health, all the 16 health extension packages are covered and are taught for adolescents, adult males and females. The same is with agriculture, related with use of animal source foods and vegetable. By our institution, we taught about water utilization and use of new technology as alternative energy source like bio mass. There are interruptions, but generally it is good.

I: Is it planned? Does it have schedule? How frequent it is?

P: yes. They have their own plan. It is given two times per week. It can be in week end or other day which suitable to community is fixed. I saw this practically. At Woreda level, the stream committee and the administration I aligned and working to achieve it as this a policy from the government, i.e. education for adolescents.

I: What about in getting advice on extra meal to both pregnant and lactating mothers? Do women get screened for their nutritional status?

P: yes it is given. In this Woreda, what we have as a structure is the women development army. Each are have 25 t0o 30 women. Each army leader identifies the number of pregnant women in her group, when do they go to health facility is audited. The HEWs also go home to home and measure children. I do not have the professional knowledge of what they do. But, I saw them measuring children. At health facility, children are weighed and if he is under weight, they gave them plump net. To mothers, education is given on antenatal and postnatal services. So do the development armies where they educate to their members. The problem here is home delivery. It is not completely solved. We have to strengthen our efforts.

I: Do adolescents get nutritional screening? Do they get the service?

P: To the in school once, yes there is service and follow up. And the out school adolescents are organized in the development army

I: Mainly the health service focuses on mothers (pregnant and lactating) and children. What is the situation here, are adolescents mobilized for nutritional screening here in Samre?

P: Here in Samre, yes we mobilize them. The town is suitable for work, and there are tea and coffee houses and shops. There are many adolescents who work there. These are inspected for sanitation. This is done by municipality. There is even stool examination for food handler. I saw this physically. They will be treated if they have the disease. They are advised to cover their hair while they prepare and handle food.

I: what about in terms of nutrition? E.g. MUAC measurement

P: I do not know.

I: What about food diversification during pregnancy and lactation? Even on those who are food secure,

P: In the town, it is good. Even in the rural food diversification is fair. The resource is available at rural community, because they produce it themselves, and the utilization is good. If they prepare ‘shiro’, they will add ‘Sils’ and vegetables like salad and pepper. This is observed. I cannot say there is some who do not have at all but, there are individuals who only use only one food item.

I: what about on the use of iodized salt? Is it common?

P: In this regard, it is properly implemented. Even though I do not cook, what I know is, the ordinary salt (Gamfur) is added while the ‘Tsebhi’ is still cooking, but the iodized salt is added after the Tsehi gets down and will be immediately covered. The utilization is good and the community knows it. But I have a reservation, if the community applied it based on the science. Thus we need to have continuous follow up.

I: Are women using home gardening? What about their involvement in safety net programs? (Pregnant/lactating mother)

P: during safety net, if woman has family member of four, she works only for three and she is waived for one. But if the head male he must work for four. During water and soil conservation, two women are parallel with one man. But if it is food for work, the task is equal for man and woman.

If a woman if pregnant, it is not acceptable if she oral declares. However, she must bring a confirmation letter from the health facility including the duration of pregnancy. This has two advantages: it is serving as an approval and check up for the mother because the agriculture extension expert will also tell her to do so. In this way both HEW and agriculture extension worker are working together. After six months of pregnancy she will be waived from work until ten months after delivery.in this way the safety net is good. In addition, home gardening is demonstrated at FTC (farmer training center). If there is no FTC, the show them in one of the farmers’ land. Women are selected and trained in this way. I, am agriculture professional; before four years, I was working in that way. Home gardening focuses mainly on poor women who are involved safety net, in order to create an alternative source of food. However, there may a gap and inconsistencies among women whether they have water source or not. In the future if all responsible boy work together, there will be a change.

I: What about regarding WASH (water, sanitation and hygiene s)? Mainly on water, what is being done regarding water hygiene, access and utilization?

P: regarding water, the direction is: In rural, the water source must be in less than one km and in urban it must be in less than 500 meter. At household 25, 80 letter of water is used and there is improvement. Regarding water hygiene, both from the health and our office, for example, we provide chlorine every three month to prevent water borne diseases and we have also an expert that test the water biologically. Besides, we provide Bishan gari (chemical) and give to health office for distribution. The health office also provides Wuha-agar as an alternative. There is also a third option; I forget the name, but it is provided by health office. At the water source, we educate the community to avoid open defecation around the water. On utilization, health professionals inform them. Mothers tie a jar inside the pot to avoid contamination. These all are expanded to all rural community. However, there are challenges. When the pump is broken, they simply wait rather trying to maintain it. And at that time the community use river water. Even though there poor follow up from the office, the community has also low motivation to maintain himself. So, this is a problem.

I: what else? Even in presence of water, what does the personal hygiene and environmental sanitation look like? Especially among mothers

P: Here, the personal hygiene and environmental sanitation is good. Sometimes it returns back. Regarding environmental sanitation, e.g. latrine utilization, the community has built a toilet. And we educate them by raising cat as example. But, still the utilization rate is low. To defecate is a must as it is natural process after you eat and drink. The problem is people defecate in open field. Other on person hygiene and washing clothes, it can be considered as good. Even the farmer do not want to join people is he is not clean.

I: what about in relation to trachoma you have mentioned before? Trachoma vs personal hygiene

P: In our community, there is trachoma, and this indicates there is a gap in the personal hygiene practice in all community. The problem (trachoma) is common across the Kola kebelle (areas that are tropical in climate) and many of them are from Amhara region. It is also present in our Woreda. The disease is not hereditary; you can bring it by yourself if you have poor personal hygiene.

Farmers know that Vitamin A cleans eye. The problem here is lack of water related to access. Thus, the poor person hygiene is not completely solved.

I: Is malaria common here? What about the use of ITN among mother?

P:In our Woreda many of the kebeles are Kola and previously malaria was at an epidemic level. But these days, because we have created awareness among the community and the government is working continuously either on awareness creation or spraying houses or provision of ITN. The community knows the advantage of ITN, but its utilization has limitations. Like what you mentioned, I went to one kebele and I was counting the number of ITNs that are used to cover hey. I counted more than two, three ITNs, and them I have communicated to the kebele leaders. I describe that “your kebele is Kola, people are sleeping without bed net and get bitten by mosquito, but the ITN is used to cover cereals to protect it from bird” why such things are happening in your kebele was our point of discussion.

The malaria endemic kebeles are known and anti-malaria is sprayed. Thus the magnitude of malaria is now decreasing. For example in this town, there was no mosquito during my child hood as I was born and grow here. But now there is mosquito. Mosquito is common here than that highland. The community complains why we are not given ITN if our house is not sprayed with anti-malaria chemical.

This is to prevent from mosquito bite. The community in the town has good attitude towards ITN utilization.

I: what about services relate to deworming mothers? Can with intention to prevent from water born disease or other parasites? Can be to mothers or children

P: There are four drugs swallowed per year, I do not know what it is used for.

I: I think, if the drugs are big and red, that is given to prevent trachoma.

P: Yes, Yes. There four drugs are swallowed to prevent from trachoma. What I see related to prevent parasites is provision of Wuha agar widely. For children and mothers while they come for vaccination, I can conclude there could be deworming service. But actually I did not know it. I see HEWs giving vitamins to mothers.

I: Did you think women (lactating/ pregnant) are getting targeted supplementary feeding, like fafa (corn) and oil for themselves after MUAC measurement and are told as stunted or wasted?

P: Yes the do. They take fafa and oil for themselves and a balanced food to their children too. It thinks it is WFP and World Vision Ethiopia who provide the supplementary feeding insecure areas to women and children who have malnutrition.

I: What about adolescents (10-19 years old girls)? Are they part of the targeted supplementary feeding? E.g. If they wasted

P: In the emergency program, there is fafa. But in specific to adolescents, I do no Know.

I: Are women getting Vitamin A supplementation? A small green tablet to prevent night blindness

P: Yes it is given. Be it based on program or monthly, the health workers provide the drug at health post or health center.

I: what about to adolescents, especially the out-school ones, do they get vitamin A?

P: it is good if you skip me this question

I: My observation: He do not know anything on adolescents

I: At health facility, is there youth friendly service? Eg. If adolescents want condom, HIV screening, STD examination; is there separate service for them at health facility.

P: At the health center, different health education services are given to all patients before they start examination. The service at health center is equal to everyone.

Regarding condom utilization, condom is available in every sector, everyone can take. But regarding the youth friendly service, I do not know if it is available or not.

I: Which of the interventions listed above are important to women? Meaning, if we work on them, you feel we could bring change. On pregnant, lactating and adolescents

P: For women, first, we have to work on these which can prevent stunting. If we do not produce health generation, the country is growing and if you did not substitute a generation, there could be crisis in the country. If we feed balanced diet, the generation will be healthy. We have to work to improve this, not only you form above but all including the farmer. If the government gives focus, it can be easily done at low level. Thus, this should be priority.

In addition, to decrease maternal and child death, it is also good if we give focus it.

A mother should not give birth outside health center or hospital. This should be promoted and need focus.

I: Which of the above interventions are effective to women (both pregnant and lactating)?

P: Example the slogan, “a mother should not die during delivery” is on ground.

I: Yes, but are there situation when mother die due to nutrition related health problems?

P: there are no mothers who die of malnutrition.

I: may not be direct but associated with it? Eg if she have malnutrition or poor hygiene, she may contract other disease.

P: Yes, even health women can die. Thus all are important. E.g. if there is no water, people may die as 70% of our body is water. These activities related to water are good. It is not because of me, it is direction from the government. Thus, we have to focus and work here. The previous ones, that you have mention for adolescents are important. And now you have oriented me about them, and we have work together the health office to address them. The youth friendly service you mention is important, as in Tigray there is an increase in HIV prevalence. Thus we have to work on youth and the other community as well. We heard many African countries are now free of HIV epidemic. To move our country to that position, we need to work hard. Even in our country if you look at the regions, Tigray is lower than Afar in its performance to prevent HIV while it is called better region. Thus we have to strength our effort.

I: in terms of nutrition, are there opportunities at school to improve adolescents’ health?

P: Yes we have many opportunities. One, every child is coming from different household, and we can teach them about home gardening, use of milk and milk products and use of egg. If introduce these packages to school including the demonstrations we want from health, e.g related to gejatat (Mitin). The student may show to his brother, mother and father. Thus, if we strongly work in this, we can bring change. At rural, in the FTC, we have to show the model activities what I have mentioned before; it can be breeding hens or it can be hybridization cattle or any other available intervention. If these things are done, there could be improvement. Even at health facility, it can be at health center or hospital, if we work on model intervention, it will be better. Latrine utilization is important. As it is a risk factor, There latrine at school, health center, but not at FTC; thus we have to build toilet when there is shortage either ourselves or together with partners. If we create awareness in the community on latrine utilization, we bring improvement in this aspect.

**Section 4: community factors affecting access to maternal nutrition interventions**

I: what are the challenges to implement/deliver the nutritional interventions that we have discussed for women (pregnant, lactating and adolescents)?

P: the barrier, from high level to low level, is shortage of budget for capacity building trainings. If there is no budget, though you want to expand service and build capacity of people, and introduce new technology to FTC, the capacity of FTC is limited. Even at school, money is collected from the community. Thus, there is generally shortage of budget. In addition there is low readiness; ‘readiness to serve with your profession’; if you do not think that I will be benefited if the farmer is benefited, then you will not be changed and the farmer will not be changed. Both frontline experts and administrators should work here. After introduction of new intervention, there is a problem in follow up. E.g. if you build a toilet, you have to follow using check list if that individual is utilizing or not. Thus the challenges are related to attitude and resources.

I: what about individual factors? E.g pregnant women’s awareness on interventions, she may prefer home delivery; or related to their educational status as it affects our level of understanding; could these are challenge?

P: these could be challenge or the challenge could be in breaking these challenges. What I mean is there is belief and backwardness. Because there are traditional birth attendants and observing previous evolvement, together with the current population size and climate change and other activities done in our area, mothers may not comprehend the things. “I gave birth to you and I did not die, so what happened to be” is common backward thinking among mothers. This is big barrier. In addition, even though the available health education is good, lack of strengthen the existed structure is a challenge. For example, if a pregnant woman is left with one month to give birth, she has to stay at hospital, but the woman is worries about the life of her children and husband. When she stays at home, and delivers at home and because of absence of traditional ambulance, she faces a lot of challenges. But, the problem is we do not work any activity to break the barriers. To the solve backwardness of women, we need to educate continuously, and to the organized development armies, be it youth or women, we need to strengthening them. This is the responsibility of the Woreda, and kebelle leaders. We need to work in an integrated way. If we continuously follow them we can bring change. For example there is a kebele called ‘Dekell’, she is always model in terms of institutional delivery. There is no connection, but they call for ambulance by climbing to a mountain. The other is, it is related to natural geography of the area and there is no road for transport. This is a challenge itself.

I: what about community level factors like the transportation you mentioned and its cost? And other like beliefs and culture to not to get acceptance etc

P: In our culture ,there are many things. E.g. a pregnant woman should eat food: she is going to drink milk, eat egg and meat. “Why are you eating on Wednesday and Friday?; fasting for forty day is order by priest, and this includes children older than seven years, so you are not allowed to eat the above food items. There are many fasting sessions: fasting for ‘Haweriyat’, fasting for ‘Nebyat’ and fasting for for St. marry. The last one is only for adults. All these problems are not yet solved. We are not if the farmer knows these things. For example, my wife is pregnant and we always nag by this issue. The belief is critical. The woman did not understand the fact. There is possibility to get penitence from priest after eating.

On the transport, there is shortage of ambulance, and the woreda’s geography is also challenging to access it. There are kebelles where a car cannot travel through it. There is a situation where it takes two to four hours to carry and bring woman to place where the can is. The direction that a woman should stay a health facility for a month is not implemented because of the inaccessibility for transportation.

I: Does cost of nutritional intervention have impact for mothers? E.g Iodine

P: Regarding the iodized salt, the cost is similar, even, the iodized salt is cheaper. Previously, there was a complaint on the cost, and tend to use the ordinary salt in which it had been used by our parent. Now this challenge is already solved. A lot of women with goiter have taken iodine, and the community has already understood the benefit. In Fire woyene keblle, there were many women with goiter on their neck. But, there is no attitude of saying the cost expensive. The problem of not using a balanced diet is because of lack of awareness. The religion as mentioned previously is a challenge to use balanced diet.

I: How convenient I the intervention given to women and adolescent girls? eg. Use iodized salt or iron supplementation or other; how acceptable are these by the community?

P: Yes, there is farmer who is fast to accept and slow to accept ones. There is also aggressive one. The solution is to continuously inform them and educate them.

There are challenges while using contraceptives; male complain why he married his wife, and this is associated with backwardness. We you show them how to prepare a balanced diet, and the found it tasty, they (farmers) will joke and say “they guys are rich”. But preparing a balanced diet is not only for the rich and it rather can be done with the available food item at every one’s house. This message must be conveyed to the community. And, there are problems in doing this.

I: Are the above nutritional intervention resource intensive? Or de we have the resource to implement them?

P: I do not think there a problem in resource. It is all about using and not using it. Mothers eat the teff, maize, wheat and egg. The have it all. The problem may be meat, but they have the goat except in few farmer. They prefer to sell the goat and own money than slaying and eating the goat. They do not understand the benefit of meat. Over all, I can say there is shortage of resource in many of the farmers. There could be some who have shortage. The major limitation is lack of awareness.

I: for the above challenges you have mentioned above, can you tell me any solution that your institution has applied for women? E.g. maintenance of broken water pumps and use of alternative energy source

P: we are working to get the community out the problems. The community has a potential to solve it. If the leader took to the initiative, it will be good. For example for one water pump, the community needs to contribute six thousand birr for maintenance. There are communities in some kebeles who saved more than this, 20 to 26 thousand birr in two years. Bringing women to leader was challenging: “how come a woman works?” was a challenge. This was not from the farmers; it rather was from the experts in that kebele. “She will not work as per my expection” was major challenge. After though discussion, they accepted it, and it is also guide set from the above level which is approved at the regional council meeting of Tigray.

We believe in equality, and women had been doing it for many years on the struggle for freedom. Shortage of water primarily affects women, and man is simply eating what is being prepared by the woman. He ploughs and weeds. Thus, we agreed that the leadership should be taken by woman. And this is already implemented.

I: Is there continues follow up for the water pump?

P: Yes, I told you because it happens in few kebeles. There is no community who don’t save money. If the pump is broken, they do not immediately seek maintenance, for example in one kebelle called Esret, a total of 4200 birr is saved by from the community. They were arguing each other rather than withdrawing the money and buy necessary material, but they stay for two months without any thing. There is money, and there are experts to maintain (a youth association organized for this purpose), but no maintenance. We met the expert and discuss with community, it finally was solved.

Regarding technology, the community is accepting the technology. The problem is in supply. The community is accepting us. In urban communities, we are orienting them associated with deforestation. They are using electric city to cook and to bake and for ironing. At rural, if there is electricity, lactating women are switching on the light to breast feed their baby at night. With the health extension workers we are working together. Use of alternative energy source is related with health and women affair, and we are working in align. But we still need more in collaborating each other.

I: what else is needed?

P: we need to align our self with partners. If we align together, there is no way that we can point to Mr. X or Y. The community is in poverty, we have to come down to the earth and convince the people. We are helping him technically; the community does not refuse to save money. Thus, we can help each other to solve the problem. The problem here is shortage of budget and transportation. In health, there is no problem; they have the vehicle, but the other sectors (youth office and women affair) do not. The transportation problem can be solved slowly. The other problem is lack of collaboration. For the future we have to work together.

**Section 5: Multi-sectorial collaboration to improve maternal nutrition**

I: Do you feel it is necessary, at your level, to work with other sectors/institutions to address maternal nutrition?

P: Be for mothers or adolescents, health is addressed by health office, women issues by women affair and youth office works not only to males but also for adolescent females. They work to assure if youth are benefiting from the services. Our office also works on water. Water is critical. If we work on water or alternative energy sources in integrated way, we can bring change. We should not say, fafa(corn) is provided by health, drug is provided by health. If we work in collaboration, we can bring change. If we collaborate at Woreda level, in the same fashion, is we integrate at the kebele as there is a structure, we can bring change. The community in our Woreda is generous.

I: Can you tell me any intervention that you work in collaboration with other sector; be it with health or other sector.

P: There is KWASH project; we work a lot in health, we build latrine, dig placenta pit and make incubator. These are led by our office, but we collaborate with health office hand to hand. There are nine kebeles under the KWASH project, and across all we build latrine. This is done with health sector. We also work at school, as all are community based services. Thus, there are many things we work together; we plan together for what should come first and what should come later. We also jointly give capacity building training on ODF and CLTH and related issues. All from woreda to kebele we work together.

I: what else do you think your institution can collaborate? With any partner to improve women’s nutrition;

P: To work for the community, what is left is with agriculture, like the KWASH, had there been any partner support, we can successfully work collaboratively.

In preventing stunting by nutrition, we use products of agriculture like milk, milk product and vegetables. This is fulfilled by agriculture. Had the agriculture was involved with us in the KWASH project, like the in the stream committee and in safety net, the work can better.

I: what about in other sectors; is there multi-sectorial collaboration? Sometimes there is competition among sectors to own intervention for the sack of resource; how do you this? How is the multi-sectorial collaboration and jointly using resources?

P: We do not have competition. We have common understanding. If there is change in agriculture, it is a change for the water office, and if there is change in health, it is a change for agriculture, we think of the entire Woreda. This is not individual interest; we are working for the government. There is no way to compete. There are few things which let us for competition: during safety net, we argue on budget allocation to sectors. There is no competition in work. When I stand for work, as a management body, I hold all check lists and supervise all activities done by all sectors.

I: what about from the beginning while planning, do you work together from planning to evaluation?

P: When plan is approved, we will be there if it mutual goal. We also have separate plan for each sector which is cascaded from the higher body and finally, we share to other sectors. What we do here is that: we have social services like health, education and social affair; the meet every two week and evaluate their performance. There is also domain call economy service which includes agriculture, water resource and finance; we evaluate together. There is also good governance service. Then each domain will meet and evaluate each other. Generally, there is no way that we work separately. In supporting poor women, women affair will go social affair and work together. If there is something related with health, the social affaire will work with health. Thus, I can say there is better situation in working in collaboration.

I: How about multi sectorial collaboration at lower level? What needs to be improved?

P: Previously, there was a habit when we all mobilize efforts to one agenda; For example, provision of fertiizers, all sectors was promoting this. Now this habit is terminated. We have six clusters in this Woreda, and then three or four persons will go together. One will lead the team. We evaluate all activities at a time.

I: Is there a coordinating plat form? Who lead the collaboration?

P: Here, for women nutrition, primary, it is the Woreda health office that takes the initiative. Then, Woreda office follows it with full attention. Even the women affair, women league and women association will supervise it. Even we ask each other. If a mother dies, the women affair will asked because se ha a structure at the low level too. Primarily women affair and health office will be asked in this regard.

If there is problem with water, office of water will be primarily in charge of it, because people are affected.

I: how effective are coordinating plat forms using multi sectorial efforts?

P: for example if we take what we did with health, the latrine construction and utilization is up was 98% and 99%. When we check it, the coverage is much lower than the figure. But generally, there is tangible change. It is one by two meter. It can be finished in two days, day and night. When we inform the farmer in this way, he will bring change. There could be something left, but what is needed follow up.

If you see a change in the community, you may wonder at your office. This creates a gap among the community and expert.

I: what opportunities do exist to promote multi sectorial coordination for nutrition in his Woreda?

P: Regarding opportunities for nutrition, we have better opportunities. We have many water alternatives. There are only few areas which have shortage of water. Even there, there are irrigation sites. Here, the problem is poor utilization of water.

**Section6: Other interventions that influence adolescent and maternal nutrition and health outcomes**

I: In your opinion, why would delayed marriage (after 18 years) improve maternal nutrition?

P: Regarding under age marriage and nutrition, we improve their nutritional status not at their adult stage, rather it must start from their infancy. If we that, we can prevent stunting and women may gain the desired stature. But, when they grow, what you can do awareness creation. This can be with expert at low level and the parent; reach at common understanding, you can let them enter in to the system. Because if a parent made his daughter marries, there will be a lot of problems to face. One, the adolescent girl may face fistula during delivery. Second, because her body is not grown well and she is under age, she could face other problem other than fistula. To avoid this, we need to mobilize and create awareness in the community. Because, what I think is that it is not preventable to eat balanced diet even after you grow (become adult). We work here properly, we can improve it.

I: what about related to increasing space between each birth and maternal nutrition?

P: In spacing births, there is an improvement from time to time. But it is not complete. In every locality, there are better situations.

I: What programs or activities do to promote increasing birth intervals and to prevent early marriage in this Woreda?

P: There is a condition when husband and wife came to agreement to stop birth and utilize long acting (the permanent one) contraceptive. For example in one health center called Koronel Dessue health center, there was a parent who decided to stop birth and applied vasectomy. This, itself, is a big change. But, we cannot be satisfied with few things like this; because, the community needs to have enough awareness in birth spacing. It has many advantages. So we should go down to the community and follow them continuously.

I: Can you tell me any programs in place to prevent early marriage?

P: There many activities done. For example, recently, every couple should be registered while marrying. There is a stream committee at the ground that oversees the situation. One, there should be birth certificated from health facility. If she do not have certificate, the girl should bring three witnesses to confirm her age. Sometimes, witnesses may speak truth and some may provide wrong evidence. At them the committee will judge based on the girl’s physical. In such cases, there could be scenarios in which the committee disapproves the marriage if the girl is stunted when she actually is18 years. Thus, in this regard, there many things left undone

I: what about in terms of law? Is there punishment

P: Here in terms of law, there is follow up. Both women affair and justice office are also working on this. A scheduled marriage had been disapproved even when all the food is prepared. The male will also be accused and brought to court. Thus, there are good initiatives. In the community, some of them have good awareness, and still few do not.

I: what are the community factors that affect age at first marriage? What is the community’s perception to early marriage? Some time there could be pressure from the male side; can you explain this?

P: one, it is the habit. But now days, both boy and the girl love each other and come for approval. But, previously, parents were selecting a girl to their son. The second reason is for the sake of benefit. If you relate with that family, you will be well benefited. There is such ideology. Therefore, as a result of these reasons, there is early marriage. We cannot conclude that these habits can be left, because it is culture. Thus, to reverse these, we have to convince with science. You need to explain “under age marriage has such negative impact” and create awareness to the community, and try to inform the government’s direction. If he refuses after this, what is next is to take legal action; because, it is about a life of individuals. We are all under the law.

I: is there any other opportunity to prevent early marriage and increase birth spacing

P: To manage family size, you can tell politically to the lower management. What is needed here is professional support. It can be at individual or group level using the various structures we have, be it women development army, it can be in network and/or in private associations. We can enter to the community and create awareness including the consequences. This can be done by those who have acceptance in the community, like religious leaders and ‘Shimagle adi’ (traditional community judges). If we do this we can bring change.

Regarding prevention of early marriage: this can be done by them (religious leaders and ‘Shimagle adi’ (traditional community judges), be it in Muslim or Christian region mutual understanding is important. Following that, in the community, there is nothing that can be done individually. Because, this is a policy and is already released; you can inform the community at any circumstances created as part of the agenda. Therefore, the community should be aware, as early marriage is backwardness. It is a time of change and the community should be changed. To promote these things, capacity building trainings should be provided. If all are done better change will come.

I: Do you have any other comments that you want to add? Any lesson you want to deliver?

P: what I say is, if all the information you provide it to me is compiled and implemented, there will be a change. I do not have to add as it is prepared, complied and corrected by higher body; everything is touched, there is no anything left to be added. We you hold should be strengthened.

I: Thank you very much for your time and energy!!

**Summary**

**Section1: common maternal nutrition**

Regarding to stunting, the problem is not serving a balanced diet. This is a critical challenge even there are choices, is poor utilization of contraceptive, associated with backwardness.

There are many people who are blind in adjacent Woreda of region three and some in this Woreda.

Home delivery is big problem in the Woreda

Out of the 151 thousand people (total population) in the Woreda 35, 000 is benefiting from safety net.

Mothers are not using the available opportunities. E.g. home gardening

**Section 2: Nutrition priorities in the Woreda**

The committee that controls the water pump is led by women, because women are facing many of the challenges.

Once we produce water, there is no follow up if the community is using it appropriately. By considering home gardening as an activity of agriculture, there is a gap in working jointly.

**Section 3: Nutrition interventions that improve adolescent and maternal health**

There is a program called “education for adolescents”, this is given not only by teachers but also by water, agriculture and health expert. And adolescents are fast to participate in the session. It is planned and is given two times per week

During safety net, if woman has family member of four, she works only for three and she is waived for one. But if the head is male he must work for four. During water and soil conservation, two women are parallel with one man. But if it is food for work, the task is equal for man and woman.

To keep water hygiene, chlorine is being provided every three month to prevent water borne diseases, and biological test is also done.

There is limitation in utilization of ITN as some people are using to cover hey and cereals to protect it from bird

There are no mothers who die of malnutrition.

**Section 4: community factors affecting access to maternal nutrition interventions**

Shortage of budget is a barrier to implement nutritional interventions across all levels.

Pregnant woman should eat food, drink milk, and eat egg and meat. At this time “Why are you eating on Wednesday and Friday? Fasting for forty days is ordered by priest, and this includes children older than seven years. All, these problems, are not yet solved.

There is shortage of ambulance, and the woreda’s geography is also challenging to access it

**Section 5: Multi-sectorial collaboration to improve maternal nutrition**

There is KWASH project led by water resource, but is done in collaboration with health. It works on building latrine, dig placenta pit and make incubator. There are nine kebeles under the KWASH project. There is capacity building training on ODF and CLTH and related issues.

There is no competition in work. When I stand for work, as a management body, I hold all check lists and supervise all activities done by all sectors.

**Section6: Other interventions that influence adolescent and maternal**

Every couple should be registered while marrying. There is a stream committee at the ground that oversees the situation.

Habit and perceived benefit from the other family are community factors affecting age at first marriage.

To manage family size, you can tell politically to the lower management. What is needed here is professional support.
