# Supplementary material for: Barriers to utilize nutrition interventions among lactating women in rural communities of Tigray, northern Ethiopia: An exploratory study
Source: PLoS One. 2021 Apr 30;16(4):e0250696. doi: 10.1371/journal.pone.0250696 (PMC8087028; doi:10.1371/journal.pone.0250696)
Supplement: S2 File — (ZIP) [file pone.0250696.s002.zip › S2_File.Doc/Woreda level and above key informants/076_IDI_MCH expert_Tanqua Abergele woreda.docx]

**In-depth interview guide for with experts** using the Guide for Nutrition focal persons (**Tool A**)

**Introduction:**

Hello my name is Haftay. I am from Mekelle University. Thank you for taking time to speak with us today. We are doing a research on factors that influence the nutrition of mothers and adolescent girls in collaboration with the regional health bureau and UNICEF. Your participation is very valuable. The things that you tell us will be used to improve nutrition programs and services for women and adolescent in the region and in the country. Your names will not share when we report our results.

However, I will record the discussion using and audio tape recorder so that we can capture all the ideas that are shared. I have several questions to ask you that we have prepared in advance, and we will ask you to say what you think about each question. To ensure the privacy of everyone here, we ask you not to repeat what to discuss outside of this group. The discussion will last for 1-2 hours. Do you have any question before I begin? If you think of any question as we proceed, please feel free to let me know. If it is all right with all of you, I will run on the tape record now.

**Section A: Interview details**

1. **Zone**: Central
2. **Woreda**: Tanqua Abergele
3. **Kebele**: Yechila
4. **Name of key Informant**: Desalegn chekole
5. **Institution of key informant**: Head of Maternal and child health
6. **Interviewer’s name**: Haftay Berhane
7. **Date of interview**: 08/11/2017
8. **Interview starting time**: 10:40 AM
9. **Interview end time**: 12:05 AM

**Section B: interviewee professional information**

1. **Gender**: Male
2. **Age**: 45 years
3. **Highest level of completed education:** College education
4. **Current job/Position:** Head, MCH in wereda health bureau.
5. **How long have you been in the current Job/position:** 07 years

**Key:**

**I**: Interview

**P**: Participant

**Section 1: Common maternal (pregnant and lactating women), and adolescent girls` nutrition problems in the community.**

**I**: In your opinion, what are the common nutrition problems in the community for women? What about for adolescent girls?

**P**: The common nutrition related problems in this wereda which mostly seen in children and adolescent girls is not balancing the diet even if the food is available. This is highly related with lack of awareness. While the food is available at home not eating the balanced food is commonly manifested. There are improvements in this regard but I cannot say it is fully addressed. There is tendency to give best food to adults than children. These are the barriers in the community.

**I**: Is it common to see individuals with severe malnutrition in this wereda?

**P**: In our wereda especially stunting is common with prevalence around forty. This is supported by many survey studies and our routine screening services in children. Severe acute malnutrition is common in mothers including pregnant and lactating, in children especially in the age less than five years and less than two years. We do screening monthly. There is wasting, stunting and underweight.

**I**: What about moderate malnutrition?

**P**: We have cases with moderate malnutrition and specially those at early warning are common. We are getting a support from different organization. This support has been continued for many years. Those who are discharged from OTP and TFU which were treated in patient also join the therapeutic supplementary feeding (TSF) program or fafa. The food support continues to three or four months to prevent relapse. This service is given by the health extension workers.

**I**: Do you think this community is suffering from Micronutrient deficiencies?

**P**: Yes. Utilization of iodine is good these days. The community is aware of goiter. The awareness of the community to use iodine increases. It is going good. However, this is not the final activity we can do. We assume there are many duties left to be done in this regard. The micronutrients are present in the wereda. There vegetables since there are irrigations in every place. However, the is a gap in utilizing them properly and there is problem of eating balanced diet. Therefore, there is prevalence of anemia in pregnant mothers. This problem is related to nutrition. Pregnant mothers are not taking food that a pregnant mother should take and hence there are anemic pregnant women.

**I**: What about night blindness?

**P**: It is there but it is not common. This disease is caused by vitamin A deficiency and commonly seen in older adults, and in mothers. The advice that should be given is to take foods that contain this vitamin A. Anyways, it is less commonly seen.

**I**: To whom do you think are these micronutrient deficiencies like anemia, goiter and night blindness affects more? Pregnant, or lactating women, or adolescent girls?

**P**: Goiter is common in women. If you compare women and men, I can it is common in women. Night blindness is also seen in few mothers and in older adults. These two are not common in children.

**I**: Do you think non-communicable diseases like hypertension and diabetes mellitus are common in this wereda?

**P**: Diabetes mellitus is exaggeratedly high. I have tried to list many diabetic patients in this wereda. Those patients are treated in abi adi hospital. I have seen three family members with diabetes in one household. Therefore, diabetes is commonly seen in this community.

**I**: How is the awareness of the community about the cause of these diseases? Do people relate them with nutrition?

**P**: There is awareness in the community about the relationship between the causes of these diseases with nutrition. The health extension workers are continually providing education about these diseases and their relationship with nutrition. However, not all members of this community are fully understood this. Any ways, we are doing to create the awareness of the community to understand the prevention of these diseases.

**I**: Do people in this community suffer from overweight?

**P**: I cannot fully say that we overweight individuals. There are very few individuals with overweight.

**I**: You have said me that this wereda is getting food support. In what situation do you think food insecurity happened?

**P**: The shortage of food is mostly seen starting May to October.

**I**: How frequent does it happen?

**P**: There are kebeles which are most affected by drought. The areas where this drought is frequently happening are those semi lowland areas.

**Section 2: Nutrition priorities in the woreda**

**I**: What priorities do your institution has in relation to maternal and adolescent health related to nutrition?

**P**: The interventions are being done. The main focus is on mothers. Nowadays, the best to male assumption is becoming minimal. Therefore, the children and adolescents are getting better attention than ever. For example, pregnant mothers were not getting what they should have to take. Now we are working focusing on what should a pregnant mother eat, how many per day. If an adolescent girl takes best foods like milk, her weight increase.

**I**: You have told me that you are teaching to pregnant on what they should eat and how many times per day. Lets` put this scenario with examples. What should a pregnant, lactating or adolescent girl eat?

**P**: pregnant women should eat a balanced diet at least four times per day. The diet should constitute foods that are rich in micronutrients. We are working towards this especially in the time of pregnancy. We are advising them to eat balanced diet that contains micronutrients such as vegetables, protein containing foods.

**I**: From the foods that contains micronutrients, which of these are growing here in this wereda?

**P**: There foods like tomato, carrot, orange, mango, papaya. The farmer is growing these fruits. The main obstacle is on the utilization. They are also available in the market. Some areas have shortage of water because of the lowland nature of the area. I do not think it is because of shortage; and I think it is because of poor utilization of these fruits.

**I**: How is the resource mobilization like water supply to escalate the home gardening?

**P**: The government is trying to secure the water source for the farmers. Especially REST is supporting us in those which are highly affected with drought and those which have shortage of water. REST is supporting us. The government is working towards solving the water problem and the coverage is around sixty percent. The government is working to secure clean water supply for drinks. Most water sources are done by REST. In the supply of clean water, NGOs are also play a role. For example, Concern is a NGO which provide us with big water containers.

**I**: What is your institution doing to improve nutrition among women and adolescents? Role of your institution in promoting home gardening, use balanced diet?

**P**: This is the focus of our institution. We are working towards the promotion of home gardening in collaboration with agriculture bureau specially in producing the above-mentioned foods. We are working as health development army in promoting maternal and child nutrition and implementing practical food preparation sessions. These sessions are given in development army, in health post during immunization and other meetings. The agriculture in collaboration with us are promoting home gardens that can easily grow in the area like tomato, mango, papaya, potato etc. We are working towards the plan that a lactating mother should eat a balanced diet five times a day. This is to be done by health extension worker and women development army. We are working in orchestration with agricultural sector.

**I**: You were telling that your institution is working on pregnant and lactating mothers` nutrition. What about adolescent girls’ nutrition?

**P**: Attention is given to adolescents currently with iYCF. There is job done in adolescents especially on girls. The activities could be on prevention of disease, nutrition and others. However, there was no consideration of adolescents in the previous times. We are working with adolescents with the age range of 10 -24 years. There is even an association of 25 adolescents in collaboration with the school to deal with HIV/AIDS prevention, nutrition, family planning. We have trained manpower and the action is started but much is needed to be done in this regard.

**I**: What are the gaps that should be focused in maternal and adolescent girls` nutrition?

**P**: The main problem is related to our mothers thinking. They do not want to give delicious food to adolescent girls. We should work on this issue. For example, one mother may have a moderate malnutrition and given a food support, she tends to share the fafa to her children. We must work on sharing. There is a problem of mothers on utilizing the micronutrient containing foods.

**I**: Who is eligible for fafa in this area?

**P**: Fafa is given to individuals who are with moderate malnutrition after a routine screening. Therefore, mothers and others on moderate malnutrition are linked to targeted supplementary feeding (TSF). If pregnant and lactating mothers have MUAC below 23 after measurement, they will join directly to the TSF.

**I**: Can a woman be given fafa because she is pregnant or lactating?

**P**: No. She will not. If her MUAC is less than 23, she will be eligible otherwise she will not. If a pregnant mother is with normal weight, we advise her to eat diversified food after assuring her and her fetus health is in good condition.

I: When is the screening done?

**P**: Screening is done every month from day 14 to day 16 and the health extension workers do daily screening. They go home to home and there is also the monthly screening.

**I**: How do you evaluate the activity done in mothers regarding nutrition?

**P**: We did a good job in the antenatal care follow up starting from zero to some number. The good success is the antenatal care four (ANC-Four) follow up which was around 40-50 and currently increases to 85-90 percent. Mothers are getting advice about nutrition in ANC follow up. The delivery service also good with skilled delivery by professionals and home delivery is minimal. The advice and support about nutrition is given in the ANC follow up. It also helps to bring behavioural change after the counselling and support. These are the changes. We evaluate ourselves at a good state. Even if home delivery is not zero, it shows remarkable change. In the ANC follow up, pregnant mothers are advised about danger signs, type of food they eat by professionals. There is also advice in the postnatal care follow up about type of food, how many times should she eat, how many times should she feed the child.

**Section 3: nutrition interventions that improve adolescent and maternal nutrition.**

**I**: You have told me about the need of extra meal for pregnant mother? How are pregnant mothers in your wereda doing? What is the role of the husband in this regard?

**P**: Not all mothers are getting enough meal. There is also a shortage of food. There are mothers who do not get food. There are also mothers who do not apply what they have been advised to do in the presence of food at home. Anyways, not all mothers are getting enough meal. This is a problem. Males are not supporting their wives including provision of nutrition in pregnancy and lactation. There are also good husbands who care their wives that apply the advice and support given to them. Anyways, the role of husbands has limitation as it is seen on the ground. We advise pregnant mothers to take one extra meal. This means, if she was taking three times per day, we advise her to take four times per day. However, there are mothers who do not apply this. Not all pregnant women are applying this even if there is enough food at home. The problem may be due to lack of awareness. We must work until the mother applied it by herself. The thing in lactating mothers is also the same. I have told you that there are mothers who do not have food but there are women who understand it and apply it and there are also women who do not want to apply it in the presence of enough food. There are mothers who care for themselves and for their children. They are also given education on the benefits of maternal nutrition on the fetus development and especially on the one thousand days, the food is for both the mother and the fetus. Mothers have enough awareness on this regard. However, there are mothers who assume eating a good meal will lead to increased weight of the fetus and then lead to difficulty in delivery.

**I**: Well. Do you do screening for adolescents?

**P**: Yes. But they are not our targets. Our targets are under five children and mothers both pregnant and lactating. However, we also include those adolescents who are sick with shortage of food.

**I**: You have mentioned that you are counselling pregnant women on food diversification? How is the practical scenario here? do you think they accept it?

**P**: Mothers are applying good in using balanced diet including use of ionized salt. We are also showing them practically. We are milling three different items including cereals and showing them how to do it. Mothers are now accepting it and applying it even to feed their children. We are assessing using check lists whether mothers are preparing this diversified flour for one or two weeks by visiting home to home. When we visit home to home, there are women who apply it but there are also who do not apply it. When we advise women to use iodized salt to prevent goitre and to give birth to a child with brilliant mind. Despite this counselling, there are women who use the normal salt without iodine.

**I**: Is iodine salt found abundantly? How about the cost?

**P**: Yes. The cost is also acceptable which is not more than 8 birrs. It is introduced currently.

**I**: Why do think are women not using iodized salt?

**P**: There are women who prefer the normal salt than iodized. The cost of the normal salt is even higher than that of iodized one. Iodized salt should be added after the stew is starting to cool. There is lack of awareness that some of them added the iodized salt just while the stew is cooking and some added with paprika. There are also some who do what they have been told to do. The highest number is seen in those who use the iodized salt properly. We cross check whether women are applying it or not using the pack of the iodized salt. Anyways, generally the awareness is becoming better.

**I**: How do women and adolescent girls in this wereda are benefited from safety net program?

**P**: women including pregnant and lactating and children are benefited from safety net program. Pregnant women are included in the program from the day of confirmation of their pregnancy. We work together with agriculture sector in safety net program. A lactating mother benefited from safety net program if she has a child with malnutrition. This mother will be included in the program and she will not participate in the water and soil conservation to care the child with malnutrition. We have done this for about two years. The mother will delegate her older child or husband to work on behalf of her.

**I**: Could a mother be benefited from the safety net program because of her pregnancy?

**P**: No. It depends on the income status. If there is shortage of food because of drought or other factors, it will be given priority. There is safety net program called emergency aid for older individuals. If a mother is having enough food and if the farmer can produce enough food supply, they will not be included in safety net program.

**I**: Pregnant mothers are benefited from safety net program without involved in the soil and water conservation to take rest. Did you advise them to take rest in their home, too? And do they apply it?

**P**: The rest is given for lactating mother with malnutrition child to care the child. If the child is with severe malnutrition and is on plumpy net, she will be free of work to support the child. She will be given advice by professionals. If the child is admitted and being treated in patient, she will get advice from health professionals while she is on the health center. Plumpy net is a medicine and she should administer the plumpy net to the child as prescribed. If the mother is pregnant, she must come to health institution to check her and the fetus`s health. She will be given advices on what food to eat and get rest in collaboration with agriculture.

**I**: In one of the study are, I have got mothers working at home more than the work expected by safety net program. How do you see this condition in this wereda?

**P**: You are right. Women are working beyond their capacity. When pregnant women are advices to work light and easy work, but they get involved in heavy work at home until they give birth.

**I**: What are the jobs done on sanitation and hygiene services in this wereda?

**P**: This personal and environmental hygiene is given attention by the government is being started to be implemented in this wereda. A mother should keep her personal hygiene from her pregnancy until she gives birth. We are advising farmers to use latrine, prevent infection, to have hand wash basin. On the ODF (open defecation free) program, we want all to have latrine, hand wash basin, and keep personal as well as environmental hygiene. In ODF, the kebele will declare to be zero open defecation as this is causing different diseases.

**I**: Is malaria common in this area?

**P**: Yes. Malaria is common in most parts are of this wereda. Malaria is common here starting from June to December.

**I**: Are women including pregnant and lactating getting advice on the need to use Insecticide treated bed nets?

**P**: Yes. The government is providing us enough bed nets. Therefore, all house hold is owner of a bed net. There are individuals who properly use the bed nets and there are also individuals who do not use it properly. Mothers and children should get priority in getting these bed nets and the spraying of insecticidal to prevent malaria.

**I**: What do you think the reason behind why individuals are not using the bed nets properly?

**P**: There are individuals who do not use the bed nets and if you ask them why, they just pretend as they are using it. If is winter, they want to defend you that malaria can attack at summer times. There are also different reasons like the bed nets can create sense of hotness and it cannot kill bedbugs.

**I**: How do you think farmers keep the hygiene of the insecticide treated bed nets?

**P**: When we distribute the bed nets, we advise them not to wash it with chemicals like soap. We also advise them not to sell it. The health extension workers are also giving advice with home to home visit. The health extension workers are visiting at least 20 homes per day and 200 homes per month to check these bed nets and sanitation practices.

**I**: Who is responsible for distribution and advice on bed nets?

**P**: First the bed nets will be supplied to wereda and it will be distributed based on the number of house hold. If the member of the household is one, the individual will receive one, if two they will receive one and if three they will receive two bed nets. The last distribution is done by health extension workers supported by surveillance on the number of the households.

**I**: What about deworming services?

**P**: It is given in extensive way. It is given in routine service and campaigns. Especially for under five with age of 24-59 months. This is given monthly and as campaign every year. Azithromycin is given before three years. This drug is effective for five years.

**I**: Do you think adolescent girls aged from 10-19 years are benefited the deworming service?

**P**: Yes. All are utilizing the service. Adolescent girls in the age range of ten to nineteen years are benefited be it in immunization or in school. They are also given iron. Iron folate was given last year to those of age 10 to 19 in school.

**I**: How do you address to the out-school adolescents?

**P**: They were also included with the help of health extension workers. The community is organized in the form of one to five network with twenty-five to thirty individuals in one developmental army (Limat gujle).

**I**: How is Targeted supplementary feeding (TSF) being applied in this area? You have told me about this but I want more detail.

**P**: Our targets are under five years age which are 6-59 months of age and mothers who are pregnant and lactating until six months. After six months, the focus is the infant. If the infant has moderate or severe malnutrition, the mother will care the infant. We are doing good in those targets. Vitamin A supplementation is also our target and will be supplemented to 6 -59 months age. Deworming is also our target that will be given to 24-59 months. Vitamin A is given twice a year. But screening is done every month. After screening, we may get MAM (moderate acute malnutrition) or SAM (severe acute malnutrition). If a child with SAM, the child will be treated in the OTP and visit the health facility every two weeks. If the child can be treated in the outpatient department, plump net will be supplied. If the child is not improving, it will be referred to health center for TFU. We have not child died of this malnutrition. There is enough supply of plump net. Here, Unicef is supporting and the government is also giving attention to the issue. We have many donors. The performance of GMT, screening, deworming, Vitamin A supplementation is currently greater than 90 percent.

**I**: Where is the supplementation of vitamin A taken place?

**P**: It has its own program. It is given monthly in the nearest kebele. It is given in pre- scheduled manner. The health extension workers also do home to home visit for support and follow up.

I: How is the youth friendly services at health facilities doing? Are adolescent girls linked to this service?

**P**: All health facilities are started the service and it is with hundred percent coverage. There are trained man powers on this regard. We have also association of youth. We are supported by UNFPA in distributing televisions for enjoyment program. We have a youth club which consists 25 students. The youth friendly service is given at OPD level for those age 15-24 years. Therefore, all health facilities are doing this service hundred percent except those who faced room overcrowd.

**I**: Do you think the use friendly service is inclusive to those who are to school and out school adolescents?

**P**: There was a gap to include adolescents who have finished their study and those who stop their school at grade 8. But currently, we have twenty-five members of the club from both school and out school adolescents. In the past days, we were only focusing students and those living in urban but we have notices it later to include out school and adolescents from rural area. The committee is working in creating awareness on HIV/AIDS, family planning, condom use. These adolescents are working to create awareness on the community with drama. The service is also a separate service to help those adolescents and get whatever services they need. This is supported by former name IFHP and now with the name transformation in collaboration with the government. The government is also giving attention to it and trained individuals to provide the service.

**I**: Do adolescent girls benefited from this youth friendly service?

**P**: Adolescent girls are equally participating in the club. Adolescent girls are involved in prevention of unplanned pregnancy, and abortion. Abortion can cause additional disease and we are currently working with the club to strengthen the prevention of unwanted pregnancy.

**I**: Which of the above interventions like deworming service, Targeted supplementary feeding, vitamin A supplementation, use of insecticide treated bed nets, for the pregnant women are being implemented in an effective way?

**P**: All the interventions are effective and useful especially for women and children

**I**: What about for the adolescent girls?

**P**: Adolescent girls are supplied with iron supplementation. This helps them during delivery and strengthening the pelvic bone.

**I**: How do the adolescent girls accept the supply of iron supplementation?

**P**: A girl starts menstruation at the age of ten. The iron supplementation is done in consideration with this. In the acceptance, there is shyness and so on. They are advised to use the iron supplement when menses starts but there are situations where these girls are not willing to use it. This is the obstacle we are facing.

**I**: Is there misconception that these adolescents can consider the iron supplementation as contraceptives? Will this condition can hinder girls not take the iron supplementation?

**P**: There are adolescents who use contraceptives and iron supplementation. The challenge we are facing is not using condoms which will help them to prevent from disease and unwanted pregnancy. Abortion is common because they are not using condom. When we give the iron supplement, we counsel them the importance of taking iron like prevention from anemia. Additionally, we also advise them to take iron containing foods. We counsel them the difference between contraceptives and iron. This counselling is mostly given in the youth friendly service.

**I**: How do you then evaluate the success of iron supplementation?

**P**: It is almost good. In the last two years, there was a good supply, but currently not. I cannot say iron supplementation is successful like the other interventions and hence, we have many things left undone.

**I**: From the above-mentioned interventions, which intervention do you think is successfully implemented?

**P**: Screening and awareness creation about screening is one of the successful interventions. GMP services are strengthen and mothers are aware of it. Mother are bringing their children to get the services properly. There are obstacles that mothers may not understand when they are told their baby is well. The successful interventions include vitamin A supplementation, deworming, awareness of mothers on screening.

**Section 4: Implementation challenges and** **Community factors affecting access to nutrition interventions**

**I**: What are the challenges to implement delivering the nutrition related interventions that we have been discussing for the pregnant and lactating women, adolescents?

**P**: It was in the previous times but currently, there are no obstacles.

**I**: What obstacles are there may be related to supply, awareness of mothers and others to hinder the implementation of nutrition related interventions? Let`s take lactating mothers.

**P**: The main obstacle is sharing. The child with malnutrition will not come to normal state where there is sharing to other family members. We have noticed sharing on the supervision and support we did on home to home basis using a check list. If you take plumpy net, mothers tend to share it to other children. Thus, there is much to be done on the awareness creation.

**I**: What about adolescent girls?

**P**: Adolescent girls in rural area are not allowed to take good meal like milk to prevent increasing in body weight. There are cultural habits like this. There many things that are not done to adolescents specially living in the rural area. In fact, there is a network for those age greater than fifteen years in one to five. In their network they are advised what to do on food. But in general, adolescents on school are better than out-school.

**I**: Are there food taboos for pregnant, lactating or adolescent girls in this area? Are there culturally prohibited food items?

**P**: Previously, delicious foods like milk were not allowed for young girls. This is because, these foods were considered to increase the development of these girls so that they can start courtship with males...(smile)... delicious foods were also not allowed for children. But these backward cultures are decreasing from time to time. Mother also apply butter and manure on the cord of new born baby. These are the obstacles. The community prevents from giving delicious foods to young girls because it will them to mature early and then put then sexually active and may be marriage.

**I**: How is early marriage in this wereda?

**P**: There is a job done on this regard. There are women affairs who give attention to early marriage. They are working with us and supporting us. They have intervened on incidence of early marriage. Early marriage is not declined to zero.

**I**: Is there any relationship between educational status of the women and access to the interventions mentioned earlier?

**P**: Anyways, education is basic tool. The education related to health professionals, education or agriculture. Education has a role. Those who attend to school are not similar with those who do not attend. For example, in awareness creation, those who attend school may accept the education easily than those who do not attend school.

**I**: What about in utilizing the intervention?

**P**: It is the same. The educated one will accept and utilize the service than those who do not attend school. The literate one will easily accept changes and counselling than those who do not attend school.

**I**: What other factors are inhibiting implementation of the interventions by mothers, and adolescent girls?

**P**: These things are decreasing. Especially in women, there were religious leaders who consider institutional delivery as sin. There was an argument that St. Virgin marry is at home and why mothers should go to health institutions for delivery. There are still elders who share this idea but it is decreased by 70 -80 percent. There is an improvement.

**I**: How do you think the improvement comes?

**P**: It is by giving education and advise. It can be solved by deep discussion with some part of the community. For example, the prohibition of delicious food to young girls was the main obstacle but it is currently solved. Women have tried to understand the situation.

**I**: Are the interventions culturally acceptable?

**P**: Currently there is may be hidden activities but they are not seen in the public. Anyways, there are few individuals that oppose these interventions. We know them personally. One of the obstacles are religious leaders. These leaders are widely accepted by the public. But, most of them are already changed these days. We are now working together and they are supporting us. However, there are also few religious leaders who should be changed.

**I**: Do you think that religious leaders allow pregnant women to get extra meal in fasting times?

**P**: They do not allow them. Even if we advise them that pregnant women and lactating women should eat extra meal, they do not allow them. Orthodox religion leaders will not allow women to eat in the big fasting times like The Great Fast (Lent), The fast of the Assumption of the Holy Virgin Mary. This is one of the challenges we have.

**I**: Are the interventions accessible to women and adolescent girls?

**P**: Supply of the interventions is good. They are all available.

**I**: What about in terms of transportation and cost?

**P**: In terms of cost, we do not have such challenge. The reason is one, the supply of the interventions is covered by donors. In terms of transport, we are using what we have but there is clear shortage of transport. For example, the provision of bed nets to end users is not done on time partly because of lack of enough transport.

**I**: What about the quality of the interventions? How do you explain it?

**P**: We are now working towards introduction of quality. We are now doing the client satisfaction including on time service, quality service and fairness of the service. But there are missing elements to satisfy clients and we are doing our best to fill these gaps?

**I**: What are the gaps?

**P**: Medicine supply is not enough. Therefore, we have to supply enough medicines if we are going to satisfy clients.

**I**: What other factors are inhibiting implementation of the interventions?

**P**: The supply for deworming and plumpy net is enough and there is no shortage. The government and the non-governmental organizations are working had in had to provide these supplies. We do not have shortage of medicines for children and mothers, drugs and supplies for ANC, iron.

**I**: What about the commitment of staffs?

**P**: the professionals are working with mothers and health extension workers are mainly with the community. The professionals are working in urban or rural areas to serve the community. There some who do not do such activities. The awareness is relatively good, where professionals are working to help mothers in the outreach programs. This is done in an organized way. We call it health development army. Even if there are some to be improved, generally the commitment of staffs is good. When we check mothers on the services provided by the professionals, most of them know and apply what they have been told but there are also few who do not understand it. This shows the commitment of the professionals and other sectors.

**I**: How are you doing in expanding best practices in mothers? If a mother is applying all interventions mentioned above, how are doing in promoting this practice so that this mother can teach others?

**P**: We are applying this. For example, we use best experience sharing in environmental hygiene. If there is model kebele in environmental hygiene, we share the experience to the low performing kebele in collaboration with other sectors including participants from the community. In network, there is experience sharing in antenatal care. If one network is good enough in hygiene, nutrition and prevention of diseases and fulfils all components will teach low performing network. But we do not do much work in expanding this activity.

**I**: Why are you not successful in expanding the best practices?

**P**: We cannot say there is no gap in the community or we just totally change the community. There may be additional tasks that hinders the expansion of the best practice. But we have started it. The network with lowest performance will be gaining best practice from best performer network.

**I**: You have told me that there is some work to be done on adolescent girls. You have also mentioned the challenge you faced in transportation. What does your institution do to solve these challenges?

**P**: There is shortage car or motor cycle. If the activities are to be accomplished, health centres should own motor cycles. The government is providing us motor cycles but there are old motorcycles and there is delay in maintenance. Anyways, the solution is maintaining the current available motorcycles. The other solution is collaboration with other sectors. The other is requesting to the government. We have enough ambulances for mothers. We use these ambulances for children under five years have developed emergency disease like pneumonia. Mother are also supported well. But these ambulances are not enough to six health centers. I prefer every health center to have one ambulance so that it can serve mothers commining to that health center. One ambulance is covering a large area around 80-90kms far. To meet the goal for decreasing maternal and child death, there should be transport services. We are using the cars assigned for sectors for mothers. The administration is giving attention to mothers and they are given priorities. We have tried to solve the shortage by this kind of collaboration.

**I**: What are the obstacles for adolescent girls?

**P**: it is not difficult to address girls in schools but the challenge is those out-school girls. In fact, the out-school girls have also their own grouping or network. We may not get the out-school girls hundred percent because of the nature of their job but the awareness is becoming good. Therefore, in the previous times, it was difficult but currently it is somewhat good. This is also related to the increased awareness among the community. It is not as such difficult to meet these out-school girls but the problem of early marriage is a problem in these population.

**I**: In your opinion, why would delayed marriage (after 18 years) improve maternal nutrition?

**P**: We cannot raise it as a big weakness. The nutrition in adolescent girls is currently becoming healthy. If you these times, adolescent girls are eating whatever they think is good. Early marriage is having big problem especially on the female. If she gives birth at age less than 18 years old, she will be hurt. The nutrition of these girls who are early married and delayed married is the same. The difference is the early marriage. If there is early marriage, there will be birth at early age. If she gives birth while she is fifteen or sixteen, she is not matured. This is done in collaboration with women affairs.

**I**: What about birth spacing? why would increase the gap between successive births improve maternal nutrition?

**P**: Death of mothers and children is mostly caused by giving births without appropriate spacing. This birth spacing has also economic impact. She is producing children. This is related with the bad culture that promotes `child will grow by chance`. This culture is still there. Males are supporting in this regard. There are women who take contraceptives hidden. From the mothers died in our wereda, all are women who do not space births. There are mothers who give birth to six and eight children at the age of forty. Nowadays, most women are changed and they give birth after four to five years. There are also mothers who use contraceptive without the knowledge of their husbands. We need to do more on this regard. There are some religious leaders who do not accept birth spacing. Anyways, if a mother is giving birth without spacing, the hurt to herself and the baby is higher. The child will not get enough breastfeeding, love and the child will be at risk of infections. There are women who get pregnant just before the uterus returns to its place and before they become strong enough to do so. Seventy to eighty percent mother are aware of the advantage of birth spacing and using the contraceptives.

**I**: Is there any program or intervention done to promote the birth spacing in this wereda?

**P**: Mothers have many choices to choose. The supply of contraceptives is good. The mother is advised by health extension workers and women development army on the available choices and their advantage as well as disadvantage. We are promoting this because it will have an impact on decreasing maternal mortality. If a mother is giving birth now and then, it is a problem. The mother will not participate in harvesting and the mother will produce children. The children and the mother will be at risk of infection and death.

**I**: what about prevention of early marriage?

**P**: We are working with women affairs on early marriage. We have cancelled marriage of adolescent girls in collaboration with women affairs with different witnesses. We have tried to convince the girls and family.

**I**: What are the factors that promote early marriage?

**P**: The is the culture. Families claim that their girl should not wait until puberty because she will be expected to create a friendship with male. They assume as she will be at risk.

**I**: What should be improved in this regard?

**P**: There should be discussion with the community and there should be possible agreement. You should educate the community as early age is danger. There is risk of death in these girls. Therefore, you should advise them by comparing the risk of marriage at age of fifteen and at above eighteen. We must work with elders and religious leaders as the community has the highest believe on them. We are starting to do this practically.

**I**: Do you think of there are any other opportunities to prevent early marriage and increasing birth spacing beyond the current intervention on these?

**P**: It is better if we strengthen the current intervention. Agreement, awareness and advice are important. There is visible change from time to time. However, it is good to work with husbands. Because there are husbands who do not want the use of contraceptives and birth spacing. Like females, we must also do remarkable work on males in continued manner.

**Section 5: Multi-sectoral collaboration to improve maternal nutrition**

**I**: Do you feel it is necessary at your level to work with other sectors/institutions to address maternal and adolescent nutrition?

**P**: Yes. For example, if a mother is to eat balanced diet we must work with agriculture to deal with what should she eat and where should the food items grow. There should also be plan of both collaborators. There should also collaboration in implementing the plan. There should also be collaboration with school to deal with school adolescent girls. There should also be collaboration with water supply to prevent water diseases caused by problem of sanitation and hygiene. We must work with water supply on what type of water we have and whether the water pumps are working or not. These are the main collaborators in health. There should also be collaboration with trade and industry to deal with food items that are expired. We have already started the collaborative work especially with agriculture that deals with balanced diet including vegetables and irrigation, home gardens that contain essential micronutrients.

**I**: Did you start to work with all sectors that mention them?

**P**: Yes of course. We have even collaborative plan. We have a committee lead by wereda administrator and the vice head is wereda health office.

**I**: Which other sectors do you feel are necessary to work with your institution?

**P**: We have ten to eleven sectors in the multi-sectoral collaboration. Associations like women association must be members in the committee to promote their duty. If we are going to prevent early marriage, we must discuss with women association and create interface.

**I**: How do you see the other institutions’ roles complementing your role in improving maternal and adolescent nutrition?

**P**: The role of agriculture is effective. We are supporting each other on the TSF. School will have a great role in awareness creation, family planning, HIV prevention, other diseases prevention, and nutrition. School is also supporting us in food demonstration. If there is no clean water, you will be exposed to different diseases. We are working with water supply to maintain and respond to any problem related to water. These are the main collaborators. Women association are working with us in orchestrated manner from this kebele to network.

**I**: How do you evaluate the collaborative plan in improving nutritional interventions?

**P**: Yes. If you work alone, you will not be effective. For example, in deworming services if we are not working in collaboration with kebele leader, and agriculture it will not be effective. If you do not work by integration, you will not be winner.

**I**: Do you think there is anything to be added to strengthen the multi-sectoral plan?

P: It is clear. The government has taken this as priority and make it clear. The government has clearly put which sectors should be included like trade and industry, all women associations, water supply, school, agriculture. At least eleven of the sectors in the multi-sectoral collaboration are evaluating the plan of action.

**I**: What needs to be done to improve the capacity of these bodies for effective coordination?

**P**: Capacity building is given by the government and the donors. For example, there is a training where health, school and water resource and supply are involved. After the training, all trainees from each sector will transfer the knowledge and skill to others under their jurisdiction. For example, we are linked with agriculture by different aspects. We have trainings where we took together.

**I**: What opportunities do exist to promote multi-sectoral coordination of nutrition in this woreda?

**P**: We have tried to form different committees. These committee will take leadership role. We have common committee which consists members from sectors like health, school even in kebele. It is linked to the networks of developmental army. We are giving support and follow up to this committee. The capacity building is being done in an organized fashion starting from the higher bodies and is linked to lower hierarchy up to the network (Limat gujle).

**Section 6: Other interventions that influence adolescent and maternal nutrition and health outcomes**

**Section 7: Additional Remarks**

**I**: Do you have any other comments on anything that we have discussed in the area of nutrition in mothers and adolescent girls?

**P**: In the area of mothers and children, we are having shortage of medicines for children. The government is declaring the treatment for pregnant women to be for free but we have much expenses. Additionally, this area is mostly affected by drought. Currently, most of our kebeles are affected by drought and need clean water supply. The areas in the tekeze basin are affected by shortage of water while is tekeze there. Strong attention should be given to the shortage of water as most diarrheal diseases are caused by lack of hygiene and sanitation.

**I**: Thank you for taking the time to discuss these issues with me today. I have learnt a lot from you. As I mentioned as the start of the discussion, I will remove all identifying information from the report of this conservation. I will make you sure that no one can identify your comments. If you have any concerns or questions, please feel free to ask me any questions. Thank you very much for your time. You cannot say everything is clear and applied by the community but there is improvement from time to time.

**Summary:**

**Section 1**:

- Stunting is common in the wereda
- Lack of awareness is the common reason for the occurrence of nutrition related problems
- Males are favoured by the culture of the community. Males are given the first priority to eat the best food the household has.
- Anemia is common in pregnant women
- Diabetes mellitus is common in the wereda
- Drought is common in the semi lowlands of the wereda

**Section 2:**

- Pregnant women are advices to take food four times a day
- Home garden that grow in the wereda include: Papaya, Tomato, Carrot, Orange
- The common challenge in women and adolescents` nutrition is sharing
- Food insecurity is common in the wereda and the wereda is benefited from the support of safety net program

**Section 3:**

- Adolescent girls are not target of the wereda nutrition agenda
- Pregnant and lactating mothers are utilizing the safety net soft conditionality including getting food support and rest.
- There is no problem in supply of plumpy net.
- Abortion is common because adolescents are not using condoms.
- Nutritional counselling is included in the antenatal care follow up counselling
- Screening and awareness creation are among the successfully implemented intervention.
- Unproperly use of insecticide treated bed net is common in the wereda

**Section 4:**

- Adolescent girls in rural areas are not allowed to take milk because of the fear of early organ development and sexual maturity
- Since religious leaders are highly accepted by the community, the wereda is planning to work together with them to overcome the misconception about birth spacing, early marriage and taking food during fasting time.
- It is difficult to get out-school adolescents for implementation of interventions because of their mobility
- Early marriage is common in the wereda
- Husbands are one of the obstacles for implementation of nutrition related interventions like extra meal and home gardening

**Section 5:**

- The string committee for nutrition in the wereda is consisting of beyond eleven sectors in it including health, agriculture, water resource and supply, women affairs etc
- For effective coordination of multi sectoral nutrition, capacity building is essential.

**Section 6**:

- It is already addressed in section 4 in details

**Section 7**:

- There are kebeles in the wereda which are highly affected by drought and shortage of water and hence, the government should focus on water resource for such kebeles
