# Supplementary material for: Barriers to utilize nutrition interventions among lactating women in rural communities of Tigray, northern Ethiopia: An exploratory study
Source: PLoS One. 2021 Apr 30;16(4):e0250696. doi: 10.1371/journal.pone.0250696 (PMC8087028; doi:10.1371/journal.pone.0250696)
Supplement: S2 File — (ZIP) [file pone.0250696.s002.zip › S2_File.Doc/Woreda level and above key informants/077_IDI_head for Youth office_Samre woreda.docx]

**Operational Research on Adolescent and Maternal Nutrition in Northern Ethiopia**

**An In-Depth Interview with Deputy of Youth and Sport Office**

**Introduction**

Good morning. Welcome and thank you for taking the time to speak with me. I am Hailemariam Tekie from Mekelle University. I came here today to study the factors that influence the nutrition of mothers and adolescents in collaboration with the Regional Health Bureau and UNICEF. As part of the community, no one knows more than you about the problems existing on adolescent girls within the community and also possible solutions for the problems. So, your participation is very valuable. The things that you tell me will be used to improve nutrition programs and services for women in the region and the country. Your name will not be included in the report. But, in order to capture all the ideas that you share me, I will record the interview. The interview will take about one and half hours. Do you have any question before we begin? If it is all right with you, I will turn on the tape recorder now.

**Section A: Interview details**

1. Zone: South-Eastern
2. Woreda: Saharti Samre
3. Kebele: Samre
4. Name of key informant: Shishay Haileselasie
5. Institution of key informant: _______
6. Interviewer name: Hailemariam Tekie
7. Date of interview: 14/11/2017
8. Interview start time: 9:46 am
9. Interview end time: 11:55 am

**Section B: Interviewee professional information**

1. Gender:
2. Female
3. **Male**
4. Age: **34 years**
5. Highest level of education:
6. No formal education
7. Primary education
8. High school
9. College education
10. **Bachelor degree**
11. Master’s degree
12. Current job/position: **Deputy of Youth and Sport Office**
13. How long have you been in the current job/position?
14. _____ Months
15. **One Years**

**Detail of the In-depth Interview**

**Key:** I – Interviewer ; P – Respondent

**Section 1: Common maternal (pregnant women, lactating women and adolescent girls) nutrition problems in the community.**

**I: In your opinion, what are the common nutrition problems in the community for women? What about for adolescent girls?**

**P:** Ok. I am not from that profession but according to my observation, one thing is that it is this time that there is an improvement otherwise I cannot consider that women are using appropriate feeding practices. This is to mean that I do not think all including the farmers understand the health benefits of the agricultural products such as meat, egg, fruits and vegetables. They only produce products that commonly are consumed at household level. The one I can say a problem for women is in relation to lack of diverse and nutritious foods. Some of them could probably be with the existence of diverse foods. The second problem is due to the lack of processing and preparation of safe foods.

**I: Ok. You told me what women do but can you also tell me what the problem are that comes after this conditions?**

**P:** I do not know much about this but ahhh.

**I: For example, if children do not eat nutritious foods, they may be thin, short, and underweight which is not proportional to their age. The other thing is in relation to different diseases such as anemia, night blindness, and goiter. So, what do think the problems in women and adolescents?**

**P:** As you said there is a science that explains about dwarfism and as to my observation in these areas, there are many incidence of wasting in children because of the reason that they do not get nutritious foods especially in the lowland areas of Finarwa kebele. The second thing is the women themselves are observed to be undersized and are facing problems during child delivery. Similarly when these kinds of women give birth, the child is found to be underweight.

**I: What about in adolescent girls?**

**P:** I do not know about that.

**I: What about micronutrient deficiencies?**

**P:** There are problems related to micronutrient deficiencies though its coverage is not considered to be high. Otherwise, there are some incidences that we observe in adolescent girls as well as in boys. For example, I know two boys who have the problem of goiter.

**I: What could be the cause of that problem?**

**P:** I do not know.

**I: What about overweight in this community?**

**P:** There is acromegaly in children or infants but I do not think there is overweight in adults in this area except that of wasting. There could be some but there is almost no problem of overweight in terms of coverage.

**I: What about diet related non-communicable diseases such as diabetics and high blood pressure? The problem is increasing in urban areas. So, is it becoming a problem in this community?**

**P:** There are some in relation to high blood pressure but according to my observation in the woreda, I do not think it is a major problem. Similarly, there is incidence of diabetics in the areas to some extent. There is a problem of diabetics in some individuals but it is not expanded and is not to be considered as a threat to the community. Unlike these problems, there is eye disease in the areas. This was observed to be a disease affecting many people due to the events organized by volunteer doctors from abroad to treat people with eye diseases in the areas. During the day, there were many people who made a long line to use the opportunity. I have seen a lot of children, adolescents, and women.

**I: What could be the reason for that problem?**

**P:** It is somewhat night blindness in many of the people. I think the cause of the problem could be because of lack of sanitation related problem such as toilet, personal hygiene and sanitation.

**I: What do you think will be the reason for lack of good hygiene and sanitation?**

**P:** Yes. Actually, water is a big problem in these areas. There are areas that travel a long distance to get water, for example Maykana kebele do not have water and then have to travel a long distance. Here there is World vision, a project that is collaborating with the government and has tried to supply water from another Kebele to Maykana kebele but still it could not be functional to solve the problem of water for the community. So, if the people could not get adequate water, it will be difficult for them to keep person hygiene and sanitation. Similarly, water is considered to be a big problem in other kebeles as well. With regard to awareness, I can say most of the people are aware of because there are different awareness creation sessions delivered for the community. There is also a habit that the people do not practice the right way due to lack of commitment or negligence though they know what to do.

**I: What about the food security condition of the woreda?**

**P:** Now the government is implementing the strategies to assure food security. One of the food security strategies is safety net program. Households who are considered to be poor are involved to improve their food security in line with the rehabilitation of the areas. The other strategies are applying the agricultural packages such as irrigation facilities, livestock, and use of agricultural inputs to improve food security. Now the food security status of the areas is almost to be ensured. Those poor mothers having different problems because of different reasons and also adolescents are supported by the government and NGOs.

**I: What do you mean by support? Is it aid or safety net?**

**P:** For those that are able to work will not be supported for free. They can only be supported in case there is a problem like that of drought. So, when there is extreme drought, there an emergency aids to support the community. But those that are unable to work, disabled individuals, having internal problems, and women that are not on the state of fitness for work are provided with these opportunities. There is screening to determine who should get free aid through safety net without working and also who should get the aid through doing the activities under safety net. In addition to that, there are also opportunities to facilitate the credit services through the saving and credit institutions for those who wanted to work to improve their food security.

**I: Do you think women are especially at the risk of malnutrition you have mentioned above?**

**P:** Yes. According to my observation, women are the most affected groups because they have a work burden at home doing so many activities the whole day in addition to the child delivery and other responsibilities. So, if they do not get nutritious foods properly, they will be affected more than males.

**I: Which women groups are most affected? PW, LW, or adolescent girls?**

**P:** The risk of malnutrition due to nutrition will be higher in pregnant and lactating mothers because it is well known that when a woman is pregnant or lactating, she needs additional foods but if she could not get foods and do not follow the proper feeding practices, the problem that can take place on the mother and her infant will be higher. Relatively, it is better in adolescents.

**I: Do you think adolescents follow better feeding practice?**

**P:** I do not mean the adolescents are getter better foods but as the pregnant and lactating women need additional foods and they are also having additional work loads, they are the most affected groups.

**Section 2: Nutrition priorities in the woreda**

**I: In your opinion, what priorities do your institution has in relation to maternal and adolescent health? Why?**

**P:** According to our institution, Youth and Sport affairs office, youth includes both male and females. Both males and females in the age category of 15-35 years old are considered to be youth. The participation and beneficence of the youth can be explained in three categories. We are working on economic, political and social sectors. So, the question that you are asking me is related to the social sectors. If we take social issues, one thing is that if we empower the women economically, we are also creating opportunity to improve the consumption of nutritious foods. The second thing with regard to social issue is that we have a mission to expand the medium aged youth. In the case of this, there is peer to peer discussion about education, reproductive health, nutrition, and other and these will help adolescents to improve awareness on these issues. In line with this, we have 11 centers (under government, we have 2 centers: Samre and Giject woredas), and also 12 kebeles with family planning in the area of health, reproductive health centers in the woreda. So, there are awareness creation sessions made in the center to improve their participation and get benefit from the centers and facilities. It is through the discussions made about nutrition and on how to handle health condition that we follow to assure a productive citizen.

**I: Are these the priorities by your institution?**

**P:** Yes. According to our mission, the one that we do as a priority is the improvement and security of youth participation and beneficence. So, we work to economically empower women.

**I: What do you do to empower women economically?**

**P:** There are packages for the rural and urban areas of the youth beneficence. As a rural woreda, we organize the youth groups to sustainably be involved in the activities based on the resources we have through capacity building activities, support them in preparing a business plan and organize them to have market center for preparation/production and selling and at end we link them with credit services . For example, to economically empower the women, we work particularly on livestock which may be cattle fattening, goat fattening, chicken production, honey production, and others. The second thing is we organize them to work on irrigation based activities. We have also seed money in which the groups transport stone products to Mekelle as construction materials such as stone, sand, and other products. The groups to be organized should have at least 50% females’ participation; otherwise it will not be accepted. The seed money facilitates a group of youth that can be involved in sand, stone, gold, and community based wells. The others that female youth mainly involved are in the five sectors: small and medium enterprise sectors, manufacturing services, urban agriculture, construction, and trade. They are highly involved in services like cafeteria, hair dresser, and others.

**I: Is it only in the woredas or kebeles as well?**

**P:** In the five sectors, the small and medium enterprise is for urban areas. The urban areas in the woredas are Samre and Gijet, and now Finarwa is also included as urban, and Adikaelay. There are also some others where there is electricity that are working on agriculture based activities.

**I: What your institution is doing currently related to the priorities you have mentioned?**

**P:** I told you the strategy and the participation of youth packages that are implemented in the ground. All are what we have been using and currently working.

**I: If that is the case, what nutrition interventions have the most resources allocated to them? Could you tell me in detail with examples?**

**P:** In 2009, the largest budget allocated from the federal revolving fund and also supported by the region as compared to others is the credit service provided to youth. Though there is credit service from Dedebit microfinance, the youth are provided only 15%. As the youth have financial problems, we give priority to provide youth a credit service because they cannot get enough money from parents and they need to have money for the economic transition from childhood to youth. So, we give much emphasis to this issue. They have to be supported and linked with credit services. In 2009, about 24,000,000.00 birr credit was taken by the youth in this woreda. This is a great job. So, this year, the one job we did with great emphasis is providing credit service. In 2009, there were 559 female youth beneficiaries and 1013 male youth beneficiaries and in total they were 1572 beneficiaries. So, these numbers of youth have been involved in sustainable jobs of the different sectors mentioned earlier in the different activities as a result of the economic support and then this improves the nutritional as well as improved economic status.

**I: Do you think it is necessary for your institution to get involved in work aimed at improving nutrition among women and adolescents? Why? If possible in relation to your institution’s mission.**

**P:** Exactly. Now we are also going to associate it with our mission. Our mission is: to have a youth with overall personality, successful citizen, ethical and competitive in sport, outstanding in national and international competitions. This means, to have a successful sportsman or woman and also to have a productive citizen, the nutrition or feeding practice should be appropriate. So, this is part and parcel of our job.

**I: How do you evaluate the priority given for the interventions for the women?**

**P:** I accept the priorities that are under implementation. I do not have differences with what the institution has given a priority. I accept that the priorities set by the institution are reasonable.

**Section 3: Nutrition interventions that improve adolescent and maternal health**

**I: What kinds of nutrition interventions are in place to improve health of the pregnants to your level? Where do they get it? Who provide it?**

**P:** It is like what I said earlier. There are also NGOs working in capacity building activities in relation to women violence, disease transmission, women rights so as to make sure that women will benefit fair and equal economic resources. So, all the governmental, private, and NGOs are involved, for example the World vision is supporting the interventions.

**I: What do they do about this? You can also tell me any interventions that are carried out by others not necessary from your institution in relation to nutrition in this woreda.**

**P:** Ok. For example there is what we call Habeney; promoting about food and here there are agents, women development groups that distribute the food.

**I: What kind of food are they promoting?**

**P:** It is a kind of complementary food. It is prepared based on one kg and half kg packed in plastic bags. So, they do promotion activities and sell the products during Saturday where a lot of people are gathered. There were also interventions and occasionally it happens these days that are conducted on food preparations, for examples they work on the importance of sweet potato, how to prepare food from it, and on how to improve nutritional status. Previously, there were experts from agriculture sector but now the interventions are implemented through the health extension workers. So, education is provided, for example the world vision is giving trainings to improve the capacity based on these issues. Similarly, REST is also involved in similar interventions. They have a health sector which also works on the capacity building in relation to nutrition.

**I: You told me that there is a complementary food distributed here. Who is preparing it? Why are they promoting it?**

**P:** Habeney is prepared in Mekelle but here there are agents that distribute the food. There are also products promoted on yellow colored cars, I have seen it even yesterday.

**I: Could it be orange fleshed sweet potato?**

**P:** Yes. Orange fleshed sweet potato. They promote products from orange fleshed sweet potato. Whereas, for Habeney food products, there are women development agents that promotes the products around administration office.

**I: Are the beneficiaries pregnant women, lactating women, adolescents, any others who have the ability to purchase?**

**P:** These complementary food products are mainly distributed to pregnant women and children. It will be another question whether the food is consumed by pregnant women and children at home. But during distribution, it is sold for pregnant women and children. The other thing is that antenatal and post-natal cares are one of the priorities by the government. What is declared always is that a mother should not die and should not give birth at home and it is considered as everybody’s job.

**I: Who is promoting the messages?**

**P:** Now as an institution though mainly it is implemented by the health sector, it is the responsibility of all of us. There is program that we have to take our responsibility to achieve for the effective implementation of nutrition though it is mainly carried out by the health sector. The promotion and coordination activities are carried out primarily by the health extension packages but as to youth and sport affairs what we contribute towards the improved nutrition is that we have youth association structure at kebele level and there are also youth affairs; chairs, youth leagues, and youth unions. They facilitate an access to have a traditional ambulance in areas where the ambulance cannot reach to the community. The youth carry and bring women using the traditional ambulance until the place where the ambulance comes or until the health centers according to their networks. We are also encouraging these activities and we are supporting them. The second thing is that any administrator has the responsibility to take his wife for check-up and should not allow his wife to give birth at home. In case the wife of the administrator gave birth at home, it is a big mistake and there could be an action taken for his mistake to the extent that he may be dismissed from his position.

**I: Counseling extra meal during pregnancy and lactation? Do pregnant women get screened for their nutritional status?**

**P:** Yes. It is right. Especially in pregnant women that are under and considered as having some problems with their nutritional status are provided a support from the health. The support is FAFA and oils and I think it is the nutritious food. Similarly, children screened with this kind of problems are also supported. The screening of women and children carried out starting from the discussions within the networks of the development armies, whether there is inconsistency or not, in which it has an opportunity for the women in same Kushet or neighbors to freely discuss and share ideas and try to understand about who is having a problem in relation to nutrition and who is in a good nutritional status. According to this, the information of the problem of nutrition of the women or adolescent is reported to the health extension packages with in the kebele and then they facilitate to support them.

**I: What about the use iodized salt in the community?**

**P:** With regard to iodine, amazingly there is a great change. Previously, people were thinking that the iodized salt does not taste well and they preferred the salt that comes from Afar, ganfur. There are traders that bring ganfur salt from Afar but I can say there is a basic change in the community. We go to the kebeles and visit many farmers’ households for different activities and we are provided with food and I have seen farmers are using iodized salt. Now, you do not even get the ganfur salt. I can say the community is using iodized salt but to some extent I suspect that in big occasions the people may use ganfur salt because they think the food will be tasty. Otherwise at household level, it is difficult to find a household that do not use iodized salt.

**I: What about home gardening?**

**P:** As a program, we do two things: keeping in mind the activities that are implemented in crop production using improved agricultural inputs to improve productivity, side-by-side we promote farmers to use irrigation on some portion of their farm lands. At the same time, we promote farmers to use home gardening packages and also to plant trees to reduce deforestation. The second thing is that what I have mentioned earlier about the health problems of women are also related to the workload to collect wood and water from a long distance. So, there should not be a means to collect wood from a long distance and it should be protected and be free from any contact. While rehabilitating the natural environment, we can satisfy our energy consumption through planting trees in the homestead. As wood is basic necessity for the rural areas for cooking, we have to either provide electricity or promote them plant eucalyptus trees in the homestead to protect deforestation. Similarly, we have to also promote and encourage farmers to produce vegetables in the farms so that there will be a means to purchase vegetables such as tomato and onion from the market. The good opportunity in the home gardening is that the areas are fertile because the homestead areas are having manure. The other thing is access to irrigation in the home garden is better. The major problem here is lack of water supply. Anyways, there are many who have home gardening activities. There are households that transport water using donkeys to grow vegetables in the home garden.

**I: Are they producing for market or for consumption?**

**P:** It depends on the size of the land and amount of the product. Primarily, they use the product for the household food consumption rather than purchasing from the market. The next is market oriented activities in which the different farmers producing vegetables using irrigation in the irrigation cluster are organized and linked to the market to produce vegetables. So, we are primarily working to promote and organize them to produce good quality and high amount of market oriented vegetable products using irrigation.

**I: You have already told me about safety net but can I ask you to explain on how the people are using and the benefit of safety net program? Do you think people will be changed from the program?**

**P:** The people in this community have the experience of working outside of these areas. They go to Humera, Addis Ababa and others to work and get changed. So, there is work experience outside of these areas and then come back here. But what they are satisfied with the safety net is that without going far away from their family and also rehabilitating the areas, they consider it is beneficial for them. In addition to that those that are supported from the agriculture in the program are in transition while getting graduated and improved their economic status. So, this is evidence that they are happy with safety net program. The second thing is that it includes those that do not have anything and do not have access to foods, elders, orphans, those that are unable to work because of internal problems, and others are addressed by the program.

**I: Earlier you have mentioned one example of workload. Are there interventions in relation to workload reduction of women?**

**P:** One thing is the water supply. The government has defined the distance where a water supply has to be established. The coverage of water supply is actually known by the water resources of the woreda but I think they are working to supply water close to the community. The other thing is the changes that are coming, for example access to milling facilities everywhere close to the communities to reduce the workload of women because it is the women who are involved in the process. Another is about the health services facilities or infrastructures in the local areas so that the community will get access to health services without going too far from home. So, I can say there are a lot of activities that are implemented.

**I: What about water, sanitation and hygiene services?**

**P:** I cannot say everyone has got access to quality water because there are kebeles that are far from the water supplies as I said earlier and drink water from the water steams, for example Frewoyane kebele does not have water even the water drilling machines could not get water in this area. So, it is very difficult to take that all the community in the woreda is getting access to quality water but the government is working to improve the water supply. With regard to hygiene and sanitation, it is the health packages that are working on it. There are 16 components within the health package that should be fulfilled to be a model. Here there are different things, for example it refers on how goods or belongings are handled properly at home, how to wash and put the goods, availability of clean toilet, and others. Here we have many kebeles that are ODF (out of defecation free). World vision project is also supporting on this regard in their own site. Similarly, REST is working good and the woreda as well. So, as I said earlier, components in the health package includes the thing that after using the toilet, you need to wash hands before preparing food, before breastfeeding a child, other personal hygiene and also sanitation. Here the municipalities are supporting such activities and follow up and monitor sanitation activities based on the rule that every house should be involved in sanitation within 20 m radius otherwise they are punished by law. There is also land administration that works on the environmental protection having experts to regulate health aspects working in a coordinated manner though they may not implement the activities efficiently but there are cases where people get punished when they do not keep the environment clean. But mainly this is carried out by the health packages. There is also a field visit, what we call SMS, which the health office is organizing as supervision. In this woreda there are five health centers and 23 health posts so that all the experts in this health post and centers are involved in the supervision. At the same time, they give education during the day of supervision to those that comes to the health center or post for medication based on the hygiene and sanitation, diseases transmission, family planning, and other aspects.

**I: How do the communities utilize/accept the interventions? Do you think there is a change?**

**P:** The community is now accepting. Toilet in the rural areas was difficult to be implemented and it was shame for the men to urinate at home in the toilet but now due to the awareness they are getting about the advantage of toilet and the incidence of cholera which affected some individuals to the extent that there were some who died because of this disease. This revealed how much toilet is important for the household; actually the community was aware of due to the trainings delivered in the previous years but in addition to that the incidence of cholera has initiated many people to give much emphasis to the use of toilet. The community is therefore accepting the messages that are promoted by the health packages; simply, we can take the example of family planning in which this time there are many women using prevention mechanisms.

I: **Are pregnant women getting advice on the need to use Insecticide treated bed nets (ITN)? Why? Who advise them? For lactating women, For adolescent girls**

**P:** ITN is one of the acknowledged interventions in these areas. ITN is provided especially to those vulnerable areas to malaria where the environment is suitable for mosquitoes. Similarly, spray of DDT is also conducted after giving trainings to those who are involved in spraying the chemicals. The community is willing to get ITN and there is supply but as to my observation, there are cases where the farmers use the ITN to collect or cover straw and other materials before the ITN has finished its originality. Actually, this has been assessed and major measures have been taken to correct these kinds of mistakes.

**I: What is the reason that they use ITN for other purposes?**

**P:** I cannot say that the community did not understand the need of ITN but the thing is that there is supply and the community is provided with ITN. As it is seasonal for the mosquito to affect the people, they ignore the value of ITN during the time when mosquitoes are not available and use the ITN for other purposes and do not have the attitude of saving the material to use it when it becomes a season suitable for mosquito because they know they will get it any time for free and I think that is the problem.

**I: Who is providing the ITN?**

**P:** It is the health office that distributes the ITN.

**I: Are pregnant women getting deworming services? For lactating women, For adolescent girls**

**P:** What I know from the parasites is tapeworm which is prevalent in the areas not only in the farmers but also in civil servants. But the good thing is that when it happens, there is access to health services and it will be treated easily if you go to the health centers.

**I: So, are there deworming services?**

**P:** I do not know. Actually, there are vaccination given but I think that is for the other problems like for example eye problems.

**I: I was going to ask you about that (vitamin A). So, do you think there is vitamin A supplementation? School feeding? Out-school adolescents?**

**P:** Yes. There is vitamin A supplementation for all. They organize on a specific intervals or a time convenient for all.

**I: Is it targeted for students or there are out-school girls who get the benefit?**

**P:** Our institution gives much emphasis on non-school adolescent girls because we consider school as one type of job. When we say work opportunity, it is for those that are not students because school by itself is a job. Those girls that do not attend school are therefore the ones that are closer with us with our activities. Those that are in school are only included in the different clubs organized by the school itself.

**I: What about the school feeding programs?**

**P:** It was just a year before last year that there was drought and then there was school feeding program for the students. Now, if am not mistaken, there is a project “Mekane Eyesus” that supports students in school like providing an incentive in those areas that do not send their children to school because of lack of food and others but I am not sure whether this time it exists or not.

**I: Do you think that approach will bring children to school? Do you think it is important?**

**P:** I think it is important during the time of drought because the children face shortage of food and it is impossible to bring children if they could not get food. So, something that can solve this problem in school by preparing food is important for the children because it will be comfortable environment rather than staying at home without taking in food. Otherwise, without solving the problem of shortage of food, it is not fair to ask students to come to school and I think that will be the basic solution for the students.

**I: As you said earlier, you are mainly working with out-school students. So, do you think that they are getting youth friendly services at health facilities?**

**P:** I can say the health services delivered by the health packages are good in terms of access and its confidentiality. There are studies about abortion and it indicates that there are also students that are facing this problems. So, there needs to be improved education or awareness creation sessions for the students on the aspects of sexual and reproductive health, for example how to prevent unwanted pregnancy. This is actually evaluated within the woreda and it was agreed that these should be carried out in a better direction and our institution is also working on that through supply of condom, make it accessible to the users, for example here in the gate of this administration office, there is one box. The health office is providing us these facilities to make it accessible for the users. We also develop and distribute brochures to improve awareness of the youth.

**I: How is the sexual and reproductive health interventions carried out for adolescents?**

**P:** It should have also been available in our institution. There are experts in the larger urban areas that provide advice and counseling but we do not have this in our institution. There is however an expert in the schools that gives advice and counseling to the adolescents. So, there is an expert that advices and give counseling following the ethical conditions in the schools and health centers.

**I: Which of the interventions listed above do you think is most important for pregnant women? For lactating women? For adolescent girls?**

**P:** One thing that I can consider important intervention for women and adolescents is supply of infrastructure such as supply of quality water. The second thing is generation of electricity using alternative technologies such as solar energy for the cooking purposes and this should be implemented in a better way. The other one is with regard to the health services including counseling should also be improved. Supply of nutritious foods should also be improved at least for those who have the money to purchase the products. If there is no any access to nutritious foods, women will not consume though they have the money to purchase. The other thing is about the hygiene and sanitation practices should also be implemented properly. One thing to add is that the women civil servants are also affected like that of the women farmers and adolescents though there is an improvement. In some offices, there are activities that are difficult and cause a workload to the women workers. These activities may be carried out that may not be comfortable when the woman is pregnant. This has to be considered and needs some improvement. The second thing is during lactation, it says there should be exclusive breastfeeding for six months but it needs modification though there is an improvement recently which becomes four months as compared to the three month previously.

**I: In your opinion, which of the above interventions for the pregnant women are being implemented in a successful way?**

**P:** The one I can say successful is the health package that has been conducted through the advice and counseling activities. In has been possible that there is a great reduction of maternal mortality in the areas and this is a great success. Previously, there were many mothers dying because of inappropriate feeding practices and other reasons but now it has been made lower. This is successful activity. For this there are lots of interventions to mention for its effective implementation so as to make it successful like, for example, the supplies, traditional ambulance, introduction of ambulance, improved agricultural productivity, job opportunity for youth, and others that contributed to the reduction of maternal and child mortality are successful.

**I: In your opinion, which of the interventions targeted to the pregnant women was less successful? Why?**

**P:** I do not know. I do not have an idea.

**Section 4: Implementation challenges and Community factors affecting access to nutrition interventions**

**I: What are the challenges to implement delivering the nutrition interventions that we have been discussing for the pregnant women?**

**P:** There can be different challenges based on the professional aspects but for me the major challenge is the attitude that needs to be changed. What I mean is, there are drawbacks in terms of attitude associated with religious and cultural issues. For example, rather than going to the health centers for check-up, there is a tendency not to go for a check-up assuming that you will get relief though you are seriously ill and there is also a tendency to go to the traditional medicines. Another simple example is that as a habit there is lack of personal hygiene. There is a need to change the community towards the modern attitudinal behaviors. There was a great problem in relation to religion though it is highly improved, women used to believe that it is not because I go to the health center but the presence of Saint Marry that I will give birth safely. It is very challenging if she is wife of a priest that she has to be at home for child delivery. This could be one of the challenges that limit the health of women and adolescents. The other challenge is economic status of the community and through the process, it has to be solved. We have to work hard to reduce poverty through economic empowerment. The other one is expansion of modern education and its accessibility that the government is working hard for its effective implementation. So, I can say, we need to work on these issues.

**I: Is there a relationship b/n educational status of the women & access to the interventions?**

**P:** Yes, there is an association between educational status and access to intervention. For example, woman who is grade 10 and a woman with no education are different. At least the one at the level of grade 10 is having better thinking capacity than the illiterate woman. She will also have the ability to accept new ideas easily. But if she does not have education, she will only take backward ideas. So, educational status is important. Whenever there is increased educational status, there will be increased keeping health status proportionally. There is direct association between educational status and care for their health and vice versa.

**I: Are the interventions accessible to the women and adolescents?**

**P:** There could a difference from one location to another location but they are accessible. Let us say for example, health services for eye diseases, check-up of diseases that are not complex, are easily accessible. The job opportunities are also accessible for women even better than men. As I said earlier, most of the services provided are at the hands of women.

**I: How convenient are interventions to the women and the adolescent girls? How do you explain the quality of the interventions?**

**P:** I do not have detailed information with regard to the quality of interventions.

**I: It is not necessarily about the health, but you can tell me the quality of interventions related to your institution.**

**P:** As much as possible we try to provide the services in a way that they are convenient for women. It does not have any problem for the women. They are implemented based on the agreements with them and in case there are problems, no one is allowed to do that activity. We are trying to provide the services that are convenient for the women and the trainings that we provide to support with the consent of the women.

**I: How do you evaluate the commitment of the intervention providers at your level?**

**P:** They are committed. You see at least 50% of the women are involved and this is how the gender issue is given much emphasis. Here there is no one leaving the women’s issues behind otherwise he will be punished by law. So, everybody is committed and there is also a gender mainstreaming that follows the activities related to women. There could be difference in the service provisions that can be of different levels, but there is no one who is going to be blamed by this time.

I: **What other factors are inhibiting implementation of the interventions? How?**

**P:** No, there is no any other challenge that I can say. If possible we need to work hard on those challenges.

**I: For these challenges that you mentioned, can you tell me of any solutions that your institution have applied to effectively implement the interventions for women and adolescent girls? Specify the each solution done for each challenges?**

**P:** I do not have much to say about this.

**I: While your institution tries to solve the challenges, what problems do it faced?**

**P:** We support the youth in two ways: we have private economic programs and cooperative economic programs. As direction, mainly we use cooperative economic programs. So, they form a cooperative and understand the importance of cooperatives to sustainably change and we are working for the better development of the individuals together. But as a challenge what we could not solve is that the cooperative are not sustainably developing together. They are separated when they get some money at some point. The other challenge is still on the formation of cooperatives; female and male are formed together but there is no willingness to work together because males have the attitude of showing superiority on females. They think that they are working extra activities and they wanted to leave women behind and become alone. So, this is a challenge that happens on the cooperatives that we establish. For example, there are challenges on the cooperatives working on stone products. The law is very strict that they have to be involved in the cooperatives by which 60% of it should be females. But the males tell them that you are benefiting from what we do and you are not doing anything. Similarly, with regard to sand cooperatives, males say that females cannot fill sand in to a car. As a solution what we do is that they have the right to use the resources of the country and they have already solved the attitude that says there is no anything that females can do but this has already been solved. They have worked a lot on the soil and water conservation equal to that of males. So, how can we say that you cannot do for it is now a business? They were doing a lot including the fight against Derg military power which is of course well known and they are equal. It is only because we do not accept it. So, what we should do is that we have to provide them appropriate work division. In case there are natural conditions such as pregnancy, she should have the opportunity to get rest. So, even if they do not carry and throw the sand to fill in to the car, they can collect the sand to facilitate the work. Similarly, in the case of stone lifting activities, it is not only the stone that is lifted but there are soils to be uplifted. Hence, they can dig and collect the soil in one place and also collect the stones. But still there are issues to be solved on the economic development activities of the cooperatives. And we have to also change the attitude of males who wanted to over dominate the females.

**Section 5: Multi-sectoral collaboration to improve maternal nutrition**

**I: Do you feel it is necessary at your level to work with other sectors/institutions to address maternal nutrition? What about for adolescent girls’ nutrition? Why?**

**P:** It is right. It is necessary to work with the stakeholders because there could not be an activity that can be finished in one office. Every activity is linked to each other. Because of this, we are also working in an interface with others and we assess our strengths and weaknesses to improve for the effective implementation of the activity. So, there should be a strong collaboration with other stakeholders. The reason is to implement the interventions successfully. There is interdisciplinary due to the reason that there is no intervention that can be implemented completely in one office. As a youth affairs, we have a mission but if we could not do to achieve the mission, there will be a lot of problems on the health of women. Similarly, if the women affairs could not do, it will be a problem, and all others. So, every institution should take the responsibility to work with the other stakeholders.

**I: Which other sectors do you feel are necessary to work with your institution?How do you see the other institutions’ roles complementing your role in improving maternal and adolescent nutrition?**

**P:** There are so many sectors and I do not think there is a sector that does not have a role to improve the adolescent and maternal nutrition. In different ways, whether directly or indirectly, the governmental and non-governmental organizations in the woreda are responsible. But mainly, the major stokeholds to work on this issue are the youth and sport affairs office, women affairs office, water resources, agriculture, water, mining and energy.

**I: Would you please explain their roles?**

**P:** Water, mining and energy – this is very important for the women to have alternative sources of energy such as biogas, solar, improved cooking materials, and others are important to improve the livelihood of women. The other one is water resources: how quality water could be supplied either through mobilizing the community from the government budget and also how to handle the quality of water after construction, for example in relation to the chlorine supply and others. Similarly, the basic thing is from agriculture office because of the reasons that we have been discussing earlier such as irrigation facilities and it is also necessary as it determines the livelihood of the household that are dependent on agriculture. But for all this, the administration office has to give an attention for the effective implantation of the interventions. Office of the political affairs (ውድብ ቤት ፅሕፈት) should also be involved to mobilize the interventions deep in to the community. The other higher administration offices should also be convinced so that it will be carried out properly.

**I: How do you evaluate the level of collaboration among sectors in nutritional interventions? Why do you think is so?**

**P:** Yes, of course. There could be a question whether there is expected change or not, otherwise there is collaboration among sectors. There is a change but it is not enough and we need to work a lot. As much as possible, we are working in collaboration with others.

**I: For multi-sectoral action that effectively works to improve maternal and adolescent nutrition at your level, what kind of change in terms of the way stakeholders work together is needed?**

**P:** There are beginnings that we are working on the activities which may need to be consolidated and may require the different sectors to prepare documents and platforms (mesrih). I am not sure whether there is a platform or not. If there is no, I suggest that we need to be coordination to work based on a platforms.

**I: What type of resistance to the needed change do you perceive, or have you experienced so far?**

**P:** The bad thing of the multi-sectoral collaboration is that the activity will not be easily implemented as compared to that of activities to be conducted at one sectors or institution. So, there will be a challenge especially if you are not the implementer. So, I can say activities that are to be implemented in collaboration with stakeholders are tame taking. They cannot be completed within a short period of time because there are regular or irregular occasions that delays the process in which one may go to another place or he may have another program and the other may have also another program which makes it challenging. So, it needs effort to solve the problem and it could be successful in doing so. If someone is committed, he will wake them up to work hard. So, it is also dependent on someone’s commitment that his strength could activate others to work hard.

**I: Is there coordinating platforms in enhancing multi-sectoral coordination in maternal and adolcent nutrition?**

**P:** I do not know about that.

**I: To what extent does your institution participate in the multi-sectoral nutrition coordinating body at this level?**

**P:** Yes, we are participants. As I said at the beginning, we have been working on the youth economic empowerment. While empowering economic status of women, we are also improving their social issues and increased participation. Let alone others, while we were working on their participation on political issues, it is to improve their thinking capacity and they will get access to this participation and we think they are benefited. So, all our activities are related to our mission and now with regard to sport, we had best athletes that are from this woreda such as Letesenbet Giedy who is internationally well recognized. This is because of the reason that we give much focus to sport and also proper feeding practices. There are also other athletes around here and Mekelle in different clubs that are initially from these areas most of which are women. So, what we are working is consolidating such activities.

**I: What needs to be done to improve the capacity of these bodies/platforms for effective coordination?**

**P:** With regard to capacity building, we have team on capacity building and quality assurance in our institution. The first thing that we need about capacity building is to fill the gap and bring a big change on the attitude of the youth. There are also others that can get support from technical and vocational education and agriculture extensions.

**I: What opportunities do exist to promote multi-sectoral coordination of nutrition in this woreda?**

**P:** In relation to nutrition, this woreda has good opportunities with the livestock potential, potential in crop varieties, there is sesame, and other fruits and vegetables using irrigation, livestock species. So, if we use the opportunity, we can produce enough and provide excess products to big markets such as Mekelle and surrounding areas. What is expected is to show the way, organize and promote on proper utilization of the resources.

**Section 6: Other interventions that influence adolescent and maternal nutrition and health outcomes**

**I: In your opinion, why would delayed marriage (after 18 years) improve maternal nutrition?**

**P:** Really early marriage is a problem in the areas. I know adolescent girls suffering by fistula because of early marriage and they are also affected because of psychological problems. There is a committee in the woreda that checks the school certificate and age of the girl with the existence of witnesses. So, it should only be above 18 years old because it has a direct association with health of the girl. I can say allowing or conducting a marriage of an adolescent girl below 18 years is just killing the health of the girl.

**I: In your opinion, why would increase the space between each birth improve maternal nutrition?**

**P:** It is well known that increasing the spacing between each birth is one thing acceptable and results in providing enough foods. In case the mother gives birth repeatedly with decreased birth spacing, she cannot provide additional foods to the children and additional foods for herself as well. She may also be suffered because of poor economic status. The other thing is giving birth with decreased spacing will hurt the mother and get tired. So, increasing birth spacing is important for the health of the mother.

**I: What programs or activities promote increasing birth intervals in this level? Can you tell me about any programs or policies in place in this woreda to prevent early marriage?**

**I:** There are meetings conducted at different times. The one I told you earlier is the network with in the development armies. The importance of increasing birth spacing and the risk of increasing birth spacing, early marriage, and others are discussed at the network level and then mobilized to the community. As there is no woman without membership of the network, they are all discussing about the issues. So, this has to be consolidated. Furthermore, there are meetings scheduled to discuss about these issues at woreda level supported with a budget. There is women’s union in the kebele: chair of women association, women’s league, and women affairs. Three of these discuses at wereda level four times a year about the issues of women.

**I: Can you think of any more programs or policies? Think about political, religious and other influences.**

**P:** There are no problems associated with policies and politics. Religions are now becoming supportive otherwise it was difficult for some to accept especially on the modern health services, for example there was resistance to allow women to delivery at the health centers. There was a belief that it is not a problem to give birth at home because there is Saint Merry.

**I: What about birth spacing and early marriage?**

**P:** There were also cases where early marriage was supported by the religions. They used to send her at early age as they are afraid that she will lose her virginity. So, these are not associated with policy, politics, and strategies but they are associated with backwardness, habits, and cultures in the areas. The religion also has its own influence. So, as I said earlier, we have to work on awareness to solve these problems.

**I: In your opinion, are these programs or policies effective? Why or why not?**

**P:** For me the process was successful. The good thing that should be consolidated is the activities implemented through the development armies. I am always excited to see the women development armies on their effective implementation of the programs. Previously, they had an incentive simply to support them with coffee and sugar to have tea and coffee ceremony during meetings because they discuss in detail and share idea freely. As a result of this, the outcomes were effective.

**I: What other community factors do you think that affect age at first marriage?**

**P:** Yes, there is factor in problem to the poverty. Poverty is becoming a factor to influence the households for the early marriage because as a habit that there are cases where there is big difference in the age of the husband and the wife; the husband is elder and the wife is under age. Whenever the girl’s families are asked to give their adolescent girl for marriage, the first criteria the girls’ families wanted to know is about the living standard of the husband. They wanted to solve the problem of the family by giving her to a rich family regardless of the age difference between the husband and the wife. The second habit is that there is a perception that a girl should have to be married otherwise she could not have life at all assuming that at later age she will not get a husband. So, the solution for this is to empower women and help them participate in the different activities in terms of economic benefits, job opportunities, and other. If the activities that are undertaken by the women participation are improved, we can change the attitude that women can also work equal to men and can generate income and then have better living by their own effort like that of me. We can also break the dependency on the economy of the husband. Otherwise, no one is happy to give his child and all the parents do understand what could be the problem of giving under age adolescent. But the other challenge is with regard to religion; the priests assume that this time there will not be virginity if the girl gets older, so they prefer to have an adolescent girl at early age to get her virginity. This time there is increased sexual behaviors and we need to work hard at school about the sexual and reproductive health to stop the unethical sex behaviors.

**I: Can you think of any other opportunities to prevent early marriage and increasing birth spacing?**

**P:** We need to improve job opportunities. If she is a housewife, she does not have another option so that the birth spacing will be short for her. But if she is busy outside, she will increase the birth spacing so as not to be interrupted with the job. So, we need to work on these issues

**Section 7: Additional Remarks**

**I: Would you please say something if you have anything to add from what we have already discussed or something that you think are important for future activities?**

**P:** The agenda that you are working now on adolescent and maternal nutrition is very important. This is basic for you are trying to assess the problems of half part of or 50% of the population. But I would be happy if you can also focus on those that are living under low economic status and low living standards, for example women living by selling local drink (sawa) because there are a lot of problems because of the lower economy and have many challenges to grow a child. So, it is good if you could also make it part of your study to improve the economic status of these neglected groups. The other groups are those that are living by prostitution. These women are also exposed to different problems and they can also influence with the dressing styles they wear and other conditions to other women that are not involved on this job and that do not have any other jobs as well. The other one is related to migration of women that are going out because of poor economic background and are highly exposed to different problems. There are also cases of psychological problems on those women who come from abroad back to home. Those who are working in prostitution could have unexpected pregnancy and also other problems that comes while working in this job even though they get money there are many challenges especially if they give birth to a child. Similarly, those who are involved in selling local drinks (Sawa), I suggest you to make it part of your study to understand their problems and come with solutions.

**I: Thank you very much for sharing me the information and time.**

**SUMMARY**

**Section 1: Common maternal (pregnant women, lactating women and adolescent girls) nutrition problems in the community.**

- There are many incidence of wasting in children because of lack of nutritious foods especially in the lowland areas.
- I have seen also a lot of children, adolescents, and women who made a long line to use the opportunity organized by volunteer doctors from abroad to treat people with eye diseases in the areas.
- Water is a big problem in these areas. So, if the people could not get adequate water, it will be difficult for them to keep person hygiene and sanitation.

**Section 2: Nutrition priorities in the woreda**

- The largest budget allocated from the federal revolving fund and also supported by the region as compared to other interventions is the credit service provided to youth groups.

**Section 3: Nutrition interventions that improve adolescent and maternal health**

- The youth carry and bring women using the traditional ambulance until the place where the ambulance comes or until the health centers according to their networks.
- The major problem here is lack of water supply. But there are many who have home gardening activities by transporting water using donkeys to grow vegetables in the home garden.

**Section 4: Implementation challenges and Community factors affecting access to nutrition interventions**

- Whenever there is increased educational status, there will be increased keeping health status proportionally.

**Section 5: Multi-sectoral collaboration to improve maternal nutrition**

- I can say activities that are to be implemented in collaboration with stakeholders are time taking.

**Section 6: Other interventions that influence adolescent and maternal nutrition and health outcomes**

- Early marriage is associated with backwardness, bad habits, cultures, poverty, and religion.

**Section 7: Additional remarks**

- It is also good to focus on those that are living under low economic status and low living standards as part of your study to improve the economic status of the neglected groups such as women living by selling local drink, those that are living by prostitution, migration of women.
