# Supplementary material for: Barriers to utilize nutrition interventions among lactating women in rural communities of Tigray, northern Ethiopia: An exploratory study
Source: PLoS One. 2021 Apr 30;16(4):e0250696. doi: 10.1371/journal.pone.0250696 (PMC8087028; doi:10.1371/journal.pone.0250696)
Supplement: S2 File — (ZIP) [file pone.0250696.s002.zip › S2_File.Doc/Woreda level and above key informants/078_IDI_ head for health office_Tanqua Abergele woreda.docx]

**Operational Research on Adolescent and Maternal Nutrition in Northern Ethiopia**

**Introduction**

Hello my name is Kiros, I am from Mekelle Universty; we are conducting a research on the factors that influences the nutrition of mothers and adolescent girls in collaboration with the regional health bureau and UNICEF. Year participation is very valuable; the information that you tell us will be used to improve nutrition programs and services for women and adolescents in the region and the country. We will not share your names when we report our results. The interview may take 1-2 hours and I would like to thank you for taking the time to speak with us today. You have the right to withdraw at any time and I will use tape recorder. Are you voluntary to participate for the interview?

**Yes** No

| **Section A: Interview details**   1. Zone: **South Eastewrn Zone of Tigray** 2. Woreda: **Thanqua Abergele** 3. Kebele: 4. Name of key informant: **Mr. Gebrehiwot Hadgu** 5. Institution of key informant: **Thanqua Abergele** **Woreda Health Office** 6. Interviewer name: **Kiros Tedla** 7. Date of interview: **14/11/2017** 8. Interview start time: **02:47AM** 9. Interview end time: **10:37AM** |
| --- |

| **Section B: Interviewee professional information**   1. Sex    1. Female    2. **Male** 2. Highest level of completed education.    1. No formal education    2. Primary education    3. High school    4. College education    5. **Bachelor degree**    6. Master’s degree    7. PhD 3. Discipline or field of educational training    1. Agriculture    2. Health (MD, nurse, **health officer**, midwife, pharmacy, etc)    3. Nutrition    4. Public health    5. Food science    6. Other (specify): 4. Current position: **Woreda Health Office Head** 5. How long have you been in the current job/position:    1. ______ Months    2. **2** Years |
| --- |

**I:** Interviewer **P:** Participant

**Section I**

**I, what are the common nutrition problems in the community for pregnant, lactating and adolescent girls?**

**P.** the main nutritional problems in our Woreda are mainly related to awareness or perception related to maternal nutrition. Hence, the role of religion and husband had a big impact as the husband should involve himself in giving care to his wife. The mother low level of awareness before pregnancy that she should eat healthy and become nutritionally fit and readiness for pregnancy in order to give healthy child. These problems and others like the old thinking such us providing the nutritionally good foods to their family and husband; getting no rest by working out of their capacity as a result their nutritional status is becoming poor. Involvement of the husband in giving care to his wife to get extra-rest, to feed extra-meal and giving care to the child are very limited or low. The role of the religion is also very huge as the mothers like pregnant mothers are fasting at least for six hours and the religion has several fasting days; so this has their own impact on maternal and child nutrition. Generally the awareness of the people in eating balanced diet using the available foods is very low as they only produce the materials like egg or other animals for marketing purposes. The people choose to sell the materials than eating these in order to get more money to pay for other purposes like dept and such thinking are might be present among our people. Anyway, what should be prioritized is the health of the child and the mother; so we have limitation in working with community in eating foods containing carbohydrate, protein, and vitamins or balanced diet.

**I. What do you mean with old thinking to maternal nutrition?**

**P.** the old thinking are like in the previous governments females were deprived of their rights similar to other population and there were also specific problems to the female as being a female. Even though; there are changes regarding problems manifesting in female as being a female but still there are specific problems mainly at house hold level.

**I. What are these problems?**

**P.** the owner ship of all the materials including money are by the male meaning the female had no chance to own the money by herself. Therefore; she could not bay what she would love to eat; the only chance they have is if the male would buy by himself. Economical owner ship at house hold level is still very poor as all of the decisions are from male. The religion had also high impact more than we expected for a mother who carries baby; as we can imagine how difficult is if we cannot eat breakfast, lunch, and diner. We have seen the impact of fasting related with the religion in Addis Ababa during the Sekota Declaration where there are 206 fasting days among the 365 days; this are practically applied among mothers mainly among women living in rural areas as it is hard to stop a mother living in the village from fasting.

**I. you told me that lack of awareness is related with malnutrition; how? Is it from the delivery or from the mothers?**

**P.** we are doing a lot in the community based nutrition with Guile limat and it is very promising mainly on ANC as we give ANC1; ANC2; ANC3; ANC4, Iron supplementation and other services. But the problem is on making permanent behavioral change in the house old as giving IRON to mother is a short term plane meaning there are limitations in the community in making the theory in to practical like we teach about food diversification by demonstrating but it is not practiced by the households related with low level of awareness and lack of resource or being poor. Even those we believe that they have the awareness they do not do it practically. There are also people who have the resources like egg or honey but they prefer to put to the market rather than eating as they do have other priorities like the as I have told you earlier they may need the money to pay for dept or other activities. So the people will prefer to sell the product to get the money rather than eating it. There is also food insecurity in the community because of the continous drought which affects our education not to be practical. Generally there are both poor and rich people not practicing their feeding habit because of lack of awareness in our community. So we need to work on this.

**I. What nutritional problems are common among mothers and adolescents?**

**P.** there is malnutrition among mothers like stunting, wasting, underweight and sever acute malnutrition as it is one among the known woredas affected by malnutrition. This is high among mothers meaning pregnant and lactating and it is studied.

**I. What about micronutrient deficiency? Like anemia?**

**P.** Yes, there is micronutrient deficiency like iron among mothers as a result there is high prevalence of anemia in our woreda. We are providing ninety plus with folic acid but still it is not enough as we have to enable the community to get iron sustainably bringing a change on feeding habit.

**I. What about the nutritional problems among adolescent girls?**

**P.** It needs to be researched as I do have very limited information in this group. We are not giving the intervention for this group so I cannot have full information but I expect to have similar problem as the mothers. This is because as we are not working on such group which is expected to be a mother in the future.

**I. What about night blindness and goiter?**

**P.** There is no such problem related night blindness as we are providing vitamin A supplementation. Goiter is also not a public health problem in this community even though there are some people with diseases as the diseases is highly prevalent in the high land areas.

**I. What about diet related non-communicable diseases?**

**P.** Yes, there is high prevalence of diabetes mellitus as it is increasing in this woreda. Eventhough the treatment is not given here. We have done a pilot study in the areas with high food insecurity or high nutritional problems like Tanqua abergele; Seharti Samre in collaboration with Mekelle University (Dr. Merhawit), diabetes association and we have got above 200 DM patients with in a single day screening. We have also tried to see the treatment demand at country level among the regions and one third of insulin is consumed in Tigray which indicates high prevalence of the disease. This is a public health problem and we have discussed with some individuals and tried to develop a proposal. We have also randomly screened pregnant mothers DM and 50% of the screened mothers had gestational DM. We have reported this to the regional health bureau and they are terrified by the finding. As a result there are trainings given to professionals by Dr. Merhawit and this will continue.

**Suggestion from the expert!!!!!**

There is high prevalence of epilepsy in this area particularly in some kebelle and we are hypothesis that it might be related to the nutrition or environment. So we need to work on this to prove that it is related with nutrition or environment.

**I. Are there areas which are affected by both malnutrition and epilepsy?**

**P.** We have identified areas with high epilepsy but we did not correlate with nutrition. What we think is like we said to prove malnutrition induced DM; we need to check if it is nutrition induced epilepsy.

**I. What about overweight?**

**P.** Yes, there are children born with overweight as they have more than 4 kg at birth. This is because of gestational DM which is very common in our area as I have indicated earlier and we have also seen such in the zonal hospitals.

**I. How prevalent is the non-communicable diseases among adolescents?**

**P.** it is prevalent as we have got 23 individuals with DM among the screened with age of less than 23 years old. Which indicates it is highly prevalent even among these groups.

**I. About food insecurity?**

**P.** even though the plane to eradicate extreme hunger and poverty is becoming diminished as country and even at woreda level; we cannot say every house hold had secured food or had food security. Our people are still suffering from shortage food resulted from internal and external factors like drought. For example; there were consecutive droughts in our area but information could be obtained from the agricultural sector as they are working on nutritional sensitive agriculture. We do have people living with safety net program and there are also other problems so we do have a lot of works to be done.

**I. Relationship of food insecurity and safety net program?**

**P.** Safety net program is a process of creating model farmers by providing aids within a five year period. These groups are food insecure groups at base line but making to become food secured. The program works on health, water and transportation. Both pregnant and lactating are also benefitted from the program; they went to the program but they did not work but get education.

**I. How do you think mothers are especially at risk for malnutrition you have mentioned above?**

**P.** For why mothers are at high risk; it is known that malnutrition has its own cause as main and underline cause. When we come to mothers they may eat foods but still they are lucking in getting balanced diet which is related with poor awareness. There is also poor husband involvement in helping to get the mother balanced diet. Generally there is poor awareness on both the male and female or husband and wife but it should be targeted to husband as he is the dominant in our setting. This was also one of the issues raised during the Sekota declaration evaluation. There is also a problem related with poverty as they cannot afford to eat their product like if they have honey or egg they would not prefer to eat but to sell and get the many which could be used to pay a dept or for other activities.

**I. Do women in this community suffer from stunting?**

**P.** It is very difficult to say they are affected by stunting as we do not have an accurate parameter; hence I am afraid to conclude that mothers are affected by stunting.

**Section two**

**I. What priorities do your institution has in relation to maternal and adolescent health? Why?**

**P.** as a sector we are working on nutrition specific but nutrition sensitive is dealt with agricultural sector as nutrition is multispectral. For example, 80% of nutrition related activities are performed by agriculture, water, education and others; only 20% is related to the health sector as explained by different researches. As nutrition specific we are working on CBNC or community based nutrition counseling which includes starting from pregnancy which includes providing ANC service follow up, iron supplementation, food demonstration, nutritional screening for both mothers and their children. And after birth we intervene like on type of food she should eat for herself and her child, on taking extra rest, and others targeting the first 1000 days. We have classified the activities in to health post, at community based activities, health center. We also give or link mothers or their children to plumplet.

**I. What are the activities done at community, health post and health center?**

**P.** At community we work the CBNC with Gujile Lemat mainly on awareness creation. At the health post activities like nutritional screening using MUAC, Iron supplementation, deworming for mothers and child, and other counseling services like SPCC are given. At the health center treatment for acute severe malnutrition where the patient is admitted to the health center. Most of our interventions are at the community or kebele level.

**I. On adolescents?**

**P.** We do not have any intervention but before 2 years we have given iron supplementation to adolescents with age of 10-19 years old but stopped. But in children mothers are advised to give complementary feeding, exclusive breast feeding starting by lactating the baby with in 1 hour of delivery. There are also other mandatory services which are done at the health center for the child and mother.

**I. What nutritional interventions are allocated the most resource?**

**P.** the interventions are not resource consuming as most of the activities are done without payment or voluntarily. We only need some money for training and we have integrated them with our routine activities. The only thing we need is to tell to the WDA to mobilize the community.

**I. What about the resources related with treating severe malnutrition or moderate as they are given Plumplet?**

**P.** we only check the availability of the medication and distribute to the lower health facilities. For example severe malnutrition is treated at health post then we send the treatment to the health post based on their request. Hence, there is no shortage of resource as it depends on the number of demand.

**I. Any shortage of the resources like plumplet or Fafa?**

**P.** there is no any shortage related with the resources except if we did not report the correct number of individuals, transportation problem meaning transporting the materials to the health facility.

**I. Do you think is necessary for your institution to involve in work aiming to improve maternal and adolescent nutrition? Why?**

**P.** regarding our role that whether we are important or not, and regardless of the 20% work distribution; health is pillar for nutrition as a science. so there is no other sector important than health sector as we need to deal with the community awareness creation and giving education and demonstration by house to house visiting. But we or the health sector could not be capable of withstanding this so the involvement of other sectors is important.

**I. How do you evaluate the priority interventions given to women?**

**P.** we do have monthly reporting and meeting to evaluate our performance and nutrition is our main agenda. But still there are limitations mainly related with quality of service such as nutritional screening for children, vitamin A supplementation, deworming for greater than 2 years old and others like vaccinations services are given but needs improvement. We assess these as they have their own focal person working on these interventions.

**I. How is the performance of these interventions?**

**P.** we cannot say they are performing very bad; as they look good like supplementation, nutritional screening and GMP activities are performing very well but we do have limitation on the quality.

**I. What are the limitations?**

**P.** Mothers related awareness problems as all mothers with normal nutritional status were expecting to get the aid and annoying when told they will not get as their child is normal and stop even coming to the health facility to use the other services. Hence; we start to give a name like “Wehale” or excellent for those who had feed their children appropriately or child with normal nutritional status and “Besero” or Lezy for mothers having a child with malnutrition. The quality of service is also affected as all mothers or children are coming at the same time which could not be addressed at the same time as the number of HEW is very limited per health post. Therefore; the HEWs will pass their time in registering of the individuals but not in educating as they have not time.

**Section three**

**I. What kind of interventions are in place to improve health of the PW to your level?**

**P.** for pregnant women advice to strictly follow its pregnancy and use the focused antenatal care at least four times starting from the health post up to the health center. At this time the nutritional screening will be taken like the weight of the mother will be measured; the status of the child or other gestational activities will be taken. And after the 3^rd^ tri-master the mother will be given deworming for intestinal parasites and iron supplementation to prevent anemia.

**I. What is the value of nutritional screening?**

**P.** Naturational screening using MUAC is important to classifay the mothers according to their nutritional status and link the pregnant mothers with low nutritional status to the nutritional intervention like to children.

**I. What is given to the pregnant mother?**

**P.** Plumpysub is given to the pregnant mothers but fafa is stopped now.

**I. What about interventions targeting lactating mothers?**

**P.** We give post natal care services; like early post natal care DA with in the first 48 hours and nutritional screening similar to the during pregnancy; and after 45 days family planning services and vaccination. There are also other services like counseling on feeding of herself and her child.

**I. What about extra-meal during pregnancy?**

**P.** This is related with SPCC interventions our main focus is creating awareness to mothers and during soil conservation activities we stop them from participation after discussing with the agriculture sector.

**I. At what time and for how long are pregnant mother exempted from the activities?**

**P.** We don’t have specific time at what time the pregnant women should stop it only depends on the mothers coming to the health service and known she has pregnancy.

**I. Is it also for lactating mothers?**

**P.** Yes, lactating mothers are also exempted from the activities for six months previously but now it is extended to be 10 months.

**I. What about advice on iodine salt?**

**P.** Yes, there is advice or we are working and most of the people are using iodine salt as indicated from the market perspective according to our random assessment. But still we cannot rule out the utilization of the old salt not alone in the village but also in the town as the salt is still in the market.

**I. What is the problem?**

**P.** The problem is related with awareness as the some of the mothers used the old salt to prepare pauper and Shiro which are used to prepare cooking’s which means the individuals are not using the iodine salt rather the old salt. Even though the salt is used appropriately in many of our cities or villages the cooking is not finished immediately so there continuous heating to make the cooking hot; then the salt will melt down because of the continuous heating.

**I. How are you participating on nutritional sensitive agriculture such as home gardening?**

**P.** Our main activity is on creating awareness and mobilization of the society to educate on all agricultural activities including home gardening, animal product utilization like meat and others.

**I. Are there advices on water, sanitation and hygiene services?**

**P.** Yes, we work though the HEWs as it is their main activity. But still there are limitations like we could not able to contract a permanent or sustainable toilet which is equipped with all the necessary materials like permanent washing service and soup.

**I. From where is the problem? Is it from provider or recipient side?**

**P.** We cannot say it is from our side as it needs a coordinated work like it needs political commitment starting from the kebele leader as it needs providing land to the toilet.

**I. Are pregnant mothers getting advice on the need to use ITN?**

**P.** Yes, they are the priorities to get the service including the ITN and free treatment if they are cases. Pregnant mothers and lactating mothers having less than five years old are given first.

**I. Why they are given first? Who provide them?**

**P.** Because the resistance capability is low among children and pregnant mothers. The other is if mother is diseased all the family member is also considered as diseases as the mother is pillar to the family. This is provided by HEWs.

**I. What about targeted supplementary feeding?**

**P.** We are giving for pregnant and lactating mothers and their children who affected by malnutrition. This has its own plane or indicator as how many of pregnant, lactating and children need the aid with its specific intervention should be known.

**I. How are the severely affected with malnutrition treated?**

**P.** They are given F100 and F75 milk during admission. They are also given folic acid and Amocsaciline.

**Vitamin A supplementation is given as you told me earlier for whom it is given?** **How?**

It is given for children with age of greater than six months. Previously it was also given to mothers immediately after delivery but now it is stopped as it is proofed that it has an effect on the mother.

**I. Is there any School feeding program in this woreda?**

**P.** As a region we can say that it is not started but in our woreda it is given in 23 schools. The students are given fafa in the form porridge with milk to eat in the school.

**I. What about on out-school adolescents about the feeding program?**

**P.** We only address these using youth friendly services as they are members of the service and we were teaching them on nutrition and perform screening services and include them in the plumplet program but now it is stopped as the partner organization or donor has stopped.

**What is youth friendly service?**

Youth friendly service or “Maere Edme” is a service provided by some age groups which have their own OPD and educate to each other.

**I. What activities are carried out by the friendly service?**

**P.** they work on different issues like about gender, delivery, nutrition and others. They present success issues in the form of drama to the community and students at school.

**I. Which of the interventions listed above do you think is the most important for pregnant and lactating mothers?**

**P.** All are very important mainly the CBNC services as they have their own effect on the health of both pregnant and lactating mothers. So there is no single intervention which is very important as all the interventions have their own independent effect or value on the mother.

**I. On adolescents related intervention?**

**P.** As I have told you earlier we are addressing the adolescents through youth service as we give education and we have also tried to give iron supplementation for six months but we did not give it again.

**I. Which of the above intervention for pregnant, lactating mothers are being implemented successfully? Why?**

**P.** nutritional screening for mother and child, iron and vitamin A supplementation and vaccination are implemented successfully. But there are limitations as well. This is because we evaluate continuously during our meetings and give credit. Hence; the credit will be used as performance indicator like BSC which will be used for staff development.

**I. What are your indicators to give the credit?**

**P.** GMP (Growth and monitoring program) and nutritional screening are the main indicators. If we call GMP we can meet all mothers and we can perform all the activities or interventions including screening for mother and child.

**I. Which of the above intervention for adolescent girls are being implemented successfully? Why?**

**P.** The school feeding program is running very well and successful as the HEWs are closely monitoring it. They check whether the material is expired or not; they will also look at hygiene and how clean is the preparation procedure.

**I. Which of the above intervention for pregnant, lactating mothers was less successfully? Why?**

**P.** as we are not working at school; we will target school to teach on how to prepare food through demonstration in order to do at his/her home practically. So need to give education to students and improve their awareness as they can make it practical in their home. If we able to change the students this are representing each house hold and the adolescents are the mothers of tomorrow and as we can see in our area all adolescent girls are the one who performs every activities in the household. So if we can change the students we can change the community easily and make the teacher to monitor for every student about their practice on nutrition.

**Section 4**

**I. What are the challenges to implement delivering the nutrition interventions that we have been discussing for the pregnant women, lactating women and adolescent girls?**

**P. individual level factors:** this is mainly related to both economical or being poor and low of awareness. Economical it is related with luck of resource as the individuals or mothers are poor they cannot get the resources even though they do the awareness. The other section is they do have the resource but they are not using it for themselves due to poor awareness on food fortification or diversification; hence, to bring change we need an integrated approach to bring the agricultural products in to his/her life that how important are the products to son or daughter and to my wife as this way of thinking is not practiced now.

**I. What about education status of the mothers?**

**P.** It is real that educated and non-educated cannot practice similarly. As an example; let’s take our self the one who writes and the other who did not write cannot score or remember similarly; hence the same is true to the mothers; as these mothers having education are practicing well compared to non-educated. So this is our main problem as most of our mothers do not read and Wright.

**What community related beliefs and norms are preventing access to the intervention? How?**

The main problem is related to religion as I have told you earlier which is related to fasting. The other is related to the husband involvement that he should help in getting the resources which are required during pregnancy and lactating.

**I. Are the interventions culturally acceptable?**

**P.** We are trying to discuss with religious leaders about fasting but still we did not go strongly as the higher Bishops are not approved it still. But we understand there could be challenges in accepting the interventions as it is related with religion.

**I. Are the interventions accessible to the women and adolescents?**

**P.** Yes most of the interventions are accessible. We have tried to assess the agricultural products of our woreda as we believe that there is no product cultivated which is not nutritionally valuable and there is maize, sorghum, teff and fish even though it is used only in same villages only.

**I. What about challenges from the interventions itself?**

**P.** there is no any problem related to the interventions.

**I. What solutions have your institution applied to effectively implement the interventions for women and adolescents?**

**P.** we have done discussions concerning the interventions with religious leaders and community leaders several times. We are also discussed with both Gujile lemat meaning male and female, kebele leaders and the total community but regardless of the changes there are also problems in which we need to strongly as I have already told you earlier.

**I. Have you got any challenge when you tried to solve the problems?**

**P.** There is no problem but I expect challenge when we enter deeply regarding the fasting days.

**Section five**

**I. Do you feel it is necessary to work with other sectors to address maternal and adolescent nutrition? Why?**

**P.** Yes, this is because nutrition is not specific to one sector and we cannot rule out other sectors as every sector is very important. As underlined in the declaration there are above nine sectors identified like agriculture, health, water, gender affairs, young and sport, finance and others.

**I. Why you need to work with these sectors?**

**P.** This is because we are working for the same purpose to improve maternal nutrition; hence to be effective we need to work closely. As for example; if we take the food processing industry they will produce foods so they need transduction with crop producers or farmers in order to produce valuable food components. The agriculture for agricultural products, water in expanding water reservoirs, small dams and providing quality water; association affaires deals with safety net program.

**I. Are you working with these sectors collaboratively?**

**P.** Yes, but it is not consistent and strong; example health and agricultural sector.

**I. What is the reason for not working consistently or strongly with the other sectors?**

**P.** The problem is related with leaders perception or way of thinking. As everything starts from the leaders meaning the leaders should establish accountability to each and every plan or report and should evaluate the performance of every sector. For example as health sector we have our own reporting and accountability process for nutritional interventions but the other sectors like agriculture should also establish accountability for the intervention or should establish reporting process or evaluation starting the lower up to the higher body. Therefore; there should be a nutritional body who had the power to lead and ask about nutrition to all sectors like water sector should be accountable what he/she had done on nutrition and the same to others as well. Hence; there should be evaluation processes to all sectors who work on nutrition.

**I. How do you think should be worked or what kind of change should be done the way the stakeholders work together?**

**P.** It needs political commitment like the Sekota declaration to make the stakeholders to work together and successful. When we say political commitment it should not only in paper it should be practical starting from the ministry up to the lower body there should accountability for the nutrition related un successful interventions for all the sectors. Example; if health has 20% part of all the activities related to nutrition and we will also work together with other sectors so we have to be accountable for not working or any failler of the intervention like if we have achieved 18% of the 20% we have to asked or accountable for not achieving our goal. Similarly the other sectors starting from the ministry level should also be accountable for their achievement.

**I. Do you mean we need to establish a system?**

**P.** No there is established system already for Sekota declaration and the leader is the dubiety prime Minister and the regional presidents. The problem is it on paper only not practical. So this should be functional and establish accountability like the president of the region should as nutrition related reports from the zonal administrator and the zone from woreda. And at woreda lever there are two committee coordinating committee and technical committee. The coordinating committee the leader is the woreda administrator and technical committee the administrator is health sector leader. So it should be run like this with established meeting and accountability to all sectors.

**I. What type of challenges or resistance to the needed change do you perceive or experience?**

**P.** There is nothing new it is already established framework but has no evaluation and monitoring system. The problem is there is specific problem in the sectors like one of the sectors are working very well and achieved the goal but the other sectors do not even give priority to the intervention. So what is needed is we should make the other sectors to prioritize and even accountable if do not achieve the goal. Therefore; there is luck of ownership as for example agriculture gives all the activities to the health sector as the sector considers nutrition is dealt with health even though we are only involved in 20% of nutrition related activities.

**I. To what extent does your institution participate in the multi-sectoral nutrition coordination body at this level?**

**P.** Yes, very few works were started with agriculture but this was by force as there were must do type of work. The work was done by force not smoothly and it was not even good or successful.

**I. What were the challenges?**

**P.** For example there is SURE program established by England which works on nutrition. Here both health and agriculture should work together and should visit the households together at the same time but if the one comes first but the other is not around the work will not be done. Hence; in our case even though the HEW is present the agricultural sector representative misses and we could not able to continue the work. This is because they do not give priority to the nutritional programs.

**I. What opportunities do exist to promote multi-sectoral coordination of nutrition in this woreda?**

**P.** We do have an established system and supporting rules and regulations. We do have also organizations or associations like Gujile lemat for both male and female; kebele leader. So we can use these associations as working platforms as they are working or involving in every sector. But the higher officials should give emphasis to the sectors if we need to bring the change.

**Section six**

**I. Why would delayed marriage (after 18 years) improve maternal nutrition?**

**P.** It is right that delayed marriage improves maternal nutrition and it is supported by science. this is because the mother with age of lower than 18 years is not well developed physically and mentally; hence may be exposed to excessive bleeding which may expose the mother to be anemic including the child or even preterm pregnancy may happen; the mother may also be exposed to other different psychological problems. So a child born from a mother who is matured in both physically and psychologically will have better health and nutrition.

**I. What activities or programs are there to prevent early marriage in the community?**

**P.** There are works done to prevent early marriage using religious leaders and WDA though education. There is also testing or screening of the adolescents to check whether they are above 18 years or not. Even though there are improvements but still we don’t prevent it completely; there are still community members doing early marriage.

**I. What are the challenges related to preventing early marriage?**

**P.** The first thing is related with resource as the male may have efficient resource so the family of the female may prefer to give him early as they do have fear that he may marry another girl.

**I. Why would increase the space between each birth improve maternal nutrition?**

We are doing on family planning very well as we are doing more than what the woreda or the region is expected to do. If family planning were not practiced we would have been populated and would have been faced with difficulties regarding population size.

**I. How it affects maternal and child nutrition?**

**P.** This if a mother do not use family planning after delivery she might get pregnancy even within the first month. Which means she may give birth within one year of the first birth; hence the first baby will not get milk from his mother in which he should have get for two years. And this will affect the nutritional status of the child and exposed to malnutrition. So it is known what will happen if she gives birth within one year spacing to the mother and child.

**I. Why your programs related to family planning are effective?**

**P.** The main thing is we have improved the awareness of the community on family planning. Even the family planning utilizes are changing from the short term like three months to the long term and becoming 50% short term and 50% long term utilizes of family planning services.

**I. What are the community factors that affect age at first marriage?**

**P.**  as I have told you earlier there are people still poor awareness and they pass their time in counting the resource like land ownership, number of cows and others for marriage. And if they have got female with such properties they will marry regardless of the age. But now the number had significantly decreased as WDA, the kebele leader and women affaires are working on investigating the age of the female.

**I. What are the policy factors that affect age at first marriage?**

**P.** Regarding to the policy it is good as it is already registered in the constitution by saying less than 18 years old girl is not matured to be married. It is also supported by the WHO definition. So it has no any problem with policy but implementation challenges like if she said I am 18 years old you do not have any scientific evidence to justify this. So we need to have scientific merit for justification like birth certificate where we are introducing now.

**I. Can you think of any other opportunities to prevent early marriage and birth spacing?**

**P.** the religious leaders, presence of different associations on both male and female; the constitution as its rules and legal agendas are supportive could be taken as an opportunities.

**I. Any additional comments or suggestions that we have discussed?**

I have no any comment but related to nutrition it should be strong starting from the higher body or at federal level focusing on the multi-sectoral collaboration with continuous meetings and monitoring and evaluations for all sectors. We at the lower level should also work together by considering as a big agenda as there is no agenda rather than this in benefiting the country.

Nutrition should start from the base; if we simply say 1000 days for mothers but the interventions before these days are very for example to create a healthy citizen you need to work on each steps of the cycle as she will give birth and become adolescent and become mother; so we need to work at each and every step of the growth cycle like starting from pregnancy and after birth; during adolescent then if she get pregnancy follow the same path; hence we can work on like this.

**Summery points**

**Section one:**

Severe malnutrition, Anemia, Stunting and underweight are very common in this area there is high prevalence of DM.

**Section two**

Main nutrition priorities in this woreda related to our sector is nutrition specific like CBNC starting from early pregnancy up to delivery and during the lactation period.

**Section three**

The interventions given include CBNC interventions such as ANC, iron and vitamin A supplementation, nutritional screening and others.

**Section four**

There are problems related with mothers like low level of awareness, economically poor, related with religion, and being illiterate.

**Section five**

Multi-sectoral collaboration is very poor even though there is established framework as this this not on practice and has no teeth.

**Section six**

Regarding to birth spacing we have achieved very well but early marriage is not stopped completely because of individual or community based challenges.

- **Finally I have finished my questions and I would like to thank for your time, patience and answering all the questions. Thank you very much!!! Thank you!!!**
