# Supplementary material for: Barriers to utilize nutrition interventions among lactating women in rural communities of Tigray, northern Ethiopia: An exploratory study
Source: PLoS One. 2021 Apr 30;16(4):e0250696. doi: 10.1371/journal.pone.0250696 (PMC8087028; doi:10.1371/journal.pone.0250696)
Supplement: S2 File — (ZIP) [file pone.0250696.s002.zip › S2_File.Doc/Woreda level and above key informants/079_IDI_Agriculture and Nutrition officer_Samre woreda.docx]

**In-depth Interview with Agriculture and Nutrition Officer in Sahartisamre** Woreda

Zone: Southeast

Woreda: Sahartisamre Kebelle: Samre

Name of Key Informant: Birhanu

Institution of key Informant: Agriculture and Rural Development

Name of interviewer: Measho G/slassie

Date of interview: 13.11.2017

Start time; 10:20 AM End time; 12:02 AM

**Interviewee Professional Information**

Age: 26

Gender: Male

Highest level of completed education: Bachelor degree

Current job/position: Agriculture and Nutrition Officer

Work experience in the current position: 3years

**I**: Interviewer **P**: Participant

**Section 1: Common maternal nutrition problems in this community**

I: Well, how do you describe the common nutrition related problems in this community? Mainly on women and adolescent girls

P: After providing trainings and other capacity building activities, and due to the encouraging collaboration with the health and other stakeholders, there are good changes on the nutrition of women and children, especially on children when we compare it with the past few years. There are big changes on the nutrition of under five children, especially on the towns and semi-town communities related with how to take balanced diet and for how long they should feed breast milk. There is also relatively good change on the maternal nutrition after I started working here when we compare it with the past. We had established an association called “HABENIO” to improve institutional delivery and maternal nutrition, and we have also established food diversification centers for women and children in all ten kebeles of this woreda. Despite there are good changes from the past, we don’t expect very high improvement because there are many problems that are not yet solved. Anyhow, when we evaluate it from what we have seen, there is good progress.

I: Good, as you have told me there are good changes and many activities have been implemented to improve the nutrition of women and children. But what are the common nutritional problems for women of this community?

P: The main problem in the women of this community is shortage of income. Even though the people have good awareness they have shortage of income to buy the required foods. For example, if they want fruits they have to buy it and in order to buy the fruits they must have the money. In fact there is still lack of awareness in some of the community, but the major problem is lack of capacity or shortage of money to buy their requirements.

I: As you know sever and moderate malnutrition are commonly observed in children and they have been treated and nutritionally supported to alleviate their problem. How do you see these problems in women especially in the pregnant and lactating women, and adolescent girls of this community?

P: Yes, there was nutritional support (e.g. provision of ‘fafa’) for women and children. But there was a problem in its utilization. Every household member including the adult men, non-lactating and non-pregnant women have been sharing the nutritional support given for the pregnant, lactating and children. However, to some extent there are changes from the past and they are now giving priority to the child. The “fafa” or other nutritional support given for the children cannot feed them for six months or one year, which means it will be consumed in a very short period of time than the expected time. In the past, almost everybody was utilizing it, but now almost only the child and the mother are using it, but the problem is not completely yet solved and there are many things that should be done.

I: Are there severely malnutrition women and adolescent girls who have been nutritionally supported in health facilities of this community?

P: By the way we have shortage of studies to speak about it, but I don’t think there is a problem to that extent.

I: Just from your own observation?

P: From my observation, there are underweight children who have been on nutritional support like Plumpy’Nut and other balanced diets and nutritional follow up in the hospital and health centers. Sometimes the problem is also observed in women, especially on those women who had bleeding after child delivery and food shortage. When we see the problem in our kebeles, it is more common in the children, but it also exists in the adult women. Therefore, there were women on nutritional follow up in the healthcare facilities.

I: What about in the adolescents?

P: I don’t have the information about them. I have not observed problems due to food shortage in the adolescent girls. It may be due to the fact that they are not coming to the health posts, health center or the hospital, but I don’t think they will be more affected by the problem because they have the awareness and they take care of themselves. The problem is not common in the adolescents; instead it is more common in the children.

I: How do you see the deficiency of micronutrients like anemia and night blindness in the pregnant and lactating women of this community?

P: Yes, the night blindness and other related problems are observed; especially it is more common in the lowland areas. So, it is sometimes observed in the pregnant and lactating women.

I: I hope you closely know the community. From your observation, in what type of women do you think the problems (night blindness and anemia) are common?

P: The problem is common in the communities which have low sanitation and hygiene. If the household and surrounding environment are not clean, they we be exposed to this kind of disease. When we assess the households in each Kebelle, the disease is associated with those who have cattle dung and other wastes around their home. So, most of the time, the disease is related with lack of sanitation. In addition, to some extent anemia is associated with feeding practice. For example, there is a situation where mothers only eat once (breakfast) per day, lack of self-care, lack of proper rest and work overload (especially the pregnant women) can lead to anemia.

I: From your observation, do you think the women are developing anemia due to food shortage or lack of attention for proper feeding?

P: We can see the cause of anemia in this community in two ways; first, there are women who are exposed anemia due to food shortage. They know that its treatment is food and want to eat balanced diet, but they don’t have the food they need. The food shortage is especially common in the young women. However, most of the people have lack of awareness on how to eat balanced diet from what they have. So, to some extent, the community has lack of access for food, but most of the time they have lack of awareness on how to use their available food and what types of food to eat to have healthy family. The common feeding habit in our community is feeding what is available at home. In addition to the lack of capacity, the main reason for the risk the problem is that they have lack of awareness on the demand of the mother for her health.

I: How do you describe the activities being undertaken to improve community awareness about maternal nutrition from the woreda to the lowest level?

P: It is relatively good when the awareness creation intervention comes from the region to the woreda and it is also good at woreda level. There are different projects at woreda level; for example the health office has its own projects and the agriculture office have recently (three years back) started nutrition interventions, and there are also activities done in collaboration with Tigray Relief Society. However, the implementations of the interventions are less effective when it comes down from the woredas. We have different activities from different sectors at Kebelle level; for example there is DA for agriculture, there is health package implemented by the health extension workers and there are also other bodies. So, the capacity building given at Kebelle level is much lower. The community may not attend the training arranged at Kebelle level, but the overall attention given for the lower cadre and the community is low. For example when you arrange training at woreda level, many people attend it because they related it with the training payments, but they don’t implement it and the awareness creation is not done as per the demand of the beneficiaries. So, the intervention implementation has been decreasing when it goes down from the top to the lowest administrative units. Overall, the awareness about nutrition is very low even at government level, for example the nutrition program was not available in GTP I rather it was started in GTPII. Since the start of the nutrition program, the understanding and emphasis given for nutrition security from the top down is limited unlike the emphasis given for food security. There is a big gap on the program because we don’t expect to bring change from the commitment of only few people or institutions, and only small numbers of people are working on it. The attention given by the governmental and nongovernmental organizations is very low. If the attention given by the region or woreda level to the program is very low, the attention given by the Kebelle will be similarly very low. As a result, the interventions will be lost before reaching to the community.

I: As you have told me, the implementation of the nutrition interventions are becoming less effective when we go down from top to down of the hierarchy. From your opinion, why do you think the interventions are not effective at the bottom line?

P: The main reason for the less effectiveness it that you are expected to implement what your leader told you to do. For example, if my leader didn’t distribute enough staff to the program, it will not be implemented to the expected level and it will be left only for me. For instance, there are more than 40 staffs in the agricultural office and 40 of them go to the fertilizer distribution program, but only I or sometimes two people are assigned to the nutrition program. So, I cannot support 23 kebeles in this situation. Even the different trainings that we have been giving for the woreda and other responsible bodies are not implemented in the community because there is no push from the top to implement it; instead their main inclination is towards the current issues. If there is not accountability for the program implementation, the attention given for it starting from the top will be very low. In addition, we don’t give high emphasis for it because the activity of one person cannot bring a change. There was a good start and linkage with the health and Tigray Relief and Rehabilitation Society and we were giving joint training for the workers from three of the sectors. There was also a good start in implementing the interventions in 10 kebeles; especially there was good start to improve the nutrition of mothers and their children by supporting them to cultivate home gardens and even to generate incomes if they have extra production of the home gardens in collaboration with the Ethiopian Orthodox Church Association. However, the program discontinued due to lack of strengthening the linkage. So, the main problem is with the overall program administration. If your leader doesn’t give emphasis for the program, it will not be implemented.

I: Good. Iodine is one of the important micronutrients. So, how do you see the magnitude of goiter in this community?

P: Goiter has decreased in this community; especially it has dramatically decreased in the past five years. It may be due to the good awareness of the community on iodized salt utilization. Almost 80/90% of the community is using iodized salt. More works have been conducted on this program.

I: So, do you think the iodized salt utilization is good in the community, especially in the women and adolescent girls?

P: Yes, it is good. Whenever we have training, we first ask them about when and how they are using iodized salt and almost all women have better understanding about the utilization of iodized salt (they can correctly tell you when and how it is used). A lot of works have been done on it. So, they have good understanding about iodized salt utilization both in mothers and in children.

I: Good. How do you see the diet related noncommunicable diseases like hypertension, diabetes mellitus and others in this community?

P: More or less our woreda is better in diet related noncommunicable diseases. Sometimes you may hear people affected by it, but generally it is not common in this community. The diabetes mellitus is more observed in the town community due to their feeding habits. You don’t expect in the rural people, but sometimes it is observed in the town people who have better income. These problems are not common in the children, but sometimes it is observed in the town residents. But, it can be sometimes observed in the rural residents, especially in the adolescents.

I: Do you think education is given for the community about the diet related noncommunicable diseases, especially how they can acquire the diseases?

P: Yes, like any other training, it is given together with health or agriculture related trainings. But, we don’t think the community has understood the problem even still the educated people have low understanding about it. Let alone the uneducated people, the educated people who counted alphabets have very low awareness about it. Sometimes training is given; we don’t think the training given is enough. There are many things left to be done.

I: As you know there are many malnutrition related problems. Stunting is among the malnutrition problems. So, how do you see the occurrence of stunting among adolescent girls (from 10-19 years of age) of this community?

P: Yes, there are stunted and underweight adolescents. Despite there is change from the past, it is observed to some extent. There are many stunted children and adolescents and also you can see similar number of underweight children and adolescents. As I have told you before, those children who have food shortage and who are on nutritional follow up are more stunted and underweight. The magnitude of underweight children is higher than the stunted ones. The stunting is not yet eliminated and it is commonly observed in the lowland areas. The problem could be related with feeding practice or it could be related with lack of awareness because some of the kebeles are difficult to provide support due to their difficult geographical setup. Thus, despite there is change, I believe there are many things that we have to do to solve the problem.

I: Good. You have well described that stunting and underweight are available in the adolescent girls of this community. Could you tell me the possible causes of the problems (is it related with food shortage or lack of awareness or others) and the measures undertaken to solve it?

P: As I have described before, the main cause of the problem is lack of awareness. But, as we have described before, shortage of food is also happening. However, the major problem is lack of awareness. To some extent, you can assure your nutrition with the limited resources you have. So, lack of awareness is the main cause of stunting and underweight. As to the intervention for these problems, apart from the capacity building and awareness creation activities, there are no another interventions as far as our office is concerned. When I was the coordinator of the nutrition program, we were distributing food support (Fafa, oil and other foods) after identifying the mostly affected kebeles by food shortage and we were also providing capacity building interventions. We have been providing the food support for the affected communities despite there are some drawbacks in its utilization. By the way there are also another interventions related with health in collaboration with FAO like provision of Plumpy’Nut and Fafa. It is also provided by food security. In addition, the Plumpy’Nut, Fafa and oil are provided for mothers and children in selected nutritionally affected communities. However, when we see the sufficiency of the food support for all affected community, sometimes there is a situation where the food support cannot be reached to all eligible beneficiaries. Even we have been using different formulas to estimate for how long to feed by the provided support. We are providing the food support only once for six months, which means the support is not enough. So, we have these kinds of interventions as starting point.

I: Good. Do you think overweight is a problem in your community or woreda?

P: More or less it may be found in the towns (laughing), but it is not a problem of the rural community. Since we have so many food insecure people, it is better to say that the problem is not common in this woreda.

I: How do you describe the food security (‘I sneezed’?)

I: Sorry

P: It’s Ok

I: How do you describe the food security of this woreda? You have of course tried to describe before.

P: In terms of food security of our community, there is a big change from the past, it could be associated with technology use or improved awareness. But, we can’t to say the community is completely food secured because we wait only one rain and if it is good they will not have the food shortage, however, if the next summer is not good, they will come back to the drought. We have not worked more on that. If the summer is good, we have farmers who are able to use improved technologies. However, despite all the efforts, we cannot conclude that the community has secured their food because if the last year’s productivity was good and this year is not good, the community will be affected by famine next year. Wastage is our main problem. But there are changes from the past, though almost half of the community is not changed especially in the lowland community. If they don’t produce more during summer due to lack of skill on the technology use and miss use of products, they may face food shortage. So, we can’t say we have secured food at woreda level due to lowland nature of the community and rain shortage.

I: Good. Are there another cultural related factors associated with food insecurity of the community in addition to shortage of rain?

P: Yes, there are wastages related with cultures. If their productivity is good this year, there will be many marriages and other religious festive meals, and they will end up with famine in the next year. There are some areas which required food support due to excessive wastage of foods in marriage and religious festive meals (“tezkar”) because the community awareness is low. There was a good start to make zero wastage of foods in this woreda, but we didn’t completely break the problem. For example, excluding the households which had marriages from the food support and limiting the number of people that should be invited and the required quantity of meal that should be prepared. So, there are these kinds of starts in the woreda, but the excessive wastages are not yet eliminated. We have unchanged communities which are at risk of food insecurity.

I: Good. Do you think the women are especially at risk of malnutrition?

P: Yes, because their feeding practice is far behind the required one. For example, while the other people eat timely, they don’t feed timey; a mother usually eats breakfast later after her children and husband ate their breakfast and after they are engaged in their respective works. The mothers eat after the breakfast time is over and become very hungry. There is also a situation where the mothers don’t eat their breakfast and lunch because they more focus on their work. If they have limitation of food at their home, they don’t eat their dinner instead they give the available food to their children and husband. The main reason why the women are affected by malnutrition is that because they give priority to their children and family members over themselves. Later they will be affected by different problems and diseases. So, the risk of malnutrition is much higher in the women than the males due to the reasons I have told you before like she don’t properly and timely eat food, she gives priority to her children and husband, and even she don’t eat tasty foods instead she gives it to her children and husband first. She may eat later if she gets a food left from her family’s meal. So, they are more affected by malnutrition.

I: As you know currently there are different structures and activities to transform the community through the health extension workers, women development army or others. How do you evaluate the efforts or activities undertaken to improve the nutrition of women and adolescents in this community?

P: The health extension workers are working more on the women’s health, even more than what we do. There are encouraging changes, as I have described before, there are limited numbers of women who equally feed themselves with their husband and there are also limited numbers of women who give more priority for the women and children in the community which have relatively good awareness. But, the problems associated with the community culture like eating in the absences or before husband are not completely solved. The health extension workers are working on delivery follow up, nutritional follow up, hygiene and sanitation follow up for improving women’s health. However, there are no big changes in the community due to high cultural influences. With all the cultural influences, trainings are being given for pregnant women to cultivate home gardening for preparing their own daily foods. For example, training is given for the pregnant women that if they have even two pieces of vegetables in their respective home, they can improve their nutrition. However, only few people implement the training.

**Section 2: Nutrition priorities in the woreda**

I: Good. Let us now come to your office, what was the full name of your office?

P: Agriculture and rural development

I: Ok, what priorities do the Agriculture and Rural Development Office has in relation to maternal and adolescent health?

P: As I have described before, the specific role of the Agriculture and Rural Development Office on women and children is provision of Fafa. Despite its coverage is low, we have been giving priorities to the women and children. We have been also providing trainings in collaboration with different stakeholders. So, to improve the nutrition of women and children, we have been providing the available foods every year by giving more priority to them.

I: Could you broadly describe the activities (with example) included in your office’s plan, especially these activities which have allocated budget or human resources for improving the nutrition of pregnant and lactating women and adolescent girls?

P: There is no any budget for the programs at woreda level. If there is NGO, we implement it, if not we stop it. There was good start with UNICEF. The UNICEF had stopped after one year support, because it was not working as NGO, rather it was as special support and we had not memorandum of understanding as well. So, there is not budgeted nutritional support for the women and adolescent girls.

**Section 3: Nutrition interventions that improve adolescent and maternal health**

I: Good. What kinds of nutrition interventions are in place to improve the health of pregnant and lactating women, and adolescent girls by your office or other sectors?

P: Yes, the services for the pregnant women are relatively good. The pregnant women come to the health center for delivery before a month and there is also free of charge ambulance service for them. In addition, the health extension workers follow the health status of the pregnant women and they also advise them on what to do, when to come to the health facility and also provide other services through going home to home. The pregnant women have also an appointment to come to the health center for delivery before a month and they also have services based on attendance or time table during their pregnancy period. These services are specifically delivered by the health office. Hence, the health and nutritional services for women and children, especially the counseling given for the pregnant women to deliver in the health center and their nutrition is relatively better. When we compare with the past, there is observable change and to some extent there is reduction of maternal death though not 100%, but it is around 99%. Though it is no marvelous change, there is improvement in the nutrition of children and mothers, and the pregnant women are delivering in the health centers due to the strong push from the community. However, there are some traditionalist people who don’t support the changes.

I: Good. As you know the pregnant and lactating women needs special nutritional support. So, what your office is currently doing for improving the nutrition of the pregnant and lactating women?

P: We are very weak it that aspect, because one or two persons cannot effectively do it. I can say almost we have done nothing with the exception of throwing Fafa for the pregnant women. Almost there was no follow up in the kebeles.

I: Good. You have told me before that nutritional support for women is not a main priority of the woreda, especially in the absence of NGO support, the woreda do not have any budget for the nutrition program. So, why do you think? Is it due to lack of budget or attention?

P: Yes, if there is budget, it is possible to do any activity. But, as I have told you in the beginning, the problem is not only the issue of budget but also the attention given for the nutrition program is very low. Nobody talks about it. For example, there is no budget for irrigation; it is only allocated for trainings. In fact there is limited budget for the summer productivity. So, there is lack of attention for the nutritional support program. When we see it at woreda level; if all offices are not responsible for it and if they don’t responsibly implemented it, there will not be any change. Even the attention given at sector level is very limited. So, the problem is not only budget, for example, the training is provided once and then you are supposed to support its implementation through the four or five experts found at Kebelle level. Had the experts providing training on nutrition as much as they give in other service, there would have been good changes in the community. So, the main problem is not lack of budget, rather it is lack of attention starting from the top. The program was dependent on one or two persons, even the program was not evaluated at region level. The top management should have known on what is going on, the strengths and gaps about the program in Sahartisamre, but they don’t include it in their annual report. So, there is lack of attention starting from the Region down to the woredas about the program.

I: Good. You have told me that the health extension workers and other providers have been following and supporting the pregnant women to deliver in health facilities, and providing other services. From your observation, how do you describe the interventions or services given for the adolescent girls and lactating women to improve their nutrition?

P: As per our extension system, the services given for pregnant women outweigh the services given for lactating women. We focus more on providing antenatal care follow up. We don’t provide equivalent follow up for the postnatal care and antenatal care periods. But, more or less though not sufficient, there is postnatal care, especially on counseling and providing child vaccination and follow up for the mother herself. Especially, there is follow up for the women who have blood shortage and who had bleeding. However, we have not worked on the adolescent girls. Our main focus is on the mothers and children. Almost, there is no any intervention on the nutrition of adolescents and there are no any other organizations working on the adolescent nutrition.

I: Ok, you have told me that attention is not given for the adolescent girls’ nutrition in this community. By the way, the attention given for adolescent girls’ nutrition is very low not only in this woreda but also in other many woredas. So, do you think it is important to give good attention for the nutrition of adolescent girls?

P: Yes, in my understanding, it is very important because they are the next mothers and they are also expected to educate their family. Since our main priority is on the pregnant women, there are drawbacks in the postnatal care follow up. The adolescents are the successor generations, they are the next mothers, and they are also the next educators of their family and community. So, if they have good awareness in their adolescence age, they can make a change.

I: Ok, how do you describe the counseling given for food diversification?

P: The health extension workers are giving the counseling. There is no anybody who gives the counseling beside the health extension workers. As it has been said, there are formal and informal trainings for the pregnant women, but the health extension workers are giving the nutrition counseling through going home to home. Telling the pregnant women what to do is counseling, telling when to go to the health facility is counseling and telling what types of foods to eat is counseling. But if you ask me whether these services are given for all, the answer is no. We are providing the service for the nearby residents, but we don’t provide the services for the residents of very far communities, instead we may send the services through other people.

I: Good. You have informed me that there are different interventions to improve the nutrition of pregnant and lactating women. How do you describe the efforts undertaken to improve the women nutrition through home gardening?

P: We have worked the home gardening in a very limited number of pregnant women. Although training is given for them, due to water shortage and other reasons, it is not well implemented. But we have started it in four or five kebeles; especially, we have worked in a place called “my tekli” (you might have seen it in your way to Mekelle) collaboration with World Vision with especial target for women. As I have told you before, the intention is first to support themselves and their children and then if there is extra production; they will supply it to the market. We have also started similar services in Samre and Gujit districts and other four or five kebeles. We have started it in some areas of the kebeles, but many more communities are not supported by the home gardening services.

I: Do you cultivate and give the gardens for the women or the women are cultivating by themselves?

P: We just provide technical support. The world vision also provides home gardening seed and technical support for around five or seven/eight kebeles and we have also provided training on where to cultivate the home gardening and when to use it. So, we support them the agricultural counseling and seed, but they are supposed to provide their own land and labor. Technically we have experts in the kebeles and they are expected to show them, and we ourselves also go there and advise them on how to prepare it in relation with the lands geographical setting.

I: So, the gardening services are not reached to all women due to the lack of seeds and technical persons?

P: Yes, the lack of home gardening coverage is due to the shortage of home gardening seeds supply and there is also shortage of water access for the garden in the communities. However, the main problem is related with their lack of awareness because very few cups of water could be enough.

I: Ok, how do you describe the implementation of productive safety net program in improving the nutrition of the pregnant, lactating and adolescent girls?

P: It is not expected to assure their nutrition through the safety net program because only wheat is given and even it is not sufficient for the large size households. Nowadays, the helpless and poor households are supported by the safety net program and even we don’t expect to improve their nutrition through the safety net program. It is from hand to mouth and it is insufficient for the households which have large number of children. So, we don’t expect to improve nutrition through safety net program because it is insufficient. Now, it is not allowed to enroll in the safety net program more than five.

I: More than five means?

P: It is not allowed to include more than five members from a single household, for example, if I have 10 household members, only five of them will be included in the safety net program support. So, if only five of the ten household members are included in the safety net program, they will not have enough food it eat and the food support given for one month will be consumed within a week or two weeks. This leads to famine and other problems.

I: Good, how do you describe the education given on water, sanitation and hygiene to the community and its implementation?

P: Much training has been given on the sanitation and hygiene, but the community is not changed yet. Capacity building interventions are being given and the community knows it, but there is a problem in its implementation. We have many gaps in implementing the sanitation and hygiene in the community.

I: From your observation, why do you think the community is not implementing it with all the trainings and awareness creation services?

P: The main cause is, there is a saying called many of many (“nay bizuh bizuh”). We couldn’t organize the community in to one. If four/five or more people are using one common water source, they don’t feel ownership about it and they become careless about the sanitation and hygiene of their environment. So, we have not changed the community in terms of sanitation and hygiene.

I: Ok, how do you describe the advice given for pregnant and lactating women to use insecticide treated bed nets in this community? How about their utilization?

P: In terms of coverage, the ITN is distributed for everyone. It is given based on the household numbers, for example, if we have three household members, we get two ITNs (the two will use one and the other one will use it alone). During its distribution, priority is given for the pregnant women and children. In terms of its utilization, despite there are changes from the past, there are still gaps. There are some people who don’t use it; instead they flex and put it in their homes and there are also some people who do not change or wash it once they starched it. So, there are good changes from the past, especially there is good utilization by the women and children.

I: Do you think the community has understood the importance of ITN utilization?

P: Interims of its importance, the community knows its relevance for the prevention of malaria and others and beyond that they are using it. But sometime, people use it for other purposes. Overall, there is better change and awareness in the utilization of ITN.

I: How do you describe the deworming services given for adolescents and pregnant women for the prevention of intestinal parasites like tapeworms and others which affects their nutritional status?

P: It is difficult to describe this question. It could be nice if a health professional describes this question. I may describe it from my observation.

I: Yes, just from your observation.

P: Yes, more or less there are parasitic diseases especially in the children and adolescents, because if the children are affected by the parasites, they will be exposed to malnutrition. But, the problem is relatively not common in the adults.

I: Do you think the deworming service is given for them? There is a tablet given in mass.

P: There is a situation where a treatment is given for all people if there is an epidemic or if there is suspicion of epidemic in the community. All the health workers in the three or four health centers and one hospital provide the treatment in campaign in the same day.

I: How do you describe the targeted supplementary feeding implementation, a special food support for the pregnant, lactating and children affected by food shortage?

P: There is no special support for those specific people who are suspected to have food shortage. However, as we have described before, there is Fafa provision for the nutritionally affected kebeles and within the kebeles priority is given for the most affected areas. So, we have general support for the community, but we don’t have a specifically studied support for the affected women alone. Before a year, during the drought time, there was such kind of support.

I: The targeted supplementary feeding is a specific provision of extra food like fafa, oil and others for the children, pregnant and lactating women identified as having nutrition problems within the identified kebeles which has food shortage, and it is given for specific period until they improve their nutritional status.

P: There is no special support for women or pregnant women in our woreda. But, in the last month there was food support, we have identified the pregnant, children and lactating women, and priority was given for them. However, it was not distributed for all communities; it was provided for only few communities.

I: Good, is there school feeding in this community?

P: There was school feeding (not specific for adolescent girls) in the form of porridge in 2008 E.C in selected schools in the communities which were seriously affected by drought. However, the school feeding program was stopped after 2008 E.C.

I: Do you think the food support program was stopped due to improved productivity of the community or not?

P: Yes, it was stopped because there was relatively better summer and better productivity of the community. But in 2008 E.C, the school feeding was provide in maintain the education of the students. Later, it was stopped because there was better rain and better agricultural productivity.

I: Ok, is there Vitamin A supplementation for lactating and adolescent girls in this community?

P: for Night blindness?

I: Yes,

P: Yes, it is given.

I: How do you describe the youth friendly services (reproductive health services given based on their demand) in this community?

P: In the past, the family health program was providing trainings, reproductive health and other services through opening different service centers in this community, but now there are no youth friendly services for the adolescents in this woreda. In the past, we have of course reached at the services given by the family health program. While we were students, we were discussing in every Sunday and different entertaining modalities were used to create awareness in the adolescents by the family health program. But now the family health program is stopped and the programs are also collapsed.

I: Do you know the reasons for the lack of availability of youth friendly services in the community?

P: Actually, I don’t know about it.

I: Good. So far we have discussed different nutritional intervention for pregnant and lactating women; to remind you; Vitamin A supplementation, Targeted supplementary feeding, ITN, iodized salt utilization, safety net program, home gardening, and related with the pregnant women we have seen the antenatal care and we have also seen the water, sanitation and hygiene. So, among the interventions that we have discussed before, which of them do you think successfully implemented in your woreda?

P: The successfully implemented interventions in our woreda are iodized salt utilization and antenatal cares follow up of pregnant women. However, we have many things to do in the other interventions. Despite there are some gaps, we and the community have owned the iodine salt utilization and antenatal cares follow up of pregnant women. There are some problems even in the antenatal care; there could be one problem in 1000, but our communities have better awareness on iodized salt utilization and antenatal care follow up. However, since we are talking at woreda level, the other interventions are owned by few individuals and not disseminated to all responsible individuals and the community. As we have discussed all the interventions one by one, there are good starts, but in my eyes, the iodized salt implementation and antenatal care follow up are being better implemented in this woreda.

I: Is there any special reason for better implementation these interventions over the others?

P: It is because more attention was given for its implementation, not another reason. But, more capacity building works have been done from the woreda to the Kebelle level. In addition, they have been promoted by different Medias.

I: Ok, from the interventions we have discussed which of them are more successfully implemented in the adolescent girls?

P: We have not done the interventions in the adolescents because attention has not been given for them. As we have described before, most of the interventions are implemented in the women and children.

I: Ok, which of the interventions we have discussed before were less successful? Shall I remind you the interventions?

P: Ok

I: Youth friendly services (for both the in school and out of school adolescents), Vitamin A supplementation (a medication for the prevention of night blindness), Targeted supplementary feeding (a nutritional support for malnourished pregnant and lactating women), advising for ITN utilization, iodized salt utilization, safety net program ( a support given for the food insecure households through working and there are also specific groups supported for free), there is also home gardening as you have broadly described before, counseling pregnant and lactating women for extra meal, counseling for antenatal care follow up and we have also seen the water, sanitation and hygiene and others. These all interventions are directly or indirectly important for improving the nutrition of pregnant, lactating and adolescent women and girls. So, which of these interventions were less successful in this woreda?

P: I forget insecticide treated bed net utilization before; similar with iodized salt utilization and antenatal care utilization, insecticide treated bed net utilization is successfully implemented in our woreda. Even Vitamin A supplementation is relatively well implemented compared with the other interventions. Vitamin A supplementation and ITN utilization are also well implemented in the adolescents because especially the ITN is distributed at household level and as a result they are benefiting from it. When I come to the interventions which are not successfully implemented; we have limited work in home gardening. A pregnant woman should prepare a home gardening but we have not worked yet. Another, we have not solved the dependency of people on safety net program. There are problems on understanding of the people in transferring from the safety net program to self-dependency. In addition, despite there are good starts, we have many drawbacks in implementing the other interventions.

I: What about in the water, sanitation and hygiene?

P: As I have described before, there are changes from the past on water handling, sanitation and hygiene. However, we have problems in water handling and sanitation that is why we are suffering from different diseases, especially acute watering diarrhea.

**Section 4: Implementation challenges and community factors affecting access to nutrition**

I: Good. Generally, we have discussed the relevant interventions for improving the maternal and adolescent nutrition. What are the challenges for implementing the interventions that we have been discussing? You can see the challenges at the individual level, community level or at the intervention level.

P: The main challenge for the implementation of the interventions was related with the community cultures, for example, in the past, our main challenge was home delivery. Mothers believed that a woman should deliver at her home. But now we have broken this kind of culture. There is also shortage of foods and unavailable of access for services despite they need it. However, the main challenge is lack of awareness. If there is lack of awareness, you cannot implement anything. There is also shortage of transport service. For example, the people travel four or five hours on foot to get health and other services in Samre Health Center. So, there is transport shortage. There are also long lasting cultures that hinders the implementation of the interventions. So, our main challenge is community awareness.

I: Is there a situation where the educational level of the women affects the implementation of the intervention?

P: Yes, the main problems in the community are associated with their educational level or illiteracy. The good changes we are observing currently are due to the educated children of the community. If you have educated children, they will educate you at your home. The community cannot understand you easily because they are not educated. Despite they have the awareness, they don’t implement it easily. For example, when you show them the picture of stunted or underweight persons and you tell them its causes they understand it but they don’t quickly implement it. So, the low education level of the community is affecting the implementation of the interventions.

I: Do you think the services or interventions are culturally acceptable?

P: As I have described before there are problems. For example, there are some community members who believe that a pregnant woman should not go out of her home and they also believe that a pregnant woman should deliver in her home. In addition, others believe that the placenta should be burred in home. There are also some people who do not like injections, especially there are some people who still resist child vaccination. But, we don’t have interventions which are against the community culture. With the exceptions of few people, most of the people are accepting the interventions. Hence, despite there are some problems related with awareness, we don’t have culturally unacceptable interventions.

I: Are there different challenges for pregnant women, lactating women and adolescent girls?

P: To some extent the pregnant women, lactating women and adolescent girls related interventions have its own challenges. As I have described before there are community related challenges for the pregnant women, but we don’t have big challenges for the adolescents like that of the pregnant women. The pregnant women may not come to the health facility and the community may not push her to come to the health facility. However, if you want to support the adolescent girls, you will not face difficult challenges. If you appoint them for vaccination, almost all of them come for the service. So, there are no big challenges in adolescent people.

I: What about the challenges related with the interventions themselves? When we say intervention related challenges, it includes quality of services, provider commitment, convenience of the services and others.

P: Yes, there could be intervention related challenges. Especially, there may be less quality services and there may providers who are not needed by the community. For example, if the community adopted only one provider, they don’t like to be served by other health service providers. For that matter, they may not come to take the services in the absence of the adopted health provider. In relation to the quality of services, there are shortages of clean delivery of beds in the health center. Otherwise, there are no major intervention related problems recognized at woreda level.

I: Ok, what other challenges are there related with the targeted supplementary feeding and other maternal nutrition related interventions?

P: There are no challenges with that. As I have described before, Fafa and other foods are provided for in the kebeles. There are some challenges related with the shortage and its utilization. The other challenge is with the diversification (“mitin”) of food. Food diversification was started in our woreda. For example, if a pregnant woman gave you 2kg of one cereal, you will give her a properly mixed Varity of food cereals and others. But, it was challenging to implement it and it is not strengthened yet.

I: For the challenges that you have mentioned, what solutions are being implemented and planned to be implemented?

P: Among the solutions that should be done primarily to solve the challenges is capacity building. If we provided better capacity building services, the challenges can be solved. If you provide continuous monitoring and evaluation, it is possible to bring change. In addition, if the community is served in their nearby area, the challenges could be solved. Instead of requesting the pregnant mother to come four hours on foot to get delivery service Samre health center, if we provide the required equipment and provides in to their nearby area ( around 30 minutes’ walk), we can solve the challenges. Since our woreda is wide and lowland, there are challenges related with geographical setup. If there is supply and capability building interventions, it is possible to solve the other challenges through time. As I have told you, we have started the food diversification in 10 kebeles of this woreda. First the women bring two or three types of cereals to the food diversification center and finally the cereals will be proportionally mixed and will be given back to the mother.

I: What is food diversification (locally call it “mitin”?

P: The food diversification has at least three types; it has cereals, legumes and it includes mainly cereals produced by the farmers. It focuses on providing proportionally balanced foods for the women. For example,

If you bring wheat and maize, you will take a mix of three types like maize, wheat and pea.

I: Is it given for all community members/

P: yes, it is given for all who bring any cereal. For instance, if you bring only wheat, you will take three types of cereals and legumes.

I: Is there separate office for food diversification?

P: Yes, it was implemented in 10 kebeles and materials also distributed to the kebeles, and there has been also one association in Samre. They prepare and grid a mix of balanced included measured sugar and other cereals. So, balanced three or four types will be prepared, packed and given to the community. We had worked very well in the last two years, but now there are problems due to the phase out of the supporting NGOs.

I: Is that food given for adults or children,

P: it is mainly given for the pregnant women and children. If three pregnant women bring three different types of cereals, they swap some portion of their cereals and they take a mix of the three types. So, many women were benefiting from the “mitin” program and were also recognized for that.

**Section 5: Multi-sectoral collaboration to improve maternal nutrition**

I: Ok, I will proceed now to the multi-sectoral collaboration part of our interview. Do you think multi-sectoral collaboration is important to improve the nutrition of women and adolescents?

P: yes, I have already touched it before; all responsible sectors should work together. We have started the multi-sectoral collaboration in our woreda by developing common plan, which is led by the deputy head of the woreda administration and the members are agriculture, health, water, education, women affairs, youth affairs and social affairs. So, multi-sectoral collaboration is mandatory. There will be change if there is common understanding between the sectors. The main problem for the lack of change is due to lack of collaboration between the sectors.

I: How do you describe the role of these sectors in improving the pregnant and lactating women?

P: It is mainly dependent on very few sectors, and we can say it is specifically dependent on the health sector. Only the health sector is worrying about it, but the other sectors are not actively working on it. The other sectors are not working on it apart from their plan.

I: Ok, is there a coordinating platform in enhancing multi-sectoral coordination in maternal and adolescent nutrition in your woreda?
P: A committee has been established.

I: Could you tell me the members of the committee?

P: The committee is chaired by the deputy head of the woreda administration and the health office is the secretary, while the remaining agriculture, water resource, education, women affairs and town development are members.

I: As you have informed me there are multi sectors established to improve the maternal and child nutrition and health. So, what do you think are the challenges for the coordination of the sectors for improving the health and nutrition of the women and adolescents?

P: The main challenge is lack of attention and ownership. They may have a plan, but they have not implemented and owned it yet unlike the health sector. Only the health sector has owned and carried it. As we have said before, if attention is not given at the top level, the work will be thrown to only one sector. So, the main challenge is lack of attention.

I: What do you recommend to bring all sectors on board and to bring changes?

P: First there should be accountability and all sectors must be asked about where they are in terms of implementing the agreed interventions. If there is no accountability, they will not work on it. The chair person should regularly evaluate all committee members. In addition, another committee should be established to lead the existing committee and it has to be evaluated quarterly. If you are not evaluated quarterly, you will repeat your current mistakes again. However, you will not repeat it if there is evaluation and monitoring. To implement, it should be part of their measurement and it should have evaluation in every quarter. If one work has evaluation, it will be successful. If it doesn’t have evaluation, it will not be successful.

I: To what extent your sector is working to improve the pregnant and lactating women and adolescent girls’ nutrition?

P: As agriculture sector, we have not worked in detail on the nutrition of women and children. As I have described before, we have good start on home gardening and agriculture in collaboration with stakeholders, but we have not worked in broader score to improve the nutrition of women and children in all kebeles. So, the activities performed are distribution of Fafa and home gardening. As agriculture sector, if we deeply work on it and there are supplies, we can improve their nutrition.

**Section 6: Other interventions that influence adolescent and maternal nutrition and health outcomes**

I: We are now in the last section of our interview. In your opinion, how do you describe the benefits of delayed marriage (after 18 years of age) for improving maternal nutrition?

P: We have not worked on the adolescents at all and we have not seen it as a program.

I: So, do you think delayed marriage (after 18 years of age) is helpful for improving maternal nutrition?

P: There are not only nutritional changes but also there are all round changes in the adolescent girls. When we compare it with the past, there are changes in their education and other aspects. When they married late, their level of maturity will be increased and they will have better awareness.

I: How do you describe the benefits of child delivery spacing for improving maternal nutrition?

P: Child spacing is very important for maternal nutrition because if the space between the births is small, the child will not get the breast milk despite he/she was expected to get it up to two years and he will not get good child care. However, if there is spacing, the child will get the breast milk as per he needs. If the mother gets pregnant within very short intervals she may be exposed to different diseases. In addition, if the family large size, they may face food shortage. Besides, if she is giving birth after birth, she will not work to support them, instead she will be engaged with the children and the provided support will not be enough for them. Hence, child spacing is very important for maternal nutrition.

I: Good. How do you describe the activities done in this woreda to prevent early marriage and to promote child birth spacing?

P: A lot of works has been done in this woreda for many years not only to for child birth spacing but also to have balanced children.

I: Do you think the community is implementing it?

P: We don’t think all of the community members have implemented it because there are observable problems like birth after birth and very large family size households. So, we don’t think all are implementing the advice given to them, but there are changes. What was the second?

I: Early marriage

P: We are working on the prevention of early marriage. Currently we have a new guideline for marriage. The marriage approval is conducted by committee at Kebelle level. If one girl wants to marry, she is expected to come to the Kebelle with her husband and three testimonies for confirming her age and for marriage registration. There is a situation where the committee cancels the marriage of underage girls. However, sometimes, early marriage is observed by cheating the committee members. Though it is not totally eliminated, there are good changes.

I: How do you describe the education given in the community and schools to prevent early marriage?

P: education is given to prevent early marriage in schools, in meetings and in other different stages. Even separate education for the adolescent girls is given to prevent early marriage in the community. However, the awareness creation may be reached to all communities and areas.

**Additional remarks**

I: I have completed my interview questions. As I have informed you in the very beginning, the objective of this study is to know the nutritional problems of pregnant, lactating and adolescent girls and to identify the individual level, community level and intervention level barriers of nutritional interventions, and to design strategies accordingly. So, if you have any additional suggestions?

P: As we have described starting from the beginning, more or less all the issues have been raised. Everything is described, what I want to recommend is that, we should not be restricted to only home gardening. We have to create linkage with the animal products and other productions. Our main problem is we follow pregnancy up to child birth and we also follow the under five children, but we ignore the adolescents. The other things are already described. Thank you.

I: Thank you very much

**Summary**

Section one: Common maternal nutrition problems in the community

- Moderate and severe malnutrition secondary to postpartum hemorrhage and food shortage are sometimes observed in the study woreda
- Night blindness and anemia are also observed in pregnant and lactating women
- Stunting and underweight are commonly observed in the adolescents

Section two: Interventions that improve adolescent nutrition

- There is no nutritional intervention for adolescent girls
- Most of the interventions given for pregnant and lactating women are provided by the health sector
- The nutritional interventions provided by the agriculture office for the women are only provision of fafa and home gardening
- Iodized salt utilization, ITN utilization and antenatal cares follow up of pregnant women are the successfully implemented interventions in the woreda
- Home gardening, youth friendly services, water, hygiene and sanitation are not successfully implemented in this woreda
- Community culture, poor community awareness and low education level are the main challenges for the implementation of the interventions
- Poor quality of services like shortages of beds and lack of transportation are the main challenges for the interventions utilization.

Section 5: Multi-sectoral collaboration to improve maternal nutrition

- The interventions for maternal and child health are almost dependent on the health sector.
- Despite there is established multi-sectoral committee for maternal nutrition, it is not functional
- Lack of attention, ownership, commitment and coordinating platform are the main problems of the sectors

Section 6: Other interventions that influence adolescent and maternal nutrition and health outcomes

Section: seven: additional remarks
