# Supplementary material for: Barriers to utilize nutrition interventions among lactating women in rural communities of Tigray, northern Ethiopia: An exploratory study
Source: PLoS One. 2021 Apr 30;16(4):e0250696. doi: 10.1371/journal.pone.0250696 (PMC8087028; doi:10.1371/journal.pone.0250696)
Supplement: S2 File — (ZIP) [file pone.0250696.s002.zip › S2_File.Doc/Woreda level and above key informants/080_IDI_head for Agricultur office_Thanqua Abergele woreda.docx]

**Operational Research on Adolescent and Maternal Nutrition in Northern Ethiopia**

## **In-depth interview responses of the agriculture and rural development head**

**Introduction**

Thank you for your acceptance of the informed consent form and for taking the time to speak with me today. I have questions to ask you which were prepared in advance. The discussion will take 1-2 hours. If you have any questions before we begin please feel free to ask.

**Section A: Interview in details**

1. Zone: Central Zone
2. Woreda: Tanqua Abergelle
3. Kebele: Mearey
4. Name of key informant: Haftom Zenebe
5. Institution of key informant: Agriculture and rural development office
6. Interviewer name: Mekonnen Haileselassie
7. Date of interview: 04/03/2010
8. Interview start time: 3:40 AM (local time)
9. Interview end time: 5:30 PM (local time)

**Section B: Socio-demographic and basic data of qualitative study participant**

| **Socio-demographic characteristic** | **KII** |
| --- | --- |
| Sex | Male |
| Age | 35 |
| Educational status | Bachler degree |
| Occupation/role in the community | Head of agriculture and rural development office |
| Service year | 10 years |

**Note:**

I: interview

P: participant

**Section 1: Common maternal (pregnant women, lactating women and adolescent girls) nutrition problems in the community**

**I:** In your opinion, what are the common nutrition problems in the community for women? What about for adolescent girls?

**P:** There was a study that indicates high prevalence of stunting among children in our woreda which was found around 41% at first study and it was reduced to 38% during the second study. As the study indicated that we are the second next to Afar region in the high prevalence of stunting in the country. Therefore, this shows that there is great unbalance of the malnutrition prevalence among the community with the agricultural production and productivity rate of the region. Our woreda has the highest prevalence of child stunting in the region.

There is high prevalence of moderate malnutrition and stunting in children. Unbalance of height and their age which is low height among children in this woreda is very common. We have reports from the health sector about stunting, underweight and wasting; and it indicates that how much the problem is devastating and that needs great concern by the responsible stakeholders. The micronutrient deficiencies are also our concern of the community. There are cases observed like anemia, and night blindness among the mothers of pregnant and lactating once.

Pregnant and lactating women and children are the most susceptible groups for malnutrition; mainly the problem is high in pregnant women, because the child inside their body needs more food who is obtained from the mother; and pregnant women could not move from place to place for search of foods unless they prepare early or before they get pregnant; and this type of early food preparation for the time of their pregnancy is not common in our community; no pregnant women could prepare foods for their pregnancy time. Relatively it is better in lactating mothers who prepare flours for porridge purpose, butter and they slaughter sheep or goat during the first lactation time. In general the pregnant and lactating women have double burdened both for their child and for themselves and that why they are sensitive to malnutrition. The body of the children is also very soft and not gets well developed who is sensitive to infection.

The root causes of malnutrition in pregnant and lactating women is lack of balance diet foods, poor knowledge of food preparation and consumption; for example they don’t know when iodized salt is added to wot, simply they add at the beginning and lost through evaporation. The same is true for vegetables and other food items that cook in high temperature and it could denature the main contents of the foods. The main problem of malnutrition in pregnant and lactating women is also the inaccessibility of the necessary food groups on time like vegetables, cereals and pulses. The other problem is the poor feeding habit of the mothers even the different food types are available at their home. We didn’t create the awareness among the mothers about their feeding system; what type of food is important during pregnancy, lactation, for children and adolescents; and the feeding interval; which food groups are included during the feeding time and so on are not well recognized by the community and also we experts didn’t educate the community in this scenario.

All the home grown food groups are not properly fed by the pregnant and lactating women. For example home grown products like honey, eggs, butter, nut, sesame and others are intended for market purposes. We didn’t coach the community to feed first for themselves so as to prevent malnutrition. Due to the awareness gaps among the community that could lead to child stunting in the community.

**I:** How do you explain problems related to overweight?

**P:** There is an understanding in the community that feeding means filling of stomach regardless of the food quality and food type. This type of thinking is not only in the illiterates but also in the literate once. If everybody has got Enjera with Shiro wot in ad libitum way, they considered as if they are feeding nutritious foods; this is the main knowledge gap among the communities. But balance diet is not consuming of one food type so as to fill our stomach, it is beyond it. There is different food groups produced in this community; all the cereals, pulses and animal products are produced from the farmers; the gap among the community is lack of knowledge on the feeding system. Although overweight is not as such our community’s problem like underweight, we observe in elders of few individuals.

In the past time, we didn’t care about balance diet rather filling of stomach was our target. We didn’t feed the home grown food types properly and on time. In our community, there is also a culture that mothers are prepare the quality foods like eggs, chicken, honey and butter to their husband rather than to eat themselves and provide to their children. They thought that the husband works a lot in the farm activities like, he ploughs, irrigate, build or weed the whole day without rest; and at the effort of him the whole family is survive. This culture is not banned. For example if a mother prepares chicken, she could not eat without the husband visit; and this type of culture is very common in our community. But the reverse is true; especially pregnant and lactating women should get the quality foods rather than the husbands. The development of the child’s mental and physical status is determined by the type and amount of mothers’ feeding condition. If a mother feeds the quality food, the child could get all the quality foods from his/her mother.

But most of our community doesn’t give much emphasis to quality foods if they get any food source to fill their stomach.

**I:** How about in relation to food insecurity?

**P**: Being we are leaders, we know the problem of malnutrition among pregnant and lactating mothers; the food shortage in the community and the existence of poor feeding habits are the main problems for the cause of malnutrition among the pregnant and lactating women of the community. In case of poor feeding habit, although there are agricultural products like sorghum, teff, bean, sesame, and livestock products like eggs, milk, honey, butter etc. in their home, due to their poor feeding system that could suffer by malnutrition.

The other groups are due to the shortage of food groups in their home; they are not food secured community and not the beneficiary of home garden vegetables, thus due to lack of food source in their home could cause malnutrition; in this case, we are providing different aids either in the form of targeting supplementary foods for children, pregnant and lactating women, and they engage in the food safety net and emergency aid program.

But when we compared the two problems that is the food shortage in the community and the existence of poor feeding system among the community, the poor feeding system is the highest problem among the communities; you can confirm by assessing their production income and their nutritional status of the community. Almost all the households are affected by the problem of malnutrition due to the lack of knowledge on the feeding system; the type, amount, and preparation method of the home grown foods could affect the nutritional status of the pregnant and lactating women in the communities. For example there are households grouped in the rich community, and they could fill their stomach without any problem of one type food deficiency but due to their poor feeding system still there are many under nutrition children from the rich. This is an indication that how much the influence of poor feeding system is prevalent in our community. Formerly, the agriculture sector was more devoted to increase the agriculture production and productivity among each household; and the health sector was also more focus to the infectious diseases; that is why the poor feeding system in the community is high prevalent.

**I:** Why the pregnant and lactating women are affected by malnutrition?

**P:** In accordance of their sensitivity to malnutrition, the pregnant, lactating women and adolescent girls are more vulnerable to malnutrition respectively. In pregnant women, the consumed food is shared between the mothers and the child; we don’t consider the dose of the pregnant women’s food, we count as one and if she could not eat the balance diet, we don’t know how much the child could affect his physical and mental development; we should think the development of the child’s cell is derived from the mother’s body. Therefore, the whole family especially the husband should understand the burden of pregnant women. Next to the pregnant, the lactating women are affected by malnutrition; because the mother provides milk to the child; here the child gets enough amount of milk if and only if the mother feeds balance diet foods. So it is clear how much the diversified food is important for both the pregnant and lactating mothers; the only difference between the pregnant and lactating women is that the former doesn’t move from place to place for search of food and the latter could move.

Both the health sector and the agricultural office didn’t give more concern to the adolescent girls’ feeding status. We have steering committee that discusses and brings a direction for malnutrition prevention in the community; in this committee, the woreda administration head is the leader, the head of health office is the secretory and we are the members; and this committee is not discuss about the adolescents, we only talk about the pregnant and lactating mothers and children. The steering committee is discussing about the overall nutritional status of pregnant and lactating women and we shared our tasks based on our fields of specialty.

We the agriculture sector encourage the community to increase the production and productivity, to produce home garden vegetables, livestock production and providing of seeds, quality breeds; the health sector also provide training about balance diet food, feeding time, amount, interval, sanitation, and screening of pregnant and lactating women and children of nutritional status; the women affairs and women association also give training to women to keep their food security, to stop underage marriage and the education sector could also promote about nutrition through the students and it is good place especially for the adolescent girls to get training in the school about nutrition and the family could get awareness through them.

**Section 2: Nutrition priorities in the woreda**

**I:** What priorities do your institution has in relation to maternal and adolescent health? Why?

**P:** We give priority on the growth of production and productivity of the agricultural products of the farmers. In this scenario, we provide training to the farmers, about the utilization of best seeds and fertilizer and we also introduce livestock packages like selective breeds of dairy cows and chicken to the farmers. The plantation of home garden vegetables among the community is the additional task of the agriculture sector. The water and soil conservation and production of compost around the homestead of each farmer are the assignment of the agriculture sectors.

**I:** What your institution is doing currently related to the priorities you have mentioned in relation to nutrition in pregnant and lactating mothers? What nutrition interventions have the most resources allocated to them?

**P:** All I have listed before as priority of our activity is related to nutrition. For example if we increase the production and productivity of farmers’ crop production, the pregnant and lactating mothers could get the food access from the produced agricultural products; and we help them to produce diversified food groups like cereals, pulses and tubers and we advise them to consume the pregnant and lactating mothers from the produced food groups. The same is true in the area of livestock production; we advise them to consume the eggs, milk and butter for themselves rather than for market purposes.

The pregnant and lactating mothers don’t participate during the soil and water conservation campaign. And in the food safety net program, the pregnant and lactating mothers get the service without involving the work. We also work on the feeding style and sanitation issues of the pregnant and lactating mothers in collaboration with the health sector. The supply of the targeted supplementary food to the pregnant and lactating mothers and to the children is given through our sector after they screened their nutritional status at the health sector.

There are organizations working on the improvement of the nutritional status of pregnant and lactating mothers like REST, SURE who works in the introduction of chicken and home garden vegetables; thus the implementation of the project is done in collaboration of our sectors.

All activities in the area of nutrition improvement are working in a collaboration mood. Nutrition improvement by its nature needs multisector collaboration that is why the activity is led by steering committee. If the agriculture sector improves the production and productivity of agricultural products; the health sector creates awareness among the community how to eat, how to prepare and who needs more nutritious food groups; the water sector also provides water access and the education sector promotes the importance of balance diet in our body etc.

**I:** How do you see the changes?

**P:** Currently the integration among all stakeholders is very strong; now as a sector, we are not only focusing on the production and productivity but what food types are more productive and more nutritious to the pregnant and lactating mothers; how to harvest the crops without losing its nutritional content and in collaboration with the health sector we are providing training to pregnant and lactating mothers on how to consume the produced food groups. For example there was no nut (ለወዝ) production in our community; now it is introduced which has production and in terms of its nutritional content is also very essential food groups. Agricultural food groups like cereals (teff, sorghum, wheat), pulses (bean, chickpea and livestock products like honey, milk, butter are the main products of this community.

**I:** How do you evaluate the priority given for the interventions to the women?

**P:** Our sector is basic for the production of the diversified food groups from the agriculture farm. As I have said before, one cause of malnutrition is the shortage of food in the farmers; if there are cereals, pulses, animal source foods and vegetables and fruits, one part of the problem could be solved. Thus the agriculture sector helps the households to keep the food security and through boosting their production and productivity; in addition to this, we educate the households’ feeding system mainly to the pregnant and lactating mothers.

Now the intention of the farmers towards production and consumption of the produced crops from the agriculture farms is improved. But we cannot say the attitude of the community is totally changed in terms of their consumption level. For example we supply more than night thousand quintal of sesame to the Ethiopian commodity exchange market every year; but we don’t consume the products for ourselves. The same is true in nut, egg, honey and butter.

Now, we the agriculture and health experts have a common plan with the objectives of improving the nutritional status of pregnant and lactating mothers. As I have said, we are working to increase the agriculture production and productivity and on the improvements of household consumption in collaboration with the health sector; and we are now observing some improvements among the community’s feeding system; for example some of household have started eating animal source foods like milk and eggs. This improvement becomes after we provide a number of trainings to the pregnant and lactating mothers.

**Section 3: Nutrition interventions that improve adolescent and maternal health**

**I:** What kinds of nutrition interventions are in place to improve health of the pregnant and lactating women to your level?

**P:** During the screening and providing of the target supplementary foods to the pregnant and lactating mothers, there is education how to improve their nutritional status like their feeding system and how to improve the production and productivity of the households’ economic status by the collaboration of the health and agriculture experts. For example the main target of SURE program is to integrate the health and agriculture sector; and how both sectors provide training to the women mainly the pregnant and lactating mothers like to produce home garden vegetables, chicken production; and their feeding system.

There is also a demonstration activity at the farmers training center for the pregnant and lactating mothers how to produce home garden vegetables, chicken production and how to prepare foods without losing its content and the like. Materials like shovel, hoe, dish, knife, spoon and pitcher are also provided by SURE program.

We also introduced sweet potato which is important for the improvement of the child’s nutritional status in the community. The SURE program also works on the introduction of inputs like chicken, the seeds of vegetables and fruits for the poor. Around 10% of the poor who are supported in the safety net will be provided the chicken and the seed of vegetables and fruits by SURE program. Training is provided by the health and agriculture experts for the poor who are intended to provide the inputs. Inputs also like the home garden seeds such as swiss chard, spinach, green paper and onion have been provided.

Now we start to create awareness among the individuals of the pregnant women and lactating one; they start to be benefited from the integration of the two sectors like production of home garden vegetables, chicken production and the consumption level of the women increases every time. They also start to think the importance of self-consumption than for market purposes.

**I:** Do you advise pregnant and lactating mothers to consume extra meal and diversified foods?

**P:** Yes, we are advising the pregnant and lactating mothers to consume extra meal and diversified foods in collaboration with the health sector. AS I have said before, during the screening of mothers, at time of the safety net support, and during the introduction of different agricultural packages like home garden and animal production technology, trainings are given how to consume diversified food groups and the importance of extra foods during pregnant and lactation period. During the training, the husbands are parts of it; because most of the husbands are the heads of the households; and we educate the husbands, how much mothers are affected by malnutrition due to the lack of balance diet foods, workload, and due to the personal hygiene and sanitation problems.

Even though the change is not as such radical, there is an improvement on the health status of pregnant and lactating mothers; for example almost all pregnant women are delivering at the health center; so this scenario could contribute for their health status. This development is due to the awareness created among the community; the same could be happened on the feeding status of the pregnant and lactating mothers. Now in most of the households, the awareness is created; the husbands start sharing the workload of women like sharing of water fetching, carrying the fire wood, providing quality food for pregnant and lactating women.

**I:** How does your institution contribute in the reduction of workloads among the pregnant and lactating women?

**P:** There is a profile about the names of all pregnant and lactating women in the health sector; thus all the pregnant women are not participating in the safety net program works and the lactating mothers could get the service until ten months of lactation without involving in the work. We also advice the husbands to fetch water by themselves using the back of donkeys so as to reduce the mothers workload; but we could not say the pregnant and lactating women get full rest; because they are busy in the household activities although we prohibit them from the soil and water conservation; since the house activities are the most restless task.

**I:** What advices do you contribute on nutrition sensitive agriculture such as home gardening?

**P:** There is a shortage of water sources in most of this community; but we are doing in collaboration with REST to plant home garden vegetables that could use carrying of water through the back of donkeys; and some of them benefited for their consumption and for the market purposes. On the other hand, there are households that don’t produce any home garden vegetables even some have the access of water source. But during the summer season every farmer plants green paper, tomato and swiss chard as home garden vegetables; this home garden planting habit is taken as a culture and every farmer leaves a plot during summer for the home garden vegetables. They use water collecting groove during the summer season and it could serve as supplementary irrigation at the end of summer; using this technology most of the farmers produce home garden vegetables during the summer season. But since the area is very high temperature, the collected water in the groove during the summer could easily evaporate and it doesn’t stay long. On the other hand there is Tabias like Felegehiwot that affect by draught; pregnant and lactating women travel long distance to fetch water although there is a plan to construct check dam.

**I:** How pregnant and lactating women benefited from the productive safety net program and food security?

**P:** There are elders and disables that are supported the whole year; and some poor individuals are supported by the safety net program and the third group is the rich who don’t need any support except in case of draught who supported in the form of emergency aid. In the case of safety net program, pregnant and lactating until ten months of lactation don’t engage in the work; they freely use the service aid.

In the case of food security, we strive to increase the farmers’ production and productivity through using different technologies like introducing of best seeds, fertilizers and selected breeds. Now a days we introduce high quality seeds in terms of production and its nutritional content to our community like special sorghum and nut. Therefore, the pregnant and lactating women are encouraged to involve in the production of high quality food groups so as to keep their food security. We also provide trainings and different inputs that could contribute on the improvement of crop production and productivity like best seeds, fertilizer and selective breeds.

As a culture, our farmers don’t eat what they produce rather they use for market purposes; there are a number of house expenses and to fill these expense, they could sell their products rather than for their own consumption; for example they sell honey and purchase sugar, here there is great price difference between honey and sugar and the extra money uses for their children schooling and other home expenses. Now in the soquota declaration, we will do how quality and quantity products are available in the home of the households; and how the cash crops could sell with high price and purchase diversified foods with fair price so as to consume and improve the nutritional status of the pregnant and lactating mothers; the feeding style of the community will be also improved.

**I:** How about your advice on water, sanitation and hygiene services?

**P:** In relation to water supply, we are working in collaboration with the water sector and we are working in the water and soil conservation activities so as to increase the ground water. There are Tabias that increase its water source as the result of the water and soil conservation activities. The other task on the water sanitation and hygiene is responsible to the water sector through constructing the nearby water sources to the community and treating the water by guard water and fencing the water site. We are also working in the environmental sanitation in collaboration with the health sector through cleaning and compost production of the wastes around the homestead. There is also the biogas production which can be produced from the agricultural waste, manure, municipal waste, plant materials to be used as fuel; it can be used for any heating purposes, such as cooking; thus the waste materials are cleaned for the biogas and compost purpose; therefore, the burden of women from using fire wood, and preventing from the smokes of fire wood that could affect the health of mothers. This technology is introduced into three Tabias as a pilot project and it will be extended throughout the woreda as the soonest possible.

But there is a weakness in relation to the use of toilet among the community although it is constructed. There is also a problem of rejecting the pipe water and they fetch water from the natural river due to the saltiness of water from the pipe which is not used to make local alcohol and other food types; due to this reason we were affected by water borne diseases last year.

I: How about the distribution of insecticide treated bed nets for the pregnant and lactating mothers?

P: The health sector could explain about this issue; but we know that these resources are given as priority to the pregnant and lactating mothers; and resources like the use of insecticide treated bed nets and iodine salt are not the problem of our community.

**I:** How is the service of targeted supplementary feeding (TSF) for pregnant and lactating mothers?

**P:** This service is done by the collaboration of health and agriculture sector; the health sector screen the pregnant, lactating women and children and those who are more affected by the problem of malnutrition could get the service. We have the focal person of the targeted supplementary feeding service organizer; we also follow their consumption level at their home so as to reduce the problem of sharing among the family households; this is mainly the task of the health extension workers and the women development group.

**I:** Which of the interventions listed before do you think is most important for the pregnant and lactating women?

**P:** The integration among the health and the agriculture sector is basic for the improvement of the nutritional status of the pregnant and lactating women in the community. Changing the feeding behaviour of the community mainly the pregnant and lactating women is very crucial; unless we do on the feeding style of the mothers, the availability and production of diversified foods could not solve the problem. It is also very important to teach on how could to offer the available foods to the pregnant and lactating mothers; time of consumption, extra food during pregnancy and lactation; how the husband could reduce the mothers’ workload; and how feeding priority is given to pregnant and lactating women in the community. We always teach the pregnant and lactating women as well as their husbands on their feeding system at any meeting days; this is given in collaboration with the health sector and which is considered as the part of the safety net activity and the training could be taken as if the pregnant and lactating women participated in the work of the safety net program; this training is given two times per week and we always evaluate the feeding status of pregnant and lactating women and the implementation of the inputs provided to them.

**I:** In your opinion, which of the interventions for the pregnant and lactating women are being implemented in successful way? Why?

**P:** It is very difficult to say our plan is fully implemented in a successful way at the ground; because as a sector, the attention of nutritional improvement to the pregnant and lactating women was given in recent times. We were focusing on the development of agricultural production and productivity; and we were working in a separate way with the health sector. Therefore, the integration between the health and the agriculture sector towards the improvement of nutrition to the pregnant and lactating women is great success. Although the practical implementation is not as per our expectation, the awareness creation about the importance of nutritious foods to the pregnant and lactating women among the community is in a better way; now the family and the community in general is understand that pregnant and lactating women needs extra and diversified food than others. Therefore, to implement all our plans and activities on the ground and to achieve the missions of Soquota declaration to get free of child stunting by 2030; we are expecting to work more on the area of nutrition to improve the nutritional status of pregnant and lactating women.

Now as I have said before, the community understands that extra meal is needed to pregnant and lactating women; but what does mean extra meal? At the community, they don’t clearly understand the occurrence of food and balance diet foods; for example many households keep their food security, they eat at least three times per day and they don’t get hungry, but their feeding style is not changed and they feed the same type of foods at every feeding time; but the availability of food is not their problem; for example they have sesame, nut, other cereals and pulses and animal source foods like eggs, milk and honey; thus they keep and eat only one type of foods and sell the other food products.

**I:** Do pregnant and lactating women prepare diversified foods for their pregnancy and lactation period?

**P:** Compared to the pregnancy time, lactating mothers are better preparing foods for their lactation period; as a culture here in the community, it is mandatory to prepare butter for lactating women; they also arrange teff flour or wheat but there is no any trend to prepare for their pregnancy time.

**I:** Which of the interventions targeted to the pregnant and lactating women was less successful? Why?

**P:** All our efforts are not properly implemented at the ground; in the community side, the awareness about the importance of consuming diversified food mainly to the pregnant and lactating women is not properly developed among the communities. In the agriculture expert side, we didn’t solve the shortage of food by increasing the agriculture production and productivity using the introduction of best seeds, fertilizer, home garden vegetables and selected breeds. The other weakness of our experts is not solving the poor feeding behaviour of the pregnant and lactating women although there is no problem of food types at their home; this is also a great gap among the community and we didn’t aware the pregnant and lactating mothers of their feeding style based on the home grown products, such as the feeding time, amount and food types.

**Section 4: Implementation challenges and community factors affecting access to nutrition interventions**

**I:** What are the challenges to implement delivering the nutrition interventions that we have been discussing for pregnant and lactating women?

**P:** As a sector we were giving high emphasis to production and productivity than nutrition improvement among the community; for example our region has high prevalence of child stunting compared to other regions, this is not due the lack of agriculture production, there is high irrigation development, high crop and animal production which was not before; but the great gap of our sector and the community in general towards the nutrition improvement was due to our focus that we were striving only to increase the agriculture production and productivity rather than nutrition improvement on the pregnant and lactating mothers.

**I**: How about the awareness of the community?

**P:** The gap of awareness on the nutrition improvement is not only the community side but it is also the experts’ knowledge gap; we didn’t assess what is excess and deficient produced in the community and how we could fill the gap; there are also deficiencies in the area of basic commodities at the market, but it is not long time problem; the main problem is less understanding of the importance of food items available in our home grown; for example if there is no oil at the market, the community could have the opportunity to use sesame; it has high oil content which could substitute the market oil; therefore, the trend of our experts to teach the community about substituting to market oil by the local product sesame is not well done; there are many households that are consuming injera with salt instead of pounding the sesame and use as wot in their dish.

Therefore, the feeding habit of the pregnant and lactating women and the community as a general is not well improved; for example, from the 365 days, the number of fasting days is as many as the non-fasting days; during fasting days the pregnant and lactating women are not allowed to eat the non-fasting foods and we can guess how much the mother and her child is affecting by the lack of balance diet foods. Although it is allowed by the religious persons to eat non-fasting foods during pregnancy and lactation time, the pregnant and lactating women could not accept to eat the non-fasting foods during fasting time; they totally accepted the habit as a culture; they also stay fasting without consuming any food type until four-six hours, so the time of feeding is also affected.

There is also the development of the sprite of dependency mentality among the community so as to get governmental aid, some households sell their quality diversified food products and purchase one food groups; this is also the other problems in the community and we are expecting to change this attitude among the community.

**I:** Is there a relationship between educational status of pregnant and lactating women access to interventions?

**P:** Yes, there is great and basic difference among the literate and illiterate community to apply the intervention that is given by our experts; for example when you give training the literate pregnant and lactating women are easily accepted and applied accordingly; whereas the illiterate once are not easily accepted the training and not applied accordingly; sometimes they suppose that the training is given as if the benefit is for the trainers. Although the comparison between the two groups is better in the literate once, the knowledge of nutritious foods on the effect of pregnant and lactating women is poor in both groups; there is no well awareness like if pregnant and lactating women eat a balance diet food the physical and mental development of the child will be improved.

The education sector also educates the students about the importance of nutritious food for pregnant and lactating women so as to influence their family; since there is a tradition that the mothers and fathers are accepting their sons/daughters’ advice; so this approach is also good method to improve the nutritional status of pregnant and lactating women.

**I:** How the belief and norms affect the feeding behaviour of pregnant and lactating women?

**P:** As a culture, there is no any assumption that pregnant and lactating women could feed better food than their husbands; if the husband doesn’t go to home during lunch time, women could stay fasting the whole day; this is very common in our community; so we should work on changing the attitudes of the community that pregnant and lactating women are demanding the quality foods on time. Concerning the religious issue, I have already discussed, fasting could affect the nutritional status of pregnant and lactating women; therefore, this is also another intervention area of the community. There is also a statement that if a pregnant woman eats meat and fruits, she may have a big baby which endangers her life by making labor difficult.

**I:** Does the intervention access affect by transportation and cost?

**P:** Yes, we have transportation problem in our community; mostly the community uses the back of donkeys for transporting of water to fetch from its source. Some may have not donkeys and they fetch water by their back; it takes about two-three hours for round trips to fetch water; in this case even the pregnant and lactating women participate in the fetching of water. The cost of some very demanding food products especially during summer season is also another preventing factor for intervention; for example the price of all cereals, pulses, animal source foods are costly during the summer season.

**I:** How convenience is interventions to the pregnant and lactating women?

**P:** Currently there is good improvement in most of the nutritional improvement of pregnant and lactating women; but it is not a radical change among all the communities; when you teach them, some of them are getting convenience about the intervention and others are not. The experts are not also clearly understanding to teach the problem with its solutions to get diversified foods for the pregnant and lactating mothers; what innovative technologies could we introduce so as to solve the problem, how could we convenience the participants to implement their trainings, how could we find and follow the pregnant and lactating women of their feeding style; how the home grown agricultural products prepare and consume in balance diet way and the commitment of our experts to solve the gaps is also our weakness.

**I:** How do you explain the quality of the intervention?

**P:** As I have said before, we have limitations on the knowledge and commitment of our experts; for example it is our weakness that the poor feeding style of pregnant and lactating women of the rich households; if they have all diversified foods in their home and they become poor nutritional status which is due to poor understanding of the importance and feeding system of them; therefore, we didn’t convenience and provide quality service. Therefore we are lagging behind in the improvement of the nutritional status among the pregnant and lactating women of the community; we were aiming the growth of production and productivity rather than improving the nutritional status of pregnant and lactating women; that is why our experts do not well understand about the improvements of nutrition in pregnant and lactating women.

**I:** How do you evaluate the commitment of the intervention providers at your level?

**P:** Since the area is low land with high temperature, there exist turnover of experts; but the main problem in our sector is the lack of attention towards the nutritional improvement in the community; for example our experts give attention to introducing best seeds, quality breeds, fertilizer, and anti-insects; rather than improving the nutritional status of the community and that is why we start an integration between the agriculture and the health sector.

Now we understand that we build healthy citizens through the improvement of nutritional status of the community, therefore, in collaboration with the health sector and other NGOs that we will create great awareness among the community about the importance of nutrition for the community especially to the pregnant and lactating women; and we will improve their food security and feeding style of the community; we will also work on the changing of attitude of the community concerning the treatment of pregnant and lactating women in their feeding aspect, reduction of workloads and the like.

**I:** What other factors are inhibiting implementation of the interventions? How?

**P:** The main factor is our poor understanding of the nutrition agenda; it was not our task and not given any concern to it; in case of the health sector, it was their main task but in the agriculture sector, the agenda of nutrition was not known; we only concentrate on the growth of agricultural production and productivity and market oriented products. Our experts also didn’t get more training and experience sharing about the improvement of nutritional status among the pregnant and lactating mothers in the community.

The other factor is the presence of poor awareness among the community; due to their poor understanding about nutrition, they don’t consume the home grown products on time and in a balance diet way. The presence of deep rooted beliefs, culture and norms like the fasting issue, engagement of pregnant and lactating women in high workloads, high respecting to husband than pregnant and lactating women to eat quality foods, the sprite of dependency mentality that could inhibit implementation of our interventions.

**I:** For the said challenges, can you tell me of any solutions that your institution have applied to effectively implement the interventions for pregnant and lactating women?

**P:** The weight of the work should be shared among the respective stakeholders; it should start from the regional bureau to the zonal administration and then to the woreda offices; it should not be done in the form of campaign rather it should be taken their own plan and evaluated its performance at the end of day; if it is performed in such a way the credit concerning the nutritional improvement could also increases; there should be an accountability for each individual’s plan performance and strict follow up of the changes that are made among the communities is very important.

The integration among the responsible bodies should be strengthening; we the leaders should act as models to follow and implement the activities; this program is not achieved as individual level rather it needs an integration with many stakeholders, like sectors of the health, agriculture, water, education, civic society, women affaire and the like.

In the community level, we should change the attitude of the community on the feeding style and more credit should be given to the mothers; we should understand that mothers are the source of life; we all are from mothers; healthy mother creates healthy child thus to build the healthy citizen, we should invest on the health and nutritional status of mothers. To create awareness, it doesn’t take time among the communities; the only needs the commitment of us.

**Section 5: Multi-sectoral collaboration to improve maternal nutrition**

**I:** Do you feel it is necessary at your level to work with other sectors/ institutions to address maternal nutrition? What about for the adolescent girls’ nutrition? Why?

**P:** Yes, the nature of nutritional improvement in the community, a sort of holistic approach is needed; it could not bring a radical change with a single sector; that is why the prevalence of malnutrition in the country and the region is very high which was run by the health sector only. Therefore, integration is the only option to improve the nutritional status of pregnant and lactating women.

In the case of adolescent girls’ nutrition status, we didn’t do any activities and even now they are not our target groups; but the education sector could work on the training aspect; they could educate in the science part and also there is a nutrition club that promotes the importance of nutrition for child development and on the improvement of mothers’ health status.

**I:** Which other sectors do you feel are necessary to work with your institution?

**P:** The health sector, agriculture, water, education, civic association, and the women affaires are the main stakeholders in the improvement of the nutritional status of the community mainly the pregnant and lactating mothers.

**I:** How do you see the other institutions’ role complementing your role in improving maternal and adolescent nutrition?

**P:** The main task of the agriculture sector is to increase the production and productivity of the agricultural products; what diversified food types are appropriate to grow in the plot and how the crops are harvested without losing their nutritional contents; how it is stored and how the pregnant and lactating women feed is the main activities of the sector.

In case of the health sector, the follow up of the nutritional and health status of the pregnant and lactating women through screening method; they also teach the pregnant and lactating women about the time of feeding, type of food and amount of feeding and other activities, like educating about keeping of personal hygiene and sanitation, early marriage follow up; family planning and the like.

The water sector also works to have the nearby water access and supply of hygienic water for pregnant and lactating women; to produce home garden and irrigation based fruits and vegetables, the water source is the fundamental component that could work in collaboration with agriculture sector; therefore, the water diversion, check dam and any ground water is produced by the water sectors.

The education sector is engaged in the education of adolescent girls to improve their nutritional status and how those girls influence to their families by educating about the importance of nutrition.

The women affair and the civic society also encourage feed the balance diet foods especially during the pregnancy and lactation time. They follow the health and feeding status of the pregnant and lactating women in collaboration to the other sectors like health, agriculture, education and water.

**I:** How do you evaluate the level of collaboration among sectors in nutritional interventions?

**P:** We are evaluating the plan that we shared as the level of steering committee and the technical experts; but during our evaluation there are many weakness in their performance of all sectors, like less access of food source, diversified food, feeding style, beliefs, culture, distant and less water, awareness creation among the communities, less training of our experts and the like.

**I:** What kind of change in terms of the way stakeholders work together is needed?

**P:** In the water sector, we in collaboration with WASH project and our resource that tried to get the water access at nearby the community;

In the agriculture sector, we introduce the home garden vegetables and there are more than thirty thousand chickens introduced to the community.

The health sector also educates the community about the improvement of nutrition mainly to pregnant and lactating women; there is also a discussion program day of the pregnant and lactating women that could talk with the religious people: they meet every month with religious people so as to bring the behavioral change on pregnant and lactating women feeding style, health follow up, to ban under age marriage.

**I:** What type of resistance to the needed change do you perceive?

**P:** The commitment of all the technical experts and all stakeholders staring from the region, zone, woreda and Tabia level is not strongly engaged into the improvement of the nutritional status among the pregnant and lactating women. Less integration among the stakeholders and we more skewed to our main task; for example we the agriculture sector mainly engage in the growing of production and productivity of the agriculture products.

**I:** Is there any tool that your activity is evaluated?

**P:** Yes, we have common plan and take our shares among the sectors based on their specialty and at the end we evaluate the performance of our plan by the technical and steering committee. We know that most of the time, the activities done in group could not successfully achieved; because most of the time the group work assignment is taken as additional task for your main activities; thus you become skewed to your main task; this type of indication is observed in this committee. That is why I said attention should be given from the above Region level to the Tabia level, since the main problem is lack of consistent effort and sustainable monitoring and evaluation among the stakeholders. Therefore, stakeholders are the opportunities for our success on our interventions but still there are works to be done with them.

**I:** What opportunities do exist to promote multi-sectoral coordination of nutrition in this woreda?

**P:** One opportunity to run the improvement of nutritional status among the pregnant and lactating mothers is: the existence of high concern that is given by national and regional level and presence of mission to end the child stunting by 2030; the other opportunity is the integration among responsible stakeholders and awareness created among the community; presence of some supportive programs like SURE could help technical and material support; we produce diversified food types in the community; the program is led by leaders of the woreda and the region; there are committee established at the Tabia and woreda level; the extensiveness of irrigation among the community is increases; technologies, like best seeds and selective breeds of chicken are introduced to the community.

**I:** To what extent does your institution participate in the multi-sectoral nutrition coordinating body at this level?

**P:** All the agricultural production like introducing of nut, sesame, chicken, dairy, soil and water conservation and the like are our main tasks. But we didn’t focus to the pregnant and lactating women; we were giving training to the community.

**Sector 6: other interventions that influence adolescent and maternal nutrition and health outcomes**

**I:** Why delayed marriages (after 18 years) improve maternal nutrition?

**P:** Here the main effect is to the mothers; if she gives birth less than eighteen years she expose to different problems like fistula, there could be delivery problem, she doesn’t not give treatment to her child.

**I:** Why would increase the space between each birth improve maternal nutrition?

**P:** If there are many children in the households, there could be the problem of source limitation; there is also lack of enough breastfeeding time; the mother could not give concern to the all children to feed complementary foods on time; and the mother herself could not get time to prepare and feed balance diet foods.

**I:** What programs or activities promote increasing birth intervals in this level?

**P:** There is an assessment on the status of underage marriage; teachers are the actors in pursue the underage marriage; we have also committee from the health sector, the education and women affaire and agriculture that follow the underage marriage; last year we exclude about fifteen adolescent girls from the underage marriage; there are beliefs in the community that insist girls to marry for deacon even they are underage; they considered as if the girl is lucky being she is the wife of deacon and the deacons are pursuing to marry underage young girl; they thought that if the girl becomes eighteen and above she couldn’t stay virgin.

The law is very tight for underage marriage; the community already has aware about underage marriage; but if someone informs about some girl’s underage marriage, the family considers to the informant as enemy; this indicates that the community is not yet totally accepted underage marriage is as illegal and corrupt activity. But through the education sector, the information is easy to check whether underage marriage is conducted or not.

**I:** What are the community factors that affect age at first marriage?

**P:** The awareness among the community is not totally changed; still the community didn’t consider the underage marriage as taboo; still there is some resistance among the community; but the improvement is very promising.

**I:** What are the community factors that affect the birth spacing?

**P:** Still there exists lack of awareness among the community; the problem here is not only they have the interest of to get birth many children but there is no birth control utilization habit; there is also a fear of remaining unproductive (sterility) among the mothers.

**I:** Can you think of any opportunities to prevent early marriage and increasing birth spacing?

**P:** For early marriage, there is a rule that prevents from it; the steering committee which is led by the women affaire and they strictly follow it; the teachers in the school are also following the marriage status, they are responsible to know the reasons why a student is stoppage the school.

In case of birth spacing there are observations that give birth after they use the birth control; so the fear of remaining unproductive is answered.

**Additional remarks**

**P:** Although there is establishing the steering committee, we couldn’t say that the activity is run in a proper way; it is clear that we are expecting to work in nutrition improvement of pregnant and lactating mothers; and stakeholders are the opportunities for our success on our interventions but still there are works to be done. We are reporting that our production and productivity is increase; on the other side there is high prevalence of child stunting in the community which is controversial report; thus we should improve and change the name of high child stunting prevalence in our region.

There is high opportunity in the community to promote nutrition among the pregnant and lactating women, like the presence of diversified agricultural food production and the community is active enough to receive and implement the program at the ground; the government is also given great attention on the improvement of nutrition among the pregnant and lactating mothers. Therefore, we stakeholders should take this program as our main tasks like the other activities.

**SUMMARY**

**Section 1: Common maternal (pregnant women, lactating women and adolescent girls) nutrition problems in the community**

- Mainly the problem of malnutrition is high in pregnant women, because the child inside their body needs more food who is obtained from the mother; and pregnant women could not move from place to place for search of foods; relatively it is better in lactating mothers who prepare foods before lactation, like flours for porridge purpose, butter and it is slaughtered sheep or goat during the first lactation time.
- The main problem of malnutrition in pregnant and lactating women is the inaccessibility of the necessary food groups on time like vegetables, animal source foods cereals and pulses.
- The other problem is the poor feeding system of the mothers even the different food types are available at their home.
- Knowledge gap among the communities, like if the pregnant and lactating mothers have eaten only injera with shiro wot in ad libitum way, they considered as if they are feeding nutritious foods.
- There is a culture that mothers are prepare the quality foods like eggs, chicken, honey and butter to their husband rather than to eat themselves and provide to their children.
- There are households grouped in the rich community, and they could eat in ad libitum and fill their stomach with only one type of food but due to their poor feeding system still there are many under nutrition children from the rich.
- We supply more than night thousand quintal of sesame to the Ethiopian commodity exchange market every year, but we don’t consume the products for ourselves; this is due to the lack of awareness among the communities.

**Section 2: Nutrition priorities in the woreda**

- We strive to produce diversified food groups like cereals, pulses, tubers and animal source foods and we advise them to consume the pregnant and lactating mothers from the produced food groups.
- The pregnant and lactating mothers don’t participate during the soil and water conservation campaign; and in the food safety net program, the pregnant and lactating mothers get the service without involving the work.
- We are not only focusing on the production and productivity but we also make sure of what food types are more productive and nutritious to the pregnant and lactating mothers; how to harvest the crops without losing its nutritional content; like production of cereals (teff, sorghum, wheat), pulses (bean, chickpea and livestock products like honey, milk, butter etc.

**Section 3: Nutrition interventions that improve adolescent and maternal health**

- In collaboration with the health sector; it is provided training to the pregnant and lactating mothers like how improve their feeding system; and there is a demonstration site at the farmers training center to show how to produce home garden vegetables, chicken production and how to prepare foods without losing its content.
- All the pregnant and ten months of lactating women are not participating in the safety net program works. We also advice the husbands to fetch water using the back of donkeys so as to reduce the mothers workload; but they are still busy in the household activities.
- During the summer season every farmer plants green paper, tomato and swiss chard as home garden vegetables; they use water collecting groove during the summer season and it could serve as supplementary irrigation at the end of summer.
- We advise households how the cash crops could sell with high price and purchase diversified foods with fair price so as to consume and improve the nutritional status of the pregnant and lactating mothers.
- There is a biogas production which can be produced from the agricultural waste, manure, municipal waste, plant materials to be used as fuel; it can be used for any heating purposes, such as cooking; thus the waste materials are cleaned for the biogas and compost purpose; thus the burden of women from using fire wood, and preventing from the smokes of fire wood.
- It is rigorously monitored the pregnant and lactating women consumption level at their home so as to reduce the problem of sharing among the family households.
- Compared to the pregnancy time, lactating mothers are better preparing foods for their lactation period; as a culture here in the community, it is mandatory to prepare butter for lactating women; they also arrange teff flour or wheat but there is no any trend to prepare for their pregnancy time.

**Section 4: Implementation challenges and community factors affecting access to nutrition interventions**

- The area of focus for agriculture sector was to increase the agriculture production and productivity rather than nutrition improvement on the pregnant and lactating mothers.
- The gap of awareness on the nutrition improvement is not only in the community side but it is also the experts’ knowledge and commitment gap; what innovative technologies could we introduce, how could we convenience the participants to implement the training, how could we improve their feeding style; how the home grown agricultural products prepare and consume in balance diet way and the commitment of our experts to solve the gaps.
- Our experts didn’t get more training and experience sharing about the improvement of nutritional status among the pregnant and lactating mothers in the community.
- Tabias like Felegehiwot that affect by draught; pregnant and lactating women travel long distance to fetch water; it takes about two-three hours for round trips to fetch water.
- There is also a problem of rejecting the pipe water and they fetch water from the natural river due to the saltiness of water from the pipe which is not used to make local alcohol and other food types.
- Their feeding style is not changed and they feed the same type of foods at every feeding time; for example they have sesame, nut, other cereals and pulses and animal source foods like eggs, milk and honey; but they keep and eat only one type of foods and sell the other food products.
- During fasting days the pregnant and lactating women are not allowed to eat the non-fasting foods; they also stay fasting without consuming any food type until four-six hours, so the time of feeding is also affected.
- The presence of deep rooted beliefs, culture and norms like the fasting issue, engagement of pregnant and lactating women in high workloads, high respecting and providing quality foods to husband than pregnant and lactating women and the sprite of dependency mentality are another challenges
- If the husband doesn’t go to his home during lunch time, women could stay fasting the whole day.
- There is a statement that if a pregnant woman eats meat and fruits, she may have a big baby which endangers her life by making labor difficult.
- In the case of adolescent girls’ nutrition status, we didn’t do any activities and even now they are not our target groups.
- There less integration among the stakeholders and we more skewed to our main tasks rather than the common work of improving the nutritional status of pregnant and lactating mothers.
- Therefore, we should understand that mothers are the source of life; we all are from mothers; healthy mother creates healthy child thus to build the healthy citizen, we should invest on the health and nutritional status of mothers.

**Section 5: Multi-sectoral collaboration to improve maternal nutrition**

Integration

- We have a common plan among all stakeholders (Agriculture, health, education, women affairs and associations) and we are evaluating the plan that we shared as the level of steering committee and the technical experts
- The agriculture sector is striving to increase the production and productivity of the agricultural products
- Health sector follows up of the nutritional and health status of the pregnant and lactating women through screening method; and they teach about the time of feeding, type of food and amount of feeding and about keeping of personal hygiene and sanitation, early marriage follow up; family planning and the like.
- The water sector also works to have the nearby water access and supply of hygienic water for pregnant and lactating women.
- The education sector is engaged in the education of adolescent girls to improve their nutritional status and how those girls influence to their families by educating about the importance of nutrition.
- The women affair and the civic society also encourage feed the balance diet foods and prevent early marriage.
- There is a discussion among the pregnant and lactating women with religious people every month so as to bring the behavioral change on feeding style, health follow up, and prevention of underage marriage.
- Opportunities like the existence of high concern is given by national and regional level and presence of mission to end the child stunting by 2030; the integration among responsible stakeholders and awareness created among the community; presence of some supportive programs like SURE could help technical and material support; the extensiveness of irrigation among the community; introduction of technologies, like best seeds and selective breeds of chicken.

**Sector 6: other interventions that influence adolescent and maternal nutrition and health outcomes**

- If there are many children in the households, there could be source limitation; lack of enough breastfeeding time; the mother could not give concern to the all children to feed complementary foods on time; and the mother herself could not get time to prepare and feed balance diet foods.
- There are beliefs in the community that insist girls to marry for deacon even they are underage; they considered as if the girl is lucky being she is the wife of deacon and the deacons are pursuing to marry underage young girl; they thought that if the girl becomes eighteen and above she couldn’t stay virgin.
- The community is not yet totally accepted underage marriage is an illegal and corrupted activity.

**Additional remarks**

- It is very important to teach on how could to offer the available foods to the pregnant and lactating mothers; time of consumption, extra food during pregnancy and lactation; how the husband could reduce the mothers’ workload; and how feeding priority is given to them in the community.
- There is high opportunity in the community to promote nutrition among the pregnant and lactating women, like the presence of diversified agricultural food production and the community is active enough to receive and implement the program at the ground; the government is also given great attention on the improvement of nutrition among the pregnant and lactating mothers.
- But we know that most of the time, the activities done in group could not successfully achieved; because most of the time the group work assignment is taken as additional task for your main activities; thus you become skewed to your main task; thus strict follow up and commitment is mandatory among all stakeholders.
