# Supplementary material for: Barriers to utilize nutrition interventions among lactating women in rural communities of Tigray, northern Ethiopia: An exploratory study
Source: PLoS One. 2021 Apr 30;16(4):e0250696. doi: 10.1371/journal.pone.0250696 (PMC8087028; doi:10.1371/journal.pone.0250696)
Supplement: S2 File — (ZIP) [file pone.0250696.s002.zip › S2_File.Doc/Woreda level and above key informants/081_IDI_head for Women affair office_Thakua Abergele woreda.docx]

**Operational Research on Adolescent and Maternal Nutrition in Northern Ethiopia**

***Date: Nov 13, 2017***

**In-depth interview with Woreda Women Affairs Head, Tankua Abergele, Tigray**

**Section A: Interview Details**

1. Zone: Southern Eastern zone
2. Woreda: Tankua Abergele
3. Kebelle: Yechila
4. Name of key informant: Migbnesh
5. Institution of key informant: Woreda Tankua Abergele
6. Interviewer name: Mengistu Mitiku
7. Date of interview: Nov 13, 2017
8. Interview start time: 8:41 AM
9. Interview end time: 10:15 AM

**Section B: Interviewee professional information**

1. Gender: Female
2. Age: 32 years
3. Highest level of completed education: BSc holder
4. Current Job position: Woreda Women Affairs Head
5. How long have you been in the current position: 2 years

**Main interview**

**I:** Interviewer

**P:** Participant [The key informant]

**I:** Okay. Let us directly go to our conversation. First of all, let me start from my name. I am Mengistu Mitiku from Mekelle University. This is a big national research on maternal and adolescent nutrition, the services and barriers. It is being conducted in collaboration with Regional health bureau, Federal and UNICEF. Since your participation is very important, you can give any response to any of the questions that I ask you. Our conversation may take 1 and half an hour to 2 hours. If there is unclear question to you, you can trace me back and ask me. If there is a question that doesn’t go with you, you can tell me to skip it and then will be out of the record. So, if you have questions before I proceed, you are welcome.

**P:** It doesn’t have problem.

**I:** Thank you for your willingness to participate.

**Section 1: Common maternal nutrition problems**

**I:** Okay. When we go to the first issue, I will ask you questions related to maternal and adolescent girls’ nutrition. As per your thoughts, which nutrition related problems are common in your community or woreda? When I say mothers, it means pregnant women, lactating mothers and adolescent girls, i.e., those whose age ranges from 10-19 years. Let us start from pregnant women.

**P:** Okay. One of the nutrition related problems in pregnant women is anemia. They experience anemia. They face also body weakness. They lose their strength. Therefore, we note that it is due lack of adequate diet. We communicate with health office regarding malnutrition which is especially common in females, i.e., in pregnant women.

**I:** What about other problems related to nutrition?

**P:** Yes. Communicable diseases linked to nutrition, most of the time, problems like anemia, body weakness are the commonly seen diseases among pregnant women. There are no commonly seen communicable diseases linked to nutrition. What are commonly seen are the diseases I mentioned so far.

**I:** What about height and weight of pregnant women which is not consistent with their age?

**P:** The imbalance between height and weight with age starts from their childhood. It is common at this time. We didn’t still solve it. We are fighting against it. With regard to thinness, pregnant mothers become thin during their pregnancy period. Their weight also decreases. Even they are sometimes short. This is something that passes to and affects the baby. Therefore, our woreda has a problem associated with nutrition, like those I mentioned so far. Assessments indicate that the problem of thinness in our woreda is about 30%. We have evaluated it.

**I:** Alright. Still we are in pregnant women. What can you say about overweight? Is there such problem?

**P:** Yes. It is rarely seen in specific areas. This is due to lack of physical exercise and in some of the pregnant women due to taking rest. But, such kinds of women with overweight are rarely seen. We don’t have such kind of problem in numbers. Such kinds of women are here again. So, problems related to anemia and reductions in weight are the commonly seen ones in this woreda.

**I:** Is there a problem with regard to food insecurity in pregnant women?

**P:** What does food security mean?

**I:** Food security means, when there is enough food that helps lead life.

**P:** Yes, there is. Malnutrition starts from this, i.e., from lack of food items and in some pregnant women, the problem arises due to the unwise utilization of resources and not knowing the appropriate way of taking diets. So, food insecurity is also common here, especially in mothers. Mothers want to feed their children and husband, ignoring themselves. Therefore, mothers don’t credit their pregnancy and don’t convince themselves that they should eat. Such conditions are seen.

**I:** Let us continue and consider lactating mothers. Like those problems which you mentioned for pregnant women, what nutrition related problems do you are commonly seen in lactating mothers? You can share me in a detailed manner.

**P:** Those I problems I mentioned in pregnant women are also seen in lactating mothers. Weight loss and also anemia are commonly seen. Cardiac related problems like decreasing pulse rate are seen. Those diseases are observed in lactating mothers.

**I:** What about the common problems in adolescent girls? Again link the problems with nutrition.

**P:** There are problems in adolescent girls also. Problem occurs. In adolescent girls, there is no proportional growth of height and weight with age of adolescent girls. Girls are so thin and our woreda’s last year’s assessment has indicated us that about 30% of adolescents are thin. The problem is there.

**I:** What else? Especially food insecurity related issues.

P: There are improvements. Our woreda is being changed. The community is improving. Of course, we haven’t achieved up to the level we wanted to. There is shortage of food. In this woreda, we have Fafa and Biscuit in schools. Distribution those resources is in place, especially in those heavily affected by food insecurity.

**I:** Okay. Let us go to the next question. This, this, this…. diseases are mentioned as common ones in lactating & pregnant women and adolescent girls. To what extent do you understand the risk of females to the listed diseases?

**P:** Yes, they understand though some are unaware due to lack of understanding. They, however, know it when health education is given to them. The point, however, is that women’s fight against the problem the have is not strong. When are told that they have the problems, they care to a little bit. They even don’t adhere to the practices they know through health education. They don’t use the resources they get from health centers to manage their problems. When, for example, they get food items from health centers in the form of aid and support, they don’t use it as per the expectations. Therefore, I would say that pregnant women do have lack of understanding. It is clear for me that, let alone women are facing the problem, I really understand even they are normal that they have the risk.

**Section 2: Nutrition priorities in the woreda**

**I:** Good. Let us talk about the nutrition priority your woreda is doing. What nutrition related interventions are being done in your woreda on mothers and adolescent girls?

**P:** Health education has been given starting from woreda level to Kebelle and then to the network of women development army. Children who are screened and found to have malnutrition problem are given complementary foods like Fafa. The same is true to pregnant women. In adolescent girls, they usually get support in schools.

**I:** You told me that health education on nutrition related issues are being given. Where do these health educations given?

**P:** Yes. We have committee consisting of members from education, health, agriculture and water resources offices. There are minor and major committees. The training is given to committee members and committee members will go down and give the health education. Those members from education will give health education at schools and those from agriculture will give at kebelle and village levels. The same is true to health. By the way, a big focus is being given to the issue we are talking about. Every concerned body is being taken to woreda and trained which in turn have the responsibility to give training at each kebelle. Every representative from health, education, agriculture and water resources are taking their assignments.

**I:** Of all the priority interventions being executed in your woreda, which interventions are the most important and most resourceful ones?

**P:** The most important intervention is the awareness creation program we are implementing in our woreda. That is it. Awareness creation is the biggest and most important one.

**I:** As head of women’s affairs, do you think that your office’s involvement in women’s and adolescent girls’ nutrition is very important?

**P:** Yes, it is necessary. I can say to a big role.

**I:** Why?

**P:** Many of the problems are challenging our female sisters. To make aware and improve the health of mothers and adolescents, we have to take the lead. We have to initiate other organization also to promote our female sisters to a better health status. In all aspects, the participation of our office should be very important.

**I:** You are telling me that your office should get involved in improving the nutrition status of women and adolescent girls. Maybe, could you tell me if you have included the issue we are talking about, i.e., about nutrition, in your mission?

**P:** Yes, we include the issue in our mission always. When we include it, we focus on mothers that include pregnant women, lactating mothers and under 5 children. We include the need to delivery health education regarding balanced diet among mothers and children. Accordingly, we also conduct evaluation activities. The main task of our office is awareness creation among our mothers.

**I:** Maybe, I have one issue in my mind. Your office focuses on lactating and pregnant mothers. But, when I think deep, it seems that the issue of adolescents is being missed. As to the mind of health professionals, the issue of adolescents is being ignored and we healthcare professionals assume that adolescent girls are the ones who would be the sources of next generation. So, what is your plan with regard to adolescents?

**P:** We are yet to prepare a plan on adolescent girls. We don’t want to tell you that we have the plan though we don’t have. But, what we are thinking about is that if mother gets balanced diet, then she will give birth to a healthy baby. We understand that children should get balanced food until the second year of their life. So, it is such understanding we have and acting accordingly on mothers and children. However, we don’t have such plan in adolescent girls.

**I:** But, adolescent are the future mothers. What should we call them?

**P:** Yes. It is right. It could be improved in the future.

**I:** Among the priority interventions we were talking about, how would you evaluate them? For example, activities being done by your woreda and even your office?

**P:** I would say that most of the interventions have been successful; especially those which focused on awareness creation and behavioral change were successful because mothers have come to know what they didn’t know. Mothers’ behavioral change on the fact that when they feed their husband and children, they face problems for themselves. Their way of thinking is being changed, I would say. However, mothers are missing some important issue. When Fafa is distributed to those mothers with moderate malnutrition, those mothers who are pregnant and lactating with normal conditions start to complain in order to get the same food aid. Therefore, the big direction we need to proceed is to do some awareness creation activities, not to keep distributing complementary foods like Fafa.

**Section 3: Other nutrition intervention that improve maternal and adolescent health**

**I:** Let us go the third category of questions. Still, we are talking about lactating, pregnant mothers and adolescent. What other activities are in place to support mothers and adolescent girls? When we say priority nutrition interventions, we mean main activities that support mothers and adolescent nutrition. Now, I am asking other activities other than the priority interventions such the other routine activities?

**P:** The other interventions are not as such different from the priority nutrition interventions. Other activities such as giving health education to mothers to the extent of home to home visit are being done. I don’t mean that every household is educated. As part of the awareness creation activity, households are being monitored on the extent households know about the nutrition related activities. However, most of the activities are those I mentioned previously as priority interventions because we believe that if those interventions are executed, the other ones would be improved.

**I:** Do you know ANC? What activities that support mothers and adolescent girls’ nutrition are being done in ANC?

**P:** This is where our woreda has excelled. It is being improved from time to time. The use of family planning is good and mothers have a good condition of health centers in that they can know their health status at any time during their visit. We, therefore, would say that we achieving changes from time to time.

**I:** Okay. Let us continue. In ANC services, what looks like the advice given to mothers to use extra meal?

**P:** Right. Regarding this issue, all those in the leadership and those mothers who have a coordination role are being called for meetings so that they can bring down the activities down. Accordingly, mothers will be advised what and how to feed themselves during and after pregnancy.

**I:** Do mothers undergo screening?

**P:** Yes.

**I:** When is that?

**P:** Every month. When mothers come to health facilities for vaccination of their babies, they themselves will also undergo screening like getting weight measurement. This is especially true in lactating women. In pregnant women, health education is being given to at least undergo health status examination and they are improving from time to time.

**I:** Let us take, for example, weight measurement. Do mothers and adolescent girls go for this practically?

**P:** Yes. Especially in pregnant women and lactating mothers and children also. I don’t want to say this is going well in adolescent girls. I have a doubt in that adolescent girls are being missed. Maybe, adolescent girls get some support in their school, especially the distribution of Fafa.

**I:** What about the preparation and consumption of diversified food among lactating, pregnant women and adolescent girls? What interventions are in place?

**P:** This is especially done by health office. Health workers advise mothers how to prepare and utilize diversified food for themselves and their children. They practically do it after mothers gave birth. There is no report regarding this issue that indicates its status in every household. But, it is practically done at health facilities.

**I:** What about iodized salt utilization?

**P:** Yes. Iodine is one things shortage of which bring down a disease. Focus is given to such issue and is in practical run. Most community members ensure that they are using plastic bag of the salt for consumption. But, they are miss using it as most declared that the salt is added while the ‘wet’ in being warmed. It was a sort of big mistake. When the iodized salt is added to the boiling ‘wet’, it will evaporate, which is a big miss.

**I:** What about nutrition sensitive agriculture and safety net? What can you tell me about?

**P:** Backyard gardening is being practiced by households, especially in Yechila town since every household has adequate supply of tape water. Most household are utilizing vegetables like salad from their home gardening. However, the sunny and hot condition of our area is a problem. Mothers have a good understanding of the benefit of vegetables. They have understood its benefit from the change their reflected in their body.

**I:** What about safety net program?

**P:** Safety net’s main purpose is to support community members who are unable to lead their life. It is not fully support mothers and others. It is to support temporarily or until life transformation takes place. Anyway, safety net has a good benefit for mothers. There are mothers who changed their life using the support they get from safety net program. There are mothers who have chicken and goat & sheep farm as a result of safety net program. Thus, I would say mothers are becoming food secured due to the support they get from safety net program. These are the opinions I have.

**I:** There is one unclear issue to me. You said ‘individuals who are unable to lead their life.’’ Who are these?

**P:** These are those community members who don’t have food cereals or farm products.

**I:** Let us consider pregnant, lactating and adolescent girls.

**P:** Yes. It is right. The safety net program gives priority to motherless children and whether there she is pregnant or lactating, it is not fully clear. There are, for example, misunderstandings for pregnant women. The principle behind for selecting the target community members is based on the possession of resources for life like food cereals, cattle and others. It is not based on the fact that pregnant and lactating women need additional food intake. Therefore, I can say that there are unfair treatments here.

**I:** Lactating and pregnant mothers are really not benefiting from safety net program as per the explanation you gave me. By one side, we are not supporting mothers and by the other side we are saying that mothers are looking after their children and the baby inside their womb who are assumed to be part of the next generation. So, how do these two things go ?

**P:** Pregnant mothers do not go to safety net activity like water and soil conservation activity when their pregnancy reaches 6 months and 10 months after delivery. This is already incorporated in the norms of the program and is having a sort of follow up for it.

**I:** In the norm you have for safety net program, you have the mentioned the rules stated above. Where did the rule for pregnant mother come from?

**P:** The rule has originated from the non-governmental organizations themselves. Of course, making pregnant women work in safety net programs until 6 months of her pregnancy is hard for her. But, our office is making a follow up for that, i.e., pregnant women should not work when their pregnancy reaches 6 months. Even woreda administration office is making a follow up by considering the rule mentioned above, like the follow up our office is making. I cannot say that the rule is devised by regional government; it is devised by the charity organizations.

**I:** What about the services related to safe drink water and sanitation give to pregnant women, lactating mothers and adolescents?

**P:** In the plan of the woreda, it is clearly indicated that mothers should get safe drinking water from a nearby source and efforts are being exerted accordingly.

**I:** What about bed net distribution to mothers and adolescents?

**P:** Bed net distribution is available here in our town, but the means by which the community utilizes it is not proper. Every bed net distributed is not wisely used in the protection of mothers and children. There is a gap in the way our community members utilize it. The distribution focuses on mothers and children.

**I:** Why such misuse has taken place?

**P:** It is due to lack of understanding.

**I:** Why that lack of understanding has taken place?

**P**: Well. I can say that the awareness creation activity is not successful as there could be a gap of delivery of the awareness creation interventions.

**I:** The other issue is deworming. What thoughts do you have regarding this ~ providing mothers and adolescent girls with tablets for worms in their intestine, eye and others?

**P:** such activities are in place. For example, I had received intestinal worm related drugs last year, 2009 E.C. However, it was not successful because some of the community members don’t have trust on the drug given and didn’t come, some came through the drug was not utilized and some had rejected the request to swallow the drug. The mobilization process was not good enough. The same was true for trachoma. So, the community is not easily mobilized.

**I:** Are these interventions particularly focusing on mothers and adolescents?

**P:** It is for the whole community.

**I:** Okay. There is what we call it targeted complementary feeding strategy for the support of targeted individuals. What idea do you have about this targeted complementary feeding where mothers undergo screening and therefore complementary foods like Fafa are given to those who have moderate malnutrition?

**P:** There is such intervention also. Mothers will undergo weight measurement and if found to be below the standard with moderate malnutrition, she will be advised to consume Fafa and that Fafa will be given to her and same is true for children.

**I:** What about Vitamin A

**P:** Same. Mothers along with their children get this vitamin A every month and those mothers who are lactating will get even after birth. The provision also applies to children.

**I:** Okay. We have been talking about different nutrition related interventions that improve maternal and adolescent health. Among all these interventions you mentioned them so far, which do you think are the most important ones?

**P:** It is the awareness creation and behavioral change based intervention that could bring about a change. It is when the community accepted the intervention that change can take place. You can give them bed net. You can give the Fafa. If you don’t change the way the community thinks, it will be meaningless. Therefore, it is the behavior change strategy that could bring about a good success. There are some community members who took bed net to their home and throw it. This is due to the fact that their behavior is not changed.

**I:** Which intervention do you think was successful?

**P:** The better intervention I can say is the awareness creation activity being done. Mothers are improving from time to time in this regard. I cannot say the impact of Fafa distribution as it is only given for some of the mothers. It is, as to me, the activity related to awareness creation that could bring about a huge change. There are some mothers who come by themselves to health facility and undergo health service related examination. They came due the change in behavior that took place in them. There is a slight change in such interventions, anyway.

**I:** What can you share about this issue in relation to lactating mothers?

**P:** That is it. Similar with pregnant women, i.e., awareness creation activity.

**I:** Adolescents?

**P:** In adolescent girls, a successful intervention to improve the nutritional status is not in place.

**I:** What about the intervention that was not successful so far?

**P:** The intervention we consider as unsuccessful is the targeted complementary feeding practice and activity which is currently in place. For us, it didn’t bring any change. The change is that the distribution is resulting in complaints among community’s pregnant women. When one pregnant women gets Fafa, the other one complains to force Fafa distribution to them.

**Section 4: Implementation challenges and community factors affecting access to utilization of nutrition interventions**

**I:** What do you think are the challenges that are preventing us from executing the nutrition interventions mentioned so far?

**P:** Most of the time, the challenges are related the lack of awareness of mothers. The transportation problem is another where the target is very titanic and difficult to manage. The willingness of the community members to participate in the interventions is not pleasant. The lack of attention from the community members is also another challenge. The role of the geographical land scape is also a challenge as it makes transportation very difficult. The community’s attention paid to health education program is also a challenge.

**I:** Let us consider lack of education. Can we say that absence of educational level is a challenge?

**P:** Yes. It is a challenge. Those educated community pregnant women do come to follow any health education than those who don’t have. So, absence of educational is a challenge.

**I:** What can you say about community’s culture related challenges?

**P:** There are some challenges in the community that still need solution. For example, there are communities that don’t believe in delivering at health facility. If a women is taken to deliver at health facility, question like ‘how wife of a priest can deliver at a health facility’, ‘how the daughter of Mr X could give birth at health facility’ and so on. There are, anyway, such problems. With regard to salt utilization, some community members want to use the famous ‘Ganfur’ as this is a salt type the community has been utilizing in the last many years and is considered as a salt inherited from our ancestors. The other challenge is the fact that some fathers say that when a child is fed by his/her mother a delicious food, they say ‘how a child can know if he/she is eating a sweet food, vital food, it is the husband that should eat vital foods.’ There are many problems that are not yet solved and therefore are barriers to implementation of the different nutrition interventions that are intended to improve the health of the mothers and adolescent girls.

**I:** Can you tell me about the cost for transportation and other costs the community incurs?

**P:** Yes. The community doesn’t want to spend its money on transport. The community doesn’t think like ‘What is the cost of missing one’s life?’ Instead, they think about the cost of the transportation. If a mother is referred to a higher health facility, like in Mekelle, the issue is the price of the transportation and the cost of service. Due to such problems, delays are being made and finally lives are being complicated. In terms of supply, there is no problem in transportation. There is easy access to transportation to Mekelle at this time.

**I:** What about the interventions themselves?

**P:** The interventions cannot be challenges. Interventions are supportive not barriers. Interventions are in place to support mothers. Maybe the distribution of Fafa could bring a problem in as it results in divisions among mothers. The use of iodized salt has its own problems ~ the means by which mothers use it. Otherwise, the supply is good. Shops are communicated to bring the iodized salt at a continuous manner to the community.

**I:** What measures are taken to solve the challenges you mentioned so far?

**P:** The challenges have occurred due to lack of awareness of the community. They are not due to the conscious act of the community. Since we understand that the challenges are due to lack of understanding of the community, we are working on awareness creating activities, especially on maternal and child cases, the act of support in transportation either from the woreda itself or other supports is being practiced at this time.

**I:** What is the role of your office in solving the challenges mentioned so far?

**P:** One of the solutions we bring to mothers who are unable to go and pay by herself is to support her transportation by communicating with different offices which can help us in using their vehicles. That is it.

**Section 5: Multi-sectorial collaboration that improve maternal nutrition**

**I:** Do you think that collaborative activities are necessary to improve the nutrition related problems of mothers and adolescents?

**P:** Yes, it does have. If there is a collaboration among sectors, mothers will have a good understanding how cultivate vegetables through the assistance of agriculture, they will have good knowledge of nutrition and balanced diet through the guidance of the health team and we, as office of women affairs, help them in how to prepare food, how to feed their children and how to care themselves while they are pregnant and lactating mothers. Thus, supporting mothers in teamed manner would bring the mother a pleasant life.

**I:** How do you evaluate the Multi-sectorial collaboration that currently exists in supporting maternal nutrition?

**P:** I can say that it is good. The collaboration that exists among the different sectors is good. The committee is working with great effort to bring a change in maternal nutrition.

**I:** What should be done to further improve the multi-sectorial collaboration that exists currently?

**P**: Well. There is an agreement that sectors produce to work together. So, the committee should work together accordingly and evaluation should be made either every week or every month. That is the idea that I have. Of course, there is a gap in coming to the practical activities. Agreements are signed but the act of each committee to the real activity does have a gap. If all these gaps are refined, improvements can come.

**I:** Who is the coordinating body of your collaboration in nutrition related interventions?

**P:** The health office. If it is nutrition, it is the health office.

**I:** In addition to the leading role of health in nutrition interventions, what is the role of your office, women’s affairs?

**P:** As I told you at the very beginning, our plan includes maternal and child health. Though our office doesn’t take the lead like the health office, as a member of the steering committee, we have a big role in sharing our efforts. The committee refines the following: Are mothers getting health education, do mothers give attention to the health education given to them? Are women development armies supporting you? All these are refined in the steering committee; even we approach them in person.

**I:** To what extent are committee members effective in doing their activities?

**P:** There could be gaps due to time constraints, but they are doing in a responsible manner. So, I would say that they are doing their jobs in good way. As I told you, there may be gaps in terms of the time they have so that that could be difficult to them to sit together and evaluate their roles. Due to this, I would say the gap is due to time constraint. In addition to this, the committee has a gap in planning and acting according to the plan.

**I:** Considering their gaps, what sort of support do you think they need?

**P:** The big support is that every committee should have the feeling of ‘it is my business.’ It should not be the health which leads us, it should not be the education which should call us for a meeting. We all should work synergically. What are the problems, what should be done…should be the agenda of all sectors. We should not expect and wait until the health office calls us all for meeting. This is the idea I have.

**I:** What are the opportunities that could enable the multi-sectorial collaboration to succeed?

**P:** The main thing is that everyone should have sense of ownership. There is nothing above this. The other opportunity is that there is a community that welcomes you easily when you approach him very wisely and appropriately.

**I:** Let us go to the next question. There are other interventions that support maternal and adolescent health. These are birth spacing and marriage when age passes 18 years. In your opinion, what do you think is the essence of marriage of adolescent girls later than 18 years?

**P:** It gives time for adolescent girls to mature. If girls get married under aged, that will bring her a big problem as she may encounter pregnancy and finally labour. It is a problem a lady who doesn’t have matured body and mind. Whereas if the girl is well above 18 years, she will not face such challenges as she is well matured physically. Anyway, if the adolescent girl have a good habit of food intake, that could also harm her. There are, for example, girls who don’t easily estimate their age due to nutrition effect. Therefore, if she is above 18 years and has been in a good nutrition situation, she may not face challenges when she became pregnant and during birth. This is the idea that I have.

**I:** What effect will it has when there is long birth spacing?

**P:** When a mother gives birth, she may have the chance to face bleeding. If this bleeding occurs repeatedly due to repeated birth, the mother will deteriorate physically. Whereas if a mother gives birth at three or four years interval, the effect of bleeding that occurred will disappear and when the next birth comes, she will not be face physical harm. If she gives birth at a year or two years interval, she will be in jeopardy.

**I:** What interventions are being done in the promotion of birth spacing?

**P:** There are non-governmental organizations like UNICF and others which are working on different activities like supplying contraceptives. The health office knows this very well. When we come to marriage related issues, there is one organization that helps us a lot where the community is empowered to organize a discussion forum so that discussions will be used as a means of conveying messages to the community.

**I:** What are the specific activities being conducted with regard to preventing under marriage?

**P:** Every year, starting from September, we conduct surveys in kebelles together with our partners from the kebelles selected for survey. We will check if there are adolescent married under aged. The partners are police of the kebelle and representatives from farmers of the kebelle. The partners study all the necessary and bring us all the information. Then, the whole team will go house to house and discuss with those who have a plan to undergo marriage. Every point will be discussed. However, if the discussion is not successful, interference at woreda level will take place. Responsible bodies, including our office and police office will reach at the kebelle and efforts will be made to solve the problem. In the discussion, community members and household members will be oriented about the problem of underage marriage and this will be supported by examples; those that encountered fistula will be taken as case in point and things will be discussed accordingly. So, the issue of underage marriage is always a focus point of our office and improvements are being achieved from time to time, I can say. Police office is working hard in this regard. The same is true to justice office; they struggle a lot.

**I:** What about the impact of religion? Can religion be used as a means to prevent early marriage or under age marriage?

**P:** Yes. Marriage has a big influence on marriage. Even the organizations that support us have indicated us to consider religion as big factors. When the issue of under marriage comes, committee will established considering representatives from religious leaders, elders from kebelle and they have the task to orient the community on the need to prevent early marriage.

**I:**  Well. You have mentioned some activities being done to prevent underage marriage. To what extent are the activities effective?

**P:** For me, I can say the activities in place are effective as changes are observed from time to time. When we consider underage marriage, there was any woreda which have a high proportion of underage marriage. This woreda was the leading one in terms of marriage. However, this woreda has improved significantly. Let alone adolescent girls, those who are well matured ones have developed the system of bringing the idea of ‘we need to be screened’ before marriage. There are three kebelles which declared that marriage is impossible without proper screening and age assessment. What these kebelles do is that they bring the couples to be marriage and undergo age and other things assessment. In this regard, we are being improved. These are the kebelles which are doing such activities by their own interest. The other kebelles are also doing similar things. However, interfering of our office and police is needed. There are instances when we as office go to the kebelle and bring the girls who are being forced to get married.

**I:** What do you think are the community factors associated with underage marriage?

**P:** The community has high desire to undergo marriage. Some families are being tensioned by the words of their neighbors. Even elders of community are very influential. Elders say ‘I want to see the son of my daughter, the daughter of my son’, etc. Well, this has a big impact on families. The other issue is that family leaders suspect that if adolescent girl is matured, she will start friendship. There are such instances. Especially when the adolescent girl is matured and is in school life, the degree of suspecting becomes high. When they go to school, they don’t hope that they will come back. They, therefore, rush to get their adolescent married. All these are taking place due to the fact that the community is illiterate. Families believe that if girls continued education and reached somewhere at higher level, they will be coupled with the person they don’t need. So, they think that terminating their school life is a big option to achieve their interest.

**I:** Let us come to the opportunities for birth spacing and marriage above 18 years. What do you think?

**P:** The law in place is one. If a family undergoes marriage, the family will get punished. The already established system of preventing underage marriage is another opportunity: the surveys done and the health educations given. The other is the strong union of women in kebelles. This is a good opportunity. When I consider birth spacing, the availability of contraceptives and the health education given to mothers at health facilities is a good opportunity.

**I:** Fine. At the end, what have you learned from what we have been discussed, i.e., the nutrition related activities.

**P:** We, the office of women affairs, have been working in mothers and children; I have learnt that our office is ignoring adolescents. But, when adolescents who are regarded as future mothers are not benefited from nutrition intervention, they will be harmed. This is a big lesson I have learnt. Also, I understood that if there exists multi-sectorial collaboration and close evaluation, then a big change will take place.

**I:** What opportunities does your woreda have to improve the nutritional status and therefore the health of mothers and adolescents? Just in general ~ the opportunities?

**P:** The opportunities of this woreda are, this woreda is famous for its cattle and honey resources. So, this woreda does have a big resource though the way we utilize them is poor. The main food cereals are those found in this woreda. For example: Fatty food cereals, protein like foods and vitamins are found here in large amount. Therefore, if the community gets a good understanding of the resources, then the resources can be very useful ones in reducing malnutrition to a lower level. This is the idea that I have.

**I:** Good. Thank you very much. I have finalized my stay with you. Thank you for the ideas you shared me.

**P:** Okay.

**Summary**

- Anemia, along with thinness, are the repeatedly mention nutrition related diseases which are commonly seen in mothers.
- Though there are many interventions being done, awareness creating activities are the main activities being done in the woreda to improve maternal nutrition.
- The nutrition od adolescent girls is a missed issue even in the woreda office of women affairs as it is not included in the mission of women affairs office.
- The most successful nutrition related intervention is the one that focus on awareness creation and behavioral change activities as it has brought a big change in the community.
- High illiteracy condition and therefore lack of understanding of community is the biggest barriers to the implementation of nutrition intervention for mothers and adolescents.
- Religion and community elders are big factors influencing marriage and birth spacing.

------ The end -----

**Summary**

- The principle ‘any mother should not die while giving life’ is among the best motivating factor for mothers as repeatedly mentioned by key informant.
- Malaria is the disease that hugely affects mothers in this kebelle as the atmospheric condition is hot and convenient for the occurrence of the disease.
- Interruption of complementary foods has hindered the fight against malnutrition in mothers.
- Key informant has constantly indicated that birth spacing has a significant effect on the mother and finally on the mental development of the child as well.
- Community mobilizations which support communications regard maternal nutrition are not as such happening in the community.

The end
