# Supplementary material for: Barriers to utilize nutrition interventions among lactating women in rural communities of Tigray, northern Ethiopia: An exploratory study
Source: PLoS One. 2021 Apr 30;16(4):e0250696. doi: 10.1371/journal.pone.0250696 (PMC8087028; doi:10.1371/journal.pone.0250696)
Supplement: S2 File — (ZIP) [file pone.0250696.s002.zip › S2_File.Doc/Woreda level and above key informants/082_IDI_Head of Woreda education office_Tankua Abergele woreda.docx]

**Operational Research on Adolescent and Maternal Nutrition in Northern Ethiopia**

## **Tool A**

## **IN-DEPTH INTERVIEW GUIDE,**

## **Woreda education office**

Hello, my name is Omer Seid. I am from Mekelle University. Thank you for taking the time to speak with me today. We are doing research on the factors that influence the nutrition of mothers and adolescents in collaboration with tahe Regional Health Bureau and UNICEF. Your participation is very valuable. The things that you tell us will be used to improve nutrition programs and services for women in the region and the country. We will not share your names when we report our results.

Do you agree to participate in this study? YES

| **Section A: Interview details** | |
| --- | --- |
| **Questions** | **Answer** |
| Zone | Central Tigray |
| Woreda | Tanqua abergele |
| Kebele | Yechila city |
| Name of key informant | Kidanemarriam W/michel |
| Institution of key informant | Tanqua abergele,Woreda education office |
| Interviewer name | Omer Seid |
| Date of interview | 13,November 2017 |
| Interview start time | 12:25 AM |
| Interview end time: | 1:58 PM |

| **Section B: Interviewee professional information** | |
| --- | --- |
| **Questions** | **Answer** |
| Gender | Male |
| Age in year | 30 years |
| Highest level of completed education. | Bachelor degree in midwifery |
| Current job/position | Woreda education office head |
| How long have you been in the current job/position: | 1 year |

**Section 1; Common maternal (pregnant women, lactating women and adolescent girls) nutrition problems in the community**

I: What are the common nutrition problems in the community for women and adolescent girls?

P: It is speak from my prior knowledge because I don’t have research based information about it. From my knowledge there is thinness, because of malnutrition or shortage of food this will be happened in their body, means if you observed them physically, have peel face which indicated that they are malnutrition. You can see this peel face character among children’s and pregnant mothers of our woreda. I know also, there are children’s, pregnant mother and women’s that have low in weight.

I: Do these problems also observed among lactating and adolescent girls?

P: ya, in this area one of the problem is early marriage, thus it is definite that this adolescent girls will give delivery/ birth below 18 years. As result the new born baby will not get sufficient milk, the girl herself will not get sufficient care and her child will not get sufficient care also and this condition will associated with malnutrition’s.

I: I will come later on about early marriages, but now I need clarification, what do mean when you say the new born baby will not get sufficient milk if the mother is below 18 years? Why?

P: It is not about milk, what I mean is there are women’s that has given deliver below 18 years. Due to poor awareness and by family and cultural pressure adolescent girls will get married and then will give births in early ages before they got matured. As a result this adolescent mother will be affected by malnutrition and her children too affected by malnutrition because if the adolescent mother is malnourished she will not produce enough milk to feed her child. Therefore this is my point that I mentioned above.

I: What about nutritional problems of adolescent girls?

P: In this woreda, there is high magnitude of fistula, and early marriage is the cause for fistula.

I: What about micronutrient deficiencies (such as anemia, night blindness, goiter)?

P: There is anemia; right now I don’t have the researched evidence that the health office do have regarding it. As of my information anemia is a problem of adolescent girls and women’s of this woreda community, but you can get further information from the woreda health office. About eye problem (night blindness) which is locally known as *hemia,* even though I don’t know the exact magnitude or percent, it is problem of the area womens.

I: Does all community segments do equally affected by night blindness?
P: If you go to out of this city (yechila), night blindness has observed rural residents or communities. Last time I was participated in a meeting, and the health office has reported night blinds is problem of the area, but right now I don’t remember its exact magnitude or percent. Anyways I heard during that meeting and from my subjective assessment in addition, night blindness is problem of this area.

I: What about goiter? Is it the problem of mother of this area?

P: Sure, there is mother that have goiter which is because of iodine problem

I: Why mother of this area are anemic, vitamin A deficient and has goiter?

P: The problem of iodine is mainly due to shortage food intake rich in iodine. If the community would consume enough iodine, iodine deficiency would be eliminated in the area. Poor awareness is the cause for this low consumption of iodine.

I: Why mother are anemic?

P: Anima is due to shortage of food intake. Vitamin A problem could also occur by inadequate dietary intake. Eg: Inadequate dietary intake of vitamin A causes night blindness, which is because of low awareness of the community. The community didn’t know the food items that have preventing night blindness. Goiter, having swellings body that is located on the anterior neck, is a problem of this area. Further information you can get from the health office of the woreda about it.

I: Ya sure, the health office my give in details and I need to assure you that, the health office is also the target participants of this study, but here I wish to know from you?

P: Oky, I am head of education office of Yechild woreda, I now schooling students that have shortage in nutrition. There is nutrition program for these students in the school by the support of WFP in which it is given oil and FAFA for schools. In these woreda 23 schools have school feeding program by the support of WFP and, in these schools porridge cooked and given for students. There are cookers for the porridge, and each school has covers the expense of cookers.

I: Are there stunting, wasted women’s and adolescent girls in this woreda?

P: The problem is I don’t have research based information regarding to you question, but if you assess subjectively you can see short and thin adolescent girls which is because of malnutrition. Because of not taking their food or breakfast you may see students that have sleep during class teachings if you are their teacher.

I: Does diet related non communicable diseases, DM and Hypertension, are problems for pregnant, lactating and adolescents of this area?

P: There are peoples that are suffered from DM and hypertension, but I don’t have Idea whither these diseases has relation with nutrition or not.

I: Who are these patients? Are they pregnant and lactating women’s or adolescent girls?

P: Yes, there are pregnant and lactating women’s and adolescent girls that are suffered from DM and hypertension. But I don’t know the cause of this none communicable diseases

I: What about food insecurity situation of pregnant and lactating women’s and adolescent girls?

P: Someone will be thin or fat which is because of nutrition or genetic related factors. For example; the person will be thin but if we see the dietary intake it is balanced in this case the problem is genetic, likely the person will be fat but if we see the dietary intake it is will not balance. Bing thin or fat is not enough indicter for nutrition shortages, but it is simple to identify malnourished students physically, they have greenish eye color. So, thinness or shortness is not only due to malnutrition because it has several factors like growth hormones.

I: What about overweight?

P: Overweight is not the problem of pregnant, lactating and adolescent girls of this area, rather anima is a problem which is because of poor dietary intake.

I: What about food insecurity situation of pregnant and lactating women’s and adolescent girls?

P: Still the community has supported by the safety nut program to get foods, this means if there is enough food the community will not be supported by the safety nut program. Both the rural and urban community of the area has food support from the safety nut program. Thus, the area is food insecured area.

I: When this food insecurity happened? Is that always?

P: It is not always and has occurred during drought seasons. If there is shortage of rain, there will not be enough agricultural production, so the community received food support from the productive safety nut programs. eg; in 2007 EC there was severe drought in this area so there was a food shortage for humans and animals which was because of shortage of rain. But still the community is food insured even with having good rain.

I: If there is good rain in the area why they are food insecure?

P: It is because of illin

I: my question is if there is good rain in the summer why the community is food insecure?

P: The area is temperate and the area is moisture shortage area and water holding capacity of the area is poor. Thus the area needs frequent regular rain for good production, if not, no good production that is the reason.

I: Which women groups are most affected by these nutrition problems?

P: In my opinion mother are anemic if are not get enough food and milk.

I: Who will be more affected by anemia female or males?

P: Females

I: Why?

P: I will compare the magnitude of anemia among pregnant Vs males. If you compare a husband and wife in one household, the wife (mother) will be more affected than the husband by anemia especially it is true during pregnancy. Thus, in my opinion if the woman is pregnant her child in the uterus takes nutrition from her, and if she is not taking adequate food to cover her and her baby need she will be anemic.

I: What about the lactating women?

P: I don’t know

I: Who will be more affected, lactating mother or males by malnutrition?

P: I told you, females are more affected than males, and out of female’s pregnant mother are more risk groups for anemia.

I: who will be more affected by other malnutrition like thins shortness or vitamin A deficiency?

P: The same, pregnant mother will affected more, if there is shortage of food there is leg edema.

**Section 2; Nutrition priorities in the woreda**

I: Do you think it is necessary for your institution to get involved in work aimed at improving maternal nutrition? Explore for pregnant women, lactating women and adolescent girls.

P: it is about nutrition?

I: yes

P: Yes definitely. We can work with social affairs office on nutrition issues, because this office supports children and women. We can work with health office of the woreda to teach the students about feeding practice and sanitation and hygiene related activities. By working with these sectors we can do awareness creation activities and then the nutrition problem will not occur in this area.

I: How can your institution at this level be involved giving examples from specific projects or work?

P: Example, with social affair office, our role will be identifying and giving malnutrition student from schools, then the social affair office will do support for this students, it could be food or other in kind materials .

I: What about another role?

P: With health office we can work on reproductive health’s, on the prevention of early marriage, HIV/AIDS and nutrition for pregnant mother. There is local NGO called OSSHD and this program has the check list forms, by using the forms we do identification of anaemic girls from schools then linking them to the health facility for treatments.

I: What maternal nutrition (pregnant, lactating and adolescent girls) interventions are the priorities in this woreda?

P: Our task is teaching the students using the curriculum, so we don’t have nutrition intervention programs for pregnant, lactating and adolescent girls. No intervention that we have doing beyond teaching students. In the school feeding program our role is identifying schools for the school feeding program, and WFP supported us all the necessary food items for the feeding program. There is a nutrition topic in the school curriculum, so we do teach the students accordingly, but we don’t have nutrition intervention in the community for pregnant and lactating mothers.

I: Why you are not intervening for pregnant and lactating mother, and adolescent girls?

P: It is clear, if the pregnant mother is not getting enough food, she will not give health baby. This is what I can say here

I: So do mean you don’t have any nutrtion intervention for pregnant and lactating mother, and adolescent girls?

P: I am telling you that we don’t have interventions for the community but nutrition is incorporated in the school books so teachers do teach students about nutrition in the school.

I: What exactly you do for adolescent girls as a wereda education?

P: What we did is identifying anemic adolescent girls then linking then to the health facility for treatment. But we don’t have intervention for pregnant and lactating mothers

**I:** In your opinion, which of the above programs are being implemented successfully (i.e. in the most effective way?) Why? Explore for pregnant women, lactating women and adolescent girls.

P: Relating to nutrition we don’t have specific intervention beyond talk. In the school we are not doing nutrition intervention for pregnant and lactating mothers. As I told you we do intervening on identification of anemic students then linking them to the health facility for treatments, but even we don’t know the treatment that has given for these students by the health facilities

**Section 3; Nutrition interventions that improve adolescent and maternal health**

I: kinds of nutrition interventions are in place to improve adolescent and maternal health in this woreda?

**P:** Beyond talk about the idea we don’t have nutrition intervention for lactating and pregnant mothers. In the school what we did is identifying anemic adolescent girls and linking them to the health facility for treatments. For adolescent girls we do this in the schools and it has social and economic importance. eg; if we get pregnant adolescent girl in our school we do link her to the health facility for ANC follow ups. Thus, I don’t believe we did successful nutrition intervention because we don’t have nutrition intervention programs. Our primary work is keeping the quality of educations. Relatively schools have better opportunity than the woreda education office for nutrition interventions.

I: Right you are and I think your office can coordinate the nutrition activity of schools, am I right?

P: yes we are coordinating the teaching and learning activity of the wored, and i am speaking you that our woreda education office has doing collecting reports from schools then reported to the next responsible bodies gain. But we don’t have nutrition intervention programs to identifying the nutritional problems in the schools and then doing interventions accordingly to tackle the malnutrition problem.

I: What is that, I think as you told me above there are some activities for school adolescent girls?

P: Yes, I already told you. For adolescent’s girls especially in the high schools, what we do is identifying adolescent pregnant women and anemic adolescent girls and linking them to the health facility for treatment.

I: How do you know anemic adolescent girls?

P: It is subjective if we suspect anemia by observing the girl physically, and her eye has green like color, then we do link this girl to the health facility.

I: Why not doing nutrition advices for adolescent girls?

P: ya, it is already doing in the schools. There is reproductive health club in the schools and this club has doing activities on the prevention of early marriage, awareness creation about menstruation and dietary practices of pregnant mother and many other things it has discussed to improve the health of adolescent girls. The health bureau has supporting the club by different materials like leaflets which the club used to teach the school students.

I: May be, could the club work on promotion of iodized salt utilization?

P: In my thought around 90 percent of the community knows I iodized and majority consumes it currently. Thus, Iodine promotion has been done by the health and by other sectors so there is an improvement on its utilization but still some households of the ommunity has using none iodized salt. There is none iodized salt locally known as “*ganfur”* which comes from Afar region in the summer season for animal consumption but still some households use this unionized salts.

I: Is productive safety net program for women’s and adolescent girls?

P: Yes it is given for food insecure households. Food insecurd households have been identified and given the support. Previously the support was food items, but now it is changed to money, so they have monthly payment. The agriculture office has doing this the productive safety nut supports.

I: What about, on nutrition sensitive agriculture such as home gardening, productive safety net program, food security?

P: In the woreda all these activities has been done by the agriculture sector

I: Do you have joint work with the agriculture sector?

P: We do jointly on water and soil conservation interventions. Jointly with the agriculture office we do water and soil conservation activities by participation of school students.

I: Do you have intervention for advice on water, sanitation and hygiene services for school adolescents?

P: Yes it is doing in the schools. Eg; there is weekly sanitation campaign program in the school, so students do clean their schools.

I: Is it only in the school or do you have such kind campaign program in the community?

P: The school reproductive health club has outreach activities in the community. The club has going into the community and teach about reproductive health, sexually transmitted diseases and do also distribution of condoms.

I: At this point, in the outreach program does the reproductive health club have activities for pregnant and lactating mother?

P: No. look the aim of schools is different from your study aim which is pregnant mother. As the education sector we are doing on keeping quality of educations, preventing student dropout from schooling, prevention of early marriage, advising delaying of pregnancy. So we don’t have outreach nutrition activity in the community. In the high schools, pills has given for adolescent girls to prevent unwanted pregnancy and modes for menstruation care which is supported by the local NGO OSSCH. There is emergency contraceptive which will be given for the adolescent girl to prevent unwanted pregnancy, if she did unsafe sex. By the support of the dignity project which is from Ayder referral hospital, punt has given for adolescent girls, but it is done before in a year ago, but no this support currently.

I: In your opinion, which of the above programs are being implemented successfully (i.e. in the most effective way?) Why? **Explore for pregnant women, lactating women and adolescent girls.**

I: The reproductive health club has doing good jobs on education of students and the community through dramas and role plays. No female student will absent from schooling during mensuration time, this is because we did good job through using the reproductive health club on awareness creation for both male and female students about menstruation. In the high schools there is a prepared private room for adolescent girls for changing their modus, have water and sops for washing during menstruation. There are mentors and training has given for these mentors in every year by OSSCH, and then mentor has doing on teaching the students on menstruation, sexual healths, and also doing on the prevention of HIV. More this mentors have doing on reproductive health intervention, not doing on nutrition’s and they do only education/promotions.

I: What are the implementation challenges that are specific to delivering the maternal nutrition interventions in the programs interims of you school? Explore adolescent girls.

P: About pregnancy ANC follow up eg; there is slogan “no women should give at home” this slogan works in every sectors. Thus, if we teach scool students about it, they will go and teach their mother at home to give birth in the health facility. This area community is not educated and mother too has none educated. There is a readymade ambulance for transportation, so woman can use this ambulance for transportation to the health facility while giving birth.

I: Dose lack of awareness on nutrition related problems affects the service utilizations?

P: I am clear with your question. For ANC follow up no cultural challenge that prevents mother form NAC follow-ups. But, some adolescent girls refused to take the immunization that has given in the school. In this situation what we did is gathering and teaching adolescent girl’s about the importance of immunization that will be given. They fear the pain that will happen due to the immunization injection. This is due to poor awareness, but as far as I know I do not know any cultural challenges that prevents mothers and adolescents girls to utilized services. There is no, it could be religion related or social related challenges that prevents the women to utilizes services.

**Section 4: community factors affecting access to maternal nutrition interventions**

I: Can you think of barriers that prevent adolescents and women from using the programs and interventions that we have discussed? **Explore for adolescent girls, pregnant women and lactating women.**

**I:** Does educational level of mother is barrier for the service utilization?

P: Education has big influence

I why?

P: Educated mother has enough understanding for the importance of ANC and PNC follow-ups and she knows appropriate foods for her consumption and has known also appropriate feeding style of her children. Thus, the educated and the non-educated mother have no equal understandings for nutrition and other services. The educated mother has given better care for their children and has given better care for her own health, but relatively none educated mother are ignorant for their children and for their own health also. Even, none educated mother will not use the homemade available foods appropriately because of low education level.

I: What about transport access?

P: There is ambulance but there are around three or four keblle that don’t have transport access.

I: What community related beliefs and norms are preventing access to the interventions? How?

P: About this, it is impossible to speak simply, eg; Me myself has bad experience, when I was a student I take a medicine which was given in our school for the prevention of eye disease (*tracuma)* unfortunately some of us feel nauseated and vomiting, and up to severe sickness in some students had, but there was also some healthy students. At that time the community hypostasized many things about the medicine we took; the medicine is bad for health and it is given for blinding student’s eye or/and the community hypostasized many other things about it. So there may be such kind of questions. The same spoken when I was school director there is red tablet which was given for adolescent girls and some students using properly but other don’t use because they perceived it is pills for pregnancy prevention. Compare to the previous five or ten years ago, the current mother’s awareness is good for health services so no such kind perceptions nowadays. If you go to the health center you can get the exact figure about home delivery, as aworeda home delivery is almost getting into zero.

I: Is it a true data?

P:There is health package in each kebelle, the health package health worker knows each pregnant mother of the kebelle, each pregnant mother has doing follow-ups and the expected date of delivery of each mother is known then the health worker of the kebelle brings mother by foot in ahead of deliver or by using ambulance to health facilities for giving delivery.

I: How do you explain the quality of the interventions?

P: I heard rumors regarding to the quality of the interventions. The community complaining that health care provider’s is not ethical, has insulting us and has nagging us. During our meeting with community this issue clearly raised by the community even they complaining by specifically mentioning the name of none ethical health care provider. So this unethical character of the health care provider is barrier for the service utilization.

I: What resources exist to provide the interventions and what do not exist?

P: The community is not getting enough services here; mainly do referring to Abi adi and Mekelle hospital. Surgical management has not given here, and another thing the community doesn’t have truest if the surgical management is given here because the hospital here is new. No fistula treatment here, so these cases has referred to Mekelle. There is also shortage of medication and other.

I: For these challenges that you mentioned, can you tell me of any solutions that your institution have applied to effectively implement the interventions for women and adolescent girls? Specify the each solution done for each challenges?

P: There was shortage of ambulances but now it is solved by the government. There are three main woredas in this area and has found three health centers and three ambulances in the area, which mean one health center and ambulance has found in each woredas.

I: Is there any solution that has made to solve health care provider’s ethics?

P: Regarding to ethics, the woreda has done evaluation of the health care givers, accordingly the correction measures has done up to firing of unethical health care providers.

Section5: **Other interventions that influence adolescent and maternal nutrition and health outcomes**

I: In your opinion, why would delayed marriage (after 18 years) improve maternal nutrition and hence both maternal and infant health?

P: Early marriage is bad, not good

I: why?

P: Early marriage has psychological and social problem. Fistula is the one which will occur due to early marriage; this case has found on students and also has found in the community. Females are not matured for pregnancy, if they get pregnancy related to early married they will develop fistula. Early marriage has several consequences, like leave from schoolings which is one of our headaches also. If we bringing this to nutrition, even they may have enough nutrition but there will be still early marriage. There is a problem, students knows the consequence of early marriage but still they have doing it.

I: why?

P: We are not changed, no behavioral change. For example, there are HIV positive students, they know how to prevent HIV but not preventing it which is because of behavioral change problem or they are not changing their behavior to prevent HIV. Knowing condom is not enough we should use it. When I come to nutrition, if one female is not matured nutritionally she will not ready for sex. Last time I heard from a study which was presented by heath sector, in this area, 76 % female’s in the rage age of 12-18 year has started sex, but in the same age range around 25 /26 % male has started sex. Therefore if we relate this in to nutrition, if you are well feed and well-nourished your body will be matured then you are ready for sex, whereas if you are not well nourished and thin you will not have sexual desire.

I: In your opinion, why would increasing the space between each birth improve maternal nutrition and hence both maternal and infant health?

P: The important is known. In our area increasing birth interval means giving birth in every 3 years intervals. This 3 year is according to our community but if you ask me the exact or the recommended year of birth interval, I don’t have response. If you asked me the importance of increasing birth interval; one; mother will be healthy, their body will recovers and will rebuild and their children can get time for adequate breast feeding and care. If there is short interval the child will not get enough time for breast feeding and care and the mother will be thin. Therefore increasing birth interval is essential for having healthy family.

I: Can you tell me about any programs or policies in place in this woreda to prevent early marriage?

P: yes there is. Nowadays early marriage is illegal and has strong law that will be asked by court. Especially it is very strong in the school if one student plans to have early marriage it will be stopped, because in the school we know the age of each student. To prevent early marriage we have been doing strong activities jointly with women affairs. Automatically the women affair will do action if we reported early marriages cases. The community knows the consequence of early marriage and, much information has been given for religious leaders and have know too much, but the problem is on the behavioral change. In the area sometimes there are early marriages under the cover of both by the community and religious leaders. So, awareness creation activity should be done to change the behavior of the community on early marriage.

I: What programs or activities promote increased birth intervals in this woreda?

P: There are family planning programs; there are different contraceptive methods currently. In each kebelle health extension works do home visiting to distribute contraceptives. There are enough health services for married women’s.

I: In your opinion, are these programs or policies effective? Why or why not?

P: In my opinion, early marriage is significantly improved; it is occurred very rare in rural areas. So it is effective. Because of economical reason and poor awareness the family needs to do early marriage.

I: What about bith spacing?

P: It is improved, but rarely can you see short bith intervals. In rural area there could be short birth interval because of poor awareness. The husband may not know about his wife pregnancy in rural area, and this is because of poor awareness. For example let me tell one story, in 2002 EC we had conducted one study, while we did collecting the data we got a women had having 14 children and that all children’s have looking equal in height and are very malnourished. At that time the mother knows contraceptives due to HEW but her has band didn’t know, if the husband know, all this would not happen. Even she showed us the scar found on her hand which is because her husband forcefully removed the contraceptive that she was applied on her hand, because the husband didn’t know the importance of contraceptive at that time. About the issues of birth spacing both wife and husband should know equally. If the husband is prince he doesn’t need to use contraceptive, because it is not encouraged by the religion so in such case the wife has use the contraceptive in the secretion of him. Therefor religion has impact on birth spacing’s.

I: Can you think of any other opportunities to prevent early marriage and increase birth spacing?

P: If we need change in the community, both wife and the husband should know family planning’s. In the rural area the husband know nothing about his wife family planning’s. For urban it is promoted by television but not reaching for rural residents. In the rural area, the husbands know nothing but his wife uses contraceptive by secreting him. In the schools it can promoted and agriculture worker can teach the community also.

**Section 6; Multi-seectoral collaboration to improve maternal nutrition**

I: Do you feel it is necessary for your woreda educational to work with other sectors/institutions to address maternal nutrition?

P: Yes absolutely.

I: why?

P: We need coordination with other sectors to address malnutrition, like mainly with health sectors, social affairs, youth associations, agricultural office because it is responsible for safety net program, women affairs office. Thus we can work jointly with these sectors.

I: Why necessary to work with other sectors/institutions?

P: If we work effectively coordinated with these sectors the current nutrition problem will be solved. All these sectors can do promote nutrition in the community. The educational sector can do teach nutrition. The agricultural sector gives money for the community by the productive safety nut supporting system but beyond giving the money, should do teach the community for investing the given money for nutrition. The women affairs can do teach nutrition women’s and similarly the youth association can promote nutrition for youths. Accordingly, though time the expected change of the community will be achieved. Considering nutrition as only the health sector issue is a problem but if the nutrition issue is shared to the education sector, agriculture and women’s affairs, and these sectors has do intervening coordinately there will be good results in nutrition.

I: Have you such kind of coordination currently?

P: We don’t have effective coordination. As I am from education sector, my main focus is education only, and the health sector also focused only on health activities only. However, as the education we can promote nutrition in schools then community awareness will be improved. If the students know nutrition they will teach their mother while they will be back to home. Therefore, working coordinately is very important.

I: For multi-sectoral action that effectively works to improve maternal nutrition at all levels, what kind of change in terms of the way stakeholders work together is needed?

P: We should come together and set around the table for discussion. All sectors I mentioned above should set around the table and discussed on the joint work. For example I will not promote like health professionals, rather I can promote general nutrition information, so I need nutrition training and the health professional can give training for me in this regard. For this reaching common understanding among sectors is important.

I: What type of resistance to the needed change do you perceive or have you experienced so far?

P: Yes there is, time constraint, shortage of finance. The government will ask me if I am not doing on education but if I’m not doing on nutrition no one will ask me so I will focus only the education activities.

I: To what extent does your institution participate in the multi-sectoral nutrition coordinating body at the woreda level?

P: We are not involving; with health sector we have trials. With agriculture as I told you above we have joint work on the water and soil conservation activities. With women affairs we have joint wok on female sexual assault, on the prevention of early marriage, but have not nutritional intervention regarding breast feedings and other. Are you clear?

I: yes

P: Therefore we filed here on nutrition. Relatively we have good joint work with health sector, on the issue of promoting HIV, sanitation and hygiene and reproductive health, however still we don’t have fruitful joint work.

I: Do you have any other comments on anything that we have discussed?

P: In the school adolescent pregnancy is significantly reduced, it is almost none. There is significant change on contraceptive utilization by students to prevent unwanted pregnancy but relatively they are poor in the prevention of HIV/AIDS. The incidence of HIV/AIDS is increasing among school students in our area.
