# Supplementary material for: Barriers to utilize nutrition interventions among lactating women in rural communities of Tigray, northern Ethiopia: An exploratory study
Source: PLoS One. 2021 Apr 30;16(4):e0250696. doi: 10.1371/journal.pone.0250696 (PMC8087028; doi:10.1371/journal.pone.0250696)
Supplement: S2 File — (ZIP) [file pone.0250696.s002.zip › S2_File.Doc/Woreda level and above key informants/083_IDI_deputy head for Youth & Sport office_Tanqua Abergelle Woreda.docx]

OPERATIONAL RESEARCH ON ADOLESCENT AND MATERNAL NOTRTION IN NOTHERN ETHIOPIA

TOOL A

IN-DEPTH INTERVIEW GUIDE WITH EXPERTS, NUTRITION FOCAL PERSONS AND HEALTH EXTENSION WORKERS

PRINCIPAL INVESTIGATOR: DR. AFEWORK MULUGETA

DATA COLLECTION

IN-DEPTH INTERVIEW REPORT OF THE VICE HEAD OF YOUTH AND SPORTS OFFICE IN YECHILA TOWN, TANQUA ABERGELLE WOREDA, SOUTH EASTERN ZONE, TIGRAY, NORTHERN ETHIOPIA

YECHILA, ETHIOPIA

NOV 13, 2017

**Information sheet and consent**

I: Good morning. My name is Yasin Jemal. I am from Mekelle University. We are doing research on the factors that influence the nutrition of mothers and adolescent girls in collaboration with the Regional Health Bureau and UNICEF. Your participation is valuable. The information that you tell us will not be shared with others. However, the information will be recorded and used to improve nutrition programs and services for women and adolescents in the region and in the country. I have several questions to ask you that we have prepared in advance and we will ask you all to say what you think about each question. Ensuring privacy of everyone here not to speak what we discuss outside of this group is strictly not allowed. The interview will take 1:30-2:00 hours. Do you have any questions before we begin? If you have any concerns or questions as we proceed please feel free to let me know. If it is alright with you, I will turn on the tape recorder now.

Participant: Ok, I agreed to participate

= = = = = = = = = = = = = = = = = = = = = = = = = = = = = = = = = = = = = = = = = = = = = = = =

**Note:**

I: Interviewer

P = Participant

= = = = = = = = = = = = = = = = = = = = = = = = = = = = = = = = = = = = = = = = = = = = = = = =

**Section A: Interview details**

| Questions | Answers |
| --- | --- |
| Zone | South eastern zone |
| Woreda | Tanqua Abergelle |
| Town | Yechila |
| Kebelle | Mearey |
| Name of key informant | Yemane G/Haweria |
| Institution of key informant | Youth and Sports Office |
| Interviewer name | Yasin Jemal |
| Date of interview | Nov 13, 2017 |
| Interview start time | 10:45 AM |
| Interview end time | 01:05 PM |

**= = = = = = = = = = = = = = = = = = = = = = = = = = = = = = = = = = = = = = = = == = = = = = =**

**Section B: Socio-demographic Information**

| **Socio-demography of Key Informant** | |
| --- | --- |
| Questions | Answers |
| Sex | Male |
| Age | 31 years |
| Higher level of completed education | Bachelor degree |
| Current job/position | Vice head, Youth and Sports Office |
| How long have you been in the current job/position | 06  Months    00  Years |

**= = = = = = = = = = = = = = = = = = = = = = = = = = = = = = = = = = = = = = = = = = = = = = = =**

**Section 1. Common maternal (pregnant women, lactating women and adolescent girls) nutrition problems in the community**

I: What do women do to stay healthy in this community/woreda?

P: As to our community if the women want to stay healthy, to have good body and to have healthy life, the first thing is to improve nutrition. When we say nutrition, there are many different food sources in the woreda. Thus, based on the available food sources and by having time schedule, women should eat food regularly to improve their nutrition. So, I have the idea of this that women do to stay healthy.

I: What else?

P: The other is related to family planning service. Previously, our woreda was rural woreda and the coverage of family planning in our woreda was low due to low awareness but now it is getting improved the coverage of family planning due to the increasing number of health professionals in the woreda and high media coverage for family planning. Thus, the family planning service is improving from time to time in the health centers and is still not in full range. Specifically, the utilization of family planning service is improving among adolescent girls because of the awareness created in the schools related to health and as they increase their level of education. Adolescent girls also had HIV/AIDS test before marriage and also use family planning service when they went to their husband in order to have birth spacing. Thus, they stay about 4 to 5 years without giving birth after marriage that is before the first birth as they have also the interest to become strong economically. Therefore, family planning helps the women to improve their body. The other is the awareness/education itself. The awareness given in farmers based lessons by health workers or by administration as a whole help improve family planning utilization. Thus, women obtain health related lessons by giving public awareness to improve their health status and this is considered as one of the pillars among the strategies to improve health.

I: What else? What do women do to stay healthy?

P: The other is the affirmative action.

I: I mean to make stay healthy

P: One is to improve nutrition and second to use family planning. This is what I know but as youth they should also obtain health information/awareness in personal hygiene, environmental sanitation and in varieties of foods (balance diet) and their nutrient contents.

I: What are the common nutrition problems in the community for women and adolescent girls (10-19 years of age)?

P: One thing that is common and seen in our community is that selling all agricultural products and not using to their own food source since they are farmers. For example, they produce honey, butter and so on but they took them to the market and sell. Others like Taff, sorghum and oily seeds are also eaten independently without mixing that is in the way that cannot give good nutrition to improve their nutritional status. In other words, they eat one type of food made of the same source without mixing with other food sources for long time. For example, if they start eating sorghum, they eat only sorghum till it ends. Thus, they didn’t obtain balanced diet.

I: Thus, what are the common nutrition problems?

P: The problems are (one) their body become emaciated/thin, (second) they become weak and during the delivery they may be passed away. They also face anemia and after delivery their body looks yellow in color. In addition, they may be susceptible to other health problems. For example, they may be sick with malaria and died easily and they also affected by common cold easily. However, if the women eat balanced diet, they can prevent the diseases early.

I: What else? Is there nutrition problems like severe and moderate malnutrition, goiter, night blindness, stunting, wasting and underweight and shortage of food?

P: Good, I have already told you earlier that there is underweight especially among underage adolescent girls (i.e. early marriage) because immediately after marriage, they become pregnant since they didn’t use family planning, didn’t eat balanced diet so that they become underweight and the child that they born is also underweight and then the child faced stunting. We have seen this last time with save the children. Thus, the children become stunted. For example, a mother may have 4 or 5 children but she is short because she is stunted. The other is as I told you earlier, they may be susceptible to other diseases as a result of eating unbalanced diet. For example, there is a diseases called falciparum (type of malaria) that wasn’t known before in our community. So, as I told you if the women were eaten balanced diet, they should not be at risk. In addition, they didn’t come early when they get sick. Thus, rather than coming early and get treated, they stay at home so that the disease may not cure. One of the reason for this is lack of getting balanced diets. The other is we have observed many mothers/women who are targets of supplementary food due to lack of eating balanced diets. For example, there are many adolescent girls who are beneficiaries of corn soya for being underweight that is caused by lacked of eating balanced diets. Even previously, in save the children program there was information/report on how many children were stunted and how many women were wasted in our woreda. However, the most common problem in adolescent girls in this community is underweight and still existent even the data may be present in woreda health office. Thus, as an office what we do is that to identify how many adolescent girls are facing underweight so that to target them improve their nutrition. Goiter is not prevalent but what you can see is prevalent underweight and also there is stunting, wasting and high exposure to diseases. For example, suffering from common cold/flue and malaria easily due to lack of eating balanced diets and as the areas is hot climate. I have seen this starting from my mother. If eating sorghum is started, we continue eating sorghum till it ends. Again, it Taff is started, eating taff is continued till it ends. So, lack of eating mixed foods causes the problem. Therefore, though goiter is not prevalent in our woreda, it is seen in two or three Kebelles such as Agbe and Shekatikh that is in semi cold climate areas where as underweight and stunting are common/known and different are also done by health workers and in different stages efforts are continued. Currently, if I am not mistaken, there is a program called SURE (Sustainable Under-nutrition Reduction in Ethiopia) that assist the technical committee in each sector by developing a plan to create a condition that we can get out the problems within a short period of time. Thus, we were requested and given an assignment to develop a program. However, there wss lack of support and follow up. The SURE program had already started the activity and we were given short time by woreda health office to develop the program. Therefore, since the time was short period, the effort that we did to get out the adolescent girls from these problems based on the plan was less. This implies that there is a problem not only in the health sector but also in all sectors in making adolescent girls eat balanced diet but I think we will do it in the future.

**Section 2: Nutrition priorities in the woreda**

I: What priorities do your institution has in relation to maternal and adolescent health?

P: As I told you earlier, one is by making adolescent girls get balanced diet to have healthy children, to make them aware and use family planning, and to know their available resource in relation to having births. The other is giving awareness about communicable diseases such as HIV/AIDS to prevent risk of exposure as an office of Youth and Sport. The main focus currently is the slogan that a woman shouldn’t lost her life while giving life. This also includes adolescent girls. Though adolescents are as you said, those aged from 15-29 or 18 to 34 years of age are considered as youth. Thus, those women who are living in rural area and uneducated are our targets not to give birth at home. Second, reducing risk of exposure to HIV/AIDS and to have HIV test and make others to have HIV test. Third, making early marriage reach zero. The forth is about balanced diet that is by making them eat balanced diet to make them live/have good life. The fifth one is to make learn those uneducated women living in rural areas using practical learning by health extension workers or by our structure/network about health related issues, balanced diet, family planning and so on. Therefore, these are the aims that we have from higher to lower levels as Youth and Sports Office to improve the health of adolescent girls.

I: Why you have these aims?

P: One of the reasons that we have these aims is that if there are no adolescent girls, the existence youth men is low because to exist one youth man, the support of a woman at home is very big. Thus, if there are no adolescent girls, the other youth men will not exist. Second, there is difference between a house that has a woman and has no woman. So, based on this there should be balanced diet and health and without health, it is impossible to do any work. Thus, we understand this and accordingly we have to apply these things and make them ready. Then, after having these and created every condition, we have to identify whether the person has a job or not and thereby to create job opportunity for job seekers but we identified that the health needs should be fulfilled first.

I: Okay but currently which activities are you implementing to the priorities among the works that you have mentioned?

P: One of the activities that we implement is HIV/AIDS test because many adolescent girls are thinking and involved in unwanted activities after they finished grade 10. Second, to get balanced diet. The third is family planning to have and lead limited number of family. Fourth, to prevent early marriage since most of the adolescent girls are susceptible as they are from rural area and thereby to make them continue their education. Fifth, to prevent adolescent girls from working in bars as bar lady by creating job opportunity.

I: What nutrition interventions have the most resources allocated to them?

P: Of course, budget is given to the health sector and we two programs that is UNFPA and UNICEF. Previously, it has its own budget to work in youth but now the budget is allocated to health office. Now, budget is allocated to us based on the number of adolescent girls that we are targeting. The question that how many adolescent girls are targets matters the amount of the budget to be allocated. Thus, the budget is allocated based on this.

I: To which activity have you allocated the highest budget?

P: The highest is to HIV/AIDS. Budget for HIV/AIDS is allocated from woreda health office and from youth and sports office as UNFPA is sending us the budget. Regarding budget for balanced diet to adolescent girls, prevention of early marriage and family planning services, it is allocated by woreda women affairs office and woreda health office and the amount of budget is not exactly known. However, the highest budget is allocated by women affairs. Therefore, what we do is we allocate budget for HIV/AIDS as an office to prevent the risk of exposure whether they are university students or youths in the community.

I: Do you think is necessary for your institution to get involved in work aimed at improving nutrition among women and adolescents? Why?

P: Must because as a youth or as youth office our duty/objective is about the youth and this should not be forgotten. This is because a woman getting pregnant and to have a healthy child, she must get balanced diet. This means the child born today will become a youth and a youth is up to 34 years. Thus, a child become adolescent and an adolescent become a youth. This means we are the producers of the work force. Therefore, as we are the producers of the work force, we have to work in three of them so that its necessity is undoubted.

I: Would you please relate the necessity to get involved in work aimed at improving nutrition among women and adolescents with your institution’s mission?

P: Now, what our institution’s mission says is that to create/have healthy and physically fitted youth within 17 years. So, if the mission is this, these adolescent girls are the beneficiaries as they are under the youth and/or parts of the youth and when we say youth, it includes both sexes. Thus, if it considers 50:50, the issues of adolescent girls that we have mentioned such as underweight, family planning and risk of exposure to communicable diseases will be considered and are at the center when we do the activity. So, there are different clubs and associations, and what we do is that we are giving training by mixing those role model females and men together with those who have bad behavior so that to make them learn those who have bad behavior from those role model females and men. Therefore, the objective of the office is to enable and empower those who have weak attitude by those role model females and men in order to bring the desired change in the community. Thus, we have been working this together with our mission and we hope that it will continue in this way.

I: How do you evaluate the priority given for the interventions for the women?

P: Good. Now, how do we evaluate is that there were poor attitudes to women from men and even the woman herself has poor attitude in the previous times. There was also a condition that this attitude was affecting the country, the region, and the woreda but now if you see women, they are involving in irrigation, education, in balanced diet preparation and health activities. For example, home delivery is almost zero now since they are given health information by health workers and apply it practically. Therefore, targeting women/adolescent girls is very necessary as they are leaders of tomorrow. The other is that previously we evaluate and consider these intervention as they are the duty of the health office whether it is balanced diet or family planning but now every sector is involved and each has its own women development army. For example, women development army identify which women is the beneficiary of targeted supplementary feeding for being wasted/underweight. In this case, women affairs and we as office (Youth and Sports Office) get involved. Specifically, we target adolescents not to involve in risk sexual behavior based on the network of women development army in women and women development army in youth. Thus, to know who is, each women development army identify/knows those who gave birth at home, who had early/child marriage, those who have underweight child and others. So, now it is better than the previous times specifically since 2006 E.C (2013/14 G.C). In addition, the health office was working separately and even this was raised in the meetings but now whether it is in zone or woreda, every sector is working to support the activity if the issues belongs to each sector. On the other hand, if the sector didn’t contribute or do anything, it would be done and considered in the next activity. Therefore, it is evaluated in this way.

**Section 3: Nutrition interventions that improve adolescent and maternal health**

I: What kind of nutrition interventions are in place to improve pregnant women, lactating women and adolescent girls health in this community/woreda?

P: Yes. The main thing that we give focus in our community for adolescent girls, pregnant and lactating women is that to identify those underweight/wasted adolescent girls/women using the network of women development army and to make them the beneficiaries of targeted supplementary food to bring changes. Second, as youth affairs or women affairs those who are exposed to risks of underweight/wasting should be given trainings by those youth who are not exposed to risks. The third is to prevent early marriage by the network of women development army (WDA) since one of the members of the WDAs is youth and this youth is again a member in Youth Development Army (YDA). This means one youth is a member of two development armies. What to do is that, in the places where there is no ambulance service, to participate in what we called traditional ambulance. Traditional ambulance is a means of carrying pregnant women or sick individual to health facility to make her get health service. In this case, the youth who is the member of the two development army participate in traditional ambulance service and to follow the husband of the pregnant women whether he is forcing his wife to deliver at home or not. Therefore, the women development army must tell the pregnant mother to give birth in health institution and get balanced diet. So, we are working in these activities very effectively. There also supports from the higher up to lower level starting from ambulance, budget and materials. For example, as a youth we have youth center in the town and in the youth center we have one nutrition club. What the nutrition club members did is that by having/making house to house visit, they bring youth of the same age to the center and have tea/ coffee together to create awareness so as to provide home based care. Now, center will become youth sports and recreation center. Thus, the nutrition club will move to the hospital and make them get support by nutrition/health experts. Regarding utilization of iodized salt, there is still gap. There are two type of salts: iodized salt and non-iodized salt. Thus, there is still gap whether the public is using the recommended one or not and we had evaluated this last time. Here, there is also lack of follow up by the technical committee. Second, there is a problem related to home gardening as there is lack of irrigation. There is safety net program. There are some problems in making pregnant women not to work while pregnant. There are partialities and inconsistency in getting the services and giving permission to pregnant women by service providers and we are working hard to address the problem. On the other hand, there are also problems from the pregnant women themselves since they are afraid of disclosing their pregnancy status. Thus, they do not to get permission not to work. As a result, the chairpersons will make them stay to work in the activity since the pregnant women didn’t disclose her status to the chairman. However, if the pregnant women brought evidence from health facility that confirms their pregnancy status, automatically they will be allowed not work and get full service. Similarly, those underweight/wasted adolescent girls are also allowed not to work but get full service if they bring evidence from the health facility. On the other hand, we have also problems in providing the service to lactating women. Though lactating mothers are allowed not to work, they make them work that is against the law. Awareness is also given by health workers on the use of extra meal and having rest to pregnant and lactating women. Advice on ANC to pregnant women is also given by health sector. Regarding water, hygiene and sanitation services, it is given. From July-September 2017, there was Acute Watery Diarrhea (AWD) outbreak. AWD was first seen in Saharti Samre woreda near to our border and then, it spread to our woreda and around 78 persons were sick of AWD. Most of the affected individual were household head females and adolescent girls. Following the outbreak, we make the water bodies to be treated. Therefore, we treated 126 water wells. However, there are two or three Kebelles which have no access to water supply. There is also cleaning program every Friday to clean our office and once a week to clean the town. Advice on the need to use insecticide treated bed nets (ITNs) is also given in the woreda. To make improve the utilization of ITN, there is follow up by WDA members to make farmers not throw the ITNs. However, if ITNs are found thrown in the community, the WDA will be punished 50 Ethiopian birr (ETB) by its members and then the WDA by its turn punish the farmer who throw the ITN. However, there is limitation in ITN utilization since the farmers fold and hung it for a while and didn’t use it. Thus, it become dirty and there is difficulty when washed as its chemical removed so that it will be ineffective to kill the mosquito as malaria is prevalent in this community. There is also shortage of supply. About deworming service, there is the program but is given by health sector. In addition, there is corn soya support mothers/adolescent girls if they are underweight/wasted. Vitamin A supplementation is also given by the health sector. However, I don’t have awareness regarding deworming and Vitamin A supplementation. However, we discussed in the meetings to make mothers use micronutrient supplements. On the other hand, no investigation why the mother lacks micronutrient using laboratory in health facility. In this regard, there are limitations. Moreover, there youth friendly service in the community. In this youth friendly service, we focus on out-school adolescent girls since they are hopeless. We have peer to peer youth center that helps us create awareness by bringing these out-school adolescent girls to the center. There is also anther club called Niway club and works on showing about early marriage, HIV/AIDS, and balanced diet/feeding. Thus, they create awareness in timely and seasonal matters.

I: Which of the above interventions are most important to pregnant women?

P: The most important interventions are ANC service and getting balanced diet. Thus, these two are most important to pregnant women.

I: Why?

P: If they have ANC follow up, they will give birth healthy baby and also know her health status. In addition, if she eats balanced diet, it helps her to give healthy baby and helps her keep her body balance. Therefore, these two interventions are most important for pregnant women.

I: What about to lactating women?

P: To lactating women balanced diet is necessary. The other is postnatal care service since it is necessary for lactating women to reduce risk of infection/exposure. Balanced diet is also necessary for the child to grow healthy and to women not to get affected. In addition, personal hygiene and environmental sanitation are necessary for the mother and the child to prevent diseases.

I: What about to adolescent girls.

P: One, balanced diet is necessary. Personal hygiene and environmental sanitation is also important. However, prevention of early marriage (as the problem is more prevalent in the area), awareness creation about HIV/AIDS, personal hygiene and balanced diet are most important for adolescent girls. Balanced diet is important to replace the blood and not to get affected by diseases.

I: Which of the above interventions for the pregnant women are being implemented in successful way?

P: Among the interventions that we have mentioned ANC service is implemented successfully. Thus, ANC follow up is the first. In the meanwhile, awareness is given to a pregnant women not to give birth at home. To have a monthly check and to get learn fetus/baby positioning, awareness is given from higher to lower level.

I: Why?

P: If a woman is pregnant, she is the power of the house and this is not exaggerated. Thus, she should not lost her life while giving life. Previously, many children were died but now emphasis should be given from the higher up to lower. Accordingly, budget is allocated to the extent that can reduce the problems. If you go to the lower level there is health worker, health extension workers and WDAs and under WDA there is network.

I: What about the other successful interventions?

P: The second, successful intervention is advice on balanced diet for treatment of underweight/wasting. Thus, the women/adolescent girls should get balanced diet and are advised by health workers to take other foods to replace their weight. The third, successful intervention is personal hygiene and environmental sanitation.

I: What about the successful interventions implemented for lactating women? Why?

P: One is balanced diet since balance diet is important to the mother and the child not to get affected and suffer from diseases. The second is personal hygiene and environmental sanitation since it help them prevent different diseases.

I: What about the successful intervention implemented for adolescent girls? Why?

P: In-school adolescent girls are easy to access them as they are in school (known place) and we follow them day and night. Balanced diet, sports/physical exercises and prevention of HIV/AIDS are among the activities implemented as they are important to improve academic performance, to have healthy mind and reduces risk of exposure to communicable diseases. On the other hand, giving interventions in organized way to out-school adolescent girls as school girls is difficult. They interventions are fragmented. However, awareness about communicable diseases (such as HIV/AIDS) and personal hygiene (cleanliness) are successful interventions for out-school adolescent girls because there are different clubs working in these interventions such as HIV/AIDS and personal hygiene (cleanliness).

I: Which of the interventions targeted to the pregnant women was less successful? Why?

P: Activities related to safety net program is less successful since there is partialities and inconsistencies in implementation. Some may works while they are pregnant and lactating women. On the other hand, other pregnant and lactating women are allowed not work and get service. This may be caused by partiality of the chairpersons and by themselves not to disclose the reality as they are pregnant women.

I: What about the intervention implemented less successfully to lactating women?

P: Provision of Vitamin A supplementation is less successful because of lack of positive attitude though vitamin A supplementation helps to reduce night blindness among lactating mothers.

I: What about the intervention implemented less successfully to adolescent girls?

P: For in-school adolescent girls vitamin A supplementation is less successful because the service is very less and is ineffective due to lack of positive attitude in the adolescent girls. On the other hand, advice on diversified food (balanced diet) is not successful intervention for out-school adolescent girls. Any ways I don’t have such detail information about interventions which are less successful as to our office.

**Section 4:** **Implementation challenges and** **Community factors affecting access to nutrition interventions**

I: What are the challenges to implement delivering the nutrition interventions that we have been discussing for the pregnant women?

P: Here, the challenges that we face is lack of budget for providing supplementary foods like corn soya for those who are underweight/wasted. The other is lack of infrastructure (such as lack of health facility in remote areas, lack of transportation facility such as no roads, no cars or no motorcycles). In addition, those pregnant women who live in remote area don’t get antenatal and postnatal services. High staff turnover is also another challenge as the area is hot climate. For example, those very energetic health workers they leave the area after they serve 2 to 3 years since there is no additional benefits/allowance other than salary for working in the hot climate. Regarding this issue we have already informed the higher officials. The other challenge is that lack of positive attitude in pregnant women themselves. For example, the pregnant women do not come by their own to deliver in health facility rather they come by the efforts of health extension workers/health workers and they don’t also come by their own to get other services. Thus, they didn’t come like those pregnant women living in urban areas. In addition, there is lack of interest to learn, accept and apply what they have learn by the public. For example, they didn’t eat honey, butter and others while they have these foods rather they want to sell but the health workers are teaching them to use these foods for themselves. Moreover, there are community perceptions about having children because they said children are gifts from Saintly Mary, having children is wealth, don’t trust health facility rather believe in Saintly Mary. Besides, there is no support and courage from husbands in making to get their spouses health services.

I: What are the challenges to implement delivering the nutrition interventions that we have been discussing for the lactating women?

P: The challenges that we have mentioned above are also similar for lactating women. There is lack of budget for providing supplementary foods such as corn soya to those underweight/wasted lactating women and their children. The other challenges are also similar that is there is lack of infrastructure (i.e. lack of health facility, transportation facility), staff turnover, lack of antenatal and postnatal services, lack of positive attitude and lack of interest to learn, accept and apply what they have learn. For example, if the child is got sick, stunted, underweight, and wasted/emaciated, they didn’t take/bring him/her to health facility by saying he will be okay and his health will return and so on. And also use some cultural/traditional activities to make the child get heal his diseases or to cure the diseases but the child suffers. The perception about having children while you are young is also another challenge and is seen in the community since having children is considered as a gift and wealth. The perception to have children without birth spacing is encouraged that is they discourage birth spacing about 6 years rather to give birth closely and then have rest at the end. For example, they said Mrs X has 4 or 5 children and now she is free, has no worries because she has already finished giving birth but you have only one and you may suffer a lot when giving births later. The community also said if you have one or two children, who will assist/help you later after the children become old or after they join universities, who will be with you, do you want to be alone by then and so on. Thus, by saying these and others they encourage mothers to give birth so that they will have many children.

I: What are the challenges to implement delivering the nutrition interventions that we have been discussing for the in-school adolescent girls?

P: The challenges that we face are lack of positive attitude, not giving weight (or taking the issue as easy; for example, when you are talking about HIV/AIDS, they didn’t take it as big problem), lack of applicability what they have learn, lack of access to transportation facility to go to schools, lack of health facility in schools and lack of health workers in schools.

I: What are the challenges to implement delivering the nutrition interventions that we have been discussing for the Out-school adolescent girls?

P: The challenge is lack of contact time to get them and discuss because after they graduate from the university, they leave this area and start living in other area, or others also went to other area after they completed grade 10 or 12. Thus, it is difficult to establish organized committee in out-school adolescent girls but in case of in-school adolescents, they have their own committee. Therefore, out-school adolescent girls are dispersed and unorganized. Mostly, what we do is that we gave them a call via telephone/mobile and then record their address in computer to discuss with them but there is always difficulty to obtain them to give awareness. The other challenge is they have lack of positive attitude since they believe as they are old/matured. Lack of interest to learn, accept and apply what they have learn is also another challenge. Besides, public attitude toward those out-school school adolescent girls is a challenge. For example, they said them, what are you doing here? You have already finished your education, don’t you get job? Again, they discourage them by saying education has no use? You have learnt up to this but you do nothing. Thus, due to these pressures the adolescent girls went to Addis Ababa, or Axum to work in hotels as bar ladies while they are graduates of chemistry, biology and others at degree or diploma level and after spending many years in education. Therefore, there is big influence after you finish education and become hopeless. The community also encourage them the adolescents to get married by different means so that you will see adolescent girls as victims.

I: How convenience is interventions to the women and the adolescent girls?

P: I don’t think that it is convenient all in all because there are challenges that I told you earlier and as far as there is no full access or coverage in the area but more or less it is convenient.

I: How do you explain the quality of the interventions?

P: Except shortage of coverage, the interventions given are good in quality

I: What resources exist to provide the interventions?

P: Our resource is human resources. The second is financial resources and the third is the public itself.

I: What do not exist?

P: Lack of adequate number of health professionals and lack of water sources (lack of ground water source).

I: How do you evaluate the commitment of the intervention providers at your level?

P: In the previous times, it wasn’t but now there is commitment. So, it is good.

I: What other factors are inhibiting implementation of the interventions?

P: That is what I have mentioned earlier.

I: Can you tell me the solutions for the challenge you mentioned?

P: Yes, their solutions are one, improving the attitude by having organized committee, through awareness, practical learning and experience sharing. Second, employing/deploying high number of health professionals. Third, improving/increasing budget and the forth is to have infrastructure facility such as to have health and transport facilities though these are beyond the capacity of the woreda. The other is involving the stakeholders. Therefore, these challenges can be broken and have standard.

I: While your institution tries to solve the challenges, what problems do it faced?

P: Nothing as far as we solved the problems.

I: What do you think needs to be done to better address the challenges you have mentioned?

P: We, as an office what we need to be done to address the challenges is that to have transport access if we really need to change the women and we youth as a youth should be organized like women development army to work in lower levels. There are 20 Kebelles in the woreda and he woreda is big. As office, we have no car, motorcycle or other means of transport. Thus, we need at least motorcycle. The other is to create sense of ownership that can support from higher to lower levels.

I: How they can be addressed better?

P: To create opportunities such as stages to reflect concerns/idea. The other is the stakeholders such as education, health and others should be included in the committee to make them supporters.

**Section 5: Multi-sectoral collaboration to improve maternal nutrition**

I: Do you feel it is necessary at your level to work with other sectors/institutions to address maternal nutrition?

P: Yes

I: Why?

P: Because there is nothing that youth affairs alone can do and also nothing that women affairs alone can do. Work should be done collaboratively. Woman is also the pillar of the house. Therefore, if we don’t help this woman, if women affairs don’t help this woman to come to health facility, and if this woman is affected, the probability of the man to live is low. So, to help women, working in collaboration and involving in committee to change the attitude, structure and infrastructure and to educate and fulfill human resource is necessary. In other words, if there is no health, you do nothing. Thus, a person to get learn or whether a person is a professor or a doctor, he/she cannot able to get life without taking balanced diet. So, he/she cannot work without getting balanced diet. Therefore, nutrition means life.

I: Which other sectors do you feel are necessary to work with your institution?

P: With health sector, women affairs, water resources, agriculture, education and finance. So, if you say agriculture, all the foods/nutrients are produced from agriculture. If go to health, you will get all the health supply. If you go to education, you will obtain the educated work force and if you go to finance, there is finance/budget. Therefore, these sectors and others like women affairs, youth affairs and others should work in collaboration.

I: How do you see the other institutions’ roles complementing your role in improving maternal and adolescent nutrition?

P: The role of other stakeholders in complementing our activity is good. Previously, especially before five years, every sector was working separately and no one was complementing. Currently, no one is working alone though there are some limitations in making it complete. For example, the health sector support us in the prevention of HIV/AIDS and early marriage among target adolescents/youths. The education sector also support us in educating nutrition education.

I: what kind of change in terms of the way stakeholders work together is needed to improve maternal and adolescent nutrition at your level?

P: There should be timely monitoring and evaluation, supervision from higher to level levels, cross-check the executives and fulfilling the required human resources. There should also discussions, common plan and so on. For example, there is interface among stakeholders with health and water resources but the interface should also be examined.

I: What type of resistance to the needed change do you perceive, or have you experienced so far?

P: The challenge that we experienced is staff turnover and this may also happen in future too due to this we are not the beneficiaries in getting adequate health professionals. We have asked the region to make some additional benefits such as allowance for working in hot climate but not yet solved. The neighboring woredas are the beneficiaries of the allowance for working in hot climate but not in our case. For this reason, doctors or health officers do not stay here to serve the public since there is no additional allowances for working in hot climate. The other challenge that we have is lack of infrastructure such as lack of health facility and transportation facility and these challenges may also be persistent in the future too. There is also insufficient/shortage of ambulance for pregnant women as there are distant places. Thus, having 1 or 2 ambulance(s) is not sufficient to give service for more than 11, 000 women in the woreda. The other challenge that we faced is early project phase out that were supported/funded by NGOs that is projects came but they early phase out. Children were supported by the projects but they phase out early. For example, UNICEF did good job in youth and health but UNICEF project aimed at supporting youth has phased out. Now, UNICEF supports the health sector only in our woreda. Therefore, if the projects phased out before the graduation of the children, the children will be affected and is a challenge. The budget allocated by UNICEF was more than the woreda budget. Many activities planned to be done by the UNICEF project are now stopped. Thus, if possible the government should discuss with donors to work in collaboration by saying please support in this, and I will do this, and when we do this, please you also do this. Therefore, the activities that we planned to be done by projects are not done and stopped since the projects are phase out early and as we have lack of budget to cover the cost from our own. So, if projects phased out, woreda alone cannot address the activities.

I: Is there coordinating platforms in enhancing multi-sectoral coordination in maternal and adolescent nutrition?

P: Yes, there are.

I: To what extent does your institution participate in the multi-sectoral nutrition coordinating body at this level?

P: We have full participation. We have many activities and we cannot do alone everything. We request the concerned body and they did it perfectly. For example, we mobile and bring the youth to the public meeting and then we would tell the health sector to teach some programs such as HIV/AIDS, nutrition, or other program to the youth. Thus, the coordinating bodies have their own time and we work accordingly but now what we left is that to identify/investigate the works/activities that we are going to do. Therefore, the extent of our participation is highest. For example, there is no nutrition education but there was education not to be sick of HIV/AIDS, malaria and about not to give birth at home. However, education about nutrition and how a person can live a healthy life based on nutrition is started in past 2 or 3 years.

I: How effective are the coordinating platforms in enhancing multi-sectoral coordination?

P: Actually this matters the area. The coordinating body wrote letter to all concerned the meeting time and place. Before the meeting, they tried all they can and even they come physically, and also they gave calls to coordinate. The other thing is that they report came by those coordinating body is more or less matches with the existed condition. This is my observation and I know based on this how they are working. When they give assignment and duties to women development army by saying do this, this and this, we understand/know that they are in work. They are also good in bringing all sectors activities by merging common activities together. So, they are effective.

I: What needs to be done to improve the capacity of these bodies/platforms for effective coordination?

P: Though I have no information about their academic status and performance, making them to improve their academic status is required. Taking training on capacity building management is also needed. Employing the required number of staff if there is lack of experts/human resources. Reference and manuals should also be provided so that to improve and update their knowledge. Training should also be given to them about nutrition. For example, training is given to us in woreda for a day or half (either in the morning or in the afternoon) and the training focuses/bases on evaluation. Thus, training should be given about what is nutrition and how nutrition can help us improve our health. For example, I have no awareness about nutrition Program Implementation. All sectors should also have awareness about nutrition. Manuals about food nutrition should also be available in all sectors such as in youth affairs, in finance, health and agriculture and so on.

I: What opportunities do exist to promote multi-sectoral coordination of nutrition in this woreda?

P: The opportunities about nutrition opportunities are presence of different stages (like conferences since there are big conferences) to create awareness on nutrition. The other is presence of experts from universities to conduct research and to participate with us. Presence of university graduates is also another opportunity. There are also other stages such as in agriculture/irrigation stages. In addition, presence of target populations are also opportunity. Awareness on nutrition in all sectors, presence of donors/funders, presence of health extension workers, presence of development agents and presence of women development army are also opportunities.

**Section 6: Other interventions that influence adolescent and maternal nutrition and health outcomes**

I: In your opinion, why would delayed marriage (after 18 years) improve maternal nutrition?

P: Since she care herself and eat balanced diets. As the woman get nutrition education from her colleagues such as women development armies, she can improve her nutrition by taking balanced diets. The other is she can improve her attitude and approaches so that she can improve her diets and thereby her nutrition.

I: In your opinion, why would increase the space between each birth improve maternal nutrition?

P: The question about how increasing birth space between each birth improves maternal nutrition is (one) the mother gets balanced diet if she increases birth space and even this is true for the child too. Thus, increasing birth space makes to get enough food both to the mother and the child. For example, the amount of balanced diet available in a household with 2 children is different from when the number of children in the household becomes four. Therefore, increasing birth space between each birth is important to obtain balanced diet.

I: What programs or activities promote increasing birth intervals in this level?

P: Though there is committee, there are sectors that fits this activities such as the health sector, and office of women affairs. In health sector, there is UNICEF program that promote increasing birth spacing. Family planning services such as injections and tablet/pills are also other programs that promote birth intervals. The other is traditional way that is releasing the sperm outside the vagina during sexual intercourse. There are contraceptive injections that can be used for 3 years, 1 year or months and tablets/pills used for 72 hours. Here, budget is required to provide all these service to the users.

I: What programs or policies are in place in this woreda to prevent early marriage?

P: I don’t have awareness/knowledge in programs that prevent early marriage. However, as sectors there are women affairs, health office, we as office, police and security and justice office/courts.

I: What about the policies?

P: By the way, I don’t have awareness/knowledge in this issue.

I: Okay, how do you stop early marriage/

P: There is law. In addition, there are manuals prepared by women affairs that states about the punishment (if you do this, you will be punished this; for example, if you get married underage, you will be punished this), the effects of early marriage and benefits of delaying marriage after 18. There is also justice office. Thus, it will go court and then punishment.

I: What else other programs or policies that prevent early marriage?

P: Religious fathers also involved in prevention of early marriage. Engagement starts from end of October to January and April to May but the wedding may be later either in August or any month. So, these periods are considered as active engagement times. On the other hand, there is also support from political parties. The health sector may also support prevention of early marriage. However, I don’t have knowledge whether the donors support prevention of early marriage or not.

I: In your opinion, are these programs or policies effective?

P: As my belief, they are effective because there is difference in the number of early marriage when compared to the previous time and the current one. Even the fathers/mothers wait until the girl reaches 18 years old but there may be early marriage in some Kebelles. In some cases, if the father/mother wants his/her daughter to get married, father/mother will come to justice office and go to the vital statistics section/division to register the age of his/her daughter and based on that we will follow the situation. There is also work force committee led by the woreda administrator that follows the marriage issue in the community. This committee has 7 or 9 members that is the woreda administrator, police, agriculture, women affairs, 3 members from three women’s league association (locally called timret), similarly from youth league and from our office (that is youth and sports office). However, if they are from out school adolescent girls the activity will be done by youth affairs, women affairs and health office (health extension worker).

I: What are the community factors that affect age at first marriage?

P: One of the factors is the physical conditions of the woman such as breast. The second is the interest of both the father and the mother to see her marriage and the third is religious influence. For example, religious father com and said what are you doing your daughter is already reached, so give her husband. Thus, the interest and chances of the girl is not considered rather influenced by these factors and get marriage while she is underage.

I: What are the policy factors that affect age at first marriage?

P: The policy factors are considering the consequences of early marriage and its impact on the health of the woman such as uterus cracking, death and giving unhealthy child. The other factor is from economic point of view that is the woman may face economic challenges to support her family. Physical and mental maturity are also other policy factors since she will face difficulties to support and lead her family.

I: How to improve prevention of early marriage?

P: Brining attitudinal change by creating awareness in all levels (from higher to lower levels) in different public meetings, improving policies, laws, involving all stakeholders and experience sharing are needed to reduce early marriage.

I: Can you think of any other opportunities to prevent early marriage and increasing birth spacing?

P: One is the public attitude itself. The other is support from religious fathers by saying that I will not be your father if you make your daughter get married while she is underage. Involvement of stakeholders from higher to lower level in creating awareness and giving training about the impacts of early marriage. Targeted schools/education, health institutions and women development armies are also other opportunities in prevention of early marriage. For example, school director is the chairman and secretary of early marriage committee at kebelle level. The health extension worker is also the minute recorder of the committee. Thus, based on these such opportunities are created and in the last 2 to 3 years, there is no early marriage. However, it may happen if the girl is close to age of 18 years (i.e. 17 years plus some months) and if she is physically big/huge and then by saying I have already reached 18 and in some cases she may get married. Moreover, presence of law is also another opportunity.

I: What lessons have you learnt regarding adolescent and maternal (pregnant, lactating and adolescent girls) nutrition at your level?

P: I have learnt about the importance of balanced diet to mothers and thereby to have a healthy child. Though it is this much, I am the user from nutrition education. If a person didn’t balance his diet, he will not have good mentality and strong physical body. The other is to have a mother a fast growing and strong baby, she should feed her child balanced diet to her child and eat to herself because if the child didn’t get balanced diet, he may get died and suffer from underweight, stunting and wasting. On the other hand, I have learnt the importance of supplements to mothers to prevent problems.

I: What lessons have you learnt regarding multi-sectoral coordination of nutrition in this woreda?

P: Previously, nutrition was given to health sector but now it is the issue of every sector from higher to lower levels by establishing committee and this is one of the lessons that I have learnt. For example, Now, I can talk about nutrition/balanced diet on behalf of youth affairs and no one say don’t talk about this but based on the previous attitude, it the duty of the health sector. Thus, this attitude is now changed and I can talk about balanced diet by delegating youth affairs, water resources can talk about clean water and other can also talk what they belong. Therefore, we have learnt such lessons. The other thing that I have learnt is if there is no balanced diet, no one can live.

I: What opportunities do exist to promote maternal (pregnant, lactating and adolescent girls) nutrition in this woreda?

P: One is the existing structure. Second, presence (establishment) of technical committee to make learn women improve their nutrition is another opportunity. The third is presence of strong women development army. For example, there was no women development army in the previous time but now there is this development army.

I: Any additional remarks

P: What I thought to be included is that food nutrition should be strengthened. There should also be media coverage to get lessons. The other point is that there should be manuals for nutrition.

I: Thank you very much. I have finished.

P: Welcome.

**SUMMARY**

**Section 1: Common maternal (pregnant women, lactating women and adolescent girls) nutrition problems in the community**

To stay healthy women/girls should eat balanced diet, keep their personal hygiene and clean their environment, use family planning and have HIV/AIDS test. The common nutrition problems in the community are stunting, wasting, underweight, goiter, anemia and night blindness.

**Section 2: Nutrition priorities in the woreda**

The priorities include: to make adolescent girls/women get balanced diet, giving awareness/advice on balanced diet, awareness on how to prevent communicable diseases such as HIV/AIDS and reducing early marriage to zero are the main priorities as youth and sports office.

**Section 3: Nutrition interventions that improve adolescent and maternal health**

Advice on ANC service, balanced diet, extra meal, use of ITNs, water, hygiene and sanitation, early marriage prevention, targeted supplementary feeding, iodized salt and giving training/awareness on different issues are the interventions which are in place. From these intervention the most effective interventions for pregnant women are ANC service and advice on balance balanced diet. Similarly, advice on balance diet, and advice on water, hygiene and environmental sanitation are the most successful interventions for lactating women. On the other hand, advice on balanced diet, sport activities and prevention of HIV/AIDS are the most successful interventions for in school adolescent girls. In contrary, safety net program is less effective intervention for pregnant women while vitamin A supplementation is less effective for both lactating and adolescent girls.

**Section 4: Community factors affecting access to maternal nutrition interventions**

The implementation challenges and community factors include: lack of budget, lack of infrastructure (such as lack of health facility, lack of transportation/no roads), high staff turnover due to lack of additional benefits as the area is hot climate, lack of interest to learn, accept and apply by the public, presence of community perceptions such as considering children as a gift and wealth, and lack of support from husbands to make use their spouses the available health services.

**Section 5: Multi-sectoral collaboration to improve maternal nutrition**

Working with other sectors such as with health, women affairs, water resources, agriculture, education and finance is necessary. However, to bring the required change timely monitoring and evaluation, supervision at all levels, cross-check the executives, having common discussion and having common plan is needed.

**Section 6: Other interventions that influence adolescent and maternal nutrition and health outcomes**

Delayed marriage (after 18 years) and increasing birth spacing between successive births can improve maternal nutrition since the mother and the child can get adequate balanced diet. Public attitude, support from religious fathers, involvement of stakeholders, presence of schools, presence of health institutions and presence of women development army are the existed opportunities that prevent early marriage. Similarly, the existing structures, presence of technical committee and presence of women development army are the opportunities to promote maternal nutrition.

THE END

REGARDS,

YASIN JEMAL
