# Supplementary material for: Barriers to utilize nutrition interventions among lactating women in rural communities of Tigray, northern Ethiopia: An exploratory study
Source: PLoS One. 2021 Apr 30;16(4):e0250696. doi: 10.1371/journal.pone.0250696 (PMC8087028; doi:10.1371/journal.pone.0250696)
Supplement: S2 File — (ZIP) [file pone.0250696.s002.zip › S2_File.Doc/Woreda level and above key informants/084_IDI_head for Women Afaire office_ Samire woreda.docx]

**Operational Research on Adolescents and Maternal Nutrition in North Ethiopia**

**Introduction**

Hello my name is Yemane , I am from Mekelle University; we are conducting a research on the factors that influences the nutrition of mothers and adolescent girls in collaboration with the regional health bureau and UNICEF. Your participation is very valuable; the information that you tell us will be used to improve nutrition programs and services for women and adolescents in the region and the country. We will not share your names when we report our results. The interview may take 1-2 hours and I would like to thank you for taking the time to speak with us today. You have the right to withdraw at any time and I will use tape recorder. Are you voluntary to participate for the interview?

**Yes** No

| **Section A: Interview details**   1. Zone: **South Eastern Zone of Tigray** 2. Woreda: **Samire** 3. Kebele: **Samire** 4. Name of key informant: **Akeza Alemseged** 5. Institution of key informant: **Women affairs** 6. Interviewer name: **Yemane G/mariam** 7. Date of interview**: 13/11/2017** 8. Interview start time: **09:30AM morning** 9. Interview end time: **5:17 AM morning**   **Section B: Interviewee professional information**   1. Gender    1. **Female B.** Male 2. Age: **32 yrs** 3. Highest level of completed education.    1. College education    2. **Bachelor degree**    3. Master’s degree 4. Current job/position: __**Women Affairs Head** 5. How long have you been in the current job/position: **4 Years** |
| --- |

**I:** Interviewer **P:** Participant

***Section1:* Common maternal (pregnant women, lactating women and adolescent girls) nutrition problems in the community.**

**I: In your opinion, what are the common nutrition problems in the community for women? What about for adolescent girls?**

P: As we know as Tigray or as woreda the focus of intervention are as listed above (PW, LW and children). PW and LW may suffer of malnutrition if they are not feed balanced diet especially during pregnancy and delivery. Due to malnutrition the child become low birth weight, anemia due to excess bleeding and unable to get enough (balanced) diet.

**I: Can you explain to me about malnutrition and its problem?**

P: The content of the food must be balanced diet in which it can able to provide good sources of nutrients. This can be obtained by eating what you have in the house like milk, eggs and other foods if you couldn’t get all this you may be exposed to different disease.

**I: What are the different diseases can tell me?**

P: For example during labour she may has excess bleeding, her child can be easily affected by cough (common cold), after delivery she may get difficulty to get strength and even though there is no maternal death but child death is common this is due the mothers were not well nourished during pregnancy and after delivery. Most of the time neonate is borne with low birth weight due to poor maternal nutrition. Their feeding practice is different before pregnancy and after pregnancy as well as during lactating time.

**I: Can elaborate what could be the difference in the feeding practice?**

P: The feeding frequency of meal is different; for example pregnant women must eat one extra meal while lactating mother should take two extra meals. But due to no awareness creation to the community they may eat as usual: even though they have enough food in the house. Whereas practically she may eats two times per day while her families are eating three times per day but she is expected to eat one or extra meal per day.

**I: In your wored is there PW, LW and adolescent girls who get fafa and Plump net? At what condition they received it?**

P: Yes, now women are receiving fafa and oil even though plump net was given for children but now it is not given. I am not expert but the health professional measures their MUAC and based on the measurement they give them fafa and oil. There is fafa coming from USA and it is balanced diet; distributed to health post and given by health extension worker (HEW) to be distributed based on the MUAC measurement.

**I: What is there evidence to distribute or to give the fafa?**

P: The HEW measures the MUAC of PW and LW; if the pregnant women are below normal she will be provided fafa since it is assumed if pregnant mother get malnourished the baby will be affected or get malnourished. The same is true for lactating mother if she is below the normal range the fafa helps her and the child to get strong and healthy even though I don’t know the cut of points of the MUAC measurement. The women development army (WDA) and HEW are responsible for handling to bring the children and mother to be screened and distribute the fafa. But now the fafa and oil is not given instead they are giving USA made food prepared from different nutrients as we observed while we was in supportive supervision and it was packed sack. Every pregnant woman received 5 kg per month.

**I: Is there micronutrient deficiency related problems among PW, LW and adolescent girl?**

P: Goiter was seen among women especially around one kebele namely fre weyane boundary with south of Ofla which is common among women and this is due to Iodine deficiency now we are teaching that all families should used Iodized salt to prevent goiter if the mother has taken iodized salt the baby will be free of iodine deficiency. Goiter can be prevented if the mother has properly used Iodized salt this was studied by ministry of health and Tigray regional health; adolescent girls with 18 to 19 year, children, and women, pregnant and lactating women are highly affected by Iodine deficiency specifically the fre weyane kebele. Even though it is reducing but still there is goiter and the community are using iodized salt by using the advice of HEW and WDA. The iodized salt should be poured after the end of cooking to prevent the evaporation of iodine but we are not sure whether the community has used iodized salt or not: this could have its own problem to follow the user of iodized salt. Now we have widely taught on the importance of using Iodized salt and the users are increasing in their house.

**I: What do you think goiter is common in Fre weyane Kebele?**

P: As I have said, goiter is common in Fre weyane even if it is presence in other kebele this could be due to its place but I am not sure but it has been studied. As administration body we have identified the kebele as high goiter prevalence this may be due to the water and the soil are not reach in iodine.

Anemia is due to low nutrition intake of balanced diet sometimes this happen due to not eating properly even though it is available in the home.

**I: Is there communicable and non communicable Diseases in your woreda?**

P: I am not sure this is beyond my expertise.

**I: Is stunting common in the community among PW, LW and adolescent girl?**

P: Scientifically stunting is due to malnutrition which begins from pregnancy and lactation till the growth of the baby but in the community they assumed it is due to natural especially children of rich family are short therefore this couldn’t be due to malnutrition since they have everything to be eat and short therefore stunting is not due to malnutrition. But now we are working a lot on why stunting is common and creating awareness to the community about the feeding practice before pregnancy, during pregnancy, during lactation and till the growth of the child by working with HEW and WDA. On some kebele we are introducing home gardening to plant vegetables. We women affairs are working with WDA and HEW. We are explaining the prevalence of stunting starting from Ethiopia, Tigray, woreda and kebeles to the community the reason for the stunting is due to malnutrition.

**I: What is the understanding of community about overweight?**

P: It is unlikely to have over weighted person but if there is over weighted one the communities perceived it reciprocally as he/she is eating well gets obese. We are in stunting and till now our problem is not over weighted.

**I: Can you say your woreda is food secured? Why?**

P: We cannot say it is food secured: we are in need of support therefore we are food insecure woreda.

**I: What are the food supports?**

**P:** The supports are like cereal, crop, oil and bean which is needed to the community.

**I: Who are receiving the food support?**

P: Before the food support is given we will do income/production analysis which means the amount of quintals of produced and number of family. Therefore the food support may be given for three, six, nine months and for 1 year based on the production analyses that have low production especially for women, lactating mother and for mothers having children. The food support is not freely they have to work in safety net based on the production analysis and for those who are totally poor they get full year chances to work in the safety net program. Whereas pregnant women the food support from the beginning of knowing her pregnancy till the baby become one year she will be provided food support without work. The problem in this community till they become three or four months of gestation they didn’t check and come to get the safety net rather they work hard in the farms which may lead her to get fatigue and exposure to sunny. Now we are working with REST on pregnant and children to reduced exposure to sunny since it lead them to be tired. The government and the NGO are supporting the food for free like for HIV patient, children, pregnant, lactating, elder and poor people.

**I: Is their precondition to be fulfilled by PW and LW to get food support?**

P: Yes, they must announce their pregnancy immediately once they tested for pregnancy therefore they can get support and reduced workload. We have registering vital events but the mothers are dinning the age of their children by reducing below one year to get the food support. Women and women having children who are working in the safety net there is an opportunity for them to get to work late and to go for lunch earlier than men since they are responsible in preparing food for the children and their husband.

**I: Is their stunting in your woreda? Which one is more affected by stunting PW, LW or adolescent girl?**

P: The percent of stunting in the Region can also explain the percent of stunting in our woreda. We don’t have separated data on stunting for PW, LW and adolescent girl.

**Section 2: Nutrition priorities in the woreda**

**I: What is/are your priority interventions related to maternal and adolescent girl nutrition as women affairs office?**

P: Yes, as women affairs we are not working by assigning budget specifically on maternal and adolescent girl nutrition. Since the office has different tasks we work with different partners or stake holders or multi-sector like NGO, Health, REST and world vision specifically on supporting pregnant and lactating women since we are more responsible for women as office. Even though we don’t have specific budget we are working with different sectors like health office, health office is our 2^nd^ office net women affairs office.

**I: What do you do with health office about maternal and adolescent girl nutrition?**

P: When we have meeting about budget we were supporting the budget for maternal and adolescent girl nutrition during the approval of budget and once the budget approved we will work together the HEW and WDA on nutrition diversification to reduce stunting. With agriculture we work on the importance of home gardening like fruits and vegetables which may help them to have variety of foods to be eaten even they can sell it to the market. And by selecting model women on using home gardening we will use them as model women and share their experiences to the community in demonstrating the importance of home gardening and using balanced nutrition like vegetables, sugar potato, egg and meats. On the last three years we are working on reducing stunting with different stake holder about maternal and adolescents nutrition. Women are expected to participate in nutrition improvement whether they are married or single in improving their nutritional status.

**I: Do you work with other sector than health and agriculture about maternal and adolescents? What is their role?**

P: Yes, we are working with Youth affairs office since they are working on youth girls, we also work with education office since we can address female students in different club and we teach them about balanced diet, early marriage, family planning and sexual and reproductive health. In addition administration office is working with us in controlling the overall activities of the different sector.

Water resources offices are working with us on digging water sources which can help for women which help women to have home garden with different vegetables. For example in this year we have 51 women who are benefited from using home garden due to water access in their villages. The overall responsibility of water resource office is to avail water for drinking and for agriculture especially in focusing for women. No offices are free of participating in women issues even though they may not be involved on maternal nutrition.

**I: What is the role of Youth affairs? Can you explain it?**

P: The office is responsible for both young male and female related issues. Its focus is based on female education like early marriage, pregnancy and delivery so if it happens what must women do to make her safe and healthy focusing on feeding practice and family planning.

**I: How do you evaluate your collaboration level with other sectors?**

P: Since we are responsible about women issues while we are working with other sectors we have difficulty in working together and having close follow up on what is going on in the ground related to women nutrition which are doing by different sectors like education, health water and health office even though we have shortage of staffs. I believe we have gap in having close follow up by assuming we are responsible and accountable for women issues since we are expected to record all activities done to women for example what has done agriculture, health, education and water office to women but we have only four workers in the office whereas the work in the office is very busy. Therefore even if we believe it is our work we couldn’t say we have made concrete change on maternal and adolescent girl nutrition. In addition in collaboration with different sectors we couldn’t say we have made all things to the women in our woreda. In the New Year we have evaluated our performance and forwarded that we must work a lot to tackle the challenges faced by the women.

**I: What must be done to improve women nutrition what you have told me above?**

P: If we need to have an improvement on maternal and adolescent girl nutrition all multi-sectors should work together collaboratively by assuming women issues are their issues. As well as the community should be responsible in caring of women, PW, LW and adolescent girls in fighting and reducing of stunting through improving feeding practice. Even if it may be difficult to bring change at a time but if each sector (agriculture, health, education and water offices) are working their responsibility and accountability on maternal nutrition we can improved the nutritional status of PW, LW and adolescent girls even though we couldn’t reach still there is promising result which is seen in some kebele. For example in our one kebele all the households have home gardening like salad, chili onion and tomato in which the kebele was model for the zone and we have visited it. Even if the women don’t have place for gardening they were renting a land which is five by five meters square and garden plant for themselves even if they sell it to the market but we haven’t pushed the work in expanding into large scale and this is our limitation in which we have to work hard.

**I: Can you tell me why it was successful? And why not expanded to other kebele?**

P: Specifically for the success of the kebele it was started by one WDA and all WDA were following her experience on home gardening as well as all households was following the same practice. Previously when water passes through their home simply they were looking it but now they turn the water and use it to drink their home garden. The model WDA was showing them practically by selling it to the market and all households were follow her path even if later on they start produce sesame. Now there are two groups of WDA having 30 members in each group with total of 60 women are using home garden and they have discussion on feeding practice of their home and shared their experiences. While they have discussion world vision were looking them and start supporting them in home gardening as well as we women affairs were following and encouraging them. During March 8 we have done experience sharing to different kebeles by taking the model WDA but still the expansion is not good this is due to our less follow up. In Gijet there are 50 women who have introduced home gardening in their home which is promising but we must to work hard in creating awareness to the community on the importance of home gardening.

**I: What are the challenges in expanding to large scale?**

P: We were planning to expand to each kebele to share the experience but due to lack of budget for transportation and personal expenses we were unable to scale up in which it may help us in creating awareness to the community. The communities don’t need to get tired while they work; let alone fetching and carrying water to home gardening they didn’t use passing water in their home and this is due to negligence and low awareness. For example water is passing through two individuals farm the one used it properly and grown his plants whereas the other one simply looking while the other works this is due to laziness. The community is short period planner for example when they to plant papaya they simply look the years of the papaya starts to give fruits for example after 5 years rather than what can be gain from the papaya after five years. Therefore simply by looking the time they give up to plant papaya and others.

**I: Is their platform which helps different multi-sector to work collaboratively about maternal and adolescent girl nutrition?**

P: We don’t have any system which allows us to work together in women nutrition issues rather simply we work in our direction. We don’t have strong committee which follow and evaluate activities related to women we are simply working individually with less coordination. Rather we assume health office has major responsibility in keeping women nutrition and agriculture has playing its own role in reducing malnutrition among women by having home garden but when we do this we don’t start by having common objective or agenda in improving women nutrition simply we are doing it haphazardly.

**I: What must be done to improve the multi-sectoral collaboration?**

P: Every sector must work collaboratively by focusing on improvement of women nutrition by establishing strong committee which focus on women nutrition like to that of other committee. The women nutrition couldn’t be improved immediately but can be improved gradually if the committee follow closely and become responsible. The committee should be established from different sectors like agriculture, health, women affair, youth affair, education and water resource office by specifying their role and expectation on how to improve women nutrition. Even though we do have many committees which is difficult to attend all but at least the above sector must established committee which works on women nutrition improvement since it is basic for continuation of life. Health office was taking the lead in improving maternal nutrition but now we must take the lead and work with different sectors to improve maternal nutrition.

**I: What are the opportunities in this woreda to work collaboratively with multi-sector to improve women nutrition?**

P: The most important opportunity by the different sectors is they have interest or have their own issues about women even though they are doing it independently. What we have to do is bringing them (Multi-sectoral committee on women nutrition ideally established during the interview) for discussion and set common goals about women nutrition to be intervene together collaboratively. And all sectors must believe that they have their own contribution for the improvement of women nutrition.

**I: Do you think working on the improvement of PW, LW and adolescent girl nutrition is related with your mission?**

P: As I have said widely this is our responsibility keeping the health status of women in our wored as well as this is also our mission. Our priority more focuses on how to make women beneficiary from different activities and with what intervention. In addition we work on what must be educated to the women to eat variety of foods and balanced diet we know that this is our responsibility. We have different women leagues in which they focus on advising child feeding, adolescent girl feeding, pregnant and lactating women. Well nourished mother can be healthy and safe as well as give birth with normal weight baby.

**Section 3: Nutrition interventions that improve adolescent and maternal health**

**I: What are community level interventions that help to improve maternal and adolescent girl in your woreda?**

P: The awareness given about nutrition feeding practice to the community is not adequate; this has lead the community to have less awareness on feeding practices of PW, LW and adolescent girl. The education given to PW and LW is not enough about their feeding even if there are some PW and LW who follow the feeding practice. For example the lactating mother is responsible for herself and the baby as sources of food therefore she needs to more extra meal. The challenges are most of the women are living in rural area and low awareness about the importance of feeding women. If adolescent girls are well nourished the child they borne will be safe and healthy therefore before pregnancy an adolescent girl must eat extra meal as compared to male since she is the responsible one in creating the next generation. But practically in our community there is no special treatment given on nutrition intervention for adolescent girls. General I couldn’t say there is good nutrition intervention for PW, LW and adolescent girls in our community whereas this all intervention the most important in creating the next generation.

**I: Do pregnant women follow ANC in your woreda?**

P: The maximum number of ANC is four times whereas as we observed most of them are not attending more than one or two times during their pregnancy till the delivery. Based on the standards it says any pregnant should attend four ANC till delivery but the community don’t followed it once they come for the first visit in the next they come for delivery.

**I: What do you think the reason they didn’t come for the ANC visit?**

P: Most of the pregnant women don’t realize the consequence that is why they are missing the visits by assuming it is enough since I have already checked and treated why I come again and again. The other reason could be due to distance and transportation since they come on foot to the health center and health post even though ambulance is available during delivery. In addition they don’t want to be seen as pregnant.

**I: What is the reason they don’t want to be seen as pregnant?**

P: Since the community speaks about the number of pregnancy she had and number of births given as well as number of abortion therefore they don’t want to be seen by the community. The other challenge to visits health center is when they come for ANC they spent one night in the nearby family houses this gas causing them not to follow their ANC and this is very difficult for pregnant women having children in her home. At this time she may assumed the examination and treatment given is enough till the delivery as well as the community tell her the first visit is more than enough for her. But when she gets difficulty in giving birth during labour due to less ANC follow up the community now understands its importance.

**I: Is their counseling and access on ITN, sanitation and hygiene of pregnant and lactating women?**

P: Since our woreda is hot all households are using ITN especially it is given for women, adolescents and children. But when we come to its (ITN) utilization they are not using it properly which means once they used it after that they used for covering other materials therefore there is on consistent utilization of ITN. Regarding to sanitation WDA are especially working on the 16 packages of the health extension packages (HEP) in introducing to the community even though it is showing some kind of slowness. There is slogan regarding sanitation among the WDA “No women without keeping the sanitation of her compound”. Previously families were sleeping in the same house with their animals but now animals have their own separated class. Regarding to latrine utilization even though everyone has latrine in their home but it is not permanent latrine; sometimes they didn’t repair the toilet while it is risky to use it. There are promising changes in keeping sanitation and personal hygiene even though we could not say it is 100%. This change is due to health WDA who are focusing in advising PW and LW about environmental sanitation and personal hygiene like washing clothes and their body due to this she is now washing her baby and herself every week while she is changing her clothes. Even though awareness is created among the community but resistances and negligence are barriers to implement the sanitation and hygiene practices.

**I: Is their Vit A supplementation for PW, LW and adolescent girls?**

P: PW, LW and adolescent girls at school are getting Vit A supplementation whereas out school students are receiving Vit A in the community day even though we don’t have that much out school adolescent.

**I: Is their youth friend services for adolescents on Sexual and Reproductive Health in you woreda?**

P: Since we have family guidance office in our woreda together with youth affairs we have established peer to peer education in 13 schools. We will follow every three months on the peer to peer education by assessing number adolescents participated in the peer to peer education and those who really bring changes will be tracked and recorded. There is also girl to girl education and youth to youth education they discuss on different issues.

**I: What do they discuss during the peer to peer education?**

P: They discuss about how to use condom, delivery and they discuss a lot as well they have library to read and refer books. They learn each other through role play, drama, poem and scripts by showing the advantage and disadvantages of using different services. That is why we women affairs and youth affairs are working on adolescent.

**I: Can you tell me any change or improvement due to peer to peer education?**

P: The peer to peer education has its important in changing the attitudes of the youth through role play and drama. For example they bring one cases either on early marriage, migration or any other issues therefore they play it in drama or role play and the youth sense/feel it what is going on and what they must do to save themselves. This has brought youth to be friendly relationship with others without fearing therefore they can ask any advice or question without fearing. In the peer to peer education they discuss with adolescent girls about how to keep their menstrual hygiene to feel shame of it.

**I: In your opinion, which of the above interventions for the pregnant women are being implemented in an effective or less effective way? Why do you think that it is effective or less effective? In what way was it implemented?**

P: We can say we have brought change on awareness about ITN utilization; environmental sanitation, feeding practice, animals need separated class and latrine utilization. The awareness is created by the effort of WDA and HEW now we are in the process of improving feeding practice and we can say that we are doing well on this.

**I: What do you mean feeding practice is improving?**

P: For example there is demonstration on food preparation in the health center to the community. Even though the cereals are available in the house but they don’t know how to prepare therefore during the demonstration day each women expected to bring cereals powder of teff, sorghum, lentils and wheat; they give to the WDA and the WDA mix it and make porridge in front of them and eat the porridge together finally the women will realize that they can made in the same in their house by the available crops.

Even though we couldn’t say we are successful in feeding practice but there is promising changes in creating awareness that feeding is important and to avoid stunting; first the mother must eat balanced diet before, during pregnancy and during lactation and it must focus on child feeding since they are responsible in creating the next generation. When we advise pregnant and lactating women must eat extra meal this cannot be effective unless her children, husband and her family take the responsibility in following her to eat properly.

Our limitation is on peer to peer education since it has bringing changes in creating awareness among the youth we must expand this experience to other school but we didn’t do it therefore this is our less effective intervention.

In addition ANC follow up is increasing even though we cannot say most successful it is promising since previously pregnant women were giving births without having ANC follow up. We have brought change on the creation of awareness on institutional delivery importance now more than 95% are giving birth in health facilities and immunization of children have reached 100%. The community has perceived immunization is saving their children like to that of holy water. Our issue is why not institutional delivery becomes 100% even though WDA are getting tired this is due to our poor support to the WDA. General we can say there is good starting on modern latrine construction, feeding practice and sanitation but we couldn’t say we have done more on this area. Now in our office we have assigned person who works on women nutrition specifically how to prepare balanced diet (balanced nutrient) “Mitin”.

**I: What is the role of the assigned person on women nutrition?**

P: She is assigned by one NGO to prepared “Mitin” balanced diet from different cereals and crops they mix and grinded it together and add vitamins, sugar and salt into the powder by using the machine. The machine is given by the orthodox tewahido church as charity and the prepared powdered will be packed and sold 25 birrs per Kg to women it can be use to feed children, PW and lactating women and everybody can feed it. It has gotten good acceptances women are buying it since it is available nearby and demonstrated by making porridge to the women. When they compare the one food porridge in their home with mixed powder porridge the test and the nutritional content is far differ therefore they are buying and using it. Since the health post has full materials for demonstration during porridge making by the WDA.

In addition in one of our kebele there are 50 women model that uses vegetables from their home gardening therefore we would like to expand it to other kebele using audio visual for evidences to show to the community even though we haven’t done it till now.

**I: Is their targeted nutrition support for PW, LW and adolescent girl I your woreda?**

P: We don’t have nutrition support for PW and LW unless they are below normal according to their MUAC measurement they get fafa and oil. If the PW and LW are poor they can get support after we have screened if really she is poor but this is not given she is pregnant rather it is given due to she is poor.

**SECTION 4: Implementation challenges and Community factors affecting access to nutrition interventions**

**I: What are the challenges to implement delivering the nutrition interventions that we have been discussing for the pregnant women?**

P: There is no strong support from the administration even though sometimes there is initiation but no continuity. Once they give training or implement a program to solve the problem no one don’t ask where does the implementation reach in other hand no close monitoring and evaluation mechanisms. When we come to the community it is due to awareness and interest even though they have income they didn’t eat based on what they have for example they didn’t eat variety of food and may eat once per day even though there is enough food in the house this due to low awareness.

One pregnant must eat four times per day but it is not implemented this is again due to low awareness.

**I: What do you think the reason not changing their awareness?**

P: We have gathered religious leader whether their belief don’t allow them to eat during pregnancy or lactation but no food restriction for pregnant and lactating women during fasting. Health worker and WDA are strongly teaching them but I couldn’t say this is the problem it is due to low awareness; unless I lack to call the term awareness. Previously pregnant women were not recommended to eat egg, butter but now no food is restricted for PW and LW. The challenge is once the community get education they don’t implement it. The community is getting education on different issues for implementation the most resistance are those who perceived themselves as knowledgeable by assuming we are working for the sake of our development and the government not for their importance.

**I: Do you think the intervention you provide is convenient, quality and acceptable by the community?**

P: We believed that it has importance and convenient to the community that is why budget is allocated for it. Its importance is already approved by the scholars and we are also witness since we have seen changes after the children or pregnant women has taken fafa and oil they increase their weight therefore it is important. As well as the child who feed properly is growing well in terms of his weight and height whereas the child not well feed is become stunting and underweight. The community don’t perceived that the fafa and oil have consumed a lot of budget even though you can take the balanced diet which is sell by 25 birr Kg once they buy they don’t consider they invest money on it. The other problem is sharing of food the food which is given to the pregnant women is shared by all the family members. Due to sharing of food the pregnant and lactating women couldn’t see or appreciate the importance of the fafa therefore they simply conclude that eating the fafa or balanced diet don’t have any contribution to their health or weight increment.

**I: What are the challenges on implementation of maternal and adolescent nutrition?**

P: Even though we do have commitment to work but we lack to work with coordination specifically working on maternal and adolescent nutrition we are not strongly working. There is no strong relationship or structure which comes from the top to the bottom level that focuses on maternal and adolescent girl nutrition. But this doesn’t mean we are not working on maternal nutrition rather we are not reaching to what level we must reach.

We do have stream committee therefore the objective of maternal and adolescent nutrition improvement should be part of the stream committee therefore they can follow and evaluate the progress accordingly. The stream committee discusses on sanitation, water, maternal nutrition, agriculture and education fortunately the chairperson of the committees from women league therefore it is an opportunity to work on maternal and adolescent nutrition. But we don’t have any committee which works specifically on maternal and adolescent nutrition.

**I: What are the challenges while you try to give solution for the intervention of maternal and adolescent girl?**

P: What we have to do is to improve environmental sanitation, latrine construction and pregnant women must have 4 ANC till their delivery since they game is with life she couldn’t do nothing once she is affected her health now our plan is to bring 100% ANC coverage. This will help us in following the mother not to give birth in home by giving advice on the importance of institutional delivery. We can solve all this challenges if we work together with WDA, HEW, Kebele leader and administration body. Regarding to the community awareness must be created all what we have produced in the farm must be the family member before it go to the market unless we have enough production the same is true for home gardening, egg and hen before w e sell to the market we have to eat them. Even if some farmers are eating hen, sheep, goat, butter and egg why not all farmers are eating all this if they are available in the house.

The other area we have to work hard is sharing experiences from one kebele to other kebele must be our priority since we have promising intervention on home gardening and youth peer to peer education. In addition we have to go to actual site since visiting is very important for those who have good performance in terms of good food production, good child feeding and handling. We couldn’t improve all at a time but we can solve it gradually for example we can follow whether women are using or buying “Mitin” powder (made of different cereals and crop) in their home or not but honestly to speak we couldn’t address all this problems in our capacity even though the change form the past two years last year was better and from last year this year is better but no dramatically change is happened. We perceived sanitation and peer to peer education can be implemented in more even though it has its own challenges. Financial problem is one challenge in implementing peer to peer education and sharing experiences among youth, women and farmers.

Awareness creation for the youth is very important since they are the one to become pregnant, mother and father.

**Section 5: Multi-sectoral collaboration to improve maternal nutrition**

*This section is already addressed in section two.*

**Section 6: Other interventions that influence adolescent and maternal nutrition and health outcomes**

**I: In your opinion, why would delayed marriage (after 18 years) improve maternal nutrition? In your opinion, why would increase the space between each birth improve maternal nutrition?**P: Women are using family planning the success is just like to that of immunization success even if we couldn’t say maximum but adequate numbers of women are using family planning. When we come to adolescent girl they had history of abortion while they are in school this is due to less awareness. Especially those adolescent girls who get married are getting counseling on family planning to prevent unwanted pregnancy.

There are committees in the kebele to protect an early marriage they also give counseling on family planning in addition to health worker. Previously women were taking her contraceptive by hiding her husband but now due to WDA have thought the community they are taking their contraceptives without fearing their husband. There are different methods of family planning with may be taken daily, injection for three months and injection for three years. Now husband has taking his wife to health center to get her family planning. Regarding to early marriage we couldn’t say totally no early marriage but all marriage should pass through the committee of the kebele before its approval as well as they must bring health status confirmation. After all the evidence presented witness will tell her history when was she borne what is her age now or her priest who impregnate her testify her birth day if all this approve her age is 18 and above she will get married if not the marriage will be cancelled. The limitation of the committee were if my daughter is below 18 and they know me or have any relationship with me they may approved the marriage not to make me sad or dissatisfied by thinking of my investment on injera and “Tella” traditional alcohol. For this case why not all evidences are come from health facilities not only HIV test but age examination must be done by the health facilities if there is any doubt on her age. For example every year we sent at least five adolescent girls since we doubt their age is less than 18 for health facilities to check their age as well as we crosses checks her age with their education certificate. Previously our woreda were affected by high number of fistula due to early marriage but now we are counseling the community on the disadvantages of early marriage like uterine incompetency, fistula and complication during delivery and early marriage is reducing though rarely it may be happen. Home delivery is decreasing from time to time even though still there are women who give birth in their home. In this year we aborted nine marriages due to their age is less than 18 and have not interest to get married since they prefer to continue their education. To use this opportunity we were prepare a discussion panel for the adolescent girls to share their experiences why they refused the early marriage. Now we have saved them from early marriage but this not guarantee for them because they may married them by saying holy day “Tsebele” since it is very difficult to control home to home unless the community brings an awareness.

**I: Why do you think the communities don’t change on early marriage?**

P: When we come to religious if a woman is 18 years and above she may not stay virgin therefore to get virgin girl she must be less than 18 years and especially if her fiancé is deacon for example we have terminated one wedding for deacon since she is less than 18 year.

**I: Is that true adolescent girls are sexually active before 18 years?**

P: No evidences, it is the perception of the community that how she can protect her virginity whiles she is 18 years and this is ridiculous with no evidences and this is due to low awareness. But this is not the perception of all community there are family who strictly follow the age limited by the government by thinking to her daughter. Especially for the past four years due to the presence of the committee which approves the wedding almost early marriage is reducing but the religious perspective is still unsolved one.

**I: What do you think the solution should be to solve it?**

P: Educating the adolescent since no couldn’t force them it is very important to create awareness about the consequences of early marriage by strengthening the peer to peer education and we can fight together to reduce early marriage. Even if she is less than 18 year sometimes she becomes interested to have the marriage at this time we send her to social justice to approved or disapproved rather than going all this step the better one is to create awareness among adolescent girls.

**I: What is the importance of having delayed marriage and birth spacing?**

P: If she is less than 18 year since she is not fully developed or growth may not tolerate the pregnancy and the delivery resulted in narrowing of pelvic and its complications will be happened. Regarding it birth spacing I is very important to use family planning if she give births head to head (without spacing) first the mother will get difficulty to handle them and get malnourished as well as her children will not be feed properly due to the capacity or due to lack of time because of her busy work. When she is lactating mother for the second child she may face bleeding as well she is responsible of caring the other child in addition as we know our feeding practice is not good when additional child is coming think of the feeding practice definitely the two child become malnourished. If she give birth interval both the mother and the baby become safe and get well nourished since she has enough time to take care of her baby as well as her family.

**Additional renarks**

**I: Do you have any other comments on anything that we have discussed?**

**I: What lessons have you learnt regarding adolescent and maternal (pregnant, lactating and adolescent girls) nutrition at your level?**

P: I have learnt that maternal and adolescent girl nutrition is very important in keeping the health status of the women, children and all the family. Everything is in our hand but we couldn’t bring something change even though we have all necessary materials for example we have food to be eat but not eating well three to four times, we have variety of food but we simply eat monotonous food whereas we can use by diversifying them and can eat by increasing the frequency specially for PW and LW which don’t need excess expenses as well as time but this can be handled at less effort. Finally when I say such change is coming I will be glad by wondering if we do hard always there is change.

When we analysis what is going on about PW, LW and adolescent girl nutrition its has its own advantages and is advantages when you think of all this it seems easy but if it is not worked properly on the nutrition of PW, LW and adolescent girls its consequences is very dangerous but if it is well implemented the change that we see in the PW and LW is more beautiful (happy, smart and attractive).

**I: What lessons have you learnt regarding multi-sectoral coordination of nutrition in this woreda?**

P: I have learnt that working with multi-sector is very important since the effect can be increased or double especially if we work on the nutrition of PW, LW and adolescent girls. Working on adolescent girl is very important since they are the one responsible for getting pregnant, giving birth and caring the baby. Therefore as sector rather than running individually each sector should work together on the improvement of maternal and adolescent nutrition. If all actors and sectors have been discussed on the issues of maternal and adolescent girl nutrition as their issues we can bring dramatic changes.

I would like to recommend if this study need to bring changes about maternal and adolescent nutrition the implementation should don’t begin from the woreda rather it should starts from the top level to the woreda and must have close follow up and support and all sectors should be participate involved like agriculture, education, health, water, youth affairs and women affairs.

I: Dear Akeza thank you for your time and discussion, I have learnt a lot from your discussion thank you again. If you have any concerns you can contact me any time take my phone number (my phone number given to her).

Thank you very much for your time and information

**Summary**

Section 1:

- Stunting and underweight are common among PW and LW
- Overweight is not the community problem
- No food support for PW and LW unless they are malnourished and given fafa and oil
- PW and LW are not eating extra meal
- Heir is nutrition screening for PW and LW but no for adolescent girl
- Balanced diet “Mitin” is prepared and sold to the community by women league established by NGO
- Goiter is common in one kebele of the woreda
- Sharing food is common problem
- Women affair are more working with on maternal nutrition
- Multi-sector collaboration is weak in the woreda
- peer to peer education for youth or adolescents in the school
- institutional delivery is high
- 4^th^ ANC is very low
- ITN is well distributed but utilization is low
- They used iodized salt
- No strong support from administration office on financial and close follow up
- Lack of support for HEW and WDA
