# Supplementary material for: Barriers to utilize nutrition interventions among lactating women in rural communities of Tigray, northern Ethiopia: An exploratory study
Source: PLoS One. 2021 Apr 30;16(4):e0250696. doi: 10.1371/journal.pone.0250696 (PMC8087028; doi:10.1371/journal.pone.0250696)
Supplement: S2 File — (ZIP) [file pone.0250696.s002.zip › S2_File.Doc/Woreda level and above key informants/086_IDI_Agriculture extenstion Worker_Felege Hiwot Keble_Tankua Abergele woreda .docx]

**Operational research on Adolescent and maternal nutrition in Northern Ethiopia**

**In-Depth interview with Agriculture Extension worker**

**Introduction**

Thank you for your willingness and for taking the time to speak with me today. I have several questions to ask you that I have prepared in advance. If you have any additional questions or comments as we do the interview, please feel free to share them with me.

| **Section A: Interview details**   1. Zone: **South eastern** 2. Woreda: **Tenqua Abregele** 3. Kebelle: **Felegehiwot** 4. Name of key informant: **Miss. Worke Mamo** 5. Institution of key informant: **Felegehiwot Kebelle Agriculture Office** 6. Interviewer name: **Abate Bekele** 7. Date of interview: **15/11/2017** 8. Interview start time: **10:15AM** 9. Interview end time: **11:30:09AM** |
| --- |
| **Section B: Interviewee professional information**   1. Sex    1. **Female**    2. Male 2. Age: **22 years** 3. Highest level of completed education.    1. **College education**    2. Bachelor degree    3. Master’s degree    4. PhD 4. Current position: **Agriculture Extension Worker** 5. How long have you been in current job/position:    1. **___6___** Months    2. ______ years |

**I:** Interviewer **P:** Participant

1. **Common maternal (Pregnant, lactating women and adolescent girls) nutrition problems in the community**

**I: What are the common nutrition problems in the community for women and adolescent girls?**

**P:** I have stayed in this kebelle for short time and I don’t know many but within this time what I have seen is there is no water at all. It takes 2-3 hours to fetch water in this community. Due to this problem the community challenged to work on nutrition through irrigation. The community has no much knowledge on various issues related with nutrition. All groups of the community like youth as well as women have insufficient knowledge on nutrition particularly the lactating women, whatever we teach them for feeding themselves and their children they don’t practice it. They say simply “this is the food that we grown-up”. Due to this reason there are nutrition related problems.

**I: What are those nutrition related problems?**

**P:** There are mother and children who receive plumpy-net due to they are thin, there are so many children receiving plumpy-net. And, there are also many pregnant and lactating mothers. But all mothers don’t receive plumpy-net rather there are also women who are taking the so called targeted supplementary foods. There are also women who don’t receive supplementary foods due to shortage of it. The eligible women are identified by health professionals through measurement and weighting. The children with nutrition related problem are identified by weight while the mothers are distinguished by MUAC.

**I: Are you saying there is thinness in mothers and children, and how sever is the problem?**

**P:** Yes, I cannot say there many cases, but it is medium.

**I: Are there women who developed anaemia?**

**P:** So far, I haven’t noticed many, but I suspect because they don’t be screened there are many diseased pregnant and lactating women like fainted but it is not confirmed by laboratory rather it is my suspicion.

**I: Are there women affected by goiter?**

**P:** So far, I haven’t seen. As to my level of stay, there is no goiter in this setting.

**I: What are the potential causes of thinness in the women of this community?**

**P:** Women are given supplementary food for three month then after they will be discharged. But they all are not improved nutritionally at time of discharge. The cause of the thinness is shortage food, meaning primarily they don’t feed diversified foods, this community never knows about feeding diversified foods. In this area the endemic crop is only sorghum and the teff is cultivated occasionally. Therefore, they feed only sorghum. The other cause is lack of water, if they don’t get clean water they become diseased and then they become thin.

**I: Are there overweight women and adolescents in this community?**

**P:** So far, I haven’t seen.

**I: Are there diet related non-communicable diseases like diabetes, hypertension?**

**P:** No, there are no such cases.

**I: Are there women and adolescents who suffer from food insecurity in this community?**

**P:** Regarding food security, it is good they are secured. For example, in this area there are many goats, there is milk and it is good. Otherwise, they are not using appropriately though they have wealth.

**I: Are there stunted women and adolescents in this community?**

**P:** Yes I have seen, in this area there are cases of very thinness and shortness women. The adolescent girls they don’t learn as they will be married early in the childhood about 13-14 years as a result they are out-of the school. You simply look them as a mother with in their early age. Though my stay is short in this community, I have seen many things. There is again thinness and stunting in adolescent girls but it is not too much.

**I: How do you think women are especially at the risk of malnutrition you have mentioned above?**

**P:** It is because frequent birth; so in my opinion women are at risk of under-nutrition due to they only focus to feed and care there kids. That means to care and women are mainly devoted to feed their children so that they don’t have concern on their nutrition or health. So the spend most of the time at caring their kids and husband.

1. **Nutrition priorities in the community**

**I: What maternal nutrition (pregnant, lactating and adolescent girls) interventions are the priorities in this community?**

**P:** We are primarily giving nutrition and disease prevention education to improve health of women, adolescent girls, and children. We educate them to consume disease preventing foods, and to use clean drinking water. Otherwise, we haven’t done other than education.

**I: Why you are educating them?**

**P:** For example, if the mothers are pregnant to give birth of healthy baby and to grow healthy, and to prevent stunting and wasting.

**I: What are activities you have done for nutrition?**

**P:** We have demonstrated food from variety of food items to women and children by calling women. We have prepared porridge from variety of food items like vegetables and cereals. It is to educate them how to prepare the food from diversified food otherwise we haven’t done anything.

**I: What nutrition interventions have the most resources allocated to them?**

**P:** None of the interventions we have done are budget allocated. We have trained at woreda and by that knowledge we are providing education to the women. The training was given by Sustainable Under-nutrition Reduction in Ethiopia (SURE) program. All experts of health and agriculture were trained.

**I: Do you think it is necessary for your institution to get involved in work aimed at improving maternal nutrition?**

**P:** Yes it very important, because the baby of a mother will be healthy and competent. The health and agriculture are in line and they should work together. For example, if there were water, the agriculture sector would provide vegetables and crops through gardening. But, there is no water in this area so we are unable to provide such crops to the community to improve their health.

**I: How do you evaluate the priority given for the interventions for the women?**

**P:** There is slight improvement in education provision, especially if we have children or pregnant in our neighbourhood due to our advice they are practicing what we thought therefore there is improvement in the use of interventions. In my opinion, the education we are providing is good. Actually, we haven’t seen any change in the community. We have started to work with health extension workers since 4-5 months but the change is not satisfactory. The priority intervention to improve mothers’ health in our institution is nutrition interventions.

**I: Why it is important?**

**P:** Because maintaining the health of mother and the child she bears will be unhealthy and at risk if we are unable to care mother. Therefore the mother should be cared well.

1. **Nutrition interventions that improve adolescent and maternal health**

**I: What kinds of nutrition interventions are in place to improve maternal health in this kebelle?**

**P:** There is food which is given every month at 16^th^ day of the month, such as Targeted Supplementary Food (TSF). So this is an intervention to improve maternal health.

**I: How they access it?**

**P:** In this setting, it is very distant to get service especially for pregnant and lactating mothers. There is 2-3 hours far village and the area is too sunny, therefore they face several challenges and many of them don’t come to health post even if they get sick. They miss clinical visits due to the distance. Further, there is problem of road; therefore only those women who are nearer to the health facility receive the care.

**I: What has been done to make the service accessible for this community?**

**P:** So far, I haven’t seen any effort made to improve access. But we were expecting the health sector will do on it but nothing has done.

**I: What kinds of nutrition interventions are in place to improve adolescent girls health in this kebelle?**

**P:** Yea, we give education at kebelle level by preparing gatherings by using holidays like Sunday we provide education.

**I: What services are available for women at kebelle level?**

**P:** There are advices given monthly such as to make them monthly check-ups, to have appropriate feeding like consuming diversified foods, to enhance healthy wellbeing of their child, there is again nutritional screening for pregnant and lactating women and children monthly at center of the kebelle here to identify those who need food support or not, and it is not done through home visit.

**I: Do women are advised on nutrition sensitive agriculture?**

**P:** Yes we do but we know they have problem of water, but when there is rain at summer we advise them to collect the rain water. Otherwise, all mothers are advised to have good sanitation. They fetch drinking water from river therefore we advise them to boil it before drinking. They are also advised to use ITN and are using it since there is malaria in this setting and ITN is used to prevent it.

**I: Who advise them?**

**P:** All sectors working here including the agriculture and health professionals are also involved wherever we go, for example, at religious institutions.

**I: Is there any health service for adolescent girls?**

**P:** It is only advice. They are advised to support their moms while their mother gets pregnant, and if their moms have children advised to care the baby and to keep their hygiene, and to have clean drinking water.

**I: Which of the interventions listed above do you think is most important for pregnant women?**

**P:** In my opinion, hum … [Silence] it is nutrition because there are diseases caused due to lack of good feeding/nutrition, due to unclean water use and lack of monthly health check-ups. So, the service should be given through each village or home visit rather than the women go long distance to get service or we reduce the distance to for getting the service.

**I: Which of the above interventions for women are being implemented in successful way?**

**P:** The successful intervention is the health service because the community believed its importance. But this doesn’t mean they are perfectly successful but it is to mean the health service use is better than any other services provided at the kebelle.

**I: Why the health service is better/ successful?**

**P:** What has been done is the advice and education otherwise nothing is done special. And the community heard them.

**I: In What way they are delivering advice?**

**P:** For example, Sunday we all go to church since the entire community member present there and there are many churches at each village. So, there we are educating them.

**I: Do you think women nutrition has been done successfully?**

**P:** No, because this is the beginning.

1. **Implementation challenges and community factors affecting access to maternal nutrition interventions**

**I: What are the challenges to implement delivering nutrition interventions that we have been discussed for women?**

**P:** We planned but the bottleneck is primarily lack of water; there is no water ever …ever...ever… and never and it is too far and it is very challenging. And, if there is no water, there is nothing. And even for hygiene and sanitation it is so difficult. Therefore the first problem is water and water. The next is we cannot mobilize all individuals to educate them through community gatherings because of the distance. Therefore they give birth at home since there is no facility around the kebelle, as they should go to “Yichila city – the woreda capital city” which very far around 38 kms even from the center of this kebelle, but there are villages at 2-3 hours distance from this center. So think how much it is worth, if they experience signs of delivery until we call ambulance, she will give birth at home or on the way. Otherwise, we have adequate human resource that is committed to deliver the service.

**I: What are the challenges to implement nutrition interventions for adolescent girls?**

**P:** The first, some are the students. We teach them at school sometimes. But it is not that much done satisfactorily. For out –of school adolescents, mainly the health professionals teach them. They advise adolescents to support their pregnant or lactating moms and to support the mothers to have time to care themselves. Otherwise, no nutrition intervention is implemented to adolescent girls.

**I: How aware are the women and girls on the need to get the interventions?**

**P:** In my opinion, currently the awareness is good and there is slight improvement even if it is not big, there are few changes. For example, every household is using toilet appropriately. They have hand washing basin around the toilet. All households are again using ITN and developed habit of drinking clean water through boiling. These are reasons why I am said there is a change. Regarding nutrition, there is slight change. Actually, they don’t have adequate agricultural products, but some women (1-2) buy some products from market to feed their children a diversified food.

**I: Is there a relationship between educational status of women and access to interventions?**

**P:** Yes, those who are literate and illiterate have very big difference in accessing nutrition intervention. There are some women who are learning in grade 8 and these mother monthly follow their clinical check-up always better than illiterate, and they care their kids very well. Therefore, the educated and non-educated have very big difference in access to care. And these women (the educated) who I am said that buy some food items from market to fed diversified meal to their family.

**I: What community related beliefs and norms are preventing access to interventions?**

**P:** It is a feeding culture, for example, there is milk and many other products there are so many goats and cattle, so they can get much milk and they can get much meat however they don’t consume them rather they like selling. I have seen they feed only one food item like sorghum as they produce only sorghum.

**I: Are the interventions acceptable culturally?**

**P:** There are cultures that don’t allow some food items for women, for example, milk is not allowed for adolescent girl especially a girl who is not married because they assumed it makes her unmannered. This is what I have seen in this community at my first contact; females don’t drink “Ergo” (Yoghurt) because they assume she will be unmannered and said to be the girl doesn’t live in peace with her family and her husband. Whereas, after marriage she can drink but they like to sell it rather than consuming for own. Further, the women give priority for their husband, for example, if her husband is not around, the stew prepared cannot be consumed until his return that can be after a day which is very surprising, then the whole day either she will wait or she eat something without stew until her husband comes back that can be at dark night. If he has gone in the morning, she will wait until his return in the night. Therefore, this leads her to be sick and other diseases.

**I: What are you doing on nutrition?**

**P:** As I have said, I am an expert of crop production. The agriculture and health are always going in line. We are here always for nutrition hence to improve the community. Therefore we work with HEWs.

**I: What resources exist to provide nutrition interventions?**

**P:** There is adequate milk for consumption in this community even which is more than enough. But, there is inappropriate use. For example, a mother doesn’t drink milk for herself priority is given for the husband and her kids. Then she left as the milk is finished by her husband and kids.

**I: Is there FTC in this community?**

**P:** Yes, this is FTC itself.

**I: Have you tried to demonstrate the community on the production?**

**P:** At summer we had started to use irrigation as there is tanker to collect rain water during summer, but they community had simply observed but not practiced. I think we have tried to show them but I think the community didn’t accept it because they say it is difficult to cultivate other crops other than sorghum since they know and accepted only sorghum.

**I: What efforts have you made to change the attitude of the community?**

**P:** We have tried to convince, but since we didn’t produced much they didn’t accept it. Therefore, I am sure that they can accept the intervention if we show them and work rigorously on demonstration of crop production at FTC. We have also improved seeds.

**I: What other factors inhibit implementation of nutrition intervention?**

**P:** Nothing but the area is too sunny therefore it is difficult to call women to discuss together and service at one center in the kebelle. The sunny condition in turn prevented us to conduct home to home or to go to their nearest village to deliver service. Regarding transportation access, we have three villages among which one called “Hirsina” has no road it is even to go foot and motor bicycle.

**I: Can you tell me any solutions that your institution have applied to solve the challenges mentioned so far?**

**P:** To solve the barrier of road to teach through home visit, we use school to teach students then they take the lesson to their family. We have work to be done though yet it was not done, we had made an effort to construct road even that can be used by motor bicycle. So there is road which is under construction by community participation. There is a productive safety net program for pregnant and lactating women. Pregnant women are exempted from 4^th^ month of pregnancy to the 2^nd^ birth day of her kid as her husband will be delegated for her. For the work they will be paid 75 birr per day.

**I: How do you think need to be done to better address the challenges you have mentioned so far?**

**P:** If we teach by going in to the community, it will be better.

1. **Multi-sectorial collaboration to improve maternal nutrition**

**I: Do you feel it is necessary for your institution to work with other sectors/institutions to address maternal nutrition?**

**P:** Yes, because if we work together we can change the community and the mothers also can aware of everything like how to care themselves and their kids.

**I: Which sectors do you feel are necessary to work with?**

**P:** The health sector, the agriculture, school, administrative of the kebelle. The school teacher advises the son of mother and the student teaches his mother when he goes back to home. The health works on immunization to prevent diseases. There might be mothers who cannot access the service due to distance therefore the student who advised at school can support her to come to the service. As the administrative is assigned from the community – but we are from other areas – is important to convince people to have meeting and discussion or education as he knows the culture and community very well. In my opinion, he can convince the community very well.

The women affairs especially important for this intervention, because women affairs head can be pregnant or lactating, for example, we have a very clever, model women affairs head and she is very influential and very well-known up-to woreda level, she is learning grade 7 now, she is very clever and she is teaching her colleagues very well by gathering the women and she can convince them very well. Therefore she is very important to work together.

**I: How the women affairs head is delivering education to women in the community?**

**P:** She teaches within 1-5 women net-work. She works to make her network model. Through gatherings or home visit she teaches her network members. For example, if the household has child, she show them how to prepare porridge and other food items as she is well trained by us.

**I: Is there any committee to work on nutrition in this kebelle?**

**P:** So far, there is no committee. However, when we think to work just we call each other to work but there is no established network to work in team.

**I: For multi-sectorial action that effectively works to improve maternal nutrition at all levels, what kind of change in terms of the way stakeholders work together is needed?**

**P:** For future, in my opinion, we have to plan and work in a way that easily understood to the community like using groups to show comedy drama. We have to convince the community by educating together with other sectors.

**I: What opportunities do exist to promote multi-sectorial collaboration of nutrition in this kebelle?**

**P:** For example, we have school, they can arrange the date convenient for them to educate our community like on Sunday everybody can go to the community as it is not working day for us and we can get many people to teach on the churches. So, the presence of religious institutions is an opportunity. We have political leaders who supervise and evaluate us at 29^th^ day of every month and we all are here to discuss and work together. So these are opportunities. Further, there are livestock that are very important to work on nutrition.

1. **Other interventions that influence adolescent and maternal nutrition and health outcomes**

**I: In your opinion, do think delayed marriage (after 18 years) improves maternal nutrition?**

**P:** In my opinion, it is very important because if a mother gives birth after 18, she can care her child very well, she maintain her health whereas a mother who had birth at early age can face so many challenges as she herself is kid and has given birth therefore she gets challenged.

**I: In your opinion, do think increasing the space between each birth improves maternal nutrition?**

**P:** Birth spacing has very big importance. The baby and mother can have good health if there is child spacing because the mother give more focus only for current birth than the eldest child. If the births are not spaced, all the children are in the same age therefore she will face a big challenge.

**I: What programs or activities promote increased birth intervals in this kebelle?**

**P:** There are interventions through health extension package, for example, they use family planning that can be removed whenever they want pregnancy. So, this is the intervention to space birth.

**I: Can you tell me about any programs or policies in place in this woreda to prevent early marriage?**

**P:** In this regard, last year January actually I was not here but as my colleagues said “this is a time for many early marriages”. There were so many adolescent girl prevented. The work is done by students if the girl is student and then the police arrest them. There are so many girls arrested.

**I: Why it happened in January, is there any community culture?**

**P:** There is early marriage because the community assumes if the girl grows faster she may decide who to marry and may be left at road by the males they decided marriage. They have also concern as she may be pregnant. Therefore, they propose the girl for early marriage.

**I: Can you think of religious leaders have influence early marriage?**

**P:** The religious leaders assume early marriage as good culture. They again assume a girl shouldn’t do what she wants. Therefore, they support early marriage and assume early marriage is good. They think as the girl should get married before she knows something.

**I: In your opinion, are these programs or policies effective?**

**P:**  The women army can handle the issue of early marriage and prevent early marriage. And this activity is very good and successful therefore the incidence of early marriage reduced substantially. Currently, for marriage the girl is checked and her age also identified before marriage. But there are some who are making marriage in secret and nobody knows.

**I: What are the policy factors that affect age at first marriage?**

**P:** Currently, yes there is, the administrative has assigned three supervisors to oversee the situation in each of the three zones of the kebelle. Therefore these people control each village to prevent early marriage.

**I: In your opinion, are these programs or policies for birth spacing effective?**

**P:**  Yes, the women are using family planning and there is improvement than previous.

**I: Is there any community factor that prevents family planning use?**

**P:** There is religious assumption which states using contraceptive as sin. But such people are not many rather sometimes there is such assumption.

**I: Can you think of any other opportunities to prevent early marriage and increase birth spacing?**

**P:** The presence of women affairs in three zones of the kebelle, they control it very well. The second, if the girls are students there is also mechanism to trace them.

**I: What lessons have you learnt regarding adolescent and maternal (pregnant, lactating and adolescent girls) nutrition in this Kebelle?**

**P:** What I understood is; the problem the women faced so far is due to lack of advice and education. But I don’t know what has happened in the past. But I feel improvements due to advice and education.

**I: What opportunities do exist to promote maternal (pregnant, lactating and adolescent girls) nutrition in this woreda?**

**P:** For example, the church, the safety net program if the mother is pregnant and lactating she doesn’t go to work. Then she goes to use health service. It is a must and she should go to health facility as she is learning. They are strongly informed that they will be accused and become to work unless they receive the care. So in this way the mother is ordered to learn and receive service for her.

**I: Any other comments?**

**P:** No

**I: Thank you very much for you time and detailed explanations!**

**Summary**

1. **Common maternal (Pregnant, lactating women and adolescent girls) nutrition problems in the community**

- There is no water at all. It takes 2-3 hours to fetch water in this community.
- No irrigation to focus on nutrition
- Insufficient community knowledge on nutrition.
- They say simply “this is the food that we grown-up”.
- There are mothers and children who receive plumpy-net due to they are thin,

1. **Nutrition priorities in the community**

- We are primarily giving nutrition and disease prevention education to improve health of women, adolescent girls, and children.
- The priority intervention to improve mothers’ health in our institution is nutrition interventions.

1. **Nutrition interventions that improve adolescent and maternal health**

- There is food support (TSF) which is given every month at 16^th^ day.
- However, in this setting, it is very distant to get service especially for pregnant and lactating mothers.
- There is problem of road; therefore only those women who are nearer to the health facility receive the care.

1. **Implementation challenges and community factors affecting access to maternal nutrition interventions**

- “The primary bottleneck is lack of water; there is no water ever …ever...ever… and never and it is too far and it is very challenging….”. The next is distance
- The community has no adequate agricultural products
- There are also food taboos for adolescent girls

1. **Multi-sectorial collaboration to improve maternal nutrition**

- There is no committee of nutrition in the kebelle but they are working together haphazardly.

1. **Other interventions that influence adolescent and maternal nutrition and health outcomes**

- The interventions through health extension package promote birth spacing.
- From religious aspect, the religious leaders assume early marriage as good culture. They again assume a girl shouldn’t do what she wants.
