# Supplementary material for: Barriers to utilize nutrition interventions among lactating women in rural communities of Tigray, northern Ethiopia: An exploratory study
Source: PLoS One. 2021 Apr 30;16(4):e0250696. doi: 10.1371/journal.pone.0250696 (PMC8087028; doi:10.1371/journal.pone.0250696)
Supplement: S2 File — (ZIP) [file pone.0250696.s002.zip › S2_File.Doc/Woreda level and above key informants/097_IDI_Tequa Abergale_School Teacher.docx]

**Operational research on Adolescent and maternal nutrition in Northern Ethiopia**

**In-Depth interview with School teacher**

**Introduction**

Thank you for your consent to take part in this study and for taking the time to speak with me today. I have several questions to ask you that I have prepared in advance. If you have any additional questions or comments as we do the interview, please feel free to share them with me.

| **Section A: Interview details**   1. Zone: **Central** 2. Woreda: **Tenqua Abergelle** 3. Kebelle: **Feleghiwot** 4. Name of key informant: **Mrs. Trihas Hagos** 5. Institution of key informant: **Feleghiwot Primary School** 6. Interviewer name: **Omer Sied** 7. Date of interview: **15/11/2017** 8. Interview start time: **10:30AM** 9. Interview end time: **12:38:10AM** |
| --- |
| Section B: Interviewee professional information   1. Gender    1. **Female**    2. Male 2. Age: **22 years** 3. Marital status: **Single** 4. Highest level of completed education.    1. **College education**    2. Bachelor degree    3. Master’s degree    4. PhD 5. Current position: **Teacher** 6. Year of Experience: **2 Years** |

**I:** Interviewer **P:** Participant

1. **Common adolescent girls’ nutrition problems in the community**

**I: In your opinion, what are the common nutrition problems in the community for women?**

**P:** As you see in this area, there is no water. The community uses from pond and it is again not clean. For example, I pay 150 birr to have drinking water from the town. Therefore, there is no water no….this community, for example, the feeding is very challenging for pregnant women, as you see there is no vegetable. Even I myself only get vegetables at Saturday and Sunday from the market. Therefore, the pregnant women are highly suffering from nutrition including the feeding, dressing and they have also no awareness regarding feeding of pregnant and caring of children, here there is lack of awareness. I don’t know how to tell you….i have no words to the problem in this community…. [Sad]. This is unsuitable place for them.

**I: For example, are there women and adolescents who are receiving plumpy-nut and TSF in this community?**

**P:** Yes, for example when we see the TSF in pregnant or in children, the women of the community talk the providers/distributers themselves give the TSF appropriately. There are women receiving TSF and even there TSF food that brought by the organization called Save the children and even there was school feeding program last year for the students, the oil and flour and everything has come but the TSF flour that has been brought by the government for example, at the health center, the providers don’t distribute appropriately to the customers. There are rumours that the providers themselves use it. When we see the children issue, it is so surprising, they are very wasted, and the same is true for pregnant and lactating women. If the women fail to feed appropriately and fail to receive the necessary thing for her, the breast milk will be little and the child fails to get enough milk. So, it is challenging in this area. When we see adolescent girl in this area, primarily as I said there is lack of awareness. The community assumption is that, if the women eat or drink some type food, they assume there is problem. For example, when I see women who came to school, leave alone nutritious food, there are so many girls coming to class without having breakfast. When we see the center of our country, the girls from 11-15 years start to menstruate whereas in this community it is very surprising, I don’t know it is late. They will be absent from school and they lack awareness.

**I: For whom TSF has been given, is it given for pregnant?**

**P:** Yes, monthly but I have seen when it is mainly given for children. I think the plumpy-nut is also given for children. The TSF is also mainly for children. For pregnant….i am not sure, but most of the time it is given for children. The children will be weighted and assessed for their health status and they provide it mainly for children.

**I: Is it given for the lactating women?**

**P:** Yes, I was saying it is given for lactating. For children less than 2 years and mothers …mother... Maybe I don’t know.

**I: Are you saying the porridge is given for adolescent girls or is there anything for them/food?**

**P:** In school, the food support is given by the organization for all students. Last year …hum..., in this area there was drought, and there was no rain that is why the support is given for school. It was provided because to prevent school drop-outs due to hunger. And it was for all students and the TSF, oil and peanut were brought. Otherwise, it is not specifically for girls, I don’t know any support specifically given for girls so far.

**I: Can you explain the issue that you have raised like community supposition about food for adolescent girls?**

**P:** The community primarily has no awareness and awareness creation was not done. There are assumptions like if women drink tea they can menstruate and then they assume the menstruation as bad. In awareness, for example, the girls don’t eat food until the father, mother, and elder brother eat. Leave it…it is still too child. When you see still there is lack of awareness.

**I: Is there a problem like anaemia and goiter in pregnant, lactating and adolescent women in this community?**

**P:** When I saw in the class, the students asleep and they don’t follow me. Therefore, what I think is; the students are coming without eating breakfast. There are many special students in this school; one of the reasons for this is the feeding. I think it is due to they fail to fed appropriately during their childhood. As they didn’t receive food, I think they exposed to this problem.

**I: But what I mean is; is there a problem like anaemia and goiter in pregnant, lactating and adolescent women in this community? Just add some**

**P:** The anaemia, may be, I think the pregnant women have clinical check-ups at health center and most of them have check-ups there. However, unfortunately those who have given birth at home, I haven’t heard someone who died due to blood loss so far.

**I: What about eye problem meaning like night blindness?**

**P:** In pregnant women I don’t know, maybe I may not know the community well. This problem [hum..] could present. But I don’t know. A little bit... [Lough]

**I: What about goiter?**

**P:** There is no goiter even there is expansion of the use of iodized salt. So it is good.

**I: When you look women in this community, is there nutrition related stunting and wasting?**

**P:** The pregnant… [Silence]...I don’t know.

**I: Are the children stunted?**

**P:** When I guess the height, I don’t know….it is medium. But what I saw special, for example, there are student who have seeing problem, movement problem and …they are thin.

**I: Do you think this thinness and shortness is related with nutrition?**

**P:** Yes, highly. If they take the food appropriately, these problems might not happen. And when I see the students, many of them are either very thin or short or unable to see.

**I: You have said this is related with nutrition, how do you think?**

**P:** Because, for example, let us take goiter, if they fail to feed iodized salt very well, this exposes them to goiter. And the night blindness cannot be occurred if they feed vitamin A.

**I: Therefore, when you look adolescent girls in this community, how is their height and thinness?**

**P:** Majority are short in height, some are very tall, and I don’t know the reason. And in general, majority are short. For example, a 14 years lady doesn’t have anything when you see her physically; she is very thin and short.

**I: Are there overweight women in this community?**

**P:** I don’t there are overweight women rather there are underweight. There are women who are very underweight. In adolescents, there is early marriage. I am 4^th^ grade teacher, there is one girl who is very fat but I don’t know the reason. She is very small and her age is 13 years. But she has been married but when I ask since I suspected her as pregnant, I asked her whether she uses contraceptive or not but she responded as she didn’t use contraceptive and told me as she is not pregnant too. She is too fat. Therefore, there are many thin than overweight.

**I: Are there non-communicable diseases such as diabetes and hypertension?**

**P:** No, no, I haven’t ever heard these.

**I: You told me as women is prohibited to drink tea, are there any foods banned for women?**

**P:** Yes, for example, the tradition is not yet broken. The women and children eat the left over foods from other family members. I don’t know why this is practiced, but if the women eat hot drinks the community assumes as they are pushed to misconduct such as sexual excitement. Therefore, women are not allowed to take hot foods.

**I: Is there any food insecurity problem in your Community?
P:** The food security varies across the households. But they have shortage even to fill their stomach, for example, there is sesame that the students bring to school, after weighing the sesame when we give the left-over to them it is very surprising, they eat it. Therefore, I think the children are hungry. Therefore, let alone balanced diet, even there are many students who come to school without eating breakfast.

**I: Who is mainly affected by the nutritional problems that we have discussed so far?**

**P:** In my opinion, children are mainly affected. When we see pregnant women, I don’t know whether they adapted the environment, the pregnant women are better while they are working very hard work. But children are mainly affected. When you look them you will be sad. I think the lactating mother has under-nutrition. Because the lactating women need food for herself and her child, she gets nutritional problem. But I don’t have information about adolescents. Pregnant women eat is known, the main problem is lack of food, rather then they prefer to feed their husband or children. They have limitation food supply to eat for themselves.

**I: Can we say women in this community are stunted?
P:** I don’t know but majority of pregnant women are short and thin.

1. **Nutrition priorities in the community**

**I: What priorities do your institution has in relation to maternal and adolescent girls’ nutrition?**

**P:** There is education about nutrition. It is only delivered by science course; you will teach how to feed pregnant women. There is education related to menstruation. It is only education that could be related with nutrition. For example, when we teach about women nutrition, it is not that much extensive because I think we are also having poor awareness.

**I: What programs are in your school?**

**P:** It is teaching and learning.

**I: Is there any work to improve nutrition, for example, school feeding?**

**P:** It was done last year, it was started around October…it was started on May then the school was closed at June and then the program ended.

**I: What is your institution doing for adolescent girls?**

**P:** Nothing rather teaching and learning.

**I: Do you think it is necessary for your institution to get involved in work aimed at improving maternal nutrition?**

**P:** Yes we can, it was due to lack the awareness but we are ready to get involved if we have awareness. For example, there are health professionals working at health center. If the awareness is created for them, this helps us to handle the adolescent girls collaboratively. For example, the adolescent girls then teach and create awareness to the community. For this collaborative work, as to me I am willing. At schools, we can create awareness primarily.

**I: On what awareness?**

**P:** Nutrition, for example, for pregnant, lactating and adolescent girls. For these, if we create awareness on how to feed, how to take rest and others, the rest will come easily.

**I: On which way can you support pregnant and lactating women?
P:** For example, through home visit we teach how to feed and counsel her to go to health center. In her home, there is balanced diet, but there is problem in how to take it, therefore we counsel her how to receive food.

**I: With whom do you think is better to work on women nutrition?**

**P:** For example, teachers with school directors, with students/the student parliament, there are also school supporters called “womeh”, farmer association, women association, and women affairs and women league. For lactating women, we teach her to feed herself appropriately, for example, before the six month to avoid extra food children, the cleanliness and dressing and feeding breast milk until 2 years, to introduce additional food at six month. And therefore, we create awareness how to feed her child at six. The mother can be educated about the child breast feeding and produce adequate milk.

**I: What can you do with farmers associations and the like you have mentioned so far?
P:** The school, if we create awareness for students that does mean transmitting some information, and then it reaches each and every household because students are from each household. Therefore, if we educate these students and then go back to their home, for example, if the members of the women association get knowledge on nutrition it reaches all women. Therefore, if we work at schools, it reaches every household.

1. **Nutrition interventions that improve adolescent and maternal health**

**I: You have said there was school feeding last year, but can you tell me what are you doing to improve adolescent nutrition?**

**P:** The school feeding, they themselves have studied it since there were students who were absentees from school due to hunger and there are also students who faced various problems due to feeding problems. By assessing, the students who were absenting from class at the community, and the support is sent us through organization. It was started due to the drought.

**I: Do you have any counselling and education session for pregnant and lactating women?**

**P:** We create awareness at school for women through students. By students for example, for those who are 5-8^th^ grade and who have reached the adolescence by gathering them we create awareness on how to prevent pregnancy. For example, I in my house, I tell whenever I have got women on disadvantages of higher birth. But their husbands don’t allow them to use contraception. Otherwise, I counselled two women last year, they have received contraceptive. So, the work is by individuals’ interest rather than in collaboration.

**I: What about advice on water services?**

**P:** In this community it is hard to get water. For example, from my rent house, as the owner said to me, it takes 4-5 hours to fetch water. To fetch the water they go with their kids on their back, the water is again highly contaminated, no water tape in this area, and I myself buy water if I want to drink. But nowadays, by creating awareness some families are using Wuha agar “water treatment drug”. Otherwise, water is very… very... very challenging.

**I: Is there Vitamin A supplementation at school?**

**P:** Yes, even yesterday, we have received two drugs the first is the eye one. The other is for the intestinal parasite. Therefore, we have received the two drugs at the school.

**I: Is there any food support for pregnant and lactating women in this community?**

**P:** It is TSF. Yes, it has given but mainly given for children. However, it may be given for women. But there is TSF. But I don’t know whether it is given for lactating women, I may ask somebody who knows.

**I: Are there adolescent girls who were out of school?**

**P:** Yes highly

**I: So how can address them, do they have association, for example, youth?**

**P:** There is an only women affair.

**I: Is there youth association?**

**P:** It may be, hum.., there is only the women association.

**I: Which of the above listed interventions do you think is most important for pregnant women?**

**P:** Yes, because for example, if we teach adolescent girls about contraception, it would be very important. We teach them about contraception, for example, if you ask the students the definition of family planning as giving birth of two children. We have to tell as it is not giving two births.

**I: In your opinion, which of the above programs are being implemented successfully (i.e. in the most effective way?)**

**P:** Hum….there is counseling about family planning at health facility during antenatal care by the health professionals. To be successful, from where did the student get feeding. If there is feeding program, it would be good. But if we tell the student to take home, the student may sell it at market. But it is better, if it is cooked at schools. For example, there was TSF that brought last year, but because lack of money paid for daily laborers, they request 10 birr per student; I don’t know why they are doing this which surprised me. For example, in a school if there are 8 students, they pay 80 birr, the community complains why this important as we are paying 80 birrs. The students themselves again complain to refuse the payment as it comes from higher level. Otherwise, school feeding is good, but this problem should be broken. The supporting agency should pay for the daily laborers though it could be difficult.

**I: So, which intervention is successful?**

**P:** When I guess, the change has come is on the utilization of family planning. Lack of knowledge of professionals about nutrition is a challenge for nutrition intervention. For example, a kid goes to take immunization they don’t know where to inject the vaccine, just they don’t know and they do simply traditionally. The government should work on the competency of graduates.

1. **Implementation challenges and community factors affecting access to maternal nutrition interventions**

**I: You have been telling me as there are food taboos for women, can you tell me the food items prohibited for pregnant women?**

**P:**  May be I don’t know about pregnant mother, but there could be taboo. For example, I think if the pregnant women drink alcohol like “Tella” – a locally prepared alcohol, they believe she will be harmed. As to my information if the pregnancy is greater than 4 month, the alcohol intake shouldn’t be high. But for example, if I don’t mistaken, the pregnant mother shouldn’t eat the banana, and egg. If she eats egg, it has its own impact.

**I: Can you tell me food taboo for lactating women?**

**P:** I don’t think so.

**I: Can you tell me food taboo for adolescent girls?**

**P:** As I said before, the adolescent girls primarily shouldn’t eat hot foods. The hot things….hum… Hum and if they took very sweetly foods, for example, they shouldn’t take sugar. If a girl takes sugar, assumed to be she will become fatty and others. Hum …hum... For example if she took hot, the mensuration will come too early. Therefore, they think as she is too child.

**I: How aware are the pregnant women on the need to get interventions?**

**P:** If there is service, the community or pregnant women use it very highly. Even it is surprising, sometimes they got excited as they here about the interventions. So, they miss the service due to lack of awareness rather they receive interventions very highly. The entire community can receive the whole care but there is awareness.

**I: How aware are the lactating women on the need to get interventions?**

**P:** Similarly, the lactating women when they are told about child care like washing and feeding, just like grade 1 student as they are illiterate they simply take the whole education provided for them. Therefore, if we create awareness the community can use the service eagerly.

**I: How aware are the adolescent girls on the need to get interventions?**

**P:** There is awareness problem, for example, when you tell about feeding and how to prepare food and use iodized salt after the stew has already ready, if they are told, they receive the education as a whole but they lack awareness. It is due to lack of awareness otherwise they receive any intervention. Regarding adolescents when we teach about how to feed a woman, cleanliness, and others …I don’t know.

**I: Good! Is there a relationship between educational status of women and access to interventions?**

**P:** It is very different in educated and uneducated women because for example, if a woman educated, if she finished grade 10, for example, if she marry and live in the community and the woman who is illiterate even who didn’t yet learn grade 1 have very different access to interventions. The woman who completed grade 10 at least, she has some awareness whereas the illiterate cannot have anything even she will be challenged to communicate with people because she doesn’t have confidence to know even for foods and other issues. Therefore, I would say there is high difference. But the problem of this community is, for example, uneducated are many than the educated one even those who said educated are for about grade 3 or 4 or 2. But, majorities are uneducated and married women in this community.

**I: So do you think literacy has importance for service utilization?**

**P:** It is very surprising, when they see us; we teachers are 22 or 23 years of age and the community is so surprised and says “we have to learn”. For example, in the rent house where I live now, a mother has 3 children, here child has reached grade one but in the past I think she was educated grade 2. She says “we should learn”. Therefore, they know the importance of education. The educated and uneducated have a big difference even to lead their home more than the husband the woman is highly involved. But the uneducated can do nothing as she doesn’t know anything. Therefore, education is very important.

**I: As a community, what community related beliefs and norms are preventing access to interventions?**

**P:** Regarding feeding, the women don’t eat with their husband even the food prepared in their home. The children eat after the husband/father eat and feels full. Similarly, the woman also eats after her husband eat. Regarding women, what surprised me is, the girls are totally prohibited to eat with their mother and father. They don’t eat…they eat alone and even after the father and mother have reached their maximum fullness. For example, if the stew is prepared then if there is no husband, no one can touch the stew, it is forbidden and it is only after he has eaten. But nowadays after the awareness has created, sometimes if the child cries they are giving stew for them even though there is no father around. In this community, when I compare the adolescent girl and pregnant woman, as mother is very humble so that she may prioritise to give food for her daughter than herself. So, the pregnant and lactating mothers are highly harmed.

**I: Are there community related beliefs and norms are preventing access to services provided at public health facilities?**

**P:** Yes, for example, last year, the eye drug given for us, first it is told as the children should eat food very well in the morning because if they take the drug in empty stomach it harms them because if they told us the drug is very dangerous. The students may lack food at their home, after taking the drug they become weak then they said is it to kill that our kids are given this drug. Finally, the students assumed it is to kill them as I heard from the students at the moment. While I say it is a drug, they refuse it as it is not drug. And regarding family planning, more than males the women are aware very well about its use. For example, a woman is so tired of high number of birth and she goes to health center and takes contraceptive secretly without informing to their husband.

**I: What other community related beliefs and norms are preventing access to services provided at public health facilities like prohibition of health care for pregnant woman?**

**P:** In the past, the pregnant woman don’t go health center alone, there was home birth but nowadays, health facility delivery is highly utilized. It is well performed than other health sectors achievements. It has improved very well. From the governments’ programs that I believe that isn’t solved is only early marriage.

**I: We will come to that, but now please tell me about community norms that hinder the women to receive health care?**

**P:** It is not that much. Even they are well aware of services related with their health and it has expanded. When you give them the drug, for example, if you tell them how to take, they apply it. Otherwise, I don’t think there are such problems.

**I: Are the interventions acceptable culturally?**

**P:** Last year, I had training; it was organized by Axum University. When we ask them during home visit, many women raised an issue about the health center. They say as visiting the health center is dangerous. They prefer to go “Yichila” – the woreda city” but here there are also professionals. The user fear, I am so surprised. For example, the health worker are absent from their work. They go Yichila town and the likes. So, there are limitation in the health center and the drug supply. For example, when the contraceptive users go to use the service, if I don’t mistaken, there is nothing more than the injectable. It might be to their level. And rather than going to Yichila to get service, it would be better to access the service here. If possible, these challenges should be solved.

**I: For example, the people may to complain about the government but don’t use the service, therefore, are there any such hidden issues regarding about service utilization?**

**P:** For example, pregnant women in each household there is ambulance telephone which s very good work. But the problem is, for example, a woman to talk as there is no health worker at health facility, she cannot talk. Otherwise, they talk in secret. For example, if you ask her, she may tell you as everything are fine. It might be because of lack of education. If they talk the problem today, they feel as a big problem will happen in the future. I think they think so. Therefore, there is failure to talk. The woman is not allowed to talk at sessions. She simply cooks food at her home. And even if she is out, that is for fetching water. This is the problem.

**I: Are the interventions accessible for women and adolescents in terms of cost like you have mentioned above about school feeding?**

**P:**  For example if we have 600 or 800 students, I don’t think the money paid for the labourers is appropriate. We should talk why this happened but it is our weakness. Last year, it was dangerous for students because if the food support has sent, why the students contribute money. Therefore, some of the students were saying rather than paying money it would be better for them to work and eat. They were saying it is difficult to pay money; rather it is better to work and eat. When I was going to class to ask whether they have brought the money, it was too challenging for me, I was feeling even to pay by myself but I cannot afford. Every morning you come to spar to collect the money. This was not good.

**I: Let us come to women; are the interventions accessible for women in terms of cost?**

**P:**  Yes they pay, for example, when the ITN comes to the health center, the community pays 15 birr for it. This is due to the community is well aware of its importance. They think 15 birr as simple and even can buy about 5 ITN for his family. We have a community that is easily changed if we give them awareness.

**I: Let say the ITN is provided; for whom the priority would be given?**

**P:**  The priority should be given for pregnant women and children. They have to be told to use it appropriately. Following them, the community as a whole should use especially those who are living in malarias area. This is what I know.

**I: Are the interventions accessible for women in terms of transportation?**

**P:**  Going to the health center is not as such difficult. But as I heard, they simply say “whether we go to the health facility or not, nothing will happen to our health”. For example, a pregnant was gone to have ANC check-up, and then after the health professional/female/ there asked about the date of menstruation, the woman said why she asked me in front of other clients, what can I say “shall I say before the other clients or what?”. She told me this. Rather they prefer to go Yichila town, actually it would be far for them. To go this health center, it is not far but the doubt the competency of health professionals there. The community worry to go there. I have heard from 3-4 individuals, complaining the health center as no service there. There is a village called Mishaza which is far. There is transportation but may be they may lack money. So, they simply come on foot to use service. Actually, I am not sure, for example, a pregnant woman after she confirmed her pregnancy, I am not sure whether she comes back for the next visit or not. However, it is recommended that a pregnant woman should have at least 4 visits till the 9^th^ month. But I am not sure whether women are using this.

**I: So, can we say the services are suitable for community?**

**P:** Yes, because they are very important. If you tell them about visit for women and if they have awareness, they can tell to their children even. For example, if they go to the health center, they can know what the women should eat even the tea.

**I: Now you have told me its importance, are the interventions acceptable by the community?**

**P:** Yes, in this regard there is no problem. Even the community believe that the government don’t bring something that harms us. They assume as the government brought the intervention since it is good for us. The community knows well. They think as the government has brought the intervention to protect them. For example, if they are told to construct toilet, they were not using it in the past but now everything I clear for them and they are aware. Therefore, they know as it is important.

**I: How do you explain the quality of interventions given for women, and adolescent girls?**

**P:** I have mentioned before. Regarding the vaccine they are taking, as I heard from 2 clients, they complain the health professionals as they don’t know how and where to inject the vaccine. Even they report some individuals who paralysed due to inappropriate injection of vaccine. There are still such cases.

**I: What about the professional ethics of the providers?**

**P:** Ethically, I haven’t yet visited them. From what I have heard, the first is; there is nothing at the health center. Even the professionals are there, they say “this is not the time to visit health facility, just say this is lunch time and I should go to my home”. Then the clients get shocked because they are illiterate. Even they don’t want to visit there anymore. Therefore, they have worry.

**I: What is the impact of end of the school feeding program?**

**P:** It was important but it would be also important to if it also given for children out of school too.

**I: What are the limitations of the interventions provided by health facility for example like lack of adequate professional, drugs?**

**P:** Actually, there are no all drugs there but when we ask the water treatment “Wuha Agar”, the community complains as they give for those who they want. They don’t distribute it in right way.

**I: What do you think needs to be done to address the challenges you have mentioned so far?**

**P:** The first, starting from myself should be involved in creating awareness at school. For health professionals, they have so many trainings but I don’t know what they have trained and I don’t understand. Therefore, the health professionals should also give training and care their customers with good face. For example, if we kick the student, they won’t come to school. Therefore, the professionals primarily should correct their conduct. They have to know what to be talked with clients and what to communicate. And they have to keep the information secret and communicate to the clients only.

**I: What solutions that your institution has applied to solve the problem you have mentioned?**

**P:** No, it was my understanding that I have been spoken to you.

1. **Multi-sectorial collaboration to improve maternal nutrition**

**I: Which other sectors do you feel are necessary to work with your institution to address women and adolescent nutrition?**

**P:** There is the so called team charter; it is an agreement to work together. For example, in our school is an agreement made between the health, police, and the kebelle administrative. For example, since we have agreement with health, therefore they should come and deliver the professional related tasks at our school. The same is true for police office, if there is misconduct in school or if we need them to teach about ethics and law very well we invite them to our school according to the agreement we had. Therefore, we have such integration. Regarding nutrition, if we have opportunity like that of last years, the school feeding program, for example, if we need their support and even how to prepare the food we have also agreement with them. We can also work with the agriculture sector. The agriculture educates the community on how to cultivate crops important for nutrition and how to utilize the products. Even everything that we were discussing about the women and adolescent nutrition are products of agriculture. If we work with women affairs we can teach the women especially how to feed the pregnant and lactating women, and how to keep clean the prepared food in detail. I have learnt so many things from your interview and such information would be better if training is given. Through nutrition trainings, for example if we need to create awareness at school, if one teacher has training then he can expand.

**I: Do you have multi-sectorial collaboration?**

**P:** Oh, it is not that much. It is due to lack of collaboration this problem occurs.

**I: What do you expect for future?**

**P:** We ourselves even have aware now, we didn’t know it before. But through time everything has been changed. Therefore, we have aware, and will create awareness for others in the future. Previously, there was nothing done though it is important to work together. By the way, collaboration is important, if we work together if we share what they know for agriculture, we can help them. Therefore, collaboration is very important.

**I: If it is important,** **what kind of change in terms of the way stakeholders work together is needed?**

**P:** To work strongly, primarily, the two of us should create awareness through suitable means. It there is no awareness created, for example, it is the only health that has a little bit awareness about nutrition. For example, when we come to education, the students absent from school however there is no training given about nutrition. But, at the health sector it is better. If we work together, we will be successful.

**I: What type of resistance to the needed change do you perceive or have you experienced so far in working together?**

**P:** First, failure to work based on the training we have took, for example, sometimes when we go to take training the 4 days training might be given with in a day that means we didn’t trained yet. So, such challenges might be there during trainings.

**I: You have mentioned as there is team charter, does it has nutrition related interventions?**

**P:** Regarding nutrition, it is not that much rather we are working with health sector.

**I: So, what works are done according to team charter?**

**P:** With health center professionals, for example, if the food support comes to the community, they show how to prepare, and how should women to be feed. It is just for talk only but in practice it is not done.

**I: So, how can multi-sectorial collaboration especially on nutrition would be strengthened?**

**P:** The education disseminated by radio is very good one. Therefore, such media information dissemination is very important. For example, we should not differentiate what women and men do. Let me tell you which is done at higher level, for example, the advertisement for “Areal Omo soap” is done by women but why don’t men do this. On the other hand, the advertisement for painting house is done by men. If we change such culture, everything will be changed. Therefore, if we correctly advertise on Medias and if we use them wisely, this can be changed.

1. **Other interventions that influence adolescent and maternal nutrition and health outcomes**

**I: In your opinion, do think delayed marriage (after 18 years) improves maternal nutrition?**

**P:** If there is early marriage, it highly affects the health and nutrition of women. If a girl has made early marriage, she has not been matured in her body structure. If she is 13 or 15 years, there are several harms to her body. Regarding her nutritional status, she didn’t yet fulfil her need. For example, early marriage exposes her to fistula. And if she give birth at 13, as she is still too child and it is surprise for me, she don’t know how to feed her child and she also become mentally ill because she herself cannot lead herself is going to care her baby. It is difficult for her as she is adding another child to her childhood life. Early marriage is one of the problems that are not yet solved. Due to this, there is disagreement with their husband. If she is 13 years and he is 25 years. So this is a problem highly practiced in this community.

**I: In your opinion, do think increasing the space between each birth improves maternal nutrition?**

**P:** It is important. If a mother has used birth spacing; she will be free and don’t worry and it is important for the household wealth. But, if a mother gives birth without spacing, the children cannot have adequate food and leads to many other problems. In this area, many problems are happening due to failure to birth spacing. As to me a woman should give birth at least at above three years gap. And again, it should be in line with our wealth/income. But, if they have adequate income and if the mother is the one who feed very well, she may not be harmed much due to birth. But, if the pregnant woman fails to feed appropriately, for example, if she gives birth in 2 years interval, she will be harmed badly. She develops physical harm, and will be exposed to various diseases. And, a woman should marry after 18 years and it is a must.

**I: What programs or activities prevent early pregnancy in this community?**

**P:** It is too late, currently it the awareness creation that is done. In schools, it is one of the top issue and we all teach and even go to visit. So, we always educate our students on the disadvantages of early marriage. But, the community creates system on the early marriage I don’t understand it, for example, a girl at grade 4^th^ should drop her education because to make the marriage secret and to be free of accusal. The community is very active in such misconduct. Therefore, this is a very big problem in this community.

**I: Is there any legal considerations that prevent early marriage?**

**P:** It has started last year. Around the November, what we have started now is, majority of such marriage is conducted in January. So, if the girl absent from school, we create awareness. The parents know the consequences of early marriage but I don’t know why they are doing this. If marriage is done, we will inform for police and women affairs. In 2008 EC, there were a grade 8 girl, actually I was not here at the moment, and everything was made ready to marry her, she is very cleaver in her education. Therefore, at the end she informed to school and her marriage was cancelled. Therefore, these all issues are happened due to lack of awareness.

**I: You have said as the prevention of early pregnancy is done by police, and others, but what should be done to eliminate for good? Or what additional works are needed to stop this problem?**

**P:** They know everything like politics and others, but creating awareness is important as to me. They know as it is an evil action. So, we have to work to create awareness through various means like drama. It is only through awareness creation that we can bring change. For example, at schools we should teach or at health center it has to be given in wider form.

**I: They have awareness as you said but they are not practicing it well, so how can we make them to practice?**

**P:** Because the community believe in small things, we have to tell them the real examples of consequences of early marriage. Using Medias to share the experiences of individual victims is very important. It would be difficult to go through religious so we also work with religious leaders.

**I: What programs or activities promote increased birth intervals in this community?**

**P:** It is good; they can get from the health center. But, utilization is still poor. For example, women are mainly receiving injectable contraceptive. Therefore, beyond the injectable, there would be other type like loop, and implant. Rather than provision, the education should be provided regarding the benefits and harms of contraceptives.

**I: What additional works can be done to increase birth interval?**

**P:** As birth spacing has importance, they community has aware of that now. With this last two years, it is good, and the ambulance was coming to the community twice but currently we are not seeing the ambulance. Therefore, the birth is reduced. The awareness about birth spacing is expanding.

**I: Why the community need to allow early marriage?**

**P:** Because, they assume the marriage as a good thing. For example, if a household has no married girl yet, they perceive as something is said by their neighbours or the girl is failed to marry due to something happened in her life. In general, it is due to perception. The parents perceive as their daughter left unmarried.

**I: Why the urge to marry their daughter before age of 18 years?**

**P:** The first reason is failure to start education at their age meaning at 7^th^ year. If they fail to start education at their early age, they will be forced to marry early. And, if she goes to school to higher level, the parents perceive as they will marry there thus they are unmannered.

**I: Why the community needs to have many children?**

**P:** Because they believe children as wealth. They think it as gift from God, therefore they say “why we need to use contraceptive”. For example, they perceive using contraceptive as sin. I think they believe the work load will be solved if they have many children. Even they use child labour they make their children to keep cattle.

**I: Can you tell me how to improve the birth spacing and early marriage in this community?**

**P:** Everyone should work together. For example, the service is given at health center; therefore I haven’t to say it is the only work of the health professionals. Therefore, we all should be strong and work.

**I: In your opinion, are these programs or policies for birth spacing and effective?**

**P:** Yes, it is important work. First, the people trained and working. Presence of educated personnel like you is also good to make effective.

**I: Can we improve it by educating at school?**

**P:** Yes indeed! Even it is through school in which much of works can be done. And everybody should teach at every community gatherings. Using some strategies like community gatherings to take some support like ITN distribution as all the community presents to take such support is important to disseminate information.

**I: Any additional suggestion to add?**

**P:** I have said everything already, but what I want to say is: I admire nutrition; there is change since the beginning of this work, so everyone including us and the government should work to expand this issue because it highly beneficial to our country. So, we have to work hard together.

**I: Thank you very much for you time!**

**Summary**

1. **Common maternal (pregnant women, lactating women and adolescent girls) nutrition problems in the community**

- There is no water.
- There is no water no… Therefore, this community, for example, the feeding is very challenging for pregnant women, as you see there is no vegetable.
- There are women receiving TSF but mainly it is given for children

1. **Nutrition priorities in the woreda**

- There is education about nutrition. It is only delivered by science course.

1. **Nutrition interventions that improve adolescent and maternal health**

- There was school feeding last year, because there were students who were absentees from school due to hunger
- We create awareness at school for women through students.
- It takes 4-5 hours to fetch water for women.

1. **Implementation challenges and community factors affecting access to maternal nutrition interventions**

- There could be food taboo for pregnant women.
- The pregnant mother shouldn’t eat the banana, and egg.
- Hot foods are not allowed for adolescent girls.
- The women don’t eat with their husband even the food prepared in their home.
- Girls eat alone and even after the father and mother have reached their maximum fullness.
- Women are also prohibited to eat stew during their husband is not around.

1. **Multi-sectorial collaboration to improve maternal nutrition**

- There is the so called team charter; it is an agreement to work together.
- The collaboration is not that much
- The way of advertisements in Medias shouldn’t be gender based, for example, soap is advertised by female whereas painting house is by males.

1. **Other interventions that influence adolescent and maternal nutrition and health outcomes**

- Early marriage is a big problem in the community
- The community assumes the marriage as a good thing.
- The parents perceive as their daughter left unmarried.
- They believe children as wealth.
- They perceive using contraceptive as sin.
