# Supplementary material for: Barriers to utilize nutrition interventions among lactating women in rural communities of Tigray, northern Ethiopia: An exploratory study
Source: PLoS One. 2021 Apr 30;16(4):e0250696. doi: 10.1371/journal.pone.0250696 (PMC8087028; doi:10.1371/journal.pone.0250696)
Supplement: S2 File — (ZIP) [file pone.0250696.s002.zip › S2_File.Doc/Woreda level and above key informants/114_IDI_WomenAffairs Head_MedabayZana woreda.docx]

**Operational research on Adolescent and maternal nutrition in Northern Ethiopia**

**In-Depth interview with Head of Women Affairs**

**Introduction**

Thank you for your consent to take part in this study and for taking the time to speak with me today. I have several questions to ask you that I have prepared in advance. If you have any additional questions or comments as we do the interview, please feel free to share them with me.

| **Section A: Interview details**   1. Zone: **Northern** 2. Woreda: **Medebayzana** 3. Kebelle 4. Name of key informant: **Mrs. Gidey Yeabeyo** 5. Institution of key informant: **Medebayzana woreda Women Affairs** 6. Interviewer name: **Abate Bekele** 7. Date of interview: **20/11/2017** 8. Interview start time: **09:10AM** 9. Interview end time: **10:15:25AM** |
| --- |
| **Section B: Interviewee professional information**   1. Gender    1. **Female**    2. Male 2. Age: **25 years** 3. Highest level of completed education.    1. College education    2. **Bachelor degree**    3. Master’s degree    4. PhD 4. Current position: **Head, Women Affairs Office (Vice)** 5. How long have you been in current job/position:    1. ______ Months    2. **___08___** years |

**I:** Interviewer **P:** Participant

1. **Common maternal (Pregnant, lactating women and adolescent girls) nutrition problems in the community**

**I: In your opinion, what are the common nutrition problems in the community for women?**

**P:** In this community, there is adequate food supply but they are not using appropriately. For example, there is egg, milk and others are available sufficiently in our community however they prefer to sell them than consuming at home. Due to this fact, the children and women face many challenges like problems related with nutrition that can be mental and physical body problems. However, this is not occurring due to shortage of food rather it is because of in appropriate feeding style of the community as they have interest to sell than consuming the food items like there is honey production in this community but they want to sell it.

**I: Are there women and adolescents have the problem of under-nutrition (thinness and stunting)?**

**P:** Yes, there are nutrition related problems especially in low land areas of the woreda. But, we don’t have report rather it can be seen from the health sectors report. It is not due to shortage of food rather it is due to inappropriate food consumptions. There were mothers who were receiving targeted supplementary feeding 2000 EC or somewhere like that…I think. At that time it was given as there were nutritional problems and there was the productive safety net program then. But now as every community has become self-sufficient in food, the support had stopped. Therefore, currently there is no food shortage problem rather there is lack of appropriate consumption.

**I: Why do you think women are especially at risk of malnutrition you have mentioned above?**

**P:** Specially, there are health related problems in mothers and children due to failure to feed diversified food. Therefore, the women will become thin.

1. **Nutrition priorities in the woreda**

**I: What priorities do your institution has in relation to maternal and adolescent health?**

**P:** In the women affairs, there are many activities. Especially, for the women who have nutritional problem we work to capacitate them to have diversified food. Primarily, we work to empower the women economically through actively supporting the women at community level. We have plan especially for those works that can be easily done by women like poultry, irrigation, and cattle production. To have diversified food especially the poultry, they use the package. The package is a list of activities that are designed to empower women economically and to enable women to feed diversified food for themselves and their kids. Therefore, it helps women to use diversified food available in their home. Especially, through irrigation women can produce so many crops and vegetables in their garden. Therefore, we have made several efforts to enable them to access the agricultural food product easily from their garden thus consume diversified food.

**I: What is the importance of such intervention for women?**

**P:** Primarily, to avoid the health effect of poor feeding through developing the culture of consuming diversified food. And if they access the food items easily, they can use it. Then if there are varieties of food items in their locality, primarily the health of women, children and the entire family will be maintained.

**I: How do you evaluate the priority given for the interventions for women?**

**P:** We plan after the evaluation has been made on the problems of women in the community. Then we go to community level to implement the planned work. So, our priority intervention depends on the evaluation result.

**I: What nutrition interventions have the most resources allocated to them?
P:** Other than training, much of our works are accomplished in the community. We have budget but we have allocated high budget for activities to prevent early marriage thus to prevent the health related problem caused by early marriage.

**I: How do you evaluate the priority given for the interventions for women?**

**P:** Through time there are improvements in the practicing early marriage. These changes are due to our work done to empower women and the decision making ability of women.

1. **Nutrition interventions that improve adolescent and maternal health**

**I: How much accessible are nutrition interventions that are in place to improve adolescent and maternal health in this woreda?**

**P:** All women have the gardening. Women are using crops that are locally available though they mainly considering to sell the products. Though there are improvements in consuming the produced items at home time to time, there are still women who focus to sell the products rather than utilizing them at home.

**I: What has been done to improve the household consumption of agricultural products?**

**P:** There has been demonstration of blended food preparation to improve nutritional status of children. This is done by kebelle HEW through demonstration at the community meeting days about the preparation of blended food. Therefore, after this work is done since 3-4 years, we have seen the improvements.

**I: What kinds of nutrition interventions are in place to improve adolescent health in this woreda?**

**P:** We have interventions for all women but not specific to adolescents. The HEW gives education also for all women. There is a development army and when they called for meeting at kebele all participate it. We have 947 Women Development Army (WDA) in this woreda. Similarly, there are also males DA and hence they are participated on education provided for WDA at kebele level. The demonstration on diversified food preparation is provided for both males and women. The males are involved because to address gender related issues on the nutrition i.e. when they get back to their home to enable them to share tasks equally.

**I: What are interventions to improve health of women (pregnant, lactating and adolescent girls) in this woereda?**

**P:** Regarding pregnant women, they always give birth at health facility. They have clinical follow-ups then before 2 weeks of birth a pregnant woman goes to nearby health clinic to stay until she gives birth. They have antenatal care follow-ups. There are HEWs at kebelle, to advise woman through women development army, and they always give orientation training at the WDA monthly. So, the WDA and Kebelle HEWs are the main actors to deliver health interventions and education to the women of the community.

**I: How WDA works?**

**P:** To avoid health problems that the women may face, the WDA always evaluate and take corrective measures on the issues in the community.

**I: Is their targeted supplementary feeding program in the woreda?**

**P:** There was TSF around 2000EC, however, the community has empowered economically therefore currently there is no such program.

**I: How such economic empowerment has come to end the TSF program?**

**P:** This has been done through agricultural works like cultivation of various crops in their whole land by using adequate amount of fertilizer and improved seeds. They have been using several agriculture mechanisms like using irrigation and poultry production hence these works empowered them economically.

**I: What are interventions to improve adolescent health in this woreda?**

**P:** The work we have done is not specific to adolescents but we were considering it as to improve all the women including adolescents. The adult women have women development army but the adolescents don’t have DA. There are HEWs to deliver services for all the community. Otherwise, there are no works done specifically for adolescent women.

**I: Which of the above listed interventions do you think is most important for pregnant women?**

**P:** Especially the delivery service is the most important one. And the nutrition interventions are also important.

**I: What interventions are important to improve adolescent health in this woreda?**

**P:** The adolescents have only education work otherwise they don’t have another work. They attend the school and live with their family so that we don’t have any work. They support their parents.

**I: How interventions like health facility delivery is being implemented successfully?**

**P:** The economic empowerments increased their need to use Ambulance; there is also transport access as the roads are improved to asphalt, thus the service use has improved. Previously, the laboring mothers were taken through caring them by colleagues.

**I: How economic empowerment improved the women access to delivery?**

**P:** The presence of community health insurance, so the households have been using it and each household saves 149 Birr per year. Previously, there was financial problem to access the health facility but currently due to community health insurance health service utilization has improved since it is applicable for all community members (the rich vs. poor).

1. **Implementation challenges and community factors affecting access to maternal nutrition interventions**

**I: What are challenges to implement nutrition interventions?**

**P:** The community has still little awareness. They mainly focus on the income/money they got by selling the crops produced on their garden. Rather they sell what have been produced in their garden; they are not concerned about the nutrition. To solve this awareness problem, several works have been implemented. Especially the HEWs work on education of the community on important health topics especially the nutrition, for example, because of lack of awareness the community is utilizing only few food items while it has so many inputs for having various food items. So, health extension workers are ding to break such misunderstanding that exists on the community. On the other hand, the agriculture sector also provides education on what to produce to address the nutrition especially the community learns about the production through demonstration at a community gathering. They together with our office and the HEWs also provide demonstration on the preparation of food from diversified food items. We also promote prevention of disease is better than getting treatment for disease in our community.

**I: Are there activities targeted adolescent girls nutrition in your community like what has done for adult women?**

**P:** They are not targeted specifically rather we are working for all women.

**I: How aware are the women and girls on the need to get interventions?**

**P:** Whenever the community has told to implement certain tasks they are willing but there are implementation problems like lack of consistent use the service and failure to apply what they trained consistently. This is because the community prioritizes on how to make money rather than concerning other important issues like nutrition. They have all inputs in their home to get balanced diet like they have egg, milk and many other food sources in their home however there is utilization problem due to lack of awareness on importance of diversified foods.

**I: Is there a relationship between educational status of women and access to interventions?**

**P:** The uneducated women are more likely to have lack of awareness than the educated one. And, to solve this it takes time for uneducated one. Whereas, the educated women have all prior knowledge and if they got a few training or sensitization they can easy implement.

**I: How can this problem of lack of awareness in uneducated women be solved?**

**P:** There is adult learning program at each kebelle then they learn on various issues by different experts working in the kebelle level. The experts such as education experts teach the basic issues like enable them to read and write, the agriculture works on demonstrating gardening of important crops, and the HEWs teach on health aspects. So, this program can bring a change in creating awareness for on nutrition especially for those uneducated one. Therefore, it has to be strengthened.

**I: What community related beliefs and norms are preventing access to interventions?**

**P:** In the past, it was said to be women is not allowed to drink and eat hot. But, currently there is some improvements. A problem of such food taboo is prevalent in the rural settings and it is less common in urban. This happened due to misunderstanding that still exists in the community. Otherwise, I don’t know food taboos other than this.

**I: Are the interventions acceptable culturally?**

**P:** Yes, they are acceptable. The only problem is awareness otherwise it is highly acceptable. If they know about the importance of the intervention they can use it.

**I: How accessible/convenience is the interventions to the women and adolescent girls?**

**P:** As the interventions are provided at the kebelle level they are accessible.

**I: What resources exist to provide interventions?**

**P:** To implement this intervention, as such many resources are not needed because the intervention is done on the existing resources of the community at the kebelle level. The community doesn’t want any additional incentives as the work is already done at kebelle level. They work on their village/home so that they don’t ask additional fee. We do have also the FTC to demonstrate the production of various crops and preparation of blended food, water source, and agricultural products. Therefore, we have model farmers in the woreda to share experiences to other farmers. And, we do have human resources at kebelles to implement the interventions.

**I: What can be done to effectively implement the interventions?**

**P:** To share experience, the both model farmer and the least performed one goes together for experience sharing session. But the least performed have still failed to implement what has been shared in the session because they have lack of awareness.

**I: How do you evaluate the commitment of the interventions providers in the kebelle?**

**P:** Their commitment is good. The problem that we have is resistant community to change the awareness; otherwise we have committed intervention providers.

**I: What other factors are inhibiting implementation of the interventions?**

**P:** To change the awareness problem, it needed much time. Therefore, to solve this problem, sharing the experience done timely, and working on making farmers a model can help to solve low awareness problem.

**I: What solutions that your institution has applied to break awareness problem so as to implement the interventions effectively for women?**

**P:** We have been working to create awareness but in nature it needs much time. There is a change from time to time though it is not satisfactory.

**I: What other work do you think needs to be done to better address the challenges you have mentioned like lack of awareness?**

**P:** The lack of awareness can be solved by the provision of consecutive education for the community through allocation of budget. Especially, the problem in agriculture can be solved through time as they aren’t using full agricultural packages like improved seeds, and fertilizer. They don’t use the package that can be enough for the land that they have, for example, the farmers receive 50 kg fertilizer for the land that requires 1000kg. Therefore, this can be solved if there is continuous experience sharing session among the farmers.

1. **Multi-sectorial collaboration to improve maternal nutrition**

**I: Do you feel it is necessary for your institution to work with other sectors/institutions to address adolescent nutrition?**

**P:** Yes, we have been again working with agriculture, health and others. Therefore, it is necessary because especially it is good to solve problems of women; it allows several professional to discuss on the same issue to bring a fruitful ideas and strategies hence the change has come.

**I: Which other sectors do you feel are necessary to work with your institution?**

**P:** The agriculture sector provides inputs to produce agricultural products; the water resource office can work on supplying water; the health sector provide education on how to use the products of agriculture whereas the agriculture shows technical issues to produce crops. The women affairs office can support the team by provision of education and linking various experts. The WDA is responsible for all sectors like health, agriculture, women affairs and …. As every body works with them at the community level.

**I: How do you evaluate the level of collaboration among sectors in nutritional interventions?**

**P:** The work has started since 3-4 years back, but it doesn’t mean all community is practicing it. But to make entire community implement the interventions, we have to expand through time. We do have nutrition technical committee that led by the agriculture office. We are working a good work through evaluation and identification of challenges and strengths on the implementation of nutrition interventions. Therefore, this is to bring change and we have monthly meeting but if there is a condition that may need fast decisions we can have every two weeks meeting. We have joint plan and we can go to work together or alone. If we go alone to work at the community, the one who has gone brings information on the activities done by various experts at the kebelle and shares it to all the team members.

**I: For multi-sectorial action that effectively works to improve maternal nutrition at all levels, what kind of change in terms of the way stakeholders work together is needed?**

**P:** There are problem that are happening all the time. We put action plan for the experts to work on certain activity but it stays undone when we go for the next supervision. For example, a group of women that have received poultry production assignments may not get timely technical support like the housing for the poultry from the agriculture experts. And the women affair goes to improve the awareness of the women. Therefore, there are inconveniences to accomplish the set tasks for different sectors and this has to be changed.

**I: To what extent does your institution participate in the multi-sectorial nutrition coordinating body at the woreda level?**

**P:** We are participating in this work in a good manner as alone we cannot accomplish many tasks, but if we work together we can do good work. For example, if I go to supervise a kebelle and the other goes to another kebelle and finally we come together to with important information regarding the roles of each sector and share them to team and respective sector.

**I: What opportunities do exist to promote multi-sectorial collaboration of nutrition in this woreda?**

**P:** There is steering committee that evaluates tasks accomplished monthly and then appreciates the pros and cons. Then, the steering committee orders to accomplish the tasks that are not done yet. Therefore, this strengthens the work of technical committee as it puts direction to perform our tasks. There is plan to work together. And, there are administrates at kebelle levels especially at the time of irrigation they come together and facilitate the implementation.

1. **Other interventions that influence adolescent and maternal nutrition and health outcomes**

**I: In your opinion, do think delayed marriage (after 18 years) improves maternal nutrition?**

**P:** Yes! As she gets older, she knows everything. She gets matured if she is older than 18 years. Otherwise, she faces challenge during birth like psychological problems.

**I: In your opinion, do think increasing the space between each birth improves maternal nutrition?**

**P:** Yes! Especially, economic problem can be solved if a mother spaces birth for a 3 years. it has an importance for the mother and her baby. If a woman has short birth interval she will be sick, and may become thin. The newborn cannot get sufficient breast milk and complementary food as well.

**I: What programs or activities promote increased birth intervals in this Woreda?**

**P:** Especially, there is work done by the HEWs at the kebelles. They provide continuous orientation and education at the WDA to space birth and all other health related issues. Therefore, those who economic limitations can be benefited more from the birth spacing. They also provide contraceptives to women at the kebelle.

**I: Can you tell me about any programs or policies in place in this woreda to prevent early marriage?**

**P:** Especially, we the women affairs have a big work on this regard. In 2009 EC there were 95 girls ready for committing early marriage and 90 were prevented from marriage whereas 5 has made marriage. To prevent challenge that can follow early marriage and school drop-outs, we have been working with police officers, and others. We also provide training to health impacts of early marriage for police, for all women leading bodies such as women affairs, league and association, and school directors. At schools, the teachers can identify the students who drop-out easily. Then we work at the lower levels through this team.

**I: How do you see the influences of religious and political leaders to prevent early marriage?**

**P:** The religious leaders are our targets for giving training. However, still there are problems from their side, especially those who are called deacon. They believe “if the girls go for education they will become unmannered”. This misunderstanding has been solved somehow but it still exists.

**I: In your opinion, are these programs or policies effective?**

**P:** Yes, there has been change through time as the awareness the community has improved.

**I: What are the community factors that promote early marriage?**

**P:** Yes! Especially related with Deacons, as they believe the girl can face challenge if she goes to education. They assume the girls will become unmannered if they go to school. On the hand, there is a community believes such that “the age don’t limit the marriage” as they assume a girl can marry at earlier ages though there are improvements currently. This is happened because the community is not well aware of the effects of early marriage.

**I: What are the policy factors that promote early marriage?**

**P:**  Yes, there is law. Those who committed early marriage will be accused and jailed.

**I: In your opinion, what could be improved to effectively inhibit early marriage?**

**P:** If there are continuous trainings for especially for religious leaders, the women and all others, there will be improvements. Only providing education at school is not sufficient to inhibit the early pregnancy as it needs interventions at the entire community because the main source of the problem is the community itself.

**I: Can you think of any other opportunities to prevent early marriage and increase birth interval?**

**P:** We are working at kebelle level and there is justice office meeting of the entire community members meeting twice per year to provide education on the effects of early marriage and the legal issues for those who committed it. All the sectors come together provide necessary education for them at the session.

**I: What lessons have you learnt regarding maternal nutrition in this woreda?**

**P:** If a woman has good nutritional status, the child has good mental wellbeing. And, it is important to maintain the health of a mother.

**I: What lessons have you learnt regarding multi-sectorial coordination of nutrition in this woreda?**

**P:** As it easy to solve the community problems with in short period of time if we work together in every task. Otherwise, if we work independently, we cannot achieve our goal.

**I: What opportunities do exist to promote maternal (pregnant, lactating and adolescent girls) nutrition in this woreda?**

**P:** The presence of all experts such as HEWs, the agriculture….. Thus the environment is smooth to work.

**I: Thank you very much for you time!**

**Summary**

1. **Common maternal (pregnant women, lactating women and adolescent girls) nutrition problems in the community**

- In this community, there is adequate food supply
- But, they are not using appropriately.
- There are nutrition related problems like thinness.
- It is not due to shortage of food rather it is due to inappropriate food consumptions.

1. **Nutrition priorities in the woreda**

- We work to capacitate the women to have diversified food.
- We have allocated high budget for activities to prevent early marriage.

1. **Nutrition interventions that improve adolescent and maternal health**

- Women are using crops that are locally available though they mainly considering to sell the products.
- We have interventions for all women but not specific to adolescents.

1. **Implementation challenges and community factors affecting access to maternal nutrition interventions**

- The community has still little awareness.
- In the past, it was said to be women is not allowed to drink and eat hot.

1. **Multi-sectorial collaboration to improve maternal nutrition**

- We have been again working with agriculture, health and others.
- There is nutrition technical committee and the woreda steering committee to implement this nutrition intervention.

1. **Other interventions that influence adolescent and maternal nutrition and health outcomes**

- The deacons believe “if the girls go for education they will become unmannered”. Therefore, they promote early marriage.
- The community believes such that “the age don’t limit the marriage” as they assume a girl can marry at earlier ages.
