# Supplementary material for: Barriers to utilize nutrition interventions among lactating women in rural communities of Tigray, northern Ethiopia: An exploratory study
Source: PLoS One. 2021 Apr 30;16(4):e0250696. doi: 10.1371/journal.pone.0250696 (PMC8087028; doi:10.1371/journal.pone.0250696)
Supplement: S2 File — (ZIP) [file pone.0250696.s002.zip › S2_File.Doc/Woreda level and above key informants/115_IDI_Woreda education office expert_Medebay zana woreda.docx]

**Operational Research on Adolescent and Maternal Nutrition in Northern Ethiopia**

## KII with Woreda education office, expert

**Introduction**

Hello, my name is Amaha Kahsay. I am from Mekelle University. Thank you for taking the time to speak with me today. We are doing research on the factors that influence the nutrition of mothers and adolescents in collaboration with the Regional Health Bureau and UNICEF.

**Section A: Interview details**

1. Zone: North-West Tigray
2. Woreda: Medebay Zana
3. Kebele: It is town administration
4. Name of key informant: Teacher Meressa Teferi
5. Institution of key informant: Medebay Zana woreda education office
6. Interviewer name: Amaha Kahsay
7. Date of interview: 20/11/2017
8. Interview start time: 10:01AM
9. Interview end time: 11:30AM

**Section B: Interviewee professional information**

1. Gender
   1. Female
   2. **Male**
2. Age: 53 yrs
3. Highest level of completed education.
   1. No formal education
   2. Primary education
   3. High school
   4. College education
   5. **Bachelor degree in Geography**
   6. Master’s degree
4. Current job/position: Developmental planning expert
5. How long have you been in the current job/position:
   1. ______ Months
   2. 03 Years

**Section 1: Common maternal (pregnant women, lactating women and adolescent girls) nutrition problems in the community.**

**I: In your opinion, what are the common nutrition problems in the community for women and adolescent girls at Medebay Zana woreda; especially at those adolescent school girls who may be under your sphere of observation?**

**P**: We thought that the total percentage of females is almost 51%; when we say this, the number of those lactating mothers at kebelle or woreda level, their number may not exceed that of the adolescent girls; but the evidence may be gotten form health sector; but as to our institution, those we call them lactating who are not found at school are not clearly contained their number by our office. But what we contain as an evidence is that those who are found at our 59 schools especially those females found at our grade one to grade eight schools who are reached the age of schooling and who are present at schools; we have their numbers; so, when we take at woreda level, let say they are from grade one or like that; we have totally 18,416 females students; indeed these are when they are clarified by their sex; and those ten years and above are found from grade five and above; when we see it in the case of gender mixture, indeed we planned about 1% at woreda level; and these females have females’ affair clubs at the schools; we have around 37 primary schools; then the females found in these schools are embedded in the clubs so that they are let attend their education well and also followed not to quit their schooling; they are also given issues about their health, their feeding practice; they are given training and share experiences by their clubs. The school also has its own plan to them that they have not to quit their schooling due to below-age marriage and again not to be endangered on their bodies due to that marriage; so, the club plays big roles in these issues. These females have good movement on improving the issues of below-age marriage and that of backward feeding practice via the club they have; thus, to strengthen these females there are also stakeholders with us like that of Co-WASH, One-WASH, Glimmer, and world vision; especially for these females to get adequate education and at their health not to be hurt by taking adequate understanding and attending better education; and also about their diets too; all in all about their families, they are playing better role.

Indeed, if we enter to the nutrition, indeed there is limitation; when we say nutrition at taking the qualified food products, the community shows gap at practicing it; even though there is adequate food sources and production, but at the preparation, there is gap which is not still peeled. Even though our community has its own living levels; but at the feeding practice, there are still problems not peeled yet. And these adolescent school girls too, at least at taking those body building foods, and disease preventive foods, they have their own problems again. Thus, for the future this nutrition needs capacity building training; because, though the food production is present, but due to lack of awareness there is problem about it. So indeed, though these adolescent girls are the ones who live and prepare foods at their families and even help being as mothers; they still and those lactating mothers too, have problems at taking those balanced foods. Indeed, what we do here is that the women affairs are working with us; the health professionals also especially those health professionals who are deployed at kebelles are providing at nutrition and about those mother who reached to deliver; and this has change indeed. And for the mothers, what should they feed, and the feeding about their children and during pregnancy, by the HEWs and women affairs and by the school through the female clubs, we believe that it has better change. Now, those who face malnutrition like scurvy and kwashiorkor and other diseases that comes due to malnutrition; it can be said almost they are decreased; what this means is that the sectors as per their own task involvements at the community specially at the mothers due to they are the main deciders of the home and they are also leaders and the life of the home and the life of the community is at their hands too, these mothers which the training and the events that they get about nutrition, it can be taken it is relatively better now. It is now taken that a baby needs eggs, milk and other enriched foods from different mixtures of cereals and whole grains. Totally, though we don’t say basic change is come, relatively there is limited change obtained; thus, it can be said that especially those adolescent girls who reached school age, those who are aged to 10 to 19 years are relatively found at better thinking level at this time. Yet, due to the social and economic condition of our area, it can’t be said that the gap of the dietary practice of the community is resolved; especially of those mothers and adolescent girls, it may not be said they take those building foods; but it can be taken that the awareness is present still.

**I: Very nice! Teacher; earlier you have told me that though there is food production and access to the community, yet, there is gap at preparation and dietary practice of the community and the mothers and adolescent girls in particular; so, what could be the reasons for this gap; is that because they have not awareness or no bodies that let them have awareness; and what would do your institution in addressing this gap?**

**P**: Okay; as I said it earlier if I am not to repeat it; currently, our community has a great role at increasing the production; that doubling of the production in relation to agricultural involvement, the community gets almost adequate production; so, it may be difficult to say that the community has lack of food or to say it is exposed to food insecurity at this time. But, though in its presence, properly in the nutritional aspect of the community like preparing from those three kinds of foods those health givers, disease preventers, and building foods; in getting adequate awareness about them and in preparing the food is present unpeeled problem until then. Thus, though we have the food production, we have still a problem of awareness in preparing the foods from those disease preventive and body building foods. For example, we have had experience of going to the kebelles with other sectors together; and we observed that though the community dresses well, but we observed that it has problem in building and having good body appearance; indeed, there is no problem of egg, no problem of meat, no problem of different cereals; yet, in preparing properly thinking that it will be important to life and health, and body, and in believing that if we eat in this way it will preserve our life; we take it there is an awareness deficiency; and we took it like that even previously. It is present; but unable to use it; thus, what it needs is that everybody at each level from agriculture, health, women affairs and other social unions, these then in creating platforms for developing awareness of the community about its nutrition is still not done; especially if we take our sector, those I have said that those female school clubs and those females at school are main owner of the home and helpers that can play their own better role at food preparation; and also at the feeding practice of the community that they would to transfer messages that they got it from the schools and also they would to apply it at their homes too; but we have a long way to do it yet; we have still a gap in what these adolescent girls would have said to their mother or father about the food preparation and also in bringing change at the community and also at their family level about the dietary awareness and use. Most of, it is said it has to be kept its hygiene in preparation; but more than that it would have been done about preparing balanced one mixing form those all available food products at home; there is still gap in adequate knowledge and awareness to eat in balanced way; but now, we at each level together with the stakeholders; there are trials that we are practicing with the females at school to create better influence at their family and the community; we have also as plan; especially those females have to play great role that we already have it as plan. We have it also at our plan with those stakeholders that beyond that hygiene and sanitation, as that of nutrition is the main condition that determine the presence or absence of life; so, it has to goes up to the ground and it has to be worked at the community; so, so as they to create awareness, we are creating platform for them; we are also creating training for them. But now being it added or subtracted, when there is production, but in using properly so that the adolescent girls to maintain their health and body and that community to keep its health, a lot is remaining on its sustainability; it needs works.

**I: Thank you; may be that of Co-WASH and One-WASH, what are they and what do they help you?**

**P**: Most of, they are involved at the health; at the latrine and its utilization; yet, side to side to this, they also help as at the personal and environmental hygiene and its relation with nutrition; they help us works and budget; we also select clubs and we give training along with them in person; especially that of World Vision, it gives big training for the females; it is to apply that of nutrition at their homes too.

**I: Are these programs or what?**

**P**: Yes, for example, One-WASH is a program centralized by the region; and that of Co-WASH is also from the region; that World Vision is alone found in this woreda; the One-WASH is the program through the education bureau, and that of Co-WASH is one program that is emerged through water resource. These then, beyond that hygiene and sanitation; they raised from the angle of that the nutrition of the community has to be changed and its health has to be improved and maintained; thus, they budgeted and being involved at capacity building and providing trainings up to the ground level.

**I: Thus, you told me that due to the awareness gap that the community has especially those adolescent girls; there is dietary practice problem to them; and you said it again it has to be done a lot for the future; so, due to the current dietary problem at them, may you observe any nutritional problems to them like that of goiter, night blindness, short stature, and others at the adolescent girls?**

**P**: Currently, we go and observe our students properly; thus, those problems like goiter due to iodine, others and that of thinness are almost not seen at this time; this might be due to earlier taking of vaccination, and then they are not seen more at the community; they are not seen more diseases; as a result, we don’t see mentally and physically hurt child. But what might be seen most of at the body is that of thinness; that thinness is seen; and this indicates that may be as we said it is due to not using properly that of production available; it is already the problem present at food preparation; otherwise, we have not critically suffered children and adolescents; it is not much concern. Now it is right, at school surroundings that used to be ill their eyes due to lack of hygiene, there is now change; even the teachers follow them about their hygiene and feeding practices as it is given related to the subject matters. So, there is no that much problem; that a student is unable to come to school due to his body is suffered due to severe problem or like that; it is not present; it can be said that there is better change. Yet as to our observation, we have still gap in deeply digging the problems of each student. But now, what we observe is that had a student had any problem or malnutrition or hunger, which would have not come to school; that is the observation that we have until then; we would to take this observation; but we don’t have any student who doesn’t come to school due to these problems; there is better change. Other than this when you compare, the feeding is different as per capacity, as per home content; there may be present difference then; otherwise, it is not being said that children and adolescents are being exposed to such kinds of diseases.

**I: You told me that thinness is a bit observed due to that feeding practice problem; is that more at boys or girls?**

**P**: Good (he laughed pleasingly showing that interested at the question); when you see the females at that age range; that maturity itself may give you something…but what is observed is that most of it the boys are a bit thinner.

**I: What do you think about it?**

**P**: Maybe (he smiled for his reasoning), here, those females with their mothers may have relation working at the home; whereas, the boy may come out of home that may pass his hour of diet or else; but, I take myself that the boys are a bit thinners.

**I: Nice; we laughed both about the reasoning he gave; then continued to ask; is there food insecurity problem here; indeed you told me that food security is not a problem here; but may there individuals especially who don’t eat either breakfast or lunch or stayed the night with no food; especially those adolescent girls who may quit schooling due to this?**

**P**: What is right then is that when we see the economic and social condition of the community, we can’t conclude that all the community gets adequate production; ehhhh…the community can remain without production due to different reasons; though at utilization is equal, but at the production there is difference; ehhh…maybe it may be difficult to say there is an individual who stays the night with no food due to lack of supporter or due to lack of capacity; now, the government is also providing support saying that no one should stay without dinner; and also maybe to come out from the expectant dominance, those who can work are currently being engaged to production by themselves observing from their neighbors and unions. Maybe, due to old age, damage to body part or others, there may present individuals who can get adequate production; and we don’t expect them to get adequate production. Yet now, that of aid expecting community is totally being changed except those due to old age and those with any body part damages; but that community part with fine body parts and mind is now engaged in productions; unless, there is shortage of land for production; it can produce what it is adequate to it. Yet, due to narrow land, due to unable to use those agriculturally introduced improved products, there is less production still; thus, for such classes of communities, it may be difficult to say they survive all the year with their food productions. Whatever so, all added the shortage of land and natural conditions, it may be difficult to say that the community is totally out of food security; considering also the social problems; because, these are not solved; the thinking, social and economic level of the community is not moving at equal level; so, seeing that haphazard gaps of the community, we can’t say the problem is totally resolved. We also take it as it is present (he means the food insecurity); because, we have areas that are remote and deserts where the productions there, are less important as foods; because, most of the cereals like legumes, barely and millet, are grown at highlands; thus, due to that geographical impact to the crop productions, it can show that there could be food insecurity; it indicates. For example, there are who have goats; but they don’t consume meat; rather, they take them to the market for selling; these are problematic then; hence, an environment has its own impact too; but totally, whatever the environment is; is that by buying or exchanging, just the awareness at getting the adequate diet, there is still gap; for example, if there is no Teff at lowland, it has to be taken from highland; if goats are reared at the lowland, it would sold goats and would have bought that Teff; but such kind of feeding system at the community and awareness about it is not that much; yet, the community thinks that its richness is as an asset; it brings it to the market being it goat, cow and even that of egg; more it brings it considering for salt or sugar, it would have used it, itself; but due to lack of knowledge, that it would have fed and produced them easily as surplus, are not being done yet; there are problems. But anyways, there are sporadic poor individuals that the problem is seen at them. Yet, what is left is that of nutrition and the quality; otherwise, even that child doesn’t go without filling its abdomen; then, content of the food is so different.

**Section 2: Nutrition priorities in the woreda**

**I: What are the priorities that your education office is working at improving the health especially the nutrition of females; along with the main tasks of your institution?**

**P**: As sector, we didn’t identify which mothers are being hurt; but we simply work at schools being together with women affairs and health sector; so, at the school using little minutes of the school queues, we tell that the health of females and mothers has to be kept; this are simply totals of awareness creation conditions; so, there is limited education; yet, we didn’t go deep into the mothers at the families level. But we consider that those females at the school may take the messages up to the community and families level; otherwise as professionals, we didn’t do anything to mothers by creating platforms; but we do at schools to be created adequate awareness. But now it is to be begun about the nutrition as Save of the children came to the woreda; it will be done for the future; otherwise previously, those adolescent girls got adequate awareness about early marriage at schools; as a result, we didn’t face much pregnant females who are at school; thus, except that short awareness creation activities at school level, we dint go to the mothers and we didn’t created deep understandings, trainings or ability or knowledge; nor to the pregnant and lactating mothers too.

**I: How about for the future, do you think it is necessary to work with other stakeholders like that of women affairs; thus, going up to the community level to improve the health and nutrition of the community and the mothers too; is that necessary for your institution?**

**P**: Good; then mainly, for all I know it would be better if it is done at schools; if you create adequate awareness at schools, at each community or household, it has student at the school; thus, if we send those adolescent girls who are at school aged 10 to 19 years by creating adequate knowledge, then it is our believe that they can change at the home too. Then maybe as program now, there is a beginning; together with the health, women affairs, and other stakeholders at least if we bring change at schools, we have the thinking that we can bring a change to the community too; because, each household has a part at the school; then these adolescent girls going to their homes, they can bring changes being it at sanitation and that of feeding if we create adequate awareness and capacity to them especially being collaborated together with health, women affairs, and those other stakeholders; but this may have its own budget constraints to address all those 59 schools; yet totally, if we do it bit by bit with small schedule and time together with the stakeholders, we believe that it can be worked and change can come; especially, we believe that our students can bring big change. Even now, it is due to this believe that being with the health and the stakeholders that it is being planned that hygiene and sanitation is to be worked at schools if we need to bring change; and we took it as initiative; and accordingly, it will be done for the future; and they will come to us in the coming foreigners’ new year so that together with them at least we will cover seven to eight schools; so, we have the believe that the rich knowledge and capacity applied at schools will be arrived at the community; and we believe that we will work giving that focus.

**I: Very nice; maybe I have heard 59 schools; how many schools do you have at your woreda?**

**P**: That is, they are 59; thirty something, are one to eight; five are secondary schools; we have 18 one to four schools; hence now, those matured females are more found from fifth to eighth and form ninth to twelfth.

**Section 3: Nutrition interventions that improve adolescent and maternal health**

**I: May there nutrition based interventions that are undergone at schools through your institution; because until then we have been talking about the overall health related interventions undergone by your education office; so, may there any activity being it counseling about their dietary practice, school gardening and others for the females at school?**

**P**: This then; it is not worked about this indeed; we don’t want to speak what is not done! Currently, there is no education given about nutrition at schools having its own schedule, plan and platform; there is no also at the education system that is given at the class by the teachers; and also we didn’t go to the community and we didn’t do what has to be done there about the feeding system of our mothers and the community. But simply in a short way, unless those by those HEWs, there is no any program planned by our sectors about the feeding system of the children and mothers at the community; yet, we expect stakeholders to be along with us and to help us to take it as program for the future; otherwise, it is not done by the sector yet.

**I: May you have schools which practice school yard vegetable gardening?**

**P**: There are no; most of our schools are at hillsides where the soil is not fertile; they have not also that of water access; ehhh… this is one problem. Plus, in the case of the backyard vegetables, may be previously in the previous education systems, there was agriculture related ability something (he needs to say education)…but this, currently at the education system, it is not applied by the education bureau; there is no agriculture related or agriculture based production introduced at schools having its own agricultural personnel at schools; and it also left out being given as a subject; so there are no schools with backyard vegetables gardens. But previously, what was present was cow for milk production was introduced to schools; those improved folk of cows were introduced to schools for the milk purpose; but now it is phased out.

**I: When was that happened?**

**P**: They were introduced four years ago; and there was good introduction of milk at schools and milk used to be sold at the school lodges. Now, I don’t know what (he smiled showing his sense of concern) maybe, but may be the work was becoming boring or its economical benefit was not profitable; it is phased out then; otherwise, we used to have Begayt (improved types) cows at four to five schools; it would have continued; but I don’t know, that was gone worn out.

**I: By who was that introduced?**

**P**: It was one working system released by the region; especially there was a thought that if there would to present experiences that link with health and agriculture sectors, that would to go down to the community; as a result, there used to be schools that were using their own budgets and the grasses of their surrounding to introduce those cows; but, that caring, and looking after them showed a gap; otherwise, at four to five schools, the cows were able to feed well milk to the school communities; and these would to be used as learning model to the community and would have been continued; but not continued (he laughed that shows a regret); and this is the problem of motivation of the schools to continue it by themselves; otherwise, they were good, for example, at the school called Bekuretsion, there was a Begayit which become six to seven ones in number; thus, this cow used to feed the school and also used to bring income to the school; because, there was adequate grass at the school and an adequate milk was milked; then, the school community was able to use at the school lodge; but that showed gaps at its management and the cows were already sold; but we didn’t support that event; it was good source; had such kinds of productions been continued, we believe that change would have come.

**I: I can see that it was successful initiative; so, what is being thought to return it for the future?**

**P**: If we need to introduce such kinds of introductions, it is needed man power; there has to be present man power that can manage it. Even though it may not have its own structure, if the schools have their own interest or motivation or initiation to introduce it, maybe it is possible; if there is adequate grass or straw at the school surrounding, it would have been possible and would have been as used as source of income for them; yet, we need to help the schools in every aspect we intend to work at our leading plans; especially, we have potential schools that can implement it successfully; so, we need to have it as our plan for the future.

**I: You have told me that you have many WASH related activities at your schools; so, can you tell me what task are being implemented to the females at school related to water, hygiene and sanitation issues?**

**P**: Good; at those stakeholders that of One-WASH and Co-WASH, they are the ones which resolve the basic problems of a school; especially, they give budget for presence of water to a school either from digging well or by roof-catchment; the same for One-WASH like that, they work on package; that is, they work at water and latrine; ehhh saying that the school community has to keep its hygiene; and we have clubs that work at how these introduced packages have to be used by the school community, and by the students; we have club WASH related with health and club females, that have great activities in the school community starting from the latrine to let all the school free from feces, rubbishes; they play great role. Thus, every school has its own clubs that have their own plan and schedules about what activities to do, and they adjusted their program; and we also gave training for many schools; for around 25 schools, we created awareness; we strengthened their capacity. Especially after the emergence of these by Co-WASH and One-WASH, there is menstruation hygiene management program; that we gave big education for females about they never quit their schooling due to menstruation. Now for the females, in this program, they are cared not to be startled and escaped due to that tinge of menstruation, they have separate classes that they use it for taking rest and to change their dresses if they see menstruation during classes; they also have water access at some of the classes which is being done at the schools which do have fine budget in addressing these classes and water access to them so that they take rest and wash; and after that they get back to their classes lessons. The same to this, the female teavhers who organize these female students are well aware about this menstruation; and go up to preparing traditional pad if there is no present modern pad for the school girls. Currently, it can be said that each school has pads; there are schools which introduced the modern pads; and the overall aim is not to miss the female students from the education system. And utterly, it is being said menstruation is a natural wealth; they have even a slogan that says menstruation is our wealth; that they have not to be startled; parallel to this, training is being given to the boys about to be alongside the females and to know of what menstruation is; thus, this is the main program that we are working with Co-WASH and One-WASH; and we have one diligent and model teacher that she is taking it to be practiced at all levels. This is better change; so, what we evaluate here is that this program beyond to the school hygiene and sanitation, it worked at making sure for the females not to quit from their schooling; and we have not females who quitted their schooling due to this. And as to the below-age marriage, our woreda is known and we are praised by the women affairs; so there is better change. And as those Co-WASH and One-WASH, we have worked well on that of SCLTHS or school community leaving total sanitation and hygiene; plus at that of MHM or menstruation hygiene management, we can say there is good change; as a result, there is no problem to the females; the same to this too, having adequate knowledge about it, there was a condition in which they transferred it down the community. So, these are our helpers and there is also a condition that we are transferred from learning into practice.

**I: Is there any food given at schools?**

**P**: Until then it is not begun; that feeding style is not present at our woreda.

**I: What could be the reason for it?**

**P**: The reason, it may have its own study.

**I: May it be due to the reason that the woreda is rich (I laughed waiting for saying rich)**

**P**: Ayiii (no), it is not because it is rich (he laughed too); we have that poor part of community; as we said it earlier, the community is not equal due to different reasons like that of narrowing of the land; thus producing and consuming adequate amount of food might be a concern; but whatever so, there is no as such natural disaster exposure, so, it was no studied by them that food is needed to them (schools). Yet; we have two sides; may be two lowlands to the direction of North and to the direction of South that we have many drought areas as they are lowlands; so then, the foods we called cannot grow adequately there; indeed there is no emergency we faced yet; but at least we guess it is needed to them; for the future it is better for these kids if they get alternative and better foods for them; it is good if they get better food; otherwise, until then it is not introduced yet.

**I: You said that there are no females who quit from their schooling at this time due to the care that is given to them; what if there are females who may quit their schooling; what do you do to trace them back and at improving their health and nutrition too?**

**P**: We work on it mainly; when we say this, any female who her age reached to school, we have study about them by their class directors and teachers; we have each study by the teachers and the female school clubs; starting from her financing if we get evidence that she is financed; because, for those females above grade five, the community has the tendency to let them marry during harvest times; so, a big study is done during that period. For example in the previous year, there were many girls who were let ready for marriage; and if they were married, either they will get lost or they will say that they will not cope academically then went despair; thus, we protect them as much as possible; we even went up to the court; up to the court! And also if she is already let marred by the family, we also let them returned by making evidence and agreement. Thus, most of due to this issue there are on students who quit their schooling; because, at the home the females are being capacitated; at the kebelle there are women affairs; and at the school there are female clubs; thus, this is being introduction to them that we are doing to protect them from missing them from the education system.

**I: Maybe, from the interventions what you have been doing at females’ health and nutrition, which one of them do you think are the most successful?**

**P**: Then, in the case of health, when the emergency conditions like that of acute watery diarrhea happened, we worked collaborated with health; not to be exposed for this disease, we go down to the schools and we discussed with the schools about what shall be done to be free form that disease, how should our students keep their hygiene, and how should they use the water too; thus, we worked together with the health; then, there is no student who is exposed to that emergency; we are free from it and we were to working at it prior to its occurrence when we hear that it was happened somewhere; we work on what they use to drink, on what they drink and on their feeding ways going to the schools in advance to the disease occurrence; we do this all collaborated with health. But what we said in the case of nutrition, indeed it is given side to side with that health though it has no its own way; even it is given as awareness by the teachers during the subject matter classes they provide. But in the practice issue, it may have its own commitment and motivation concerns; otherwise, together with the health and other concerned bodies, we give awareness creation sessions; especially at what the female should do about to help the mothers who are at home about what can they create to them; about their nutrition, the production is present; but at the preparation and at the utilization what can you help them; what you have taken from school has to be applied there. Even though it is short period, we also teach them some practical capacity building sessions too; thus, there is no one exposed to emergency conditions. Yet, we take it that there is still gap; especially at the nutrition, there is production access; but unable to utilize it is the problem in capacity and knowledge; so, starting from the school community together with other bodies, we have to work at this.

**Section 4: Implementation challenges and community factors affecting access to nutrition interventions**

**I: Maybe, in your attempts to achieve the success of your interventions in all what you have tried until then, what challenges do you face not to reach at your intended successes?**

**P**: One, it will be that of budget; I mean, if you need to create as such platforms and to give trainings, it needs its own budget; thus, the one who gives trainings needs budget. Plus, there are also schools which are far out from transportation access; so, it is needed transportation too; there is shortage of transportation; thus, if these two are adjusted, there is no problem of giving education going down there. Yet, that of transportation and budget capacity deficiency are taken as the ones that cause problem; due to this, we don’t get all our schools as the intended time that we would to get them; we have transportation problem; plus, the one who go there and gives training and even the one to be given the training, if it is needed to create better awareness, it is mean budget is needed; budget is needed; this is the deficiency. Because, the schools are ready and volunteer to get the awareness; they have not problem if you schedule them; their accepting sprit can be raised as a problem; rather, we have to do it for them clearly having its own time and schedule and having our own budget and transportation to do that; otherwise, there is no complain that is raised by the schools.

**I: What did your education office do to resolve the problem of budget and transportation deficit as a solution?**

**P**: Now, now (He laughed to mention as what is given as a solution); as to the transportation, the education bureau gave us around two to three motors-cycles; especially that of One-WASH, they gave us. Now maybe (he laughed in showing there is problem); what is at the budget is that, if there is someone to have driving license for them again, there will not present that budget for training (he laughed again); anyways, this is personal interest; but, we stayed with no license due to these reasons; thus, we are exposed to punishments; so, if we come to our office in this year, motors are come to us; and we can say that our transportation problem is almost solved; and also, one car is deployed to us at this current period. But in the case of the budget, there is no much in this year unfortunately; even as our office, there is no much budget planned for this year; it has its own scarcity; perhaps for the future, it may be searched from concerned bodies; but fortunately this woreda has good opportunities with those like Glimmer malt, and those WASHs, and that of REST (relief society of Tigray) that work for us at water sources and they also gives for us some trainings. So, even though there is haphazard collaboration with these bodies in using our maximum and deep link in addressing common plans due to our overloaded internal tasks; and also though they don’t give us as such clear budgets, they are helping us for real; and yet, we still can have better collaboration and achievement of plans together with them for the future too.

**I: Very good; how about for the future, what solution do you suggest as solution to resolve these challenges?**

**P**: So then, what do you think that is; all everything cannot be adequate at once; if we say budget, we know the economic capacity of the country, the region and this woreda and all the area; but at least, level by level, those small budgets that we get, we have to use them properly; if there is commitment, there are tasks to be done with limited budget and even with no budget to be done; for example, you may go to schools with on budget if you have commitment, motivation and interest to work; yet, perhaps here there may be shortage of man power and then that routine task in the office may influence you too; which then it causes you problem to schedule and to go down to the schools.

**I: How about in the community that maybe barrier to your interventions related to the nutrition of the females that may hinder your interventions to reach to the intended females and that community itself; in the cases of culture, religion and beliefs, may there barriers that you can tell me?**

**P**: Ayii (no), we don’t take that the community creates much gap about this; now, the thinking can be taken as better; the community especially this generation is moving by understanding the purpose of the education; it doesn’t hate those modern types of introductions, productions and capacity buildings. But that of being inclined with religion is another issue; we don’t have even issues that take us to the religion; there is no religion that aggress the right of these females in being educated and capacities at their schools too. Rather, the opportunity has to be gotten to give better awareness creation to the community as there is no any refrain by the community to get better education; there is better awareness by the community, and it is not challenging. Even, if there are modern introductions, the community doesn’t resist; if you go down and let it understand about the importance, it accepts you well as far as it is benefited from them; it has not problem. Maybe, that I can suspect little is that there were very few religion bigoted individuals that used to oppose the females at their education; but, we convinced them then and there is no problem even with them now.

**Section 5: multi-sectoral collaboration to improve maternal and adolescent girls’ nutrition**

**I: Do you feel it is necessary for your education sector starting from higher level up to this woreda and lower levels, to work with other sectors/institutions to address maternal and adolescent girls’ nutrition?**

**P**: He soothed his throat; then, yes it is; if we are to decide about the nutrition of these females to let it into better status; it has not to be us alone; for all I know, they learn it at class; but the focus is less; if an additional force is added, there could be better change; if we get the mothers by one side ,and those adolescent that we teach them by another side, we believe that of their both additional result can bring better change; this then, if go down alone as sector, it may have its own something; if possible, those women affairs as much as possible if they meet those female students, they can bring change; thus, sectors like women affairs, unions of women, health itself, agriculture itself those who are at the woreda and even at that kebelle who teaches, then if they see that which products are producing and if they able to create awareness about it, there can come change. Currently there is practice-based introduction of education; thus, in this practice-based education, it is practical and if that of agriculture, health, water, and education if we work together, I believe that there can come change. So, if we go alone, there will not present change; hence, those sectors if collaborate together, there will not be any means that the nutritional condition of the community will not be difficult to be changed. So, had we been able applied it at the practice-based education, I believe that there would have been changed; because, there are health professional there who need to work, there are agricultural workers, there are women affairs; so, if these are critically given that awareness starting from its production up to its consumption, the change will not difficult to come. Thus, running only for education may not bring change and we have to collaborate; because, the current generation, that young generation is becoming so modern about the way it thinks, dresses and eats; so, if we teach it about to change its feeding, sanitation and dressing, there will no one that can’t apply it; that our previous backward feeding, dressing and sleeping is almost being changed currently; it is working better work; but if to come still better change, I believe that we have to collaborate with those stakeholders.

**I: Do you think is there current working in collaboration with other sectors; how do you see that?**

**P**: Yes, there is, but it is a kind of cut down into pieces; there is problem at some of us; for example, those HEWs go alone; as to us too, there is a condition that we go alone; now there is a problem of not being organized; now at the ground, the HEW work teaches alone, the agriculture is also being cut down; so, there is a problem of being interrupted; it is not going as per it was intended in the way to go; the needed program is not being applied; the one concerned body which would to organize the schedule that would let health gives awareness on that date, agriculture on that date, and education on that date; it is not present; it is being interrupted. Thus this is the problem; so, it has to be gone in collaboration; for example at our schools, if we need basic change, we have to go in collaboration; but as I said it, this will need budget and it will need transportation; so that the community can get what it wants at its any convenient time; and would have been changed; because, our community is at the edge to be changed, it needs only little push; thus, it would to accept it.

**I: Do you have any nutrition related coordination platforms at your woreda level; does you woreda education office coordinate or participate at nutrition related platforms?**

**P**: Coincidently, I have heard about it at this moment; I have heard from the women affairs that they were that they were invited to Axum and have had orientation or like training about nutrition; perhaps it may be needed to be introduce to them or. Again from us, at this near moment, they were invited; I think it is about nutrition by save the children and two from us are invited and went there; so, there is such kind of beginning; but previously, I don’t know; before my coming to here, I never participated at such kinds of platforms; perhaps other bodies may talk about it, the information that I have about it is not much; I don’t think big awareness is given to it.

**I: So, how would it be improved these kinds of platforms for the future at your woreda level?**

**P**: Yes what is right here is that without having the good health of children and mothers, there will not present change to come; if we are to prosper about our social and economic status; it has to be proven that there is healthy citizen; and if the health of the citizens is to be maintained, the production that is being obtained has to be utilized properly; yet, we have to have adequate awareness about how to let our community use that; and thus, I say that there has to be present collaborated program to do that then. Because, we are watching that though there is production, there are people who may not able use it properly; so, we have to at least create a knowhow to it about its nutrition that about how to prepare it, how to utilize it and how to bring change on itself; we have to let the community know what it has to know by having adequate awareness we ourselves; so, it is mean that training is needed; our community is nearby to change if we provide it what it has to do; since nutrition is about life; but due to lack of preparation and readiness, so as not to be endangered by malnutrition, at least it has to be done on utilizing of those available productions at the community; and thus, it needs training.

**I: So, if we get back y to your woreda based on the recommendations you gave us to improve these nutrition platforms, what opportunities would we get for the success of the improvement?**

**P**: From the opportunities, ehhh…indeed, we said that we are going separately; thus, I would say that these separate movements would have be better collaborated with those concerned bodies. It would have be better identified that who can better lead that of nutrition of our community and it has to be scheduled; and it has to be identified that where should be our implementation population; is that directly to the community, or at schools, or at women development armies and others too; we have to be collaborate. Now, nutrition is more related to health and women; though we say that education can create big influence; but we have at the ground those women development armies that can accept well; and these WDAs should be collaborated with the woreda women affairs and union of women; so, these have to let them well organized; so, mainly health professionals plus women affairs, agriculture, water also itself, all these if well organized, there has to be present collaborated environment. Perhaps, if it is clearly identified the time and place and if they are well organized as a team, and also if one organizing body is emerge from these bodies that can then monitor things s independently and can let us all go down to the ground to work accordingly, I can it say it this is an opportunity.

**Section 6: Other intervention that influence adolescent and maternal nutrition and health outcomes**

**I: You told me that you are sacrificing a lot on preventing that early marriage to those adolescent school girls and also you have experiences even going up to the courts in safeguarding them; so, what do you think about its importance to the health and nutrition of the school girls that you sacrifice this much; do you think it has impact on them?**

**P**: Good; our current effort is that, one, it is that the girl has to get the chance of education equal to the boy. And if you help a girl, we know that she has sharp mind; if they are treated they have equal capacity. So, we need them to be equal number of female students with male students; and also we need them to sustain academically up to the end. Because, we don’t need the females be interrupted from the school; while having equal capacity and equal right to the education; development without females is also impossible. Thus, one, she has not be interrupted from the education system; plus, if she is married, physically she is hurt; especially, due to the current below-age marriage happening, their body system can be hurt, ehhh…what you call that is followed at the Ham…lin; yeah, fistula; this itself, they are exposed to this if they are below-age; we don’t need them again be exposed to this emergency. Plus, if this girl is pregnant at this age, she will not have adequate body; and her baby will not be also adequate. Again, she will be economically endangered; and if she is not well economically, it will let her be in problem of her nutrition; she will not get nutritious food; it will influence her living; thus, she will be hurt physically and academically too. So, our main target is to let these adolescent girls equal to those boys both in their academy and their nutrition.

**I: Great; so, who is promoting all this starting from the schools up to the community level in such an extensive way?**

**P**: (He soothed his throat); then, this is a program and it is a structure as at the bureau and as our office level; it is our extensive work that we have to do it as our plan, program and it is as our structure utterly. It is very strict, let alone to be interrupted from their schooling, we work to bring them up to leadership; up to leadership! At least, even at the class and at the department, she has to be present; that is it! Thus, as program, as bureau and as office, we work actively here. Even other bodies if they come, they support us this; everyone come with a program that says females should not be hurt; they have to be capacitated; because, without them (he laughed to give stress on the issue), there are no any developmental activities to be undergone. So, it is as our program, and as our plan; and mandatorily, every expert here has to prepare checklist about it; and also has to give orientations and trainings about it. For example currently, our supervisors look after this; our school directors, look after this; those established clubs look after this; thus, this is just like a program.

**I: How about that of birth spacing, how much focus you give it?**

**P**: (He laughed to show that what it looks like); this is what is said at the families; so in the case of birth spacing, indeed, there is that our community doesn’t peel it yet; there is long stay thinking by the community that says you have to have relatives that can gear you well; but here, it is not considered with that delivering mother about what hurt can happen to her; it is only seen to the number of the relatives; it doesn’t know either the delivering mother is being hurt or. On the other hand, the awareness about what importance has that birth spacing in the social and economic condition and in the health of the mother is limited. Thus, we also work to introduce those family planning methods in the trainings we give. Ehhh…giving birth at longer space has for the mother and for the baby let them be healthy; you will not be hurt economically; it can also let those newly born babies to reach at the intended condition well. Then, for our schools, we think that it would be better be provided as portion or chapter though it couldn’t have come as its own curriculum; because, it would to bring those advantages we mentioned. Thus, you have to stand from the socio-economic condition of the environment; thus, it has to know that if you have many children in number, you will be hurt economically; so, giving planned birth at least has something at economy and feeding condition; for example, if you have seven children, but if you feed only two children, then it is problematic with your socio-economic status that you have. Thus, we give this program at all our schools that family planning methods have to be introduced at the community and at household levels. And at health, those HEWs let the lactating mothers go having that family planning method. Hence, it is crucial for health; it is also crucial for economy; the mother is not hurt, the baby is not hurt; and the community is not also hurt. But we are not moving sustainably.

**I: Thus, how do you see this work that of early marriage prevention and increasing birth spacing; is that successful?**

**P**: (He soothed his throat); now, we can’t say it is totally peeled; like in the case of below-age marriage, there may present missed ones; it is difficult to say we controlled it 100%. There may present scram ones; even we found them in control, they say that their age is reached to be married; especially those who have not birth certificates; even, you let neighbors be witness, they tell you that her age is reached; so, what can you do here? Thus, there are adolescent girls who are scrammed and enter to the danger; and also our community is a bit far from that of something thought about it due to living at far rural; and also due to absence of exposure to the modernization like watching television and like that. And this is because something is still left about creating awareness to the community; and it can’t be said then the problem is totally peeled; it is a bit difficult to say we introduced it; something is left yet.

**I: Are there any community factors that hinder for this work to b successful?**

**P**: Yes, there are; for example, when we say that the female has to learn; the community says that what did Mr. someone’s son or daughter do after completing grade 10^th^ or like that they say; due to observing at some lazy individuals fail at grade 10^th^ and get back to a kebelle then wander here and there at that area; which then such kind of things influence us to some extent. Then, there will be found who say that, it’s okay for her to have her marriage; because, what did do that Mr. someone’s daughter after competing her grade 10^th^.; because, it is observed that some students who didn’t achieve academically may get married; thus, this creates influence to the community; this is logic; they are not missed.

**I: How about at policy level; may there any challenging factor?**

**P**: Ayii (he laughed with nodding his head to express albescence of any factor); there is no problem at policy level. It is good it has not any problem (he laughed again). Our policy is good; it has not problem.

**Section 7: additional remarks**

**I: What lessons did you learn from the interventions that intend to improve maternal and adolescent nutrition at your woreda level?**

**P**: (He soothed his throat); so good; currently in the case of nutrition, as per to the science or academy, the activity undergone at school, we take that it has better change; at least, currently, there is a community which needs to have better feeding and better dressing; what is this is that, it is its alertness and the time that brings to it; it is due to such kind of opportunity that it brings to it. Currently, there is a community which needs to feed goo; except economically deficiency; having what is available with it, there is a community which needs to dress good, needs to eat well, and needs to drink well and others as well. Now, due to our academic provision and as provision of external trainings are going on, we can say that the nutrition of the community is good. Perhaps, economically those found today, may not be repeated tomorrow; but the knowhow is present; that is it; and also the feeding is still better; perhaps, there may present who rears hen, who practice vegetables and like that; yet, I cannot say it is utterly absent. As to the condition and time itself due to the extension of schools especially with us, there is extension done up to the villages and it can be taken that better at thinking is created too. And as bureau and as woreda education office together with other sectors as we said it, with better awareness platforms and events are being created; that nutrition has better change still; for example, mothers unless their weakness or other management technical something, there is no mother who is said to be died of due to food shortage; otherwise, the doctor is late or if she doesn’t come on time; unless, it is not due to shortage of food or not due to lack of balanced diet or like that, is not much; because, there is follow up by the HEWs and by us too that can have its own something; so, it is not said because she is due to shortage of food. But, at occasional coincidences as we said it, economically it is not missed that they have blood shortage and other shortages during delivery and like that. But that one, due to that food shortage, it can be taken that more or less it is not present.

**I: How about at the multi-sectoral collaboration, is there any lesson that you learned due you worked together or not?**

**P**: Ayii (to express presence of the collaboration), it is benefited us because of working together; especially with those stakeholders and sectors that we mentioned them, it benefited us. We take that there is better thinking; we can take that our student have better dressing; currently, you judge that their (female students) dressing and feeding is well; we can take that it is almost fine; that working in coordination and collaboration has better change; if we continue, we say that it has well acceptance and we think that it has good change; perhaps, if it goes in a strengthened way, what might be the outcome is; it will be seen in the future. But until then, working with the stakeholders at capacity building trainings and awareness creation platforms, is present better work that we take it. But as we said it, it has not gone a bit sustainably; yet, we can take that we are at better change or at better level.

**Section 7: Additional remarks**

**I: Thank you so much teacher; but if you have any additional idea that we didn’t raise; I can give you the chance; in my side, I am done with my questions and I am learned a lot from you; thank you!**

**P**: So good; (he soothed his throat), now undergoing such kind of study is, I think, it is to put solution at those visible problems; yeah, a study that is undergone to identify visible problems and to searching a solution together for them is good; and we as woreda, I think, we are mentioning that what has been done until then and what problems to we have that we need support and solution for the future; thus currently, our starts have to be encouraged even up to the individual level; may be as gave me the information it is from Mekelle University; and if you give us even your full address it is good that we will have it; whatever so, I say that this study has to get back to us and has to be our supportive; ehhh…when we say this, the work that you work it collaboratively and being together is, we believe that is for one prosperity. Hence, as the information we provided, that is it, and those that we did, we have to maintain; and those not done yet, we have to go through them. Indeed, we were not ready; we would have been aware (he laughed as if he missed to tell information); but what is needed now is the logic; what is needed is the logic which is at the ground. Thus, what we are doing at the ground is, obviously we tried it; anyway, based on the information we gave, we need this data to be well documented to be used back for the woreda at least for those wealth that we have at our hands; and we also expect other stakeholders to come to us; so, we expect this as a woreda. Thank you.

**I: Thank you**

**Summary (home take messages)**

**Section 1: Common maternal (pregnant women, lactating women and adolescent girls) nutrition problems in the community.**

Food production is finely present; but unable to use it; thus, what it needs is that everybody at each level from agriculture, health, women affairs and other social unions, these then in creating platforms for developing awareness of the community about its nutrition is still needs a long way to go.

**Section 2: Nutrition priorities in the woreda**

As sector, we didn’t identify which mothers are being hurt; but we simply work at schools being together with women affairs and health sector.

**Section 3: Nutrition interventions that improve adolescent and maternal health**

There are school vegetables gardening as most of our schools are at hillsides where the soil is not fertile; they have not also that of water access.

**Section 4: Implementation challenges and community factors affecting access to nutrition interventions**

There are budget and transportation challenges to the woreda.

**Section 5: multi-sectoral collaboration to improve maternal and adolescent girls’ nutrition**

Running only for education may not bring change and we have to collaborate; because, the current generation, that young generation is becoming so modern about the way it thinks and dresses and eats.

**Section 6: Other intervention that influence adolescent and maternal nutrition and health outcomes**

The main target of preventing early marriage by the education bureau and woreda education office is to let the adolescent girls equal to those boys both in their academy and their nutrition.

**Section 7: additional remarks**

This study has to get back to us and has to be our supportive
