# Supplementary material for: Barriers to utilize nutrition interventions among lactating women in rural communities of Tigray, northern Ethiopia: An exploratory study
Source: PLoS One. 2021 Apr 30;16(4):e0250696. doi: 10.1371/journal.pone.0250696 (PMC8087028; doi:10.1371/journal.pone.0250696)
Supplement: S2 File — (ZIP) [file pone.0250696.s002.zip › S2_File.Doc/Woreda level and above key informants/116_IDI_MCH expert_Medebay Zana wored health office.docx]

Date:

IDI with :

Name of KT:

Place :

Position:

Education:

Name of interviewer:

Start time

End time

Experience:

**Section one: Common maternal nutrition problems**

I: We will start. Thank you for being voluntary. I will start from mothers, when i say mothers the purpose of the study are mothers; they might be pregnant, lactating or adolescents who age is between 10 to 19 years. what mothers themselves do to make their health complete? What did they do to keep herself at home out of the formal health services . what is your observation as an MCH expert of Medebay Zana?

P: What mothers do by themselves to keep their health particularly those at urban and semi urban; those who have better awareness, to avoid unnecessary pregnancy they use contraception. Secondly, they visit health facility and checked even there mothers who come more than seven of eight times. This are those who are close to urban and semi urban. In far rural, there are difficult places in our woreda and their awareness is not that much. Activities to keep their health is almost null. Here related to early marriage; together with women affairs there are good movement. Particularly in rural, if the girl is under age like 16 or 15 and stop her school they work to interrupt by providing information. they have good awareness on early marriage. Beyond this, they are not that much. What people do for a pregnant women which is special from the normal. what the pregnant women herself do. How do you see?

I: In the community, now it is at least they know a women should not be harmed, when must give birth a health facility. She need to make check up at health facility is understood well in the community. But, in its implementation, there are irregularities. In theory, they know what to do in pregnancy. For example, there is what we call water and soil conservation. The pregnant women is free at the community. She will be free from different labor works. They need only evidence of her pregnancy from health professional. they do not bring any reason.

I: What about in lactating women, is she has an infant?

P: It is similar, of course there is focus for less than 2 year. At least for the first 2 or 3 months similar to the employed mothers she will get rest. After that she will be treated as anyone else.

I: Well, what about adolescents? What did they do regarding nutrition and what the community recommend them to do in their age? Related what she must eat in terms of its frequency, duration. Is this common thing?

P: Here we have gaps. They rather say " she should not eat". They assume that they will grow fast and it is the time they start to feel shyness. There is a lot of gaps here. There is no work about this.

I: What are the common nutrition related health problems among mothers in Medebay Zana. What are the nutrition related problems in pregnant, lactating and adolescent one by one.

P: In adolescent it is i told you before. We mainly work with partners on them regarding family planning, prevention of unsafe abortion, prevention of HIV/STI. These are the services for them. In nutrition we did not work and it is not common. The common one is about infant feeding, complementary feeding. we did not work on adolescents. In pregnant and lactating[interruption]. Now in pregnant and lactating, our Woreda has no supply shortage, there good harvest. So, unless it is the lack of knowledge regarding how she should eat, there resource limitation cannot be a problem. In addition, we use to conduct trainings with some NGOs. If you ask to women, she will explain you well, but if you asked her how she is using you will find gaps in utilization. she may not get the required things. she may not get iodine. It is fine and the awareness is good but still we have some gaps.

I: Is there a problem of malnutrition in children, adult mothers and young. Are there conditions or seasons where this things will happen?

P: Now our focus is in children not in adults. We only give general health education. We do not have a mechanism to measure them. we work of mothers and under five. It is not exaggerated. As to the report we do not have exaggerated malnutrition. We do have plempy-net. we have few and they are not sever.

I: How much? It may not be in terms of number but which setting are affected more, is it related to food shortage or utilization or awareness. What do you thing is the source of malnutrition case seen children though they are few? what are the things that exposed to malnutrition. As you know this is among food secured area. How the case are occurring?

P: These case are coming for far places which is 50 km far from the Woreda. In these areas, husbands will go to other places as the topography of the areas bad to plough and crop that produce is only sorghum which is one type of food. They do not get sauce crops.

I: Is it Kola?

P: Yes, instead of eating with stew, they prefer to eat using ድቁስ. They are too far and will not get access to healthcare. she will come once from 3 or 4 hours walk to take planpy net and she will not come again due the distance. we have two Tabias having health problems which you may know it. Liver disease which is commonly seen around Zana and Asgede Thsimblae

I: Kalazar?

P: They call it HUD, it kill several people for long time and then as i told you due to lack of awareness of mothers despite they have food, they do not use, they provide other foods on top of breast feeding at 3 or 4 months.

I: Micronutrient deficiency, is there problems of anemia, goiter and night blindness

P: Goiter is not seen in children but there in adults to some extent. Micronutrient deficiency is good but there interruption of supply. Now we have brought. There were several mothers whom we refer due to lack of iron. Is there s no micronutrient supply, we will consider as deficiency. Because mothers are not getting what they are supposed to get. All under five children need to take. If it is not our store, mothers and children won't take and if they do to take there will be deficiency too. Now it is fine.

I: Out of the health service, how common is anemia in the community. Is there a risk of being anemic for women?

P: We have anemic mothers. Some of them will get treatment and some will be referred. We have also come cases of night blindness. Goiter is fine except for older mothers. It is not common in adult. Much is being done on nutrition together with donors and health extension workers. So, there is good change.

I: Related to non communicable; are there problems like hypertension, obesity in mothers

P: It is not much

I: Diabetic, hypertension?

P: Hypertension can happen in pregnant women due the pregnancy but we do not have much case treated or referred related to hypertension. Anyways, it is fine. Most of our referrals are due to anemia.

I: How is the prevalence of stunting in children and adults in the community?

P: We do not have a specific rate of Stunting In our Woreda but study report 44 %. But, we cannot say there is no stunting as long as a feeding problem exists.

I: How do evaluate and related stunting and wasting with nutrition?

P: Well, this is what we usually say. The community should get enough food. Breast feeding, complementary feeding until 2 years, monthly growth monitoring, early treatment during illness, additional food to mothers. It is proved that stunting and wasting are due to problems in these areas. It is known that they are outcomes of malnutrition.

I: Are these seasons for food insecurity?

P: It is fine for the last 3 to 5 years there is no food insecurity in our Woreda though it happens in other places. The is an assessment made by the food security office to identify top three Tabias. There is support given to more than 300 families. We have identified areas near to Yechila and support we given to them. Just when you see it mostly the far and lowland areas the affect one. We do not reach the far places and the donors also do not reach the far areas. Mostly there is a message which is left in space; without reaching to them.

I: Well, compared to male how the risk of women to malnutrition

P: Yes, when we look their physical to the meeting, most women looks uncomfortable. There is influence. They have a workload at home. They are not like the males. They won't eat what they get. They will wait their husband all the day even if he when to party where food will be served such as wedding or holy events. There is culture of not eating in the absence of husband. Again if she went to market in the morning, she will eat up on her return to home during the night. They cannot buy and eat from outside, particularly in our Woreda. this is not common. They went carrying the baby, they breast feeding, they work and even if she has some food it with her, she will feel shy to eat publicly. They try to cover themselves while eating to be out people's focus. The culture is yet unimproved.

I: What about students or adolescent girl age 10 to 19 years. How do see their exposure to malnutrition.

P: We do not have any study. We can talk only from our observation. I do not what to say

I: Have you seen cases of malnutrition in adolescents from your report?

P: We do not have on our report

**Section two: Nutrition priorities**

I: What are the priorities on maternal nutrition in your Woreda?

P:In mothers, our priorities are creating awareness, securing important supplies, women conference and food demonstration for pregnant and lactating women on monthly bases. We also provide training to religious leaders regarding fasting for pregnant women which was lead by save the children and it was a nice training and we create understanding. We have activities related to this.

I: As a Woreda, is there a resource or budget for nutrition? Compared to other service do you think that nutrition is getting better or equal attention?

P:There is no budget. We use the available opportunities. There is a budget for prevention. We use from that source if you develop an action plan. Otherwise there is no specific budget for nutrition only.

I: What about plan about nutrition? How do you plan to implement nutrition services? How do secure resource?

P: At Woreda there is nutrition expert which works focusing on this issue. they have plan for CMAM, SAM. The plan will be there. another there is a plan on food demonstration; when, where and how many. This will evaluated. Similarly in children, there is a plan to provide vitamin A, deworming, measuring MUAC, weight for 17% of the children every month. Of course there some interruption here. There some gaps but there is plan for that.

I: You have mentioned some cultural influences such as feeling shy. Is there any priority you plan on this Woreda similar to skilled delivery

P:It is the same. Nutrition is one of the health extension packages. We will identify how to influence the issue through religious leaders or other acceptable people. We also have contact with the Woreda administrator. now it is becoming good. In theory, even the religious leader say she need to eat different foods. This attitude is becoming eroded. but the mothers themselves still do have several problems. if you give her to eat food restricted during fasting, she will not eat. There is gap in mothers, religious leaders and the husbands in terms of supply of food for his wife. They might not supply her with meat if she wants

I: Well, how do see the accessibility of those activities that we mention are being provided to improve women nutrition? Are the services accessible for all mothers; the young and the old

P: In terms of accessibility we have gaps. i mentioned it before. that is either they won't come or we fail to go to them. There are relevant messages which did not reach to mothers partly due to cancellation of programmed meeting related the local events such as religious events, funerals. we sometimes go with our checking their convenience of attend the events. It could be due to Government meetings, lack of transport or training, the people fail to attend our programs.

**Section three: Nutrition interventions**

I: Ok well, When we look at the services, how is the messages for making women aware being provided. who should teach them. How should the modality of teaching,; house to house, counseling

P: In ANC is no mother who give birth without making at least one visit. if you say when did they come some come at 3 months and the other come at six months and some come near to term. she is missing the services she supposed to get before 6 or 7 months. They have to start the visit at 3 months so that they will have 4 visits. We have health extension workers in each Tabia. There is a health guide and the health extension workers tell the message inside the manual to mothers and women development army. The manual is contains all important information. The manual is prepared in the way they mothers can understand it. If they health extension workers transmit the message, That is enough for them. By written and by picture. They get via different means. All community member knows that a pregnant women must get four ANC visit and take all kinds of screening services. There belief that having one ANC visit can be sufficient. Having one ANC visit is not sufficient to get the required services due shortage of supply. We have supervisors, health extension workers. we do not have shortage in terms of human power.

I: How do you see the advice given regarding extra food during pregnancy?

P:It is as i told you. all know about feeding. The issue is in the implementation. The theory; everyone knows it. If you ask them they will tell you pregnant women should eat take one extra meal and two extra meal when they are lactating, the child should get additional food in the form of soft porridge .All knows about it, for the if we are asked did they use or apply as it is recommended, we did not study and we do know it.

I: Ok, well, how do you the advice about home gardening?

P:Yes training is given in collaboration with Agriculture. After the training, there are so many individuals in the Woreda who have home garden. Therefore, it is about 40%. some take to market.

I: Ok, well, they do have the awareness, the problem is in implementation. For example take ANC Why she did not come after one visit. Is it the distance, or the road? What the community says as a reason? What are the challenges for the implementation of the advices?

P; As i told it is too far, ups and downs. Some visit up to 7 times particularity those who become pregnant for the first time. They feel a sort of and visit frequently to health facility.

I: What about services and advise given about sanitation and deworming

P: In terms of sanitation, our Woreda is model. There are far places with are not addressed but from other places they come to us to visit and take our experience. Now there is bit tendency of lagging behind

I: What are the challenges?

P: You can say this the problem but there a tendency of feeing bored, taking the issue less serious and close attachment with the health extension workers.

I: Is there malaria in this area

P: Yes the area is malarious. But we have ITN distributed based on the households.

I: Did they use it ?

P: They use

I; What about the deworming services?

P: For pregnant women will be given by the midwifery during ANC. For children they be given during outreach.

I: What about for adolescents?

P: In school some times in the form of campaign. There was a campaign this year but it is not common.

I: What about targeted supplementary feeding? Do you have people who are addressed through targeted supplementary feeding in the Woreda? I mean children or mothers who are given special foods such as Fafa, or people who are getting treatment or follow up at outpatient?

P: No

I: What about vitamin A supplementation? Are you providing them?

P: For pregnant, we did not give. Now it is already restricted. for children we provide. For 6 to 59 months. 17 % of them should get vitamin A monthly. There was some shortage. Yesterday, we have got the supply and we will continue to provide them.

I: Is it through campaign?

P: In the past it was given as a campaign through community health days. But, now from all children 17% should get per month. if we do like this at 6 months we will achieve 100% because all children did not reach six at the same time. one will be six month to day while the other will be tomorrow.

I: Is there a condition where the adolescents will get Vitamin A

P: No

I:Do you have youth friendly service? Services about sexual and reproductive health at health center.

P: All provides but there is shortage of space. Compared to the standard there is gap. youth friendly service is introduced to make all services for the at one center. the professional working there might be a health office who do not take abortion, family planning, HIV Counseling training, turn over and un availability of refreshment center. There is good start.

I: For the service stated, which once are considered unsuccessful regarding pregnant and lactating women and adolescent nutrition in the Woreda? Similarly what are the successful once as per your evaluation?

P:We do not have services which are not started? We did not start service regarding adolescent nutrition and there is no direction. we do not have awareness about that. The other services it will a matter of assessing how much we become successful. At least, we achieve more that 70% and we are left 30%.

**Section four and five: Community factors affecting access to maternal nutrition interventions**

I: Up on your effort to implement these services, what are the barriers in the community that limit the success of these services in could be awareness or attitude or culture ?

P: We are fine in other aspects. the problem that we identify as an obstacle is partner or male involvement. We have gap. When a mother get pregnant, they did not support her in terms of support, they will go to the farm leaving the women alone. These will limit the use of necessary services for mothers. Specially around zana, we have a village where the male will out of the house for months. we face difficult of even getting a person who will transport her to health facility during labor. Only women left at home. During term if she must come to the waiting room, who will care for children. It is challenging. Males do not come with their wife for ANC check up.

I: How did the males express their attitude about it

P: They took pregnancy as a women's job. They relate their status/ክብሪ" they consider it as effeminate/ሴታ ሴት" . We have included in our plan. we have much to do with them. they did not involved yet. [Mobile phone call] Because, If the male allows her to stay at the waiting room. She will be willing as she won't be out of her husband's interest. They create influence on the woman in terms of attitude and implementation. this our evaluation. Better service users are employed mothers.

[silence]

I: What do the community say for overweight mother, thin mother and about adolescent nutrition. Community may have their own description things that might affect nutrition.

P: regarding fat and thin, there is such talk in the community. But, as i told you before, a women will not ear before husband. And there are foods restricted for women.

I: What are these?

P: She cannot eat chicken if her husband is outside and again she won't eat if she do not have husband. There are also food that women are not allowed to eat in our Woreda which are harmful. for example "ሕልበት" and yoghurt as this will make the baby big and make the labor very difficult. This too traditional. We have encounter this condition when we ask them about the foods to be eaten during pregnancy. They say she should not eat white colored such as ሕልበት ,milk and yoghurt.

I:With what they relate the white colored foods

P: I do not know but they say she should not eat

I: What about in lactating, adolescents?

P: In adolescent it is as said you. There is not service or we do not have awareness

I: what the community says?

P: Of course there will people who may think that she will grow fast

I: So, what should be done to solve the existing challenges; cultural, failing to implement advices

P: A lot can be done in the community, particularly by providing video assisted teaching

I: What kind of video? what content?

P: A video containing nutrition and how to get good nutrition, several trainings in schools, for adolescents, trying to understand the thinking of adolescents. We did not effectively work on IYS but we can do a lot. In school as well we have to work and make discussion in schools how the young understand these issues.

**Section six: multi sectoral collaborations to improve maternal nutrition**

I: In the last point you mentioned about collaboration with other sectors; who should work in collaboration at Woreda, Kebele and Tabia level to improve the nutrition of the women and adolescents and what can be their role

P: Agriculture, school, women affair and health.

I: What should be their role?

P: Agriculture should be about home gardening, irrigation, crop production; women affair will arrange discussion forums about harmful practices such early marriage' school in establishing clubs at school, teachers should not entertain any one who is not allowed from the recognized authority. That is the government structure. Administration office, social affairs can also be contributors.

I: We can agree that they should work together. But, are they currently working in collaboration

P: There is a start but it needs a person who is formally assigned to coordinate all. we use to take trainings together nut our work schedule is different. so it is difficult to consistently meet and work effectively. we might have campaigns the schools will have exams.. so, it is important to have one coordinator

I; Is the a mechanism of evaluating the joint activities

P: Now, it is not strong. when somebody gathers us we will meet and discuss. when the administrator asks us about our job, every sector will report what they did. The women affairs work very close to health sector. There is no tangible work that can be considered done by the collaboration. It is weak anyways

I: How is the resistance of the sectors for collaboration; the tendency of being confined to your own work, the collaboration also need a budget. What challenges you observe for the collaboration.

P: We did not take it seriously. there is no a kind of resistance since we do not have common plan and did not attempt to work together in organized way. There is no a package that we try to implement and failed.

I: What about at Woreda level? Is there nutrition coordinating body?

P: There is

I: Who are the members?

P: The nutrition expert from us, Food security and from other sectors. They evaluate every six months? i do not know much on this

I: Did the expert bring what is discussed with the team

P: He might report to the head. i do not know

I: Ok, well as per your evaluation is the platform strong?

P: The coordinating body is the food security. It is not something to be appreciated.

I: What else about adolescent intervention may be from your office and support from other donors

P; In adolescents as i told you, there is no service, But, on maternal health, there is support and we conduct evaluation together and we notice the changes.

I: Is there a work on early marriage?

P: yes, there is. The women affairs and school offices and health extension workers in each Tabia are the members of the committee. A lot of girls are saved. they did it in hidden way which they call it "ሙለቅ". They push to get married internally. Any ways the student from school, are working on it. they provide information about it to the committee. Due to several girls are saved from being married.

I: What is Mulk, is it to mean divorced?

P: They got married without any ceremony and without the awareness of the community. Once she get married, they went to court and of course stop the process.

I: what are the major activities do ne to prevent this problem

p: Mainly it is done thorough schools. There are clubs with the school. the teachers will coordinate. If they find cases they find cases communicate to the committee and check the condition together with school director. they will try to convince the community to stop it. If they did not accept it they will go to court. It is women who coordinate it at Woreda level.

P: OK, well, why the community are pretending to practice early marriage?

P: They want to have a child, to get a heir if the parent are getting old. To see the their children get married" ብርሃን ንምርአይ"

I: What is the tolerable age limit to get married by the community?

P: Now the community has awareness about it. It is already declared and there is a stand on that. It is 18 years and they accept it but some girls get married at 12 year, 14 and 15 years. All the members of the community at various levels know it. Nothing will be hidden. The system works up to Tabia level.

I: What policies do you have to avoid the practices at the bottom level

P: They law is the same across all level

I: Is there a law that is added regarding the punishments for those who commit early marriage.

P: There is no, It is based on the law

I: Ok well what the community belief regarding delay in birth spacing. How did they perceive its advantage to the women?

p: It is fine. there are some mothers in lowland who give birth after one year their marriage. most of them they did not give birth for 7 to 8 years. they continue their education after marriage. sO, IT IS FINE.

I: How the birth spacing and family planning use. It is good utilization

P:As a Woreda we achieve 70%

I: Do the community believe having more children as a good thing.

P: The communities interest like that. There women who use Family planning in hidden was without disclosing to their husbands. There is huge effort on that. we provide home to home counseling, health education and awareness activities. The women development army has good understanding. we need to work more to address some of the problems

I: Do we involve those who can have influence; is the way of delivering the message invites the males to send their wife and daughters to use family planning. How do you see?

P: We also work at schools similar to YFS particularly to avoid unwanted pregnancy. Beyond this there are parents who brought their daughters before marriage. They come and ask to let her get married but she will continue her education. They are good thinkers and there are who think their education is no important for women. They are mostly from far places whom we fail to address awareness creation activities. there are also girls who are not attending school despite they are supposed to attend. There also who interrupt their education and engaged in marriage. I do not when i think of addressing these area in the future. The health extension worker traveling 7 to 8 hours to give message is very difficult.

I: What are addition opportunities that can support efforts of dealing with early marriage.

P: The women development army. they are working as health professionals. this is good opportunity. the health post also goes down to the comminute. we are going closed to the community. we are inviting individuals for the lowest levels well. we are involving religious leaders as everyone goes to these institutions. We need to work more on that.

I: Ok well what lessons you got to improve the services as an MCH expert or something that you think that it is good do?

P: There was a good training given about blended nutrition. Particularly, regarding micronutrients, sources of iron, vitamin A, i get good lesson.

[Motor vehicle]

P: At least training the supervisors at health center, we have got a good understanding on nutrition. With the collaboration of others sectors much can be done . in fact we did not work.

I: Ok, well what about the lessons you got from the collaborations and find it important experience.

P: There is a common work that we achieve together, except the early marriage. We did a good job on that. we save 30 to 40 mothers who continue their education up to university level. some of them, they inform the plan of their family to the concerned body. But, in nutrition, there is no good job.

I: I have finished on my side. If you have ideas that you want to raise and suggest us to include in our study

P: Most of the time in maternal nutrition; since the donors and we ourselves are working on it, there is good work on that. However, as we said before we need to work on adolescent nutrition. I find it new for me. i want to say; we shall work with schools on what to do on them. Otherwise, in our Woreda, there is no such gap. If possible again we need support in areas that we cannot reach. UNICEF was supporting in this area, but i do not know currently. The support shall continue. We were getting materials for screening but now there is gap. During nutrition screening we get short of many materials. Otherwise much of it is mentioned

I: Ok, i thank you very much for the information you give me. It is important and for the study as well it is important.

P:Thank you for coming and asking us. This data of interview might bring something and thank you.
