# Supplementary material for: Barriers to utilize nutrition interventions among lactating women in rural communities of Tigray, northern Ethiopia: An exploratory study
Source: PLoS One. 2021 Apr 30;16(4):e0250696. doi: 10.1371/journal.pone.0250696 (PMC8087028; doi:10.1371/journal.pone.0250696)
Supplement: S2 File — (ZIP) [file pone.0250696.s002.zip › S2_File.Doc/Woreda level and above key informants/117_IDI_Head of Water Resource Office Head_Lalay Maychew.docx]

## Lealay Machew Woreda – Water Resource Office Head KII

| **Introduction:**  Hello, my name is **Dejen Yemane**. I am from Mekelle University. Thank you for taking the time to speak with me today. We are doing research on the factors that influence the nutrition of mothers and adolescents in collaboration with the Regional Health Bureau and UNICEF. Your participation is very valuable. The things that you tell us will be used to improve nutrition programs and services for women in the region and the country. We will not share your names when we report our results.  However, I will record the discussion so that I can capture all the ideas that are shared. I have several questions to ask you that I have prepared in advance, and I will ask you to say what you think about each question. The interview will last for 1:30 -2:00 hours. Do you have any questions before we begin? If you think of any questions as we proceed, please feel free to let me know. If it is all right with you, I will turn on the tape recorder now.  **Section A: Interview details**   1. Zone: **Central** 2. Woreda: **Lealay Machew** 3. Kebele: **__________** 4. Name of key informant: **Amare Abraha** 5. Institution of key informant: **Water Resource, Mining and Energy Office** 6. Interviewer name: **Dejen Yemane** 7. Date of interview: **November 20, 2017** 8. Interview start time: _________________________ 9. Interview end time: __________________________ |
| --- |
| **Section B: Interviewee professional information**   1. Gender    1. Female    2. **Male** 2. Age: **50** yrs 3. Highest level of completed education.    1. No formal education    2. Primary education    3. High school    4. College education    5. **Bachelor degree**    6. Master’s degree 4. Current job/position: **Water Resource, Mining and Energy Office Head** 5. How long have you been in the current job/position?    1. ______ Months    2. **Three** Years |

**Section 1: Common maternal (pregnant women, lactating women and adolescent girls) nutrition problems in the community.**

**Interviewer: In your opinion, what are the common nutrition problems among pregnant/lactating women and adolescent girls in your community?**

**Participant:** well, though I don’t have exposure on health-related interventions, before I proceed to the problem, people consider food is injera, bread, or others but since water is very important there are efforts to make water accessible. But before I proceed to the problem though there are some problems in the community, especially our democratic and developmental government has designed strategies to minimize the distance a woman goes to fetch water and to avail clean water by providing water alternatives. Previously the recommended distance was with in 2km radius but now we are working to achieve access to adequate water with in 1km radius. As a Woreda we have 464 water sources serving the community. We add chlorines to these water sources quarterly to prevent water borne diseases and in collaboration with health sector we check the quality of the water sources by taking samples so that it will help us to take necessary measures. Coming to nutrition, it is not about quantity of the food rather it is food variety, and this is not yet solved and needs much effort. Though it is available and there is change but giving vegetables, fruits, milk and egg products to mothers is not yet solved. Though it is not completely solved, there is also change in the feeding and wearing style of the community because of the national growth. Especially as water resource office since irrigation is part of our work, we need to see women to participate in irrigation activities by conserving water and planting home gardens that can be harvested with in very short period. There are efforts farmers to supply their family with substantial quantities of a variety of foods through home gardening and from the home garden they can be sold for income to buy other essential things. But it still needs further work collaboratively with stakeholders in changing the attitude of the community so that they will optimally utilize every drop of water for home gardening to plant onion, vegetables like salad, swiss chard, carrot and potato. There are many efforts to change behavior of farmers to have home gardens not only in the summer even in the autumn season.

In the past there was hunger but now we don’t have households suffering of hunger. The problem is not on the quantity rather in the variety of foods. Even though they have at hand, they do not eat enough of the right kinds of food.

**Interviewer: you told me that it is not about unavailability of food rather it is lack of variety and low awareness that not yet solved? So, could you please tell me common nutrition problems among pregnant/lactating women and adolescent girls in your community that are associated with not eating variety of foods? For example, anemia, night blindness, goiter, wasting and stunting**

**Participant:** the national development by itself is changing things from time to time but it is not sufficient. Maybe due to our utilization there is goiter problem that we see occasionally. From my observation, regarding stunting the genetic makeup is changing from time to time though I did not scientifically measure the weight and height. Overweight is not a problem in our Woreda except rarely in urban areas.

**Interviewer: what about wasting in pregnant/lactating women and adolescent girls?**

**Participant:** wasting is common in children of mothers who have health problems due to malaria and cold conditions (ናይ ቁሪ ሕማም). This are due to lack of optimal breastfeeding. Wasting is commonly seen if the women is not properly breastfeeding her child and has food shortage. But such symptoms are seen in not more than 5%.

**Interviewer: Do you think there could be any association between nutrition and occurrence of non-communicable diseases among the women/girls? Are there such diseases in this community? For example, diabetes, cancer and hypertension.**

**Participant:** though it is rare there are cancer cases who have gone to Ayder referral hospital. But their number is insignificant. Similarly, there is also hypertension. Still it is rear but there is diabetes in school children.

**Interviewer: Is there a situation when women suffer from shortage of food?**

**Participant:** since the community is almost farmers they are dependent on rainfall distribution. Therefore, if the farmer doesn’t have reserve at home sometimes due to snow and pests especially in dry areas found in lowlands there happens food shortage.

**Interviewer: which kebelles are more prone to food shortage in your Woreda?**

**Participant:** in kebelles like Welel, Awleo, and Natk bilae because they are lowlands and if there is short summer the grains dry quickly, and the community will suffer shortage of food. Generally, to compensate such things the farmers are using irrigation. In such situations a committee led by Agriculture and rural development office, we study to know the intensity of the problem and based on that government will support from the reserve food grains to avoid death and school dropouts dye to shortage of food.

**Interviewer: though there is change there is still stunting, wasting anemia, night blindness and goiter. Tell me comparing illiterate vs literate, rich and poor, male vs female, Christian vs Muslim, pregnant vs lactating, pregnant vs adolescent,**

**Participant:** just guess, health problems are common in lower and upper age. In children it is because their feeding and prevention capacity. To grow healthy there are food requirements and there is also immunity comptonization. From my observation, goiter is more prevalent among females. In rich households there are rich people diseases like diabetes due to use of excess fat and sugar. Whereas, stunting and wasting are common in poor households. However, there is no economic disparity among the farmers because their source of income is land and the land are equitably distributed what makes difference is their effort and using technological inputs.

**Interviewer: which do you think is** **more affected by the mentioned nutritional problems (pregnant, lactating and adolescents)?**

**Participant:** the worst effect is on lactating women because she had blood loss during delivery, secondly being breastfeeding by itself compounded with the life style affects her more. Because whatever food she took is shared by her child. Therefore, special treatment and support is needed to pregnant and lactating women. But culturally the mother gives priority to her children and husband than herself. There is tradition a mother to eat first before she feed her family. Understanding this, we should support our mothers because being pregnant and lactating needs extra food and rest more than anybody else. There are changes due to consistent health education by the health professionals at schools and churches. Though there is implementation gap that needs further effort, HEWs teach us using different posters that promote feeding pregnant and lactating women mixing from carrot, vegetables, potato, meat, egg and milk.

**Interviewer: why are these implementation gaps happened?**

**Participant:** because you can’t solve the existing perceptions. We are saying there is change but it should be beyond that. Though there are many attempts but still the problems are persisting. Nobody can give love to his/her family like mothers.

**Interviewer: is stunting common in pregnant/lactating women and adolescents?**

**Participant:** if we say there is stunting in the community it is common to see in women and adolescents regardless of sex and age. If you are stunted during your childhood, then it will appear when you reach adulthood. Your adulthood structure the reflection of your childhood status. To bring change on stunting interventions should start from conception. During pregnancy and lactation period the mother should be adequately fed because until the 2^nd^ birthday of the child the greatest food of the child is mother’s breast milk. In addition, child feeding practice is detrimental, because there is no that much growth after the age of 18-20 years. Therefore, to end stunting we need to work hard on child and maternal nutrition.

**Summary**

- The common nutrition problems among pregnant/lactating women and adolescent girls are goiter, anemia, stunting and wasting and lactating women are more affected by those nutritional problems because they have blood loss during delivery and are breastfeeding.
- There is no food shortage and starvation, everybody is eating at least three times but still there is a problem in eating balanced diet due to attitudinal problem
- AWD, different skin diseases, intestinal worms and opportunistic infections are the commonest communicable diseases whereas, cancer, hypertension and diabetes in the woreda

**Section 2: Nutrition priorities in the Woreda**

**Interviewer: In your opinion, what** **priorities do your institution has in relation to maternal and adolescent health? Why?**

**Participant:** I will tell you focusing on four pillars of the office. These are irrigation, drinking water, mineral mining, and energy. Great works are being done by our democratic and developmental government focusing on policies and strategies that increase participation of women and youth. There are clear classifications, for example, women, farmer and youth and our reports are evaluated based on that.

In the irrigation activities, we provide irrigation alternatives especially for female headed household. If we have one or two wells we give priority to women who are heading households to change her life. In our Woreda there are women who produce irrigation products more than men, for example, in Medego kebelle there are special women whose children are in the highest level.

In the drinking water, most of the time traditionally women are responsible to fetch water though there are significant changes because there are few men involved in fetching water. In rural areas, when a man goes to rivers to water the animals they bring water via donkey on their way. Previously access to water was measured by the availability of water at 2km radius whereas now there are efforts to avail water alternatives at 1km radius. As a Woreda, 52% of our community get water at its 1km radius. Previously we were reporting the water access was 80%, but in GTP-II the government is working to avail water alternatives with in 1km radius in collaboration with development partners especially REST and WASH projects. Our Woreda is included in such initiatives and currently REST is digging water in 4 sites. The government is giving much attention to water. Secondly in schools, if a child is diseased s/he will transmit it his/her mother therefor we are working to avail water alternatives at each school. Of the 4 sites I mentioned the two sites are with in schools.

**Interviewer: what health problems could be occurred in women and adolescents due to inaccessibility and shortage of water?**

**Participant:** if water is not accessible, women will not take rest and especially if she is pregnant she will carry both the fetus within and jerrycan, then if water source at a far distant women and fetus will be affected. If it is in their proximity, though you can not avoid the effects it can be minimized, and they will get a chance to take rest. If the water is not accessible the household sanitation and hygiene is also affected because they can not wash their cloths, body and cleaning their household. Therefore, if water is accessible you can keep environmental and personal hygiene and you can prevent different communicable diseases. Though, they are insignificant in number, there are households who have self-supply by digging wells at their compound. For example, if they have roof catchment though they can’t use it for drinking purpose it can use for washing purposes. Though it is rare it is already started, and we have a plan to work on that. If the farmer is supported cement they can easily give you the labor for free. The problem is it is difficult for the farmers to afford expenses for sand, stone and cement if development partners support us with funds the farmers can easily implement it. Water requirement for drinking water is not that much rather washing and animal consumption are water consuming.

If water is contaminated, it leads to different health problems. As water is inaccessible there is a probability of contamination during transportation especially if the water collection material has no cover. Therefore, if it is accessible contamination can be also minimized. In addition, water needs protection, fencing, lock and rules but if it is far from the households its sustainability issue is questionable because it is difficult to protect it. For your surprise, the policy of the government also directs water to be administered by women. There are 6 committee members in the water committee and we are constituting the committee to have 4 women members, especially the chair of the committee is woman. We have water committee at kebelle level and the chair is woman. In the water committee the deputy is female, youth office, women association, therefore more than half is female and practically we are working that way. If it is not possible women will be equally represented in the committee and the committee will be led by the women because the problem affects women more. On top of that if women are convinced, water need money. If a woman convinces her family and the family can contribute up to 120 birrs per year for maintenance, fencing, treatment and salary of the guards. If she is convinced the 120 birrs is very simple because it the cost of half hen or 5-6 plates of teff.

Thirdly, in energy. As it is promoted in medias and schools we need to use green/renewable energy. For survival we need food, cloth and shelter. To eat you need to have food but food alone is not enough by itself. If it is not cooked it has impacts and for cooking purpose, we need fire wood. To get fire wood we need to cut plants and if you cut plants the environmental contamination will be increased, and such contaminated environment will affect women. Because plants naturally take carbon dioxide and gives oxygen then it helps in protecting health of the community and prevents environmental contamination. Therefore, though it is unavoidable we should minimize cutting trees and instead we should use energy efficient stoves. Easy stoves are energy efficient stoves that can be used by minimum fire woods and minimizes smoke. If you are using much fire woods, you will emit much smoke and the smoke will affect the lung of the mother and it will lead her to cancer. If you minimize smoke cancer disease can be minimized and this is called ‘killing two birds with one stone’ because her health will be maintained, and the contamination is reduced. It has also economic beneficence, for example if the family is buying fire wood they can save the amount of money spent for fire wood and for those who collect fire wood by themselves, they can sell and generate economic earnings. Therefor energy efficient stoves, increases 40-50% efficiency of energy utilization. Then the one who were using 100 kg fire wood will use 60 kg firewood then he will minimize his/her expenses. The cost of energy efficient stoves is 90 birrs. Furthermore, there is solar energy. In the past, when I was a student we were using lamp which was exposing us to respiratory problems but now in the rural areas they are using solar energy for lighting and cooking purposes. Because of using solar energy, children are comfortably studying and minimizing contamination. We have revolving fund as a Woreda that will be used for purchasing water supply equipment and energy supplies. Thirdly, we have biogas. Biogases will be fed mixture of cow dung and human feces and this will serve as latrine and source of energy. For those who constructed biogases government is subsidizing funds for labor and materials if the farmers could avail sand, stone and around 3000 birrs for cement. In urban areas you have dislodging cost but if you have biogas it can serve the family sustainably, maintains cleanliness and avoids energy cost.

Lastly, to empower women we involve them in mining activities that lasts for one year. Youth association is responsible to organize them and if number of female members is less than 50% we don’t allow them.

**Interviewer: tell me the association of access to water, mining and energy to maternal and adolescent nutrition**

**Participant:** it is already known. If she has clean water at her proximity she can minimize water related diseases like AWD and different skin diseases. If there is contaminated water, there she be exposed to intestinal worms and opportunistic infections. In addition, if you provide them clean energy you are preventing respiratory health problems. Similarly, if they are involved in mining activities they will be economically empowered and as a result they will maintain their environmental and personal cleanliness and purchase foods. Lastly, if they are involved in irrigation activities they can get variety of vegetables from their home gardens and they can also sell vegetables and purchase their needs like hen and meat.

**Interviewer: What nutrition interventions have the most resources allocated your institution?**

**Participant:** most of our resources are invested in drinking water. The biggest health problem is associated with water and access to clean water has the biggest role in maternal health. If there is no water, you cannot think of irrigation. If there is no access to water contaminated water will leads to poor work ability and illness, illness means visiting the health center and too many visits to the health center mean lost working time as well as lost money and to the worst you will die. If there is access to water the community can use it for irrigation, and if there is irrigation they will have income and if s/he economically empowered they can purchase energy efficient stove or solar energy. By the way, mining is not sustainable opportunity, it is temporary because the resource is not renewables. Generally, water is very important even your carcass needs water and for the mother it is important to keep her child’s cleanliness.

**Intervention: Do you think is necessary for your institution to get involved in work aimed at improving nutrition among women and adolescents? Why? If you think it is important, how do you evaluate your participation?**

**Participant:** there is no doubt that participation of our institution would improving nutrition among women and adolescents. Because water is important to maintain health if the mother is not healthy the family will be affected. We are promoting to participate on irrigation and mining activities and to dig well in their compound to use it for different purposes. If the household loves their family we encourage them to create alternative water sources, to handle water properly and to treat it. We also mobilize the community to contribute 120 birrs every year for maintenance, fencing and salary of guards. We have also more than 30 technical personnel. We have 16 kebelles and of them we support 15 kebelles. In those 15 kebelles we have also 8 females and 7 males, technical personnel. The females are effective on their work.

**Summary**

- We promote energy efficient stoves and renewable energy like biogas and solar to minimize environmental contamination and respiratory health problems
- Water resource, mining and energy office priority to irrigation, safe drinking water supply, green energy and mining to improve health and empower economy of women and adolescent girls

**Section 3: Nutrition interventions that improve adolescent and maternal health**

**Interviewer: What kinds of nutrition interventions are in place to improve health of the pregnant/lactating women and adolescent girls to your level?**

**Participant:** the main thing is the policy. The policy promotes mother should not die, starved and diseased. There is also slogan that says, “a mother should not die while she gives life”. Based on that the Woreda council, offices and the justice office are collaboratively working. The water resource office has better implementation performance. HEWs are teaching the community in a home to home basis on sanitation and hygiene, institutional delivery integrated with other sectors. If there is home delivery, then the executive body assigned in that village will be evaluated. Health sector is promoting mothers to use ANC services because if they did not follow ANC she will face during delivery. If she is anemic or hypertensive, she should know ahead. If she is hypertensive, HEWs, advise them to avoid fat, salt and sugar. I am not health expert but from our meetings I understand what is going on. We the council members also supports kebelles and during our supportive supervisions we don’t only look on water issues we look also education, health, agriculture, and women empowerment. We are promoting institutional delivery, but we need to construct enough waiting rooms, sanitation and water facilities. In addition, the community is also contributing in kind or on money to prepare porridge in health facility. Not only that, there should be road access so that she will get ambulance service. Ambulance drivers are always on call to give service to pregnant mothers and some pregnant mothers even come to health facility a week ahead.

As education office, teachers always teach students to keep their health through the health clubs. The nitration issues are part of our curriculums. Except its depth students are thought starting from grade 1. Because our students are thought about digestion system and which kinds of foods are rich in carbohydrate, fat, vitamin and proteins. In addition, they are thought which kinds of foods produce energy, promote the growth/development and repair of tissue, promote resistance against diseases and maintain and regulate vital functions of the body.

Agriculture office also promote communities to use irrigation, to boost their productivity, to use technologies.

**Interviewer: you told me that there are many interventions on pregnant and lactating women, what nutritional interventions are in place for adolescent girls**

**Participant:** as a Woreda we don’t have kidnapping practices and it is not also common in Tigray. Most of the interventions related to adolescents is given in schools. Fortunately, I have also served as head of education office, so I will tell you the interventions done in adolescents at schools. We teach them about the behavioral changes that occur during adolescence age because they have unique blessing the so-called ministration. We disseminate information for example, if she has unplanned sexual intercourse she will get pregnancy, if she is pregnant it will lead her for dropout and unwanted pregnancy is harmful for her. Mainly their uterus is not matured, and they are physically immature then the uterus can’t carry the fetus and we inform them that it will also lead them to fistula. We also teach the male adolescents not to abduct them because they are their sisters and mothers and they should give them the love they give for their mothers. We also teach to adolescent girls how to manage their menstruation especially at school, how to use sanitary pad, for how long does menstruation lasts, not to be absent from school and stressed. There is club where they discuss and share their experiences. There is peer to peer education and in such discussion the one who has experience will share her experiences.

**Interviewer: WASH facilities at school is very important for menstrual hygiene management, how do you see availability and adequacy of WASH facilities and sanitary pad**

**Participant:** in all schools there is separate latrine for male and female. If we did not avail separate latrines for females where is the equality and female affair? Unless otherwise it is a slogan. Therefor, they have separate latrine and it is must to avail water. If there is water, they can use it, but it is not complete because there are inconsistencies. But as an awareness we all, the teachers, families, students and the leaders know it what it should be. We also recruit cleaners for the latrines in every school.

**Interviewer: the reason why I asked you this is that, before three months there was a study that assess school WASH facilities, which I was involved. In our assessment we found most of the schools don’t have separate latrines and if any it is not clean, the student to latrine ratio is beyond standard, there were water infrastructure with out water … therefor, in such conditions we observed that adolescent girls to defecate openly which is associated with their dignity and sometimes they may also keep it until they return home which leads to urinary infections like kidney. So, what could be the main reason?**

**Participant:** this is changing because in the past using latrine was considered as humiliation for girls. Since most of the kebelles are rural, in most of the schools there is no latrine with piped water. The schools have hand dug well or shallow well and the adolescent girls use water from that. But if the development allows it would have been good to collect water in water towers and connecting with every latrine. But we don’t have enough water supply though there is water in the school compound at least hand pump which is not adequate to the number of users.

**Interviewer: In your opinion, which of the above interventions for the pregnant/lactating women and adolescent girls are being implemented in successful way and which interventions are implemented unsuccessfully? Why? For example (counseling for food diversification, extra meal, checkup and services, Insecticide treated bed nets, Targeted supplementary feeding, advice on WASH, ANC, institutional delivery and PNC)**

**Participant:** we are successful in ANC, PNC and institutional delivery though it is not 100%. But we are not successful in nutrition. Though they take adequate quantity it lacks variety. Individuals can talk but if you go practically they don’t provide variety food consisting honey, egg and milk even though it is available. Instead of eating the butter and hen they prefer to take it to market. Therefore, we should work hard to change such behaviors that prioritize income than consumption pathway.

**Interviewer: Is there a situation in this community how that you think that women need to be addressed through Targeted supplementary feeding (TSF) for women? Why? Could you tell me specific examples and situations?**

**Participant:** this can be better answered by health personnel. They have balances and they are weighed but you can get better information from health personnel.

**Summary**

- Nutrition interventions that improve adolescent and maternal health in Lealay Machew Woreda are ANC, PNC, institutional delivery, nutrition counselling, home gardening, and WASH.
- ANC, PNC and institutional delivery are the most effective intervention and food diversification interventions are the less effective once.
- The nitration issues are part of primary and secondary school curriculums and students are thought about digestion system, food stuffs and which kinds of foods produce energy, promote the growth/development and repair of tissue, promote resistance against diseases and maintain and regulate vital functions of the body.
- Adolescent girls are thought how to manage their menstruation especially at school, how to use sanitary pad, for how long does menstruation lasts, not to be absent from school.

**Section 4:** **Implementation challenges and community factors affecting access to nutrition interventions**

**Interviewer: What are the challenges to implement delivering the nutrition interventions that we have been discussing for the pregnant/lactating women and adolescent girls? When you answer think of individual level factors, community level, intervention level.**

**Participant:** I will try to answer to your question focusing in four things; government, community, family and individual level factors.

The government should avail water in every health posts and not availability only it should be clean. In addition, there should be latrine and adequate waiting rooms. Government should do this in collaboration with development partners. If the health centers are attractive and secured mothers who got service in those facilities will promote it. Development partners and government should avail water supplies in health facilities because there are gaps. We are using hand dug wells but instead it is better to collect water in water tankers and then the water will be supplied to each room via gravitational force. This are the bottle necks, so we need to solve this in collaboration. In addition, we are availing ambulances to bring only laboring mothers to health facilities. But, when pregnant women travel by public transports they will be harmed so if our development allows it is good to avail 2-3 ambulances in the woreda. Because one is not adequate. Secondly, after bringing the pregnant women for delivery we don’t give service to return them back home and this should be solved. They don’t have rest, and as to me if the other civil servants could work like health professional we could bring big change in our region. Health professionals are serving the community day and night but access to ambulances should be increased to serve them to bring them to health facility and return them back home. If they came by ambulance and returned back on foot, it will affect their institutional delivery utilization because there are many rituals in the 7^th^ day and baptism ceremonies. If you treat them well they will promote it and the community will be attracted.

As a community factor, there are areas where ambulance can not reach. In such areas there are tendencies to deliver at home because of inaccessibility to ambulances instead of bringing pregnant using traditional ambulances (stretcher) to place where ambulance can reach. They do it by campaigns in they leave it when the administrative activities are loos. There are such failures, so we need to aware the community.

In a family there are mother, father and children. A husband who loves his mother should love his wife. Because in delivery there is bleeding and if she bleeds, she is losing blood that will lead her to death. For example, leaf bean of a been will dry if it did not get water and similarly a mother will die if the bleeds. During delivery there is probability of birth complications, tear, and operation. Therefore, to avoid such birth complications we need to work on creating awareness on the family. The government should also create awareness through media like TV, radio and in schools.

It is the husband that would give rest for his wife. Therefor, our supporting culture for women should be improved especially during pregnancy. Especially husband should give special treatment during pregnancy either by recruiting servant or collecting water and fair woods by himself. In addition, by availing animal and plant source foods. Especially for taking rest husband is detrimental. In some households they even cut grasses and carry heavy loads while she was pregnant. If a pregnant is carrying loads both the fetus and the mother will be affected. In addition to the husband the pregnant woman should also give value for herself without modesty.

**Interviewer: what about at community level? do they give extra meals and animal source foods to pregnant and lactating women and is there gender disparity?**

**Participant:** currently there is no gender disparity and surprisingly the community is now loving to have female. This was there in the past, though it is not 100% it is significantly changed, and it needs further work.

**Interviewer: For these challenges that you mentioned, can you tell me of any solutions that your institution has applied to effectively implement the interventions for women and adolescent girls? Specify each solution done for each challenge. While your institution tries to solve the challenges, what problems do it faced? What do you think needs to be done to better address the challenges you have mentioned? How they can be addressed better?**

**Participant:** we all have job descriptions and our job description is availing water and energy. Even irrigation is not ours, our role is availing irrigation water. We are working on treat and maintain water supplies, by the way last year many people were affected by AWD. One of the challenges is that, our community is not willing to pay because they feel that why should I pay for naturally available resource. If the water scheme is broken it needs money for maintenance but still, there is challenge in the willingness to pay. We have 464 water sources. While you use them, it is obvious that they will be broken but our community don’t have habit of storing reserve water and paying annual 120 birrs payment. So, this is the greatest challenge for us. To solve this challenge, we are creating awareness at each level, but it is not yet solved. Especially after the occurrence of AWD the community is treating water and our workers are also shocked. What we did is we assigned accountable person from our office so that the assigned person will be evaluated for what will happen in that specific kebelle. In addition, to avoid home delivery the health professionals will follow.

**Summary**

- Lack of adequate water, latrine and waiting rooms in health facilities; ambulance service; willingness to pay for water service; road inaccessibility; and low husband support were mentioned as implementation challenges for nutritional interventions

**Section 5: Multi-sectoral collaboration to improve maternal nutrition**

**Interviewer: Do you feel it is necessary at your level to work with other sectors/institutions to address maternal and adolescent girls’ nutrition? Why? Which other sectors do you feel are necessary to work with your institution?**

**Participant:** we are working in interface. For the question do you think it is necessary, yes, it is necessary. I have told you, availing water is role of the water sector. Health sector alone can’t do everything. If you take AWD, the health problem is due to unclean water. Therefore, there should be contribution of water resource. In addition, there should be variety of foods. Even though you have money if you buy vegetables from far distant market it will die until it reaches home. Therefore, water resource and agriculture should work in collaboration on irrigation and home gardening. If water resource avails irrigation water, then agricultural productivity will be increased then this will boost the economy. Schools should also promote personal hygiene and home gardening. If we work on schools, students can transmit information to their family in the so-called family discussion (መኣዲ ምይይጥ). When they eat dinner the father, mother, adolescents and the student will discuss on what they have been thought at school. We also promote on the Parents’ Day. Media and public relations office of the woreda should also be involved. In addition, religious leaders should be involved because every household has faith fathers and the community complies to what their faith fathers say. If faith fathers orders to fast or work they obey, and they listen them more than health, education, agriculture and water experts. Therefore, to bring behavioral and attitudinal changes it is good to involve religious leaders. To benefit women, government is organizing women in league, association and women affairs. Similarly, it needs collaboration with youth office. Therefore, there is multi-sectoral collaboration among health office, agriculture office, education office, water resource office, public relation office, women affairs office, youth office, and associations. In addition, it is good to include TPLF and EPRDF party leading the regional and national government respectively because they have members in the grassroot level and these members have higher convincing ability because they have better understanding compared to the community. We were bringing change but if we integrate these all we can bring better changes in short period of time.

**Interviewer: How do you see the other institutions’ roles complementing your role in improving maternal and adolescent nutrition?**

**Participant:** I am happy because in all aspects but specially in we did a good job in bringing women in to mining activities. Similarly, previously efficient energy stoves and solar energy were not known but now we came to consensus. Every water sites should have reserve of 5000-6000 birrs but in our woreda there are kebelles who collected up to 20000 birrs. Yesterday, there was water problem in Diege because board is broken due to electricity and to maintain it we have assigned technical persons to bring material for maintenance. Because water shortage is becoming big good governance issue. Due to lack of understanding of the work people complain at our office. Thus, to solve the problem we discussed with the Diege town municipality to maintain from the budget they have, and the municipality promised to give us 500000 birrs. Therefore, we are trying our best to supply water and government is training water experts. There are also electro-mechanical experts that would support us. The regional water resource, energy and mining office has also given us mandate to identify 10 women who can avail sand and stone to construct biogas for free. If work collaboratively, for example we are working on biogas, but it will benefit for health also because if there is biogas there is latrine. But HEWs are going home to home to promote latrine construction. If they construct the traditional latrine it may collapse of be affected by termites, but latrines connected with biogas tankers will last long because it is made of concrete. In urban areas when the tanker is full it needs dislodging cost. But latrines connected with biogas will not only serve as source of energy for lighting and cooking purposes, the biggest benefit is economic empowerment. Because the biogas sludge serves as a fertilizer because it produces humus/compost. This humus is free of pests and weeds. We were invited by Mr. Leake, a model farmer living in Debrekal kebelle who implemented energy efficient stove, biogas and solar energy to see grains grown by artificial fertilizer and humus. The one grown with humus has higher productivity than the artificial fertilizer. Secondly, humus is natural manure it is not artificial. Grains grown with this natural manure is very important for the health of the mother. I recommend the farmers not to sell grains grown with natural manures because though all grains are equally sold in the market grains grown with natural manures are healthier.

**Interviewer: you have mentioned many stakeholders, for multi-sectoral action that effectively works** **to improve maternal and adolescent nutrition at your level, what kind of change in terms of the way stakeholders work together is needed? What type of resistance to the needed change do you perceive, or have you experienced so far?**

**Participant:** the existing collaboration is enough rather it needs strengthening and this can be strengthened by the woreda head. Because he is the one who lead the activities as a chair person. It needs also capacity building and the different sectors must be aware of stunting is an indicator of backwardness. Because stunting is contradicting with our development and it affects our image we need to end stunting. The wealth and nutritional status of my family when I was kid and my wealth and nutritional status is incomparable. This shows there is change but it is not enough, it needs much work. I use ‘shiro’ but if sell it in the market bean has the highest price even more expensive than teff. So, by selling beans you can purchase egg and vegetables, what matters is the attitude unless otherwise the wealth is at the hand of the farmer. Thus, to bring change public relation and education offices must work hard. If the teacher transmits in the form of drama, text and if medias participate we can change it because we have all at hand. There is no food shortage and starvation, everybody is eating at least three times but still there is a problem in eating balanced diet due to attitudinal problem.

**Interviewer: would you please elaborate more about the capacity buildings? What opportunities do exist to promote multi-sectoral coordination of nutrition in this woreda?**

**Participant:** army must be trained but when you deploy them they will not sit down because they are once trained, they will do exercise day and night to improve their capacity from time to time and to defend and attack their enemies. Though there are many platforms but there is sense of forgetting things and lack of transmission ability. By the way I have many experiences because I have worked in USAID and from my experience I learned that you need to give education separating your audience in sex, age. For example, if you bring an adolescent and a woman together they can’t raise question and answer for questions, therefore you need to identify your audiences. To do that you need to raise the WH questions like where, when, whom and why to teach. There is a proverb that says, “repetition is the medicine of medicine”, so we need to repeatedly tell the community. Health bureau cannot do anything alone, so Universities and development partners should collaboratively work with the bureau.

There is an opportunity that promote multi-sectoral coordination of nutrition in our woreda because the committees and council members evaluate achievements of every sector quarterly, biannually and annually and gives direction on the identifies problems. If there are families who forbid to go to health institution and promote home delivery, we punish them in collaboration with justice office.

**Summary**

- In Lealay Machew Woreda there is good multi-sectoral collaboration among media, public relations office, justice office, religious leaders, health office, agriculture office, education office, water resource office, women affairs office, youth office and associations to improve maternal nutrition and adolescent nutrition.
- Existing collaboration is enough to improve maternal and adolescent nutrition, but it needs strengthening and to do that it should be led by woreda head with the understanding stunting is an indicator of backwardness and it is affecting the country’s image.

**Section 6: Other interventions that influence adolescent and maternal nutrition and health outcomes**

**Interviewer: Do you think delaying the age at first birth to after 18 is better for the health of the women? How? What other benefits does it have for the women? What about for the baby? Does this delay would have a benefit to the nutritional status of the women? Do you think this message is being promoted in the community? Who are working on it? How do they promote?**

**Participant:** I can not conclude that there is no early marriage because there are interests. In preventing early marriage there is multi-sectoral collaboration. For example, if there is early marriage, to stop the process schools report to women affairs and women affairs take the issue to prosecutor and police. As a woreda, there are good things on this regard and as an education office they are working to minimize school dropout. School teachers can conclude that it is early marriage if a lady is married before grade 10 because if she starts education at her 7 years old she will be 17 years old at grade 10. Though there are changes, but still there are interests marry their adolescent girls to government employee to leave surrounded by their children and maintain their dignity. Instead of aspiring to see their adolescent girls to be lecturer, scientist and doctor, they aspire to send them to school until they complete 10^th^ grade. So, to prevent early marriage there should be multi-sectoral collaboration among education, women affairs, health and justice office. There are adolescents who were referred to hospitals for checkup and there were circumstances where we were defeated because the adolescents were reached puberty. If she is not well nourished she might not be physically matured and have no breast development. Now we promote to check their HIV status and to be 18 years old. Here the multi-sectoral collaboration, the health professional, prosecutor and police should tell the right age of the adolescent. The adolescent herself and the education office should also involve. Furthermore, priests should be involved because they know when does she baptized and know the exact age of the adolescents.

**Interviewer: what are the health and nutritional impacts of early marriage for the women and baby?**

**Participant:** being a teacher exposes you to see many things. If there is early marriage, she doesn’t have physical maturity to carry the fetus. If she experiences sexual intercourse before age of 18 years she will face big health problem. Though physically mature she is not psychologically mature if she marries before 18 years old. She should have psychological, physical and economic maturity that can shoulder household responsibility. if she doesn’t have psychological, physical and economic maturity it will lead her to death, unnecessary bleeding, fistula and birth complications. Take for example a plastic bag, if it carries beyond its capacity it will tear. Similarly, if she gets pregnant before her uterus wall are strengthened, it will lead her to different health impacts on herself and her baby. Secondly, she will be also economically affected. When you marry you should be economically independent because you your families can’t feed you forever. Therefore, when you get married you need to be economically secured to lead your life. If you are economically secured your marriage will be durable and there will not be separation and there will be good feeding practice that will lead to health child.

**Interviewer: How many years do you think the gap should be between successive births for women? Why? What about if shorter than it? What other benefits does it have for the women and the baby? What do you suggest promoting it in a better way?**

**Participant:** we should take Strength, Weakness, Opportunity and Threats (SWOT) analysis. If there is short interval between births, it has no benefit instead of impairment. The impairment will affect three bodies, on the child, mother and family. Number one, the child will be affected because the child will not get food because at least children must have breastfed for two years scientifically. Because breast milk is balanced food by itself more than egg, milk and others. If the child did not get balanced food s/he will be stunted and if s/he is stunted s/he will die, disabled, or s/he will have poor mental development. The mother also faces equivalent problems. If the birth interval is very short, the mother will have fetus with in and she will be forced to stop breastfeeding the younger child. Because it is impossible to climb to two trees though you have two legs. I am not health professional and I don’t know what will happen to a child breastfed from pregnant women but traditionally it is not recommended. But mainly, since she has delivery experience her uterus wall is not recovered. Therefore, being married early and having short birth intervals has its own impact because after delivery the uterus needs rest. Even farmland needs rest and care, and this should be changed. in addition, it will have economic impact on the family. Because the family will be economically disturbed because the mother has great role and if she is pregnant she can’t work as usual. Thus, the household will be economically affected and if mother is debilitated every household member will be disturbed. The feeding and studying potential of the children will be affected. If there is short birth interval the mother can’t join social events like wedding, baptism and funeral ceremonies. In addition, children will not optimally feed, and they will be affected as a family because if the child is ill you will have medical cost and there will be lose of working time.

**Additional Remarks**

**Interviewer: Do you have any other comments on anything that we have discussed? What lessons have you learnt regarding adolescent and maternal (pregnant, lactating and adolescent girls) nutrition at your level? What lessons have you learnt regarding multi-sectoral coordination of nutrition in this Woreda? What opportunities do exist to promote maternal (pregnant, lactating and adolescent girls) nutrition in this Woreda?**

**Participant:** your questionnaire is very comprehensive that assess everything. But the way forward is to strengthen what we have started because nobody can do our job. Creating awareness is very important, especially discussing with respective stakeholders that we have listed before.

**Thank you**

Thank you for taking the time to discuss these issues with me today. We have learned a lot from you. As I mentioned at the start of the interview, we will remove all identifying information from the transcript of this conversation. We will make sure that no one can identify you from your comments. If you have any concerns or questions, please feel free to contact me (contact info). Thank you very much for your time.
