# Supplementary material for: Barriers to utilize nutrition interventions among lactating women in rural communities of Tigray, northern Ethiopia: An exploratory study
Source: PLoS One. 2021 Apr 30;16(4):e0250696. doi: 10.1371/journal.pone.0250696 (PMC8087028; doi:10.1371/journal.pone.0250696)
Supplement: S2 File — (ZIP) [file pone.0250696.s002.zip › S2_File.Doc/Woreda level and above key informants/118_IDI_Youth office_Lalay Machew woreda.docx]

**Day9: 11 /03/2010 E.C**

**Translation: In-depth interview of youth affair**

**Section A: Interview details**

Zone: Central

Woreda: Laelay Maychew

Name of participant: W/gebriel Tewelde

Interviewer: G/medhin.B

Date: 11/03/2010 E.C.

Interview start time: 3:26 Am; local time

Interview end time: 4:58 Am; local time

|  | Socio demographic information | | | |
| --- | --- | --- | --- | --- |
| **Sex** | **Age** | **Marital status** | **Education level** | **occupation** |
| Male | 31 | Married | Bachelor degree | Government employee |

Position: Head

How long have you been in the current job/position: 4 years

**Section1: common maternal nutrition**

I: In your opinion what are the common nutritional problems for adolescent (10-19 years old) girls in the community? Any problems you observe, in both in and out school ones, when go around different areas and structures;

P: When we go to different kebeles and schools, we observe that because females are not eating balanced and variety of foods, we see its impact on their body. These are: their weight is small though it I better than girls from other area. Even though it is not among many students, few of them do not use diversity of foods. Students bring one type of food from their family when they come from rural to town. There is such a kind trend. The good thing in this community is a lot of vegetables grow here. You can easily buy and use it. There for, this area (Hatsebo and Madego) is better than other areas. Rarely, there could be nutritional problem among women. Most of adolescents today are good at nutrition. If it is not because of unavailability, there is no problem in awareness. There could be some problems associated with shortage of food. But, majority is fine. And education is given timely.

I: what about in terms of type? Example stunting, disproportion of height to age and weight to age, and others

P: Yes, like what I said before. Associated with imbalanced diet they use, there are very short and small girls. There are also under weight ones. Last week, I had an observation. There was a screening program among students for military purpose. Unfortunately 34 adolescent girls were less than 45 kg. Their age is eligible but their weight was very low; it was a dead one.

I: As you know this area is one of the food secure Woredas, so what do you think the cause? Where is the problem?

P: That is the point. There is a problem in feeding to some of them. And there is shortage in few Kebles. The kebeles differ one from the other. There are three kebeles in Kola (tropical), and the girls came from one of these kebele, so called ‘Welel’ which is near to Merebleke. I do not know whether it is because of shortage of food or due to improper use of food, but almost all became underweight. We brought by looking their height, but their weight was low though their height well.

I: what about problems related with micronutrient deficiencies (such as anemia, night blindness, and goiter) in adolescents?

P: Yes, there is. Fortunately I saw everything there during the screening. Out of 35 girls, four of them had goiter. Even though it is not seen among many of the adolescents, few have such problems.

I: what do you think is the reason for goiter, night blindness, and anemia?

P: The night blindness and anemia are related with problem in nutrition. But the goiter is because of ...emm

I: is it related with iodine?

P: Yes goiter is caused by shortage of iodine. That is what I think. But, I do not think these are hereditary ones.

I: what about the diet related non- communicable diseases like hypertension, diabetes, and others among adolescent girls? May be related to overweight;

P: I have never seen adolescents, both in male and female, with hypertension till now. I do not see any case of diabetes among females. But I saw diabetes among male adolescents, and they use medication.

I: Is there a situation where the community suffers from food insecurity? In your Woreda, are there kebeles with food insecurity?

P: Yes we have.

I: where?

P: Related with the climate change, in one kebele call ‘Ableo’, there are some Kushets with food insecurity. In that area, there was drought, and there was rain. Late alone for human, even there was difficulty to get water for their livestock. There was no rain the whole summer in this year.

I: Is it frequently? Does it happen many times?

P: No, it was only in this summer.

I: So do you think this has affected the maternal and adolescent nutrition in the community?

P: Yes, it will create a problem from now onwards. So far, they are using food that they had produced last year, but from now onwards, they will face a shortage, even they openly announce the government. Especially among women and children, there could be a problem. Personally that is what I think.

I: So do you have any plan to support the adolescent girls in repose to the situation?

P: Our office does not give any food support. What we do is we organize them in associations and we provide them a loan based on their project proposal and they can deploy themselves on different activities. They can participate either on animal breeding, trade or mining. We, together with other responsible bodies, arrange a loan for them. With help, they can lead their life. Providing temporary food support is a duty of other sectors; they saw it at Woreda level,

I: When there is shortage of rain, male adolescents may move and find a job. But adolescent girls do not. So, how do you think is women/ adolescent girls are at risk of malnutrition?

P: Because they are at home, they cannot move. Especially those girls who are at school mat face shortage of food, and they may not get balanced diet. If the food is not available in the community, there is no way that they can choice and eat of variety of foods. Thus, they may affected be malnutrition in this way.

I: What else?

P: In this area, by the way, the seeds what they want is Teff and sorghum. These are what they want. There are no any vegetables and they may not get animal products too. Now, there is no anything. They may not get milk and egg, but even when get it, they do not consume it, they rather take to the market. This will lead them to malnutrition. Because of these, women may be affected by nutrition related problems. “What kind of support is given by the Woreda?” this is what we will see it in the future together.

**Section 2: Nutrition priorities in the Woreda**

I: To solve the above problems so that women and adolescent would be self-support and care for them themselves, what priorities as plan do your institution has in relation to adolescent’s health? It can in collaboration with other sectors too;

P: For adolescents, we coordinate activities with health sector. What we talked about is family planning. Second, there were also short trainings on nutrition; we were also involved in it. Therefore once we arrange the program, we mobilize the community. There is youth’s conference. And the health sector works with us.

To deliver the education, we mobilize the adolescents. This year we will have conference starting from zone up to kebele. We announce the program, because we want the health sector to be present there, thus, we came to common understanding with them. It is not only meeting; youths donate blood at that time.

Thus we consider this activity and the health sector also works based on its action plan. And we discuss each other frequently. This year, this is our program: we will create awareness about nutrition extensively among adolescents on the conference. If females are changed, they can change the whole community as they are care a family. They are coming as insurance to children and the other community. We also help each other with health insurance. We usually collect money from youths for health insurance even though it is a health program. But, at Wereda, we came to understanding that every sector should collect money itself from its structure.one youth, be it male or female, pays 55 birr and we collect it. Everything regarding use is mobilized by our office; they only take the collected money.

I: When we call it adolescents or youths, it commonly considers the single ones. What about to the married adolescents who may be pregnant or lactating? Does your office include these categories of women?

P: Prevention of early marriage is an agenda of women office and our office. Their age matters, if their age is in the range of youth, they will be included with us. If there is any support they will be led by the principle. To under nutrition, adolescents are more vulnerable than the older mothers. There are conditions when adolescents are not ready and underage, but become pregnant suddenly. This may lead them to anemia and other problems. This is caused by under nutrition, there many situations where we mobilize. So the problem is severing among females. This is what we do with the health sector. The older mothers already have an experience. To the issues you mention, we are working extensively.

I: What your institution can do independently or with other sectors, to improve adolescent nutrition? It can be in mobilization or promotion or else;

P: Some times, there is food that comes for children. It is also given to mothers and adolescents who are low in weight. It would be good if our office included during the screening. Second, what I think it is good, is regarding health streaming committee; it will be good if our office included in streaming committee. It could be easy to solve youth’s problem. What are in the committee are health, women affaire and agriculture. For youths, both female and male, information can be provided us. Therefore had we been member of the stream committee, we can perform better.

I: what else? Can you tell me what your institution have done regarding adolescent nutrition is effective? Any nutrition related activities that your office had worked it with health or agriculture sector or others, and finally was effective?

P: There is no any work that we have done it so far.

I: What about activities that your office had coordinated or mobilized for other sectors regarding adolescent nutrition?

P: If we talk openly, the people in this area have better feeding habit as compared to other areas. If we are to take the solid truth, there is better nutrition in the area, and they also give education; we saw them when we move together. The under nutrition is not because of short food, it is because of lack of awareness. For this, education is given every time. Because goods activities are done, there is good change in nutrition. Now, the feeding among children, adults and even adolescents is good. Previously an individual wants to collect and save money, but now there is a desire to eat various types of foods. The situation is changing. Therefore in this area, in majority, there are good things in nutrition. Especially the health sector extensively works in nutrition.

I: Do you have any thing you know in detail? Activities done by health or agriculture sector adolescents’ nutrition that you can mention to me as one, two, three;

P: Now, there is ‘Balta’, they did it currently.

I: Who coordinate? Who works it?

P: it is done by agriculture. We identify youth from them. We and women affair identify the adolescents (female) for them. Then, adolescents prepare balanced food (Mitin) for children and supply to the community. We have two sites, in Madigo and Dura. We believe they are doing good job. We conclude it has contribution to balanced diet.

**Section 3: Nutrition interventions that improve adolescent and maternal health**

I: Do you think adolescents are getting advice to visit health facility if are pregnant or lactating?

P: Yes, there is advice, because there are professional in every kebele in health post and health center. Anyways, we give support at kebele level. As we work together, there is advice.

I: Do adolescents get advice to eat extra meal during pregnancy and after delivery? And, does this advice get practiced?

P: This is right. Such things are talken in all kebeles we move, because there are professional in all kebeles. Each individual pregnant adolescent or the older mother has a follow up in the health post. The HEWs follow them. Before three days there was an incident in Hatsebo. We were giving land for residence to youths, and there was one pregnant youth waiting for getting the residential land. In that time there was HEW educating the youths regarding health insurance. The HEW said “when I saw you (to the pregnant woman), you look that you are anemic.” Therefore, tomorrow you need to come to health post; you will be examined and take the medicine. This much advice is given, and this is what I saw it practically.

I: How about the advice to eat extra meal during pregnancy? Ideally, they have to eat at least one extra meal from what their family eats, so how is this in practice?

P: In the rural, there is a long stood culture that affects the eating habit. But, as a health extension package, there is daily follow up. All pregnant women found in the kebele have follow up. It can be in health center or health post. This is what we consider it as a change. There was a situation when husband is advised. Therefore, they advise “the pregnant woman is found in this status because of shortage of food or others. So, you need to care her very well in this way.” Every time, there is a change in the health sector. This is what we observe. The community is accepting it. For pregnant woman all services are given without any cost. Therefore it is easy to teach too and the community can accept you with interest. There is change from time to time.

I: How do you see the use of food diversification during pregnancy among women/adolescents? How do you see in practice? As some times women may eat the usual food staff although they have all type of food items. Do they get even counseling by the HEWs or agriculture or you network?

P: There is repeated advice.

I: Who provide it?

P: There is health professional. Besides, the management also took it as part of his job. Even though I am not professional, during field visit, I have different activities given to me, out of my sector. And during meeting, I will deliver/report all things done to the responsible body. Apart from us, there are professional from agriculture, health and education offices. Therefore, we have to deliver all the activities to the community. Out of the meeting, there is situation where we go home to home to educate the community.

I: Ho w o you see the necessity of the consuming diversified food?

P: The health extension package is needed for this. Before being diseased, prevent it. Prevention is necessary and it must be taken by the community. If you use balanced diet in different way, it will protect you from disease, and you will not be easily affected. This is its importance. “Before being diseased, prevent it” and this is already taken. The community has also awareness on this.

I: How do you the utilization of iodized salt? So far our parents were using Gamfur, and there are many people with goiter. How do you see this? Even, is iodized salt accessible in shops, retailers and in other places?

P: Now, in some of them there is lack of awareness and practice. And as habit, there are people who use the ordinary one. But, as the iodized salt is packed and suitable to handle, they buy it. Second, in order to use iodized salt, education is given to the community. The iodized salt is available in unions/associations. There are shops where the unions put different materials for sale. One, every member of the union should buy salt from the shop. It is the union who supply the iodized salt. The community buys the salt from the shop as it is nearest to them. Second, the union supplies only the iodized salt. There is no ordinary salt, and even there is no anyone who provides it and there is no anyone who carries it. The community took the packed one as it is. In addition, the people have understood the situation and there is also health education. The education is given not only to female but also to other community. This is what is in ground.

I: What about home gardening by adolescent? Planting and using different vegetables; for adolescents it could be also a source of income if they sell some of it. It is important for health and as nutrition. So, how do you see this?

P: Here in community, not only for food but what is good here in the community regarding youth, be it female and male is irrigation. E.g. in Hatsebo, Lesalso, Madigo and Mudura separately, these are irrigation sites. The adolescents do not have land, but the use the land in rent. They pay some amount money to the owner and they plant non-permanent vegetables like green vegetables, potato, onion and carrot. When they plant these vegetables, one, they will get income; second, they also consume from it. In areas where there is no irrigation, they plant in summer. It can be in their parent’s or in their own land. Therefore, it is not only a source for profit but it is also for consumption. In the market, there are fruits and it is good for youth and they can consume in that way.

I: How about the safety net? Even though it is good at Woreda level, in areas where there is food insecurity, are there adolescents who are involved in safety net program? Or the Woreda has no problem regarding this;

P: Yes there are. But they are few. The safety net quota given to the Woreda is few. Much of the safety net program is use by three kebeles: Niwel, Edaga arbi and Ableo; the fourth is Natka ablaeli; many safety net beneficiaries are from these kebeles.

I: How do adolescents benefit from the safety net? Adolescents are those who are 10 to 19 years old girls. Which age group can involve here?

P: If they can work for themselves, they are involved. Girls older than 17 years are involved and there are registered by their name. Youth participation in safety net including the local community has its own program, and youths are involved; they have the chance. In many of the kebeles, these who not have land are identified. One, they are getting two things. This is additional work, and from the safety net, he getting income. Third, the environment is reviving. So it is good for the youth. Thus, we can conclude that safety net has three advantages. Therefore when we see it at Woreda level, we have many youths who are involved in safety net even though we do not have the exact figure at hand. Food security office identifies the kebele need for safety net program. If a farmer has fertile land and he is also productive, he will not be involved in safety net program. Here in our Woreda, Laelay mayche, to tell you frank, many of the farmers are part of the safety net program. Because there is a work that needs energy, many of the people want to pass his time in his job and often they do not look for safety net. The youth, especially females are involved in it because they do not move to other place to find job.

I: if adolescent girl get pregnant or she is lactating, how does the safety net program entertains them?

P: There is a program for them until 12 months.

I: How is it? Do they work or are they waived like the elder people?

P: it is similar; they freely get the support.

I: What about regarding water, sanitation and hygiene practices among adolescents? Do they get advice on this regard?

P: Regarding sanitation, it is not such a problem in this area. Though the Woreda is classified as rural, it actually is semi urban; it is near to Axum. One, there is education. Second, in relation to ‘Atet’, extensive education is given to the community. Therefore the sanitation is good.

I: Is it at school or out school?

P: In all places. At school, the information is delivered regularly by professionals. Second, at kebele and home to home, there is health education. Therefore a lot of education is given to prevent problems associated with poor sanitation.

I: Are there adolescents who have trachoma because of poor personal hygiene?

P: Last year, we have had one youth with trachoma in Welel, near to Rama. He had traveled to Rama and affected by the disease there. Else, the Woreda is fine. For example ‘Atet’, it had occurred in Woreleke and Tanqua. But here we were fine.

I: What about the other waterborne diseases? It is not only ‘Atet’ which can be caused by poor sanitation.

P: It is fine. There are no more people affected by this. And there is also health education.

I: What about regarding personal hygiene? Do adolescents know when to wash, keep environment including toilet ownership? In towns, the youth could administer the toilet and provide service to the community. How are these things in your Woreda?

P: In rural areas, education is given to youths to wash their hand and face when they wake up in the morning. It is told that “after toilet they have to wash their hands.” The time taking one is awareness creation in toilet utilization. In this area, toilet construction was affected. Now permanent and temporary toilets are under construction. There are people who show you a toilet which they have never used it. Now, what is going on here is, one the area, Laelay Maychew, is flat. The situation itself affects you. If you want to use open field, there is no place that you can use in it. Even though there is farm field, it is free (there are no bushes). There is health education for people to use toilet. Now there are farmers who built standard latrine in the rural area. The same is among youths. In one kushet called ‘Debo’ youth are organized to produce and sell slap to the community.

Previously they were producing, and now we have again reorganized them to produce slap and provide to farmers. They get an income and in parallel, they provide service to the community. We did this in collaboration with the health office.

I: Is malaria common here in Laelay Maychew? How about adolescents, are they affected? What about the use of ITN?

P: Maria is not that much common here. But our youth are movable, usually to Humera. After they get back from Humera, we make a lot of efforts, together with the health sector, to let them screen for malaria. Here what we commonly do is to mobilize adolescents who have been in Humera for screening rather than spraying the houses. Because the youth is near to us, we give him an education to directly go to health center immediately after he gets back from Humera. This is because there could be probability of disease transmission to others. And for him, it helps from being victim unknowingly.

Regarding ITN utilization, it is good. Even though the mosquito is not malaria causing one, people use ITN to prevent from mosquito bite, especially in this season. What is interesting here that the habit of ITN utilization is very good. There is good supply and ITN is also provided on time. The problem here is anti-malaria spray is not available. There are some kebeles who complain of it. They said “all of us do not get the service.” In one kebele called Miab, we held a meeting with youths. When we identify problems, they have mentioned us “there is shortage of anti-malaria spray. It is sprayed in some of the houses and some houses are left unsprayed. Thus, the problem should be solved.

I:

I: Do anti parasite drugs (deworming) give to adolescents? Like what is give at school to children?

P: Here, I have not seen anything. I did not know. There is a drug given for trachoma.

I: After MUAC measurement, lactating or pregnant women get supplementary feeding, like fafa (corn) and oil if they are thin. Is there a similar thing done for adolescents?

P: The adolescent must be either a pregnant or lactating. Out of this, no adolescent get such support even if she is thin and with low weight. I have information on this in all kebeles. After measurement, pregnant or lactating woman will be given additional food if she low. But such supports is not given to adolescents

I: What about Vitamin A supplementation? A small green tablet to prevent night blindness;

P: That one is given.

I: It is may be commonly given to in school as it suitable a mass of students. What about the out school adolescents? How do they get it? Is there home to home provision of Vitamin A or any campaign?

P: That one, they (out school) get it. It is carried out inform of campaign and it is not frequently given. During the campaign, mobilization is done. It is given at different five or six sites. It reaches every household. Because, it is planed per household, there is no problem. It is equal to everyone.

I: Is there school feeding program for in school girls? Especially at the food insecure Woreda; to prevent withdrawal from school;

P: There is no. Last year, there was feeding program in Edaga arbi. They had started it.

I: Is that in other Woreda?

P: No, it is in our Woreda. I did not remember the partner, is it Save the children or else, they had started school feeding. This was because the community had faced a problem as their cereals were destroyed by heavy rain. The students were supported to prevent school withdrawal. Out of this, there is nothing.

I: At health facility, adolescent should seek service. But they might get frustrated or shay. So, is there youth friendly service in separate room? E.g. If adolescents want HIV screening, take condom and others like STD examination; thus, we want them to feel free. So, is there youth friendly service?

P: Now, there is no. until now I haven’t seen like this.

I: This mean they are getting service together with adults. Do agree?

P: Yes. Education is given to tell they can take condom, utilize contraceptive. To prevent unwanted pregnancy, more lessons are given to adolescent girls about contraceptive freely, without frustration. Females are not frustrated. They go to the health post. Sometime when they feel frustration associated with being served in their locality, they go to town to utilize. With being free, it is good among adolescents. But, there is separate youth friendly service. Major of male adolescents do not go to health faculty to get lesson on condom utilization as many of the health professional at health post are females.

Here there are many youth clients, because the comfort itself governs you. Here, there is condom which is brought by health sector for our office. In the outside, we put it for adolescents, there youth many who took it. Here, they feel freedom, and they are not shay with us.

I: what if it had been in health facility? How do you see it?

P: In health facility, there is condom.

I: It is not only for condom, but for other services too? E.g if a young man to wants to know his HIV status;

P: Had there been a health professional for this purpose, it will be good. But, in all health facilities, there is no such thing and I did not see anything.

I: Which of the interventions listed above are important to adolescent girls? Meaning, you feel that it can bring change on adolescents’ nutrition (like ITN utilization, taking extra meal, school feeding, vitamin A supplementation, supplementary feeding, deworming service and others).

P: The additional food brought by other body will not bring change. It will only let people develop expectation. The basic one is the awareness creation that we made to eat balanced diet. Thus, it is good if adolescents are given education. So far what is being done is for children. But now, it will be good if adolescents get specific nutritional education. It would be very effective. Providing an education to adolescent girls means it like educating the whole family as she can form family.

If the government works primarily on adolescents, s/he can be change agent. Second, if peer to peer to advice is in place at health center, it may contribute to other especially on communicable diseases and others such as unwanted pregnancy and anemia, and under nutrition among females. Females will be careful if special counseling service at separate and confidential place is available. It may have contribution for the citizens. Regarding the additional food that comes and vitamin- A, there is no problem because it being given. Even if it is left, it will not affect. I say if the government works with full attention in the first two things, it is very important. One, the youth has readiness to accept faster than the other category of community. He can also teach to others. So, it will be good if education is given here. It will be balanced.

I: Which of the above nutritional interventions are effective to adolescents? Which you think it is transformed from advice to practice.

P: When we choose from the above ones, advice on the feeding habit is better. From all, the advice on feeding habit has better performance. From time to time, there is education, and from time to time, there is improvement. The existing change itself brought you to the current situation. The feeding habit is better now. When you compare the advice given on health conditions and this, there is significance difference. People hide from others. It is not yet changed, people hide. Youths do not come freely to seek advice. Therefore when we look it, it is better in the feeding habit. ]

I: What about the less effective one?

P: From where?

I: From the above interventions;

P: It is that one, the peer to peer advice or service at health facility separately for adolescents (he is to mean the youth friendly service). It lagged behind as per my observation. Even the magnitude of HIV is increasing from time to time than decrement. Because these things are getting back, special attention is needed here.

Second, had these things are given to us, adolescents, we could have been effective.

**Section 4: community factors affecting access to maternal nutrition interventions**

I: what are the challenges to implement/deliver the nutritional interventions that we have discussed for adolescents, be it pregnant, lactating or none? It can be at individual level like awareness or community level like religion or it can be related with resource, like lack of resource and the like; what are barriers that have to be solved?

P: One, adolescents are not visiting heath facility freely, even when she has anemia. Unfortunately, there is also unwanted pregnancy at an underage. There is also underage marriage as barrier which is related with culture. They pursue her to marry; with that level, she becomes pregnant while she is not ready. She will face two things: anemia and other things. Then she suffers. This is it what should be corrected. Then, she can be affected by communicable diseases. The community also affects adolescents not go to health facility freely. If you go there, by the way, the health professional has also his own problem. The health care professional is not confidential. It should be in secret. But, if you go the rural area, it is not like that. Even it would be good if this thing researched. I would say it should be corrected. What is there is, if an individual is examined at health center or health post including HIV screening test done by health professionals, the health extension works assigned there in the health post do not keep any type of secret. The information gets exposed to the community and everyone knows about it. This time, community retreats to visit health facility. Now, the adolescents do not go to health facility; they undermine the profession because of the error s/he made by himself. They may hate the professional, and the community may not visit the health facility. They prefer to go to big town, Axum, for service than serving in local health facility. This is because the gap in keeping confidentiality and security.

I: What about in relation the health care provider skill and quality of service? In addition to the non-confidentiality, a big thing, you told me.

P: Second, the advice should be delivered in an attractive way. If there is skill and capacity, you can convince the community and bring the adolescents to health service. Therefore there is also limitation in capacity. All these created barriers to the community, and these should be corrected. In the future, if they are corrected, they will be difficult.

I: Anything else you can add as a challenge.

P: The other is there is limitation in supply in the market. Even though you understood it and wanted to buy something you may not get it. There is a problem in transportation, and lack of market in the area. Therefore, these are the challenges, even though the problem can basically by solved by building infrastructure.

I: For the challenges you have mentioned above, can you tell me any solution that your institution has applied for adolescents? Our office, independently or jointly with other sector, has solved the problem in this way, and it can be taken as experience for others.

P: Regarding balanced diet (nutrition), we did not work anything. But indirectly, it can be, because we have established a club at Woreda level for adolescent to get peer to peer advice freely. It has its own contribution

I: What is the club?

P: It is a youth club. The club move to Kebeles and it provides information to adolescents in form of poem and drama. We create forum for this. They inform youths that how much effects and damages it has while working with these things in shoulder. The youths are telling us openly that these services in the kebele, what I have mentioned you before, are not enabling us to come freely to health facility; these are barriers. The ability of health care providers to keep confidentiality is weak. Because the meet with the farmer there in the community. Even there is an education given by HEW to get service in towns. Therefore there is a change from time to time. With regard to prevention of unwanted pregnancy, how to use contraceptive and condom they tell them to come openly and freely to get the service. If possible they have to abstain, if not we educate them how to use it. There is a club and it is working better things among adolescents.

I: How does the club involves adolescent girls?

P: There are 16 youths in the club, of which only six are male, more are females. Because it is delivered by females, it is easy to accept it.

**Section 5: Other interventions that influence adolescent and maternal nutrition and health outcomes**

I: In your opinion, why would delayed marriage (after 18 years) improve maternal nutrition including adolescents? How do see the advantage and disadvantage underage marriage for mothers?

P: Now, what I was explaining before is if a girl marries in underage, she may get pregnant when her body is not developed and ready to feed a baby. Second, because she encounters under nutrition while she bleed during delivery as her body was not ready for pregnancy. Anyways it has damage. For this reason, we and women affair are giving education. The male is also a productive citizen. So, when he is caught, he should not pass his time in prison. He is hurting her, and at the same time, this guy is hurting himself. There are two damages. When he is found, he will be asked by law. If he is not found, the girl faces difficulty and he will also be hurt. Both are hurt. That is why we are giving education jointly on underage marriage. When there is underage marriage, when they are nominated for that purpose, she should say “no.” We give them such information, and there are many who don’t accept the marriage. They also come openly to women affair to talk about it. They say “we are facing a marriage, and our parents are forcing us to marry, so what should we do” they asked us. There is a situation when we, together with police, terminate a marriage. As a Woreda, there are lots things done to prevent underage marriage.

I: What about related to increasing space between each birth among adolescents? Do adolescents use contraceptive? How do you see this?

P: I have been working at Kebele as an expert for many years. What is there for some of them, especially the wife of a priest will not take contraceptive. And, she was considering it as religious activity and they gave birth double. Now, this had changed. Now with the situation, be it priest’s wife or other, they are using. Even some are deciding not to give birth. Because of the change in the community, they plan to have three or four children. Previously, there was proverb called “he will grow by his chance” now there is no such things. In the community, there is an interest of family planning. To use contraceptive, some women have discussed with their husband, and some has decided without notifying their husband. If they want they can take injectable or pill. Because, short birth interval has problem, people have awareness even though it is not as such much as we want.

I: What programs or activities done by your office or the health sector or others to promote increasing birth intervals and to prevent early marriage among adolescents?

P: Now, there is a limited change. But, the family planning activity has a decreasing pattern than it has been before. The community has understood the use of contraceptives. But, the health education on family planning given by health sector tends to weaken. When I look this in the entire Woreda, and Tigray, it has weakened. The promotion of birth spacing either by radio or by professionals at home to home is very weak. I would say there has to be one principle. But in the community itself, because the situation is governing him and understood it, the tendency of family planning is good. For us, now, we took the education on family planning is part of our work among youths. In every opportunity, we mobilize. But, in measuring how much change we brought has huge gap. Therefore, it is good if we work more.

I: Can you tell me any programs in place to prevent early marriage? What about in terms of law?

P: Underage marriage has its own regulation and declaration. To those who let the children married, there is its own regulation.

I: what does the regulation say?

P: If the youths are married in underage, both the parents of the bride and the groom will asked by law. But, in the community, even though the awareness is increasing some times what is happening is covering. That means, when the girl is in underage, they provide witness as if she is above age (greater than 18 years). While it is not actually their concern, they speak her age and count back when she was born in calendar year. This, itself, supports the existing backwardness, and problems in the community. But, underage marriage has its own regulation.

I: What about in terms of political commitment, religious and other influences? What if she is selected by a deacon, she early marries.

P: Here now, there is no religious influence. “Because he is deacon and he should marry a child” this does not work, and there is no such influence. And at school their age is registered correctly, there is no religious influence in this locality.

I: What about others?

P: what we can take is the political commitment is low. Even when underage marriage occurs, the fast response in terms of legal action we took is limited. When we compare the religious influence, this one is bigger.

I: What are the community factors that affect age at first marriage? What is the community’s perception to early marriage? Sometime, a mother may say “I was married and gave born to you at age of 14 t but, nothing has happen to me” there could be such sayings; so how do you explain this?

P: Leave it; it is there in some females. But, no one accepts them. This time, the adolescent girls do not accept such idea. Now days, there is no any work done without interest. Because girls have awareness, they came early to women affair when they face underage marriage.

I: What other opportunity do we have to prevent early marriage and increase birth spacing? For example, building awareness at schools;

P: Now, the opportunity that we can use for family planning is: there are different forums like religious leaders meeting including the other community, especially priest’s wife that tends not to use contraceptive. There are pressures, so if we give education here, they can understand themselves. And if you teach them in relation to the current situation, like the presence of market inflation, scarcity of land from time to time, they can understand it. If we work here on family planning, I think, it will be good. Plus, if we work among males, it would be good, because many of the influences are from male. They want her to give birth. Even when he has interest which his wife should not give birth, there is lack of awareness on utilization. Even the females do create problem during utilization. They stay using contraceptive, but they do not know when their appointment is. Second, if they want to take injectable, there is pressure the community like you will be infertile. If these things are not changed using extensive health education, it will influence the family planning. Thus, we have to work here.

To prevent underage marriage, one education has to be given in the schools. Second, we have to educate religious leaders, parents, Abeytiadi (community leaders) and youth and adolescents. We have to work here by creating forum and preparing manual together with professionals and that fits to him. It is necessary to work here.

**Section6: Multi-sectorial collaboration to improve maternal nutrition**

I: Do you feel it is necessary, at your level, to work with other sectors/institutions to address maternal nutrition? How is your experience in multi-sectorial collaboration?

P: Now, we as sector are working with 13 sectors. But, in regard to mothers, we work with health, agriculture, and women affair and education sector.

I: is this a potential or it is done practically from higher to lower structure?

P: It is practical. Now, it has its own committee. We plan together and we work together in an integrated way; especially with women affair, agriculture and health, we work in special way. With agriculture, it related with the safety net program. We are involved there. One, we evaluate that if it involves the youth. We have a forum to evaluate it, and there is a responsibility we took and sign for it. Plus, with health, we are involved at work related to HIV, balance diet, family planning and latrine construction and environmental hygiene. There is a condition where we made an interface and work together. With women affair, we work in area of underage marriage, adolescent girls’ beneficence. We work together in these things. Regarding to mothers, these are the bodies that we work together.

I: What other sector do you think is necessary to collaborate with your institution? Any partner that you feel that can improve women’s nutrition;

P: Other, to improve adolescents’ nutrition, there is no other sector that I can say we can collaborate with him. But if we take generally for women’s, we can collaborate with justice and security (police). It is related with them. All of them have contribution, and they are already included as there is another stream committee for women. But with us, as beneficiary, we work with what I have mentioned before including water resource office. We are connected with water resource, so that female youths should benefit from mining activities. We create an opportunity, and they can get some sort of income. From the 13 sectors that exist, with water resource is one. We also work with all the sectors in one way another. For example with small scale industry, we made the sector arrange small and easy manufacturing activities for female youths, so that they can generate income.

We also work with a new sector called small and medium scale manufacturing office.

I: Therefore, when you are working together, what should be done or change is needed to improve maternal and adolescent nutrition? For example, earlier you have mentioned me that it is better if your office is included in stream committee. What else can you tell me?

P: What I say stream committee previously is to stream committee of women. Here, the members are health, agriculture, and education. We are not included here till the kebele. Our network is not available at Woreda as well as kebele. If we are to work among women, if we have to meet, we have to be part of the stream committee. This is one and I would say we could have worked better job. Second, at women affair, had special training is organized to female youths, it would have been better. It should be in especially way and for only females.

I: How do you see the initiative you took to lead multi sectorial collaboration having in mind that it will improve maternal and adolescent nutrition? Or you are seconded to the primary role of others.

P: As a sector, what we lead is the utilization of the different interventions; for example if we look at balance diet, we made women to have its own income. The education on how to prepare and consume balance diet is a duty other party, the health sector. But, the activities we made women to generate income is led by us. We do not give it other, even the women get it the service from our sector. We empower them to generate income. If they have income, the probability that they can eat balanced diet is increasing. Second, what our sector works is on the peer to peer advice we arrange for adolescents. We take responsibility to work on screening with waiting to the health sector. Generally, these are the activities what we do.

I: Once multi sectorial collaboration is in place, what kind of changes are there among women and adolescents’ health and nutrition? Or is it simply an ideal which is not implemented in ground?

P: Now, what I can tell you is: there is a so call development arm for women. In the development army, we organize the youths and the mothers together. Now, their interest will not come be uniformly. Now, there is farmers’ army and youths’ army organized as new network. The youths’ network has an interest to isolate and stand alone, and they ask repeatedly. Finally, it is rearranged for them. In the guideline, it says, if there are female adolescents, they can be organized with the male youth development army. If you see them in practice, they have the interest to join with male development army, but, the women: women’s affair and women’s league and women association in the Woreda do not want and influence them. They say “you do not have to be in male youth development army and you should not leave women development army.” There are many challenges here. And there are complains that they mention as “women are under pressure.” You are taking us youths. The health sector also claims the same. The health sector also does not want them to join the youth.

I: They want them to manage together with mothers.

P: Yes. But, their interest especially related with communicable disease and others service related with the peer to peer advice is with the youth s; and thus, it is good if they are organized with us.

I: May be a last question, do you have any other comments that you want to add? Any lesson you want to deliver?

P: Here, what I want to say is if the mothers’ and adolescents’ nutrition is going to bring big change, only women should not take the responsibility. The husband should also take the responsibility. The men should be investigated and aligned with the maternal nutrition. There should be awareness creation with men and implement it in practice. If not, especially in Tigray, it would be worse for us than others region. That is what I feel. It should be researched; if it exists, it should be changed to practice, so that men should be helper to women. Even to manage for themselves and family, it is important to include men in the intervention. If you create awareness to only women, because it is dedicated for them, there will be influence. But, if you also create awareness to the man, he will help and because he also takes responsibility. Thus, my conclusion is we did not work among men. And to the youths, you need to create a forum and educate them by inviting individuals that fit to level of the youth’s thinking. If you provide education to male youths through a female HEW, they will not listen to her. They, the youth, may undermine and will not accept her. There should be someone who can shape it and give education. In that way they listen very well. If we provide information to the community, there will be a change from time to time.

I: Thank you very much for your time and energy!!

**Summary**

**Section1: common maternal nutrition**

Because of imbalanced diet they use, there are very short and small girls.

Even though goiter is not seen among many of the adolescents, few have such problems.

There is no hypertension among adolescents, both in male and female. Diabetes is seen among few male adolescents

In few kebeles, there is community who suffers from food insecurity

**Section 2: Nutrition priorities in the Woreda**

To deliver the education, youth office mobilizes the adolescents. When there is meeting; youths donate blood.

We (youth office) usually collect money from youths for health insurance even though it is a health program.

It can be easy to solve adolescents’ problem if youth office is include in health streaming committee.

**Section 3: Nutrition interventions that improve adolescent and maternal health**

For pregnant woman all services are given without any cost, therefore it is easy to teach.

If you use balanced diet in different way, it will protect you from disease, and you will not be easily affected. “Before being diseased, prevent it.”

The safety net quota given to the Woreda is few, thus, there are only few adolescents who are involved in safety net.

Sanitation is not such a problem in this Woreda. Almost all the community has toilet. There is health education for people to use toilet. Now there are farmers who built standard latrine in the rural area.

Commonly as program, adolescents who have been in Humera are screened for malaria, and the mobilization I done by youth office.

There is no deworming service given to adolescents.

**Section 4: Community factors affecting access to maternal nutrition interventions**

Adolescents are not visiting heath facility freely, because of the community’s culture and health professional violation of ethics. The health care professionals are not keeping secret and confidentiality.

At Woreda level there is a youth club established for adolescent to get peer to peer advice in form of poem and drama freely.

**Section 5: Other interventions that influence adolescent and maternal nutrition and health outcomes**

Adolescent girls come to women affair to talk about underage; they say “we are facing a marriage, and our parents are forcing us to marry, so what should we do” they asked, and then the marriage is terminated by police.

Previously, there was proverb called “the baby will grow by his chance” now there is no such things. In the community, there is an interest to us family planning.

The political commitment to prevent underage marriage is low.

**Section6: Multi-sectorial collaboration to improve maternal nutrition**

With regard to mothers, youth office primarily collaborates with health, agriculture, and women affair and education sector.

Youth office collaborates also with water resource, to benefit adolescent girls from mining activities, so that get some sort of income.

As a sector, youth office leads the utilization of the different interventions by adolescents.
