# Supplementary material for: Barriers to utilize nutrition interventions among lactating women in rural communities of Tigray, northern Ethiopia: An exploratory study
Source: PLoS One. 2021 Apr 30;16(4):e0250696. doi: 10.1371/journal.pone.0250696 (PMC8087028; doi:10.1371/journal.pone.0250696)
Supplement: S2 File — (ZIP) [file pone.0250696.s002.zip › S2_File.Doc/Woreda level and above key informants/120 _IDI_ woreda education office_Lalay Machew woreda.docx]

**Operational Research on Adolescent and Maternal Nutrition in Northern Ethiopia**

**An In-Depth Interview with Woreda Education**

**Introduction**

Good morning. Welcome and thank you for taking the time to speak with me. I am Hailemariam Tekie from Mekelle University. I came here today to study the factors that influence the nutrition of mothers and adolescents in collaboration with the Regional Health Bureau and UNICEF. As part of the community, no one knows more than you about the problems existing on adolescent girls within the community and also possible solutions for the problems. So, your participation is very valuable. The things that you tell me will be used to improve nutrition programs and services for women in the region and the country. Your name will not be included in the report. But, in order to capture all the ideas that you share me, I will record the interview. The interview will take about one and half hours. Do you have any question before we begin? If it is all right with you, I will turn on the tape recorder now.

**Section A: Interview details**

1. Zone: Central
2. Woreda: Laelay Maichew
3. Kebele: ___________
4. Name of key informant: Mebrhit Werkneh
5. Institution of key informant: _______
6. Interviewer name: Hailemariam Tekie
7. Date of interview: 20/11/2017
8. Interview start time: 9:44 am
9. Interview end time: 11:18 am

**Section B: Interviewee professional information**

1. Gender:
2. **Female**
3. Male
4. Age: **30 years**
5. Highest level of education:
6. No formal education
7. Primary education
8. High school
9. College education
10. **Bachelor degree**
11. Master’s degree
12. Current job/position: **Deputy of Woreda Education Office**
13. How long have you been in the current job/position?
14. **Six Months**
15. Years

**Detail of the In-depth Interview**

**Key:** I – Interviewer ; P – Respondent

**Section 1: Common maternal (pregnant women, lactating women and adolescent girls) nutrition problems in the community.**

**I: In your opinion, what are the common nutrition problems in the community for women? What about for adolescent girls?**

**P:** When we look at the nutrition of mothers and adolescents in our woreda, it is not a great problem. I can say it is ok as compared to the previous times because of some of the educations provided by the health extension workers through home-to-home visiting. This time it is ok. When we say it is ok, the main focus given is on the hygiene practices. As the local areas are not far from the urban areas, they have good handling practices of the hygienic conditions. They follow better nutritional feeding styles. But there are some problems, for example there are some who simply eat without washing hands. There is still a gap with regard to awareness creation on the hygienic practices and as a result there are incidences of different diseases though they are not affected, especially on the lowland areas where malaria is prevalent, people are exposed to malaria. So, this is due to the sanitation and hygienic conditions in the local areas. If you do not make your house clean and if you do not make the environment clean, there could be different insects that can be generated from the unhygienic areas and therefore, people will be exposed to malaria. But I do not think there are problems related to lack of food. The main thing is on the hygienic practices otherwise, I do think there is a problem related to nutrition.

**I: Ok. You are telling me that there is no problem in relation to nutrition but can you tell me is there are some incidences? For example it happens in children that they can be underweight or thin in other areas. What about the status of mothers and adolescents in this woreda?**

**P:** Actually, there are incidences, for example there are wasted children and also there are mothers that are malnourished. There is a difference among the mothers taking nutritious foods and those that do not. We observe during our field visit that there are incidences on mothers that they are thin and also you can understand the problem looking at their skin glossiness. We could not understand what is inside health condition of the mothers but we can see the problem from the physical appearances that they are thin and not glossy. Similarly, there are incidences on children that they are undersized and thin.

**I: What about micronutrient deficiencies?**

**P:** There are such problems, especially goiter. It is ok after the introduction and consumption of iodized salt in this generation but there are mothers that have goiter in the areas. The other thing is that mothers say I feel dizzy especially when I stand from sitting.

**I: Ok. You said there is reduction of goiter because of iodized salt and what could be the reason for dizziness and night blindness, if there is any.**

**P:** Actually, we do not ask in detail but when we ask them why you feel dizzy, they say the health expert told me it is anemia (ደም ዋሕዲ) the reason for this as they are told by the health experts is lack of consumptions of nutritious foods. Though they produce vegetable, they sell them in the market. So, they do not consume vegetables. They do not take nutritious foods, vitamins, and proteins, and others, and when you ask them why not you take these nutritious foods, they tell you that they do not take them. But we do not ask them in detail.

**I: What about diet related non-communicable diseases such as diabetics and high blood pressure?**

**P:** When we go to the field activities, we talk with the farmers. So, they say I have blood pressure. When we ask them their feeling, they tell us that they feel dizzy. And with regard to diabetics, there is a person I know who was a guard in the office I was working before. He is taking treatments but that will not avoid the problem if you do not follow-up the case. So, there are incidences that people say I have diabetics, I have high blood pressure, and others and there could be a variation from kebel to kebele.

**I: What could be the cause of high blood pressure? And what is the perception of the community for these diseases?**

**P:** Most of the community understands that these problems are happening in urban areas in relation to eating nutritious foods. Though they did not have the know-how, now they understand that it will also happen in rural areas when there is shortage of food or shortage of eating nutritious foods, especially those that are affected by the diseases have the know-how that the diabetics could also happen at rural areas.

**I: What about overweight in this community?**

**P:** I have not seen this kind of problem in the areas.

**I: What about the perception of the community to overweight?**

**P:** As we are talking in rural areas, the community thinks that there could not be overweight in rural areas because they are working in the tedious activities that demand more energy and if there is loss of energy, there will be balanced weight. So that they think overweight will not happen in rural areas.

**I: What about the food security condition of the woreda?**

**P:** It is ok. It is moderate. You cannot say it is good and you cannot say it is bad. The people have moderate living conditions. As to my observation, previously, it was not that much good, especially with regard to irrigation facilities that there was no much awareness but now in most of the community in this area there are at least irrigation activities. Though the people did not use them for their own consummation, they bring and sell it in the rural areas so that the urban areas are beneficiaries of their products. So it is ok. It is not that much bad and it is not also that much good and the productivity are also fair.

**I: You told me that malnutrition is not that much risky in the area but as there are some incidences can you tell me which women groups are most affected? PW, LW, or adolescent girls?**

**P:** Mostly it is the women that are affected.

**I: Women can be pregnant, lactating, and adolescents. So, which groups are most affected?**

**P:** With regard to nutrition, most of the time, it is the pregnant and lactating women that are highly affected. They are highly affected because they need more foods when they are breastfeeding, they need balanced diet. Similarly, during pregnancy, they need balanced diets, they need a lot of things but they do not properly utilize them. They do not think that they need them and do not prepare and consume what is important for them. That is why they take the foods that they have produced to the market. If you try to see in Laelay Maichew woreda, mostly it is happening like that. They do not think that this is important for their child rather they take the products to the urban market. There is a gap on the awareness to the utilization of balanced diets.

**I: What about in adolescents? Can we say they are not exposed to the problem?**

**P:** When you compare it with the pregnant and lactating women, adolescents are not that much affected.

**I: What about adolescents as compared to other groups of the community?**

**P:** They are not that much highly affected. It is similar to that of other groups of the community. Adolescent are getting what other groups are also getting. They do not have anything that they could be affected.

**I: Are there any challenges to maternal and adolescent nutrition?**

**P:** There could be a challenge within the community but I do not think that there is a challenge from other institutions. Mostly, there is education provided, especially in the rural areas for women and adolescents in relation to the balanced diets that are important for them by the health sectors, focusing on the health aspects. But as a habit, what they do is that they take the nutritious foods that are produced in the farms to the market rather than using it for their own consumption. This is due to lack of awareness. They do not think that the foods that I am taking to the market are also important for me. So, they do not keep some portion or the full portion of the products at home for consumption. This is due to lack of awareness on the community that they have to think this is important for me and i should not only produce for the market. So, it is because of the awareness of the community but I do not think there is a challenge or gap on the institutions.

**Section 2: Nutrition priorities in the woreda**

**I: In your opinion, what priorities do your institution has in relation to maternal and adolescent health? Why?**

**P:** Most of the time, it is on hygienic practice. If we see in Laelay Maichew, mostly the activities that are given much focus are on the hygienic practices, especially on the use of toilet, personal hygiene and sanitation. The first thing is therefore to create awareness on these issues by the respective institutions. The second focus is about nutrition. But the first focus given is about hygienic conditions.

**I: What your institution is doing currently related to the priorities you have mentioned?**

**P:** As education office of the woreda, we have activities that we implement; especially in schools where there is “health and anti-HIV AIDS club”. In this club, there are different activities that are organized in the form of drama, literary, discussions by students because if the students, especially girls understand the messages, they can easily change the families in the community. Awareness on how to keep hygienic condition, how to wash hands, and how to clean the environment are provided in the school. Through this, we are trying to mobilize the activities deep in to the communities.

**I: If that is the case, what nutrition interventions have the most resources allocated to them? Could you tell me in detail with examples?**

**P:** We do not have a budgeted resource for this prioritized intervention. There are NGOs like that of Save the children that have budget for those schools that are included in the project. The budgets are for soap, modes for the adolescent girls, and as education office, we promote to use local materials to use as hygienic practices. Otherwise, we do not have a budget for that.

**I: Do you think it is necessary for your institution to get involved in work aimed at improving nutrition among women and adolescents? Why? If possible in relation to your institution’s mission.**

**P:** Yes, of course. The messages that are conveyed, especially at school are very crucial. So, if they are crucial, we as education office, have to work hard because the students can change the whole community in a specific area in case they really learn and understand the message. So, it is due to lack of budget otherwise there is a need to have a budget as a start-up for training, workshops, and demonstrations. It is only the limited capacity of the woreda that it is not given emphasis otherwise it is very important.

**I: What about in relation to your mission?**

**P:** It goes in line with our mission. Most of the education provided to students on how to keep personal hygiene and sanitation is one of the missions of woreda education office because if we want to produce children of healthy citizen, first of all he has to be hygienic. Based on this mission, we try to accomplish the activities to achieve the mission. We establish clubs in schools, and create awareness on the students, and providing messages to achieve the missions.

**I: How do you evaluate the priority given for the interventions for the women?**

**P:** The prioritized activities under implementation are very important. Most of the diseases that are transmitted are because of lack of hygiene and sanitation conditions. As most of the transmitted diseases are caused by lack of hygienic conditions, we give the priority to the hygienic conditions and then we put the thing to be the second priorities. Otherwise, I do not accept, for example, nutrition to be the first priority because if you do not handle or utilize the food properly, it will be meaningless. This is due to the reason that if we are eating unsafe foods, we will be infected by different diseases. So, based on my personal point of view, I prefer hygiene and sanitation to be the priority and I believe I will do it in a better way for the future because there are many diseases that could be raised by lack of hygiene and sanitation.

**Section 3: Nutrition interventions that improve adolescent and maternal health**

**I: What kinds of nutrition interventions are in place to improve health of the pregnants to your level? Where do they get it? Who provide it?**

**P:** As we go to most of the kebeles for field activities, we observed that most of the mothers have group discussions either in a week or two weeks in each kebele. These all have different activities; what the lactating women should work? What the pregnant women should get a balanced diet? And then woreda health office is giving them educations. While education is provided, the women development groups and the mothers discuss together about the issues. So, they discuss and share on the ideas such as: how do you feed to your child? What kind of balanced diet do you provide to your child? And then after the discussion, they share ideas that need to be filled based on the identified gaps. Similarly, for the pregnant women, there are activities with the discussions on how they check health conditions, what kind of food they consume, what shortages they face, and others.

**I: What about counseling extra meal during pregnancy and lactation? Are there advises on ANC during pregnancy?**

**P:** As to my knowledge, they get support and follow-up or advice and counseling otherwise, there is no any financial support provided to them with in the worda or the health office. But there are support, follow-up, advices and counseling activities for the proper utilization of balanced diets that can be available based on the products that are produced at household level.

**I: Do pregnant women get screened for their nutritional status?**

**P:** As to my knowledge, there is no anything with regard to this. There are advices and counseling given to the mothers to go to the health center for child delivery, for check-up, follow pre and post-natal check-ups, and others otherwise there is no anything that give advice to the mothers that you have this problem and you need to take this food and there is no one that tells you have this one to take from us. There is nothing yet started in our woreda.

**I: What about food diversification for pregnant and lactating women?**

**P:** As I said earlier, they are told to take balanced diets. Whether they eat it or not, it needs an assessment. But the health extension workers give education to the mothers to take balance diets. They tell them not to take the food today that you ate yesterday and the like but you cannot be sure whether they are really taking the balance diet or not as far as you do not follow what they are doing.

**I: As you said it needs its own study but what do you understand from the community while you discus with them and if there are also barriers that you know?**

**P:** Ok. As the mothers explained, the health extension teaches us not to take foods today that we have taken yesterday for diversification but they say how can we use like this?

**I: What could be their reason?**

**P:** When we talk to some of the mothers and ask them why do not you eat food different from the food that you ate yesterday? They say as we have so many activities and agricultural activities to do, there is no one to prepare the foods. So, as we do not have time to do that, we simply eat what we had yesterday for today. We do not have time to diversify our foods because we go for agricultural activities.

**I: You told me earlier about iodized salt but I wanted to know about the current conditions. So, would you please tell me the status of using iodized salt in the community?**

**P:** We cannot say all the community has been changed. When you observe in the earlier times, mothers were having goiter but now it is decreasing not only because of iodine but also by taking other nutritious foods. But you cannot say all the people are using it; there is a gap, especially with regard to the rural conditions that there is negligence in that they use other type of salt (gela salt) though there are educations provided to them. So, it is difficult to say all are using iodized salt.

**I: If they are aware of it, what could be the reason for those that do not use iodized salt?**

**P:** As to me, it is negligence. It is a matter of not taking that it is important. Otherwise there is education provided and the educated individuals are trying to make the people well aware of but there is negligence. They say why should I use it? It is just after they get diseased that they sensed its importance. There is no prevention before they get sick because of negligence.

**I: What about home gardening? Do you think most of the people have access to water for home gardening?**

**P:** It is difficult to estimate how much of the people are having home garden activities but there is no problem of water in most of the areas in this woreda. But those that do not have access to water are also using mixed cropping systems for vegetables with cereal crops. But the vegetables that are produced even by those households having access to water are mainly targeted for market.

**I: What are the vegetables that are produced by the farmers? I wanted to know whether is only tomato or other diverse vegetables as well?**

**P:** It depends on the access to water. For example, tomato does not need water and matures early so that generally most of the areas produce tomato. In some of the areas, there is spinach, lettuce, cabbage, tomato, and onion. So, there is a difference from one location to another location.

**I: Do you think that there are farmers that do not produce vegetables even with access to water? If available, what could be the barriers?**

**P:** Yes, there are farmers that do not produce vegetables when you tell them to use the access of water. When you ask them why not you produce vegetables, they say you will only be tired of the activities from vegetable production. So, this is because of lack of awareness. They do not think that we can produce and be changed and they do not have the attitude that at least I can be self-sufficient. This is lack of awareness that they say why should I work to be tired? And other reasons that they would like to reflect.

**I: What about safety net program? If the people are involved, how do they get a benefit from safety net program? Do you think people will be changed from the program?**

**P:** There is safety net program. When I see safety net in the areas, most of the beneficiaries are screened by the local administrations and the support is provided based on the labor they are involved in the activities. For example, the women are involved in soil and water conservation in June or they are involved in any of the development activities so that they get the support from safety net program. They are graduated from the program to recognize that they are changed but when you see them, they are not really changed. As they were only involved in the program and when they are excluded after graduation, you see that the program does not change them. But in order to give the chance for those who were not involved in the program, the ones that were involved are excluded to replace them with those that did not get the chance. So, those that were involved are graduated and recognized assuming that they are changed and enabled to be self-sufficient but really they are not changed.

**I: What could be the reason for they are not changed? Is it because of the beneficiaries’ management problem or because the support is not enough to let the people changed?**

**P:** As to my observation, the reason is that you know the people that are involved are the poor that do not have anything to eat. So, these people were only dependent on the support and the support is small which could not help them to be changed. So, if the support is to change them, they have to have their own other duties otherwise if they are dependent on waiting until support comes, it will only be from hand to mouth as it is also small for the household. So, it would be important to support them with other development activities in addition to that of the food aid. Otherwise, this support will not change the livelihood of the beneficiaries. It will only be temporary solution.

**I: So, do you think safety net program is important or not? What could be done for sustainable change of the community?**

**P:** The support provided by safety net is important but the importance of the support would be better if the support could be provided in terms of materials by adding the money or the food of some months in which they can provide them the materials that could make the beneficiaries more productive such cow, ox, or sheep instead of giving them the food or the money. So, they can change their livelihood through the productive materials for the future otherwise once they are provided the money, they will finish it for food which will not be replaced tomorrow. So, the support should be something that can be replaced.

**I: Earlier you mentioned that women do not have enough time to prepare diverse foods. So, do you think that there are interventions in relation to workload reduction of women?**

**P:** There are some interventions but the question is whether they are practically applied or not. There are educations provided about the gender equality; there are no activities different for males and females. But the community is still not changed; especially the attitude of males is not solved. There is no one to be rational to consider that she was with me working the whole day outside and let me help here at home, especially in the rural areas. So, the workload on women is that they are involved in the agricultural activities like that of males and then they will come back to do all the home activities again. So, the community is not that much changed. They are not well aware of these problems which I can say they are not changed because the males reject some of the activities telling the female that ‘this is not my job’ ‘just it is your job’. To change the attitudes, there are educations during meetings that the people discuss on the issues of gender equality in that they are told there is no difference on the activities though there is biological difference. There are no activities different for males and females; we have to be involved in the activities equally. So, there are educations provided but still there is no change.

**I: What about introduction of technologies to reduce the workload of women?**

**P:** There are some indications with regard to solar energy for electricity (lighting) but not for cooking purposes. In most of the rural areas, there is no one without solar energy. But these are only for lighting, not for cooking foods and other purposes. Similarly, there are technologies to reduce workload of women in the agricultural activities but they are not introduced in the areas and there is no activity to introduce and promote them to reduce the workload even at woreda level. But there is a great change within the community with regard to lighting using solar energy in which previously it was the traditional lighting using spirit lamp.

**I: What about water, sanitation and hygiene services?**

**P:** With regard to water in Laelay Maichew woreda, it is good that there is access to water. In some areas, they have water hand-pumps but the problem is that there is improper usage of the pumps while using them. Most of the time, they are broken because proper care is not given to them. There are actually kebeles where they do not have even one hand pump. So, there are people that drink water from wells. Of course, there are some interventions for those that are using water from the wells to treat the water using water-guards. The health extension workers inform to use water-guards as it will kill the germs in the water but I do not have the information whether the community uses the water-guards properly or not. With regard to hygiene and sanitation, we said there is education but there is a gap on the application. Most of the time, we spend much of our time with students. While we expect student to come to school hygienic, they come unhygienic, stay the whole day without washing their hand, and come to school with their dirt. So, the people are not changed. There is a gap in its application though they are provided with education. So, it is difficult to say they are changed.

**I: So, what could be the gap? It can be anything that you observe.**

**P:** It is not because of lack of water that the people are not keeping their hygienic condition. It is because of negligence and because of lack of awareness creations. They simply say let us eat, nothing will happen. So, you see most of the community eat foods without removing their soils and muds. This is the problem of lack of predicting the consequences after eating foods unhygienic. So, it is due to negligence and thinking what will happen by eating this food otherwise there is no shortage of water. It is due to negligence and lack of awareness and also lack of predicting the problems that may come by eating foods unhygienic.

**I: So, what should be done? As you said there is training provided to them but still there negligence. So, what should be done?**

**P:** As the community is not yet changed, there should be repeated education, training, support, and follow-up until the community is changed and become self-sufficient. There is a need to follow-up the process until the community is changed so that we should not stop assuming that we have already delivered trainings and provided education. There is a need of continues education and short term trainings offered so as to change the community

**I: Earlier, you mentioned about malaria. Are pregnant women getting advice on the need to use Insecticide treated bed nets (ITN)? Why? Is it because of lack of access to ITN or because of sanitation?**

**P:** There is supply of ITN but they do not use it; there is a problem of proper utilization. Most of the time, we observe during our field visit that they use it for collecting straw and covering other materials. As they do not think ITN is used to prevent the diseases that can affect them, they do not properly handle the mater in a safe condition. So, the problem is due to lack of proper utilization. Otherwise there is no one without ITN and there is no one that does not know what and how ITN is used in the community. They know it but there is a gap on its proper use.

**I: What do you observe from the community? Is it because the people prefer the straw better than their health or they have another reason?**

**P:** If you ask the people whether straw is better than his health or not, they will tell you that health is better. Of course they are true, health is better than anything because it is if you are healthy that you can do anything. But the thing is that the community thinks that there will not be a problem and there is negligence that I will not be sick so that they prefer to use it for collecting straw rather than keeping it at home. So, what can you say about this?

**I: Are pregnant women getting deworming services? For lactating women, For adolescent girls**

**P:** What I know is in children that they are provided with tablets to protect worms at about six months if I am not mistaken. But I do not have the information whether there is deworming or not in the case of women.

**I: Do you think that women should be targeted for supplementary feeding?**

**P:** It could be done but what is important thing here is the supply. If there is supply of supplementary food, it will be good at least in some sampled areas in case the supply may not reach all. If you provide it in selected areas, the community will also be initiated to prepare similar foods at home. So, there will not be a problem to provide supplementary foods such as FAFA in selected areas, especially in the areas where they are expected to have shortages of proper nutrition.

**I: What about Vitamin A supplementation and school feeding programs?**

**P:** With regard to this, there is no any intervention within the woreda. There is no school feeding program for students and there are no supplies of vitamins. In the case of mothers, especially for pregnant women, there is provision of vitamin A and to prevent deficiency of iron. But I do not think there is in infants, children, and others.

**I: What about out-school adolescents? Are they supported to improve their nutrition and health condition?**

**P:** Focus is not given to adolescent girls, especially if they dropout from school. There is no activity about adolescent girls to know where they are? What is happening to them? And how are they becoming? They are already dropout and went to the community, and then no one considered them. There is nothing an activity focusing on out-school adolescent girls.

**I: Are the services youth friendly at health facilities or schools for adolescent girls (in-school and out-school)?**

**P:** While training is provided, it is only for 5 or 10 minutes during the regular classes in the morning being in front of the students where most of them are available. So, it is about giving some important issues that has to be delivered otherwise, it is difficult to say they have been given training because of shortage of time during the regular classes. If you call them another time for training, they will not come to the school because they are sons of farmers’. So, the training is only for about 10 minutes which is very short and they will not also capture the information. But as we give them repeated trainings, I cannot say there is a gap on delivery if not on its application though we cannot be 100% sure that they will capture the information within that 5 or 10 minutes training. But they need follow-up on how they are making the trainings practical. So, there is a gap on the follow-up; some of the good teachers tell the students to show what the students do at home related to the training provided. For that time the students may show what they did but there is a problem of forgetting after a time.

**I: Which of the interventions listed above do you think is most important for pregnant women? For lactating women? For adolescent girls?**

**P:** All are important points and cannot be observed differently. It is very difficult to differential the points listed. But as I said earlier, the major one is hygiene and sanitation. The most important thing is hygiene. Fifty percent of the diseases are due to lack of hygienic conditions. So, if we can protect 50% of the diseases by hygienic practices, the other thing that comes into concern is taking balanced diets. And again, if there is balanced diet, everything will improve following these conditions.

**I: In your opinion, which of the above interventions for the pregnant women are being implemented in a successful way?**

**P:** In the woreda, most of the mothers are going to the health post or hospital for child delivery. It is also rule and I can say it is successful. This time, there is no woman who gives birth at home. There is no woman that does not go for check-up. This time there is no advocacy to brink woman to the health center because they are well aware of the importance of health services. The community is changed with regard to this and a lot of promotion activities have been carried out on this issue for its effective implementation. The woreda is still working hard for this successful activity.

**I: In your opinion, which of the interventions targeted to the pregnant women was less successful? Why?**

**P:** Now, the major problem in the community is the hygiene and balanced diet. There is a problem within the community to practically apply what they know about the importance of hygienic practices and balancing the diet. So, we need to work hard giving much emphasis on these issues. If a mother takes balanced diet and keep her hygiene, she will produce healthy child and healthy citizen. We need to also work on awareness creations to the community and follow-up for the application of the activity in which it has to be confirmed for its implementation going down to the ground level. We should not come back after simply talking or giving trainings rather we have to take a sample and then crosscheck whether it is really working or not at the household level that have been involved in the training programs, support, and follow-up activities. Otherwise, saying we have provided training does not make sense. But going down to the ground and observing all the intervention is important, especially for the consumption of balanced nutrition and hygiene practices. It is said that keeping hygiene is good and washing hands is also good and the like but it is only becoming talk. So, I have the idea that it has to be confirmed by going down to the ground.

**Section 4: Implementation challenges and Community factors affecting access to nutrition interventions**

**I: What are the challenges to implement delivering the nutrition interventions that we have been discussing for the pregnant women?**

**P:** When we talk about the challenges, one thing is that there may be shortage of food or lack of access to supplies. So, if you need to ask the community to do this, there may be shortage in his house, for example, if they have teff they can only use teff and they may not have wheat. So, there could also be shortage of supplies which could be considered as a challenge. The second thing is there is lack of awareness, for example there is negligence on taking into consideration that this issue is important for me and it will help me to improve my health. I will consider this as challenge. If this is solved, there will not be a reason for the community not to be changed.

**I: What about the challenges that are observed at community level?**

**P:** As I said earlier, it is mainly the awareness. If you know it, you have to apply it. For example, if you ask them what are the diseases that are caused by hygienic conditions, anyone may tell you but it does not mean that it is applied only because they know it.

**I: What about community factors that limits the community for the improved nutrition and health of women and adolescents.**

**P:** Now, there is no challenge at community level. There can be a problem or lack of applying and awareness on the intervention. Otherwise, I do not think that there is like that.

**I: Is there a relationship b/n educational status of the women & access to the interventions?**

**P:** Yes, there is relationship. There is a great difference. For example, one educated woman and one uneducated woman have great difference. Let us take hygienic condition of the women in the rural areas; the educated woman is better in terms of personal hygiene and also in the hygienic condition of her house and also on the preparation of foods. So, there is a difference compared to the uneducated woman. There is a large gap that the educated woman is better than the uneducated one in all matters.

**I: How are the interventions accessible and convenient to the women and the adolescent girls? How do you explain the quality of the interventions?**

**P:** Mostly, the interventions are carried out not only to be accessible or convenient for women but also for the community in general. There is no activity that is carried out giving focus to women’s convenience. But it should not be too far for the community, at least near to the school, health center, and near to other services. So, it is a matter of making convenient for the community, not specifically targeting the women. So, there is no activity that assumes it should not be far for the women or it has to be convenient for the women. But generally, the interventions are carried out targeting the community, not giving a specific focus to the community.

**I: How do you explain the quality of interventions?**

**P:** Actually, I do not have much to say about this but women tell us that they go to the health center for check-up and get good health services. The women are getting the health services that they wanted. Till now, I have never heard of women saying I could not get health services.

**I: How do you evaluate the commitment of the intervention providers at your level?**

**P:** There are interruptions but the experts that are assigned to do the activities are giving services, and they are working hard to create awareness on the community but while working hard, you should not say this is enough as you think you said it today. It has to be daily; there should not be interruptions. If it is interrupted, it will also be forgotten. As we are giving trainings to uneducated communities, it should not be interrupted and you should not conclude by giving educations at six months or a month. It has to be repeated and I do not think it is enough.

**I: What other factors are inhibiting implementation of the interventions? How?**

**P:** There are ambulances but they are few in number. So, if one ambulance goes to one kebele, a pregnant woman who wanted an ambulance service in another kebele will get the service after she gets tired. Hence, there is shortage of supply of ambulance. So, there are incidences like this that are raised by the community.

**I: For these challenges that you mentioned, can you tell me of any solutions that your institution have applied to effectively implement the interventions for women and adolescent girls? Specify the each solution done for each challenges?**

**P:** There are educations provided about nutrition and others that are introduced recently, especially in these areas as there are schools that are included in the NGO projects. They are few but there are for example trainings focusing on the nutrition, training for teachers and students. Anyways, it should have covered larger areas otherwise we cannot say it is introduced by considering these sample areas. When we take as education office of the woreda, what we do most is on the hygienic condition. But there are challenges when we provide education about hygienic practices; for example, when you give training to a child, it will be a challenge if he could not apply it practically. There are students who come to school without washing face, eat lunch without washing hands, and other challenges. When you try to assess why these are happening, you will understand that you need an assistant at home to make it practical. So, if there is no parent that follows and tells him to wash his hands before eating, wash hand and face before doing something, it will be meaningless if only the teacher is working for the change. So, there is a need of assistance and we take the community awareness as a major challenge. So, the community has to help the teachers on these issues. The thing that should be done in the future is that there are committees at school from parents in which they should come to the school at least once in a week and should assess overall conditions of the child; not only his performance but also his hygienic conditions. They have to assess whether he is washing his hands before eating and follow what is happening up to individual households. If we consolidate this experience and change the child, the community will be changed as he is following that trend and this is what we are doing as a woreda education office.

**Section 5: Multi-sectoral collaboration to improve maternal nutrition**

**I: Do you feel it is necessary at your level to work with other sectors/institutions to address maternal nutrition? What about for adolescent girls’ nutrition? Why?**

**P:** Yes, of course. You will do the activities effectively if you work together. Otherwise, if you run alone, it is like clapping using only one hand. So, if you work together with education, agriculture, health, and other NGOs, the community will also have common understanding. For example, if education sector talks the whole day alone and if the agriculture and health are also talking alone, the community will be bored with these sectors and will not understand what the sectors are talking about. So, if you want to change the community, it is important to work in collaboration with other sectors.

**I: Which other sectors do you feel are necessary to work with your institution?How do you see the other institutions’ roles complementing your role in improving maternal and adolescent nutrition?**

**P:** Good. The stakeholders are agriculture, health, education, and other NGOs that are working in the local areas. So, if health works on hygiene and nutrition; if agriculture works on vegetables, cereals, and on home garden activities; and if education works on activities like that of hygiene and nutrition; then if they all go together having their own roles, they will make it successful. If this is made possible, there will not be any difference on the community. If they understand that the education provided by health is the same with what the other sectors are giving them, it will not be difficult for them to make it practical.

**I: How do you evaluate the level of collaboration among sectors in nutritional interventions? Why do you think is so?**

**P:** There are incidences where they do work together; totally we cannot say there is no collaboration. For example, heath sector are giving education to the adolescent girls at school about hygienic practices and nutrition. Similarly, agriculture is also giving educations about home garden vegetables at school. But the education provided is not complete; there are interruptions. There is some collaboration that has been done though there are some challenges in the program but its continuity is not that much good.

**I: For multi-sectoral action that effectively works to improve maternal and adolescent nutrition at your level, what kind of change in terms of the way stakeholders work together is needed? What type of resistance to the needed change do you perceive, or have you experienced so far?**

**P:** By the way, everybody is going to his activities on his own program. So, there are some interruptions in the program. For example, there can be an urgent activity after the health, education, and agriculture discussed to go together one day. That urgent activity may bring back some of the individuals and disturb the program. Instead of working the planned program what so ever comes an urgent activity, there are cases where we interrupt the planned programs. So, I suggest that it should not be like that.

**I: Is there coordinating platforms in enhancing multi-sectoral coordination in maternal and adolescent nutrition?**

**P:** Yes, there is. We call it “Memorandum of Understanding” (ውዕል ስምምዕነት). For example, there is memorandum of understanding on what should be the focus that education could work on health activities? Similarly, what should the health sector need to focus to work on education activities? What you have agreed on the memorandum of understanding; you have to plan it together with the education, health, and agriculture sectors through discussion but the problem is that there is gap on implementing the activities based on the plan. As I said earlier, we simply run to do only our program, not in collaboration with other stakeholders. So, there are a lot of gaps.

**I: To what extent does your institution participate in the multi-sectoral nutrition coordinating body at this level?**

**P:** As education office of the woreda, we work to help others. I mean without increasing the work burden we have, we try to help others. For example, if the health sector wanted to conduct education today, they ask us to send an expert from the education office and then we send an expert from our office. Similarly, when agriculture office wanted to conduct education in the school, they ask us to send an expert from our office and we do not resist on that, we become voluntary to help them. But the thing is that we should not wait until they activate us or until they told us that they have to work in collaboration. It is also good that our institution need to promote others to work together. So, we have some gaps on that.

**I: What needs to be done to improve the capacity of these bodies/platforms for effective coordination?**

**P:** There are gaps and these gaps could be solved through discussions. You need to meet every month and during the meeting you need to identify what has been done? And what is not done properly within that month? If they are clearly identified, the ones that are effectively done will be encouraged to be continued and the ones that are not implemented, you need to know who is not implementing? What are the gaps for its lack of implementation? and then solve the gaps through discussion. The other thing is that with regard to supplies. For example, while we give trainings, we ask them to come to the urban areas. So, there is shortage of budget to do the activity. Let us say for example, the health sector will call participants only one or two from each kebele or woreda because of shortage of budget and you cannot do beyond that if there is no release of budget from the higher administration. So, as there is a gap with regard to budget supply, it will also be good if this is solved for the future.

**Section 6: Other interventions that influence adolescent and maternal nutrition and health outcomes**

**I: In your opinion, why would delayed marriage (after 18 years) improve maternal nutrition?**

**P:** As we are now talking about the woreda, I do not think that there is a problem of early marriage. But, it is difficult to guess that there is no early marriage because there could be some incidences. There is a difference on the nutritional and health conditions in relation to age of the marriage. When someone tells you that this girl was married at the age of this, you will see something that you observe on her condition. You will see that she is thin; she is in a rough condition; and the like. But for those that are married above 18, there is a great difference compared to those married at early age.

**I: What could be the reason for the difference?**

**P:** The difference is that one thing the girl is getting married while she is not matured. She may not know what is needed for her, what is needed for the house. She is not getting married knowing all these issues. So, as she does not know about these, she will face so many problems that may affect her condition. As she is not getting matured in terms of psychological and physical status, there will be an effect on het living conditions.

**I: In your opinion, what would you say about increasing the space between each birth to improve maternal nutrition?**

**P:** This time, there is no education about increasing birth spacing because the community has already been adopted it. So, it is good that there is increased birth spacing.

**I: How much is the spacing between each birth in the areas? And can you also explain in relation to nutrition and health of the mother and the child?**

**P:** With regard to the birth spacing, it is ok. I have not seen mothers with short birth spacing (year after year) but there could be as I do not have much information about it. But I do not think that there is someone who does not have the knowledge of increasing birth spacing. It is good that the community is well aware of increasing birth spacing and this awareness has changed the community to accept is easily. But if you ask me about the growth of the child in the area, it is not good in terms of clothing, nutrition, hygiene, and other visible conditions. We cannot say children are growing well within the community.

**I: What are the challenges that affect increasing birth spacing or early marriage may be in terms of policy, religion, or others that observe within the community?**

**P:** There are no questions raised by the community with regard to the religion and policy issues. But as to me, there is some in relation to religion aspect, for example if he is priest or deacon, they do not accept these issues because it is associated with religion. They say what is the problem if it is the willing of God? And I do not think there is a problem with the policy issues though the community did not speak out freely.

**I: What about social or economic factors?**

**P:** I do not have anything about this.

**Section 7: Additional Remarks**

**I: Would you please say something if you have anything to add from what we have already discussed or something that you think are important for future activities?**

**P:** As you are asking me about women and adolescents, what I would like to suggest or give comments is that all the necessary trainings should be provided based on the gaps identified for pregnant, lactating, adolescents, and also children nutritional conditions, especially for women. The training provided should focus on women; separating the women alone. But we should not give training only to women; rather men should also be provided with training that has been delivered to women because they are supporting women at home. So, we need to inform them that this training is also provided to women and you need to make this training practical at home even in relation to the workload. So, this is still not solved because women are working with men outside the whole day and then they work many activities at home alone. So, I have the idea that there should be training provided to create awareness on the community. The other thing is in relation to working with stakeholders in which education, agriculture, and health are the major sectors. Here there are trainings provided with in the stakeholders; for example, health is providing training but it has to be beneficial for the women and the training provide by agriculture is also simply general for the farmers and does not focus on women. So, agriculture should understand and provide training based on what women looks like in agriculture, how is the utilization at home, how is she preparing the food, how are they using vegetables, and other important points that will be convenient and improve the capacity of women. In addition to that, there should be collaboration in relation to education. It also needs follow-up to check at what level and what is happening to the activities rather than simply giving trainings. So, I suggest that there should be follow-up and evaluating the activities for their application.

**I: Thank you very much for sharing me the information and time.**

**SUMMARY**

**Section 1: Common maternal (pregnant women, lactating women and adolescent girls) nutrition problems in the community.**

- As a habit, what people do in the areas is that they take the nutritious foods that are produced in the farms to the market rather than using it for their own consumption mainly due to lack of awareness.

**Section 2: Nutrition priorities in the woreda**

- In Laelay Maichew woreda, the priority interventions are the hygiene and sanitation practices.
- We do not have a budgeted resource for this prioritized intervention.
- Most of the diseases that are transmitted are because of lack of hygiene and sanitation.

**Section 3: Nutrition interventions that improve adolescent and maternal health**

- We do not have time to prepare and diversify our foods because we go for agricultural and other activities. So that we simply eat what we had yesterday for today.
- Lack of keeping hygiene in the area is because of negligence and lack of awareness.
- Focus is not given to adolescent girls, especially if they dropout from school. There is nothing an activity focusing on out-school adolescent girls.

**Section 4: Implementation challenges and Community factors affecting access to nutrition interventions**

- If you ask the community what are the diseases that are caused by hygienic conditions, anyone may tell you but it does not mean that it is applied only because they know it.

**Section 5: Multi-sectoral collaboration to improve maternal nutrition**

- If the different sectors talk the whole day alone separately, the community will be bored with these sectors and will not understand what the sectors are talking about. So, if you want to change the community, it is important to work in collaboration with other sectors.

**Section 6: Other interventions that influence adolescent and maternal nutrition and health outcomes**

- This time, there is no education about increasing birth spacing because the community has already been adopted it. But we cannot say children are growing well within the community.

**Section 7: Additional remarks**

- The necessary trainings should be provided based on the gaps identified for pregnant, lactating, adolescents, and children’ nutritional conditions.
- We should not give training only to women; rather men should also be provided with training that has been delivered to women.
- There should be follow-up and evaluating the activities for their application going down to the ground level.
