# Supplementary material for: Barriers to utilize nutrition interventions among lactating women in rural communities of Tigray, northern Ethiopia: An exploratory study
Source: PLoS One. 2021 Apr 30;16(4):e0250696. doi: 10.1371/journal.pone.0250696 (PMC8087028; doi:10.1371/journal.pone.0250696)
Supplement: S2 File — (ZIP) [file pone.0250696.s002.zip › S2_File.Doc/Woreda level and above key informants/121_IDI_Agriculture office_Laelay Maychew woreda.docx]

**Operational Research on Adolescent and Maternal Nutrition in Northern Ethiopia**

**Introduction**

Hello my name is kiros, I am from Mekelle Universty; we are conducting a research on the factors that influences the nutrition of mothers and adolescent girls in collaboration with the regional health bureau and UNICEF. Year participation is very valuable; the information that you tell us will be used to improve nutrition programs and services for women and adolescents in the region and the country. We will not share your names when we report our results. The interview may take 1-2 hours and I would like to thank you for taking the time to speak with us today. You have the right to withdraw at any time and I will use tape recorder. Are you voluntary to participate for the interview?

**Yes** No

| **Section A: Interview details**   1. Zone: **Central Zone of Tigray** 2. Woreda: **Lealay Machew** 3. Kebele: 4. Name of key informant: **Mr. Negacy Fitsum** 5. Institution of key informant: **Woreda Agriculture** 6. Interviewer name: **Kiros Tedla** 7. Date of interview: **20/11/2017** 8. Interview start time: **10:50AM** 9. Interview end time: **12:27AM** |
| --- |

| **Section B: Interviewee professional information**   1. Sex    1. Female    2. **Male** 2. Highest level of completed education.    1. No formal education    2. Primary education    3. High school    4. College education    5. **Bachelor degree**    6. Master’s degree    7. PhD 3. Discipline or field of educational training    1. **Agriculture**    2. Health (MD, nurse, health officer, midwife, pharmacy, etc)    3. Nutrition    4. Public health    5. Food science    6. Other (specify): 4. Current position: **Woreda Agriculture Office Head** 5. How long have you been in the current job/position:    1. ______ Months    2. **2** Years |
| --- |

**I:** Interviewer **P:** Participant

**Section I: Common maternal lactating women nutrition problems in the community**

**I:What are the common nutrition problems in the community for pregnant, lactating and adolescent girls?**

**P.** yes there are nutrition related problems among pregnant mothers. The problems are like having poor energy and unable withstand diseases or easily affected to different diseases. The problems seen in the child also includes direct physical effects like they will become underweight or having kg of below the standard during birth and they could also be affected by wasting and stunting. The other problem manifested among the children is internal diseases which could be known by measuring. These are the direct physical problems related malnutrition in which the children become underweight during birth and become dwarf or short. This is resulted from the mother as she do not eat balanced diet during pregnancy and if the child do not get enough care and diet after birth. The main problem is related with inappropriate feeding habit during pregnancy. So generally mothers are easily affected by diseases and there are also health problems which could be corrected using appropriate feeding habit like anemia and others. So generally having medication is right but it is valueless of used without appropriate feeding.

**I, What about common nutritional problems among lactating mothers?**

P. on lactating mothers it similar but lactating mothers living in the village when you look them they are physically very thin. This is resulted from food nutrition as she do not get what is required to her but she simply eat with her family with no change during pregnancy and after pregnancy. Yes there is food change during delivery but this is only for one week which is not enough and the whole lactating time she eats what is present like if shiro only shiro without any change. Hence, the mother could be affected by multiple problems if she could not get the appropriate food. The child will also suffer from absence of milk and this could be resulted in to several problems.

**I,** **What about common nutritional problems among adolescent girls?**

**P.** there is no any different new thing given among this group as they are part of the community. They are members of a family so they are also part of the problem and at the end this adolescents are mothers by themselves and the problem related with balanced diet is cumulated effect and we can see this among these groups when they become pregnant or during delivery as nutrition cannot be resolved once for all in the community. Hence, as the community has a problem related with eating of balanced diet they are also affected as they are part of the community. The community has the resource but they do have a problem related with utilization of the resources. Similarly there is no any different feeding style among this groups as well as the farmer’s say I have get good food when he has eaten meat for two or three weeks during holy days. Having meat by itself is not nutritional sufficient meaning eating meat for long time or constantly do not mean you have got balanced diet. But the community thinks like this; the community is rich in animal products like milk, honey and meat but they do not utilize it as they use for marketing purpose. Vegetables and cereals are considered as foods for poor; hence, there are problems like this and they eat only one type of injera with one type of sauces. The adolescents in the community have no other chance as they are living within the community and are part of the problem.

**I. how sever is the nutritional problem among mothers and adolescents?**

**P.** generally in all the three groups we have mentioned earlier; adolescents with age of 10-19 years have good nutritional status or affected very mildly. This is because they do not need extra-energy like the mothers as they are only living and feeding for themselves. Pregnant mothers had also better nutritional compared to lactating mothers as they can get diversified or balanced diet as they loss their appetite or hate food then there is a habit of changing food during pregnancy and this makes the pregnant mother to have better nutritional status compared to lactating mother. But after delivery the community assumes that she can eat everything she finds so there is a problem in getting good nutrition which can substitute the milk she gives; plus she is living in the village where is not only lactating her child she also involves in different tiresome outdoor activities. But the mother is suffering from lack of obtaining balanced diet which contains carbohydrate, protein, and vitamin which can balance the energy lost through her activities. So lactating mothers are highly affected as during pregnancy the women is not allowed to participate on different hard works and relatively pregnant mothers have the chance to feed diversified food compared to lactating mothers. By my own personal judgment not based on study but from what I have observed lactating mothers are highly affected by malnutrition.

**I. What about micronutrient deficiency? Like anemia?**

**P.** anemia is more common among pregnant and lactating mothers but not among adolescent girls. The most affected groups are still lactating mothers as I said earlier they are giving milk to their children and are involved in many other energy consuming hard works so they are the vulnerable groups then the other. The secondly affected groups by anemia are pregnant mothers as they do not get balanced diet or related to poor nutrition during pregnancy as most of our community living in the village believes in amount not quality and satisfy when they get only injera regardless of the other type of foods like vegetable where it is very important mainly for pregnant mother. So there is a problem related to this in our community but still the main problem related to anemia is among lactating mothers. But this is I do not mean that we do not have any change or their no community members who use vegetables; there are community members who cultivate and utilize vegetables but we still have problems related to this which needs to be worked on it.

**I. What about night blindness and goiter?**

**P.** yes there is goiter and night blindness. Goiter occurs starting from 10 years old and become larger in size through time and this is present in our community even though there is change from time to time. This disease was very common previously but now becoming very rare. Night blindness is also common and occurs among all age groups. This is also is the result of poor nutrition.

**I. What about diet related non-communicable diseases?**

**P.** Yes, there are non-communicable diseases like diabetes and hypertension in the community which are related with nutrition. But our farmers are not manifesting the diseases as they are always exposed to different works and physical activities which may protect the farmers from becoming severely affected by these diseases. Relatively these types of diseases are very common in the urban than in the rural areas.

**I, Is the height of the children or adolescents proportional to their age? Why?**

**P.** there is change from time to time but still there is a problem mainly among poor people. For example; there are a number of mothers who owned hen and feed their children but previously they were only use for market purpose. There are also community members who have milk and milk products and use for themselves. The community is utilizing the energy producing foods and vegetables because of the education given. But still we do have a community member who could not afford this even if small in number. The reason is first it should start from early pregnancy but should not only on the 1000 days; the other is related to genetics or hereditary from his parents and it is also related to feeding habit or our nutrition as weight could not be given other reason rather than our nutrition or our feeding habit but fro height it could be related with genetics or from parts genetics. So there are problems related with this in our community we still lagging behind and we do have both stunting and wasting in our community.

**I. What about overweight?**

**P.** overweight is not very common but still it could be present in the urban areas but not common in the village or rural areas but still there could be some individuals even in the village. But we can generally say it is not very common.

**I. About food insecurity?**

**P.** most of our community member is food secured; this is because the area is highly potential area and not susceptible to drought; the soil is highly fertile and could give high crop production but in other areas even though they have higher land but the crop produced is very small. The most important thing is that most of the crops with high nutritional value are produced and cultivated here. For example our main crop here is TAFF which is utilized by the community for consumption in the form of injera, Kitsa, porridge and soup. The other is white which is produced here; the cereals like been are produced here; so the community is not baying these cereals used to make sauces from other areas. The community is utilizing these types of foods during breakfast or during feeding and it is known that these crops have high nutritional value. We have also small farm irrigation potential to produce vegetables; so the community has the chance to get these vegetables closely from the market but previously the community were not using his own vegetable for consumption but now this is changed they produce to use it and for market purposes. But we still have food insecure family or community members mainly elders as they have no supporter and all their lands are given to other farmers in the form of partial or half payment or division from what is obtained from the land. The main important in this area to be food secure is the availability of different industries and university and the city is large so those youth can work here and get money and become food secured. But we do not mean that our all people are food secured; there are individuals who do not get farm land and having farm land but unable to produce because of health; there are also very large family members where the farmland is very small and the family members do not afford to get the food they need.

**I, What are the reasons for the mothers not getting balanced diet? It is related with lack of resource or lack of knowledge or awareness?**

**P.** the big problem is related with lack of awareness or knowledge as the village or rural community has better resource than the urban community but they fill only their stomach regardless of the type of food they eat and they do have low level of knowledge about what type of food has high nutritional value when eaten. But the community has all the resources and there is also lack of knowledge technically as well like for example eating the some type of food for long time like eating meat for long without any other additional type of food. There are also Scio-cultural barriers in the community like for example pregnant, lactating and severely diseased individual; even GOD will not consider them as sin if they do not fast. But this is not allowed in our community; for example lactating mother or mother who give birth now are not allowed to drink milk even it is present in their house during fasting even if they are interested to drink. She could not eat egg or meat where she has in her house because of the fasting; so there are such problems in the community. Except very small children; even children with age of seven and above are ordered to fast. Particularly pregnant and lactating mothers do not eat during the fasting of sent marry fasting which is for two weeks and the larger fasting period or called “Aby-Tsom” fasting for two months. There are also other different types of fasting periods like Wednesday and Friday, and others.

**I, Have you communicated or tried to solve the problem with religious leaders?**

P. we have communicated and rose the issue many times. If the pregnant mother is close to deliver and give birth she is allowed to eat but this is only for one month after delivery until epiphany of the child. The problem is after that as she lactates the baby for two or two and half and even some mothers for three years; hence the mother needs extra-meal or food in order to produce high amount of milk but she could not eat as it is fasting even though they like to eat they will not do it because they consider this as sine.

**Section two:**

**I. What priorities do your institution has in relation to maternal and adolescent health? Why?**

**P.** our first priority is awareness creation using the one to five net-works or structure of our farmer to discuses and takes as a big agenda about maternal nutrition. For where should it start; care should start from early pregnancy in order to have healthy child; and the child should get appropriate care starting from exclusive breast feeding up to six months and after that should get extra-food. So the awareness program is very huge and should start from early as the farmer in our community has the resources in his hand. The only problem is lack of knowledge or awareness.

**I. What about prioritized agricultural products for pregnant or lactating and adolescent girls?**

**P.** **yes** there is concerning the use of vegetables first we enable them to be aware of the benefit of these vegetables for example they know what are the other types of crops used with it. For example for a mother to feed her child she should use crops like TAFF, cereals, egg and vegetable. So these types of production and cultivation of crops and vegetables are given the with awareness creation are priorities by our sector. So we work on what pregnant mother should eat during pregnancy; what about for lactating and the child should eat to be healthy together with health sector. We also advice the farmers to practice home gardening cultivate vegetables.

**I. What nutritional prioritized interventions are allocated the most resource?**

**P.** the highest resource is allocated for farmers to increase agricultural production like irrigation, animal production, crop production and to achieve food security for all farmers. This is the area where the highest resource is allocated. Our first goal is to enable the formers to own the resources and then we will go to awareness creation together with health sector up to the lower level as we have a structure up to the community and they were given trainings previously and are working together.

**I. which of the prioritized interventions are effective in improving maternal and adolescent nutrition?**

**P.** the intervention targeting pregnant mothers is effective which are found in the constitution like pregnant women should not hold or try to stand heavy materials are becoming effective. Secondly pregnant mothers feeding style or habit and follow up and check up in health institution had improved very well and had scored big changes. There are also changes like both pregnant and lactating mother should not work very heavy works or the need of extra-rest is also doing well. But our big achievement is the change we brought regarding the utilization or consumption of vegetables as this was not consumed by the community previously even considered as taboo was now eating by everyone. The other is the utilization of animal products as the communities were not utilizing the animal products for themselves now they consume for themselves. Even religious related barriers are also becoming resolved though time as there are mothers who believe and eat meat or other animal products during fasting after delivery.

**Section three**

**I. What kind of interventions is in place to improve health of the PW to your level?**

**P.** pregnant mothers are excluded from governmental activities or works like soil conservation and if they are part of safety net program they are exempted from the activity starting from pregnancy and for one year after delivery; but she is given the payment. This is official and we do not involve them in any activities.

**I, What is safety net program?**

P. safety net is also called productive safety net where those food insecure individuals had get aid. This are two types first is those who could not work like elders and are given the aid yearly or for full one year without work. There are also other like productive safety net or public work where these are groups where they get the aid by work like involving in soil conservation, and other activities. Pregnant and lactating mothers are given the aid freely without participating in these activities. There is also emergency aid for the community which is given when the community is participated in soil conservation and other activities but pregnant and lactating mothers are also given the aid freely without participating in the activities.

**I. Is the emergency aid given to all pregnant and lactating mothers?**

P. not to all it is given to those who are selected from the community based on the criteria. For example one pregnant woman will not be included in the program as she is pregnant. But if she very poor she will be included and given the opportunity of rest.

**I. Tell me about nutritional sensitive agriculture such as home gardening and productive safety net? What you are doing to improve maternal nutrition?**

**P.** We are doing a lot of things within the safety net program like it has gender affairs and nutrition as one component. This safety net program is already planned and in practice and the community is expected to work for five days per month and paid for these number of days but the two days among this five days are for education on nutrition. Above 11,000 people are included in the safety net program and nutrition related trainings and education are given by both health sector and agriculture found at the community. We are also giving assistance or help and education using WDAs, HEWs and DAs on home gardening to cultivate different vegetables in their home.

**I, Is there any adolescent related intervention by your sector?**

**P.** As nutrition cannot discriminate or is the issue of all we give school base education to students as education sector is our one stake holder. We do have education for adolescents which excludes pregnant and lactating mothers and they work on water, health and agriculture. And nutrition is also part of the education; nutrition has its own manual here which focuses on how to eat or general nutrition. Education for adolescents is a providing education for those who do not get the opportunity to learn in the formal education and who do not able to write and read; this could be male or female.

**I. Which of the above intervention for pregnant, lactating mothers are being implemented successfully? Why?**

**P.** The safety net program based training given is very successful. Previously there were carelessness from the community on receiving the training but now the community had understand the value of the education on nutrition given for two days. This had shown changes and the need of extra-rest to mothers is practical in our area and any mother is utilizing the service. This has given to the mother another massage that we as governmental sector we say no you have to take rest as this is your right and this has influenced to their personal activities at home that lactating and pregnant mother should not lost her energy or should not do heavy works; they should get rest. Such awareness was created by our intervention to the mothers or due to exemption of the mothers from work.

**I, Why are these interventions effective?**

**P.** Because the community has the resources like they do have the crops or they produce crops and they do have the animals, irrigation and vegetables and their products in their hand. We ask them to compare from people living in the urban areas with themselves or rural; the rural people are not created to be always ugly; those living in urban are better simply because they do have good awareness. So we had worked repeatedly on awareness creation and we have brought the change. Secondly it is not because our work only; now each family or household has student or educated which could in collage or private education; but by any means they are exposed to the living style of the urban people. Thus then leads in to awareness creation it could be in nutrition or feeding style or as I said earlier related to religion pressure these are now becoming easier and easier. This is because the one of the family member is living outside the family in the urban areas and these influences to the awareness of the family and brings change.

**I. Which of the above intervention for pregnant, lactating mothers was less successfully? Why?**

**P.** Religious related pressure or influence on resource wastage like Teskar we have tried to stop many times and give education but still tens of thousands of birr is wasted. Others like epiphany and marriage ceremony this are the main source of wastage for money and a lot of family is becoming poor because of such activities which could be removed. We do have trials but the trials were not strong and we will do it strongly. We need to work on the effect religion on the mother and we should even study its effect like when do the mother should be free from the religious effect or fasting. As we need to have the logic but now we simply say it has an effect. The other problem is on awareness creation on nutrition as it is less than to the other programs; for example we are not working like we work to create awareness on productivity like animal or crops or irrigation and vegetation every day and night. But we did not work similar to this for nutrition by preparing its on manual.

**Section 4**

**I. What are the challenges to implement delivering the nutrition interventions that we have been discussing for the pregnant women, lactating women and adolescent girls?**

**P.** there is no any barrier for nutrition only than I said before like increasing productivity but the main problem is lack of focus. Why do not give focus is because the other programs like increasing productivity is measurable and have an indicator like you can transfer data or count the outcome and evaluate the performance. And everybody is accountable for any failure but nutrition is additional work that we have the resource so let’s use or modify our eating habit; it is done by willingness but it is not a must do job. And has no any accountability for its failure.

**I. how can we solve the barriers like for example how can we make nutrition get the focus?**

**P.** as there is no bigger issue than this; there should be owner of the program or nutrition expert. At this country to bring changes like educated individuals, creativity and researchers, all these are brought by human being. Human being can only invent and work if only they do have health and energy. So to fulfill this we need nutrition; hence nutrition should at least have owner ship at the prominent sectors like agriculture to give education on how the produced agricultural products should be eaten; then there should be a nutritional expert at the woreda level. So if we make like this it will work very well as it his responsibility; as it is also similar in the health sector as well they are not working very well in an organized way. For example when we get training on nutrition we try to work but when you get another work you will leave it. So if we can establish its own expert it will be successful. Secondly all the participating individuals at the lower level should also be evaluated and it should not be like catch and go. This should be evaluated and conduct meetings at woreda, region and the country level and should be identified the problems.

**I. What solutions have your institution applied to effectively implement the interventions for women and adolescents?**

**P.** we repeatedly do education to improve the awareness of the community but we do have problem related with having continues meetings and evaluating our performance. We are working very well on the nutrition of pregnant and lactating mothers but this should not be the only targets; we should also target the whole community as if the whole community have correct understanding about nutrition the our intervention will be effective. But if we only give education on nutrition on pregnant mother but the community had no awareness; it is valueless. As this may lead the community in to another dimension like lactating and pregnant mothers should only get care only during this time but not out of this time.

**I. what are the barriers related to the mothers which prevents from utilizing the interventions?**

**P.** there are several barriers; first there is economic problem like lactating or pregnant mother cannot get what she needs to eat because she lacks resources like money. The other is related to culture like they do have milk but they do not utilize for themselves even if there is pregnant or lactating mother. There is also the opposite to this like they do not have the milk or the resource in the house but have the money to bay but they consider baying as taboo mainly in the village. There is no goat or sheep in the house but there is the need like pregnant mother; but they do not do this by baying from the market. The third is related to religion by saying what could they say if I eat this or could they say sine if I eat during fasting will say the mothers. The other people also do not advice the mother to eat.

**I, How can we solve these problems or barriers?**

**P.** we solve it through continuous awareness creation. As there are also technical problems like what type of food should I eat, with what and others; religious and cultural barriers; we should give continuous awareness creation to solve these all barriers.

**Section 5**

**I. do you think early marriage have effect on maternal and child nutrition? How?**

**P.** it is scientifically known that early marriage has psychological problem to the mother and the mother also fill ashamed to hold the child in front of people as she is not ready to have baby. It has also a problem in giving care to the child related to nutrition. The main think why we say less than 18 years old should not marry from nutrition perspective is that she is not ready or mature to hold the pressure. Adding early marriage to the nutritional problem which existed with mother and over this she is not physically ready will multiply the problem in the mother. Hence; this affects to both maternal and child nutrition.

**I. is there early marriage now?**

**P.** now there is no early; we are now totally illuminating it and it is one of our success among the other programs. But still there are trials from the community to do early marriage but the girl will be controlled from school and if she is absent and told she is to be married; the family members will be imprisoned. I cannot say we make it zero for early marriage as there is such trial and thinking and the community can do it yesterday even if they are not doing now as there is such thinking in the community. But still we have made very big progress and achieved bigger changes regarding early marriage.

**I. What activities or programs are there to prevent early marriage in the community?**

**P.** this is because we make serious meetings or evaluations not like nutrition where there is no accountability but here we do have accountability and serious meetings and the one with problem will be punished like for example the family of the daughter allowing the early marriage will be imprisoned; this is practical as there are family members imprisoned after wasting all the resources for the marriage and such people are used as examples to teach the community. Hence; as the community has this idea and do not do early marriage; it is also part of legal issue and the main agents working are lawyers; schools or education sector, women affaires together are working and it is effective. .

**I, any additional we should work to prevent early marriage?**

**P.** I do not see any problem related with preventing early marriage as the rules are working very well and the awareness of the community is also improving because of work done at school and though WDAs . So as it is working very well; what is needed is only to strengthen this but we do not need any new thing.

**I, What are the community related barriers related early marriages as you told me earlier that there are trials still to do early marriage?**

**P.** this is related to back wardens as there is a fear in the community that if the girl matures she may have boy friend by her own interest without the consent of the family members. This considers the community as a big loose or looser or taboo as they believe that a girl should go through her family willingness and should marry through the community culture. Hence; the family members marry the girl early to avoid this an necessary or out of law friend ship of the girl which is considered as taboo in the community.

**I. what do you think should be the gap of birth interval? Why would increase the space between each birth improve maternal nutrition?**

P. for me I choose to be at least three years gap. First for the child to have good health and nutrition; also have psychological effect if give birth immediately as they do not get the actual care from their mother and the child may develop stress and even affected disease as most of the mothers were giving birth within one year or two years of first birth. This means the child would develop stress and cannot get milk up to two years if the mother had pregnancy within one year. But if the mother had got pregnancy after lactating for two year she should give birth at third when the child is matured and he has got all the necessary things from his mother; so he will not be affected by malnutrition. The mother is also affected if she give birth consecutively as she is giving with having poor nutrition and her body is not ready to give birth without interval. So the mother and the child will be both affected or become victims.

**I. Is the family planning services doing well and why the programs related to family planning are effective?**

**P.** Family planning services are doing well and there are a lot of things done on this issue. Awareness creation has done many times and it is done with plane or goal inside the health sector like how many mothers are utilizing the family planning. The awareness creation is done also through WDAs or gujile lemat repeatedly and the mothers are becoming aware of the program and utilizing it. The other thing is now the family members are educated or at least know the importance of family planning and they do not need consecutive birth by themselves. There is also full access of family planning services including the treatment and it is close to their home. I cannot say that we have achieved 100% but we have done very well.

**I. Can you think of any other opportunities to prevent early marriage and birth spacing?**

**P.** For both nutrition and family planning we should target school; even though they may found within the subjects given like biology, civics but there is no subject which gives only nutrition and the importance of family planning. I believe that nutrition should have its own curriculum and given starting from early schooling; and then own many experts as we do not have more than or bigger than this issue. This means we can change the awareness of the students or adolescents which are the mothers of tomorrow and the male are the fathers of tomorrow so that we will create healthy society. Every community member has a student and if we work on student from early on nutrition, early marriage and family planning they can make or translate in to their life easily. The importance of this is; first it benefits the student himself as they will be fathers or mothers for tomorrow. Secondly every community member or family may have one or two students hence they can influence their family members. Hence, I would be very happy if nutrition has got the attention and given in the form subject in the schools.

**Section 6**

**I. Do you feel it is necessary to work with other sectors to address maternal and adolescent nutrition? Why?**

**P.** Yes, it is must as the community is ours for all the sectors and nutrition cannot be given to one sector only to work. For example it is mandatory for us to work with health sector, with educational sector as it reaches every household and we use the students for teaching purposes. We also need to work with affaires like young and women affaires; hence we can reach all the family members about nutrition. These are our direct stakeholders and there are also other stakeholders were their value or contribution is very small.

**I, Tell me the role of your institution in working or complementing the role of the other sectors to improve maternal and adolescent nutrition like how do you work with health and others?**

**P.** with health sector and women affaires we directly meet through pregnant and lactating mothers as we all work on them. The women affaires have their own structure like Guijile Lemat which reaches up to the lower level or community; hence to get the mothers it is must to use this organization or structure. And we are working with these sectors using this structure manly on nutrition for example; the health extension teaches to pregnant or lactating mothers on what they should eat and the agricultural sector should work like when it is produced, how it is produced, and how it is processed; so this is done collaboratively. The amount or quantity of the food should also be worked by the agricultural sector. The health extension may have only the above mentioned activities but when to produce; how to produce, how to handle the product after farm is dealt with agricultural sector; hence we need to work together. The women affairs office mainly works on mobilization and awareness creation of the mothers in making the mothers confident to come to front and get the intervention. With education to make the students understand about nutrition and get ready for it. They also give or arrange us time to teach the agricultural expert to the students about nutrition.

**I. How do you think should be worked or what kind of change should be done the way the stakeholders work together?**

**P.** the first thing is the one I told you earlier that nutrition should have his own expert at woreda level. It could be very difficult to have experts in all the sectors but we can put on the most important sectors then the other stakeholders would become supporters as it is his only activity he can design how and when to work with agriculture, with health, with education and other sectors as well. There will not be forgetfulness and lack of focus as there is responsible person working on it; and the accountability and the focus will come. Hence; for me it is better if we have an expert who works on nutrition only and coordinates to all the other sectors.

**I, Tell me how should be the platform or the structure the multi-sectoral collaboration to be functional?**

**P.** for me it should be in the agricultural sector as we do have DAs or agricultural extension workers working at community on seeds or crops. Here the nutritional expert with all the manuals on how it should work and other planes and strategies; it could also be within the health sector as they are also working on nutrition if they have the same responsible body working on nutrition. So I believe that nutrition should have its own expert to work consistently.

**I. Are you working with these sectors collaboratively for example with health sector?**

**P.** Yes, for example the safety net program; we are working collaboratively in the safety net program and other at the lower level or community based on our focus area.

**I. What type of challenges or resistance have you experienced when you are doing collaboratively?**

**P.** when working collaboratively there is a problem mainly with appointment as when the health sector is available the other sector may have another agenda or may not come or absent. And there is no mandate to order or account for a person working in the other sector; there is a problem related to ordering even among the officials like the authorized body cannot force for the lower workers outside his sector. So these are the problems I said we need responsible body or nutritional expert to solve such problems.

**I. To what extent does your institution participate in the multi-sectoral nutrition coordination body at this level?**

**P.** yes there is, there are committees established for example there is stream committee and budget was allocated in the past in 2006/7 EC and there were activities or movements at that time. But starting from that time the committee is not functioning very well. They were trained and there were from education, health, agriculture. Budget was allocated to work on awareness creation on nutrition.

**I. Why is the committee not working very well or what are the reasons?**

**P.** As I said earlier the main problem is lack of focus and giving priority to your sector only. If there is budget you will work as there is budget simply because there is budget; if there is no budget you will shift towards your area or another program and give all your focus here and the nutrition activity or activity of the committee will lack the focus.

**Section 7**

**I. Any additional comments or suggestions that we have discussed?**

**P. F**or me what should be in the future is; first if something is started it should not be done if it is not to be practical and energy should not be wasted. Any way when you are working this project you have invested a lot of energy and budget; for example you may be outside from your home for a month and you have other activities left behind so if this project or data do not bring practical benefit to the community; all your energy and the budget is becoming valueless. When the information collected is becoming very large and having very important information but left out without any benefit because of the size as it become very difficult organize all the information and only remain in the papers and on shelf. What I want to say is we have to be concrete and reach up to the local community and dealt on how this should be implemented. For example we are over burdened with many programs and we only implement only one of the programs and forget the other. This is not because the other program is not important but due to the over load. So there should be owner ship for nutrition as if there is no ownership it could not be effective; so we have to take this lesson. If we work like this the lower community will also be benefited. Mainly concerning nutrition previously there was curriculum like “Niro-Zedie” but now it is not present in our curriculum. So for the future we should include as a curriculum and give education. The other is the awareness program as we starting from the higher body are giving less focus to awareness from time to time but for me it should not be left but at least we select the problems like for example stunting in Tigray it is very common we are only above Affar. When you heard about this it immediately feels and starts working but then lift it; so we need to continuously evaluate and meet to discuss the issue. We need also to evaluate what is done in the lower level; do they have the plane and do they achieved it; like every other sector is doing. So it needs constant monitoring and evaluation process and capacity building but if it is still by word meaning by saying do it from the higher body; we do receive such orders from the different higher officials and it is no more functional as we do have many other programs and planes to be worked we could forget it. For me I do believe we should make nutrition functional by creating conducive environment but the others I have said it before.

**Summery points**

**Section one:**

Both pregnant and lactating mothers are affected by malnutrition and to some extent adolescent girls.

**Section two**

- Our main priorities related to improving maternal nutrition is awareness creation as they do have the resource but poor utilization.

**Section three**

- Our main intervention regarding maternal nutrition is exemption of the both lactating and pregnant mothers from work during safety net program and emergency aid activities.

**Section four**

- Our main barrier related with to implementation of the nutrition interventions is lack of focus as nutrition is not our priority and has no ownership.

**Section five**

- We have worked on preventing early marriage and increasing space of birth interval but there are socio-cultural barriers.

**Section six**

- We are working with health, women affairs and education but our organizing platform needs reform like allocating responsible body or nutrition expert at the most important sectors.
- **Finally I have finished my questions and I would like to thank for your time, patience and answering all the questions. Thank you very much!!! Thank you!!!**
