# Supplementary material for: Barriers to utilize nutrition interventions among lactating women in rural communities of Tigray, northern Ethiopia: An exploratory study
Source: PLoS One. 2021 Apr 30;16(4):e0250696. doi: 10.1371/journal.pone.0250696 (PMC8087028; doi:10.1371/journal.pone.0250696)
Supplement: S2 File — (ZIP) [file pone.0250696.s002.zip › S2_File.Doc/Woreda level and above key informants/122_Zoneal Women association_Axum_Lalay Machew wored.docx]

**Operational Research on Adolescent and Maternal Nutrition in Northern Ethiopia**

**Introduction**

Hello my name is kiros, I am from Mekelle Universty; we are conducting a research on the factors that influences the nutrition of mothers and adolescent girls in collaboration with the regional health bureau and UNICEF. Year participation is very valuable; the information that you tell us will be used to improve nutrition programs and services for women and adolescents in the region and the country. We will not share your names when we report our results. The interview may take 1-2 hours and I would like to thank you for taking the time to speak with us today. You have the right to withdraw at any time and I will use tape recorder. Are you voluntary to participate for the interview?

**Yes** No

| **Section A: Interview details**   1. Zone: **Central Zone of Tigray** 2. Woreda: **Axum** 3. Kebele: 4. Name of key informant: **Miss Freweyni Yibrah** 5. Institution of key informant: **Women Association** 6. Interviewer name: **Kiros Tedla** 7. Date of interview: **20/11/2017** 8. Interview start time: **4:00PM** 9. Interview end time: **11:34PM** |
| --- |

| **Section B: Interviewee professional information**   1. Sex    1. **Female**    2. Male 2. Highest level of completed education.    1. No formal education    2. Primary education    3. High school    4. College education    5. **Bachelor degree**    6. Master’s degree    7. PhD 3. Discipline or field of educational training    1. Agriculture    2. Health (MD, nurse, health officer, midwife, pharmacy, etc)    3. Nutrition    4. Public health    5. Food science    6. Other (specify): **Accounting** 4. Current position: **Vice Head of Women Association (was delegated by the head as the head was not present during the interview)** 5. How long have you been in the current job/position:    1. ______ Months    2. **10** Years on women related issues |
| --- |

**I:** Interviewer **P:** Participant

**Section I**

**I, what are the common nutrition problems in the community for pregnant, lactating and adolescent girls?**

**P.** when we see from time to time as we all know there is change in economy as region and as country and this is also true in the households as there is also economic change and they do have the resource. But there is no awareness on the community and we have been working to improve the awareness with different stakeholders when I was in the woreda or in my previous work and I was member of the committee who works in food security. I learnt from this that we do have a problem on utilization regardless of the resource is there. And as leaders we have been observing and discussing to each other. If you say are the problems there; yes they are present. Mainly in some households even though they do have the resource you see very thin child and treated for malnutrition and given Plumpnet and resist the malnutrition are very large in different woreda and Kebelles. This is only because of malnutrition but not due to any other disease. But when you ask that is it because the households have no food or resource it is not the reason; it is lack of balanced diet even among those having the resource as they only eat meat as they consider eating meat is valuable and considers themselves as getting good nutrition. There is also a problem related to utilization of cereals and vegetables. But now there are changes because of the different meetings among mothers; for example previously as I am born in the village I have never seen a farmer baying onion and tomato from the market but now I see farmers baying vegetables and others from market. But equivalent to this there is a lot of work left to be done. Comparing the resources present and the economical growth still the children, mother are not benefited to actual level. When we see who is the most affected; there are mothers who are affected and there are also children but I have not seen among the adolescents. But still we can see on the adolescents that they do not get the actual calorie when we see their physical; but we Tigrians are short and thin naturally; hence it is very difficult to evaluate the youngsters as they are young and childish. But malnutrition is very visible among children and mothers. So there is work to be done to make the mothers and children to be benefited.

**I, You told me that children were given Plumpnet; what about pregnant or lactating mothers?**

**P.** Before two years in Rama where I had been working but I do not have the information now; plumpnet was given to children with severe malnutrition as treatment; and Fafa were given to thin children and mother with oil and they were given only for the affected mother or child but not to the whole family. They were also advised to feed only to the affected child or mother but should not share with other family members. More than this there is also education given by the professionals to improve awareness on what was missed to become the child or mother very thin and on how this could be treated.

**I. What about micronutrient deficiency? Like anemia?**

**P.** Yes there is anemia as I told you previously it is because the mother do not get balanced diet and in the low lands there is also malaria and other problems cause anemia.

**I. What about night blindness and goiter?**

**P.** Night blindness I have not seen but when I was a child I had seen but now I have not.

**I, What about goiter?**

**P.** Goiter depends on the areas; in the area where I had been there were no but I don’t have recent information. Adi-ahferom there were signs of goiter but with different vaccinations given I don’t have information regarding the changes.

**I. About food insecurity?**

**P.** food security activities were done and had shown big achievements from time to time; there are many individuals who had nothing but using the established packages like those who work on agriculture using the new technologies and most individuals have achieved their food security. We cannot say all the individuals have food security at this time. There are still food insecure individuals even though I do not have the percent; hence there are still people with food insecurity. But most of our people are out of this meaning they do have food which can feed them yearly or for one year adding with daily work and agricultural product.

**I, Which women groups meaning from pregnant, lactating and adolescent girls are the most affected by the above mentioned nutrition problems?**

P. I do have a problem in number as I said it is present in all but more on pregnant women as she hold two lives. To put consecutively the highest malnutrition is among pregnant mothers followed by children and then lactating and adolescents respectively

**I, Why pregnant, and lactating mothers are at higher risk for malnutrition?**

**P.** the problem is related with lack of awareness; as pregnancy is considered as gift only with no need to do special things like food by the mother and the husband as well; they do not consider that she has second person inside but she thinks like she was with no pregnancy. Hence; there is lack of awareness on the need of extra-meal during pregnancy as the mother should eat for herself and her child. So this is the problem.

**I. What about on children and lactating mothers?**

**P.** it is similar lack of awareness also on the children and lactating mothers; we cannot say being poor or lack of resource as there are very thin children and mother even among the rich people. What she must do and what she want to do like pregnant mother as lactating mother is also feeding two lives so it is if she eats she can feed the child but there is no one who thinks like this. So if the mother does not get enough food she would lost her weight duet to lactating to her child.

**Section two**

**I, Do you think it is necessary for your institution to get involved in work aimed to improve maternal nutrition?**

**P.** yes, we are also working mainly with health and agricultural sector; in some occasions on awareness creation and intensive training consecutively; not alone as trainers when we told by these sectors we also get a number of new ideas and we start to practice and share with our family members in our home. So if we work repeatedly we can bring change as we had brought other changes.

**I. What priorities do this woreda has in relation to maternal and adolescent health? Why?**

**P.** It defers from woreda to woreda but we are talking about zone as the woredas have their own priority. The sectors I have mentioned earlier like agriculture they do have office of food security and this with other affaires like women and young; they work on awareness creation. There are also kebele based activities on mothers to protect before they are affected by malnutrition and included in the aid to use all the resources in their house and if they are already affected by malnutrition to go to the health facility; so there are such activities. HEWs are mainly working these activities and they also show them how to prepare porridge and how to prepare soup from different crops by calling the pregnant and lactating mothers and give education by entertaining the mothers. They advice to use resources available in the house as at least there could be egg, milk of sheep or goat and if they do not have money they also could use the available crops or animal products; this are the daily works of the HEWs.

**I. Is your institution involved in such activities? How?**

**P.** yes we work through the women association found at lower level and women leg and these groups are working with health extension workers and women developmental arm. This is because the HEW worker to get the community they should go through these associations.

**Section 3**

**I, Is there any intervention given through your sector which is very effective?**

**P.** for effective interventions we have participated the gujile lemat or women developmental army (WDA) are two types there is Gujile lemat for male and there is gujile lemat for female. And when we measure the strength the female WDA is very strong than the male. The reason is that they believe that health and any other issue is very important for them and they meet always. They also have very strong performance even though there is limitation on both of them. And when you ask them they answer first health related issues.

**I, Do pregnant or lactating mothers get all nutrition related interventions?**

**P.** after the slogan which is “mother should not lost her life while giving life” there are works but before the health professionals were not giving the appropriate care but after the slogan giving birth at home is considered as the mother is died. As if she give birth at home the mother may be exposed to excessive bleeding or may have got positional problem and other problems may be occurred so these types of problems could not be solved at home unless the mother comes to the health facility. What services she get are like check up but there is a problem here like the pregnant mothers do not start early or at the right time. They should be followed for four phases but they only follow the last two phases; they do not come early.

**I, Why are these pregnant mothers coming late?**

P. This is because they do not feel any discomfort so that they say why should I go to the clinic as I am healthy. And they say I am ok but when the delivery date is approaching they come to the HF; this could be due to fear or to see the status of their child. This is for some mothers as there are also mothers who check and follow their pregnancy at the health facility fully. And when they come to the health service they are given all the interventions including treatment for anemia and others is given. Home delivery is achieved in some kebelles and they make it zero but as woreda and zone we are still not achieving or not zero. Mortality is decreased and the post natal follow up and check up are also present.

**I, Are pregnant and lactating mothers advised on the need of extra-meal and rest?**

P. It is mainly done by the health sector but our members also work with them like WDA and the health professionals give advice as at this time there is no mother leaving without any advice; they are advised.

**I, what about on water quality, sanitation and hygiene?**

**P.** we work collectively as we are at zone and the woreda or kebelle are so different as they have their own hierarchy. So if we talk about any sector there is responsible person for that sector like if we take water there is a professional who works at the kebelle level and the other sectors like agriculture they do have their own structure up to the kebele level who works with other sectors. Mainly the last two years related with water; those with high prevalence malaria are advised to clear stagnant water and keep clean their environment and their house until they fulfill the graduating packages as they will be graduated and it is one of the components of the graduating package. They are graduated based on their hygiene like how the drinking water is kept, how the kitchen or the baking and cooking area is, how the overall are the feeding utensils, and other environmental and how is the living houses of animals and human. If they have done this they will be certified and recognized as model family. This is done with our women association with the health extension worker.

**I, Tell me all the criteria used to select the model family or to graduate the farmers or mothers?**

**P.** I cannot name all the criteria but as leaders we are invited in different graduations and heard and see some of the criteria’s. For example we were invited in one model kebelle because they made free from feaces or avoid open defecation. So if the household is to be graduated it will be assessed internally like how the ITN is used; how clean is the house and the feeding materials or utensils; how is the traditional baking look like to where is the direction of the smoke to inside or outside; how clean is the house; is the drinking cup tied with the water reservoir and is it clean; how about their surrounding is it clean; are animals and human living separately or together; as the animal should kept at different place; what about toilet do they have toilet with washing hands. This all are considered during the graduation.

**I, Tell me about safety net program? What it is doing in relation to maternal nutrition?**

**P.** Safety net program is given for those who are poor in order not to move other places to avoid the wasting of time and energy and work in his place by conserving his place and also working for his own and getting the aid. Mothers are also benefited from this program based on the work and they do have their own work which is different from the work done by the male. They are also exempted from the activities during pregnancy and for one year after delivery. This is the rule but is it practiced in all the areas I do not know but previously there were knowledge gap but now they know that it is there right it get the rest.

**I, Is there treatment given to the pregnant or lactating or adolescent girls to prevent intestinal parasites?**

**P.** I do not know but I know that pregnant mothers are given health service freely.

**I, What do you do on adolescents like youth-friendly service?**

**P.** I do not know but when I see from the health sector even there were some NGO aids here and it is related with health and there is work done by this. But still it is not distributed to all the youth.

**I. Is there school feeding program?**

**P.** yes there are schools who give porridge of Fafa to students in central zone of Tigray.

**I. Where are these schools?**

**P.** I don’t know now but they were feeding students when I was in Mereb-Leke and last year a number of materials were given by NGO.

**I. You told me that children were given Plumpnet; how is this plumplet given to the children?**

**P.** they give them by measuring using I forget the name- MUAC; it has three colors if the child is in the red color she has problem.

**I. What about for mothers?**

**P.** yes they are also measured

**I, What was given after measuring and for whom?**

**P.** as I have told you this plumpnet and fafa were given; plumpnet is given for severely affected or malnutrition child and fafa is given for child with moderate malnutrition. The mother is also measured. There were problems from the mother like firstly they were not voluntary to screen their children and after measure they complain because they are not given plumpnet to their children while the child is normal. Child feeding is also has changed from time to time.

**I. Why are these interventions effective?**

**P.** Institutional delivery is effective because all other sectors are also involved but previously it was only given to one sector and then the communities were not addressed in creating awareness. But now for mother to give birth at health institution there is no health professional talking about it; there are no leaders who do not speak about mother and during meeting we evaluate it and we also work on it; So that the changes are achieved because we make it ours and work together.

**I. Which of the above intervention for pregnant, lactating mothers was less successfully? Why?**

**P.** there is no unsuccessful because of our work or because we did not work but do not stop teaching and working; there are very successful interventions like institutional delivery, feeding habit or nutrition in general to some extent. But we need to work strongly without any rest as there is no anything which could achieved immediately as it needs time. For example our plane is to stop home delivery but still is not stopped; it is not only home delivery which we do not stopped but maternal death is not still stopped for example 11 mothers were died last year because of delivery. But our plane is to stop home delivery and stop even single maternal death as zone and a region. This shows that we need very hard as before there were not focus given regardless of the maternal death but now everyone from the professionals up to leaders there is accountability if mother is died during delivery. But there are husbands who say she should deliver at home and there are also family members who have backwardness or with old thinking. Hence; if the mother died we accuse the husband as he is responsible for death of the mother and we teach the community by taking the husband as an example. There is also delayed presentation of the pregnant mother during delivery or after she had become tired but it should have been coming immediately when she had felt the pain. But now there are resting rooms prepared for the pregnant mothers to live in the institutions when there delivery time is close and they feed porridge prepared by the government.

**I, Why are the individuals resisting to send to the health facility to give birth?**

**P.** culture as previously it was not known giving birth at health facility and the community believes Sent Marry will help her and she is there for all bad or good happenings; so she should give birth at home.

**I, Are there interventions used to solve these challenges to improve maternal and adolescent nutrition?**

**P.** yes there are like together with women association and women affaires we went to the house of the resisting husband or family to counsel and advice them; also to know why they are resisting but still there are mothers died but still we are working to solve the problem. Now all pregnant mothers in the kebele are registered by the health extension worker and the women association. Hence, the pregnant mothers will be categorized in to the WDAs and these WDAs will follow who is correctly following her pregnancy and who do not go to the health facility. If there is a problem from the husband or family in sending the mother to the health service; they went to home and try to convince and if she died he will be used as example to teach the community; to prevent from happening for the second time.

**Section 4**

**I. What are the barriers that prevent adolescent and women from using the nutrition interventions that we have been discussing for the pregnant women, lactating women and adolescent girls?**

**P.** As I said before the problem is related with awareness as they do not think what would happen if they are died to their children and family. They only think of the good think or surviving but not the other side like death may happen; so such problems are hindering the mothers to use the services. When the mothers are told to stay in the health facility until delivery they refuse and there are even few mothers who want to their home without permission before giving birth. Their reason is how my children or my family will live without me for two or three weeks. The husband is also do not pressure her to stay in the health facility rather he prefers to stay at home because of lack of awareness.

**I, How about community related a barrier like religious related believe for example there is Sent Marry here and Sent Marry there at the health facility?**

**P.** yes there are but now there are changes even though there are also still such type of believes in the community. That is why there is home delivery and death of mothers still now but if the mother went to the health service she would have been saved her life as there are health facilities everywhere. This indicates that there are still works left to be done.

**I, What about from the health provider or health service related barriers who prevents mothers from utilizing the health service?**

**P.** Previously meaning before the slogan there were carelessness related with the health professionals like insulting the mother, un appropriate delivery and they were also saying the mother like you are not doing for the benefit of me and others were manifested to the mother either from the female or male working in the delivery. But now they know that they are accountable and get difficulty if they do not give the right service. But I cannot rule out the presence of unethical professionals for example before two years there were one event occur in Adwa hospital by one health professional which were even covered by mass-media and the health professional were accused. But most of our health professionals work appropriately and know that if they do not do the right thing they will be accountable for that. The mothers are also aware that they have the right to get the service if they do not get the appropriate service they can complain and this is also is known by the health professionals. The health experts have their own catchment area so they know that how the mother is advised and get the service. So now the mothers are appreciated to come to health facility by providing even motivators or services. Health professionals are also given duty when they give service at night but this could have limitation in different areas related with budget but it is legalized by the government.

**I. how can we solve the barriers mentioned above and do you think this are easy to solve?**

**P.** there is no any problem which could not be solved and it is not easy how far we come and achieved goals. We can take our previous achievements as positives like children vaccination as there is no nay problem regardless of the place of delivery at home or at health facility his mother will brought to the health facility; this is because the mother had understand the value of vaccine as they know that if measles or cough is entered in one family a number of children were died so they know this; so if there is vaccination they vaccinate their children as they have seen the value of vaccine as the number of children died because of such diseases is decreasing after the vaccination and even if the children are exposed to the disease they see they are not diseased severely. So the problems related with home delivery and the feeding habit or nutrition of the mother and her child will be solved if we work hard by learning from our previous achievements.

**Section 5**

**I. do you think early marriage have effect on maternal and child nutrition? How?**

**P.** I do not believe on nutrition but I do believe that early marriage affects the mother and her child. And it is a problem to the mother if she gives birth before 18 years old as she is not physically strong she may face difficulty to carry the baby. But if she is above 18 years old she is matured and physically strong so she can carry the child and even have better nutrition. But as to me when I see from nutrition perspective she has no matured physical and could not able to carry the child during pregnancy if she is lower than 18 years old and may be exposed to fistula. Such types of problems had been occurring and even occurring now. Even 18 years old is not enough to give birth as giving birth at 18 years old and after some years like at 25 is very different. For example myself the child I born when I was in the 20s year old and the child I born when I was in the 18s year old is very different; when I was in the 18s year old when the baby cry I was beating him as I do not know what he wants but he may be hungry or may have any other disease. So imagine if she gives birth lower than 18 years old; this could be worse than my experience as she is not mature and child for herself. So it is true that there might be many problems related to this; as it is difficult to the mother to carry the husband and the whole family.

**I, You told me that when the age increases the care to the child also increases; could you tell me the actual age where the girls should give birth?**

**P.** It is not scientific based but from my experience; as the 18 years old more give priority to sexual desire but to give birth for me it should be above 25 years old as they are becoming mature but if it is before the mother will not give appropriate care starting from understanding the need of the child up to feeding the child. As when the baby cries you could not understand him why he is crying but you simply complain about him as you could not understand him.

**I. do you think it is promoted in the community?**

**P.** yes it promoted in the community as our party or TPLF had started this when the party was in struggle as well. But still we cannot say it is totally stopped as there are still girls who stopped their education because of marriage and there are also many girls who are deceived by male and stopped their education. There are school based cotrolling mechanisms but the problem is it is only work for the winter season or during February but the family members can send their daughter at summer as there is no education at summer and there is no teacher who stops this. There are also like sending the daughter without wedding silently in the lowland areas. But if this is recognized this will be stopped by lawyers; women affairs and teachers; and the daughter will continue education and the male as well.

**I, Who are working to prevent early marriage?**

**P.** justice office, women association this are free service women representatives and women affairs are governmental, there are also leg which are political members. These are the three who are working on preventing early marriage.

**I, What are they doing?**

**P.** they give education and teachers study who are students stopped education and why they stopped. And after the study the justice office will give education on the issue like if they are doing the marriage they will be accused legally and this will be given together with the associations.

**I, What are the community related barriers related early marriages as you told me earlier that there are trials still to do early marriage?**

**P.** the policy as I have told you has no problem and the community is also educating their children as there are many girls in school and when we see in competitive they are very competitive as you are observing in your University; this is to mean we are doing very well in terms of education as previously for female marriage was the first choice by the family members so when we see now there is huge change. But still there are problems like girls have boyfriends without permission to their family and this is not acceptable in our community and then the community says if I did not marry at lower age she should have boyfriend and become outsider or unable to control; and she will bring herself and this thatches my morale; so I should give her my preference. So we do not eradicate at all on the old thinking’s even though there are also changes.

**I, What about the effect of religion on early marriage like Diakon has to get virgin girl?**

**P.** I have not considered as a problem but as general knowledge if he is “diakon” he marries virgin girl and to get such girl he will prefer the lower age girls as he could not believe the girls with age of above 18 years old would have the virginity. As a problem yes it has an effect but their number is very small.

**I. what do you think should be the gap of birth interval? Why would increase the space between each birth improve maternal nutrition?**

P. It is not what I said but based on the scientific evidence and it is also give support as well; as it has its own advantage and disadvantage in saying above or lower than this. It should be four years gap of birth interval. But still four years old child is still very small; this simply our community awareness is so poor. But the child is not still mature as he do need to separate from his mother and he still loves to play with his mother but at least he could eat what he has got by himself. So I prefer the gap to be four years birth interval which is already put by the government.

**I, Why four years birth interval why not the women give birth consecutively?**

**P.** this is not because of hating having child but the mother also hates but she could give birth every nine months; the problem is the mother will suffer in giving care to all the children as they are so close; she will even confused to whom she would give care and finally reach at which the mother would not able to resist and may die. Give child first she should survive and to survive she should have stable pregnancy period unless this is not happening forget the economical implication she could not able to withstand her health related problems. On one hand she is lactating and on the other hand she is giving birth and loses blood; hence she could not survive. Imagine blood lost during one delivery at what time could be substituted so she could not able and the child is also affected as he could not get the actual treatment or care like lactation and food. So the child will be exposed to different diseases as he did not enough milk from his mother and he has not finished his love to his mother; these all together would affect the child. Finally the family will be affected including the husband even though he is not direct victim he is not free from the pressure as the family will not have any peace; there will be always disputes among husband and wife as we live peacefully when get our interest unless we will fight each other.

**I. Is this program promoted effectively in the community?**

**P.** yes it is promoted in the community and there are changes in the community as we do not observe very crowded family in the community. There are children born at the exact gap or at an interval as the women are becoming aware of the importance of giving birth at certain interval. But still there are problems on the gap as there are mothers still give birth at two years gap and you see very physically small children which may be related with nutrition of the child as he may not get the care. But still there are mothers who give birth at one or two year birth interval so still there is the problem related with poor awareness. And the mothers themselves who give birth after one year then feel sad when coming to the health service.

**I, Who are working on birth spacing?**

**P.** it is given at the community by the HEWs during the community education. They teach the mothers the importance of family planning and the effect of giving birth without spacing to the mother and to the child. The health professionals also give advice to the mother when they come to the health service to deliver to come back for contraceptive and its importance as may see period during lactation she is also advised what she must do.

**I. Is there any a problem or barriers to the implementation of birth spacing?**

P. previously yes there were problems related with religion by religious leaders and the community members as well that the children are given from GOD so it is not correct. But now this is changed; rather this now it is because of carelessness. If the influence of the religion was there we have been observing family members with 12 and above family size so this day mothers are better than they were the past times in terms of economy so they could have many children like 12 or 15 children per family but such thinking is not present or the religious related barriers are not a problem now.

**I. Can you think of any other opportunities to prevent early marriage and birth spacing?**

**P.** there is school based education but it is not like it was given previously as it was given using banners and posters. For example our children were telling us when they come back from school like the poster of a mother with many children at her back, in front and carry woods at her head; so such type of teaching should be given to the students at school as the mothers are already getting from the HEWs. We need to teach the students about the problem related with having two or three children and six or seven children as the students or the children are victims themselves because it easy to bay shoes to three or four but difficult to bay for seven or eight children; so the children should be learned like this at school. This could be then the issue of the family as the children will tell to their family and there is no now families who have no student. Starting from grade one they should be learnt about how happy family is created and how the children will have bright brain and active in schooling, clothing and over all living condition to be better and others should be given.

**Section 6**

**I. Do you feel it is necessary to work with other sectors to address maternal and adolescent nutrition? Why?**

**P.** it is present and we are working mainly with the health sector on nutrition and agriculture as I have already told you they do have food security program; so I believe that it should be continued strongly. There is a need of help from NGO to provide help to the poor; the awareness creation is mainly worked by us or the team. Here the problem is it is not consistent as it is started and then left and the meeting is also similar so if we work consistently with continued meeting there will be a change.

**I, Tell me the role of your institution in working or complementing the role of the other sectors to improve maternal and adolescent nutrition like how do you work with health and others?**

**P.** our role will be mobilizing the community and then the other sectors will give their education based on their role; like health sector will teach the community about health and the agriculture will teach about productivity and how to get their food from this products.

**I. How do you think should be worked or what kind of change should be done the way the stakeholders work together? Are you for example working together now?**

**P.** yes we are working with health and agriculture but it is not consistent; there are interruptions. For example health if they want to teach the community about health they cannot work without the women associations and women affaires which are found in the community and working voluntarily; health sector the HWE; agriculture has also has representative these are working together. As there could not be change by working separately as the health cannot bring change by working alone and the women affaires similarly cannot bring change by working alone. But we need to work closely and strongly.

**I. What type of challenges or resistance have you experienced when you are doing collaboratively?**

**P.** there is no nay other barrier but the problem is only related to their sector as they give priority to their sector first and when you have an appointment you may not get the other sector because he may have another plane from his sector; so this is the problem which prevents the program to be very effective or strong.

**I. To what extent does your institution participate in the multi-sectoral nutrition coordination body at this level?**

**P.** when you take our association it is based on the interest of the society; it is free association and if take the women affairs it is governmental organization but it has no any it own activity; their main job is mobilization and collaboration with other sectors. For example if we take women affairs they do not have its own activity but work collaboratively with health sector and agriculture.

**I. is it very effective in working together as collaboration?**

**P.** yes it is good as I said before.

**I. what needs to be done to improve the capacity of these bodies?**

**P.** as I said before we have to work closely. It should have its own time and check list and should go up to the lower level. The check list must have what we have achieved, how are we performing, what problems we have and how we should solve those problems. There should be also accountability and the person who is working there should also be evaluated and we should use the measurements like the nutritional status of the child and mother; and in areas where it is not working well should be identified and work intensively; so for me we need to work like this.

**Section 7**

**I. Any additional comments or suggestions that we have discussed?**

**P.** What I should say finally which is related to this topic is that our development is incredible and never seen in other countries. But still we do have stunting or “Mknchar” and the child is affected by having poor thinking, physically and he would have poor brain like we were at our childhood. And when we see our economy and level of the technology they do not have cry such type of children; so it needs brilliant or active mind and to get this even if very few we need to feed our children balanced diet as the countries development depends on these children. There are works which are started and stopped type but this should be worked consistently similar to the mother issue in which we make it our issue daily. So our nutrition should be similar to our development and we should continuously bring change in awareness of the different cities. But finally the others like early marriage is doing well and our policy is good and what is left is to work very strongly to be successful. All our planes need to be worked closely and collaboratively to be effective. This is all what I have.

**Summery points**

**Section one:**

- Both pregnant and lactating mothers are affected by malnutrition and most notably pregnant mothers are the most affected.

**Section two**

- Our main priorities related to improving maternal nutrition is awareness creation and mobilization together with health and agriculture.

**Section three**

- Our main intervention regarding maternal nutrition is giving support to the health service and agricultural services to the community by mobilizing the community..

**Section four**

- Our main barrier related with to implementation of the nutrition interventions is lack of awareness of husband and the community.

**Section five**

- We have worked on preventing early marriage and increasing space of birth interval but girls should start giving birth after 25 years old.

**Section six**

- We are working with health and agriculture as our institution as no any other activities but supporting and implementing the other sector activities.
- **Finally I have finished my questions and I would like to thank for your time, patience and answering all the questions. Thank you very much!!! Thank you!!!**
