# Supplementary material for: Barriers to utilize nutrition interventions among lactating women in rural communities of Tigray, northern Ethiopia: An exploratory study
Source: PLoS One. 2021 Apr 30;16(4):e0250696. doi: 10.1371/journal.pone.0250696 (PMC8087028; doi:10.1371/journal.pone.0250696)
Supplement: S2 File — (ZIP) [file pone.0250696.s002.zip › S2_File.Doc/Woreda level and above key informants/123-IDI_Woreda health office_Medebay Zana woreda.docx]

**In-depth Interview with V/Head of Health Office in Medebay Zana Woreda**

Zone: Northwest

Woreda: Medebay Zana Kebelle: Selekleka

Name of Key Informant: Berhane W/aregay

Institution of key Informant: Woreda Health Office

Name of interviewer: Measho G/slassie

Date of interview: 20/11/2017

Start time; 10:45 AM End time; 12:18AM

**Interviewee Professional Information**

Age: 29

Gender: Male

Highest level of completed education: Bachelor degree

Current job/position: WHO, vice head

Work experience in the current position: 2 years

**I**: Interviewer **P**: Participant

**Section 1: Common maternal nutrition problems in this community**

I: Thank you once again for your willingness to participate in this study. What do you think are the common nutritional problems of the women and adolescent girls in this community?

P: The fundamental problem in this community is not about shortage of food but it is related with utilization. When we take the mothers and children, there is nutritional screening and there are also findings. There are mothers and children who have moderate malnutrition, but its basic cause is related with the problems of proper utilization of the available foods at their home.

I: Good. You have told me that the moderate malnutrition is observed in the children. Is it common in the pregnant and lactating women?

P: Similarly, it is observed in the pregnant and lactating women. It is not something that we can judge by our naked eyes. However, there are some malnourished women identified during our monthly nutritional screening. They look like normal but when we measure them they are malnourished. Anyhow, there are some women found to have the moderate or hidden malnutrition during our nutritional screening which is supported by evidence. The region is following it as its priority and the health centers; especially it is a monthly duty of the health extension workers and the women development army.

I: How do you see the occurrence of severe malnutrition in the pregnant and lactating women of this community?

P: It is also similar with what we have been talking. There are changes when we compare it with the past. Unlike the past, there are changes in their level of understanding, especially after strengthening the women development army and they are also getting sufficient education. As much as possible the pregnant women have been getting antenatal care visits at least four times with sufficient counseling based on the principle of 1000 days (starting from pregnancy up to 2 years after pregnancy). Similarly, they are counseled to follow their nutritional status after delivery. There is change from where it is because they are receiving sufficient education. However, it is impossible to say that the problem is completely solved.

I: Good. How do you see the nutrition problems in the adolescent girls?

P: Nutritional screening is mainly done on the pregnant and lactating women, and the children, but there are also activities done on the adolescents. For example, if we take our health facilities, we have separate diagnosis room for adolescents and counseling service is also given to them in line with this services. But the main activities are done in the schools. First, health education is given to maintain their menstrual hygiene, and there is also nutritional education concomitant with the menstrual hygiene services. Awareness was the main problem, but now at lot of things has been done in collaboration with our stakeholders like schools and social affairs.

I: Are there adolescent girls who have moderate or severe malnutrition in this community?

P: We have not done anything apart from the health education because as I have told you before, the nutritional screening activity (MUAC measurement) is done on the pregnant and lactating women and of course in the under five children. There is a situation where follow up is conducted if such problems are happened; however, there is no regular activity pertaining to the adolescents.

I: why do you think adolescent nutrition is not given special priority like the pregnant and lactating women?

P: In terms of health education, we have almost equivalent services for the adolescents as much as we give for the pregnant and lactating women, especially in collaboration with schools through integrating with the clubs and providing materials to improve their awareness. But, we don’t screen the adolescent girls monthly using MUAC through going to their schools like what we have been doing for the pregnant and lactating women. We don’t classify the nutritional status of the adolescents through measuring their MUAC.

I: Good. How do you see the prevalence of micronutrient deficiencies like anemia and night blindness in the pregnant and lactating women of this community?

P: As much as possible we have been trying to diagnose them as early as possible. Out of our seven health centers only one has laboratory service while the rest are treating clinically. The laboratory problem of this woreda is well known by the region and it cannot be solved at woreda level. Despite we have only one laboratory service, of course we have now allocated laboratory for one additional health center, one comprehensive laboratory services including anemia test are done for all pregnant women. There are problems identified during the laboratory examination. We have been also providing early treatments for those who require it. Hence, despite we have the accessibility problem; there is a situation where they can get the laboratory diagnosis once in their pregnancy and solutions accordingly in the woreda.

I: Is goiter common in the women of this community?

P: Yes, it is observed in this community. There are existing problems, but there is no new incidence of goiter. There are changes in iodized salt utilization, but in terms of percentage of iodized salt utilization, we cannot say there is increment. As much as possible, we are collaborating with the associations in each kebeles to have iodized salt with them, and then to distribute it to the households through the health extension workers and women development army. We are also confirming the utilization of iodized salt in each household and as a result the disease is decreasing in the community.

I: Do you think the iodized salt utilization is well promoted in the community?

P: There is supervision service from the region every six months. During their supervision they start from the health centers, health extension workers, women development army up to the households. Iodized salt utilization is one main part of their supervision. It is also our main priority as a woreda up to the women development army. Giving priority is not enough; the fundamental thing is assuring the accessibility of iodized salt in each community and improving the community awareness about its benefits if they used it and the consequences of not using it. In addition, not only the availability of the iodized salt, but also we have done many things for improving the proper usage of the iodized salt.

I: Good. Do the diets related noncommunicable diseases like hypertension, diabetes mellitus and others are commonly observed in this community?

P: Yes, they are observed, especially diabetes mellitus is commonly observed in the towns (Ezana and Selekleka). There are many people who have been under diabetes mellitus treatment.

I: Why do you think diabetes mellitus is commonly observed in this community in relation with the community feeding practices?

P: There are many diet related influences that we have not solved yet. Despite there are many changes, there are challenges in the accessibility of foods, which is our assignment for the next. Diabetes mellitus, cancer or hypertension could be happened due of improper feeding, because, there are people who repeatedly use fatty foods, repetitive sugar intake and inconsistent salt utilization. Still the community has low awareness about how to prepare balanced diet and what types of foods have what benefit and consequences.

I: What are the activities being undertaken to prevent the diets related non-communicative disease?

P: When we say the nutritional problem, the community considers it as under nutrition. There is an awareness gap on the problems associated with over nutrition. Most of the problems were associated with under nutrition and lack of proper food intake. There is widespread lack of awareness on the diet related non-communicable diseases, which will be our assignment.

I: As you have mentioning, is overweight common in this community?

P: Yes, there is to some extent. The problems is observed in two poles (over weight and underweight), but the overweight is not a major problem of this community. However, there are some signs of it.

I: Good. How do you see the availability of stunting and underweight in the adolescent girls and the women of this community?

P: Since we have been screening the underweight in every time, the OTP services are available up to the health post level. Thus, we have been following it and giving the OTP services and as a result many people have recovered from. To prevent the reversion of the problem, counseling is being given to them. With regards to stunting, to some extent it is observed. There are some adolescents who have unbalanced height and their age. This is irreversible problem, but it is not common in most of them. However, we are left more to do to on its assessment.

I: You have told me that underweight and stunting problems are sometimes observed in this community. So, could it be related with food shortage or unable to properly use the available foods?

P: The basic problem is unable to use the available foods. If you see our woreda, it is not as such affected by food shortage. As you can see, it is green and it is not also economically affected. In addition, there is no water shortage compared with other areas. In fact we have some lowland areas which can be affected by food shortage, especially they have unsolved liver disease in the areas despite many researches have been conducted. The disease is progressing and they are not productive. As a result, they will be economically poor and this could be a cause for their underweight and stunting. Overall, majority of the problem is related with lack of proper utilization of the available foods. For example, they take their eggs to the market. There is also irrigation to some extent, but they take the vegetables to the market. They see it on the sides of the money but they don’t see the benefits of feeding for themselves.

I: How do you describe the education given to prevent malnutrition?

P: The rural people take their balanced foods to the market to get sugar and coffee or sugar and salt or others which do not bring any change on its health or nutrition. For example, the sell honey to get something. Even though we have done many things and we have also seen some changes, but we have to strengthen what we have started to bring fundamental change. There are many collaborating stakeholders on nutrition, for example, Tigray Relief Society is widely working nutrition. Agricultural Growth Program (AGP) organizes and supports different association. The health sectors is also actively working on nutrition, especially we are working in the 1000 days for preventing underweight and stunting. Different training has been given for the agricultural workers, health extension workers and other community leaders and religious leaders mainly within these two years. World vision and Tigray Relief Society have been working on the community nutrition for the last many years. Nutritional services are also part of the main mission of the Woreda Health Office. Therefore, the health office has been aggressively working on it by taking the stakeholders as main resources and supplementing its activities.

I: You have told me that the lowland communities are affected by liver disease. Do you think the liver disease is associated with their nutrition?

P: It needs further research, in fact there are some attempts and there are also some conclusions about it. The researchers found that there is a toxic substance inside the venous of the liver that causes the disease. There are some planned activities for the forthcoming. The liver disease has very high influence on nutrition. Those people who have liver disease cannot feed whatever they get; instead they eat only selective foods. They don’t eat the relevant balanced foods for their body like the normal persons; rather they only eat foods that cannot aggravate their disease. There is also a situation where they don’t get the other alternatives. So, there is a situation where they will be at risk for malnutrition.

I: You told me that those women who have moderate malnutrition are given OTP.

P: Yes, it is given in every health centers and health post.

I: Is OTP an abbreviation?

P: Oh! It represents Outpatient Therapy.

I: Thank you for the clarification

P: It is a packed balanced diet mainly given for the underweight children for two months or eight weeks. It is a nutritional treatment.

I: Is it Plumpy’Nut or else?

P: it is Plumpy’Nut and there are also other complementary medications. If there are people who cannot take this treatment, they will be referred to the primary hospital for inpatient treatment. In the inpatient, they will be given in the form of milk, F100, F75 and others. Therefore, we have not faced anyone who died from the screened underweight people so far. There is a situation where they can improve after treatment. The treatment services are available in their nearby health centers and health posts. The OTP and TF services are available in the health centers. So, there is good service provision and outcome.

I: Good. Are there pregnant or lactating women who take Plumpy’Nut in this woreda?

P: We don’t have any women who have been taking Plumpy’Nut in this woreda. It is only given for the malnourished under five children. It has its own criteria for starting the service. The women are given nutritional counseling. The main source of the problem is unable to use the available foods in home. So, she will be advised how to improve her nutrition by preparing balanced diet from the available foods.

I: How do you describe the food security of this woreda?

P: As I have told you the main problem is related with unable to use the available foods properly. However, the previously mentioned lowland areas complemented with the disease that we have described before have food shortage. They survive by eating only the dry enjera with salt; even some of them may not get this. Despite we have many things to do for improving the community awareness; we cannot conclude that there is no food shortage. Especially the food shortage is observed in the lowlands, but the highlands are relatively better.

I: where are the lowlands which have food insecurity?

P: Maylemon, some parts of Toklo, but the worst is found in Kuwudto and then there are also other places like tsadalaka, shame and adinigisti.

I: Do you think the women and adolescent girls are specifically at risk for malnutrition?

P: Scientifically the children are more affected for malnutrition and we are also observing it practically. The women are also at risk for malnutrition due to different internal and external influences. The children cannot decide by themselves in addition to their immunity and it is also similar with the women. The environment by itself also contributes for it. The women could have nutritional influences associated with their pregnancy and delivery similarly the adolescent girls could have the influence associated with their menstrual cycle. So, they are at risk in different forms.

**Section 2: Nutrition priorities in the woreda**

I: Good. Let us come to your office, what priorities do you have for improving the maternal and adolescent health?

P: Mainly there are people conferences especially in the nutritionally affected areas. Giving health education at health facility or by the health extension workers is not sufficient. In the conference, the administrative bodies, religious leaders, influential people in the community, especially the pregnant and lactating women are included. During the conference, you give them the topic to provide the solution by themselves with specific deadline, and evaluating on the progressive changes. There are also good woreda based services, especially on the priority areas like health education. In terms of accessibility, the kuberto, mylemon and tselemti are bordered with Tekeze. So, some of the communities have lack of access for information due to its long distance from the center.

I: Good. You have informed me about the priority health services of your woreda; what prioritized activities do you have for improving the nutrition of women and adolescents?

P: Mainly there is a women union which includes women association, women affairs and women league. The main priority of the women union and of course the assignment of the health office is strengthening the networking (‘wudabe) of women. The women development army is part of its solution. Currently, 25 to 30 women are grouped in to one in each Kebelle. They have been discussing in their respective groups about their health nothing more and they have been providing solution by themselves. They don’t have any reason to wait for the decision of the Kebelle or woreda administrators, even their husbands. They share experiences from each other and they don’t have to beg money from her husband, instead she can engage in poultry agriculture to fulfill her demands. They are getting out from their dependency. Equal numbers of girls are also integrated with the males during distribution of land for irrigation. So, they are equally participating in irrigation and they are also educating their children. In the past, they were obliged for early marriage and as a result there were susceptible for malnutrition, but now awareness about prevention of early marriage has been created. So, great work has been done on networking and grouping the women. There is a situation where they are solving their problems by themselves.

I: Good. How do you evaluate the attention given for the women and adolescent girls?

P: Big attention is given for the women in this community. The government policy and direction by itself also gives due emphasis for them. Similarly, the woredas are also giving more attention for them.

**Section 3: Nutrition interventions that improve adolescent and maternal health**

I: Good. You have described about the general priorities and women networking activities undertaken for improving the women’s’ health and nutrition. What specific kinds of nutrition interventions are in place to improve the nutrition of women in this community?

P: Mainly at woreda level, there are efforts to establish alternative water sources for every household. If they have the alternative water sources, at least they will have the home gardens. If you have the water source, you will not have to wait for other persons rather you can improve your nutrition by cultivating your own gardens in a piece of land behind your house. This doesn’t require more energy and land to produce onions, vegetables and related things for your own personal consumption. So, we have been working to improve their nutrition in such a way.

I: Do you think the pregnant and lactating women are implementing the home gardening activities as per the education given to them?

P: I don’t think they are efficiently utilizing the available water for home gardening. Despite there are gaps, when you compare it with the past, there is good progress. At least in the past, with the exception of salt and oil, onion was not used in the rural community, but now they are preparing their food using onion. Green colored vegetables were not commonly used during the winter, but the summer season. However, currently they are preparing their foods from tomato and other vegetable from their home gardens during the winter. They are not waiting a season for producing their vegetables though it depends on their efforts. Therefore, there is change in their awareness and they are also practically implementing it.

I: How do you describe the counseling given on the antenatal and postnatal care follow up in this community?

P: It is implemented in this community with special attention. There is a situation where a pregnant woman is diagnosed before her third month’s pregnancy. We have already prepared the pregnancy format and the health extension worker diagnoses the pregnancy based on the prepared formant. Similar to the drug balance, we have reporting system in every month. For example, if 20 women delivered in one Kebelle, we also identify the number of pregnant women diagnosed within the month. Then we estimate our balance through identifying our entries and exits. If they are diagnosed in their early pregnancy, it will help them to get the services timely. If you diagnose the pregnant women, it will be very easy to know who delivered where. You don’t need to search for the mother to bring her to the health facility for delivery; you can easily find her in the document. We classify the pregnant women in three levels; red level (pregnant women whose delivery date is arrived), Yellow level (pregnant women on antenatal care and they also have special follow up for other services) and green level (timely diagnosed pregnant women who required at least four ANC visits). Therefore, every mother is registered starting from her early pregnancy and she will have also at least four ANC visits and will be advised to deliver in the health facility. After delivery, the follow up continued in collaboration with all concerned bodies to vaccinate the child not by searching home to home but through your registration document. Second, we have pregnant and lactating women day (“maalti birhan tinusatin harasatin”), aimed for encouraging the pregnant women not to stop her ANC, deliver in health facility and to follow her postnatal care after delivery. In addition, if the pregnant women implemented what she has been advised about nutrition, her family members may have a concern on why she is eating every testy food for herself. Therefore, the pregnant and lactating women, husband, her mother or mother of her husband are included in the pregnant and lactating women day to improve their awareness.

I: What is the schedule for the pregnant and lactating women day?

P: It is conducted once a month in every Kebeles. Mainly, it is conducted in wholly days. For example, we have 20 kebeles and some of them conduct is on the 7^th^, 12^th^, 19^th^, 21^st^, and 27^th^ days, they don’t conduct it on the 29^th^ day because there is another meeting. So, their discussion is conducted in every wholly days, even it is conducted in a very hot manner. For example, one mother promises to bring roasted cereals, there are also others who come with sugar, coffee, wood. Hence, they discuss while enjoying their coffee and tea, and there are also staff from health extension workers and staff from the health center and sometimes there is a situation where staff from the woreda joins in their discussion.

I: How do you describe the counseling given for the pregnant and lactating women to eat extra meal and diversified foods either during pregnant and lactating women day or other occasions?

P: We are about the women centered activities in the woreda. When we come to the details, the women are counseled not only to eat balanced diet but also how many times to eat per day during her pregnancy and lactation period. Awareness is also given for whom she is eating. They are being told that the food she took is not only for the mother but it is also shared for her baby. They have been also discussing about the nutritional alternatives, for example, they discuss about what they have in their houses. In the first place, when you advise them about balanced diet, they tell you that we don’t need advice if we get this kind of foods. When you hear their opinion after advising the potential alternatives, they will tell that i don’t have it. However, when you ask them what they have in their home, they will tell you more than the alternatives you proposed for them. They have the most expensive and important things in their hand, but they swap it to the cheaper things in the outside. So, we have been discussing with them to reach in common understanding. When we come to the children, they weight is regularly measured and their status is reported during pregnant and lactating women day. Counseling is also given for those children who have malnutrition. There is also demonstration session on how to prepare the balanced diet during the meeting. The kebeles were given the relevant equipment for the demonstration. Education is also given for the adolescents in the schools.

I: How do you describe the implementation of productive safety net program in this community?

P: Yes, there is safety net program and it is aimed to support those who have food shortage. To avoid dependency and to improve their work culture, the support is not freely provided rather they have to work in order to get it and they are also advised to use it. For example, there were some people who were selling the oils and pea for other people. But, the purpose of the safety net support is to improve their food shortage but not for selling. They are also advised that the support is not sustainable. If they are supported by the safety net food, they can buy a poultry or goat through their efforts. After they secured their food, they will be graduated from the safety net program.

I: For how long do they stay in the safety net program before their graduation?

P: I don’t have full information about that, but I think it is every five years. But, if the household secured his food before five years, there is a situation where the household is graduated from the safety net program. Graduation means self-change. So, they will be self-sufficient after graduation. However, there are many activities done to break the intention of the households to become dependent on it.

I: How do you describe the counseling given for the pregnant and lactating women for improving their water, hygiene and sanitation?

P: There are some pipes waters under construction, but many people are still using from the river water. Mainly, we have been working to create open defecation free community. In the communities where pipe water is construction; initial assessment is conducted to know what it looks like and then training is given to the community. After triggering, post triggering will be given and evaluation is also conducted, and 8 people are also selected as water committee. So, it is relatively better in this regard because the water committees are also responsibly working on it. Despite it is very difficult to say there is big change in hygiene and sanitation because if there is big change, the Kebelle has to declare open defecation free. But, there are changes when we see our data. In the past, when the community was told to construct toilet, they just dig very small hole and it will be covered during the summer season. *In terms of number, it will be reported as 90% or more toilets are constructed, but you will not get them next year. The number will exist because it is already reported, but whether the reported number is on the ground or not is another issue.* We have established two big associations in collaboration with WASH to alleviate the problems mentioned before. The main role of the associations are constructing slab and supplying to the community. The community collects the money and they provide them the slab. There are two in Zana sub woreda and there is also one here. They are also constructing in the kebeles, if there are thirty and above households are willing to willing to collect the money. The sustainability of the toilets has increased after the slab is in place. Even if the toilet if full, there is an opportunity to take the slab for another new toilet. In the past, most of the toilets were constructed and demolished within very short period of time and it was only dependent on the numbers or data, but not practical.

I: why do you think the community is not concerned about the construction of their toilets similar with what they give for constructing their house?

P: The main problem is lack of awareness about its associated risks. For example, if we take our last year’s scenario, around 73 acute watery diarrhea cases were reported in this woreda though most of were come from the western zone. The 73 cases were young and productive people. Among the 73 cases, 69 were come from the western whereas the four were from our woreda. The main cause of the problem is because they don’t have toilet and they are defecating outside their homes like animals. Therefore, the foods and water sources will be contaminated. So, the there is a gap in understanding its implication on personal health. We have widely discussed with them. *For example, some of them asked about which one is easily between digging burial or toilet. The toilet in the rural community is almost similar with the size of the burial. During the funeral everybody collaborates like an army and a lot of energy is wasted, but they don’t give similar emphasis for digging the toilets.* Therefore, there is good movement to work on permanent toilets and it may solve the exiting problems. Mainly, it is very difficult to change about the community perception about its severity and it needs repetitive discussions.

I: How do you describe the counseling given for the pregnant and lactating women to use insecticides treated bed nets and their utilization?

P: Priority is given for the pregnant, lactating and the children during the ITN distribution. When priority is given to them, it is about favoring them rather it is because they have many influences and tiredness and if they also sick, they will be primarily at risk for many problems. When we see in terms of coverage, we have received 83,000 ITNs this year and it was distributed to the households timely. We haven’t only distributed it but we have showed them on how to stretch and use it in their beds. We have also discussed on who should get it first. We give them by opening its covers to prevent the misuse of the ITN (like selling, taking to other places). When we visit the households, some of them use it as fence for seedlings, house cover and some of them also use it for carrying straws. We know it how it is coming and it is used to save lives. Despite we working to improve its utilization, there are still some challenges in its implementation. We are working to achieve 100% utilization of the ITN.

I: what actions are taken for those who don’t properly utilized the ITN during your home to home assessment apart from health education?

P: There is no punishment; rather we discuss with them and we also give them health education. If you punish them, they may not show you during the day but they may not use it during the night. So, we should work of their understanding. We don’t have a specific rule to punish the persons, who misuse it, however, if we get someone using it for carrying straws, immediately the ITN will be taken away because it was not come for that purpose.

I: How do you describe the deworming service in this community?

P: Most of the time it is given for the children mainly from two to five years old in every six months. In the past, we were waiting for six months to provide the deworming service, but now it is given in monthly bases. When we say in monthly bases, it doesn’t mean that one child is getting in monthly bases. For example, out of the 100% children, only 17% of them will get the service in one month and the rest will get is the rest of the months accordingly. So, the children will get the service in every six months, but the service is given in every month. In the past they were coming only at once, but now they are classified in to different months based on their age. So, the deworming service is relatively better.

I: Is the deworming service given for the adolescent girls (10-19 years of age)?

P: It was given before two years, but it is stopped since last year.

I: Do you know why it is stopped?

P: It was regionally centralized service and it is implemented by regional program.

I: Do you mean that it is not implemented based on the woreda plan?

P: yes, even the drug supply for deworming is coming from the region.

I: How do you describe the availability and implementation of targeted supplementary feeding in this community?

P: In the past, the pregnant and lactating women who were screened to have moderate malnutrition were given Fafa like the OTP service for the children, but now the service is not available. However, after assessing their household status, there is a situation where they can be supported through the safety net program.

I: Is the vitamin A supplementation given for the pregnant and lactating women and the adolescent girls given in this community?

P: Yes, it is given to them.

I: Is the vitamin A supplementation given for the adolescent girls?

P: It is given only for the pregnant and lactating women, but it is not given for the adolescent girls in schools and other areas.

I: Do you think it is important for the in school and out school girls of this community?

P: It is not only important but also it is mandatory, but there is shortage of supply and it is coming based on the targeted priority. So, priority is given for the pregnant and lactating women.

I: How do you describe the implementation of youth friendly services in this community?

P: Mainly, it was started in one health facility which was supported to become a model for the other health facilities. The youth were also working in organized manner. The youth were coming to the health center in every three months for evaluation and to take their assignments. Similarly, the other health facilities are also working on it. We have also separate youth OPD in every health center.

I: Are there youth friendly services in the schools and community beyond the health facilities?

P: There is an opportunity to discuss about it in the community the by the women development army, one to five networking and health extension workers with the community. There are also discussion opportunities in the schools about their nutrition, especially as I have told you before; there are awareness creation discussions about their menstrual hygiene.

I: Do the health workers provide the health and nutrition education or the teachers?

P: Mainly there are health clubs. Sometimes in a situation where the students are gathered, the health workers give the nutrition and health education and also they provide professional feedback for their questions. Most of the time, we provide support and training for the clubs. But, whenever needed, they health workers went to the schools to follow, educate and to provide feedback.

I: Good. From the nutritional interventions implemented for improving the nutrition of women and adolescent girls, for example, youth friendly services, Vitamin A supplementation, deworming, counseling to use ITN, counseling to use iodized salt utilization, safety net program, nutrition sensitive agricultures like home gardening, counseling to follow antenatal care and counseling to take diversified foods. So, among these interventions, which of them do you think is successfully implemented in this woreda?

P: The successfully implemented intervention is related with home gardening. With regards to antenatal care follow up, in the past they were resistant to come to the health facility and even were bringing them to the health facility by searching in home to home bases, but now, they are coming by themselves as soon as they become pregnant. They attitude has been widely changed currently. In the past, the community was saying that it is not testy, but now it is widely utilized in the community. In terms of its proper utilization, we have a lot of assignments to do. There might be some people who don’t use iodized salt, but among the users, there are so many people who cook is together with the stew. Anyhow, there is little improvement in awareness of the community about iodized salt. Though it is important to give Vitamin A supplementation for the adolescent girls, we have not yet worked on it. We will see it together in the future if solution is given from the higher administration. There should be supply and in line with that there should be a reporting system about who works what. But the Vitamin A supplementation for the children (from 6 moths to five years old) is well implemented. With regards to deworming, years back it was given for the adolescents, but now the service is not available for them. I wish to continue the deworming service for the adolescents, but it is not something we could decide it rather it needs directions from the top. No body prevents us from giving the services but there is shortage of supply to do it.

I: Good. Which of the interventions that we have been discussing are not successfully implemented in this woreda?

P: All the interventions are successful, but there are some gaps in utilizing the services due to their lack of awareness. The main point is not about how much we tired, rather it is do the community accepted it. We have to mobile all implementers up to the women development army to reach the message to the community, implement it, and to create a community whom benefited from the intervention. Thus, despite there are many progresses, we cannot say everything is completely implemented. Even we have problems in iodized salt utilization; first we have to assess the availability of the iodized salt and we have to follow whether the supplied one is reached at the household or not and finally we have to follow whether the community is properly utilizing it or not. Even there is iodine tester. The health extension workers can take sample to test the availability of iodine in the salt. So, we will strengthen the implementation of iodized salt utilization in this community. The community is also widely using the ITN, but still we have implement more follow up activities. We are not implemented the deworming and vitamin A supplementation for the adolescent girls not due to lack of attention but lack of supply.

**Section 4: Implementation challenges and community factors affecting access to nutrition**

I: Good. What do you think are the main challenges or obstacles for implementing the interventions that we have been discussing?

P: One challenge is lack of the availability of Fafa services for the undernourished women identified during screening. We have been only linking the malnourished women with the safety net program. We have also shortage of deworming drugs supply.

I: We can see the challenges in three sides; one; from the individual beneficiary side, second; by the community side and third; by the interventions side. So, what challenges have you been facing from the individual beneficiaries side related with their educational level, awareness and others?

P: Nobody dislikes the benefits, but the problem is unable to understand the benefits. We are facing many challenges in this community. Even, we have discussed before about sanitation. It will take us more time and effort to change it. Therefore, the perception about *“if we don’t have toilet, we will loss life”* is not yet accepted. So, the toilets are not constructed and the people are dying associated with it.

I: From your statistical report observation, do you think the educated or uneducated people are more challenging you to implement the interventions?

P: There are two things here; there are uneducated people who implement it if you properly educated them. There are also other uneducated people who resist you. They may say ok while you advise him, but they don’t implement it. Mostly the uneducated people are challenging to change. It is also related with our failure to create awareness in a way they can understand it.

I: Ok, do you think there are challenges related with the community culture, attitude and traditions for implementing the interventions?

P: Currently, there are no challenges related with that, but there were problems in the past. For example, there were influences for ANC in the past, but now everybody including the priests and other religious leaders encourage her to go to the health facility and they are also implementing it. However, there were a lot of influences in the past for the pregnant women not to attend her ANC and they were coming for delivery after their delivery becomes more complicated as last alternative. But now, let alone at her delivery started, she is coming before her delivery to stay in the health facility.

I: Are there any challenges related with accessibility like transportation, geographical setting, cost or others?

P: Yes, we have two kebeles which doesn’t have transport access during summer season. The one Kebelle has health center. If there is no transport and if ambulance is unable to reach there, it has its own influence. Second, the transport shortage has its own influence to introduce supplies like iodine and others.

I: Are there any challenges related with the interventions themselves, related with its quality, convenience, professional commitment like CRC and others?

P: There is no overt problem, but there are some signs. Especially if you take the health centers related with the cards, if the customer told you that his card is lost, you shouldn’t tell him to bring his card because he will not get it or he will not come back; unable to serve the customers by saying time is over. So, there are some problems related with this. When we see it generally, especially after the implementation of health insurance, the services has increased because once the community paid in the beginning, they will get the services for free in each visit. When the services become almost double from the previous one, some ethical problems are observed, but it is not as such magnificent problem.

I: You have raised good point about insurance. How do you describe the role of health insurance for improving maternal health and nutrition?

P: There were times where the woman wants to get treatment service, but she fail to come due to shortage of money or she hides her illness due to her shortage of money or due to fear of incurring too much money. But, now there is no anything like that. She just come with her cards and gets the treatment services. Therefore, it has great role for maintaining her health. When it comes for its payment, it is a concern of the whole family, but later it doesn’t have any influence during the service utilization.

I: You have told me that there are some challenges for implementing the nutritional interventions, for example; lack of transport access, lack of community awareness and others related with the interventions. Thus, what actions did you take to solve the problems?

P: Even though there is transport shortage, there is no interruption of service provision. The inaccessible areas for transport are very few and the required services like drugs, vaccines, vitamin A, deworming and other supplies are reached there either by camel, messenger, or by the commitment of the health workers. At most there could be extra financial cost and people may be tiered because the manpower is used instead of car to reach there. There could be delay in providing the services in the inaccessible areas. For example, we have delayed to provide the ITN in one Kebelle due to lack of transport service. Therefore, we cannot say they are being served timely.

**Section 5: Multi-sectoral collaboration to improve maternal nutrition**

I: As you have described before, your health office is working together with different NGOs’ for improving the health and nutrition of mothers. Do you think it is important to work with different sectors for improving maternal nutrition and health?

P: This is correct. As I have told you before, they are working very well on this issue. World Vision and Pathfinder are working primarily on the maternal health. The Tigray Relief Society is also working on it. They have been measuring the changes in every time.

I: In addition to working with the NGO’s, how do you describe the collaboration of your organization with different sectors like education, water, education and others?

P: We are connected with them by the established stream committee. The stream committee includes four main components, health, agriculture, education and good governance. They have a scheduled meeting in every two weeks. They also have a joint plan. Therefore, they have been working on nutrition. The health sector presents about nutrition activities, the agriculture also presents about green home gardens and irrigations, good governance helps to assure fair distribution and beneficence from the resources and it also helps to prevent inferiority of women, the education helps to eliminate illiteracy. This stream committee is available at the bottom as it is. They evaluate their performances collaboratively. Here we have the education, health, agriculture, three of the women union. The women union is standing for assuring the benefits of the women. Therefore, we have good collaboration with the sectors. For instance, there is adult learning in every kebeles. So, the health extension worker presents about healthcare activities, the education worker comes with what they have worked on adult reading and writing. The sum of all activities is for improving nutrition of the community. Above all, since nutrition has its own contribution in development, the woreda is working on it and similarly different activities are being undertaken at the bottom.

I: When we see the multi-sectoral collaboration in other woredas, similar to this woreda they have steering committee and platform at woreda level, but the sectors work independently at Kebelle level and they don’t collaborate for improving the maternal nutrition. So, is there joint plan and implementation at the lower level like what is available at woreda level?

P: If you ask me about the steering committee at Kebelle level; there is health extension worker, three of the women unions (women league, women association and women affairs) and there is also the part. More the activities are inclined towards the health sectors. The women in their networking like the women development army is more working on health. There are differences from Kebelle to Kebelle, because in some kebeles there are very strong implementing committees and to that extent there are also independent works. But, when we see the average, the woreda level steering committee is functional and it is implemented up the lower level. Currently the steering committee has taken training and they have evaluated what they have done. Therefore, generally the steering committee is very strong.

I: Ok, for example, do the assigned Kebelle level agriculture, water and other sectors work together with the health extension workers about maternal nutrition?

P: They don’t work only for nutrition; even the steering committee at woreda level assesses many activities. But, nutrition is one part of their assessment. They assess about sanitation, early marriage, antenatal care follow up, institutional delivery and others. Hence, they don’t specifically start and end their discussion with nutrition.

I: Are there any challenges or resistances while collaborating and working with different sectors?

P: Regularly, the steering committee have meeting in every two weeks. But, all of them do not come to the meeting. There is a situation where only few members may come and undertake the meeting. There are also problems in converting the evaluated activities in to results. For example, our meeting and evaluation is to bring result, it is not for the sake of meeting. However, the results vary from Kebelle to Kebelle. It is successful in some of the kebeles, whereas as it is weak in other kebeles.

I: What opportunities do you have to implement the multi-sectoral collaboration?

P: It only needs attention to implement it. If we take the agriculture, every one including the administrators are concerned on fertilizer distribution for improving productivity. Similarly, the health issues are given attention from the woreda down to the bottom. So, the main issue is about giving attention for it.

**Section 6: Other interventions that influence adolescent and maternal nutrition and health outcomes**

I: What activities are being undertaken in this woreda for encouraging delayed first birth (after 18 years of age) or for preventing under age childbirth?

P: Above all, many activities are being undertaken to increase their school enrollment starting from ‘O’ class and then to enroll to grade one at their 7^th^ age. So, they are working aggressively in education. There is women affairs representative in each school to assess and identify the adolescent girls who are a candidate for early marriage. For example, if we take last year’s report, 84 girls were proposed for early marriage. Among these girls, the early marriage of 80 girls was successfully canceled, but the remaining four was married by different means. I don’t know the decision but the cases were under law investigation. Therefore, you can see how big attention is given for prevention of early marriage and we have been also discussing on how harmful practice early marriage is. Above all, canceling the early marriage of 80 children to continue their education indicates how much attention is given for it.

I: Good. As you have described it, many things have been done to prevent early marriage. However, for example, still the community has proposed more than 80 girls for early marriage in this woreda. So, what opportunities are there in the community (including religions) for preventing early marriage?

P: As you are saying, the main thing is on its prevention. Those 80 girls should not have been proposed for that. Even after cancelation of the marriage, it has big social influence on the both parents and the candidates. When it comes to prevention of early marriage, we have still many things to do. From the very beginning, there should not be proposition of early marriage. This indicates there are still cultural influences for early marriage. For example, there were some women development army and women affairs that proposed their girls for early marriage. There was a situation where the judges undertaken the verdict of those who implemented early marriage in school compound to create awareness in the students, but you know the level of respect the judges have. You can understand the commitment of the woreda for prevention of early marriage.

I: What strategies, activities and policies do the woreda has for childbirth spacing?

P: The main strategy for birth spacing is providing the alternatives. In the past, as you know, the community had not good attitude towards contraceptive method utilization in relation with their religion and other influences. But now, these kinds of problems are solved. It is difficult to say that the husband and other influences are completely eliminated, but there is good improvement. Second, as I have told you, we have been creating awareness in different meetings and stages about its benefits and the disadvantages of not using it. If they delivered birth after birth, there are social, physical, and mental problems could happen on the mother and her child. In the past, the contraceptive methods were prescribed for the mother, but now all information about the alternative methods is provided. For example, the mother may come to take Depo-Provera for three months, but she will be informed about the availability other alternatives like Implanol, IUCD and if she don’t like to give birth at all, the possibility of undertaking tuba ligation. In most of the health centers, the alternatives are displayed by big flip charts. The information related with all methods is given, but she can decide at any time she wants. So, we are good in terms of contraceptive utilization. In the last the long acting contraceptive use was 20%, but now we are working to make it 50% by this year. In terms of advantage, the Depo-Provera is 150mg and is taken every three months, but the implant is 68mg and is taken only once for three months and even if she wants to become pregnant she can take out it simply without problem. Thus, there is good awareness about it and the alternatives are available in every health facility. At regional level, we don’t have anyone up to the health extension workers who didn’t take the training about it. Therefore, it is good in terms of accessibility of methods and providers. In addition, there is no any health center which don’t have at least three midwifes. We have 22 health posts and all of them are providing implanol. So, the alternatives are available, the education is also given and the beneficiaries are improving.

I: Good. What opportunities are there in the community for promoting childbirth spacing and prevention of early marriage?

P: The main thing is learning each other. They can learn from their neighbors. For example, they can compare between their early married child and the other neighbor who is attending her education. They can see the benefits of continuing education. There was one mother died after they tried traditional practices, of course it is not related with the early marriage and with what you are saying. On the contrary, there was another mother who saved her life after she came to the health facility due to very strong effort of a health worker despite her family was very resistant. Conference was organized to create awareness and to understand the situation from each other.

I: Was the death associated with early marriage?

P: Yes, the mother was early married. She was early married, there is lack of awareness and there are also environmental influences not to deliver in health facility. There are also many women who have been suffering from fistula. But, we have been creating awareness about its courses. In addition, there are some women who were treated from fistula in Hamlin hospital engaged in awareness creation. These kinds of women are ambassadors and we enroll them to the women development army to create more awareness in the community. The women development are also arranging awareness creation forum by inviting the early married women to share their bad experiences.

I: What about for childbirth spacing?

P; we discuss about its consequences. Problems related with providing educational materials, cloths, food are discussed. They can assume how much they could be suffered to develop the children and how much the mother could be suffered to develop the children if she delivered birth after birth. So, mainly we focus on awareness creation.

**Additional remarks**

I: Thank you very much. As I have informed you in the very beginning, the objective of this study is identify the access and implementation barriers of nutrition interventions for pregnant, lactating and adolescent women. So, if you have any additional suggestions?

I: We should be concerned about not only the transfer of the messages but also we have to follow its implementation including its results in the ground. We will strengthen its implementation. What we have discussed is part of our mission and most of them are what we have been doing it, but there are also many things that should be improved especially on the adolescent girls. The adolescent girls are the future mothers and it will strongly work on them. Thank you for the time we had.

I: Thank you very much for scarifying your time to participate in this study.

**Summary**

Section one: Common maternal nutrition problems in the community

- The fundamental problem in this community is not about shortage of food but it is related with utilization
- There are some malnourished pregnant and lactating women identified during monthly nutritional screening
- There is no nutritional screening for the adolescent girls
- Food shortage is observed in the lowland areas of this community
- Liver disease is also a common problem in the food insecure lowland communities

Section two: Interventions that improve adolescent nutrition

- This woreda has monthly pregnant and lactating women day for creating awareness about their nutrition and health
- There is no nutritional screening and interventions for the adolescent girls except the health education.
- TSF is not provided for the malnourished pregnant and lactating women

Section 4: Implementation challenges and community factors affecting access to nutrition

- Lack of community awareness, poor provider commitment and transport shortage are the main challenges of intervention implementation

Section 5: Multi-sectoral collaboration to improve maternal nutrition

- There is established steering committee, but its implementation is low.

Section 6: Other interventions that influence adolescent and maternal nutrition and health outcomes

- The community has birth spacing, but still there are some cultural barriers for the prevention of early marriage
