# Supplementary material for: Barriers to utilize nutrition interventions among lactating women in rural communities of Tigray, northern Ethiopia: An exploratory study
Source: PLoS One. 2021 Apr 30;16(4):e0250696. doi: 10.1371/journal.pone.0250696 (PMC8087028; doi:10.1371/journal.pone.0250696)
Supplement: S2 File — (ZIP) [file pone.0250696.s002.zip › S2_File.Doc/Woreda level and above key informants/124_Nutrition expert_Agriculture and Rural development Office_Medebay Zana woreda.docx]

**Operational Research on Adolescent and Maternal Nutrition in Northern Ethiopia**

## **In-depth interview responses of the nutrition expert**

**Introduction**

Thank you for your acceptance of the informed consent form and for taking the time to speak with me today. I have questions to ask you which were prepared in advance. The discussion will take 1-2 hours. If you have any questions before we begin please feel free to ask.

**Section A: Interview in details**

1. Zone: North Western
2. Woreda: Medebay Ezana
3. Kebele: Selkleka
4. Name of key informant: Kiros G/selassie
5. Institution of key informant: Agriculture and rural development office
6. Interviewer name: Mekonnen Haileselassie
7. Date of interview: 11/03/2010
8. Interview start time: 3:40 AM (local time)
9. Interview end time: 5:30 PM (local time)

**Section B: Socio-demographic and basic data of qualitative study participant**

| **Socio-demographic characteristic** | **KII** |
| --- | --- |
| Sex | Female |
| Age | 44 |
| Educational status | Bachler degree |
| Occupation/role in the community | Nutrition expert |
| Service year | 20 years |

**Note:**

I: interview

P: participant

**Section 1: Common maternal (pregnant women, lactating women and adolescent girls) nutrition problems in the community**

What do women do to stay healthy in this community/woreda?

P: We are doing a demonstration in each Tabia so as to be benefited all women from the training. We are striving to consume the pregnant women with diversified food in our community. Currently we are working in collaboration with health sector on overall conditions of nutrition; in the agriculture side, it is introduced the high quality protein maize (ሙቁር ዕልዎ) and we are working to distribute this agricultural product to each households. Since the maize is rich in carbohydrate and protein that could support the pregnant and lactating mothers in the improvement of their nutritional status. But there is also a problem on the feeding style of the community although it is introduced to all communities.

We advise mainly to the pregnant and lactating mothers to consume diversified food in the community; we demonstration site in each Tabia and we train the pregnant and lactating mothers how to plant sweet potato and we show practically how to prepare the food from sweet potato like porridge, bread and the like. But it is impossible to say that full awareness is created in the community. The weight which is given by the office is poor; for example there is a committee that was established in such a way that the woreda leader is the chairman, the head of health office secretary and the office of agriculture, education women affaire are members. But this committee is… (She showed frown face which was an indication of unhappiness).

The feeding habit of the community is poor and we didn’t do a lot in this area; the awareness creation among the community in the improvement of feeding diversified food is not well done; because the nutrition field expert as structure was lately introduced to the agriculture and rural development bureau which is only two years old. You know that let alone nutrition in our department, the old age agricultural activity that is the development of agricultural production and productivity requires large effort to develop well.

It has not produced great awareness from the high ups/government to the woreda then to Tabia and to the community levels about nutrition. As a starting we have introduced awareness to the community but we couldn’t say that nutrition is well popularized.

**I:** In your opinion, what are the common nutrition problems in the community for women? What about for adolescent girls?

P: It is difficult to evaluate whether there is nutrition problem or not in the community; there is no as such food problem in the community but the feeding system is poor; this is mainly due to lack of awareness. They produce all types of crops and vegetables like teff, millet, maize, sorghum, carrot, potato, spinach,

Most of the married women are not participating in the trainings and demonstration; now we are starting to invite both the husband and wife in the training. At least if the husband is participating he could insist the mother to prepare and consume the diversified foods.

There is resistance from the women like why we stove two, three dishes at a time; now some NGOs like Save the children introduces new technology that the women could prepare different food types at a time. For example they cook lentil, then add spinach then add potato then add oil, thus from the recommended six food groups this food preparation contains at least four food groups at a time.

I: What are the six food groups?

P: Cereals, pulses, vegetables, oil seeds, animal source foods; from these six foods groups if it is not possible to get utilized all the six food groups you should consume at least four. Some food types like the high quality protein maize contains protein, carbohydrate, oil; so our problem is less attention to the feeding style. We could produce swiss chard, eggs but they take to market to purchase sugar, coffee and other house expenses; so to solve this type of problem, it needs great effort that could convince the community. It may take time to make aware the community.

I: How do observe the prevalence of severe and moderate malnutrition?

P: Relative to other woredas, this community is food secure woreda; now nobody is get hungered; the problem is poor consumption habit of diversified foods among the community. There are very few individuals that don’t keep their food security who are supported by the safety net food programs. Save the children wants to select the poor pregnant and lactating mothers to support small ruminants and poultry production; but they didn’t get poor pregnant and lactating mothers in some Tabias. To select them from the seventeen Tabias, it has taken more than a month.

To improve the nutritional status of mothers, many NGOs are participating; but to cover all Tabias in the woreda it could need great efforts. There is an access of tomato, spinach and others in the home of the households; the only problem is the presence of poor feeding habit and the poor knowledge of nutrition importance among the community.

I: Do you think that pregnant, lactating women and adolescent girls in this community are suffering from micronutrient deficiencies?

P: Yes, goiter is observed in very few individuals in the community; and stunting is shown in some households in the children which indicate there is a shortage of balance diet food. But it doesn’t mean that they have the problem of food or the lack of food security. The nutritional problem is common in this community which is related to poor feeding system.

Anemia is also observed in some mothers; it produces a problem during delivery; but nowadays, there is an improvement on the prevalence of anemia among the pregnant and lactating mothers. Now the community starts to purchase edible oil, tomato, spinach, carrot; especially the Tabias nearby to the market; now you can observe some improvements in the consumption of diversified foods among the community.

In case of night blindness, I didn’t see the case but it could happen; because there is a problem of vitamin A deficiency in this community.

I: Do pregnant, lactating women and adolescent girls in this community suffer from overweight?

P: In the rural community this type of case is not observed; but in the urban community, it is observed in rare case of individuals, but there is a case of diabetes. Instead it is shown in high prevalence the case of thinness, stunting, underweight and anemia in the urban and rural community.

The case of diabetes in the community could be caused either due to the lack of balance diet food or ease;

I: What is the nutrition problems related to food insecurity?

P: As I have said before, food insecurity is not the serious problem of the community; but there are few female headed households face the problem of food security; because they give their farm land for common profit to male headed households; so they don’t treat properly as their own farm like they seed without fertilizer as the result they produce less products and they could face the problem of food security in some mothers.

Mostly the female headed household, who has not farm land and livestock production, could face the problem of food insecurity. Due to the lack of manpower, the female headed household doesn’t produce high agricultural products. There is also a culture that women have feeling the sprite of inferiority compared with male; like they feel as if they have no power to work their agricultural farm by themselves.

There is poor habit of feeding system; they are not more eager to eat for themselves. If they are married, they wait to eat until the husband arrives. They are also affected by high workloads; if both the husband and wife were engaging the whole day in the agriculture farm, then the husband take rest and ordered her to supply him like coffee and foods; the wife could also engage in the housework activities. This type of trend is not avoided still now although there are some improvements are shown; for example I am governmental employer and my husband too. I am doing in one Tabia and he is the same; then when we go to our home, I prepare food for dinner but he seats and observe TV until I prepared dinner for him. Imagine I am literate, how much this dominancy is applied in the illiterate ones. In the urban community, it is relatively better and if a chicken is prepared the women could eat part of it with no waiting for their husband but in the rural community, a woman could not eat until he visits home; this is not banned yet. The past culture is totally affected the women in the community yet.

**Section 2: Nutrition priorities in the woreda**

**I:** What priorities do your institution has in relation to maternal and adolescent health? Why?

P: If there is a problem to purchase a fertilizer by the women community, the agricultural office provides fertilizer with credit for women headed households only. But for others credit is not allowed. This is intended to boost their agricultural production and productivity. To bring 50% of the female participation in any position, training is given as priority for them.

To improve the nutritional status of mothers, women are participating in the demonstration site to show porridge, how to prepare it, how they feed, what are the main food types recommended for them are given a training. The source of the demonstrative materials like balance diet foods is purchased from Axum. We also make ourselves by mixing all types of food groups like cereals which is collected by the women farmers and we add small amount of spinach and carrot, then they porridge by milk or water and then adding iodized salt at the end.

I: Have you taken any training about food preparation?

P: We are taken training by agricultural bureau; there is regional nutrition expert who shows us how the balance diet is prepare, how mothers could feed, what are the basic food types are important for a mother and a child; there is also NGO (World vision) who trains for mothers about nutrition and annually they provide training for 50 women individuals and their husbands; first they have given training about which crop is basic for their nutritional status improvement, which animal or crop product is basic for body building foods like vegetables, fruits, milk, egg

**I:** What your institution is doing currently related to the priorities in relation to nutrition in pregnant and lactating mothers? What nutrition interventions have the most resources allocated to them?

P: As I have said before, the NGOs are providing six chickens and the seeds of home garden vegetables, such as orange color sweet potato, carrot and spinach and they also provide vegetable materials like hoe to each trainee. After the vegetables are grown they also provide training about the preparation of the food in a balanced way at their local sites; then the women by themselves also exercised the preparation technique. They provide the training for the last successive three years in four Tabias. There is some improvement in their awareness and they could also influence to their neighbours.

There is also a support from an agricultural growth program (AGP) to demonstrate in eighteen Tabias about preparation of food nutrition to pregnant and lactating mothers. Therefore, in collaboration with the different NGOs, we are providing training about how to improve the production and productivity of agricultural nutritious products to the pregnant and lactating mothers; and creates an awareness among them how to eat, how to prepare and who needs more nutritious food groups in the community.

**I:** How do you evaluate the changes?

P: As I told you before, it was much neglected field in our sector; now there is an improvement in our office. There are more than five development agents in each Tabia, who are specialized in different fields (like experts in agronomy, livestock, natural resource, cooperative, irrigation, and apiculture), so they are ordered to include nutrition promotion in addition to their main task. All the development agents were taken training about nutrition issues for three rounds. We trained them theoretically and all the practical section was done through demonstration of food preparation like how to prepare porridge containing five types of food groups; we also trained about moringa which is not commonly adapted for food. Moringa is very common plant in our community; so we provide training about how moringa chips, moringa tea and sweet potato is prepared.

We also trained them about the preparation of the high quality protein maize (ሙቁር ዕልዎ) in the form of bread with help of AGP and UNICEF budgets. As I told you before, the focus to nutrition in the agriculture sector was very poor; every expert was striving to promote the agricultural production and productivity. This nutrition field is an infant which was introduced two years ago in the agriculture sector; so now we are more focus on creating awareness among the community and the agricultural experts like the agricultural development agents.

**I:** How do you evaluate the priority given for the interventions to the women?

P: If there is well expansion of the new introduced technology to the neighboring communities after the women were trained, it is clearly that the promotion of nutrition among the community is conducted. We don’t have other measurements; but in the health sector, they could measure it in terms of stunting, wasting and underweight. I took the data last year from the health office but not this year.

I: How is the relation doing between agriculture and health sector in nutrition improvement?

P: There is a nutritionist in the health sector who works in collaboration with me; we always participate in the same training and observe all the activities that are performed in each office; but we didn’t evaluate the changes as the result of our intervention. Still now we don’t develop common plan between our sector and the health sector on the area of improving the nutritional status of pregnant and lactating mothers.

**Section 3: Nutrition interventions that improve adolescent and maternal health**

**I:** What kinds of nutrition interventions are in place to improve health of the pregnant and lactating women to your level?

P: During the training, we tell the pregnant and lactating mothers how much they are the most vulnerable groups to nutrition; but we didn’t follow how they feed in their home.

As I told you before, we always provide training about nutrition improvement for the mothers; for example we have given training for about 306 mothers in the last month; the main contents of the training was about the importance of nutrition on pregnant and lactating mothers; the quality quantity and its effect during pregnancy and lactation; we advise them to eat diversified food during pregnancy and if so they don’t face any problem during their deliver; we also advise them about the benefit of balance diet for the children. Advice about feeding of diversified food during pregnancy and lactation time is given by the agriculture and health sector; advices like to visit to the health facility, exclusive breastfeeding until six months of the child age then complementary food is needed after six months are common.

Now what I hesitate that whether all advises that are given to the trainee is applied practically at their home; in our side we didn’t evaluate and follow them. There are health extension workers and women development group that follows each women in the household; but we didn’t evaluate with the health sector expert about its implementation; now we have a plan to check the status of the interventions in each households.

I: Is there any nutrition screening program for pregnant, lactating women and adolescent girls?

P: Yes, they are screened at the health center; during the screening time, the pregnant and lactating mothers get advise how to improve their nutritional status like their feeding system especially if they fall below the standard during screening, they are advised to eat diversified foods like vegetables, milk, eggs, porridge, soup. They are also given tablets if it is deemed necessary.

I: How about targeted supplementary feeding?

P: They are only provided advice how to prepare and consume the home grown foods; unless there is no any supply of targeted supplementary foods in our community. But they are counseled to eat extra diversified and food.

There is advises about consumption of diversified foods that is given to the mothers by our sector and the health sector. For example all the agricultural development agents are visiting to each household to advice how to plant home garden vegetables and how to eat the produced vegetables properly. They advise to consume the pregnant and lactating mothers like spinach, carrot, sweet potato, high protein maize. Lactating mothers are also advised to provide eggs for their child by comparing the importance of one egg could substitute one kilo of meat. Training is routinely given to the beneficiaries; the only our problem is lack of evaluation for each households in person at their home.

I: How about counseling on the use of iodized salt?

We are advised to utilize iodized salt mainly to the pregnant women, because it has great importance for the minds of the child; thus we advise them to add the iodized salt after the food is flowed from the stove. Now the consumers are asking whether the salt is animal salt (without iodine) or human salt (iodized salt) before purchasing it. The price of salt with iodine and without iodine is the same. Therefore, all our community utilizes the iodized salt.

**I:** What advices do you contribute on nutrition sensitive agriculture such as home gardening?

P: They are advised to plant home garden vegetables regardless of the water availability in their home. Especially those who were trained by World vision Ethiopia are mandatory to have home garden vegetables. Organizations like save the children, World vision, and the Greener are encouraging women to have home garden vegetables; those organizations provide seeds, hoe and watering materials; they also provide small ruminant animals (three sheep per head) and they have plan to provide chickens. Here the intention is to feed the mothers from the sheep milk and the home garden vegetables are also used as diversified food for them. We also advise them to plant the home garden vegetables in a very small plot by fetching water from the river. Although there may be a shortage of water source in few Tabias, there could have an opportunity to purchase the vegetables from those who plant it with high water source.

I: What about on the need to be involved in safety net program?

02: Those who are elders that could not produce food by themselves are supported by the program. Some youngsters also with no farmable land are involving in safety net program and the poor are also supported by the program. There is no special favour for pregnant and lactating mothers to be supported in the program; but if they are poor, who are supported like the other community; in this case pregnant mothers get the support without involving the work and lactating mothers allowed to get the service without involving the work until six months of the child age.

I: How does your institution contribute in the reduction of workloads among the pregnant and lactating women?

P: As I told you before, all the pregnant women are not participating in the work of safety net program and the lactating mothers could get the service until six months of lactation without involving in the work.

The favour is not as such attractive (nodding her head to indicate unsatisfactory). There is also an introduction of best stove (ምርጥ እቶን) that could contribute on the reduction of workload for the pregnant and lactating mothers; they could not lost much time to search of firewood since best stove limits the consumption of much firewood. There is also an introduction of light solar in the households; but it is not possible to say the workload of women is radically reduced.

There is also an establishment of grinding mill in their nearby home; this could also reduce the workload of women. But still the workload in women is very high; for example they fetch water, collecting firewood, engaging in the agricultural and housework activities and the like. I could not say that we are creating the behavioral change on the reduction of women’s workloads in the community. Pregnant women are engaging in all activities of the agriculture, housework and others what a men could work. For example a pregnant woman prepares food for their family and she takes food to the husband in the agriculture farm; then she collects the crop what the husband sickled it; so how could we say the workload is reduced in women. We are talking only in theoretical rather than practically.

There could be support from the husbands; but let’s analyse the time for how much time the mothers are get rest. I don’t mean that the husband could not fetch water or collect firewood if the mother is pregnant or lactating; but there is very little support in terms of calculating the total time that she needs rest.

I: How about your advice on water, sanitation and hygiene services?

P: It was my main task before I joined to this field; I was doing in home and economics; I was dealing about personal hygiene, environmental sanitation, collecting of wastes, toilet and any garbage. I was showing the arrangement of house furniture, and I gave advice to separate the residence of human and animals. Now this activity is changed into health sector. Since I have the experiences on this field, I am working in the environmental sanitation in collaboration with the health sector through cleaning and compost production of the wastes around the homestead. I also educate the mothers how to keep their personal and environmental hygiene and the effect of dirty environment on their health and the health of their family.

I: How about the distribution of insecticide treated bed nets for the pregnant and lactating mothers?

P: In any service, priority is given for pregnant and lactating mothers. Especially we believed that a pregnant and lactating mothers with less than one year age child needs more advice. Therefore, the service of insecticide treated bed nets is given first for them. In case of World vision, they support mainly the pregnant and lactating mothers of female headed households.

**I:** How is the service of targeted supplementary feeding (TSF) for pregnant and lactating mothers?

P: If a child is below standard after the child has been screened, a supplementary food like fafa is provided. But an advice to consume diversified foods is provided for pregnant and lactating mothers; there is no any food support for them in our community.

**I:** What kinds of nutrition interventions are in place to improve health of the adolescent girls to your level?

P: In our side, we didn’t support them, except some training. We invite teachers to get training with the pregnant and lactating mothers and we supposed that teachers could get TOT so as to provide training to the adolescents in school; we train the teachers about the improvement of nutrition for adolescents and how much nutrition is important for them so as to develop their brain and they will be moms by tomorrow. I gave training about importance of nutrition to the teachers and the male and female students. But we didn’t do any about nutrition for out-school students.

I: Which of the interventions listed before do you think is most important for the pregnant and lactating women?

P: It is impossible to say we did best; but relatively we are doing fair in the area of mothers and children of their nutritional improvement. There is somewhat progress in the nutritional improvement of mothers and children; for example after we trained to the pregnant and lactating mothers, you could see a sort of expansion the introduced technologies; you could observe the technology in other households who didn’t participate in the training; if you train for ten individuals then you could see other ten, twenty individuals who get involved; you also appreciate the peer to peer training. Therefore, awareness is slightly created among the mothers on the importance of nutrition. Although the implementation isn’t as such catchy, as a start it is ok.

I: In your opinion, which of the interventions for the pregnant and lactating women are being implemented in successful way? Why?

P: Even though there is no specific measurement for effect of our intervention, you could notice that women purchase different vegetables from the market; you could also observe home garden vegetables in some households; they also give witness of benefit from the training like they understand the importance of sweet potato. Regardless of its coverage, we could appreciate the creation of awareness in limited mothers. Now most of the mothers know at least the importance of diversified food although they don’t fully apply into practice; this is great success. Sometimes they ask you about their child feeding; so this indicates we are starting to create demanding society.

Mainly our main focus is the pregnant and lactating mothers and their children; but I don’t mean that others didn’t participate in the intervention.

During our supervision, one woman showed us the cooking technique of cabbage, in the past the fluid part of the boiled cabbage was spilled to the ground; but now they utilize the fluid which is the main component of the cabbage; the same is true in sweet potato and others; this is the results of our education.

I: Which of the programs mentioned above are less effective? Why?

P: We didn’t do more in the adolescent girls like the pregnant and lactating mothers; we didn’t also address the coverage of each household; you mightn’t get enough time to address each household in each woreda. Regardless of your arrival to each kushet, they could also not involve all mothers in the training or demonstration site.

I: How is the structure of your service to address each household?

P: There is a structure like women development groups, agriculture development agent that could diffuse the information; the problem is the development of dependency mentality among the mothers; for example if you invite to the woreda, every invited individual is attending because it has perdium; but if the training is at their kushet few mothers attend due to no perdium. Even they could produce many things in their home, they are very eager to attend training at woreda to get even ten birr perdium; only very few individuals are complaining about the invitation mainly during the working days. The mothers give great value for perdium; sometimes they resist you to apply the technology into practice if they didn’t participate perdium based training. If we call them for training to Woreda, every woman/man will come even those who are not invited. Because perdium is given if they get training at woreda level, and they are accustomed to taking perdium by the non-governmental organizations during the trainings.

**Section 4: Implementation challenges and community factors affecting access to nutrition interventions**

I: What are the challenges to implement delivering the nutrition interventions that we have been discussing for pregnant and lactating women?

P: The main challenge for the implementation of the intervention is due to the lack of focus by all responsible bodies from the above to the household level; especially the agricultural sector is more focus to increase production and we don’t understand as it could return back. Had it been a focus to nutrition like boosting to production, it could bring great change in nutrition improvement among the community. The other problem is the presence of dependency mentality; shortage of diversified food in some individuals is also another challenge.

I: Is there a relationship between educational status of pregnant and lactating women access to interventions?

P: Education is also a factor to accept and implement the interventions; for example those who married after they complete their education are very fast to accept it; the illiterate one resist to accept the new technology and they act as if they know it and they always correlate with shortage of resource; but some are regret for what they did, they said that we maltreatment our children with lack of knowledge.

I: How the belief and norms affect the feeding behaviour of pregnant and lactating women?

P: In the community, clearly they don’t say that this type of food is for male and this for female; but they compared with their way of growth; for example some individuals said that what is the special now, we ourselves grew without any access of diversified food; we remembered how we grew and still we are strong; why we should care more for diversified food if we filled our stomach. Some elders supposed that as if there was high access of honey and butter in the past time but not now.

On the other hand, most people reflect that the importance of diversified foods and they appreciate the attention of the government towards the nutrition improvement. Some people also agreed that the home grown food is possible to prepare in a balance diet food; the problem is not due to the lack of food but to our negligence. For example we were giving training last week, one man who seat at the back then during the presentation he was impressed and came to the front and he explained that the training is very attractive and this should be strengthened; we were assuming the stunting is derived from their patents; our children were suffered unknowingly.

I: What food types do pregnant, lactating women and adolescent girls avoid?

P: In our community, there is no food type that could not edible by mothers and adolescent girls; but when I was in Tsegede, egg and milk is not recommended to eat by pregnant women.

The main problem of women in this community is stay without eating until the husband arrives. If they prepare chicken, they don’t taste until the husband attains to home. Some individuals also give their leftover to their children. Some women also said that if a pregnant woman takes coffee, it could stay on the child head during delivery; and some women also don’t eat meat and egg if so the size of the child get large who could difficult during delivery. But we advise them to eat more food so as to become softer their body and they get deliver easily.

I: Does the intervention access affect by transportation and cost?

P: For pregnant women, there is ambulance service. For other purpose, it is not more affected by transportation or cost; most of the Tabias have an access of transportation. There is also car assigned for transportation of the community in the far Tabias. But the pregnant and lactating mothers are fetching water whether from the near or far distances.

In case of access, there is no the problems of supply in the market; the problem is the lack of awareness in some individuals that may not purchase the nutritious food items instead they buy coffee and the like.

I: How convenience is interventions to the pregnant and lactating women?

P: Compared to the past time, there is great change in the community awareness; for example the people request insecticide treated bed nets for the health expert which indicates the awareness of the community. They also brought quality seed from Humera to seed it here, for example the seed of onion is brought from Humera which is border of Sudan; this indicates they understand the importance of each introduced technology; now there is no as such difficulty to implement new technology; the problem is feeding habit; they more focus to produce market oriented. In some training also, the representative body may not attend unless you visit to each household’s home. Especially it is very difficult to conduct training without perdium in Tabias, very few individuals are attending; everybody develops the sprite of dependency like the perdium or any aid from the government or non-government bodies; but in case of the pregnant and lactating mothers, there is strict follow up by the women development group and by the health extension workers; so there is no difficulty to give training in every site; they don’t associate with perdium except few mothers.

I: How do you explain the quality of the intervention?

P: Now we have development agents in each Tabia, although their main task is not nutrition promotion, they have been given the assignment to promote nutrition in addition to their main task. For example the expert of irrigation could encourage the mothers to plant home garden vegetables; it could be nice had it been a structure at the Tabia level, but the assignment was given to each development agents and they are evaluated about performance of the nutrition intervention.

Now we have a specific plan to seed the total size of the plot that could be covered by selected seeds in the given year; for example in case of the high protein maize, we plan that how much hectare will be covered in each Tabias by the high protein maize crop, and the same is true for other vegetables like spinach, and then you communicate with crop and irrigation experts in the Tabia, and the experts take their share of tasks and they will be evaluated based on their performance at the end of the time. All the agricultural development agents include the nutrition agenda with their main tasks. For example the agronomy expert encourage the households to crop the high quality protein maize in their plot; s/he also advises to plant the home garden vegetables; the same is true the other experts.

As I told you before the quality issue is not well addressed; you can easily understand that the time taken for boosting of agriculture production and productivity in the region; thus it is difficult to evaluate the quality of service in nutrition interventions within two years; for example I am the only food science expert in this woreda and I could support one Tabia through practical demonstration; the other Tabias are covered by agricultural development agents which is a secondary task for them, they could not eagerly work as their main task; this could affect the quality of the service. To improve the quality service we share the activities in the woreda agriculture extension workers and diffuse the information to the Tabia agricultural development agents and then we evaluate their performance at every quarter of the year. But this year, we didn’t do in such approach due to the JEG civil service reform program.

Sometimes the assigned experts could forget the given assignments of nutrition intervention in the community; because, it is not the main task of the expert which is secondary task.

I: How do you evaluate the commitment of the experts in diffusion of the nutrition interventions?

P: With in the extension department, the self-learning activity is performed among all the experts; in this perspective I present my plan with clear objective of the activities and then as I have told you that we share the assignments of the nutrition intervention activity to all experts based on the yearly plan. But during their time, they may miss the nutrition plan being it is their secondary task. It has an infant age since the nutrition program was introduced to the agriculture sector like the other fields; so now, we are doing the creation of awareness among the experts and the community.

This year, we will demonstrate in the eighteen Tabias in collaboration with the health sector since we have budgets subsidized by the agricultural growth program.

I: What resources exist to provide the interventions and what don’t exist?

P: This woreda is known by the presence of high burden activities, thus the agriculture development agents release from the assigned Tabias to other woredas with less workload; then untrained expert is recruited or shifted from other woredas; in this case you are required to train the new comer experts and you need financial support to provide training for such individuals. Unless we get from different projects, it is not allocated any budget for such cases. Due to the nature of the field it needs a sort of demonstration unless it is difficult to convince the mothers in theory only. For example you should demonstrate the preparation of sweet potato in terms of biscuit, bread, kelo (ቐሎ), porridge, soup, and the like so as to convince the mothers to apply practically in their home.

The agriculture development agents also need the practical refreshment training so as to strengthen their responsibility to promote nutrition in each Tabias.

I: What about your existing resources to provide interventions?

P: We don’t have any resources to provide the nutrition demonstration interventions; but we have the agriculture development agents with different professions in each Tabia; as I have told you before, there is turnover of experts and it is a secondary task for the agriculture development agents.

I: What are the solutions that your institution have applied to effectively implement the interventions for women and adolescent girls?

P: The presence of nutrition structure to Tabia level could very important to implement the nutrition interventions effectively at the ground level like the health sector; because it is not taken as a secondary task like to other fields. There should be also budget allocated to give the theoretical and practical nutrition training for mothers and other responsible bodies. If there is budget allocated, you could accomplish your plans timely. There should be also given an attention to its promotion and implementation of the activities at the ground; the attention should start from the woreda administration head, agriculture and rural development head and the other responsible stakeholders. The Tabia leaders should be also given the orientation about the nutrition promotion and intervention activities from the woreda administration heads like the other fields. For example when the Tabia leaders are coming to woreda, they are given an orientation about irrigation, natural resource, livestock and crop production but not about nutrition activities. Therefore, the influence from the upper position is mandatory in order to implement the nutrition interventions effectively at the household level.

In the area of education, we start now, how to influence the students to their family in the consumption of diversified foods; I communicate with one school teacher and he developed a teaching chart in the form of pyramid and get started promoting the importance of diversified foods in the improvement of nutrition in the community. One responsible teacher has got training from each school about nutrition by World vision. Now the trained teachers requested us the seed of home garden vegetables to plant in their school to serve as demonstration to the students. So the students could initiate their family to plant the home garden vegetables in their homestead.

I: Is there nutrition education as a subject in the school?

P: No, had it been nutrition education in school, it could be nicely promoted the importance of nutrition in each family; I believe that it could be eradicated the malnutrition problem in our region; because almost every household has a student except very few individuals; so the students in their family could change their feeding system and by tomorrow they will establish family by themselves. When I was a student, there was a teacher that deals about personal and environmental hygiene, how to sanitize the house and house furniture and I was learned a lot and I developed the interest of the field; it had great value in changing the community awareness about food hygiene and environmental sanitation.

I: What your institution can be addressed the challenges in a better way?

P: Now it is started to considered as an agenda in the agricultural sector although the influence is not like the other fields; but the office head has started to train the community about the depressing of malnutrition prevalence in the region and he advised them to crop the high quality protein and carbohydrate maize; therefore this starting is an indicative how much he could influence the community to apply practically at the ground. This high quality protein maize is also high in production compared to the previous once.

**Section 5: Multi-sectoral collaboration to improve maternal nutrition**

I: Do you feel it is necessary at your level to work with other sectors/ institutions to address maternal nutrition? What about for the adolescent girls’ nutrition? Why?

I: Our stakeholders who work in the improvement of nutrition in pregnant and lactating mothers are the heath sector, education sector, water sector, women affaire and from the non-governmental organizations like the World vision, agriculture growth program, save the children, Amsalu, REST.

There is steering committee that the woreda leader is the chairman and the head of the health office is the secretory and our bureau is a member and the vice of every sector is technical committees; but in practice it is almost nominal that doesn’t work the given assignment. For say an example, I took a letter to the woreda administrator about how to promote nutrition in pregnant and lactating mothers, but I did get any response; mainly they focused in to other big assignments.

The World vision provides trainings to education sector how to make awareness the importance of consuming diversified foods to the teachers and the students.

I: How do you see the other institutions’ role complementing your role in improving maternal and adolescent nutrition?

P: The non-governmental organizations support us in providing training about nutrition improvement to the pregnant and lactating mothers; for example REST provide seed vegetables and training how to plant the vegetables in the women’s homestead for sixty women; I also train them about the importance of vegetables in the improvement of their nutritional status and how to prepare and consume the vegetables for themselves. REST also brought the sweet potato from other areas and distributes the seedlings to the mothers.

The World vision also provides vegetable seed and training for about fifty lactating mothers; and they will train them about nutrition after the vegetables are produced. They also provide us financial support to show the nutritious food preparation for the agricultural experts through practical demonstration.

The health sector is measuring the nutritional status of mothers and children and they counsel how to feed the diversified food types; and we advise the mothers to produce diversified foods, such as home garden vegetables and production of small ruminants and poultry. There are also religious leaders that are invited by the REST and get training with the mothers about nutrition; so as to give advice to the community in consumption of diversified foods.

Therefore, the importance of stakeholders are supporting in capacity building of experts, strengthening the integration of sectors and gaining of resource and materials to the pregnant and lactating women.

I: How do you evaluate the level of collaboration among sectors in nutritional interventions?

P: At the end of their intervention, there is a supervision conducted by all stakeholders and they observe the status of the home garden vegetables, how it grows, how it was treated, how the mothers could consume for them and their children. The mothers could give their witness how much they are benefited and we also appreciate the diffusion of the introduced technology to other communities. We didn’t evaluate in details in such a way that how much production they get, how many of the communities are benefited, what nutritional change they bring and so on;

The women are called four times to attend training, such as training about environmental sanitation, water, nutrition, vegetable planting by the non-governmental organizations; and during this time, they are provided some financial support in terms of perdium during the training time and get seed and irrigation materials for the trainee and other inputs like chicken; therefore, the women are benefited from the support but I have reservation in their attendance during training; had it been prepared by our bureau without perdium, they might not be attended.

I: What kind of change in terms of the way stakeholders work together is taken?

P: Relatively to the past time, there is great change is observed; when I went to the community, they have positive attitude towards the nutrition intervention activities, they acknowledged to the non-governmental organizations and they liked the nutrition field. Actually you could observe the changes in person; at least the community has developed awareness on the importance of feeding diversified foods, home garden vegetables, and the need of diversified foods for pregnant and lactating mothers. Plus the engagement of fifty pregnant and lactating mothers in the nutrition programs like getting the small ruminants, seeds and chicken every year is not a small figure.

I: What type of resistance to the needed change do you perceive or have you experienced so far?

P: Mostly our activity is done in collaboration of the non-governmental organizations; but the women affaire is also doing in boosting of the women benefits and they suggested that the large number of women in any intervention should be encouraged. However, in the side of the governmental organizations, there is no any organized plan about nutrition improvement that we shared among all stakeholders; to evaluate the status of nutrition intervention activities, the governmental stakeholders need perdium and in this case we don’t have the budget to run it.

I: How effective are the coordinating platforms in enhancing multi-sectoral coordination?

P: In the health sector, we are doing in integration way that we share the reports, and we discuss together about the nutrition problems and its solution. With World vision, they provide four water pumps to the community and other irrigation materials; the REST also provides training to 306 mothers and they are purchasing three sheep per individuals and they will also provide chicken. Therefore, the presence of office financial problem is solved by the non-governmental organizations. So it could be possible to use this media so as to promote the nutrition agendas in the community.

We aware the mothers that how the child feed diversified foods, at what time interval the child feeds, the amount, the type like eggs, vegetables, carrot, milk.

Any vegetables can grow in our community, such as carrot, sweet potato, potato, tomato, spinach, swiss chard, and crops like, the high protein maize, teff, maize, millet. In the past, the mothers sold the eggs and butter and purchase coffee and sugar, but now you could observe the women that purchase the vegetables such as spinach, swiss chard, tomato, potato for their consumption in the rural communities.

We also advise them to consume eggs for themselves and for their children. If the product is surplus, we advise them to sell and change other house expenses.

I: What opportunities do exist to promote multi-sectoral coordination of nutrition in this woreda?

P: we have a hardworking community that could introduce new technologies and diffuse to other communities in short period of time. The women become exposing to different forums is great success; since in the past time, women didn’t participate in the community meetings and it was difficult to introduce any new technique or technologies; the attention of the government and the presence of nutrition supporting non -government organizations, the presence of different more than five development agents in each Tabia.

**Section 6: Other interventions that influence adolescent and maternal nutrition and health outcomes**

I: Why delayed marriages (after 18 years) improve maternal nutrition?

P: Now days, it could possible to say that there is no underage marriage in the community; you could observe that some people may cheat the rule by having different evidences; it is conducted in rare case. Last year about four family heads were prisoned; there are women affairs and the women development groups that seriously follow the underage marriage. We are doing better in this area; now the community own that underage marriage is illegal activity.

The community believes that the underage marriage is not supported by low; and if the underage marriage is conducted, the community believes that delivery problems like fistula could be encountered and the problem could also affect the child.

I: Why would increase the space between each birth improve maternal nutrition?

P: The long birth spacing is started now, but it is not banned like underage marriage. Although there is good awareness among the community, in practice you could see some children with short birth spacing between the successive birth children.

Majority the birth spacing between successive birth children is three years.

I: What programs or activities promote increasing birth intervals in this level?

P: The training was given for long time; and the community aware very well, some may do intentionally and others could be unknowingly; we give training about the importance of increasing birth intervals between successive birth child by giving an example of using raw planting technology to plant vegetables or to seed crops; if vegetables are planted with enough space you can see the development difference compared with the narrowed one.

Religious leaders are participating in the training and help in promoting of long birth spacing between successive birth children. Therefore, the awareness creation among the community is well done; but I don’t mean that some individuals did unknowingly but they think that the principle of growing the child by chance and they thought that child is an asset. Therefore, we can’t say that the family planning is totally applied among the communities like the underage marriage.

I: What are the community factors that affect underage marriage and birth spacing?

P: In case of underage marriage, if a girl is asked for marriage the family of that girl thought as if it is golden opportunity for her; others also think that if a girl stays unmarried, she will have boyfriend without the permission of her family, this is also uncommon culture in the community. Even some families could like to divorce the girl after she has married; but now this type of thinking is more reduced.

In case of birth spacing, as I have said before, some families think that more children are an asset and they don’t show willingness to use birth control. There is also a fear of remaining unproductive (sterility) among the mothers if they used birth control drugs. Nowadays this type of thinking is reduced; because they show women get birth after they used the birth control drug.

Generally what we understand that we should work hard to make great awareness among the community mainly in the area of family planning like what we did in the prevention of underage marriage. As we have discussed before, this issue should also work in schools; if it is given awareness to students, they could influence and make aware to their families.

I: Can you think of any opportunities to prevent early marriage and increasing birth spacing?

P: If we take as great concern, we are many stakeholders like the health sector, education, water, and agriculture that could make awareness among the community through our fields. Therefore, integration among all stakeholders is very important. The presence of religious leaders is also an opportunity to convince the community with easily manner about this issue.

**Additional remarks**

We will extend the different technologies in the demonstration site to get benefited the pregnant, lactating mothers and the adolescent girls. We will do in schools to promote nutrition among the school students and their family. Unlike we grew, if we could do in the pregnant and lactating mothers we will have healthy citizen.

All stakeholders should also take as our priority task and we should use the introduced technologies properly. We have supportive non-governmental organization in the promotion of nutrition improvement in each Tabias of our community; the health sector has structure to diffuse the technology and the agriculture sector has also agricultural development agents that could promote it.

Thank you I am very much happy having this type of discussion since you give more attention to this agenda and I have get encouraged to work a lot. You are coming here which is an indication of how much the sector is given more attention. Once again thank you, I hope it will be improved.

**SUMMARY**

**Section 1: Common maternal (pregnant women, lactating women and adolescent girls) nutrition problems in the community**

- The agriculture sector introduces the high quality protein maize (ሙቁር ዕልዎ) and we are working to distribute this agricultural product to each household. Since it is rich in carbohydrate and protein.
- The weight which is given to the nutrition activity in our office is poor; for example there is a committee that was established in such a way that the woreda leader is the chairman, the head of health office secretary and the office of agriculture, education women affaire are members. But this committee is… (She showed frown face which was an indication of unhappiness).
- There is no as such food problem in the community but the feeding system is poor; this is mainly due to lack of awareness.
- Save the children wants to select the poor pregnant and lactating mothers to support small ruminants and poultry production; but they didn’t get poor pregnant and lactating mothers in some Tabias.
- There is a culture that women have feeling the sprite of inferiority compared with male to do something; like they feel as if they have no power to work their agricultural farm by themselves.
- The married women could wait without eating until the husband arrives; especially like chicken food.
- If both the husband and wife were engaging the whole day in the agriculture farm, then the husband take rest and ordered her to supply him like coffee and foods.

**Section 2: Nutrition priorities in the woreda**

- If there is a problem to purchase a fertilizer by the women community, the agricultural office provides fertilizer with credit for women headed households only.
- World vision trains for mothers about nutrition and nutrition improvement and annually they provide training for 50 women and their husbands in the community.
- The World vision are providing six chickens and the seeds of home garden vegetables, such as orange color sweet potato, carrot and spinach and they also provide vegetable materials like hoe to each trainee.
- Moringa is very common plant in our community; so we provide training about how moringa chips, moringa tea and sweet potato is prepared for the mothers and to the agricultural development agents.

**Section 3: Nutrition interventions that improve adolescent and maternal health**

- Advice about feeding of diversified food during pregnancy and lactation time is given by the agriculture and health sector; but we didn’t evaluate the changes that arise as the result of our intervention.
- We advise them to add the iodized salt after the food is flowed from the stove. Now the consumers are asking whether the salt is (without iodine) or human salt (iodized salt) before purchasing from the market.
- The favour for the pregnant and lactating mothers in our community is….. (Nodding her head to indicate unsatisfactory).
- There is an introduction of best stove (ምርጥ እቶን) and light solar that could contribute on the reduction of workload for the pregnant and lactating mothers by reducing much time to search of firewood.
- But still the workload in women is very high; for example they fetch water, collecting firewood, engaging in the agricultural and housework activities….
- We invite teachers to get training with the pregnant and lactating mothers about the nutrition improvement and we supposed that teachers could get TOT so as to provide training to the adolescents in school.
- During our supervision, one woman showed us the cooking technique of cabbage, in the past the fluid part of the boiled cabbage was spilled to the ground; but now they utilize the fluid which is the main component of the cabbage.

**Section 4: Implementation challenges and community factors affecting access to nutrition interventions**

- There is the lack of focus by all stakeholders from the Region to the Tabia level.
- The development of dependency mentality among the mothers; for example if you invite to the woreda, every invited individual is attending because it has perdium; but if the training is at their kushet few mothers attend due to no perdium.
- Some women also don’t eat meat and egg if so the size of the child gets large who could difficult during delivery.
- The agricultural development agents could not eagerly work as their main task since it is a secondary task for them and this could affect the quality of the service.
- The presences of high turnover among the agricultural development agents affect the diffusion of nutrition intervention technologies among the communities.
- We didn’t evaluate the status of nutrition intervention in such a way that how much production they get, how many of the communities are benefited, and what nutritional change they bring and so on.

**Section 5: Multi-sectoral collaboration to improve maternal nutrition**

- There is steering committee like the woreda leader, health, education, water, agriculture and women affair, but in practice it is almost nominal that doesn’t work the given assignment.
- The non-governmental organizations like World vision, REST, Save the children, AGP that support us in providing training about nutrition improvement to the pregnant and lactating mothers; and they provide seed vegetables, chicken, sheep and they train how to plant the vegetables, how to prepare food and how to feed the products.
- The importance of stakeholders are supporting in capacity building of experts, strengthening the integration of sectors and gaining of resource and materials to the pregnant and lactating women in the community.
- We have an opportunity of having hardworking community that could introduce new technologies and diffuse to other communities in short period of time.

**Section 6: Other interventions that influence adolescent and maternal nutrition and health outcomes**

- We are doing better in prevention of underage marriage; now the community own that underage marriage is illegal activity.
- Although there is good awareness among the community about long birth spacing, in practice you could see some children with short birth spacing in the community.
- We give training about the importance of increasing birth intervals between successive birth children by giving an example of raw planting technology; like if vegetables are planted with enough space you can see the development difference compared with the narrowed one.
- In some families think that more children are considered as an asset and they don’t show willingness to use birth control.

**Additional remarks**

- We will extend the different technologies in the demonstration site to get benefited the pregnant, lactating mothers and the adolescent girls.
- We should do in schools to promote nutrition among the school students and their family.
