# Supplementary material for: Barriers to utilize nutrition interventions among lactating women in rural communities of Tigray, northern Ethiopia: An exploratory study
Source: PLoS One. 2021 Apr 30;16(4):e0250696. doi: 10.1371/journal.pone.0250696 (PMC8087028; doi:10.1371/journal.pone.0250696)
Supplement: S2 File — (ZIP) [file pone.0250696.s002.zip › S2_File.Doc/Woreda level and above key informants/125_IDI_Woreda health office_Lalay Maychew woreda.docx]

**Operational Research on Adolescent and Maternal Nutrition in Northern Ethiopia**

*Date: Nov 20, 2017*

In-depth interview with Woreda health office Head, Laelay Maychew, Tigray.

**Section A: Interview Details**

1. Zone: Central zone
2. Woreda: Laelay Maichew
3. Kebelle: Axum
4. Name of key informant: Gidey
5. Institution of key informant: Woreda Laelay Maichew
6. Interviewer name: Mengistu Mitiku
7. Date of interview: Nov 20, 2017
8. Interview start time: 2:41 PM
9. Interview end time: 4:07 PM

**Section B: Interviewee professional information**

1. Gender: Male
2. Age: 52 years
3. Highest level of completed education: BSc holder
4. Current Job position: Woreda Health Office Head
5. How long have you been in the current position: 6 years

**Main interview**

**I:** Interviewer

**P:** Participant [The key informant]

**I:** I am Mengistu Mitiku from Mekelle University. This is a research on maternal and adolescent nutrition. It is being conducted in collaboration with Regional health bureau, Federal and UNICEF. Our conversation may take 1 and half an hour to 2 hours. If there is unclear question, you can ask me. If there is a question that doesn’t go with you, you can tell me to skip it and then will be out of the record. Any question before we start.

**Section 1: Common pregnant women nutrition problems in the community**

**I:** The first question says, considering the current situation, what are mothers and those we call them adolescents in your community doing? It is not what your office and their families are doing for them. It is regarding what they themselves are doing?

**P:** The mothers in the mentioned category are doing different activities to make themselves healthy considering the existing situation and unity of the community. One of the activities which we consider it good is the efficient utilization by mothers. Mothers are improving their efficient utilization of the economy they have without any support. The other is regarding their condition of hygiene. So, the condition of mothers in keeping their clothes hygienic and their person hygiene also is increasing. When we consider adolescents, they exchange advice from one another in a transparent manner and this is improving from time to time?

**I:** Let us continue. We are still in pregnant women, lactating mothers and adolescents. What nutrition related problems are common among them in this community, Laelay Maichew?

**P:** As there are some kebelles which are semi-low lands, the nutrition related problems that occasionally occur among pregnant and lactating mothers and adolescents is anemia. This occurs rarely especially in pregnant women. When we come to the time after delivery, the problem of bleeding occurs occasionally. This has an implication of the nutritional status of the mother. There are no other problems related to nutrition in mothers.

**I:** Maybe, is there anything you would like to share me with regard to the disproportionate imbalance that exists between the height and weight of mothers with their age?

**P:** When we see the relative height and weight of mothers compared to their age, it is good in most kebelles. As to us, it is balanced in most kebelles. However, there is imbalance of age with weight and height in limited number of kebelles. We conduct surveys in schools and we have confirmed that the problem exists. There are adolescent girls who have the problem of imbalance between age and height as well age and weight.

**I:** What about the issue of overweight? Does this problem occur in your community?

**P:** When we see it in relation to previous data, overweight among mothers is occurring though we don’t consider it as a problem. There is an increasing tendency of overweight especially among females who live at the periphery of the city, Axum. However, we still don’t consider as a problem though evidences are appearing.

**I:** What about the issue of food security among mothers and adolescents in this woreda in relation to the fact that your woreda is among the ones where food security is declared. Thus, considering the three categories of community members, what can be said?

**P:** When we see the food security condition of mothers in this woreda, it is a better one as far as woredas are considered. This is a general view. Howeve, the condition of food security varies from kebelle to kebelle. Its socio-economic status is better and there is no dependence from any economic support. When we, as woreda health office, view it in pregnant women, lactating mothers and adolescents, we would say that they are almost food sufficient. Their habit of diet in take is also good. The community as a whole also supports mothers, unlike other areas where the community expects the support to come from the government itself. Here, we don’t have such problems. But, there are kebelles at the periphery like those neighboring to Aydet, Mereb woerdas where the food security issue might not be like the other kebelles.

**I:** With regard to the group of mothers who are being attacked by nutrition related problems, what can tell me? Are pregnant women, lactating mothers and adolescents who are easily attacked by nutrition related problems?

**P:** As to our professional view and evidence we have, pregnant women are the ones easily attacked. If you ask me why, first they need support though the understanding of those who can support is low. In this woreda, mother are left alone as their children are at schools and even they have the burden of looking after their cattle and also they have additional burden of agricultural activities. So, when we see things considering those all, pregnant women are easily attacked in terms of health issues relative to lactating mothers and adolescent girls. When we consider lactating mothers, they get good care after delivery. When we also consider adolescent girls of these days, they get good respect from their parents. They are at the age of schooling. Thus, parents support them to go to school, to develop their capability and become good citizens. It is not like the previous time of putting pressure that parents are practicing now. So, it is a better condition with regard to adolescent girls.

**I:** Okay. You told me that lactating mothers get care after giving birth. What is that care?

**P:** When we consider the support pregnant women get in terms of food and the like, it is improving. Farmers have developed a good habit of giving care to their lactating wives. Since most of them give birth at health facility, they get advice and when they go home after delivery, the husband is communicated to take the responsibility. Relatives also give her a good care. This is in terms of food support the lactating mother gets. The second point is the rest they get. They take rest and this enables them to get a good benefit. Therefore, the support they get until they come to the normal health status is good. That is why we see a good outcome in the mother itself and her baby. Their physical is good.

**Section 2: Nutrition priorities in the woreda**

**I:** Okay. We were in the introductory section. We will talk about the nutritional interventions which are given priorities and are being executed. Of course, there could be much intervention. But, which are the interventions with priorities on the three groups of females mentioned so far? Let us start from pregnant mothers.

**P:** When we start with pregnant women, the better job we are doing in pregnant women is awareness creation. We know becoming pregnant and giving birth is a normal process. Therefore, we are doing continuous awareness creation activities in pregnant women. The first is about what nutrition. The second is about the economic wise utilization of what mothers have and also the impact of pregnant mothers’ nutrition on the fetus, the support families and relatives should have for pregnant women. Generally, education related to nutrition as indicated by the regional health bureau is being given focus. The other is the screening of mothers for their nutritional status every month. This is to check whether they have good diet taking habits or not.

**I:** Let us consider only the activities given priorities first and then we will proceed to the other activities.

**P:** Okay. Therefore, as I told you the activity given priority is the nutrition related health education. This is the one which we have a big focus on. We are working here as to the network they have already established. More specifically, pregnant women are made to have conference on monthly basis that will help them upgrade their understanding. In addition, we also provide them iron-folate to prevent them from developing iron deficiency anemia. Husbands are also communicated to make them supportive. Finally, we always focus on pregnant women when there are campaigns.

**I:** It is my first time to hear that your woreda have a system in place to communicate with husbands. Do you meet the husbands on weekly, monthly or every six month? What does the communication focus on?

**P:** Primarily, the discussion focuses in making the husbands participatory in the health of their wives. In previous times, there was pressure on the wife; with the husband placed on higher level and the wife on lower level. If the mother comes to health facility, we give her letter appointment that also includes that her husband should accompany her while she comes back to health facility. This avoids the inferiority complex pregnant mother encounter. When they come together, many things are done. It is not only on nutrition that we focus on. Discussion will be held on how the husband gives her care, about her food intake habits, where she gives birth, what the role of the husband is. The husband will clearly be communicated in relation to all these issues. After making him know everything clearly and then convincing him, we present them the request to undergo voluntary counseling and testing of HIV. If the husbands are convinced in such a way, they bring their husbands the day they are probably to give birth. Therefore, it is mostly on feeding practice that husbands will be oriented. If it is possible, she will use everything the household owns and if there is something which the mother wants but not available in their household, the husband will buy what he has and bring what she wants. Husbands will advised in such a way very properly.

**I:** Let us go to lactating women, what prioritized nutrition interventions are you doing to improve their health?

**P:** As I told you, families have a good support in terms of food delivery to the lactating mother. As a health office, the one we give exceptional focus is we are working on the fact that lactating mothers should eat foods which enables her to produce adequate breast milk to the baby. This is because the baby should exclusively get breast milk for the next 6 months after birth. This is possible if the mother produces adequate breast milk. Therefore, the lactating mother will be advised to eat foods that enable her to produce adequate breast milk and to take adequate rest. If she faces a problem, she is also advised to call and communicate with women development army around. It is in such a way that they get the support.

**I:** For how many months after delivery does she take rest?

**P:** They take rest for six months. They are free of any public work for six months. Lactating mothers have no pressure and if she has extraordinary medical permission, she may have the opportunity to have additional time to take rest.

**I:** How do you see the 6 months’ time given for lactating women to have rest? In some areas, they are exempted to take rest for 10 months. But, here it is six months. So, on one hand, we are saying the baby should grow well, having well matured mind. On the other hand, we are giving the lactating mother 6 months. So, what do you think? Don’t you think 6 months is short?

**P:** Yes, it is short. You are right. But, when they start public work, they don’t deal with activities that need physical strength. They do things seated. There are many activities in the soil and water conservation activity. For example, if a mother resumes work 6 months after giving birth and if there are 2 or 3 pregnant women who came from the same village, they spend the water and soil conservation time caring children, communicating and preparing some refreshment related services like tea and coffee. Otherwise, they don’t deal with strong work that needs physical strength. As you said, 6 months is short and it could have been better should the mother been given more than 6 months.

**I:** What is your woreda’s biggest intervention to adolescent nutrition?

**P:** Alongside the plan we have for changing the community’s diet intake habits, we give health education for adolescents in schools about nutrition as they are the factory of future generations. As adolescent girls are living with mothers, the experience will help them and we also support them through health education to protect them from any jeopardy. We also work in preventing under age marriage as it is harmful for them. In few schools, the practical demonstration of food preparation is being implemented as it is believed that adolescent girls are future mothers.

**Section 3: Nutrition interventions that improve maternal and adolescent nutrition**

**I:** You told me about the prioritized nutrition related interventions. Let us go to the routine interventions. What routine activities related to nutrition support of pregnant women, lactating mothers and adolescent can you tell me and are in place? Let us consider pregnant mothers first.

**P:** In pregnant mothers, one of the routine nutrition related interventions is to assess the weight of pregnant women at different intervals. The second is to distribute iron tablets as per the standards set. In lactating women, there is distribution of vitamin A. In addition to this, pregnant women receive advice though our health workers and give them the opportunity to learn from each other. There are three members who always avail themselves during the monthly conference of pregnant women. These are: the pregnant women herself, a lactating mother who gave birth at health facility, elder mothers (those who have extensive maternal experience) and religious leaders as their role is very important. We believe that if we do all these, pregnant mothers will be happy with regard to their health.

**I:** Do you mean that the religious leaders always avail themselves during the conference? I am considering this issues as religious leaders are the most influential ones.

**P:** Yes. The meeting is conducted at village level. When they meet monthly, the meeting is accompanied and commenced by the religious leaders. If this is true, then there is lesson and advice. Therefore, since the meeting is conducted at village level, religious leaders avail themselves based on rotation system. This is acceptable by the mothers.

**I:** What efforts are in place to support mothers in home gardening? I mean what support do you give for mothers who try to cultivate vegetables in backyards of their house?

**P:** We are on work with regard to home gardening and some activities being executed at this time. One: The introduction of farming activity by farmers is increasing from time to time in those who have easy access to water. What we do is to do gardening activities at health post or health center and make it public and the example of kebelle ‘Mahber Dego’ can be taken. When mothers are invited to a visit, they see it practically and also make them taste it. We tell them that let alone cultivating vegetables in the area where there is access to water, plot of lands around health facilities is possible. This gives them lesson. But, we cannot say that we have gone a long distance in this regard. It is the activity executed in the farm areas that excels as compared to the vegetable cultivation in backyards. But, there are still gaps that need action. So, understanding the importance of home gardening, there are almost no mothers that don’t use the product of such activity. Of course, the product can be obtained from nearby markets, but it cannot be said that mothers have a full control of home gardening.

**I:** With regard to access to clean water service, person hygiene and sanitation, what has been done so far? Anything you would like to say.

**P:** Good jobs are being done to improve the issue of clean water supply of mothers, and of course of the community. Since mothers are vulnerable groups, efforts are being made to improve the access to clean water. As a government, many activities are being done for mothers to have access to clean water, especially health office and water resources are working very closely in this regard. The last two years has been the time we operated well due to the occurrence of watery diarrhea epidemic. Overall, efforts are being made to address the clean water demand of the community as a whole. Therefore, one of the actions is to prepare clean water to mothers from the nearest possible area. The other is to make at least 50% of the committee members females. Therefore, more than 50% of the committee members are females at this time. Water access has its own committee. The committee monitors the activities done such as washing its surrounding and protecting from any external factors. There are protectors. In all these activities, females are empowered to have the lion-share. The other is, as health office we have a regulatory team which assures the safety of the water, including bacteriological investigations provided we suspect that the water is contaminated. Moreover, our office treats the drinking water sources in time. It is the committee which accomplishes this task. The problem we have is with regard to adolescent girls. Since every school doesn’t have access to drinking water, we cannot say that adolescent girls have good access to clean water. The water coverage of schools is very low. Therefore, we are struggling to solve this problem. However, with regard to the other category of females, we have done good job though it is not time to declare that water coverage is completely addressed.

**I:** In your perspective, what can be said about the distribution of the water sources? For example, we have been to ‘Hatsebo’ kebelle and observed that there are hand pumped drinking water sources, one around the health post and 3 or 4 somewhere in the kebelle. It is also indicated that another adjacent kebelle to Hatsebo has no access to drinking water at nearby area. So, what can be said about the distribution water sources?

**P:** In this woreda, the distribution of water sources is the same for all kebelles if the interest of the government is considered. It is the same. The problem we have is the fact that digging and detecting water access points is not possible in some kebelles. It varies from kebelle to kebelle and accordingly the distribution is not the same. For example, if we consider our health facilities, we have 5 big health centers of which only two of them have access to water. It is tried many times, but it couldn’t be successful. We are using other option, in this case using big containers. Therefore, to improve the community’s access to clean water, many efforts are made.

**I:** What other things can you share us, for example bed net distribution for pregnant mothers.

**P:** Many things related to maternal health are done. For example, as part of malaria prevention, bed net distribution is in place. Above all, our targets are pregnant women and children. So, we are working with a big focus that makes mothers and children full beneficiaries of bed net. The anti-mosquito spray is another intervention which we are dealing with. Here, mothers are very important as they are the individuals who are close to the wall of their house, who knows how to keep the wall clean for more than six months. The other activity is the issue of blood donation. Here, we are making our female sisters to actively participate in community mobilization to donate blood as it will help mothers to improve the state of anemia which is seen occasionally in mothers. We are also mobilizing mothers to safety net program to the nearest site of their village as females sisters are the most important element for the sustainability of any program as tangible evidence from our experience tells us. Mothers participate in different activities, especially in rural development and water resources development activities.

**I:** Let us consider adolescent girls. What can you tell us about nutritional screening done for them?

**P:** There is no notable and sustainable nutritional screening activity done for adolescent girls. Activities related to health education and deworming are sometimes done for adolescent girls at woreda level. However, nutritional screening is not something which our health office focuses on at woreda level. Maybe, the screening can be done for special cases in few woredas. As a strategy, the implementation of health related interventions in schools is planned to be executed in the current Growth and Transformation Plan.

**I:** What about school feeding in adolescents?

**P:** We have no school feeding related activities in our woreda as this woreda is food secured one.

**I:** What can you tell us with regard to iron distribution?

**P:** We have that in place.

**I:** Where do you distribute the iron?

**P:** In schools. It is distributed along with Vitamin A and Mebendazol. Well, the schedule is to be done every quarter, but we are not adhering to that standard due to supply issues.

**I:** One point remaining regarding adolescents is the issue of youth friendly service. So, what can be said about this issue?

**P:** For youth friendly service, we have made 1-2 staff members from health centers to receive training and the service is currently in place in health centers. So, the awareness creation activity is done in schools and the service is made ready at health centers.

**I:** You have told me many interventions done for pregnant women, lactating mothers and adolescents. Of the interventions implemented so far, which do you think were successful and which were unsuccessful? And why?

**P:** We can say the biggest intervention done so far for mothers is the intervention done for pregnant women. The caring and advising activities done for pregnant women along with the health service packages, especially the efforts made for mothers to deliver at health facility was successful though it is not as such consistent with the plan we had.

**I:** What the main reason behind the success?

**P:** The main reason behind the success is that the community’s critical thinking is being improved. Because, the community is recognizing the benefit obtained from the successful works done and is further understanding the essence of the services being done. Of course, this is the result of the policy and the truthful government’s day and night commitment and accordingly the result is good, especially when we consider pregnant women, the result is good particularly the breast feeding practice of mothers. Their experience of breast feeding for 6 months after delivery and vaccination of children is improved. So, we can say we have done well in this regard. The issue we would say is lagging behind is the issue of adolescent girls. We have a significant number of adolescent girls in schools. It was not successful because there is no system which helps us to deal with them from top to down, not because we don’t want to work. Even we don’t know what packages are for adolescent girls. We believe that if we don’t work for the significant number of adolescent girls we have in schools, they would face difficulty of becoming the future mothers.

**I:** You told us the interventions that were successful and not successful. Your woreda is among the recognized ones in terms of food security though there are things lagging behind due to some barriers. So, what can be said about these barriers?

**P:** As I said, one of the challenges is the absence of clear packages to deal with in the case of adolescent girls. We are doing things from experience as we believe that operating in schools would have benefits. But, the steps are not clear as to where to start and where to end. The other point is that it is not only health office which should work on adolescent girls. The collaborative activity that should exist among stakeholders is also not clear. The packages to be done for farmers are clear. Only the awareness creation activities are done and the directives which could help us do other things are being missed. The other barrier is the shortage of health work force. You know there are only two health extension workers in a health center. Two health extension workers are not enough for the currently increasing number of population which is a burden for the work. This doesn’t allow you to do as per the expectations you have and should be considered while formulating the packages in the future.

**I:** What can you tell me about the human resources you have? Can it be considered a challenge?

**P:** As a woreda, the shortage of human resource is a challenge. The reason behind for this shortage of human resource is shortage of budget. The woreda is working its best to fulfill its human resource demand. However, it is found to be above its scope. Therefore, the challenge we have with regard to human capital is not a simple problem in all aspects.

**I:** With whom can you relate the shortage of trained health workers?

**P:** There are two points here. The big reason is the shortage of budget and the other point which the woreda is currently facing is the absence of trained man power. For example, laboratory and pharmacy. We have almost stopped working as a result of shortage of these professions. There is budget. The government has allocated budget for those professions but we don’t find them in the market. This is also another burden.

**I:** What special and innovative activities have your office devised to make the interventions mentioned so far very successful.

**P:** As a woreda, we don’t have a special innovation so far. But, one thing we consider as special is the existence of strong relationship between the woreda health office and the lower level management. For example, we have weekly agendas. After a week, the management at our office and health center directors will seat together and evaluate. The other big issue is that we have created an opportunity for the community to evaluate the health service activities we are doing at least ones in three months’ time. Evaluating our work and confirming whether it is going well or bad is assumed to be good for us. The other thing is the example of Mahber Dego kebelle where there is a very exemplary agricultural activity (vegetable gardening) even they use water storage containers provided there is no water. The plantation of health centers aiming them to be green is a good activity. This is a good mobilizer of the community as per our believe. Above all, we have made the community to feel and to be concerned about that the health service activities done. Any problem which occurs in our health facilities will not be hidden and will automatically reach us and we will give feedback for them immediately.

**Section 4: Community factors affecting access to maternal nutrition interventions**

**I:** The next category of questions is regarding community factors. What factors were considered relevant and affecting the nutrition related interventions of pregnant & lactating mothers and adolescent? Let us consider mothers and then to adolescents.

**P:** One of the factors is the thinking ability and understanding power of the community is not developed as required. When I say it is not well developed, the understanding of elder community members is not satisfactory in the stand they have regarding the care that should pregnant women receive. We don’t like to say we defeated it as there are remnants of bad thinkers. The other point is the shyness of the mother. She knows that she has to get everything first but she serves her children and other family members first. This exists among women in the periphery of our kebelles. With regard to adolescents, community members’ power of thinking that adolescent girls are not like children with no fully matured physical and nutritional body is low. The community doesn’t think that adolescent girls will bring children in the future. Anyway, the community doesn’t have a big concern on adolescent girls. Another point is that adolescent girls participate in activities as part of income generation. So, instead of using the money for nutrition purposes, they use it for building of houses and luxury. So, if we don’t take action here, they can face problems.

**I:** What can be said with regard to quality of care? Quality of care is founded imbedded in the concerns of the community. So, is quality of care a factor?

**P:** The quality of care our health facilities are providing cannot be a factor for mothers not to come and receive the services. Our services are usually surveyed every quarter and external bodies from civil service will evaluate it every six months. The third body is woerda congress evaluates our services every quarter and undergoes conference. We don’t have notable complaints in the health services provided to mothers and the woreda has a well-recognized performance. Therefore, I cannot say that there is a basic problem in the woreda with regard to quality of care. Overall, there is no any negative impact of the services we are providing on mothers.

**Section 5: Other interventions that influence adolescent and maternal nutrition and health outcomes.**

**I:** Let us go to the fifth point which is about birth spacing and under age marriage. What are you doing in this regard? For example let us take under age marriage share us what is being done in this woreda.

**P:** Regarding under age, we are doing well in creating the awareness of the community along with our stakeholders. We focus on previous scenarios and experiences and use it as key idea for creating awareness in the community. The other point is that under marriage is backed by the Law and accordingly there are families punished for undergoing underage marriage. This is also taken as a good example for teaching the community. In addition to this, awareness creation activity is being done on adolescent girls themselves pertaining to the harmful effect of underage marriage, whom to report if they face the problem and whom to communicate with. In all those aspects, we see that there is a change from time to time. Our office, women affairs, women development and justice are working together. Due to this collaboration, the situation of underage marriage is almost avoided.

**I:** Okay. Withregard to under age marriage, there is consistent data from the woreda we started our study up to now ~ under age marriage is almost disappearing. However, a data declared this week indicated that out of the marriage of girls in 2009 in western zone of Tigrai, 53% were underage. So, what can be said?

**P:** For the time being, I have no data. But, the evaluation of underage marriage in the woreda congress is that out of the 13 adolescent girls who faced under age marriage, the involvement of stakeholders including justice office have dissolved the problem and 2 of them realized the problem and measures were taken accordingly. So, what I want to tell you is that the community is doing such things as it does have its own belief. Anyway, the achievement is good; managing the issue of 11 of them out of 13 is good, even the under aged marriage of two of them is not something that should be taken as simple.

**I:** What is the contribution of police office in under age marriage?

**P:** When under age marriage takes place, the issue if mainly managed by justice and women affairs offices. However, police have a leading role in refining the issue pertaining to under age marriage that has taken place in the community. If a gossips are detected somewhere in the kebelle, police have a good contribution. Women affairs, justice and police offices are the leading players. If there is a need for us to be element of the issue, we will get involved. So, as I have said, if the issue of under marriage takesplace, it will initially go to the police and justice offices.

**I:** What can be said about the effect of religion on the occurrence of underage marriage?

**P:** Religious leaders have a good contribution and stages are prepared for them to have their ideas and thoughts. Therefore, we don’t have the opportunity to say that religious leaders are acting as barriers rather they are afraid of the law and refrain themselves from such issues and sometimes are very supportive as they give their advice to adolescent girls to continue learning. Repeated surveys were done and even representatives from regional bureau have confirmed that religious leaders have no pressure to apply on under age marriage. However, there are some religious leaders who act as if they don’t know though they are well aware. They say ‘I was out of the kebelle’, ‘it has occurred because I was not around to apply pressure on the family’, etc. this is not gambling.

**I:** Let us go to birth spacing. To develop the spirit of birth spacing, what interventions are done by your health office?

**P:** The issue of family planning is assumed to be among the main activities that can result in good outcome in maternal and child health. Its essence is evaluated at woreda level. Therefore, one thing which we considered is best in this woreda is that family planning does have multiple options. From these options, the community’s preference of long term family planning methods is upgraded from time to time. More than 60% percent of mothers use long term contraceptives including ICD and Permanent. This is a good achievement. But, when we compare it to our plan, we understand that there are still gaps that need to be filled. Of course, there are mothers who experienced unwanted pregnancy as opposed to the policy in place and this is the area which we have to work on in the future. Otherwise, the progress is good as far as the process is well going, especially with regard to trained man power, supply, critical thinking and having sense of ownership.

**I:** With regard to family planning, the data that I obtained has indicated that males are also good actors. There are males who said ‘enough’ and passed the decision to use permanent family planning methods. To what extent is this real?

**P:** With regard to family planning, many of the users are mothers. But, these days males are also putting some considerations and started thinking how they could help their female counterparts as the options are being made open to all categories. If the approach of family planning is not good to mothers and if giving birth poses a threat to mothers, then males decision to use permanent method of family planning method is increasing from time to time. Of course, a long distance is not travelled, but the awareness developed is enough. Most of the time, mothers are the users of permanent family planning methods. When we take the coverage of the woreda, around 68% are permanent family planning users, considering this month. Of the total coverage of 70%, around 68% are long term family planning methods. Anyway, the progress is going good.

**I:** The data I have is that those that used permanent family planning methods are clergy men. Thus, what have been done for those clergy men to pass the decision?

**P:** When we start to introduce the program of family planning, we started from them, i.e., clergy men**.**  We started to communicate with them because family planning was treated as a depraved activity. Even the community believes that something not approved by religious leaders is a sin and therefore we started the job from them. How should a mother/family control the growth of a child? Is it in the bible to give birth a baby that you don’t give care to? If you don’t give care, is it not giving birth that outweighs or giving birth and passing through suffers? Which one is better? Which one brings you peace and sin? All these points were raised and common understanding was taken. So many efforts were made, there were arguments many times. The influence of religion is very big. Therefore, since we believed that the role of religious leaders is essential, we started from them and after they are convinced that they issue brings about benefits to themselves *(themselves is to mean religious leaders).* It was challenging. It was due to the fact that religious leaders are convinced that the intervention of family planning was successful. Church men started to be exemplary. Therefore, our plan to make religious leaders beneficiaries and otherwise not to act as barriers has been achieved.

**I:** It is really nice to see such kind of achievement as this is one that I didn’t see in other areas. So, how did you run the activities that helped you convince religious leaders?

**P:** For this issue, we have conducted woreda level conference of religious leaders. A conference was held in the presence of community elders and representatives of religious leaders. As a continuation of this, conference conducted at the lower level management assisted by those who got orientation at the first conference. Now, religious leaders didn’t act as barriers when women development armies start persuading mothers to give birth at health facility. The main problem was that there was a debate in that using family planning methods is an action that makes you sinful or not. So, when we come to consensus at woreda level and then at cluster level where evaluations are done every quarter.

**I:** What can be done to prevent under age marriage and to enhance optimum birth spacing?

**P:** One: as I said, the information about under age marriage’s consequences should be disseminated to the community, especially awareness creation activities should be done at school levels. Two: prevention of underage marriage and enhancement of birth spacing is not something that needs the commitment of health office only. The collaboration among stakeholders should be considered, especially with education office, women association, women affairs, justice and police office. Even the participation of security personnel and Militia should be considered. The other task that should be done is to convince priest that under age marriage is law backed and therefore committing error will result in imprisonment and further punishment. The distribution of lesson carrying materials to the community as the number of community members who don’t read is low. Educating the networked community is also another intervention; especially focus should be given to female networks because it is the female which suffers from the consequence of underage marriage. With regard to family planning services, we should do additional tasks along with the stakeholders mentioned so far and we have to work to upgrade the convincing power of our health professionals. Moreover, the supply issues should be complete. If we do these, we can excel. The involvement of other stakeholders in funding is also important in addition to the support from the government. There are NGOs which can support in terms of coverage and quality. So, creating a sort of opportunity for those NGOs to apply their contribution is important.

**Section 6: Multi-sectorial collaboration to improve maternal nutrition**

**I:** Do you think that multi-sectorial collaboration is important to improve the implementation nutritional interventions?

**P:** The essence of collaboration of nutritional interventions that exists among stakeholders is unquestionable. It is not the health office which can accomplish all the tasks as per the expectations of the government. The health can only deal with some of the agendas, especially when we think of improving the diet taking habit and bringing behavioral change of the community, it should be acted though the tactics of politics. It is not professional work only; it is also a political agenda. For example, when you take someone who has a big wealth and a lot of money, there are times that that person wears bad clothes. This is due to poverty and this poverty is not something related to wealth; it is related to the way that person thinks. So, if every concerned body does what he/she can, especially agriculture and rural development, water resources office, women networks can contribute a lot. When all those bodies mentioned come together and work as a team, the impact will be big. Thus, collaboration is a very important thing and is option less.

**I:** For all the sectors including the health office to work together and bring about a better and productive outcome, what support do you think will they need?

**P:** When we think of the collaboration and the support needed, there will not be a difference. But, when we think of the outcome of working together, there should be the need for benchmarking of the experience of other areas where collaborative work among stakeholders have been practical. The other point is there should be a system. The systems should be adjusted to work collaboratively. When the health office is in danger, the office should not push stakeholders and say please let us do this this and this. A system should be in place that brings all stakeholders to the same point and feeling. In most instances, we have the problem of not undergoing things through a system. When we face problems, we say ‘what shall we do?’ But, before things happen, what should be done? What should we do to prevent the problem from occurring? There should be a system in place that answers all these things that keeps all things going starting from the top level management.

**I:** Okay. What lessons did you get from the nutrition interventions that have been done and you told me so far?

**P:** When we consider what we have done so far, the community is benefiting as the diet utilization behavior and habit of the community has been improved. Previously, the community had the habit of bringing valuable products to the market, but this is changed right this time. Especially the behavioral change that occurred among pregnant, lactating women and adolescents has been good. This is a big achievement. We consider it as a big achievement. Previously, better foods had been given to males. That was the way the community was thinking. But, this way of thinking is disappearing. The community has started to think that better and delicious foods are also important for mothers and the children. This by itself is a big achievement. The other point is the upper level management that is still working on nutrition is the health office. The share of health in nutrition is 20%. However, if think that the other sectors are managing the 80%, it is questionable. Therefore, when we consider the involvement of health in nutrition, it is improving from time to time as health has close relation with nutrition. This is a good thing. The relationship that exists among sectors, though it is not complete, like the relationship that exists between health and agriculture and women network and agriculture is very good. Furthermore, the other thing which could make us successful is our consideration of religious leaders in every aspect of the activity we are doing, like the nutritional programs we have and others. This has brought a pleasant change. Above all, we would like to thank the support we have got from our government or the regional health bureau during the implementation of the nutrition interventions.

**I:** What lesson has dropped you the multi-sectorial collaboration that existed in your woreda?

**P:** The lesson we picked from multi-sectorial collaboration could be important for us in the future is that the collaborative activity that exists between us and the water resource office in assuring the community to have access to drinking water is being improved from day to day and this has given us a huge lesson. When we come to agriculture, the fact that women are exempted from labour related duty and are benefiting from the interventions has pleased us and is a good success. Furthermore, our woreda’s congress evaluation of the maternal and adolescent services and telling us our weakness has been a good success.

**I:** With regard to sectorial collaboration, we know and are saying that adolescent girls are the future mothers on one hand and we say also they are the neglected segment of the community. This is an issue of the globe. You know women affairs office can put a big pressure in the issue of adolescent girls. So, to what extent do you collaborate with women affairs office?

**P:** This is a question which is frequently evaluated by concerned bodies. For women’s issue, we don’t act like women affairs office. We act if we believe that the job we will do is helpful for us, we really act. The network and accountability of women is to the women affairs office. Therefore, things are not going as expected. There are gaps left. If we encounter good and bad things while working for the maternal and adolescent nutrition, we seat together with women affairs office and discuss. Then, if there is something that requires their involvement, we request them to act. But, things are not stretched to the maximum in this regard. In adolescents, we have taken the lesson that a big activity is ahead of us. Therefore, if there is a concerned body that directs the things that should be improved, we have understood that a long distance cannot be travelled. Otherwise, it is frequently evaluated. To your surprise, there has been a committee from regional women affairs office which had the mission of evaluating the relationship we have women affairs office. We had been evaluating what you raised about right now word by word. Word by word. Therefore, there is a gap here. We have shortcoming in acting on the gaps we have.

**I:** What opportunities does this woreda have in supporting the maternal and adolescent nutriton?

**P:** To accomplish the tasks we have as a woreda, we have a committed administration. When we think as a government, we can say that we have an interested administration. The other is the association of women. They established the association to keep their benefits alive. The women association and women development army are a working for the benefit of women. This is a big opportunity if we utilize it. The fact that women, especially adolescent girls are in the line of education is another opportunity because instead of teaching them by going home to home, you will find them at school. In addition, if we work on female teachers, we can work on adolescent girls as well. This is another opportunity. A very good opportunity. The other is that we have health post in every kebelle and as per the standard; we have achieved the access to health services in each health center. Finally, as mothers and adolescents are parts of the community, we have a strong woreda congress that controls and evaluates the conditions of mothers, whether they are getting benefits from what they have to get. A very strong congress at woreda and at kebelle level.

**I:** Why do we call the congress ‘strong’?

**P:** Today, I ignored you to contact the congress as they were here to assess the progress of health. There was a conference a month ago and they were here to know whether the assignments we took from the conference are going well or not. Therefore, they congress is working to assure that the activity we are doing is bringing benefits to the community. Fortunately, I have noted all the points they raised *[key informant indicating the note book where he wrote the points asked by the congress].* For example, how do you narrow the gap of ANC utilization in the first and fourth ANCs? There is a low utilization in the fourth and higher in the first. Why? What did you do so far? In postpartum service, what are you doing? In order mothers not to deliver at home, what are doing? HIV is said to be increasing in Tigrai, what are you doing, then? How is the progress of quality of care sectorial collaboration? Etc. they have brought a lot of questions. Therefore, the congress is very strong. It doesn’t give you the time to easily skip things. This is a big opportunity. The fact that Tigrai regional health bureau is enhancing our relation with stakeholders like NGOs, Mekelle University and Aksum University to support us and conduct such researches which could show us the gaps. This is another opportunity. Above all, the government and the policy in place are facilitating the implementation of the different tasks in health which is another opportunity.

**I:** In the collaboration of sectors, what opportunities are in this woreda for all the sectors like the NGOs, the health office and other to work effectively?

**P:** The big opportunity is that as a government, the presence of the thinking that ‘multi-sectorial collaboration is important’ is one opportunity. There is a committee which ties all the sectors to work collaboratively. The committee gives all sectors an assignment based on the plan and all are evaluated depending on the performance they showed by the committee. Then all is ranked. Therefore, the benefit of working together is not blurred to all sectors. The problem is that sectors incline to their own activity. So, the collaborative activity is already structured. However, it is not working as a system and we should adjust that. So, the presence of the committee is an opportunity for us. If we have faced a problem, this committee discusses and brings the solution. The committee requests what should be done to solve the problem.

**I:** To whom is the committee accountable to?

**P:** It is accountable to women affairs office. Therefore, health is considered as one member of the committee.

**I:** I have finalized my questions. If there is anything you would like to convey, you are welcome. Otherwise, I have finalized.

**P:** I have nothing to add. The assessment you made has dropped us a lesson. The questions raised have conveyed to us a lesson and based on the question raised; we have understood where to give focus. Therefore, it helps for our work. In the future, the outcome will bring us a benefit to our woreda. I have finished. Thank you.

**I:** Thank you

**Summary**

- As per the key informant’s information, the recognized nutrition related problem in the woreda is anemia though the disease is not as such common.
- The key informant has consistently pointed out that empowering villagers to stick to vegetable farm is among the main activities the woreda is supporting.
- There are no sustained nutrition screening programs for mothers and adolescent girls in the woreda as the economic activities like farming are bringing a good impact on the lives of the people.
- Community’s lack of awareness and low level of understanding are common barriers in the implementation of nutrition interventions.
- Interventions on adolescent girls are almost none as the nutrition packages of adolescent girls are not clear.
- The key informant repeatedly indicated that the nutrition intervention focused multi-sectorial collaboration that involves different stakeholders is not as such effective as there is no system for its management.

------ The end -----

.
