# Supplementary material for: Barriers to utilize nutrition interventions among lactating women in rural communities of Tigray, northern Ethiopia: An exploratory study
Source: PLoS One. 2021 Apr 30;16(4):e0250696. doi: 10.1371/journal.pone.0250696 (PMC8087028; doi:10.1371/journal.pone.0250696)
Supplement: S2 File — (ZIP) [file pone.0250696.s002.zip › S2_File.Doc/Woreda level and above key informants/126_IDI_head of Women affaries office_Laelay Maychew woreda.docx]

**Operational Research on Adolescent and Maternal Nutrition in Northern Ethiopia**

## **Tool A**

## **IN-DEPTH INTERVIEW GUIDE,**

## **Lealy Michew Woreda women affairs**

Hello, my name is Omer Seid. I am from Mekelle University. Thank you for taking the time to speak with me today. We are doing research on the factors that influence the nutrition of mothers and adolescents in collaboration with the Regional Health Bureau and UNICEF. Your participation is very valuable. The things that you tell us will be used to improve nutrition programs and services for women in the region and the country. We will not share your names when we report our results.

Do you agree to participate in this study? YES

| **Section A: Interview details** | |
| --- | --- |
| **Questions** | **Answer** |
| Zone | Central Tigray |
| Woreda | Lealay Michew |
| Kebele | Axum |
| Name of key informant | Letebrahan Berehe |
| Institution of key informant | Lealay michew women affairs |
| Interviewer name | Omer Seid |
| Date of interview | 20,November 2017 |
| Interview start time | 9:34 AM |
| Interview end time: | 11:41 AM |

| **Section B: Interviewee professional information** | |
| --- | --- |
| **Questions** | **Answer** |
| Gender | Female |
| Age in year | 29 years |
| Highest level of completed education. | Bachelor Degree |
| Current job/position | Head, Lealay Michew women affairs |
| How long have you been in the current job/position: | 6 Months |

**Section 1: Common maternal (pregnant women, lactating women and adolescent girls) nutrition problems in the community.**

**I:** In your opinion, what are the common nutrition problems in the community for women (pregnant and lactating)? What about for adolescent girls?

P: Ok, to explain about these three (pregnant, lactating and adolescents) as a woreda, the last six months I served in this office as head and even before here, in the last four years during I worked as Vice leader, as women, we leader has been evaluating women issue. During evaluating the issue of women’s, particularly we evaluated the pregnant women; what sort of food recommended for pregnant women’s and what sort of foods are not recommended for pregnant mother and then in this area we did give education for pregnant mothers jointly with the health sector. There were different problems on feeding, even on feeding practice of lactating mother and adolescent girls but now there is improvement progress. When I say this, before now a pregnant mother feeds, and drinks like alcohol and its likes, but nowadays it is changed. When i say alcohol like beer, pregnant mother had been drinking it before, but nowadays it is not allowed. Women have getting education at the health institution that means women have getting awareness about feeding. There is improvements on feeding but not totally removed the feeding problems.

**I**: Is there any pregnant, lactating and adolescent girls have supplement of plump nut or FAFA in this woreda?

P: The woreda has 16 kebelles, and last time there was supplementation but not currently. Time to time there is an improvement on pregnant mother, lactating mother and the children, so no supplementation of FAFA and plump nuts. Measuring the child and the measurement of children indicated it is healthy so no needs of supplement pump nuts and FAFA. The education by itself has good outcome results, because of this the child born with good things so not problems. No problem on pregnant and lactating mother.

I: Does micronutrient deficiencies (such as anemia, night blindness, goiter) are problems for pregnant, lactating mother and adolescent girls?

P: Anemia is problem in this woreda women’s. But our blood donation sates is good, any person having greater than 45 kg in weight and checked, and has good health states should donate blood. So in the 16 kebelle we have traditional and up-to-date ambulances so that pregnant women’s that lived in the sixteen kebelle will use during delivery. The community of these 16 kebelles knows the importance of blood donations, and they have practicing it voluntary also.

I: What about night blinds problem among womes?

P: About night blindness locally known as “*Hemia” every* person has taking vitamin A supplementation. Even for children’s, if I am not mistaken up to five years childen, there is droplet intake of vitamin A in every six menthes interval. Thus, night blindness situation of the previous time is not found now, it is possible to say it is totally ending. It is removed because the health institution has doing very nice jobs. Beyond any other sector, the health institution has doing theoretical and practical activities and health sector is the first by this. If all other institution do like health institution it will be very good, because the health institution has doing fruitful jobs for life. So in our woreda it is possible to say night blindness is not the problem of pregnant and lactating women and adolescent girls.

I: What about anemia among lactate women’s and adolescent girls?

P: About it I don’t have idea. Goiter I don’t know goiter cases in our woreda.

I: Does women of this woreda are stunting (low height for their age)? What about Underweight (low weight for their age)?

P: What do mean thins to weigh? And stunting? Is that like me? and she is Laughing.

I: I explain stunting and underweight interims of age?

P: It is short and thin. Physically there are thin and short women’s in the woreda, but relatively the children borne form this mother are quite different from them, because by the education of the health institution the dietary intake of women’s during pre-pregnancy and pregnancy is improved, so these thin and short women’s has not giving children like them (short and thin), rather they are giving very nice beautiful children. I can take myself as the witness.. with laughing

I: let me ask you diet related non communicable diseases like DM, hypertension and cancer among pregnant, lactating and adolescent girls?

P: DM and hypertension cases are not common in our kebelless, but when I see in the town, practically DM affected majority of the people nowadays and I don’t know its reason. Me, myself has tried to check from the Facebook, and have got the causes and the privation of DM, but, once it happened it doesn’t have medication, thus there are many affected women’s by DM. In our rural kebelle, the problem is anemia but not hypertension.

I: What about the food insecurity situation of the woreda among pregnant and lactating women’s and adolescent girls?

P: Ok, when we see the food security situation of the woreda, it is possible to say the woreda is rich by food availability. In the sixteen kebelle the main product is Teff and previously around two or three kebelle had produces sorghum and wheat but now they turned to produce Teff starts from the last two year. The food intake states is good, it is common that every person takes three meals per a day’s. So, have no food shortage in our woreda.

I: Which women groups are most affected by these nutrition problems?

P: It means nutrition?

I: Yes

P: Pregnant mother. Do you need the reason?

I: Yes

P: She will not eat the available food because during pregnancy you will dislike all foods. There are food that you dislikes and the also foods that you likes. So mainly pregnant mother are affected groups but, other women can eat any food they gets so it is not the problem for other. Lactating mother will not affect like pregnant mother. Pregnant mother are more affected groups but lactating mother and adolescents are good. During 2-5 month pregnancy of there are many health obstacles, like nauseating, headaches and hating to take foods. Hating to eat food is not 100%, rather it is fifty, fifty which means there are sort of food that she like to eat, on the other side there are sort of foods that she hates to eat also. The problem during pregnancy is you will not eat all food that you have, as a result the pregnant mother will be affected.

**Section 2: Nutrition priorities in the woreda**

I: Do you think it is necessary for your institution to get involved in work aimed at improving maternal nutrition? Explore for pregnant women, lactating women and adolescent girls.

P: Yes

I: Why?

P: To improve the nutrition of this three (the pregnant and lactating women and adolescent girls), the women affairs office should involve; because it is women issue and woman affaires office is for women. And women are affected groups, because if the pregnant mothers are affected or if one woman is affected the whole community will be affected.

I: What maternal nutrition (pregnant, lactating and adolescent girls) interventions are the priorities in this woreda?

P: Yes, related to these three, specifically for pregnant and lactating women and adolescent girls, we are intervening in group jointly with other sectors. In our woreda we have steering committee (the group) that contains nine members but has eleven members if go down to keblle level which is to work on women’s nutrition’s. The steering committee has doing effectively on maternal nutrition. Particularly in the woreda there are 1294 women developmental army’s and each woman has network with the women developmental group. Through the network the women developmental group has educating the pregnant women about taking rest and about feedings. Starting from the wored to kebelle this women developmental group has linkages networks. Regarding to adolescents in particularly between 7-18 years age in the sixteen kebelle we are working to prevent school dropout through using the women developmental group. Regarding to this from sixteen kebelle, four kebelle is mine, in this woreda women affairs office we are four female staffs so each staff has get four kebelles which means we shared the sixteen kebelle to four staffs. In these four kebelle each of us has doing the prevention of school dropout of young females and doing follow ups to check the situation of pregnant and lactating women and children also.

I: How do you check the pregnant women, lactating women situation?

P: By going in to the community. If we get a report from the women developmental group that the community is not accepting their education, we directly do go in to the community together with the other steering committee members like health sectors, because our education is different from the health institution education and the main target explanation will be given by the health sectors. In the sixteen kebelle the health sector has going with us to do education eg; last year in Medego kebelle seven mother has gave birth at their home, which is the first in the history of our woreda, then we did discussion with the women developmental group to identify the reason why mother do home delivery. The reason we got from the women group, which is because of low awareness. Then together with the health sector we going into the community and did discussion, and their reason was they are not comfortable by the woreda so that had declined to use health facility to give birth. In this case by bringing the woreda officials we did discussion with the community and have solved the problem for the future. Thus, to improve the nutrition of pregnant and lactating women’s and adolescent girls we are working by group, and this group has found starting from woreda to kebelle level. This group has network strong starting from woreda to kebelle level so using this network we do education. Mainly we give education and then evaluate the change. There is improvement on women feeding and feeding of the community in general though the women’s education. The community has enough understanding for women’s nutrition so there is an improvement currently.

I: What maternal nutrition (pregnant, lactating and adolescent girls) interventions are the priorities in this woreda?

P: We give priority of pregnant mother. Mainly we do interventions for pregnant mothers because it is life. We are not doing equally for the three groups (pregnant, lactating and adolescents). We do give strong care for pregnant mother, we do visit her at her home and also we evaluate the women developmental group activities for pregnant mothers. We do assess the husband support for pregnant mother and her pregnancy follow ups by the health institution. However we are not doing for lactating and adolescent equally with pregnant mothers. All are not equal, so we give priority of pregnant mother.

I: Can you tell me some of the successful maternal nutrition interventions that you have implemented in this woreda?

P: Primary we are focusing on pregnant mother’s nutrition, so we are not doing like pregnant mother for lactating mothers and adolescent girls. We are not doing strong for lactating mother and adolescent girls but I would be done.

**Section 3: Nutrition interventions that improve adolescent and maternal health**

I: What kinds of nutrition interventions are in place to improve adolescent and maternal health in this woreda?

I: We give education about good eating’s for pregnant and lactating women’s and adolescent girls in the sixteen kebelles. Me Letebirahan only will not cover all sixteen kebelles, we women affairs are four in staffs, so we all staffs has going in to the community together with the health sector which is done programmatically then give educations. What we did is giving general introductions to the community and the health sector will give detail explanation about women’s nutrition. So together with the health sector we give education for women developmental groups , then after the women developmental groups will give the education for women’s by gathering . For this, there are 502 women developmental groups in this woreda, we give education for the women developmental group, and then they accepted and transfer the education messages to women’s of their kebelles. Then after, we did evaluate the change achieved due to the education we give. If there is no change we give the education again. To educate the community we use also best experiences, if one women developmental group has best experience we used it to educate for other groups or communities. The community or the women developmental group will achieve good change by understanding the best experience. For your information in our woreda or kebelle, there is farmer’s developmental group, youth’s developmental group and women’s development group. Of these all the women developmental group is the best performer and the model. There are some incentives, for example last year the federal government had allocated budget for youth’s for job creation, and from this finance adolescent girls were the primary beneficiaries which is because of the women developmental group work. Our role is showing the women developmental group, so this women group is the main performer of the task practically at the ground level.

I: Can you tell me the specific activities you have doing for pregnant, lactating and adolescent girls in you woreda?

P: Yes, about pregnancy and post pregnancy follow-ups, regarding to their nutrition, I already mentioned above. The women developmental group is the main actor of doing activities by making mother to do follow-ups during pregnancy and post pregnancy of women’s. In the sixteen kebelle this women group has working on identifying and linking of pregnant and lactating mother with health facilities for follow ups. First estimated calculation has made for pregnant and lactating mothers then distributing the estimated calculation to the sixteen kebelles, so now it is known the number of pregnant and lactating mothers that are found in each of the sixteen kebelles. The health worker of the kebelle together with the women developmental group has working to bring these mothers for follow-ups during pregnancy and post pregnancy.

I: Good, what kind of salt you advise for mother?

P: Iodine salt (iodized salt she said), and we educate mother about how to keep hygiene of her child. Iodine salt has many befits; to prevent goiter the pregnant mother should use this iodized salts. In the sixteen kebelle before now the community uses none iodized salt that comes from Afar region but currently now the community turns to use the iodine salt. It is not only for mother the whole community stared to use iodized salts. Mainly the health institutions do give education for the community about the importance of iodized salts coordinated with us. We don’t have detail like health workers to educate the community about the importance of iodized salts.

I: Any other intervention?

P: In general the education given for pregnant mother is about all cares needed for pregnant mothers. May be my language is not clear for you, what I am saying is jointly with the health institution we do give all education that is necessary for pregnant mothers.

I: It is clear dear Letish, my intention is to knowing all each intervention that you are doing for pregnant, lactating and adolescent girls. For example are you educating mother about deworming?

P: What is deworming?

I: I explain about deworming’s?

P: During pregnancy there is TT vaccination. Generally we give education about all things that are necessary for pregnant mothers.

I: Can you tell me the feeding advice that you are giving for pregnant and lactating mother?

P: Pregnant mother should eat four meals per day and lactating mother should eat also four meals per day.

I: In your opinion, which of the above programs are being implemented successfully (i.e. in the most effective way?) Why? Explore for pregnant women, lactating women and adolescent girls.

P: Simple exercise is very important for pregnant mother, like waking by foot for short distances, doing some simple work in the house. It is important because the mother will not be stressed during delivery. So during our education we educate mother to do simple exercise during the morning and in the evenings. We advise, between 12:00 AM to 2:00PM the pregnant mother should take rest, and we advise to do simple exercise in the morning between 7:00 AM -9:00 AM and in the evening between 5:PM -6:PM which is important to reduce the damage and pain that has occurring during delivery.

I: In your opinion, which of the above programs are being implemented successfully for lactating mother (i.e. in the most effective way?) Why?

P: It is the same for lactating mothers, like that of pregnant mothers she should take rest because important for the content of the breast milk. For lactating mother care should be given like pregnant mothers, if she is loaded by work, and haven’t rest her breast milk content will be reduced and will also stop even. But practically lactating mother has not getting care like that of pregnant mothers.

I: In your opinion, which of the above programs are being implemented successfully for adolescent (i.e. in the most effective way?) Why?

P: Adolescents are next mother, thus adolescents should learn about nutrition to prevent stunting and thinness. Adolescent nutrition is not like nutrition of women’s around 30 years’ age, adolescent nutrition is more important, because it is adolescent age even marriage is not simple or it is challenging life. During educating women and adolescents, adolescent easily accepts the education message but women’s do not because women will not have concentration like adolescents, women will be destructed by thinking about issue that she has left at homes. Adolescent don’t have destruction by thinking home related issues. So adolescent should learn nutrition because they are the next mother.

I: That is very nice Letish, my question was, in your opinion, which of the above programs are being implemented successfully (i.e. in the most effective way?) Why? Explore for pregnant women, lactating women and adolescent girls.

P: We did the successful job on pregnant mother because the illness related to pregnancy and during delivery is reduced. The successful work has been done on maternal nutrition and resting if we see in kebelle. The next good job has done adolescent girls, and the third good job we did on lactating mothers. For lactating mother by considering her child risks in nutrition we are not doing good jobs like that pregnant mother. Thus, when I see and explain interims of the community we did very nice job on pregnant mother’s nutrition.

I: Of all intervention of pregnant mother which is successful intervention?

P: I think exercise is simple for rural community, but bout dietary intake/nutrition there is still the problem. Thus, doing simple experience is simple for pregnant mother and has change on it, regarding nutrition there is good progress but still it is not good like doing simple exercise.

I: In your opinion, which of the programs mentioned above are less effective? Why? Explore for pregnant women, lactating women and adolescent girls.

P: Less effective was dietary intake of mothers. Mother has doing four ANC but has weak in dietary intake, by the evaluation we did it is identified as gab, and we planned to work on it in the future. The steering committee approved this gap also, that the feeding practice achievement is not comparable to the effort we did to educate the community.

I: What is the reason for it?

P: The imputes are not found in some kebelle like vegetables

I: What about successful program for lactating mother?

P: Dietary intake is good, successful because for the purpose of improving breast milk production lactating mother takes better foods compared to pregnant.

**Section 4:** **Implementation challenges and** **Community factors affecting access to nutrition interventions**

I: Can you think of barriers that prevent adolescents and women from using the programs and interventions that we have discussed? Explore for adolescent girls, pregnant women and lactating women.

I: Does the literate and the illiterate mother has used equality the available services?

P: No, not use equally, the literate and the illiterate person is not the same

I: Who will use better? Why?

P: The educated one uses better. Regarding this as I already mentioned above, the literate and the illiterate person is not equal eg. I and my mother are not the same, because during my education I learn about health, I learn about the surrounding area , I learn about feeding and others, but my mother did now this and to educate me she can’t did her education and she didn’t take any education. Thus, am better than her because I learned it theoretically and in my life I have doing it practically.

I: What about awareness of women and adolescent girls?

P: The women that have awareness can use the service better than none aware women are because the aware women know the importance and the consequence then she will go to use services but if the women don’t know the importance and the consequence the service she will not going to use. That is way we are giving education women’s by the establishing steering committee, by using women developmental group and the networks and others. Our aim is by creating awareness bringing mothers to good nutrition’s. So it is the main barrier.

I: What community related beliefs and norms are preventing access to the interventions? How?

P: In our sixteen kebelle, no community perception barriers at all currently. Before now there are preemptions even schooling allowed only for males, but now it is changed and also allowed for females. Before now only the family has the mandated body for selecting for the husband of the female, but now she has doing to select her husband. Now females are equal with males, she has equally participated with males in every actively. Females should come first, because she is development of the nation, she is development of the house and she is the candle light of the house and this is the slogan for improving females. So there is change, no community perception barrier currently.

I: Are the interventions culturally acceptable? Why and why not?

P: Aha, about it I need to speak. Among pregnant mother there is improvement, but in schools there is something injected by needles for students and female students are not interested to take the injection because they perceived it is contraceptive so they did leave form school and running away for not taking the injection. So still there is problem. For pregnant it is good but there is some problems like taking of yogurt and pea is not recommended during pregnancy because the child will get bigger in the mothers uterus, then it will be difficult to born.

I: Are the interventions accessible to the women and adolescents? Probe in terms of transportation and cost?

P: In the sixteen keblle there is equipped health facility and all miner cases are managed by the health institution that are established in each keblle, and for major cases they will not managed and will do refer it. So the community has getting well services. Transportation, we have a keblle named Awule, this kebelle is a afar of all sixteen keblles , it has health facility, it has school and has all service but don’t have ambulance. If a woman has referred from this kebelle, there is no ambulance to come here (Axum), and in all kebelle there is road but not quality road, difficult for transportation of mother using ambulance, rods are not comfortable for pregnant mother.

I: How convenience is interventions to the women and the adolescent girls?

P: No problem relating to this.

I: How do you explain the quality of the interventions?

P: The service is good and no problem related to the quality

I: What resources exist to provide the interventions and what do not exist?

P: Regarding to this, as a women affairs office, it is known we have sixteen kebelles, we use public transportation to going these kebelles and sometimes we beg and used also other offices vehicle. Most of the time we use telephone calls to follow the activities of all sixteen kebelles, because of transportation problem. The community attitude towards women has good progress eg: regarding to this, the point that I need explain about transport which is the main thing, only the Mekelle head office of women affairs has transportation, but primarily transport should be given for women offices, become as I told you women’s are mothers, women’s are sister, and women are candles lights of the house. So, if there is unfairness the work will not do appropriately. Health institution can educate about main nutrition related things, but we are appropriate to educate woman about modus utilizations, but if we need do this we don’t have transportation to going in to community. So what I did is in most of the time using telephones instead of going there. This transportation problem is not only for our woreda, as region all Tigray region women affairs offices have transportation problem. The women office should get attention equally with other sector like health, water and other.

I: How do you evaluate the commitment of the intervention providers at your level?

P: Regarding this, most of the time it is good. When we changed our responsibility in to practice most of the time it is good. However, sometimes by some health care givers eg: adolescents may get pregnancy; I have seen many things related to this during my university study, adolescent girl going in to the health facility for abortion care at this time we should keep the psychology of the girl. If the health care provider is shouting on her instead of helping then the adolescent girls will do suicide. Regarding to this I have seen many things. In our sixteen kebelle it is good regarding to this because I did assessment and found nice. If we see psychologically, male health care provider will give better care for female clients and female health care provider will give better care for male clients. But if female’s health care provider is for female clients, there will be grandercity like behavior. So we should consider the psychology of the clients, while giving care. This issue needs further work in the future and we should do.

I: What other factors are inhibiting implementation of the interventions? how?

P: About duty workers for health professionals, some do sleep the entire night and other may work the entire night so the payment should be performance based.

I: For these challenges that you mentioned, can you tell me of any solutions that your institution have applied to effectively implement the interventions for women and adolescent girls? Specify the each solution done for each challenges?

P: Psychologically we should approach professionals to improve their commitment. Punishment is not a solution here, we should approached psychologically then tell them their mistakes, then they will improve it. My myself, I personally need money and will satisfy, but I changed this mentality and I will satisfy if the community satisfied by the serviced that I give. So we should approach the works and helping them to think and do like this.

I: What about other challenges/

P: Regarding to the transportation our country economy has growing, the transportation problem will be solved in the near futures. The country has striving to reach middle income countries and the meantime this problem be solved. About the education, the government has doing many things to improve education. Through the education office many things are done to improve education. But still there is no enough education chance for offices like us; per year, one or two chances, which is not enough. About universities should use own citizen, should stop to recruiting expatriates by three or four fold salary of impetrates for one expatriates. As result we should educate ourselves, the community should get the education important at their level. Everybody should get education; the educated person can get knowledge and this person will have change on thinking’s.

**Section 5: Other interventions that influence adolescent and maternal nutrition and health outcomes**

I: In your opinion, why would delayed marriage (after 18 years) improve maternal nutrition?

P: Early marriages is harmful for females

I: Why?

P: Regarding it, there will be uterus infections if the marriage is early, there will be major problems like fistula and raptured uterus and beyond this she will be affected psychological. Early marriage is not good for marriage life, no love and it is like dark. Her life will be dark because not love, so early marriage is harmful.

I: Can you tell me about any programs or policies in place in this woreda to prevent early marriage?

P: By understanding its harmful effect in our woreda, when we evaluated our one year activity we prevented 11 female early marriages coordinately with education office, women developmental groups. We bring all this females to schooling by discontinuing their marriage. So, currently strong effort has underdoing to prevent early marriage in our wored. Even beyond terminating the marriage we asked the families of these 11 students by their trial. So if we see our woreda and kebelle, the community has not doing early marriages but sometimes if we get one or two early marriage trial, we have strong coordination then we will interpret the marriage.

I: In your opinion, are these programs or policies effective? Why or why not?

P: It is very effective, eg. Those 11 females are enrolled in schooling, by terminating the marriage. If they going get marriage, there will b faced different problems, she will dropout out from schooling, she will face different psychological problem. By considering these things we are working to prevent early marriage in our woreda and kebelle.

I: Can you think of any other opportunities to prevent early marriage? For example, building awareness at schools

P: Regarding this as I mentioned above the problems like uterus related problems and psychological related problem and other marriage life problems of early marriage, so education been giving in schools on these topics. There is one selected female school teachers in each of the 47 schools of the woreda and this school teacher has selected by us for this purpose, then every two week she educate the students about the problem of early marriage. Thus, early marriage is around ending in our woreda.

I: Can you think of any more programs or policies? Think about political, religious and other influences.

P: Religious leader, the *kese* and the *shek* already accepted and have working to stop early marriages. This regions leader beyond giving the religious aspect of Bible or Quran, they are also giving such things for the community to stop early marriages. About the politics, it is already known and no need to discuss because has doing strong efforts already.

I: Why the community needs to do early marriage?

P: Because the community perception is not changed, especially in rural area the family perceived if the adolescent girls see/going in to town she will have boyfriend which is unethical, shame for the family *“Tawardenalech”*. Thus, for females after grade eight majority of the community decided to do early marriages.

I: In your opinion, why would increase the space between each birth improve maternal nutrition?

P: Increasing birth interval is important because it is economical, the child will get enough care, clothes and feedings.

I: What is the importance of increasing birth interval for the mother?

P: She will be burdened, her body will be affected, and giving one child per three year or two children per three is not the same, she will not have enough power for giving birth. So majority of the community knows it very well.

I: What programs or activities promote increasing birth intervals in this level?

P: Yes there are activities to increase birth intervals, before now the mother had get the contraceptive after gaining three or four hours walking, but now it is available in each kebelle in their nearby with five or twenty munities she can collect. But the point I need speak about contraceptives many women’s have facing many problems. I heard complains, like there is continuous vaginal blinding, not preventing the pregnancy, totally stopping menstruation. Even the community has developing negative perceptions for contraceptives, they associated with cancers using contraceptive, so the community is not happy by contraceptive. I think we should consider to solve the side effectives of contraceptives. Majority of females has damaged face because of the contraceptive, and their hair also.

I: Can you think of any more programs or policies? Think about political, religious and other influences.

P: By correcting the community perception we can increase birth intervals. We should do educate the science for the mothers. The expert should know the side effects of the contracts.

**Section 6; Multi-sectoral collaboration to improve maternal nutrition**

I: Do you feel it is necessary for your institution to work with other sectors/institutions to address maternal nutrition?

P: Yes

I: I think you have steering committee about this can you tell about it?

P: The steering committee members sectors are; health sector, agriculture, micro finance, Joint “*timret*” has contain three women’s (women affaires office, women’s league and women’s associations), TPLF office, water sector, education and justice office. I can explain the role of all these sectors but the main sectors are; health, agriculture and education should work jointly with us to prevent adolescent drop out from school, as we discussed above to educating women’s like pregnant women feedings, and the agriculture will educate production that is by identifying the appropriate crop for the soils to improve production. These are the main sectors for joint work. The first thing that we should know is the women office do not has separate tasks, all our work will be done with other sectors. The issue is to benefiting the women in feedings, even for home planning’s and others, so working jointly with other sectors is mandatory because we can’t do separately. Even beyond this 9 sectors, we though to work with the total 23 government sectors and for this case we did many discussion with the woreda administrator. So we are planned and starving to work with the whole 23 government sectors.

I: For multi-sectoral action that effectively works to improve maternal nutrition at all levels, what kind of change in terms of the way stakeholders work together is needed? What type of resistance to the needed change do you perceive or have you experienced so far?

P: Yes there are many challenges that we faced. There is a say “Women’s are coming” (*setoch metu*) this is what other sectors said for us while we are going to them to work jointly. The attitude for females is not changed fully, still there is problem. There are many challenges like all 23 government sectors don’t have plan and activity reports for women’s. When we going in to the 23 government sectors, majorities of sectors are not cooperative and not giving fast response for us, they haven’t sense of ownership. This perception should change and this change should began form top officials, if not we can’t change it at our level. There is a regional steering committee, this committee should evaluate the lower at woreda and kebelle level the nutrition activities of agriculture, small scale enterprise, and water and education sector. If we are not questioning each sector, at lower level it will be implemented haphazardly. If the top leader is not doing strictly, there will be problem at our level.

I: Is there coordinating platforms in enhancing multi-sectoral coordination in maternal and adolcent nutrition?

P: About this I can take the blame, because I don’t do such trials by developing proposal. But still on this issue two; with Axum University, and the new development hope organization which is partner that we work jointly on women’s nutrition. With Axum University we are working on supporting female students that don’t have mother and father. Around 320 young female students of this area has getting educational material support from Axum university, this to prevent them from school dropout.

**Additional Remarks**

P: About women’s I said already many things, but what I want to add is, priority should be given for women, we women’s should get education learn and the attitude for women should be changed.

Summery

**Section 1: Common maternal (pregnant women, lactating women and adolescent girls) nutrition problems in the community.**

Common nutritional problems

- Anemia
- Underweight
- Stunting
- diet related non communicable diseases

Most affected women group is pregnant mother

**Section 2: Nutrition priorities in the woreda**

Our involvement is necessary and currently we are doing promotion activities.

**Section 3: Nutrition interventions that improve adolescent and maternal health**

As a woreda what they do is coordinating with the health sector promoting women health like,

Promoting ANC and PNC

**Section 4:** **Implementation challenges and** **Community factors affecting access to nutrition interventions**

The barriers are

- Educational level
- Awareness
- cultural related
- caregiver giver related

**Section 5: Other interventions that influence adolescent and maternal nutrition and health outcomes**

An early marriage is harmful for females, there will be;

- Uterus infections
- Fistula and raptured uterus
- Psychological related problems

Increasing birth interval is important

- it is economical for the family,
- The child will get enough care, clothes and feedings.
- Not good for mother body

**Section 6; Multi-sectoral collaboration to improve maternal nutrition**

Yes, it is necessary to work with other institution mainly with

- Health,
- Agriculture
- Education Should
