# Supplementary material for: Barriers to utilize nutrition interventions among lactating women in rural communities of Tigray, northern Ethiopia: An exploratory study
Source: PLoS One. 2021 Apr 30;16(4):e0250696. doi: 10.1371/journal.pone.0250696 (PMC8087028; doi:10.1371/journal.pone.0250696)
Supplement: S2 File — (ZIP) [file pone.0250696.s002.zip › S2_File.Doc/Woreda level and above key informants/127_IDI_Vice head for Water resource office_Medabay Zana Woreda.docx]

OPERATIONAL RESEARCH ON ADOLESCENT AND MATERNAL NOTRTION IN NOTHERN ETHIOPIA

TOOL A

IN-DEPTH INTERVIEW GUIDE WITH EXPERTS, NUTRITION FOCAL PERSONS AND HEALTH EXTENSION WORKERS

PRINCIPAL INVESTIGATOR: DR. AFEWORK MULUGETA

DATA COLLECTION

IN-DEPTH INTERVIEW REPORT OF THE VICE HEAD OF WATER RESOURCES, MINING AND ENERGY OFFICE IN SELEKLEKA TOWN, MEDABAY ZANA WOREDA, NORTH WEST ZONE, TIGRAY, NORTHERN ETHIOPIA

SELEKLEKA, ETHIOPIA

NOV 27, 2017

**Information sheet and consent**

I: Good morning. My name is Yasin Jemal. I am from Mekelle University. We are doing research on the factors that influence the nutrition of mothers and adolescent girls in collaboration with the Regional Health Bureau and UNICEF. Your participation is valuable. The information that you tell us will not be shared with others. However, the information will be recorded and used to improve nutrition programs and services for women and adolescents in the region and in the country. I have several questions to ask you that we have prepared in advance and we will ask you all to say what you think about each question. Ensuring privacy of everyone here not to speak what we discuss outside of this group is strictly not allowed. The interview will take 1:30-2:00 hours. Do you have any questions before we begin? If you have any concerns or questions as we proceed please feel free to let me know. If it is alright with you, I will turn on the tape recorder now.

Participant: Ok, I agreed to participate

= = = = = = = = = = = = = = = = = = = = = = = = = = = = = = = = = = = = = = = = = = = = = = = =

**Note:**

I: Interviewer

P = Participant

= = = = = = = = = = = = = = = = = = = = = = = = = = = = = = = = = = = = = = = = = = = = = = = =

**Section A: Interview details**

| Questions | Answers |
| --- | --- |
| Zone | North West zone |
| Woreda | Medabay Zana |
| Town | Selekleka |
| Name of key informant | Goshu Tewelde |
| Institution of key informant | Water resources, mining and energy |
| Interviewer name | Yasin Jemal |
| Date of interview | Nov 20, 2017 |
| Interview start time | 10:32 AM |
| Interview end time | 11:45 AM |

**= = = = = = = = = = = = = = = = = = = = = = = = = = = = = = = = = = = = = = = = == = = = = = =**

**Section B: Socio-demographic Information**

| **Socio-demography of Key Informant** | |
| --- | --- |
| Questions | Answers |
| Sex | Male |
| Age | 26 years |
| Higher level of completed education | Bachelor degree |
| Current job/position | Expert in water resource management and acting as vice head of water resources, mining and energy office |
| How long have you been in the current job/position | 10  Months    03  Years |

**= = = = = = = = = = = = = = = = = = = = = = = = = = = = = = = = = = = = = = = = = = = = = = = =**

**Section 1: Common maternal (pregnant women, lactating women and adolescent girls) nutrition problems in the community.**

I: What do women/adolescent girls do to stay healthy in this community?

P: To stay healthy what women/adolescent girls need to do is (one) to eat balanced diet and when I related this balanced diet with our sector, clean water is part of nutrition. Thus, if they get clean water and then by getting balanced diet and if they get all services and if they improve their socio-economic condition, they can stay healthy. Thus, if you see from water sector perspective, what is expected from us is just to provide and bring water to their nearby. Currently, our standard is to have/get water access within 1 Km radius and 20 L/C/D. Thus, if we provide based on this standard, they (women) will fulfill their nutrition demand.

I: Good, what else do women/girls do to stay healthy? For example, what do pregnant women do to stay healthy?

P: To have regular medical checkups and follow up, and apply what they are advised by health workers.

I: For example, what?

P: Let say during pregnancy when they go to health facility, they are advised what type of food they should eat, and for how many days they should check. Thus, overall they should apply these advices and based on this they can stay healthy.

I: What else?

P: The other is to apply what is told.

I: Okay, what about those lactating women?

P: Lactating women should also breastfeed properly to their children at least for 6 months since they are future generations.

I: What else?

P: They should keep personal hygiene of their children as well as their own personal hygiene. And as I told you, based on the government directions there are conditions what a child needs to grow healthy and there is what we called family planning. Thus, they should use such services on their areas and taking/accepting the lessons and then applying these advices. They should also eat vegetables and fruits and as I told you they should keep their personal hygiene.

I: What else should keep clean?

P: They should keep their environmental sanitation. At least if they clean their own area and to that extent if others share, the environment will be clean. Thus, they should keep their environmental sanitation.

I: What else?

P: …Silent…

I: Okay, what about the adolescent girls (10-19 years of age) do to stay healthy?

P: In what? ….Ehhhh….in nutrition?

I: To stay healthy

P: Is that to stay healthy? As I told you, they should also…. as adolescent girls (10-19 years of age), they know some. Thus, they should also keep their personal hygiene plus as I told you earlier, it is important to eat fruits. In addition, as far as they are in education, there are health aspects given in education sector about personal hygiene, what problems they should face during period (menstruation) and how they should keep their hygiene. Thus, in that there are different issues and it has full trainings. Thus, to apply this, it is expected from them.

I: What else?

P: ….Silent…

I: What else?

P: …Silent…

I: Okay, good. In your opinion, what are the common nutrition problems in the community for women?

P: The common nutrition problems observed in our community are like stunting (Dinki). Is that called dwarf?

I: Stunting

P: Like stunting and lack having physical fitness or physical appearance at the right age (seen as prevalent) are seen in our community.

I: Do you mean underweight?

P: Yes, underweight

I: What else?

P: Mental retardation is also faced. …..silent….the other is eye problem (trachoma).

I: Night blindness

P: Okay, night blindness is faced.

I: Okay, any other?

P: Ehhhh…..The other ….quaaaaaaaaaa….

I: Okay, good. What about for adolescent girls?

P: The nutritional problems in adolescents can mostly be seen separately as rural and urban. Now, if it is in urban, it is better than rural

I: Problems caused by nutrition problem (malnutrition)

P: As I told you there is stunting as main problem and underweight as told you earlier.

I: Is there wasting?

P: Yes, there is wasting.

I: What else?

P: Nothing else.

I: Okay, good. In your opinion what are the common nutrition problems in pregnant women?

P: The problem faced in pregnant women is reducing their weight (wasting) and the fetus/baby inside (womb) may not also have normal weight.

I: How do you think pregnant women are especially at the risk of malnutrition you have mentioned above?

P: Lack of eating balanced diet, lack of awareness and lack of availability of the food due to poverty (for example, he/she may not get balanced diet though you told him/her to eat balanced diet).

I: What else?

P: Lack of emphasis and awareness and others

I: Okay, what about the risk of malnutrition in lactating women?

P: The one we mentioned are also the causes

I: What about in adolescent girls?

P: That is not out of them.

I: Okay, good. How sever the nutrition related problems you have mentioned are among the pregnant women in this community?

P: I cannot measure its severity but currently I thought that it is low since awareness is given in every area but it is difficult measure the amount so that to say this much is improved or not.

I: What about in the lactating women

P: The same to that

I: What about in adolescent girls?

P: The same

I: Okay. Do you think that women/adolescent girls in this community are suffering from micronutrient deficiencies such as anemia, night blindness, goiter and others?

P: Yes, there are.

I: Which one among these problems?

P: There is goiter, night blindness and dizziness (anemia)

I: How prevalent they are?

P: To provide the magnitude in percentage may be known with the experts

I: Why you agree with these problem?

P: Since they are seen.

I: What do you think are the reasons for the deficiencies?

P: Lack of balanced diet

I: What non-communicable diseases are common among pregnant and lactating women in this community? Are there blood pressure/hypertensive and diabetes type two among pregnant and lactating women in this community?

P: Yes, there are.

I: Why do you think the reasons are?

P: Lack of balanced diet

I: Do you think these could be associated with their nutritional status?

P: Yes, there is association. If your food is not balanced diet, the diseases mentioned may be caused.

I: Okay, are there any non-communicable diseases?

P: …Silent…

I: But are there these diseases really exist? Is there blood pressure?

P: Yes, there is

I: Are there diabetes problem?

P: Yes, there is

I: Good. Do you think pregnant/lactating women and adolescent girls in this community increase their height proportional to their age?

P: No

I: Why?

P: Since they don’t’ eat balanced diet.

I: Are there such problems?

P: Yes, there are.

I: Could it have relationship with their nutrition?

P: Yes, it is associated.

I: How it is associated?

P: It directly associated if they don’t eat balanced diet.

I: Can you give me example?

P: For example, if they eat vegetables, they will face night blindness

I: Just with height

P: Dwarf (stunting).

I: Do you think women/adolescent girls in this community increase their weight proportional to their age?

P: No, I don’t

I: Could it have relationship with their nutrition?

P: Yes, it has.

I: Why?

P: By reducing weight

I: Do you think women/adolescent girls in this community suffer from overweight?

P: Yes, I think and there are

I: Why?

P: Because it shows that the food is not balanced diet or it shows over, and may be not eating the food based on the blood type. As to me it is due to this when I understand it.

I: Is there a situation when the community suffer from shortage of food?

P: It may get reduced

I: But is there a situation even one suffer from shortage of food?

P: Yes, there is. It is a must

I: In what situation do you think this happened?

P: It happened more of in summer.

I: From which month to which month?

P: Starting from June up to September, shortage of food happened especially at home.

I: How frequent does it happen?

P: At that time they may not get enough food. A person may 2 times but still 2 times may not be enough.

I: Why it happened like that?

P: Since there is lack of water and the produced food is finished by that time. In addition, there is land scarcity, and as the population increases, there is no enough production that serve the population. Thus, based on this and as it is summer, shortage of food happened.

I: For which of the above problems do you think pregnant women are especially at risk?

P: Reducing weight (wasting) and night blindness

I: What about the lactating women?

P: Like that, the same. When they breastfeed, they become emaciated/wasted.

I: What about the adolescent girls?

P: In adolescents being stunted, wasting and mental retardation.

**Section 2: Nutrition priorities in the woreda**

I: In your opinion, what priorities do your institution has in relation to maternal and adolescent health?

P: From our sector, it is water that is to provide water to their nearby community and the standard of the government is to provide water within 1 Km radius. Thus, the aim is to provide water based on the standard.

I: Why water?

P: As far as our mission is to provide water

I: For what water can used?

P: It is part of nutrition and can be used for cleanliness, and since water is important to work every activity

I: What else other priorities?

P: In irrigation activities. We are doing check dam diversion infrastructure development to empower women to be independent. Energy is also the other priority area especially in making them use energy saving stoves and thereby to prevent the impact of smoke that causes to their health. In addition, we are working to make them use solar energy and biogas. We are doing these in a places where there are women.

I: Why you are working the irrigation activities and provision of energy in areas where there are women?

P: To make them beneficiaries as an affirmative action and thereby to improve/empower their economy and so as to improve their nutrition and health. For example, they eat vegetable and prevent smoke.

I: Good. What your institution is doing currently related to the priorities you have mentioned?

P: We are doing all the priorities but mainly we are working in provision of clean water and irrigation.

I: What nutrition interventions have the most resources allocated to them?

P: In provision of clean water the budget is covered by development partners (Non-Governmental Organizations) and the woreda budget is not this much. However, if we calculate, the highest budget is allocated for provision of clean water than others.

I: Do you think is necessary for your institution to get involved in work aimed at improving nutrition among women and adolescents?

P: Yes, it is necessary

I: Why?

P: Let from our sector through provision of clean drinking water, women can improve their health. Thus, working on that is necessary.

I: What is your institution’s mission?

P: Our mission is to provide quality and adequate water supply for drinking and irrigation activities to their nearby community by studying water resources and to make the public use the mining and energy sources and thereby to improve their economic development.

I: Would you please related your institution’s mission with the work aimed at improving nutrition among women and adolescent girls?

P: Since women are more affected by malnutrition and by others related to caring children, we have activities that we focused on women especially in places where there are high number of women, it would be preferable if there are irrigation activities in their nearby.

Thus, are focusing on works in irrigation activities especially in places where there are high number of women. And it is best if there is irrigation activities in their nearby so as to produce and cultivate by their own. Similarly, in drinking water since it helps them for cleanliness/sanitation as well as to nutrition itself.

I: How do you evaluate the priority given for the interventions for the women?

P: As to our sector, it is good (fair). Though we don’t compare it with others or health sector, at least it is good. Even the trend is increasing the women’s beneficiary.

**Section 3: Nutrition interventions that improve adolescent and maternal health**

I: What kinds of nutrition interventions are in place to improve health of the pregnant women to your level?

P: There may be by health sector but by our sector not this much.

I: In the community that you observed?

P: By giving different trainings

I: What services are given in the community?

P: Provision of water and energy (like energy saving stoves, solar energy and biogas fuels)

I: What else?

P: Irrigation

I: What services are given even by health sector that you have observed in your community?

P: I didn’t observed.

I: For example, service like examination during pregnancy

P: Yes, there is in their community.

I: Okay, tell me. What else?

P: To have pregnancy checkups. Ehhhh

I: What else?

P: Ehhh…the other is advice on nutrition, family planning and trainings are given.

I: Any other?

P: …Silent…

I: What else? Nothing else?

P: That is it

I: What about for lactating women? What interventions are in place?

P: Advice on how to eat balanced diet is also given to lactating women. The health extension workers showed them practically what balanced diet it would contain in each community/cluster.

I: Okay, what else?

P: The other is regular vaccination service

I: What else?

P: The other, nothing.

I: Okay, what about for adolescent girls?

P: In adolescents that you said me 10-19 years of age?

I: Yes. I mean the services given to them

P: Advices are given in schools such as in keeping personal hygiene, advice related to the food they eat, and services related to provision of sanitary napkins/pads.

I: Do pregnant women advised to visit health facility for checkups and services?

P: Yes. They are advised.

I: What services do they get?

P: Have examination and know the position of the fetus/baby

I: What would it help them?

P: To know their health status and thereby to take appropriate action

I: Do you think that pregnant women receive advice on the need of extra meal?

P: Yes, they receive advice especially health workers advise them.

I: What about the lactating women/

P: They all advised

I: Do pregnant women get screened for their nutritional status?

P: Yes, they are screened.

I: How they are screened?

P: Most of the time, it is done by health workers. The health workers have their own observation and advised them to get examined and based on that they took samples and measured them their upper arm.

I: What do they tell them after they measure their arm?

P: I don’t know. Health workers know this.

I: How do you think that it would help for?

P: To know their health status and then act accordingly

I: What about for lactating women?

P: Yes, there is everything.

I: How?

P: It is done by health workers and helps them to get out of the problem.

I: What about to the child?

P: To have good mental development and physical growth.

I: What about for adolescent girls?

P: It is like that.

I: Are pregnant women getting counseling for food diversification during pregnancy?

P: Yes, they are advised.

I: Could you tell me about it with examples?

P: They are advised to eat vegetables (when I say they are advised to eat vegetables, it is the one prepared at home, not in restaurant). They are also advised to take milk, meat, and to be free from alcohol (that is not to take alcohol).

I: Why do you think this is necessary?

P: Since it help for the health of the child/fetus and to keep the health of the mother.

I: What about for lactating women/during lactation?

P: Yes, they are told/advised.

I: Are pregnant women getting advice for the need to use iodized salt?

P: Yes, they are advised.

I: Why?

P: Since it help to prevent goiter

I: What about the lactating women?

P: They are advised to all

I: What about for adolescent girls?

P: To all

I: Are pregnant women getting advice on nutrition sensitive agriculture such as home gardening?

P: Yes, they advise and trainings are given on nutrition.

I: What about on the need to be involved in safety net programs?

P: Yes, they are involved. They involve in water/irrigation activities, agriculture, health, education and in road construction.

I: Are they different activities?

P: They are safety net activities but they may involve; for example, in water/irrigation activities and natural resource conservation.

I: What else interventions related to this?

P: Ehh… there are natural resources activities and they get involved in schools. Thus, they involved in all activities.

I: What about lactating women? Do they get involved?

P: Yes, they get involved.

I: What about adolescent girls?

P: Ehhh…adolescents they don’t not get involved.

I: Are pregnant women getting advice on water, sanitation and hygiene services?

P: Yes, they get advices and get training.

I: What about lactating women and adolescent girls?

P: Yes, they get advice.

I: Are women/girls getting advice on the need to use Insecticide treated bed nets (ITN)?

P: Yes, they get advised by health workers.

I: Why?

P: To prevent mosquito and thereby to prevent malaria

I: Are Women/girls getting deworming services?

P: Yes, they get advice

I: How do they get service?

P: There are health extension workers in each community and through that they get the service

I: Who provides the service?

P: Health extension workers

I: Why do you think is necessary for women in this community?

P: It helps them improve their health by killing the worm in their stomach.

I: Is there a situation, women/girls need to be addressed through Targeted supplementary feeding (TSF) in this community?

P: Yes, there is to help them get food support.

I: Could you tell me specific examples and situations?

P: For example, they are given corn soya plus currently there is what we called save the children gives them balanced diet to children and mothers.

I: What type of balanced diet?

P: Actually, I don’t know the food specifically. Any ways, it targets and gives them.

I: What about for lactating women?

P: It is also like that to them

I: What about for adolescent girls?

P: For adolescents, no.

I: Are lactating women getting vitamin-A supplementation after they give birth?

P: Yes, they are getting.

I: Is it necessary?

P: Yes, it is

I: Why is it necessary?

P: To prevent night blindness

I: What about for adolescent girls?

P: I don’t this much knowledge/information about vitamin-A supplementation on them.

I: In your opinion, are adolescent girls provided school feeding?

P: I didn’t observed till now.

I: But, is it necessary?

P: Yes

I: Why?

P: To improve their health

I: In your opinion, are adolescent girls linked to youth friendly services at health facilities?

P: Yes, they are linked through clubs.

I: Is it necessary?

P: Yes, it is necessary.

I: Why?

P: As they are adolescents and are at the fire age, it is necessary to get services on time that are needed them.

I: What about for out-school adolescent girls?

P: Ayyy…I don’t see. I didn’t observe.

I: Which of the interventions listed above do you think is most important for pregnant women?

P: Eating balanced diet, advice on cleanliness (such as water, sanitation and hygiene services) and medical check ups

I: What about for lactating women?

P: Eating balanced diet, advice on cleanliness (such as water, sanitation and hygiene services) and deworming services

I: What about for adolescent girls?

P: Advice on cleanliness (such aswater, sanitation and hygiene services) and vitamin-A supplementation

I: In your opinion, which of the above interventions for the pregnant women are being implemented in an effective way?

P: Medical checkup and insecticides treated bed nets (ITNs)

I: Why do you think that they are effective?

P: There is emphasis on medical checkups and awareness is given regularly, and there is also provision of ITNs.

I: In what way was they implemented?

P: Regarding ITNs, it is given and distributed in each community. Medical checkups are also given in their nearby by health extension workers.

I: What about for lactating women? I mean interventions implemented in an effective way?

P: For lactating women that are implemented effectively/successfully, one is ITNs and the other is medical checkups. It is the same with that of pregnant women.

I: Why do you think that they are effective?

P: The reasons are similar.

I: What about for in-school adolescent girls?

P: Advice on cleanliness such as water, sanitation and hygiene services

I: Why it is effective?

P: Since it is part of the education plus provision of clean water is done/undergoing in each area. Thus, since there is access, they get the service.

I: What about for out-school adolescents?

P: Medical examination

I: In your opinion, which of the above interventions for pregnant women are less effective?

P: Having extra rest are less effective. The other is having extra meal and/or balanced diet

I: What about for lactating women?

P: Here also having extra rest is less effective and I thought that deworming service is also less effective.

I: What about for in-school adolescent girls?

P: Youth friendly service is less effective.

I: What else?

P: School feeding is also less effective.

I: What about for out-school adolescent girls?

P: Having rest is less effective since they are involving in different activities

I: What else?

P: The other less effective is ITN service and its utilization.

**Section 4:** **Implementation challenges and** **Community factors affecting access to nutrition interventions**

I: What are the challenges to implement delivering the nutrition interventions that we have been discussing for the pregnant women?

P: One is lack of awareness, lack of provision of the service (such as lack of food provision) and poverty.

I: What else even from the service providers?

P: From the service providers there is lack of emphasis.

I: What about from their own?

P: Lack of emphasis from their own.

I: What about from the community

P: There is less emphasis from the community too. For example, when women get pregnant, the community make pregnant women get to involve in activities like other persons while special care should have to be given to them. Even in the community activity, the care (treatment) given to them is less.

I: What about the challenges for lactating women/

P: It is similar with that of pregnant women.

I: What about the challenges for in-school adolescent girls/

P: For in-school adolescent girls?

I: Yes

P: That is no provision such as no school feeding. The other is the support and emphasis given is less from the schools.

I: What else the challenges from themselves, family and community?

P: There is a challenge from the school. There is no challenge from themselves and if there is service, they can use.

I: What is the challenge from the community?

P: From the community, there is lack of awareness.

I: Is it the awareness that the community has about adolescent girls?

P: The awareness of the community to make support to adolescent girls is less

I: What about the challenges for out-school adolescent girls?

P: Lack of emphasis. Those in-school are better but for out-school even the service are almost not available.

I: What are the individual level factors for out-school adolescents not use the interventions?

P: They have lack of awareness and lack of motivation to think as it is useful to them.

I: How aware are the women and girls on the need to get the interventions?

P: They are aware of medical checkups/examinations, balanced diet/food and ITNs service/use but they don’t have awareness on others services/interventions.

I: Is there a relationship between educational status of the women & access to the interventions?

P: Yes, it has relationship.

I: What community related beliefs and norms are preventing access to the interventions?

P: There is traditional belief

I: Would you make it clear please?

P: There is an attitude like I grow up like this, so why you are now saying or doing this? This attitude makes them not to eat balanced diet. There is also an attitude, we were born like this and what happened to us? In addition, there is an attitude, we gave birth at home and nothing happened to us, so why don’t you give birth at your home and the likes. We grow up like this, it is the order from God if he wills.

I: Are the interventions culturally acceptable?

P: At this time, they are getting acceptance.

I: Why

P: Since they see/observe the changes in the public

I: What change is there?

P: For example, they are observing that child and maternal mortality is reducing.

I: What else?

P: They also see the difference between the one who eat and who do not eat.

I: How they observe that?

P: They know it; for example; they said the urban eats better but not the rural due to lack of awareness though we have all access.

I: Are the interventions accessible to the women and adolescents?

P: Yes, they are accessible. For example, there is ambulance service

I: What about to come by their own?

I: They can also come by their own.

I: What about the cost? To pay and get service?

P: Cost of services for mothers and children, I didn’t see them to pay that much.

I: They can afford it?

P: Yes

I: How convenience is interventions to the women and the adolescent girls?

P: They are convenient.

I: How do we know they are convenient?

P: Women/adolescents have regular medical checkups to know their health status and also children have to that extent, and may give birth a healthy baby.

I: How convenient is it to care herself; for example, by balanced diet, personal hygiene and environmental sanitation?

P: To care herself is also convenient through counseling and advice given by health workers, so she can care.

I: How do you explain the quality of the interventions?

P: Ayyy..The quality is not this much. You cannot say this much. The quality is poor.

I: What resources do exist to provide the interventions?

P: To provide the health service, there are health extension workers in each community.

I: What else?

P: There is also ambulance service, water service, ITN service and vitamin A supplementation.

I: What else?

P: Having agriculture and human resource (civil servant) are also available resources.

I: Okay, what do not exist to provide the service?

P: There is nothing the resources that do not exist.

I: How do you evaluate the commitment of the intervention providers at your level?

P: It varies but there is commitment in health.

I: What about in your institution?

P: It is like that

I: What else?

P: In terms of commitment, currently there is commitment to achieve the mission using the available resources. Thus, commitment is good at this level. However, when you see the results, it will much with the commitment.

I: Why?

P: As we said earlier, there are challenges in the public such not to accept and culture/tradition plus not working based on the system/procedure/approaches by service providers.

I: What other factors are inhibiting implementation of the interventions?

P: Nothing else.

I: For these challenges that you mentioned, can you tell me of any solutions that your institution have applied to effectively implement the interventions for women and adolescent girls?

P: Awareness creation is needed and regular monitoring and follow up and to have demonstration area for practical learning,

I: What else? Any other?

P: Experience sharing is also needed. Taking those who do not have good experience to those who have good experience so that to make them learn each other.

I: While your institution tries to solve the challenges, what problems do it faced?

P: There are challenges from the public.

I: What kind of challenges?

P: The service that you give them do not understand/know especially in some individuals but there are also individuals who understand positively. For example, when you provide them water and told them to fence it, they said grow up like this, so if you want do it yourself. Thus, there are such challenges.

I: What else other problems that you face while solving others?

P: In the activities we did, we face problems. For example, you made for them irrigation check dams but there are farmers who distract it at night. They distract it using hummer and they can use the materials for making utensils.

I: What do you think needs to be done to better address the challenges you have mentioned?

P: Creating awareness by going in to the community.

I: What else?

P: And as I said earlier, to have experience sharing. For example, those who do not have with those who care and maintain water supply service as well as irrigation activities.

I: What needs to be done to better address the challenges?

P: Working by approach. For example, if we take irrigation activity there is an approach called PIDM (participatory Irrigation Development and Management). Thus, if we work using this approach in irrigation, everything can be solved. There are works that we did good using PIDM.

I: What else?

P: Like in irrigation, if you use this approach in drinking water, you can solve the problems.

**Section 5: Multi-sectoral collaboration to improve** maternal **nutrition**

I: Do you feel it is necessary at your level to work with other sectors/institutions to address maternal nutrition?

P: Yes, it is necessary.

I: What about for adolescent girls’ nutrition?

P: It is necessary/key to all

I: Why it is necessary?

P: As they are part of the public, they have to be the beneficiaries.

I: What I am saying is that why it is necessary to work with other sectors to address maternal/adolescent girls’ nutrition?

P: It makes you to be effective and to serve the public that is the pregnant, lactating or adolescent girls.

I: What else other reasons to work together?

P: If you work together, you will be successful. You know there is a proverb called united fingers may bend the stick (Habirenya ziwetsa atsabi’etis arqay yetsembe’a).’ The other is to achieve the mission.

I: Which other sectors do you feel are necessary to work with your institution?

P: We, for example, we work with agriculture, health, women affairs and youth affairs. We did good job with these.

I: What else?

P: These are the stakeholders

I: Good. How do you see the other institutions’ roles complementing your role in improving maternal and adolescent nutrition?

P: It is poor as per my evaluation

I: Why it is poor?

P: With health sector, it is better since clean water is a component but the integration with other sectors is poor.

I: Why it is like that?

P: Not working as committed

I: What else?

P: Lack of emphasis

I: What else?

P: The other, nothing.

I: Good. How do you evaluate the level of collaboration among sectors in nutritional interventions?

P: It is poor/weak

I: Why it is weak/poor?

P: I told you earlier

I: I want understand/know it more?

P: Focusing on own work and not working together as well as not considering as useful

I: Okay

I: What kind of change in terms of the way stakeholders work together is needed to improve maternal and adolescent nutrition at your level?

P: It will be best if you work together in integrated way such as by having interface that is to identify the works that you are going to do on nutrition and others also identify the works that they will do in their part on nutrition and then by having common activities, you will have common agreement and consensus to the work together and act accordingly. The other is to have evaluation.

I: What else?

P: Leadership is also needed.

I: What else?

P: That is it.

I: What type of resistance to the needed change do you perceive, or have you experienced so far?

P: Not considering as useful for activities to be done, lack of integration, focusing on seasonal (timely) activities, focusing on own work and lack of commitment.

I: Are there coordinating platforms in enhancing multi-sectoral coordination in maternal and adolescent nutrition?

P: Yes, there are coordinating platforms

I: To what extent does your institution participate in the multi-sectoral nutrition coordinating body at this level?

P: It participates.

I: To what extent?

P: It has; for example, in clean drinking water supply we work in special way with health and education

I: How effective are the coordinating platforms in enhancing multi-sectoral coordination?

P: In the outcome, it cannot be said effective.

I: I am saying how effective are the coordinating platforms in enhancing multi-sectoral coordination?

P: They are effective but the outcome at the ground level, they are not. However, at the higher level, there is a trend of working together. Thus, they are effective at the higher level.

I: What needs to be done to improve the capacity of these bodies/platforms for effective coordination?

P: The concerned individuals to give them different trainings and awareness regularly. To have experience sharing

I: On which area should be training given?

P: On nutrition, sanitation and hygiene and overall in health

I: What opportunities do exist to promote multi-sectoral coordination of nutrition in this woreda?

P: What we say the opportunities include: currently, NGOs are pushing/encouraging you to work in integrated way. Let, when NGOs are working in water, they will not come without the health sector, women affairs and education. They come followed by these sectors is an opportunity by itself.

I: What else?

P: The other is the policy itself. The policy we have is also encouraging

I: What else?

P: …Silent…

**Section 6: Other interventions that influence adolescent and maternal nutrition and health outcomes**

I: In your opinion, why would delayed marriage (after 18 years) improve maternal nutrition?

P: It improves their nutrition.

I: How?

P: If they get married after 18 years, their mental attitude will be good so that they may resist the challenges they face and thereby they may reduce

I: In your opinion, why would increase the space between each birth improve maternal nutrition?

P: If she give birth with spacing, her body will be good and the food she eat will help her to recover. However, if she give birth continuously at a time, she may not get balanced diet. In addition, if she didn’t eat in the way that help both herself and the child, she would get affected. Thus, birth spacing is important to improve maternal nutrition.

I: What programs or activities promote increasing birth intervals in this level?

P: There is family planning program and awareness given by health extension workers and by every institutions.

I: Is there any programs or policies in place in this woreda to prevent early marriage?

P: Yes, there are.

I: What are these programs/policies?

P: There are activities done by women affairs.

I: What are these activities?

P: One study will be done to identify those under engagement/fiancé especially in those under 18 years old and who are student. The other is assessment is done in community to identify those who are preparing for wedding.

I: Okay, what other programs/policies prevent early marriage?

P: There is also law

I: What else?

P: …Silent…

I: Can you think of any more programs or policies like political, religious and other influences?

P: There is high political commitment but religious commitment is not this much.

I: What about the influence of marriage committee?

P: Yes, there is influence.

I: In your opinion, are these programs or policies effective?

P: You can say, it is good.

I: Why?

P: Even the emphasis given is good and the action taken or the punishment itself is a good lesson to others. The other is the awareness that the public has on early marriage is better. Thus, it is better from the previous.

I: What are the community factors that affect age at first marriage?

P: Would please repeat the question?

I: What are the community factors that affect age at first marriage?

P: One is the maturity and physical changes/appearance such as having breast, and not to have sex before marriage (adultery) or not to look others for having sex.

I: What else?

P: To make her support herself/independent

I: What else?

P: …Silent…

I: What are the policy factors that affect age at first marriage?

P: I think it is based on study to prevent physical effects (not to address/have physical effects) in the girl. .

I: In your opinion, what could be improved to prevent early marriage?

P: One is to implement policy. The second is to create awareness in every stages in each community.

I: Can you think of any other opportunities to prevent early marriage?

P: The policy itself.

I: What else?

P: The other is education. For example, if she is student, it is an opportunity. Thus, creating awareness at schools is an opportunity.

I: Can you think of any other opportunities to increasing birth spacing?

P: Here, the awareness given by the government is an opportunity.

I: What are these?

P: There is what we called family planning.

I: What else?

P: The other is the changes that having birth spacing brings in the health status of the women

I: What else?

P: …Silent…

**Section 7: Additional Remarks**

I: What lessons have you learnt regarding adolescent and maternal (pregnant, lactating and adolescent girls) nutrition at your level?

P: In irrigation that is in PIDM (Participatory Irrigation Development and Management) ther is nutrition. Thus, I got training on nutrition.

I: Any other lessons you learnt?

P: Not this much

I: What lessons have you learnt regarding multi-sectoral coordination of nutrition in this woreda?

P: I learnt the importance of working together and how it is effective, and even when you see the approaches and if you work based on that, you will understand how it is useful/important. Currently, there are trainings that I took with agriculture. They involve on nutrition and in vegetables and by water on our part. So, we participate in that and it is good as both of us are complementary to each other since the result/output is towards one.

I: So, what lessons have you learnt while working with health and agriculture sectors?

P: For example, I have learnt about cleanliness while working with health sector

I: What about with agriculture?

P: I have learnt about feeding/nutrition

I: What opportunities do exist to promote multi-sectoral collaboration on nutrition in this woreda?

I: One as I told you the programs which are coming followed integration approach. Any NGO or governmental sector says the programs should be done in integrated way. However, the previous one was independent way. Thus, it makes us successful.

I: What else?

P: The policy itself

I: What opportunities do exist to promote maternal (pregnant, lactating and adolescent girls) nutrition in this woreda?

P: One, there are services in each community and women/adolescents are getting the services plus they get awareness/lessons given from television or radio regularly.

I: What else?

P: The policy

I: What else?

P: …Ehhh…silent…

I: Okay. Thank you. I have finished

P: Thank you.

**SUMMARY**

**Section 1: Common maternal (pregnant women, lactating women and adolescent girls) nutrition problems in the community.**

The common nutrition problems among women/adolescent girls are stunting, underweight, wasting, night blindness, goiter and anemia.

**Section 2: Nutrition priorities in the woreda**

The priorities include: provision of clean water as part of nutrition within 1Km radius, irrigation activities (such as developing check dams) and provision of clean energy (such as provision of electricity, solar energy, provision of energy saving stoves and biogas fuels)

**Section 3: Nutrition interventions that improve adolescent and maternal health**

Nutrition interventions that are in place to improve maternal and adolescent health include: water service, energy service, irrigation activities, medical checkup, advice on nutrition, family planning service, vaccination service, advice on sanitation and hygiene, advice on nutrition screening, ITN service (advice on ITN), advice on use of Iodized salt, deworming service and so on.

**Section 4:** **Implementation challenges and** **Community factors affecting access to nutrition interventions**

The implementation challenges are lack of awareness, lack of service (lack of service provision e.g. no school feeding), poverty, lack of emphasis and lack of motivation.

**Section 5: Multi-sectoral collaboration to improve maternal nutrition**

Working with health, agriculture, women affairs and youth affairs is necessary to address maternal nutrition. To do this, working together in an integrated way is needed.

**Section 6: Other interventions that influence adolescent and maternal nutrition and health outcomes**

Delaying marriage after 18 years of age and increasing birth spacing improves maternal nutrition. The existed law, presence of women affairs and education (awareness in schools) are the opportunities to prevent early marriage while presence of family planning service and the policy itself are the opportunities to increasing birth spacing.

**Section 7: Additional Remarks**

Lessons learnt include: knowledge on nutrition and sanitation.

THE END

REGARDS,

YASIN JEMAL
