# Supplementary material for: Barriers to utilize nutrition interventions among lactating women in rural communities of Tigray, northern Ethiopia: An exploratory study
Source: PLoS One. 2021 Apr 30;16(4):e0250696. doi: 10.1371/journal.pone.0250696 (PMC8087028; doi:10.1371/journal.pone.0250696)
Supplement: S2 File — (ZIP) [file pone.0250696.s002.zip › S2_File.Doc/Woreda level and above key informants/128_IDI_Nutrition expert_Laely Maichew woreda.docx]

**Operational Research on Adolescents and Maternal Nutrition in North Ethiopia**

## Tool A

## IDI GUIDE WITH NUTRITION FOCAL PERSONS

| **Introduction:**  Hello, my name is Yemane G/mariam. I am from Mekelle University. Thank you for taking the time to speak with me today. We are doing research on the factors that influence the nutrition of mothers and adolescents in collaboration with the Regional Health Bureau and UNICEF. Your participation is very valuable. The things that you tell us will be used to improve nutrition programs and services for women in the region and the country. We will not share your names when we report our results.  However, I will record the discussion so that I can capture all the ideas that are shared. I have several questions to ask you that I have prepared in advance, and I will ask you to say what you think about each question. The interview will last for 1:30 -2:00 hours. Do you have any questions before we begin? If you think of any questions as we proceed, please feel free to let me know. If it is all right with you, I will turn on the tape recorder now.  **Section A: Interview details**   1. Zone:___North West_ 2. Woreda: ----------Laelay Maichew 3. Kebele: -Axum 4. Name of key informant: -Bitwded Birhan 5. Institution of key informant: --- 6. Interviewer name: Yemane G/mariam 7. Date of interview ------ 8. Interview start time: __8:30 Afternoon 9. Interview end time: __10:06 Afternoon |
| --- |
| **Section B: Interviewee professional information**   1. Gender    1. Female    2. **Male** 2. Age: 46 yrs 3. Highest level of completed education.    1. No formal education    2. Primary education    3. High school    4. College education    5. **Bachelor degree**    6. Master’s degree 4. Current job/position: _**woreda nutrition expert** 5. How long have you been in the current job/position:    1. ______ Months    2. 5 years Years |

**I:** Interviewer **P:** Participant

***Section1:* Common maternal (pregnant women, lactating women and adolescent girls) nutrition problems in the community.**

**I: In your opinion, what are the common nutrition problems in the community for women? What about for adolescent girls?**

P: Most of the time on mother especially on PW and LW malnutrition may happen. Since our woreda is rural most of the mothers are giving more children and don’t care themselves. For example during pregnancy they don’t eat extra meal and after delivery it is recommended to take two extra meal but they didn’t take two extra meal. Therefore they can be affected by malnutrition during pregnancy and after delivery. We check their nutritional status by measuring the entire mother their MUAC every month. Previously pregnant women were not coming but all are coming and follow their ANC. As we know our woreda is among one of that have good income or production or potential (secured). Previously nutrition screening were every three months we call it community health day but now the nutrition screening is every month and we call it mothers health day. When we measure the mother under nutrition was common especially when we measure. In some woreda there is food support like Tanqua abergele, Tenben and other given by world food program (WFP) and we were giving fafa and oil for mothers who are below 21 cm of MUAC. The support of WFP were also given to our woreda for those below 21cm in terms of fafa and oil and when the support of WFP stopped we considered that our woreda don’t have a problem of food production therefore we decided to measure the mother and counsel her on feeding and food diversification if she is below normal rather than giving fafa and oil. Even if this is good messages but since they expect the fafa and oil at the beginning it was difficult to implement. In 2008E.C the prevalence of normal nutrition was 40 to 50% among PW and LW but now in the first quarter it has raised to 70%. Regarding to the quality the WDA measures the mother in their community and sends them to us but the challenge is once they measured her they don’t send her immediately to health center. If the mother is with the normal range we ask her we she become normal and appreciated her work and for the mother below the normal we ask her why she become below the normal and the challenges finally we advice her to eat variety of foods for and her family. The challenge was when the HEW measures the MUAC of the mother simply they were sending the mother without advice that is why they become below the normal range. The mother say what is the importance being measured once we come after two hours of journey to the health post if it is only MUAC measurement. Once we identified this gap we were discussing with HEW and woreda health office by evaluating our performance on the quality of nutrition screening now it has increased to 71% since we give counseling on nutrition once they have been measured. The MUAC standard world widely said below 23 cm for mother is below normal but Tigray region was set the cutoff point to be less than 21 cm since most of them are thin but since the life is getting improved we have agreed to us the standard one which is 23cm as cutoff point. Since we have raised the range the numbers of women below the range are increasing but we are working to reduce the percentage while using this cutoff point.

Some time has taken to look for document review (searching) to display the magnitude of under nutrition mother.

Therefore the prevalence of women less than 23cm of MUAC was 41% among those who are screened for nutrition.

**I: Do you have separate report among PW and LW?**

P: We haven’t separated as pregnant and lactating women.

**I: What do they get if they are too below normal?**

P: Since our woreda yearly production is good we don’t give any food support rather we teach or advice those to eat food in diversified way because the challenge is even though the food is available in the house they eat today shiro, tomorrow shiro and next time shiro. No we are advising them how to use milk, egg and other production their child and family. Previously the focus of our government on nutrition program was if food is available in the house no matter on how they eat; they will be in good nutritional status. Now the government has change the direction to National nutrition program which is led by the prime minister that having food is not enough it must be eaten in balanced way to keep the nutritional status of the family. By assuming the problem is on feeding practice because children of rich person are facing malnutrition. Previously we were not working on this but in the past four years we are working on women nutrition by saying “A” (to indicate they are starting the work …).

**I: Does it have acceptance by the mother once the fafa and oil support is stopped? Who provide the information?**

P: At the beginning the community was expecting the food support and it was challenging but now in terms of their attitude it is getting acceptance. But they were raising question once we come after 2 to 3 hours of journey and the screening counseling was short they were complaining why the fafa and oil is not given. This the time were high amount of under nutrition was observed and the normal range of nutrition were increasing from 40% to 71% since the result is achieved within two years gap this show that there is promising achievement. Now once the mother screening we tell us her status and ask her what is available in her home and advice how to eat all this in balanced way this has brought change in terms of achievement to 71%.

**I: Can you tell in detail how this change is come? What was the role of WDA?**

P: First of all we identified the gap among us and the gap in the community why women are not getting nutrition screening; we were assuming they are not coming due to fafa and oil is not given but we try to discuss about our weakness than pointing our fingers to the community. We conclude the problem is with us because the attitude of needing fafa and oil is not broken because we don’t work on the creation of awareness in our community therefore we decided to advice the mother when they come for screening that previously we were giving fafa and oil since our production were less but now we have good production and the food in our house can replace even more than the fafa and oil that is why we have stopped giving fafa and oil. After all this discussion once the mother go and implent the advice she will observe there is change on her and her family therefore she will realized the importance and why she go more than 2 and 3 hours. We were working with supervisor and HEW and we have seen changes the coverage of nutrition screening and above normal range to be changed from time to time it was 40% to 50% to 60% and to 70% this is promising and we are interested to reach 100%. This change is achieved due to the acceptance of nutrition screening by the community. When pregnant women come she will be screened for her nutritional status in addition to other services given in addition to nutrition counseling. Once she goes and implements the nutrition advice she realized there is change and her attitude becomes good. We have registration book for mothers and children who are screened and unscreened and we follow them why they don’t come and measure. Once a pregnant woman gives births we register her to lactating mother and follow them accordingly. We have observed there is false report in other woreda they report none screened mother as screened mother therefore we put a registration book section by section for pregnant, lactating women and children based on this when a lactating women reaches one year we cancelled her from the registration book and we will follow like this. All the HEW are expected to be in the health post but since they have also expected to go to the community they are giving the screening in some day of the week especially during holiday where most mothers come and screened sometimes we go and actually observed the process and give them support as well as feedback this helps the HEW worker to give advice on nutrition to the mother.

**I: Is their micro nutrient deficiency in your woreda?**

P: We give iron for pregnant and lactating women since they may be bleeding during labour and after delivery it is must to take the iron. We are fulfilling all this now we have reached 95% coverage in giving iron for PW and LW. Regarding to Vit A supplementation we are giving for lactating women its coverage is reaching 92%. Others we don’t start like zinc except for children.

**I: Is there any observed micro nutrient deficiency among PW and LW?**

P: Yes, on pregnant mother if they have bleeding sometimes they develop anemia previously blurring vision what we call xerophytalimia was common but now it is totally not seen. And goiter is not common since 95% of the community used iodized salt based on the last three years study done. It is not seen among children and adolescents except rarely in some women.

**I: Is their stunting among PW, LW and adolescent girls in you woreda?**

P: Regarding to adolescentgirl they get iron and vaccination at school even though it is on and off but when we come to stunting it is cyclic the child grow, become adolescent, get married, become pregnant, give birth and become mother therefore starting from the beginning if the child is stunted and get pregnant there is more likely her baby to be stunted so it is cyclic pattern. If the pregnant women is stunted her pelvic may be narrow or incompetent to give birth. Now we are giving deworming by giving albendazol for adolescent girl based at school on the NNP I said previously but we are not working on it 100% we remain a lot to work and we have plan to work on adolescent girl.

**I: Is their nutritional screening at school for Adolescent girl?**

P: No nutritional screening for adolescent girl.

**I: Is their overweight in you woreda?**

P: Do you know malnutrition is both under nutrition and over nutrition we know that overweight is malnutrition but since we don’t have the measurement we are working on it even though due to the development some women are increasing their weight become overweight but till now we are not focusing on it. Since focus is not given no format to report or to measure but due to obesity some disease are now observed like diabetes mellitus (DM) and hypertension.

**I: Who is more affected by the hypertension and DM?**

P: Most of the times due to the pregnancy pregnant women are affected by preeclampsia and eclampsia even thought DM is common among men it is also seen in women.

**I: Do you think your woreda is affected by food insecurity?**

P: We can say this woreda is food secured based on the production but their traditional culture memorial ceremony “Teskare”, babtism day “Kiristna”, and wedding and graduation al l this has wasted the production this has lead the woreda to be food insecure. For example due to the “Elino” effect there were draught in our woreda due to no rainfall but we have tolerated the draught due to our production. If they planned to make memorial ceremony Teskare they will be penalized 5000 birrs now it is increasing therefore they will have double expenses and some of them have stopped teskare instead they give some money to the church and poor people. But practically we have seen that the woreda can tolerate drought for one year.

**I: Who are more at risk for malnutrition PW, LW or adolescent girl?**

P: Before deciding who is more affected or at risk for malnutrition first the adolescent girl must be screened but we can guess it may happen among the adolescent girl. Till now no direction is given about adolescent girl that is we are not giving focus on it but if it is introduced we are ready to work on adolescent girl. Since we focus and measure PW and LW the risk is more on PW and LW since they are on pregnancy and lactation for the baby. Even though they (PW) are taking iron and drug in addition they have ANC follow up it was very low even if it has reached now to 80%.

**I: What was the reason for low ANC follow up?**

P: The ANC has four visits; the first ANC is high but the fourth ANC is very low these is due to low follow up and close supervision but now the 4^th^ ANC is reached 80% because as I have said above we counsel them its importance during nutrition screening I addition we revised our performance and set direction how to improve them. For example we have structure how to address the community starting from the top to the WDA level and the WDA follow by asking the pregnant whether the pregnant women go to ANC how many times based on this she advice and inform the mother to go to ANC and nutrition screening. The WDA and HEW are linked which was established by L10K. The WDA has her sketch map showing the number of PW and LW by putting sticker the same has the HEW. Therefore the HEW asks the WDA how many Pw and LW are in her network, are they following ANC and other services if she says no the HEW and WDA go and advice the PW and LW to get the services to the health post. We were assuming it is difficult to handle in terms of incentive and keeping the link between WDA and HEW since L10Q was phased out but we have handle it because we have give training for WDA and some partners are giving incentives for HEW therefore the work is continuing without any problem.

**Section 2: Nutrition priorities in the woreda**

**I: What is/are your priority interventions related to maternal and adolescent girl nutrition?**

P: I couldn’t say we have done this on adolescent girl as I have said previously rather we give them deworming using praziquantil, vaccination and iron and Vit A supplementation which was facilitated by OSA and we don’t have nutrition screening for adolescent girl even regional heal don’t give focus on it when we see the HMIS format it doesn’t have any format about adolescent girl but we believe it must be included and we have raised it in regional meeting. But on PW and LW we have not any problem we are implementing it in a good way since the community has understood the importance. We are giving all services to the PW and LW with no challenges and we are increasing the nutrition screening. I am sure if we go like this we will achieve the nutrition screening coverage to 90 to 100% by the end of 2010 or 2011 E.C.

**I: Which nutrition intervention is more consuming your time and resources on maternal and adolescent nutrition?**

P: We are giving iron for six months for pregnant women and advice her to have one extra meal during pregnancy, to prepare clothes for her and the baby, prepare money and food preparation from different cereals and crop so called “Mitin”. Nutrition screening and counseling on how to prepare balanced diet every month is given and now it is increasing from time to time.

**I: What advice is given on nutrition counseling?**

P: the nutrition counseling is given how to feed herself during and after pregnancy and her child especially after six months of birth. The mother eat monotonous food for example they eat bean sauces and teff injera for long time but we advice them to make variety of food and sauces which available in their home. We have an interface agreement with agriculture to train the WDA to plant home gardening vegetables like salad, chili and tomato which can help to have variety of food in addition we advice on the importance of eating honey, milk, egg and meat all this must be eat in the home if they are available. This can be prepared in porridge form and given to her children and herself since if they child is health the mother will be health due to happiness her child. We show them how to prepare porridge for children above six months using “Mitin” prepared from variety of vegetables, cereal and crop they can buy it from shop, In our woreda together with Mekelle university there is supply of Mitin in Hatsebo, Mahber degu and Duwa kebele it is low cost as compared to other shop and have good quality there is big difference since we were followed for its quality together with one foreigner. The communities were adapting it therefore together with the home gardening vegetables it give good nutrition for the mother and the child. Related to micro nutrient we were following them.

**I: Do you think it is necessary for your institution to get involved in work aimed at improving nutrition among women and adolescents? Why?**

P: Yes, definitely it is relate to mission; our mission is to create health generation in each household in our woreda therefore to achieve this we have to teach the mothers how to feed their family, how to make balanced diet and home to keep their family sanitation and hygiene while they come for nutrition screening. Therefore if all individual are well nourished in each household then we are achieving the mission stated by laely maichew since very one become healthy. If you see most of the diseases are resulted due to malnutrition like measles, respiratory infection and others are common since our body resistance is decreased therefore as we improves our feeding the diseases decrease and we can achieve our mission but the challenge is on the quality of the service that we provides since there are health center and HEW who works hard and have quality and in other hand there are health center and HEW who works as usual with the same quality. In 2010 we have planned to work more on quality than the quantity.

**Section 3: Nutrition interventions that improve adolescent and maternal health**

**I: Is there any other nutrition intervention support given for PW and LW?**

P: Yes, the one component of ANC is about the need of rest for pregnant women in which now we are 80% of coverage therefore this indicate we must work hard on it. PW and LW are involved in safety net will be free from work starting from their pregnancy till one year after delivery since they are expected to care for her baby and her family. After one year she will continue to work in the safety net program but she will be allowed to start the work late than men and to go home earlier than men because she is expected to make sauces and prepare food where as the men are simply go home wait until she brings the lunch that is why we exempted her to come to work lately and to go home early. I don’t remember the amount of money but give them in cash. Since the child needs care while she is working in the safety net we have prepared day care using plastic home for those less than five years children therefore the mother can breast her child at any time there is caregiver for the child and from the safety net we buy for the children biscuits and soft drinks we have an interface agreement with the safety net program office. The mother is to come to the work at 3:30 if the working time is 2:00 and she will go home at 5:30 if the work ends at 6:30 this help her to get time food preparation for herself and her family.

**I: Is this advantage is given to all women?**

P: As I have said PW and LW are exempted not to work but all women and LW more than one year are expected to come and go based on the above schedule who are participated in the safety net.

**I: When did you start this intervention?**

P: We have started this in last year but now we have planned to scale up with the support of Tigray regional health and UNICEF.

**I: Do you advise them on water, sanitation and hygiene?**

P: We have given hand out which is given for NNP which starts on nutrition about stunting and wasting and finally it concludes with sanitation and hygiene implies that whatever you work on nutrition if there is no hygiene and sanitation we couldn’t improves the health status of the community even though this is beyond my profession but much must be done on this. We are facilitating the community to construct latrine most of them have latrine still some of them don’t have latrine we are working on it even though most of the toilet were temporary now we are working to change them into permanent latrine together with small enterprise which can serve the toilet for 7 to 10 years. There is what we call second generation HEW which is forwarded by Federal ministry of health which focuses on building permanent construction and we are preparing slab and the community buy it with balanced cost and expected to surround the latrine, water basin with soap, must have shadow and must cover the toilet. The face of the child must enter to the toilet and cleanness of the mother and the child must be keep. She must wash her hand on the five events during before eating, after working, after toilet used, before food preparation and after cleaning baby which is given by HEW and WDA. We are advising them to separate place for animals and the big problem almost in every one of us is low attention is given to kitchen. Kitchen is the most important in which we have to its cleanness and must have separate room but most of us don’t give care for kitchen now we are giving awareness on kitchen handling because all the food is made and store in the kitchen.

**I: What is the source of water for the community?**

P: We have hand pump water now we have made an interface agreement with water resource office previously treating the water with chlorine was our job but now we have given them to treat the water butt we follow them since sometimes they may not treat it for example last time acute watery diarrhea AWD was occurred it is due to untreated water and we inform them to treat it.

Sometimes the water pump may be injure the water resource office maintain it but to create sense of ownership by the community there is annual fee by the community which may helps to buy key for locking the door of the pump water, to repair the water pump and payment for the technician even though the community is not paying on time. REST has studied all the woreda with no water access therefore all woreda has water pump with minimum of one.

**I: Do they use ITN?**

P: We have plenty of ITN and it is distributed to each household based on their family size we may give one, two or three based on size. In addition we give additional ITN for those households who have PW, LW and children. But we when we come to our community they assume the ITN as wealth and they use one save the two ITN or may use to cover their property in the house. Based on this we have call meeting with the community and we presented by influential person of the community in order to use ITN properly some change were achieved but it is not fully therefore we are expected to work more on this. For those who saved the ITN we inform them about the sustainability of ITN supply and to use it now.

**I: Is their Vit A supplementation for PW, LW and adolescent?**

P: Please remember Vit A supplementation is on and off for both in and out school adolescent girls if you’re working to implement this; our focus is only on PW and LW even though sometimes we give immunization for adolescent girl.

**I: What nutrition interventions are more important for PW and LW?**

P: Monthly Nutrition screening is one of our interventions together with nutrition counseling if they are below the normal and normal. The advice has its own importance in promoting ANC follow up and to take micronutrients during lactation.

**I: What is your successful nutrition intervention on PW and LW?**

P: Vit A and Iron supplementation is our successful intervention we are 100% achieved on PW, LW and children. We are also successful in following growth and monitoring of children. But we are not satisfied on mother nutrition which is 70% we need it to be 100% like to that of Vit A and Iron supplementation.

**I: Why do you become successful in the above intervention?**

P: The reason for our success is we assume that all the root cause for the intervention is among us rather than pointing our hands to the community. If the community don’t understand or don’t implement what we teach them; we have to ask ourselves why they couldn’t implement based on this we discuss ourselves and ask the community to tell us our problem finally we will improve by making common understanding among us. If you clearly tell to the community and understood by the community it will implement it and they see change again they will expand it. Let me tell you one story there was one foreigner who teach starting from five months of gestational age the baby play when hear music, moves when they exposed to morning sunlight this was told for the mother and they approved it finally he taught the mother if you feed well yourself the baby will be happy like this. The same is true if we show and train the community you can change a lot.

**I: Which of the intervention is less successful?**

P: That is what you have said; we don’t work adolescent girl and we are not successful this due to no direction give to us from the top even when we work it is on and off.

**Section 4: Implementation challenges and Community factors affecting access to nutrition interventions**

**I: What are the challenges to implement delivering the nutrition interventions that we have been discussing for the pregnant women?**

P: Previously the intention for the need of fafa and oil were one challenge which make us to stay on 40% achievement for good notional status but now it has been improved. The other challenge is poor commitment especially HEW were reluctant in working and supporting WDA. The second most challenge is replacement for WDA when they withdraw; this may be due to their husband influence since the husband perceived working outside home as WDA is like **sex worker or widowed “maemin”.** There is what we call CBN community based nutrition it has big role in creating community awareness.

**I: What do they discuss in the CBN?**

P: The core message in the CBN is nutrition and sanitation; all women and their husband gather in between the message of exclusive breast feeding will be raised by the WDA if a woman who started complementary feeding earlier than six months will stand and justify why she start feeding, does she has toilet and how she is keeping her sanitation and hygiene finally she will gives explanation and she will takes lesson from other women even though it has importance people are not coming for different reason during CBN.

**I: What are the reasons not to come to CBN?**

P: We couldn’t say we have addressed all in awareness creation about maternal nutrition but during CBN people may not come for different reasons like if it is during summer they said I will go to farm and if it is in January they said I have wedding this all reasons are part of life and cannot be removed what we do is we tell them the discussion is not too long it will take half to one hour. Last time on the first quarter we have discussed that we have to see ourselves how we are serving our people and what must be done to improve by putting solution. We have the five percent rigid person that says” no and this is the problem of the community and the line.”

**I: Is there any belief or norms of the community that affects maternal nutrition?**

P: Previously they feed their children by giving tell during holyday “Tsbele” but now no problem. Though pregnant women and lactating women are not expected to fast but they are not allowed to eat meat and milk I think this cannot be changed.

**I: Why meat is not allowed for PW and LW during fasting?**

P: We have discussed with priest but they said unless she is sick it is not allowed to eat meat during fasting it is very difficult to change may be in the next generation.

**I: Do you think the nutrition interventions are accessible by the women?**

P: For pregnant women since we have ambulance they can give birth in the health center and we return them to their home after delivery in order to motivate the community. Before delivery we bring pregnant women if she is term and we fulfill the health center with waiting room to the mother and materials to make injera and others in each health center. We allow them to use chili from the health center and to make porridge from wheat, sorghum and teff. We did this to bring to zero death of mother as you now labour comes suddenly therefore it can happens any where any time which may lead to death of the mother and the baby.

**I: Do you think your nutrition intervention has quality and have enough resources? How?**

P: Yes, for example we get fund/support from UNICEF every quarter specific to nutrition intervention. The challenge regarding to the fund it depends on the report of the past quarter therefore if one woreda delay the report we will wait till they report this was our challenges. Where us our wored has gotten award due to on time report of performance. Regarding to other resources we get on time we distributed it on time no problem with resource distribution. Since 29% of mothers are not come for nutrition screening therefore the resource which is allocated for this mother is wasting this indicates that we must work hard by going to the community level.

**I: How do you evaluate the commitment of the intervention providers at your level?**

P: We can say there is commitment that is why we are discussing and fighting to improve maternal nutrition. We have an evaluation indicator for each staff performance we agreed that if our performance is high our work in the ground must be high otherwise it is reasonable that our performance is high and actual work is low. After long debate we agreed now we are working based on balanced scorecard. Some of them were defending us this blocks our chance to get competition at region level but finally it sharpen us now we are using it. Therefore everybody is aware of it unless they work hard they could not get good performance evaluation and they will get difficulty in competing for scholarship and other benefits; because of this they are working hard now. Now there is fatigue among HEW in other hand they are right all programs are implemented by them for example one HEW may expected to report more than 70 report formats per month since all program coming to the ground like nutrition, Tb now it is given in health post and all programs are down this has brought fatigue among HEW. But we are working with HEW to bring the commitment.

**I: What solution do you recommend for these challenges?**

P: it is obvious the programs will be implemented by the HEW in order to bring change but we have to add the number of HEW in the health post for example if you see Hatsebo we have assigned three HEW in the health post since it is wide. This must be introduced to other health post for example if they are four HEW per health post the 70 format report will be divided by four HEW in which they are expected to report 15 format this can reduced the burden they can get time to work with the community.

**Section 5: Multi-sectoral collaboration to improve maternal nutrition**

**I: Do you feel it is necessary at your level to work with other sectors/institutions to address maternal nutrition? What about for adolescent girls’ nutrition? Why?**

P: Now we are working with different sector for example we work with agriculture and we have interface agreement on safety net program now they have assigned budget for us. Safety net means the farmer will work either in health post construction, road construction or at school once they work they paid money. Now agriculture has financed us 100,000 birrs which use to build latrine or surrounding for the health post or latrine for school based on our demand. It also serves free safety net payment for PW and LW till one year.

We have agreed with agriculture any PW and LW less than year child will get free safety net the challenge is some LW denied the year of their child by saying the child is less than one year while the child is above one year. And PW once she brings the evidence that confirms her pregnancy she will get free safety net. We have also an agreement interface with agriculture about emergency preparedness for example last time some kebele were affected by ice rainfall therefore we study what was the income or production which kebele and farmer is affected by the draught or high rainfall. Once we have screened all this the letter will be send to federal level and they checked it based on the given data but we work it carefully since it may create dependency. Monthly we have report together with agriculture if any problem is seen I am the one sign on it.

With water resource office we work on home gardening, latrine and water access and quality we work also on HIV. We work with women affairs and we give them report if there is any problem.

**I: What do you work with women affairs?**

P: The women affairs have their own focus for example if the women are divorced and widowed and have more children we prepare to get free safety net since she is responsible in caring and feeding her children as well as other works. We work on the equality of women participation and representation starting from the HEW to the woreda office in terms of employment. In addition they are working with us on immunization and maternal issue since we give them the report they discuss and help us in giving advice during their discussion with women.

**I: How do you evaluate the level of collaboration among sectors in nutritional interventions? Why do you think is so?**

P: Yes, it is good because if women affairs, agriculture and water resources were not help us it is very difficult to achieve what we have achieved now. Therefore it is very important working with multi sectors integrated before the occurrence of the problem as well to solve them and we share information based on our performance. Therefore I can say it is effective.

**I: Is there coordinating platforms in enhancing multi-sectoral coordination in maternal and adolescent nutrition?**

P: For example as I have said to you there is NNP which is lead by vice prime minister; every sector have their contribution on maternal nutrition for example the role of health is 20% previously it was 100% our role, agriculture and other sectors have their own contribution. This helps to reach to the target if we work together collaboratively.

**I: What are the challenges that affect you to work collaboratively?**

P: We are working well with agriculture but with water resources office we need to improve; you know water means nutrition unless the community get clean water they can be affected by diseases but water resources don’t treat the pump water they have scarcity of structure and don’t have branches till the ground like health structure we have branches till the health post. When AWD was seen they didn’t respond immediately we were informing them to treat the water and we were discussed on these issues in the office.

**I: What opportunities do exist to promote multi-sectoral coordination of nutrition in this woreda?**

P: Yes, the presence of safety net is an opportunity which helps us to work together and will help us in improving maternal nutrition.

**Section 6: Other interventions that influence adolescent and maternal nutrition and health outcomes**

**I: In your opinion, why would delayed marriage (after 18 years) improve maternal nutrition?**

P: Yes, it doesn’t have direct relationship with us and I cannot say we are working it. Now they work on it even though adolescent girl are hurrying to get married and pregnant.

**I: Do you think nutrition and delayed pregnancy and birth spacing have relation?**

P: As you know when adolescent girl gets married they are affected by malnutrition when they become pregnant they will be challenged during delivery and she becomes responsible for her family while is she is too young less than 18 years. But if she married above 18 years she is matured in terms of thinking and handling responsibility as well as tolerates pregnancy and delivery since she is well nourished and the baby will be safe.

I: **In your opinion, why would increase the space between each birth improve maternal nutrition?**

**P:** Family planning is a base for the development of a country if you have more children than your capacity the share of love decrease, the share of care decrease and the share of decrease finally the children will become stunting and sick and become wasting become stunted and finally may die. If there is birth spacing the child grow well, eat well, get care well and educated well be free from infectious disease specifically till two years since more than 70% of the child growth is within two years starting from pregnancy. If no birth space the child becomes stunted and unable to learn and become load of the society.

**I: What are the community perceptions on birth spacing?**

P: There are some women who take family planning hiding from their husband and the community says why you use family planning; the child may become leader of the country why we are taking family planning. Still the community is having more children.

**I: Is there any police on birth spacing?**

P: Yes for example there is long term and short term family planning; but in our woreda long term family planning is more used 50 plus and marie stop and family planning guidance are working on it. Short term family planning user is low but we are working on it.

Now we have bring change on delivery by using religious leader but on family planning they are not happy instead we are using other influential person.

**I: Is there any police on early marriage?**

P: I don’t know the article but less than 15 years is not allowed since the women affairs screened it.

**Additional Remarks**

**I: What lessons have you learnt regarding adolescent and maternal (pregnant, lactating and adolescent girls) nutrition at your level?**

P: Generally I believe nutrition is very important for our community when I read nutrition related book, trained as health profession and get many training on nutrition I realized that we have real missed the importance of nutrition. But now we are using the opportunity to feed the mother starting from pregnancy by giving different micro nutrient and counseling on nutrition for PW and LW.

The introduction of CBN by UNICEF was very important since there is direct measurement of the child and the PW and LW by WDA it was very important learning forum since the discussion is in front of the people and women with low weight child will improve the weight of her child for the next month not to be shame again. Once she start feeding her child if she observed the improvement she will developed it as behavior. This is important in all like for latrine, sanitation and hygiene. In CBN every issue can be raised like family planning, latrine and sanitation.

**I: What lessons have you learnt regarding multi-sectoral coordination of nutrition in this woreda?**

P: For example in safety net when I heard of interface agreement I was think it is simply for protocol but now I realized we concerned in every work and we must integrated to work together to bring sustainable change. Since all the activities are interrelated therefore we must do together by involving the HEW, WDA and other sector to bring the changed. We have an interface with education to eradicate illiteracy through practical teaching on sanitation and personal hygiene given by the WDA side by side they teach them how to count. Previously we were resisting this is the responsibility of education office whereas when we see the WDA can write and read we become part of it.

Now the WDA are using the sketch map by them to track and follow PW and LW. During the session all sector give them a practical teaching on home gardening, education, sanitation and hygiene the work that was irritated us now it is fruitful and has bring change.

If one of the WDA member can read and write she teach her group member under the close supervision of teachers and the HEW is with them and all sector teach them one by one. In other hand the WDA that couldn’t and write are become writer and reader in which they are helping us in many ways like by following the PW and LW who are following the visit or not.

**I: Do you have any other comments on anything that we have discussed?**

P: Ok all what you have asked me is related to our work and may help you for different finding. Since you have said you come from Mekelle University I would like to speak about “Mitin” where balanced diet is prepared it has may impotence in supporting people mothers are getting Mitin from it. There was one foreigner working with agriculture they stop following them including Dr Afework why they stop their follow up since it has importance to the child as well as the mother and it was affordable and owned by some group of women to support themselves. We follow them but there is no link created between us and the women therefore link should be created and it is better if they follow them since the quality of the Mitin may reduced from time to time or not. Finally I would like to recommend the support of Mekelle University should continue. I: Dear Bitweded thank you for your time and discussion, I have learnt a lot from your discussion thank you again. If you have any concerns you can contact me any time take my phone number (my phone number given to her). Thank you very much for your time and information

**Summary**

Section 1:

- Stunting and underweight are common among PW and LW
- Overweight is not the community problem
- No food support for PW and LW unless they are malnourished and given fafa and oil
- PW and LW are not eating extra meal
- Heir is nutrition screening for PW and LW but no for adolescent girl
- Balanced diet “Mitin” is prepared and sold to the community by women league established by NGO
- Goiter is common in one kebele of the woreda
- Sharing food is common problem
- Women affair are more working with on maternal nutrition
- Multi-sector collaboration is weak in the woreda
- peer to peer education for youth or adolescents in the school
- institutional delivery is high
- 4^th^ ANC is very low
- ITN is well distributed but utilization is low
- They used iodized salt
- No strong support from administration office on financial and close follow up
- Lack of support for HEW and WDA
