# Supplementary material for: Barriers to utilize nutrition interventions among lactating women in rural communities of Tigray, northern Ethiopia: An exploratory study
Source: PLoS One. 2021 Apr 30;16(4):e0250696. doi: 10.1371/journal.pone.0250696 (PMC8087028; doi:10.1371/journal.pone.0250696)
Supplement: S2 File — (ZIP) [file pone.0250696.s002.zip › S2_File.Doc/Woreda level and above key informants/143_IDI_Regional Education bureau_Tigray region.docx]

**Operational research on Adolescent and maternal nutrition in Northern Ethiopia**

**In-Depth interview with Head of Regional Education Bureau**

**Introduction**

Thank you for your consent to take part in this study and for taking the time to speak with me today. I have several questions to ask you that I have prepared in advance. If you have any additional questions or comments as we do the interview, please feel free to share them with me.

| **Section A: Interview details**   1. Region: **Tigray** 2. Name of key informant: **Mrs. Selas Araya** 3. Institution of key informant: **Tigray Regional Education Bureau** 4. Interviewer name: **Abate Bekele** 5. Date of interview: **23/11/2017** 6. Interview start time: **03:10PM** 7. Interview end time: **04:15:41PM** |
| --- |
| **Section B: Interviewee professional information**   1. Gender    1. **Female**    2. Male 2. Age: **47 years** 3. Highest level of completed education.    1. College education    2. Bachelor degree    3. **Master’s degree**    4. PhD 4. Current position: **Head, Regional Education Bureau (Delegate), Curriculum Team leader** 5. How long have you been in current job/position:    1. ______ Months    2. **___29___** years |

**I:** Interviewer **P:** Participant

1. **Common maternal (pregnant women, lactating women and adolescent girls) nutrition problems in the community**

**I: In your opinion, what are the common nutrition problems in the community for women?**

**P:** The nutrition related problems, first, especially for pregnant it can have effect on the baby and on the mother herself can inhibit her to cope the effect of pregnancy. The child can be thin or may not have appropriate growth. Therefore, this mother may not have knowledge to feed balanced diet, and there could be shortage of food.

**I: Are there problems related with nutrition among women of Tigray region like thinness?**

**P:** There could be various problems in different areas. Though I cannot guess how much the problem is, there is under-nutrition. When we see women who are very suffering in their lives, and there is large number of births therefore there are problems that we can understand even though I cannot guess the exact level of the problem.

**I: Do you know any women who are receiving food support from health facilities?**

**P:** In this regard, it may not be in line with our profession. Therefore, I am not good to tell you about this issue.

**I: Are there micronutrient deficiencies like Goiter, night blindness and anaemia in women of Tigray?**

**P:** Yes there are! Especially there was goiter in high land areas of the region; there was an area that I was worked in which both males and females were affected by the goiter. It was in the southern Tigray and it was long time ago around 20 years back. However, nowadays, I don’t know the current status after iodized salt has introduced. There might be improvements after this intervention. But I know as there is goiter in the past.

**I: Is there a problem of overweight in women of this community?**

**P:** It is not that much and what I guess is there are high numbers of thin. It is sometimes visible in urban areas.

**I: Is there any food insecurity problem in your Community?
P:** Hum…yes there is problem and it can happen. As per my understanding I know there are households that have no sufficient meal though there are improvements through time. Even if there is adequate food, there is limitation in feeding diversified food.

**I: In which areas of the region there is food insecurity problem?**

**P:** There is a problem in both urban and rural settings.

**I: Why do you think women are especially at risk of malnutrition you have mentioned above?**

**P:** The women have double responsibility. The first, they manage the family. Secondly, they become pregnant and give birth then feed breast milk, feeding balanced diet for the family, the works in side home and outside the home, and a women have so many duties. Therefore, she has no rest and due to high number of responsibility, she is at higher risk to develop malnutrition.

**I: So, how they become under-nourished/thin?**

**P:** Primarily, they might not have knowledge on prevention of nutrition related problems. They simply feed what they have got then especially when a woman gets pregnant and lactating she suffers a lot.

1. **Nutrition priorities in the region**

**I: What priorities do your institution has in relation to maternal and adolescent health?**

**P:**  Our targets are schools, and at schools there are nutrition educations in the education system. The students learn and to practice it especially in schools that have water supply there are practical demonstration of gardening and food preparation. They acquire theoretical knowledge from the class like they have knowledge of food items that can provide us energy, body building, and prevention from form disease. And practically, in schools who have access to water source they can demonstrate and learn and the students again start to exercise at their home.

**I: What is the importance of prioritizing on such intervention?**

**P:** As a girl learns, she can apply in her life and it is easy to her to practice in her life unless she doesn’t have the problem of sufficient food supply. Therefore, a girl should learn and know. This is for school adolescents and they are from the community and go back to the community. Then if they practice it in their household, they take information to parents. And in the future, the girl will be manager of her family, therefore her current knowledge will help her to lead her family and help her to plan early to lead her family. Therefore, the women should learn, it is not about educating school gardening but it is to mean she has to have academic knowledge that will be helpful for the future. The entire community should learn and education is a base to achieve every goal and can bring change.

**I: How we can address those illiterate parents as they are older and stopped education once?**

**P:** There is the so called integrated functional adults literacy (IFAL). In this program there are various sectors involved such as the health professionals, and education professional. In this program, when they learn alphabet, they learn about food as there are nutrition professionals there in the team. Therefore, you cannot say this people have stopped learning, though the work is not that much strong, it can be addressed through this way. There are HEWs to teach about health related issues. Therefore, in various means they can acquire knowledge and learn. But the main thing is the children should grow through learning. The following generation should learn and be educated.

**I: What nutrition interventions have the most resources allocated to them?
P:** As bureau of education, the main task that we have done we have included it in our education system. Starting from the lower grades through higher at every level there is nutrition related topics even till the 12^th^ grade. For example, there is environmental science course for 1-4^th^ grades and for 5-6^th^ grades there is integrated science course (it is the integration of three sciences), and starting from 7^th^ grade there is biology course. Therefore, in each of these course there is nutrition related topics at every grade level.

**I: When was such integration of nutrition to education curriculum has started?**

**P:** We all have been passed through this way. There is such education in the past too. It already exists in the past and continued till today.

**I: Do you think it is necessary for your institution to get involved in work aimed at improving maternal nutrition?**

**P:** Yes! Hum...as education, to bring what should be included in the education system the education sector has been working on it. Hum... in this way it is important to work with education sector. For example, let us take school feeding; it is implemented in drought situations, it is done at schools. The nutrition experts implement it in schools, so it is important to work with education sector.

**I: How do you evaluate the priority given for the interventions for women?**

**P:** We don’t have separate work for nutrition rather than during planning of school feeding program. Otherwise, we don’t have separate work implemented besides the education system. In another way, we have girls club and sexual and reproductive club at schools at this clubs though it is not directly/specific towards nutrition, general works regarding female students have been done like for their education including tutorials and support, in prevention of early marriage, prevent school drop-outs, to prevent gender related discriminations and challenges. Otherwise, there are no specific nutrition related activities. So, we are working on the education aspect of nutrition. Otherwise, we are not giving them what to eat.

**I: As you told me there is school gardening, what is your intention about this intervention; is it intended to feed them or what?**

**P:** It is for demonstration purpose only. It is intended to practice the student and then when the student goes to his parents he can exercise at his home if there is irrigation opportunity. And to create awareness on the foods that can be cultivated used to prevent from disease, and the student learns as it has other importance and helps him to utilize his knowledge at summer or use them by buying. Therefore, this is important to create awareness to his parents about nutrition. Otherwise, the work is not to feed the students. It is just as demonstration.

**I: How accessible are the interventions for women and adolescent girls?**

**P:** As I said, the intervention is only for demonstration rather the women cannot use products from site.

**I: For example, in education do all schools have demonstration on gardening?**

**P:** For example, we have 2156 primary and 215 secondary schools among these only very few have school gardening due to lack of water. The majority learn only through the education system otherwise they don’t have school gardening. Only around 36% of schools have water supply. Even the water is only for drinking and it cannot be adequate for gardening. So I cannot say there is sufficient school gardening because when we compare the number existing school gardening with the number of schools it is too few.

3. **Nutrition interventions that improve adolescent and maternal health**

**I: What kinds of nutrition interventions are in place to improve adolescent health?**

**P:** Rather than the education system to provide nutrition education we don’t have any specific interventions for them.

**I: What kinds of nutrition interventions are in place to improve maternal health?**

**P:** Starting from the antenatal period and as I have experienced as being female, there are several antenatal clinical investigations, for example, those related with nutrition the pregnant woman is counselled on what to eat and in any other activities that should be done during pregnancy beyond the immunization and other health care services for them. After birth, she also counselled by health professionals on what has to be done for herself and her baby.

**I: Do the adolescents are receiving iron foliate supplementation at schools?**

**P:** These interventions are what the schools and health facilities work together. So this question is relevant for the health professional. Otherwise, sometimes we hear from our children, as they received supplements like iron foliate but I cannot tell the details about such interventions like when and where had it be provided.

**I: Are there women receiving targeted supplementary feeding?**

**P:** There could be, I know as there are cases in the past. I know as there are vitamin A, iron foliate and tetanus supplementation for adolescent girls in schools. There are no food supplements for school adolescent girls rather the school feeding program that is used by the all the students of the school.

**I: Which of the above listed interventions do you think is most important for pregnant women?**

**P:** For mother, the medical follow-up is important. Then she may be diagnosed for nutritional deficiencies and if in case there is problem she will be treated. For example, she will be told about what to do for here problem but she might not get the treatment for her case. So she is supported for such condition at clinics. She might get health care for free and helps her to get support for conditions occurring during pregnancy.

**I: In your opinion, which of the above programs are being implemented successfully (i.e. in the most effective way?)**

**P:** The effective program is the one that is provided by the health sector. But, the one that is important for their life time is education as it goes through their lives. The knowledge they have got through education will help them in the future life. Though I haven’t measured the result of interventions, I know if a woman learns, she will be effective and she can get solutions for her conditions. Regarding the agriculture, if the school gardening is done well, it will be effective.

**I: What are interventions confined to adolescent girls at schools?**

**P:** Regarding education, there are interventions. As I already said, regarding health, they receive anti-tetanus vaccination, iron foliate supplementation, and there are other life skill and academic related interventions too. But, there are no interventions focused for their nutrition.

1. **Implementation challenges and community factors affecting access to maternal nutrition interventions**

**I: What are challenges to implement nutrition interventions like school gardening, and nutrition education?**

**P:** The challenge is the issue of water. It is the shortage of water. If there is water, the school can be a site for demonstration. Otherwise, there are no challenges. To deliver nutrition education, they do have various clubs to deliver education and they also learn it from the science course. We teach them to cultivate crops at their summer season and if they feed various diversified food items, they will be benefited more. For example, if they include the carrot in their diet, they will have health eyes, and regarding other vitamins, the teacher demonstrates as there are various food items to have nutrients. The teachers also use the garden as teaching aid.

**I: How aware are the women and girls on the need to get interventions?**

**P:** What we believe is that the students can take messages to the community. The students are the bridges between us and the community otherwise we didn’t have planned to teach nutrition to women. We provide nutrition education for females together with males. But we don’t have education for adolescent girls separately.

**I: Which one do you think is effective, educating separately or altogether with males?**

**P:** The education system doesn’t have a room for separation. They have to learn together for example, at elementary level the males and females cannot be easily separated therefore in the education system it is not good to teach them separately rather it should be better to have separate nutrition education manual for interventions together with the sexual and reproductive and girls club. If the separate nutrition manual is prepared, it would be beneficial.

**I: Is there a relationship between educational status of women to access interventions?**

**P:** The educated women are better in accessing the interventions. First, the educated woman can read and she has also previous knowledge. Therefore, educated women are better.

**I: What community related beliefs and norms are preventing access to interventions?**

**P:** In this area where I grown-up, there are foods prohibited for women. I have something that I hear from people, but actually I haven’t seen them while practiced, for example, pregnant woman is not allowed to eat cheese, yoghurt and egg but I haven’t seen in practice rather they are supposition by others that I have heard. I have got this information while I took training on harmful traditional practices. This is happened as they assume the food items will be posted on the body of their baby. But when we compare the assumption with science it has no relation at all. It is just simply a supposition. I haven’t got the chance to visit the areas that have such practices; I hope it has been solved today. The area was to the western Tigray. I do think such practices are there today because there are HEWs working there.

**I: Are the interventions acceptable culturally?**

**P:** It is acceptable especially at urban areas they know about these interventions and use it very well. Again in rural areas, as the health coverage is increased therefore the acceptability is good.

**I: How convenience is the intervention to the women and the adolescent girls’?**

**P:** Regarding gardening, it is only implemented for sample in areas where there is water supply. Majority of our schools are in rural areas, they don’t have water supply if not there is irrigation in the areas. If the school is chanceful, it can use water.

**I: What resources exist to provide nutrition interventions?**

**P:** As the government capacity, yet there are no resources mobilized rather the UNICEF has taken sample woredas to implement the nutrition intervention such as school gardening.

**I: How do you evaluate the commitment of the nutrition interventions providers in this level?**

**P:** Regarding the school feeding program, the federal government of Ethiopia has allocated budget so far but it is not sustainable. For example, there was work done on nutrition last year but there are no works this year. Therefore, there are no sustainable interventions regarding nutrition.

**I: What are the causes for unsustainability of the interventions?**

**P:** It could be the problem of budget. Meaning, the government didn’t give assignment for nutrition as assignment given for other programs. But the work is done only during emergencies by securing budget from several bodies. Then it can be done for one year and in the next year it may stop. Therefore it is difficult to say even there is intervention like school feeding. Otherwise, there are no works done in plan with budget.

**I: What solutions that your institution has applied to effectively implement the interventions for women?**

**P:** Nowadays, we are planning to provide the program to be handled by the community. Just we are thinking to do so. For example, in winter season to contribute various crops to prepare diversified and balanced diet for students. Then the students can use it in the schools. It is just what we are thinking now rather is not started yet. But we go through, the intervention can be sustainable and we may also get other collaborating organization.

**I: What do you think needs to be done to better address the challenges you have mentioned like budget?**

**P:** The challenges should be identified scientifically and should be discussed and presented to government and if the government can solve it through mobilizing the partners. Then the challenges can be addressed.

1. **Multi-sectorial collaboration to improve maternal nutrition**

**I: Do you feel it is necessary for your institution to work with other sectors/institutions to address adolescent nutrition?**

**P:** The adolescents are in the schools, the majority are in school. Therefore, it is good if the education sector work together with others.

**I: Which other sectors do you feel are necessary to work with your institution?**

**P:** The agriculture, health, [hum…. Other….]. These two are the main ones as they have direct link with nutrition. Others, the government of region should work as a core since we are to ask budget therefore he should know the activities to be done.

**I: Is there multi-sectorial collaboration on nutrition in this region?**

**P:** I cannot say as there is collaboration. But for emergency so many sectors such as agriculture, university, WFP, UNICEF and …hum... had meeting together therefore multi-sectorial collaboration is necessary. However, it has to have its budget and should be sustained. But currently, it is not sustainable and it is only called whenever there is temporary works like emergency.

**I: Why it is not sustainable and how it be sustained?**

**P:** To make it sustainable, the government has to believe its importance and should work on it. The government should understand our problem and make us to work sustainable work.

**I: For multi-sectorial action that effectively works to improve maternal nutrition at all levels, what kind of change in terms of the way stakeholders work together is needed?**

**P:** We all should know our role on nutrition. Then if we work on our share and finally working for one goal, then it can be effective.

**I: To what extent does your institution participate in the multi-sectorial nutrition coordinating body at the woreda level?**

**P:** We have nutrition focal person under our planning department. He participates on nutrition related works like sekota declaration. He shared the information on how to strengthen school gardening for us. The detailed works is reported to my boss therefore I may not give you much information on this.

**I: What should be done to improve the capacity of collaborating bodies?**

**P:** It would be better if the specific organization is formed for nutrition, because now there is on-off type of work. Therefore there should be its own unit that leads the nutrition in the region. For example, it shouldn’t be done starting from adolescence rather it should be done from the birth of the child. Starting to work during adolescence may not be effective to halt nutrition related problems. Therefore, it would be good if we start to intervene from time of pregnancy.

**I: What opportunities do exist to promote multi-sectorial collaboration of nutrition in this region?**

**P:** The opportunities are, for example, as an education sector we provide theoretical lessons, and in practice we demonstrate using school gardening. There are also health professionals to teach about health related problems. Therefore, if each sector has done its own share or duty it would be good.

1. **Other interventions that influence adolescent and maternal nutrition and health outcomes**

**I: In your opinion, do think delayed marriage (after 18 years) improves maternal nutrition?**

**P:** If a girl is less than 18 years, her body is not matured and she cannot have strength to become pregnant and give birth. Then she will be exposed for several diseases. Therefore, it would be better for her health and next generation if she is matured physically and mentally for pregnancy and child birth and to cope it. Therefore, she should be matured by her age and enable herself by education to lead her livelihood.

**I: In your opinion, do think increasing the space between each birth improves maternal nutrition?**

**P:** If a mother gives birth with spacing, she can get enough breaks for herself. She will have rest from the pregnancy and breastfeeding. She will be also economically good since she is prepared for having next birth. A mother loses blood when she give birth, that blood should be replaced and she should be stronger before considering next birth therefore this needs certain period of time. And again she should get balanced diet in turn to get the diet she should work. Therefore, the work also needs break to do some trade. Therefore, birth spacing is important.

**I: What programs or activities promote increased birth intervals in the region?**

**P:** There are contraceptives. Though there are uptakes issues as women are complaining health complications due to the drugs, those who need are using it. But there might be complaints on their side effects.

**I: Can you tell me about any programs or policies in place in this woreda to prevent early marriage?**

**P:** This work is done well at schools. The age of students is already known, therefore there is students’ 1-5 network, there are also women affairs, and there is again education development army (EDA). These all work well to prevent early marriage. The school is working harder in this issue.

**I: What are the political factors that prevent early marriage and promote birth spacing?**

**P:** It is due to the political acceptance and commitment that the schools are implementing such interventions. There is political and legal support to prohibit early marriage. Likewise, there are also political support to promote birth spacing that is why contraceptives are advertised through medias. The health professionals are also working.

**I: Can you tell me about any religious issues in this woreda to prevent early marriage?**

**P:** It might be difficult to me to discuss about religious bases. I don’t think there is written thing that promote early marriage rather it is the decision of parents that forces the girl to marry early. Otherwise, there is no religious issue that promote early marriage.

**I: In your opinion, are these programs or policies effective?**

**P:** The lady doesn’t marry by force unless she is willing. Her age is known by her neighbours and the community and is followed. So far there have been works to prevent early marriage however the achievement may not be 100%.

**I: What are the community factors that promote early marriage?**

**P:**  Nowadays, the parents can understand. Though we cannot say the schools are accessible, there are interventions to prevent early marriage so that a girl does have education starting from her childhood age and she is unwilling to marry, and the community itself has awareness. However, in the past, there were even suppositions stating the education is unnecessary for women -“What is the importance of education for women?” but this has broken today.

**I: So, how can we address the existing problem of birth spacing and early marriage successfully?**

**P:** We have to work harder on the community and we have to convince them. For example, explaining the harm of the conditions, for example, what would be the problems that will be occurred if a girl marries under 18 years? We have been seeing our sisters that have exposed to the risks of early marriage like fistula and other problems. Therefore, we have to educate the community the harms of early marriage and the advantages of marriage at recommended age.

**I: Can you think of any other opportunities to prevent early marriage and increase birth interval?**

**P:** At school, there are interventions to prevent early marriage and they also learn about importance of birth spacing in Biology.

**I: What lessons have you learnt regarding adolescent and women of nutrition in this region?**

**P:** [silence]…… [Lough] ………….it could be from my own knowledge otherwise I don’t have lessons from the works done. I have lessons on nutrition, and I have thought students, so from these I have got nutrition related issues that are applied in life and that I am using. For example, I know how to prepare and provide the disease preventive diets to my children, body builders, and energy providers.

**I: What lessons have you learnt regarding multi-sectorial coordination of nutrition in this region?**

**P:** There are things that I have got which I have limitations. For example, I have got knowledge on preparing blended food during school feeding program, and as the child can get what is appropriate for him from locally available food items rather than buying costly packed foods. Therefore, I have learnt how to prepare blended food from locally available crops.

**I: What opportunities do exist to promote maternal (pregnant, lactating and adolescent girls) nutrition in this region?**

**P:**  The presence health extension workers to the level of household. And the agriculture sector also has nutrition expert, the education has also nutrition expert, and the presence of integrated functional adult literacy. The regular students again have session to provide knowledge and practices on nutrition and the presence of media information. So, these are the opportunities.

**I: Any suggestions to add?**

**P:** No

**I: Thank you very much for you time!**

**Summary**

1. **Common maternal (pregnant women, lactating women and adolescent girls) nutrition problems in the community**

- There could be various problems in different areas.
- Though I cannot guess how much the problem is, there is under-nutrition.

1. **Nutrition priorities in the woreda**

- Our targets are schools, and at schools there are nutrition educations in the education system.
- The entire community should learn and education is a base to achieve every goal and can bring change.

1. **Nutrition interventions that improve adolescent and maternal health**

- Rather than the education system to provide nutrition education, we don’t have any specific interventions for them.

1. **Implementation challenges and community factors affecting access to maternal nutrition interventions**

- The challenge is the issue of water. It is the shortage of water.
- In the education system it is not good to teach girls separately rather it should be better to have separate nutrition education manual.
- There are foods prohibited for women.

1. **Multi-sectorial collaboration to improve maternal nutrition**

- The majority of adolescents are in school. Therefore, it is good if the education sector work together with others.
- Currently, collaboration is not sustainable and it is only called whenever there is temporary works like emergency.
- It would be better if the specific organization is formed for nutrition, because now there is on-off type of work.

1. **Other interventions that influence adolescent and maternal nutrition and health outcomes**

- In the past, there were even suppositions stating the education is unnecessary for women -“What is the importance of education for women?” But this has broken today.
