# Supplementary material for: Barriers to utilize nutrition interventions among lactating women in rural communities of Tigray, northern Ethiopia: An exploratory study
Source: PLoS One. 2021 Apr 30;16(4):e0250696. doi: 10.1371/journal.pone.0250696 (PMC8087028; doi:10.1371/journal.pone.0250696)
Supplement: S2 File — (ZIP) [file pone.0250696.s002.zip › S2_File.Doc/Woreda level and above key informants/144_IDI_head of Regional Youth Association_Tigray region.docx]

**Operational research on Adolescent and maternal nutrition in Northern Ethiopia**

**In-Depth interview with Head of Regional Youth Association**

**Introduction**

Thank you for your consent to take part in this study and for taking the time to speak with me today. I have several questions to ask you that I have prepared in advance. If you have any additional questions or comments as we do the interview, please feel free to share them with me.

| **Section A: Interview details**   1. Region: **Tigray** 2. Name of key informant: **Mrs. Hidiat Negash** 3. Institution of key informant: **Tigray Regional Youth Association Office** 4. Interviewer name: **Abate Bekele** 5. Date of interview: **23/11/2017** 6. Interview start time: **11:20AM** 7. Interview end time: **12:21:07AM** |
| --- |
| **Section B: Interviewee professional information**   1. Gender    1. **Female**    2. Male 2. Age: **28 years** 3. Highest level of completed education.    1. College education    2. **Bachelor degree**    3. Master’s degree    4. PhD 4. Current position: **Head, Regional Youth Association (Delegate), Social Department Head** 5. How long have you been in current job/position:    1. ______ Months    2. **___3___** years |

**I:** Interviewer **P:** Participant

1. **Common maternal (pregnant women, lactating women and adolescent girls) nutrition problems in the community**

**I: In your opinion, what are the common nutrition problems in the community for adolescent?**

**P:** The nutritional problems aren’t occurring during adolescence rather it starts from the birth of children. For example, a woman should feed balanced diet starting from the time of pregnancy. However, to do so there are some limitations because we have passed through the poverty hence it hasn’t allowed good feeding habit; the mothers have the awareness and livelihood problem. Due to this fact, the child grows in the face of this problem till his adolescence. Especially, in Tigray region, majority of the population are rural residents. And, adolescent girls are one of the parts of our community, and they follow the feeding habit of their family and they had awareness gap on feeding well, and less focus has been given for their nutrition therefore there are nutrition related problems in the adolescent girls of the region.

**I: Can you tell me the specific nutritional related problems happening in the adolescent girls of the region like thinness?**

**P:** At every meeting like forums, we hear as there are thinness and stunting in our region and at nation at large from the research findings, and the workshops we have participated. There are awareness creation works done in our institution level. But we didn’t yet clearly identify what to work regarding the problem. Otherwise, we are hearing and looking the nutritional problems.

**I: What are the possible causes for the nutritional problems?**

**P:** Nowadays, the people have improved awareness therefore the feeding style has improved, for example, the people are asking what to eat to maintain their health currently. However, in the past, there was thought that food is to fill the stomach and only feeding single food item was also considered satisfactory in our community. Therefore, this is one of the causes to the occurrence of nutritional problems. On the other hand, currently our rural community are producing poultry and milk but they sell the products rather than consuming them at home. They are not fully aware of the importance of feeding them. The third possible cause, in the past, the awareness creation mechanisms were poor and the government even didn’t give attention to nutritional problem. However, nowadays, this has changed because the community is receiving awareness from various sources of information like Medias and other sources. And it needs additional works to alleviate the problem.

**I: What are interventions done for improving the nutritional status of adolescent girls in the region?**

**P:** As our youth association, we didn’t have interventions done specifically for adolescent girls. But, there might be interventions done from other sectors like health, and women affairs. I don’t have exact information on what has been done by these sectors.

**I: Are there adolescent girls who are overweight?**

**P:** Yes, there are cases but being overweight can be due to other health problems though it needs professional explanation. There are also people who are told to correct their feeding style.

**I: Is there any food insecurity problem in your Community?
P:** As the region, the studies have shown as we are still under poverty. Therefore, this is indicative of shortage of food in the community. To solve the problem of food insecurity the government has implemented program therefore there are changes nowadays. For example, the government promotes use of irrigation to produce various crops such as gardening, and promoting the youth to get involved in different income generation activities like herding cattle. And there are other packages especially for youth to empower them economically and to secure themselves in terms of food. The effort that has been made to produce more secured community is very high in our region and it is indicative of the changes through time.

**I: How do you think women are especially at risk of malnutrition you have mentioned above?**

**P:** Nowadays the awareness has improved. However, in women of remote rural areas there are still traditional thinking like a woman shouldn’t to eat egg and meat. So, there might be things that should be abolished. Since there are WDAs, therefore through discussion with them on what the child should feed and what type of balanced diet should he take and this might lead the women more at risk for nutritional problems. The other thing is the women have work overload therefore they might take their meal on time like at the breakfast, lunch, snack and dinner. They mainly busy in home works therefore this might lead them at risk.

**I: In your opinion, what are the common nutrition problems in the community for pregnant and lactating women?**

**P:** We have been working in reduction of maternal and child mortality for several years. There were activities done during the MDG and currently in the SDGs to reduce stunting and mortality. Nowadays, I would say it has been reduced. As the reports we have heard, there was high maternal mortality in the past, but currently it has been significantly reduced and the region is also a model. For example, as of youth office, the youth are contributing to reduce maternal mortality through donation of blood, by transporting pregnant women to the respective health facility in the absence of ambulance and road to enable women to get health services, supporting women to get appropriate food and to participate in activities of the health extension packages. To do so, we have been working in creating awareness to youth population. However, still there might be some mothers who need support because of livelihood problem since they might have economic problem, there could be stunting and wasting in women. There are also reports showing the existence of the problem.

1. **Nutrition priorities in the region**

**I: What priorities do your institution has in relation to adolescent girls’ health?**

**P:** We have priorities for mothers too but not only adolescent girls. As I explained you earlier, we have age related organization and it doesn’t differentiate the females and males rather it works on both sexes. However, there are high numbers of males in the group than females but to include the women to the association we have done several tasks. Like the WDA, the youth grouping is done similarly. There is working system but we have done very huge activities on issues related with mothers before having the youth grouping system. We have done several tasks like donation of blood to prevent death of our mothers due to birth. So, for mothers who need help, we have done several tasks to reduce maternal death. As I have said ambulance before; the youth have prepared traditional ambulance to promote access to health facility in areas where ambulance is not accessible. But we didn’t focus on maternal and adolescent nutrition like what we have done for HIV/AIDS prevention and other health related services. We have awareness but we didn’t focus on nutrition and may not tell you the details on the problems related with nutrition. Otherwise, we were working to improve the health of women.

**I: So, which is the priority task for women and adolescent girls?**

**P:** I have repeatedly explained it to you like donating blood we have done several tasks and again we have worked on the traditional ambulance. These are works that we are well known. As evidence we can see the woredas. There also activities related with voluntarism. Therefore these are our main task that we are known. For example, the girl not only at youth stage even after marriage, she can manage her home clean, and her husband might be again youth therefore we create awareness on how to lead their life.

**I: Which interventions have the most resources allocated to them?
P:** For your information, the structure of youth association is different than the government organization structure. The income of our organization is from its members. Our work is not focused on tasks that need budget allocation. The main task of our organization is primarily mobilizing the youth and then we work to bring those mobilized youth to the youth organization structure, and it has its own rule and there is a fee for members. For example, a youth who is a member of the association should pay yearly and this is the source of the organization budget. In addition to this, we have been working by designing projects. For example, we had big projects in HIV that are done and there are also projects that are currently under the implementation. To create the awareness of youth towards HIV and enable them what to do, we are working through condom distribution and peer to peer activities. We have been working in people most at risk for HIV (such as commercial sex workers, long track drivers, waiters and daily labourers) through projects like with PSI NGO. The other project is environmental entrepreneurship to enable youth to generate/increase its own economy and changed the environment on the way to improve its livelihood. But the main task of our organization is to work on mobilization to enable the youth to utilize the package delivered by the government. We promote the utilization of packages by our youths at every forum/meetings. Otherwise, we don’t prioritize and allocate budget.

**I: Do you think it is necessary for your institution to get involved in work aimed at improving maternal nutrition?**

**P:** Yes, it is very important. For example, to work at program level we understand and want but the youth is one of the parts of the community. If we work on them, they are the next generation replacers. To prevent food insecurity and stunting, we have planned to work on the youth and it has started now. For example, we are stakeholders with health bureau, we work together many activities. The youth that our institution mobilized and provided awareness on the nutrition then together we can be stronger in working towards the nutrition.

**I: How do you evaluate the priority given for the interventions for women?**

**P:** In the past, especially when there is no works to construct roads we have saved several mothers. Even the women have witnessed the work of youth to support them and they have given feedback for us. Therefore, I believe we have done good work. Every year, we are working with youth come back from the Universities like on the donation of blood. We have planned what to do for mothers and we have also strategic plan and we work accordingly based on the plan. So I believe we have done good work, but we can implement it more than the current level like the youth organization. Youth organization structure is a big structure and beyond supporting mothers by blood donation and ambulance services, it should work on nutrition.

1. **Nutrition interventions that improve adolescent and maternal health**

**I: What kinds of nutrition interventions are in place to improve adolescent health in this region?**

**P:** Regarding nutrition, I don’t have say, more than we are working on the awareness creation. The awareness creation has started at lower levels together with health bureau and other sectors through committee. But independently as youth office, we have been working for youth. We don’t have any specific task from our side, as I told you before, rather we are implementing in the directions/packages set by the government. This work has been started and should be strengthened. Otherwise, we don’t have any activity related with nutrition for adolescents unlike that of HIV. To give you an explanation, for example in commercial sex workers to stop them from the work …primarily to make them to utilize condom for non-users, to change their awareness and to change their work, and therefore there are youth who get out of this work and started other work. There are girls who are started to use condom and educating others to utilize condom. There are a lot of works that we have done on HIV together with the NGOs such as transaction and ERA. The other to bring the behaviour change we have been working on peer to peer activities. Currently, there are also assumptions that stating there is no HIV infection since the education has reduced. Therefore, we are revising our plan to act as what we have done in the past, we are planned to work on creation of awareness and to bring behavioural change, and we have prepared session and have been creating the awareness.

**I: What are services that are in place to improve pregnant women health in this region?**

**P:** The intervention that has been done more on women ….hum... The women health is the one that our regional health bureau as well as the government of the region has given emphasis. And I am the one who is the beneficiary of the services. From my experiences as a woman, when I went to the health facility for the first time after getting pregnant, I had overall medical check-ups. Primarily the pregnant mother service has given and then the HIV test has conducted, and there are many treatments while I am pregnant. And during delivery, the current works done by women is better than what had been done in the past. There is care by peers and post-delivery there is also follow-up, immunization and so many ceremonies. Therefore, I think these are good activities and should be continued. Otherwise, I don’t understand more than these activities.

**I: Are there women receiving targeted supplementary feeding?**

**P:** I don’t have information regarding this issue... [Lough].

**I: Are there services like vitamin A and iron-foliate supplementation for in school and out school adolescent girls?**

**P:** These activities are provided by health sector and they are given to them. Like immunization, and so many other tasks has been done at both school and out of school. For example, iron supplementation is given for pregnant at health facility. And these activities are done. But I don’t know the details and we may get the exact information from the health sectors.

**I: In your opinion, which of the above listed interventions is implemented for women and adolescents successfully?**

**P:** As to me, the work that has been done on mothers to reduce their death as we are recognized on it. It is very big success for me.

**I: Why reduction of maternal mortality was successful?**

**P:** In the past, there was high maternal death however based on the MDGs related to mothers and children, since we have planned to reduce maternal mortality to zero, and the entire people including the governmental and non-governmental sectors have been involved in the implementation of activities to reduce maternal death. Due to this fact, I would say, the goal is achieved. Therefore, due to the focus given the reduction of maternal death has achieved.

**I: Which of the interventions for adolescent girls are being implemented successfully?**

**P:** We didn’t have evaluation, but there are organizations working. There should be awareness creation works on in-school and out-school girls. Especially, nowadays, at high schools there are pregnant girls, and undergone abortion. There are no many tasks done on the creation of awareness for these issues therefore it should be done. Though there are activities done, but they are not that can bring change. For example, we hear high number of abortion cases from high schools while we attend meetings. And the university students have similar cases of abortion. There studies that reported high number of abortions in adolescent girls. Now, I am talking as a layman, but there might be studies done by the professionals. What I know in this region is working on nutrition has started but if we work on nutrition like what has been done to reduce maternal and child death, we can be successful. Even though this is a suggestion by layman…. [hahaha]...

**4**. **Implementation challenges and community factors affecting access to maternal nutrition interventions**

**I: You were telling me as you are started to work on nutrition, what are challenges to implement nutrition interventions?**

**P:** We had collaboration on nutrition. But, stakeholders’ failure to work with emphasis is challenged as they don’t have good awareness on importance of nutrition and they didn’t give due attention to implement interventions related with nutrition. If all the people have awareness on nutrition and work on nutrition as what has been done for reduction of maternal death, we can be successful. Therefore, all sectors are less committed.

**I: How aware are the women and girls on the need to get interventions?**

**P:** Regarding adolescent girls, in rural setting there could be lack of awareness. But in schools, there is education about nutrition in courses like biology. And even me my self has learnt about appropriate feeding style for the human beings like vitamins A, B, C. but we have community that have limited due to their livelihood. Again we have community that has good living status. For example, in urban setting, the feeding style has been improved to more good level. But in rural setting if we strengthen the implementation of nutrition activities, they will be also changed but this can be achieved if there are changes in their livelihood meaning if we have sufficient income since there might be community that are still in poor livelihoods.

**I: What they can do if they have sufficient income?**

**P:** If they have income, they can select appropriate foods and can purchase what they want to feed. It is because they can feed what they want.

**I: Is there a relationship between educational status of women and access to interventions?**

**P:** Yes of-course! The educated woman knows many things like the feeding style for herself, she can have opportunity to read, can search as nowadays there is advancement in technologies therefore if you are educated there are several opportunities like internet to know about nutrition. For example, I read what to eat to either it can be to reduce weight or to improve my feeding style or to feed my baby. However, if a mother is uneducated, she only gets information from the community or family members. Despite this fact, currently we have a good women organization structure, like the WDA, therefore this is an opportunity to discuss about issues in group. For example, they discuss on what to feed for their child, and the feeding and cleanliness styles for their home. However, I believe the educated mother is better than uneducated in accessing the interventions.

**I: What community related beliefs and norms are preventing access to interventions?**

**P:**  Nowadays, it is left. But there might be settings still have misunderstandings like assumptions stating women don’t eat some food items might be still in some remote settings where the interventions has not yet reached. But, nowadays, the awareness of females has improved greatly. In history, in the past what we hear is the women shouldn’t eat in front of her husband and she cannot eat food or stew in the absence of her husband, she not allowed to eat egg, and milk. These were traditional assumptions that our parents told us. I hope this has completely ended nowadays. But there might be the case in some amount in areas where the awareness creation has not be yet provided well. So such areas need strong work by all concerned body including the government and us ourselves.

**I: Are the interventions acceptable culturally?**

**P:** As to my understanding, every area has its own culture and livelihood. So it goes through their culture to persist the culture. But if it is said to be more helpful especially at health facility, like the education that is provided by nurses or the HEWs are acceptable even by the farmers. They receive education for their consumption. I have said earlier, it is to mean majority of the community has changed otherwise it doesn’t mean there are no community that fails implement what has been thought. It doesn’t mean all the community of the region has completely changed. It is from my observation.

**I: Are the interventions accessible for women and adolescents?**

**P:** There are model women development armies in the community and we are seeing them in Medias. And, most of the time WDA has discussion on the food item that should be feed by their kids and pregnant mother. There is also information dissemination of the feeding style and food items for children and pregnant women through Medias many times, and they have been done at community level through the WDA as well. So I feel the interventions are accessible for them. There are also radio messages and messages delivered on meetings are nearer to the community. But there might be community members who are not participating on meetings due to the reason of having other individual commitments. Otherwise, the interventions are accessible like the education and to create awareness.

**I: What about the quality of the interventions?**

**P:** There might be quality problem but the professionals can guess though it might be different across various areas.

**I: What resources exist to provide interventions?**

**P:** There are resources that are given at lower levels to my level of understanding like for those who are stunted and thin, there are supports given at health centers, such as plumpy-nut and their might be other services given that I may not guess. But I have information as there are resources at the facilities.

**I: How do you evaluate the commitment of the interventions providers in the region?**

**P:** Whenever there is meeting to report by health professionals and other government officials, there are messages that inform the need to focus on interventions related with nutrition. Nowadays the health professionals are again working several activities regarding nutrition because there are more stunting problems. But currently born children especially at urban area there are well nourished children that could be due to the intervention.

**I: What other factors are inhibiting implementation of the interventions?**

**P:** As I said before, the community situations like existing culture. Otherwise there are no other factors.

**I: What solutions that your institution has applied to effectively implement the interventions for women?**

**P:** As i said before, we didn’t specially focus on the nutrition therefore it would be difficult to me to say something on this regard.

1. **Multi-sectorial collaboration to improve maternal nutrition**

**I: You have said as it is necessary for your institution to work with other sectors/institutions to address women nutrition, but what about for adolescents’ girl?**

**P:**  Yes it is important. We have understood as it is important to work collaboratively and we are working now. Still we are involved in multi-sectorial works like with health, sport, youth and other sectors. And we are on work and it has to be continued.

**I: How do you evaluate the level of collaboration among sectors in nutritional interventions?**

**P:** Especially the youth association has become the member of multi-sectorial groups. I think there are various challenges. There might be lack of commitment to work there. And they should sensitize all the members of the collaboration.

**I: What type of resistance to the needed change do you perceive or have you experienced so far?**

**P:** Most of the time when we work with stakeholders there might be increased work load, therefore there might be less commitment and less focus. But these have to be overcome to be successful in nutrition.

**I: How effective are the coordinating platforms in enhancing multi-sectorial collaboration?**

**P:** As a sector, we believe in collaboration. Even in our institution, most of the time good works have been done when there are collaborations. If clump with only one hand you cannot do much work, so when we collaborate we can do effective and good works. For example, what I have told about the maternal and children health achievements were done by multi-sectorial collaboration.

**I: What should be done to improve the capacity of the multi-sectorial collaboration?**

**P:** As I have tried to explain before, when there is meeting of the multiple sectors, you prepare schedule and an action plan like the stakeholders meeting for approving plan, and evaluate report. And if we work on the prepared action plan, and we increased our commitment more, the works of each sector can be effective. Therefore, I would say all should share their inputs towards the implementation. Not only creating multi-sectorial collaboration plate-form but also if each sector should share its role then we will be successful.

**I: What opportunities do exist to promote multi-sectorial collaboration of nutrition in this region?**

**P:** We have opportunity to work with all stakeholders, we have own structure, have also high number of members like more than 400,000, and it is the structure that has high number of members, therefore these are opportunities. The next, the health bureau is ready to work with us. And it is to mean we have readiness to work together with other sectors.

1. **Other interventions that influence adolescent and maternal nutrition and health outcomes**

**I: In your opinion, do think delayed marriage (after 18 years) improves maternal nutrition?**

**P:** This marriage should be conducted after 18 years law is set due to the girl become mature physically after this age, and she can have good nutritional status and physically well, then there is evidence that a girl cannot be harmed due to marriage. She may not get much problem in the time of delivery but if she is at her earlier age she might develop fistula and any other problems. Therefore, the marriage should be based on the set law for marriage. There is a law that prevents early marriage (<18 years) for females and I think before 24 years for males.

**I: In your opinion, do think increasing the space between each birth improves maternal nutrition?**

**P:** Lough…In the past, it could be of our grandpas, there was a tendency of frequent birth, the mother bears problems, she become always breast feeder, caring the child, always pregnant, then her body may not have much strength regarding nutrition, she will be more injured. She will become always busy by caring her child. Therefore, birth spacing has much importance, the first, it limits population size, then it also helps to maintain her healthy and lastly it has benefit for her wellbeing and feeding of her child. Therefore, increasing birth interval is important.

**I: How long should it take to space birth?**

**P:** This is up to the mother who wants to give birth. Nowadays, there are also mothers giving birth yearly. But for me, it would be better if I gave birth with in the 4^th^ year interval. Actually, I don’t understand what I have been advised regarding birth interval rather it is my own plan my baby is 3 years old but yet I didn’t plan to give birth but I may give birth after his fourth year.

**I: What programs or activities promote increased birth intervals in this region?**

**P:**  There is a good contraceptive service. It is highly expanded. We are also providing it as we have place to deliver the service and then we also give awareness creation educations, we have also project working on it. It is for free in health facilities especially the governmental facilities. There are also so many types of contraceptives, like the injections that is given at every month, there are also given every day, there is also called loop that is embedded to the arm to increase birth interval the health professionals are currently promoting the long-lasting contraceptive like for five years, the contraceptive that can take long time like loop if I don’t mistaken. This promotion of long term contraceptives is to avoid short interval birth. Besides there are also awareness given regarding birth spacing.

**I: How should be the awareness created more?
P:** It has been given at the community level, at religious bases, and it has been given at various health facilities. Due to advancements in technology the people are more aware but at the rural community we should provide awareness creation through WDA and our youth structure. For example, the youth are marrying early nowadays so we should work on this.

**I: Can you tell me about any programs or policies in place in this region to prevent early marriage?**

**P:** Yes, there are programs that prevent it. For example, especially there are education given at schools on the consequences of early marriage, therefore the teacher provide such education and there are also such education on other sides. The justice office, women affairs, other women organization structure and all these involved in preventing early marriage and they also work up to accusing the misconduct. There are also situations where the girl and community can fight against early marriage legally. Moreover, they were prevented from marriage. I would say it is a good work.

**I: Can you tell me about any religious issues in this region to prevent early marriage?**

**P:** I haven’t seen at every place, but the church that I visit teaches to prevent early marriage and the priest while he comes to your home. In the past, there were issues that promote early marriage and giving birth. Nowadays, the religious leaders are even aware of the importance of birth spacing because of the education they have got.

**I: In your opinion, are these programs or policies effective?**

**P:** Yes indeed, it is happened due to collaboration and hard work. However, there are settings still practicing early marriages as I have seen from reports of Medias. They were reporting as they have prevented specific number of girls from early marriage with in the community. So still to prevent early marriage, there has to be done strongly.

**I: What are the community factors that promote early marriage?**

**P:** There were assumption such that “my daughter shouldn’t be rude” in the community. Without considering her age, the community assume she will become protected from rudeness if she gets married early. Rather than teaching the badness of early marriage the parents force her to marry especially if she failed to pass grade 10, there are parents that promote marriage for their daughter. Therefore, we have to work on this. There might be problem of livelihood that may force them to marry at early age if I don’t mistaken. There might be a need to live in urban setting. There might be assumption that the girls may assume they get married with the expectation to live in urban setting.

**I: What are the policy factors that promote early marriage?**

**P:** There is policy and even there is ban through the law of the country. Our policy discourages early marriage and it can accuse the individuals that have participated in such misconduct.

**I: In your opinion, what could be improved to effectively prevent early marriage?**

**P:** Everybody should take his role especially the community. We should work at our home with our children to alleviate the misunderstandings. We have to explain the consequences of life events. We should break the assumption that some talks are not conducted in front of kids, rather than this we should discuss with our kids. We should increase the work done through communication with our children should be increased. Multi-sectorial collaboration has to be done.

**I: Can you think of any other opportunities to prevent early marriage and increase birth interval?**

**P:** We have NGOs that work only on contraceptives, and the presence of Medias to share information is the good opportunities for us. The awareness creation activities at school are also important. The teacher should have awareness and work on creating awareness for his students in a way stronger than what has been done so far.

**I: What lessons have you learnt regarding adolescent and maternal nutrition at regional level?
P:** I have understood my weakness and strengths. Now I understood that if we work in collaboration in a well committed way we can be successful. Otherwise, if there are gaps in the collaboration and in the community, there could be less success.

**I: What opportunities do exist to promote maternal (pregnant, lactating and adolescent girls) nutrition in this region?**

**P:** In this region, there are different grouping structures such as that of women, youth, farmers, and more the community has included in such groupings. We have various media coverage like FMs, TV and facebook and the presence of religious leaders and the policy are again opportunities.

I: Do you think working on maternal and adolescent girls’ nutrition has importance?

P: Yes... [lough] even the half of the total population are women, therefore if we work on them the adolescents are our future generations, and mothers are also that who produce the next generation. If we work on women, we can easily change the community. For example, if we work on nutrition on woman, she will teach all the family members about nutrition and she can practice it at her home. Therefore, she can protect her family as well as the next generation.

**I: Thank you very much for you time and responses!**

**Summary**

1. **Common maternal (pregnant women, lactating women and adolescent girls) nutrition problems in the community**

- We are hearing and looking the nutritional problems.
- The studies have shown as we are still under poverty
- There could be stunting and wasting in women. There are also reports showing the existence of the problem.

1. **Nutrition priorities in the region**

- We have awareness but we didn’t focus on nutrition

1. **Nutrition interventions that improve adolescent and maternal health**

- I don’t have say, besides we are working on the awareness creation.
- The awareness creation has started at lower levels together with health bureau and other sectors through committee.

1. **Implementation challenges and community factors affecting access to maternal nutrition interventions**

- We had collaboration on nutrition. But, stakeholders’ failure to work with emphasis is challenging as they don’t have good awareness on the importance of nutrition and,
- They didn’t give due attention to implement interventions related with nutrition.

1. **Multi-sectorial collaboration to improve maternal nutrition**

- It is important to collaborate for maternal nutrition.
- We are involved in multi-sectorial works like with health, sport, youth and other sectors.

1. **Other interventions that influence adolescent and maternal nutrition and health outcomes**

- Early marriage is not beneficial and it hurts the girl.
- There are policies and programs to prevent early marriage and increase birth space in the region.
- Rather than teaching the badness of early marriage the parents force their daughter to marry especially if she failed to pass grade 10.
