# Supplementary material for: Barriers to utilize nutrition interventions among lactating women in rural communities of Tigray, northern Ethiopia: An exploratory study
Source: PLoS One. 2021 Apr 30;16(4):e0250696. doi: 10.1371/journal.pone.0250696 (PMC8087028; doi:10.1371/journal.pone.0250696)
Supplement: S2 File — (ZIP) [file pone.0250696.s002.zip › S2_File.Doc/Woreda level and above key informants/145_ IDI_ Zonal Women affair_South Easter Zone_Tigray.docx]

**Day11: 14 /03/2010 E.C**

**Translation: In-depth interview of zonal women affair**

**Section A: Interview details**

Zone: South Eastern

Name of participant: Mrs. Almaz Zewdu

Institution of Key informant: Social affair office (including women affair)

Interviewer: G/medhin.B

Date: 14/03/2010 E.C.

Interview start time: 11:00 Pm; local time

Interview end time: 12:18 Pm; local time

|  | Socio demographic information | | | |
| --- | --- | --- | --- | --- |
| **Sex** | **Age** | **Marital status** | **Education level** | **occupation** |
| Female | 39 | Married | Bachelor degree | Government employee |

Position: Head

How long have you been in the current job/position: 6 months

**Section1: common maternal nutrition**

I: In your opinion what are the common nutritional problems for women (pregnant, lactating and adolescent) in the community?

P: Among mother what I saw is, they did not resist disease; they have weakness and specially if they lactating, because they have addition job at home beyond breast feeding. They also frequently get diseased, because most the time disease affects women. This is what we observe at health facility and in the kebels where we visit for supervision.

I: What about related to nutrition?

P: Regarding to nutrition, as habit mothers tend to work; when there is under nutrition, which does not mean there is no food in the house. It is rather the thoughtfulness, meaning, when there are children they first care to the child or the family than for themselves. They are breasting but, there is problem in feeding for them. The hours itself is a challenge. Women work for many hours, and related with this, they loss their appetite. They cook themselves; they bake themselves. Therefore rather than eating for them, they run to feed others. Hence, there are problems here. And because of this under nutrition may occur.

I: what about problems related with micronutrient deficiencies (such as anemia, night blindness, and goiter) in adolescents?

P: Yes. It is common to see goiter and anemia among women. One day in Hintalo wajirat, there was a meeting with women, and fortunately, they want to donate blood. In almost all, there was anemia. Only few had normal blood. Therefore mothers are acing shortage of blood. Regarding the goiter, it related with iodine, and water utilization. On iodized salt utilization, women do not have awareness.

I: Are there many women with goiter.

P: Yes, there are; goiter mainly affects women.

I: What about mother night blindness? A different from trachoma;

P: Night blindness: unable to see at night. This is related with smoke, and even though it is not confirmed by examination, it is seen among mothers.

I: Diseases can be communicable or non- communicable; so how do you the non-communicable diseases among mothers? Like hypertension, diabetes, and others in relationship with nutrition?

P: Yes, they are seen. The diseases like hypertension and diabetes are seen especially at rural. But in semi urban, we cannot say it is not observing among women. But, we cannot take it as big number. In my observation diabetes is common among male than female. When you compare urban and rural dwellers, hypertension and diabetes are common in urban.

I: How stunting (low height for their age) among mothers, especially among adolescents, 10 -19 year old girls? How you see this in your zone, as stunting is becoming a peculiar characteristic of Tigray?

P: In our zone, when you see at stunting, it has its own age. If you see on the current time, it is better. In previous years, many people were stunted and thin. There were thinness; and these things are seen in our zone (south eastern zone) too. The stunting is related with the nutrition and awareness; and I believe these can cause stunting.

I: what do women think the reason for stunting?

P: They do not have an attitude that it is related with food. I saw them while we give training together with health. They think, it is comes from God. In addition, they say, it comes from family. His father is short; his mother is short. This is from grandmother, grandfather. Else it is not because of the feeding. Such kinds of thoughts are there with women. We cannot say this is solved completely. But, when you train them, they will be surprised how it is. There are women who told us there is a change in their children after they feed them based on the training they took.

I: How about underweight (low weight for age) among mothers/adolescents?

P: Yes, now on weight scale, there is a problem in awareness. They think my child is thin. So that he will be measured. This is to get the support given by the health extension workers. But, the thinness is not few. Although is it decreasing as to compared to the previous one. There are children who are born small and thin.

I: what about among the adolescents?

P: I think the problem is common among mothers. The lowest weight is seen among mother; adolescent are measured together with mothers (lactating and pregnant), and with them there is decrement.

I: Are overweight mothers and adolescents?

P: Overweight, it is not seen; it cannot explain it, the Woreda. She laughed. The problem is under weight than overweight. We can rarely see one out of hundred.

I: Is there a situation where the women suffer from food insecurity? Are there involved in safety net?

P: We have head of households; women with low economic status and they are identified and get involved in the safety net. There are more women in the safety net program. Their economy is low and they are supported. There are women who take direct and indirect supports. When it is direct, it is for women who are elder and cannot work. And, the indirect ones are those who can work for food. Therefore, we can take many of the beneficiaries are women that are head of households. The identification/investigation also works in that way based on them.

I: Who commonly suffers from stunting? Pregnant or lactating;

P: How it comes matter. There can stunting among lactating and among pregnant. Now, it may be difficult it explain it is more in this group, but there is stunting in both of them. We do not have an exact figure on the number stunting women who are pregnant or lactating separately. I do not know it.

**Section 2: Nutrition priorities in the Woreda**

I: what priorities do your institution has in relation to maternal and adolescents’ health?

P: Here we work a lot of things. As office of women affair, health related and social affair related activities are done here. Especially on women’s health regarding balanced diet; when we call it balanced diet, it starts with what they have at home. The problem regarding balanced diet is women are not utilizing the food they have at hand. Related to this we gave training on nutrition. And then, we establish association and thereby let prepare balanced diet like sugar potato; we train them process it and sell to women in the community. This is organized in collaboration with NGO. We are expanding it. The same is among adolescent whose age it 10 to 15 years. Therefore the job is to increase awareness. This was being done with partners.

I: What interventions that have the most resource are done by your institution?

P: Now, such activities are decreased, from 2008 E.C onwards, there are no such interventions. Even the charity organizations (NGOs) are leaving; when I compare them with what has been there in 2004, 2005 and 2006, there many activities; but now days, they are decreasing. There is no activity which is supported by budget in our office. May be in health sector, together with maternal education they give, they are doing better activities. In the other sector, there are no many things done to women on nutrition, and food.

I: Do you think it is necessary for your institution to get involved in work aimed to improve maternal nutrition? In relation to your organization mission;

P: Yes. This is the one we should work out; because, it about killing the generation if we do not work on nutrition especially at stunting and thinness. Related to this, the productive force can be decreased. Therefore based on this, Mekelle University should work, especially on awareness. The awareness problem should be solved; based on that we can solve the poverty too. There are two things: lack of awareness and poverty. The women may say “I will do this” but, there is no capacity (economy). She may not get the food if we only create the awareness. Thus, first we have to create awareness, and then to some women who are head of household education should be given. Related with shortage of budget, much is not done on this regard. If it is given they can increase some sort awareness.

I: How do you evaluate the priority given for interventions for women?

P: It is not that much. And no one is working on it. There is no office, I am not sure may be the health office, which works on women’s nutrition. There is budge for women, but there is no activity done on nutrition and mothers separately. There a number of activities done for women; example to involve them in irrigation; use inputs. Women are participated; but separately for women nutrition, even the Woreda does not put budge for it, and nothing is being done. Therefore, this is the area which we have to work on it, separately for women. But in general things, they are beneficiary.

**Section 3: Nutrition interventions that improve adolescent and maternal health**

I: What nutritional interventions are in place to improve women health? E.g. for pregnant women;

P: For pregnant women, counseling service is given; there is no other thing. There are women development army and HEWs who identify and send pregnant women to health facility. She is advised what she should eat, what should she do; including the four checkups (ANC follow up); to vaccinate at health center, and to deliver at health facility. Pregnant women are advised to do all these things. Even to the feeding after deliver, they educate them at the health center how to feed the baby and for her. Then when they get to the seventh and eighth month, they go to the nearby health post. The HEW has a list of pregnant women; she advise the when they to the health facility.

I: So, do you think they have the access to health facility?

P: This is very difficult. In some kebeles, the Kushets are very wide, and there is a problem in transport. For a six or seven month pregnant woman, it difficult for her to visit health facility for the sack of the advice; the health post is located at the center of the kebele. So to come to health post, they have lot difficulties. There are no such kind advices and vaccination services every kushet. It is mandatory for to go the health post, and this is very difficult.

I: What about for adolescent? Is there any intervention in place for adolescent separately? For both in and out school girls;

P: For the in school girls, lots of things are given in relation to HIV/AIDS. Much information is given in relation to ethics. But, for the out school girls, it is only the education given with development armies; however, there is no education given separately for them. There is a network: there is youth association; there is women association and there is women affair. But, there is no education for adolescent girls, and I have not any observation.

I: how do you see it necessity in relation risk of malnutrition they have?

P: Its importance is good, especially for adolescents; because, they are the generation who receives the country. The education given to adolescents, especially to out-school girls is important. Those who are at school can get education from the teacher and others. But the out-school girls are in darkness. If you go to some kebeles, there are surprisingly large number girls who do go to school. You will be shocked yourself and ask “where were they?” With these girls, it would have been good if something is done for them.

I: Is there a counseling service to eat extra meal during pregnancy or lactation? Do pregnant women screened for their nutritional status?

P: Yes, there is measurement (MUAC). The problem is thy do not come because of the remoteness of the health facility. We have many women who die. This is because they do not come; they are not measured and checked up. But if they come all the counseling services are available at the health facility. But, because of the distance, they do not come.

I: What about food diversification during pregnancy and lactation? Is there any advice given to women?

P: It is given for pregnant women.

I: What about if she delivers and become locating? How to prepare balanced diet for children;

P: They gave them, but it is limited. Even though it not in all sites, there is something prepared. It is given by the health professionals. There is no other body who works it. Now, the HEWs demonstrate for women how to prepare balanced diet using porridge. This is done at each kebele, and rarely at Woreda by calling the mothers. But, this is not enough with the existed problem.

I: Are women using home gardening? Do they get the advice?

P: ‘Eha’ she laughed. There are only few mothers who use home gardening. Now here, we go to each kebele; we see what is going on; we take experiences. We also visit by taking women. You simply can see one or two (Few) women selling vegetables. But intentionally, for personal use, they do not plant vegetables like pepper, spinach, tomato and onion. There are efforts to make women have alternative water sources. But, it is not effective and we cannot say women are changed by this. There are lots of things left undone.

I: What about their involvement in safety net programs, and work reduction and other related issues? Is it practical?

P: Yes, there is. If women are pregnant is in six months and above, they are waived from the work. The will get rest?

I: In that time, during the work reduction, is there any preconditions set for the women, like they have to visit health facility and make checkups? And bring evidence when needed;

P: They just bring evidence that she is six month pregnant; but I do not there is a question to be asked if she has followed the ANC, get advice and other vaccination service. The letter confirms only the period of pregnancy; if she is four month, it says four month; it does not explain the follow up; even it is good if this is addressed in the future. Because the community relates it with benefit, had use put that precondition, they could use from it; because, it pushes.

I: Are women advised to use iodized salt? Is it accessible? Do they use it?

P: Now it is good. We made a lot of efforts. There were a number of problems in the community, because people want the ordinary salt (nay Arho chew). Because, we give a number of educations, there is better iodine usage in rural. Even the access, now you can find it in every kebele.

I: how about its cost?

P: It is not such much; and no one raises a question on the cost. But in its application there some gap. And women say it is not tasty. But it is better now; and do not leave because of it.

I: What about regarding WASH (water, sanitation and hygiene s)? And utilization, e.g. toilet; access to water and other;

P: Regarding personal hygiene, education is given. But to keep your personal hygiene water is important. In south eastern zone, access to water is very difficult. They are in difficult situation. If you go you can check it. There in Saharti samre, there is water, and women travel for many hours to get water. There is Wajirat; especially those which are commonly affected by drought. Even here in Degua temben, it is “Degua’ (temperate), but there is still shortage of water. In Gonka kebele, Hintalowajirat, women go to at night, with hyenas, to bring water. The spring rises at night. And people complain that we are drinking with hyena at night. The water access at town is good. But in remote and drought areas, there is a problem. If you tell her to keep personal hygiene at that time, is she going to give it to her children or wash her cloth? It is a problem. This is true in some kebeles. The rest is good.

I: what about regarding toilet ownership and utilization?

P: There were many toilets dag. But, there is a problem in utilization. Fortunately now, I was compiling a report. There is a plan called ODF. But if you go to the kebele, there is no one who practices it. This means, people are defecating outside. If you look based on that, the toilets they construct are not permanent. The other is, even though they construct it, they do not utilize. They simple paint it and use it for show. Here, it we should work. Even, a study was conducted. The number of toilets reported in Hitalo wajirat was assessed, it decreases from the above 70% coverage to down to a lower number. When you hear from the research, it is destroyed; I do not know why; may be they want the land or else, you cannot I understand it. But they destroy it, and make house for cattle. Latrine utilization is a big problem that we do not transform it.

I: Who provide the education?

P: HEWs

I: Is there any other sector who involves in WASH, e.g. Water resource;

P: The office of water resource attaches the toilet with biogas; they also work something. But the primary body is the health profession including the management body. The activity is evaluated and the management also gets down to it. But we can’t say the desired change is brought.

I: Because of the personal hygiene and poor access to water, women may be affected by water born disease. Therefore, do women get deworming services? It can be per six months or per year; e.g. school children are given deworming service. What about to women? Lactating, pregnant, and adolescent girls;

P: I do not know this. Only chlorine is added water pump; by office of water resource. Even this has a problem; sometimes they do not add chlorine. There is complaining in kebele. The people say, they do not come and see it. There is also a chemical given to women which they can add it to water. But, I do not know the table given to be swallowed by women.

I: Did you think women (lactating/ pregnant) are getting targeted supplementary feeding, like fafa (corn) and oil for themselves after MUAC measurement?

P: Children are given additional food after measurement.

I: What about to the mother

P: I do not know it. But for the children, they are given fafa and oil if they are underweight after measurement. But for the mothers during pregnancy, I do not know it.

I: Are women getting Vitamin A supplementation? A small green tablet to prevent night blindness;

P: Vitamin A is given to women

I: when?

P: During vaccination campaign, there is time for it, and all women and children are given vitamin A.

I: What about to adolescents, to both in and out school girls?

P: For adolescents, I do not know it. I do not think they are given?

I: Is there school feeding program at school to prevent school absenteeism and withdrawal?

P: Yes, it was. I do not have any information in other Woreda, but in Hintalowajirat, there was school feeding program provided by ‘X’ project. In two or three schools, meat and egg were provided. But, the kebele was not selected by purposively based on drought status. I do not know how these were selected, but in Mesanu, there was feeding program; in Waza, there was school feeding program. Previously, I was a teacher, and I saw Fafa was given by UNICE. But now, there are no such things.

I: Which of the interventions listed above are important to women?

P: It is good if the underweight pregnant mothers are supported. It could be because of lack of awareness or due to shortage of food. Especially to those who are pregnant and have poverty, supporting them is not bad. This is because, it is important to the baby and for her. But we have to select them; it cannot be for all, because the government cannot afford it.

I: Is ITN given to women and do they utilize it?

P: Yes, it is distributed; even, if you go to some places, there is shortage because there is a need. However, during the utilization, there is a problem. Because they simply pack and put it; use to cover hey; early take off, after September where they think is malarious. Therefore there is a problem in utilization. But there is no problem on distribution, except shortage.

I: Do they get advice how and when to use ITN?

P: When they come to take ITN, they will be demonstrated how to use it in the health post; There is demonstration and education, but there is still problem. In some of them they use it appropriately.

I: So, how do you see the magnitude of malaria? Is it decreasing in the community and the women specifically?

P: It is better; if you simply look at the Woreda report, you will 59, is has decreased from the previous when it was in hundreds. There is change; the basic for the change is the ITN utilization; besides, awareness creation and the general prevention activities like drainage of swampy areas. Now, if you see the evidence, it shows there is decrement.

I: Which of the above interventions are effective to women (both pregnant and lactating)?

P: Regarding the change, it is the vaccination. The vaccination is of two types: there pre delivery and post-delivery. The most effective one is pre delivery (ante partum); in the post-delivery (post-partum), there is a problem. The follow up coverage (PNC) is low. It is about 68%. They left the follow up, because they have already given birth. The one that we call women have taken it is the vaccination (ANC follow up), and its coverage is100%. So we can conclude that the vaccination (ANC) is effective. Regarding the adolescents, they are using contraceptives; this itself is a change. Related to HIV and early marriage, there is also a change. There was no condition where couples come for HIV screening; but now there is a change among adolescents, because they come to health facility and check their HIV status before marriage. These all are changed.

I: Which of the above interventions are less effective to women?

P: Even though we cannot conclude these things are not implemented, we rather have activities we should work for. In relation to maternal health, we have to work on sanitation; we can work on toilet utilization. Even the feeding habit should be corrected, because we cannot say women have taken it well. You should eat balanced diet from what you at home. This time such educations are left, because the trainings are getting few. So it is good if we work on maternal and child nutrition. To adolescents that are out school we can do lot things: with their life; with job opportunity, with HIV/AIDS screening. There is nothing done more on out school adolescents especially those who are at rural. So, awareness should be created for them.

**Section 4: Implementation challenges and community factors affecting access to nutrition interventions**

I: what are the challenges to implement/deliver the nutritional interventions that we have discussed for women (pregnant, lactating and adolescents)?

P: I do not know if there is education given for adolescents for nutritional screening. They do not come to health facility and there are measured. May be if they are sick, they will be examined. Among mothers is lack of awareness. They do not know what is anemia; what is hypertension. The problem is lack of awareness. As the number of HEWs is few that is, they are three, two, and even one in some kebele. Thus, they cannot reach to every community in the Kushet or Got. Now, there is development army established, and their leaders are farmers like them, they can easily teach them. The problem is lack of awareness as there is on human power; there is no support.

I: What about factors related with community? Religion, cultural acceptance;

P: There could be such problems. It is not much, but there are barriers related to religion; what is the advantage of having examination and vaccination; only God and St Marry knows. Even there are men who become barrier to their wives. Her husband and relatives return her back from visiting health facility. Even though the women have an interest to visit health facility, there are such obstacles at the household which h people relate it with religious beliefs.

I: How about in terms of transportation and cost? Sometimes the Ambulance may not get in to the Kushet;

P: Yes, there is transport problem. What the government finally takes as solution is preparation of traditional ambulance from the community. Even this one is decreasing. Therefore there may transportation problem. Therefore, there could be a pregnant woman that prefers to stay home that traveling the up and downs across the mountain. Even in the presence of transportation a woman may not come associated with her capacity and economy, thus she may not get money.

I: How about the intervention? How convenient is the intervention to women and adolescents? E.g. may dislike to swallow; or tastelessness of the iodized salt;

P: Such things are not common in pregnant women; these are seen while using contraceptives; they complain of gastritis; and if you tell them to put it under their arm, they may say my hair will be lost; there is something called blood blockage. In this time, they feel shock. Such a kind of individual factors are there.

I: How about in terms of service quality? Related with skill of provider, and giving respected care;

P: This is not too much, but especially in the rural areas, when the midwives are men, they just do not want to be assisted by men care provider during delivery. “Our wives should not go to men at health facility” this was talked by husbands. Otherwise there is nothing said related to care provider in this time. Rarely, it happens with lack of giving respected care. When the women come for delivery, there is complaining and unnecessary talks among care providers. Thus the women may prefer to diver at home. Related to culture, when one gives birth, there should be porridge and coffee prepared on time. Even it is also related with Mielal (shouting). As solution the heath bureau provides flour and oil at the health center; and there is also a separate room to get rest and prepare porridge. These all are done to let woman happy and feel as if she in her home. Therefore there are lots of improvements in this regard. Thus, the service provision is better.

I: For the above challenges you have mentioned above, can you tell me any solution that can be applied for women?

P: What is important is the comfort to the mothers. Mothers, both lactating sand pregnant, need comfort. It is difficult, but if it is possible the health posts should be promoted to health centers. This is because many of the health facilities are health posts. And there, the HEW does not have the skill to assist delivery except if it is emergency case; they have a duty of prevention, not treatment. Thus, they bleed; and if the health center is far, she will lose lot of blood until she arrives in, and death might occur. Had they get access at nearest place it would be good. Related with the family and culture, awareness creation activities should be done. When we to the community we only meet the pregnant. But, it is also good if we also inform the family, especially her husband who can influence her decisions. When you tell her about nutrition, like eat egg, meat and other variety of foods, the family will understand her easily. They will understand the care needed for pregnant woman. But we, traditionally, train only the pregnant mother.

I: You are right; what do you think the role of husband can be to improve maternal nutrition? Regarding decision making, money ownership, and others;

P: Here, there is gender issue in women affair which introduces such things. By gender issue, there is an office which provides training on gender. But we can say it is changed. There are only some household heads who own money. Many of it is owned by men. Therefore, there must be activities which should be done for men to improve women nutrition. This could be done in general, for both men and women or separately for men. Here, there are no other things needed rather than awareness through trainings and community conversations.

I: What do your institution tries to solve to the challenges mentioned above?

P: Yes, especially on health, regarding sanitation, we work together with water resource. At health post, there is no water, thus if a women delivers, there is shortage of water. The health office and water resource works together to solve the situation, there by health posts will have water. And regarding the toilet, research should be conducted to verify the coverage, and leave the false report. There should be solution that can bring basic change. There are plans to mobilize the community from the scratch for 2010 E.C.

I: What about the practical ones? activities which can be seen on ground

P: In practice, what I have mentioned above is reading the water; we gave trainings to women, and also held community conversation in all the kebele; is it effect or not matter. There is development armies established there. Therefore they are established to work these activities.

I: What challenges do you face while working aiming to improve maternal nutrition?

P: We, as zonal office do not have much budget allocated for us to work. Many activities are done at Woreda level and we work with them. But the challenge in this year, related with the budget, we were not able to organized trainings, because we do not have budget for this purpose. Even the Woreda do not have budget; the budgets are known. Therefore, there is a problem here. You cannot reach to every kushet, but if you are to give training in one place, by inviting all women to one point, you need budget. In this regard, the women affair and even the health office is not with previous tempo. It is decreasing. Even it is getting back. Because they are farmers, the may not write what you taught hem and may forget it. Therefore, you need to refresh him, but here, there is shortage of budget. But, if you really want to work, the farmer is not a challenge.

**Section 5: Multi-sectorial collaboration to improve maternal nutrition**

I: Do you feel it is necessary, at your level, to work with other sectors/institutions to address maternal nutrition?

P: Working with partners is very effective. There is nothing you can work alone especially related with women affair; there is nothing they can work alone. There multi –sectorial team established, but there is nothing it did in ground. But all activities are done with other sectors. If it is health issue, we work with heath, if it related with adolescents, we work youth office. Even with agriculture, we work together on home gardening, and water resource. We all work together. When there is plan, we approve it together. Especially of women affair, we all together see it; there is a committee which is established for his purpose. We work based on this. It could be effective; but, there are a number of challenges while working with partners. One is absent and the other is present; agriculture is present bur education is absent and health is present but water resource is absent. Because of this, what you have planned to work and complied will be missed. As in the women affair, there is difficulty to mobilize and give activities to collaborating sectors. But, because we have no option, we have to work together and sustained it. We woke on mobilization activities.

I: Which other sectors do feel are necessary to work with you institution?

P: Other sector, what is? With education, we are working; we are working with all sectors; with health, we are work; if you see construction, there is job opportunity. If there is a job, an investor planned to work; it is done to together with women affair. If you say road, it is constructed for women too.

I: How do you see the other institution’s role in complementing your activity to improve maternal nutrition?

P: They are helping us; anyways there is 50% proportion of women in the community, and they are benefiting from all activities. However, separately, there is no any sector that works for women considering the importance of the intervention for women. This is true, except health office, and education where 50% of students are female. Let alone others, even in the report, it is difficult to identify which is for male which is for female.

What you can see is, it simply to work and achieve the activity; however, “let get sit and look for if women are benefiting from this; what can they benefit from; how are we going to help them; how should we change it to comfort them” such thing are weak. But, simply the work is done; they think women can benefit from it. No sector is going and oriented in this way.

I: How do you think this can be improved? What kind of change in terms of stallholders work together is needed?

P: You know what it is: if it can be improved, it should start from beginning while planning. During planning, this year, I will do this separately for women. The problem is it gets mixed with other activities of the office. If there is a plan, based on that, it has to be implemented. There should be at one person who follows this, the beneficence of women in the sector. This is not difficult; and even he can do in parallel to other activity. If is he is irrigation expert, he can at same time work the irrigation and the women’s issue. But it should be done separately for women. They have planned to construct alternative water source for women, but, it later get mixed with that the men. The follow up and support is also small. Therefore, if it is separated, she should be equally supported as men, because she is physically lower than the men. So she needs extra support, e.g. during the construction of alternative water sources like well. This could be in agriculture and the same should be done in the water resource related with energy like technologies that saves energy e.g. ‘Eton’, because women are suffering from smoke. Thus, there are such activities that can possibly be done. General the sector needs attention.

I: Is there a coordinating plat form in enhancing multi sectorial collaboration? Who lead the collaboration?

P: Is it at the women’s affair?

I: No, just it can be at Woreda level or zone level; any ways s/he coordinates plat form.

P: At regional level, there is women’s affair office; the office coordinates activities to be done among women. And at the sector, it has its own office.

I: what about at zone

P: At zone, it is mixed with other five activities

I: who lead the entire coordinating plat form?

P: At zone, this is led by the deputy and we all evaluate our performance at a time. But, to coordinate the activity, there is one person assigned, like the position I have, who led the social affair issues; there are five activities under this. The evaluation is led by the deputy zone administrator, and here reports are seen and get evaluated. Performance is ordered. At zone level, it has attention and it is good, because there is trial to benefit women.

I: What about regarding the evaluation of joint performance on women nutrition?

P: Yet there is no such kind of things; at zone we are expected to do this. We are not doing at the expect level. As report, it comes from the women affairs, but it is simply a collection of activities done to women by different sectors independently. But centrally our office, women affair, is evaluated based on its performance. But in evaluation of joint performance sectors on women issue is not yet practiced.

I: To what extent does your institution participate in multi sectorial collaboration? Is there something left?

P: Yes, we have lot of gaps. At zone level, we are coordinators. The women are 50% the population. Had one person is placed as affair at zone level it would be good, because, currently the activities are many; reports come from health, agriculture, education, women affair and social affair itself. Thus it is difficult to compile. We do not do many things beyond compiling report.

I: what opportunities do exist to promote multi sectorial coordination for nutrition among women?

P: we have no other option; we have to work in collaboration with other sectors. That is only it. Additional; it is human power, and it is good if they are added, because he can coordinate. Because, there is burden with currently coordinator while running to health, agriculture and others. With the sectors that are put by the government, we have work in collaboration. The other is with charity organization (NGO), if sectors collaborate with charity organizations, it will be good. Previously, for sack of women, there were experts that are assigned women. And, they were supportive in terms of material and human power; one of them gives Motor, and other give other thing; there was good support. So here, related with charity organization and investors, we are not doing more. Even when you look at the local investors, there is no one who works separately for women; they may simply give support to the Woreda. These things are left with investors that are local dwellers.

**Section6: Other interventions that influence adolescent and maternal nutrition and health outcomes**

I: In your opinion, why would delayed marriage (after 18 years) improve maternal nutrition?

P: Now, we are preventing underage marriage; because there are problems when she marries in under age. The big problem is related delivery; even it may risk her to death if she marries in under age. Other, it is related with disease, she may be exposed to fistula. The second is she is not psychologically ready.

Then after marriage, she may early divorce, because of misunderstanding and disagreement. These are common among adolescents married in underage.

I: How about related to nutrition?

P: Because they do not have awareness, they cannot predicate what is going on them; second, they are economically dependent; they expect for man. So here, there are problems which they encounter when they are married in underage.

I: what about related to increasing space between each birth and maternal nutrition?

P: Now, regarding birth spacing, there is change. Increasing birth spacing is good for the health of the women. There are some who give birth within a year and two years; and here you can see women get emaciated physical; there is also problem in their economy. Especially in this time, when they give born one over the other, they will face a big problem. They may think, there is no problem if he born; this related with poor utilization of contraceptives like what I have mentioned before. There are also pressures from family; husband has ordered her not to use; then she keeps giving births. Finally she will face health problems. The children are not using balanced diet and the mother is suffering.

I: What programs or activities are done to promote increasing birth intervals in this Woreda?

P: Information is given by professional and media using radio. We work together with responsible bodies; the same is the health sector, they work. The problem is what I have mentioned it before. That is, we cannot conclude that women have understood the message and have changed it to practice; their life is not improved, and still we have to work.

I: Can you tell me about any programs and policies in place in this Woreda to increase birth space.

P: This is provision of contraceptive at health facility, the rest easy even the health extension workers can do it. Contraceptives should be available at the nearest health facility. There are conditions when the HEW took it with her when she moves to each kushet. These are done.

I: what about in terms of political direction and religion and other activities?

P: There is education which involves religious leaders; there is community conversation at kebele, and Woreda level. Especially on religion and taking pill, ideas like the pill is killing baby; it is sin and likes have decreased; there is change. The religious leaders are now influencing positively. Because if he born and get suffered, it is sin. Therefore, the religious leaders, and community leaders are giving education on this matter. There are good things done in the community.

I: People explained me that during the safety net program, if a household have a family size of ten, he will be given support for only of the five members. Is this to discourage giving birth? Or else?

P: “Because I have ten children, I will use from the safety net” no one will give birth to befit from safety net.

I: Yes, my point is, if a household have a family size of ten, he will be given support for only of the five members. Is this to discourage birth, indirectly? Or else? Or is it to share the quota to all households in the kebele.

P: No, she laughs. It is not to control birth. It is because the quota is not enough. The safety net is a support that comes from outside. You cannot give to all family members. If you give to all of the 10 members, a household with five family sizes will die. Therefore this is to balance the support; it is not a birth control method. I have never heard of it so far that the government is doing this in the safety net program.

I: I do not mean it. My point is, do we have a political commitment to limit family size, for example, one can say the average family size should be 4 or 5; do we such kind of commitment?

P: To say “it should be four” such things are there. The government is trying and we are also trying. This is to increase awareness and thereby use family planning. But it is not acceptable to control family size by discouraging economically.

I: So, in your opinion, are these programs and polices effective?

P: Yes,

I: Do priest’ wife take contraceptive?

P:” eh’ she laughed. Yes there are some who takes contraceptive. Even the priests come voluntary with their wife, and they are taking. There are some who use contraceptive by hiding their husband. Now it is better, people in the rural want to plan the family size. But, this takes lot of efforts. Because of there is some change. Even in the rural, there is no anyone who does not us family planning. Rarely, those who are very stringent, there are priest’s wife who do not use contraceptive. However the awareness is better.

I: What are the community factors that affect age at first marriage?

P: Here there influence which is not yet solved. The influence if the parent is not educated is: now she tall and she will bring me a problem so she must be married. Because they saw some girls who have boyfriend and they do not accept it. So they think they would be humiliated or would be undermined if she is big. There is such backwardness. Then what they do, if she is tall and she starts menarche, pushing her to marry. They think they would be humiliated. This is common among the kebele leaders too. The men attach this with respect, they feel they disrespected if something taken. With regard to religion, if a girl is proposed by deacon, they think she should not get tall, and she should not menarche. There is such a kind backward thinking. Therefore they conclude she have to marry early during childhood.

I: what about the assumption that at least the girl will support her economically by her husband; when there is shortage of food in the family.

P: yes, there may be such kind of problems. But in my opinion, it cannot explain it. Because, those who are making their child marry are the rich ones. Even there is also wastage. Two things are missing. There is wastage, and the girl is hurting. In our zone, there is no way she gets married because of poverty. The reverse is true; the one who are not married are the poor ones. This is because; in the rural they will see if she has an ox, a cow or other things. Because of this he runs to the daughter of rich. Thus, underage marriage is common among the daughters of the rich. Therefore marriage for economic benefit cannot explain to our zone.

I: What are the policy factors that affect age at first marriage?

P: Regarding under age marriage it is put under the law. If someone marries his daughter at age less than 15 years, there is punishment?

I: Is it less than 18 or 15.

P: less than 18 year. And there is a law for it.

I: How is it confirmed?

P: This is the big problem. If you look them physically, there are thin, short girls, like what you have mentioned before. There are girls who seem they are 20 while they are actually less than 18 years. So when you look the physical, you cannot correctly know them. What we are doing is to send them bring medical certificate. Even the certificate is clear cut; it put it from this to this e.g.15-18 years. And this is very difficult for decision. Even when you go to justice you will get defeated.

Second, it is on the witness. In the kebele, there is Marriage committee. Then if she relative, they say this girl is born in ‘XXXX’ calendar year. Then they will make them (the girls) all above 18 years. Here there great gap. I do not know if another regulation/law is in place by government. With the existing law, we are always defeated.

I: what if the emanation is done at hospital?

P: Yes, they bring certificate from hospital. For example we all send them to hospital accompanied by police. Even the result from hospital does not tell sing number. Therefore here there are big problems.

I: can you think any other opportunities to prevent early marriage and increase birth spacing?

P: Regarding the underage marriage, it would be good if birth certificate is introduced to the community from now onwards, the previous ones, the have already escaped, all girls have birth certificate even now without telling it is for marriage.

I: What about the school certificated?

P: There is false age; it can increase or decrease. However, you relate it with religion, for example date of birth is available at church; because you cannot take evidence from church to law. Had such thing is introduced, and all females have certificate, it will be good. It is better if certification is done at kebeles. Be it their age is old or young, they have to have a certificate. Especially to those whom you expect they are underage.

I: what about building awareness at school?

P: Yes. Especially on the problem they can face after marriage.

They are given education at school; because there girls club. They teach each other. There is experience sharing at school from girls who face problem after early marriage using procures distributed by women affair. But, this is not enough. At kebele, there should a forum for out-school adolescent girls in the community, so that they can teach each other at that time. It can be at the health post in each kebele. It would be good if committee if formed from only adolescents under the supervision of women affair.

I: I have finished my question, and if you have any other comments that you want to add? Any lesson you want to deliver? You are welcome.

P: that is enough! There is nothing untouched. “Haha” she is laughing.

I: Thank you very much for your time and energy!!

**Summary**

**Section1: common maternal nutrition**

- When there is under nutrition, it does not mean there is no food in the house.
- In South eastern zone anemia is very common.
- Many women thought stunting comes from God, and some thought it comes from family.

**Section 2: Nutrition priorities in the Woreda**

- Women are not utilizing the food they have at hand; thus awareness creation is important.
- It is about killing a generation if we do not work on nutrition especially at stunting and thinness.

**Section 3: Nutrition interventions that improve adolescent and maternal health**

- In some kebeles, access to health facility is very difficult for pregnant women.
- There is no education/advice given separately for the out school girls; they sometimes may be given with development armies.
- In some kebeles, there are surprisingly large number girls who do go to school.
- At the health post, health extension workers demonstrate women how to prepare balanced diet inform of porridge.
- At some kebeles, because there is shortage of water, it is difficult for women to keep their personal hygiene.
- There is no deworming service for mothers

**Section 4: Implementation challenges and community factors affecting access to nutrition interventions**

- There is no nutritional screening for adolescents
- Lack of awareness, dis approval of husbands, religion and transport problem are among the barrier for women to utilize services.

**Section 5: Multi-sectorial collaboration to improve maternal nutrition**

- Working with partners is very effective. There is nothing you can work alone especially related with women affairs.
- There is no coordinating plat form in enhancing multi sectorial collaboration in Woreda.

**Section6: Other interventions that influence adolescent and maternal nutrition and health outcomes**

- Information is given by professional and media using radio to promote increasing birth intervals.
- To prevent underage marriage, it is good if birth certificate is introduced to the community.
