# Supplementary material for: Barriers to utilize nutrition interventions among lactating women in rural communities of Tigray, northern Ethiopia: An exploratory study
Source: PLoS One. 2021 Apr 30;16(4):e0250696. doi: 10.1371/journal.pone.0250696 (PMC8087028; doi:10.1371/journal.pone.0250696)
Supplement: S2 File — (ZIP) [file pone.0250696.s002.zip › S2_File.Doc/Woreda level and above key informants/147_IDI_Nutrition Expert at RHB_Tigray region.docx]

**In-depth interview guide for with experts** using the Guide for Nutrition focal persons (**Tool A**)

**Introduction:**

Hello my name is Haftay. I am from Mekelle University. Thank you for taking time to speak with us today. We are doing a research on factors that influence the nutrition of mothers and adolescent girls in collaboration with the regional health bureau and UNICEF. Your participation is very valuable. The things that you tell us will be used to improve nutrition programs and services for women and adolescent in the region and in the country. Your names will not share when we report our results.

However, I will record the discussion using and audio tape recorder so that we can capture all the ideas that are shared. I have several questions to ask you that we have prepared in advance, and we will ask you to say what you think about each question. To ensure the privacy of everyone here, we ask you not to repeat what to discuss outside of this group. The discussion will last for 1-2 hours. Do you have any question before I begin? If you think of any question as we proceed, please feel free to let me know. If it is all right with all of you, I will run on the tape record now.

**Section A: Interview details**

1. **Zone**: Mekelle
2. **Woreda**: Semen
3. **Kebele**: 03
4. **Name of key Informant**: Tsegazeab Hailu
5. **Institution of key informant**: Tigray regional health bureau
6. **Interviewer’s name**: Haftay Berhane
7. **Date of interview**: 24/11/2017
8. **Interview starting time**: 10:20 AM
9. **Interview end time**: 12:01 PM

**Section B: interviewee professional information**

1. **Gender**: Male
2. **Age**: 31 years
3. **Highest level of completed education:** Master`s degree
4. **Current job/Position:** Manager soketa declaration
5. **How long have you been in the current Job/position:** 07 Months as manager of Sekota declaration program but 5 years in TRHB

**Key:**

**I**: Interview

**P**: Participant

**M and E**: Monitoring and Evaluation

**Section 1: Common maternal (pregnant and lactating women), and adolescent girls` nutrition problems in the community.**

**I**: What are the nutrition related problems that are common especially in women including pregnant and lactating and adolescent girls?

**P**: Well. Actually, the malnutrition is a vicious cycle that starts from mothers, from adolescents. One of the important age segment in this cycle is the so called vulnerable which include women and adolescent girls. Thus, what we can see here is, as results of different studies and surveys showed, one of the malnutrition that commonly occur in these population is anemia. This anemia is seen widely. Another is the preparedness of their body as they are mothers; the adolescents are almost ready for pregnancy. We called adolescents for those aged 10-19 years. The pregnant and lactating women additionally have a child with them. When we see here, there is also nutritional problem in children in these population segment. For example, if we take pregnant and lactating mothers, during pregnancy, there will be stunting. Fifteen to seventeen percent of stunting occurs during pregnancy. When we see the prevalence of stunting regional and nationally especially in those under five years of age, it is very high. This is shown in different health demographic survey results that are done every five years. Therefore, when we see the malnutrition in women and adolescent girls, these are not the only problems. There are different health problems related to malnutrition. There is neural tube defect which is related to folic acid deficiency. It is known that such problems are reported nationally from big hospitals like Ayder comprehensive specialized hospital. Malnutrition is very big in these population segments. If you take adult stunting, mothers and adolescents of our country and specially our region, are stunted. They are also wasted. We have many mothers whose BMI is less than 18.5. Therefore, malnutrition in these mothers have many explanations. It could be related with fertility, educational achievement and hence, the consequence of malnutrition in these population segment can be explained in many ways.

**I**: Thank you. You have mentioned many problems that can be caused by malnutrition. You have also mentioned anemia as a problem which is one of the micronutrient deficiency. What about night blindness, and goiter in these population? Are these diseases common in women and adolescents?

**P**: Good. It is a good question. You have reminded me a good point. Micronutrient deficiencies are anemia as we have mentioned earlier. Goiter, which especially caused by iodine deficiency is usually seen in mothers. This goiter is common specially in the highland areas of Tigray. The problem is still expensively seen in women and adolescent girls because the insufficient supply of iodized salt, low behavioural change in understanding the importance of iodized salt to pregnant women, lack of awareness is not completely solved yet. Many studies showed that goitre that can be palpated is still common. This problem is highly linked with mental development. Iodine deficiency is one of the reasons that contributes to children to have low intelligent quotient which accounts for about ten points. Night blindness is caused by vitamin A deficiency. Of course, foods that contain vitamin A are not easily accessible to mothers and adolescents. There is also lack of awareness on the importance of vitamin A. Therefore, there also a problem in this regard. The micronutrition deficiencies are not properly addressed in every survey. As we can see as global indicators and global established facts especially in Africa and developing countries like Ethiopia, the problem is huge. As we can take visible signs like night blindness, there is huge problem.

**I**: How do you explain the lack of awareness? Is it because there is no education? What do you mean by lack of awareness?

**P**: Nutrition is not a matured discipline or science. Especially in developing countries like us, it is not long time that we raise the issue of nutrition security. Therefore, when we see the political focus, enabling environment like policies, research based interventions, etc, they do not have long time. Because, as it known, the government direction was food security. As we can see the conceptual frame work, from the malnutrition interventions, sufficient and accessible food supply or food security is one of essential elements. Therefore, we say this after short period of time off course beyond 8 and 9 years, the government and scholars was trying to establish the problem on the ground, nutrition security was identified as one of the big problems of our society. Thus, different nutrition specific interventions were applied especially for those under five years of age. Nutrition specific interventions that can be implemented at ministry of health and bureau of health at region level were applied. Those nutrition specific interventions which are mainly focusing with emergency related issues in under five children. When we say emergency, it is related to the management of severe acute malnutrition. This has been done by the government in collaboration of stake holders. It could be considered as a good move. Then, we can say activities related to development. We have said that malnutrition is a vicious cycle. Therefore, there are age groups that should be addressed here. One is at the age of adolescents, then at the age pregnancy and lactation, at the age less than two years, at the age under five years and preschool age children. The interventions are focused on children under five years of age and are mainly emergency related once. However, there is a huge gap in the other development works. We can even conclude as there is no activity started in this regard. The Ethiopian healthy policy is prevention focused. The health extension program which is considered as the flagship of the health system has been functioned for more than ten years. When we see the ten years implementation of health extension program, nutrition is one component. It is one of the components of family health. But, when we see here, there are no remarkable activities done in the developmental stage in mothers including pregnant and lactating and adolescent, pre-school and school except the activities done for the emergency related which mainly focused for the under five years children. Thus, the awareness about nutrition depends on the program maturity, and the width of the interventions. Off course, you cannot say there is totally nothing done. But, one of the problem here is, there are limitations even on the interventions that are already started, in organizing and preparing a doable social behavioural change communication tools that can change the awareness of the society. There a big gap the strategic NBCC or Nutrition behavioural change communication tools. Therefore, the intervention on creating awareness of the society at low level including the mothers at household level is minimal. The next is the educational level of our mothers. Forty-seven to forty eight percent of mothers living in rural areas are illiterate. These mothers are illiterates who even do not start grade one and this indicates that there is high illiteracy rate. This has also its own impact in behavioural change communication. This is one of the barriers in our interventions that can hinder mothers to grasp the content of the awareness so that they can bring behavioural change. The other is the gender value. In Ethiopia as well as in Tigray the society is paternal means it is male dominated society. There are gender values in the society itself. It does not need to define gender for you. There are many gender issues that are contextualized in each the society. Therefore, this will negatively affect mothers. Most of their time is spent on work. They do not even eat the food available at household level alone. These things have many implications. Therefore, the awareness on nutrition was affected by males’ involvement. The other thing that should be mentioned here is, related to religious organizations especially orthodox. If you take Tigray as a region, more than ninety percent of the population is orthodox in religion. There are around 266 fasting days. This is off course a religious issue. We as a government, as a stake holder need to do something. Government is a secular but the impact of fasting is huge. If we for example take one pregnant mother, out of the 266 fasting days almost in the nine months, even if she has the food at home, she will not allow to eat animal source foods. Most of the nutrients in nutrition are found from animal sources and there are many important nutrients. These animal sources are better nutritious that can help herself and the fetus than the fasting foods. Therefore, this is a hug problem in pregnant and lactating mothers. When we say fasting, one, we can raise it with animal products. The other is, when we say fasting, mothers do not eat even the fasting food itself because it is fasting. Therefore, the involvement of stake holders that can improve the nutrition of mothers and adolescent girls is limited. In general, the actions taken for awareness creation is not remarkable because of the program is new; we do not use those tactics or methodologies that can create awareness properly; immaturity of BCC (behavioural change communication) material; and low women literacy or high women illiteracy; cultural and religious issues. By the way, when we say awareness, there are limited actions done on creating awareness even on the literate people on nutrition as it is newly developing science. Thus, all these are the factors that hinders the nutrition intervention on mothers and adolescent girls. Therefore, nutrition interventions could not grow fast programs such as immunization. Immunization is one of the successful public health interventions in history. Immunization has long age and has better performance in the things that we were mentioning above like advocacy and the SBCC tools are well organized and developed compare to nutrition. It is to mention that leave alone with mothers, there are many things that are not done by the higher bodies at program level.

**I**: Thanks. You have tried to relate nutrition with food security in the above discussion. But, how do you see the food security currently in our region?

**P**: When we say food security, there are methods that we use. What do we mean food security? Food security has at least three pillars. One, when we say food security, it is the availability. Availability of food. The next is the access. If it is available, we should see whether it is accessible to mothers and adolescent girls or not. We must look for accessibility. And then, the next is the utilization. Therefore, when we say food security, we should look for availability, accessibility and utilization. When we see these pillars, and when we see specifically related to availability, the level of poverty in Tigray is 27% as it is indicated in the recent study done by central statistics agency. This is studied at household level. This indicates that 27% of households in Tigray are food insecure. This means, mothers in the 27% are having shortage of food availability. Mothers out of this or those who are food secured, even if there is food in the house hold but it is not accessible to the mother or the adolescent girl because of gender values, food taboos, fasting and religion etc. it could also affect the child but since our focus is mothers and adolescent girls, there are factors that affects the access to food. When we go to utilization, we are saying as there is lack of awareness even if they are food secured. A pregnant mother should take two more dishes than the normal dish. An adolescent girl will not know what to eat, what type of malnutrition will occur in this age, while this age is critical for her to be ready for marriage. As an adolescent girl, she may develop anemia. This could be related to malnutrition, menses and puberty. Therefore, she needs carbohydrates and iron containing foods. There is lack of knowledge on which foods at which age group and this will affect the third pillar which is utilization of food. This is about food security.

**I**: We were talking about what a malnutrition especially under nutrition can cause. How do you see overweight on pregnant and lactating mothers and adolescent girls?

**P**: When we say malnutrition, you are right, it needs definition. When we say malnutrition, it includes over and under nutrition. Therefore, malnutrition is divided in to two; undernutrition and overnutrition. It is important to mention why we are giving focus to under nutrition. We as Ethiopian, as Tigrean, our big problem is undernutrition. If we say why, we are having a problem with availability, access and utilization. Off course, the overnutrition also are linked with this but it is caused increased amount and size. However, we have low prevalence of this problem. Malnutrition is one of the major public health problems in our case. But, we should also see overnutrition. When we see overnutrition, according to EDHS 2011 report, the prevalence in mothers and adolescents is almost 4%. I am not sure about the prevalence of overweight in the EDHS 2106 report. However, we should not also for get this overweight. As policy, you must prioritize problems. Interventions are done by prioritization in every country. Therefore, undernutrition is prioritized by the government and stake holders. We are now at transition. Almost, the food insecurity and the unemployment is being solved. Since our society is in transition from poverty to medium income, the above mentioned overnutrition problems will happen. They should not forget this problem. When you see it currently, this is not a big problem of our society and especially these women and adolescent girls.

**I**: Do you think diet related non-communicable diseases like hypertension, diabetes mellitus, cancer etc are common in women and adolescent girls?

**P**: Actually, nutrition is one of the causes of cancers especially colon cancers as established facts in developed countries. It is also known that malnutrition especially overnutrition is highly linked with the diet related non-communicable diseases like hypertension and diabetes mellites. But, I think there are no nation wise or region wise studies that leads to established facts that showed these diseases are present in the population segments, women and adolescent girls. However, as general population, these problems are linked with life style. When we say life style, it can be linked with physical exercise, stress etc and it is also related with alcoholism, smoking etc. When we see in the age category of these population, including women and adolescent girls in relation to these problems, which cancer is related to diet, is the hypertension because of diet or others is a next homework to scholars. But, hypertension is not easy in pregnant women. Otherwise, you could not talk confidently about these problems in these segments of the population.

**I**: Well. As we can see it, we have talked about the priority intervention is undernutrition. What are the nutritional interventions done on adolescent girls and women until today?

**P**: When we see the interventions currently available in adolescents and pregnant and lactating women, let`s start to talk about interventions done for adolescents. There are no interventions started for adolescent girls. But, the absence of these interventions does not mean that it is not possible to do activities related to prevention. But, it is to mean that we did not start these interventions. There are activities that should be done here. One is women empowerment; off course, this is being done. When we say women empowerment, it is related to education, enrolment rate, gender parity index, I mean what does the sex party index looks. There activities done related to education. There are no other interventions that empower adolescent girls such as economic empowerment by giving meaning to nutrition by organizing them separately as they are the base of the next generation. There are another cooperative. But, there are no programs that organizes the adolescent girls focusing on nutrition to work together in producing, improving their nutrition, and improving the nutrition of the coming babies. The current associations and cooperatives are mainly focusing one intergeneration. Income generation and nutrition security are different. Therefore, there is a need to twine these things. As international nutrition recommendations, specific nutrition interventions like iron folic acid should be given to adolescents every week. This means, they should take a tablet that contain iron and folic acid every week. This one intervention. Therefore, this is given in school based. When we see this, off course, it is not started yet. It will be started in one or two wereda may be this year. Off course, near Raya Azebo area, but it is not started yet. If there is assumptions that shows the presence of this intervention, it is the school deworming. Adolescent girls in school specifically and all students generally are provided with deworming medications every year based on the load of intestinal parasitosis studied. There are no other interventions in adolescent girls than this intervention. Here, attention to the current cooperative association to focus on nutrition in its organization should be given. For example, if there is one cooperative of females that produce milk, there should be a way that these females should be benefited from the milk. It should be organized that these females should take certain millilitre of the milk every day and by selling the excess milk to grow their income on the other side. In this cooperative, the current income should be linked with their own nutrition. There may be different cooperatives. When we see iron folic acid supplementation, the prevalence of anemia currently is increasing alarmingly. There is a need to think about what should be done when it increases alarmingly. It may need to mobilize resources and establish evidences. Therefore, it is difficult to say that there are activities done in adolescent girls towards this. When we take, pregnant and lactating women, there are better interventions. From these interventions, one is the nutritional assessments and counselling services for pregnant and lactating mothers. This starts at the antenatal care. When they visit the health facility for antenatal care, they are provided with nutritional counselling. There is also supplementation of iron folic acid for six consecutive months from the starting date. There is also nutritional screening every month. This nutritional screening is done by health extension workers by putting MUAC measure on their upper arm. Based on this screening, women will join and benefited from the targeted supplementary feeding program. This targeted supplementary feeding is limited to limited area and it does not cover many areas. When we see the upper arm screening, we cannot say every pregnant woman is screened. This is to indicate that the program is not strong enough. It is only 20-21% of mothers who get screened and sometimes this may reach up to 30%. This is therefore to indicate that, the program is not strong but the intervention in pregnant and lactating mothers is somewhat better. The other is, the pregnant and lactating mothers are members of the lowest administration in the community which we call it women development army and they are taking educations on awareness creation. They will teach other on what should a pregnant woman eat, and what problems she may face. Related to institutional delivery, there is a better work here. There is also a plat form for pregnant and lactating in health post, health center. But, in school adolescent girls may benefited. However, the school health and nutrition is not strong. Therefore, we can take the interventions in pregnant and lactating mothers is better because of the presence of delivery plat form, since the service is given with other related services like with antenatal care, delivery, postnatal care.

**I**: Well. You have told me as deworming is given to school adolescents. How do you think we address the deworming for those adolescent girls who are out of school? How do they be benefited from this intervention?

**P**: Yes. It is a nice question. They are not being addressed. The intestinal parasitosis of our wereda is different. Therefore, this deworming is given in schools based on the load of this intestinal parasitosis every time. Those that are out of this are missed. Therefore, there is no other way to reach the out-school adolescents. Even if their number is significant, they are not getting this service. They are not benefited from this intervention.

**I**: Ok. You were telling me that the nutritional screening in pregnant and lactating women is low. Do we have opportunities to scale up this intervention? Or if there are future direction on this?

**P**: When we see the coverage of screening of mother in every month, it is not remarkable. But, there are delivery plat forms. One, when we think of pregnant and lactating mothers is it is not given separately as nutrition. When a mother comes to health facility to take essential health service packages, one is nutrition. Therefore, a mother should visit the health facility for antenatal care a minimum of four time. But the screening is expected to be done every month. This is to follow what the mother`s MUAC looks like. This is not accurate indicator for pregnant women, but proxy indicator instead. When you compare the MUAC of a first trimester pregnant with third trimester pregnant woman, it is difficult to decide. This is related to the measurement itself. Off course, the measurement lacks accuracy and validity. But, there are many efforts done to be done in agreement with the other programs like ANC, delivery, PSC, immunization, etc. But, awareness on nutrition even in the front level health worker which health extension worker is low. The health extension workers do not sense the importance of MUAC measurement. The other is related to work load. When we say work load, since the program is new even if it is essential, they consider it as additional work. These the factors that have their own role in impact on the strengthening and increase the status of nutrition screening program. But, many activities are being done towards the strengthen of the screening program in coordinating with other programs.

**I**: You have been mentioning that there could be gaps in utilization. You have also told me that pregnant woman should take two extra meal than the normal dish. How do you think this taking extra meal in practical scenario? Do mothers take extra meal during pregnancy?

**P**: There is education on extra meal that a pregnant mother should take two extra meal over the normal meal, a lactating woman should take one extra meal over the normal meal. The advice is off course, being provided. But, when you see in the ground, one, our routine monitoring mechanism cannot capture this. These should be obtained by kind of surveys, operation researches. Otherwise, beyond giving general opinion, it is difficult to the exact number of women that eat their food based on the scientific way while they are pregnant and during lactation.

**I**: In taking extra meal could you explain it more by linking with the above-mentioned factors like religion, male dominance, and others? For example, since there is male dominance, pregnant woman may not eat in the absence of her husband; during fasting leave alone the extra meal, she may not eat the normal meal. Therefore, what is your observation on taking the extra meal during pregnancy and lactation in general terms?

**P**: Taking extra meal may be measured by current mothers BMI. I may not exactly remember the figure in the study result of 2016, we can it from that study. The other is the level of stunting in babies. We can take stunting in children is decreasing from time to time. From the past sixteen years from 2010 G.C until this year, the prevalence of child stunting is lowered from 55% to 39%. Thus mothers` diet also has its own contribution as 15-17% of stunting occurs in the womb. You can decide by looking at the trend whether the extra meal taking habit is improving or not. But, still it is difficult as an expert to say mothers are taking their meal based on the counselling service they are provided, until the awareness on fasting is not known and the current awareness is not known. In the rural area, how many mothers do you think can eat before their husbands even if they are pregnant and lactating? Therefore, during fasting how many mothers do you think will they eat the available food at home? If mothers are having hen, how many of them do you think they will eat one egg during the great fasting? You cannot therefore take this as solved. The problem is there.

**P**: How do you see the food diversification in pregnant and lactating mothers grossly? Do you think pregnant and lactating mothers get counselling to use food diversification?

**I**: When we say diversification, thinking that those who live in rural areas as farmers, they harvest different crops. However, mothers are not eating diversified food. The reason is, there is lack of awareness. When we are saying awareness, it is related to which crops contain what. When we take food groups, there are those that give energy, prevent disease, body builders, those that give heat etc. Therefore, most of our mothers can take, if they get, the food that can give energy. Because most farmers harvest either sorghum or barely or wheat etc. Thus, all the cereals are energy givers. May be the body builder which is out of the animal products such as meat and other animal products are legumes. These legumes are consumed in the form of stew. There is limitation in taking these legumes that specifically added to our food to make diversification without in the form of stew. Live alone for mothers, there is problems in giving complementary feeding for children that are nutritious and diversified. Therefore, one, the extension activities must be strengthening, since there is a problem in the agricultural extension. If food is to be diversified, the current agriculture should also produce diversified products. For example, there are irrigation cluster areas. Farmers must harvest diversified products. And then, they should also eat diversified food as a family. Therefore, when you take food diversification for mothers, there is a big problem. There are challenges that affect the food diversification beyond that of awareness. There is a problem in introducing biofortified crops that can be ready for diversification based on the agroecology that can supplement the nutrition deficiencies. When we say mothers should diversify, we should also solve challenges that can affect the diversification. Therefore, there are food so called biofortified like orange flesh with potato, quality protein maize, iron rich bean etc. The livestock activities, especially poultry, small ruminant, should be accessible to mothers in the eye of nutrition by targeting them in each area and then diversified their food. The activity of sectors is also one barrier. Therefore, all these are problems for food diversification.

**I**: Is there intervention done in home gardening?

P: Off course, it is difficult to know how many households are having home gardening in practical scenario. But, as intervention, agriculture is one of the activities that perform better. There is a problem in this regard, too…smile…When we think of home gardening, how many of our population have access to water. Therefore, there is also a problem here. We have been doing home to home visit in the past ten to eleven rounds and there are home gardens in few areas. When we see how many of rural households have home gardening or back yard gardening that is ready for nutrition purpose and preparation of diversified food that will help mothers and children is questionable. Even, when you see if it is successful with the current lack of water, needs attention. Here, there is a need for customizing. In which area should home gardening be introduced and in which area which intervention to be introduced. Sometimes we will be biased in that if an intervention is successful in one applicable area, you can not apply it in overall areas. If we take Tigray, it is highly affected by lack of water. When you think of home garden as my perspective, there is a situation that you will be difficulty. This is because, mothers are fetching water with jericans on their back from rivers. Therefore, it will be difficult for them to do home gardening or back yard gardening by fetching water from rivers. It will be a problem to garden by fetching and consume to themselves. Therefore, helpful technologies should be introduced and areas should be selected. If you see the work done on this regard, it is not significant.

**I**: Well. How do you see the work done on WASH; hygiene and sanitation especially on mothers and adolescent girls? This hygiene and sanitation may have direct or indirect relationship with nutrition and how do you explain the works done on this regard?

**P**: Ok. When we say WASH; sanitation and hygiene, actually, it has a direct relationship with nutrition. When we see this in perspective of pregnant and lactating mothers, the main problem here is not focusing the vulnerable groups. There are interventions on open defecation free program. ODF if one of the health transformation agenda of our country. It is a part. It is one of the indicators of a modelling. The problem here is not working with vulnerable groups in focus. Here, what you do is, you will calculate the reports on kebeles that do ODF, Households with latrine, latrine utilization. You will not specifically know how many pregnant and lactating women from these households who have latrine are utilizing the latrine. It is also difficult to know how many of these women from those households who have hand washing basin are utilizing this hand washing basin. Therefore, the interventions are not yet addressing the vulnerable groups. You will only see a gross. If you see WASH activities, it is possible to get reports on what the latrine coverage is and utilization in Tigray, with the false reports we have as a problem. When you come to households with pregnant and lactating women, you will not get it. Thus, the WASH activities in these pregnant and lactating women specifically will not be clear except as a general population.

**I**: Some parts of Tigray are malaria endemic. Are women including pregnant and lactating getting and adolescent girls get advice on the need to use Insecticide treated bed nets?

**P**: This is also similar. Bed net distribution is done in surveillance. There will be a report on how many bed nets are distributed to the general population. But, there is no focus targeting to these population segments including pregnant and lactating mothers, adolescent girls, the same with under five-year children. Service qualification is not yet done. There is limitation in qualifying the service and addressing the vulnerable group. You will get difficulty in getting information on how many of the pregnant women are addressed with bed net distribution, from the malaria endemic areas in Tigray. In general population, Tanqua Abergele is a malaria endemic area. Then you will see how many households in the wereda are distributed with bed nets but it is difficult to get the number of the pregnant and lactating mothers getting these bed nets and those that do not get these bed nets from the wereda.

**I**: How is the youth friendly services at health facilities going on? Are adolescent girls linked to this service?

**P**: Our friends here may better explain about this youth friendly services but I was also part of the program. Youth friendly health service is currently provided in different places and it is given special emphasis by the ministry of health. It is given emphasis because it contains the large segment of the population. There are many activities done in youth friendly health service. I doubt whether nutrition is raised as a point in the youth health service. When we say youth friendly health service, there is education about early pregnancy, STI etc. This has its own input. But, in youth friendly health service, it does not focus specifically on adolescent girls’ nutrition. Anyways, youth friendly health service is existing.

**I**: Which of the above-mentioned interventions in women and adolescent girls like deworming service, Targeted supplementary feeding, vitamin A supplementation, use of insecticide treated bed nets, iodized salt utilization, do you think are most successful? Can we classify them in to successful and less successful?

**P**: Deworming is not yet started. Maternal deworming is there in the national program strategy. But still, it is not started due to different reasons including budget. This is not started activity. Vitamin A supplementation off course, was given in postpartum but currently it is not recommended. We, as implementers have suppressed this intervention based on the evidence from the WHO recommendation reported before two years. It does not have effect resource wastage based on the recommendation given. If you take target supplementary feeding it is better from the wereda that have the intervention. Actually, the problem here is we do not have robust M and E or data management system on nutrition. Targeted supplementary feeding is given in areas where there are donors in agricultural bureau and rural development. And then, even if the work to be done is in coordination between health extension and agriculture, there is a problem in implementing the work of both sectors in a meaningful way. But, in the target wereda which we called them hot spot one by different parameters, you can say, targeted supplementary feeding is better performer.

**I**: We have seen some challenges that hinder the implementation of nutrition related interventions. What other challenges do you think are there that hinder the implementation of these nutrition related interventions or programs?

P: One of the challenges that hinder implementation of nutrition related interventions is the structure of the implementation itself. This structure is weak. The structure at each level is not accommodative to nutrition professionals. When we see nutrition, it is mostly found in one program, off course it is a big program, which we call it maternal health. Therefore, one of the problem is related to structure. To undergo the nutritional interventions, when we see the malnutrition conceptual frame work, it touches multisector. This is because, it has different levels; basic, underline, midline, direct. Therefore, these touches different sectors. The common nutrition interventions in multi-sectoral coordination`s plan is not equally distributed between the sectors. The role of health in nutrition is very minimal which is around 20%. This role is even given if the interventions are executed at 90% scale level. The other sectors handle 80%. Therefore, the structure is one of the problems as I mentioned earlier. Therefore, the absence of a strong structure in each sector is a problem. And then, there is a problem in addressing the nutrition interventions for malnutrition in common. As we have been discussing before, WASH should be targeted to pregnant and lactating mothers. Targeted supplementary feeding should also should also be addressed to this population. Gender issue should be addressed. Male involvement should be addressed and safety net program should also be addressed. The above interventions are touched by different sectors. When there are different sector involvements, there is a problem that these sectors do not come onboard together to work on it. The other problem is the nutrition governance. When we say multi-sectoral nutrition governance, one is fund, which is resource mobilization. Therefore, there are many interventions that are not implemented. The development works done on mothers and adolescent girls is very weak. In fact, there are limited activities in the development work of under five children. Therefore, this needs fund, resource mobilization and harmonization. Budget should be allocated. There is no budget allocated. It is donor based. It is good, donor is important but the government should allocate budget in each sector. Budget is always limited but there should be budget allocation based on the resource you have. Therefore, in the nutritional governance, there is budget. There should be resource harmonization which could be related with the multi-sectoral activities. There other is the M and E system. There is not robust data for common plat form. Therefore, there is no common M and E plat form that shows who works what. These are big problems. They are barriers that hinders the development of nutrition forward. The other thing related to nutrition governance is the number of professional. The number of professional is very limited. You may get one or two nutritionists at regional level. But at wereda level, there is problem in human resource development who has knowledge of nutrition and can stimulate the structure in that area. There is shortage of professional. When we say professional, it does not only mean that nutrition needs in MSc, level. But there should be nutrition professional at diploma level, at degree level. When we see other African countries like Rwanda, there is human resource in diploma level and degree level. These individuals are deployed at each level. But, when we in our case, individuals are graduated with MSc in nutrition from different disciplines and then you will get them somewhere at regional or federal level. Therefore, the professional is also a big problem. These are the barriers that affect the nutrition intervention activities not to move forward.

**I**: Well. We have been talking the barriers at program level. Do we have barriers at individual level and at community level that hinders the implementation of the nutrition related interventions?

**P**: Could you elaborate it more, please?

**I**: We have been mentioning that because of lack of awareness, the utilization could be low even if the system is there. Therefore, could there be other individual related or barriers at community level that hinders the implementation of the nutrition related interventions, or utilization of the service, may be related to community beliefs, etc?

**P**: Those are the things that we have been described them above but one is the lack of awareness. When we see the prevalence of malnutrition in those who have food and those who do not have food, it is equal. Therefore, it is not a matter of being food secure or food insecure. When we compare the two at community or household level, different studies showed that the prevalence is almost the similar. This means, the problem is lack of awareness. The others are related to religion and culture. As culture in one family, if you take the case of you or I grow, there is no consideration that feeding is very important to the future of the individual. Child is growth is dependent on what the child eats. There is no one who thinks that a child`s ability to critical think, ability to produce, ability to innovate is dependent on the current feeding of the child especially at the age of less than 2 years and specially what the pregnant mother eats today during the one thousand days will affect the child future development. It is already a culture. Eating what is available. Whether a pregnant or a child will eat what is available. This is the culture that should be cut. These are the big problems.

**I**: We have raised the issue of food taboos in our discussion. Are there food taboos that are known for pregnant, lactating or adolescent girls?

**P**: You can talk food taboos from the area you are well familiar. I can assume that there may be different food taboos. When we see in urban areas, pregnant women are not allowed to eat more food because they assume that the fetus will be big enough to be delivered and hence, the mother will face a problem during delivery. The other food taboo is, mothers are not allowed to eat eggs especially at the western part of Tigray.

**I**: Why is that?

**P**: This is gender constructed issue. Thus, it is prohibited. Animal products are not allowed to be taken by female considering that the females will look for sex with individuals other than their husbands. They consider these animal products as sexual triggering factors. These are the food taboos.

**I**: What about with adolescent girls?

**P**: There are no food taboos that I clearly know in these girls but I think there are food taboos. I do not have basic study result that shows food taboos in each area. But, when you go visits for different activities, you will observe different food taboos. I have already told you about the food taboos in western part. I do not know whether these are food taboos or not but there are related with pre- lacteal feeding. There are practices that are done for new baby before it starts breastfeeding especially, related to colostrum. They consider it as a dirty liquid and remove it first. Despite, it is very import but removing the colostrum considering a dirty fluid is practicing. These things could be considered as food taboos.

I: Well. We have mentioned many challenges at individual level. For these challenges that you mentioned, what does your institution do to solve this challenges or if there are future direction to solve the challenges?

**P**: There many improvements by the government to solve the problems related to malnutrition such as program revision, strategic development etc. The government has avowed a sekota declaration that aimed at solving the problem especially in pregnant and lactating mothers as well as children less than 2 years. This is aimed at terminating the problem by 2030. There are many works done to realize this aim. Works related to creating enabling environment, establishing food and nutrition policy starting from federal level, human development in nutrition with structure and salary arrangements by ministry of health. This structure and salary arrangement is already endorsed by the government. The Ethiopian orthodox has endorsed a ceremony guide by the year 2015 which is related to fasting. It dictates that pregnant and lactating mothers as well as children under 5 years of age should not fast. To facilitate this, it is distributed to every master head of a church so that every kebele and every holly father will teach each pregnant and lactating woman. The work is already started. We have mentioned that nutrition is a multi-sectoral activity. Therefore, especially, health, agriculture, water resource, school and social affairs should prepare a plan that is should be intervened by each sector on the nutrition of pregnant, lactating women and under two children. These sectors should also plan for budget. The government is also allocating budget to solve the problem of governance. There is a specific budget for nutrition. Not only budgeting but it should also have budget line with specific code. It will also help how much the government has invested in nutrition. This will also help in resource trucking and cost mapping. Therefore, there are activities towards this. The other is, on the issue of M and E common plat form as well as robust data management. Stake holders like UNICEF, ministry of health and sekota declaration has developed a data management called UNIS which is unified nutrition information system. There are efforts to finalize this data management tool and to expand to the lower levels.

**I**: We have tried to describe the multi-sectoral collaboration in our discussion. Do you feel it is necessary to include other sectors than the five sectors you mentioned above to address maternal and adolescent nutrition?

**P**: By the way, all the sectors and all ministers are called line ministers in the national nutrition program. They are implementing ministers. Those are all the ministers at federal level and then down level to the sectors here. Therefore here, there are sectors like sport, women affairs, youth affairs. There are also other associations than the sectors mentioned including farmers association, religious organizations network etc. If we take all the sectors mentioned, they are implementation sectors. They are signatory of the national nutrition program. But, sometimes if there are many sectors, it is very difficult for the pan across management. The five sectors are chosen based on the multiplier effect of the sectors. Every sector has contribution but you must choose which sector has more multiplier effect than the others. Thus, is a prioritization. Therefore, it is good to prioritize and strengthen the multi-sectoral collaboration by induction. These sectors are chosen to induce other sectors. Otherwise, all line ministers are implementation sectors in the national nutrition program. All sectors at federal level, down to all sectors here, sectors in wereda, kebele are implementation sectors.

**I**: Well. How do you see the current level of collaboration? How do you evaluate the current level of collaboration especially in improving maternal and adolescent nutrition?

**P**: We can see the level of collaboration by categorizing in some points. When we think of the current level of collaboration, let`s see it by thoughtful of multi-sectoral governance. When we see the intention of government in terms of multi-sectoral coordination thoughtful, we can take it is very strong. We are saying it strong because, starting from 2013, it is found to be effective in launching the need for multi-sectoral intervention, signing all line ministers to be signatory. Then, the document is revised to install accountability by including the roles and responsibilities of all line ministers. It includes the roles and responsibility of all sectors. Now, when we see the thoughtful of the government for multi-sectoral nutrition governance, it is very nice. It will also address the food and nutrition policy, school health program. School health will be started. Therefore, when you all these things, you can consider it as a good thoughtful.

The other point in collaboration is the communication. Let`s see communication alone and communication and accountability together. When we see communication, there is horizontal and vertical communication. In communication, the multi-sectoral coordination has prepared schedules for meetings. There is a technical committee at federal, regional and wereda level. There is a big problem in communication between the nutrition coordination body and the technical committee. Meetings are not done based on schedule. When meetings are not done on schedule basis, there will not be communication from the federal, regional wereda line. Therefore, there is missing element in communication.

When we say accountability, there is a working guide for accountability based on the roles and responsibility of each sectors. There is no measure who do what. The roles and responsibility of each sectors are there and there are also outcome indicators. Therefore, there is no measure on the indicators. It is not known where the sectors are in terms of outcome indicators, output indicators and impact indicators. Off course, when we measure the impact indicators, we come to stunting, wasting and underweight and then to BMI, anemia etc. This is indicated by who contribute more which could be measured by the output and outcome indicators. But, you can measure the effort of all sectors by impact indicator because, this indicator is comprehensive indicator. Therefore, there is still a problem in communication and accountability.

The third category that should be seen here in collaboration is related to robust data management system. As I have mentioned earlier, when we think that these sectors have their own jobs, their own measurements, each sector should have a system that will help them in manging their data. Not only data management at each sector, but they should have also a common plat form. There is also a big problem in this regard. Therefore, the coordination status seems like this based on the three points.

**I**: You have told me some points about early marriage. What would be the effect of delayed marriage (after 18 years) on improving maternal nutrition?

**P**: Early marriage has significant effects. If there is marriage before 18 years, it will have negative effects on the mother. For example, if 16 years old girl is married, she is already a mother. The body maturity of a girl is like a land prepared by a farmer to sow a crop. I am, fortunately, is a farmer boy. If the farmer wants to sow a crop, he should prepare the land carefully, ploughing it two to three times. Similarly, the body development of a girl should be ready for pregnancy like that of land prepared by the farmer. Therefore, if a girl is having pregnancy before the normal time she should get pregnant, her body is not ready for pregnancy. If a baby is conceived, there is no fertile environment for the baby to grow well. The other is psychosocial effect. When you say this, the girl may develop fistula (breaking of uterus) and the psychosocial effect is related with these problems. A mother will not think about breastfeeding and child caring being on this kind of problem. Since the body of the girl is not matured, there will be many problems that may occur during labour. Even she did not have maturity for child caring. In most of the time, child caring is done by mothers. Even if she gives birth peacefully, there child caring will have its own effect. Therefore, early marriage has its own effect. It is one of the reasons for the viscous cycle of undernutrition in maternal and child nutrition.

**I**: What about child spacing? What is the effect of increased the space between each birth in improving maternal nutrition?

**P**: Child spacing has a big meaning in malnutrition. This is because, when a mother gets pregnant, her body condition will be changed. When she breastfeeds with energy she has, weight she has, it will be a burden other activity of the mother. Thus, if a mother give birth ever year, let`s say it every two years as it does not have problem, it will have an impact on the child and her own nutrition when compared to a mother who give birth every four two years. This because, when the mother with spacing is giving birth, her body will not be harmed. She will grow her baby well and as we have seen what the farmer do to his land, her body will get ready for the next baby with a good weight and enough rest. The baby born from this mother will have a good nutrition. Therefore, when we say birth spacing, it should also be related with number of children. Since we are talking about food security, it will be linked with number of children at household level. When we compare a mother with five children and a mother with one child, the availability, access and utilization of food will not be equal. Therefore, this is linked by many ways. The other, is related to the mother`s family caring time and energy. This because, as the mother gives birth every time and as the number of child increases, she will spend much time and energy in caring her children. How many jerican will the mother fetch for drinking if she has seven to eight children. If you ask yourself how many times will she bake injera, how many times she prepare sticks for fire, it will have a big impact with the family size. Therefore, this has many impacts. This is related to family planning which will affect the family nutrition. The difference between households who control their family size and those who do not properly control their family size have a visible difference.

**I**: What opportunities do we have to promote child spacing and preventing early marriage?

**P**: If we see the opportunity we have, the early marriage has a law protected. There are working elements in every level. There is an established legal issue related to women affairs. If you asked yourself, whether the intervention is effective because of the legal issue, it is another issue. This to mean that, the first thing that should be done is to change awareness of the community. Otherwise, if a 15 years old girl is going to be married because of the need of the family or her father, he may let her marry by claiming as she is 18 years of age. The after, the women affair may accuse the family and them it will be seen by court etc. But there will challenges here. This may happen since the girl`s family wants to create a bond with the husband`s family. Since the awareness of the community is not changed, and they may assume that the girl may be mature enough, may commit sex. These are manifestations of the backward thinking. But, the good thing is early marriage has legal protection.

But, with family size, I do not know whether it has legal protection or not. As the government is democratic, I think it is not difficult. There is no child policy, too. Then, what you should do is, there should be awareness creation as this can negatively affect maternal nutrition. Because most children are born by without need. If you see it by the way, this child is born with or without knowledge or mistakenly. Otherwise, it does not mean mothers will get tired to give birth every year, grow carrying the child for two years. It is also difficult with the work load they have at home. But, there is need to create awareness. We have a family planning services in every area. Thus, it should be strengthen taking it as opportunity. Family planning in Tigrigna means is not controlling births but it is planning for number of births. When you plan you will take in to consideration the number planned number of children. Therefore, when we talk about planning, it depends on the income and ability to grow the number of children you planned. When you plan, you should consider the nutrition of the mother and the coming baby. When you create such thinking in the community, the leader of the family will consider the effect of malnutrition on the health of the mother and the baby. Therefore, it is important to create responsiveness on maternal and child nutrition in the community. The family planning methods should also be considered in planning. These methods include short and long acting family planning methods. But, know, we must increase the coverage of the long acting family planning methods utilization. Therefore, it is important to use this opportunity. It important to use the family planning methods properly. It is important to understand the dogma of religious leader on child bearing. These leaders should be involved in family planning as stakeholders. This is because the community we have is following a religion for long time and some part of the religion is becoming a culture. Therefore, it is good to properly involve these religious leaders at every level of the church including master head of churches, priests especially in family planning.

**I**: Thank you. May be last point. Do you have any ideas that should be included or that should not be missed on the area of maternal and adolescent girls` nutrition?

**P**: Off course, we have discussed many things. Since adolescents and mother are the foundation of the tomorrow`s generation, focus should be given on the maternal and adolescent girls nutrition. The current screening and prevention interventions should be strengthening. As you have told me in the introduction, promising operational researches that can influence policies should be done focusing on the maternal and child nutrition. This is because, a country can be changed by generation. Hence, the first thing in doing this is creating a citizen with a well-developed mind. The fountain of the next generation today is the segment of the vulnerable group. Therefore, the interventions working around these should be strengthened.

**I**: Thank you for taking the time to discuss these issues with me today. I have learnt a lot from you. As I mentioned as the start of the discussion, I will remove all identifying information from the report of this conservation. I will make you sure that no one can identify your comments. If you have any concerns or questions, please feel free to ask me any questions. Thank you very much for your time. You cannot say everything is clear and applied by the community but there is improvement from time to time.

**Summary:**

**Section 1**:

**Section 2:**

**Section 3:**

**Section 4:**

**Section 5:**

**Section 6**:

**Section 7**:
