# Supplementary material for: Barriers to utilize nutrition interventions among lactating women in rural communities of Tigray, northern Ethiopia: An exploratory study
Source: PLoS One. 2021 Apr 30;16(4):e0250696. doi: 10.1371/journal.pone.0250696 (PMC8087028; doi:10.1371/journal.pone.0250696)
Supplement: S2 File — (ZIP) [file pone.0250696.s002.zip › S2_File.Doc/Woreda level and above key informants/148-IDI-Nutrition expert_Regional Agri and rural development Bureau_Tigray.docx]

**In-depth Interview with Nutrition extension expert in Tigray Region Agriculture and Rural Development Office**

Zone: Mekelle

Woreda: Semen Kebelle: 05

Name of Key Informant: Fitsum Abay

Institution of key Informant: **Tigray Region Agriculture and Rural Development Office**

Name of interviewer: Measho G/slassie

Date of interview: 23/11/2017

Start time; 3:00 PM End time; 4:39PM

**Interviewee Professional Information**

Age: 45

Gender: Female

Highest level of completed education: Bachelor degree

Current job/position: Nutrition Expert

Work experience in the current position: 4 years

**I**: Interviewer **P**: Participant

**Section 1: Common maternal and adolescent nutrition problems in this community**

I: Thank you. What do you think are the common nutritional problems of the women and adolescent girls in Tigray Region?

P: Mainly the common nutritional problems are; anemia or iron deficiency, iodine deficiency, and vitamin A deficiency and iron folic acid defects. We understand that the mothers are at risk for developing malnutrition due to the presence of the above mentioned micronutrient deficiencies. In addition, due to the shortage of protein or carbohydrate intake, the women are affected by underweight. We also understand that there are problems related with the unable to take nutrition rich nutrients associated with fasting and harmful traditional practices.

I: Good. You have told me that the micronutrient deficiencies like anemia, goiter and night blindness are commonly observed in the women of Tigray region. Could you describe the magnitude of the problems in this region and in which areas are commonly observed?

P: When I see the problem from time to time, even when I practically visit the farmers (because the farmers are our main customers) and when we show them cooking demonstration together with different partners; I understand that there are big observable problems in the communities, even you can see from their face. When you see the way they take care of their children and their sanitation, you can learn that there are many things to do. When you also see most of the agricultural land of Tigray, it is eroded because it has been ploughed for many years. For example, when you go to the highlands of Tigray, goiter is visibly observed and you can’t explain the number of mentally retarded people in the communities. They may look like normal physically, but there are so many people with mental problems. For example, if you got to around Mychew, Ofal and other highlands that I have visited in my field work, I have observed many people with different problems, like, physical disability and mental retardation with my naked eyes. This indicates, there are very big observable problems in the communities.

I: Good. As you have described very well, there are widespread nutritional problems, especially the micronutrient deficiency in the communities. So, do you think the problems are due to shortage of food or due to unable to use the available foods?

P: The problem is due to lack of properly using the available foods. Let alone the farmers, the educated people do not know about the food pyramid. Therefore, this is an indicator for us that we are expected to work more on what type of foods to take more, with what quantity and quality. Despite the resource is available and productivity is increasing, we are expected to do more on our nutrition.

I: Good. How do you describe the moderate and severe malnutrition in women of this region?

P: These problems are related with sudden problems like drinking unhygienic water and improper handling of our resources or products. These problems are also commonly affecting the communities. These problems are associated with the chronic problems like stunting. The sudden problems lead to sunken eye, loss of body nutrients in the form of diarrhea or vomiting and other nutritional problems. So, these problems are widely observed in different communities.

I: Do you think the nutritional problems that we have been discussing are also observed in the adolescent girls?

P: Most of the time in my understanding, we have not worked on the adolescent girls. We know how the intergenerational malnutrition is happening through different trainings. Therefore, we have not given due emphasis to the adolescent girls, instead our main focus is no the mothers.

I: Do you know why attention is not given for the adolescent girls’ nutrition?

P: I think it is related with our plan. The problem starts with our plan. The target age groups are from 15-49 years of age and we have also activities on the 1000 days. However, starting with our plan, we have not worked on the adolescent girls. We focus on agriculture, mainly on the farmers. I think it may be due to the adolescents could get the services in schools. As per the national nutrition program, the adolescent girls are given health services like deworming, vitamin A supplementation and other different services. So, I think the adolescents could get the services by the health sector through their schools. So, our main focus is on the farmer women, not on the adolescents.

I: Good. Do you have a plan to work on the adolescent girls’ nutrition in the forthcoming?

P: For the adolescents?

I: Yes, as a region agriculture and rural development office, do you have a plan to work on the adolescent girls’ nutrition in forthcoming?

P: Regionally, we have a plan for improving the beneficence of youth, women, and male farmers.

I: What about adolescent girls?

P: We don’t have a plan for the adolescent girls.

I: How do you describe the diet related noncommunicable diseases in the women and adolescent girls?

P: The noncommunicable diseases are observed. When we compare with the past, the malnutrition is resulting in different problems like heart disease, hypertension or diabetes mellitus. If we have worked on the fetus, we can expect his adulthood lifecycle. If the mother is nourished from the very beginning, during pregnancy, up to two years and if proper feeding is practiced after six months of age, then he will have good adulthood life. If the mother was malnourished and if the child becomes malnourished after delivery, and if he doesn’t improve his feeding practice, he will be malnourished during his adulthood life or he will be at risk for different diseases. If we failed to work during the first none months of his early childhood, whatever food you provide later, there will be what we call it “metabolic syndrome”. So, the lifecycle of the person will continue with the problem of metabolic syndrome. Therefore, if possible the mother should learn about her health situation before pregnancy, if not she have to improve her nutrition during pregnancy.

I: How do you describe the occurrence of stunting and underweight in the adolescent and lactating women of this region?

P: Mostly we have been using the information from EDHS. In addition, as nutrition expert, to roughly evaluate the successfulness of our activities we have been using studies done by the emergency preparedness as an input for ourselves. Mostly, we have been providing the capacity building trainings based on the children’s stunting, wasting and underweight measurement status reports to create common understanding and to inform the frontline nutrition experts to works accordingly. Therefore, most of the time we have not been using the maternal nutrition status; rather we have been using the EDHS report of under five children nutrition for our implementation. As we know the EDHS is conducted in Tanqua bergele, Raya azebo and saisietsaedaeba. So, the report will help us to have evidence based information about the children’s nutritional status at global, national and woreda level. In addition, it will also help us to work based on the evidences at the lower level. So, we have been using the nutritional status of the children, but we have never measured the MUAC or other nutritional measurements of the mothers so far.

I: Have you been using the above mentioned woredas as research centers?

P: These are the representative areas in which the Ethiopian Demographic and Health Survey is conducted. Therefore, we don’t directly measure the nutritional status of the children. For example, I am nutrition extension expert. The Agriculture office has make it an extension because its structure goes down up to the lower level and works based on that structure. So, our capacity building interventions depend on the studies conducted by the food security and emergency preparedness offices.

I: Good. How do you see the problem of overweight in the women and adolescent girls of this region?

P: It may be available, as we have described before, the malnutrition related problems like blood vessels closure or hypertension. From my observation, in the past the overweight was the problem of the Europe, but now it is coming to our community. The people say that someone has died suddenly due to stroke or due to other related disease; this indicates the availability of the problem in this community. However, my attention and of course our national focus is on stunting and it has also highest coverage. For example, if you see the global coverage of stunting, it is around 168 million, but there are only 44 million overweight children. Therefore, at national level, especially in Tigray, we have been giving more priority for the stunting, underweight and wasting. However, it doesn’t mean that there is no awareness creation on the overweight. We have been indicating the emergence of cancer and the consequences of overweight. We have been also creating awareness to eat locally available natural foods based on what I have said before, i.e the food Pyramid.

I: Good. You have described that different activities are being implemented for improving the nutrition of the people. How do you describe the food security of the communities?

P: There is improvement from time to time. What we have evaluated is that we have not worked on malnutrition as much as we have worked on food security because the poverty of our region has decreased from where it is to 20 something percent. If I am not mistaken it is around 23%. This indicates an achievement of our food security. However, when we see our level of nutrition, stunting is 39% and this is the reason why we are providing the training on that. Had the agriculture office worked on nutrition as much as it has worked on the elimination of hunger and on the same pace as it has started, we would have improved the nutrition because as I have told you before we were already working on it. Therefore, due to the unavailability of the lower level implementers, the work has been restricted only on increasing productivity of the households. Our assumption was if they have the food they will feed it and as a result the priority was only given for assuring the availability of the food.

I: Good. As per different evidences, the women are more at risk for malnutrition. So, why do you think the women are more at risk for malnutrition? You can describe it in terms of our region’s context.

P: Because they give first priority to their family members feeding over themselves. We know our traditional practices. The woman first gives the meal to her husband and then the children and the wife eats later. Even there are many problems in their way of feeding, for example we know what and how the women eats if chicken is prepared, there are also traditional influences for the women not to take milk. They believe that if the woman drinks milk, she will be full hearted and she will be out of control. Still now egg is not allowed for the women in some communities due to the traditional influences. The other problem of the women is work overload. Still now we don’t have technologies convenient for the women that can reduce her workload. What technologies can reduce her workload? The mother forgers her meal after she spent very long time on work. Therefore, the women has work overload because there is no division of work in their homes. The duties of the family are primarily given for the women. We have started a new project at region level to solve this kind of problems. It is very small project and we have only started it in two woredas (I forget its name, but I will tell you later). The project aims to reduce the work overload of women. The project name is gender model family. The gender model family helps to improve gender equality and division of works at home. In some kebeles, a situation in which the husband waits until the wife comes for sleeping has been created. Even forget the work, knowing the time she sleeps by itself is a big change. When you see the gender centered trainings, they analyses the countless duets of the women and even it is more in the towns. If you are married, you wear your cloths and prepare for the breakfast, but your wife has many activities to do in the house. Therefore, the cultural influences are primary affecting the nutrition of the women and adolescent girls.

I: You have told me that you have introduced the gender model family in some communities… (Interrupted)

P: We are just starting it

I: Ok, it is a good start, could you describe about it (who is implementing it and is it a kind of awareness creation or there is another support)?

P: Mainly it was started by small scale micro irrigation (SSMI). To assure the participation of women, they have to participate from its plan and structure development and they have to reflect their opinion about how and where to establish the drinking and washing water. This project is designed due to the fact that there was a failure study indicating the women are not benefiting from the women development initiatives designed to benefit the women because despite the women development initiative was designed for them, they were not doing it by themselves. As a result, the overall development process lagged behind and they were given by rent. Therefore, after studying how the previous experience, this project was designed to bring the women in to the development. Then it has been started in Adwa, tahtay logomti, laelay logomti and Wukro. All of the people included in the project are married households. It has been implemented in a way they can accept the technologies that reduces the work overload of the women. Some husbands have already started preparing coffee in their homes. One husband said that at least I have stopped insulting my wife that I used it before. What we did was, during the trainings we let them to discuss by themselves about their nutrition. For example, this year we have discussed with the nutrition and water committee. The wives are also included in the committee during the training. I can show you, the drama they have done about gender equality was really very impressive. So, there are many efforts to improve the nutrition of the community by creating dialogue among each other. In addition, they have their own logo and the good thing they have started is when they go to meetings, the husband and wife go together in pair. The other people become amazed by the households who are enrolled in the gender model family. There are also efforts to improve the gender equality through creating discussion forums in the community. But, this project at its infancy stage (laugh).

I: It is good model. What priorities do the Tigray Agriculture Office has in relation with improving the maternal and adolescent health?

P: Repeat it

I: what priorities do your office has for improving the maternal and adolescent health?

P: As per the directions of the national nutrition program, the agriculture office is working on producing the fruits and vegetables which has better bio fortification like, “hibri aranshi sikar dinish”, peas with high iron contents, introducing vegetables and fruits, and overall improving the productivity of the farmers. Improving productivity of farmers helps to increase the farmers’ benefit from what they produced and access in the market. To bring social and behavioral change, we are working on increasing their awareness; especially we are working to increase the beneficence of women and the youths. Mainly we are working with the households to cultivate home garden in 5 by 5 land and as I have told you before we are working to increase their productivity based on their agro ecological, for example, the women are supported to introduce poultry agriculture. Therefore there are big activities and packages to improve their nutrition and their children’s nutrition. Training is provided on animal resources like nutrition sensitive dairy, nutrition sensitive poultry, nutrition sensitive fishery and we have also similar nutrition sensitive activities in crops. We have also started nutrition sensitive activities. Specially, ToT was provided at federal level and we have started its implementation at woreda level. We have also evaluated it in the last month, but our hope is on the capacity building trainings.

I: Good. Do you think the above mentioned priories are implemented in the ground?

P: When I see the experts at my level, I don’t think all activities are handled by the experts. So, I can that the awareness at the bottom is low. We have problems in properly leading and provision of services. Despite we have the checklist; there are problems in giving attention to the checklist and working based on that checklist. However, there are activities properly implemented with the strong efforts of the experts. Especially, I can testify the good integration between the health extension worker and the agriculture DA in cooking demonstration for the children.

I: Do you think the attention given for the women is sufficient in Tigray region?

P: No, it is not sufficient. We have started it, but it is very far from the sufficiency starting from the top because I have seen it 2007 during the coordination body and technical committee meeting to implement the national nutrition program. The challenges raised in the meeting indicated that the problems are also available at the top. So, there are challenges from the top to the bottom. The attention given for productivity is not equal with the attention given for the nutrition. The direction is 60% should be used for consumption while the remaining 40% can be used for the market. However, since our farmers have many problems, they may be obliged to see it in order to take other important things. For example, we have evaluated the cow milk productivity and utilization. There is one kebeles which has good experience, for example, they sell the cow milk produced in the morning, while the milk produced in the evening will be used for their own consumption and for the baby of the cow. So, we have applied this good experience in all milk associations. The problem is our farmers were shaped to have money oriented mind and this is the main reason why social behavioral change is required at all levels starting from ourselves. When you think of nutrition, there are many problems that should be improved. We have agreed with animal resources office to increase productivity because we assumed that the community will start using their products after they produced extra resources. But, we have many things to do on the nutrition aspect.

I: Good. You have told me that lack of awareness on nutrition is widely observed. So, what activities are you doing alleviate this problem?

P: Yes, we are working to improve their awareness on nutrition. One activity is the capacity building activity; our office is mainly working on nutrition in the form of extension communication capacity. Our direction is to create more awareness on beneficence (“tetekaminet”); by the way the word beneficence (“tetekaminet”) is misleading, instead it better to change it by nutritional beneficence. It has been misleading us for many times. It has been reported that this number of women are benefited from the productivity, but I feel this is not correct and as a result we have started to revise our checklist. We have to assess their nutrition instead of the beneficence. For example, we can ask them how much they consumed from their products. Therefore, we are working to improve our activities in the checklist to make it more informative even by listing the food groups. So, the checklist can clarify the important activities that should be done. Therefore, there are efforts and the main sign of their beneficence is that they are buying vegetables from the market by selling their products. They have been observing that the farmers have started to buy and use vegetables and fruits by selling their cereals. This indicates there is a change from the past. During the time I started professional work, the farmers were eating enjera with salt and red pepper and they were taking milk once a week (if they have it). Therefore, there are big awareness changes from the past. However, there are many problems in terms of improving nutrition; for example, we have not worked more on what types of foods to eat and how to eat a mix of important foods.

I: Good. What additional activities do you have in addition to what you have mentioned for improving the nutrition of pregnant and lactating women and adolescent girls?

P: We have different projects with CONCRN, GIZ, UNICEF (we are working on Nutrition Though the Growth (GTN)), World Vision, Ginea Community and others. We are working to improve the beneficence of the women through introducing different supportive packages like what we call it productive safety nets programs. Besides, we are working to improve their nutrition through supporting their home gardening, introducing poultry agriculture, capacitating the women through providing different trainings, printing and disseminating different messages and we are also working with different Medias like Dimtsi Weyane Tigray to improve their awareness. As I have told you before, during the training provision, there are role plays. Especially, the important materials developed by the sustainable, under nutrition reduction in Ethiopia (SURE) project are found in our office. The material has details on the role of agriculture on nutrition and how the training should be given. They have observed how the health extension and agriculture extension workers are doing through going to the communities and they have agreed on how to work together. The project was implemented in five woredas, and they were providing selected seeds and different agricultural tools for the poor women. The government has made an effort to establish a structure and has assigned nutrition and water experts up to the woreda level. The problem is there is lack of budget to hire nutrition experts at woreda level or I can say there is lack of attention to allocate budget or divert the available budget for nutrition interventions. Therefore, there are visible problems because work its being done by delegation. Despite there are still attitudinal problems, the agriculture office has been responsibly working on capacity building and introducing quality maize protein. I would be very happy if you have visited the beet market in Alamata. The cooked beet is being sold in the street roads of Alamata. The mothers could give the cooked beet to their child and the daily workers could eat after their work. This is very good experience that I have seen recently. So, this indicated that if the agriculture office worked very hard, many nutritional problems can be solved. Eating vitamin A rich foods is better than taking vitamin A drug. So, I believe the agriculture office can solve many nutritional problems. However, whether we believe or not, the wide activities done by the agriculture office should not be ignored.

I: How do you describe the counseling given for the pregnant and lactating women to follow their antenatal and postnatal care follow up and to take extra meal more than the ordinary person?

P: We are not providing the services by assessing their health status, rather our focus in assuring the availability of folic acid rich near their homes. For example, the importance pumpkin was forgotten for many years. I have been always taking the seeds of pumpkin to all training that I have provided. I always counsel the women to cultivate the pumpkin. We have not worked on the important fruits that can easily grow in the communities. The agricultural experts are experts are expected work together with the health extension workers on the health packages. We are expected to work on creating food access and counseling on what type of food can replace what, what types of foods can give us iron, what type of foods can give us vitamin A. We can also get folic acid from pumpkin or papaya. However, I think the health message about institutional delivery should be provided by the health workers. We are expected to create awareness based on our professional expertise.

I: Good. How do you describe the role of the productive safety net program on improving maternal and adolescent girls’ nutrition?

P: As it has been clearly documented, the pregnant women are not expected to work in the productive safety net program as well us in the environmental conservation activities rather they are supported for free. Different trainings were given to them to increase awareness about their nutrition through the safety net program. Even there is one day training about their nutrition, which is deducted from their productive safety net working hours in the community. But, the time taken for the training will be accounted as if they have worked in the productive safety net program.

I: Good. At what time do the pregnant women stop working in the productive safety net program?

P: The pregnant women will be supported for free starting from the time she reported her pregnancy.

I: Good. You have told me that you are widely working on the nutrition sensitive agricultures like home gardening in different parts of the region, but in some areas, despite they have the awareness they are not implementing it due to shortage of water and vegetable seeds. So, what activities do you have in order to solve this kind of problems?

P: The agriculture office is working based on the agro-ecology. And this is the reason why there are no home gardens in different places. After identifying the agro-ecology, there are an agricultural activity that goes with the lowland, highland, and middle land. So, we are working based on the whether condition or agro-ecology of the communities. We have now assessed the agro-ecology of the communities, for example, the irrigation area are identified, the crop production is undertaken in cluster bases and even the animal resources production is cluster bases. So, it is not possible to implement all resources in all areas. *For example, if you take the dry lands like Tanqabergele, the agro-ecology is conducive for goat production. So, it is impossible to introduce home gardens in such kinds of agro-ecologies because there is no water, otherwise the seeds will be wasted. Therefore, we are implementing everything based on its agro-ecology. So, there are identified agro-ecologies for crop production, animal resources and irrigation.*

I: As you know the iodized salt and ITN utilization are nutrition interventions. So, how do you describe the efforts undertaken for creating awareness in the community?

P: We don’t mention the insecticide treated net in our capacity building or awareness creation activities. I understand that if someone is sick with malaria, he may be suffered with acute malnutrition due to the fact that if there is high fever, there will be high nutritional lost. However, we have been giving more emphasis in iodized salt utilization. It doesn’t mean that there is lack of iodized salt access in the households. Majority of the households have iodized salt in their home. However, they don’t know the volatile and evaporative nature of it. Therefore, we show them during demonstration not only the iodized salt but also lemon helps the bioavailability of foods in our body. So, during demonstration I show them by adding squeezed lemon to the food and also show them how to properly use iodine, even they should not leave it open because it can be evaporated. During my field visit, one woman told me that she has been drying the iodized salt to eliminate its moisture. They are not properly using it because we didn’t teach them how to use it. They are only looking for the iodized salt, but they don’t have any information whether the iodine can be eliminated by heat or air. But, in 2009 e.c, we have provided capacity building trainings with practical demonstration on iodized salt utilization, lemon use, and how to enrich the porridge (complementary food for the children) including its ratio of the cereals, legumes and other important ingredients. Even we have been telling our experts to start it from their homes because we have to work for our children and grandchildren. If we don’t practice proper feeding in our homes, we cannot teach to the other community.

I: Good. You have told me that deworming service is given for the children. Do the adolescent girls get deworming services?

P: As I have told you before, the deworming and vitamin A supplementation are not our interventions. I understand that the deworming drugs help the availability of the taken foods for the children. Similarly, I believe that they should take the vitamin A supplementation. In addition, I believe that the antenatal and postnatal cares are very important and they should follow it. However, technically we have different responsibilities. Ii is possible to give information, but it is not good to enter to the profession of others. So, I don’t have information about its coverage and implementation.

I: Ok, you are not supposed to speak on behalf of their office (Health office), this is about your personal opinion.

P: I know that deworming is given for the adolescents in the rural and urban schools. However, there attitudinal problems in the community, for example, some people believe that the injections given for the prevention of tetanus are contraceptive methods. Some women were escaping from the treatment because they didn’t believe that they are getting preventive treatments for tetanus disease. But, the coverage is increasing from time to time. Especially, when I was working in the woreda, they were getting the deworming services in the schools.

I: You have told me many nutritional interventions for improving the nutrition of the women and adolescents. So, could you tell me the most successful and less successful nutritional interventions in this region?

P: Skill and understanding are very important things in nutrition. When we say skill, a person should not only focus on eating but he also needs skill on how to prepare required foods per meal. We can prepare from vegetables, lentil and carrot to get vitamin A, we can also add oil. Therefore, we have gain skill to prepare balanced diet from the food items. Some people believe that balanced diet is the meal of the reach people. They also say that we don’t have the food to prepare balanced diet. But, it is about awareness, knowledge and skill to prepare a balanced diet from the available food items. First, we have to create awareness, and then the skill comes later. Many good things have been done on the complementary foods of the children, especially by the women development army. I feel some of the good works done before are going backwards. The Orthodox had worked very well, especially in swapping cereals. Even many job opportunities were created for the women and there are good experiences in Adwa, Mokoni, Saharti Samre, rayaazebo, Degia temben and mychew. So, the nutritional interventions in the children are relatively better, but we have not worked for the mothers. Just we say the mother should eat extra meal, but we have not worked on what types of foods the mother should eat. Even the main concern of the health sector is about complementary feeding. I have not seen anything recipe developed by Mekelle University, Ayder Health College. If I am not mistaken, there is no recipe developed for indicating what types of foods the pregnant should take.

I: Good. Which of the nutritional interventions that we have discussed before like nutrition sensitive agricultures, productive safety net program, counseling on diversified and extra feeding and others are successfully implemented for improving maternal nutrition?

P: Some excellent women dry the important foods like tomato and pumpkin use it thought the year and they also know the quality maize protein. When you ask them about it, they can tell you more about it including its benefits (building the body). But, we have not worked specifically for the pregnant or lactating women, rather as I have told you our main customers are the farmers. We have evaluated that our data should be improved. For example, the health sector has data for different category of children like from 0-6 month, from 6 month up to one year and others. So, we don’t have this kind of data in our office, but we are planning to differentiate the pregnant and lactating women. This will help us to know the nutritional interventions undertaken on them and it will also help us to know our primary targets. We were working by focusing on our customer (farmer), and I learned that this was a misleading concept. We were assuming that the farmers are the productive citizens and if they are sick the productivity will be lowered (laugh). For that matter our plan for GTP II was to improve the nutrition of all farmers (100%) and 70% of women. However, when we plan to improve the nutrition of 70% women, we have not differentiated the different category of women.

I: You mean that your office does not have specific nutrition intervention for pregnant women?

P: Yes, we have not differentiated the pregnant women in our data. So, we don’t have any nutritional intervention specific for the pregnant women, but now, I we have agreed that we have to have different category of data at woreda and region level and I have also started some activities to implement the nutritional interventions specific to the pregnant women.

I: Is there any plan about that?

P: Yes, we have identified what to do for whom. As you have said, sometimes you can see things in different aspect. Our target was emphasizing on “this number of farmers have cultivated home gardens, this amount of chickens are introduced for the unemployed persons and related activities”. Did you understand me? Our understanding was, if there is home gardening, the nutrition of the family will be improved and their productivity will be improved. But now, we understood that if we think that the under 2 years children are part of the 1000 days then we will correctly implement the nutrition interventions. So, this is what we have proposed to do for the next. The activities of the SURE project were reported based on specific age groups. Since the activities of the SURE were on the under 2 years of age children, we hadn’t problem on that. However, when we see at Agriculture Office level, our activities are broad and we were focusing on improving productivity through focusing on the productive citizens without study in order to create food access for everybody.

I: Is SURE an NGO?

P: SURE is sustainable under nutrition reduction in Ethiopia.

I: So, their target is… (Interrupted)

P: Their primary target is 1000 days. The same thing, GIZ and Nutrition Sensitive Agriculture also works on 1000 days. There are many NGOs working on 1000 days. There are some NGOs working on the nutrition of pregnant and lactating women of some specific areas, however, our office is not yet working in this direction. The government (agriculture office) focuses on the overall customer, which is the farmer. The office is working to improve food access and feeding practice of the farmers. As I have told you before, we have been creating awareness on the farmers to eat balanced diet from what they have, but the women development army are responsible for creating awareness about the feeding of the children and the mothers. In collaboration with unicef, cluster based training was given for the women and men development armies.

I: How do you describe the implementation of Targeted Supplementary Feeding for the malnourished mothers and adolescents?

P: TSF is available.

I: Is it available in all communities?

P: It is given for the mothers and children who have malnutrition in the areas where there is food shortage. By measuring their nutritional status; there is fafa, plumpy’Nut, and even there are also inpatient and outpatient nutritional treatments.

I: Good. As you have mentioned, there are many nutritional interventions for improving the nutrition of the communities. What are the challenges for its implementation? The challenges could be related with the individual beneficiary side, community side or the intervention itself side.

P: The main challenge is lack of awareness/understanding/, especially cultural practices are common in Tigray region and the people are restricted by religion and fasting. Therefore we have to implement wide awareness creation activities that can overpower the existing challenges. There is no good understanding from the top to the bottom that working in nutrition can help the achievement of national growth and transformation plan. If everyone including the administrators and religious leaders has worked the attitudinal change, the challenges will be solved. So, the main thing that we should work is on the community culture, traditions, religious practices that affect their nutrition. There are some activities started by the religious bodies on fasting, but only this change cannot solve the existing nutrition problems. There are other alternative activities that should be done to improve nutrition, for example, mushroom can replace proteins and it can solve the challenges related with fasting. We have to also introduce other alternatives that can solve the challenges. We are focusing only on production, but we have to also focus on nutrition. Our research centers should work in improving biofortifiction.

I: What activities are being done to solve the main nutritional challenges like the cultural, traditional, lack of awareness, and religious and production orientation?

P: Some activities have been started to alleviate the challenging. For example, the first is using public Medias to improve our communication with the people. The second is community mobilization to create awareness about malnutrition. We are also working with GIZ to bring social behavioral change in the community. For exampling, we are preparing to release a poem written by Mahlet about nutrition through the public Medias to create awareness. In addition, in collaboration with SURE project a short drama is formatted by the Association of Tigray Traditional Artists in the farmers training center and it will be transmitted in the ETV Tigrigna program after editing. There are also efforts in printing and disseminating newsletters and leaflets. There are also capacity building trainings to bring social behavioral change in the community. At office level, we have established a nutrition technical team to plan and implement nutrition related activities. So, this are the activities started to solve the existing nutrition challenges. But, our worry is the nutritional budget is only given for the health sector and we are also maligning the government for that. Most of the time, starting from the federal level the nutritional budget is given for the health. Even there is no budget for the M&E. There is budget shortage in the agriculture. Thanks for some NGOs; they are working on the nutrition sensitive activities for their own mission. The main obstacle for lack of support, follow up and evaluation at the region, woreda and the bottom line is lack of budget. We have also discussed about it during our review meeting in August 2009 E.C. Therefore, we have evaluated that we have not worked as expected despite it is very important area. However, to improve the capacity building trainings and community awareness, the government has to allocate budget for it.

Multis

I: We are through to finish our interview. As you know nutrition of the women and adolescents is not an activity of one sector. So, besides the agriculture sector, what sectors should work on the nutrition of the women and adolescents?

P: In addition to the agriculture, there is heath, education and if you don’t have the water all what we are talking cannot be implemented, and there is also trade and industry to play key role in the imported foods like iodine, women affairs, social affairs, youth and sport affairs, Media, women association and farmer association (because creating awareness only on the women cannot solve the problem. The awareness should also be created on the men because they are the main sources of the resources and they can buy the required foods.)

I: How do you describe the existing collaboration between the different sectors you have mentioned?

P: If you see it based on Sekota declaration, it is centralized and it has given big emphasis by the government. There are started activities and in my observation, they are strengthening from time to time and I hope the technologies will bring changes in the ground because the sectors have come together.

I: What do you think are the existing challenges of the multi-sectoral collaboration for improving maternal and adolescent nutrition?

P: In the past, since all the sectors had the same status, there were problems of accountability and responsibility. But, now some of the challenges are solved because the multi sectors are led by the president of the region. I think the remaining problems will be further solved because the multi-sectoral collaboration is given more attention by the top management of the region.

I: Good. Some of the woredas told us that they have muti-sectoral technical committee, but they are not practically collaborating in the ground, instead they are independently acting and they also have problems of accountability. Do you know this kind of problems?

P: Yes, it is known. As I have told you before there is problem of understanding from the top to the bottom. There is big challenge in understand the importance of collaborative working for achieving the nutrition targets of the GTP. The impacts of nutrition cannot be achieved in a short period of time. Therefore, the nutrition activities are neglected because the sectors focus on their urgent priorities. In the past, as you have said, the sectors were connected, but now there is very high turnover. The problem is you will not get it tomorrow what you have established today. The one you have established today very well, then it will be dispersed by the next day. As long as there is turnover, you are expected again to re-establish it. There is also scarcity of budget to do the activities. But now, the multi-sectoral collaboration is strengthening at region level because responsibility was given for the president to lead it and Mr. Assefa is delegated by the president to lead it. Similarly, formal letter will be written to the woredas to implement it. This indicates, the multi-sectoral collaboration is at plan level. Since it is at plan level, it requires very strong effort to create collaboration between the sectors. With all the challenges, we have been advising to our staff at different levels to be optimistic and to feel sense of ownership in implementing the nutritional activities by entering to the community. But, what they asked us was the availability policy to do the activities, however we have to see the problem whether there is policy or not because policy is not our mandate and nobody has restricted us to work our mandate. Therefore, we have to work on attitudinal changes. Nutrition is the priority of all sectors, even the administrator politicians. Due to the high level turnover or replacement, we have not influenced or created awareness the politicians as to the expected level. If there is multi-sectoral collaboration at the top level then it will create a pressure for the establishment of multi-sectoral collaboration at the lower level. However, the sectors are going independently, but we cannot chive valuable results by going independently. We have not influenced others to understand that they are not working on nutrition. Due to lack of influence, the others think as if they are working on it.

I: Is there a multi-sectoral common plan and working system which states what to do, when to do and how to do?

P: It is different from woreda to woreda. The woredas which have GTN, and in which engine was working have known separated minute and meeting schedule for nutrition. Since it has budget, the woreda cabinet visits the kebeles for arranging and following its implementation. The problem is the project works are not sustainable. Therefore, we are left with many things to do in implementing the nutrition activities as a routine activity without budget. Whenever you talk about nutrition, they believe that it has a lot of budget and benefit despite it is their routine duty.

I: Finally, how do you describe the available strategies and activities for child birth spacing and prevention of early marriage and the community attitude about it in Tigray?

P: Nowadays it early marriage is improved. However, there are still early marriages in the community. When you go deep to the rural communities there are still many unsolved problems. Though the policy allows the marriage of above 18 for female and above 22 for male, there are still influences for the females. It is known that there are many problems what we have not reached for. The tendency for early marriage is decreased, but still it is available.

I: Do you think our policies and strategies on early marriage are effective?

P: Depending on their understanding and interpretation of the law, there may be gaps in its implementation. This could reduce the follow up and monitoring of the early marriage. There are rapes and other problems. So, more assessment is needed at the lower level. If we went to the rural community not as a government employee but as a relative we could have faced many problems. We are observing adolescent girls employed in the urban community who escaped from early marriage. When you ask their age, they are either 15 or 16 years old. This is indicates the problem is still existing. For example, there was similar girl in my house in this year. She told me that she escaped from the market while she was sent to the town buy new cloth for her marriage. Therefore, they are stopping their education and they are also coming to the towns. Those girls who have some education are escaping to save themselves from the early marriage.

I: How do you describe the influence of early marriage on their nutrition?

P: As we have been discussing, if the girl is not mature, this will lead her to intergenerational malnutrition. In addition, her newborn will be underweight and his survival probability is very less. Even, if the child is grown, she will have intergenerational malnutrition. These problems are happening because the girl does not have mature physical body.

I: What activities are being undertaken for encouraging child spacing?

P: In the towns, the mothers space by themselves due to the economic influences. There are also improvement indicators in the rural communities due to the economic influences. However, there could be some problems due to the religious influences. But, there are big changes from the time I was working in the woredas.

I: Thank you. Finally, as I have described in the very beginning, the objective of this study is to identify the barriers related with maternal and adolescent nutrition and to provide evidences for policy development. So, you are welcome if you have additional suggestions.

P: We have described it. Anyhow, it would be better if the agriculture of the region had its own workers up to the Kebelle level. The government believes that it could be implemented by the existing development workers. But, there are good signs that better work could be done if it has its own experts up to the Kebelle level. Therefore, if women nutrition becomes sensitive by everyone and if the agriculture office established its own experts at lower level (like the efforts it had in 1984 for training us) that can support the implementation of the home gardens, introduction of poultry agriculture, cooking demonstration to solve skill gaps in the community, counseling on diversified foods, better works will be done.

I: Don’t you have now nutrition experts that works in the lower level?

P: We have four experts (animal resources, natural resources and other different experts) at Kebelle level, but they cannot solve the skill related problems of the women. We might think that the skill related problems can be solved by the health workers, but the production belongs to the agriculture. Therefore, we need female agricultural workers who can closely show the women on how to use the available agricultural resources in their homes using locally available cooking equipment (“disti”) because the available agricultural experts have been working on production. So, if we sense the existing problems, it needs its own expert who can solve the awareness and skill related problems.

I: So, do you have a plan to implement that?

P: They don’t accept it because they believe that there are excess workers. So, they didn’t sense it due to budget problems. However, if we believe on the policies and strategies and if there is pressure from the top, it will be implemented in the lower levels. If you ask me on how we are working, the activities are given to the woreda and to the Kebelle leaders and then they are working on improving productivity by deploying their own workers. The Kebelle level agricultural workers are also involving in the cooking demonstration for complementary feeding, but this is not enough. We have to work to solve the skill related problems.

I: Thank you for scarifying your time to participate in this study.

P: Thank you.

**Summary**

Section 1: Common maternal and adolescent nutrition problems in the community

- Protein energy malnutrition, Iron deficiency anemia, iodine deficiency, and vitamin A deficiency and iron folic acid defects are visibly observed in different communities of the region
- There are also many mentally retarded people due to micronutrient deficiency
- Severe and moderate malnutrition are also commonly observed in the region
- Mostly the problems are occurring due to lack of awareness and understanding on nutrition
- As per the EDHS report, the stunting and underweight are commonly observed in the region

Section 2: Interventions that improve adolescent nutrition

- The agriculture office do not have any nutritional intervention for the adolescent girls
- Gender model family project is under implementation for improving gender equality
- As per the directions of the national nutrition program, the agriculture office is working on producing on the fruits and vegetables which has better bio fortification.
- The office is also working on home gardening with the households
- The office is also providing capacity building training on animal resources like nutrition sensitive dairy, nutrition sensitive poultry, nutrition sensitive fishery and nutrition sensitive crops.

Section 3: Implementation challenges and community factors affecting access to nutrition

- Cultural, traditional, and religious influences are affecting the nutrition interventions of the women, for example, egg, milk, honey are not allowed for the women in some areas.
- The agriculture office has been focusing on improving agricultural productivity, but not on nutrition
- Still now the focus of the agriculture office is on household level (farmer) level interventions and they don’t have specific nutritional interventions for the pregnant, lactating and adolescent girls

**Section 4: Multi-sectoral collaboration to improve maternal nutrition**

- Agriculture, health, education, trade and industry, women affairs, social affairs, youth and sport affairs, Media, women association and farmer association are the steering committee for nutrition. The steering committee is led by the president of the region.
- The multi-sectoral collaboration is still not well implemented at all levels of the region.

**Section 5: Other interventions that influence adolescent and maternal nutrition and health outcomes**

- Early marriage is still widely practiced in the rural communities

**Section 6: Additional remarks**

- The existing agricultural workers in the kebeles are working with especial emphasis to agricultural productivity.
- The interviewee recommended deploying new female agricultural experts at kebeles level to improve the skill of the women on nutritious food preparation with the existing resources.
