# Supplementary material for: Barriers to utilize nutrition interventions among lactating women in rural communities of Tigray, northern Ethiopia: An exploratory study
Source: PLoS One. 2021 Apr 30;16(4):e0250696. doi: 10.1371/journal.pone.0250696 (PMC8087028; doi:10.1371/journal.pone.0250696)
Supplement: S2 File — (ZIP) [file pone.0250696.s002.zip › S2_File.Doc/Woreda level and above key informants/149_IDI_Head for Agriculture Extension in rewgional Agri and Rural development Bureau_Tigray.docx]

**Operational Research on Adolescent and Maternal Nutrition in Northern Ethiopia**

## **In-depth interview responses of the agriculture and rural development extension training officer**

**Introduction**

Thank you for your acceptance of the informed consent form and for taking the time to speak with me today. I have questions to ask you which were prepared in advance. The discussion will take 1-2 hours. If you have any questions before we begin please feel free to ask.

**Section A: Interview in details**

1. Zone: Mekelle Zone
2. Woreda: Mekelle
3. Kebele: 05
4. Name of key informant: HailuKiros
5. Institution of key informant: Bureau of Agriculture and rural development
6. Interviewer name: MekonnenHaileselassie
7. Date of interview: 14/03/2010
8. Interview start time: 4:50 AM (local time)
9. Interview end time: 6:20 PM (local time)

**Section B: Socio-demographic and basic data of qualitative study participant**

| **Socio-demographic characteristic** | **KII** |
| --- | --- |
| Sex | Male |
| Age | 52 |
| Educational status | Bachler degree |
| Occupation/role in the community | Extension training officer |
| Service year | 28 years |

**Note:**

I: interview

P: participant

**Section 1: Common maternal (Pregnant women) nutrition problems in the community**

I: Before I proceed to the main question, do you have any general view on the main problems related to the pregnant women, lactating women and adolescent girls considering as agricultural bureau?

P: There were no activities that conducted before we were focusing on pregnant women, lactating women and adolescent girls in our sector. In the past, the main focus area of our bureau was on how to boost the agricultural productivities. But there wasn’t any linkage that gives attention on how and in what manner the pregnant women, lactating women as well as the adolescent girls are get benefit from the agricultural production. But now, especially based on the plane of GTP two and national NNP, we derives what can we work on nutrition and in regional as well as in woreda level (in around 19 woredas) experts were employed to work on nutrition. And there is also a focus on how to create a linkage with health extension experts and the production and productivity of the agricultural sector. Therefore, we give emphasis to link the production of our livestock, crop, and irrigation department with nutrition.

I: In your opinion what are the common nutrition problems in the community for women?

P: As you have said, especially with those lactating women and pregnant women in relation to productive safety net program, there is a department/group that works on that activity to follow up the challenge of children and women in relation to nutrition.

I: what is the main mission of that group/department?

P: The department focuses on the food securities of children and women of those screened below standard, and provide service in order to get a balanced diet. This department works in majority of the regional woredas.

I: what do you think the consistency of service? In some woredas; the support is given by health bureau and in others is given by bureau of agriculture.

P: Of course, the coverage is dominated by health bureau, but also here in our bureau the responsible team/department works on that as health bureau did.

I: what do you think about the coverage of the micronutrient deficiency (goiter, anemia, night blindness…) do you believe that the problem has a wide coverage?

P: There is no any study conducted by us focused on that problem. But since we work closely with the farmers, we know that these areas with iron, calcium, phosphors deficiency. Therefore, based on this deficiency, we have documented information. The problem coverage of stunting (low height for their age) is almost known. Now we are working on nutrition sensitive agriculture and we are doing the awareness. When we say nutrition sensitive agriculture, we think that where we are and where to go in relation to stunting, underweight, wasting and we have the existing data. We now almost all the agricultural woreda sectors and we have never seen overweight in rural areas. In relation to diabetes in children, since it is hidden they may think that as another disease but there is no any information that we get from the health bureau. But in regard to overweight some changes are existed in urban areas like what we see in other countries. As we observe the students in school, there is overweight in some of them. The reason is just we can say that it is due to the style/system of our diet.

I: How do relate malnutrition and food insecurity?

P: Now it doesn’t mean that there is no problem due to food insecurity and lack of education. At least we all know that the agricultural productivity is increased from time to time. But when we assume that, is it in all over the community? There might be a part of the community that does not produce that product. Therefore, we cannot conclude that there are no affected women or child due to lack of that product, there is. Even though the productivity increases, the coverage required an effort to address, that’s what we see in the ground.

I: Therefore, it may not be goes in a balanced way (increment of productivity and coverage). Do you think that?

P: Of course, but now the magnitude comes to become narrower in different ways. For example, if she/he does not have a land how can we take care of her/him? It may be by engaged her in poultry production or honey bee production or small ruminant production. In somewhat, to change the way she lived. If it is a youth and has no land by doing the same thing, there is a system/strategy how to make them equally benefited. But we cannot say there is no gap.

I: How do you think women are especially at the risk of malnutrition you have mentioned above (in pregnant women, lactating women)?

P: The reason what we see is workload. When we go to the rural area all the activities are on the shoulder of the women, it might be on the lactating women, it might be on the seven/eight month pregnant women that carry out the work. But the women instead of substitute the energy that she loses during the work, either there is no food or she serves first the family. She is the one who goes to the market, almost as we see in the rural areas she is the one who goes to grinding mill, to fetch water, and you can see that she is pregnant or something. But instead of having a food in order to substitute the energy she loses, she feeds first the family either for her husband or her kids. This is the major problem that we observed individually, even we can observe in the field work. The other thing is even there is food; there is a gap in what kind of food to be taken, lack of proper feeding system, lack of feeding diversified food types. She may have honey, butter, but there is lack of awareness on the feeding system.

I: Do you think the women/mother is more sensitive from the community? Why?

P: When you see in the agricultural works, you may know that the women have started plowing, leave it that, even there is a husband in the household, and the women participate in all agricultural activities/work. She works with him and back to home, and go carrying agricultural equipment to the farm area, she carrying baby, those all burdens are taken on by the women.

**Section 2: Nutrition priorities in the woreda**

I: As institution, are there any prioritized activities that you work in relation to the women?

P: The prioritized activities that we work in relation to women are: the feeding system that what kind of food should feed the women; when she should feed; what she should grow in here backyard; it might be homestead fruit, it might be vegetable or it might be home garden. In general those all things that related to women up to cooking demonstration were carried out. Cooking demonstration in the farmers training center, we supply the equipment of cooking demonstration, then mothers/women were learned there; not only the mothers but also the man that can help them should be trained on production and cooking demonstration; such kind of activities are now working as agricultural bureau. Beyond this, even those foods believed which have good content/nutrition; crops, for example maize, introducing maize with high protein content (quality protein maize), bio-fortification in relation to technology; to grow up the crops with all contents from the ground were practiced in some areas like in Tanqua Abergele and Kola Tenben. These are what we work in agriculture in relation to pregnant and lactating women.

I: What nutrition interventions have the most resources allocated to them considering the coverage?

P: Of course, we have non-governmental partners that work with us like UNICEF, Arish Aid, and others. Currently, we are working with them after they tell us their plan, based on their plan we have a professional experts and then as I told you before, starting from the training up to production and then there is feeding demonstration in the farmers training center. Therefore, based on this, we follow up/monitor the allocated resources in each woreda for what they used it. There may be another organ that monitor the finance, but in relation to women and pregnant we follow up the activities that carried out at the ground.

I: Do you think the activities that carried out are basic especially in changing the women’s nutritional status? And what is the weight given to the pregnant women, lactating women and the adolescent girls?

P: In solving the problem, when we see in the past three years there is a good achievement. Though it is not as we expected, they are changing in feeding habit, in production; especially when you see on the targeted women’s, if there is water even in small amount, all of them in their back yards there is something to grow (vegetable, fruits). And there is a change not to sell the products rather they consume for themselves. Before that, there was a trend to take chicken, butter, and milk to the market; but now there is a change to consume for them, and it is also described in the training guide.

I: What do you think about the availability of water for home garden vegetables? And the adolescent girls are not part of the intervention in many woredas?

P: It is obvious that there is problem of enough water availability/distribution. But the strategy of the government indicates that everyone should have more than one possibility water source. And we in agricultural sector followed that strategy. But, are we go through that strategy? No, it has its own gap. There is shortage of water, but in the area with water, it might be in irrigation or boreholes/low land areas/, there are many places that become irrigated and green. For example, if you go to Quola Temben, there is an improvement; any drops of water could be changed into effect. Currently we receive around four good report of water usage technique from Quola Temben, Malbere and Negsh.

I: Do you think is necessary for your institution to get involved in work aimed at improving nutrition among women and adolescents? Why?

P: You know agricultural in nutrition improvement is basic, mandatory. In health, it has its own part, but most of the time the change is in children, women, and in pregnant women. However, in agriculture we are not working what we should work, there is something gap. But the change was evaluated according to the plan versus report that we have. After the report, there are technical team that work in collaboration with external organizations like AGP, SMIS that work in small projects. Currently there is a good work conducted with SMIS, we have a good approach with SMIS. The wife and husband after gets training together what they bring a change and how the change could disseminate, it has its own approach. Now there is a good thing that we evaluate it. As a team, there are conducted activities. There are listed the gaps by the SMS group (subject matter specialist) and every quarter, the performed activities are reported to the regional bureau. In the ToR a list of criteria focused on what are performed in nutrition and we evaluate based on the criteria. The nutritional criteria included in the ToR and then they bring to regional bureau, we evaluate that whether there is a change or not, are we work based on the plan or not; these all are the way that we evaluated. The one that you raised before, the focused given to the adolescent girls, of course it is true that stated on the program but the suggestion/complain is right.

I: What do you suggest effectiveness of your intervention?

P: Yes, as we mention before it is visible that there is vegetables and fruits that are grown in the back yard. Based on the correct diversified; now let us say if it is fruit, the amount that recommended for one household is known. Currently in collaboration with SURE, there is good activities that we conduct; the SURE supports even agricultural equipment to women, lactating women and pregnant women. Supporting from agricultural equipment starts from watering jar up to giving cultivating tools. Beyond this, seed fruits which are found in highland areas, they purchased and giving as support for the pregnant and lactating mothers. There is also ongoing plan that related with poultry; a lot of budget is allocated to purchase and to distribute to the local areas. But before that, we were working on activities related to poultry, milk/dairy, and small ruminants. Especially on irrigation activities, it may not be lead as for discussion. There is scarcity of water, but we focused on what can we work on that area, it may be related with poultry production;

We also encourage them to exploit their maximum resource that could afford and even water is not available nearby; to work by transporting water using donkey are some of the activities that are conducted still now; these activities are carried out by SURE, and by Irish Aid. The same things in other activities like in poultry and in small ruminants are done by the pregnant and lactating mothers. Currently there are about three components that are performed by Irish Aid program in the enhancement of nutrition in pregnant and lactating mothers in the region. The first component is strengthening the farmers training center so as to serve as demonstration site and the women could be benefited. Secondly, by identifying the sources of income generation, there is a trend that to support and to engage them on these identified activities. The third component is related with increasing the production and productivities, as what we called improved quality maize protein; such kind of activities was already implemented on ground.

**Section 3: Nutrition interventions that improve adolescent and maternal health**

I: What kinds of nutrition interventions are in place to improve health of the pregnant and lactating women to your level?

P: Now, what I have said before, the intervention is mainly making awareness among the communities which is done through training; the training is practical training delivered to them. What products do we have at home, and how the product is utilized; therefore, the training is given both theoretically and through practical demonstration. We let them to practice by themselves. As I mentioned before, there are demonstrating materials in farmers training center which were purchased and introduced by the government and other non-governmental institutions. Thus, the pregnant and lactating mothers are given the training and practice it by themselves and they also consume it. After that the pregnant and lactating mothers are applying practically at their home; this type of activity is done in different woredas like Tanqua Abergele. This woreda is our model in terms of preparing such types of practical activities.

I: How does your institution contribute in the reduction of workloads among the pregnant and lactating women?

P: Of course I don’t catch up the norm; but during the productive safety net program, the pregnant and lactating women, have special favour as compared with the past time and other communities. Currently the norm is improved that could not affect their health status. To reduce the workload in productive safety net program, it has its own norm that could help the pregnant and lactating women to get rest.

I: What about the workload of pregnant and lactating women in agriculture and housework activities?

P: In this regard, as I told you before, we are developing a mechanism that how husbands could help his pregnant or lactating wife; If the husband is a model in helping his pregnant or lactating wife, we put a sign on his house that indicates as a good or role model husband. This kind of work is conducted in collaboration with agriculture growing program. I will tell you the approach later on. At least if there is an improvement or change in the feeding of the pregnant or lactating mother, or helping them in the household, there is something that post in his house. Then after, he encourages and considered as role model to others. Then in the community there is an evaluation among the community by setting the time interval in order to identify who improves first that bring a change and who is not. If someone claims as he improves in supporting of his wife, we evaluate the improvements. Mainly the evaluation points are like what did he helps to his lactating or pregnant wife; and each performance is evaluated by the community. I think before a month, there was a workshop conducted at Wukro with the targeted group and we discussed about the encouraging of husbands in supporting their wives and during that workshop we suggested that such kind of best experiences should be expanded its coverage throughout the region.

I: How about your advice on water, sanitation and hygiene services?

P: Currently we don’t have an activity in relation to sanitation and hygiene, most of the time we don’t give a focus on this area.

I: what about the activities worked in targeted supplementary feeding?

P: There is one team that organizes the targeted supplementary feeding in women; it is served for the poor women which correlated with food insecurity which is given as supplementary feeding.

I: Do you mean it works for those below the standard?

P: Yes, it is given for those who are screened below the standard.

I: What are the most important intervention activities that implemented by your institution?

P: We take the poultry production easily; for a household it was supported up to 50 chicken and from their product they could consume the eggs and to serve as a source of income generation. This is almost we can say as successful activity. Now the capacity of supplying to the market, we was agreed with many suppliers. That is the one thing successfully achieved. The other good work is the production of fruits and vegetables in the irrigation area that showed the successful improvement; here the product could consume for them and also they could sell for their other house expenses. Therefore, the home garden activity is an easy task that could consume for them and it could as a source of income. If you take in the area of livestock, in addition to poultry production, there is honey bee production, now women start to own either individually or in group as cooperative which shows better improvement. In this case they get money and could purchase diversified foods from the market. Generally in agriculture there are improvements that we observe at the ground in improving of the pregnant and lactating mothers’ nutritional status.

I: what about the women in creating awareness in their home, to get help from their husband, or from the community?

P: As you have said, in the past time, the awareness creation was exclusively given to the women, which was mainly focused on women’s feeding system, production etc. But currently the husband is included to have awareness. Currently, the overall of our work is family based approach. When we give training to the women, the husband/household leader and students are included. This is the general approach. But when we see in nutrition both of them should be included. If it is need to get help the mothers from their husband, what should expected to know the husband. This is the awareness that we need to have them.

I: In your opinion, which of the nutrition interventions for the pregnant women was less successful? Why?

P: In this case, we have not anything that failed in our plan; however, the success was not according to the expectation or sated goals. Even that of we have discussed before, it was not diffused to other woredas or throughout the region. Here the problem is lack of giving continuous focus; there is in and out activities and less human power; for example, if you remember in the past, there were home economics experts in each village that popularized all the housework activities. But now, there is no such kind of human power or network.

I: What is that? Can you explain that home economics expert please?

P: Home agent means a profession of home economics just like agricultural development agent and worked at village level focused on agriculture. It was part of the agricultural structure that promotes nutrition. We have got information that because of the absent of this structure (i.e., home agents), the nutritional improvement was reduced. Currently the professionals graduated in home science from Mekelle University are working in around 19 woredas, but since it is not well strengthen the plan and the structure, their performance was not satisfactory.

The other thing that is not effectively diffuse throughout the region is the introduction of milk Holstein; it is very important to support the lactating women, pregnant women in the improvement of nutritional status; but as it was said that we didn’t implement in massive coverage. Second, the milk produced from small ruminants is very important thing for nutrition; but still we could not give attention and less effort was done in its coverage in the region. Currently the plan that we have in Soquota declaration is to produce milk in a good way from cattle and to make benefited the women from it; but still we are not working on this area. The product from the cattle is used only either for kids, or to produce some butter; but still the pregnant women and lactating women are not benefited from the cattle milk. In general, these are the most our weaknesses that we didn’t do effectively in pregnant and lactating mothers.

I: what about in relation to awareness? It might be the awareness gap of our expert, the community, even the educational level of the society are the factors for less success. What do you think on that?

P: Of course there is awareness gap at the expert and community level. But when we are working, we assess ourselves including me, if I am going to talk to expert in woreda what should I know, what I should look like, the same thing the woreda expert when they go to kebles just like what the region expert look like; therefore there is a sort of self-preparation so as to convince the community, currently such kind of preparation are encouraged and improved. However, still it shows that there is a gap in nutrition improvement of the pregnant and lactating women. There is also similar gap in the farmers. The awareness coverage is still low.

In case of education it is obvious that the educational status is increase the level of acceptance to new technology is increased.

**Section 4: Implementation challenges and community factors affecting access to nutrition interventions**

I: What are the challenges to implement delivering the nutrition interventions that we have been discussing for pregnant and lactating women?

P: Now, there is a gap as you said. Since nutrition is multi-sectoral, it is led by the vice director of each sectors. And there are different directors of the multi-sector. Here, in order to have a discussion on the main problems of the nutrition and how to solve that, some of them may present on the discussion time and some of them are not; that is what I observed. The other thing is when we come to our bureau; there is gap of integration in work towards the nutrition. Here internally we have established nutrition technical working team, but in evaluating the implementation of the plan, which was prepared by the technical team has its own gap. Therefore, there is integration problem starting from the multi-sector and here in our bureau. Currently, according to Soquota declaration, there is new thinking that one plan, one goal, and one MND. Now, to have the same plan among the governmental sectors, non-governmental sectors that is within our institution and out of our institution all sectors that work in nutrition, like FAO, Irish aid, REST and other projects that works in nutrition improvement have the same template of plan as well as report. This was decided and the template was approved by the government at the presence of Ato Abay, Ato kiros and the Federal coordinator of Sequota declaration. There is an assigned expert that crosscheck whether we are working based on the template or not in each sectors. From now there will be a change in our plan, support, and in our report.

I: what about the community cultures, beliefs, norms that are preventing or supporting to the intervention? How?

P: In relation to region there is approved by synods that could help to consume a non-fasting food for the pregnant and lactating women in during the fasting time. And this was known and popularized through creating forums by the regional, woreda and other concerned religious bodies. But it is still challenging to accept even if it is approved by synods, unless the local bodies are not participated on the awareness creation.

There is also practiced that women don’t take milk, butter, egg, etc; still this kind of culture is existing in some places regardless of the fasting time. When we ask them why females don’t take milk, egg, is the reason that they take the lead of the household and in such thing female would be dominating or influence the man.

I: What about the resistance level of the community during introducing a new technology?

P: During introducing of new technology, resistance is common among the communities. For example, if take Beless/cactus, except the fruit part the other part is not edible. But you can make juice and other thing from that part. But now there are some improvements among the community to the new technology.

I: Are the interventions accessible to the women and adolescents, in terms of transportation and cost?

P: In relation to water access, this is just under the water sector. But out of this, the strategy that each household should have more than one option to get water; but at the ground, who have that and who have not? We just assess, even if the women does not able to work in productive safety net program, we should also support the water access by excavating a borehole /Horeye/ to use for irrigation.

I: How convenience is interventions to the women, the quality of the intervention?

P: The approach of the extension work is starting from the training, for example in the previous time the husband was only involved in the training. But starting from the last year we follow a family based approach. Both the husband and wife should take the training. Therefore, the awareness is started from the household. During the past time of the supporting system was husband based approach, when the experts (subject matter specialist) went to supervise the households and if the husband only were stay at home just we discuss and counseling to him only and we assume that the women could take the information from her husband. But, currently at least if there is supervision and when the supervisor goes to household, it must be get both the husband and the wife and make a discussion with both. It is not recommend getting the husband only.

I: Is there any channel that contact the households or someone goes to the households from the regional bureau?

P: The structure is just we are at region level, then at woreda level and Tabia or kebele level. In one kebelle at least there are four agricultural development agents. In cluster level for three to four kebele, there is one agricultural expert. The development agent may be the expert of apiculture or artificial insemination and the like; in general, we have almost 9 development agent in one Tabia. Hence, the structure is up to this. When the structure goes down, there is women development team, there is men development team; and then our support is based on the teams. There is special thing that, the subject matter experts are from the regional bureau and then directly go to household.

I: Do you mean that there is cross-checking?

P: Yes, it is cross-checking at household level; the feedback got from the household is addressed to the development agent, then to the woreda expert and up to the regional bureau. Therefore, this approach is the way of supporting system we follow.

I: what resources exist to provide the intervention and what do not exist?

P: As I told you before, there are some mothers that bring better impact and could generate better income. Now they are benefited ether in income or in consumption. But we credit fattening or dairy, can they afford it? Fattening, it costs high investment, dairy it costs high investment, and then if you provide as credit such kind of finance for the lactating women, or pregnant women, they might not afford it. Therefore, there is great gap in the areas of that need higher investment.

Sometimes if we introduce something new technology with high product, then there will be a market linkage problem. Currently there is high productivity of wheat but no market. If you see now there is a complaint in milk market, there is no market particularly for milk. In Aksum, Shire, during fasting days, egg is surplus and no demand in the market. So, still we are not working in market and value chain analysis, agro processing. Currently there are some activities started on that with agricultural transformation agency. And also we are working effectively in post harvesting technology. Let say if she produce tomato how can she reserved for a long time? Therefore, we have to work in supplying the preserving materials and how to use it. Hence, it needs to work on in relation to post harvest technologies such as milk, egg, tomato etc.

I: How do you evaluate the commitment of the intervention providers at your level? Towards nutrition improvements

P: Now in commitment, since we did not work on, now we have to work on and we should make awareness among the communities. In particular we need here to bring a change, but sometimes we discuss general things. When general things raised this implies that the commitment is masked. Therefore, raising an idea in particular to improve the nutrition gap is very important. But with regard to commitment, even the bureau head is focused on that issue and raised the idea in different stages. At present, bureau head believes that any agricultural work should be implementing with the perspective of nutrition improvement. Therefore this is the reflection of the need and commitment to work in nutrition improvement. If the bureau head gives more focus and emphasis on nutrition improvement; hence, in the lower structure (woreda and kebelle level) there could produce good need and commitment to work on nutrition improvement.

I: As you mentioned before, there are some challenges/gaps, like post-harvest problem, technology gap to preserve perishable fruits and vegetables, milk products, coverage of food products, but what else that can you mention as challenges?

P: Beyond these challenges; in general, there is also awareness, knowledge and skill gap; though, the awareness and skill differs from one to another. There are gaps in the presence of experienced skilled persons; therefore, there is gap of experience, exposure, knowledge. Let say in nutrition it is not only gap of knowledge but also there is gap of skill. Therefore, if we solve it in short term, the improvement of nutrition will be promoted. The challenges are already I mentioned before.

I: What do you think the solutions? In what way can we solved these problems?

P: The solution will be possible, even though the awareness is good; in general we have to work on the skill gap. The occupational standard (OS), we have OS of crop, livestock, natural resource, there should be manual, curriculum. There are experts and farmers that trained in agricultural college. But there is no curriculum in the OS about nutrition. Therefore, we have to incorporate the curriculum on nutrition on the occupational standard. Then if we incorporate the curriculum of nutrition, we can solve the problem or gaps of knowledge and skill with regard to nutrition. Secondly, there is a difference being the partners work independently and work in collaboration. Hence, if the partners come together to work in collaboration there will be one plan, one goal, and one MND, there will be nutrition improvement.

Any project that works in nutrition must have the same plan, must have the same evaluation method, and must have the same goal. Therefore, the integration among stakeholders should be strong enough. There is no change being work individually. We could see in stages or conferences, but even now if we go in a strong way it is possible to solve the problem. Especially, in introducing effective technologies, as I mentioned like high protein maize, through searching, introducing variety and by conducting research, we can solve the challenges. Currently, our bureau the researchers are advised to work any kind of research in terms of nutrition improvement. Therefore, if we conduct research to improve nutrition, if there is strong linkage among stakeholders, and if we introduce effective technologies; the other things can be solved without difficulty.

**Section 5: Multi-sectoral collaboration to improve maternal nutrition**

I: What do you think the effect being nutrition is a multi-sectoral issue?

P: Being nutrition is a multi-sectoral issue, it has its own advantage, but there should be strong collaboration among the stakeholders. If we think that to bring into one sector, I don’t know, is it possible especially with the condition we live is the question. You can think that establishing one agency or bureau that gives focus to nutrition, it might take a long time. But within the existing situation, if we make the integration strong the improvement/change will be overcome.

I: Which other sectors do you feel are necessary to work with your institution?

P: From the governmental sectors: just health bureau which is the basic, water bureau, bureau of education, bureau of women affairs.

I: What about from the non-governmental institutions?

P: From the non-governmental organizations: UNICEF, which is the first and basic one; there is also Irish aid, which has its own team in our sector; AGP, which works in terms of fund and others in nutrition improvement; Save the Children.

I: What they help for your institutions? What roles have both the governmental and non-governmental organization?

P: The non-governmental organizations are help in capacity building; from the regional level up to the developmental team; they allocate budget for capacity building, like UNICEF. Irish aid is also help in income generation activities of the women, it supports in terms of providing chicken, small ruminants, other thing like quality maize protein. Beyond this, in strengthening the center of farmers training center Irish aid has the greatest share. Moreover, currently there is a project that works on emigrant refugee. This project is also working in environmental management, in maximization of production and productivities, in income generation, generally work in introducing financial capacity and physical capacity building.

I: What about the governmental sectors?

P: With the health bureau; currently what is working on with SURE is: in the past time, our development agents and the health extension worker went to households lonely and there was not collaboration in between them. Currently both the development agents and the health extension worker go to households together so as to provide advice on the improvement of nutrition in pregnant and lactating mothers. As I told you in AGP there is both financial and physical capacity building for both groups.

I: What is your evaluation mechanism?

P: Yes, as I told you before, there is technical working team established from UNICEF, Finance, and other multi-sectoral organizations, who are the concerned body goes to woreda and kebele in order to evaluate the implementation of the nutrition improvement interventions.

I: What type of resistance to the needed change do you perceive, or have you experienced so far?

P: The reason that cannot see a change in the multi-sectoral is: in drafting the plan together, and during evaluating the plan, I tried to participate myself two or three times and what I observed is that; let say health bureau is the coordinator but they may not present during planning or evaluation. And let say the representative of bureau of agriculture is vise coordinator but they may send lower expert instead of participating the director in planning and evaluation. The same thing in bureau of education; such kinds of challenges are there, this is by itself implication of resistance. So here, if at the above level or reginal level of the multi-sectoral becomes well strengthened; at the lower stage the challenges might be solved. But still up to this time it is difficult to conclude such kinds of challenges are solved.

I: How effective are the coordinating platform in enhancing multi-sectoral coordination?

P: Here as I told you before, such kind of work should not be work by delegation. Though there is delegation, the one who is going to delegate for this work should be committed and feel the work as its own work. For example in our institution, when the manager assigned a person, the manager believes that the assigned person is well oriented, committed, even there is evaluation after the work; however, there is just similar weight it is difficult to say our institution is better than health bureau or other sector and vis versa.

I: What do you think to strengthen it?

P: First, the integration among the multi-sector should be strengthening. Another thing the Soqouta declaration even it works in six woredas, it should be strengthened it in order to have the same plan, the same goal and the same MND.

I: What opportunities do we have to promote the multi-sectoral coordination?

P: The opportunity that we have is: first there is ready organ for this nutrition improvement, with NNP-1, even NNP-2 the government is the main actor to bring a change on this issue. If we see in Soqouta declaration, the regional president, Ato Abay was participating in two three conferences or stages. The nutrition regional committee is also coordinated by Ato Abay. This is a good opportunity what we have. We have also enough man power, when we compare to other regions, here is better in man power especially the development agent at Tabia level. Here in our region we have up to 9 development agents in one kebelle, but in other region it is up to three or four. This can be considered as an opportunity for us. Other thing in reducing the challenge of man power, we have four universities now, so there are a lot of man powers in nutrition, for example, from Mekelle university professor Fetyen and Dr. Afework are working on this.

**Section 6: other interventions that influence adolescent and maternal nutrition and health outcomes**

I: What do you think about under marriage? What is the knowhow you have as an institution?

P: In this regard of curse it is not evaluated, but still there is such kind of under marriage. The problem is not totally changed, as we hear in different media. If the social behavioral communication change is strengthening and this works with all sectors may bring a change. When we go to field work, you see that thing, and there are girls absent from education because of that under marriage. Even with regard to production it has an effect on girls. But as institution there is no focus towards underage marriage. It may not belong to all sectors may be with those teams work in nutrition improvement.

I: What about with family planning? Is your institution work on that?

P: When we work with nutrition of course we raised in relation to the kids feeding system, family planning, under marriage we discuss on that. As institution, when we mainly focus on production and productivities but it is important to discuss on other factors that affect the production, like under marriage, family planning etc.

I: What do you say the barriers for under marriage, family planning /space between each birth/ as your institution?

P: Concerning the barriers, we could not understand the problem in depth. If the family have a girl, they think that it is better to get married, because they afraid for other problems that the girl bring to home. Therefore, the awareness level of the effect of under marriage, giving birth under age on nutrition challenges is very low. Just they believe the child will grow by their fate. This one may the most barriers in this issue. Other thing is attitude; they think that if their daughter gets married with a rich person, the family problem may solve. That family is better, and if I give them my daughter, I can get help from them; they may help me in agricultural activities; this is the understanding problem.

I: What about in increasing space between each birth?

P: In increasing space between each birth, the problem is in both the awareness giver and the awareness taker.

I: What solution do you recommend for this problem? As institution what is expected towards the solution?

P: As institution we are teaching and capacitating the production and productivities and we work on nutritional improvements, therefore, here we can work side by side on the increasing space between each birth. We can teach the advantage of increasing space between each birth and the disadvantage of increasing space between each birth; and we have to include in our program. If we go through this, there will be a change since we have a structure up to a lower stage of kebele.

I: What opportunities do we have to support this issue?

P: We have the opportunity that the production and productivity of the agricultural product is increased. We will provide farm land to the landless youth; we are also doing on the adult education so as to easily accept the training. We have already developed a curriculum in the adult education in collaboration with health and education sector; so this could maximize the awareness of the community in the region.

**Additional remarks**

I: Do you have any other comments on anything that we have discussed?

P: We have learned a lot and it could be nice had it been started in this type of approach in early time; we are observing the change now; for example during the soil and water conservation campaign, pregnant and lactating mothers were working with their child; now we give them rest, and you can observe healthy mothers and their children.

Now we have the opportunity of Soquota declaration, I was participating in the forum and I am working as focal person here, so it will bring great change and it will be diffused into other woredas of the region other than the six woredas. The attention of the government that is given to the improvement of nutritional status in mothers and children is great opportunity.

I learned a lot from what you observed in the ground; so let’s do together in this area so as to bring a change on the coming generation.

**SUMMARY**

**Section 1: Common maternal (Pregnant women) nutrition problems in the community**

- Currently, there is a commitment from the government side on how to create a linkage with health extension experts and the production and productivity of the agricultural sector.
- In regard to overweight some changes are existed in urban areas like what we see in other countries. We observe that there is overweight in some school students, this is due to the style of our diet.
- When we go to the rural areas, all the activities are on the shoulder of the women regardless of their pregnancy or lactation. She is the one who goes to the market, fetch water and involve in the agricultural activities.

**Section 2: Nutrition priorities in the woreda**

- Currently the agricultural bureau introduces maize with high protein content and energy content for the pregnant and lactating mothers.
- There is an improvement in the consumption of home grown foods in the community; for example, if there is water even in small amount, they plant vegetables in their back yards and utilize the products for themselves.
- There is less focus on the nutritional improvement of adolescent girls in the community.

**Section 3: Nutrition interventions that improve adolescent and maternal health**

- We are developing a mechanism that how husbands could help his pregnant or lactating wife; If the husband is a model in helping his pregnant or lactating wife, we put a sign on his house that indicates as a good or role model husband.
- In some woredas it was supported up to five chicken per households and from their product they could consume the eggs and to serve as a source income.
- There is honey bee production that could produce either individually or in group as cooperative which shows better improvement in the income generation and they purchase diversified foods from the market.
- In the past time, the awareness creation was exclusively given to the women, about nutrition improvement, but currently the husband is part of the trainee.

**Section 4: Implementation challenges and community factors affecting access to nutrition interventions**

- Since nutrition improvement program is a multi-sectoral agenda, stakeholders take as secondary activity and the representative body is not attending during the common plan development and evaluation of the activities.
- All government and non-governmental organizations who work in the improvement of nutrition in pregnant and lactating mothers in nutrition have the same template of plan as well as report.
- There are experts and farmers that were trained in agricultural college; but there is no curriculum in the occupational standard about nutrition.
- If we conduct research to improve nutrition, if there is strong linkage among stakeholders, and if we introduce effective technologies; the other things can be solved without difficulty.

**Section 5: Multi-sectoral collaboration to improve maternal nutrition**

- There is technical working team established from governmental and non-governmental organizations Finance, who is the concerned body, goes to woreda and kebele so as to evaluate the implementation of the nutrition improvement interventions.
- The opportunities are, there is ready organ for this nutrition improvement, with NNP-1, even NNP-2 the government is the main actor to bring a change on this issue.

**Section 6: other interventions that influence adolescent and maternal nutrition and health outcomes**

- When we go to field work, we observe that there are girls absent from education because of that under marriage. But as institution there is no focus towards underage marriage.
- If the family has a girl, they think that it is better to get married early, because they afraid for other problems that the girl could bring to home like unwanted pregnancy.
- There is adult education program in the region that could support the ban of early marriage, since education has a great factor on easily acceptance of any training.

**Additional remarks**

- The attention of the government that is given to the improvement of nutritional status in mothers and children is great opportunity.
