# Supplementary material for: Barriers to utilize nutrition interventions among lactating women in rural communities of Tigray, northern Ethiopia: An exploratory study
Source: PLoS One. 2021 Apr 30;16(4):e0250696. doi: 10.1371/journal.pone.0250696 (PMC8087028; doi:10.1371/journal.pone.0250696)
Supplement: S1 File — (ZIP) [file pone.0250696.s001.zip › S1_File.Doc/IDI with Experts.doc]

## Tool A

## IDI GUIDE WITH EXPERTS, NUTRITION FOCAL PERSONS AND HEALTH EXTENSION WORKERS

**Introduction:**

Hello, my name is _____. I am from Mekelle University. Thank you for taking the time to speak with me today. We are doing research on the factors that influence the nutrition of mothers and adolescents in collaboration with the Regional Health Bureau and UNICEF. Your participation is very valuable. The things that you tell us will be used to improve nutrition programs and services for women in the region and the country. We will not share your names when we report our results.

However, I will record the discussion so that I can capture all the ideas that are shared. I have several questions to ask you that I have prepared in advance, and I will ask you to say what you think about each question. The interview will last for 1:30 -2:00 hours. Do you have any questions before we begin? If you think of any questions as we proceed, please feel free to let me know. If it is all right with you, I will turn on the tape recorder now.

Do you agree to participate in the study?

1. Yes
2. No

**Section A: Interview details**

1. Zone:_____________
2. Woreda: -----------------------------------------
3. Kebele: ------------------------------------------
4. Name of key informant: -----------------------------
5. Institution of key informant: ---------------------------------------
6. Interviewer name: ----------------------------------------------
7. Date of interview ------------------------------------------------
8. Interview start time: _________________________
9. Interview end time: __________________________

**Section B: Interviewee professional information**

1. Gender
   a. Female
   b. Male
2. Age: _________ yrs
3. Highest level of completed education.
   a. No formal education
   b. Primary education
   c. High school
   d. College education
   e. Bachelor degree
   f. Master’s degree
4. Current job/position: ____________________________________________
5. How long have you been in the current job/position:
   a. ______ Months
   b. ______ Years

| **Section 1: Common maternal (pregnant women, lactating women and adolescent girls) nutrition problems in the community.** | |
| --- | --- |
| 1.1 | In your opinion, what are the common nutrition problems in the community for women? What about for adolescent girls?  Probe for:   - Severe and moderate malnutrition? - Micronutrient deficiencies (such as anemia, night blindness, goiter)? - Diet related non communicable diseases? - Stunting (low height for their age)? - Under weight (low weight for their age)? - Overweight? - Food insecurity? - How do you think women are especially at the risk of malnutrition you have mentioned above? - Do women in this community suffer from stunting? Probe for LW and AG? |
| **Section 2: Nutrition priorities in the woreda** | |
| 2.2 | - What priorities do your institution has in relation to maternal and adolescent health? Why? - What your institution is doing currently related to the priorities you have mentioned? What nutrition interventions have the most resources allocated to them? Could you tell me in detail with examples? - Do you think is necessary for your institution to get involved in work aimed at improving nutrition among women and adolescents? Why? [Probe to relate it to his/her institution’s mission] - How do you evaluate the priority given for the interventions for the women? |
| **Section 3: Nutrition interventions that improve adolescent and maternal health** | |
| 3.1 | What kinds of nutrition interventions are in place to improve health of the PW to your level?   - How they access it? What about for LW? - What about for adolescent girls (AG)?   **Probe for:**   - Advise ANC during pregnancy? - Counseling extra meal during pregnancy and lactation? Do pregnant women get screened for their nutritional status?   - During pregnancy?   - During Lactation?   - For Adolescence? Probe:   - Counseling for food diversification during pregnancy? Probe: during Lactation? - Advice iodized salt? [**Probe for all**] - Advice on nutrition sensitive agriculture such as home gardening? Probe: **Productive safety net program, food security, workload reduction and others? [Probe for ALL]** - Advice on water, sanitation and hygiene services?[Probe for all] - Are pregnant women getting advice on the need to use Insecticide treated bed nets (ITN)? Why? Who advise them? - Deworming services? [Probe for all] - Targeted supplementary feeding (TSF)? [**Probe for all**] - Vitamin A supplementation? [Probe for LW and AG] school feeding?   - Probe: for out-school adolescents? - Youth friendly services at health facilities? Probe:   - For out- school adolescent girls - Which of the interventions listed above do you think is most important for pregnant women?   Probe:   - For lactating women - For adolescent girls |
| 3.2 | In your opinion, which of the above interventions for the pregnant women are being implemented in successful way? Why??  Probe:   - For lactating women - For in-school adolescent girls - For out-school adolescent girls |
| 3.3 | In your opinion, which of the interventions targeted to the pregnant women was less successful? Why?  Probe:   - For lactating women - For in-school adolescent girls - For out-school adolescent girls |
| **Section 4:** **Implementation challenges and** **Community factors affecting access to nutrition interventions** | |
| 4.1 | What are the challenges to implement delivering the nutrition interventions that we have been discussing for the pregnant women? Probe:   - For lactating women - For in-school adolescent girls - For out-school adolescent girls   **Probe for pregnant, lactating and adolescent girls:**   - Individual level factors:   - How aware are the women and girls on the need to get the interventions?   - Is there a relationship b/n educational status of the women & access to the interventions - Community level factors:   - What community related beliefs and norms are preventing access to the interventions? How?   - Are the interventions culturally acceptable? Why and why not?   - Are the interventions accessible to the women and adolescents? Probe in terms of transportation and cost? - The intervention itself:   - How convenience is interventions to the women and the adolescent girls?   - How do you explain the quality of the interventions   - What resources exist to provide the interventions and what do not exist?   - How do you evaluate the commitment of the intervention providers at your level? - What other factors are inhibiting implementation of the interventions? how? |
| 4.2 | - For these challenges that you mentioned, can you tell me of any solutions that your institution have applied to effectively implement the interventions for women and adolescent girls? Specify the each solution done for each challenges? - While your institution tries to solve the challenges, what problems do it faced? - What do you think needs to be done to better address the challenges you have mentioned? How they can be addressed better? |
| **Section 5: Multi-sectoral collaboration to improve maternal nutrition** | |
| 5.1 | Do you feel it is necessary at your level to work with other sectors/institutions to address maternal nutrition? What about for adolescent girls’ nutrition? Why?  Probe for:   - Which other sectors do you feel are necessary to work with your institution? - How do you see the other institutions’ roles complementing your role in improving maternal and adolescent nutrition? - How do you evaluate the level of collaboration among sectors in nutritional interventions? Why do you think is so? |
| 5.2 | - For multi-sectoral action that effectively works to improve maternal and adolescent nutrition at your level, what kind of change in terms of the way stakeholders work together is needed? - What type of resistance to the needed change do you perceive, or have you experienced so far? |
| 5.3 | - Is there coordinating platforms in enhancing multi-sectoral coordination in maternal and adolescent nutrition? - To what extent does your institution participate in the multi-sectoral nutrition coordinating body at this level?   Probe for:   - How effective are the coordinating platforms in enhancing multi-sectoral coordination? - What needs to be done to improve the capacity of these bodies/platforms for effective coordination? - What opportunities do exist to promote multi-sectoral coordination of nutrition in this woreda? |
| **Section 6: Other interventions that influence adolescent and maternal nutrition and health outcomes** | |
| 6.1 | - In your opinion, why would delayed marriage (after 18 years) improve maternal nutrition? - In your opinion, why would increase the space between each birth improve maternal nutrition?   PROBE:   - What programs or activities promote increasing birth intervals in this level? Can you tell me about any programs or policies in place in this woreda to prevent early marriage?   Can you think of any more programs or policies? Think about political, religious and other influences. |
| 6.2 | In your opinion, are these programs or policies effective? Why or why not?  PROBE:   - What are the community factors that affect age at first marriage? - What are the policy factors that affect age at first marriage?   In your opinion, what could be improved? |
| 6.3 | Can you think of any other opportunities to prevent early marriage and increasing birth spacing?  PROBE:  For example, building awareness at schools |
| Additional Remarks | |
|  | Do you have any other comments on anything that we have discussed?  Probe for:   - What lessons have you learnt regarding adolescent and maternal (pregnant, lactating and adolescent girls) nutrition at your level? - What lessons have you learnt regarding multi-sectoral coordination of nutrition in this woreda?   What opportunities do exist to promote maternal (pregnant, lactating and adolescent girls) nutrition in this woreda? |
| Thank you for taking the time to discuss these issues with me today. We have learned a lot from you. As I mentioned at the start of the interview, we will remove all identifying information from the transcript of this conversation. We will make sure that no one can identify you from your comments. If you have any concerns or questions, please feel free to contact me (contact info). Thank you very much for your time. | |
