# Supplementary material for: Barriers to utilize nutrition interventions among lactating women in rural communities of Tigray, northern Ethiopia: An exploratory study
Source: PLoS One. 2021 Apr 30;16(4):e0250696. doi: 10.1371/journal.pone.0250696 (PMC8087028; doi:10.1371/journal.pone.0250696)
Supplement: S1 File — (ZIP) [file pone.0250696.s001.zip › S1_File.Doc/FGD[Men&WDA] and IDI [kebele leader[&WDA].doc]

## Tool B

## FGD GUIDE FOR MEN OF REPRODUCTIVE AGE

## [also for IDI for Kebele leaders, WDA]

| **Introduction:**  Hello, my name is _____. This is my colleague ______. We are from Mekelle University. Thank you for taking the time to speak with us today. We are doing research on the factors that influence the nutrition of mothers and adolescent girls in collaboration with the Regional Health Bureau and UNICEF. Your participation is very valuable. The things that you tell us will be used to improve nutrition programs and services for women and adolescents in the region and the country. We will not share your names when we report our results.  However, we will record the discussion so that we can capture all the ideas that are shared. We have several questions to ask you that we have prepared in advance, and we will ask you all to say what you think about each question. To ensure the privacy of everyone here, we ask you not to repeat what we discuss outside of this group. The discussion will last for 1-2 hours. Do you have any questions before we begin? If you think of any questions as we proceed, please feel free to let me know. If it is all right with all of you, we will turn on the tape recorder now.  Do you agree to participate in the study?   1. Yes 2. No     **Section B: Interview details**   1. Zone:_____________ 2. Woreda: ----------------------------------------- 3. Kebele: ------------------------------------------ 4. Facilitator’s name: ---------------------------------------------- 5. Date of discussion: ------------------------------------------------ 6. Discussion start time: _________________________ 7. Discussion end time: __________________________ | | | | |
| --- | --- | --- | --- | --- |
| **Section B: Socio-demographic Information** | | | | |
| Name of the FGD participant | Age | Marital status | Education level | Occupation |
| 01. |  |  |  |  |
| 02. |  |  |  |  |
| 03. |  |  |  |  |
| 4. |  |  |  |  |
| 05. |  |  |  |  |
| 06. |  |  |  |  |
| 07. |  |  |  |  |
| 08. |  |  |  |  |
| 09. |  |  |  |  |
| 10. |  |  |  |  |
| 11. |  |  |  |  |
| 12. |  |  |  |  |

| **Section 1: Common maternal (PW, LW and adolescent girls) nutrition problems in the community.** | |
| --- | --- |
| 1.1 | - What do pregnant women do to stay healthy in this community? Why?   - What about Lactating women do? Why?   - What about Adolescent girls do? Why? |
| 1.2 | In your opinion, what are the common nutrition problems in the community for Women/Girls?   - In your opinion, how do you explain the risk of malnutrition for women? - Why do you think are especially affected by malnutrition? - How sever the nutrition related problems you have mentioned are among the WOMEN in this community? - Do you think that women in this community are suffering from Micronutrient deficiencies? (Probe for anemia, night blindness, goiter and others)?   - Why do you agree or disagree with this?   - What do you think are the reasons for the deficiencies? - What communicable diseases are common among women in this community?   - Why do you think the reasons are?   - Do you think these could be associated with their nutritional status? - What do you think on why women/girls in this community would not increase their height proportional to their age?   - Could it have relationship with their nutrition?   - Could you tell me about it with example? - Do women/girls in this community increase their weight proportional to their age?   - If no, what do you think the reasons would be?   - Do women in this community suffer from overweight? If yes, what do you think the reasons would be? - Is there a situation when women suffer from shortage of food?   - In what situation do you think this happened? - How frequent does it happen? Why? What other problems do women in this community suffer from? Why? |
| **Section 2: Barriers to access and utilization of nutrition services** | |
| 2.1 | - What kinds of nutrition interventions are in place to improve health of the pregnant in this woreda? Where do they get it? Who provide it?   Probe:   - Do WOMEN advised to visit HFs for checkup and services during pregnancy? What services do they get? What would it help to them? - Do you think that WOMEN receive advice on the need to get extra meal during pregnancy and lactation?? How? How do you think that it would help for? - Are Women getting counseling for food diversification during pregnancy and lactation?   - Could you tell me about it with examples? Do you think it is necessary? How?   - Are WOMEN getting advice for the need to use iodized salt? Why - Are WOMEN/adolescent girls getting advice on nutrition sensitive agriculture such as home gardening?   - What about on the need to be involved in safety net programs?   - What else interventions related to this? - Are Women/Girls getting advice on water, sanitation and hygiene services? How they do get it? Its necessity? - Is Malaria common in this community?   - Are women getting advice on the need to use Insecticide treated bed nets (ITN)? Why? Who advise them? - Are Women/Girls getting deworming services?   - How do they get it? Who provides the service?   - Why do you think is necessary for women in this community? - Is there a situation in this community how that you think that women need to be addressed through Targeted supplementary feeding (TSF) for women? Why? Could you tell me specific examples and situations? - Which of the interventions listed above do you think is most important for Women/Girls? - What are the barriers in the implementation of these nutrition services? ( counseling for food diversification , extra meal, checkup and services, Insecticide treated bed nets, Targeted supplementary feeding, advice on water, sanitation and hygiene services ) |
| **Section 3: Perceived needs of women for relevant services during pregnancy** | |
| 3.1 | What special things do PW,LW and Adolescent need in your community? [Ask them to customize it to the LW in their community and themselves?]  Probe:   - Visiting health facility - Taking extra meal - Taking rest - Taking supplements - Others - What should be the role of a husband to improve nutrition for LW? |
| 3.2 | Do WOMEN in this community typically change their diets when they are pregnant?  Probe for:   - How is this diet different from when they are not pregnant? Probe for More or Less food? - What foods are recommended for Women/Girls? What foods do Women/Girls avoid? - What affects WOMEN’s diet during pregnancy? - Are there gender disparities in women’s diets before pregnancy and during pregnancy? |
| **Section 4: Other interventions that improve pregnant, lactating and adolescent nutrition** | |
| 4.1 | - Have you ever gone for nutrition screening? Where? Who provide you? - Probe for: - Do you think community health days would have benefits for the WOMEN? How? What about routine service delivery?. - What are the challenges to attending community health days? what about to accessing the routine service delivery? - Do you think WOMEN needs to be targets for supplementary foods? Why and why not? |
| 4.2 | Are WOMEN beneficiaries of the soft conditionality of the productive safety net program, PSNP? How?  Probe for:   - Do women in this community know why they are targets of the program? |
| **Section 5: Understanding perceptions of age at first birth and birth spacing** | |
| 5.1 | Do you think delaying the age at first birth to after 18 is better for the health of the women? How? What other benefits does it have for the women? What about for the baby?   - Does this delay would have a benefit to the nutritional status of the women? - Do you think this message is being promoted in the community? Who are working on it? How do they promote? - In your opinion, what does the reaction of the community looks like to the promotion? Why? - In your opinion, how could this message be better promoted? Who should be involved? - Can you think of any other opportunities for promoting birth spacing? |
| 5.2 | - How many years do you think the gap should be between successive births for women? Why? What about if shorter than it? - From who have you ever heard? For the last time? Who are involving on it? - What do suggest promoting it in a better way? Which population in this community is not addressed with the promotion? - Can you think of any other opportunities to prevent early marriage? How? |
| **Section 6: Understanding communication and information sources** | |
| 6.1 | - Is there an opportunity in the community to discuss Nutrition for women? What are the opportunities? Probe: for community conversation, community mobilizations? - What are the common sources for nutrition during pregnancy? What messages? - Do all women get the messages easily? What are the barriers for access to information for nutrition during pregnancy? - Which information is effective for you to change your practice in nutrition during pregnancy? Why? - Which source of information about nutrition is essential for you? |
| **Additional remarks** | |
| 7.1 | - Any other additional suggestions or comments on pregnant, lactating and adolescent nutrition in this community. |
| Thank you for taking the time to discuss these issues with us today. We have learned a lot from you. If you have any concerns or questions, please feel free to contact us (contact info). Thank you very much for your time. | |
